# Supplementary material for: Analysis of the Compositional Features and Codon Usage Pattern of Genes Involved in Human Autophagy
Source: Cells. 2022 Oct 12;11(20):3203. doi: 10.3390/cells11203203 (PMC9601114; doi:10.3390/cells11203203)
Supplement: Supplementary file 1 [file cells-11-03203-s001.zip › Supplementary Figure S1.pdf]

# Supplementary Figure

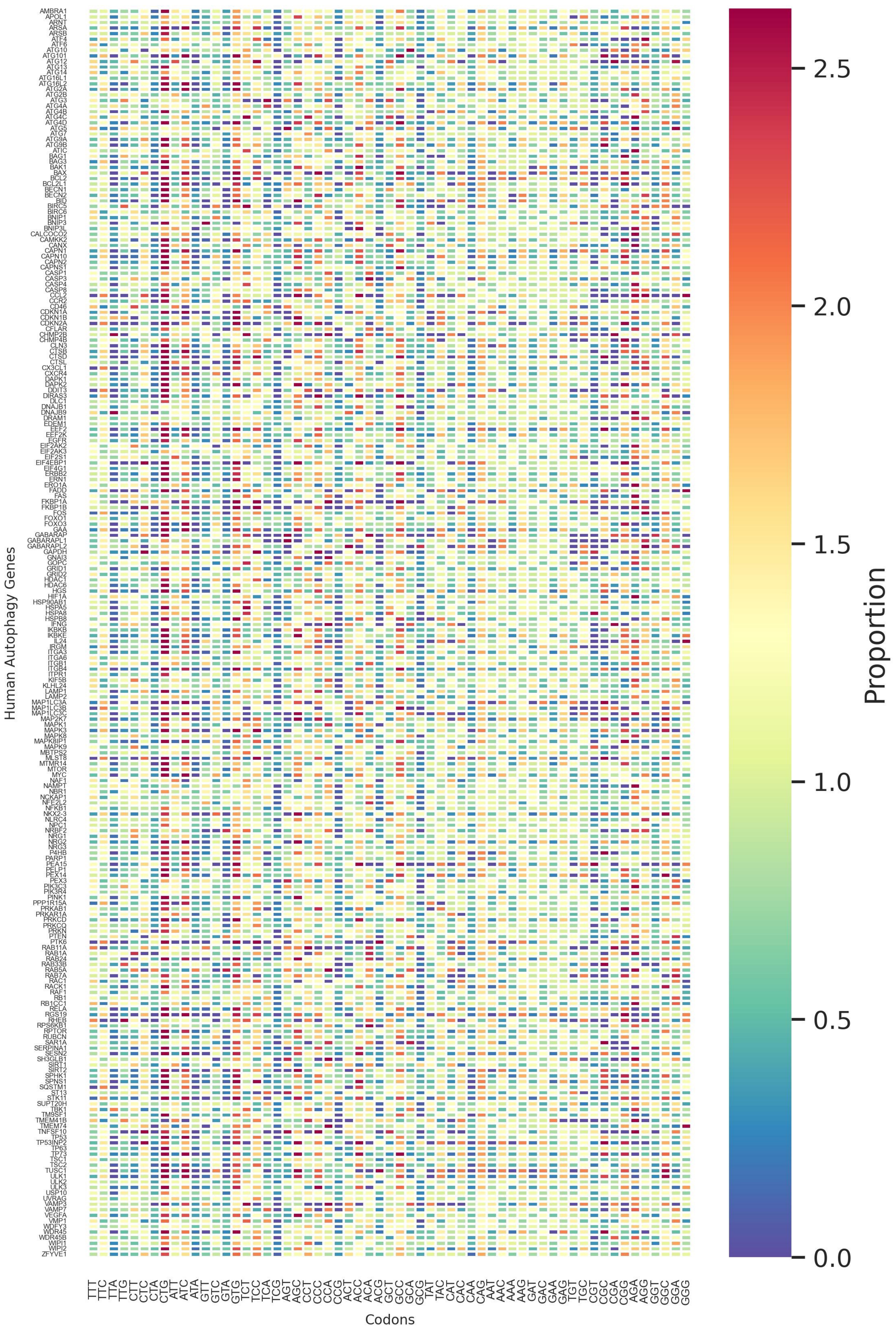


**Supplementary Figure S1.** Heatmap comprising of all RSCU values of 59 degenerate codons of human autophagy genes.
