# Supplementary material for: Analysis of the Compositional Features and Codon Usage Pattern of Genes Involved in Human Autophagy
Source: Cells. 2022 Oct 12;11(20):3203. doi: 10.3390/cells11203203 (PMC9601114; doi:10.3390/cells11203203)
Supplement: Supplementary file 1 [file cells-11-03203-s001.zip › Supplementary Table S2.pdf]

Supplementary Table S2. Coding sequences (CDS) of all the included 224 human autophagy genes.

>lcl|NM_017749.3_cds_NP_060219.2_1 [gene=AMBRA1] [db_xref=CCDS:CCDS31475.1] [protein=activating molecule in BECN1-regulated autophagy protein 1 isoform 2] [protein_id=NP_060219.2] [location=361..3987] [gbkey=CDS]

ATGAAGGTTGTCCCAGAAAAGAATGCTGTCCGGATACTCTGGGGGCGAGAACGGGGTGCTCGGGCCATGG

GAGCTCAGCGGCTTCTGCAGGAGCTGGTAGAAGATAAAACCCGGTGGATGAAATGGGAGGGCAAGAGAGT

AGAACTGCCGGATAGTCCACGCTCTACCTTCTTATTGGCCTTCAGCCCAGACAGGACTCTCTTAGCCTCC

ACCCATGTGAACCATAATATCTATATTACGGAGGTGAAGACTGGCAAGTGTGTTCATTCCCTGATTGGAC

ACCGCCGTACTCCATGGTGTGTCACTTTTCATCCCACCATCTCAGGCCTTATTGCTTCTGGCTGCCTAGA

TGGGGAGGTTAGGATTTGGGATTTACACGGTGGCAGTGAAAGCTGGTTCACAGATAGCAACAATGCCATT

GCCTCCCTGGCTTTCCACCCTACGGCTCAGCTCCTGCTGATTGCCACTGCCAATGAGATCCACTTCTGGG

ACTGGAGTCGACGGGAACCCTTTGCTGTGGTGAAGACAGCTAGTGAGATGGAACGGGTCCGTCTGGTGAG

ATTTGATCCACTTGGACACTACTTACTCACAGCAATTGTTAACCCCTCTAATCAACAGGGTGATGACGAA

CCAGAGATCCCCATAGATGGAACAGAATTATCCCACTACCGTCAGCGTGCCCTCCTGCAATCACAGCCAG

TTCGCCGGACGCCTCTCCTCCACAATTTCCTGCACATGCTGTCCTCCCGCTCTTCTGGCATCCAGACCGA

GCCCTTCCATCCCCCGGAGCAGGCCTCGTCAACGCAGCAGGACCAGGGCCTCCTGAACCGGCCGTCTGCC

TTCAGTACAGTCCAGAGCAGCACTGCCGGCAACACGCTCCGCAACCTCAGTCTGGGTCCTACCCGCCGCT

CTTTGGGAGGGCCTCTGTCTAGCCACCCTTCTAGGTATCACCGAGAAATAGCTCCTGGGTTGACAGGATC

TGAGTGGACCCGGACAGTACTCAGTCTGAACTCCCGCTCTGAGGCGGAATCCATGCCCCCGCCCAGAACC

AGTGCCTCTTCGGTGAGTTTGCTGTCTGTGCTGAGACAGCAGGAAGGTGGCTCTCAGGCATCTGTGTACA

CTTCAGCCACAGAAGGGAGGGGTTTTCCGGCATCAGGGTTGGCAACTGAGTCAGATGGAGGGAATGGCTC

CAGCCAAAACAACTCGGGCAGCATTCGCCATGAGCTTCAGTGTGACCTGAGACGCTTCTTTCTGGAGTAT

GACCGGCTTCAGGAGCTGGATCAGAGCCTGAGTGGGGAAGCTCCCCAGACCCAACAGGCCCAGGAAATGC

TCAACAATAACATTGAATCTGAGAGGCCAGGCCCTTCCCACCAGCCCACCCCACACAGCAGTGAGAACAA

CTCCAACCTGTCCCGTGGCCACCTGAATCGCTGTCGTGCTTGCCACAATCTCCTGACCTTCAACAACGAT

ACCCTGCGCTGGGAAAGAACCACACCTAACTACTCCTCTGGCGAGGCTAGTTCCTCTTGGCAGGTCCCCA

GCTCCTTTGAGAGTGTGCCATCAAGTGGCAGCCAGTTGCCACCTCTCGAGCGGACTGAGGGCCAAACGCC

CAGCTCCAGCAGGCTGGAGTTGAGCAGCTCTGCTAGTCCGCAGGAGGAGAGGACTGTGGGGGTGGCCTTT

AACCAGGAGACAGGCCACTGGGAAAGAATTTACACCCAGTCCAGCAGATCTGGAACTGTGTCACAGGAGG

CCTTACATCAGGATATGCCTGAGGAGAGCTCTGAGGAGGATTCACTCAGGAGGAGGCTGCTGGAATCTTC

CCTCATTTCATTATCCCGTTATGATGGAGCAGGATCCAGAGAGCACCCAATTTACCCAGACCCAGCGAGA

TTATCTCCTGCTGCATACTACGCCCAGAGGATGATCCAGTATCTCTCACGGAGAGACAGTATTCGCCAGC

GCTCCATGCGCTACCAACAGAACCGTCTCCGTTCTTCCACCTCCTCCTCTTCCTCAGACAACCAGGGTCC

ATCAGTAGAGGGAACCGACTTGGAATTTGAGGACTTTGAGGACAATGGTGACAGATCCAGGCACCGAGCT

CCACGCAATGCCCGGATGTCTGCACCTTCGCTTGGACGCTTTGTCCCAAGGCGTTTCTTGCTGCCTGAGT

ACTTGCCTTATGCTGGGATTTTTCATGAACGTGGACAGCCTGGCTTGGCTACTCACTCTTCTGTTAACAG

GGTCCTGGCAGGGGCAGTGATCGGTGATGGACAGTCTGCTGTGGCCAGTAACATTGCCAATACTACCTAC

CGGCTCCAGTGGTGGGACTTCACTAAGTTTGACCTCCCTGAAATCAGTAATGCTTCCGTGAATGTGCTGG

TGCAGAACTGCAAGATCTACAATGATGCCAGCTGTGACATTTCTGCAGATGGCCAGCTCCTGGCAGCTTT

CATCCCCAGCAGCCAGAGGGGCTTTCCTGATGAAGGCATCCTGGCAGTGTACTCCCTGGCCCCCCATAAC

CTGGGCGAAATGCTCTACACCAAGCGATTTGGTCCCAATGCCATTTCGGTGAGCCTGTCCCCAATGGGCA

GATATGTAATGGTGGGCTTGGCCTCACGAAGGATCCTGCTGCACCCCTCCACAGAGCACATGGTGGCCCA

GGTCTTCAGGCTGCAACAGGCCCATGGTGGAGAGACCTCCATGAGGAGAGTTTTCAACGTCCTTTATCCC

ATGCCTGCCGACCAGCGGAGACATGTCAGTATCAACTCTGCCCGTTGGCTGCCTGAGCCAGGGCTTGGCT

TGGCCTATGGTACTAACAAAGGAGACCTGGTGATCTGCCGACCAGAGGCCTTAAACTCTGGTGTTGAGTA

CTACTGGGACCAGCTGAACGAGACGGTCTTCACTGTCCATTCCAACAGCAGGAGCAGCGAGCGGCCTGGA

ACCAGCAGAGCCACATGGAGGACAGACAGAGACATGGGGCTGATGAATGCCATTGGGCTTCAGCCCCGGA

ACCCTGCCACCTCAGTGACATCTCAGGGCACCCAGACTCTGGCCCTTCAGCTGCAGAATGCCGAAACACA

GACTGAGAGGGAGGTGCCGGAGCCAGGGACAGCCGCCTCAGGTCCTGGTGAAGGTGAGGGTTCAGAGTAT

GGTGCCAGTGGAGAAGATGCGCTCAGCAGGATCCAGAGGCTGATGGCGGAGGGCGGCATGACAGCCGTGG

TGCAGCGGGAGCAGAGCACCACCATGGCCTCCATGGGCGGCTTCGGCAACAACATCATCGTCAGCCACCG

CATTCACCGCAGCTCTCAGACGGGCACTGAACCTGGTGCCGCCCACACCTCCTCACCCCAGCCCTCCACC

TCTCGGGGACTGCTCCCAGAGGCCGGGCAACTGGCAGAGCGAGGCCTAAGCCCCCGGACAGCTTCCTGGG

ACCAGCCTGGTACCCCTGGGCGGGAGCCAACCCAGCCAACCCTGCCCTCTTCCTCCCCTGTCCCCATTCC

TGTTTCCCTTCCCAGCGCTGAGGGACCAACCCTCCACTGCGAGTTGACCAATAACAACCACCTTCTGGAT

GGTGGCAGCAGCAGGGGGGACGCTGCAGGCCCTAGGGGAGAACCACGGAACAGGTAG

>lcl|NM_145343.3_cds_NP_663318.1_1 [gene=APOL1] [db_xref=CCDS:CCDS13925.1] [protein=apolipoprotein L1 isoform b precursor] [protein_id=NP_663318.1] [location=213..1457] [gbkey=CDS]

ATGAGATTCAAAAGCCACACTGTGGAATTGAGGAGGCCCTGCAGCGACATGGAGGGAGCTGCTTTGCTGA

GAGTCTCTGTCCTCTGCATCTGGATGAGTGCACTTTTCCTTGGTGTGGGAGTGAGGGCAGAGGAAGCTGG

AGCGAGGGTGCAACAAAACGTTCCAAGTGGGACAGATACTGGAGATCCTCAAAGTAAGCCCCTCGGTGAC

TGGGCTGCTGGCACCATGGACCCAGAGAGCAGTATCTTTATTGAGGATGCCATTAAGTATTTCAAGGAAA

AAGTGAGCACACAGAATCTGCTACTCCTGCTGACTGATAATGAGGCCTGGAACGGATTCGTGGCTGCTGC

TGAACTGCCCAGGAATGAGGCAGATGAGCTCCGTAAAGCTCTGGACAACCTTGCAAGACAAATGATCATG

AAAGACAAAAACTGGCACGATAAAGGCCAGCAGTACAGAAACTGGTTTCTGAAAGAGTTTCCTCGGTTGA

AAAGTGAGCTTGAGGATAACATAAGAAGGCTCCGTGCCCTTGCAGATGGGGTTCAGAAGGTCCACAAAGG

CACCACCATCGCCAATGTGGTGTCTGGCTCTCTCAGCATTTCCTCTGGCATCCTGACCCTCGTCGGCATG

GGTCTGGCACCCTTCACAGAGGGAGGCAGCCTTGTACTCTTGGAACCTGGGATGGAGTTGGGAATCACAG

CCGCTTTGACCGGGATTACCAGCAGTACCATGGACTACGGAAAGAAGTGGTGGACACAAGCCCAAGCCCA

CGACCTGGTCATCAAAAGCCTTGACAAATTGAAGGAGGTGAGGGAGTTTTTGGGTGAGAACATATCCAAC

TTTCTTTCCTTAGCTGGCAATACTTACCAACTCACACGAGGCATTGGGAAGGACATCCGTGCCCTCAGAC

GAGCCAGAGCCAATCTTCAGTCAGTACCGCATGCCTCAGCCTCACGCCCCCGGGTCACTGAGCCAATCTC

AGCTGAAAGCGGTGAACAGGTGGAGAGGGTTAATGAACCCAGCATCCTGGAAATGAGCAGAGGAGTCAAG

CTCACGGATGTGGCCCCTGTAAGCTTCTTTCTTGTGCTGGATGTAGTCTACCTCGTGTACGAATCAAAGC

ACTTACATGAGGGGGCAAAGTCAGAGACAGCTGAGGAGCTGAAGAAGGTGGCTCAGGAGCTGGAGGAGAA

GCTAAACATTCTCAACAATAATTATAAGATTCTGCAGGCGGACCAAGAACTGTGA

>lcl|NM_001197325.2_cds_NP_001184254.1_1 [gene=ARNT]
[db_xref=GeneID:405,HGNC:HGNC:700,MIM:126110] [protein=aryl hydrocarbon receptor nuclear translocator isoform 4] [protein_id=NP_001184254.1] [location=33..2354] [gbkey=CDS]

ATGGCGGCGACTACTGCCAACCCCGAAATGACATCAGATGTACCATCACTGGGTCCAGCCATTGCCTCTG

GAAACTCTGGACCTGGAATTCAAGGTGGAGGAGCCATTGTCCAGAGGGCTATTAAGCGGCGACCAGGGCT

GGATTTTGATGATGATGGAGAAGGGAACAGTAAATTTTTGAGGTGTGATGATGATCAGATGTCTAACGAT

AAGGAGCGGTTTGCCAGGGAAAATCACAGTGAAATTGAACGGCGGCGACGGAACAAGATGACAGCCTACA

TCACAGAACTGTCAGATATGGTACCCACCTGTAGTGCCCTGGCTCGAAAACCAGACAAGCTAACCATCTT

ACGCATGGCAGTTTCTCACATGAAGTCCTTGCGGGGAACTGGCAACACATCCACTGATGGCTCCTATAAG

CCGTCTTTCCTCACTGATCAGGAACTGAAACATTTGATCTTGGAGGCAGCAGATGGCTTTCTGTTTATTG

TCTCATGTGAGACAGGCAGGGTGGTGTATGTGTCTGACTCCGTGACTCCTGTTTTGAACCAGCCACAGTC

TGAATGGTTTGGCAGCACACTCTATGATCAGGTGCACCCAGATGATGTGGATAAACTTCGTGAGCAGCTT

TCCACTTCAGAAAATGCCCTGACAGGGCGTATCCTGGATCTAAAGACTGGAACAGTGAAAAAGGAAGGTC

AGCAGTCTTCCATGAGAATGTGTATGGGCTCAAGGAGATCGTTTATTTGCCGAATGAGGTGTGGCAGTAG

CTCTGTGGACCCAGTTTCTGTGAATAGGCTGAGCTTTGTGAGGAACAGATGCAGGAATGGACTTGGCTCT

GTAAAGGATGGGGAACCTCACTTCGTGGTGGTCCACTGCACAGGCTACATCAAGGCCTGGCCCCCAGCAG

GTGTTTCCCTCCCAGATGATGACCCAGAGGCTGGCCAGGGAAGCAAGTTTTGCCTAGTGGCCATTGGCAG

ATTGCAGGTAACTAGTTCTCCCAACTGTACAGACATGAGTAATGTTTGTCAACCAACAGAGTTCATCTCC

CGACACAACATTGAGGGTATCTTCACTTTTGTGGATCACCGCTGTGTGGCTACTGTTGGCTACCAGCCAC

AGGAACTCTTAGGAAAGAATATTGTAGAATTCTGTCATCCTGAAGACCAGCAGCTTCTAAGAGACAGCTT

CCAACAGGTAGTGAAATTAAAAGGCCAAGTGCTGTCTGTCATGTTCCGGTTCCGGTCTAAGAACCAAGAA

TGGCTCTGGATGAGAACCAGCTCCTTTACTTTCCAGAACCCTTACTCAGATGAAATTGAGTACATCATCT

GTACCAACACCAATGTGAAGAACTCTAGCCAAGAACCACGGCCTACACTCTCCAACACAATCCAGAGGCC

ACAACTAGGTCCCACAGCTAATTTACCCCTGGAGATGGGCTCAGGACAGCTGGCACCCAGGCAGCAGCAA

CAGCAAACAGAATTGGACATGGTACCAGGAAGAGATGGACTGGCCAGCTACAATCATTCCCAGGTGGTTC

AGCCTGTGACAACCACAGGACCAGAACACAGCAAGCCCCTTGAGAAGTCAGATGGTTTATTTGCCCAGGA

TAGAGATCCAAGATTTTCAGAAATCTATCACAACATCAATGCGGATCAGAGTAAAGGCATCTCCTCCAGC

ACTGTCCCTGCCACCCAACAGCTATTCTCCCAGGGCAACACATTCCCTCCTACCCCCCGGCCGGCAGAGA

ATTTCAGGAATAGTGGCCTAGCCCCTCCTGTAACCATTGTCCAGCCATCAGCTTCTGCAGGACAGATGTT

GGCCCAGATTTCCCGCCACTCCAACCCCACCCAAGGAGCAACCCCAACTTGGACCCCTACTACCCGCTCA

GGCTTTTCTGCCCAGGTGGCTACCCAGGCTACTGCTAAGACTCGTACTTCCCAGTTTGGTGTGGGCAGCT

TTCAGACTCCATCCTCCTTCAGCTCCATGTCCCTCCCTGGTGCCCCAACTGCATCGCCTGGTGCTGCTGC

CTACCCTAGTCTCACCAATCGTGGATCTAACTTTGCTCCTGAGACTGGACAGACTGCAGGACAATTCCAG

ACACGGACAGCAGAGGGTGTGGGTGTCTGGCCACAGTGGCAGGGCCAGCAGCCTCATCATCGTTCAAGTT

CTAGTGAGCAACATGTTCAACAACCGCCAGCACAGCAACCTGGCCAGCCTGAGGTCTTCCAGGAGATGCT

GTCCATGCTGGGAGATCAGAGCAACAGCTACAACAATGAAGAATTCCCTGATCTAACTATGTTTCCCCCC

TTTTCAGAATAG

>lcl|NM_000487.6_cds_NP_000478.3_1 [gene=ARSA] [db_xref=CCDS:CCDS14100.2] [protein=arylsulfatase A isoform a precursor] [protein_id=NP_000478.3] [location=374..1903] [gbkey=CDS]

ATGTCCATGGGGGCACCGCGGTCCCTCCTCCTGGCCCTGGCTGCTGGCCTGGCCGTTGCCCGTCCGCCCA

ACATCGTGCTGATCTTTGCCGACGACCTCGGCTATGGGGACCTGGGCTGCTATGGGCACCCCAGCTCTAC

CACTCCCAACCTGGACCAGCTGGCGGCGGGAGGGCTGCGGTTCACAGACTTCTACGTGCCTGTGTCTCTG

TGCACACCCTCTAGGGCCGCCCTCCTGACCGGCCGGCTCCCGGTTCGGATGGGCATGTACCCTGGCGTCC

TGGTGCCCAGCTCCCGGGGGGGCCTGCCCCTGGAGGAGGTGACCGTGGCCGAAGTCCTGGCTGCCCGAGG

CTACCTCACAGGAATGGCCGGCAAGTGGCACCTTGGGGTGGGGCCTGAGGGGGCCTTCCTGCCCCCCCAT

CAGGGCTTCCATCGATTTCTAGGCATCCCGTACTCCCACGACCAGGGCCCCTGCCAGAACCTGACCTGCT

TCCCGCCGGCCACTCCTTGCGACGGTGGCTGTGACCAGGGCCTGGTCCCCATCCCACTGTTGGCCAACCT

GTCCGTGGAGGCGCAGCCCCCCTGGCTGCCCGGACTAGAGGCCCGCTACATGGCTTTCGCCCATGACCTC

ATGGCCGACGCCCAGCGCCAGGATCGCCCCTTCTTCCTGTACTATGCCTCTCACCACACCCACTACCCTC

AGTTCAGTGGGCAGAGCTTTGCAGAGCGTTCAGGCCGCGGGCCATTTGGGGACTCCCTGATGGAGCTGGA

TGCAGCTGTGGGGACCCTGATGACAGCCATAGGGGACCTGGGGCTGCTTGAAGAGACGCTGGTCATCTTC

ACTGCAGACAATGGACCTGAGACCATGCGTATGTCCCGAGGCGGCTGCTCCGGTCTCTTGCGGTGTGGAA

AGGGAACGACCTACGAGGGCGGTGTCCGAGAGCCTGCCTTGGCCTTCTGGCCAGGTCATATCGCTCCCGG

CGTGACCCACGAGCTGGCCAGCTCCCTGGACCTGCTGCCTACCCTGGCAGCCCTGGCTGGGGCCCCACTG

CCCAATGTCACCTTGGATGGCTTTGACCTCAGCCCCCTGCTGCTGGGCACAGGCAAGAGCCCTCGGCAGT

CTCTCTTCTTCTACCCGTCCTACCCAGACGAGGTCCGTGGGGTTTTTGCTGTGCGGACTGGAAAGTACAA

GGCTCACTTCTTCACCCAGGGCTCTGCCCACAGTGATACCACTGCAGACCCTGCCTGCCACGCCTCCAGC

TCTCTGACTGCTCATGAGCCCCCGCTGCTCTATGACCTGTCCAAGGACCCTGGTGAGAACTACAACCTGC

TGGGGGGTGTGGCCGGGGCCACCCCAGAGGTGCTGCAAGCCCTGAAACAGCTTCAGCTGCTCAAGGCCCA

GTTAGACGCAGCTGTGACCTTCGGCCCCAGCCAGGTGGCCCGGGGCGAGGACCCCGCCCTGCAGATCTGC

TGTCATCCTGGCTGCACCCCCCGCCCAGCTTGCTGCCATTGCCCAGATCCCCATGCCTGA

>lcl|NM_000046.5_cds_NP_000037.2_1 [gene=ARSB] [db_xref=CCDS:CCDS4043.1] [protein=arylsulfatase B isoform 1 precursor] [protein_id=NP_000037.2] [location=63..1664] [gbkey=CDS]

ATGGGTCCGCGCGGCGCGGCGAGCTTGCCCCGAGGCCCCGGACCTCGGCGGCTGCTCCTCCCCGTCGTCC

TCCCGCTGCTGCTGCTGCTGTTGTTGGCGCCGCCGGGCTCGGGCGCCGGGGCCAGCCGGCCGCCCCACCT

GGTCTTCTTGCTGGCAGACGACCTAGGCTGGAACGACGTCGGCTTCCACGGCTCCCGCATCCGCACGCCG

CACCTGGACGCGCTGGCGGCCGGCGGGGTGCTCCTGGACAACTACTACACGCAGCCGCTGTGCACGCCGT

CGCGGAGCCAGCTGCTCACTGGCCGCTACCAGATCCGTACAGGTTTACAGCACCAAATAATCTGGCCCTG

TCAGCCCAGCTGTGTTCCTCTGGATGAAAAACTCCTGCCCCAGCTCCTAAAAGAAGCAGGTTATACTACC

CATATGGTCGGAAAATGGCACCTGGGAATGTACCGGAAAGAATGCCTTCCAACCCGCCGAGGATTTGATA

CCTACTTTGGATATCTCCTGGGTAGTGAAGATTATTATTCCCATGAACGCTGTACATTAATTGACGCTCT

GAATGTCACACGATGTGCTCTTGATTTTCGAGATGGCGAAGAAGTTGCAACAGGATATAAAAATATGTAT

TCAACAAACATATTCACCAAAAGGGCTATAGCCCTCATAACTAACCATCCACCAGAGAAGCCTCTGTTTC

TCTACCTTGCTCTCCAGTCTGTGCATGAGCCCCTTCAGGTCCCTGAGGAATACTTGAAGCCATATGACTT

TATCCAAGACAAGAACAGGCATCACTATGCAGGAATGGTGTCCCTTATGGATGAAGCAGTAGGAAATGTC

ACTGCAGCTTTAAAAAGCAGTGGGCTCTGGAACAACACGGTGTTCATCTTTTCTACAGATAACGGAGGGC

AGACTTTGGCAGGGGGTAATAACTGGCCCCTTCGAGGAAGAAAATGGAGCCTGTGGGAAGGAGGCGTCCG

AGGGGTGGGCTTTGTGGCAAGCCCCTTGCTGAAGCAGAAGGGCGTGAAGAACCGGGAGCTCATCCACATC

TCTGACTGGCTGCCAACACTCGTGAAGCTGGCCAGGGGACACACCAATGGCACAAAGCCTCTGGATGGCT

TCGACGTGTGGAAAACCATCAGTGAAGGAAGCCCATCCCCCAGAATTGAGCTGCTGCATAATATTGACCC

GAACTTCGTGGACTCTTCACCGTGTCCCAGGAACAGCATGGCTCCAGCAAAGGATGACTCTTCTCTTCCA

GAATATTCAGCCTTTAACACATCTGTCCATGCTGCAATTAGACATGGAAATTGGAAACTCCTCACGGGCT

ACCCAGGCTGTGGTTACTGGTTCCCTCCACCGTCTCAATACAATGTTTCTGAGATACCCTCATCAGACCC

ACCAACCAAGACCCTCTGGCTCTTTGATATTGATCGGGACCCTGAAGAAAGACATGACCTGTCCAGAGAA

TATCCTCACATCGTCACAAAGCTCCTGTCCCGCCTACAGTTCTACCATAAACACTCAGTCCCCGTGTACT

TCCCTGCACAGGACCCCCGCTGTGATCCCAAGGCCACTGGGGTGTGGGGCCCTTGGATGTAG

>lcl|NM_001675.4_cds_NP_001666.2_1 [gene=ATF4] [db_xref=CCDS:CCDS13996.1] [protein=cyclic AMP-dependent transcription factor ATF-4] [protein_id=NP_001666.2] [location=888..1943] [gbkey=CDS]

ATGACCGAAATGAGCTTCCTGAGCAGCGAGGTGTTGGTGGGGGACTTGATGTCCCCCTTCGACCAGTCGG

GTTTGGGGGCTGAAGAAAGCCTAGGTCTCTTAGATGATTACCTGGAGGTGGCCAAGCACTTCAAACCTCA

TGGGTTCTCCAGCGACAAGGCTAAGGCGGGCTCCTCCGAATGGCTGGCTGTGGATGGGTTGGTCAGTCCC

TCCAACAACAGCAAGGAGGATGCCTTCTCCGGGACAGATTGGATGTTGGAGAAAATGGATTTGAAGGAGT

TCGACTTGGATGCCCTGTTGGGTATAGATGACCTGGAAACCATGCCAGATGACCTTCTGACCACGTTGGA

TGACACTTGTGATCTCTTTGCCCCCCTAGTCCAGGAGACTAATAAGCAGCCCCCCCAGACGGTGAACCCA

ATTGGCCATCTCCCAGAAAGTTTAACAAAACCCGACCAGGTTGCCCCCTTCACCTTCTTACAACCTCTTC

CCCTTTCCCCAGGGGTCCTGTCCTCCACTCCAGATCATTCCTTTAGTTTAGAGCTGGGCAGTGAAGTGGA

TATCACTGAAGGAGATAGGAAGCCAGACTACACTGCTTACGTTGCCATGATCCCTCAGTGCATAAAGGAG

GAAGACACCCCTTCAGATAATGATAGTGGCATCTGTATGAGCCCAGAGTCCTATCTGGGGTCTCCTCAGC

ACAGCCCCTCTACCAGGGGCTCTCCAAATAGGAGCCTCCCATCTCCAGGTGTTCTCTGTGGGTCTGCCCG

TCCCAAACCTTACGATCCTCCTGGAGAGAAGATGGTAGCAGCAAAAGTAAAGGGTGAGAAACTGGATAAG

AAGCTGAAAAAAATGGAGCAAAACAAGACAGCAGCCACTAGGTACCGCCAGAAGAAGAGGGCGGAGCAGG

AGGCTCTTACTGGTGAGTGCAAAGAGCTGGAAAAGAAGAACGAGGCTCTAAAAGAGAGGGCGGATTCCCT

GGCCAAGGAGATCCAGTACCTGAAAGATTTGATAGAAGAGGTCCGCAAGGCAAGGGGGAAGAAAAGGGTC

CCCTAG

>lcl|NM_007348.4_cds_NP_031374.2_1 [gene=ATF6] [db_xref=CCDS:CCDS1235.1] [protein=cyclic AMP-dependent transcription factor ATF-6 alpha] [protein_id=NP_031374.2] [location=42..2054] [gbkey=CDS]

ATGGGGGAGCCGGCTGGGGTTGCCGGCACCATGGAGTCACCTTTTAGCCCGGGACTCTTTCACAGGCTGG

ATGAAGATTGGGATTCTGCTCTCTTTGCTGAACTCGGTTATTTCACAGACACTGATGAGCTGCAATTGGA

AGCAGCAAATGAGACGTATGAAAACAATTTTGATAATCTTGATTTTGATTTGGATTTGATGCCTTGGGAG

TCAGACATTTGGGACATCAACAACCAAATCTGTACAGTTAAAGATATTAAGGCAGAACCTCAGCCACTTT

CTCCAGCCTCCTCAAGTTATTCAGTCTCGTCTCCTCGGTCAGTGGACTCTTATTCTTCAACTCAGCATGT

TCCTGAGGAGTTGGATTTGTCTTCTAGTTCTCAGATGTCTCCCCTTTCCTTATATGGTGAAAACTCTAAT

AGTCTCTCTTCAGCGGAGCCACTGAAGGAAGATAAGCCTGTCACTGGTCCTAGGAACAAGACTGAAAATG

GACTGACTCCAAAGAAAAAAATTCAGGTGAATTCAAAACCTTCAATTCAGCCCAAGCCTTTATTGCTTCC

AGCAGCACCCAAGACTCAAACAAACTCCAGTGTTCCAGCAAAAACCATCATTATTCAGACAGTACCAACG

CTTATGCCATTGGCAAAGCAGCAACCAATTATCAGTTTACAACCTGCACCCACTAAAGGCCAGACGGTTT

TGCTGTCTCAGCCTACTGTGGTACAACTTCAAGCACCTGGAGTTCTGCCCTCTGCTCAGCCAGTCCTTGC

TGTTGCTGGGGGAGTCACACAGCTCCCTAATCACGTGGTGAATGTGGTACCAGCCCCTTCAGCGAATAGC

CCAGTGAATGGAAAACTTTCCGTGACTAAACCTGTCCTACAAAGTACCATGAGAAATGTCGGTTCAGATA

TTGCTGTGCTAAGGAGACAGCAACGTATGATAAAAAATCGAGAATCCGCTTGTCAGTCTCGCAAGAAGAA

GAAAGAATATATGCTAGGGTTAGAGGCGAGATTAAAGGCTGCCCTCTCAGAAAACGAGCAACTGAAGAAA

GAAAATGGAACACTGAAGCGGCAGCTGGATGAAGTTGTGTCAGAGAACCAGAGGCTTAAAGTCCCTAGTC

CAAAGCGAAGAGTTGTCTGTGTGATGATAGTATTGGCATTTATAATACTGAACTATGGACCTATGAGCAT

GTTGGAACAGGATTCCAGGAGAATGAACCCTAGTGTGAGCCCTGCAAATCAAAGGAGGCACCTTCTAGGA

TTTTCTGCTAAAGAGGCACAGGACACATCAGATGGTATTATCCAGAAAAACAGCTACAGATATGATCATT

CTGTTTCAAATGACAAAGCCCTGATGGTGCTAACTGAAGAACCATTGCTTTACATTCCTCCACCTCCTTG

TCAGCCCCTAATTAACACAACAGAGTCTCTCAGGTTAAATCATGAACTTCGAGGATGGGTTCATAGACAT

GAAGTAGAAAGGACCAAGTCAAGAAGAATGACAAATAATCAACAGAAAACCCGTATTCTTCAGGGTGCTC

TGGAACAGGGCTCAAATTCTCAGCTGATGGCTGTTCAATACACAGAAACCACTAGTAGTATCAGCAGGAA

CTCAGGGAGTGAGCTACAAGTGTATTATGCTTCACCCAGAAGTTATCAAGACTTTTTTGAAGCCATCCGC

AGAAGGGGAGACACATTTTATGTTGTGTCATTTCGAAGGGATCACCTGCTGTTACCAGCTACCACCCATA

ACAAGACCACAAGACCAAAAATGTCAATTGTGTTACCAGCAATAAACATAAATGAGAATGTGATCAATGG

GCAGGACTACGAAGTGATGATGCAGATTGACTGTCAGGTGATGGACACCAGGATCCTCCATATCAAAAGT

TCGTCAGTTCCTCCTTACCTCCGAGATCAGCAGAGGAATCAAACCAACACCTTCTTTGGCTCCCCTCCCG

CAGCCACAGAGGCAACCCACGTTGTCAGCACCATCCCTGAGTCATTACAATAG

>lcl|NM_001131028.2_cds_NP_001124500.1_1 [gene=ATG10] [db_xref=CCDS:CCDS4057.1] [protein=ubiquitin-like-conjugating enzyme ATG10] [protein_id=NP_001124500.1] [location=432..1094] [gbkey=CDS]

ATGGAAGAAGATGAGTTCATTGGAGAAAAAACATTCCAACGTTATTGTGCAGAATTCATTAAACATTCAC

AACAGATAGGTGATAGTTGGGAATGGAGACCATCAAAGGACTGTTCTGATGGCTACATGTGCAAAATACA

CTTTCAAATTAAGAATGGGTCTGTGATGTCACATCTAGGAGCATCTACCCATGGACAGACATGTCTTCCC

ATGGAGGAGGCTTTCGAGCTACCCTTGGATGATTGTGAAGTGATTGAAACTGCAGCAGCGTCCGAAGTGA

TTAAATATGAGTATCATGTCTTATATTCCTGTAGCTACCAAGTGCCTGTACTTTACTTTAGGGCAAGCTT

TTTAGATGGGAGACCTTTAACTCTGAAGGACATATGGGAAGGAGTTCATGAGTGCTATAAGATGCGACTG

CTACAGGGACCATGGGACACTATTACGCAACAGGAACATCCAATACTTGGGCAACCCTTTTTTGTACTTC

ATCCCTGCAAGACGAATGAATTCATGACTCCTGTATTAAAGAATTCTCAGAAAATCAATAAGAATGTCAA

CTATATCACATCATGGCTGAGCATTGTAGGGCCAGTTGTTGGGCTGAATCTACCTCTGAGTTATGCCAAA

GCAACGTCTCAGGATGAACGAAATGTCCCTTAA

>lcl|NM_021934.5_cds_NP_068753.2_1 [gene=ATG101] [db_xref=CCDS:CCDS8820.1]
[protein=autophagy-related protein 101] [protein_id=NP_068753.2] [location=472..1128] [gbkey=CDS]

ATGAACTGTCGCTCGGAGGTGCTGGAGGTGTCGGTGGAGGGGCGGCAGGTGGAGGAGGCCATGCTGGCTG

TGCTGCACACGGTGCTTCTGCACCGCAGCACAGGCAAGTTCCACTACAAGAAGGAGGGCACCTACTCCAT

TGGCACCGTGGGCACCCAGGATGTTGACTGTGACTTCATCGACTTCACTTATGTGCGTGTCTCTTCTGAG

GAACTGGATCGTGCCCTGCGCAAGGTTGTTGGGGAGTTCAAGGATGCACTGCGCAACTCTGGTGGCGATG

GGCTGGGGCAGATGTCCTTGGAGTTCTACCAGAAGAAGAAGTCTCGCTGGCCATTCTCAGACGAGTGCAT

CCCATGGGAAGTGTGGACGGTCAAGGTGCATGTGGTAGCCCTGGCCACGGAGCAGGAGCGGCAGATCTGC

CGGGAGAAGGTGGGTGAGAAACTCTGCGAGAAGATCATCAACATCGTGGAGGTGATGAATCGGCATGAGT

ACTTGCCCAAGATGCCCACACAGTCGGAGGTGGATAACGTGTTTGACACAGGCTTGCGGGACGTGCAGCC

CTACCTGTACAAGATCTCCTTCCAGATCACTGATGCCCTGGGCACCTCAGTCACCACCACCATGCGCAGG

CTCATCAAAGACACCCTTGCCCTCTGA

>lcl|NM_004707.4_cds_NP_004698.3_1 [gene=ATG12] [db_xref=CCDS:CCDS4122.2] [protein=ubiquitin-like protein ATG12 isoform 1] [protein_id=NP_004698.3] [location=14..436] [gbkey=CDS]

ATGGCGGAGGAGCCGCAGTCTGTGTTGCAGCTTCCTACTTCAATTGCTGCTGGAGGGGAAGGACTTACGG

ATGTCTCCCCAGAAACAACCACCCCGGAGCCCCCGTCTTCCGCTGCAGTTTCCCCGGGAACAGAGGAACC

TGCTGGCGACACCAAGAAAAAAATTGACATTTTGCTAAAGGCTGTGGGAGACACTCCTATTATGAAAACA

AAGAAGTGGGCAGTAGAGCGAACACGAACCATCCAAGGACTCATTGACTTCATCAAAAAGTTTCTTAAAC

TTGTGGCCTCAGAACAGTTGTTTATTTATGTGAATCAGTCCTTTGCTCCTTCCCCAGACCAAGAAGTTGG

AACTCTCTATGAGTGTTTTGGCAGTGATGGTAAACTGGTTTTACATTACTGCAAGTCTCAGGCGTGGGGA

TGA

>lcl|NM_014741.5_cds_NP_055556.2_1 [gene=ATG13] [db_xref=CCDS:CCDS7921.1] [protein=autophagy-related protein 13 isoform f] [protein_id=NP_055556.2] [location=408..1850] [gbkey=CDS]

ATGGAAACTGATCTCAATTCCCAGGACAGAAAGGACCTGGACAAGTTTATTAAATTTTTTGCCCTCAAGA

CTGTCCAAGTGATTGTCCAGGCTCGGCTTGGTGAAAAGATTTGCACTCGTTCATCATCTTCTCCAACGGG

TTCAGATTGGTTCAACTTAGCAATCAAAGACATCCCAGAGGTTACACATGAAGCAAAGAAGGCACTGGCA

GGACAGCTGCCTGCAGTCGGGAGGTCCATGTGTGTGGAGATTTCACTTAAGACTTCTGAGGGAGATTCCA

TGGAGCTGGAAATATGGTGTCTTGAAATGAATGAAAAGTGTGATAAAGAAATCAAAGTTTCCTACACGGT

GTACAACAGACTGTCATTGCTGCTGAAGTCCCTTCTTGCTATAACTAGGGTGACACCAGCCTATAGGCTC

TCCAGGAAACAAGGGCATGAATATGTCATATTATACAGGATATATTTTGGAGAAGTTCAGCTGAGTGGCT

TAGGAGAAGGCTTCCAGACAGTTCGTGTTGGGACAGTGGGCACCCCTGTGGGCACCATCACTCTTTCTTG

TGCTTACAGAATTAACTTGGCATTCATGTCTACCAGGCAATTTGAGAGGACCCCACCTATCATGGGGATT

ATTATTGATCACTTTGTGGACCGTCCCTATCCCAGCTCCTCTCCCATGCACCCCTGCAATTACAGAACTG

CTGGTGAGGACACTGGAGTAATATACCCGTCTGTAGAAGACTCTCAAGAAGTGTGTACCACCTCTTTTTC

CACCTCCCCACCATCCCAGCTGATGGTTCCTGGGAAGGAAGGTGGGGTACCCCTTGCTCCCAACCAGCCT

GTCCATGGTACCCAGGCTGACCAGGAGAGACTGGCAACCTGCACCCCTTCTGACAGAACCCACTGTGCTG

CCACACCCTCCAGTAGTGAGGATACTGAAACCGTATCAAACAGCAGTGAGGGACGGGCCTCCCCTCACGA

TGTCTTGGAGACCATCTTTGTCCGAAAAGTGGGGGCTTTTGTCAACAAACCCATTAACCAGGTGACCCTG

ACGAGTTTGGATATACCCTTTGCCATGTTTGCTCCCAAGAATTTGGAGCTGGAGGATACCGATCCAATGG

TGAATCCTCCAGATTCCCCAGAGACTGAATCTCCTCTCCAGGGCAGCCTGCACTCAGATGGCTCCAGCGG

GGGCAGCAGTGGCAATACCCATGATGACTTTGTTATGATAGACTTTAAACCAGCTTTTTCTAAAGATGAC

ATTCTTCCGATGGACCTGGGGACCTTCTATCGGGAGTTTCAGAACCCACCTCAGCTGAGCAGCCTCTCCA

TAGATATTGGAGCACAGTCCATGGCTGAAGACTTGGACTCATTACCAGAGAAGCTGGCTGTGCATGAGAA

GAATGTCCGCGAGTTTGATGCCTTTGTGGAAACCCTGCAGTAA

>lcl|NM_014924.5_cds_NP_055739.2_1 [gene=ATG14] [db_xref=CCDS:CCDS32087.1] [protein=beclin 1-associated autophagy-related key regulator] [protein_id=NP_055739.2] [location=9..1487] [gbkey=CDS]

ATGGCGTCTCCCAGTGGGAAGGGAGCCCGGGCGCTGGAGGCTCCTGGCTGCGGGCCCCGGCCGCTCGCCC

GGGACCTGGTGGACTCCGTGGACGATGCGGAGGGGCTGTACGTGGCTGTGGAGCGCTGCCCGCTGTGCAA

CACTACCCGCCGGCGGCTGACCTGCGCCAAATGCGTTCAGAGCGGCGATTTCGTCTACTTCGACGGCCGC

GACCGGGAGAGGTTTATCGACAAGAAGGAAAGGTTAAGCCGACTTAAGAGCAAGCAAGAAGAATTTCAGA

AAGAAGTGTTAAAAGCTATGGAAGGAAAATGGATAACAGATCAGTTGAGATGGAAAATAATGTCCTGCAA

GATGAGGATTGAACAGTTAAAACAAACAATATGTAAAGGAAATGAAGAAATGGAGAAAAATTCTGAAGGC

CTTCTCAAAACCAAGGAAAAGAATCAGAAGCTTTACAGTCGAGCACAACGGCACCAAGAGAAAAAGGAGA

AGATTCAGAGGCATAATCGCAAACTTGGTGACCTGGTAGAAAAAAAGACCATTGACTTAAGAAGTCATTA

TGAGCGTCTGGCAAATCTTCGACGATCCCATATATTAGAGCTCACCTCTGTCATTTTTCCAATCGAGGAA

GTAAAGACGGGTGTGAGAGACCCCGCAGATGTGTCTTCAGAGAGTGACAGTGCCATGACCTCCAGCACTG

TGAGCAAGCTTGCTGAAGCCCGGAGGACAACTTACCTCTCAGGACGATGGGTCTGTGACGATCACAACGG

AGACACCAGCATTAGCATTACAGGGCCTTGGATTAGCCTCCCTAACAATGGGGACTACTCTGCCTACTAC

AGCTGGGTGGAGGAGAAGAAAACAACCCAGGGGCCTGACATGGAGCAGAGTAACCCTGCCTACACCATCA

GTGCTGCGCTGTGCTATGCAACTCAGCTGGTCAACATTCTGTCTCATATACTTGATGTAAATCTTCCCAA

AAAGCTCTGCAACAGTGAATTTTGTGGCGAAAATCTAAGCAAGCAGAAATTTACTCGAGCAGTGAAGAAA

CTGAATGCAAATATTCTTTACCTTTGTTTTTCTCAGCATGTAAATTTAGATCAATTACAACCACTGCATA

CCCTCAGGAATCTAATGTACCTGGTCAGTCCAAGCTCTGAACACCTAGGCAGGTCAGGGCCCTTTGAAGT

ACGAGCAGACCTTGAGGAGTCCATGGAATTTGTGGATCCCGGAGTTGCTGGAGAATCAGATGAGAGCGGA

GATGAGCGCGTCAGCGATGAAGAAACCGACCTGGGCACAGACTGGGAGAACTTGCCTAGTCCCCGGTTTT

GTGATATCCCTTCCCAGTCTGTGGAAGTCTCCCAGAGTCAGAGCACCCAGGCGTCCCCACCCATCGCGAG

CAGCAGTGCAGGTGGGATGATCTCCTCTGCAGCAGCCTCGGTGACCTCCTGGTTTAAAGCTTACACTGGA

CACCGTTAA

>lcl|NM_017974.4_cds_NP_060444.3_1 [gene=ATG16L1] [db_xref=CCDS:CCDS2502.2] [protein=autophagy-related protein 16-1 isoform 2] [protein_id=NP_060444.3] [location=156..1922] [gbkey=CDS]

ATGTCGTCGGGCCTCCGCGCCGCTGACTTCCCCCGCTGGAAGCGCCACATCTCGGAGCAACTGAGGCGCC

GGGACCGGCTGCAGAGACAGGCGTTCGAGGAGATCATCCTGCAGTATAACAAATTGCTGGAAAAGTCAGA

TCTTCATTCAGTGTTGGCCCAGAAACTACAGGCTGAAAAGCATGACGTACCAAACAGGCACGAGATAAGT

CCCGGACATGATGGCACATGGAATGACAATCAGCTACAAGAAATGGCCCAACTGAGGATTAAGCACCAAG

AGGAACTGACTGAATTACACAAGAAACGTGGGGAGTTAGCTCAACTGGTGATTGACCTGAATAACCAAAT

GCAGCGGAAGGACAGGGAGATGCAGATGAATGAAGCAAAAATTGCAGAATGTTTGCAGACTATCTCTGAC

CTGGAGACGGAGTGCCTAGACCTGCGCACTAAGCTTTGTGACCTTGAAAGAGCCAACCAGACCCTGAAGG

ATGAATATGATGCCCTGCAGATCACTTTTACTGCCTTGGAGGGAAAACTGAGGAAAACTACGGAAGAGAA

CCAGGAGCTGGTCACCAGATGGATGGCTGAGAAAGCCCAGGAAGCCAATCGGCTTAATGCAGAGAATGAA

AAAGACTCCAGGAGGCGGCAAGCCCGGCTGCAGAAAGAGCTTGCAGAAGCAGCAAAGGAACCTCTACCAG

TCGAACAGGATGATGACATTGAGGTCATTGTGGATGAAACTTCTGATCACACAGAAGAGACCTCTCCTGT

GCGAGCCATCAGCAGAGCAGCCACGAGACGCTCTGTCTCTTCCTTCCCAGTCCCCCAGGACAATGTGGAT

ACTCATCCTGGTTCTGGTAAAGAAGTGAGGGTACCAGCTACTGCCTTGTGTGTCTTCGATGCACATGATG

GGGAAGTCAACGCTGTGCAGTTCAGTCCAGGTTCCCGGTTACTGGCCACTGGAGGCATGGACCGCAGGGT

TAAGCTTTGGGAAGTATTTGGAGAAAAATGTGAGTTCAAGGGTTCCCTATCTGGCAGTAATGCAGGAATT

ACAAGCATTGAATTTGATAGTGCTGGATCTTACCTCTTAGCAGCTTCAAATGATTTTGCAAGCCGAATCT

GGACTGTGGATGATTATCGATTACGGCACACACTCACGGGACACAGTGGGAAAGTGCTGTCTGCTAAGTT

CCTGCTGGACAATGCGCGGATTGTCTCAGGAAGTCACGACCGGACTCTCAAACTCTGGGATCTACGCAGC

AAAGTCTGCATAAAGACAGTGTTTGCAGGATCCAGTTGCAATGATATTGTCTGCACAGAGCAATGTGTAA

TGAGTGGACATTTTGACAAGAAAATTCGTTTCTGGGACATTCGATCAGAGAGCATAGTTCGAGAGATGGA

GCTGTTGGGAAAGATTACTGCCCTGGACTTAAACCCAGAAAGGACTGAGCTCCTGAGCTGCTCCCGTGAT

GACTTGCTAAAAGTTATTGATCTCCGAACAAATGCTATCAAGCAGACATTCAGTGCACCTGGGTTCAAGT

GCGGCTCTGACTGGACCAGAGTTGTCTTCAGCCCTGATGGCAGTTACGTGGCGGCAGGCTCTGCTGAGGG

CTCTCTGTATATCTGGAGTGTGCTCACAGGGAAAGTGGAAAAGGTTCTTTCAAAGCAGCACAGCTCATCC

ATCAATGCGGTGGCGTGGTCGCCCTCTGGCTCGCACGTTGTCAGTGTGGACAAAGGATGCAAAGCTGTGC

TGTGGGCACAGTACTGA

>lcl|NM_033388.2_cds_NP_203746.1_1 [gene=ATG16L2] [db_xref=CCDS:CCDS31634.1] [protein=protein Atg16l2 isoform 1] [protein_id=NP_203746.1] [location=36..1895] [gbkey=CDS]

ATGGCGGGGCCGGGCGTCCCCGGTGCCCCCGCAGCGCGCTGGAAACGCCACATCGTGCGGCAGCTGCGGC

TTCGGGACCGTACGCAAAAGGCGCTTTTCCTGGAGCTGGTGCCGGCCTATAACCATCTCTTAGAGAAGGC

TGAGCTGCTGGACAAGTTCTCAAAGAAGCTGCAGCCGGAGCCAAACAGTGTCACTCCCACCACCCACCAG

GGCCCCTGGGAGGAGTCAGAGCTTGACTCAGACCAAGTCCCATCACTGGTCGCACTGAGGGTGAAGTGGC

AGGAGGAGGAGGAGGGGCTCCGGCTGGTCTGTGGTGAGATGGCCTACCAGGTGGTGGAGAAGGGCGCGGC

CCTGGGCACGCTGGAGTCGGAGCTGCAGCAGAGGCAAAGCAGGCTGGCAGCCCTGGAGGCCCGCGTGGCG

CAGCTGCGAGAGGCGCGGGCGCAGCAGGCCCAGCAGGTGGAGGAGTGGCGGGCGCAGAATGCGGTGCAGC

GGGCAGCCTACGAGGCGCTGCGCGCGCACGTCGGGCTCCGGGAGGCGGCACTGCGCAGGCTCCAGGAAGA

GGCGCGCGACCTGCTGGAGAGGCTCGTGCAGCGCAAGGCGCGCGCCGCGGCCGAGCGCAACCTGCGCAAC

GAGCGCCGGGAGCGGGCCAAGCAGGCGCGGGTGTCCCAGGAGCTGAAGAAGGCTGCCAAGCGGACCGTGA

GCATCAGCGAGGGCCCGGACACCCTAGGCGATGGGATGAGGGAGAGAAGGGAGACTCTGGCTCTGGCCCC

TGAGCCAGAGCCCCTGGAGAAGGAAGCTTGTGAGAAGTGGAAGAGGCCCTTCAGGTCTGCCTCAGCCACC

TCCCTGACGCTGTCCCACTGTGTGGATGTGGTGAAGGGGCTTCTGGATTTTAAGAAGAGGAGAGGTCACT

CAATTGGGGGAGCCCCTGAGCAGCGATACCAGATCATCCCTGTGTGTGTGGCTGCCCGACTTCCTACCCG

GGCTCAGGATGTGCTGGATGCCCACCTCTCTGAGGTCAATGCTGTTCGTTTTGGCCCCAACAGCAGCCTC

CTGGCCACTGGAGGGGCTGACCGCCTGATCCACCTCTGGAATGTTGTGGGAAGTCGCCTGGAGGCCAACC

AGACCCTGGAGGGAGCTGGTGGCAGCATCACCAGTGTGGACTTTGACCCCTCGGGCTACCAGGTTTTAGC

AGCAACTTACAACCAGGCTGCCCAGCTCTGGAAGGTGGGGGAGGCACAGTCCAAGGAGACACTGTCTGGA

CACAAGGATAAGGTGACAGCTGCCAAATTCAAGCTAACGAGGCACCAGGCAGTGACTGGGAGCCGCGACC

GGACAGTGAAGGAGTGGGACCTCGGCCGTGCCTATTGCTCCAGGACCATCAATGTCCTTTCCTACTGTAA

TGACGTGGTGTGTGGGGACCATATCATCATTAGTGGCCACAATGACCAGAAGATCCGGTTCTGGGACAGC

AGGGGGCCCCACTGCACCCAGGTCATCCCTGTGCAGGGCCGGGTCACCTCCCTGAGCCTCAGCCACGACC

AACTGCACCTGCTCAGCTGTTCCCGAGACAACACACTCAAGGTCATCGACCTGCGTGTCAGCAACATCCG

CCAGGTGTTCAGGGCCGATGGCTTCAAGTGTGGTTCTGACTGGACCAAAGCTGTGTTCAGCCCGGACAGA

AGCTATGCACTGGCAGGCTCCTGTGATGGGGCCCTTTACATCTGGGATGTGGACACCGGGAAACTGGAGA

GCAGACTACAGGGACCCCATTGCGCTGCCGTCAACGCCGTGGCCTGGTGCTACTCCGGGAGCCACATGGT

GAGCGTGGACCAGGGCAGGAAGGTTGTGCTCTGGCAGTAG

>lcl|NM_015104.3_cds_NP_055919.2_1 [gene=ATG2A] [db_xref=CCDS:CCDS31602.1] [protein=autophagy-related protein 2 homolog A isoform 1] [protein_id=NP_055919.2] [location=75..5891] [gbkey=CDS]

ATGTCACGATGGCTGTGGCCATGGTCAAACTGTGTGAAAGAGCGGGTCTGCCGCTACTTGCTGCACCACT

ACTTAGGTCACTTCTTCCAAGAGCACCTCAGCCTGGACCAGCTCAGCCTCGATCTGTACAAGGGCAGCGT

TGCCCTGCGAGACATCCACCTGGAAATCTGGTCTGTGAACGAGGTGCTGGAGTCAATGGAGTCACCGCTG

GAGCTGGTGGAAGGCTTCGTGGGCTCCATCGAGGTGGCCGTGCCCTGGGCTGCTCTGCTCACCGACCACT

GCACAGTGCGCGTGTCCGGCCTCCAGCTCACCTTGCAGCCCCGCCGGGGTCCAGCGCCAGGGGCTGCCGA

CTCACAGAGCTGGGCCTCATGCATGACCACAAGCCTGCAGCTGGCCCAGGAGTGTCTGCGGGATGGGCTA

CCGGAGCCCTCTGAGCCACCACAGCCCCTGGAGGGGCTGGAGATGTTTGCCCAGACCATTGAGACTGTGC

TTCGGAGGATCAAAGTGACCTTCCTGGACACTGTCGTGAGGGTGGAGCACTCTCCGGGTGATGGGGAACG

TGGTGTGGCCGTCGAGGTCCGTGTGCAGAGACTGGAGTACTGTGATGAGGCAGTGCGGGACCCAAGCCAG

GCGCCGCCGGTGGACGTGCATCAGCCGCCTGCCTTCCTGCACAAGCTGCTGCAGCTGGCAGGGGTCCGCC

TGCACTACGAGGAGCTCCCGGCACAGGAAGAGCCTCCAGAGCCCCCCTTGCAGATCGGCAGCTGCTCAGG

GTACATGGAGCTGATGGTGAAGTTGAAGCAAAATGAGGCCTTCCCTGGCCCCAAGTTGGAGGTGGCGGGA

CAGCTGGGCTCCCTGCACCTGCTCCTGACCCCGAGGCAGCTCCAGCAACTTCAGGAACTGCTCAGCGCCG

TGAGCCTTACAGACCACGAGGGCCTGGCTGACAAGCTGAACAAGAGCCGCCCGCTAGGTGCCGAAGACCT

GTGGCTGATTGAGCAGGACCTGAACCAGCAGCTGCAGGCAGGGGCAGTGGCTGAGCCCCTCAGCCCAGAC

CCCCTTACCAACCCCCTTCTCAACCTGGATAACACTGACCTCTTCTTCTCCATGGCTGGCCTCACAAGCA

GTGTGGCCTCAGCCCTCTCTGAGCTCTCCCTCTCCGATGTAGACCTGGCCTCCTCTGTGCGCAGCGACAT

GGCCTCCCGCCGGCTCTCTGCCCAGGCCCACCCAGCTGGCAAGATGGCCCCCAACCCCCTCCTGGACACC

ATGCGCCCTGACTCGCTGCTGAAGATGACCTTGGGGGGTGTGACCCTGACCTTGCTTCAGACGTCTGCCC

CATCTTCCGGACCACCTGACCTCGCCACGCACTTTTTCACCGAGTTTGATGCCACCAAGGATGGGCCCTT

CGGTTCCCGAGACTTCCATCACCTTCGACCACGCTTCCAGAGGGCCTGTCCCTGTAGCCATGTTCGGCTA

ACGGGCACAGCCGTGCAGCTGTCCTGGGAGCTGCGGACGGGCAGTCGGGGCCGGCGGACAACCAGCATGG

AAGTGCACTTCGGGCAGCTGGAGGTGCTGGAGTGTCTGTGGCCCCGGGGCACCTCTGAGCCTGAGTACAC

GGAGATCCTGACCTTTCCTGGTACGCTGGGCTCCCAGGCCTCAGCTCGGCCCTGCGCCCATCTGCGCCAC

ACACAGATCCTGCGCCGTGTGCCTAAGAGCCGACCCCGGCGCTCAGTTGCCTGCCATTGCCACTCAGAAC

TGGCCCTGGACCTGGCCAACTTCCAGGCGGACGTGGAGCTGGGGGCCCTGGACCGGCTGGCCGCCCTACT

GCGCCTGGCCACCGTACCTGCTGAGCCTCCAGCCGGCCTGCTGACAGAGCCCCTGCCGGCGATGGAGCAG

CAGACGGTATTTCGGCTCTCTGCACCCCGGGCCACGCTGCGGCTGCGCTTCCCCATTGCCGACCTGCGGC

CTGAGCCGGACCCCTGGGCGGGCCAGGCCGTGCGGGCTGAGCAGCTTCGGCTGGAGCTGAGTGAGCCCCA

GTTCCGGTCAGAGCTTAGCAGTGGGCCTGGTCCCCCAGTCCCCACCCACCTGGAACTCACCTGCTCCGAC

CTACATGGTATCTATGAAGATGGAGGGAAGCCACCTGTCCCTTGCCTGCGTGTCTCCAAAGCCCTGGACC

CCAAGAGCACTGGGCGCAAGTACTTCCTGCCCCAGGTAGTGGTGACTGTGAACCCCCAGTCCAGCAGCAC

ACAGTGGGAGGTGGCCCCGGAGAAGGGAGAGGAACTGGAGCTGTCAGTGGAGAGTCCCTGTGAGCTGCGG

GAACCTGAGCCCTCGCCCTTCTCCTCTAAGAGGACCATGTATGAGACAGAGGAGATGGTGATCCCTGGAG

ACCCTGAGGAGATGAGGACGTTCCAGAGCCGGACCCTGGCACTGTCCCGCTGCAGCCTGGAAGTGATCCT

GCCCAGTGTCCACATCTTTCTGCCCAGCAAGGAGGTCTACGAGAGCATCTACAACAGGATCAACAACGAC

CTGCTCATGTGGGAGCCTGCAGATCTGCTTCCCACCCCCGACCCCGCCGCCCAGCCCTCGGGCTTCCCCG

GCCCCTCAGGCTTCTGGCACGACAGCTTTAAGATGTGCAAGTCAGCCTTCAAGCTGGCCAACTGCTTTGA

TCTCACCCCAGACTCGGACTCGGATGACGAGGATGCCCACTTCTTCTCAGTGGGGGCATCAGGTGGCCCA

CAGGCCGCTGCCCCTGAGGCCCCAAGTCTTCACTTGCAGAGCACCTTCTCTACACTGGTGACAGTGCTGA

AGGGGCGGATCACAGCCCTCTGTGAGACCAAGGATGAGGGTGGGAAGCGGCTGGAGGCTGTGCACGGGGA

GCTGGTGCTGGACATGGAGCACGGTACCCTCTTCAGCGTCTCCCAGTACTGTGGCCAGCCAGGACTTGGC

TACTTCTGTCTGGAAGCTGAAAAGGCAACACTCTACCACCGAGCGGCCGTGGATGACTACCCGCTGCCCA

GTCACCTGGACCTTCCCAGTTTCGCTCCCCCGGCTCAGCTGGCCCCAACCATCTACCCATCGGAGGAAGG

GGTGACCGAGCGGGGAGCCTCGGGCCGCAAGGGCCAGGGCCGGGGACCCCACATGTTGTCCACTGCTGTG

CGCATCCACCTGGACCCCCACAAGAATGTGAAGGAGTTCCTGGTGACACTGCGGTTGCACAAAGCCACCT

TGCGCCACTACATGGCCCTGCCCGAGCAGAGCTGGCATTCCCAGTTGTTGGAGTTCCTAGACGTGCTGGA

TGACCCTGTGCTGGGCTACCTGCCCCCGACGGTCATCACCATCCTGCACACACACCTGTTCTCCTGCTCT

GTGGACTATAGGCCACTCTACCTCCCAGTGCGTGTCCTCATCACCGCGGAGACCTTCACTCTCTCCAGCA

ACATCATCATGGACACCTCCACCTTCCTGCTCAGGTTCATCCTCGATGACTCCGCCTTGTACCTGTCCGA

CAAGTGTGAGGTGGAGACCCTGGACCTGCGGCGAGATTATGTCTGTGTTTTGGATGTTGACCTCTTGGAA

CTTGTGATTAAAACCTGGAAAGGGAGCACCGAGGGCAAACTGAGCCAGCCACTATTCGAGCTGCGCTGCT

CCAACAATGTGGTACACGTGCACAGCTGTGCCGACTCCTGTGCCCTGCTGGTCAACCTGCTCCAGTACGT

AATGAGCACAGGCGATCTGCACCCCCCACCCCGGCCCCCCAGCCCCACGGAGATCGCCGGCCAGAAGCTC

TCGGAGAGTCCTGCCTCTCTGCCCTCGTGCCCCCCAGTGGAGACGGCCCTCATCAACCAGCGTGACCTGG

CCGACGCCCTCCTGGACACCGAGCGCAGCCTACGGGAGCTGGCCCAGCCTTCAGGTGGCCACCTCCCTCA

GGCGTCGCCCATCTCCGTCTACCTATTCCCAGGTGAACGGAGTGGGGCCCCACCCCCTTCACCACCTGTC

GGGGGCCCTGCTGGCAGCTTAGGGTCATGCTCAGAGGAGAAGGAAGATGAAAGGGAAGAGGAGGGCGATG

GAGACACCCTGGACAGTGATGAGTTCTGCATCCTTGATGCTCCCGGCCTGGGCATCCCGCCCCGAGATGG

GGAGCCTGTGGTGACACAGCTGCATCCCGGCCCCATCGTTGTGAGGGACGGTTACTTCTCACGGCCGATC

GGCAGCACGGACTTGCTGCGGGCACCTGCCCATTTCCCAGTGCCCAGCACTCGGGTGGTGCTACGTGAGG

TCTCCCTCGTCTGGCACCTCTATGGGGGCCGAGACTTTGGCCCCCACCCCGGCCACAGGGCAAGAACTGG

CCTCTCAGGTCCCAGGAGCTCCCCTTCCCGCTGCTCTGGCCCCAACCGGCCCCAGAACTCATGGCGCACG

CAGGGGGGCAGCGGGCGGCAGCACCATGTCCTCATGGAGATCCAGCTGAGCAAGGTAAGCTTCCAGCACG

AGGTGTACCCAGCGGAGCCAGCCACAGGCCCTGCGGCCCCCAGCCAGGAGCTGGAGGAGCGACCGCTGTC

CCGTCAGGTGTTCATCGTGCAGGAGCTGGAGGTCCGAGACCGGCTCGCCTCCTCCCAGATCAACAAGTTC

CTGTACCTACACACGAGTGAGCGGATGCCGCGACGTGCCCACTCTAACATGCTCACCATCAAAGCGCTGC

ATGTGGCCCCCACTACCAACCTGGGTGGGCCTGAGTGCTGTCTCCGCGTCTCGCTGATGCCCCTGCGGCT

CAATGTGGACCAGGATGCCCTCTTCTTCCTCAAGGACTTCTTCACTAGTCTGGTGGCCGGCATCAACCCC

GTGGTCCCAGGGGAGACCTCCGCTGAGGCTCGCCCCGAGACTCGAGCCCAGCCCAGCAGCCCCCTGGAAG

GGCAGGCCGAAGGCGTAGAGACCACTGGTTCGCAGGAGGCCCCAGGAGGTGGACACAGCCCCTCCCCTCC

TGACCAGCAGCCCATCTACTTCAGAGAGTTCCGCTTCACGTCTGAGGTCCCCATCTGGCTGGATTACCAT

GGCAAGCACGTCACGATGGACCAGGTGGGCACTTTTGCTGGCCTCCTCATCGGCCTGGCCCAACTCAACT

GCTCCGAGCTGAAGCTAAAGCGGCTCTGTTGCAGGCACGGGCTCCTGGGTGTGGACAAGGTGCTGGGCTA

TGCCCTCAACGAGTGGCTGCAGGACATCCGCAAGAACCAGCTGCCCGGCCTGCTGGGAGGCGTGGGCCCC

ATGCACTCGGTTGTCCAGCTCTTCCAAGGGTTCCGGGACCTGCTGTGGCTGCCCATTGAGCAGTACAGGA

AGGATGGCCGCCTCATGCGGGGGCTGCAGCGAGGGGCTGCCTCCTTTGGCTCATCCACAGCCTCTGCCGC

CCTGGAACTCAGCAACCGGTTGGTACAGGCTATCCAGGCCACAGCTGAGACCGTGTATGACATCCTGTCC

CCGGCAGCCCCCGTCTCCCGCTCCCTGCAGGATAAGCGCTCTGCGCGGAGGCTGCGCAGGGGCCAGCAGC

CTGCCGACCTGCGGGAGGGTGTGGCCAAGGCCTACGACACAGTGCGAGAGGGCATCTTGGATACAGCTCA

GACCATCTGTGACGTGGCATCGCGGGGCCATGAGCAGAAGGGGCTGACGGGCGCCGTGGGGGGCGTGATC

CGCCAGCTGCCCCCGACTGTGGTGAAGCCGCTCATCCTGGCCACGGAGGCCACGTCCAGCCTGCTCGGGG

GCATGCGCAACCAGATTGTCCCCGACGCCCACAAGGACCACGCCCTCAAGTGGCGCTCGGACAGTGCCCA

AGACTGA

>lcl|NM_018036.7_cds_NP_060506.6_1 [gene=ATG2B] [db_xref=CCDS:CCDS9944.2] [protein=autophagy-related protein 2 homolog B] [protein_id=NP_060506.6] [location=366..6602] [gbkey=CDS]

ATGCCTTGGCCGTTTTCGGAGTCCATCAAGAAGAGGGCCTGCCGGTACCTCCTGCAGAGGTACCTGGGCC

ACTTTCTGCAGGAGAAGCTGAGCCTGGAGCAGCTCAGCCTGGACCTGTACCAAGGCACCGGGTCCCTCGC

CCAGGTCCCCTTGGACAAATGGTGTCTCAATGAGATCTTGGAGTCAGCAGATGCACCCTTAGAAGTCACT

GAAGGATTCATTCAGTCAATTTCCCTGTCAGTTCCATGGGGCTCTTTACTGCAGGATAATTGTGCACTGG

AAGTGAGAGGATTAGAAATGGTCTTCCGGCCTAGACCTCGCCCAGCAACTGGTTCTGAGCCTATGTATTG

GTCAAGTTTTATGACCAGCAGTATGCAATTGGCAAAAGAATGTCTTAGCCAGAAACTAACAGATGAACAA

GGAGAAGGATCCCAGCCTTTTGAAGGACTTGAAAAGTTTGCTGAAACCATTGAAACAGTACTAAGAAGAG

TAAAAGTCACTTTTATAGATACTGTTTTGAGAATTGAACATGTGCCAGAAAATTCCAAAACTGGAACTGC

ACTTGAAATTCGAATAGAAAGAACTGTGTACTGTGATGAAACTGCTGACGAATCCTCAGGAATTAATGTG

CATCAACCCACTGCTTTTGCTCACAAGTTACTTCAGCTCTCTGGAGTGTCTCTCTTCTGGGATGAGTTTT

CTGCATCAGCCAAATCTTCCCCAGTGTGTTCAACTGCACCAGTGGAAACTGAGCCAAAGCTCTCACCTAG

CTGGAACCCCAAAATTATTTATGAGCCACACCCACAGCTAACTAGAAATTTACCAGAGATAGCACCTTCT

GACCCAGTGCAGATTGGACGGTTAATTGGTAGGTTGGAGTTGAGTCTCACGTTGAAACAGAATGAAGTGC

TTCCTGGAGCTAAGTTGGATGTTGATGGACAGATAGACTCTATTCATCTACTCCTGTCACCAAGACAGGT

GCACTTGCTTTTGGATATGTTGGCAGCTATTGCTGGACCAGAAAATTCTAGCAAAATAGGGTTAGCTAAT

AAAGATAGGAAAAATCGACCCATGCAGCAGGAAGACGAGTATCGAATTCAGATGGAATTAAACCGGTATT

ATTTGAGAAAAGATTCCCTCTCTGTGGGTGTATCTTCAGAGCAAAGCTTTTATGAGACAGAAACAGCTCG

TACACCTTCTAGCCGTGAAGAAGAAGTATTCTTCTCCATGGCTGATATGGACATGTCTCATAGTCTCTCT

TCTCTTCCACCCCTTGGGGACCCCCCAAACATGGACCTTGAGTTATCATTAACTAGTACATATACAAATA

CCCCAGCAGGATCTCCATTAAGTGCTACTGTGCTTCAGCCCACTTGGGGAGAGTTCCTTGATCATCATAA

AGAACAGCCAGTAAGAGGGTCAACATTTCCATCCAACCTAGTTCACCCAACACCTTTACAGAAGACATCT

CTCCCATCTAGATCTGTTTCAGTGGATGAATCAAGGCCTGAACTTATTTTTAGACTAGCTGTGGGAACTT

TTTCAATCTCTGTGCTTCACATTGATCCTTTATCTCCACCTGAAACGTCACAGAACCTTAATCCATTGAC

ACCTATGGCAGTAGCTTTCTTTACTTGTATAGAAAAGATTGATCCAGCAAGATTTTCAACAGAAGATTTT

AAGTCTTTCCGAGCAGTGTTTGCAGAAGCTTGCTCACACGATCACCTTAGATTTATAGGTACTGGCATTA

AAGTATCCTATGAACAAAGACAAAGATCAGCTTCCCGATATTTTAGTACTGATATGTCCATTGGGCAAAT

GGAATTTTTGGAGTGCCTATTTCCAACTGATTTTCATTCTGTTCCTCCTCACTATACAGAGCTTTTAACG

TTCCATTCCAAAGAAGAAACTGGTTCCCATTCCCCTGTGTGTCTTCAGCTTCATTATAAGCATTCTGAGA

ATAGAGGGCCCCAGGGTAATCAAGCAAGACTTAGTTCAGTTCCTCACAAGGCAGAATTGCAAATTAAATT

AAATCCAGTGTGTTGTGAGCTGGATATCAGTATTGTGGACAGGTTAAATTCCTTGCTTCAACCACAGAAA

CTTGCCACAGTAGAGATGATGGCATCCCACATGTATACTTCATATAATAAACATATTAGTCTGCACAAGG

CTTTCACTGAAGTGTTTCTAGATGATTCACATAGTCCTGCAAATTGTCGGATATCAGTACAAGTTGCCAC

ACCAGCATTAAACCTTTCTGTTCGCTTCCCAATACCTGATCTTCGATCTGATCAAGAAAGAGGACCATGG

TTTAAGAAGTCACTTCAGAAGGAGATCCTTTATTTAGCCTTCACAGATCTAGAATTTAAGACTGAATTTA

TAGGAGGATCAACCCCAGAACAAATTAAATTGGAACTTACCTTTAGAGAACTAATTGGATCGTTCCAGGA

AGAGAAAGGAGATCCATCTATTAAGTTTTTCCATGTGTCTAGTGGAGTAGATGGAGATACAACATCGTCA

GATGACTTTGACTGGCCACGAATTGTACTGAAAATAAATCCACCAGCCATGCATTCCATTTTGGAGAGAA

TTGCAGCTGAAGAAGAAGAGGAGAATGATGGTCACTACCAGGAGGAAGAGGAAGGAGGTGCTCATTCCTT

GAAAGATGTTTGTGATCTAAGAAGACCAGCCCCATCTCCTTTTTCTTCTCGTAGAGTAATGTTTGAAAAT

GAACAGATGGTGATGCCAGGAGACCCTGTAGAAATGACAGAATTTCAGGATAAAGCAATCAGCAATTCTC

ACTATGTGCTGGAACTTACGTTACCAAATATTTATGTAACACTACCTAATAAGAGCTTTTATGAGAAGCT

TTATAATAGGATCTTTAATGACTTGCTACTGTGGGAACCAACAGCTCCTTCACCAGTGGAGACATTCGAG

AATATTTCCTATGGCATTGGGCTTTCAGTAGCCAGTCAGCTCATTAATACTTTCAACAAAGATAGTTTTA

GTGCATTTAAATCTGCAGTTCACTATGATGAGGAAAGTGGATCTGAGGAGGAGACTTTGCAGTATTTTTC

CACTGTTGATCCCAACTATCGTTCTCGCAGGAAAAAAAAATTAGACTCTCAGAACAAGAACTCTCAGAGT

TTTCTCTCAGTTCTTCTGAATATTAATCATGGATTAATAGCAGTGTTCACAGATGTGAAGCAAGATAATG

GAGATCTGTTGGAAAACAAGCATGGTGAATTCTGGTTAGAGTTCAATAGTGGTTCATTATTTTGTGTGAC

AAAATATGAAGGTTTTGATGACAAGCACTACATTTGCCTTCATTCTAGCAGTTTCAGTCTGTACCATAAA

GGCATAGTGAATGGAGTGATTCTCCCGACAGAAACACGACTTCCCAGCTCAACCCGCCCACACTGGTTGG

AACCTACTATTTATTCCTCTGAAGAAGATGGCCTCAGTAAAACTTCTTCAGATGGAGTTGGAGGAGACAG

TTTGAATATGCTGTCTGTTGCCGTTAAAATATTGTCTGATAAATCAGAGTCCAATACAAAGGAATTTCTC

ATTGCCGTAGGACTGAAAGGAGCCACTCTCCAGCATAGAATGCTTCCTTCTGGGCTTAGCTGGCATGAGC

AGATTTTATACTTCTTGAATATTGCTGATGAACCTGTTTTGGGATATAATCCTCCAACTTCATTTACAAC

TTTTCATGTTCATCTTTGGAGCTGTGCACTTGATTATAGACCCCTTTATTTGCCAATCCGATCTCTTCTT

ACCGTGGAAACATTCAGTGTTTCCAGTAGCGTTGCATTGGATAAATCTTCCTCTACTCTCAGAATAATCT

TGGATGAAGCTGCTTTACATCTATCTGACAAATGCAATACTGTCACTATAAATCTGAGTAGAGATTATGT

TCGTGTGATGGATATGGGGCTTTTGGAGTTAACCATAACTGCAGTGAAGTCTGATTCTGATGGAGAGCAA

ACTGAGCCCCGCTTTGAGTTACACTGTTCCAGCGATGTTGTCCATATCAGAACGTGCTCAGACTCTTGTG

CTGCGTTAATGAATCTCATTCAGTACATTGCAAGCTATGGTGACTTGCAGACACCTAACAAGGCAGATAT

GAAGCCTGGAGCCTTTCAAAGAAGGTCTAAGGTAGATTCCAGTGGTCGATCATCCTCACGTGGTCCAGTA

CTTCCTGAAGCAGATCAACAAATGTTACGAGATCTGATGAGTGATGCTATGGAGGAGATCGACATGCAAC

AAGGCACCTCGTCAGTAAAACCACAGGCTAATGGTGTTTTGGATGAAAAATCTCAAATTCAGGAGCCATG

TTGTTCAGACCTCTTCCTGTTTCCTGACGAGAGTGGGAATGTATCCCAGGAGTCCGGCCCCACCTATGCC

TCATTCTCTCACCATTTCATCAGTGATGCAATGACAGGTGTGCCCACTGAGAATGATGACTTTTGCATTC

TTTTTGCACCAAAAGCAGCCATGCAGGAGAAGGAAGAAGAACCAGTTATAAAAATCATGGTTGATGATGC

AATTGTGATAAGAGACAATTATTTCAGTCTGCCCGTTAATAAGACCGATACGAGCAAAGCCCCCTTACAC

TTTCCCATTCCTGTGATTCGCTATGTGGTGAAGGAGGTCTCTCTTGTCTGGCATCTTTATGGAGGAAAGG

ATTTTGGAATAGTCCCTCCCACTTCTCCGGCTAAAAGTTATATTAGTCCCCACAGTTCGCCTTCTCACAC

ACCCACGAGACATGGACGTAATACAGTATGTGGGGGAAAAGGAAGGAACCATGACTTTTTAATGGAAATA

CAGCTAAGCAAGGTGAAGTTTCAGCATGAAGTCTACCCGCCATGCAAACCTGATTGTGATTCCAGCCTCT

CAGAACACCCAGTCTCCCGGCAGGTGTTCATTGTTCAGGATCTTGAGATTCGAGATCGTTTGGCAACATC

ACAAATGAATAAATTTTTATACCTGTATTGCAGTAAAGAAATGCCTCGAAAAGCTCACTCCAACATGTTG

ACAGTGAAAGCCTTACACGTGTGTCCAGAATCTGGCAGGTCCCCACAGGAGTGCTGCTTGAGAGTGTCGC

TGATGCCGCTCCGCCTCAATATTGACCAGGATGCTTTGTTCTTCCTGAAGGATTTCTTCACAAGTCTTTC

TGCAGAAGTAGAGCTTCAAATGACTCCAGATCCAGAAGTTAAAAAGTCTCCTGGAGCTGATGTCACCTGC

AGTTTGCCAAGGCATTTGAGTACCTCAAAGGAGCCAAATCTGGTTATTTCTTTCTCTGGGCCAAAACAGC

CTTCCCAAAATGATAGTGCCAATTCAGTGGAAGTGGTTAATGGCATGGAAGAGAAGAACTTCTCTGCTGA

AGAAGCATCTTTTAGGGATCAGCCTGTGTTTTTTAGAGAATTTAGATTCACGTCAGAAGTTCCCATTCGA

CTTGATTATCATGGCAAACATGTATCAATGGATCAGGGTACGCTAGCTGGGATTTTGATTGGTCTGGCTC

AGTTAAACTGCTCTGAACTAAAGCTCAAGAGGCTTTCCTATCGACATGGTTTACTAGGCGTTGACAAATT

ATTCTCATATGCAATCACTGAGTGGCTTAATGACATTAAGAAGAACCAGCTACCAGGAATCCTGGGAGGT

GTTGGACCTATGCATTCACTAGTACAATTAGTACAAGGCCTAAAGGACTTGGTCTGGCTCCCAATAGAGC

AGTACCGGAAGGATGGCCGCATTGTCAGAGGGTTTCAGAGAGGCGCTGCTTCCTTTGGTACCTCGACAGC

GATGGCTGCTCTAGAACTCACAAACAGAATGGTTCAAACCATACAGGCAGCTGCAGAGACTGCTTATGAT

ATGGTGTCTCCTGGTACCCTTTCTATCGAGCCCAAGAAGACCAAAAGGTTTCCTCATCACCGGTTAGCCC

ACCAGCCAGTAGACCTGAGGGAAGGTGTGGCCAAGGCCTACAGTGTTGTGAAAGAGGGAATCACAGACAC

GGCTCAGACCATTTATGAAACTGCGGCTCGAGAACACGAGAGCAGAGGGGTGACTGGTGCCGTGGGCGAG

GTTCTGCGCCAGATTCCTCCGGCAGTGGTGAAACCTCTGATTGTTGCCACAGAAGCAACGTCAAACGTGC

TGGGTGGCATGAGAAACCAAATTAGGCCAGATGTCCGGCAAGACGAGTCACAGAAATGGCGCCACGGGGA

TGACTGA

>lcl|NM_022488.5_cds_NP_071933.2_1 [gene=ATG3] [db_xref=CCDS:CCDS2966.1] [protein=ubiquitin-like-conjugating enzyme ATG3 isoform 1] [protein_id=NP_071933.2] [location=435..1379] [gbkey=CDS]

ATGCAGAATGTGATTAATACTGTGAAGGGAAAGGCACTGGAAGTGGCTGAGTACCTGACCCCGGTCCTCA

AGGAATCAAAGTTTAAGGAAACAGGTGTAATTACCCCAGAAGAGTTTGTGGCAGCTGGAGATCACCTAGT

CCACCACTGTCCAACATGGCAATGGGCTACAGGGGAAGAATTGAAAGTGAAGGCATACCTACCAACAGGC

AAACAATTTTTGGTAACCAAAAATGTGCCGTGCTATAAGCGGTGCAAACAGATGGAATATTCAGATGAAT

TGGAAGCTATCATTGAAGAAGATGATGGTGATGGCGGATGGGTAGATACATATCACAACACAGGTATTAC

AGGAATAACGGAAGCCGTTAAAGAGATCACACTGGAAAATAAGGACAATATAAGGCTTCAAGATTGCTCA

GCACTATGTGAAGAGGAAGAAGATGAAGATGAAGGAGAAGCTGCAGATATGGAAGAATATGAAGAGAGTG

GATTGTTGGAAACAGATGAGGCTACCCTAGATACAAGGAAAATAGTAGAAGCTTGTAAAGCCAAAACTGA

TGCTGGCGGTGAAGATGCTATTTTGCAAACCAGAACTTATGACCTTTACATCACTTATGATAAATATTAC

CAGACTCCACGATTATGGTTGTTTGGCTATGATGAGCAACGGCAGCCTTTAACAGTTGAGCACATGTATG

AAGACATCAGTCAGGATCATGTGAAGAAAACAGTGACCATTGAAAATCACCCTCATCTGCCACCACCTCC

CATGTGTTCAGTTCACCCATGCAGGCATGCTGAGGTGATGAAGAAAATCATTGAGACTGTTGCAGAAGGA

GGGGGAGAACTTGGAGTTCATATGTATCTTCTTATTTTCTTGAAATTTGTACAAGCTGTCATTCCAACAA

TAGAATATGACTACACAAGACACTTCACAATGTAA

>lcl|NM_052936.5_cds_NP_443168.2_1 [gene=ATG4A] [db_xref=CCDS:CCDS14538.1] [protein=cysteine protease ATG4A isoform a] [protein_id=NP_443168.2] [location=50..1246] [gbkey=CDS]

ATGGAGTCAGTTTTATCCAAGTATGAAGATCAGATTACTATTTTCACTGACTACCTAGAAGAATATCCAG

ATACAGATGAGCTGGTATGGATCTTAGGGAAGCAGCATCTCCTTAAAACAGAAAAATCTAAGCTGTTGTC

TGATATAAGTGCTCGTCTATGGTTTACATACAGAAGGAAATTTTCACCAATTGGTGGAACGGGCCCTTCA

TCAGATGCTGGTTGGGGATGTATGCTACGCTGTGGACAGATGATGCTGGCTCAAGCCCTTATCTGTAGAC

ACTTGGGAAGGGACTGGAGCTGGGAGAAACAAAAAGAACAACCCAAAGAATACCAACGCATCCTACAGTG

CTTCTTAGATAGAAAAGATTGTTGCTACTCTATCCATCAAATGGCACAAATGGGTGTAGGAGAAGGGAAA

TCAATTGGAGAATGGTTTGGACCAAATACAGTTGCACAGGTGTTAAAAAAACTTGCTTTATTTGACGAAT

GGAATTCCTTGGCTGTTTATGTTTCAATGGATAACACAGTGGTCATTGAAGATATCAAAAAAATGTGCCG

TGTCCTTCCCTTGAGTGCTGACACAGCTGGTGACAGGCCTCCCGATTCTTTAACTGCTTCAAACCAGAGT

AAGGGCACCTCTGCCTACTGCTCAGCCTGGAAACCCCTGCTGCTCATTGTGCCCCTTCGCCTGGGCATAA

ACCAAATCAATCCTGTCTATGTTGATGCATTCAAAGAGTGTTTTAAGATGCCACAGTCTTTAGGGGCATT

AGGAGGAAAACCAAATAACGCGTATTATTTCATAGGATTCTTAGGTGACGAGCTCATCTTCTTGGACCCT

CATACAACCCAGACCTTTGTTGACACTGAAGAGAATGGAACGGTTAATGACCAGACTTTCCATTGCCTGC

AGTCCCCACAGCGAATGAACATCCTAAACCTGGATCCTTCAGTTGCATTGGGATTTTTCTGCAAAGAAGA

AAAAGACTTTGATAACTGGTGTAGCCTTGTTCAGAAGGAAATTCTAAAGGAGAATTTAAGGATGTTTGAA

TTAGTTCAGAAACATCCATCACACTGGCCTCCCTTTGTACCTCCAGCCAAGCCAGAAGTGACAACCACTG

GGGCAGAATTCATTGACTCTACTGAGCAACTGGAGGAGTTTGATCTGGAGGAAGATTTTGAGATTCTGAG

TGTGTAG

>lcl|NM_013325.5_cds_NP_037457.3_1 [gene=ATG4B] [db_xref=CCDS:CCDS46564.1] [protein=cysteine protease ATG4B isoform a] [protein_id=NP_037457.3] [location=23..1204] [gbkey=CDS]

ATGGACGCAGCTACTCTGACCTACGACACTCTCCGGTTTGCTGAGTTTGAAGATTTTCCTGAGACCTCAG

AGCCCGTTTGGATACTGGGTAGAAAATACAGCATTTTCACAGAAAAGGACGAGATCTTGTCTGATGTGGC

ATCTAGACTTTGGTTTACATACAGGAAAAACTTTCCAGCCATTGGGGGGACAGGCCCCACCTCGGACACA

GGCTGGGGCTGCATGCTGCGGTGTGGACAGATGATCTTTGCCCAAGCCCTGGTGTGCCGGCACCTAGGCC

GAGATTGGAGGTGGACACAAAGGAAGAGGCAGCCAGACAGCTACTTCAGCGTCCTCAACGCATTCATCGA

CAGGAAGGACAGTTACTACTCCATTCACCAGATAGCGCAAATGGGAGTTGGCGAAGGCAAGTCCATAGGC

CAGTGGTACGGGCCCAACACTGTCGCCCAGGTCCTGAAGAAGCTTGCTGTCTTCGATACGTGGAGCTCCT

TGGCGGTCCACATTGCAATGGACAACACTGTTGTGATGGAGGAAATCAGAAGGTTGTGCAGGACCAGCGT

TCCCTGTGCAGGCGCCACTGCGTTTCCTGCAGATTCCGACCGGCACTGCAACGGATTCCCTGCCGGAGCT

GAGGTCACCAACAGGCCGTCGCCATGGAGACCCCTGGTACTTCTCATTCCCCTGCGCCTGGGGCTCACGG

ACATCAACGAGGCCTACGTGGAGACGCTGAAGCACTGCTTCATGATGCCCCAGTCCCTGGGCGTCATCGG

AGGGAAGCCCAACAGCGCCCACTACTTCATCGGCTACGTTGGTGAGGAGCTCATCTACCTGGACCCCCAC

ACCACGCAGCCAGCCGTGGAGCCCACTGATGGCTGCTTCATCCCGGACGAGAGCTTCCACTGCCAGCACC

CGCCGTGCCGCATGAGCATCGCGGAGCTTGACCCGTCCATCGCTGTGGGGTTTTTCTGTAAGACTGAAGA

TGACTTCAATGATTGGTGCCAGCAAGTCAAAAAGCTGTCTCTGCTTGGAGGTGCCCTGCCCATGTTTGAG

CTGGTGGAGCTGCAGCCTTCACATCTGGCCTGCCCCGACGTCCTGAACCTGTCCCTAGATTCTTCTGATG

TAGAGCGACTGGAAAGATTCTTCGACTCAGAAGATGAAGACTTTGAAATCCTGTCCCTTTGA

>lcl|NM_032852.4_cds_NP_116241.2_1 [gene=ATG4C] [db_xref=CCDS:CCDS623.1] [protein=cysteine protease ATG4C] [protein_id=NP_116241.2] [location=211..1587] [gbkey=CDS]

ATGGAGGCTACAGGAACAGATGAAGTTGACAAGCTAAAAACCAAATTTATATCTGCTTGGAACAACATGA

AATATAGTTGGGTGTTGAAAACAAAGACGTATTTTAGTAGAAATTCTCCTGTATTATTGCTTGGAAAATG

TTACCATTTTAAATATGAAGATGAAGATAAAACGTTACCTGCAGAGTCGGGATGTACAATAGAGGATCAC

GTAATTGCAGGAAATGTAGAAGAATTTCGTAAAGATTTCATTTCTAGAATATGGCTGACCTACAGGGAAG

AATTCCCTCAAATAGAAGGCTCAGCTTTGACAACAGACTGTGGGTGGGGCTGCACATTGAGAACTGGCCA

GATGCTCTTGGCTCAAGGACTCATACTACACTTTCTTGGTAGAGCTTGGACCTGGCCTGATGCTTTGAAT

ATTGAAAATTCAGACTCTGAATCATGGACTTCCCACACTGTCAAAAAATTTACTGCATCATTTGAAGCAT

CACTTTCAGGGGAAAGAGAATTCAAAACCCCAACAATTTCTCTGAAGGAAACAATTGGGAAATATTCTGA

TGATCATGAAATGCGAAATGAAGTTTATCATAGGAAAATCATCTCTTGGTTTGGTGATTCCCCCTTGGCT

CTTTTTGGCTTACATCAACTAATAGAATATGGAAAGAAGTCTGGGAAAAAAGCAGGAGATTGGTATGGAC

CAGCTGTGGTTGCTCACATTTTAAGAAAAGCAGTTGAAGAAGCAAGGCATCCTGATTTACAAGGAATAAC

TATTTATGTTGCACAAGATTGTACAGTTTACAATTCTGATGTAATTGATAAACAGAGTGCTTCCATGACT

TCTGATAATGCAGATGACAAAGCTGTTATTATTCTAGTTCCTGTTAGACTTGGTGGAGAAAGAACCAACA

CCGACTACTTAGAATTTGTGAAGGGTATTTTAAGCCTGGAATATTGTGTGGGTATTATTGGTGGCAAACC

TAAACAGTCATATTACTTTGCTGGATTTCAAGATGACAGTTTGATTTACATGGATCCTCATTACTGCCAA

TCTTTTGTAGATGTCAGCATAAAGGATTTCCCTCTTGAGACATTCCACTGCCCTTCTCCCAAAAAGATGT

CTTTTCGAAAAATGGATCCCAGCTGTACAATAGGATTTTACTGTCGAAATGTTCAGGACTTCAAACGAGC

TTCTGAAGAAATCACCAAGATGCTGAAATTTTCTTCTAAGGAGAAATATCCCTTATTTACTTTTGTAAAT

GGTCATTCCAGAGACTATGATTTTACATCTACTACAACCAATGAAGAAGACCTTTTTTCAGAGGATGAAA

AGAAACAATTAAAAAGATTTAGCACGGAAGAGTTTGTCTTGCTTTAA

>lcl|NM_032885.6_cds_NP_116274.3_1 [gene=ATG4D] [db_xref=CCDS:CCDS12241.1] [protein=cysteine protease ATG4D isoform 1 precursor] [protein_id=NP_116274.3] [location=188..1612] [gbkey=CDS]

ATGAACTCAGTGTCGCCGGCCGCCGCGCAGTACCGGAGCAGCAGCCCGGAGGACGCGCGCCGCCGGCCCG

AGGCCCGCAGGCCGCGGGGTCCCAGAGGCCCAGACCCCAACGGCCTGGGGCCTTCCGGAGCCAGCGGCCC

CGCTCTTGGCTCTCCCGGGGCTGGCCCGAGTGAGCCGGACGAAGTGGACAAGTTCAAGGCCAAGTTCCTG

ACAGCCTGGAACAACGTCAAGTACGGTTGGGTGGTTAAAAGCCGGACCAGCTTTAGCAAGATCTCCAGCA

TCCACCTCTGTGGCCGCCGCTACCGTTTCGAGGGCGAGGGTGACATACAGCGTTTCCAGCGGGACTTTGT

GTCCCGCCTGTGGCTCACATACCGCCGGGACTTCCCGCCCCTTCCTGGGGGCTGCCTGACCTCGGACTGT

GGCTGGGGGTGCATGTTACGCAGCGGCCAGATGATGCTGGCACAGGGCCTTCTGCTGCATTTCCTGCCCA

GAGACTGGACATGGGCCGAGGGCATGGGCCTGGGCCCCCCTGAGCTGTCAGGGTCAGCCTCTCCCAGCCG

GTACCATGGGCCTGCCCGCTGGATGCCCCCACGCTGGGCCCAGGGTGCCCCTGAGCTGGAGCAGGAACGC

CGGCACCGGCAGATTGTGTCCTGGTTCGCCGACCACCCCCGGGCCCCCTTTGGCCTACACCGGCTGGTGG

AGCTTGGGCAGAGCTCAGGCAAGAAGGCAGGTGACTGGTATGGGCCATCGCTAGTGGCACACATCCTCAG

GAAAGCCGTGGAGAGCTGCTCCGACGTCACCCGCCTGGTGGTGTACGTTTCTCAGGACTGCACAGTGTAC

AAGGCGGATGTGGCACGCCTGGTGGCCAGGCCAGACCCCACAGCCGAGTGGAAGTCTGTGGTCATCCTGG

TGCCCGTGCGACTGGGTGGCGAGACTCTCAACCCCGTGTATGTGCCCTGCGTGAAGGAACTCCTGCGTTG

CGAGCTGTGCCTGGGCATCATGGGTGGGAAACCGCGACACTCACTGTACTTCATTGGCTACCAAGATGAC

TTCCTGCTGTACCTGGACCCTCACTACTGCCAGCCCACTGTGGATGTCAGCCAGGCCGACTTCCCCCTGG

AGTCCTTCCACTGCACCTCGCCCCGCAAGATGGCCTTTGCCAAGATGGACCCAAGCTGTACCGTGGGCTT

CTATGCTGGAGACAGGAAGGAGTTTGAGACACTCTGCTCAGAGCTGACCAGGGTCCTCAGCTCCTCCTCA

GCCACAGAGCGGTACCCCATGTTCACCCTGGCCGAGGGCCATGCTCAGGACCACAGCCTGGACGACCTCT

GCTCCCAGCTCGCCCAGCCCACACTCCGGCTCCCTCGCACAGGGCGGCTCCTCAGGGCCAAACGCCCCAG

CTCTGAGGACTTTGTGTTTTTATAA

>lcl|NM_004849.4_cds_NP_004840.1_1 [gene=ATG5] [db_xref=CCDS:CCDS5055.1] [protein=autophagy protein 5 isoform a] [protein_id=NP_004840.1] [location=294..1121] [gbkey=CDS]

ATGACAGATGACAAAGATGTGCTTCGAGATGTGTGGTTTGGACGAATTCCAACTTGTTTCACGCTATATC

AGGATGAGATAACTGAAAGGGAAGCAGAACCATACTATTTGCTTTTGCCAAGAGTAAGTTATTTGACGTT

GGTAACTGACAAAGTGAAAAAGCACTTTCAGAAGGTTATGAGACAAGAAGACATTAGTGAGATATGGTTT

GAATATGAAGGCACACCACTGAAATGGCATTATCCAATTGGTTTGCTATTTGATCTTCTTGCATCAAGTT

CAGCTCTTCCTTGGAACATCACAGTACATTTTAAGAGTTTTCCAGAAAAAGACCTTCTGCACTGTCCATC

TAAGGATGCAATTGAAGCTCATTTTATGTCATGTATGAAAGAAGCTGATGCTTTAAAACATAAAAGTCAA

GTAATCAATGAAATGCAGAAAAAAGATCACAAGCAACTCTGGATGGGATTGCAAAATGACAGATTTGACC

AGTTTTGGGCCATCAATCGGAAACTCATGGAATATCCTGCAGAAGAAAATGGATTTCGTTATATCCCCTT

TAGAATATATCAGACAACGACTGAAAGACCTTTCATTCAGAAGCTGTTTCGTCCTGTGGCTGCAGATGGA

CAGTTGCACACACTAGGAGATCTCCTCAAAGAAGTTTGTCCTTCTGCTATTGATCCTGAAGATGGGGAAA

AAAAGAATCAAGTGATGATTCATGGAATTGAGCCAATGTTGGAAACACCTCTGCAGTGGCTGAGTGAACA

TCTGAGCTACCCGGATAATTTTCTTCATATTAGTATCATCCCACAGCCAACAGATTGA

>lcl|NM_006395.3_cds_NP_006386.1_1 [gene=ATG7] [db_xref=CCDS:CCDS2605.1] [protein=ubiquitin-like modifier-activating enzyme ATG7 isoform a] [protein_id=NP_006386.1] [location=45..2156] [gbkey=CDS]

ATGGCGGCAGCTACGGGGGATCCTGGACTCTCTAAACTGCAGTTTGCCCCTTTTAGTAGTGCCTTGGATG

TTGGGTTTTGGCATGAGTTGACCCAGAAGAAGCTGAACGAGTATCGGCTGGATGAAGCTCCCAAGGACAT

TAAGGGTTATTACTACAATGGTGACTCTGCTGGGCTGCCAGCTCGCTTAACATTGGAGTTCAGTGCTTTT

GACATGAGTGCTCCCACCCCAGCCCGTTGCTGCCCAGCTATTGGAACACTGTATAACACCAACACACTCG

AGTCTTTCAAGACTGCAGATAAGAAGCTCCTTTTGGAACAAGCAGCAAATGAGATATGGGAATCCATAAA

ATCAGGCACTGCTCTTGAAAACCCTGTACTCCTCAACAAGTTCCTCCTCTTGACATTTGCAGATCTAAAG

AAGTACCACTTCTACTATTGGTTTTGCTATCCTGCCCTCTGTCTTCCAGAGAGTTTACCTCTCATTCAGG

GGCCAGTGGGTTTGGATCAAAGGTTTTCACTAAAACAGATTGAAGCACTAGAGTGTGCATATGATAATCT

TTGTCAAACAGAAGGAGTCACAGCTCTTCCTTACTTCTTAATCAAGTATGATGAGAACATGGTGCTGGTT

TCCTTGCTTAAACACTACAGTGATTTCTTCCAAGGTCAAAGGACGAAGATAACAATTGGTGTATATGATC

CCTGTAACTTAGCCCAGTACCCTGGATGGCCTTTGAGGAATTTTTTGGTCCTAGCAGCCCACAGATGGAG

TAGCAGTTTCCAGTCTGTTGAAGTTGTTTGCTTCCGTGACCGTACCATGCAGGGGGCGAGAGACGTTGCC

CACAGCATCATCTTCGAAGTGAAGCTTCCAGAAATGGCATTTAGCCCAGATTGTCCTAAAGCAGTTGGAT

GGGAAAAGAACCAGAAAGGAGGCATGGGACCAAGGATGGTGAACCTCAGTGAATGTATGGACCCTAAAAG

GTTAGCTGAGTCATCAGTGGATCTAAATCTCAAACTGATGTGTTGGAGATTGGTTCCTACTTTAGACTTG

GACAAGGTTGTGTCTGTCAAATGTCTGCTGCTTGGAGCCGGCACCTTGGGTTGCAATGTAGCTAGGACGT

TGATGGGTTGGGGCGTGAGACACATCACATTTGTGGACAATGCCAAGATCTCCTACTCCAATCCTGTGAG

GCAGCCTCTCTATGAGTTTGAAGATTGCCTAGGGGGTGGTAAGCCCAAGGCTCTGGCAGCAGCGGACCGG

CTCCAGAAAATATTCCCCGGTGTGAATGCCAGAGGATTCAACATGAGCATACCTATGCCTGGGCATCCAG

TGAACTTCTCCAGTGTCACTCTGGAGCAAGCCCGCAGAGATGTGGAGCAACTGGAGCAGCTCATCGAAAG

CCATGATGTCGTCTTCCTATTGATGGACACCAGGGAGAGCCGGTGGCTTCCTGCCGTCATTGCTGCAAGC

AAGAGAAAGCTGGTCATCAATGCTGCTTTGGGATTTGACACATTTGTTGTCATGAGACATGGTCTGAAGA

AACCAAAGCAGCAAGGAGCTGGGGACTTGTGTCCAAACCACCCTGTGGCATCTGCTGACCTCCTGGGCTC

ATCGCTTTTTGCCAACATCCCTGGTTACAAGCTTGGCTGCTACTTCTGCAATGATGTGGTGGCCCCAGGA

GATTCAACCAGAGACCGGACCTTGGACCAGCAGTGCACTGTGAGTCGTCCAGGACTGGCCGTGATTGCAG

GAGCCCTGGCCGTGGAATTGATGGTATCTGTTTTGCAGCATCCAGAAGGGGGCTATGCCATTGCCAGCAG

CAGTGACGATCGGATGAATGAGCCTCCAACCTCTCTTGGGCTTGTGCCTCACCAGATCCGGGGATTTCTT

TCACGGTTTGATAATGTCCTTCCCGTCAGCCTGGCATTTGACAAATGTACAGCTTGTTCTTCCAAAGTTC

TTGATCAATATGAACGAGAAGGATTTAACTTCCTAGCCAAGGTGTTTAATTCTTCACATTCCTTCTTAGA

AGACTTGACTGGTCTTACATTGCTGCATCAAGAAACCCAAGCTGCTGAGATCTGGGACATGAGCGATGAT

GAGACCATCTGA

>lcl|NM_024085.5_cds_NP_076990.4_1 [gene=ATG9A] [db_xref=CCDS:CCDS42820.1] [protein=autophagy-related protein 9A] [protein_id=NP_076990.4] [location=132..2651] [gbkey=CDS]

ATGGCGCAGTTTGACACTGAATACCAGCGCCTAGAGGCCTCCTATAGTGATTCACCCCCAGGGGAGGAGG

ACCTGTTGGTGCACGTCGCCGAGGGGAGCAAGTCACCTTGGCACCATATTGAAAACCTTGACCTCTTCTT

CTCTCGAGTTTATAATCTGCACCAGAAGAATGGCTTCACATGTATGCTCATCGGGGAGATCTTTGAGCTC

ATGCAGTTCCTCTTTGTGGTTGCCTTCACTACCTTCCTGGTCAGCTGCGTGGACTATGACATCCTATTTG

CCAACAAGATGGTGAACCACAGTCTTCACCCTACTGAACCCGTCAAGGTCACTCTGCCAGACGCCTTTTT

GCCTGCTCAAGTCTGTAGTGCCAGGATTCAGGAAAATGGCTCCCTTATCACCATCCTGGTCATTGCTGGT

GTCTTCTGGATCCACCGGCTTATCAAGTTCATCTATAACATTTGCTGCTACTGGGAGATCCACTCCTTCT

ACCTGCACGCTCTGCGCATCCCTATGTCTGCCCTTCCGTATTGCACGTGGCAAGAAGTGCAGGCCCGGAT

CGTGCAGACGCAGAAGGAGCACCAGATCTGCATCCACAAACGTGAGCTGACAGAACTGGACATCTACCAC

CGCATCCTCCGTTTCCAGAACTACATGGTGGCACTGGTTAACAAATCCCTCCTGCCTCTGCGCTTCCGCC

TGCCTGGCCTCGGGGAAGCTGTCTTCTTCACCCGTGGTCTCAAGTACAACTTTGAGCTGATCCTCTTCTG

GGGACCTGGCTCTCTGTTTCTCAATGAATGGAGCCTCAAGGCCGAGTACAAACGTGGGGGGCAACGGCTA

GAGCTGGCCCAGCGCCTCAGCAACCGCATCCTGTGGATTGGCATCGCTAACTTCCTGCTGTGCCCCCTCA

TCCTCATATGGCAAATCCTCTATGCCTTCTTCAGCTATGCTGAGGTGCTGAAGCGGGAGCCGGGGGCCCT

GGGAGCACGCTGCTGGTCACTCTATGGCCGCTGCTACCTCCGCCACTTCAACGAGCTGGAGCACGAGCTG

CAGTCCCGCCTCAACCGTGGCTACAAGCCCGCCTCCAAGTACATGAATTGCTTCTTGTCACCTCTTTTGA

CACTGCTGGCCAAGAATGGAGCCTTCTTCGCTGGCTCCATCCTGGCTGTGCTTATTGCCCTCACCATTTA

TGACGAAGATGTGTTGGCTGTGGAACATGTGCTGACCACCGTCACACTCCTGGGGGTCACCGTGACCGTG

TGCAGGTCCTTTATCCCGGACCAGCACATGGTGTTCTGCCCTGAGCAGCTGCTCCGCGTGATCCTCGCTC

ACATCCACTACATGCCTGACCACTGGCAGGGTAATGCCCACCGCTCGCAGACCCGGGACGAGTTTGCCCA

GCTCTTCCAGTACAAGGCAGTGTTCATTTTGGAAGAGTTGCTGAGCCCCATTGTCACACCCCTCATCCTC

ATCTTCTGCCTGCGCCCACGGGCCCTGGAGATTATAGACTTCTTCCGAAACTTCACCGTGGAGGTCGTTG

GTGTGGGAGATACCTGCTCCTTTGCTCAGATGGATGTTCGCCAGCATGGTCATCCCCAGTGGCTATCTGC

TGGGCAGACAGAGGCCTCAGTGTACCAGCAAGCTGAGGATGGAAAGACAGAGTTGTCACTCATGCACTTT

GCCATCACCAACCCTGGCTGGCAGCCACCACGTGAGAGCACAGCCTTCCTAGGCTTCCTCAAGGAGCAGG

TTCAGCGGGATGGAGCAGCTGCTAGCCTCGCCCAAGGGGGTCTGCTCCCTGAAAATGCCCTCTTTACGTC

TATCCAGTCCTTACAATCTGAGTCTGAGCCCCTGAGCCTTATCGCAAATGTGGTAGCTGGCTCATCCTGC

CGGGGCCCTCCACTGCCCAGAGACCTGCAGGGCTCCAGGCACAGGGCTGAAGTCGCCTCTGCCCTGCGCT

CCTTCTCCCCGCTGCAACCCGGGCAGGCGCCCACAGGCCGGGCTCACAGCACCATGACAGGCTCTGGGGT

GGATGCCAGGACAGCCAGCTCCGGGAGCAGCGTGTGGGAAGGACAGCTGCAGAGCCTGGTGCTGTCAGAA

TATGCATCCACAGAGATGAGCCTGCATGCCCTCTATATGCACCAGCTCCACAAGCAGCAGGCCCAGGCTG

AACCTGAGCGGCATGTATGGCACCGCCGGGAGAGTGATGAGAGTGGAGAAAGCGCCCCTGATGAAGGGGG

AGAGGGCGCCCGGGCCCCCCAGTCTATCCCTCGCTCTGCTAGCTATCCCTGTGCAGCACCCCGGCCTGGA

GCTCCTGAGACCACTGCCCTGCATGGGGGCTTCCAGAGGCGCTACGGTGGCATCACAGATCCTGGCACAG

TGCCCAGGGTTCCCTCTCATTTCTCTCGGCTGCCTCTTGGAGGGTGGGCAGAAGATGGGCAGTCGGCATC

AAGGCACCCTGAGCCCGTGCCCGAAGAGGGCTCGGAGGATGAGCTACCCCCTCAGGTGCACAAGGTATAG

>lcl|NM_001317056.2_cds_NP_001303985.1_1 [gene=ATG9B] [db_xref=CCDS:CCDS83242.1] [protein=autophagy-related protein 9B] [protein_id=NP_001303985.1] [location=72..2846] [gbkey=CDS]

ATGGTGAGCCGAATGGGCTGGGGGGGGAGAAGAAGGCGGCTGGGGCGGTGGGGAGATCTGGGGCCCGGAT

CGGTGCCCCTCCTCCCCATGCCACTGCCACCTCCTCCTCCTCCTTCATGCCGGGGACCTGGGGGAGGGAG

GATCTCCATCTTCTCTCTGTCCCCTGCCCCTCATACAAGAAGCTCCCCCTCCTCATTTTCCCCTCCCACC

GCAGGGCCCCCTTGCTCAGTGCTACAGGGGACAGGGGCTTCTCAGTCTTGCCACAGTGCTCTCCCTATCC

CAGCCACCCCCCCAACACAGGCTCAACCTGCAATGACACCTGCCTCTGCATCTCCCTCCTGGGGATCCCA

CTCCACCCCACCCCTGGCCCCGGCAACCCCCACTCCCTCACAGCAGTGCCCCCAGGACTCTCCTGGGCTG

CGGGTAGGCCCTTTGATCCCTGAACAGGATTATGAGCGGCTGGAGGACTGTGACCCTGAGGGGTCCCAAG

ACTCACCCATCCACGGGGAGGAGCAGCAACCCCTGCTTCATGTCCCTGAAGGGCTCCGAGGCTCCTGGCA

TCACATCCAGAACCTGGACAGTTTCTTCACCAAGATCTACAGCTACCACCAGCGGAATGGCTTTGCCTGC

ATCTTGCTGGAGGATGTCTTCCAGCTGGGACAATTTATTTTCATTGTCACCTTCACAACCTTCCTCCTTC

GATGCGTGGATTACAATGTTCTCTTTGCCAACCAACCAAGTAACCATACCAGACCTGGGCCGTTCCACAG

CAAAGTGACCCTGTCAGATGCCATCCTACCCTCAGCCCAGTGTGCTGAGAGGATCCGCTCCAGCCCGCTG

CTGGTCCTCCTCCTGGTCCTGGCTGCCGGCTTCTGGCTGGTCCAACTGCTTCGCTCAGTCTGCAACCTCT

TCAGCTACTGGGACATCCAGGTGTTTTACAGGGAGGCCCTGCACATCCCCCCGGAGGAGCTGAGCTCGGT

TCCCTGGGCAGAGGTGCAGTCCCGCCTCTTGGCACTGCAGCGGAGCGGGGGCCTGTGCGTGCAGCCGCGG

CCCCTGACGGAGCTGGACATCCACCACCGCATCCTGCGCTACACCAACTACCAGGTGGCGCTGGCCAACA

AAGGCCTGCTGCCGGCCCGCTGCCCGCTGCCCTGGGGAGGCAGTGCGGCTTTCCTCAGCCGCGGCCTGGC

GCTCAATGTCGACCTGCTGCTCTTCCGCGGTCCCTTCTCGCTCTTCCGCGGGGGCTGGGAGCTGCCGCAC

GCCTACAAGCGCAGCGACCAGCGGGGCGCCCTAGCAGCGCGCTGGGGGCGCACAGTGCTGCTGCTGGCCG

CCCTGAACCTGGCGCTGAGCCCGCTGGTGCTGGCCTGGCAGGTTCTGCACGTCTTCTATAGCCACGTGGA

GCTGCTGCGGCGCGAGCCTGGCGCGCTGGGGGCGCGCGGCTGGTCCCGCCTGGCGCGCTTGCAGCTGCGC

CACTTCAACGAGCTGCCGCACGAGCTGCGCGCGCGCCTGGCCCGCGCCTACCGCCCCGCCGCCGCCTTCC

TGCGCACCGCTGCGCCCCCCGCGCCCCTGCGCACGCTGCTGGCCCGCCAGCTCGTTTTCTTCGCGGGTGC

ACTCTTCGCCGCGCTGCTTGTGCTCACCGTCTACGACGAGGACGTGCTAGCCGTGGAGCACGTGCTCACC

GCCATGACCGCGCTCGGGGTCACCGCCACCGTCGCCAGGTCTTTCATTCCGGAAGAGCAGTGCCAGGGTC

GTGCGCCGCAGCTCCTGCTGCAGACAGCCCTGGCCCACATGCACTACCTCCCGGAGGAGCCCGGCCCCGG

CGGCAGGGACCGCGCCTACCGGCAGATGGCGCAGCTGCTGCAGTACCGAGCGGTCTCCCTCCTGGAGGAG

CTCCTGTCCCCGCTCCTCACCCCGCTGTTTCTGCTTTTCTGGTTCCGCCCTCGTGCCCTGGAGATTATCG

ACTTTTTTCATCACTTCACTGTGGATGTGGCTGGGGTTGGGGACATCTGTTCCTTTGCCCTTATGGATGT

GAAGCGCCACGGACACCCTCAGTGGCTCTCGGCGGGACAGACTGAGGCCTCGCTGTCTCAGCGTGCGGAG

GACGGCAAGACTGAGCTTTCTTTGATGCGGTTCTCCCTGGCGCATCCACTCTGGCGCCCCCCAGGGCACA

GCTCTAAGTTTCTTGGGCACCTCTGGGGCCGAGTACAACAAGATGCAGCTGCCTGGGGTGCCACCTCGGC

TCGCGGCCCCTCCACCCCGGGGGTGCTCAGCAACTGCACCTCGCCCCTGCCTGAGGCCTTCCTGGCCAAC

CTCTTCGTGCACCCTCTCCTGCCTCCGAGAGATCTGAGCCCGACAGCCCCCTGTCCAGCTGCGGCCACAG

CCAGCCTCCTTGCCTCCATTTCCCGAATTGCCCAGGACCCCAGCTCTGTGTCCCCAGGAGGCACTGGGGG

CCAGAAGCTGGCCCAGCTCCCAGAACTTGCTTCTGCCGAGATGAGTCTCCATGTCATCTACCTGCACCAG

CTTCACCAGCAGCAGCAGCAGCAGGAGCCGTGGGGTGAGGCTGCAGCCTCCATCCTGTCCAGGCCCTGCT

CCAGCCCCTCACAGCCACCCTCGCCTGATGAGGAGAAGCCATCCTGGTCAAGTGACGGCTCCAGTCCTGC

CTCTAGCCCCAGACAACAGTGGGGAACCCAGAAGGCCCGGAATCTGTTCCCCGGAGGGTTTCAGGTGACC

ACAGACACCCAGAAGGAGCCTGACCGGGCCTCTTGCACTGACTGA

>lcl|NM_004044.7_cds_NP_004035.2_1 [gene=ATIC] [db_xref=CCDS:CCDS2398.1] [protein=bifunctional purine biosynthesis protein ATIC] [protein_id=NP_004035.2] [location=85..1863] [gbkey=CDS]

ATGGCTCCCGGCCAGCTCGCCTTATTTAGTGTCTCTGACAAAACCGGCCTTGTGGAATTTGCAAGAAACC

TGACCGCTCTTGGTTTGAATCTGGTCGCTTCCGGAGGGACTGCAAAAGCTCTCAGGGATGCTGGTCTGGC

AGTCAGAGATGTCTCTGAGTTGACGGGATTTCCTGAAATGTTGGGGGGACGTGTGAAAACTTTGCATCCT

GCAGTCCATGCTGGAATCCTAGCTCGTAATATTCCAGAAGATAATGCTGACATGGCCAGACTTGATTTCA

ATCTTATAAGAGTTGTTGCCTGCAATCTCTATCCCTTTGTAAAGACAGTGGCTTCTCCAGGTGTAACTGT

TGAGGAGGCTGTGGAGCAAATTGACATTGGTGGAGTAACCTTACTGAGAGCTGCAGCCAAAAACCACGCT

CGAGTGACAGTGGTGTGTGAACCAGAGGACTATGTGGTGGTGTCCACGGAGATGCAGAGCTCCGAGAGTA

AGGACACCTCCTTGGAGACTAGACGCCAGTTAGCCTTGAAGGCATTCACTCATACGGCACAATATGATGA

AGCAATTTCAGATTATTTCAGGAAACAGTACAGCAAAGGCGTATCTCAGATGCCCTTGAGATATGGAATG

AACCCACATCAGACCCCTGCCCAGCTGTACACACTGCAGCCCAAGCTTCCCATCACAGTTCTAAATGGAG

CCCCTGGATTTATAAACTTGTGCGATGCTTTGAACGCCTGGCAGCTGGTGAAGGAACTCAAGGAGGCTTT

AGGTATTCCAGCCGCTGCCTCTTTCAAACATGTCAGCCCAGCAGGTGCTGCTGTTGGAATTCCACTCAGT

GAAGATGAGGCCAAAGTCTGCATGGTTTATGATCTCTATAAAACCCTCACACCCATCTCAGCGGCATATG

CAAGAGCAAGAGGGGCTGATAGGATGTCTTCATTTGGTGATTTTGTTGCATTGTCCGATGTTTGTGATGT

ACCAACTGCAAAAATTATTTCCAGAGAAGTATCTGATGGTATAATTGCCCCAGGATATGAAGAAGAAGCC

TTGACAATACTTTCCAAAAAGAAAAATGGAAACTATTGTGTCCTTCAGATGGACCAATCTTACAAACCAG

ATGAAAATGAAGTTCGAACTCTCTTTGGTCTTCATTTAAGCCAGAAGAGAAATAATGGTGTCGTCGACAA

GTCATTATTTAGCAATGTTGTTACCAAAAATAAAGATTTGCCAGAGTCTGCCCTCCGAGACCTCATCGTA

GCCACCATTGCTGTCAAGTACACTCAGTCTAACTCTGTGTGCTACGCCAAGAACGGGCAGGTTATCGGCA

TTGGAGCAGGACAGCAGTCTCGTATACACTGCACTCGCCTTGCAGGAGATAAGGCAAACTATTGGTGGCT

TAGACACCATCCACAAGTGCTTTCGATGAAGTTTAAAACAGGAGTGAAGAGAGCAGAAATCTCCAATGCC

ATCGATCAATATGTGACTGGAACCATTGGCGAGGATGAAGATTTGATAAAGTGGAAGGCACTGTTTGAGG

AAGTCCCTGAGTTACTCACTGAGGCAGAGAAGAAGGAATGGGTTGAGAAACTGACTGAAGTTTCTATCAG

CTCTGATGCCTTCTTCCCTTTCCGAGATAACGTAGACAGAGCTAAAAGGAGTGGTGTGGCGTACATTGCG

GCTCCCTCCGGTTCTGCTGCTGACAAAGTTGTGATTGAGGCCTGCGACGAACTGGGAATCATCCTCGCTC

ATACGAACCTTCGGCTCTTCCACCACTGA

>lcl|NM_004323.6_cds_NP_004314.6_1 [gene=BAG1] [db_xref=CCDS:CCDS35004.1] [protein=BAG family molecular chaperone regulator 1 isoform BAG-1L] [protein_id=NP_004314.6] [location=35..1072] [gbkey=CDS]

CTGGCTCAGCGCGGGGGGGCGCGGAGACCGCGAGGCGACCGGGAGCGGCTGGGTTCCCGGCTGCGCGCCC

TTCGGCCAGGCCGGGAGCCGCGCCAGTCGGAGCCCCCGGCCCAGCGTGGTCCGCCTCCCTCTGGGCGTCC

ACCTGCCCGGAGTACTGCCAGCGGGCATGACCGACCCACCAGGGGCGCCGCCGCCGGCGCTCGCAGGCCG

CGGATGAAGAAGAAAACCCGGCGCCGCTCGACCCGGAGCGAGGAGTTGACCCGGAGCGAGGAGTTGACCC

TGAGTGAGGAAGCGACCTGGAGTGAAGAGGCGACCCAGAGTGAGGAGGCGACCCAGGGCGAAGAGATGAA

TCGGAGCCAGGAGGTGACCCGGGACGAGGAGTCGACCCGGAGCGAGGAGGTGACCAGGGAGGAAATGGCG

GCAGCTGGGCTCACCGTGACTGTCACCCACAGCAATGAGAAGCACGACCTTCATGTTACCTCCCAGCAGG

GCAGCAGTGAACCAGTTGTCCAAGACCTGGCCCAGGTTGTTGAAGAGGTCATAGGGGTTCCACAGTCTTT

TCAGAAACTCATATTTAAGGGAAAATCTCTGAAGGAAATGGAAACACCGTTGTCAGCACTTGGAATACAA

GATGGTTGCCGGGTCATGTTAATTGGGAAAAAGAACAGTCCACAGGAAGAGGTTGAACTAAAGAAGTTGA

AACATTTGGAGAAGTCTGTGGAGAAGATAGCTGACCAGCTGGAAGAGTTGAATAAAGAGCTTACTGGAAT

CCAGCAGGGTTTTCTGCCCAAGGATTTGCAAGCTGAAGCTCTCTGCAAACTTGATAGGAGAGTAAAAGCC

ACAATAGAGCAGTTTATGAAGATCTTGGAGGAGATTGACACACTGATCCTGCCAGAAAATTTCAAAGACA

GTAGATTGAAAAGGAAAGGCTTGGTAAAAAAGGTTCAGGCATTCCTAGCCGAGTGTGACACAGTGGAGCA

GAACATCTGCCAGGAGACTGAGCGGCTGCAGTCTACAAACTTTGCCCTGGCCGAGTGA

>lcl|NM_004281.4_cds_NP_004272.2_1 [gene=BAG3] [db_xref=CCDS:CCDS7615.1] [protein=BAG family molecular chaperone regulator 3] [protein_id=NP_004272.2] [location=297..2024] [gbkey=CDS]

ATGAGCGCCGCCACCCACTCGCCCATGATGCAGGTGGCGTCCGGCAACGGTGACCGCGACCCTTTGCCCC

CCGGATGGGAGATCAAGATCGACCCGCAGACCGGCTGGCCCTTCTTCGTGGACCACAACAGCCGCACCAC

TACGTGGAACGACCCGCGCGTGCCCTCTGAGGGCCCCAAGGAGACTCCATCCTCTGCCAATGGCCCTTCC

CGGGAGGGCTCTAGGCTGCCGCCTGCTAGGGAAGGCCACCCTGTGTACCCCCAGCTCCGACCAGGCTACA

TTCCCATTCCTGTGCTCCATGAAGGCGCTGAGAACCGGCAGGTGCACCCTTTCCATGTCTATCCCCAGCC

TGGGATGCAGCGATTCCGAACTGAGGCGGCAGCAGCGGCTCCTCAGAGGTCCCAGTCACCTCTGCGGGGC

ATGCCAGAAACCACTCAGCCAGATAAACAGTGTGGACAGGTGGCAGCGGCGGCGGCAGCCCAGCCCCCAG

CCTCCCACGGACCTGAGCGGTCCCAGTCTCCAGCTGCCTCTGACTGCTCATCCTCATCCTCCTCGGCCAG

CCTGCCTTCCTCCGGCAGGAGCAGCCTGGGCAGTCACCAGCTCCCGCGGGGGTACATCTCCATTCCGGTG

ATACACGAGCAGAACGTTACCCGGCCAGCAGCCCAGCCCTCCTTCCACCAAGCCCAGAAGACGCACTACC

CAGCGCAGCAGGGGGAGTACCAGACCCACCAGCCTGTGTACCACAAGATCCAGGGGGATGACTGGGAGCC

CCGGCCCCTGCGGGCGGCATCCCCGTTCAGGTCATCTGTCCAGGGTGCATCGAGCCGGGAGGGCTCACCA

GCCAGGAGCAGCACGCCACTCCACTCCCCCTCGCCCATCCGTGTGCACACCGTGGTCGACAGGCCTCAGC

AGCCCATGACCCATCGAGAAACTGCACCTGTTTCCCAGCCTGAAAACAAACCAGAAAGTAAGCCAGGCCC

AGTTGGACCAGAACTCCCTCCTGGACACATCCCAATTCAAGTGATCCGCAAAGAGGTGGATTCTAAACCT

GTTTCCCAGAAGCCCCCACCTCCCTCTGAGAAGGTAGAGGTGAAAGTTCCCCCTGCTCCAGTTCCTTGTC

CTCCTCCCAGCCCTGGCCCTTCTGCTGTCCCCTCTTCCCCCAAGAGTGTGGCTACAGAAGAGAGGGCAGC

CCCCAGCACTGCCCCTGCAGAAGCTACACCTCCAAAACCAGGAGAAGCCGAGGCTCCCCCAAAACATCCA

GGAGTGCTGAAAGTGGAAGCCATCCTGGAGAAGGTACAGGGGCTGGAGCAGGCTGTAGACAACTTTGAAG

GCAAGAAGACTGACAAAAAGTACCTGATGATCGAAGAGTATTTGACCAAAGAGCTGCTGGCCCTGGATTC

AGTGGACCCCGAGGGACGAGCCGATGTGCGTCAGGCCAGGAGAGACGGTGTCAGGAAGGTTCAGACCATC

TTGGAAAAACTTGAACAGAAAGCCATTGATGTCCCAGGTCAAGTCCAGGTCTATGAACTCCAGCCCAGCA

ACCTTGAAGCAGATCAGCCACTGCAGGCAATCATGGAGATGGGTGCCGTGGCAGCAGACAAGGGCAAGAA

AAATGCTGGAAATGCAGAAGATCCCCACACAGAAACCCAGCAGCCAGAAGCCACAGCAGCAGCGACTTCA

AACCCCAGCAGCATGACAGACACCCCTGGTAACCCAGCAGCACCGTAG

>lcl|NM_001188.4_cds_NP_001179.1_1 [gene=BAK1] [db_xref=CCDS:CCDS4781.1] [protein=bcl-2 homologous antagonist/killer] [protein_id=NP_001179.1] [location=284..919] [gbkey=CDS]

ATGGCTTCGGGGCAAGGCCCAGGTCCTCCCAGGCAGGAGTGCGGAGAGCCTGCCCTGCCCTCTGCTTCTG

AGGAGCAGGTAGCCCAGGACACAGAGGAGGTTTTCCGCAGCTACGTTTTTTACCGCCATCAGCAGGAACA

GGAGGCTGAAGGGGTGGCTGCCCCTGCCGACCCAGAGATGGTCACCTTACCTCTGCAACCTAGCAGCACC

ATGGGGCAGGTGGGACGGCAGCTCGCCATCATCGGGGACGACATCAACCGACGCTATGACTCAGAGTTCC

AGACCATGTTGCAGCACCTGCAGCCCACGGCAGAGAATGCCTATGAGTACTTCACCAAGATTGCCACCAG

CCTGTTTGAGAGTGGCATCAATTGGGGCCGTGTGGTGGCTCTTCTGGGCTTCGGCTACCGTCTGGCCCTA

CACGTCTACCAGCATGGCCTGACTGGCTTCCTAGGCCAGGTGACCCGCTTCGTGGTCGACTTCATGCTGC

ATCACTGCATTGCCCGGTGGATTGCACAGAGGGGTGGCTGGGTGGCAGCCCTGAACTTGGGCAATGGTCC

CATCCTGAACGTGCTGGTGGTTCTGGGTGTGGTTCTGTTGGGCCAGTTTGTGGTACGAAGATTCTTCAAA

TCATGA

>lcl|NM_138763.4_cds_NP_620118.1_1 [gene=BAX] [db_xref=CCDS:CCDS12743.1] [protein=apoptosis regulator BAX isoform delta] [protein_id=NP_620118.1] [location=55..486] [gbkey=CDS]

ATGGACGGGTCCGGGGAGCAGCCCAGAGGCGGGGGGCCCACCAGCTCTGAGCAGATCATGAAGACAGGGG

CCCTTTTGCTTCAGGGGATGATTGCCGCCGTGGACACAGACTCCCCCCGAGAGGTCTTTTTCCGAGTGGC

AGCTGACATGTTTTCTGACGGCAACTTCAACTGGGGCCGGGTTGTCGCCCTTTTCTACTTTGCCAGCAAA

CTGGTGCTCAAGGCCCTGTGCACCAAGGTGCCGGAACTGATCAGAACCATCATGGGCTGGACATTGGACT

TCCTCCGGGAGCGGCTGTTGGGCTGGATCCAAGACCAGGGTGGTTGGGACGGCCTCCTCTCCTACTTTGG

GACGCCCACGTGGCAGACCGTGACCATCTTTGTGGCGGGAGTGCTCACCGCCTCACTCACCATCTGGAAG

AAGATGGGCTGA

>lcl|NM_000633.3_cds_NP_000624.2_1 [gene=BCL2] [db_xref=CCDS:CCDS11981.1] [protein=apoptosis regulator Bcl-2 isoform alpha] [protein_id=NP_000624.2] [location=883..1602] [gbkey=CDS]

ATGGCGCACGCTGGGAGAACAGGGTACGATAACCGGGAGATAGTGATGAAGTACATCCATTATAAGCTGT

CGCAGAGGGGCTACGAGTGGGATGCGGGAGATGTGGGCGCCGCGCCCCCGGGGGCCGCCCCCGCACCGGG

CATCTTCTCCTCCCAGCCCGGGCACACGCCCCATCCAGCCGCATCCCGGGACCCGGTCGCCAGGACCTCG

CCGCTGCAGACCCCGGCTGCCCCCGGCGCCGCCGCGGGGCCTGCGCTCAGCCCGGTGCCACCTGTGGTCC

ACCTGACCCTCCGCCAGGCCGGCGACGACTTCTCCCGCCGCTACCGCCGCGACTTCGCCGAGATGTCCAG

CCAGCTGCACCTGACGCCCTTCACCGCGCGGGGACGCTTTGCCACGGTGGTGGAGGAGCTCTTCAGGGAC

GGGGTGAACTGGGGGAGGATTGTGGCCTTCTTTGAGTTCGGTGGGGTCATGTGTGTGGAGAGCGTCAACC

GGGAGATGTCGCCCCTGGTGGACAACATCGCCCTGTGGATGACTGAGTACCTGAACCGGCACCTGCACAC

CTGGATCCAGGATAACGGAGGCTGGGATGCCTTTGTGGAACTGTACGGCCCCAGCATGCGGCCTCTGTTT

GATTTCTCCTGGCTGTCTCTGAAGACTCTGCTCAGTTTGGCCCTGGTGGGAGCTTGCATCACCCTGGGTG

CCTATCTGGGCCACAAGTGA

>lcl|NM_138578.3_cds_NP_612815.1_1 [gene=BCL2L1] [db_xref=CCDS:CCDS13189.1] [protein=bcl-2-like protein 1 isoform Bcl-X(L)] [protein_id=NP_612815.1] [location=382..1083] [gbkey=CDS]

ATGTCTCAGAGCAACCGGGAGCTGGTGGTTGACTTTCTCTCCTACAAGCTTTCCCAGAAAGGATACAGCT

GGAGTCAGTTTAGTGATGTGGAAGAGAACAGGACTGAGGCCCCAGAAGGGACTGAATCGGAGATGGAGAC

CCCCAGTGCCATCAATGGCAACCCATCCTGGCACCTGGCAGACAGCCCCGCGGTGAATGGAGCCACTGGC

CACAGCAGCAGTTTGGATGCCCGGGAGGTGATCCCCATGGCAGCAGTAAAGCAAGCGCTGAGGGAGGCAG

GCGACGAGTTTGAACTGCGGTACCGGCGGGCATTCAGTGACCTGACATCCCAGCTCCACATCACCCCAGG

GACAGCATATCAGAGCTTTGAACAGGTAGTGAATGAACTCTTCCGGGATGGGGTAAACTGGGGTCGCATT

GTGGCCTTTTTCTCCTTCGGCGGGGCACTGTGCGTGGAAAGCGTAGACAAGGAGATGCAGGTATTGGTGA

GTCGGATCGCAGCTTGGATGGCCACTTACCTGAATGACCACCTAGAGCCTTGGATCCAGGAGAACGGCGG

CTGGGATACTTTTGTGGAACTCTATGGGAACAATGCAGCAGCCGAGAGCCGAAAGGGCCAGGAACGCTTC

AACCGCTGGTTCCTGACGGGCATGACTGTGGCCGGCGTGGTTCTGCTGGGCTCACTCTTCAGTCGGAAAT

GA

>lcl|NM_003766.5_cds_NP_003757.1_1 [gene=BECN1] [db_xref=CCDS:CCDS11441.1] [protein=beclin-1 isoform a] [protein_id=NP_003757.1] [location=153..1505] [gbkey=CDS]

ATGGAAGGGTCTAAGACGTCCAACAACAGCACCATGCAGGTGAGCTTCGTGTGCCAGCGCTGCAGCCAGC

CCCTGAAACTGGACACGAGTTTCAAGATCCTGGACCGTGTCACCATCCAGGAACTCACAGCTCCATTACT

TACCACAGCCCAGGCGAAACCAGGAGAGACCCAGGAGGAAGAGACTAACTCAGGAGAGGAGCCATTTATT

GAAACTCCTCGCCAGGATGGTGTCTCTCGCAGATTCATCCCCCCAGCCAGGATGATGTCCACAGAAAGTG

CCAACAGCTTCACTCTGATTGGGGAGGCATCTGATGGCGGCACCATGGAGAACCTCAGCCGAAGACTGAA

GGTCACTGGGGACCTTTTTGACATCATGTCGGGCCAGACAGATGTGGATCACCCACTCTGTGAGGAATGC

ACAGATACTCTTTTAGACCAGCTGGACACTCAGCTCAACGTCACTGAAAATGAGTGTCAGAACTACAAAC

GCTGTTTGGAGATCTTAGAGCAAATGAATGAGGATGACAGTGAACAGTTACAGATGGAGCTAAAGGAGCT

GGCACTAGAGGAGGAGAGGCTGATCCAGGAGCTGGAAGACGTGGAAAAGAACCGCAAGATAGTGGCAGAA

AATCTCGAGAAGGTCCAGGCTGAGGCTGAGAGACTGGATCAGGAGGAAGCTCAGTATCAGAGAGAATACA

GTGAATTTAAACGACAGCAGCTGGAGCTGGATGATGAGCTGAAGAGTGTTGAAAACCAGATGCGTTATGC

CCAGACGCAGCTGGATAAGCTGAAGAAAACCAACGTCTTTAATGCAACCTTCCACATCTGGCACAGTGGA

CAGTTTGGCACAATCAATAACTTCAGGCTGGGTCGCCTGCCCAGTGTTCCCGTGGAATGGAATGAGATTA

ATGCTGCTTGGGGCCAGACTGTGTTGCTGCTCCATGCTCTGGCCAATAAGATGGGTCTGAAATTTCAGAG

ATACCGACTTGTTCCTTACGGAAACCATTCATATCTGGAGTCTCTGACAGACAAATCTAAGGAGCTGCCG

TTATACTGTTCTGGGGGGTTGCGGTTTTTCTGGGACAACAAGTTTGACCATGCAATGGTGGCTTTCCTGG

ACTGTGTGCAGCAGTTCAAAGAAGAGGTTGAGAAAGGCGAGACACGTTTTTGTCTTCCCTACAGGATGGA

TGTGGAGAAAGGCAAGATTGAAGACACAGGAGGCAGTGGCGGCTCCTATTCCATCAAAACCCAGTTTAAC

TCTGAGGAGCAGTGGACAAAAGCTCTCAAGTTCATGCTGACGAATCTTAAGTGGGGTCTTGCTTGGGTGT

CCTCACAATTTTATAACAAATGA

>lcl|NM_001290693.1_cds_NP_001277622.1_1 [gene=BECN2] [db_xref=CCDS:CCDS81433.1] [protein=beclin-2] [protein_id=NP_001277622.1] [location=1..1296] [gbkey=CDS]

ATGTCTTCCATCCGCTTCCTGTGCCAGCGCTGCCACCAGGCCCTGAAGCTGAGCGGCTCCTCGGAGTCTA

GGAGCCTCCCTGCAGCCCCGGCGCCCACCTCTGGGCAGGCTGAGCCCGGAGACACCCGGGAGCCCGGCGT

CACCACCAGGGAGGTGACAGACGCTGAGGAGCAACAGGACGGTGCCTCTAGCAGATCCCCTCCAGGCGAT

GGCAGTGTGTCCAAGGGCCATGCCAACATCTTCACCCTGCTGGGGGAGCTTGGCGCCATGCACATGCTCA

GTAGCATCCAGAAGGCAGCTGGTGACATTTTTGACATAGTCTCTGGCCAAGCAGTTGTGGACCATCCCCT

GTGTGAAGAATGCACCGACAGTCTTTTAGAGCAGCTGGACATCCAGCTCGCTCTCACAGAAGCTGACAGT

CAGAACTACCAACGCTGCCTGGAGACCGGGGAGCTGGCGACCAGCGAGGACGAGGCGGCGGCGCTGCGGG

CGGAGCTGCGGGACCTGGAGCTGGAGGAGGCCAGGCTGGTGCAGGAGCTGGAGGATGTGGACAGGAACAA

TGCAAGAGCAGCGGCGGATCTCCAGGCAGCCCAGGCAGAGGCTGCGGAGCTGGACCAGCAGGAGAGGCAG

CACTACAGGGACTACAGTGCCTTGAAGCGGCAGCAGCTGGAACTGCTTGATCAGCTGGGGAACGTGGAGA

ACCAGCTGCAGTATGCCAGGGTCCAGAGGGACCGGCTGAAGGAAATCAACTGTTTCACCGCCACGTTTGA

GATCTGGGTGGAGGGCCCCTTGGGCGTCATCAATAACTTCAGGTTGGGCCGCCTCCCCACTGTCCGTGTG

GGCTGGAATGAGATTAACACTGCCTGGGGACAGGCGGCCTTGCTGCTCCTTACCCTGGCCAATACAATTG

GACTGCAGTTTCAGAGGTATCGACTCATCCCCTGCGGAAACCATTCGTATCTGAAGTCTTTAACAGATGA

CCGCACTGAGCTGCCGTTGTTCTGTTATGGGGGGCAGGATGTTTTCCTCAATAACAAGTATGACCGCGCG

ATGGTGGCCTTCCTGGACTGCATGCAGCAGTTCAAGGAAGAGGCTGAGAAGGGTGAGCTGGGCCTCTCTC

TGCCCTACGGGATCCAGGTGGAGACAGGCCTGATGGAGGACGTTGGCGGCCGAGGGGAATGCTATTCCAT

CAGAACCCATCTGAACACGCAGGAGCTGTGGACAAAGGCACTCAAGTTCATGCTTATAAATTTCAAGTGG

AGTCTCATCTGGGTTGCCTCAAGGTATCAAAAGTAG

>lcl|NM_197966.3_cds_NP_932070.1_1 [gene=BID] [db_xref=CCDS:CCDS13747.1] [protein=BH3-interacting domain death agonist isoform 1] [protein_id=NP_932070.1] [location=295..1020] [gbkey=CDS]

ATGTGCAGCGGTGCTGGGGTCATGATGGCTCGGTGGGCAGCGAGGGGCCGGGCCGGCTGGAGGAGCACAG

TGCGGATTCTGTCGCCACTGGGACACTGTGAACCAGGAGTGAGTCGGAGCTGCCGCGCTGCCCAGGCCAT

GGACTGTGAGGTCAACAACGGTTCCAGCCTCAGGGATGAGTGCATCACAAACCTACTGGTGTTTGGCTTC

CTCCAAAGCTGTTCTGACAACAGCTTCCGCAGAGAGCTGGACGCACTGGGCCACGAGCTGCCAGTGCTGG

CTCCCCAGTGGGAGGGCTACGATGAGCTGCAGACTGATGGCAACCGCAGCAGCCACTCCCGCTTGGGAAG

AATAGAGGCAGATTCTGAAAGTCAAGAAGACATCATCCGGAATATTGCCAGGCACCTCGCCCAGGTCGGG

GACAGCATGGACCGTAGCATCCCTCCGGGCCTGGTGAACGGCCTGGCCCTGCAGCTCAGGAACACCAGCC

GGTCGGAGGAGGACCGGAACAGGGACCTGGCCACTGCCCTGGAGCAGCTGCTGCAGGCCTACCCTAGAGA

CATGGAGAAGGAGAAGACCATGCTGGTGCTGGCCCTGCTGCTGGCCAAGAAGGTGGCCAGTCACACGCCG

TCCTTGCTCCGTGATGTCTTTCACACAACAGTGAATTTTATTAACCAGAACCTACGCACCTACGTGAGGA

GCTTAGCCAGAAATGGGATGGACTGA

>lcl|NM_001168.3_cds_NP_001159.2_1 [gene=BIRC5] [db_xref=CCDS:CCDS11755.1] [protein=baculoviral IAP repeat-containing protein 5 isoform 1] [protein_id=NP_001159.2] [location=65..493] [gbkey=CDS]

ATGGGTGCCCCGACGTTGCCCCCTGCCTGGCAGCCCTTTCTCAAGGACCACCGCATCTCTACATTCAAGA

ACTGGCCCTTCTTGGAGGGCTGCGCCTGCACCCCGGAGCGGATGGCCGAGGCTGGCTTCATCCACTGCCC

CACTGAGAACGAGCCAGACTTGGCCCAGTGTTTCTTCTGCTTCAAGGAGCTGGAAGGCTGGGAGCCAGAT

GACGACCCCATAGAGGAACATAAAAAGCATTCGTCCGGTTGCGCTTTCCTTTCTGTCAAGAAGCAGTTTG

AAGAATTAACCCTTGGTGAATTTTTGAAACTGGACAGAGAAAGAGCCAAGAACAAAATTGCAAAGGAAAC

CAACAATAAGAAGAAAGAATTTGAGGAAACTGCGGAGAAAGTGCGCCGTGCCATCGAGCAGCTGGCTGCC

ATGGATTGA

>lcl|NM_016252.4_cds_NP_057336.3_1 [gene=BIRC6] [db_xref=CCDS:CCDS33175.2] [protein=baculoviral IAP repeat-containing protein 6 isoform 1] [protein_id=NP_057336.3] [location=140..14713] [gbkey=CDS]

ATGGTGACTGGTGGTGGTGCTGCACCTCCCGGGACTGTCACTGAGCCGCTTCCCAGTGTGATTGTGCTGA

GCGCAGGCCGGAAGATGGCGGCTGCGGCTGCGGCGGCCTCGGGCCCCGGCTGCTCCTCGGCGGCGGGGGC

GGGGGCGGCCGGGGTCTCAGAGTGGCTGGTGCTGCGGGACGGCTGCATGCACTGCGACGCCGACGGGCTG

CACAGCCTGTCCTACCACCCTGCGCTCAACGCCATCCTGGCCGTCACTAGCCGCGGGACCATCAAAGTCA

TCGACGGCACCTCGGGGGCCACACTGCAGGCCTCCGCGCTCAGTGCTAAACCAGGTGGACAGGTGAAATG

TCAGTATATCTCTGCTGTGGATAAAGTTATATTTGTGGATGATTATGCAGTAGGGTGTAGGAAGGACCTT

AATGGAATCTTGTTGTTAGACACTGCTCTGCAAACTCCAGTTTCAAAGCAGGATGATGTGGTTCAGCTTG

AATTACCCGTTACAGAGGCACAGCAGCTCTTATCAGCATGTTTAGAAAAGGTAGATATTTCTAGTACAGA

GGGTTATGATTTGTTCATCACACAGCTCAAAGATGGTTTAAAAAATACATCTCATGAGACTGCAGCAAAC

CACAAAGTTGCTAAGTGGGCCACAGTTACATTTCATCTTCCTCATCATGTGTTGAAGTCCATTGCCAGTG

CCATTGTAAATGAACTCAAGAAAATAAATCAAAATGTTGCTGCCTTACCTGTGGCGTCCTCAGTGATGGA

CAGATTGTCTTACCTCTTACCTAGTGCACGTCCAGAACTCGGAGTGGGGCCAGGCCGTTCTGTAGACAGA

TCACTGATGTATAGTGAAGCTAACAGACGGGAGACATTTACCTCATGGCCTCATGTAGGCTATAGGTGGG

CACAACCAGATCCCATGGCTCAAGCTGGATTTTATCATCAGCCTGCCTCATCTGGAGATGATAGAGCCAT

GTGTTTTACTTGTAGTGTATGCCTCGTTTGTTGGGAACCTACTGATGAACCTTGGTCTGAACACGAAAGA

CATTCCCCAAACTGCCCATTTGTGAAAGGTGAGCACACACAGAATGTGCCATTGTCAGTCACTCTTGCAA

CAAGTCCTGCACAGTTTCCTTGTACGGATGGAACTGACAGAATATCTTGCTTTGGGTCGGGGAGCTGCCC

TCATTTTCTAGCTGCTGCAACTAAACGAGGAAAGATCTGCATATGGGATGTTTCCAAACTTATGAAGGTG

CACTTAAAGTTTGAAATTAATGCCTATGATCCAGCAATTGTACAACAGCTTATTCTATCAGGAGACCCAA

GCTCAGGAGTTGATTCAAGGAGACCAACTTTGGCGTGGCTGGAGGACTCCTCTAGTTGCTCAGATATACC

AAAATTGGAAGGAGATAGTGATGATTTACTGGAGGATTCAGACAGTGAAGAGCATTCCAGATCAGATTCT

GTGACAGGGCATACATCACAGAAGGAAGCCATGGAAGTAAGCCTTGATATAACAGCACTCAGCATTCTCC

AACAGCCAGAAAAACTTCAGTGGGAGATTGTTGCAAATGTGCTTGAAGATACTGTTAAGGATCTTGAAGA

ACTTGGGGCAAATCCTTGTTTAACAAACTCTAAGAGTGAAAAGACAAAGGAAAAGCACCAGGAGCAACAC

AACATTCCTTTTCCATGTTTATTAGCTGGAGGTTTATTAACATATAAATCTCCTGCTACCTCACCCATTA

GTAGTAATTCTCACAGGTCACTGGATGGTTTAAGCAGAACTCAGGGTGAAAGTATATCAGAACAAGGGTC

AACTGACAATGAATCCTGCACTAATTCAGAACTAAATTCTCCTCTGGTAAGGAGGACTTTACCGGTTTTG

CTTCTTTATAGCATCAAGGAATCTGATGAGAAAGCAGGAAAGATCTTTTCACAGATGAACAATATTATGA

GTAAAAGTTTGCATGATGATGGTTTTACTGTTCCACAGATTATTGAAATGGAGCTGGATAGTCAGGAGCA

GTTGTTATTGCAGGATCCTCCTGTGACTTACATTCAGCAATTTGCAGATGCAGCAGCCAACCTTACCTCT

CCGGATTCTGAGAAGTGGAACTCTGTGTTTCCCAAGCCTGGGACTTTGGTTCAGTGCTTGAGGCTGCCAA

AGTTTGCAGAGGAGGAGAATCTTTGTATAGACTCAATAACTCCTTGTGCTGACGGAATTCATTTGTTGGT

AGGACTGCGGACATGCCCTGTTGAATCCTTGAGTGCAATAAATCAAGTAGAGGCCTTGAATAATTTAAAT

AAATTAAACTCTGCACTATGTAATAGACGGAAAGGTGAGCTGGAATCAAATCTTGCTGTAGTGAATGGTG

CAAATATTAGTGTAATCCAACATGAATCACCAGCAGATGTACAGACTCCTTTAATAATTCAGCCTGAGCA

GAGGAATGTTAGTGGTGGATATTTAGTGCTTTATAAAATGAATTATGCCACTCGGATAGTGACTTTAGAA

GAGGAGCCAATAAAAATACAACATATCAAAGATCCCCAGGACACAATTACCTCGCTCATTTTGCTTCCAC

CCGATATATTGGATAATCGAGAGGATGACTGTGAGGAACCTATTGAGGACATGCAGTTAACCTCAAAGAA

TGGTTTTGAGAGAGAAAAAACGTCTGACATTTCTACTCTTGGACACCTGGTAATAACCACTCAGGGAGGA

TATGTAAAAATACTAGATCTTTCAAACTTTGAAATTTTGGCCAAAGTGGAGCCTCCCAAAAAGGAGGGCA

CTGAGGAACAGGACACATTTGTTTCTGTGATTTACTGTTCTGGCACAGACAGGCTGTGTGCATGCACCAA

AGGTGGTGAGCTTCATTTTCTCCAAATTGGAGGAACCTGTGATGATATTGATGAAGCTGATATACTAGTG

GATGGATCTCTTTCTAAAGGAATAGAACCATCTTCAGAAGGTTCCAAACCTTTATCAAATCCTTCAAGTC

CTGGCATTTCAGGAGTTGATTTATTGGTGGACCAGCCATTCACCCTTGAAATCTTGACATCCCTAGTGGA

GCTAACCCGCTTTGAGACTTTGACTCCAAGGTTTTCAGCGACTGTTCCTCCATGCTGGGTAGAAGTTCAA

CAAGAACAGCAGCAAAGGAGGCATCCTCAACATTTGCATCAGCAACACCATGGTGATGCTGCTCAGCATA

CTCGAACTTGGAAACTACAGACCGACAGCAACAGCTGGGATGAACATGTATTTGAATTAGTACTACCTAA

AGCTTGTATGGTTGGACATGTGGACTTCAAATTCGTTTTGAACTCAAACATCACCAATATTCCACAGATA

CAAGTGACACTGCTGAAAAATAAAGCTCCAGGATTAGGGAAAGTCAATGCTCTTAACATTGAAGTGGAAC

AAAATGGGAAACCGTCCCTGGTTGATTTGAATGAAGAAATGCAGCACATGGATGTAGAGGAATCACAGTG

TCTTAGATTATGTCCATTTTTGGAGGATCATAAAGAAGACATTCTATGTGGGCCAGTATGGCTTGCTAGT

GGCCTTGATCTATCAGGGCATGCTGGAATGTTGACGTTAACAAGCCCCAAACTTGTTAAAGGTATGGCAG

GAGGAAAATATCGTTCGTTTTTAATCCATGTCAAGGCAGTGAATGAAAGAGGAACAGAAGAGATTTGTAA

TGGTGGTATGCGTCCTGTAGTAAGGCTTCCATCCCTAAAACACCAGAGTAACAAGGGTTATTCACTTGCT

TCACTTTTGGCTAAAGTTGCAGCAGGCAAGGAAAAATCATCTAATGTTAAGAATGAAAATACAAGTGGCA

CCCGTAAATCTGAAAACCTCCGGGGCTGTGATTTACTTCAAGAGGTCTCAGTCACCATTCGAAGATTTAA

GAAAACCTCAATTTCTAAGGAAAGAGTGCAACGATGTGCCATGTTACAGTTTTCAGAATTTCATGAGAAG

CTTCTTAATACTCTTTGCAGAAAAACAGATGATGGCCAGATCACAGAACATGCCCAGAGCCTTGTGTTGG

ATACTCTCTGTTGGTTAGCTGGAGTTCATTCAAATGGACCCGGAAGCTCAAAGGAAGGAAATGAGAACCT

ACTTTCAAAAACACGAAAATTTCTGTCAGACATCGTACGTGTTTGCTTCTTTGAGGCAGGACGAAGTATA

GCCCATAAGTGTGCCCGATTTCTAGCCTTGTGCATTAGTAATGGCAAATGTGACCCATGTCAACCAGCAT

TTGGACCTGTTCTGTTGAAGGCTTTACTTGATAATATGTCATTTTTACCTGCAGCAACAACTGGTGGTTC

TGTCTATTGGTATTTTGTCTTACTGAATTATGTGAAAGATGAAGATCTGGCTGGATGCAGTACAGCTTGT

GCATCTTTGCTTACTGCAGTGTCCAGACAGTTACAGGACAGGCTAACACCAATGGAGGCTTTACTTCAGA

CAAGATATGGATTATATAGCTCACCATTTGATCCAGTCCTCTTTGATTTGGAGATGAGTGGCTCTTCTTG

TAAAAATGTTTATAACAGCAGCATTGGTGTCCAGTCAGATGAAATTGATTTATCAGATGTCCTTTCAGGA

AATGGAAAGGTCAGTAGTTGCACAGCTGCTGAGGGTAGTTTCACATCTCTCACTGGACTTTTGGAAGTTG

AACCTCTGCACTTTACTTGTGTGTCAACTAGTGATGGAACCAGAATAGAAAGGGATGATGCAATGAGTTC

CTTCGGGGTTACTCCTGCAGTAGGTGGACTATCATCTGGGACAGTTGGGGAAGCCTCGACAGCCCTGAGT

TCAGCAGCCCAGGTAGCTTTGCAGTCTCTCTCTCATGCAATGGCTTCAGCCGAGCAACAGCTACAGGTGC

TGCAAGAGAAACAGCAGCAGCTTTTGAAGCTTCAGCAACAGAAAGCAAAGCTGGAAGCCAAGTTACATCA

GACAACAGCTGCAGCAGCTGCAGCAGCATCAGCAGTAGGTCCTGTTCACAACTCTGTGCCTTCCAACCCA

GTGGCTGCCCCTGGATTCTTCATTCATCCATCTGATGTTATTCCACCCACTCCAAAAACAACACCTCTTT

TTATGACTCCACCACTCACTCCACCCAATGAAGCAGTTTCCGTTGTGATTAATGCCGAACTTGCACAGCT

TTTCCCAGGCTCAGTCATTGATCCCCCAGCAGTCAATCTTGCTGCACATAACAAAAATTCCAACAAGTCC

AGAATGAATCCACTTGGTTCTGGTCTAGCCCTTGCAATTTCTCATGCTTCACATTTTCTTCAACCTCCGC

CTCACCAGTCCATTATTATAGAGCGAATGCATTCAGGAGCAAGAAGATTTGTGACCTTGGATTTTGGGAG

GCCTATATTGTTGACTGATGTATTGATTCCCACTTGTGGAGACTTGGCCTCTTTGTCAATTGACATTTGG

ACATTAGGAGAAGAGGTGGATGGAAGGCGGTTGGTAGTGGCAACTGATATAAGCACTCATTCACTAATTC

TTCATGACTTAATACCACCTCCCGTGTGCAGATTCATGAAGATCACTGTTATTGGACGTTACGGGAGTAC

AAATGCCAGAGCCAAAATCCCATTAGGATTTTACTATGGTCATACCTACATCTTGCCTTGGGAAAGTGAA

CTGAAGTTAATGCATGATCCTCTAAAGGGAGAGGGAGAATCTGCAAACCAGCCAGAAATTGACCAGCATT

TAGCAATGATGGTTGCTTTGCAGGAGGATATACAGTGCAGGTATAACTTGGCTTGTCATCGTCTGGAAAC

CCTTTTGCAAAGTATTGATCTTCCTCCTCTAAACAGTGCTAACAATGCACAGTACTTTTTACGAAAACCA

GATAAGGCAGTTGAGGAAGACAGTAGGGTTTTTTCTGCTTATCAAGATTGTATTCAGCTACAGCTTCAAC

TAAATTTGGCTCATAATGCAGTGCAGAGGCTCAAAGTGGCGCTAGGTGCAAGCCGGAAGATGTTGAGTGA

AACATCAAATCCAGAAGATTTAATTCAGACATCTTCCACAGAGCAGTTACGTACTATCATCAGATATTTA

CTGGACACTTTGCTCAGCCTGCTTCATGCTTCTAATGGACACTCTGTTCCTGCAGTTTTGCAGAGCACAT

TTCATGCCCAGGCCTGTGAAGAGCTCTTTAAACACTTGTGCATCAGTGGAACCCCAAAGATACGGTTACA

TACTGGTCTTCTTCTTGTTCAACTGTGTGGTGGTGAAAGGTGGTGGGGTCAATTTCTTTCTAATGTCCTT

CAGGAATTGTACAATTCGGAACAGCTTCTCATCTTTCCACAGGATAGGGTCTTCATGTTACTTTCCTGCA

TTGGTCAAAGATCACTTAGTAATAGTGGAGTATTAGAAAGCTTACTTAATCTCTTGGATAATTTATTGTC

ACCTCTTCAGCCACAGTTACCCATGCATAGGAGGACAGAAGGAGTACTAGATATTCCCATGATCAGTTGG

GTTGTTATGCTGGTGTCCAGGTTGCTGGATTATGTGGCAACTGTTGAAGATGAAGCAGCAGCTGCAAAGA

AACCTTTGAATGGTAATCAGTGGAGTTTTATTAACAATAATCTACACACTCAGAGCTTAAATAGATCTTC

TAAAGGCAGCAGTAGCCTTGATAGATTATATTCCAGAAAAATCAGAAAGCAGCTTGTTCATCATAAACAG

CAACTTAACCTACTAAAAGCAAAGCAGAAGGCATTGGTAGAACAGATGGAAAAAGAAAAAATACAAAGTA

ACAAAGGATCATCATATAAACTCCTGGTAGAACAAGCAAAACTAAAGCAGGCCACTTCAAAGCACTTTAA

GGATTTAATTCGTTTACGTCGGACAGCAGAATGGTCCCGTTCTAATTTAGACACAGAAGTTACAACAGCA

AAAGAAAGTCCTGAGATAGAACCACTTCCATTTACTCTGGCCCATGAGCGTTGTATCTCAGTAGTCCAGA

AACTTGTTCTGTTTCTTCTCTCCATGGACTTTACATGTCATGCAGATCTCTTATTGTTTGTTTGTAAGGT

TCTTGCACGCATTGCAAATGCCACGAGGCCAACTATTCATCTGTGTGAGATTGTGAACGAACCCCAGCTG

GAAAGACTGCTGTTACTTTTGGTTGGAACTGACTTCAATAGAGGAGATATATCTTGGGGTGGTGCTTGGG

CTCAGTATTCCTTAACTTGCATGCTACAAGATATTTTAGCAGGAGAATTACTGGCTCCAGTAGCCGCAGA

AGCCATGGAGGAAGGAACAGTGGGTGATGATGTAGGTGCGACAGCTGGTGACTCTGATGACTCCCTTCAA

CAGTCCTCAGTTCAGTTGCTGGAAACTATAGATGAACCTTTGACACATGACATAACAGGTGCACCTCCTC

TGTCCTCTTTGGAAAAAGATAAAGAAATTGACCTTGAGTTACTTCAGGATCTAATGGAAGTTGACATTGA

TCCTTTAGATATTGATTTGGAAAAGGACCCTCTTGCAGCCAAGGTTTTTAAGCCAATAAGCAGTACATGG

TATGATTATTGGGGTGCTGATTATGGGACCTACAATTACAACCCTTACATTGGAGGTCTGGGAATTCCTG

TAGCAAAGCCACCAGCAAACACGGAGAAGAACGGATCACAGACAGTTAGCGTTTCAGTCTCTCAGGCCCT

GGATGCTCGCCTAGAAGTTGGACTTGAACAGCAAGCAGAACTGATGTTGAAAATGATGTCTACTCTGGAG

GCAGATTCCATTTTACAGGCATTAACAAATACATCTCCTACATTATCACAGTCTCCCACTGGAACAGATG

ATTCACTTCTAGGGGGTTTACAAGCAGCAAACCAAACCAGCCAGCTTATTATACAGTTATCATCTGTCCC

AATGTTAAATGTTTGTTTCAACAAACTTTTTTCCATGCTTCAAGTCCATCATGTTCAGTTGGAGTCACTT

CTCCAATTGTGGCTCACACTGAGCCTGAATTCTAGTTCAACTGGAAACAAAGAAAATGGAGCAGACATAT

TTTTATATAATGCTAATAGGATACCTGTTATTTCATTAAATCAAGCATCAATAACTAGCTTTCTCACAGT

GTTAGCTTGGTATCCCAATACTTTGCTCCGGACATGGTGCCTTGTGCTTCATAGCCTAACACTCATGACA

AACATGCAGCTTAATTCTGGTTCCAGCAGTGCCATTGGAACTCAGGAGAGTACTGCTCATTTGTTGGTTT

CAGATCCAAACCTAATTCATGTATTAGTGAAATTTCTTTCTGGCACCAGTCCACATGGAACAAATCAACA

CAGTCCACAGGTTGGTCCTACAGCTACACAAGCTATGCAAGAATTTCTTACTCGATTACAAGTGCATCTT

TCTTCAACATGTCCTCAGATATTCAGTGAATTTTTGCTCAAGCTAATTCATATACTTTCAACTGAAAGGG

GTGCTTTCCAGACAGGCCAAGGACCTCTCGATGCCCAAGTGAAGCTCTTAGAATTCACTCTGGAGCAGAA

TTTTGAAGTCGTTTCAGTTAGTACTATTTCTGCCGTGATAGAATCGGTTACATTTTTAGTGCACCACTAT

ATCACTTGCTCAGACAAAGTAATGTCAAGAAGTGGATCAGATAGCTCCGTGGGTGCTCGAGCATGCTTTG

GGGGACTCTTTGCCAATCTTATTCGTCCGGGTGATGCAAAAGCAGTTTGTGGCGAAATGACAAGAGATCA

ACTCATGTTTGATTTGTTAAAACTTGTTAACATTTTAGTGCAGCTGCCTCTTTCAGGCAATAGGGAATAC

AGTGCAAGAGTGTCTGTGACCACAAATACAACAGATAGTGTTTCAGATGAAGAAAAAGTCTCAGGAGGCA

AAGATGGCAATGGAAGCAGTACCAGTGTTCAAGGATCGCCTGCATATGTTGCTGACTTAGTCTTAGCCAA

CCAACAAATTATGAGCCAGATTTTGTCTGCTCTGGGCCTGTGTAATAGCAGTGCCATGGCAATGATAATT

GGAGCAAGTGGATTACATCTCACTAAACATGAAAACTTTCATGGTGGGTTGGATGCCATATCAGTTGGGG

ATGGATTATTTACCATACTGACAACCCTTAGTAAAAAAGCTTCTACAGTCCACATGATGCTGCAGCCAAT

TTTAACATACATGGCCTGTGGATATATGGGCAGACAAGGCTCTCTTGCTACTTGCCAGTTATCTGAGCCA

TTATTGTGGTTCATTTTGAGAGTATTGGATACTAGTGATGCCTTGAAAGCATTTCATGATATGGGTGGTG

TTCAGCTCATATGCAATAATATGGTTACTAGTACAAGGGCTATTGTGAACACTGCAAGAAGTATGGTATC

AACTATTATGAAATTTCTTGACTCTGGTCCAAATAAAGCTGTTGACAGCACATTGAAAACAAGAATACTA

GCTTCTGAGCCTGACAATGCTGAAGGGATTCATAACTTTGCACCCCTCGGTACAATCACATCTAGCAGTC

CTACTGCCCAACCAGCTGAAGTGCTATTGCAGGCCACACCTCCTCACAGAAGAGCTCGCTCTGCTGCTTG

GTCCTACATCTTTCTTCCAGAGGAGGCTTGGTGTGACCTTACCATTCACCTTCCTGCAGCAGTGCTGCTT

AAGGAGATACATATCCAGCCTCATCTTGCATCTCTTGCAACCTGCCCTTCCTCAGTGTCTGTTGAAGTAA

GTGCAGATGGGGTAAATATGCTACCTTTGTCCACTCCTGTTGTCACAAGTGGCCTCACCTACATAAAAAT

TCAGCTTGTAAAAGCCGAAGTAGCTTCTGCTGTCTGCCTTAGACTACATCGTCCACGGGATGCCAGCACA

TTAGGCCTTTCACAAATTAAATTATTGGGGCTCACTGCTTTTGGTACCACCTCTTCTGCAACAGTTAATA

ATCCATTCCTTCCATCTGAAGATCAGGTATCCAAAACAAGTATTGGATGGTTACGGTTATTACATCATTG

CCTTACTCACATAAGTGATCTAGAAGGAATGATGGCAAGTGCAGCTGCACCTACTGCTAATCTGCTGCAG

ACTTGTGCGGCCTTATTGATGTCACCTTACTGTGGAATGCATTCACCCAACATCGAGGTTGTGCTTGTAA

AGATAGGACTGCAGTCTACTAGAATTGGCCTGAAGCTCATAGACATTCTCCTGAGAAATTGTGCAGCATC

AGGCAGTGATCCTACAGATTTGAATAGTCCTTTACTTTTTGGAAGACTAAATGGACTCTCTTCTGACTCT

ACGATAGATATTCTTTACCAGCTTGGAACAACTCAGGATCCTGGTACAAAAGACAGAATTCAGGCCTTGT

TAAAATGGGTTAGTGATTCTGCAAGAGTGGCTGCTATGAAGAGAAGTGGCAGGATGAACTACATGTGTCC

TAACTCCTCAACAGTAGAGTATGGTCTTCTGATGCCATCTCCTTCTCATTTGCACTGTGTAGCAGCCATT

CTGTGGCATAGTTATGAGCTGCTTGTAGAATATGACTTACCAGCACTCCTGGACCAAGAGCTCTTTGAGT

TACTTTTTAATTGGTCCATGTCTCTTCCCTGCAATATGGTTTTGAAGAAAGCTGTTGACAGTCTACTTTG

CTCAATGTGTCACGTACACCCAAACTATTTTTCTTTGCTCATGGGCTGGATGGGAATTACCCCTCCTCCA

GTGCAATGTCATCATAGACTGTCCATGACAGATGATAGCAAAAAGCAGGATCTTAGTTCATCTTTAACAG

ATGACTCTAAAAATGCACAAGCACCTCTCGCATTAACTGAATCACATTTGGCTACCCTTGCTTCCTCTTC

TCAATCTCCTGAAGCTATTAAACAATTACTAGACTCAGGTTTGCCTTCTCTTCTTGTGAGGAGTCTGGCT

AGTTTCTGCTTTAGCCACATTTCTAGCTCAGAAAGCATTGCCCAGTCAATAGATATTTCCCAGGACAAAC

TCAGGCGCCATCATGTCCCACAACAATGTAATAAGATGCCTATCACAGCCGACCTAGTTGCTCCTATTCT

TAGGTTTTTGACAGAAGTTGGCAATAGCCATATTATGAAAGATTGGCTTGGTGGTTCTGAAGTCAATCCA

CTATGGACAGCACTTCTGTTTTTATTGTGTCACTCTGGGTCCACTTCTGGAAGCCATAATTTAGGTGCAC

AACAGACCAGTGCAAGATCAGCTTCTCTTTCTTCAGCTGCTACAACAGGACTGACTACTCAACAGCGCAC

AGCAATTGAGAATGCAACTGTTGCGTTCTTTCTACAGTGCATTTCATGCCATCCTAATAATCAAAAGCTG

ATGGCACAGGTTCTTTGTGAACTATTTCAGACATCTCCTCAAAGAGGGAACCTTCCAACATCTGGGAACA

TTTCAGGGTTTATACGAAGATTATTTTTACAGTTGATGCTGGAAGATGAGAAAGTGACAATGTTTCTTCA

GTCTCCATGTCCACTGTACAAAGGTAGAATTAATGCTACTAGCCACGTCATCCAGCATCCAATGTATGGA

GCAGGCCACAAATTCCGTACTCTTCATTTGCCAGTCTCAACAACATTATCAGATGTTCTTGACAGAGTGT

CAGATACTCCAAGTATCACAGCTAAATTAATTAGTGAACAAAAAGATGACAAAGAAAAGAAAAACCATGA

AGAGAAAGAAAAAGTTAAAGCGGAAAATGGATTTCAAGACAATTACAGTGTTGTTGTTGCCTCTGGGCTG

AAGTCTCAATCTAAACGTGCTGTGTCAGCTACACCACCTCGCCCACCATCCAGGAGGGGGAGGACAATAC

CTGATAAAATAGGAAGTACTTCAGGAGCAGAGGCTGCCAACAAAATAATTACTGTCCCAGTGTTTCACCT

GTTTCACAAACTCTTGGCAGGCCAGCCATTGCCAGCTGAAATGACACTTGCCCAGCTTTTAACTCTCCTA

TATGACCGAAAACTTCCTCAGGGTTACCGCTCAATAGATCTGACTGTTAAATTGGGATCAAGAGTTATAA

CAGACCCCAGTCTATCAAAAACAGATTCTTATAAAAGACTACACCCTGAAAAAGATCATGGAGACTTACT

TGCTAGCTGTCCAGAAGATGAGGCTCTCACTCCAGGTGATGAATGCATGGATGGGATACTGGATGAATCT

TTGCTTGAAACCTGTCCAATTCAGTCACCATTACAAGTTTTTGCAGGAATGGGTGGACTGGCTCTTATTG

CTGAAAGACTACCCATGCTATATCCAGAAGTAATTCAACAGGTGAGTGCTCCAGTTGTAACATCTACCAC

TCAGGAAAAGCCGAAGGATAGCGATCAGTTTGAATGGGTGACCATTGAACAGTCAGGGGAGTTAGTTTAT

GAAGCACCAGAAACTGTTGCGGCTGAACCTCCACCTATCAAGTCAGCAGTACAGACCATGTCTCCCATAC

CTGCCCATTCTTTGGCTGCTTTTGGATTATTTCTTCGTCTTCCGGGCTATGCGGAAGTGCTACTGAAAGA

GAGAAAACATGCCCAGTGCCTTCTTCGATTGGTATTGGGAGTGACAGATGATGGAGAAGGAAGTCATATT

CTTCAATCTCCATCAGCCAATGTGCTTCCAACCCTTCCTTTCCACGTCCTTCGTAGCTTGTTTAGCACTA

CACCTTTGACAACTGATGATGGTGTACTTCTAAGGCGGATGGCATTGGAAATTGGAGCCTTACACCTCAT

TCTTGTCTGTCTCTCTGCTTTGAGCCACCATTCCCCACGAGTTCCAAACTCTAGCGTGAATCAAACTGAG

CCACAGGTGTCAAGCTCTCATAACCCTACATCAACAGAAGAACAACAGTTATATTGGGCCAAAGGGACTG

GCTTTGGAACAGGCTCTACAGCTTCTGGGTGGGATGTGGAACAAGCCTTAACTAAGCAAAGGCTGGAAGA

GGAACATGTTACCTGCCTTCTGCAGGTTCTTGCCAGTTACATAAATCCCGTCAGTAGTGCGGTAAATGGA

GAAGCTCAGTCATCTCATGAGACTAGAGGGCAGAACAGTAATGCCCTTCCTTCTGTACTTCTCGAGCTTC

TCAGTCAGTCCTGCCTCATCCCAGCCATGTCATCTTATCTACGAAATGATTCAGTTCTGGACATGGCAAG

ACATGTGCCACTCTATCGGGCACTGCTGGAATTGCTTCGGGCCATTGCTTCTTGTGCTGCCATGGTGCCC

CTATTGTTGCCCCTTTCTACAGAGAACGGTGAAGAGGAAGAAGAACAGTCAGAATGTCAAACTTCTGTTG

GTACATTGTTAGCCAAAATGAAGACCTGTGTTGATACCTATACCAACCGTTTAAGATCTAAAAGGGAAAA

TGTTAAAACAGGAGTAAAACCAGATGCGTCTGATCAAGAACCAGAAGGACTTACTCTTTTGGTACCAGAC

ATCCAAAAGACTGCTGAGATAGTTTATGCAGCCACCACCAGTTTGCGGCAAGCAAATCAGGAAAAAAAAC

TGGGTGAATACTCCAAGAAGGCGGCTATGAAACCCAAACCTTTGTCAGTATTAAAGTCACTTGAAGAAAA

ATATGTGGCTGTTATGAAGAAATTACAGTTTGATACGTTTGAAATGGTTTCTGAAGATGAAGATGGGAAA

TTGGGATTTAAAGTAAATTACCACTACATGTCTCAGGTGAAAAATGCTAATGATGCGAACAGTGCTGCCA

GAGCTCGCCGCCTTGCCCAGGAAGCTGTGACGCTTTCAACCTCACTGCCTCTGTCTTCATCCTCTAGTGT

GTTTGTACGCTGTGATGAGGAGCGACTTGATATCATGAAGGTTCTAATAACTGGTCCAGCGGACACCCCT

TATGCAAATGGCTGCTTTGAGTTTGATGTGTATTTTCCTCAAGATTATCCCAGTTCACCCCCTCTTGTGA

ATCTAGAGACAACTGGTGGTCATAGCGTGCGATTCAATCCAAACCTTTATAATGATGGCAAGGTTTGTTT

AAGCATCTTAAACACGTGGCATGGAAGACCAGAAGAGAAGTGGAATCCTCAGACCTCAAGCTTTTTGCAA

GTGTTGGTGTCTGTCCAGTCCCTTATATTAGTAGCTGAGCCTTATTTTAATGAACCGGGATATGAACGGT

CTAGAGGCACTCCCAGTGGCACACAGAGTTCTCGAGAATATGATGGAAACATTCGACAAGCAACAGTTAA

GTGGGCAATGCTAGAACAAATCAGAAACCCTTCACCATGTTTTAAAGAGGTAATACACAAACATTTTTAC

TTGAAAAGAGTTGAGATAATGGCCCAATGTGAGGAGTGGATTGCGGATATCCAGCAGTACAGCAGTGATA

AGCGGGTAGGCAGGACTATGTCTCACCATGCAGCAGCTCTCAAGCGTCACACTGCTCAGCTCCGCGAAGA

GTTGCTGAAACTTCCCTGCCCTGAAGGCTTGGATCCTGACACTGACGATGCCCCAGAGGTGTGCAGAGCC

ACAACAGGTGCTGAGGAGACTCTAATGCATGATCAGGTTAAACCCAGCAGCAGCAAAGAACTCCCCAGTG

ACTTCCAGTTATGA

>lcl|NM_013979.3_cds_NP_053582.2_1 [gene=BNIP1] [db_xref=CCDS:CCDS4385.1] [protein=vesicle transport protein SEC20 isoform BNIP1-b] [protein_id=NP_053582.2] [location=16..831] [gbkey=CDS]

ATGGCGGCTCCCCAAGACGTCCACGTCCGGATCTGTAACCAAGAGATTGTCAAATTTGACCTGGAGGTGA

AGGCGCTTATTCAGGATATCCGTGATTGTTCAGGACCCTTAAGTGCTCTTACTGAACTGAATACTAAAGT

AAAAGAGAAATTTCAACAGTTGCGTCACAGAATACAGCCAGTTCTCTATCAAAGGGCATTTATTTGGACT

GCTTCCACATTTTTTTTTAAGCTAACTTATTCCCTGACAGACTTTTCTTCAACTCAGCATGACTTCAACT

CTCCAACTACACCTGTTACCTTCAGTGACCTGGAGCAGTTGGCTAAAGAGCAAGACAAAGAATCAGAGAA

ACAACTTCTACTCCAGGAAGTGGAGAATCACAAAAAGCAGATGCTCAGCAATCAGGCCTCATGGAGGAAA

GCTAATCTCACCTGCAAAATTGCAATCGACAATCTAGAGAAAGCAGAACTTCTTCAGGGAGGAGATCTCT

TAAGGCAAAGGAAAACCACCAAAGAGAGCCTGGCCCAGACATCCAGTACCATCACTGAGAGCCTCATGGG

GATCAGCAGGATGATGGCCCAGCAGGTCCAGCAGAGCGAGGAGGCCATGCAGTCTCTAGTCACTTCTTCA

CGAACGATCCTGGATGCAAATGAAGAATTTAAGTCCATGTCGGGCACCATCCAGCTGGGCCGGAAGCTTA

TCACAAAATACAATCGCCGGGAGCTGACGGACAAGCTTCTCATCTTCCTTGCGCTAGCCCTGTTTCTTGC

TACGGTCCTCTATATTGTGAAAAAGCGGCTCTTTCCATTTTTGTGA

>lcl|NM_004052.4_cds_NP_004043.4_1 [gene=BNIP3] [db_xref=CCDS:CCDS7663.2] [protein=BCL2/adenovirus E1B 19 kDa protein-interacting protein 3] [protein_id=NP_004043.4] [location=118..702] [gbkey=CDS]

ATGTCGCAGAACGGAGCGCCCGGGATGCAGGAGGAGAGCCTGCAGGGCTCCTGGGTAGAACTGCACTTCA

GCAATAATGGGAACGGGGGCAGCGTTCCAGCCTCGGTTTCTATTTATAATGGAGACATGGAAAAAATACT

GCTGGACGCACAGCATGAGTCTGGACGGAGTAGCTCCAAGAGCTCTCACTGTGACAGCCCACCTCGCTCG

CAGACACCACAAGATACCAACAGAGCTTCTGAAACAGATACCCATAGCATTGGAGAGAAAAACAGCTCAC

AGTCTGAGGAAGATGATATTGAAAGAAGGAAAGAAGTTGAAAGCATCTTGAAGAAAAACTCAGATTGGAT

ATGGGATTGGTCAAGTCGGCCGGAAAATATTCCCCCCAAGGAGTTCCTCTTTAAACACCCGAAGCGCACG

GCCACCCTCAGCATGAGGAACACGAGCGTCATGAAGAAAGGGGGCATATTCTCTGCAGAATTTCTGAAAG

TTTTCCTTCCATCTCTGCTGCTCTCTCATTTGCTGGCCATCGGATTGGGGATCTATATTGGAAGGCGTCT

GACAACCTCCACCAGCACCTTTTGA

>lcl|NM_004331.3_cds_NP_004322.1_1 [gene=BNIP3L] [db_xref=CCDS:CCDS6050.1] [protein=BCL2/adenovirus E1B 19 kDa protein-interacting protein 3-like isoform 1] [protein_id=NP_004322.1] [location=78..737] [gbkey=CDS]

ATGTCGTCCCACCTAGTCGAGCCGCCGCCGCCCCTGCACAACAACAACAACAACTGCGAGGAAAATGAGC

AGTCTCTGCCCCCGCCGGCCGGCCTCAACAGTTCCTGGGTGGAGCTACCCATGAACAGCAGCAATGGCAA

TGATAATGGCAATGGGAAAAATGGGGGGCTGGAACACGTACCATCCTCATCCTCCATCCACAATGGAGAC

ATGGAGAAGATTCTTTTGGATGCACAACATGAATCAGGACAGAGTAGTTCCAGAGGCAGTTCTCACTGTG

ACAGCCCTTCGCCACAAGAAGATGGGCAGATCATGTTTGATGTGGAAATGCACACCAGCAGGGACCATAG

CTCTCAGTCAGAAGAAGAAGTTGTAGAAGGAGAGAAGGAAGTCGAGGCTTTGAAGAAAAGTGCGGACTGG

GTATCAGACTGGTCCAGTAGACCCGAAAACATTCCACCCAAGGAGTTCCACTTCAGACACCCTAAACGTT

CTGTGTCTTTAAGCATGAGGAAAAGTGGAGCCATGAAGAAAGGGGGTATTTTCTCCGCAGAATTTCTGAA

GGTGTTCATTCCATCTCTCTTCCTTTCTCATGTTTTGGCTTTGGGGCTAGGCATCTATATTGGAAAGCGA

CTGAGCACACCCTCTGCCAGCACCTACTGA

>lcl|NM_005831.5_cds_NP_005822.1_1 [gene=CALCOCO2] [db_xref=CCDS:CCDS11538.1] [protein=calcium-binding and coiled-coil domain-containing protein 2 isoform 3] [protein_id=NP_005822.1] [location=55..1395] [gbkey=CDS]

ATGGAGGAGACCATCAAAGATCCCCCCACATCAGCTGTCTTGCTGGATCACTGTCATTTCTCTCAGGTCA

TCTTTAACAGTGTGGAGAAGTTCTACATCCCTGGAGGGGACGTCACATGTCATTATACCTTCACCCAGCA

TTTCATCCCTCGTCGAAAGGATTGGATTGGCATCTTTAGAGTGGGGTGGAAGACAACCCGTGAGTATTAC

ACCTTCATGTGGGTTACTTTGCCCATTGACCTAAACAACAAATCAGCTAAACAGCAGGAAGTCCAATTCA

AAGCTTACTACCTGCCCAAGGATGATGAGTATTACCAGTTCTGCTATGTGGATGAGGATGGTGTGGTCCG

GGGAGCAAGTATTCCTTTCCAATTCCGTCCAGAAAATGAGGAAGACATCCTGGTTGTTACCACTCAGGGA

GAGGTGGAAGAGATTGAGCAGCACAACAAGGAGCTTTGCAAAGAAAACCAGGAGCTGAAGGACAGCTGTA

TCAGCCTCCAGAAGCAGAACTCAGACATGCAGGCTGAGCTCCAAAAGAAGCAGGAGGAGCTAGAAACCCT

ACAGAGCATCAATAAGAAGTTGGAACTGAAAGTGAAAGAACAGAAGGACTATTGGGAGACAGAGCTGCTT

CAACTGAAAGAACAAAACCAGAAGATGTCCTCAGAAAATGAGAAGATGGGAATCAGAGTGGATCAGCTTC

AGGCCCAGCTGTCAACTCAAGAGAAAGAAATGGAGAAGCTTGTTCAGGGAGATCAAGATAAGACAGAGCA

GTTAGAGCAGCTGAAAAAGGAAAATGACCACCTCTTTCTCAGTTTAACTGAACAGAGGAAGGACCAGAAG

AAGCTCGAGCAGACAGTGGAGCAAATGAAGCAGAATGAAACTACTGCAATGAAGAAACAACAGGAATTAA

TGGATGAAAACTTTGACCTGTCAAAAAGACTGAGTGAGAACGAAATTATATGTAATGCTCTGCAGAGACA

GAAAGAGAGATTGGAAGGAGAAAATGATCTTTTGAAGAGGGAGAACAGCAGATTGCTCAGTTACATGGGT

CTGGATTTTAATTCTTTGCCGTATCAAGTACCTACTTCAGATGAAGGAGGCGCAAGACAAAATCCAGGAC

TTGCCTATGGAAACCCATATTCTGGTATCCAAGAAAGTTCTTCCCCCAGCCCGCTCTCCATCAAGAAATG

CCCTATCTGCAAAGCAGATGATATTTGTGATCACACCTTGGAGCAACAGCAGATGCAGCCCCTTTGTTTC

AATTGTCCAATTTGTGACAAGATCTTCCCAGCTACAGAGAAGCAGATCTTTGAAGACCACGTGTTCTGCC

ACTCTCTCTGA

>lcl|NM_172226.3_cds_NP_757380.1_1 [gene=CAMKK2] [db_xref=CCDS:CCDS9218.1] [protein=calcium/calmodulin-dependent protein kinase kinase 2 isoform 2] [protein_id=NP_757380.1] [location=132..1757] [gbkey=CDS]

ATGTCATCATGTGTCTCTAGCCAGCCCAGCAGCAACCGGGCCGCCCCCCAGGATGAGCTGGGGGGCAGGG

GCAGCAGCAGCAGCGAAAGCCAGAAGCCCTGTGAGGCCCTGCGGGGCCTCTCATCCTTGAGCATCCACCT

GGGCATGGAGTCCTTCATTGTGGTCACCGAGTGTGAGCCGGGCTGTGCTGTGGACCTCGGCTTGGCGCGG

GACCGGCCCCTGGAGGCCGATGGCCAAGAGGTCCCCCTTGACACCTCCGGGTCCCAGGCCCGGCCCCACC

TCTCCGGTCGCAAGCTGTCTCTGCAAGAGCGGTCCCAGGGTGGGCTGGCAGCCGGTGGCAGCCTGGACAT

GAACGGACGCTGCATCTGCCCGTCCCTGCCCTACTCACCCGTCAGCTCCCCGCAGTCCTCGCCTCGGCTG

CCCCGGCGGCCGACAGTGGAGTCTCACCACGTCTCCATCACGGGTATGCAGGACTGTGTGCAGCTGAATC

AGTATACCCTGAAGGATGAAATTGGAAAGGGCTCCTATGGTGTCGTCAAGTTGGCCTACAATGAAAATGA

CAATACCTACTATGCAATGAAGGTGCTGTCCAAAAAGAAGCTGATCCGGCAGGCCGGCTTTCCACGTCGC

CCTCCACCCCGAGGCACCCGGCCAGCTCCTGGAGGCTGCATCCAGCCCAGGGGCCCCATTGAGCAGGTGT

ACCAGGAAATTGCCATCCTCAAGAAGCTGGACCACCCCAATGTGGTGAAGCTGGTGGAGGTCCTGGATGA

CCCCAATGAGGACCATCTGTACATGGTGTTCGAACTGGTCAACCAAGGGCCCGTGATGGAAGTGCCCACC

CTCAAACCACTCTCTGAAGACCAGGCCCGTTTCTACTTCCAGGATCTGATCAAAGGCATCGAGTACTTAC

ACTACCAGAAGATCATCCACCGTGACATCAAACCTTCCAACCTCCTGGTCGGAGAAGATGGGCACATCAA

GATCGCTGACTTTGGTGTGAGCAATGAATTCAAGGGCAGTGACGCGCTCCTCTCCAACACCGTGGGCACG

CCCGCCTTCATGGCACCCGAGTCGCTCTCTGAGACCCGCAAGATCTTCTCTGGGAAGGCCTTGGATGTTT

GGGCCATGGGTGTGACACTATACTGCTTTGTCTTTGGCCAGTGCCCATTCATGGACGAGCGGATCATGTG

TTTACACAGTAAGATCAAGAGTCAGGCCCTGGAATTTCCAGACCAGCCCGACATAGCTGAGGACTTGAAG

GACCTGATCACCCGTATGCTGGACAAGAACCCCGAGTCGAGGATCGTGGTGCCGGAAATCAAGCTGCACC

CCTGGGTCACGAGGCATGGGGCGGAGCCGTTGCCGTCGGAGGATGAGAACTGCACGCTGGTCGAAGTGAC

TGAAGAGGAGGTCGAGAACTCAGTCAAACACATTCCCAGCTTGGCAACCGTGATCCTGGTGAAGACCATG

ATACGTAAACGCTCCTTTGGGAACCCATTCGAGGGCAGCCGGCGGGAGGAACGCTCACTGTCAGCGCCTG

GAAACTTGCTCACGAAGCAAGGCAGCGAAGACAACCTCCAGGGCACCGACCCGCCCCCCGTGGGGGAGGA

GGAAGTGCTCTTGTGA

>lcl|NM_001024649.2_cds_NP_001019820.1_1 [gene=CANX] [db_xref=CCDS:CCDS4447.1] [protein=calnexin isoform d precursor] [protein_id=NP_001019820.1] [location=87..1865] [gbkey=CDS]

ATGGAAGGGAAGTGGTTGCTGTGTATGTTACTGGTGCTTGGAACTGCTATTGTTGAGGCTCATGATGGAC

ATGATGATGATGTGATTGATATTGAGGATGACCTTGACGATGTCATTGAAGAGGTAGAAGACTCAAAACC

AGATACCACTGCTCCTCCTTCATCTCCCAAGGTTACTTACAAAGCTCCAGTTCCAACAGGGGAAGTATAT

TTTGCTGATTCTTTTGACAGAGGAACTCTGTCAGGGTGGATTTTATCCAAAGCCAAGAAAGACGATACCG

ATGATGAAATTGCCAAATATGATGGAAAGTGGGAGGTAGAGGAAATGAAGGAGTCAAAGCTTCCAGGTGA

TAAAGGACTTGTGTTGATGTCTCGGGCCAAGCATCATGCCATCTCTGCTAAACTGAACAAGCCCTTCCTG

TTTGACACCAAGCCTCTCATTGTTCAGTATGAGGTTAATTTCCAAAATGGAATAGAATGTGGTGGTGCCT

ATGTGAAACTGCTTTCTAAAACACCAGAACTCAACCTGGATCAGTTCCATGACAAGACCCCTTATACGAT

TATGTTTGGTCCAGATAAATGTGGAGAGGACTATAAACTGCACTTCATCTTCCGACACAAAAACCCCAAA

ACGGGTATCTATGAAGAAAAACATGCTAAGAGGCCAGATGCAGATCTGAAGACCTATTTTACTGATAAGA

AAACACATCTTTACACACTAATCTTGAATCCAGATAATAGTTTTGAAATACTGGTTGACCAATCTGTGGT

GAATAGTGGAAATCTGCTCAATGACATGACTCCTCCTGTAAATCCTTCACGTGAAATTGAGGACCCAGAA

GACCGGAAGCCCGAGGATTGGGATGAAAGACCAAAAATCCCAGATCCAGAAGCTGTCAAGCCAGATGACT

GGGATGAAGATGCCCCTGCTAAGATTCCAGATGAAGAGGCCACAAAACCCGAAGGCTGGTTAGATGATGA

GCCTGAGTACGTACCTGATCCAGACGCAGAGAAACCTGAGGATTGGGATGAAGACATGGATGGAGAATGG

GAGGCTCCTCAGATTGCCAACCCTAGATGTGAGTCAGCTCCTGGATGTGGTGTCTGGCAGCGACCTGTGA

TTGACAACCCCAATTATAAAGGCAAATGGAAGCCTCCTATGATTGACAATCCCAGTTACCAGGGAATCTG

GAAACCCAGGAAAATACCAAATCCAGATTTCTTTGAAGATCTGGAACCTTTCAGAATGACTCCTTTTAGT

GCTATTGGTTTGGAGCTGTGGTCCATGACCTCTGACATTTTTTTTGACAACTTTATCATTTGTGCTGATC

GAAGAATAGTTGATGATTGGGCCAATGATGGATGGGGCCTGAAGAAAGCTGCTGATGGGGCTGCTGAGCC

AGGCGTTGTGGGGCAGATGATCGAGGCAGCTGAAGAGCGCCCGTGGCTGTGGGTAGTCTATATTCTAACT

GTAGCCCTTCCTGTGTTCCTGGTTATCCTCTTCTGCTGTTCTGGAAAGAAACAGACCAGTGGTATGGAGT

ATAAGAAAACTGATGCACCTCAACCGGATGTGAAGGAAGAGGAAGAAGAGAAGGAAGAGGAAAAGGACAA

GGGAGATGAGGAGGAGGAAGGAGAAGAGAAACTTGAAGAGAAACAGAAAAGTGATGCTGAAGAAGATGGT

GGCACTGTCAGTCAAGAGGAGGAAGACAGAAAACCTAAAGCAGAGGAGGATGAAATTTTGAACAGATCAC

CAAGAAACAGAAAGCCACGAAGAGAGTGA

>lcl|NM_001198868.2_cds_NP_001185797.1_1 [gene=CAPN1] [db_xref=CCDS:CCDS44644.1] [protein=calpain-1 catalytic subunit] [protein_id=NP_001185797.1] [location=40..2184] [gbkey=CDS]

ATGTCGGAGGAGATCATCACGCCGGTGTACTGCACTGGGGTGTCAGCCCAAGTGCAGAAGCAGCGGGCCA

GGGAGCTGGGCCTGGGCCGCCATGAGAATGCCATCAAGTACCTGGGCCAGGATTATGAGCAGCTGCGGGT

GCGATGCCTGCAGAGTGGGACCCTCTTCCGTGATGAGGCCTTCCCCCCGGTACCCCAGAGCCTGGGTTAC

AAGGACCTGGGTCCCAATTCCTCCAAGACCTATGGCATCAAGTGGAAGCGTCCCACGGAACTGCTGTCAA

ACCCCCAGTTCATTGTGGATGGAGCTACCCGCACAGACATCTGCCAGGGAGCACTGGGGGACTGCTGGCT

CTTGGCGGCCATCGCCTCCCTCACTCTCAACGACACCCTCCTGCACCGAGTGGTTCCGCACGGCCAGAGC

TTCCAGAATGGCTATGCCGGCATCTTCCATTTCCAGCTGTGGCAATTTGGGGAGTGGGTGGACGTGGTCG

TGGATGACCTGCTGCCCATCAAGGACGGGAAGCTAGTGTTCGTGCACTCTGCCGAAGGCAACGAGTTCTG

GAGCGCCCTGCTTGAGAAGGCCTATGCCAAGGTAAATGGCAGCTACGAGGCCCTGTCAGGGGGCAGCACC

TCAGAGGGCTTTGAGGACTTCACAGGCGGGGTTACCGAGTGGTACGAGTTGCGCAAGGCTCCCAGTGACC

TCTACCAGATCATCCTCAAGGCGCTGGAGCGGGGCTCCCTGCTGGGCTGCTCCATAGACATCTCCAGCGT

TCTAGACATGGAGGCCATCACTTTCAAGAAGTTGGTGAAGGGCCATGCCTACTCTGTGACCGGGGCCAAG

CAGGTGAACTACCGAGGCCAGGTGGTGAGCCTGATCCGGATGCGGAACCCCTGGGGCGAGGTGGAGTGGA

CGGGAGCCTGGAGCGACAGCTCCTCAGAGTGGAACAACGTGGACCCATATGAACGGGACCAGCTCCGGGT

CAAGATGGAGGACGGGGAGTTCTGGATGTCATTCCGAGACTTCATGCGGGAGTTCACCCGCCTGGAGATC

TGCAACCTCACACCCGACGCCCTCAAGAGCCGGACCATCCGCAAATGGAACACCACACTCTACGAAGGCA

CCTGGCGGCGGGGGAGCACCGCGGGGGGCTGCCGAAACTACCCAGCCACCTTCTGGGTGAACCCTCAGTT

CAAGATCCGGCTGGATGAGACGGATGACCCGGACGACTACGGGGACCGCGAGTCAGGCTGCAGCTTCGTG

CTCGCCCTTATGCAGAAGCACCGTCGCCGCGAGCGCCGCTTCGGCCGCGACATGGAGACTATTGGCTTCG

CGGTCTACGAGGTCCCTCCGGAGCTGGTGGGCCAGCCGGCCGTACACTTGAAGCGTGACTTCTTCCTGGC

CAATGCGTCTCGGGCGCGCTCAGAGCAGTTCATCAACCTGCGAGAGGTCAGCACCCGCTTCCGCCTGCCA

CCCGGGGAGTATGTGGTGGTGCCCTCCACCTTCGAGCCCAACAAGGAGGGCGACTTCGTGCTGCGCTTCT

TCTCAGAGAAGAGTGCTGGGACTGTGGAGCTGGATGACCAGATCCAGGCCAATCTCCCCGATGAGCAAGT

GCTCTCAGAAGAGGAGATTGACGAGAACTTCAAGGCCCTCTTCAGGCAGCTGGCAGGGGAGGACATGGAG

ATCAGCGTGAAGGAGTTGCGGACAATCCTCAATAGGATCATCAGCAAACACAAAGACCTGCGGACCAAGG

GCTTCAGCCTAGAGTCGTGCCGCAGCATGGTGAACCTCATGGATCGTGATGGCAATGGGAAGCTGGGCCT

GGTGGAGTTCAACATCCTGTGGAACCGCATCCGGAATTACCTGTCCATCTTCCGGAAGTTTGACCTGGAC

AAGTCGGGCAGCATGAGTGCCTACGAGATGCGGATGGCCATTGAGTCGGCAGGCTTCAAGCTCAACAAGA

AGCTGTACGAGCTCATCATCACCCGCTACTCGGAGCCCGACCTGGCGGTCGACTTTGACAATTTCGTTTG

CTGCCTGGTGCGGCTAGAGACCATGTTCCGATTTTTCAAAACTCTGGACACAGATCTGGATGGAGTTGTG

ACCTTTGACTTGTTTAAGTGGTTGCAGCTGACCATGTTTGCATGA

>lcl|NM_023083.4_cds_NP_075571.2_1 [gene=CAPN10] [db_xref=CCDS:CCDS42838.1] [protein=calpain-10 isoform a] [protein_id=NP_075571.2] [location=179..2197] [gbkey=CDS]

ATGCGGGCGGGCCGGGGCGCGACGCCGGCGAGGGAGCTGTTCCGGGACGCCGCCTTCCCCGCCGCGGACT

CCTCGCTCTTCTGCGACTTGTCTACGCCGCTGGCCCAGTTCCGCGAGGACATCACGTGGAGGCGGCCCCA

GGAGATTTGTGCCACACCCCGGCTGTTTCCAGATGACCCACGGGAAGGGCAGGTGAAGCAGGGGCTGCTG

GGGGATTGCTGGTTCCTGTGTGCCTGCGCCGCGCTGCAGAAGAGCAGGCACCTCCTGGACCAGGTCATTC

CTCCGGGACAGCCGAGCTGGGCCGACCAGGAGTACCGGGGCTCCTTCACCTGTCGCATTTGGCAGTTTGG

ACGCTGGGTGGAGGTGACCACAGATGACCGCCTGCCGTGCCTTGCAGGGAGACTCTGTTTCTCCCGCTGC

CAGAGGGAGGATGTGTTCTGGCTCCCCTTACTGGAAAAGGTCTACGCCAAGGTCCATGGGTCCTACGAGC

ACCTGTGGGCCGGGCAGGTGGCGGATGCCCTGGTGGACCTGACCGGCGGCCTGGCAGAAAGATGGAACCT

GAAGGGCGTAGCAGGAAGCGGAGGCCAGCAGGACAGGCCAGGCCGCTGGGAGCACAGGACTTGTCGGCAG

CTGCTCCACCTGAAGGACCAGTGTCTGATCAGCTGCTGCGTGCTCAGCCCCAGAGCAGGTGCCCGGGAGC

TGGGGGAGTTCCATGCCTTCATTGTCTCGGACCTGCGGGAGCTCCAGGGTCAGGCGGGCCAGTGCATCCT

GCTGCTGCGGATCCAGAACCCCTGGGGCCGGCGGTGCTGGCAGGGGCTCTGGAGAGAGGGGGGTGAAGGG

TGGAGCCAGGTAGATGCAGCGGTAGCATCTGAGCTCCTGTCCCAGCTCCAGGAAGGGGAGTTCTGGGTGG

AGGAGGAGGAGTTCCTCAGGGAGTTTGACGAGCTCACCGTTGGCTACCCGGTCACGGAGGCCGGCCACCT

GCAGAGCCTCTACACAGAGAGGCTGCTCTGCCATACGCGGGCGCTGCCTGGGGCCTGGGTCAAGGGCCAG

TCAGCAGGAGGCTGCCGGAACAACAGCGGCTTTCCCAGCAACCCCAAATTCTGGCTGCGGGTCTCAGAAC

CGAGTGAGGTGTACATTGCCGTCCTGCAGAGATCCAGGCTGCACGCGGCGGACTGGGCAGGCCGGGCCCG

GGCACTGGTGGGTGACAGTCATACTTCGTGGAGCCCAGCGAGCATCCCGGGCAAGCACTACCAGGCTGTG

GGTCTGCACCTCTGGAAGGTAGAGAAGCGGCGGGTCAATCTGCCTAGGGTCCTGTCCATGCCCCCCGTGG

CTGGCACCGCGTGCCATGCATACGACCGGGAGGTCCACCTGCGTTGTGAGCTCTCACCGGGCTACTACCT

GGCTGTCCCCAGCACCTTCCTGAAGGACGCGCCAGGGGAGTTCCTGCTCCGAGTCTTCTCTACCGGGCGA

GTCTCCCTTAGCGCCATCAGGGCAGTGGCCAAGAACACCACCCCCGGGGCAGCCCTGCCTGCGGGGGAGT

GGGGGACCGTGCAGCTACGGGGTTCTTGGAGAGTCGGCCAGACGGCGGGGGGCAGCAGGAACTTTGCCTC

ATACCCCACCAACCCCTGCTTCCCCTTCTCGGTCCCCGAGGGCCCTGGCCCCCGCTGCGTCCGCATCACT

CTGCATCAGCACTGCCGGCCCAGTGACACCGAGTTCCACCCCATCGGCTTCCATATCTTCCAGGTCCCAG

AGGGTGGAAGGAGCCAGGACGCACCCCCACTGCTGCTGCAGGAGCCGCTGCTGAGCTGCGTGCCACATCG

CTACGCCCAGGAGGTGAGCCGGCTCTGCCTCCTGCCTGCGGGCACCTACAAGGTTGTGCCCTCCACCTAC

CTGCCGGACACAGAGGGGGCCTTCACAGTGACCATCGCAACCAGGATTGACAGGCCATCCATTCACAGCC

AGGAGATGCTGGGCCAGTTCCTCCAAGAGGTCTCCATCATGGCAGTGATGAAAACCTAA

>lcl|NM_001748.5_cds_NP_001739.3_1 [gene=CAPN2] [db_xref=CCDS:CCDS31035.1] [protein=calpain-2 catalytic subunit isoform 1] [protein_id=NP_001739.3] [location=103..2205] [gbkey=CDS]

ATGGCGGGCATCGCGGCCAAGCTGGCGAAGGACCGGGAGGCGGCCGAGGGGCTGGGCTCCCACGACAGGG

CCATCAAGTACCTCAACCAGGACTACGAGGCGCTGCGGAACGAGTGCCTGGAGGCCGGGACGCTCTTCCA

GGACCCGTCCTTCCCGGCCATCCCCTCGGCCCTGGGCTTCAAGGAGTTGGGGCCCTACTCCAGCAAAACC

CGGGGCATCGAGTGGAAGCGCCCCACGGAGATCTGCGCTGACCCCCAGTTTATCATTGGAGGAGCCACCC

GCACAGACATCTGCCAAGGAGCCCTGGGTGACTGCTGGCTGCTGGCAGCCATTGCCTCCCTCACCTTGAA

TGAAGAAATCCTGGCTCGAGTCGTCCCCCTAAACCAGAGCTTCCAGGAAAACTATGCAGGGATCTTTCAC

TTCCAGTTCTGGCAATACGGCGAGTGGGTGGAGGTGGTGGTGGATGACAGGCTGCCCACCAAGGACGGGG

AGCTGCTCTTTGTGCATTCAGCCGAAGGGAGCGAGTTCTGGAGCGCCCTGCTGGAGAAGGCATACGCCAA

GATCAACGGATGCTATGAAGCGCTATCAGGGGGTGCCACCACTGAGGGCTTCGAAGACTTCACCGGAGGC

ATTGCTGAGTGGTATGAGTTGAAGAAGCCCCCTCCCAACCTGTTCAAGATCATCCAGAAAGCTCTGCAAA

AAGGCTCTCTCCTTGGCTGCTCCATCGACATCACCAGCGCCGCGGACTCGGAGGCCATCACGTTTCAGAA

GCTGGTGAAGGGGCACGCGTACTCGGTCACCGGAGCCGAGGAGGTTGAAAGTAACGGAAGCCTACAGAAA

CTGATCCGCATCCGAAATCCCTGGGGAGAAGTGGAGTGGACAGGGCGGTGGAATGACAACTGCCCAAGCT

GGAACACTATAGACCCAGAGGAGAGGGAAAGGCTGACCAGACGGCATGAAGATGGAGAATTCTGGATGTC

TTTCAGTGACTTCCTGAGGCACTATTCCCGCCTGGAGATCTGTAACCTGACCCCAGACACTCTCACCAGC

GATACCTACAAGAAGTGGAAACTCACCAAAATGGATGGGAACTGGAGGCGGGGCTCCACCGCGGGAGGTT

GCAGGAACTACCCGAACACATTCTGGATGAACCCTCAGTACCTGATCAAGCTGGAGGAGGAGGATGAGGA

CGAGGAGGATGGGGAGAGCGGCTGCACCTTCCTGGTGGGGCTCATTCAGAAGCACCGACGGCGGCAGAGG

AAGATGGGCGAGGACATGCACACCATCGGCTTTGGCATCTATGAGGTTCCAGAGGAGTTAAGTGGGCAGA

CCAACATCCACCTCAGCAAAAACTTCTTCCTGACGAATCGCGCCAGGGAGCGCTCAGACACCTTCATCAA

CCTCCGGGAGGTGCTCAACCGCTTCAAGCTGCCGCCAGGAGAGTACATTCTCGTGCCTTCCACCTTCGAA

CCCAACAAGGATGGGGATTTCTGCATCCGGGTCTTTTCTGAAAAGAAAGCTGACTACCAAGCTGTCGATG

ATGAAATCGAGGCCAATCTTGAAGAGTTCGACATCAGCGAGGATGACATTGATGATGGATTCAGGAGACT

GTTTGCCCAGTTGGCAGGAGAGGATGCGGAGATCTCTGCCTTTGAGCTGCAGACCATCCTGAGAAGGGTT

CTAGCAAAGCGCCAAGATATCAAGTCAGATGGCTTCAGCATCGAGACATGCAAAATTATGGTTGACATGC

TAGATTCGGACGGGAGTGGCAAGCTGGGGCTGAAGGAGTTCTACATTCTCTGGACGAAGATTCAAAAATA

CCAAAAAAATTTACCGAGAAATCGACGTTGACAGGTCTGGTACCATGAATTCCTATGAAATGCGGAAGGCA

TTAGAAGAAGCAGGTTTCAAGATGCCCTGTCAACTCCACCAAGTCATCGTTGCTCGGTTTGCAGATGACC

AGCTCATCATCGATTTTGATAATTTTGTTCGGTGTTTGGTTCGGCTGGAAACGCTATTCAAGATATTTAA

GCAGCTGGATCCCGAGAATACTGGAACAATAGAGCTCGACCTTATCTCTTGGCTCTGTTTCTCAGTACTT

TGA

>lcl|NM_001003962.3_cds_NP_001003962.1_1 [gene=CAPNS1] [db_xref=CCDS:CCDS12489.1] [protein=calpain small subunit 1 isoform 1] [protein_id=NP_001003962.1] [location=105..911] [gbkey=CDS]

ATGTTCCTGGTTAACTCGTTCTTGAAGGGCGGCGGCGGCGGCGGGGGAGGCGGGGGCCTGGGTGGGG

GCCTGGGAAATGTGCTTGGAGGCCTGATCAGCGGGGCCGGGGGCGGCGGCGGCGGCGGCGGCGGCGG

CGGTGGTGGAGGCGGCGGTGGCGGTGGAACGGCCATGCGCATCCTAGGCGGAGTCATCAGCGCCATCAGC

GAGGCGGCTGCGCAGTACAACCCGGAGCCCCCGCCCCCACGCACACATTACTCCAACATTGAGGCCAACG

AGAGTGAGGAGGTCCGGCAGTTCCGGAGACTCTTTGCCCAGCTGGCTGGAGATGACATGGAGGTCAGCGC

CACAGAACTCATGAACATTCTCAATAAGGTTGTGACACGACACCCTGATCTGAAGACTGATGGTTTTGGC

ATTGACACATGTCGCAGCATGGTGGCCGTGATGGATAGCGACACCACAGGCAAGCTGGGCTTTGAGGAAT

TCAAGTACTTGTGGAACAACATCAAAAGGTGGCAGGCCATATACAAACAGTTCGACACTGACCGATCAGG

GACCATTTGCAGTAGTGAACTCCCAGGTGCCTTTGAGGCAGCAGGGTTCCACCTGAATGAGCATCTCTAT

AACATGATCATCCGACGCTACTCAGATGAAAGTGGGAACATGGATTTTGACAACTTCATCAGCTGCTTGG

TCAGGCTGGACGCCATGTTCCGTGCCTTCAAATCTCTTGACAAAGATGGCACTGGACAAATCCAGGTGAA

CATCCAGGAGTGGCTGCAGCTGACTATGTATTCCTGA

>lcl|NM_033292.4_cds_NP_150634.1_1 [gene=CASP1] [db_xref=CCDS:CCDS8330.1] [protein=caspase-1 isoform alpha precursor] [protein_id=NP_150634.1] [location=32..1246] [gbkey=CDS]

ATGGCCGACAAGGTCCTGAAGGAGAAGAGAAAGCTGTTTATCCGTTCCATGGGTGAAGGTACAATAAATG

GCTTACTGGATGAATTATTACAGACAAGGGTGCTGAACAAGGAAGAGATGGAGAAAGTAAAACGTGAAAA

TGCTACAGTTATGGATAAGACCCGAGCTTTGATTGACTCCGTTATTCCGAAAGGGGCACAGGCATGCCAA

ATTTGCATCACATACATTTGTGAAGAAGACAGTTACCTGGCAGGGACGCTGGGACTCTCAGCAGATCAAA

CATCTGGAAATTACCTTAATATGCAAGACTCTCAAGGAGTACTTTCTTCCTTTCCAGCTCCTCAGGCAGT

GCAGGACAACCCAGCTATGCCCACATCCTCAGGCTCAGAAGGGAATGTCAAGCTTTGCTCCCTAGAAGAA

GCTCAAAGGATATGGAAACAAAAGTCGGCAGAGATTTATCCAATAATGGACAAGTCAAGCCGCACACGTC

TTGCTCTCATTATCTGCAATGAAGAATTTGACAGTATTCCTAGAAGAACTGGAGCTGAGGTTGACATCAC

AGGCATGACAATGCTGCTACAAAATCTGGGGTACAGCGTAGATGTGAAAAAAAATCTCACTGCTTCGGAC

ATGACTACAGAGCTGGAGGCATTTGCACACCGCCCAGAGCACAAGACCTCTGACAGCACGTTCCTGGTGT

TCATGTCTCATGGTATTCGGGAAGGCATTTGTGGGAAGAAACACTCTGAGCAAGTCCCAGATATACTACA

ACTCAATGCAATCTTTAACATGTTGAATACCAAGAACTGCCCAAGTTTGAAGGACAAACCGAAGGTGATC

ATCATCCAGGCCTGCCGTGGTGACAGCCCTGGTGTGGTGTGGTTTAAAGATTCAGTAGGAGTTTCTGGAA

ACCTATCTTTACCAACTACAGAAGAGTTTGAGGATGATGCTATTAAGAAAGCCCACATAGAGAAGGATTT

TATCGCTTTCTGCTCTTCCACACCAGATAATGTTTCTTGGAGACATCCCACAATGGGCTCTGTTTTTATT

GGAAGACTCATTGAACATATGCAAGAATATGCCTGTTCCTGTGATGTGGAGGAAATTTTCCGCAAGGTTC

GATTTTCATTTGAGCAGCCAGATGGTAGAGCGCAGATGCCCACCACTGAAAGAGTGACTTTGACAAGATG

TTTCTACCTCTTCCCAGGACATTAA

>lcl|NM_004346.4_cds_NP_004337.2_1 [gene=CASP3] [db_xref=CCDS:CCDS3836.1] [protein=caspase-3 isoform a preproprotein] [protein_id=NP_004337.2] [location=236..1069] [gbkey=CDS]

ATGGAGAACACTGAAAACTCAGTGGATTCAAAATCCATTAAAAATTTGGAACCAAAGATCATACATGGAA

GCGAATCAATGGACTCTGGAATATCCCTGGACAACAGTTATAAAATGGATTATCCTGAGATGGGTTTATG

TATAATAATTAATAATAAGAATTTTCATAAAAGCACTGGAATGACATCTCGGTCTGGTACAGATGTCGAT

GCAGCAAACCTCAGGGAAACATTCAGAAACTTGAAATATGAAGTCAGGAATAAAAATGATCTTACACGTG

AAGAAATTGTGGAATTGATGCGTGATGTTTCTAAAGAAGATCACAGCAAAAGGAGCAGTTTTGTTTGTGT

GCTTCTGAGCCATGGTGAAGAAGGAATAATTTTTGGAACAAATGGACCTGTTGACCTGAAAAAAATAACA

AACTTTTTCAGAGGGGATCGTTGTAGAAGTCTAACTGGAAAACCCAAACTTTTCATTATTCAGGCCTGCC

GTGGTACAGAACTGGACTGTGGCATTGAGACAGACAGTGGTGTTGATGATGACATGGCGTGTCATAAAAT

ACCAGTGGAGGCCGACTTCTTGTATGCATACTCCACAGCACCTGGTTATTATTCTTGGCGAAATTCAAAG

GATGGCTCCTGGTTCATCCAGTCGCTTTGTGCCATGCTGAAACAGTATGCCGACAAGCTTGAATTTATGC

ACATTCTTACCCGGGTTAACCGAAAGGTGGCAACAGAATTTGAGTCCTTTTCCTTTGACGCTACTTTTCA

TGCAAAGAAACAGATTCCATGTATTGTTTCCATGCTCACAAAAGAACTCTATTTTTATCACTAA

>lcl|NM_001225.4_cds_NP_001216.1_1 [gene=CASP4] [db_xref=CCDS:CCDS8327.1] [protein=caspase-4 isoform alpha precursor] [protein_id=NP_001216.1] [location=50..1183] [gbkey=CDS]

ATGGCAGAAGGCAACCACAGAAAAAAGCCACTTAAGGTGTTGGAATCCCTGGGCAAAGATTTCCTCACTG

GTGTTTTGGATAACTTGGTGGAACAAAATGTACTGAACTGGAAGGAAGAGGAAAAAAAGAAATATTACGA

TGCTAAAACTGAAGACAAAGTTCGGGTCATGGCAGACTCTATGCAAGAGAAGCAACGTATGGCAGGACAA

ATGCTTCTTCAAACCTTTTTTAACATAGACCAAATATCCCCCAATAAAAAAGCTCATCCGAATATGGAGG

CTGGACCACCTGAGTCAGGAGAATCTACAGATGCCCTCAAGCTTTGTCCTCATGAAGAATTCCTGAGACT

ATGTAAAGAAAGAGCTGAAGAGATCTATCCAATAAAGGAGAGAAACAACCGCACACGCCTGGCTCTCATC

ATATGCAATACAGAGTTTGACCATCTGCCTCCGAGGAATGGAGCTGACTTTGACATCACAGGGATGAAGG

AGCTACTTGAGGGTCTGGACTATAGTGTAGATGTAGAAGAGAATCTGACAGCCAGGGATATGGAGTCAGC

GCTGAGGGCATTTGCTACCAGACCAGAGCACAAGTCCTCTGACAGCACATTCTTGGTACTCATGTCTCAT

GGCATCCTGGAGGGAATCTGCGGAACTGTGCATGATGAGAAAAAACCAGATGTGCTGCTTTATGACACCA

TCTTCCAGATATTCAACAACCGCAACTGCCTCAGTCTGAAGGACAAACCCAAGGTCATCATTGTCCAGGC

CTGCAGAGGTGCAAACCGTGGGGAACTGTGGGTCAGAGACTCTCCAGCATCCTTGGAAGTGGCCTCTTCA

CAGTCATCTGAGAACCTAGAGGAAGATGCTGTTTACAAGACCCACGTGGAGAAGGACTTCATTGCTTTCT

GCTCTTCAACGCCACACAACGTGTCCTGGAGAGACAGCACAATGGGCTCTATCTTCATCACACAACTCAT

CACATGCTTCCAGAAATATTCTTGGTGCTGCCACCTAGAGGAAGTATTTCGGAAGGTACAGCAATCATTT

GAAACTCCAAGGGCCAAAGCTCAAATGCCCACCATAGAACGACTGTCCATGACAAGATATTTCTACCTCT

TTCCTGGCAATTGA

>lcl|NM_001228.4_cds_NP_001219.2_1 [gene=CASP8] [db_xref=CCDS:CCDS42799.1] [protein=caspase-8 isoform 1 precursor] [protein_id=NP_001219.2] [location=304..1794] [gbkey=CDS]

ATGGACTTCAGCAGAAATCTTTATGATATTGGGGAACAACTGGACAGTGAAGATCTGGCCTCCCTCAAGT

TCCTGAGCCTGGACTACATTCCGCAAAGGAAGCAAGAACCCATCAAGGATGCCTTGATGTTATTCCAGAG

ACTCCAGGAAAAGAGAATGTTGGAGGAAAGCAATCTGTCCTTCCTGAAGGAGCTGCTCTTCCGAATTAAT

AGACTGGATTTGCTGATTACCTACCTAAACACTAGAAAGGAGGAGATGGAAAGGGAACTTCAGACACCAG

GCAGGGCTCAAATTTCTGCCTACAGGTTCCACTTCTGCCGCATGAGCTGGGCTGAAGCAAACAGCCAGTG

CCAGACACAGTCTGTACCTTTCTGGCGGAGGGTCGATCATCTATTAATAAGGGTCATGCTCTATCAGATT

TCAGAAGAAGTGAGCAGATCAGAATTGAGGTCTTTTAAGTTTCTTTTGCAAGAGGAAATCTCCAAATGCA

AACTGGATGATGACATGAACCTGCTGGATATTTTCATAGAGATGGAGAAGAGGGTCATCCTGGGAGAAGG

AAAGTTGGACATCCTGAAAAGAGTCTGTGCCCAAATCAACAAGAGCCTGCTGAAGATAATCAACGACTAT

GAAGAATTCAGCAAAGGGGAGGAGTTGTGTGGGGTAATGACAATCTCGGACTCTCCAAGAGAACAGGATA

GTGAATCACAGACTTTGGACAAAGTTTACCAAATGAAAAGCAAACCTCGGGGATACTGTCTGATCATCAA

CAATCACAATTTTGCAAAAGCACGGGAGAAAGTGCCCAAACTTCACAGCATTAGGGACAGGAATGGAACA

CACTTGGATGCAGGGGCTTTGACCACGACCTTTGAAGAGCTTCATTTTGAGATCAAGCCCCACGATGACT

GCACAGTAGAGCAAATCTATGAGATTTTGAAAATCTACCAACTCATGGACCACAGTAACATGGACTGCTT

CATCTGCTGTATCCTCTCCCATGGAGACAAGGGCATCATCTATGGCACTGATGGACAGGAGGCCCCCATC

TATGAGCTGACATCTCAGTTCACTGGTTTGAAGTGCCCTTCCCTTGCTGGAAAACCCAAAGTGTTTTTTA

TTCAGGCTTGTCAGGGGGATAACTACCAGAAAGGTATACCTGTTGAGACTGATTCAGAGGAGCAACCCTA

TTTAGAAATGGATTTATCATCACCTCAAACGAGATATATCCCGGATGAGGCTGACTTTCTGCTGGGGATG

GCCACTGTGAATAACTGTGTTTCCTACCGAAACCCTGCAGAGGGAACCTGGTACATCCAGTCACTTTGCC

AGAGCCTGAGAGAGCGATGTCCTCGAGGCGATGATATTCTCACCATCCTGACTGAAGTGAACTATGAAGT

AAGCAACAAGGATGACAAGAAAAACATGGGGAAACAGATGCCTCAGCCTACTTTCACACTAAGAAAAAAA

CTTGTCTTCCCTTCTGATTGA

>lcl|NM_002982.4_cds_NP_002973.1_1 [gene=CCL2] [db_xref=CCDS:CCDS11277.1] [protein=C-C motif chemokine 2 precursor] [protein_id=NP_002973.1] [location=66..365] [gbkey=CDS]

ATGAAAGTCTCTGCCGCCCTTCTGTGCCTGCTGCTCATAGCAGCCACCTTCATTCCCCAAGGGCTCGCTC

AGCCAGATGCAATCAATGCCCCAGTCACCTGCTGTTATAACTTCACCAATAGGAAGATCTCAGTGCAGAG

GCTCGCGAGCTATAGAAGAATCACCAGCAGCAAGTGTCCCAAAGAAGCTGTGATCTTCAAGACCATTGTG

GCCAAGGAGATCTGTGCTGACCCCAAGCAGAAGTGGGTTCAGGATTCCATGGACCACCTGGACAAGCAAA

CCCAAACTCCGAAGACTTGA

>lcl|NM_001123041.3_cds_NP_001116513.2_1 [gene=CCR2] [db_xref=CCDS:CCDS43078.1] [protein=C-C chemokine receptor type 2 isoform A] [protein_id=NP_001116513.2] [location=119..1243] [gbkey=CDS]

ATGCTGTCCACATCTCGTTCTCGGTTTATCAGAAATACCAACGAGAGCGGTGAAGAAGTCACCACCTTTT

TTGATTATGATTACGGTGCTCCCTGTCATAAATTTGACGTGAAGCAAATTGGGGCCCAACTCCTGCCTCC

GCTCTACTCGCTGGTGTTCATCTTTGGTTTTGTGGGCAACATGCTGGTCGTCCTCATCTTAATAAACTGC

AAAAAGCTGAAGTGCTTGACTGACATTTACCTGCTCAACCTGGCCATCTCTGATCTGCTTTTTCTTATTA

CTCTCCCATTGTGGGCTCACTCTGCTGCAAATGAGTGGGTCTTTGGGAATGCAATGTGCAAATTATTCAC

AGGGCTGTATCACATCGGTTATTTTGGCGGAATCTTCTTCATCATCCTCCTGACAATCGATAGATACCTG

GCTATTGTCCATGCTGTGTTTGCTTTAAAAGCCAGGACGGTCACCTTTGGGGTGGTGACAAGTGTGATCA

CCTGGTTGGTGGCTGTGTTTGCTTCTGTCCCAGGAATCATCTTTACTAAATGCCAGAAAGAAGATTCTGT

TTATGTCTGTGGCCCTTATTTTCCACGAGGATGGAATAATTTCCACACAATAATGAGGAACATTTTGGGG

CTGGTCCTGCCGCTGCTCATCATGGTCATCTGCTACTCGGGAATCCTGAAAACCCTGCTTCGGTGTCGAA

ACGAGAAGAAGAGGCATAGGGCAGTGAGAGTCATCTTCACCATCATGATTGTTTACTTTCTCTTCTGGAC

TCCCTATAATATTGTCATTCTCCTGAACACCTTCCAGGAATTCTTCGGCCTGAGTAACTGTGAAAGCACC

AGTCAACTGGACCAAGCCACGCAGGTGACAGAGACTCTTGGGATGACTCACTGCTGCATCAATCCCATCA

TCTATGCCTTCGTTGGGGAGAAGTTCAGAAGCCTTTTTCACATAGCTCTTGGCTGTAGGATTGCCCCACT

CCAAAAACCAGTGTGTGGAGGTCCAGGAGTGAGACCAGGAAAGAATGTGAAAGTGACTACACAAGGACTC

CTCGATGGTCGTGGAAAAGGAAAGTCAATTGGCAGAGCCCCTGAAGCCAGTCTTCAGGACAAAGAAGGAG

CCTAG

>lcl|NM_172361.3_cds_NP_758871.1_1 [gene=CD46] [db_xref=CCDS:CCDS1484.1] [protein=membrane cofactor protein isoform 12 precursor] [protein_id=NP_758871.1] [location=160..1227] [gbkey=CDS]

ATGGAGCCTCCCGGCCGCCGCGAGTGTCCCTTTCCTTCCTGGCGCTTTCCTGGGTTGCTTCTGGCGGCCA

TGGTGTTGCTGCTGTACTCCTTCTCCGATGCCTGTGAGGAGCCACCAACATTTGAAGCTATGGAGCTCAT

TGGTAAACCAAAACCCTACTATGAGATTGGTGAACGAGTAGATTATAAGTGTAAAAAAGGATACTTCTAT

ATACCTCCTCTTGCCACCCATACTATTTGTGATCGGAATCATACATGGCTACCTGTCTCAGATGACGCCT

GTTATAGAGAAACATGTCCATATATACGGGATCCTTTAAATGGCCAAGCAGTCCCTGCAAATGGGACTTA

CGAGTTTGGTTATCAGATGCACTTTATTTGTAATGAGGGTTATTACTTAATTGGTGAAGAAATTCTATAT

TGTGAACTTAAAGGATCAGTAGCAATTTGGAGCGGTAAGCCCCCAATATGTGAAAAGGTTTTGTGTACAC

CACCTCCAAAAATAAAAAATGGAAAACACACCTTTAGTGAAGTAGAAGTATTTGAGTATCTTGATGCAGT

AACTTATAGTTGTGATCCTGCACCTGGACCAGATCCATTTTCACTTATTGGAGAGAGCACGATTTATTGT

GGTGACAATTCAGTGTGGAGTCGTGCTGCTCCAGAGTGTAAAGTGGTCAAATGTCGATTTCCAGTAGTCG

AAAATGGAAAACAGATATCAGGATTTGGAAAAAAATTTTACTACAAAGCAACAGTTATGTTTGAATGCGA

TAAGGGTTTTTACCTCGATGGCAGCGACACAATTGTCTGTGACAGTAACAGTACTTGGGATCCCCCAGTT

CCAAAGTGTCTTAAAGGATATCCTAAACCTGAGGAAGGAATACTTGACAGTTTGGATGTTTGGGTCATTG

CTGTGATTGTTATTGCCATAGTTGTTGGAGTTGCAGTAATTTGTGTTGTCCCGTACAGATATCTTCAAAG

GAGGAAGAAGAAAGGGAAAGCAGATGGTGGAGCTGAATATGCCACTTACCAGACTAAATCAACCACTCCA

GCAGAGCAGAGAGGCTGA

>lcl|NM_078467.3_cds_NP_510867.1_1 [gene=CDKN1A] [db_xref=CCDS:CCDS4824.1] [protein=cyclin-dependent kinase inhibitor 1 isoform 1] [protein_id=NP_510867.1] [location=100..594] [gbkey=CDS]

ATGTCAGAACCGGCTGGGGATGTCCGTCAGAACCCATGCGGCAGCAAGGCCTGCCGCCGCCTCTTCGGCC

CAGTGGACAGCGAGCAGCTGAGCCGCGACTGTGATGCGCTAATGGCGGGCTGCATCCAGGAGGCCCGTGA

GCGATGGAACTTCGACTTTGTCACCGAGACACCACTGGAGGGTGACTTCGCCTGGGAGCGTGTGCGGGGC

CTTGGCCTGCCCAAGCTCTACCTTCCCACGGGGCCCCGGCGAGGCCGGGATGAGTTGGGAGGAGGCAGGC

GGCCTGGCACCTCACCTGCTCTGCTGCAGGGGACAGCAGAGGAAGACCATGTGGACCTGTCACTGTCTTG

TACCCTTGTGCCTCGCTCAGGGGAGCAGGCTGAAGGGTCCCCAGGTGGACCTGGAGACTCTCAGGGTCGA

AAACGGCGGCAGACCAGCATGACAGATTTCTACCACTCCAAACGCCGGCTGATCTTCTCCAAGAGGAAGC

CCTAA

>lcl|NM_004064.5_cds_NP_004055.1_1 [gene=CDKN1B] [db_xref=CCDS:CCDS8653.1] [protein=cyclin-dependent kinase inhibitor 1B] [protein_id=NP_004055.1] [location=473..1069] [gbkey=CDS]

ATGTCAAACGTGCGAGTGTCTAACGGGAGCCCTAGCCTGGAGCGGATGGACGCCAGGCAGGCGGAGCACC

CCAAGCCCTCGGCCTGCAGGAACCTCTTCGGCCCGGTGGACCACGAAGAGTTAACCCGGGACTTGGAGAA

GCACTGCAGAGACATGGAAGAGGCGAGCCAGCGCAAGTGGAATTTCGATTTTCAGAATCACAAACCCCTA

GAGGGCAAGTACGAGTGGCAAGAGGTGGAGAAGGGCAGCTTGCCCGAGTTCTACTACAGACCCCCGCGGC

CCCCCAAAGGTGCCTGCAAGGTGCCGGCGCAGGAGAGCCAGGATGTCAGCGGGAGCCGCCCGGCGGCGCC

TTTAATTGGGGCTCCGGCTAACTCTGAGGACACGCATTTGGTGGACCCAAAGACTGATCCGTCGGACAGC

CAGACGGGGTTAGCGGAGCAATGCGCAGGAATAAGGAAGCGACCTGCAACCGACGATTCTTCTACTCAAA

ACAAAAGAGCCAACAGAACAGAAGAAAATGTTTCAGACGGTTCCCCAAATGCCGGTTCTGTGGAGCAGAC

GCCCAAGAAGCCTGGCCTCAGAAGACGTCAAACGTAA

>lcl|NM_000077.5_cds_NP_000068.1_1 [gene=CDKN2A] [db_xref=CCDS:CCDS6510.1] [protein=cyclin-dependent kinase inhibitor 2A isoform p16INK4a] [protein_id=NP_000068.1] [location=31..501] [gbkey=CDS]

ATGGAGCCGGCGGCGGGGAGCAGCATGGAGCCTTCGGCTGACTGGCTGGCCACGGCCGCGGCCCGGGGTC

GGGTAGAGGAGGTGCGGGCGCTGCTGGAGGCGGGGGCGCTGCCCAACGCACCGAATAGTTACGGTCGGAG

GCCGATCCAGGTCATGATGATGGGCAGCGCCCGAGTGGCGGAGCTGCTGCTGCTCCACGGCGCGGAGCCC

AACTGCGCCGACCCCGCCACTCTCACCCGACCCGTGCACGACGCTGCCCGGGAGGGCTTCCTGGACACGC

TGGTGGTGCTGCACCGGGCCGGGGCGCGGCTGGACGTGCGCGATGCCTGGGGCCGTCTGCCCGTGGACCT

GGCTGAGGAGCTGGGCCATCGCGATGTCGCACGGTACCTGCGCGCGGCTGCGGGGGGCACCAGAGGCAGT

AACCATGCCCGCATAGATGCCGCGGAAGGTCCCTCAGACATCCCCGATTGA

>lcl|NM_003879.7_cds_NP_003870.4_1 [gene=CFLAR] [db_xref=CCDS:CCDS2337.1] [protein=CASP8 and FADD-like apoptosis regulator isoform 1] [protein_id=NP_003870.4] [location=456..1898] [gbkey=CDS]

ATGTCTGCTGAAGTCATCCATCAGGTTGAAGAAGCACTTGATACAGATGAGAAGGAGATGCTGCTCTTTT

TGTGCCGGGATGTTGCTATAGATGTGGTTCCACCTAATGTCAGGGACCTTCTGGATATTTTACGGGAAAG

AGGTAAGCTGTCTGTCGGGGACTTGGCTGAACTGCTCTACAGAGTGAGGCGATTTGACCTGCTCAAACGT

ATCTTGAAGATGGACAGAAAAGCTGTGGAGACCCACCTGCTCAGGAACCCTCACCTTGTTTCGGACTATA

GAGTGCTGATGGCAGAGATTGGTGAGGATTTGGATAAATCTGATGTGTCCTCATTAATTTTCCTCATGAA

GGATTACATGGGCCGAGGCAAGATAAGCAAGGAGAAGAGTTTCTTGGACCTTGTGGTTGAGTTGGAGAAA

CTAAATCTGGTTGCCCCAGATCAACTGGATTTATTAGAAAAATGCCTAAAGAACATCCACAGAATAGACC

TGAAGACAAAAATCCAGAAGTACAAGCAGTCTGTTCAAGGAGCAGGGACAAGTTACAGGAATGTTCTCCA

AGCAGCAATCCAAAAGAGTCTCAAGGATCCTTCAAATAACTTCAGGCTCCATAATGGGAGAAGTAAAGAA

CAAAGACTTAAGGAACAGCTTGGCGCTCAACAAGAACCAGTGAAGAAATCCATTCAGGAATCAGAAGCTT

TTTTGCCTCAGAGCATACCTGAAGAGAGATACAAGATGAAGAGCAAGCCCCTAGGAATCTGCCTGATAAT

CGATTGCATTGGCAATGAGACAGAGCTTCTTCGAGACACCTTCACTTCCCTGGGCTATGAAGTCCAGAAA

TTCTTGCATCTCAGTATGCATGGTATATCCCAGATTCTTGGCCAATTTGCCTGTATGCCCGAGCACCGAG

ACTACGACAGCTTTGTGTGTGTCCTGGTGAGCCGAGGAGGCTCCCAGAGTGTGTATGGTGTGGATCAGAC

TCACTCAGGGCTCCCCCTGCATCACATCAGGAGGATGTTCATGGGAGATTCATGCCCTTATCTAGCAGGG

AAGCCAAAGATGTTTTTTATTCAGAACTATGTGGTGTCAGAGGGCCAGCTGGAGGACAGCAGCCTCTTGG

AGGTGGATGGGCCAGCGATGAAGAATGTGGAATTCAAGGCTCAGAAGCGAGGGCTGTGCACAGTTCACCG

AGAAGCTGACTTCTTCTGGAGCCTGTGTACTGCGGACATGTCCCTGCTGGAGCAGTCTCACAGCTCACCA

TCCCTGTACCTGCAGTGCCTCTCCCAGAAACTGAGACAAGAAAGAAAACGCCCACTCCTGGATCTTCACA

TTGAACTCAATGGCTACATGTATGATTGGAACAGCAGAGTTTCTGCCAAGGAGAAATATTATGTCTGGCT

GCAGCACACTCTGAGAAAGAAACTTATCCTCTCCTACACATAA

>lcl|NM_014043.4_cds_NP_054762.2_1 [gene=CHMP2B] [db_xref=CCDS:CCDS2918.1] [protein=charged multivesicular body protein 2b isoform 1] [protein_id=NP_054762.2] [location=215..856] [gbkey=CDS]

ATGGCGTCCCTCTTCAAGAAGAAAACCGTGGATGATGTAATAAAGGAACAGAATCGAGAGTTACGAGGTA

CACAGAGGGCTATAATCAGAGATCGAGCAGCTTTAGAGAAACAAGAAAAACAGCTGGAATTAGAAATTAA

GAAAATGGCCAAGATTGGTAATAAGGAAGCTTGCAAAGTTTTAGCCAAACAACTTGTGCATCTACGGAAA

CAGAAGACGAGAACTTTTGCTGTAAGTTCAAAAGTTACTTCTATGTCTACACAAACAAAAGTGATGAATT

CCCAAATGAAGATGGCTGGAGCAATGTCTACTACAGCAAAAACAATGCAGGCAGTTAACAAGAAGATGGA

TCCACAAAAGACATTACAAACAATGCAGAATTTCCAGAAGGAAAACATGAAAATGGAAATGACTGAAGAA

ATGATCAATGATACACTTGATGACATCTTTGACGGTTCTGATGACGAAGAAGAAAGCCAGGATATTGTGA

ATCAAGTTCTTGATGAAATTGGAATTGAAATTTCTGGAAAGATGGCCAAAGCTCCATCAGCTGCTCGAAG

CTTACCATCTGCCTCTACTTCAAAGGCTACAATCTCAGATGAAGAGATTGAACGGCAACTCAAGGCTTTA

GGAGTAGATTAG

>lcl|NM_176812.5_cds_NP_789782.1_1 [gene=CHMP4B] [db_xref=CCDS:CCDS13228.1] [protein=charged multivesicular body protein 4b] [protein_id=NP_789782.1] [location=122..796] [gbkey=CDS]

ATGTCGGTGTTCGGGAAGCTGTTCGGGGCTGGAGGGGGTAAGGCCGGCAAGGGCGGCCCGACCCCCCAGG

AGGCCATCCAGCGGCTGCGGGACACGGAAGAGATGTTAAGCAAGAAACAGGAGTTCCTGGAGAAGAAAAT

CGAGCAGGAGCTGACGGCCGCCAAGAAGCACGGCACCAAAAACAAGCGCGGCCCTCCAGGCACTGAAG

CGTAAGAAGAGGTATGAGAAGCAGCTGGCGCAGATCGACGGCACATTATCAACCATCGAGTTCCAGCGGG

AGGCCCTGGAGAATGCCAACACCAACACCGAGGTGCTCAAGAACATGGGCTATGCCGCCAAGGCCATGAA

GGCGGCCCATGACAACATGGACATCGATAAAGTTGATGAGTTAATGCAGGACATTGCTGACCAGCAAGAA

CTTGCAGAGGAGATTTCAACAGCAATTTCGAAACCTGTAGGGTTTGGAGAAGAGTTTGACGAGGATGAGC

TCATGGCGGAATTAGAAGAACTAGAACAGGAGGAACTAGACAAGAATTTGCTGGAAATCAGTGGACCCGA

AACAGTCCCTCTACCAAATGTTCCCTCTATAGCCCTACCATCAAAACCCGCCAAGAAGAAAGAAGAGGAG

GACGACGACATGAAGGAATTGGAGAACTGGGCTGGATCCATGTAA

>lcl|NM_000086.2_cds_NP_000077.1_1 [gene=CLN3] [db_xref=CCDS:CCDS10632.1] [protein=battenin isoform a] [protein_id=NP_000077.1] [location=324..1640] [gbkey=CDS]

ATGGGAGGCTGTGCAGGCTCGCGGCGGCGCTTTTCGGATTCCGAGGGGGAGGAGACCGTCCCGGAGCCCC

GGCTCCCTCTGTTGGACCATCAGGGCGCGCATTGGAAGAACGCGGTGGGCTTCTGGCTGCTGGGCCTTTG

CAACAACTTCTCTTATGTGGTGATGCTGAGTGCCGCCCACGACATCCTTAGCCACAAGAGGACATCGGGA

AACCAGAGCCATGTGGACCCAGGCCCAACGCCGATCCCCCACAACAGCTCATCACGATTTGACTGCAACT

CTGTCTCTACGGCTGCTGTGCTCCTGGCGGACATCCTCCCCACACTCGTCATCAAATTGTTGGCTCCTCT

TGGCCTTCACCTGCTGCCCTACAGCCCCCGGGTTCTCGTCAGTGGGATTTGTGCTGCTGGAAGCTTCGTC

CTGGTTGCCTTTTCTCATTCTGTGGGGACCAGCCTGTGTGGTGTGGTCTTCGCTAGCATCTCATCAGGCC

TTGGGGAGGTCACCTTCCTCTCCCTCACTGCCTTCTACCCCAGGGCCGTGATCTCCTGGTGGTCCTCAGG

GACTGGGGGAGCTGGGCTGCTGGGGGCCCTGTCCTACCTGGGCCTCACCCAGGCCGGCCTCTCCCCTCAG

CAGACCCTGCTGTCCATGCTGGGTATCCCTGCCCTGCTGCTGGCCAGCTATTTCTTGTTGCTCACATCTC

CTGAGGCCCAGGACCCTGGAGGGGAAGAAGAAGCAGAGAGCGCAGCCCGGCAGCCCCTCATAAGAACCGA

GGCCCCGGAGTCGAAGCCAGGCTCCAGCTCCAGCCTCTCCCTTCGGGAAAGGTGGACAGTGTTCAAGGGT

CTGCTGTGGTACATTGTTCCCTTGGTCGTAGTTTACTTTGCCGAGTATTTCATTAACCAGGGACTTTTTG

AACTCCTCTTTTTCTGGAACACTTCCCTGAGTCACGCTCAGCAATACCGCTGGTACCAGATGCTGTACCA

GGCTGGCGTCTTTGCCTCCCGCTCTTCTCTCCGCTGCTGTCGCATCCGTTTCACCTGGGCCCTGGCCCTG

CTGCAGTGCCTCAACCTGGTGTTCCTGCTGGCAGACGTGTGGTTCGGCTTTCTGCCAAGCATCTACCTCG

TCTTCCTGATCATTCTGTATGAGGGGCTCCTGGGAGGCGCAGCCTACGTGAACACCTTCCACAACATCGC

CCTGGAGACCAGTGATGAGCACCGGGAGTTTGCAATGGCGGCCACCTGCATCTCTGACACACTGGGGATC

TCCCTGTCGGGGCTCCTGGCTTTGCCTCTGCATGACTTCCTCTGCCAGCTCTCCTGA

>lcl|NM_147780.4_cds_NP_680090.1_1 [gene=CTSB] [db_xref=CCDS:CCDS5986.1] [protein=cathepsin B isoform 1 preproprotein] [protein_id=NP_680090.1] [location=275..1294] [gbkey=CDS]

ATGTGGCAGCTCTGGGCCTCCCTCTGCTGCCTGCTGGTGTTGGCCAATGCCCGGAGCAGGCCCTCTTTCC

ATCCCCTGTCGGATGAGCTGGTCAACTATGTCAACAAACGGAATACCACGTGGCAGGCCGGGCACAACTT

CTACAACGTGGACATGAGCTACTTGAAGAGGCTATGTGGTACCTTCCTGGGTGGGCCCAAGCCACCCCAG

AGAGTTATGTTTACCGAGGACCTGAAGCTGCCTGCAAGCTTCGATGCACGGGAACAATGGCCACAGTGTC

CCACCATCAAAGAGATCAGAGACCAGGGCTCCTGTGGCTCCTGCTGGGCCTTCGGGGCTGTGGAAGCCAT

CTCTGACCGGATCTGCATCCACACCAATGCGCACGTCAGCGTGGAGGTGTCGGCGGAGGACCTGCTCACA

TGCTGTGGCAGCATGTGTGGGGACGGCTGTAATGGTGGCTATCCTGCTGAAGCTTGGAACTTCTGGACAA

GAAAAGGCCTGGTTTCTGGTGGCCTCTATGAATCCCATGTAGGGTGCAGACCGTACTCCATCCCTCCCTG

TGAGCACCACGTCAACGGCTCCCGGCCCCCATGCACGGGGGAGGGAGATACCCCCAAGTGTAGCAAGATC

TGTGAGCCTGGCTACAGCCCGACCTACAAACAGGACAAGCACTACGGATACAATTCCTACAGCGTCTCCA

ATAGCGAGAAGGACATCATGGCCGAGATCTACAAAAACGGCCCCGTGGAGGGAGCTTTCTCTGTGTATTC

GGACTTCCTGCTCTACAAGTCAGGAGTGTACCAACACGTCACCGGAGAGATGATGGGTGGCCATGCCATC

CGCATCCTGGGCTGGGGAGTGGAGAATGGCACACCCTACTGGCTGGTTGCCAACTCCTGGAACACTGACT

GGGGTGACAATGGCTTCTTTAAAATACTCAGAGGACAGGATCACTGTGGAATCGAATCAGAAGTGGTGGC

TGGAATTCCACGCACCGATCAGTACTGGGAAAAGATCTAA

>lcl|NM_001909.5_cds_NP_001900.1_1 [gene=CTSD] [db_xref=CCDS:CCDS7725.1] [protein=cathepsin D preproprotein] [protein_id=NP_001900.1] [location=69..1307] [gbkey=CDS]

ATGCAGCCCTCCAGCCTTCTGCCGCTCGCCCTCTGCCTGCTGGCTGCACCCGCCTCCGCGCTCGTCAGGA

TCCCGCTGCACAAGTTCACGTCCATCCGCCGGACCATGTCGGAGGTTGGGGGCTCTGTGGAGGACCTGAT

TGCCAAAGGCCCCGTCTCAAAGTACTCCCAGGCGGTGCCAGCCGTGACCGAGGGGCCCATTCCCGAGGTG

CTCAAGAACTACATGGACGCCCAGTACTACGGGGAGATTGGCATCGGGACGCCCCCCCAGTGCTTCACAG

TCGTCTTCGACACGGGCTCCTCCAACCTGTGGGTCCCCTCCATCCACTGCAAACTGCTGGACATCGCTTG

CTGGATCCACCACAAGTACAACAGCGACAAGTCCAGCACCTACGTGAAGAATGGTACCTCGTTTGACATC

CACTATGGCTCGGGCAGCCTCTCCGGGTACCTGAGCCAGGACACTGTGTCGGTGCCCTGCCAGTCAGCGT

CGTCAGCCTCTGCCCTGGGCGGTGTCAAAGTGGAGAGGCAGGTCTTTGGGGAGGCCACCAAGCAGCCAGG

CATCACCTTCATCGCAGCCAAGTTCGATGGCATCCTGGGCATGGCCTACCCCCGCATCTCCGTCAACAAC

GTGCTGCCCGTCTTCGACAACCTGATGCAGCAGAAGCTGGTGGACCAGAACATCTTCTCCTTCTACCTGA

GCAGGGACCCAGATGCGCAGCCTGGGGGTGAGCTGATGCTGGGTGGCACAGACTCCAAGTATTACAAGGG

TTCTCTGTCCTACCTGAATGTCACCCGCAAGGCCTACTGGCAGGTCCACCTGGACCAGGTGGAGGTGGCC

AGCGGGCTGACCCTGTGCAAGGAGGGCTGTGAGGCCATTGTGGACACAGGCACTTCCCTCATGGTGGGCC

CGGTGGATGAGGTGCGCGAGCTGCAGAAGGCCATCGGGGCCGTGCCGCTGATTCAGGGCGAGTACATGAT

CCCCTGTGAGAAGGTGTCCACCCTGCCCGCGATCACACTGAAGCTGGGAGGCAAAGGCTACAAGCTGTCC

CCAGAGGACTACACGCTCAAGGTGTCGCAGGCCGGGAAGACCCTCTGCCTGAGCGGCTTCATGGGCATGG

ACATCCCGCCACCCAGCGGGCCACTCTGGATCCTGGGCGACGTCTTCATCGGCCGCTACTACACTGTGTT

TGACCGTGACAACAACAGGGTGGGCTTCGCCGAGGCTGCCCGCCTCTAG

>lcl|NM_001912.5_cds_NP_001903.1_1 [gene=CTSL] [db_xref=CCDS:CCDS6675.1] [protein=procathepsin L isoform 1 preproprotein] [protein_id=NP_001903.1] [location=291..1292] [gbkey=CDS]

ATGAATCCTACACTCATCCTTGCTGCCTTTTGCCTGGGAATTGCCTCAGCTACTCTAACATTTGATCACA

GTTTAGAGGCACAGTGGACCAAGTGGAAGGCGATGCACAACAGATTATACGGCATGAATGAAGAAGGATG

GAGGAGAGCAGTGTGGGAGAAGAACATGAAGATGATTGAACTGCACAATCAGGAATACAGGGAAGGGAAA

CACAGCTTCACAATGGCCATGAACGCCTTTGGAGACATGACCAGTGAAGAATTCAGGCAGGTGATGAATG

GCTTTCAAAACCGTAAGCCCAGGAAGGGGAAAGTGTTCCAGGAACCTCTGTTTTATGAGGCCCCCAGATC

TGTGGATTGGAGAGAGAAAGGCTACGTGACTCCTGTGAAGAATCAGGGTCAGTGTGGTTCTTGTTGGGCT

TTTAGTGCTACTGGTGCTCTTGAAGGACAGATGTTCCGGAAAACTGGGAGGCTTATCTCACTGAGTGAGC

AGAATCTGGTAGACTGCTCTGGGCCTCAAGGCAATGAAGGCTGCAATGGTGGCCTAATGGATTATGCTTT

CCAGTATGTTCAGGATAATGGAGGCCTGGACTCTGAGGAATCCTATCCATATGAGGCAACAGAAGAATCC

TGTAAGTACAATCCCAAGTATTCTGTTGCTAATGACACCGGCTTTGTGGACATCCCTAAGCAGGAGAAGG

CCCTGATGAAGGCAGTTGCAACTGTGGGGCCCATTTCTGTTGCTATTGATGCAGGTCATGAGTCCTTCCT

GTTCTATAAAGAAGGCATTTATTTTGAGCCAGACTGTAGCAGTGAAGACATGGATCATGGTGTGCTGGTG

GTTGGCTACGGATTTGAAAGCACAGAATCAGATAACAATAAATATTGGCTGGTGAAGAACAGCTGGGGTG

AAGAATGGGGCATGGGTGGCTACGTAAAGATGGCCAAAGACCGGAGAAACCATTGTGGAATTGCCTCAGC

AGCCAGCTACCCCACTGTGTGA

>lcl|NM_002996.6_cds_NP_002987.1_1 [gene=CX3CL1] [db_xref=CCDS:CCDS10779.1] [protein=fractalkine isoform 1 precursor] [protein_id=NP_002987.1] [location=80..1273] [gbkey=CDS]

ATGGCTCCGATATCTCTGTCGTGGCTGCTCCGCTTGGCCACCTTCTGCCATCTGACTGTCCTGCTGGCTG

GACAGCACCACGGTGTGACGAAATGCAACATCACGTGCAGCAAGATGACATCAAAGATACCTGTAGCTTT

GCTCATCCACTATCAACAGAACCAGGCATCATGCGGCAAACGCGCAATCATCTTGGAGACGAGACAGCAC

AGGCTGTTCTGTGCCGACCCGAAGGAGCAATGGGTCAAGGACGCGATGCAGCATCTGGACCGCCAGGCTG

CTGCCCTAACTCGAAATGGCGGCACCTTCGAGAAGCAGATCGGCGAGGTGAAGCCCAGGACCACCCCTGC

CGCCGGGGGAATGGACGAGTCTGTGGTCCTGGAGCCCGAAGCCACAGGCGAAAGCAGTAGCCTGGAGCCG

ACTCCTTCTTCCCAGGAAGCACAGAGGGCCCTGGGGACCTCCCCAGAGCTGCCGACGGGCGTGACTGGTT

CCTCAGGGACCAGGCTCCCCCCGACGCCAAAGGCTCAGGATGGAGGGCCTGTGGGCACGGAGCTTTTCCG

AGTGCCTCCCGTCTCCACTGCCGCCACGTGGCAGAGTTCTGCTCCCCACCAACCTGGGCCCAGCCTCTGG

GCTGAGGCAAAGACCTCTGAGGCCCCGTCCACCCAGGACCCCTCCACCCAGGCCTCCACTGCGTCCTCCC

CAGCCCCAGAGGAGAATGCTCCGTCTGAAGGCCAGCGTGTGTGGGGTCAGGGACAGAGCCCCAGGCCAGA

GAACTCTCTGGAGCGGGAGGAGATGGGTCCCGTGCCAGCGCACACGGATGCCTTCCAGGACTGGGGGCCT

GGCAGCATGGCCCACGTCTCTGTGGTCCCTGTCTCCTCAGAAGGGACCCCCAGCAGGGAGCCAGTGGCTT

CAGGCAGCTGGACCCCTAAGGCTGAGGAACCCATCCATGCCACCATGGACCCCCAGAGGCTGGGCGTCCT

TATCACTCCTGTCCCTGACGCCCAGGCTGCCACCCGGAGGCAGGCGGTGGGGCTGCTGGCCTTCCTTGGC

CTCCTCTTCTGCCTGGGGGTGGCCATGTTCACCTACCAGAGCCTCCAGGGCTGCCCTCGAAAGATGGCAG

GAGAGATGGCGGAGGGCCTTCGCTACATCCCCCGGAGCTGTGGTAGTAATTCATATGTCCTGGTGCCCGT

GTGA

>lcl|NM_001008540.2_cds_NP_001008540.1_1 [gene=CXCR4] [db_xref=CCDS:CCDS33295.1] [protein=C-X-C chemokine receptor type 4 isoform a] [protein_id=NP_001008540.1] [location=297..1367] [gbkey=CDS]

ATGTCCATTCCTTTGCCTCTTTTGCAGATATACACTTCAGATAACTACACCGAGGAAATGGGCTCAGGGG

ACTATGACTCCATGAAGGAACCCTGTTTCCGTGAAGAAAATGCTAATTTCAATAAAATCTTCCTGCCCAC

CATCTACTCCATCATCTTCTTAACTGGCATTGTGGGCAATGGATTGGTCATCCTGGTCATGGGTTACCAG

AAGAAACTGAGAAGCATGACGGACAAGTACAGGCTGCACCTGTCAGTGGCCGACCTCCTCTTTGTCATCA

CGCTTCCCTTCTGGGCAGTTGATGCCGTGGCAAACTGGTACTTTGGGAACTTCCTATGCAAGGCAGTCCA

TGTCATCTACACAGTCAACCTCTACAGCAGTGTCCTCATCCTGGCCTTCATCAGTCTGGACCGCTACCTG

GCCATCGTCCACGCCACCAACAGTCAGAGGCCAAGGAAGCTGTTGGCTGAAAAGGTGGTCTATGTTGGCG

TCTGGATCCCTGCCCTCCTGCTGACTATTCCCGACTTCATCTTTGCCAACGTCAGTGAGGCAGATGACAG

ATATATCTGTGACCGCTTCTACCCCAATGACTTGTGGGTGGTTGTGTTCCAGTTTCAGCACATCATGGTT

GGCCTTATCCTGCCTGGTATTGTCATCCTGTCCTGCTATTGCATTATCATCTCCAAGCTGTCACACTCCA

AGGGCCACCAGAAGCGCAAGGCCCTCAAGACCACAGTCATCCTCATCCTGGCTTTCTTCGCCTGTTGGCT

GCCTTACTACATTGGGATCAGCATCGACTCCTTCATCCTCCTGGAAATCATCAAGCAAGGGTGTGAGTTT

GAGAACACTGTGCACAAGTGGATTTCCATCACCGAGGCCCTAGCTTTCTTCCACTGTTGTCTGAACCCCA

TCCTCTATGCTTTCCTTGGAGCCAAATTTAAAACCTCTGCCCAGCACGCACTCACCTCTGTGAGCAGAGG

GTCCAGCCTCAAGATCCTCTCCAAAGGAAAGCGAGGTGGACATTCATCTGTTTCCACTGAGTCTGAGTCT

TCAAGTTTTCACTCCAGCTAA

>lcl|NM_004938.4_cds_NP_004929.2_1 [gene=DAPK1] [db_xref=CCDS:CCDS43842.1] [protein=death-associated protein kinase 1] [protein_id=NP_004929.2] [location=350..4642] [gbkey=CDS]

ATGACCGTGTTCAGGCAGGAAAACGTGGATGATTACTACGACACCGGCGAGGAACTTGGCAGTGGACAGT

TTGCGGTTGTGAAGAAATGCCGTGAGAAAAGCACCGGCCTCCAGTATGCCGCCAAATTCATCAAGAAAAG

GAGGACTAAGTCCAGCCGGCGGGGTGTGAGCCGCGAGGACATCGAGCGGGAGGTCAGCATCCTGAAGGAG

ATCCAGCACCCCAATGTCATCACCCTGCACGAGGTCTATGAGAACAAGACGGACGTCATCCTGATCTTGG

AACTCGTTGCAGGTGGCGAGCTGTTTGACTTCTTAGCTGAAAAGGAATCTTTAACTGAAGAGGAAGCAAC

TGAATTTCTCAAACAAATTCTTAATGGTGTTTACTACCTGCACTCCCTTCAAATCGCCCACTTTGATCTT

AAGCCTGAGAACATAATGCTTTTGGATAGAAATGTCCCCAAACCTCGGATCAAGATCATTGACTTTGGGT

TGGCCCATAAAATTGACTTTGGAAATGAATTTAAAAACATATTTGGGACTCCAGAGTTTGTCGCTCCTGA

GATAGTCAACTATGAACCTCTTGGTCTTGAGGCAGATATGTGGAGTATCGGGGTAATAACCTATATCCTC

CTAAGTGGGGCCTCCCCATTTCTTGGAGACACTAAGCAAGAAACGTTAGCAAATGTATCCGCTGTCAACT

ACGAATTTGAGGATGAATACTTCAGTAATACCAGTGCCCTAGCCAAAGATTTCATAAGAAGACTTCTGGT

CAAGGATCCAAAGAAGAGAATGACAATTCAAGATAGTTTGCAGCATCCCTGGATCAAGCCTAAAGATACA

CAACAGGCACTTAGTAGAAAAGCATCAGCAGTAAACATGGAGAAATTCAAGAAGTTTGCAGCCCGGAAAA

AATGGAAACAATCCGTTCGCTTGATATCACTGTGCCAAAGATTATCCAGGTCATTCCTGTCCAGAAGTAA

CATGAGTGTTGCCAGAAGCGATGATACTCTGGATGAGGAAGACTCCTTTGTGATGAAAGCCATCATCCAT

GCCATCAACGATGACAATGTCCCAGGCCTGCAGCACCTTCTGGGCTCATTATCCAACTATGATGTTAACC

AACCCAACAAGCACGGGACACCTCCATTACTCATTGCTGCTGGCTGTGGGAATATTCAAATACTACAGTT

GCTCATTAAAAGAGGCTCGAGAATCGATGTCCAGGATAAGGGCGGGTCCAATGCCGTCTACTGGGCTGCT

CGGCATGGCCACGTCGATACCTTGAAATTTCTCAGTGAGAACAAATGCCCTTTGGATGTGAAAGACAAGT

CTGGAGAGATGGCCCTCCACGTGGCAGCTCGCTATGGCCATGCTGACGTGGCTCAGTTACTGTGCAGCTT

CGGCTCAAATCCCAATATCCAGGACAAGGAAGAAGAAACCCCCCTGCACTGTGCTGCTTGGCACGGCTAT

TACTCTGTGGCCAAAGCCCTTTGTGAAGCCGGCTGTAACGTGAACATCAAGAACCGAGAAGGAGAGACGC

CCCTCCTGACAGCCTCTGCCAGGGGCTACCACGACATCGTGGAGTGTCTGGCCGAACATGGAGCCGACCT

TAATGCTTGCGACAAGGACGGACACATTGCCCTTCATCTGGCTGTAAGACGGTGTCAGATGGAGGTAATC

AAGACTCTCCTCAGCCAAGGGTGTTTCGTCGATTATCAAGACAGGCACGGCAATACTCCCCTCCATGTGG

CATGTAAAGATGGCAACATGCCTATCGTGGTGGCCCTCTGTGAAGCAAACTGCAATTTGGACATCTCCAA

CAAGTATGGGCGAACGCCTCTGCACCTTGCGGCCAACAACGGAATCCTAGACGTGGTCCGGTATCTCTGT

CTGATGGGAGCCAGCGTTGAGGCGCTGACCACGGACGGAAAGACGGCAGAAGATCTTGCTAGATCGGAAC

AGCACGAGCACGTAGCAGGTCTCCTTGCAAGACTTCGAAAGGATACGCACCGAGGACTCTTCATCCAGCA

GCTCCGACCCACACAGAACCTGCAGCCAAGAATTAAGCTCAAGCTGTTTGGCCACTCGGGATCCGGGAAA

ACCACCCTTGTAGAATCTCTCAAGTGTGGGCTGCTGAGGAGCTTTTTCAGAAGGCGTCGGCCCAGACTGT

CTTCCACCAACTCCAGCAGGTTCCCACCTTCACCCCTGGCTTCTAAGCCCACAGTCTCAGTGAGCATCAA

CAACCTGTACCCAGGCTGCGAGAACGTGAGTGTGAGGAGCCGCAGCATGATGTTCGAGCCGGGTCTTACC

AAAGGGATGCTGGAGGTGTTTGTGGCCCCGACCCACCACCCGCACTGCTCGGCCGATGACCAGTCCACCA

AGGCCATCGACATCCAGAACGCTTATTTGAATGGAGTTGGCGATTTCAGCGTGTGGGAGTTCTCTGGAAA

TCCTGTGTATTTCTGCTGTTATGACTATTTTGCTGCAAATGATCCCACGTCAATCCATGTTGTTGTCTTT

AGTCTAGAAGAGCCCTATGAGATCCAGCTGAACCAAGTGATTTTCTGGCTCAGTTTCCTGAAGTCCCTTG

TCCCAGTTGAAGAACCCATAGCCTTCGGTGGCAAGCTGAAGAACCCACTCCAAGTTGTCCTGGTGGCCAC

CCACGCTGACATCATGAATGTTCCTCGACCGGCTGGAGGCGAGTTTGGATATGACAAAGACACATCGTTG

CTGAAAGAGATTAGGAACAGGTTTGGAAATGATCTTCACATTTCAAATAAGCTGTTTGTTCTGGATGCTG

GGGCTTCTGGGTCAAAGGACATGAAGGTACTTCGAAATCATCTGCAAGAAATACGAAGCCAGATTGTTTC

GGTCTGTCCTCCCATGACTCACCTGTGTGAGAAAATCATCTCCACGCTGCCTTCCTGGAGGAAGCTCAAT

GGACCCAACCAGCTGATGTCGCTGCAGCAGTTTGTGTACGACGTGCAGGACCAGCTGAACCCCCTGGCCA

GCGAGGAGGACCTCAGGCGCATTGCTCAGCAGCTCCACAGCACAGGCGAGATCAACATCATGCAAAGTGA

AACAGTTCAGGACGTGCTGCTCCTGGACCCCCGCTGGCTCTGCACAAACGTCCTGGGGAAGTTGCTGTCC

GTGGAGACCCCACGGGCGCTGCACCACTACCGGGGCCGCTACACCGTGGAGGACATCCAGCGCCTGGTGC

CCGACAGCGACGTGGAGGAGCTGCTGCAGATCCTCGATGCCATGGACATCTGCGCCCGGGACCTGAGCAG

CGGGACCATGGTGGACGTCCCAGCCCTGATCAAGACAGACAACCTGCACCGCTCCTGGGCTGATGAGGAG

GACGAGGTGATGGTGTATGGTGGCGTGCGCATCGTGCCCGTGGAACACCTCACCCCCTTCCCATGTGGCA

TCTTTCACAAGGTCCAGGTGAACCTGTGCCGGTGGATCCACCAGCAAAGCACAGAGGGCGACGCGGACAT

CCGCCTGTGGGTGAATGGCTGCAAGCTGGCCAACCGTGGGGCCGAGCTGCTGGTGCTGCTGGTCAACCAC

GGCCAGGGCATTGAGGTCCAGGTCCGCGGCCTGGAGACGGAGAAGATCAAGTGCTGCCTGCTGCTGGACT

CGGTGTGCAGCACCATTGAGAACGTCATGGCCACCACGCTGCCAGGGCTCCTGACCGTGAAGCATTACCT

GAGCCCCCAGCAGCTGCGGGAGCACCATGAGCCCGTCATGATCTACCAGCCACGGGACTTCTTCCGGGCA

CAGACTCTGAAGGAAACCTCACTGACCAACACCATGGGGGGGTACAAGGAAAGCTTCAGCAGCATCATGT

GCTTCGGGTGTCACGACGTCTACTCACAGGCCAGCCTCGGCATGGACATCCATGCATCAGACCTGAACCT

CCTCACTCGGAGGAAACTGAGTCGCCTGCTGGACCCGCCCGACCCCCTGGGGAAGGACTGGTGCCTTCTC

GCCATGAACTTAGGCCTCCCTGACCTCGTGGCAAAGTACAACACCAGTAACGGGGCTCCCAAGGATTTCC

TCCCCAGCCCCCTCCACGCCCTGCTGCGGGAATGGACCACCTACCCTGAGAGCACAGTGGGCACCCTCAT

GTCCAAACTGAGGGAGCTGGGTCGCCGGGATGCCGCAGACTTTTTGCTGAAGGCATCCTCTGTGTTCAAA

ATCAACCTGGATGGCAATGGCCAGGAGGCCTATGCCTCGAGCTGCAACAGCGGCACCTCTTACAATTCCA

TTAGCTCTGTTGTATCCCGGTGA

>lcl|NM_014326.5_cds_NP_055141.2_1 [gene=DAPK2] [db_xref=CCDS:CCDS10188.1] [protein=death-associated protein kinase 2 isoform 1] [protein_id=NP_055141.2] [location=195..1307] [gbkey=CDS]

ATGTTCCAGGCCTCAATGAGGAGTCCAAACATGGAGCCATTCAAGCAGCAGAAGGTGGAGGACTTTTATG

ACATCGGAGAGGAGCTGGGGAGTGGCCAGTTTGCCATCGTGAAGAAGTGCCGGGAGAAGAGCACGGGGCT

TGAGTATGCAGCCAAGTTCATCAAGAAGCGGCAGAGCCGGGCGAGCCGGCGCGGTGTGAGCCGGGAGGAG

ATCGAGCGGGAGGTGAGCATCCTGCGGCAGGTGCTGCACCACAATGTCATCACGCTGCACGACGTCTATG

AGAACCGCACCGACGTGGTGCTCATCCTTGAGCTAGTGTCTGGAGGAGAGCTCTTCGATTTCCTGGCCCA

GAAGGAGTCACTGAGTGAGGAGGAGGCCACCAGCTTCATTAAGCAGATCCTGGATGGGGTGAACTACCTT

CACACAAAGAAAATTGCTCACTTTGATCTCAAGCCAGAAAACATTATGTTGTTAGACAAGAATATTCCCA

TTCCACACATCAAGCTGATTGACTTTGGTCTGGCTCACGAAATAGAAGATGGAGTTGAATTTAAGAATAT

TTTTGGGACGCCGGAATTTGTTGCTCCAGAAATTGTGAACTACGAGCCCCTGGGTCTGGAGGCTGACATG

TGGAGCATAGGCGTCATCACCTACATCCTCTTAAGTGGAGCATCCCCTTTCCTGGGAGACACGAAGCAGG

AAACACTGGCAAATATCACAGCAGTGAGTTACGACTTTGATGAGGAATTCTTCAGCCAGACGAGCGAGCT

GGCCAAGGACTTTATTCGGAAGCTTCTGGTTAAAGAGACCCGGAAACGGCTCACAATCCAAGAGGCTCTC

AGACACCCCTGGATCACGCCGGTGGACAACCAGCAAGCCATGGTGCGCAGGGAGTCTGTGGTCAATCTGG

AGAACTTCAGGAAGCAGTATGTCCGCAGGCGGTGGAAGCTTTCCTTCAGCATCGTGTCCCTGTGCAACCA

CCTCACCCGCTCGCTGATGAAGAAGGTGCACCTGAGGCCGGATGAGGACCTGAGGAACTGTGAGAGTGAC

ACTGAGGAGGACATCGCCAGGAGGAAAGCCCTCCACCCACGGAGGAGGAGCAGCACCTCCTAA

>lcl|NM_004083.6_cds_NP_004074.2_1 [gene=DDIT3] [db_xref=CCDS:CCDS8943.1] [protein=DNA damage-inducible transcript 3 protein isoform 2] [protein_id=NP_004074.2] [location=178..687] [gbkey=CDS]

ATGGCAGCTGAGTCATTGCCTTTCTCCTTCGGGACACTGTCCAGCTGGGAGCTGGAAGCCTGGTATGAGG

ACCTGCAAGAGGTCCTGTCTTCAGATGAAAATGGGGGTACCTATGTTTCACCTCCTGGAAATGAAGAGGA

AGAATCAAAAATCTTCACCACTCTTGACCCTGCTTCTCTGGCTTGGCTGACTGAGGAGGAGCCAGAACCA

GCAGAGGTCACAAGCACCTCCCAGAGCCCTCACTCTCCAGATTCCAGTCAGAGCTCCCTGGCTCAGGAGG

AAGAGGAGGAAGACCAAGGGAGAACCAGGAAACGGAAACAGAGTGGTCATTCCCCAGCCCGGGCTGGAAA

GCAGCGCATGAAGGAGAAAGAACAGGAGAATGAAAGGAAAGTGGCACAGCTAGCTGAAGAGAATGAACGG

CTCAAGCAGGAAATCGAGCGCCTGACCAGGGAAGTAGAGGCGACTCGCCGAGCTCTGATTGACCGAATGG

TGAATCTGCACCAAGCATGA

>lcl|NM_004675.5_cds_NP_004666.1_1 [gene=DIRAS3] [db_xref=CCDS:CCDS641.1] [protein=GTP-binding protein Di-Ras3 precursor] [protein_id=NP_004666.1] [location=146..835] [gbkey=CDS]

ATGGGTAACGCCAGCTTTGGCTCCAAGGAACAGAAGCTGCTGAAGCGGTTGCGGCTTCTGCCCGCCCTGC

TTATCCTCCGCGCCTTCAAGCCCCACAGGAAGATCAGAGATTACCGCGTCGTGGTAGTCGGCACCGCTGG

TGTGGGGAAAAGTACGCTGCTGCACAAGTGGGCGAGCGGCAACTTCCGTCATGAGTACCTGCCGACCATT

GAAAATACCTACTGCCAGTTGCTGGGCTGCAGCCACGGTGTGCTTTCCCTGCACATCACCGACAGCAAGA

GTGGCGACGGCAACCGCGCTCTGCAGCGCCACGTTATAGCCCGGGGCCACGCCTTCGTCCTGGTCTACTC

AGTCACCAAGAAGGAAACCCTGGAAGAGCTGAAGGCCTTCTATGAGCTGATCTGCAAGATCAAAGGTAAC

AACCTGCATAAGTTCCCCATCGTGCTGGTGGGCAATAAAAGTGATGACACCCACCGGGAGGTGGCCCTGA

ATGATGGTGCCACCTGTGCGATGGAGTGGAATTGCGCCTTCATGGAGATTTCAGCCAAGACCGATGTGAA

TGTGCAGGAGCTGTTCCACATGCTGCTGAATTACAAGAAAAAGCCCACCACCGGCCTCCAGGAGCCCGAG

AAGAAATCCCAGATGCCCAACACCACTGAGAAGCTGCTTGACAAGTGCATAATCATGTGA

>lcl|NM_182643.3_cds_NP_872584.2_1 [gene=DLC1] [db_xref=CCDS:CCDS5989.1] [protein=rho GTPase-activating protein 7 isoform 1] [protein_id=NP_872584.2] [location=376..4962] [gbkey=CDS]

ATGTCTGTAGCTATCAGAAAGAGAAGCTGGGAAGAACATGTGACCCACTGGATGGGACAGCCTTTTAATT

CTGATGATCGTAACACAGCATGTCATCATGGACTAGTAGCTGACAGCTTGCAGGCAAGTATGGAAAAAGA

TGCAACTCTAAATGTGGACCGCAAAGAGAAGTGTGTTTCACTACCTGACTGCTGTCATGGATCAGAGCTG

AGAGATTTTCCTGGGAGGCCAATGGGTCATCTTTCAAAGGATGTGGACGAAAATGACAGCCATGAAGGTG

AAGATCAGTTTCTTTCTCTGGAAGCCAGCACAGAAACACTAGTGCATGTTTCTGATGAGGATAACAATGC

TGATTTATGCCTTACAGATGATAAACAGGTTTTAAATACCCAAGGGCAGAAAACATCAGGCCAACATATG

ATCCAAGGAGCAGGCTCCTTAGAAAAGGCACTGCCCATCATACAAAGTAACCAAGTTTCTTCTAACTCCT

GGGGAATAGCTGGTGAAACTGAATTAGCACTGGTAAAAGAAAGTGGGGAGAGAAAAGTTACTGACTCTAT

AAGTAAAAGCCTGGAGCTTTGCAATGAAATAAGCTTAAGTGAAATAAAAGATGCACCCAAAGTAAATGCA

GTGGATACTTTGAACGTGAAAGATATTGCACCTGAGAAACAATTGCTTAACTCTGCTGTAATTGCTCAGC

AACGAAGGAAACCTGACCCCCCTAAAGATGAAAATGAAAGAAGCACCTGCAATGTAGTACAAAATGAGTT

CTTGGATACTCCTTGCACAAACAGAGGACTGCCATTATTAAAAACAGATTTTGGAAGCTGCCTTCTGCAG

CCTCCTTCCTGCCCCAATGGAATGTCAGCTGAAAATGGCCTGGAGAAGAGTGGTTTTTCACAACATCAAA

ACAAAAGTCCACCAAAGGTCAAGGCAGAAGATGGCATGCAGTGTTTACAATTAAAGGAGACCCTGGCCAC

CCAGGAACCCACAGATAACCAAGTCAGACTTCGTAAGAGAAAGGAAATAAGAGAAGATCGAGATAGGGCG

CGGCTGGACTCCATGGTGCTGCTGATTATGAAACTGGACCAGCTTGATCAGGACATAGAAAATGCCCTCA

GCACCAGCTCCTCTCCATCAGGCACACCAACAAACCTGCGGCGGCACGTTCCTGATCTGGAATCAGGATC

TGAAAGTGGAGCAGATACCATTTCAGTAAATCAGACACGAGTAAATTTGTCTTCTGACACTGAGTCCACG

GACCTCCCATCTTCCACTCCAGTAGCCAATTCTGGAACCAAACCCAAGACTACGGCTATTCAAGGTATTT

CAGAGAAGGAAAAGGCTGAAATTGAAGCCAAGGAAGCTTGTGATTGGCTACGGGCAACTGGTTTCCCCCA

GTATGCACAGCTTTATGAAGATTTCCTGTTCCCCATCGATATTTCCTTGGTCAAGAGAGAGCATGATTTT

TTGGACAGAGATGCCATTGAGGCTCTATGCAGGCGTCTAAATACTTTAAACAAATGTGCGGTGATGAAGC

TAGAAATTAGTCCTCATCGGAAACGAAGTGACGATTCAGACGAGGATGAGCCTTGTGCCATCAGTGGCAA

ATGGACTTTCCAAAGGGACAGCAAGAGGTGGTCCCGGCTTGAAGAGTTTGATGTCTTTTCTCCAAAACAA

GACCTGGTCCCTGGGTCCCCAGACGACTCCCACCCGAAGGACGGCCCCAGCCCCGGAGGCACGCTGATGG

ACCTCAGCGAGCGCCAGGAGGTGTCTTCCGTCCGCAGCCTCAGCAGCACTGGCAGCCTCCCCAGCCACGC

GCCCCCCAGCGAGGATGCTGCCACCCCCCGGACTAACTCCGTCATCAGCGTTTGCTCCTCCAGCAACTTG

GCAGGCAATGACGACTCTTTCGGCAGCCTGCCCTCTCCCAAGGAACTGTCCAGCTTCAGCTTCAGCATGA

AAGGCCACGAAAAAACTGCCAAGTCCAAGACGCGCAGTCTGCTGAAACGGATGGAGAGCCTGAAGCTCAA

GAGCTCCCATCACAGCAAGCACAAAGCGCCCTCAAAGCTGGGGTTGATCATCAGCGGGCCCATCTTGCAA

GAGGGGATGGATGAGGAGAAGCTGAAGCAGCTCAACTGCGTGGAGATCTCCGCCCTCAATGGCAACCGCA

TCAACGTCCCCATGGTACGAAAGAGGAGCGTTTCCAACTCCACGCAGACCAGCAGCAGCAGCAGCCAGTC

GGAGACCAGCAGCGCGGTCAGCACGCCCAGCCCTGTTACGAGGACCCGGAGCCTCAGTGCGTGCAACAAG

CGGGTGGGCATGTACTTAGAGGGCTTCGATCCTTTCAATCAGTCAACATTTAACAACGTGGTGGAGCAGA

ACTTTAAGAACCGCGAGAGCTACCCAGAGGACACGGTGTTCTACATCCCTGAAGATCACAAGCCTGGCAC

TTTCCCCAAAGCTCTCACCAATGGCAGTTTCTCCCCCTCGGGGAATAACGGCTCTGTGAACTGGAGGACG

GGAAGCTTCCACGGCCCTGGCCACATCAGCCTCAGGAGGGAAAACAGTAGCGACAGCCCCAAGGAACTGA

AGAGACGCAATTCTTCCAGCTCCATGAGCAGCCGCCTGAGCATCTACGACAACGTGCCGGGCTCCATCCT

CTACTCCAGTTCAGGGGACCTGGCGGATCTGGAGAACGAGGACATCTTCCCCGAGCTGGACGACATCCTC

TACCACGTGAAGGGGATGCAGCGGATAGTCAATCAGTGGTCGGAGAAGTTTTCTGATGAGGGAGATTCGG

ACTCAGCCCTGGACTCGGTCTCTCCCTGCCCGTCCTCTCCAAAACAGATACACCTGGATGTGGACAACGA

CCGAACCACACCCAGCGACCTGGACAGCACAGGCAACTCCCTGAATGAACCGGAAGAGCCCTCCGAGATC

CCGGAAAGAAGGGATTCTGGGGTTGGGGCTTCCCTAACCAGGTCCAACAGGCACCGACTGAGATGGCACA

GTTTCCAGAGCTCACATCGGCCAAGCCTCAACTCTGTATCACTACAGATTAACTGCCAGTCTGTGGCCCA

GATGAACCTGCTGCAGAAATACTCACTCCTAAAGCTAACGGCCCTGCTGGAGAAATACACACCTTCTAAC

AAGCATGGTTTTAGCTGGGCCGTGCCCAAGTTCATGAAGAGGATCAAGGTTCCAGACTACAAGGACCGGA

GTGTGTTTGGGGTCCCACTGACGGTCAACGTGCAGCGCACAGGACAACCGTTGCCTCAGAGCATCCAGCA

GGCCATGCGATACCTCCGGAACCATTGTTTGGATCAGGTTGGGCTCTTCAGAAAATCGGGGGTCAAGTCC

CGGATTCAGGCTCTGCGCCAGATGAATGAAGGTGCCATAGACTGTGTCAACTACGAAGGACAGTCTGCTT

ATGACGTGGCAGACATGCTGAAGCAGTATTTTCGAGATCTTCCTGAGCCACTAATGACGAACAAACTCTC

GGAAACCTTTCTACAGATCTACCAATATGTGCCCAAGGACCAGCGCCTGCAGGCCATCAAGGCTGCCATC

ATGCTGCTGCCTGACGAGAACCGGGAGGTTCTGCAGACCCTGCTTTATTTCCTGAGCGATGTCACAGCAG

CCGTAAAAGAAAACCAGATGACCCCAACCAACCTGGCCGTGTGCTTAGCGCCTTCCCTCTTCCATCTCAA

CACCCTGAAGAGAGAGAATTCCTCTCCCAGGGTAATGCAAAGAAAACAAAGTTTGGGCAAACCAGATCAG

AAAGATTTGAATGAAAACCTAGCTGCCACTCAAGGGCTGGCCCATATGATCGCCGAGTGCAAGAAGCTTT

TCCAGGTTCCCGAGGAAATGAGCCGATGTCGTAATTCCTATACCGAACAAGAGCTGAAGCCCCTCACTCT

GGAAGCACTCGGGCACCTGGGTAATGATGACTCAGCTGACTACCAACACTTCCTCCAGGACTGTGTGGAT

GGCCTGTTTAAAGAAGTCAAAGAGAAGTTTAAAGGCTGGGTCAGCTACTCCACTTCGGAGCAGGCTGAGC

TGTCCTATAAGAAGGTGAGCGAAGGACCCCCTCTGAGGCTTTGGAGGTCAGTCATTGAAGTCCCTGCTGT

GCCAGAGGAAATCTTAAAGCGCCTACTTAAAGAACAGCACCTCTGGGATGTAGACCTGTTGGATTCAAAA

GTGATCGAAATTCTGGACAGCCAAACTGAAATTTACCAGTATGTCCAAAACAGTATGGCACCTCATCCTG

CTCGAGACTACGTTGTTTTAAGAACCTGGAGGACTAATTTACCCAAAGGAGCCTGTGCCCTTTTACTAAC

CTCTGTGGATCACGATCGCGCACCTGTGGTGGGTGTGAGGGTTAATGTGCTCTTGTCCAGGTATTTGATT

GAACCCTGTGGGCCAGGAAAATCCAAACTCACCTACATGTGCAGAGTTGACTTAAGGGGCCACATGCCAG

AATGGTACACAAAATCTTTTGGACATTTGTGTGCAGCTGAAGTTGTAAAGATCCGGGATTCCTTCAGTAA

CCAGAACACTGAAACCAAAGACACCAAATCTAGGTGA

>lcl|NM_006145.3_cds_NP_006136.1_1 [gene=DNAJB1] [db_xref=CCDS:CCDS12312.1] [protein=dnaJ homolog subfamily B member 1 isoform 1] [protein_id=NP_006136.1] [location=49..1071] [gbkey=CDS]

ATGGGTAAAGACTACTACCAGACGTTGGGCCTGGCCCGCGGCGCGTCGGACGAGGAGATCAAGCGGGCCT

ACCGCCGCCAGGCGCTGCGCTACCACCCGGACAAGAACAAGGAGCCCGGCGCCGAGGAGAAGTTCAAGGA

GATCGCTGAGGCCTACGACGTGCTCAGCGACCCGCGCAAGCGCGAGATCTTCGACCGCTACGGGGAGGAA

GGCCTAAAGGGGAGTGGCCCCAGTGGCGGTAGCGGCGGTGGTGCCAATGGTACCTCTTTCAGCTACACAT

TCCATGGAGACCCTCATGCCATGTTTGCTGAGTTCTTCGGTGGCAGAAATCCCTTTGACACCTTTTTTGG

GCAGCGGAACGGGGAGGAAGGCATGGACATTGATGACCCATTCTCTGGCTTCCCTATGGGCATGGGTGGC

TTCACCAACGTGAACTTTGGCCGCTCCCGCTCTGCCCAAGAGCCCGCCCGAAAGAAGCAAGATCCCCCAG

TCACCCACGACCTTCGAGTCTCCCTTGAAGAGATCTACAGCGGCTGTACCAAGAAGATGAAAATCTCCCA

CAAGCGGCTAAACCCCGACGGAAAGAGCATTCGAAACGAAGACAAAATATTGACCATCGAAGTGAAGAAG

GGGTGGAAAGAAGGAACCAAAATCACTTTCCCCAAGGAAGGAGACCAGACCTCCAACAACATTCCAGCTG

ATATCGTCTTTGTTTTAAAGGACAAGCCCCACAATATCTTTAAGAGAGATGGCTCTGATGTCATTTATCC

TGCCAGGATCAGCCTCCGGGAGGCTCTGTGTGGCTGCACAGTGAACGTCCCCACTCTGGACGGCAGGACG

ATACCCGTCGTATTCAAAGATGTTATCAGGCCTGGCATGCGGCGAAAAGTTCCTGGAGAAGGCCTCCCCC

TCCCCAAAACACCCGAGAAACGTGGGGACCTCATTATTGAGTTTGAAGTGATCTTCCCCGAAAGGATTCC

CCAGACATCAAGAACCGTACTTGAGCAGGTTCTTCCAATATAG

>lcl|NM_012328.3_cds_NP_036460.1_1 [gene=DNAJB9] [db_xref=CCDS:CCDS5752.1] [protein=dnaJ homolog subfamily B member 9 precursor] [protein_id=NP_036460.1] [location=241..912] [gbkey=CDS]

ATGGCTACTCCCCAGTCAATTTTCATCTTTGCAATCTGCATTTTAATGATAACAGAATTAATTCTGGCCT

CAAAAAGCTACTATGATATCTTAGGTGTGCCAAAATCGGCATCAGAGCGCCAAATCAAGAAGGCCTTTCA

CAAGTTGGCCATGAAGTACCACCCTGACAAAAATAAGAGCCCGGATGCTGAAGCAAAATTCAGAGAGATT

GCAGAAGCATATGAAACACTCTCAGATGCTAATAGACGAAAAGAGTATGATACACTTGGACACAGTGCTT

TTACTAGTGGTAAAGGACAAAGAGGTAGTGGAAGTTCTTTTGAGCAGTCATTTAACTTCAATTTTGATGA

CTTATTTAAAGACTTTGGCTTTTTTGGTCAAAACCAAAACACTGGATCCAAGAAGCGTTTTGAAAATCAT

TTCCAGACACGCCAGGATGGTGGTTCCAGTAGACAAAGGCATCATTTCCAAGAATTTTCTTTTGGAGGTG

GATTATTTGATGACATGTTTGAAGATATGGAGAAAATGTTTTCTTTTAGTGGTTTTGACTCTACCAATCA

GCATACAGTACAGACTGAAAATAGATTTCATGGATCTAGCAAGCACTGCAGGACTGTCACTCAACGAAGA

GGAAATATGGTTACTACATACACTGACTGTTCAGGACAGTAG

>lcl|NM_018370.3_cds_NP_060840.2_1 [gene=DRAM1] [db_xref=CCDS:CCDS41823.1] [protein=DNA damage-regulated autophagy modulator protein 1] [protein_id=NP_060840.2] [location=211..927] [gbkey=CDS]

ATGCTGTGCTTCCTGAGGGGAATGGCTTTCGTCCCCTTCCTCTTGGTGACCTGGTCGTCAGCCGCCTTCA

TTATCTCCTACGTGGTCGCCGTGCTCTCCGGGCACGTCAACCCCTTCCTCCCGTATATCAGTGATACGGG

AACAACACCTCCAGAGAGTGGTATTTTTGGATTTATGATAAACTTCTCTGCATTTCTTGGTGCAGCCACG

ATGTATACAAGATACAAAATAGTACAGAAGCAAAATCAAACCTGCTATTTCAGCACTCCTGTTTTTAACT

TGGTGTCTTTAGTGCTTGGATTGGTGGGATGTTTCGGAATGGGCATTGTCGCCAATTTTCAGGAGTTAGC

TGTGCCAGTGGTTCATGACGGGGGCGCTCTTTTGGCCTTTGTCTGTGGTGTCGTGTACACGCTCCTACAG

TCCATCATCTCTTACAAATCATGTCCCCAGTGGAACAGTCTCTCGACATGCCACATACGGATGGTCATCT

CTGCCGTTTCTTGCGCAGCTGTCATCCCCATGATTGTCTGTGCTTCACTAATTTCCATAACCAAGCTGGA

GTGGAATCCAAGAGAAAAGGATTATGTATATCACGTAGTGAGTGCGATCTGTGAATGGACAGTGGCCTTT

GGTTTTATTTTCTACTTCCTAACTTTCATCCAAGATTTCCAGAGTGTCACCCTAAGGATATCCACAGAAA

TCAATGGTGATATTTGA

>lcl|NM_014674.3_cds_NP_055489.1_1 [gene=EDEM1] [db_xref=CCDS:CCDS33686.1] [protein=ER degradation-enhancing alpha-mannosidase-like protein 1] [protein_id=NP_055489.1] [location=100..2073] [gbkey=CDS]

ATGCAATGGCGAGCGCTCGTCCTGGGGCTGGTGCTCCTCCGGCTTGGCCTCCATGGAGTATTGTGGCTCG

TCTTCGGGCTGGGGCCCAGCATGGGCTTCTACCAGCGCTTTCCGCTCAGCTTCGGCTTCCAGCGTCTGAG

GAGCCCCGACGGCCCCGCGTCGCCCACCTCGGGGCCCGTGGGCCGGCCTGGGGGGGTATCCGGGCCGTCG

TGGCTGCAGCCGCCGGGGACCGGGGCAGCGCAGAGCCCGCGCAAGGCTCCGCGGCGTCCTGGGCCGGGGA

TGTGCGGCCCAGCCAACTGGGGCTACGTGCTGGGCGGCCGGGGCCGCGGCCCGGACGAGTACGAGAAGCG

CTACAGCGGCGCCTTCCCTCCGCAGCTGCGTGCCCAGATGCGCGACCTGGCACGGGGCATGTTCGTCTTT

GGCTACGACAACTACATGGCTCACGCCTTCCCCCAGGACGAGCTCAACCCCATCCACTGCCGCGGCCGTG

GGCCCGACCGCGGGGACCCTTCAAATCTGAACATCAATGATGTACTAGGGAACTACTCATTGACTCTTGT

TGATGCATTGGATACACTTGCAATAATGGGAAATTCATCCGAGTTCCAGAAAGCCGTCAAGTTAGTGATC

AACACAGTTTCATTTGACAAAGATTCCACCGTCCAAGTCTTTGAGGCCACGATAAGGGTCCTGGGAAGCC

TCCTTTCTGCTCACAGAATAATAACTGACTCCAAGCAGCCCTTTGGTGACATGACAATTAAGGACTATGA

TAATGAGTTGTTATACATGGCCCATGACCTGGCGGTGCGGCTCCTCCCTGCTTTTGAAAACACCAAGACA

GGGATTCCATATCCTCGGGTGAATCTAAAGACAGGAGTTCCTCCTGACACCAATAATGAGACATGCACAG

CGGGAGCCGGTTCCCTCCTGGTGGAATTTGGGATTCTGAGTCGACTCCTGGGGGACTCCACATTTGAGTG

GGTGGCCAGACGAGCAGTGAAAGCCCTTTGGAACCTCCGGAGCAATGATACAGGATTACTAGGCAATGTC

GTGAACATTCAGACGGGCCACTGGGTTGGAAAGCAGAGTGGCCTGGGTGCCGGGCTGGACTCCTTCTATG

AATACCTCTTGAAATCTTACATTCTCTTTGGAGAAAAAGAAGACCTAGAAATGTTTAATGCTGCATATCA

GAGTATTCAGAACTACTTAAGAAGAGGGCGGGAAGCCTGCAATGAAGGAGAAGGAGACCCTCCACTCTAT

GTCAACGTGAACATGTTCAGTGGGCAGCTGATGAACACCTGGATTGACTCTCTGCAGGCCTTTTTCCCTG

GACTGCAGGTGCTGATAGGAGATGTGGAAGATGCCATCTGCCTTCATGCCTTCTACTATGCCATATGGAA

ACGATATGGTGCCCTCCCTGAGAGATATAACTGGCAGCTGCAGGCCCCTGACGTTCTCTTCTACCCACTG

AGACCAGAGTTAGTGGAATCCACATATCTCCTCTACCAGGCAACCAAGAATCCCTTCTACCTCCATGTAG

GAATGGATATTCTGCAGAGTCTGGAAAAGTACACAAAAGTCAAGTGTGGGTACGCCACGCTGCATCACGT

CATTGACAAGTCCACAGAAGACCGGATGGAGAGCTTCTTTCTCAGTGAGACCTGTAAATATTTGTATCTG

CTGTTTGATGAAGACAATCCAGTACACAAGTCTGGAACCAGATACATGTTCACAACAGAGGGACACATTG

TATCTGTGGATGAGCATCTTCGGGAATTGCCATGGAAGGAATTCTTCTCTGAAGAGGGAGGGCAGGACCA

AGGGGGAAAGTCTGTGCACAGGCCGAAACCTCATGAGTTAAAAGTCATCAACTCCAGCTCCAACTGCAAT

CGTGTACCTGATGAGAGGAGGTACTCCCTGCCCTTAAAGAGCATCTACATGCGACAGATTGACCAGATGG

TTGGTTTGATTTGA

>lcl|NM_001961.4_cds_NP_001952.1_1 [gene=EEF2] [db_xref=CCDS:CCDS12117.1] [protein=elongation factor 2] [protein_id=NP_001952.1] [location=84..2660] [gbkey=CDS]

ATGGTGAACTTCACGGTAGACCAGATCCGCGCCATCATGGACAAGAAGGCCAACATCCGCAACATGTCTG

TCATCGCCCACGTGGACCATGGCAAGTCCACGCTGACAGACTCCCTGGTGTGCAAGGCGGGCATCATCGC

CTCGGCCCGGGCCGGGGAGACACGCTTCACTGATACCCGGAAGGACGAGCAGGAGCGTTGCATCACCATC

AAGTCAACTGCCATCTCCCTCTTCTACGAGCTCTCGGAGAATGACTTGAACTTCATCAAGCAGAGCAAGG

ACGGTGCCGGCTTCCTCATCAACCTCATTGACTCCCCCGGGCATGTCGACTTCTCCTCGGAGGTGACTGC

TGCCCTCCGAGTCACCGATGGCGCATTGGTGGTGGTGGACTGCGTGTCAGGCGTGTGCGTGCAGACGGAG

ACAGTGCTGCGGCAGGCCATTGCCGAGCGCATCAAGCCTGTGCTGATGATGAACAAGATGGACCGCGCCC

TGCTGGAGCTGCAGCTGGAGCCCGAGGAGCTCTACCAGACTTTCCAGCGCATCGTGGAGAACGTGAACGT

CATCATCTCCACCTACGGCGAGGGCGAGAGCGGCCCCATGGGCAACATCATGATCGATCCTGTCCTCGGT

ACCGTGGGCTTTGGGTCTGGCCTCCACGGGTGGGCCTTCACCCTGAAGCAGTTTGCCGAGATGTATGTGG

CCAAGTTCGCCGCCAAGGGGGAGGGCCAGTTGGGGCCTGCCGAGCGGGCCAAGAAAGTAGAGGACATGAT

GAAGAAGCTGTGGGGTGACAGGTACTTTGACCCAGCCAACGGCAAGTTCAGCAAGTCAGCCACCAGCCCC

GAAGGGAAGAAGCTGCCACGCACCTTCTGCCAGCTGATCCTGGACCCCATCTTCAAGGTGTTTGATGCGA

TCATGAATTTCAAGAAAGAGGAGACAGCAAAACTGATAGAGAAACTGGACATCAAACTGGACAGCGAGGA

CAAGGACAAAGAAGGCAAACCCCTGCTGAAGGCTGTGATGCGCCGCTGGCTGCCTGCCGGAGACGCCTTG

TTGCAGATGATCACCATCCACCTGCCCTCCCCTGTGACGGCCCAGAAGTACCGCTGCGAGCTCCTGTACG

AGGGGCCCCCGGACGACGAGGCTGCCATGGGCATTAAAAGCTGTGACCCCAAAGGCCCTCTTATGATGTA

TATTTCCAAAATGGTGCCAACCTCCGACAAAGGTCGGTTCTACGCCTTTGGACGAGTCTTCTCGGGGCTG

GTCTCCACTGGCCTGAAGGTCAGGATCATGGGGCCCAACTATACCCCTGGGAAGAAGGAGGACCTCTACC

TGAAGCCAATCCAGAGAACAATCTTGATGATGGGCCGCTACGTGGAGCCCATCGAGGATGTGCCTTGTGG

GAACATTGTGGGCCTCGTGGGCGTGGACCAGTTCCTGGTGAAGACGGGCACCATCACCACCTTCGAGCAC

GCGCACAACATGCGGGTGATGAAGTTCAGCGTCAGCCCTGTTGTCAGAGTGGCCGTGGAGGCCAAGAACC

CGGCTGACCTGCCCAAGCTGGTGGAGGGGCTGAAGCGGCTGGCCAAGTCCGACCCCATGGTGCAGTGCAT

CATCGAGGAGTCGGGAGAGCATATCATCGCGGGCGCCGGCGAGCTGCACCTGGAGATCTGCCTGAAGGAC

CTGGAGGAGGACCACGCCTGCATCCCCATCAAGAAATCTGACCCGGTCGTCTCGTACCGCGAGACGGTCA

GTGAAGAGTCGAACGTGCTCTGCCTCTCCAAGTCCCCCAACAAGCACAACCGGCTGTACATGAAGGCGCG

GCCCTTCCCCGACGGCCTGGCCGAGGACATCGATAAAGGCGAGGTGTCCGCCCGTCAGGAGCTCAAGCAG

CGGGCGCGCTACCTGGCCGAGAAGTACGAGTGGGACGTGGCTGAGGCCCGCAAGATCTGGTGCTTTGGGC

CCGACGGCACCGGCCCCAACATCCTCACCGACATCACCAAGGGTGTGCAGTACCTCAACGAGATCAAGGA

CAGTGTGGTGGCCGGCTTCCAGTGGGCCACCAAGGAGGGCGCACTGTGTGAGGAGAACATGCGGGGTGTG

CGCTTCGACGTCCACGACGTCACCCTGCACGCCGACGCCATCCACCGCGGAGGGGGCCAGATCATCCCCA

CAGCACGGCGCTGCCTCTATGCCAGTGTGCTGACCGCCCAGCCACGCCTCATGGAGCCCATCTACCTTGT

GGAGATCCAGTGTCCAGAGCAGGTGGTCGGTGGCATCTACGGGGTTTTGAACAGGAAGCGGGGCCACGTG

TTCGAGGAGTCCCAGGTGGCCGGCACCCCCATGTTTGTGGTCAAGGCCTATCTGCCCGTCAACGAGTCCT

TTGGCTTCACCGCTGACCTGAGGTCCAACACGGGCGGCCAGGCGTTCCCCCAGTGTGTGTTTGACCACTG

GCAGATCCTGCCCGGAGACCCCTTCGACAACAGCAGCCGCCCCAGCCAGGTGGTGGCGGAGACCCGCAAG

CGCAAGGGCCTGAAAGAAGGCATCCCTGCCCTGGACAACTTCCTGGACAAATTGTAG

>lcl|NM_013302.5_cds_NP_037434.2_1 [gene=EEF2K] [db_xref=CCDS:CCDS10604.1] [protein=eukaryotic elongation factor 2 kinase] [protein_id=NP_037434.2] [location=479..2656] [gbkey=CDS]

ATGGCAGACGAAGATCTCATCTTCCGCCTGGAAGGCGTTGATGGCGGCCAGTCCCCCCGAGCTGGCCATG

ATGGTGATTCTGATGGGGACAGCGACGATGAGGAAGGTTACTTCATCTGCCCCATCACGGATGACCCAAG

CTCGAACCAGAATGTCAATTCCAAGGTTAATAAGTACTACAGCAACCTAACAAAAAGTGAGCGGTATAGC

TCCAGCGGGTCCCCGGCAAACTCCTTCCACTTCAAGGAAGCCTGGAAGCACGCAATCCAGAAGGCCAAGC

ACATGCCCGACCCCTGGGCTGAGTTCCACCTGGAAGATATTGCCACCGAACGTGCTACTCGACACAGGTA

CAACGCCGTCACCGGGGAATGGCTGGATGATGAAGTTCTGATCAAGATGGCATCTCAGCCCTTCGGCCGA

GGAGCAATGAGGGAGTGCTTCCGGACGAAGAAGCTCTCCAACTTCTTGCATGCCCAGCAGTGGAAGGGCG

CCTCCAACTACGTGGCGAAGCGCTACATCGAGCCCGTAGACCGGGATGTGTACTTTGAGGACGTGCGTCT

ACAGATGGAGGCCAAGCTCTGGGGGGAGGAGTATAATCGGCACAAGCCCCCCAAGCAGGTGGACATCATG

CAGATGTGCATCATCGAGCTGAAGGACAGACCGGGCAAGCCCCTCTTCCACCTGGAGCACTACATCGAGG

GCAAGTACATCAAGTACAACTCCAACTCTGGCTTTGTCCGCGATGACAACATCCGCCTGACGCCGCAGGC

CTTCAGCCACTTCACTTTTGAGCGTTCCGGCCATCAGCTGATAGTGGTGGACATCCAGGGAGTTGGGGAT

CTCTACACTGACCCACAGATCCACACGGAGACGGGCACTGACTTTGGAGACGGCAACCTAGGTGTCCGCG

GGATGGCGCTCTTCTTCTACTCTCATGCCTGCAACCGGATTTGCGAGAGCATGGGCCTTGCTCCCTTTGA

CCTCTCGCCCCGGGAGAGGGATGCAGTGAATCAGAACACCAAGCTGCTGCAATCAGCCAAGACCATCTTG

AGAGGAACAGAGGAAAAATGTGGGAGCCCCCAAGTAAGGACCCTCTCTGGGAGCCGGCCACCCCTGCTCC

GTCCCCTTTCAGAGAACTCTGGAGACGAGAACATGAGCGACGTGACCTTCGACTCTCTCCCTTCTTCCCC

ATCTTCGGCCACACCACACAGCCAGAAGCTAGACCACCTCCATTGGCCAGTGTTCAGTGACCTCGATAAC

ATGGCATCCAGAGACCATGATCATCTAGACAACCACCGGGAGTCTGAGAATAGTGGGGACAGCGGATACC

CCAGTGAGAAGCGGGGTGAGCTGGATGACCCTGAGCCCCGAGAACATGGCCACTCATACAGTAATCGGAA

GTACGAGTCTGACGAAGACAGCCTGGGCAGCTCTGGACGGGTATGTGTAGAGAAGTGGAATCTCCTCAAC

TCCTCCCGCCTCCACCTGCCGAGGGCTTCGGCCGTGGCCCTGGAAGTGCAAAGGCTTAATGCTCTGGACC

TCGAAAAGAAAATCGGGAAGTCCATTTTGGGGAAGGTCCATCTGGCCATGGTGCGCTACCACGAGGGTGG

GCGCTTCTGCGAGAAGGGCGAGGAGTGGGACCAGGAGTCGGCTGTCTTCCACCTGGAGCACGCAGCCAAC

CTGGGCGAGCTGGAGGCCATCGTGGGCCTGGGACTCATGTACTCGCAGTTGCCTCATCACATCCTAGCCG

ATGTCTCTCTGAAGGAGACAGAAGAGAACAAAACCAAAGGATTTGATTACTTACTAAAGGCCGCTGAAGC

TGGCGACAGGCAGTCCATGATCCTAGTGGCGCGAGCTTTTGACTCTGGCCAGAACCTCAGCCCGGACAGG

TGCCAAGACTGGCTAGAGGCCCTGCACTGGTACAACACTGCCCTGGAGATGACGGACTGTGATGAGGGCG

GTGAGTACGACGGAATGCAGGACGAGCCCCGGTACATGATGCTGGCCAGGGAGGCCGAGATGCTGTTCAC

AGGAGGCTACGGGCTGGAGAAGGACCCGCAGAGATCAGGGGACTTGTATACCCAGGCAGCAGAGGCAGCG

ATGGAAGCCATGAAGGGCCGACTGGCCAACCAGTACTACCAAAAGGCTGAAGAGGCCTGGGCCCAGATGG

AGGAGTAA

>lcl|NM_005228.5_cds_NP_005219.2_1 [gene=EGFR] [db_xref=CCDS:CCDS5514.1] [protein=epidermal growth factor receptor isoform a precursor] [protein_id=NP_005219.2] [location=262..3894] [gbkey=CDS]

ATGCGACCCTCCGGGACGGCCGGGGCAGCGCTCCTGGCGCTGCTGGCTGCGCTCTGCCCGGCGAGTCGGG

CTCTGGAGGAAAAGAAAGTTTGCCAAGGCACGAGTAACAAGCTCACGCAGTTGGGCACTTTTGAAGATCA

TTTTCTCAGCCTCCAGAGGATGTTCAATAACTGTGAGGTGGTCCTTGGGAATTTGGAAATTACCTATGTG

CAGAGGAATTATGATCTTTCCTTCTTAAAGACCATCCAGGAGGTGGCTGGTTATGTCCTCATTGCCCTCA

ACACAGTGGAGCGAATTCCTTTGGAAAACCTGCAGATCATCAGAGGAAATATGTACTACGAAAATTCCTA

TGCCTTAGCAGTCTTATCTAACTATGATGCAAATAAAACCGGACTGAAGGAGCTGCCCATGAGAAATTTA

CAGGAAATCCTGCATGGCGCCGTGCGGTTCAGCAACAACCCTGCCCTGTGCAACGTGGAGAGCATCCAGT

GGCGGGACATAGTCAGCAGTGACTTTCTCAGCAACATGTCGATGGACTTCCAGAACCACCTGGGCAGCTG

CCAAAAGTGTGATCCAAGCTGTCCCAATGGGAGCTGCTGGGGTGCAGGAGAGGAGAACTGCCAGAAACTG

ACCAAAATCATCTGTGCCCAGCAGTGCTCCGGGCGCTGCCGTGGCAAGTCCCCCAGTGACTGCTGCCACA

ACCAGTGTGCTGCAGGCTGCACAGGCCCCCGGGAGAGCGACTGCCTGGTCTGCCGCAAATTCCGAGACGA

AGCCACGTGCAAGGACACCTGCCCCCCACTCATGCTCTACAACCCCACCACGTACCAGATGGATGTGAAC

CCCGAGGGCAAATACAGCTTTGGTGCCACCTGCGTGAAGAAGTGTCCCCGTAATTATGTGGTGACAGATC

ACGGCTCGTGCGTCCGAGCCTGTGGGGCCGACAGCTATGAGATGGAGGAAGACGGCGTCCGCAAGTGTAA

GAAGTGCGAAGGGCCTTGCCGCAAAGTGTGTAACGGAATAGGTATTGGTGAATTTAAAGACTCACTCTCC

ATAAATGCTACGAATATTAAACACTTCAAAAACTGCACCTCCATCAGTGGCGATCTCCACATCCTGCCGG

TGGCATTTAGGGGTGACTCCTTCACACATACTCCTCCTCTGGATCCACAGGAACTGGATATTCTGAAAAC

CGTAAAGGAAATCACAGGGTTTTTGCTGATTCAGGCTTGGCCTGAAAACAGGACGGACCTCCATGCCTTT

GAGAACCTAGAAATCATACGCGGCAGGACCAAGCAACATGGTCAGTTTTCTCTTGCAGTCGTCAGCCTGA

ACATAACATCCTTGGGATTACGCTCCCTCAAGGAGATAAGTGATGGAGATGTGATAATTTCAGGAAACAA

AAATTTGTGCTATGCAAATACAATAAACTGGAAAAAACTGTTTGGGACCTCCGGTCAGAAAACCAAAATT

ATAAGCAACAGAGGTGAAAACAGCTGCAAGGCCACAGGCCAGGTCTGCCATGCCTTGTGCTCCCCCGAGG

GCTGCTGGGGCCCGGAGCCCAGGGACTGCGTCTCTTGCCGGAATGTCAGCCGAGGCAGGGAATGCGTGGA

CAAGTGCAACCTTCTGGAGGGTGAGCCAAGGGAGTTTGTGGAGAACTCTGAGTGCATACAGTGCCACCCA

GAGTGCCTGCCTCAGGCCATGAACATCACCTGCACAGGACGGGGACCAGACAACTGTATCCAGTGTGCCC

ACTACATTGACGGCCCCCACTGCGTCAAGACCTGCCCGGCAGGAGTCATGGGAGAAAACAACACCCTGGT

CTGGAAGTACGCAGACGCCGGCCATGTGTGCCACCTGTGCCATCCAAACTGCACCTACGGATGCACTGGG

CCAGGTCTTGAAGGCTGTCCAACGAATGGGCCTAAGATCCCGTCCATCGCCACTGGGATGGTGGGGGCCC

TCCTCTTGCTGCTGGTGGTGGCCCTGGGGATCGGCCTCTTCATGCGAAGGCGCCACATCGTTCGGAAGCG

CACGCTGCGGAGGCTGCTGCAGGAGAGGGAGCTTGTGGAGCCTCTTACACCCAGTGGAGAAGCTCCCAAC

CAAGCTCTCTTGAGGATCTTGAAGGAAACTGAATTCAAAAAGATCAAAGTGCTGGGCTCCGGTGCGTTCG

GCACGGTGTATAAGGGACTCTGGATCCCAGAAGGTGAGAAAGTTAAAATTCCCGTCGCTATCAAGGAATT

AAGAGAAGCAACATCTCCGAAAGCCAACAAGGAAATCCTCGATGAAGCCTACGTGATGGCCAGCGTGGAC

AACCCCCACGTGTGCCGCCTGCTGGGCATCTGCCTCACCTCCACCGTGCAGCTCATCACGCAGCTCATGC

CCTTCGGCTGCCTCCTGGACTATGTCCGGGAACACAAAGACAATATTGGCTCCCAGTACCTGCTCAACTG

GTGTGTGCAGATCGCAAAGGGCATGAACTACTTGGAGGACCGTCGCTTGGTGCACCGCGACCTGGCAGCC

AGGAACGTACTGGTGAAAACACCGCAGCATGTCAAGATCACAGATTTTGGGCTGGCCAAACTGCTGGGTG

CGGAAGAGAAAGAATACCATGCAGAAGGAGGCAAAGTGCCTATCAAGTGGATGGCATTGGAATCAATTTT

ACACAGAATCTATACCCACCAGAGTGATGTCTGGAGCTACGGGGTGACTGTTTGGGAGTTGATGACCTTT

GGATCCAAGCCATATGACGGAATCCCTGCCAGCGAGATCTCCTCCATCCTGGAGAAAGGAGAACGCCTCC

CTCAGCCACCCATATGTACCATCGATGTCTACATGATCATGGTCAAGTGCTGGATGATAGACGCAGATAG

TCGCCCAAAGTTCCGTGAGTTGATCATCGAATTCTCCAAAATGGCCCGAGACCCCCAGCGCTACCTTGTC

ATTCAGGGGGATGAAAGAATGCATTTGCCAAGTCCTACAGACTCCAACTTCTACCGTGCCCTGATGGATG

AAGAAGACATGGACGACGTGGTGGATGCCGACGAGTACCTCATCCCACAGCAGGGCTTCTTCAGCAGCCC

CTCCACGTCACGGACTCCCCTCCTGAGCTCTCTGAGTGCAACCAGCAACAATTCCACCGTGGCTTGCATT

GATAGAAATGGGCTGCAAAGCTGTCCCATCAAGGAAGACAGCTTCTTGCAGCGATACAGCTCAGACCCCA

CAGGCGCCTTGACTGAGGACAGCATAGACGACACCTTCCTCCCAGTGCCTGAATACATAAACCAGTCCGT

TCCCAAAAGGCCCGCTGGCTCTGTGCAGAATCCTGTCTATCACAATCAGCCTCTGAACCCCGCGCCCAGC

AGAGACCCACACTACCAGGACCCCCACAGCACTGCAGTGGGCAACCCCGAGTATCTCAACACTGTCCAGC

CCACCTGTGTCAACAGCACATTCGACAGCCCTGCCCACTGGGCCCAGAAAGGCAGCCACCAAATTAGCCT

GGACAACCCTGACTACCAGCAGGACTTCTTTCCCAAGGAAGCCAAGCCAAATGGCATCTTTAAGGGCTCC

ACAGCTGAAAATGCAGAATACCTAAGGGTCGCGCCACAAAGCAGTGAATTTATTGGAGCATGA

>lcl|NM_002759.4_cds_NP_002750.1_1 [gene=EIF2AK2] [db_xref=CCDS:CCDS1786.1] [protein=interferon-induced, double-stranded RNA-activated protein kinase isoform a] [protein_id=NP_002750.1] [location=491..2146] [gbkey=CDS]

ATGGCTGGTGATCTTTCAGCAGGTTTCTTCATGGAGGAACTTAATACATACCGTCAGAAGCAGGGAGTAG

TACTTAAATATCAAGAACTGCCTAATTCAGGACCTCCACATGATAGGAGGTTTACATTTCAAGTTATAAT

AGATGGAAGAGAATTTCCAGAAGGTGAAGGTAGATCAAAGAAGGAAGCAAAAAATGCCGCAGCCAAATTA

GCTGTTGAGATACTTAATAAGGAAAAGAAGGCAGTTAGTCCTTTATTATTGACAACAACGAATTCTTCAG

AAGGATTATCCATGGGGAATTACATAGGCCTTATCAATAGAATTGCCCAGAAGAAAAGACTAACTGTAAA

TTATGAACAGTGTGCATCGGGGGTGCATGGGCCAGAAGGATTTCATTATAAATGCAAAATGGGACAGAAA

GAATATAGTATTGGTACAGGTTCTACTAAACAGGAAGCAAAACAATTGGCCGCTAAACTTGCATATCTTC

AGATATTATCAGAAGAAACCTCAGTGAAATCTGACTACCTGTCCTCTGGTTCTTTTGCTACTACGTGTGA

GTCCCAAAGCAACTCTTTAGTGACCAGCACACTCGCTTCTGAATCATCATCTGAAGGTGACTTCTCAGCA

GATACATCAGAGATAAATTCTAACAGTGACAGTTTAAACAGTTCTTCGTTGCTTATGAATGGTCTCAGAA

ATAATCAAAGGAAGGCAAAAAGATCTTTGGCACCCAGATTTGACCTTCCTGACATGAAAGAAACAAAGTA

TACTGTGGACAAGAGGTTTGGCATGGATTTTAAAGAAATAGAATTAATTGGCTCAGGTGGATTTGGCCAA

GTTTTCAAAGCAAAACACAGAATTGACGGAAAGACTTACGTTATTAAACGTGTTAAATATAATAACGAGA

AGGCGGAGCGTGAAGTAAAAGCATTGGCAAAACTTGATCATGTAAATATTGTTCACTACAATGGCTGTTG

GGATGGATTTGATTATGATCCTGAGACCAGTGATGATTCTCTTGAGAGCAGTGATTATGATCCTGAGAAC

AGCAAAAATAGTTCAAGGTCAAAGACTAAGTGCCTTTTCATCCAAATGGAATTCTGTGATAAAGGGACCT

TGGAACAATGGATTGAAAAAAGAAGAGGCGAGAAACTAGACAAAGTTTTGGCTTTGGAACTCTTTGAACA

AATAACAAAAGGGGTGGATTATATACATTCAAAAAAATTAATTCATAGAGATCTTAAGCCAAGTAATATA

TTCTTAGTAGATACAAAACAAGTAAAGATTGGAGACTTTGGACTTGTAACATCTCTGAAAAATGATGGAA

AGCGAACAAGGAGTAAGGGAACTTTGCGATACATGAGCCCAGAACAGATTTCTTCGCAAGACTATGGAAA

GGAAGTGGACCTCTACGCTTTGGGGCTAATTCTTGCTGAACTTCTTCATGTATGTGACACTGCTTTTGAA

ACATCAAAGTTTTTCACAGACCTACGGGATGGCATCATCTCAGATATATTTGATAAAAAAGAAAAAACTC

TTCTACAGAAATTACTCTCAAAGAAACCTGAGGATCGACCTAACACATCTGAAATACTAAGGACCTTGAC

TGTGTGGAAGAAAAGCCCAGAGAAAAATGAACGACACACATGTTAG

>lcl|NM_004836.7_cds_NP_004827.4_1 [gene=EIF2AK3] [db_xref=CCDS:CCDS33241.1] [protein=eukaryotic translation initiation factor 2-alpha kinase 3 isoform 1 precursor] [protein_id=NP_004827.4] [location=191..3541] [gbkey=CDS]

ATGGAGCGCGCCATCAGCCCGGGGCTGCTGGTACGGGCGCTGCTGCTGCTGCTGCTGCTGCTGGGGCTCG

CGGCAAGGACGGTGGCCGCGGGGCGCGCCCGTGGCCTCCCAGCGCCGACGGCGGAGGCGGCGTTCGGCCT

CGGGGCGGCCGCTGCTCCCACCTCAGCGACGCGAGTACCGGCGGCGGGCGCCGTGGCTGCGGCCGAGGTG

ACTGTGGAGGACGCTGAGGCGCTGCCGGCAGCCGCGGGAGAGCAGGAGCCTCGGGGTCCGGAACCAGACG

ATGAGACAGAGTTGCGACCGCGCGGCAGGTCATTAGTAATTATCAGCACTTTAGATGGGAGAATTGCTGC

CTTGGATCCTGAAAATCATGGTAAAAAGCAGTGGGATTTGGATGTGGGATCCGGTTCCTTGGTGTCATCC

AGCCTTAGCAAACCAGAGGTATTTGGGAATAAGATGATCATTCCTTCCCTGGATGGAGCCCTCTTCCAGT

GGGACCAAGACCGTGAAAGCATGGAAACAGTTCCTTTCACAGTTGAATCACTTCTTGAATCTTCTTATAA

ATTTGGAGATGATGTTGTTTTGGTTGGAGGAAAATCTCTGACTACATATGGACTCAGTGCATATAGTGGA

AAGGTGAGGTATATCTGTTCAGCTCTGGGTTGTCGCCAATGGGATAGTGACGAAATGGAACAAGAGGAAG

ACATCCTGCTTCTACAGCGTACCCAAAAAACTGTTAGAGCTGTCGGACCTCGCAGTGGCAATGAGAAGTG

GAATTTCAGTGTTGGCCACTTTGAACTTCGGTATATTCCAGACATGGAAACGAGAGCCGGATTTATTGAA

AGCACCTTTAAGCCCAATGAGAACACAGAAGAGTCTAAAATTATTTCAGATGTGGAAGAACAGGAAGCTG

CCATAATGGACATAGTGATAAAGGTTTCGGTTGCTGACTGGAAAGTTATGGCATTCAGTAAGAAGGGAGG

ACATCTGGAATGGGAGTACCAGTTTTGTACTCCAATTGCATCTGCCTGGTTACTTAAGGATGGGAAAGTC

ATTCCCATCAGTCTTTTTGATGATACAAGTTATACATCTAATGATGATGTTTTAGAAGATGAAGAAGACA

TTGTAGAAGCTGCCAGAGGAGCCACAGAAAACAGTGTTTACTTGGGAATGTATAGAGGCCAGCTGTATCT

GCAGTCATCAGTCAGAATTTCAGAAAAGTTTCCTTCAAGTCCCAAGGCTTTGGAATCTGTCACTAATGAA

AACGCAATTATTCCTTTACCAACAATCAAATGGAAACCCTTAATTCATTCTCCTTCCAGAACTCCTGTCT

TGGTAGGATCTGATGAATTTGACAAATGTCTCAGTAATGATAAGTTTTCTCATGAAGAATATAGTAATGG

TGCACTTTCAATCTTGCAGTATCCATATGATAATGGTTATTATCTACCATACTACAAGAGGGAGAGGAAC

AAACGAAGCACACAGATTACAGTCAGATTCCTCGACAACCCACATTACAACAAGAATATCCGCAAAAAGG

ATCCTGTTCTTCTTTTACACTGGTGGAAAGAAATAGTTGCAACGATTTTGTTTTGTATCATAGCAACAAC

GTTTATTGTGCGCAGGCTTTTCCATCCTCATCCTCACAGGCAAAGGAAGGAGTCTGAAACTCAGTGTCAA

ACTGAAAATAAATATGATTCTGTAAGTGGTGAAGCCAATGACAGTAGCTGGAATGACATAAAAAACTCTG

GATATATATCACGATATCTAACTGATTTTGAGCCAATTCAATGCCTGGGACGTGGTGGCTTTGGAGTTGT

TTTTGAAGCTAAAAACAAAGTAGATGACTGCAATTATGCTATCAAGAGGATCCGTCTCCCCAATAGGGAA

TTGGCTCGGGAAAAGGTAATGCGAGAAGTTAAAGCCTTAGCCAAGCTTGAACACCCGGGCATTGTTAGAT

ATTTCAATGCCTGGCTCGAAGCACCACCAGAGAAGTGGCAAGAAAAGATGGATGAAATTTGGCTGAAAGA

TGAAAGCACAGACTGGCCACTCAGCTCTCCTAGCCCAATGGATGCACCATCAGTTAAAATACGCAGAATG

GATCCTTTCGCTACAAAAGAACATATTGAAATCATAGCTCCTTCACCACAAAGAAGCAGGTCTTTTTCAG

TAGGGATTTCCTGTGACCAGACAAGTTCATCTGAGAGCCAGTTCTCACCACTGGAATTCTCAGGAATGGA

CCATGAGGACATCAGTGAGTCAGTGGATGCAGCATACAACCTCCAGGACAGTTGCCTTACAGACTGTGAT

GTGGAAGATGGGACTATGGATGGCAATGATGAGGGGCACTCCTTTGAACTTTGTCCTTCTGAAGCTTCTC

CTTATGTAAGGTCAAGGGAGAGAACCTCCTCTTCAATAGTATTTGAAGATTCTGGCTGTGATAATGCTTC

CAGTAAAGAAGAGCCGAAAACTAATCGATTGCATATTGGCAACCATTGTGCTAATAAACTAACTGCTTTC

AAGCCCACCAGTAGCAAATCTTCTTCTGAAGCTACATTGTCTATTTCTCCTCCAAGACCAACCACTTTAA

GTTTAGATCTCACTAAAAACACCACAGAAAAACTCCAGCCCAGTTCACCAAAGGTGTATCTTTACATTCA

AATGCAGCTGTGCAGAAAAGAAAACCTCAAAGACTGGATGAATGGACGATGTACCATAGAGGAGAGAGAG

AGGAGCGTGTGTCTGCACATCTTCCTGCAGATCGCAGAGGCAGTGGAGTTTCTTCACAGTAAAGGACTGA

TGCACAGGGACCTCAAGCCATCCAACATATTCTTTACAATGGATGATGTGGTCAAGGTTGGAGACTTTGG

GTTAGTGACTGCAATGGACCAGGATGAGGAAGAGCAGACGGTTCTGACCCCAATGCCAGCTTATGCCAGA

CACACAGGACAAGTAGGGACCAAACTGTATATGAGCCCAGAGCAGATTCATGGAAACAGCTATTCTCATA

AAGTGGACATCTTTTCTTTAGGCCTGATTCTATTTGAATTGCTGTATCCATTCAGCACTCAGATGGAGAG

AGTCAGGACCTTAACTGATGTAAGAAATCTCAAATTTCCACCATTATTTACTCAGAAATATCCTTGTGAG

TACGTGATGGTTCAAGACATGCTCTCTCCATCCCCCATGGAACGACCTGAAGCTATAAACATCATTGAAA

ATGCTGTATTTGAGGACTTGGACTTTCCAGGAAAAACAGTGCTCAGACAGAGGTCTCGCTCCTTGAGTTC

ATCGGGAACAAAACATTCAAGACAGTCCAACAACTCCCATAGCCCTTTGCCAAGCAATTAG

>lcl|NM_004094.5_cds_NP_004085.1_1 [gene=EIF2S1] [db_xref=CCDS:CCDS9781.1] [protein=eukaryotic translation initiation factor 2 subunit 1] [protein_id=NP_004085.1] [location=131..1078] [gbkey=CDS]

ATGCCGGGTCTAAGTTGTAGATTTTATCAACACAAATTTCCTGAGGTGGAAGATGTAGTGATGGTGAATG

TCAGATCCATTGCTGAAATGGGGGCTTATGTCAGCTTGCTGGAATACAACAACATTGAAGGCATGATTCT

TCTTAGTGAATTATCCAGAAGGCGTATCCGTTCTATCAACAAACTCATCCGAATTGGCAGGAATGAGTGT

GTGGTTGTCATTAGGGTGGACAAAGAAAAAGGATATATTGATTTGTCAAAAAGAAGAGTTTCTCCAGAGG

AAGCAATCAAATGTGAAGACAAATTCACAAAATCCAAAACTGTTTATAGCATTCTTCGTCATGTTGCTGA

GGTGTTAGAATACACCAAGGATGAGCAGCTGGAAAGCCTATTCCAGAGGACTGCCTGGGTCTTTGATGAC

AAGTACAAGAGACCTGGATATGGTGCCTATGATGCATTTAAGCATGCAGTCTCAGACCCATCTATTTTGG

ATAGTTTAGATTTGAATGAAGATGAACGGGAAGTACTCATTAATAATATTAATAGGCGCTTGACCCCACA

GGCTGTCAAAATTCGAGCAGATATTGAAGTGGCTTGTTATGGTTATGAAGGCATTGATGCTGTAAAAGAA

GCCCTAAGAGCAGGTTTGAATTGTTCTACAGAAAACATGCCCATTAAGATTAATCTAATAGCTCCTCCTC

GGTATGTAATGACTACGACAACCCTGGAGAGAACAGAAGGCCTTTCTGTCCTCAGTCAAGCTATGGCTGT

TATCAAAGAGAAGATTGAGGAAAAGAGGGGTGTGTTCAATGTTCAAATGGAGCCCAAAGTGGTCACAGAT

ACAGATGAGACTGAACTTGCGAGGCAGATGGAGAGGCTTGAAAGAGAAAATGCCGAAGTGGATGGAGATG

ATGATGCAGAAGAAATGGAAGCCAAAGCTGAAGATTAA

>lcl|NM_004095.4_cds_NP_004086.1_1 [gene=EIF4EBP1] [db_xref=CCDS:CCDS6100.1] [protein=eukaryotic translation initiation factor 4E-binding protein 1] [protein_id=NP_004086.1] [location=41..397] [gbkey=CDS]

ATGTCCGGGGGCAGCAGCTGCAGCCAGACCCCAAGCCGGGCCATCCCCGCCACTCGCCGGGTGGTGCTCG

GCGACGGCGTGCAGCTCCCGCCCGGGGACTACAGCACGACCCCCGGCGGCACGCTCTTCAGCACCACCCC

GGGAGGTACCAGGATCATCTATGACCGGAAATTCCTGATGGAGTGTCGGAACTCACCTGTGACCAAAACA

CCCCCAAGGGATCTGCCCACCATTCCGGGGGTCACCAGCCCTTCCAGTGATGAGCCCCCCATGGAAGCCA

GCCAGAGCCACCTGCGCAATAGCCCAGAAGATAAGCGGGCGGGCGGTGAAGAGTCACAGTTTGAGATGGA

CATTTAA

>lcl|NM_182917.4_cds_NP_886553.3_1 [gene=EIF4G1] [db_xref=CCDS:CCDS54688.1] [protein=eukaryotic translation initiation factor 4 gamma 1 isoform 1] [protein_id=NP_886553.3] [location=417..5219] [gbkey=CDS]

ATGAACAAAGCTCCACAGTCCACAGGCCCCCCACCCGCCCCATCCCCCGGACTCCCACAGCCAGCGTTTC

CCCCGGGGCAGACAGCGCCGGTGGTGTTCAGTACGCCACAAGCGACACAAATGAACACGCCTTCTCAGCC

CCGCCAGCACTTCTACCCTAGCCGGGCCCAGCCCCCGAGCAGTGCAGCCTCCCGAGTGCAGAGTGCAGCC

CCTGCCCGCCCTGGCCCAGCTGCCCATGTCTACCCTGCTGGATCCCAAGTAATGATGATCCCTTCCCAGA

TCTCCTACCCAGCCTCCCAGGGGGCCTACTACATCCCTGGACAGGGGCGTTCCACATACGTTGTCCCGAC

ACAGCAGTACCCTGTGCAGCCAGGAGCCCCAGGCTTCTATCCAGGTGCAAGCCCTACAGAATTTGGGACC

TACGCTGGCGCCTACTATCCAGCCCAAGGGGTGCAGCAGTTTCCCACTGGCGTGGCCCCCGCCCCAGTTT

TGATGAACCAGCCACCCCAGATTGCTCCCAAGAGGGAGCGTAAGACGATCCGAATTCGAGATCCAAACCA

AGGAGGAAAGGATATCACAGAGGAGATCATGTCTGGGGCCCGCACTGCCTCCACACCCACCCCTCCCCAG

ACGGGAGGCGGTCTGGAGCCTCAAGCTAATGGGGAGACGCCCCAGGTTGCTGTCATTGTCCGGCCAGATG

ACCGGTCACAGGGAGCAATCATTGCTGACCGGCCAGGGCTGCCTGGCCCAGAGCATAGCCCTTCAGAATC

CCAGCCTTCGTCGCCTTCTCCGACCCCATCACCATCCCCAGTCTTGGAACCGGGGTCTGAGCCTAATCTC

GCAGTCCTCTCTATTCCTGGGGACACTATGACAACTATACAAATGTCTGTAGAAGAATCAACCCCCATCT

CCCGTGAAACTGGGGAGCCATATCGCCTCTCTCCAGAACCCACTCCTCTCGCCGAACCCATACTGGAAGT

AGAAGTGACACTTAGCAAACCGGTTCCAGAATCTGAGTTTTCTTCCAGTCCTCTCCAGGCTCCCACCCCT

TTGGCATCTCACACAGTGGAAATTCATGAGCCTAATGGCATGGTCCCATCTGAAGATCTGGAACCAGAGG

TGGAGTCAAGCCCAGAGCTTGCTCCTCCCCCAGCTTGCCCCTCCGAATCCCCTGTGCCCATTGCTCCAAC

TGCCCAACCTGAGGAACTGCTCAACGGAGCCCCCTCGCCACCAGCTGTGGACTTAAGCCCAGTCAGTGAG

CCAGAGGAGCAGGCCAAGGAGGTGACAGCATCAATGGCGCCCCCCACCATCCCCTCTGCTACTCCAGCTA

CGGCTCCTTCAGCTACTTCCCCAGCTCAGGAGGAGGAAATGGAAGAAGAAGAAGAAGAGGAAGAAGGAGA

AGCAGGAGAAGCAGGAGAAGCTGAGAGTGAGAAAGGAGGAGAGGAACTGCTCCCCCCAGAGAGTACCCCT

ATTCCAGCCAACTTGTCTCAGAATTTGGAGGCAGCAGCAGCCACTCAAGTGGCAGTATCTGTGCCAAAGA

GGAGACGGAAAATTAAGGAGCTAAATAAGAAGGAGGCTGTTGGAGACCTTCTGGATGCCTTCAAGGAGGC

GAACCCGGCAGTACCAGAGGTGGAAAATCAGCCTCCTGCAGGCAGCAATCCAGGCCCAGAGTCTGAGGGC

AGTGGTGTGCCCCCACGTCCTGAGGAAGCAGATGAGACCTGGGACTCAAAGGAAGACAAAATTCACAATG

CTGAGAACATCCAGCCCGGGGAACAGAAGTATGAATATAAGTCAGATCAGTGGAAGCCTCTAAACCTAGA

GGAGAAAAAACGTTACGACCGTGAGTTCCTGCTTGGTTTTCAGTTCATCTTTGCCAGTATGCAGAAGCCA

GAGGGATTGCCACATATCAGTGACGTGGTGCTGGACAAGGCCAATAAAACACCACTGCGGCCACTGGATC

CCACTAGACTACAAGGCATAAATTGTGGCCCAGACTTCACTCCATCCTTTGCCAACCTTGGCCGGACAAC

CCTTAGCACCCGTGGGCCCCCAAGGGGTGGGCCAGGTGGGGAGCTGCCCCGTGGGCCGCAGGCTGGCCTG

GGACCCCGGCGCTCTCAGCAGGGACCCCGAAAAGAACCACGCAAGATCATTGCCACAGTGTTAATGACCG

AAGATATAAAACTGAACAAAGCAGAGAAAGCCTGGAAACCCAGCAGCAAGCGGACGGCGGCTGATAAGGA

TCGAGGGGAAGAAGATGCTGATGGCAGCAAAACCCAGGACCTATTCCGCAGGGTGCGCTCCATCCTGAAT

AAACTGACACCCCAGATGTTCCAGCAGCTGATGAAGCAAGTGACGCAGCTGGCCATCGACACCGAGGAAC

GCCTCAAAGGGGTCATTGACCTCATTTTTGAGAAGGCCATTTCAGAGCCCAACTTCTCTGTGGCCTATGC

CAACATGTGCCGCTGCCTCATGGCGCTGAAAGTGCCCACTACGGAAAAGCCAACAGTGACTGTGAACTTC

CGAAAGCTGTTGTTGAATCGATGTCAGAAGGAGTTTGAGAAAGACAAAGATGATGATGAGGTTTTTGAGA

AGAAGCAAAAAGAGATGGATGAAGCTGCTACGGCAGAGGAACGAGGACGCCTGAAGGAAGAGCTGGAAGA

GGCTCGGGACATAGCCCGGCGGCGCTCTTTAGGGAATATCAAGTTTATTGGAGAGTTGTTCAAACTGAAG

ATGTTAACAGAGGCAATAATGCATGACTGTGTGGTCAAACTGCTTAAGAACCATGATGAAGAGTCCCTTG

AGTGCCTTTGTCGTCTGCTCACCACCATTGGCAAAGACCTGGACTTTGAAAAAGCCAAGCCCCGAATGGA

TCAGTATTTCAACCAGATGGAAAAAATCATTAAAGAAAAGAAGACGTCATCCCGCATCCGCTTTATGCTG

CAGGACGTGCTGGATCTGCGAGGGAGCAATTGGGTGCCACGCCGAGGGGATCAGGGTCCCAAGACCATTG

ACCAGATCCATAAGGAGGCTGAGATGGAAGAACATCGAGAGCACATCAAAGTGCAGCAGCTCATGGCCAA

GGGCAGTGACAAGCGTCGGGGCGGTCCTCCAGGCCCTCCCATCAGCCGTGGACTTCCCCTTGTGGATGAT

GGTGGCTGGAACACAGTTCCCATCAGCAAAGGTAGCCGCCCCATTGACACCTCACGACTCACCAAGATCA

CCAAGCCTGGCTCCATCGATTCTAACAACCAGCTCTTTGCACCTGGAGGGCGACTGAGCTGGGGCAAGGG

CAGCAGCGGAGGCTCAGGAGCCAAGCCCTCAGACGCAGCATCAGAAGCTGCTCGCCCAGCTACTAGTACT

TTGAATCGCTTCTCAGCCCTTCAACAAGCGGTACCCACAGAAAGCACAGATAATAGACGTGTGGTGCAGA

GGAGTAGCTTGAGCCGAGAACGAGGCGAGAAAGCTGGAGACCGAGGAGACCGCCTAGAGCGGAGTGAACG

GGGAGGGGACCGTGGGGACCGGCTTGATCGTGCGCGGACACCTGCTACCAAGCGGAGCTTCAGCAAGGAA

GTGGAGGAGCGGAGTAGAGAACGGCCCTCCCAGCCTGAGGGGCTGCGCAAGGCAGCTAGCCTCACGGAGG

ATCGGGACCGTGGGCGGGATGCCGTGAAGCGAGAAGCTGCCCTACCCCCAGTGAGCCCCCTGAAGGCGGC

TCTCTCTGAGGAGGAGTTAGAGAAGAAATCCAAGGCTATCATTGAGGAATATCTCCATCTCAATGACATG

AAAGAGGCAGTCCAGTGCGTGCAGGAGCTGGCCTCACCCTCCTTGCTCTTCATCTTTGTACGGCATGGTG

TCGAGTCTACGCTGGAGCGCAGTGCCATTGCTCGTGAGCATATGGGGCAGCTGCTGCACCAGCTGCTCTG

TGCTGGGCATCTGTCTACTGCTCAGTACTACCAAGGGTTGTATGAAATCTTGGAATTGGCTGAGGACATG

GAAATTGACATCCCCCACGTGTGGCTCTACCTAGCGGAACTGGTAACACCCATTCTGCAGGAAGGTGGGG

TGCCCATGGGGGAGCTGTTCAGGGAGATTACAAAGCCTCTGAGACCGTTGGGCAAAGCTGCTTCCCTGTT

GCTGGAGATCCTGGGCCTCCTGTGCAAAAGCATGGGTCCTAAAAAGGTGGGGACGCTGTGGCGAGAAGCC

GGGCTTAGCTGGAAGGAATTTCTACCTGAAGGCCAGGACATTGGTGCATTCGTCGCTGAACAGAAGGTGG

AGTATACCCTGGGAGAGGAGTCGGAAGCCCCTGGCCAGAGGGCACTCCCCTCCGAGGAGCTGAACAGGCA

GCTGGAGAAGCTGCTGAAGGAGGGCAGCAGTAACCAGCGGGTGTTCGACTGGATAGAGGCCAACCTGAGT

GAGCAGCAGATAGTATCCAACACGTTAGTTCGAGCCCTCATGACGGCTGTCTGCTATTCTGCAATTATTT

TTGAGACTCCCCTCCGAGTGGACGTTGCAGTGCTGAAAGCGCGAGCGAAGCTGCTGCAGAAATACCTGTG

TGACGAGCAGAAGGAGCTACAGGCGCTCTACGCCCTCCAGGCCCTTGTAGTGACCTTAGAACAGCCTCCC

AACCTGCTGCGGATGTTCTTTGACGCACTGTATGACGAGGACGTGGTGAAGGAGGATGCCTTCTACAGTT

GGGAGAGTAGCAAGGACCCCGCTGAGCAGCAGGGCAAGGGTGTGGCCCTTAAATCTGTCACAGCCTTCTT

CAAGTGGCTCCGTGAAGCAGAGGAGGAGTCTGACCACAACTGA

>lcl|NM_004448.4_cds_NP_004439.2_1 [gene=ERBB2] [db_xref=CCDS:CCDS32642.1] [protein=receptor tyrosine-protein kinase erbB-2 isoform a precursor] [protein_id=NP_004439.2] [location=176..3943] [gbkey=CDS]

ATGGAGCTGGCGGCCTTGTGCCGCTGGGGGCTCCTCCTCGCCCTCTTGCCCCCCGGAGCCGCGAGCACCC
AAGTGTGCACCGGCACAGACATGAAGCTGCGGCTCCCTGCCAGTCCCGAGACCCACCTGGACATGCTCCG
CCACCTCTACCAGGGCTGCCAGGTGGTGCAGGGAAACCTGGAACTCACCTACCTGCCCACCAATGCCAGC
CTGTCCTTCCTGCAGGATATCCAGGAGGTGCAGGGCTACGTGCTCATCGCTCACAACCAAGTGAGGCAGG
TCCCACTGCAGAGGCTGCGGATTGTGCGAGGCACCCAGCTCTTTGAGGACAACTATGCCCTGGCCGTGCT
AGACAATGGAGACCCGCTGAACAATACCACCCCTGTCACAGGGGCCTCCCCAGGAGGCCTGCGGGAGCTG
CAGCTTCGAAGCCTCACAGAGATCTTGAAAGGAGGGGTCTTGATCCAGCGGAACCCCCAGCTCTGCTACC
AGGACACGATTTTGTGGAAGGACATCTTCCACAAGAACAACCAGCTGGCTCTCACACTGATAGACACCAA
CCGCTCTCGGGCCTGCCACCCCTGTTCTCCGATGTGTAAGGGCTCCCGCTGCTGGGGAGAGAGTTCTGAG
GATTGTCAGAGCCTGACGCGCACTGTCTGTGCCGGTGGCTGTGCCCGCTGCAAGGGGCCACTGCCCACTG
ACTGCTGCCATGAGCAGTGTGCTGCCGGCTGCACGGGCCCCAAGCACTCTGACTGCCTGGCCTGCCTCCA
CTTCAACCACAGTGGCATCTGTGAGCTGCACTGCCCAGCCCTGGTCACCTACAACACAGACACGTTTGAG
TCCATGCCCAATCCCGAGGGCCGGTATACATTCGGCGCCAGCTGTGTGACTGCCTGTCCCTACAACTACC
TTTCTACGGACGTGGGATCCTGCACCCTCGTCTGCCCCCTGCACAACCAAGAGGTGACAGCAGAGGATGG
AACACAGCGGTGTGAGAAGTGCAGCAAGCCCTGTGCCCGAGTGTGCTATGGTCTGGGCATGGAGCACTTG
CGAGAGGTGAGGGCAGTTACCAGTGCCAATATCCAGGAGTTTGCTGGCTGCAAGAAGATCTTTGGGAGCC
TGGCATTTCTGCCGGAGAGCTTTGATGGGGACCCAGCCTCCAACACTGCCCCGCTCCAGCCAGAGCAGCT
CCAAGTGTTTGAGACTCTGGAAGAGATCACAGGTTACCTATACATCTCAGCATGGCCGGACAGCCTGCCT
GACCTCAGCGTCTTCCAGAACCTGCAAGTAATCCGGGGACGAATTCTGCACAATGGCGCCTACTCGCTGA
CCCTGCAAGGGCTGGGCATCAGCTGGCTGGGGCTGCGCTCACTGAGGGAACTGGGCAGTGGACTGGCCCT
CATCCACCATAACACCCACCTCTGCTTCGTGCACACGGTGCCCTGGGACCAGCTCTTTCGGAACCCGCAC
CAAGCTCTGCTCCACACTGCCAACCGGCCAGAGGACGAGTGTGTGGGCGAGGGCCTGGCCTGCCACCAGC
TGTGCGCCCGAGGGCACTGCTGGGGTCCAGGGCCCACCCAGTGTGTCAACTGCAGCCAGTTCCTTCGGGG
CCAGGAGTGCGTGGAGGAATGCCGAGTACTGCAGGGGCTCCCCAGGGAGTATGTGAATGCCAGGCACTGT
TTGCCGTGCCACCCTGAGTGTCAGCCCCAGAATGGCTCAGTGACCTGTTTTGGACCGGAGGCTGACCAGT

GTGTGGCCTGTGCCCACTATAAGGACCCTCCCTTCTGCGTGGCCCGCTGCCCCAGCGGTGTGAAACCTGA

CCTCTCCTACATGCCCATCTGGAAGTTTCCAGATGAGGAGGGCGCATGCCAGCCTTGCCCCATCAACTGC

ACCCACTCCTGTGTGGACCTGGATGACAAGGGCTGCCCCGCCGAGCAGAGAGCCAGCCCTCTGACGTCCA

TCATCTCTGCGGTGGTTGGCATTCTGCTGGTCGTGGTCTTGGGGGTGGTCTTTGGGATCCTCATCAAGCG

ACGGCAGCAGAAGATCCGGAAGTACACGATGCGGAGACTGCTGCAGGAAACGGAGCTGGTGGAGCCGCTG

ACACCTAGCGGAGCGATGCCCAACCAGGCGCAGATGCGGATCCTGAAAGAGACGGAGCTGAGGAAGGTGA

AGGTGCTTGGATCTGGCGCTTTTGGCACAGTCTACAAGGGCATCTGGATCCCTGATGGGGAGAATGTGAA

AATTCCAGTGGCCATCAAAGTGTTGAGGGAAAACACATCCCCCAAAGCCAACAAAGAAATCTTAGACGAA

GCATACGTGATGGCTGGTGTGGGCTCCCCATATGTCTCCCGCCTTCTGGGCATCTGCCTGACATCCACGG

TGCAGCTGGTGACACAGCTTATGCCCTATGGCTGCCTCTTAGACCATGTCCGGGAAAACCGCGGACGCCT

GGGCTCCCAGGACCTGCTGAACTGGTGTATGCAGATTGCCAAGGGGATGAGCTACCTGGAGGATGTGCGG

CTCGTACACAGGGACTTGGCCGCTCGGAACGTGCTGGTCAAGAGTCCCAACCATGTCAAAATTACAGACT

TCGGGCTGGCTCGGCTGCTGGACATTGACGAGACAGAGTACCATGCAGATGGGGGCAAGGTGCCCATCAA

GTGGATGGCGCTGGAGTCCATTCTCCGCCGGCGGTTCACCCACCAGAGTGATGTGTGGAGTTATGGTGTG

ACTGTGTGGGAGCTGATGACTTTTGGGGCCAAACCTTACGATGGGATCCCAGCCCGGGAGATCCCTGACC

TGCTGGAAAAGGGGGAGCGGCTGCCCCAGCCCCCCATCTGCACCATTGATGTCTACATGATCATGGTCAA

ATGTTGGATGATTGACTCTGAATGTCGGCCAAGATTCCGGGAGTTGGTGTCTGAATTCTCCCGCATGGCC

AGGGACCCCCAGCGCTTTGTGGTCATCCAGAATGAGGACTTGGGCCCAGCCAGTCCCTTGGACAGCACCT

TCTACCGCTCACTGCTGGAGGACGATGACATGGGGGACCTGGTGGATGCTGAGGAGTATCTGGTACCCCA

GCAGGGCTTCTTCTGTCCAGACCCTGCCCCGGGCGCTGGGGGCATGGTCCACCACAGGCACCGCAGCTCA

TCTACCAGGAGTGGCGGTGGGGACCTGACACTAGGGCTGGAGCCCTCTGAAGAGGAGGCCCCCAGGTCTC

CACTGGCACCCTCCGAAGGGGCTGGCTCCGATGTATTTGATGGTGACCTGGGAATGGGGGCAGCCAAGGG

GCTGCAAAGCCTCCCCACACATGACCCCAGCCCTCTACAGCGGTACAGTGAGGACCCCACAGTACCCCTG

CCCTCTGAGACTGATGGCTACGTTGCCCCCCTGACCTGCAGCCCCCAGCCTGAATATGTGAACCAGCCAG

ATGTTCGGCCCCAGCCCCCTTCGCCCCGAGAGGGCCCTCTGCCTGCTGCCCGACCTGCTGGTGCCACTCT

GGAAAGGCCCAAGACTCTCTCCCCAGGGAAGAATGGGGTCGTCAAAGACGTTTTTGCCTTTGGGGGTGCC

GTGGAGAACCCCGAGTACTTGACACCCCAGGGAGGAGCTGCCCCTCAGCCCCACCCTCCTCCTGCCTTCA

GCCCAGCCTTCGACAACCTCTATTACTGGGACCAGGACCCACCAGAGCGGGGGGCTCCACCCAGCACCTT

CAAAGGGACACCTACGGCAGAGAACCCAGAGTACCTGGGTCTGGACGTGCCAGTGTGA

>lcl|NM_001433.5_cds_NP_001424.3_1 [gene=ERN1] [db_xref=CCDS:CCDS45762.1] [protein=serine/threonine-protein kinase/endoribonuclease IRE1 precursor] [protein_id=NP_001424.3] [location=116..3049] [gbkey=CDS]

ATGCCGGCCCGGCGGCTGCTGCTGCTGCTGACGCTGCTGCTGCCCGGCCTCGGGATTTTTGGAAGTACCA

GCACAGTGACGCTTCCTGAAACCTTGTTGTTTGTGTCAACGCTGGATGGAAGTTTGCATGCTGTCAGCAA

GAGGACAGGCTCAATCAAATGGACTTTAAAAGAAGATCCAGTCCTGCAGGTCCCAACACATGTGGAAGAG

CCTGCCTTTCTCCCAGATCCTAATGATGGCAGCCTGTATACGCTTGGAAGCAAGAATAATGAAGGCCTGA

CGAAACTTCCTTTTACCATCCCAGAATTGGTGCAGGCATCCCCATGCCGAAGTTCAGATGGAATCCTCTA

CATGGGTAAAAAGCAGGACATCTGGTATGTTATTGACCTCCTGACCGGAGAGAAGCAGCAGACTTTGTCA

TCGGCCTTTGCAGATAGTCTCTGCCCATCAACCTCTCTTCTGTATCTTGGGCGAACAGAATACACCATCA

CCATGTACGACACCAAAACCCGAGAGCTCCGGTGGAATGCCACCTACTTTGACTATGCGGCCTCACTGCC

TGAGGACGACGTGGACTACAAGATGTCCCACTTTGTGTCCAATGGTGATGGGCTGGTGGTGACTGTGGAC

AGTGAATCTGGGGACGTCCTGTGGATCCAAAACTACGCCTCCCCTGTGGTGGCCTTTTATGTCTGGCAGC

GGGAGGGTCTGAGGAAGGTGATGCACATCAATGTCGCTGTGGAGACCCTGCGCTATCTGACCTTCATGTC

TGGGGAGGTGGGGCGCATCACAAAGTGGAAGTACCCGTTCCCCAAGGAGACAGAGGCCAAGAGCAAGCTG

ACGCCCACTCTGTATGTTGGGAAATACTCTACCAGCCTCTATGCCTCTCCCTCAATGGTACACGAGGGGG

TTGCTGTCGTGCCCCGCGGCAGCACACTTCCTTTGCTGGAAGGGCCCCAGACTGATGGCGTCACCATTGG

GGACAAGGGGGAGTGTGTGATCACGCCCAGCACGGACGTCAAGTTTGATCCCGGACTCAAAAGCAAGAAC

AAGCTCAACTACTTGAGGAATTACTGGCTTCTGATAGGACACCATGAAACCCCACTGTCTGCGTCTACCA

AGATGCTGGAGAGATTTCCCAACAATCTACCCAAACATCGGGAAAATGTGATTCCTGCTGATTCAGAGAA

AAAGAGCTTTGAGGAAGTTATCAACCTGGTTGACCAGACTTCAGAAAACGCACCTACCACCGTGTCTCGG

GATGTGGAGGAGAAGCCCGCCCATGCCCCTGCCCGGCCCGAGGCCCCCGTGGACTCCATGCTTAAGGACA

TGGCTACCATCATCCTGAGCACCTTCCTGCTGATTGGCTGGGTGGCCTTCATCATCACCTATCCCCTGAG

CATGCATCAGCAGCAGCAGCTCCAGCACCAGCAGTTCCAGAAGGAACTGGAGAAGATCCAGCTCCTGCAG

CAGCAGCAGCAGCAGCTGCCCTTCCACCCACCTGGAGACACGGCTCAGGACGGCGAGCTCCTGGACACGT

CTGGCCCGTACTCAGAGAGCTCGGGCACCAGCAGCCCCAGCACGTCCCCCAGGGCCTCCAACCACTCGCT

CTGCTCCGGCAGCTCTGCCTCCAAGGCTGGCAGCAGCCCCTCCCTGGAACAAGACGATGGAGATGAGGAA

ACCAGCGTGGTGATAGTTGGGAAAATTTCCTTCTGTCCCAAGGATGTCCTGGGCCATGGAGCTGAGGGCA

CAATTGTGTACCGGGGCATGTTTGACAACCGCGACGTGGCCGTGAAGAGGATCCTCCCCGAGTGTTTTAG

CTTCGCAGACCGTGAGGTCCAGCTGTTGCGAGAATCGGATGAGCACCCGAACGTGATCCGCTACTTCTGC

ACGGAGAAGGACCGGCAATTCCAGTACATTGCCATCGAGCTGTGTGCAGCCACCCTGCAAGAGTATGTGG

AGCAGAAGGACTTTGCGCATCTCGGCCTGGAGCCCATCACCTTGCTGCAGCAGACCACCTCGGGCCTGGC

CCACCTCCACTCCCTCAACATCGTTCACAGAGACCTAAAGCCACACAACATCCTCATATCCATGCCCAAT

GCACACGGCAAGATCAAGGCCATGATCTCCGACTTTGGCCTCTGCAAGAAGCTGGCAGTGGGCAGACACA

GTTTCAGCCGCCGATCTGGGGTGCCTGGCACAGAAGGCTGGATCGCTCCAGAGATGCTGAGCGAAGACTG

TAAGGAGAACCCTACCTACACGGTGGACATCTTTTCTGCAGGCTGCGTCTTTTACTACGTAATCTCTGAG

GGCAGCCACCCTTTTGGCAAGTCCCTGCAGCGGCAGGCCAACATCCTCCTGGGTGCCTGCAGCCTTGACT

GCTTGCACCCAGAGAAGCACGAAGACGTCATTGCACGTGAATTGATAGAGAAGATGATTGCGATGGATCC

TCAGAAACGCCCCTCAGCGAAGCATGTGCTCAAACACCCGTTCTTCTGGAGCCTAGAGAAGCAGCTCCAG

TTCTTCCAGGACGTGAGCGACAGAATAGAAAAGGAATCCCTGGATGGCCCGATCGTGAAGCAGTTAGAGA

GAGGCGGGAGAGCCGTGGTGAAGATGGACTGGCGGGAGAACATCACTGTCCCCCTCCAGACAGACCTGCG

TAAATTCAGGACCTATAAAGGTGGTTCTGTCAGAGATCTCCTCCGAGCCATGAGAAATAAGAAGCACCAC

TACCGGGAGCTGCCTGCAGAGGTGCGGGAGACGCTGGGGTCCCTCCCCGACGACTTCGTGTGCTACTTCA

CATCTCGCTTCCCCCACCTCCTCGCACACACCTACCGGGCCATGGAGCTGTGCAGCCACGAGAGACTCTT

CCAGCCCTACTACTTCCACGAGCCCCCAGAGCCCCAGCCCCCAGTGACTCCAGACGCCCTCTGA

>lcl|NM_014584.3_cds_NP_055399.1_1 [gene=ERO1A] [db_xref=CCDS:CCDS9709.1] [protein=ERO1-like protein alpha isoform 2 precursor] [protein_id=NP_055399.1] [location=78..1484] [gbkey=CDS]

ATGGGCCGCGGCTGGGGATTCTTGTTTGGCCTCCTGGGCGCCGTGTGGCTGCTCAGCTCGGGCCACGGAG

AGGAGCAGCCCCCGGAGACAGCGGCACAGAGGTGCTTCTGCCAGGTTAGTGGTTACTTGGATGATTGTAC

CTGTGATGTTGAAACCATTGATAGATTTAATAACTACAGGCTTTTCCCAAGACTACAAAAACTTCTTGAA

AGTGACTACTTTAGGTATTACAAGGTAAACCTGAAGAGGCCGTGTCCTTTCTGGAATGACATCAGCCAGT

GTGGAAGAAGGGACTGTGCTGTCAAACCATGTCAATCTGATGAAGTTCCTGATGGAATTAAATCTGCGAG

CTACAAGTATTCTGAAGAAGCCAATAATCTCATTGAAGAATGTGAACAAGCTGAACGACTTGGAGCAGTG

GATGAATCTCTGAGTGAGGAAACACAGAAGGCTGTTCTTCAGTGGACCAAGCATGATGATTCTTCAGATA

ACTTCTGTGAAGCTGATGACATTCAGTCCCCTGAAGCTGAATATGTAGATTTGCTTCTTAATCCTGAGCG

CTACACTGGTTACAAGGGACCAGATGCTTGGAAAATATGGAATGTCATCTACGAAGAAAACTGTTTTAAG

CCACAGACAATTAAAAGACCTTTAAATCCTTTGGCTTCTGGTCAAGGGACAAGTGAAGAGAACACTTTTT

ACAGTTGGCTAGAAGGTCTCTGTGTAGAAAAAAGAGCATTCTACAGACTTATATCTGGCCTACATGCAAG

CATTAATGTGCATTTGAGTGCAAGATATCTTTTACAAGAGACCTGGTTAGAAAAGAAATGGGGACACAAC

ATTACAGAATTTCAACAGCGATTTGATGGAATTTTGACTGAAGGAGAAGGTCCAAGAAGGCTTAAGAACT

TGTATTTTCTCTACTTAATAGAACTAAGGGCTTTATCCAAAGTGTTACCATTCTTCGAGCGCCCAGATTT

TCAACTCTTTACTGGAAATAAAATTCAGGATGAGGAAAACAAAATGTTACTTCTGGAAATACTTCATGAA

ATCAAGTCATTTCCTTTGCATTTTGATGAGAATTCATTTTTTGCTGGGGATAAAAAAGAAGCACACAAAC

TAAAGGAGGACTTTCGACTGCATTTTAGAAATATTTCAAGAATTATGGATTGTGTTGGTTGTTTTAAATG

TCGTCTGTGGGGAAAGCTTCAGACTCAGGGTTTGGGCACTGCTCTGAAGATCTTATTTTCTGAGAAATTG

ATAGCAAATATGCCAGAAAGTGGACCTAGTTATGAATTCCATCTAACCAGACAAGAAATAGTATCATTAT

TCAACGCATTTGGAAGAATTTCTACAAGTGTGAAAGAATTAGAAAACTTCAGGAACTTGTTACAGAATAT

TCATTAA

>lcl|NM_003824.4_cds_NP_003815.1_1 [gene=FADD] [db_xref=CCDS:CCDS8196.1] [protein=FAS-associated death domain protein] [protein_id=NP_003815.1] [location=165..791] [gbkey=CDS]

ATGGACCCGTTCCTGGTGCTGCTGCACTCGGTGTCGTCCAGCCTGTCGAGCAGCGAGCTGACCGAGCTCA

AGTTCCTATGCCTCGGGCGCGTGGGCAAGCGCAAGCTGGAGCGCGTGCAGAGCGGCCTAGACCTCTTCTC

CATGCTGCTGGAGCAGAACGACCTGGAGCCCGGGCACACCGAGCTCCTGCGCGAGCTGCTCGCCTCCCTG

CGGCGCCACGACCTGCTGCGGCGCGTCGACGACTTCGAGGCGGGGGCGGCGGCCGGGGCCGCGCCTGGGG

AAGAAGACCTGTGTGCAGCATTTAACGTCATATGTGATAATGTGGGGAAAGATTGGAGAAGGCTGGCTCG

TCAGCTCAAAGTCTCAGACACCAAGATCGACAGCATCGAGGACAGATACCCCCGCAACCTGACAGAGCGT

GTGCGGGAGTCACTGAGAATCTGGAAGAACACAGAGAAGGAGAACGCAACAGTGGCCCACCTGGTGGGGG

CTCTCAGGTCCTGCCAGATGAACCTGGTGGCTGACCTGGTACAAGAGGTTCAGCAGGCCCGTGACCTCCA

GAACAGGAGTGGGGCCATGTCCCCGATGTCATGGAACTCAGACGCATCTACCTCCGAAGCGTCCTGA

>lcl|NM_000043.6_cds_NP_000034.1_1 [gene=FAS] [db_xref=CCDS:CCDS7393.1] [protein=tumor necrosis factor receptor superfamily member 6 isoform 1 precursor] [protein_id=NP_000034.1] [location=80..1087] [gbkey=CDS]

ATGCTGGGCATCTGGACCCTCCTACCTCTGGTTCTTACGTCTGTTGCTAGATTATCGTCCAAAAGTGTTA

ATGCCCAAGTGACTGACATCAACTCCAAGGGATTGGAATTGAGGAAGACTGTTACTACAGTTGAGACTCA

GAACTTGGAAGGCCTGCATCATGATGGCCAATTCTGCCATAAGCCCTGTCCTCCAGGTGAAAGGAAAGCT

AGGGACTGCACAGTCAATGGGGATGAACCAGACTGCGTGCCCTGCCAAGAAGGGAAGGAGTACACAGACA

AAGCCCATTTTTCTTCCAAATGCAGAAGATGTAGATTGTGTGATGAAGGACATGGCTTAGAAGTGGAAAT

AAACTGCACCCGGACCCAGAATACCAAGTGCAGATGTAAACCAAACTTTTTTTGTAACTCTACTGTATGT

GAACACTGTGACCCTTGCACCAAATGTGAACATGGAATCATCAAGGAATGCACACTCACCAGCAACACCA

AGTGCAAAGAGGAAGGATCCAGATCTAACTTGGGGTGGCTTTGTCTTCTTCTTTTGCCAATTCCACTAAT

TGTTTGGGTGAAGAGAAAGGAAGTACAGAAAACATGCAGAAAGCACAGAAAGGAAAACCAAGGTTCTCAT

GAATCTCCAACTTTAAATCCTGAAACAGTGGCAATAAATTTATCTGATGTTGACTTGAGTAAATATATCA

CCACTATTGCTGGAGTCATGACACTAAGTCAAGTTAAAGGCTTTGTTCGAAAGAATGGTGTCAATGAAGC

CAAAATAGATGAGATCAAGAATGACAATGTCCAAGACACAGCAGAACAGAAAGTTCAACTGCTTCGTAAT

TGGCATCAACTTCATGGAAAGAAAGAAGCGTATGACACATTGATTAAAGATCTCAAAAAAGCCAATCTTT

GTACTCTTGCAGAGAAAATTCAGACTATCATCCTCAAGGACATTACTAGTGACTCAGAAAATTCAAACTT

CAGAAATGAAATCCAAAGCTTGGTCTAG

>lcl|NM_000801.5_cds_NP_000792.1_1 [gene=FKBP1A] [db_xref=CCDS:CCDS13014.1] [protein=peptidyl-prolyl cis-trans isomerase FKBP1A isoform a] [protein_id=NP_000792.1] [location=57..383] [gbkey=CDS]

ATGGGAGTGCAGGTGGAAACCATCTCCCCAGGAGACGGGCGCACCTTCCCCAAGCGCGGCCAGACCTGCG

TGGTGCACTACACCGGGATGCTTGAAGATGGAAAGAAATTTGATTCCTCCCGGGACAGAAACAAGCCCTT

TAAGTTTATGCTAGGCAAGCAGGAGGTGATCCGAGGCTGGGAAGAAGGGGTTGCCCAGATGAGTGTGGGT

CAGAGAGCCAAACTGACTATATCTCCAGATTATGCCTATGGTGCCACTGGGCACCCAGGCATCATCCCAC

CACATGCCACTCTCGTCTTCGATGTGGAGCTTCTAAAACTGGAATGA

>lcl|NM_004116.5_cds_NP_004107.1_1 [gene=FKBP1B] [db_xref=CCDS:CCDS1706.1] [protein=peptidyl-prolyl cis-trans isomerase FKBP1B isoform a] [protein_id=NP_004107.1] [location=110..436] [gbkey=CDS]

ATGGGCGTGGAGATCGAGACCATCTCCCCCGGAGACGGAAGGACATTCCCCAAGAAGGGCCAAACGTGTG

TGGTGCACTACACAGGAATGCTCCAAAATGGGAAGAAGTTTGATTCATCCAGAGACAGAAACAAACCTTT

CAAGTTCAGAATTGGCAAACAGGAAGTCATCAAAGGTTTTGAAGAGGGTGCAGCCCAGATGAGCTTGGGG

CAGAGGGCGAAGCTGACCTGCACCCCTGATGTGGCATATGGAGCCACGGGCCACCCCGGTGTCATCCCTC

CCAATGCCACCCTCATCTTTGACGTGGAGCTGCTCAACTTAGAGTGA

>lcl|NM_005252.4_cds_NP_005243.1_1 [gene=FOS] [db_xref=CCDS:CCDS9841.1] [protein=protein c-Fos] [protein_id=NP_005243.1] [location=156..1298] [gbkey=CDS]

ATGATGTTCTCGGGCTTCAACGCAGACTACGAGGCGTCATCCTCCCGCTGCAGCAGCGCGTCCCCGGCCG

GGGATAGCCTCTCTTACTACCACTCACCCGCAGACTCCTTCTCCAGCATGGGCTCGCCTGTCAACGCGCA

GGACTTCTGCACGGACCTGGCCGTCTCCAGTGCCAACTTCATTCCCACGGTCACTGCCATCTCGACCAGT

CCGGACCTGCAGTGGCTGGTGCAGCCCGCCCTCGTCTCCTCCGTGGCCCCATCGCAGACCAGAGCCCCTC

ACCCTTTCGGAGTCCCCGCCCCCTCCGCTGGGGCTTACTCCAGGGCTGGCGTTGTGAAGACCATGACAGG

AGGCCGAGCGCAGAGCATTGGCAGGAGGGGCAAGGTGGAACAGTTATCTCCAGAAGAAGAAGAGAAAAGG

AGAATCCGAAGGGAAAGGAATAAGATGGCTGCAGCCAAATGCCGCAACCGGAGGAGGGAGCTGACTGATA

CACTCCAAGCGGAGACAGACCAACTAGAAGATGAGAAGTCTGCTTTGCAGACCGAGATTGCCAACCTGCT

GAAGGAGAAGGAAAAACTAGAGTTCATCCTGGCAGCTCACCGACCTGCCTGCAAGATCCCTGATGACCTG

GGCTTCCCAGAAGAGATGTCTGTGGCTTCCCTTGATCTGACTGGGGGCCTGCCAGAGGTTGCCACCCCGG

AGTCTGAGGAGGCCTTCACCCTGCCTCTCCTCAATGACCCTGAGCCCAAGCCCTCAGTGGAACCTGTCAA

GAGCATCAGCAGCATGGAGCTGAAGACCGAGCCCTTTGATGACTTCCTGTTCCCAGCATCATCCAGGCCC

AGTGGCTCTGAGACAGCCCGCTCCGTGCCAGACATGGACCTATCTGGGTCCTTCTATGCAGCAGACTGGG

AGCCTCTGCACAGTGGCTCCCTGGGGATGGGGCCCATGGCCACAGAGCTGGAGCCCCTGTGCACTCCGGT

GGTCACCTGTACTCCCAGCTGCACTGCTTACACGTCTTCCTTCGTCTTCACCTACCCCGAGGCTGACTCC

TTCCCCAGCTGTGCAGCTGCCCACCGCAAGGGCAGCAGCAGCAATGAGCCTTCCTCTGACTCGCTCAGCT

CACCCACGCTGCTGGCCCTGTGA

>lcl|NM_002015.4_cds_NP_002006.2_1 [gene=FOXO1] [db_xref=CCDS:CCDS9371.1] [protein=forkhead box protein O1] [protein_id=NP_002006.2] [location=430..2397] [gbkey=CDS]

ATGGCCGAGGCGCCTCAGGTGGTGGAGATCGACCCGGACTTCGAGCCGCTGCCCCGGCCGCGCTCGTGCA

CCTGGCCGCTGCCCAGGCCGGAGTTTAGCCAGTCCAACTCGGCCACCTCCAGCCCGGCGCCGTCGGGCAG

CGCGGCTGCCAACCCCGACGCCGCGGCGGGCCTGCCCTCGGCCTCGGCTGCCGCTGTCAGCGCCGACTTC

ATGAGCAACCTGAGCTTGCTGGAGGAGAGCGAGGACTTCCCGCAGGCGCCCGGCTCCGTGGCGGCGGCGG

TGGCGGCGGCGGCCGCCGCGGCCGCCACCGGGGGGCTGTGCGGGGACTTCCAGGGCCCGGAGGCGGGCTG

CCTGCACCCAGCGCCACCGCAGCCCCCGCCGCCCGGGCCGCTGTCGCAGCACCCGCCGGTGCCCCCCGCC

GCCGCTGGGCCGCTCGCGGGGCAGCCGCGCAAGAGCAGCTCGTCCCGCCGCAACGCGTGGGGCAACCTGT

CCTACGCCGACCTCATCACCAAGGCCATCGAGAGCTCGGCGGAGAAGCGGCTCACGCTGTCGCAGATCTA

CGAGTGGATGGTCAAGAGCGTGCCCTACTTCAAGGATAAGGGTGACAGCAACAGCTCGGCGGGCTGGAAG

AATTCAATTCGTCATAATCTGTCCCTACACAGCAAGTTCATTCGTGTGCAGAATGAAGGAACTGGAAAAA

GTTCTTGGTGGATGCTCAATCCAGAGGGTGGCAAGAGCGGGAAATCTCCTAGGAGAAGAGCTGCATCCAT

GGACAACAACAGTAAATTTGCTAAGAGCCGAAGCCGAGCTGCCAAGAAGAAAGCATCTCTCCAGTCTGGC

CAGGAGGGTGCTGGGGACAGCCCTGGATCACAGTTTTCCAAATGGCCTGCAAGCCCTGGCTCTCACAGCA

ATGATGACTTTGATAACTGGAGTACATTTCGCCCTCGAACTAGCTCAAATGCTAGTACTATTAGTGGGAG

ACTCTCACCCATTATGACCGAACAGGATGATCTTGGAGAAGGGGATGTGCATTCTATGGTGTACCCGCCA

TCTGCCGCAAAGATGGCCTCTACTTTACCCAGTCTGTCTGAGATAAGCAATCCCGAAAACATGGAAAATC

TTTTGGATAATCTCAACCTTCTCTCATCACCAACATCATTAACTGTTTCGACCCAGTCCTCACCTGGCAC

CATGATGCAGCAGACGCCGTGCTACTCGTTTGCGCCACCAAACACCAGTTTGAATTCACCCAGCCCAAAC

TACCAAAAATATACATATGGCCAATCCAGCATGAGCCCTTTGCCCCAGATGCCTATACAAACACTTCAGG

ACAATAAGTCGAGTTATGGAGGTATGAGTCAGTATAACTGTGCGCCTGGACTCTTGAAGGAGTTGCTGAC

TTCTGACTCTCCTCCCCATAATGACATTATGACACCAGTTGATCCTGGGGTAGCCCAGCCCAACAGCCGG

GTTCTGGGCCAGAACGTCATGATGGGCCCTAATTCGGTCATGTCAACCTATGGCAGCCAGGCATCTCATA

ACAAAATGATGAATCCCAGCTCCCATACCCACCCTGGACATGCTCAGCAGACATCTGCAGTTAACGGGCG

TCCCCTGCCCCACACGGTAAGCACCATGCCCCACACCTCGGGTATGAACCGCCTGACCCAAGTGAAGACA

CCTGTACAAGTGCCTCTGCCCCACCCCATGCAGATGAGTGCCCTGGGGGGCTACTCCTCCGTGAGCAGCT

GCAATGGCTATGGCAGAATGGGCCTTCTCCACCAGGAGAAGCTCCCAAGTGACTTGGATGGCATGTTCAT

TGAGCGCTTAGACTGTGACATGGAATCCATCATTCGGAATGACCTCATGGATGGAGATACATTGGATTTT

AACTTTGACAATGTGTTGCCCAACCAAAGCTTCCCACACAGTGTCAAGACAACGACACATAGCTGGGTGT

CAGGCTGA

>lcl|NM_001455.4_cds_NP_001446.1_1 [gene=FOXO3] [db_xref=CCDS:CCDS5068.1] [protein=forkhead box protein O3] [protein_id=NP_001446.1] [location=293..2314] [gbkey=CDS]

ATGGCAGAGGCACCGGCTTCCCCGGCCCCGCTCTCTCCGCTCGAAGTGGAGCTGGACCCGGAGTTCGAGC

CCCAGAGCCGTCCGCGATCCTGTACGTGGCCCCTGCAAAGGCCGGAGCTCCAAGCGAGCCCTGCCAAGCC

CTCGGGGGAGACGGCCGCCGACTCCATGATCCCCGAGGAGGAGGACGATGAAGACGACGAGGACGGCGGG

GGACGGGCCGGCTCGGCCATGGCGATCGGCGGCGGCGGCGGGAGCGGCACGCTGGGCTCCGGGCTGCTCC

TTGAGGACTCGGCCCGGGTGCTGGCACCCGGAGGGCAAGACCCCGGGTCTGGGCCAGCCACCGCGGCGGG

CGGGCTGAGCGGGGGTACACAGGCGCTGCTGCAGCCTCAGCAACCGCTGCCACCGCCGCAGCCGGGGGCG

GCTGGGGGCTCCGGGCAGCCGAGGAAATGTTCGTCGCGGCGGAACGCCTGGGGAAACCTGTCCTACGCGG

ACCTGATCACCCGCGCCATCGAGAGCTCCCCGGACAAACGGCTCACTCTGTCCCAGATCTACGAGTGGAT

GGTGCGTTGCGTGCCCTACTTCAAGGATAAGGGCGACAGCAACAGCTCTGCCGGCTGGAAGAACTCCATC

CGGCACAACCTGTCACTGCATAGTCGATTCATGCGGGTCCAGAATGAGGGAACTGGCAAGAGCTCTTGGT

GGATCATCAACCCTGATGGGGGGAAGAGCGGAAAAGCCCCCGGCGGCGGGCTGTCTCCATGGACAATAG

CAACAAGTATACCAAGAGCCGTGGCCGCGCAGCCAAGAAGAAGGCAGCCCTGCAGACAGCCCCCGAATCA

GCTGACGACAGTCCCTCCCAGCTCTCCAAGTGGCCTGGCAGCCCCACGTCACGCAGCAGTGATGAGCTGG

ATGCGTGGACGGACTTCCGTTCACGCACCAATTCTAACGCCAGCACAGTCAGTGGCCGCCTGTCGCCCAT

CATGGCAAGCACAGAGTTGGATGAAGTCCAGGACGATGATGCGCCTCTCTCGCCCATGCTCTACAGCAGC

TCAGCCAGCCTGTCACCTTCAGTAAGCAAGCCGTGCACGGTGGAACTGCCACGGCTGACTGATATGGCAG

GCACCATGAATCTGAATGATGGGCTGACTGAAAACCTCATGGACGACCTGCTGGATAACATCACGCTCCC

GCCATCCCAGCCATCGCCCACTGGGGGACTCATGCAGCGGAGCTCTAGCTTCCCGTATACCACCAAGGGC

TCGGGCCTGGGCTCCCCAACCAGCTCCTTTAACAGCACGGTGTTCGGACCTTCATCTCTGAACTCCCTAC

GCCAGTCTCCCATGCAGACCATCCAAGAGAACAAGCCAGCTACCTTCTCTTCCATGTCACACTATGGTAA

CCAGACACTCCAGGACCTGCTCACTTCGGACTCACTTAGCCACAGCGATGTCATGATGACACAGTCGGAC

CCCTTGATGTCTCAGGCCAGCACCGCTGTGTCTGCCCAGAATTCCCGCCGGAACGTGATGCTTCGCAATG

ATCCGATGATGTCCTTTGCTGCCCAGCCTAACCAGGGAAGTTTGGTCAATCAGAACTTGCTCCACCACCA

GCACCAAACCCAGGGCGCTCTTGGTGGCAGCCGTGCCTTGTCGAATTCTGTCAGCAACATGGGCTTGAGT

GAGTCCAGCAGCCTTGGGTCAGCCAAACACCAGCAGCAGTCTCCTGTCAGCCAGTCTATGCAAACCCTCT

CGGACTCTCTCTCAGGCTCCTCCTTGTACTCAACTAGTGCAAACCTGCCCGTCATGGGCCATGAGAAGTT

CCCCAGCGACTTGGACCTGGACATGTTCAATGGGAGCTTGGAATGTGACATGGAGTCCATTATCCGTAGT

GAACTCATGGATGCTGATGGGTTGGATTTTAACTTTGATTCCCTCATCTCCACACAGAATGTTGTTGGTT

TGAACGTGGGGAACTTCACTGGTGCTAAGCAGGCCTCATCTCAGAGCTGGGTGCCAGGCTGA

>lcl|NM_000152.5_cds_NP_000143.2_1 [gene=GAA] [db_xref=CCDS:CCDS32760.1] [protein=lysosomal alpha-glucosidase preproprotein] [protein_id=NP_000143.2] [location=343..3201] [gbkey=CDS]

ATGGGAGTGAGGCACCCGCCCTGCTCCCACCGGCTCCTGGCCGTCTGCGCCCTCGTGTCCTTGGCAACCG

CTGCACTCCTGGGGCACATCCTACTCCATGATTTCCTGCTGGTTCCCCGAGAGCTGAGTGGCTCCTCCCC

AGTCCTGGAGGAGACTCACCCAGCTCACCAGCAGGGAGCCAGCAGACCAGGGCCCCGGGATGCCCAGGCA

CACCCCGGCCGTCCCAGAGCAGTGCCCACACAGTGCGACGTCCCCCCCAACAGCCGCTTCGATTGCGCCC

CTGACAAGGCCATCACCCAGGAACAGTGCGAGGCCCGCGGCTGTTGCTACATCCCTGCAAAGCAGGGGCT

GCAGGGAGCCCAGATGGGGCAGCCCTGGTGCTTCTTCCCACCCAGCTACCCCAGCTACAAGCTGGAGAAC

CTGAGCTCCTCTGAAATGGGCTACACGGCCACCCTGACCCGTACCACCCCCACCTTCTTCCCCAAGGACA

TCCTGACCCTGCGGCTGGACGTGATGATGGAGACTGAGAACCGCCTCCACTTCACGATCAAAGATCCAGC

TAACAGGCGCTACGAGGTGCCCTTGGAGACCCCGCATGTCCACAGCCGGGCACCGTCCCCACTCTACAGC

GTGGAGTTCTCCGAGGAGCCCTTCGGGGTGATCGTGCGCCGGCAGCTGGACGGCCGCGTGCTGCTGAACA

CGACGGTGGCGCCCCTGTTCTTTGCGGACCAGTTCCTTCAGCTGTCCACCTCGCTGCCCTCGCAGTATAT

CACAGGCCTCGCCGAGCACCTCAGTCCCCTGATGCTCAGCACCAGCTGGACCAGGATCACCCTGTGGAAC

CGGGACCTTGCGCCCACGCCCGGTGCGAACCTCTACGGGTCTCACCCTTTCTACCTGGCGCTGGAGGACG

GCGGGTCGGCACACGGGGTGTTCCTGCTAAACAGCAATGCCATGGATGTGGTCCTGCAGCCGAGCCCTGC

CCTTAGCTGGAGGTCGACAGGTGGGATCCTGGATGTCTACATCTTCCTGGGCCCAGAGCCCAAGAGCGTG

GTGCAGCAGTACCTGGACGTTGTGGGATACCCGTTCATGCCGCCATACTGGGGCCTGGGCTTCCACCTGT

GCCGCTGGGGCTACTCCTCCACCGCTATCACCCGCCAGGTGGTGGAGAACATGACCAGGGCCCACTTCCC

CCTGGACGTCCAGTGGAACGACCTGGACTACATGGACTCCCGGAGGGACTTCACGTTCAACAAGGATGGC

TTCCGGGACTTCCCGGCCATGGTGCAGGAGCTGCACCAGGGCGGCCGGCGCTACATGATGATCGTGGATC

CTGCCATCAGCAGCTCGGGCCCTGCCGGGAGCTACAGGCCCTACGACGAGGGTCTGCGGAGGGGGGTTTT

CATCACCAACGAGACCGGCCAGCCGCTGATTGGGAAGGTATGGCCCGGGTCCACTGCCTTCCCCGACTTC

ACCAACCCCACAGCCCTGGCCTGGTGGGAGGACATGGTGGCTGAGTTCCATGACCAGGTGCCCTTCGACG

GCATGTGGATTGACATGAACGAGCCTTCCAACTTCATCAGGGGCTCTGAGGACGGCTGCCCCAACAATGA

GCTGGAGAACCCACCCTACGTGCCTGGGGTGGTTGGGGGGACCCTCCAGGCGGCCACCATCTGTGCCTCC

AGCCACCAGTTTCTCTCCACACACTACAACCTGCACAACCTCTACGGCCTGACCGAAGCCATCGCCTCCC

ACAGGGCGCTGGTGAAGGCTCGGGGGACACGCCCATTTGTGATCTCCCGCTCGACCTTTGCTGGCCACGG

CCGATACGCCGGCCACTGGACGGGGGACGTGTGGAGCTCCTGGGAGCAGCTCGCCTCCTCCGTGCCAGAA

ATCCTGCAGTTTAACCTGCTGGGGGTGCCTCTGGTCGGGGCCGACGTCTGCGGCTTCCTGGGCAACACCT

CAGAGGAGCTGTGTGTGCGCTGGACCCAGCTGGGGGCCTTCTACCCCTTCATGCGGAACCACAACAGCCT

GCTCAGTCTGCCCCAGGAGCCGTACAGCTTCAGCGAGCCGGCCCAGCAGGCCATGAGGAAGGCCCTCACC

CTGCGCTACGCACTCCTCCCCCACCTCTACACACTGTTCCACCAGGCCCACGTCGCGGGGGAGACCGTGG

CCCGGCCCCTCTTCCTGGAGTTCCCCAAGGACTCTAGCACCTGGACTGTGGACCACCAGCTCCTGTGGGG

GGAGGCCCTGCTCATCACCCCAGTGCTCCAGGCCGGGAAGGCCGAAGTGACTGGCTACTTCCCCTTGGGC

ACATGGTACGACCTGCAGACGGTGCCAGTAGAGGCCCTTGGCAGCCTCCCACCCCCACCTGCAGCTCCCC

GTGAGCCAGCCATCCACAGCGAGGGGCAGTGGGTGACGCTGCCGGCCCCCCTGGACACCATCAACGTCCA

CCTCCGGGCTGGGTACATCATCCCCCTGCAGGGCCCTGGCCTCACAACCACAGAGTCCCGCCAGCAGCCC

ATGGCCCTGGCTGTGGCCCTGACCAAGGGTGGGGAGGCCCGAGGGGAGCTGTTCTGGGACGATGGAGAGA

GCCTGGAAGTGCTGGAGCGAGGGGCCTACACACAGGTCATCTTCCTGGCCAGGAATAACACGATCGTGAA

TGAGCTGGTACGTGTGACCAGTGAGGGAGCTGGCCTGCAGCTGCAGAAGGTGACTGTCCTGGGCGTGGCC

ACGGCGCCCCAGCAGGTCCTCTCCAACGGTGTCCCTGTCTCCAACTTCACCTACAGCCCCGACACCAAGG

TCCTGGACATCTGTGTCTCGCTGTTGATGGGAGAGCAGTTTCTCGTCAGCTGGTGTTAG

>lcl|NM_007278.2_cds_NP_009209.1_1 [gene=GABARAP] [db_xref=CCDS:CCDS11092.1] [protein=gamma-aminobutyric acid receptor-associated protein] [protein_id=NP_009209.1] [location=120..473] [gbkey=CDS]

ATGAAGTTCGTGTACAAAGAAGAGCATCCGTTCGAGAAGCGCCGCTCTGAGGGCGAGAAGATCCGAAAGA

AATACCCGGACCGGGTGCCGGTGATAGTAGAAAAGGCTCCCAAAGCTCGGATAGGAGACCTGGACAAAAA

GAAATACCTGGTGCCTTCTGATCTCACAGTTGGTCAGTTCTACTTCTTGATCCGGAAGCGAATTCATCTC

CGAGCTGAGGATGCCTTGTTTTTCTTTGTCAACAATGTCATTCCACCCACCAGTGCCACAATGGGTCAGC

TGTACCAGGAACACCATGAAGAAGACTTCTTTCTCTACATTGCCTACAGTGACGAAAGTGTCTACGGTCT

GTGA

>lcl|NM_001363598.2_cds_NP_001350527.1_1 [gene=GABARAPL1] [db_xref=CCDS:CCDS86280.1] [protein=gamma-aminobutyric acid receptor-associated protein-like 1 isoform 1] [protein_id=NP_001350527.1] [location=254..694] [gbkey=CDS]

ATGAAGTTCCAGTACAAGGAGGACCATCCCTTTGAGTATCGGAAAAAGGAAGGAGAAAAGATCCGGAAGA

AATATCCGGACAGGGTCCCCGTGATTGTAGAGAAGGCTCCAAAAGCCAGGGTGCCTGATCTGGACAAGAG

GAAGTACCTAGTGCCCTCTGACCTTACTGTTGGCCAGTTCTACTTCTTAATCCGGAAGAGAATCCACCTG

AGACCTGAGGACGCCTTATTCTTCTTTGTCAACAACACCATCCCTCCCACCAGTGCTACCATGGGCCAAC

TGTATGAGGTAATGGTTCTGGTTGCACAATACTGGATGCCGTCCAGTGCAGTCTGGCATCCTCTAGCCCT

TGTTCTAGATGCGTTGATAACACATCTGAGAAGTGGGGCAGAAGGTGTTATTTATCCGGATCCTCTTACA

TATGGCAGTGTAAGGCTCTGA

>lcl|NM_007285.7_cds_NP_009216.1_1 [gene=GABARAPL2] [db_xref=CCDS:CCDS10921.1] [protein=gamma-aminobutyric acid receptor-associated protein-like 2] [protein_id=NP_009216.1] [location=109..462] [gbkey=CDS]

ATGAAGTGGATGTTCAAGGAGGACCACTCGCTGGAACACAGATGCGTGGAGTCCGCGAAGATTCGAGCGA

AATATCCCGACAGGGTTCCGGTGATTGTGGAAAAGGTCTCAGGCTCTCAGATTGTTGACATTGACAAACG

GAAGTACTTGGTTCCATCTGATATCACTGTGGCTCAGTTCATGTGGATCATCAGGAAAAGGATCCAGCTT

CCTTCTGAAAAGGCGATCTTCCTGTTTGTGGATAAGACAGTCCCACAGTCCAGCCTAACTATGGGACAGC

TTTACGAGAAGGAAAAAGATGAAGATGGATTCTTATATGTGGCCTACAGCGGAGAGAACACTTTTGGCTT

CTGA

>lcl|NM_002046.7_cds_NP_002037.2_1 [gene=GAPDH] [db_xref=CCDS:CCDS8549.1] [protein=glyceraldehyde-3-phosphate dehydrogenase isoform 1] [protein_id=NP_002037.2] [location=77..1084] [gbkey=CDS]

ATGGGGAAGGTGAAGGTCGGAGTCAACGGATTTGGTCGTATTGGGCGCCTGGTCACCAGGGCTGCTTTTA

ACTCTGGTAAAGTGGATATTGTTGCCATCAATGACCCCTTCATTGACCTCAACTACATGGTTTACATGTT

CCAATATGATTCCACCCATGGCAAATTCCATGGCACCGTCAAGGCTGAGAACGGGAAGCTTGTCATCAAT

GGAAATCCCATCACCATCTTCCAGGAGCGAGATCCCTCCAAAATCAAGTGGGGCGATGCTGGCGCTGAGT

ACGTCGTGGAGTCCACTGGCGTCTTCACCACCATGGAGAAGGCTGGGGCTCATTTGCAGGGGGGAGCCAA

AAGGGTCATCATCTCTGCCCCCTCTGCTGATGCCCCCATGTTCGTCATGGGTGTGAACCATGAGAAGTAT

GACAACAGCCTCAAGATCATCAGCAATGCCTCCTGCACCACCAACTGCTTAGCACCCCTGGCCAAGGTCA

TCCATGACAACTTTGGTATCGTGGAAGGACTCATGACCACAGTCCATGCCATCACTGCCACCCAGAAGAC

TGTGGATGGCCCCTCCGGGAAACTGTGGCGTGATGGCCGCGGGGCTCTCCAGAACATCATCCCTGCCTCT

ACTGGCGCTGCCAAGGCTGTGGGCAAGGTCATCCCTGAGCTGAACGGGAAGCTCACTGGCATGGCCTTCC

GTGTCCCCACTGCCAACGTGTCAGTGGTGGACCTGACCTGCCGTCTAGAAAAACCTGCCAAATATGATGA

CATCAAGAAGGTGGTGAAGCAGGCGTCGGAGGGCCCCCTCAAGGGCATCCTGGGCTACACTGAGCACCAG

GTGGTCTCCTCTGACTTCAACAGCGACACCCACTCCTCCACCTTTGACGCTGGGGCTGGCATTGCCCTCA

ACGACCACTTTGTCAAGCTCATTTCCTGGTATGACAACGAATTTGGCTACAGCAACAGGGTGGTGGACCT

CATGGCCCACATGGCCTCCAAGGAGTAA

>lcl|NM_006496.4_cds_NP_006487.1_1 [gene=GNAI3] [db_xref=CCDS:CCDS802.1] [protein=guanine nucleotide-binding protein G(i) subunit alpha-3] [protein_id=NP_006487.1] [location=107..1171] [gbkey=CDS]

ATGGGCTGCACGTTGAGCGCCGAAGACAAGGCGGCAGTGGAGCGAAGCAAGATGATCGACCGCAACTTAC

GGGAGGACGGGGAAAAAGCGGCCAAAGAAGTGAAGCTGCTGCTACTCGGTGCTGGAGAATCTGGTAAAAG

CACCATTGTGAAACAGATGAAAATCATTCATGAGGATGGCTATTCAGAGGATGAATGTAAACAATATAAA

GTAGTTGTCTACAGCAATACTATACAGTCCATCATTGCAATCATAAGAGCCATGGGACGGCTAAAGATTG

ACTTTGGGGAAGCTGCCAGGGCAGATGATGCCCGGCAATTATTTGTTTTAGCTGGCAGTGCTGAAGAAGG

AGTCATGACTCCAGAACTAGCAGGAGTGATTAAACGGTTATGGCGAGATGGTGGGGTACAAGCTTGCTTC

AGCAGATCCAGGGAATATCAGCTCAATGATTCTGCTTCATATTATCTAAATGATCTGGATAGAATATCCC

AGTCTAACTACATTCCAACTCAGCAAGATGTTCTTCGGACGAGAGTGAAGACCACAGGCATTGTAGAAAC

ACATTTCACCTTCAAAGACCTATACTTCAAGATGTTTGATGTAGGTGGCCAAAGATCAGAACGAAAAAAG

TGGATTCACTGTTTTGAGGGAGTGACAGCAATTATCTTCTGTGTGGCCCTCAGTGATTATGACCTTGTTC

TGGCTGAGGACGAGGAGATGAACCGAATGCATGAAAGCATGAAACTGTTTGACAGCATTTGTAATAACAA

ATGGTTTACAGAAACTTCAATCATTCTCTTCCTTAACAAGAAAGACCTTTTTGAGGAAAAAATAAAGAGG

AGTCCGTTAACTATCTGTTATCCAGAATACACAGGTTCCAATACATATGAAGAGGCAGCTGCCTATATTC

AATGCCAGTTTGAAGATCTGAACAGAAGAAAAGATACCAAGGAGATCTATACTCACTTCACCTGTGCCAC

AGACACGAAGAATGTGCAGTTTGTTTTTGATGCTGTTACAGATGTCATCATTAAAAACAACTTAAAGGAA

TGTGGACTTTATTGA

>lcl|NM_020399.4_cds_NP_065132.1_1 [gene=GOPC] [db_xref=CCDS:CCDS5117.1] [protein=Golgi-associated PDZ and coiled-coil motif-containing protein isoform a] [protein_id=NP_065132.1] [location=224..1612] [gbkey=CDS]

ATGTCGGCGGGCGGTCCATGCCCAGCAGCAGCCGGAGGGGGCCCAGGGGGCGCCTCCTGCTCCGTGGGGG

CCCCTGGCGGGGTATCCATGTTCCGGTGGCTGGAGGTGCTGGAGAAGGAGTTCGACAAAGCTTTTGTGGA

TGTGGATCTGCTCCTGGGAGAGATCGATCCAGACCAAGCGGACATCACTTATGAGGGGCGACAGAAGATG

ACCAGCCTGAGCTCCTGCTTTGCACAGCTTTGCCACAAAGCCCAGTCTGTGTCTCAAATCAACCACAAGC

TGGAGGCACAGTTGGTGGATCTGAAATCTGAACTGACAGAAACCCAAGCAGAGAAAGTTGTTTTGGAGAA

AGAAGTACATGATCAGCTTTTACAGCTGCACTCTATTCAGCTGCAGCTTCATGCTAAAACTGGTCAAAGT

GCTGACTCTGGTACCATTAAGGCAAAATTGTCTGGCCCCTCTGTGGAGGAGCTGGAAAGAGAGCTTGAGG

CAAACAAAAAAGAAAAAATGAAAGAAGCACAACTTGAAGCTGAAGTGAAATTGTTGAGAAAAGAGAATGA

AGCCCTTCGTAGACATATAGCTGTTCTCCAGGCTGAAGTATATGGGGCGAGACTAGCTGCCAAGTACTTG

GATAAGGAACTGGCAGGAAGGGTCCAACAGATACAATTGCTAGGACGAGATATGAAGGGACCTGCTCATG

ATAAGCTTTGGAACCAATTAGAAGCTGAAATACATTTGCATCGTCACAAAACTGTGATCCGAGCCTGCAG

AGGACGTAATGACTTGAAACGACCAATGCAAGCACCACCAGGCCATGATCAAGATTCCCTAAAGAAAAGC

CAAGGTGTTGGTCCAATTAGAAAAGTTCTCCTCCTTAAGGAAGATCATGAAGGCCTTGGCATTTCAATTA

CAGGTGGGAAAGAACATGGTGTTCCAATCCTCATCTCTGAGATCCATCCGGGGCAACCTGCTGATAGATG

CGGAGGGCTGCACGTTGGGGATGCTATTTTGGCAGTCAACGGAGTTAACCTAAGGGACACAAAGCATAAA

GAAGCTGTAACTATTCTTTCTCAGCAGAGAGGAGAGATTGAATTTGAAGTAGTTTATGTGGCTCCTGAAG

TGGATTCTGATGATGAAAACGTAGAGTATGAAGATGAGAGTGGACATCGTTACCGTTTGTACCTTGATGA

GTTAGAAGGAGGTGGTAACCCTGGTGCTAGTTGCAAAGACACAAGTGGGGAAATCAAAGTATTACAAGGA

TTTAATAAGAAGGCAGTAACTGACACACATGAAAATGGAGACCTGGGCACTGCAAGTGAAACTCCGCTAG

ATGACGGTGCTTCAAAATTAGATGATCTGCACACTCTGTATCATAAAAAATCTTATTAA

>lcl|NM_017551.3_cds_NP_060021.1_1 [gene=GRID1] [db_xref=CCDS:CCDS31236.1] [protein=glutamate receptor ionotropic, delta-1 precursor] [protein_id=NP_060021.1] [location=404..3433] [gbkey=CDS]

ATGGAAGCGCTGACGCTGTGGCTTCTCCCCTGGATATGCCAGTGCGTGTCGGTGCGGGCCGACTCCATCA

TCCACATCGGTGCCATCTTCGAGGAGAACGCGGCCAAGGACGACAGGGTGTTCCAGTTGGCGGTATCCGA

CCTGAGCCTCAACGATGACATCCTGCAGAGCGAGAAGATCACCTACTCCATCAAGGTCATCGAGGCCAAC

AACCCATTCCAGGCTGTGCAGGAAGCCTGTGACCTCATGACCCAGGGGATTTTGGCCTTGGTCACGTCCA

CTGGCTGTGCATCTGCCAATGCCCTGCAGTCCCTCACGGATGCCATGCACATCCCACACCTCTTTGTCCA

GCGCAACCCGGGAGGGTCGCCACGCACCGCATGCCACCTGAACCCCAGCCCCGATGGTGAGGCCTACACA

CTGGCTTCGAGACCACCCGTCCGCCTCAATGATGTCATGCTCAGGCTGGTGACGGAGCTGCGCTGGCAGA

AGTTCGTCATGTTCTACGACAGCGAGTATGATATCCGTGGGCTTCAAAGCTTTCTGGACCAGGCCTCGCG

GCTGGGCCTTGACGTCTCTTTACAAAAGGTGGACAAGAACATTAGCCACGTATTCACCAGCCTCTTCACC

ACGATGAAGACAGAGGAGCTGAATCGCTACCGGGACACGCTTCGCCGCGCCATCCTGCTGCTCAGCCCAC

AGGGAGCCCACTCCTTCATCAACGAGGCCGTGGAGACCAACCTGGCTTCCAAGGACAGCCACTGGGTCTT

TGTGAATGAGGAAATCAGTGACCCGGAGATCCTGGATCTGGTCCATAGTGCCCTTGGAAGGATGACCGTG

GTCCGGCAAATCTTTCCGTCTGCAAAGGACAATCAGAAATGCACGAGGAACAACCACCGCATCTCCTCCC

TGCTCTGCGACCCCCAGGAAGGCTACCTCCAGATGCTGCAGATCTCCAACCTCTATCTGTATGACAGTGT

TCTGATGCTGGCCAACGCCTTTCACAGGAAGCTGGAGGACCGGAAGTGGCATAGCATGGCGAGCCTCAAC

TGCATACGGAAATCCACTAAGCCATGGAATGGTGGGAGGTCCATGCTGGATACCATCAAAAAGGGCCACA

TCACTGGCCTCACTGGGGTGATGGAGTTTCGGGAGGACAGTTCGAATCCCTATGTCCAGTTTGAAATCCT

TGGCACTACCTATAGTGAGACTTTTGGCAAAGACATGCGCAAGTTGGCGACATGGGACTCAGAGAAGGGC

TTGAATGGCAGCTTGCAAGAGAGGCCCATGGGCAGCCGCCTCCAAGGATTGACTCTTAAAGTGGTGACTG

TCTTGGAAGAGCCTTTCGTGATGGTGGCTGAGAACATCCTAGGACAGCCCAAGCGCTACAAAGGGTTCTC

CATAGATGTCCTGGATGCACTGGCCAAGGCTCTGGGCTTTAAATATGAGATTTACCAAGCCCCTGATGGC

AGGTACGGTCACCAGCTCCATAACACCTCCTGGAACGGGATGATCGGGGAGCTCATCAGCAAGAGAGCAG

ACTTGGCCATCTCTGCCATCACCATCACCCCAGAGAGGGAGAGCGTTGTGGACTTCAGCAAGCGGTACAT

GGACTATTCAGTGGGGATTCTAATTAAGAAGCCCGAGGAGAAAATCAGCATCTTCTCCCTCTTTGCTCCA

TTTGATTTCGCTGTGTGGGCCTGCATTGCAGCAGCCATCCCTGTGGTTGGTGTGCTGATATTTGTGTTGA

ACAGGATACAGGCTGTGAGGGCTCAGAGTGCTGCCCAGCCCAGGCCGTCAGCTTCTGCCACTCTGCACAG

CGCCATCTGGATTGTCTATGGAGCCTTCGTACAGCAAGGTGGCGAATCTTCCGTGAACTCCATGGCCATG

CGCATCGTGATGGGCAGCTGGTGGCTCTTCACGCTCATTGTGTGCTCCTCCTACACAGCCAACCTTGCTG

CCTTCCTCACAGTGTCCAGGATGGACAACCCCATAAGGACTTTCCAGGACCTGTCCAAACAAGTGGAAAT

GTCTTATGGCACTGTCCGGGATTCTGCTGTATATGAGTACTTCCGAGCCAAGGGCACCAACCCCCTGGAG

CAGGACAGCACGTTTGCTGAACTCTGGCGGACCATCAGCAAGAACGGAGGGGCTGACAACTGCGTGTCCA

GTCCTTCAGAAGGCATCAGGAAGGCAAAGAAGGGGAACTACGCCTTCCTGTGGGATGTGGCCGTGGTGGA

ATACGCAGCCCTGACGGATGACGACTGCTCGGTGACTGTCATCGGCAACAGCATCAGCAGCAAGGGTTAC

GGGATTGCCCTGCAGCATGGCAGCCCCTACAGGGACCTCTTCTCCCAGAGGATCCTGGAGCTGCAGGACA

CAGGGGACCTGGATGTGCTGAAGCAGAAGTGGTGGCCGCACATGGGCCGCTGTGACCTCACCAGCCATGC

CAGCGCCCAGGCCGACGGCAAATCCCTCAAGCTGCACAGCTTCGCCGGGGTCTTCTGCATCCTGGCCATT

GGCCTGCTCCTGGCCTGCCTGGTGGCTGCCCTGGAGTTGTGGTGGAACAGCAACCGGTGCCACCAGGAGA

CCCCCAAGGAGGACAAAGAAGTGAACTTGGAGCAGGTCCACCGGCGCATGAACAGCCTCATGGATGAAGA

CATTGCTCACAAGCAGATTTCCCCAGCGTCGATTGAGCTCTCGGCCCTGGAGATGGGGGGCCTGGCTCCC

ACCCAGACCTTGGAGCCGACACGGGAGTACCAGAACACCCAGCTCTCGGTCAGCACCTTTCTGCCAGAGC

AGAGCAGCCATGGCACCAGCCGGACACTCTCATCAGGGCCCAGCAGCAACCTGCCGCTGCCGCTGAGCAG

CTCGGCGACCATGCCCTCCATGCAGTGCAAACACAGGTCACCCAACGGGGGGCTGTTCCGGCAGAGCCCG

GTGAAGACCCCCATCCCCATGTCCTTCCAGCCCGTGCCTGGAGGCGTCCTTCCAGAGGCTCTGGACACCT

CCCACGGGACCTCCATCTGA

>lcl|NM_001286838.1_cds_NP_001273767.1_1 [gene=GRID2] [db_xref=CCDS:CCDS68758.1] [protein=glutamate receptor ionotropic, delta-2 isoform 2 precursor] [protein_id=NP_001273767.1] [location=259..2997] [gbkey=CDS]

ATGGAAGTTTTCCCCTTTCTCTTGGTTTTGTCCGTCTGGTGGTCTCGAACCTGGGACTCGGCGAATGCGG

ATTCGATCATTCACATCGGAGCAATTTTTGATGAATCTGCCAAAAAGGATGATGAGGTATTTCGCACTGC

GGTTGGTGACCTTAACCAGAATGAGGAGATCTTACAGACTGAGAAAATCACATTTTCAGTGACGTTTGTT

GATGGCAACAACCCTTTCCAAGCAGTTCAAGAAGATATCCGTGGAATACAGGAGTTCTTGGACAAAGTCT

CTCAGCAGGGAATGGATGTTGCACTTCAGAAGGTAGAAAACAACATCAATAAAATGATTACCACTCTCTT

TGACACCATGAGAATAGAAGAACTGAATCGCTATCGAGACACTCTTAGGCGAGCGATCCTTGTTATGAAT

CCTGCTACAGCCAAATCCTTCATTACTGAGGTTGTGGAGACTAATTTGGTTGCTTTTGACTGTCACTGGA

TCATTATAAATGAGGAAATAAACGATGTGGACGTACAGGAACTTGTAAGAAGGTCAATTGGAAGGTTAAC

GATTATTCGGCAGACATTTCCAGTTCCCCAGAACATAAGTCAGCGGTGTTTCCGTGGCAACCATCGAATA

TCTTCAACATTGTGTGATCCAAAGGATCCATTTGCTCAGAATATGGAGATTTCCAACCTTTACATATATG

ACACGGTGCTTCTGCTTGCTAATGCTTTTCATAAGAAGCTGGAGGACCGAAAGTGGCACAGCATGGCAAG

TCTGTCATGTATCAGAAAGAACTCAAAGCCCTGGCAGGGTGGGCGCTCCATGTTGGAGACCATCAAGAAG

GGTGGAGTTAGTGGGTTGACTGGAGAGCTAGAATTTGGAGAAAATGGAGGCAATCCCAATGTCCACTTTG

AAATCCTTGGAACCAACTATGGAGAAGAGCTTGGCAGAGGTGTTCGAAAACTTGGTTGCTGGAATCCTGT

CACAGGTCTGAATGGGTCACTGACTGACAAGAAATTGGAGAATAACATGCGTGGAGTGGTTCTACGTGTA

GTAACTGTTCTGGAAGAACCTTTTGTGATGGTCTCTGAAAATGTCTTGGGTAAGCCGAAGAAATACCAGG

GCTTCTCCATTGATGTTTTGGATGCCTTATCTAACTACCTGGGTTTTAACTACGAAATTTACGTAGCACC

GGATCACAAATACGGAAGCCCACAAGAAGATGGGACATGGAATGGCTTGGTAGGAGAACTTGTCTTTAAG

AGAGCCGACATAGGGATTTCTGCTTTAACCATCACTCCAGATCGTGAAAATGTGGTGGACTTTACGACAC

GTTACATGGACTACTCAGTGGGGGTACTACTTCGAAGGGCTGAAAAGACAGTGGATATGTTTGCCTGTCT

TGCACCATTTGATCTCTCTCTATGGGCTTGCATTGCTGGCACAGTCCTTCTGGTGGGTCTACTGGTCTAC

CTCTTGAACTGGCTTAATCCCCCACGATTACAAATGGGATCAATGACGTCTACTACTCTCTACAACTCCA

TGTGGTTTGTGTATGGATCTTTTGTACAACAAGGCGGGGAAGTCCCGTACACGACTCTGGCTACCCGAAT

GATGATGGGGGCTTGGTGGCTATTTGCTTTGATTGTTATCTCATCTTACACGGCAAACCTCGCTGCTTTC

CTCACTATTACACGCATTGAAAGTTCCATCCAGTCTCTCCAGGACCTTTCCAAGCAAACAGAAATCCCTT

ATGGCACAGTCCTAGACTCTGCGGTATATGAGCATGTCCGCATGAAAGGACTGAATCCTTTTGAGAGGGA

CAGCATGTATTCCCAAATGTGGCGGATGATCAACCGAAGCAATGGATCGGAGAACAATGTTCTGGAGTCC

CAGGCAGGCATTCAAAAGGTAAAATATGGAAATTATGCTTTCGTATGGGATGCAGCTGTATTGGAATATG

TGGCTATCAATGACCCAGATTGTTCCTTTTACACCATTGGAAATACTGTTGCTGATCGGGGATATGGAAT

TGCATTACAACATGGCAGTCCTTACCGAGATGTTTTTTCACAAAGGATCCTGGAGCTTCAGCAGAATGGT

GACATGGACATCCTGAAGCACAAATGGTGGCCTAAGAATGGCCAGTGTGACCTGTACTCGTCAGTGGACA

CAAAGCAGAAAGGAGGCGCCCTGGACATAAAGAGCTTTGCAGGGGTCTTTTGTATCCTGGCTGCTGGAAT

TGTCCTCTCCTGCTTCATAGCCATGCTGGAGACGTGGTGGAACAAGAGGAAAGGCTCCCGGGTTCCATCA

AAAGAGGATGACAAGGAAATTGACCTGGAGCACCTCCATAGACGTGTAAATAGCTTGTGCACAGATGACG

ACAGCCCCCATAAACAGTTTTCCACCTCGTCAATTGATTTGACCCCTCTGGACATTGACACTTTGCCAAC

ACGACAAGCACTGGAGCAAATCAGTGATTTCAGGAACACTCATATTACCACAACAACCTTTATCCCAGAG

CAGATCCAGACTCTTAGCCGCACACTGTCAGCTAAAGCTGCTTCTGGTTTCACTTTTGGCAACGTGCCTG

AGCACCGAACTGGCCCTTTTAGGCACAGGGCACCTAATGGGGGCTTTTTCAGGAGTCCTATAAAAACAAT

GTCATCTATTCCTTATCAACCAACTCCTACCCTGGGGCTCAATCTGGGTAATGATCCAGACCGAGGCACC

TCCATATGA

>lcl|NM_004964.3_cds_NP_004955.2_1 [gene=HDAC1] [db_xref=CCDS:CCDS360.1] [protein=histone deacetylase 1] [protein_id=NP_004955.2] [location=88..1536] [gbkey=CDS]

ATGGCGCAGACGCAGGGCACCCGGAGGAAAGTCTGTTACTACTACGACGGGGATGTTGGAAATTACTATT

ATGGACAAGGCCACCCAATGAAGCCTCACCGAATCCGCATGACTCATAATTTGCTGCTCAACTATGGTCT

CTACCGAAAAATGGAAATCTATCGCCCTCACAAAGCCAATGCTGAGGAGATGACCAAGTACCACAGCGAT

GACTACATTAAATTCTTGCGCTCCATCCGTCCAGATAACATGTCGGAGTACAGCAAGCAGATGCAGAGAT

TCAACGTTGGTGAGGACTGTCCAGTATTCGATGGCCTGTTTGAGTTCTGTCAGTTGTCTACTGGTGGTTC

TGTGGCAAGTGCTGTGAAACTTAATAAGCAGCAGACGGACATCGCTGTGAATTGGGCTGGGGGCCTGCAC

CATGCAAAGAAGTCCGAGGCATCTGGCTTCTGTTACGTCAATGATATCGTCTTGGCCATCCTGGAACTGC

TAAAGTATCACCAGAGGGTGCTGTACATTGACATTGATATTCACCATGGTGACGGCGTGGAAGAGGCCTT

CTACACCACGGACCGGGTCATGACTGTGTCCTTTCATAAGTATGGAGAGTACTTCCCAGGAACTGGGGAC

CTACGGGATATCGGGGCTGGCAAAGGCAAGTATTATGCTGTTAACTACCCGCTCCGAGACGGGATTGATG

ACGAGTCCTATGAGGCCATTTTCAAGCCGGTCATGTCCAAAGTAATGGAGATGTTCCAGCCTAGTGCGGT

GGTCTTACAGTGTGGCTCAGACTCCCTATCTGGGGATCGGTTAGGTTGCTTCAATCTAACTATCAAAGGA

CACGCCAAGTGTGTGGAATTTGTCAAGAGCTTTAACCTGCCTATGCTGATGCTGGGAGGCGGTGGTTACA

CCATTCGTAACGTTGCCCGGTGCTGGACATATGAGACAGCTGTGGCCCTGGATACGGAGATCCCTAATGA

GCTTCCATACAATGACTACTTTGAATACTTTGGACCAGATTTCAAGCTCCACATCAGTCCTTCCAATATG

ACTAACCAGAACACGAATGAGTACCTGGAGAAGATCAAACAGCGACTGTTTGAGAACCTTAGAATGCTGC

CGCACGCACCTGGGGTCCAAATGCAGGCGATTCCTGAGGACGCCATCCCTGAGGAGAGTGGCGATGAGGA

CGAAGACGACCCTGACAAGCGCATCTCGATCTGCTCCTCTGACAAACGAATTGCCTGTGAGGAAGAGTTC

TCCGATTCTGAAGAGGAGGGAGAGGGGGGCCGCAAGAACTCTTCCAACTTCAAAAAAGCCAAGAGAGTCA

AAACAGAGGATGAAAAAGAGAAAGACCCAGAGGAGAAGAAAGAAGTCACCGAAGAGGAGAAAACCAAGGA

GGAGAAGCCAGAAGCCAAAGGGGTCAAGGAGGAGGTCAAGTTGGCCTGA

>lcl|NM_006044.4_cds_NP_006035.2_1 [gene=HDAC6] [db_xref=CCDS:CCDS14306.1] [protein=histone deacetylase 6 isoform b] [protein_id=NP_006035.2] [location=107..3754] [gbkey=CDS]

ATGACCTCAACCGGCCAGGATTCCACCACAACCAGGCAGCGAAGAAGTAGGCAGAACCCCCAGTCGCCCC

CTCAGGACTCCAGTGTCACTTCGAAGCGAAATATTAAAAAGGGAGCCGTTCCCCGCTCTATCCCCAATCT

AGCGGAGGTAAAGAAGAAAGGCAAAATGAAGAAGCTCGGCCAAGCAATGGAAGAAGACCTAATCGTGGGA

CTGCAAGGGATGGATCTGAACCTTGAGGCTGAAGCACTGGCTGGCACTGGCTTGGTGTTGGATGAGCAGT

TAAATGAATTCCATTGCCTCTGGGATGACAGCTTCCCGGAAGGCCCTGAGCGGCTCCATGCCATCAAGGA

GCAACTGATCCAGGAGGGCCTCCTAGATCGCTGCGTGTCCTTTCAGGCCCGGTTTGCTGAAAAGGAAGAG

CTGATGTTGGTTCACAGCCTAGAATATATTGATCTGATGGAAACAACCCAGTACATGAATGAGGGAGAAC

TCCGTGTCCTAGCAGACACCTACGACTCAGTTTATCTGCATCCGAACTCATACTCCTGTGCCTGCCTGGC

CTCAGGCTCTGTCCTCAGGCTGGTGGATGCGGTCCTGGGGGCTGAGATCCGGAATGGCATGGCCATCATT

AGGCCTCCTGGACATCACGCCCAGCACAGTCTTATGGATGGCTATTGCATGTTCAACCACGTGGCTGTGG

CAGCCCGCTATGCTCAACAGAAACACCGCATCCGGAGGGTCCTTATCGTAGATTGGGATGTGCACCACGG

TCAAGGAACACAGTTCACCTTCGACCAGGACCCCAGTGTCCTCTATTTCTCCATCCACCGCTACGAGCAG

GGTAGGTTCTGGCCCCACCTGAAGGCCTCTAACTGGTCCACCACAGGTTTCGGCCAAGGCCAAGGATATA

CCATCAATGTGCCTTGGAACCAGGTGGGGATGCGGGATGCTGACTACATTGCTGCTTTCCTGCACGTCCT

GCTGCCAGTCGCCCTCGAGTTCCAGCCTCAGCTGGTCCTGGTGGCTGCTGGATTTGATGCCCTGCAAGGG

GACCCCAAGGGTGAGATGGCCGCCACTCCGGCAGGGTTCGCCCAGCTAACCCACCTGCTCATGGGTCTGG

CAGGAGGCAAGCTGATCCTGTCTCTGGAGGGTGGCTACAACCTCCGCGCCCTGGCTGAAGGCGTCAGTGC

TTCGCTCCACACCCTTCTGGGAGACCCTTGCCCCATGCTGGAGTCACCTGGTGCCCCCTGCCGGAGTGCC

CAGGCTTCAGTTTCCTGTGCTCTGGAAGCCCTTGAGCCCTTCTGGGAGGTTCTTGTGAGATCAACTGAGA

CCGTGGAGAGGGACAACATGGAGGAGGACAATGTAGAGGAGAGCGAGGAGGAAGGACCCTGGGAGCCCCC

TGTGCTCCCAATCCTGACATGGCCAGTGCTACAGTCTCGCACAGGGCTGGTCTATGACCAAAATATGATG

AATCACTGCAACTTGTGGGACAGCCACCACCCTGAGGTACCCCAGCGCATCTTGCGGATCATGTGCCGTC

TGGAGGAGCTGGGCCTTGCCGGGCGCTGCCTCACCCTGACACCGCGCCCTGCCACAGAGGCTGAGCTGCT

CACCTGTCACAGTGCTGAGTACGTGGGTCATCTCCGGGCCACAGAGAAAATGAAAACCCGGGAGCTGCAC

CGTGAGAGTTCCAACTTTGACTCCATCTATATCTGCCCCAGTACCTTCGCCTGTGCACAGCTTGCCACTG

GCGCTGCCTGCCGCCTGGTGGAGGCTGTGCTCTCAGGAGAGGTTCTGAATGGTGCTGCTGTGGTGCGTCC

CCCAGGACACCACGCAGAGCAGGATGCAGCTTGCGGTTTTTGCTTTTTCAACTCTGTGGCTGTGGCTGCT

CGCCATGCCCAGACTATCAGTGGGCATGCCCTACGGATCCTGATTGTGGATTGGGATGTCCACCACGGTA

ATGGAACTCAGCACATGTTTGAGGATGACCCCAGTGTGCTATATGTGTCCCTGCACCGCTATGATCATGG

CACCTTCTTCCCCATGGGGGATGAGGGTGCCAGCAGCCAGATCGGCCGGGCTGCGGGCACAGGCTTCACC

GTCAACGTGGCATGGAACGGGCCCCGCATGGGTGATGCTGACTACCTAGCTGCCTGGCATCGCCTGGTGC

TTCCCATTGCCTACGAGTTTAACCCAGAACTGGTGCTGGTCTCAGCTGGCTTTGATGCTGCACGGGGGGA

TCCGCTGGGGGGCTGCCAGGTGTCACCTGAGGGTTATGCCCACCTCACCCACCTGCTGATGGGCCTTGCC

AGTGGCCGCATTATCCTTATCCTAGAGGGTGGCTATAACCTGACATCCATCTCAGAGTCCATGGCTGCCT

GCACTCGCTCCCTCCTTGGAGACCCACCACCCCTGCTGACCCTGCCACGGCCCCCACTATCAGGGGCCCT

GGCCTCAATCACTGAGACCATCCAAGTCCATCGCAGATACTGGCGCAGCTTACGGGTCATGAAGGTAGAA

GACAGAGAAGGACCCTCCAGTTCTAAGTTGGTCACCAAGAAGGCACCCCAACCAGCCAAACCTAGGTTAG

CTGAGCGGATGACCACACGAGAAAAGAAGGTTCTGGAAGCAGGCATGGGGAAAGTCACCTCGGCATCATT

TGGGGAAGAGTCCACTCCAGGCCAGACTAACTCAGAGACAGCTGTGGTGGCCCTCACTCAGGACCAGCCC

TCAGAGGCAGCCACAGGGGGAGCCACTCTGGCCCAGACCATTTCTGAGGCAGCCATTGGGGGAGCCATGC

TGGGCCAGACCACCTCAGAGGAGGCTGTCGGGGGAGCCACTCCGGACCAGACCACCTCAGAGGAGACTGT

GGGAGGAGCCATTCTGGACCAGACCACCTCAGAGGATGCTGTTGGGGGAGCCACGCTGGGCCAGACTACC

TCAGAGGAGGCTGTAGGAGGAGCTACACTGGCCCAGACCACCTCGGAGGCAGCCATGGAGGGAGCCACAC

TGGACCAGACTACGTCAGAGGAGGCTCCAGGGGGCACCGAGCTGATCCAAACTCCTCTAGCCTCGAGCAC

AGACCACCAGACCCCCCCAACCTCACCTGTGCAGGGAACTACACCCCAGATATCTCCCAGTACACTGATT

GGGAGTCTCAGGACCTTGGAGCTAGGCAGCGAATCTCAGGGGGCCTCAGAATCTCAGGCCCCAGGAGAGG

AGAACCTACTAGGAGAGGCAGCTGGAGGTCAGGACATGGCTGATTCGATGCTGATGCAGGGATCTAGGGG

CCTCACTGATCAGGCCATATTTTATGCTGTGACACCACTGCCCTGGTGTCCCCATTTGGTGGCAGTATGC

CCCATACCTGCAGCAGGCCTAGACGTGACCCAACCTTGTGGGGACTGTGGAACAATCCAAGAGAATTGGG

TGTGTCTCTCTTGCTATCAGGTCTACTGTGGTCGTTACATCAATGGCCACATGCTCCAACACCATGGAAA

TTCTGGACACCCGCTGGTCCTCAGCTACATCGACCTGTCAGCCTGGTGTTACTACTGTCAGGCCTATGTC

CACCACCAGGCTCTCCTAGATGTGAAGAACATCGCCCACCAGAACAAGTTTGGGGAGGATATGCCCCACC

CACACTAA

>lcl|NM_004712.5_cds_NP_004703.1_1 [gene=HGS] [db_xref=CCDS:CCDS11784.1] [protein=hepatocyte growth factor-regulated tyrosine kinase substrate] [protein_id=NP_004703.1] [location=57..2390] [gbkey=CDS]

ATGGGGCGAGGCAGCGGCACCTTCGAGCGTCTCCTAGACAAGGCGACCAGCCAGCTCCTGTTGGAGACAG

ATTGGGAGTCCATTTTGCAGATCTGCGACCTGATCCGCCAAGGGGACACACAAGCAAAATATGCTGTGAA

TTCCATCAAGAAGAAAGTCAACGACAAGAACCCACACGTCGCCTTGTATGCCCTGGAGGTCATGGAATCT

GTGGTAAAGAACTGTGGCCAGACAGTTCATGATGAGGTGGCCAACAAGCAGACCATGGAGGAGCTGAAGG

ACCTGCTGAAGAGACAAGTGGAGGTAAACGTCCGTAACAAGATCCTGTACCTGATCCAGGCCTGGGCGCA

TGCCTTCCGGAACGAGCCCAAGTACAAGGTGGTCCAGGACACCTACCAGATCATGAAGGTGGAGGGGCAC

GTCTTTCCAGAATTCAAAGAGAGCGATGCCATGTTTGCTGCCGAGAGAGCCCCAGACTGGGTGGACGCTG

AGGAATGCCACCGCTGCAGGGTGCAGTTCGGGGTGATGACCCGTAAGCACCACTGCCGGGCGTGTGGGCA

GATATTCTGTGGAAAGTGTTCTTCCAAGTACTCCACCATCCCCAAGTTTGGCATCGAGAAGGAGGTGCGC

GTGTGTGAGCCCTGCTACGAGCAGCTGAACAGGAAAGCGGAGGGAAAGGCCACTTCCACCACTGAGCTGC

CCCCCGAGTACCTGACCAGCCCCCTGTCTCAGCAGTCCCAGCTGCCCCCCAAGAGGGACGAGACGGCCCT

GCAGGAGGAGGAGGAGCTGCAGCTGGCCCTGGCGCTGTCACAGTCAGAGGCGGAGGAGAAGGAGAGGCTG

AGACAGAAGTCCACGTACACTTCGTACCCCAAGGCGGAGCCCATGCCCTCGGCCTCCTCAGCGCCCCCCG

CCAGCAGCCTGTACTCTTCACCTGTGAACTCGTCGGCGCCTCTGGCTGAGGACATCGACCCTGAGCTCGC

ACGGTATCTCAACCGGAACTACTGGGAGAAGAAGCAGGAGGAGGCTCGCAAGAGCCCCACGCCATCTGCG

CCCGTGCCCCTGACGGAGCCGGCTGCACAGCCTGGGGAAGGGCACGCAGCCCCCACCAACGTGGTGGAGA

ACCCCCTCCCGGAGACAGACTCTCAGCCCATTCCTCCCTCTGGTGGCCCCTTTAGTGAGCCACAGTTCCA

CAATGGCGAGTCTGAGGAGAGCCACGAGCAGTTCCTGAAGGCGCTGCAGAACGCCGTCACCACCTTCGTG

AACCGCATGAAGAGTAACCACATGCGGGGCCGCAGCATCACCAATGACTCGGCCGTGCTCTCACTCTTCC

AGTCCATCAACGGCATGCACCCGCAGCTGCTGGAGCTGCTCAACCAGCTGGACGAGCGCAGGCTGTACTA

TGAGGGGCTGCAGGACAAGCTGGCACAGATCCGCGATGCCCGGGGGGCGCTGAGTGCCCTGCGCGAAGAG

CACCGGGAGAAGCTTCGCCGGGCAGCCGAGGAGGCAGAGCGCCAGCGCCAGATCCAGCTGGCCCAGAAGC

TGGAGATAATGCGGCAGAAGAAGCAGGAGTACCTGGAGGTGCAGAGGCAGCTGGCCATCCAGCGCCTGCA

GGAGCAGGAGAAGGAGCGGCAGATGCGGCTGGAGCAGCAGAAGCAGACGGTCCAGATGCGCGCGCAGATG

CCCGCCTTCCCCCTGCCCTACGCCCAGCTCCAGGCCATGCCCGCAGCCGGAGGTGTGCTCTACCAGCCCT

CGGGACCAGCCAGCTTCCCCAGCACCTTCAGCCCTGCCGGCTCGGTGGAGGGCTCCCCAATGCACGGCGT

GTACATGAGCCAGCCGGCCCCTGCCGCTGGCCCCTACCCCAGCATGCCCAGCACTGCGGCTGATCCCAGC

ATGGTGAGTGCCTACATGTACCCAGCAGGGGCCACTGGGGCGCAGGCGGCCCCCCAGGCCCAGGCCGGAC

CCACCGCCAGCCCCGCTTACTCATCCTACCAGCCTACTCCCACAGCGGGCTACCAGAACGTGGCCTCCCA

GGCCCCACAGAGCCTCCCGGCCATCTCTCAGCCTCCGCAGTCCAGCACCATGGGCTACATGGGGAGCCAG

TCAGTCTCCATGGGCTACCAGCCTTACAACATGCAGAATCTCATGACCACCCTCCCAAGCCAGGATGCGT

CTCTGCCACCCCAGCAGCCCTACATCGCGGGGCAGCAGCCCATGTACCAGCAGATGGCACCCTCTGGCGG

TCCCCCCCAGCAGCAGCCCCCCGTGGCCCAGCAACCGCAGGCACAGGGGCCGCCGGCACAGGGCAGCGAG

GCCCAGCTCATTTCATTCGACTGA

>lcl|NM_001530.4_cds_NP_001521.1_1 [gene=HIF1A] [db_xref=CCDS:CCDS9753.1] [protein=hypoxia-inducible factor 1-alpha isoform 1] [protein_id=NP_001521.1] [location=293..2773] [gbkey=CDS]

ATGGAGGGCGCCGGCGGCGCGAACGACAAGAAAAAGATAAGTTCTGAACGTCGAAAAGAAAAGTCTCGAG

ATGCAGCCAGATCTCGGCGAAGTAAAGAATCTGAAGTTTTTTATGAGCTTGCTCATCAGTTGCCACTTCC

ACATAATGTGAGTTCGCATCTTGATAAGGCCTCTGTGATGAGGCTTACCATCAGCTATTTGCGTGTGAGG

AAACTTCTGGATGCTGGTGATTTGGATATTGAAGATGACATGAAAGCACAGATGAATTGCTTTTATTTGA

AAGCCTTGGATGGTTTTGTTATGGTTCTCACAGATGATGGTGACATGATTTACATTTCTGATAATGTGAA

CAAATACATGGGATTAACTCAGTTTGAACTAACTGGACACAGTGTGTTTGATTTTACTCATCCATGTGAC

CATGAGGAAATGAGAGAAATGCTTACACACAGAAATGGCCTTGTGAAAAAGGGTAAAGAACAAAACACAC

AGCGAAGCTTTTTTCTCAGAATGAAGTGTACCCTAACTAGCCGAGGAAGAACTATGAACATAAAGTCTGC

AACATGGAAGGTATTGCACTGCACAGGCCACATTCACGTATATGATACCAACAGTAACCAACCTCAGTGT

GGGTATAAGAAACCACCTATGACCTGCTTGGTGCTGATTTGTGAACCCATTCCTCACCCATCAAATATTG

AAATTCCTTTAGATAGCAAGACTTTCCTCAGTCGACACAGCCTGGATATGAAATTTTCTTATTGTGATGA

AAGAATTACCGAATTGATGGGATATGAGCCAGAAGAACTTTTAGGCCGCTCAATTTATGAATATTATCAT

GCTTTGGACTCTGATCATCTGACCAAAACTCATCATGATATGTTTACTAAAGGACAAGTCACCACAGGAC

AGTACAGGATGCTTGCCAAAAGAGGTGGATATGTCTGGGTTGAAACTCAAGCAACTGTCATATATAACAC

CAAGAATTCTCAACCACAGTGCATTGTATGTGTGAATTACGTTGTGAGTGGTATTATTCAGCACGACTTG

ATTTTCTCCCTTCAACAAACAGAATGTGTCCTTAAACCGGTTGAATCTTCAGATATGAAAATGACTCAGC

TATTCACCAAAGTTGAATCAGAAGATACAAGTAGCCTCTTTGACAAACTTAAGAAGGAACCTGATGCTTT

AACTTTGCTGGCCCCAGCCGCTGGAGACACAATCATATCTTTAGATTTTGGCAGCAACGACACAGAAACT

GATGACCAGCAACTTGAGGAAGTACCATTATATAATGATGTAATGCTCCCCTCACCCAACGAAAAATTAC

AGAATATAAATTTGGCAATGTCTCCATTACCCACCGCTGAAACGCCAAAGCCACTTCGAAGTAGTGCTGA

CCCTGCACTCAATCAAGAAGTTGCATTAAAATTAGAACCAAATCCAGAGTCACTGGAACTTTCTTTTACC

ATGCCCCAGATTCAGGATCAGACACCTAGTCCTTCCGATGGAAGCACTAGACAAAGTTCACCTGAGCCTA

ATAGTCCCAGTGAATATTGTTTTTATGTGGATAGTGATATGGTCAATGAATTCAAGTTGGAATTGGTAGA

AAAACTTTTTGCTGAAGACACAGAAGCAAAGAACCCATTTTCTACTCAGGACACAGATTTAGACTTGGAG

ATGTTAGCTCCCTATATCCCAATGGATGATGACTTCCAGTTACGTTCCTTCGATCAGTTGTCACCATTAG

AAAGCAGTTCCGCAAGCCCTGAAAGCGCAAGTCCTCAAAGCACAGTTACAGTATTCCAGCAGACTCAAAT

ACAAGAACCTACTGCTAATGCCACCACTACCACTGCCACCACTGATGAATTAAAAACAGTGACAAAAGAC

CGTATGGAAGACATTAAAATATTGATTGCATCTCCATCTCCTACCCACATACATAAAGAAACTACTAGTG

CCACATCATCACCATATAGAGATACTCAAAGTCGGACAGCCTCACCAAACAGAGCAGGAAAAGGAGTCAT

AGAACAGACAGAAAAATCTCATCCAAGAAGCCCTAACGTGTTATCTGTCGCTTTGAGTCAAAGAACTACA

GTTCCTGAGGAAGAACTAAATCCAAAGATACTAGCTTTGCAGAATGCTCAGAGAAAGCGAAAAATGGAAC

ATGATGGTTCACTTTTTCAAGCAGTAGGAATTGGAACATTATTACAGCAGCCAGACGATCATGCAGCTAC

TACATCACTTTCTTGGAAACGTGTAAAAGGATGCAAATCTAGTGAACAGAATGGAATGGAGCAAAAGACA

ATTATTTTAATACCCTCTGATTTAGCATGTAGACTGCTGGGGCAATCAATGGATGAAAGTGGATTACCAC

AGCTGACCAGTTATGATTGTGAAGTTAATGCTCCTATACAAGGCAGCAGAAACCTACTGCAGGGTGAAGA

ATTACTCAGAGCTTTGGATCAAGTTAACTGA

>lcl|NM_007355.4_cds_NP_031381.2_1 [gene=HSP90AB1] [db_xref=CCDS:CCDS4909.1] [protein=heat shock protein HSP 90-beta isoform a] [protein_id=NP_031381.2] [location=96..2270] [gbkey=CDS]

ATGCCTGAGGAAGTGCACCATGGAGAGGAGGAGGTGGAGACTTTTGCCTTTCAGGCAGAAATTGCCCAAC

TCATGTCCCTCATCATCAATACCTTCTATTCCAACAAGGAGATTTTCCTTCGGGAGTTGATCTCTAATGC

TTCTGATGCCTTGGACAAGATTCGCTATGAGAGCCTGACAGACCCTTCGAAGTTGGACAGTGGTAAAGAG

CTGAAAATTGACATCATCCCCAACCCTCAGGAACGTACCCTGACTTTGGTAGACACAGGCATTGGCATGA

CCAAAGCTGATCTCATAAATAATTTGGGAACCATTGCCAAGTCTGGTACTAAAGCATTCATGGAGGCTCT

TCAGGCTGGTGCAGACATCTCCATGATTGGGCAGTTTGGTGTTGGCTTTTATTCTGCCTACTTGGTGGCA

GAGAAAGTGGTTGTGATCACAAAGCACAACGATGATGAACAGTATGCTTGGGAGTCTTCTGCTGGAGGTT

CCTTCACTGTGCGTGCTGACCATGGTGAGCCCATTGGCAGGGGTACCAAAGTGATCCTCCATCTTAAAGA

AGATCAGACAGAGTACCTAGAAGAGAGGCGGGTCAAAGAAGTAGTGAAGAAGCATTCTCAGTTCATAGGC

TATCCCATCACCCTTTATTTGGAGAAGGAACGAGAGAAGGAAATTAGTGATGATGAGGCAGAGGAAGAGA

AAGGTGAGAAAGAAGAGGAAGATAAAGATGATGAAGAAAAACCCAAGATCGAAGATGTGGGTTCAGATGA

GGAGGATGACAGCGGTAAGGATAAGAAGAAGAAAACTAAGAAGATCAAAGAGAAATACATTGATCAGGAA

GAACTAAACAAGACCAAGCCTATTTGGACCAGAAACCCTGATGACATCACCCAAGAGGAGTATGGAGAAT

TCTACAAGAGCCTCACTAATGACTGGGAAGACCACTTGGCAGTCAAGCACTTTTCTGTAGAAGGTCAGTT

GGAATTCAGGGCATTGCTATTTATTCCTCGTCGGGCTCCCTTTGACCTTTTTGAGAACAAGAAGAAAAAG

AACAACATCAAACTCTATGTCCGCCGTGTGTTCATCATGGACAGCTGTGATGAGTTGATACCAGAGTATC

TCAATTTTATCCGTGGTGTGGTTGACTCTGAGGATCTGCCCCTGAACATCTCCCGAGAAATGCTCCAGCA

GAGCAAAATCTTGAAAGTCATTCGCAAAAACATTGTTAAGAAGTGCCTTGAGCTCTTCTCTGAGCTGGCA

GAAGACAAGGAGAATTACAAGAAATTCTATGAGGCATTCTCTAAAAATCTCAAGCTTGGAATCCACGAAG

ACTCCACTAACCGCCGCCGCCTGTCTGAGCTGCTGCGCTATCATACCTCCCAGTCTGGAGATGAGATGAC

ATCTCTGTCAGAGTATGTTTCTCGCATGAAGGAGACACAGAAGTCCATCTATTACATCACTGGTGAGAGC

AAAGAGCAGGTGGCCAACTCAGCTTTTGTGGAGCGAGTGCGGAAACGGGGCTTCGAGGTGGTATATATGA

CCGAGCCCATTGACGAGTACTGTGTGCAGCAGCTCAAGGAATTTGATGGGAAGAGCCTGGTCTCAGTTAC

CAAGGAGGGTCTGGAGCTGCCTGAGGATGAGGAGGAGAAGAAGAAGATGGAAGAGAGCAAGGCAAAGTTT

GAGAACCTCTGCAAGCTCATGAAAGAAATCTTAGATAAGAAGGTTGAGAAGGTGACAATCTCCAATAGAC

TTGTGTCTTCACCTTGCTGCATTGTGACCAGCACCTACGGCTGGACAGCCAATATGGAGCGGATCATGAA

AGCCCAGGCACTTCGGGACAACTCCACCATGGGCTATATGATGGCCAAAAAGCACCTGGAGATCAACCCT

GACCACCCCATTGTGGAGACGCTGCGGCAGAAGGCTGAGGCCGACAAGAATGATAAGGCAGTTAAGGACC

TGGTGGTGCTGCTGTTTGAAACCGCCCTGCTATCTTCTGGCTTTTCCCTTGAGGATCCCCAGACCCACTC

CAACCGCATCTATCGCATGATCAAGCTAGGTCTAGGTATTGATGAAGATGAAGTGGCAGCAGAGGAACCC

AATGCTGCAGTTCCTGATGAGATCCCCCCTCTCGAGGGCGATGAGGATGCGTCTCGCATGGAAGAAGTCG

ATTAG

>lcl|NM_005347.5_cds_NP_005338.1_1 [gene=HSPA5] [db_xref=CCDS:CCDS6863.1] [protein=endoplasmic reticulum chaperone BiP precursor] [protein_id=NP_005338.1] [location=218..2182] [gbkey=CDS]

ATGAAGCTCTCCCTGGTGGCCGCGATGCTGCTGCTGCTCAGCGCGGCGCGGGCCGAGGAGGAGGACAAGA

AGGAGGACGTGGGCACGGTGGTCGGCATCGACCTGGGGACCACCTACTCCTGCGTCGGCGTGTTCAAGAA

CGGCCGCGTGGAGATCATCGCCAACGATCAGGGCAACCGCATCACGCCGTCCTATGTCGCCTTCACTCCT

GAAGGGGAACGTCTGATTGGCGATGCCGCCAAGAACCAGCTCACCTCCAACCCCGAGAACACGGTCTTTG

ACGCCAAGCGGCTCATCGGCCGCACGTGGAATGACCCGTCTGTGCAGCAGGACATCAAGTTCTTGCCGTT

CAAGGTGGTTGAAAAGAAAACTAAACCATACATTCAAGTTGATATTGGAGGTGGGCAAACAAAGACATTT

GCTCCTGAAGAAATTTCTGCCATGGTTCTCACTAAAATGAAAGAAACCGCTGAGGCTTATTTGGGAAAGA

AGGTTACCCATGCAGTTGTTACTGTACCAGCCTATTTTAATGATGCCCAACGCCAAGCAACCAAAGACGC

TGGAACTATTGCTGGCCTAAATGTTATGAGGATCATCAACGAGCCTACGGCAGCTGCTATTGCTTATGGC

CTGGATAAGAGGGAGGGGGAGAAGAACATCCTGGTGTTTGACCTGGGTGGCGGAACCTTCGATGTGTCTC

TTCTCACCATTGACAATGGTGTCTTCGAAGTTGTGGCCACTAATGGAGATACTCATCTGGGTGGAGAAGA

CTTTGACCAGCGTGTCATGGAACACTTCATCAAACTGTACAAAAAGAAGACGGGCAAAGATGTCAGGAAA

GACAATAGAGCTGTGCAGAAACTCCGGCGCGAGGTAGAAAAGGCCAAACGGGCCCTGTCTTCTCAGCATC

AAGCAAGAATTGAAATTGAGTCCTTCTATGAAGGAGAAGACTTTTCTGAGACCCTGACTCGGGCCAAATT

TGAAGAGCTCAACATGGATCTGTTCCGGTCTACTATGAAGCCCGTCCAGAAAGTGTTGGAAGATTCTGAT

TTGAAGAAGTCTGATATTGATGAAATTGTTCTTGTTGGTGGCTCGACTCGAATTCCAAAGATTCAGCAAC

TGGTTAAAGAGTTCTTCAATGGCAAGGAACCATCCCGTGGCATAAACCCAGATGAAGCTGTAGCGTATGG

TGCTGCTGTCCAGGCTGGTGTGCTCTCTGGTGATCAAGATACAGGTGACCTGGTACTGCTTGATGTATGT

CCCCTTACACTTGGTATTGAAACTGTGGGAGGTGTCATGACCAAACTGATTCCAAGGAACACAGTGGTGC

CTACCAAGAAGTCTCAGATCTTTTCTACAGCTTCTGATAATCAACCAACTGTTACAATCAAGGTCTATGA

AGGTGAAAGACCCCTGACAAAAGACAATCATCTTCTGGGTACATTTGATCTGACTGGAATTCCTCCTGCT

CCTCGTGGGGTCCCACAGATTGAAGTCACCTTTGAGATAGATGTGAATGGTATTCTTCGAGTGACAGCTG

AAGACAAGGGTACAGGGAACAAAAATAAGATCACAATCACCAATGACCAGAATCGCCTGACACCTGAAGA

AATCGAAAGGATGGTTAATGATGCTGAGAAGTTTGCTGAGGAAGACAAAAAGCTCAAGGAGCGCATTGAT

ACTAGAAATGAGTTGGAAAGCTATGCCTATTCTCTAAAGAATCAGATTGGAGATAAAGAAAAGCTGGGAG

GTAAACTTTCCTCTGAAGATAAGGAGACCATGGAAAAAGCTGTAGAAGAAAAGATTGAATGGCTGGAAAG

CCACCAAGATGCTGACATTGAAGACTTCAAAGCTAAGAAGAAGGAACTGGAAGAAATTGTTCAACCAATT

ATCAGCAAACTCTATGGAAGTGCAGGCCCTCCCCCAACTGGTGAAGAGGATACAGCAGAAAAAGATGAGT

TGTAG

>lcl|NM_006597.6_cds_NP_006588.1_1 [gene=HSPA8] [db_xref=CCDS:CCDS8440.1] [protein=heat shock cognate 71 kDa protein isoform 1] [protein_id=NP_006588.1] [location=79..2019] [gbkey=CDS]

ATGTCCAAGGGACCTGCAGTTGGTATTGATCTTGGCACCACCTACTCTTGTGTGGGTGTTTTCCAGCACG

GAAAAGTCGAGATAATTGCCAATGATCAGGGAAACCGAACCACTCCAAGCTATGTCGCCTTTACGGACAC

TGAACGGTTGATCGGTGATGCCGCAAAGAATCAAGTTGCAATGAACCCCACCAACACAGTTTTTGATGCC

AAACGTCTGATTGGACGCAGATTTGATGATGCTGTTGTCCAGTCTGATATGAAACATTGGCCCTTTATGG

TGGTGAATGATGCTGGCAGGCCCAAGGTCCAAGTAGAATACAAGGGAGAGACCAAAAGCTTCTATCCAGA

GGAGGTGTCTTCTATGGTTCTGACAAAGATGAAGGAAATTGCAGAAGCCTACCTTGGGAAGACTGTTACC

AATGCTGTGGTCACAGTGCCAGCTTACTTTAATGACTCTCAGCGTCAGGCTACCAAAGATGCTGGAACTA

TTGCTGGTCTCAATGTACTTAGAATTATTAATGAGCCAACTGCTGCTGCTATTGCTTACGGCTTAGACAA

AAAGGTTGGAGCAGAAAGAAACGTGCTCATCTTTGACCTGGGAGGTGGCACTTTTGATGTGTCAATCCTC

ACTATTGAGGATGGAATCTTTGAGGTCAAGTCTACAGCTGGAGACACCCACTTGGGTGGAGAAGATTTTG

ACAACCGAATGGTCAACCATTTTATTGCTGAGTTTAAGCGCAAGCATAAGAAGGACATCAGTGAGAACAA

GAGAGCTGTAAGACGCCTCCGTACTGCTTGTGAACGTGCTAAGCGTACCCTCTCTTCCAGCACCCAGGCC

AGTATTGAGATCGATTCTCTCTATGAAGGAATCGACTTCTATACCTCCATTACCCGTGCCCGATTTGAAG

AACTGAATGCTGACCTGTTCCGTGGCACCCTGGACCCAGTAGAGAAAGCCCTTCGAGATGCCAAACTAGA

CAAGTCACAGATTCATGATATTGTCCTGGTTGGTGGTTCTACTCGTATCCCCAAGATTCAGAAGCTTCTC

CAAGACTTCTTCAATGGAAAAGAACTGAATAAGAGCATCAACCCTGATGAAGCTGTTGCTTATGGTGCAG

CTGTCCAGGCAGCCATCTTGTCTGGAGACAAGTCTGAGAATGTTCAAGATTTGCTGCTCTTGGATGTCAC

TCCTCTTTCCCTTGGTATTGAAACTGCTGGTGGAGTCATGACTGTCCTCATCAAGCGTAATACCACCATT

CCTACCAAGCAGACACAGACCTTCACTACCTATTCTGACAACCAGCCTGGTGTGCTTATTCAGGTTTATG

AAGGCGAGCGTGCCATGACAAAGGATAACAACCTGCTTGGCAAGTTTGAACTCACAGGCATACCTCCTGC

ACCCCGAGGTGTTCCTCAGATTGAAGTCACTTTTGACATTGATGCCAATGGTATACTCAATGTCTCTGCT

GTGGACAAGAGTACGGGAAAAGAGAACAAGATTACTATCACTAATGACAAGGGCCGTTTGAGCAAGGAAG

ACATTGAACGTATGGTCCAGGAAGCTGAGAAGTACAAAGCTGAAGATGAGAAGCAGAGGGACAAGGTGTC

ATCCAAGAATTCACTTGAGTCCTATGCCTTCAACATGAAAGCAACTGTTGAAGATGAGAAACTTCAAGGC

AAGATTAACGATGAGGACAAACAGAAGATTCTGGACAAGTGTAATGAAATTATCAACTGGCTTGATAAGA

ATCAGACTGCTGAGAAGGAAGAATTTGAACATCAACAGAAAGAGCTGGAGAAAGTTTGCAACCCCATCAT

CACCAAGCTGTACCAGAGTGCAGGAGGCATGCCAGGAGGAATGCCTGGGGGATTTCCTGGTGGTGGAGCT

CCTCCCTCTGGTGGTGCTTCCTCAGGGCCCACCATTGAAGAGGTTGATTAA

>lcl|NM_014365.3_cds_NP_055180.1_1 [gene=HSPB8] [db_xref=CCDS:CCDS9189.1] [protein=heat shock protein beta-8] [protein_id=NP_055180.1] [location=383..973] [gbkey=CDS]

ATGGCTGACGGTCAGATGCCCTTCTCCTGCCACTACCCAAGCCGCCTGCGCCGAGACCCCTTCCGGGACT

CTCCCCTCTCCTCTCGCCTGCTGGATGATGGCTTTGGCATGGACCCCTTCCCAGACGACTTGACAGCCTC

TTGGCCCGACTGGGCTCTGCCTCGTCTCTCCTCCGCCTGGCCAGGCACCCTAAGGTCGGGCATGGTGCCC

CGGGGCCCCACTGCCACCGCCAGGTTTGGGGTGCCTGCCGAGGGCAGGACCCCCCCACCCTTCCCTGGGG

AGCCCTGGAAAGTGTGTGTGAATGTGCACAGCTTCAAGCCAGAGGAGTTGATGGTGAAGACCAAAGATGG

ATACGTGGAGGTGTCTGGCAAACATGAAGAGAAACAGCAAGAAGGTGGCATTGTTTCTAAGAACTTCACA

AAGAAAATCCAGCTTCCTGCAGAGGTGGATCCTGTGACAGTATTTGCCTCACTTTCCCCAGAGGGTCTGC

TGATCATCGAAGCTCCCCAGGTCCCTCCTTACTCAACATTTGGAGAGAGCAGTTTCAACAACGAGCTTCC

CCAGGACAGCCAGGAAGTCACCTGTACCTGA

>lcl|NM_000619.3_cds_NP_000610.2_1 [gene=IFNG] [db_xref=CCDS:CCDS8980.1] [protein=interferon gamma precursor] [protein_id=NP_000610.2] [location=126..626] [gbkey=CDS]

ATGAAATATACAAGTTATATCTTGGCTTTTCAGCTCTGCATCGTTTTGGGTTCTCTTGGCTGTTACTGCC

AGGACCCATATGTAAAAGAAGCAGAAAACCTTAAGAAATATTTTAATGCAGGTCATTCAGATGTAGCGGA

TAATGGAACTCTTTTCTTAGGCATTTTGAAGAATTGGAAAGAGGAGAGTGACAGAAAAATAATGCAGAGC

CAAATTGTCTCCTTTTACTTCAAACTTTTTAAAAACTTTAAAGATGACCAGAGCATCCAAAAGAGTGTGG

AGACCATCAAGGAAGACATGAATGTCAAGTTTTTCAATAGCAACAAAAAGAAACGAGATGACTTCGAAAA

GCTGACTAATTATTCGGTAACTGACTTGAATGTCCAACGCAAAGCAATACATGAACTCATCCAAGTGATG

GCTGAACTGTCGCCAGCAGCTAAAACAGGGAAGCGAAAAAGGAGTCAGATGCTGTTTCGAGGTCGAAGAG

CATCCCAGTAA

>lcl|NM_001190720.3_cds_NP_001177649.2_1 [gene=IKBKB] [db_xref=CCDS:CCDS55228.1] [protein=inhibitor of nuclear factor kappa-B kinase subunit beta isoform 2] [protein_id=NP_001177649.2] [location=256..2334] [gbkey=CDS]

ATGAGAAGGCTGACCCACCCCAATGTGGTGGCTGCCCGAGATGTCCCTGAGGGGATGCAGAACTTGGCGC

CCAATGACCTGCCCCTGCTGGCCATGGAGTACTGCCAAGGAGGAGATCTCCGGAAGTACCTGAACCAGTT

TGAGAACTGCTGTGGTCTGCGGGAAGGTGCCATCCTCACCTTGCTGAGTGACATTGCCTCTGCGCTTAGA

TACCTTCATGAAAACAGAATCATCCATCGGGATCTAAAGCCAGAAAACATCGTCCTGCAGCAAGGAGAAC

AGAGGTTAATACACAAAATTATTGACCTAGGATATGCCAAGGAGCTGGATCAGGGCAGTCTTTGCACATC

ATTCGTGGGGACCCTGCAGTACCTGGCCCCAGAGCTACTGGAGCAGCAGAAGTACACAGTGACCGTCGAC

TACTGGAGCTTCGGCACCCTGGCCTTTGAGTGCATCACGGGCTTCCGGCCCTTCCTCCCCAACTGGCAGC

CCGTGCAGTGGCATTCAAAAGTGCGGCAGAAGAGTGAGGTGGACATTGTTGTTAGCGAAGACTTGAATGG

AACGGTGAAGTTTTCAAGCTCTTTACCCTACCCCAATAATCTTAACAGTGTCCTGGCTGAGCGACTGGAG

AAGTGGCTGCAACTGATGCTGATGTGGCACCCCCGACAGAGGGGCACGGATCCCACGTATGGGCCCAATG

GCTGCTTCAAGGCCCTGGATGACATCTTAAACTTAAAGCTGGTTCATATCTTGAACATGGTCACGGGCAC

CATCCACACCTACCCTGTGACAGAGGATGAGAGTCTGCAGAGCTTGAAGGCCAGAATCCAACAGGACACG

GGCATCCCAGAGGAGGACCAGGAGCTGCTGCAGGAAGCGGGCCTGGCGTTGATCCCCGATAAGCCTGCCA

CTCAGTGTATTTCAGACGGCAAGTTAAATGAGGGCCACACATTGGACATGGATCTTGTTTTTCTCTTTGA

CAACAGTAAAATCACCTATGAGACTCAGATCTCCCCACGGCCCCAACCTGAAAGTGTCAGCTGTATCCTT

CAAGAGCCCAAGAGGAATCTCGCCTTCTTCCAGCTGAGGAAGGTGTGGGGCCAGGTCTGGCACAGCATCC

AGACCCTGAAGGAAGATTGCAACCGGCTGCAGCAGGGACAGCGAGCCGCCATGATGAATCTCCTCCGAAA

CAACAGCTGCCTCTCCAAAATGAAGAATTCCATGGCTTCCATGTCTCAGCAGCTCAAGGCCAAGTTGGAT

TTCTTCAAAACCAGCATCCAGATTGACCTGGAGAAGTACAGCGAGCAAACCGAGTTTGGGATCACATCAG

ATAAACTGCTGCTGGCCTGGAGGGAAATGGAGCAGGCTGTGGAGCTCTGTGGGCGGGAGAACGAAGTGAA

ACTCCTGGTAGAACGGATGATGGCTCTGCAGACCGACATTGTGGACTTACAGAGGAGCCCCATGGGCCGG

AAGCAGGGGGGAACGCTGGACGACCTAGAGGAGCAAGCAAGGGAGCTGTACAGGAGACTAAGGGAAAAAC

CTCGAGACCAGCGAACTGAGGGTGACAGTCAGGAAATGGTACGGCTGCTGCTTCAGGCAATTCAGAGCTT

CGAGAAGAAAGTGCGAGTGATCTATACGCAGCTCAGTAAAACTGTGGTTTGCAAGCAGAAGGCGCTGGAA

CTGTTGCCCAAGGTGGAAGAGGTGGTGAGCTTAATGAATGAGGATGAGAAGACTGTTGTCCGGCTGCAGG

AGAAGCGGCAGAAGGAGCTCTGGAATCTCCTGAAGATTGCTTGTAGCAAGGTCCGTGGTCCTGTCAGTGG

AAGCCCGGATAGCATGAATGCCTCTCGACTTAGCCAGCCTGGGCAGCTGATGTCTCAGCCCTCCACGGCC

TCCAACAGCTTACCTGAGCCAGCCAAGAAGAGTGAAGAACTGGTGGCTGAAGCACATAACCTCTGCACCC

TGCTAGAAAATGCCATACAGGACACTGTGAGGGAACAAGACCAGAGTTTCACGGCCCTAGACTGGAGCTG

GTTACAGACGGAAGAAGAAGAGCACAGCTGCCTGGAGCAGGCCTCATGA

>lcl|NM_001193321.2_cds_NP_001180250.1_1 [gene=IKBKE] [db_xref=CCDS:CCDS53464.1] [protein=inhibitor of nuclear factor kappa-B kinase subunit epsilon isoform 3] [protein_id=NP_001180250.1] [location=482..2377] [gbkey=CDS]

ATGGAGTACTGCTCCAGTGGGAGCCTGCTGAGTGTGCTGGAGAGCCCTGAGAATGCCTTTGGGCTGCCTG

AGGATGAGTTCCTGGTGGTGCTGCGCTGTGTGGTGGCCGGCATGAACCACCTGCGGGAGAACGGCATTGT

GCATCGCGACATCAAGCCGGGGAACATCATGCGCCTCGTAGGGGAGGAGGGGCAGAGCATCTACAAGCTG

ACAGACTTCGGCGCTGCCCGGGAGCTGGATGATGATGAGAAGTTCGTCTCGGTCTATGGGACTGAGGAGT

ACCTGCATCCCGACATGTATGAGCGGGCGGTGCTTCGAAAGCCCCAGCAAAAAGCGTTCGGGGTGACTGT

GGATCTCTGGAGCATTGGAGTGACCTTGTACCATGCAGCCACTGGCAGCCTGCCCTTCATCCCCTTTGGT

GGGCCACGGCGGAACAAGGAGATCATGTACCGGATCACCACGGAGAAGCCGGCTGGGGCCATTGCAGGTG

CCCAGAGGCGGGAGAACGGGCCCCTGGAGTGGAGCTACACCCTCCCCATCACCTGCCAGCTGTCACTGGG

GCTGCAGAGCCAGCTGGTGCCCATCCTGGCCAACATCCTGGAGGTGGAGCAGGCCAAGTGCTGGGGCTTC

GACCAGTTCTTTGCGGAGACCAGTGACATCCTGCAGCGAGTTGTCGTCCATGTCTTCTCCCTGTCCCAGG

CAGTCCTGCACCACATCTATATCCATGCCCACAACACGATAGCCATTTTCCAGGAGGCCGTGCACAAGCA

GACCAGTGTGGCCCCCCGACACCAGGAGTACCTCTTTGAGGGTCACCTCTGTGTCCTCGAGCCCAGCGTC

TCAGCACAGCACATCGCCCACACGACGGCAAGCAGCCCCCTGACCCTCTTCAGCACAGCCATCCCTAAGG

GGCTGGCCTTCAGGGACCCTGCTCTGGACGTCCCCAAGTTCGTCCCCAAAGTGGACCTGCAGGCGGATTA

CAACACTGCCAAGGGCGTGTTGGGCGCCGGCTACCAGGCCCTGCGGCTGGCACGGGCCCTGCTGGATGGG

CAGGAGCTAATGTTTCGGGGGCTGCACTGGGTCATGGAGGTGCTCCAGGCCACATGCAGACGGACTCTGG

AAGTGGCAAGGACATCCCTCCTCTACCTCAGCAGCAGCCTGGGAACTGAGAGGTTCAGCAGCGTGGCTGG

AACGCCTGAGATCCAGGAACTGAAGGCGGCTGCAGAACTGAGGTCCAGGCTGCGGACTCTAGCGGAGGTC

CTCTCCAGATGCTCCCAAAATATCACGGAGACCCAGGAGAGCCTGAGCAGCCTGAACCGGGAGCTGGTGA

AGAGCCGGGATCAGGTACATGAGGACAGAAGCATCCAGCAGATTCAGTGCTGTTTGGACAAGATGAACTT

CATCTACAAACAGTTCAAGAAGTCTAGGATGAGGCCAGGGCTTGGCTACAACGAGGAGCAGATTCACAAG

CTGGATAAGGTGAATTTCAGTCATTTAGCCAAAAGACTCCTGCAGGTGTTCCAGGAGGAGTGCGTGCAGA

AGTATCAAGCGTCCTTAGTCACACACGGCAAGAGGATGAGGGTGGTGCACGAGACCAGGAACCACCTGCG

CCTGGTTGGCTGTTCTGTGGCTGCCTGTAACACAGAAGCCCAGGGGGTCCAGGAGAGTCTCAGCAAGCTC

CTGGAAGAGCTATCTCACCAGCTCCTTCAGGACCGAGCAAAGGGGGCTCAGGCCTCGCCGCCTCCCATAG

CTCCTTACCCCAGCCCTACACGAAAGGACCTGCTTCTCCACATGCAAGAGCTCTGCGAGGGGATGAAGCT

GCTGGCATCTGACCTCCTGGACAACAACCGCATCATCGAACGGCTAAATAGAGTCCCAGCACCTCCTGAT

GTCTGA

>lcl|NM_006850.3_cds_NP_006841.1_1 [gene=IL24] [db_xref=CCDS:CCDS1471.1] [protein=interleukin-24 isoform 1 precursor] [protein_id=NP_006841.1] [location=276..896] [gbkey=CDS]

ATGAATTTTCAACAGAGGCTGCAAAGCCTGTGGACTTTAGCCAGACCCTTCTGCCCTCCTTTGCTGGCGA

CAGCCTCTCAAATGCAGATGGTTGTGCTCCCTTGCCTGGGTTTTACCCTGCTTCTCTGGAGCCAGGTATC

AGGGGCCCAGGGCCAAGAATTCCACTTTGGGCCCTGCCAAGTGAAGGGGGTTGTTCCCCAGAAACTGTGG

GAAGCCTTCTGGGCTGTGAAAGACACTATGCAAGCTCAGGATAACATCACGAGTGCCCGGCTGCTGCAGC

AGGAGGTTCTGCAGAACGTCTCGGATGCTGAGAGCTGTTACCTTGTCCACACCCTGCTGGAGTTCTACTT

GAAAACTGTTTTCAAAAACTACCACAATAGAACAGTTGAAGTCAGGACTCTGAAGTCATTCTCTACTCTG

GCCAACAACTTTGTTCTCATCGTGTCACAACTGCAACCCAGTCAAGAAAATGAGATGTTTTCCATCAGAG

ACAGTGCACACAGGCGGTTTCTGCTATTCCGGAGAGCATTCAAACAGTTGGACGTAGAAGCAGCTCTGAC

CAAAGCCCTTGGGGAAGTGGACATTCTTCTGACCTGGATGCAGAAATTCTACAAGCTCTGA

>lcl|NM_001145805.2_cds_NP_001139277.1_1 [gene=IRGM] [db_xref=CCDS:CCDS47313.1] [protein=immunity-related GTPase family M protein isoform 2] [protein_id=NP_001139277.1] [location=1116..1661] [gbkey=CDS]

ATGGAAGCCATGAATGTTGAGAAAGCCTCAGCAGATGGGAACTTGCCAGAGGTGATCTCTAACATCAAGG

AGACTCTGAAGATAGTGTCCAGGACACCAGTTAACATCACTATGGCAGGGGACTCTGGCAATGGGATGTC

CACCTTCATCAGTGCCCTTCGAAACACAGGACATGAGGGTAAGGCCTCACCTCCTACTGAGCTGGTAAAA

GCTACCCAAAGATGTGCCTCCTATTTCTCTTCCCACTTTTCAAATGTGGTGTTGTGGGACCTGCCTGGCA

CAGGGTCTGCCACCACAACCCTGGAGAACTACCTGATGGAAATGCAGTTCAACCGGTATGACTTCATCAT

GGTTGCATCTGCACAATTCAGCATGAATCATGTGATGCTTGCCAAAACCGCTGAGGACATGGGAAAGAAG

TTCTACATTGTCTGGACCAAGCTAGACATGGACCTCAGCACAGGTGCCCTCCCAGAAGTGCAGCTACTGC

AGATCAGAGAAAATGTCCTGGAAAATCTCCAGAAGGAGCGGGTATGTGAATACTAA

>lcl|NM_002204.4_cds_NP_002195.1_1 [gene=ITGA3] [db_xref=CCDS:CCDS11558.1] [protein=integrin alpha-3 preproprotein] [protein_id=NP_002195.1] [location=331..3486] [gbkey=CDS]

ATGGGCCCCGGCCCCAGCCGCGCGCCCCGCGCCCCACGCCTGATGCTCTGTGCGCTCGCCTTGATGGTGG

CGGCCGGCGGCTGCGTCGTCTCCGCCTTCAACCTGGATACCCGATTCCTGGTAGTGAAGGAGGCCGGGAA

CCCGGGCAGCCTCTTCGGCTACTCGGTCGCCCTCCATCGGCAGACAGAGCGGCAGCAGCGCTACCTGCTC

CTGGCTGGTGCCCCCCGGGAGCTCGCTGTGCCCGATGGCTACACCAACCGGACTGGTGCTGTGTACCTGT

GCCCACTCACTGCCCACAAGGATGACTGTGAGCGGATGAACATCACAGTGAAAAATGACCCTGGCCATCA

CATTATTGAGGACATGTGGCTTGGAGTGACTGTGGCCAGCCAGGGCCCTGCAGGCAGAGTTCTGGTCTGT

GCCCACCGCTACACCCAGGTGCTGTGGTCAGGGTCAGAAGACCAGCGGCGCATGGTGGGCAAGTGCTACG

TGCGAGGCAATGACCTAGAGCTGGACTCCAGTGATGACTGGCAGACCTACCACAACGAGATGTGCAATAG

CAACACAGACTACCTGGAGACGGGCATGTGCCAGCTGGGCACCAGCGGTGGCTTCACCCAGAACACTGTG

TACTTCGGCGCCCCCGGTGCCTACAACTGGAAAGGAAACAGCTACATGATTCAGCGCAAGGAGTGGGACT

TATCTGAGTATAGTTACAAGGACCCAGAGGACCAAGGAAACCTCTATATTGGGTACACGATGCAGGTAGG

CAGCTTCATCCTGCACCCCAAAAACATCACCATTGTGACAGGTGCCCCACGGCACCGACATATGGGCGCG

GTGTTCTTGCTGAGCCAGGAGGCAGGCGGAGACCTGCGGAGGAGGCAGGTGCTGGAGGGCTCGCAGGTGG

GCGCCTATTTTGGCAGCGCCATTGCCCTGGCAGACCTGAACAATGATGGGTGGCAGGACCTCCTGGTGGG

CGCCCCCTACTACTTCGAGAGGAAAGAGGAAGTAGGGGGTGCCATCTATGTCTTCATGAACCAGGCGGGA

ACCTCCTTCCCTGCTCACCCCTCACTCCTTCTTCATGGCCCCAGTGGCTCTGCCTTTGGTTTATCTGTGG

CCAGCATTGGTGACATCAACCAGGATGGATTTCAGGATATTGCTGTGGGAGCTCCGTTTGAAGGCTTGGG

CAAAGTGTACATCTATCACAGTAGCTCTAAGGGGCTCCTTAGACAGCCCCAGCAGGTAATCCATGGAGAG

AAGCTGGGACTGCCTGGGTTGGCCACCTTCGGCTATTCCCTCAGTGGGCAGATGGATGTGGATGAGAACT

TCTACCCAGACCTTCTAGTGGGAAGCCTGTCAGACCACATTGTGCTGCTGCGGGCCCGGCCCGTCATCAA

CATCGTCCACAAGACCTTGGTGCCCAGGCCAGCTGTGCTGGACCCTGCACTTTGCACGGCCACCTCTTGT

GTGCAAGTGGAGCTGTGCTTTGCTTACAACCAGAGTGCCGGGAACCCCAACTACAGGCGAAACATCACCC

TGGCCTACACTCTGGAGGCTGACAGGGACCGCCGGCCGCCCCGGCTCCGCTTTGCCGGCAGTGAGTCCGC

TGTCTTCCACGGCTTCTTCTCCATGCCCGAGATGCGCTGCCAGAAGCTGGAGCTGCTCCTGATGGACAAC

CTCCGTGACAAACTCCGCCCCATCATCATCTCCATGAACTACTCTTTACCTTTGCGGATGCCCGATCGCC

CCCGGCTGGGGCTGCGGTCCCTGGACGCCTACCCGATCCTCAACCAGGCACAGGCTCTGGAGAACCACAC

TGAGGTCCAGTTCCAGAAGGAGTGCGGGCCTGACAACAAGTGTGAGAGCAACTTGCAGATGCGGGCAGCC

TTCGTGTCAGAGCAGCAGCAGAAGCTGAGCAGGCTCCAGTACAGCAGAGACGTCCGGAAATTGCTCCTGA

GCATCAACGTGACGAACACCCGGACCTCGGAGCGCTCCGGGGAGGACGCCCACGAGGCGCTGCTCACCCT

GGTGGTGCCTCCCGCCCTGCTGCTGTCCTCAGTGCGCCCCCCCGGGGCCTGCCAAGCTAATGAGACCATC

TTTTGCGAGCTGGGGAACCCCTTCAAACGGAACCAGAGGATGGAGCTGCTCATCGCCTTTGAGGTCATCG

GGGTGACCCTGCACACAAGGGACCTTCAGGTGCAGCTGCAGCTCTCCACGTCGAGTCACCAGGACAACCT

GTGGCCCATGATCCTCACTCTGCTGGTGGACTATACACTCCAGACCTCGCTTAGCATGGTAAATCACCGG

CTACAAAGCTTCTTTGGGGGGACAGTGATGGGTGAGTCTGGCATGAAAACTGTGGAGGATGTAGGAAGCC

CCCTCAAGTATGAATTCCAGGTGGGCCCAATGGGGGAGGGGCTGGTGGGCCTGGGGACCCTGGTCCTAGG

TCTGGAGTGGCCCTACGAAGTCAGCAATGGCAAGTGGCTGCTGTATCCCACGGAGATCACCGTCCATGGC

AATGGGTCCTGGCCCTGCCGACCACCTGGAGACCTTATCAACCCTCTCAACCTCACTCTTTCTGACCCTG

GGGACAGGCCATCATCCCCACAGCGCAGGCGGCGACAGCTGGATCCAGGGGGAGGCCAGGGCCCCCCACC

TGTCACTCTGGCTGCTGCCAAAAAAGCCAAGTCTGAGACTGTGCTGACCTGTGCCACAGGGCGTGCCCAC

TGTGTGTGGCTAGAGTGCCCCATCCCTGATGCCCCCGTTGTCACCAACGTGACTGTGAAGGCACGAGTGT

GGAACAGCACCTTCATCGAGGATTACAGAGACTTTGACCGAGTCCGGGTAAATGGCTGGGCTACCCTATT

CCTCCGAACCAGCATCCCCACCATCAACATGGAGAACAAGACCACGTGGTTCTCTGTGGACATTGACTCG

GAGCTGGTGGAGGAGCTGCCGGCCGAAATCGAGCTGTGGCTGGTGCTGGTGGCCGTGGGTGCAGGGCTGC

TGCTGCTGGGGCTGATCATCCTCCTGCTGTGGAAGTGCGGCTTCTTCAAGCGAGCCCGCACTCGCGCCCT

GTATGAAGCTAAGAGGCAGAAGGCGGAGATGAAGAGCCAGCCGTCAGAGACAGAGAGGCTGACCGACGAC

TACTGA

>lcl|NM_000210.4_cds_NP_000201.2_1 [gene=ITGA6] [db_xref=CCDS:CCDS2249.1] [protein=integrin alpha-6 isoform b preproprotein] [protein_id=NP_000201.2] [location=204..3425] [gbkey=CDS]

ATGGCCGCCGCCGGGCAGCTGTGCTTGCTCTACCTGTCGGCGGGGCTCCTGTCCCGGCTCGGCGCAGCCT

TCAACTTGGACACTCGGGAGGACAACGTGATCCGGAAATATGGAGACCCCGGGAGCCTCTTCGGCTTCTC

GCTGGCCATGCACTGGCAACTGCAGCCCGAGGACAAGCGGCTGTTGCTCGTGGGGGCCCCGCGGGCAGAA

GCGCTTCCACTGCAGAGAGCCAACAGAACGGGAGGGCTGTACAGCTGCGACATCACCGCCCGGGGGCCAT

GCACGCGGATCGAGTTTGATAACGATGCTGACCCCACGTCAGAAAGCAAGGAAGATCAGTGGATGGGGGT

CACCGTCCAGAGCCAAGGTCCAGGGGGCAAGGTCGTGACATGTGCTCACCGATATGAAAAAAGGCAGCAT

GTTAATACGAAGCAGGAATCCCGAGACATCTTTGGGCGGTGTTATGTCCTGAGTCAGAATCTCAGGATTG

AAGACGATATGGATGGGGGAGATTGGAGCTTTTGTGATGGGCGATTGAGAGGCCATGAGAAATTTGGCTC

TTGCCAGCAAGGTGTAGCAGCTACTTTTACTAAAGACTTTCATTACATTGTATTTGGAGCCCCGGGTACT

TATAACTGGAAAGGGATTGTTCGTGTAGAGCAAAAGAATAACACTTTTTTTGACATGAACATCTTTGAAG

ATGGGCCTTATGAAGTTGGTGGAGAGACTGAGCATGATGAAAGTCTCGTTCCTGTTCCTGCTAACAGTTA

CTTAGGTTTTTCTTTGGACTCAGGGAAAGGTATTGTTTCTAAAGATGAGATCACTTTTGTATCTGGTGCT

CCCAGAGCCAATCACAGTGGAGCCGTGGTTTTGCTGAAGAGAGACATGAAGTCTGCACATCTCCTCCCTG

AGCACATATTCGATGGAGAAGGTCTGGCCTCTTCATTTGGCTATGATGTGGCGGTGGTGGACCTCAACAA

GGATGGGTGGCAAGATATAGTTATTGGAGCCCCACAGTATTTTGATAGAGATGGAGAAGTTGGAGGTGCA

GTGTATGTCTACATGAACCAGCAAGGCAGATGGAATAATGTGAAGCCAATTCGTCTTAATGGAACCAAAG

ATTCTATGTTTGGCATTGCAGTAAAAAATATTGGAGATATTAATCAAGATGGCTACCCAGATATTGCAGT

TGGAGCTCCGTATGATGACTTGGGAAAGGTTTTTATCTATCATGGATCTGCAAATGGAATAAATACCAAA

CCAACACAGGTTCTCAAGGGTATATCACCTTATTTTGGATATTCAATTGCTGGAAACATGGACCTTGATC

GAAATTCCTACCCTGATGTTGCTGTTGGTTCCCTCTCAGATTCAGTAACTATTTTCAGATCCCGGCCTGT

GATTAATATTCAGAAAACCATCACAGTAACTCCTAACAGAATTGACCTCCGCCAGAAAACAGCGTGTGGG

GCGCCTAGTGGGATATGCCTCCAGGTTAAATCCTGTTTTGAATATACTGCTAACCCCGCTGGTTATAATC

CTTCAATATCAATTGTGGGCACACTTGAAGCTGAAAAAGAAAGAAGAAAATCTGGGCTATCCTCAAGAGT

TCAGTTTCGAAACCAAGGTTCTGAGCCCAAATATACTCAAGAACTAACTCTGAAGAGGCAGAAACAGAAA

GTGTGCATGGAGGAAACCCTGTGGCTACAGGATAATATCAGAGATAAACTGCGTCCCATTCCCATAACTG

CCTCAGTGGAGATCCAAGAGCCAAGCTCTCGTAGGCGAGTGAATTCACTTCCAGAAGTTCTTCCAATTCT
GAATTCAGATGAACCCAAGACAGCTCATATTGATGTTCACTTCTTAAAAGAGGGATGTGGAGACGACAAT
GTATGTAACAGCAACCTTAAACTAGAATATAAATTTTGCACCCGAGAAGGAAATCAAGACAAATTTTCTT
ATTTACCAATTCAAAAAGGTGTACCAGAACTAGTTCTAAAAGATCAGAAGGATATTGCTTTAGAAATAAC
AGTGACAAACAGCCCTTCCAACCCAAGGAATCCCACAAAAGATGGCGATGACGCCCATGAGGCTAAACTG
ATTGCAACGTTTCCAGACACTTTAACCTATTCTGCATATAGAGAACTGAGGGCTTTCCCTGAGAAACAGT
TGAGTTGTGTTGCCAACCAGAATGGCTCGCAAGCTGACTGTGAGCTCGGAAATCCTTTTAAAAGAAATTC
AAATGTCACTTTTTATTTGGTTTTAAGTACAACTGAAGTCACCTTTGACACCCCAGATCTGGATATTAAT
CTGAAGTTAGAAACAACAAGCAATCAAGATAATTTGGCTCCAATTACAGCTAAAGCAAAAGTGGTTATTG
AACTGCTTTTATCGGTCTCGGGAGTTGCTAAACCTTCCCAGGTGTATTTTGGAGGTACAGTTGTTGGCGA
GCAAGCTATGAAATCTGAAGATGAAGTGGGAAGTTTAATAGAGTATGAATTCAGGGTAATAAACTTAGGT
AAACCTCTTACAAACCTCGGCACAGCAACCTTGAACATTCAGTGGCCAAAAGAAATTAGCAATGGGAAAT
GGTTGCTTTATTTGGTGAAAGTAGAATCCAAAGGATTGGAAAAGGTAACTTGTGAGCCACAAAAGGAGAT
AAACTCCCTGAACCTAACGGAGTCTCACAACTCAAGAAAGAAACGGGAAATTACTGAAAAACAGATAGAT
GATAACAGAAAATTTTCTTTATTTGCTGAAAGAAAATACCAGACTCTTAACTGTAGCGTGAACGTGAACT
GTGTGAACATCAGATGCCCGCTGCGGGGGCTGGACAGCAAGGCGTCTCTTATTTTGCGCTCGAGGTTATG
GAACAGCACATTTCTAGAGGAATATTCCAAACTGAACTACTTGGACATTCTCATGCGAGCCTTCATTGAT
GTGACTGCTGCTGCCGAAAATATCAGGCTGCCAAATGCAGGCACTCAGGTTCGAGTGACTGTGTTTCCCT
CAAAGACTGTAGCTCAGTATTCGGGAGTACCTTGGTGGATCATCCTAGTGGCTATTCTCGCTGGGATCTT
GATGCTTGCTTTATTAGTGTTTATACTATGGAAGTGTGGTTTCTTCAAGAGAAATAAGAAAGATCATTAT
GATGCCACATATCACAAGGCTGAGATCCATGCTCAGCCATCTGATAAAGAGAGGCTTACTTCTGATGCAT
AG

>lcl|NM_002211.4_cds_NP_002202.2_1 [gene=ITGB1] [db_xref=CCDS:CCDS7174.1] [protein=integrin beta-1 isoform 1A precursor] [protein_id=NP_002202.2] [location=87..2483] [gbkey=CDS]

ATGAATTTACAACCAATTTTCTGGATTGGACTGATCAGTTCAGTTTGCTGTGTGTTTGCTCAAACAGATG

AAAATAGATGTTTAAAAGCAAATGCCAAATCATGTGGAGAATGTATACAAGCAGGGCCAAATTGTGGGTG

GTGCACAAATTCAACATTTTTACAGGAAGGAATGCCTACTTCTGCACGATGTGATGATTTAGAAGCCTTA

AAAAAGAAGGGTTGCCCTCCAGATGACATAGAAAATCCCAGAGGCTCCAAAGATATAAAGAAAAATAAAA

ATGTAACCAACCGTAGCAAAGGAACAGCAGAGAAGCTCAAGCCAGAGGATATTACTCAGATCCAACCACA

GCAGTTGGTTTTGCGATTAAGATCAGGGGAGCCACAGACATTTACATTAAAATTCAAGAGAGCTGAAGAC

TATCCCATTGACCTCTACTACCTTATGGACCTGTCTTACTCAATGAAAGACGATTTGGAGAATGTAAAAA

GTCTTGGAACAGATCTGATGAATGAAATGAGGAGGATTACTTCGGACTTCAGAATTGGATTTGGCTCATT

TGTGGAAAAGACTGTGATGCCTTACATTAGCACAACACCAGCTAAGCTCAGGAACCCTTGCACAAGTGAA

CAGAACTGCACCAGCCCATTTAGCTACAAAAATGTGCTCAGTCTTACTAATAAAGGAGAAGTATTTAATG

AACTTGTTGGAAAACAGCGCATATCTGGAAATTTGGATTCTCCAGAAGGTGGTTTCGATGCCATCATGCA

AGTTGCAGTTTGTGGATCACTGATTGGCTGGAGGAATGTTACACGGCTGCTGGTGTTTTCCACAGATGCC

GGGTTTCACTTTGCTGGAGATGGGAAACTTGGTGGCATTGTTTTACCAAATGATGGACAATGTCACCTGG

AAAATAATATGTACACAATGAGCCATTATTATGATTATCCTTCTATTGCTCACCTTGTCCAGAAACTGAG

TGAAAATAATATTCAGACAATTTTTGCAGTTACTGAAGAATTTCAGCCTGTTTACAAGGAGCTGAAAAAC

TTGATCCCTAAGTCAGCAGTAGGAACATTATCTGCAAATTCTAGCAATGTAATTCAGTTGATCATTGATG

CATACAATTCCCTTTCCTCAGAAGTCATTTTGGAAAACGGCAAATTGTCAGAAGGCGTAACAATAAGTTA

CAAATCTTACTGCAAGAACGGGGTGAATGGAACAGGGGAAAATGGAAGAAAATGTTCCAATATTTCCATT

GGAGATGAGGTTCAATTTGAAATTAGCATAACTTCAAATAAGTGTCCAAAAAAGGATTCTGACAGCTTTA

AAATTAGGCCTCTGGGCTTTACGGAGGAAGTAGAGGTTATTCTTCAGTACATCTGTGAATGTGAATGCCA

AAGCGAAGGCATCCCTGAAAGTCCCAAGTGTCATGAAGGAAATGGGACATTTGAGTGTGGCGCGTGCAGG

TGCAATGAAGGGCGTGTTGGTAGACATTGTGAATGCAGCACAGATGAAGTTAACAGTGAAGACATGGATG

CTTACTGCAGGAAAGAAAACAGTTCAGAAATCTGCAGTAACAATGGAGAGTGCGTCTGCGGACAGTGTGT

TTGTAGGAAGAGGGATAATACAAATGAAATTTATTCTGGCAAATTCTGCGAGTGTGATAATTTCAACTGT

GATAGATCCAATGGCTTAATTTGTGGAGGAAATGGTGTTTGCAAGTGTCGTGTGTGTGAGTGCAACCCCA

ACTACACTGGCAGTGCATGTGACTGTTCTTTGGATACTAGTACTTGTGAAGCCAGCAACGGACAGATCTG

CAATGGCCGGGGCATCTGCGAGTGTGGTGTCTGTAAGTGTACAGATCCGAAGTTTCAAGGGCAAACGTGT

GAGATGTGTCAGACCTGCCTTGGTGTCTGTGCTGAGCATAAAGAATGTGTTCAGTGCAGAGCCTTCAATA

AAGGAGAAAAGAAAGACACATGCACACAGGAATGTTCCTATTTTAACATTACCAAGGTAGAAAGTCGGGA

CAAATTACCCCAGCCGGTCCAACCTGATCCTGTGTCCCATTGTAAGGAGAAGGATGTTGACGACTGTTGG

TTCTATTTTACGTATTCAGTGAATGGGAACAACGAGGTCATGGTTCATGTTGTGGAGAATCCAGAGTGTC

CCACTGGTCCAGACATCATTCCAATTGTAGCTGGTGTGGTTGCTGGAATTGTTCTTATTGGCCTTGCATT

ACTGCTGATATGGAAGCTTTTAATGATAATTCATGACAGAAGGGAGTTTGCTAAATTTGAAAAGGAGAAA

ATGAATGCCAAATGGGACACGGGTGAAAATCCTATTTATAAGAGTGCCGTAACAACTGTGGTCAATCCGA

AGTATGAGGGAAAATGA

>lcl|NM_000213.5_cds_NP_000204.3_1 [gene=ITGB4] [db_xref=CCDS:CCDS11727.1] [protein=integrin beta-4 isoform 1 precursor] [protein_id=NP_000204.3] [location=165..5633] [gbkey=CDS]

ATGGCAGGGCCACGCCCCAGCCCATGGGCCAGGCTGCTCCTGGCAGCCTTGATCAGCGTCAGCCTCTCTG

GGACCTTGGCAAACCGCTGCAAGAAGGCCCCAGTGAAGAGCTGCACGGAGTGTGTCCGTGTGGATAAGGA

CTGCGCCTACTGCACAGACGAGATGTTCAGGGACCGGCGCTGCAACACCCAGGCGGAGCTGCTGGCCGCG

GGCTGCCAGCGGGAGAGCATCGTGGTCATGGAGAGCAGCTTCCAAATCACAGAGGAGACCCAGATTGACA

CCACCCTGCGGCGCAGCCAGATGTCCCCCCAAGGCCTGCGGGTCCGTCTGCGGCCCGGTGAGGAGCGGCA

TTTTGAGCTGGAGGTGTTTGAGCCACTGGAGAGCCCCGTGGACCTGTACATCCTCATGGACTTCTCCAAC

TCCATGTCCGATGATCTGGACAACCTCAAGAAGATGGGGCAGAACCTGGCTCGGGTCCTGAGCCAGCTCA

CCAGCGACTACACTATTGGATTTGGCAAGTTTGTGGACAAAGTCAGCGTCCCGCAGACGGACATGAGGCC

TGAGAAGCTGAAGGAGCCCTGGCCCAACAGTGACCCCCCCTTCTCCTTCAAGAACGTCATCAGCCTGACA

GAAGATGTGGATGAGTTCCGGAATAAACTGCAGGGAGAGCGGATCTCAGGCAACCTGGATGCTCCTGAGG

GCGGCTTCGATGCCATCCTGCAGACAGCTGTGTGCACGAGGGACATTGGCTGGCGCCCGGACAGCACCCA

CCTGCTGGTCTTCTCCACCGAGTCAGCCTTCCACTATGAGGCTGATGGCGCCAACGTGCTGGCTGGCATC

ATGAGCCGCAACGATGAACGGTGCCACCTGGACACCACGGGCACCTACACCCAGTACAGGACACAGGACT

ACCCGTCGGTGCCCACCCTGGTGCGCCTGCTCGCCAAGCACAACATCATCCCCATCTTTGCTGTCACCAA

CTACTCCTATAGCTACTACGAGAAGCTTCACACCTATTTCCCTGTCTCCTCACTGGGGGTGCTGCAGGAG

GACTCGTCCAACATCGTGGAGCTGCTGGAGGAGGCCTTCAATCGGATCCGCTCCAACCTGGACATCCGGG

CCCTAGACAGCCCCCGAGGCCTTCGGACAGAGGTCACCTCCAAGATGTTCCAGAAGACGAGGACTGGGTC

CTTTCACATCCGGCGGGGGGAAGTGGGTATATACCAGGTGCAGCTGCGGGCCCTTGAGCACGTGGATGGG

ACGCACGTGTGCCAGCTGCCGGAGGACCAGAAGGGCAACATCCATCTGAAACCTTCCTTCTCCGACGGCC

TCAAGATGGACGCGGGCATCATCTGTGATGTGTGCACCTGCGAGCTGCAAAAAGAGGTGCGGTCAGCTCG

CTGCAGCTTCAACGGAGACTTCGTGTGCGGACAGTGTGTGTGCAGCGAGGGCTGGAGTGGCCAGACCTGC

AACTGCTCCACCGGCTCTCTGAGTGACATTCAGCCCTGCCTGCGGGAGGGCGAGGACAAGCCGTGCTCCG

GCCGTGGGGAGTGCCAGTGCGGGCACTGTGTGTGCTACGGCGAAGGCCGCTACGAGGGTCAGTTCTGCGA

GTATGACAACTTCCAGTGTCCCCGCACTTCCGGGTTCCTCTGCAATGACCGAGGACGCTGCTCCATGGGC

CAGTGTGTGTGTGAGCCTGGTTGGACAGGCCCAAGCTGTGACTGTCCCCTCAGCAATGCCACCTGCATCG

ACAGCAATGGGGGCATCTGTAATGGACGTGGCCACTGTGAGTGTGGCCGCTGCCACTGCCACCAGCAGTC

GCTCTACACGGACACCATCTGCGAGATCAACTACTCGGCGATCCACCCGGGCCTCTGCGAGGACCTACGC

TCCTGCGTGCAGTGCCAGGCGTGGGGCACCGGCGAGAAGAAGGGGCGCACGTGTGAGGAATGCAACTTCA

AGGTCAAGATGGTGGACGAGCTTAAGAGAGCCGAGGAGGTGGTGGTGCGCTGCTCCTTCCGGGACGAGGA

TGACGACTGCACCTACAGCTACACCATGGAAGGTGACGGCGCCCCTGGGCCCAACAGCACTGTCCTGGTG

CACAAGAAGAAGGACTGCCCTCCGGGCTCCTTCTGGTGGCTCATCCCCCTGCTCCTCCTCCTGCCGC

TCCTGGCCCTGCTACTGCTGCTATGCTGGAAGTACTGTGCCTGCTGCAAGGCCTGCCTGGCACTTCTCCC

GTGCTGCAACCGAGGTCACATGGTGGGCTTTAAGGAAGACCACTACATGCTGCGGGAGAACCTGATGGCC

TCTGACCACTTGGACACGCCCATGCTGCGCAGCGGGAACCTCAAGGGCCGTGACGTGGTCCGCTGGAAGG

TCACCAACAACATGCAGCGGCCTGGCTTTGCCACTCATGCCGCCAGCATCAACCCCACAGAGCTGGTGCC

CTACGGGCTGTCCTTGCGCCTGGCCCGCCTTTGCACCGAGAACCTGCTGAAGCCTGACACTCGGGAGTGC

GCCCAGCTGCGCCAGGAGGTGGAGGAGAACCTGAACGAGGTCTACAGGCAGATCTCCGGTGTACACAAGC

TCCAGCAGACCAAGTTCCGGCAGCAGCCCAATGCCGGGAAAAAGCAAGACCACACCATTGTGGACACAGT

GCTGATGGCGCCCCGCTCGGCCAAGCCGGCCCTGCTGAAGCTTACAGAGAAGCAGGTGGAACAGAGGGCC

TTCCACGACCTCAAGGTGGCCCCCGGCTACTACACCCTCACTGCAGACCAGGACGCCCGGGGCATGGTGG

AGTTCCAGGAGGGCGTGGAGCTGGTGGACGTACGGGTGCCCCTCTTTATCCGGCCTGAGGATGACGACGA

GAAGCAGCTGCTGGTGGAGGCCATCGACGTGCCCGCAGGCACTGCCACCCTCGGCCGCCGCCTGGTAAAC

ATCACCATCATCAAGGAGCAAGCCAGAGACGTGGTGTCCTTTGAGCAGCCTGAGTTCTCGGTCAGCCGCG

GGGACCAGGTGGCCCGCATCCCTGTCATCCGGCGTGTCCTGGACGGCGGGAAGTCCCAGGTCTCCTACCG

CACACAGGATGGCACCGCGCAGGGCAACCGGGACTACATCCCCGTGGAGGGTGAGCTGCTGTTCCAGCCT

GGGGAGGCCTGGAAAGAGCTGCAGGTGAAGCTCCTGGAGCTGCAAGAAGTTGACTCCCTCCTGCGGGGCC

GCCAGGTCCGCCGTTTCCACGTCCAGCTCAGCAACCCTAAGTTTGGGGCCCACCTGGGCCAGCCCCACTC

CACCACCATCATCATCAGGGACCCAGATGAACTGGACCGGAGCTTCACGAGTCAGATGTTGTCATCACAG

CCACCCCCTCACGGCGACCTGGGCGCCCCGCAGAACCCCAATGCTAAGGCCGCTGGGTCCAGGAAGATCC

ATTTCAACTGGCTGCCCCCTTCTGGCAAGCCAATGGGGTACAGGGTAAAGTACTGGATTCAGGGTGACTC

CGAATCCGAAGCCCACCTGCTCGACAGCAAGGTGCCCTCAGTGGAGCTCACCAACCTGTACCCGTATTGC

GACTATGAGATGAAGGTGTGCGCCTACGGGGCTCAGGGCGAGGGACCCTACAGCTCCCTGGTGTCCTGCC

GCACCCACCAGGAAGTGCCCAGCGAGCCAGGGCGTCTGGCCTTCAATGTCGTCTCCTCCACGGTGACCCA

GCTGAGCTGGGCTGAGCCGGCTGAGACCAACGGTGAGATCACAGCCTACGAGGTCTGCTATGGCCTGGTC

AACGATGACAACCGACCTATTGGGCCCATGAAGAAAGTGCTGGTTGACAACCCTAAGAACCGGATGCTGC

TTATTGAGAACCTTCGGGAGTCCCAGCCCTACCGCTACACGGTGAAGGCGCGCAACGGGGCCGGCTGGGG

GCCTGAGCGGGAGGCCATCATCAACCTGGCCACCCAGCCCAAGAGGCCCATGTCCATCCCCATCATCCCT

GACATCCCTATCGTGGACGCCCAGAGCGGGGAGGACTACGACAGCTTCCTTATGTACAGCGATGACGTTC

TACGCTCTCCATCGGGCAGCCAGAGGCCCAGCGTCTCCGATGACACTGGCTGCGGCTGGAAGTTCGAGCC

CCTGCTGGGGGAGGAGCTGGACCTGCGGCGCGTCACGTGGCGGCTGCCCCCGGAGCTCATCCCGCGCCTG

TCGGCCAGCAGCGGGCGCTCCTCCGACGCCGAGGCGCCCCACGGGCCCCCGGACGACGGCGGCGCGGGCG

GGAAGGGCGGCAGCCTGCCCCGCAGTGCGACACCCGGGCCCCCCGGAGAGCACCTGGTGAATGGCCGGAT

GGACTTTGCCTTCCCGGGCAGCACCAACTCCCTGCACAGGATGACCACGACCAGTGCTGCTGCCTATGGC

ACCCACCTGAGCCCACACGTGCCCCACCGCGTGCTAAGCACATCCTCCACCCTCACACGGGACTACAACT

CACTGACCCGCTCAGAACACTCACACTCGACCACACTGCCCAGGGACTACTCCACCCTCACCTCCGTCTC

CTCCCACGACTCTCGCCTGACTGCTGGTGTGCCCGACACGCCCACCCGCCTGGTGTTCTCTGCCCTGGGG

CCCACATCTCTCAGAGTGAGCTGGCAGGAGCCGCGGTGCGAGCGGCCGCTGCAGGGCTACAGTGTGGAGT

ACCAGCTGCTGAACGGCGGTGAGCTGCATCGGCTCAACATCCCCAACCCTGCCCAGACCTCGGTGGTGGT

GGAAGACCTCCTGCCCAACCACTCCTACGTGTTCCGCGTGCGGGCCCAGAGCCAGGAAGGCTGGGGCCGA

GAGCGTGAGGGTGTCATCACCATTGAATCCCAGGTGCACCCGCAGAGCCCACTGTGTCCCCTGCCAGGCT

CCGCCTTCACTTTGAGCACTCCCAGTGCCCCAGGCCCGCTGGTGTTCACTGCCCTGAGCCCAGACTCGCT

GCAGCTGAGCTGGGAGCGGCCACGGAGGCCCAATGGGGATATCGTCGGCTACCTGGTGACCTGTGAGATG

GCCCAAGGAGGAGGGCCAGCCACCGCATTCCGGGTGGATGGAGACAGCCCCGAGAGCCGGCTGACCGTGC

CGGGCCTCAGCGAGAACGTGCCCTACAAGTTCAAGGTGCAGGCCAGGACCACTGAGGGCTTCGGGCCAGA

GCGCGAGGGCATCATCACCATAGAGTCCCAGGATGGAGGACCCTTCCCGCAGCTGGGCAGCCGTGCCGGG

CTCTTCCAGCACCCGCTGCAAAGCGAGTACAGCAGCATCACCACCACCCACACCAGCGCCACCGAGCCCT

TCCTAGTGGATGGGCTGACCCTGGGGGCCCAGCACCTGGAGGCAGGCGGCTCCCTCACCCGGCATGTGAC

CCAGGAGTTTGTGAGCCGGACACTGACCACCAGCGGAACCCTTAGCACCCACATGGACCAACAGTTCTTC

CAAACTTGA

>lcl|NM_002222.7_cds_NP_002213.5_1 [gene=ITPR1] [db_xref=CCDS:CCDS54550.1] [protein=inositol 1,4,5-trisphosphate receptor type 1 isoform 2] [protein_id=NP_002213.5] [location=351..8438] [gbkey=CDS]

ATGTCTGACAAAATGTCTAGCTTCCTACATATTGGAGACATTTGTTCTCTGTACGCGGAGGGATCGACAA

ATGGATTTATTAGCACCTTGGGCCTGGTTGATGATCGTTGTGTTGTACAGCCAGAAACCGGGGACCTTAA

CAATCCACCTAAGAAATTCAGAGACTGCCTCTTTAAGCTATGTCCCATGAACCGCTACTCTGCCCAAAAG

CAGTTCTGGAAAGCCGCTAAGCCTGGGGCCAACAGCACCACAGACGCAGTGCTACTCAACAAACTGCACC

ACGCTGCAGACTTGGAAAAGAAGCAGAATGAGACAGAAAACAGGAAATTGCTGGGGACCGTAATCCAGTA

TGGCAATGTGATCCAGCTCCTGCATTTGAAAAGTAATAAATACCTAACAGTGAATAAGAGGCTTCCTGCT

CTGTTGGAGAAGAATGCCATGAGAGTCACATTGGACGAGGCTGGAAATGAAGGGTCCTGGTTTTATATTC

AGCCATTCTACAAGCTGCGATCCATTGGAGACAGCGTGGTCATAGGTGACAAGGTGGTTCTGAACCCCGT

CAATGCTGGTCAGCCCCTACATGCTAGCAGCCATCAACTGGTAGATAACCCAGGCTGCAATGAGGTCAAT

TCCGTCAACTGCAATACAAGCTGGAAAATAGTCCTTTTCATGAAATGGAGTGATAACAAAGACGACATAT

TAAAGGGGGGTGACGTGGTGAGGCTGTTTCATGCTGAGCAGGAGAAGTTTCTCACCTGTGACGAACACAG

GAAGAAGCAGCACGTCTTCCTGAGAACCACGGGCCGGCAGTCGGCCACATCTGCCACCAGTTCAAAAGCC

CTGTGGGAGGTGGAGGTGGTCCAGCATGACCCATGTCGGGGCGGAGCAGGGTATTGGAACAGCCTTTTCC

GTTTCAAGCATCTGGCCACGGGGCATTACTTGGCAGCAGAGGTGGACCCTGATCAGGACGCCTCTCGAAG

TAGGTTGCGGAATGCCCAAGAAAAGATGGTATACTCCCTGGTCTCTGTGCCTGAAGGCAATGACATCTCC

TCCATTTTCGAGCTAGATCCCACCACTCTGCGTGGAGGTGACAGCCTTGTCCCAAGGAACTCTTATGTTC

GGCTCAGACACCTATGTACTAATACCTGGGTTCACAGCACAAATATTCCTATTGACAAGGAAGAAGAAAA

GCCCGTGATGCTGAAAATTGGCACCTCTCCTGTGAAGGAGGATAAGGAAGCATTTGCCATAGTTCCGGTT

TCTCCTGCTGAAGTTCGGGACCTGGACTTTGCCAATGATGCCAGCAAGGTGCTGGGCTCCATTGCTGGGA

AGCTAGAGAAGGGCACCATCACCCAGAATGAAAGGAGGTCTGTAACCAAGCTGCTAGAAGATTTGGTTTA

CTTCGTCACTGGTGGAACTAATTCTGGTCAAGATGTTCTCGAAGTTGTCTTCTCCAAGCCCAACAGAGAA

CGGCAGAAACTGATGAGAGAACAGAATATTCTCAAGCAGATCTTCAAGTTGTTACAAGCCCCATTCACAG

ACTGCGGTGATGGCCCAATGCTTCGGCTGGAAGAGCTCGGGGACCAGCGGCACGCTCCTTTCAGACACAT

CTGCCGGCTCTGCTACAGGGTGCTGAGACACTCGCAGCAAGACTACAGGAAGAACCAGGAGTATATAGCC

AAGCAGTTTGGCTTCATGCAGAAGCAGATTGGCTATGATGTGTTGGCTGAAGACACTATCACTGCCCTGC

TCCACAATAATCGGAAACTCCTGGAAAAACACATTACCGCGGCAGAGATTGACACATTTGTCAGCCTGGT

GCGAAAGAACAGGGAGCCCAGATTCTTAGATTACCTCTCCGACCTCTGTGTCTCCATGAACAAATCAATT

CCAGTGACCCAGGAACTGATATGTAAAGCTGTGCTGAACCCCACCAACGCTGACATCCTGATTGAGACCA

AGTTGGTTCTTTCTCGTTTTGAATTTGAAGGTGTCTCTTCCACTGGAGAGAATGCTCTGGAGGCAGGAGA

AGACGAGGAAGAGGTGTGGCTGTTTTGGAGGGACAGCAACAAAGAGATTCGCAGCAAGAGTGTGAGGGAA

TTGGCTCAGGATGCTAAAGAAGGGCAGAAGGAGGACCGAGACGTTCTCAGCTACTACAGATATCAGCTGA

ACCTCTTTGCGAGGATGTGTCTGGACCGCCAATACCTGGCCATCAACGAAATCTCAGGCCAGCTGGATGT

CGATCTCATTCTCCGCTGCATGTCTGACGAGAACCTGCCCTATGACCTCAGGGCGTCCTTCTGCCGCCTC

ATGCTTCACATGCATGTGGACCGAGATCCCCAGGAACAAGTCACCCCCGTGAAATATGCCCGCCTCTGGT

CGGAGATTCCCTCGGAGATCGCCATTGACGACTATGATAGTAGTGGAGCTTCCAAAGATGAAATTAAGGA

GAGATTTGCTCAGACCATGGAGTTTGTGGAGGAGTATTTAAGAGATGTGGTTTGTCAGAGGTTCCCTTTC

TCTGATAAAGAGAAGAATAAGCTTACGTTTGAGGTTGTAAATTTAGCTAGGAATCTCATATACTTTGGTT

TCTACAACTTCTCTGACCTTCTACGATTAACTAAGATCCTTCTGGCCATATTGGACTGTGTACATGTGAC

AACAATCTTCCCCATTAGCAAGATGGCGAAAGGAGAAGAGAATAAAGGCAGTAACGTGATGAGATCTATT

CATGGCGTGGGAGAGCTGATGACCCAGGTGGTGCTCCGGGGAGGAGGCTTTTTGCCCATGACTCCCATGG

CTGCTGCCCCTGAAGGCAATGTGAAGCAGGCAGAGCCTGAGAAGGAGGACATCATGGTCATGGACACCAA

GCTGAAGATCATTGAGATACTCCAGTTTATTTTGAATGTGAGGTTGGATTATAGGATCTCCTGCCTCCTG

TGTATATTTAAGCGAGAGTTTGATGAAAGCAATTCCCAGACTTCAGAAACATCCTCCGGAAACAGCAGCC

AAGAAGGGCCAAGTAATGTACCAGGTGCTCTTGACTTTGAACACATTGAAGAACAAGCAGAAGGCATCTT

TGGAGGAAGTGAGGAGAACACCCCACTGGACTTGGATGACCACGGCGGCAGAACCTTTCTCCGTGTCCTG

CTCCACTTGACGATGCATGACTACCCACCCCTGGTGTCAGGGGCCCTGCAGCTCCTCTTCCGGCACTTCA

GCCAGAGGCAGGAGGTGCTCCAGGCCTTCAAACAGGTTCAACTGCTGGTTACCAGCCAAGATGTGGACAA

CTACAAACAGATCAAACAAGACTTGGATCAACTGAGGTCCATCGTGGAAAAGTCAGAGCTTTGGGTGTAC

AAAGGGCAGGGCCCCGATGAGACTATGGATGGTGCATCTGGAGAAAATGAACATAAGAAAACGGAGGAGG

GAAATAACAAGCCACAAAAGCATGAAAGCACCAGCAGCTACAACTACAGAGTGGTCAAAGAGATTTTGAT

TCGGCTTAGCAAACTCTGTGTTCAAGAGAGTGCCTCAGTGAGAAAGAGCAGGAAGCAGCAACAGCGTCTG

CTCCGGAACATGGGCGCGCACGCCGTGGTGCTGGAGCTGCTGCAGATTCCCTATGAGAAGGCCGAAGATA

CCAAGATGCAAGAGATAATGAGGTTGGCTCATGAATTTTTGCAGAATTTCTGCGCAGGCAACCAGCAGAA

TCAAGCTTTGCTACATAAACACATAAACCTGTTTCTCAACCCAGGGATCCTGGAGGCAGTAACCATGCAG

CACATCTTCATGAACAATTTCCAGCTTTGCAGTGAGATCAACGAGAGAGTTGTTCAGCACTTCGTTCACT

GCATAGAGACTCACGGTCGGAATGTCCAGTATATAAAGTTCTTACAGACAATTGTCAAGGCAGAAGGGAA

ATTTATTAAAAAATGCCAAGACATGGTTATGGCCGAGCTGGTCAATTCGGGAGAGGATGTCCTCGTGTTC

TACAACGACAGAGCCTCTTTCCAGACTCTGATCCAGATGATGCGGTCAGAACGGGATCGGATGGATGAGA

ACAGCCCTCTCATGTACCACATCCACTTGGTCGAGCTCCTGGCTGTGTGCACGGAGGGTAAGAATGTCTA

CACAGAGATCAAGTGCAACTCCCTGCTCCCGCTGGATGACATCGTTCGCGTGGTGACCCACGAGGACTGC

ATCCCTGAGGTTAAAATTGCATACATTAACTTCCTGAATCACTGCTATGTGGATACAGAGGTGGAAATGA

AGGAGATTTATACCAGCAATCACATGTGGAAATTGTTTGAGAATTTCCTTGTAGACATCTGCAGGGCCTG

TAACAACACTAGTGACAGGAAACATGCAGACTCGATTTTGGAGAAGTATGTCACCGAAATCGTCATGAGT

ATTGTTACTACTTTCTTCAGCTCTCCCTTCTCAGACCAGAGTACGACTTTGCAGACTCGCCAGCCTGTCT

TTGTGCAACTGCTGCAAGGCGTGTTCAGGGTTTACCACTGCAACTGGTTAATGCCAAGCCAAAAAGCCTC

CGTGGAGAGCTGTATTCGGGTGCTGTCTGATGTAGCCAAGAGCCGGGCCATTGCCATTCCCGTGGACCTG

GACAGCCAAGTCAACAACCTCTTTCTCAAGTCCCACAGCATTGTGCAGAAAACAGCCATGAACTGGCGGC

TCTCAGCCCGCAATGCCGCACGCAGGGACTCTGTTCTGGCAGCTTCCAGAGACTACCGGAATATCATTGA

GAGATTGCAGGACATCGTCTCCGCGCTGGAGGACCGTCTCAGGCCCCTGGTGCAGGCAGAGTTATCTGTG

CTCGTGGATGTTCTCCACAGACCCGAGCTGCTTTTCCCAGAGAACACAGACGCCAGAAGGAAATGTGAAA

GTGGCGGTTTCATTTGCAAGTTAATAAAGCATACAAAACAGCTGCTAGAAGAAAATGAAGAGAAGCTCTG

CATTAAGGTCCTACAGACCCTGAGGGAAATGATGACCAAAGATAGAGGCTATGGAGAAAAGGGTGAGGCG

CTCAGGCAAGTTCTGGTCAACCGTTACTATGGAAACGTCAGACCTTCGGGACGAAGAGAGAGCCTTACCA

GCTTTGGCAATGGCCCACTGTCAGCAGGAGGACCCGGCAAGCCCGGGGGAGGAGGGGGAGGTTCCGGATC

CAGCTCTATGAGCAGGGGTGAGATGAGTCTGGCCGAGGTTCAGTGTCACCTTGACAAGGAGGGGGCTTCC

AATCTAGTTATCGACCTCATCATGAACGCATCCAGTGACCGAGTGTTCCATGAAAGCATTCTCCTGGCCA

TTGCCCTTCTGGAAGGAGGCAACACCACCATCCAGCACTCCTTTTTCTGTCGCTTGACAGAAGATAAGAA

GTCAGAGAAATTCTTTAAGGTGTTTTATGACCGGATGAAGGTGGCCCAGCAAGAAATCAAAGCAACAGTG

ACAGTGAACACCAGTGACTTGGGAAATAAAAAGAAAGACGATGAGGTAGACAGGGATGCCCCATCACGGA

AAAAAGCTAAAGAGCCCACAACACAGATAACAGAAGAGGTCCGGGATCAGCTCCTGGAGGCCTCCGCTGC

CACCAGGAAAGCCTTCACCACTTTCAGGAGGGAGGCTGATCCCGACGACCACTACCAGCCTGGAGAGGGC

ACCCAGGCCACTGCCGACAAGGCCAAGGACGACCTGGAGATGAGCGCGGTCATCACCATCATGCAGCCCA

TCCTCCGCTTCCTTCAGCTCCTGTGTGAAAACCACAACCGAGACCTGCAGAACTTCCTCCGTTGCCAAAA

TAACAAGACCAACTACAATTTGGTATGTGAGACCCTGCAGTTTCTGGACTGTATTTGTGGAAGCACAACT

GGAGGCCTTGGTCTTCTGGGCTTGTATATAAATGAAAAGAATGTAGCGCTTATCAACCAAACCCTGGAAA

GTCTGACCGAATACTGTCAAGGACCTTGCCATGAGAACCAGAACTGCATAGCCACCCATGAATCCAATGG

CATTGACATCATCACAGCCCTGATCCTCAATGATATCAATCCTTTGGGAAAGAAGAGGATGGACCTTGTG

TTAGAACTGAAGAACAATGCCTCGAAGTTGCTCCTGGCCATCATGGAAAGCAGGCACGACAGTGAAAACG

CAGAGAGGATACTTTATAACATGAGGCCCAAGGAACTGGTGGAAGTGATCAAGAAAGCCTACATGCAAGG

TGAAGTGGAATTTGAGGATGGAGAAAACGGTGAGGATGGGGCGGCGTCCCCCAGGAACGTGGGGCACAAC

ATCTACATATTAGCCCATCAGTTGGCTCGGCATAACAAAGAACTTCAGAGCATGCTGAAACCTGGTGGCC

AAGTGGACGGAGATGAAGCCCTGGAGTTTTATGCCAAGCACACGGCGCAGATAGAGATTGTCAGATTAGA

CCGAACAATGGAACAGATAGTCTTTCCCGTGCCCAGCATATGTGAATTCCTAACCAAGGAGTCAAAACTA

CGAATTTACTATACTACAGAGAGAGACGAACAAGGCAGCAAAATCAATGATTTCTTTCTGCGGTCTGAAG

ACCTCTTCAATGAAATGAATTGGCAGAAGAAACTGAGAGCCCAGCCCGTGTTGTACTGGTGTGCCCGCAA

CATGTCTTTCTGGAGCAGCATTTCGTTTAACCTGGCCGTCCTGATGAACCTGCTGGTGGCGTTTTTCTAC

CCGTTTAAGGGAGTCCGAGGAGGAACCCTGGAGCCCCACTGGTCGGGACTCCTGTGGACAGCCATGCTCA

TCTCTCTGGCCATCGTCATTGCCCTCCCCAAGCCCCATGGCATCCGGGCCTTAATTGCCTCCACAATTCT

ACGACTGATATTTTCAGTCGGGTTACAACCCACGTTGTTTCTTCTGGGCGCTTTCAATGTATGCAATAAA

ATCATCTTTCTAATGAGCTTTGTGGGCAACTGTGGGACATTCACAAGAGGCTACCGAGCCATGGTTCTGG

ATGTTGAGTTCCTCTATCATTTGTTGTATCTGGTGATCTGTGCCATGGGGCTCTTTGTCCATGAATTCTT

CTACAGTCTGCTGCTTTTTGATTTAGTGTACAGAGAAGAGACTTTGCTTAATGTCATTAAAAGTGTCACT

CGCAATGGACGGTCCATCATCCTGACAGCAGTTCTGGCTCTGATCCTCGTTTACCTGTTCTCAATAGTGG

GCTATCTTTTCTTCAAGGATGACTTTATCTTGGAAGTAGATAGGCTGCCCAATGAAACAGCTGTTCCAGA

AACCGGCGAGAGTTTGGCAAGCGAGTTCCTGTTCTCCGATGTGTGTAGGGTGGAGAGTGGGGAGAACTGC

TCCTCTCCTGCACCCAGAGAAGAGCTGGTCCCTGCAGAAGAGACGGAACAGGATAAAGAGCACACATGTG

AGACGCTGCTGATGTGCATTGTCACTGTGCTGAGTCACGGGCTGCGGAGCGGGGGTGGAGTAGGAGATGT

ACTCAGGAAGCCGTCCAAAGAGGAACCCCTGTTTGCTGCTAGAGTTATTTATGACCTCTTGTTCTTCTTC

ATGGTCATCATCATTGTTCTTAACCTGATTTTTGGGGTTATCATTGACACTTTTGCTGACCTGAGGAGTG

AGAAGCAGAAGAAGGAAGAGATCTTGAAGACCACGTGCTTTATCTGTGGCTTGGAAAGAGACAAGTTTGA

CAACAAGACTGTCACCTTTGAAGAGCACATCAAGGAAGAACACAACATGTGGCACTATCTGTGCTTCATC

GTCCTGGTGAAAGTAAAGGACTCCACCGAATATACTGGGCCTGAGAGTTACGTGGCAGAAATGATCAAGG

AAAGAAACCTTGACTGGTTCCCCAGGATGAGAGCCATGTCATTGGTCAGCAGTGATTCTGAAGGAGAACA

GAATGAGCTGAGAAACCTGCAGGAGAAGCTGGAGTCCACCATGAAACTTGTCACGAACCTTTCTGGCCAG

CTGTCGGAATTAAAGGATCAGATGACAGAACAAAGGAAGCAGAAACAAAGAATTGGTCTTCTAGGACATC

CTCCTCACATGAATGTCAACCCACAACAACCAGCATAA

>lcl|NM_004521.3_cds_NP_004512.1_1 [gene=KIF5B] [db_xref=CCDS:CCDS7171.1] [protein=kinesin-1 heavy chain] [protein_id=NP_004512.1] [location=453..3344] [gbkey=CDS]

ATGGCGGACCTGGCCGAGTGCAACATCAAAGTGATGTGTCGCTTCAGACCTCTCAACGAGTCTGAAGTGA

ACCGCGGCGACAAGTACATCGCCAAGTTTCAGGGAGAAGACACGGTCGTGATCGCGTCCAAGCCTTATGC

ATTTGATCGGGTGTTCCAGTCAAGCACATCTCAAGAGCAAGTGTATAATGACTGTGCAAAGAAGATTGTT

AAAGATGTACTTGAAGGATATAATGGAACAATATTTGCATATGGACAAACATCCTCTGGGAAGACACACA

CAATGGAGGGTAAACTTCATGATCCAGAAGGCATGGGAATTATTCCAAGAATAGTGCAAGATATTTTTAA

TTATATTTACTCCATGGATGAAAATTTGGAATTTCATATTAAGGTTTCATATTTTGAAATATATTTGGAT

AAGATAAGGGACCTGTTAGATGTTTCAAAGACCAACCTTTCAGTTCATGAAGACAAAAACCGAGTTCCCT

ATGTAAAGGGGTGCACAGAGCGTTTTGTATGTAGTCCAGATGAAGTTATGGATACCATAGATGAAGGAAA

ATCCAACAGACATGTAGCAGTTACAAATATGAATGAACATAGCTCTAGGAGTCACAGTATATTTCTTATT

AATGTCAAACAAGAGAACACACAAACGGAACAAAAGCTGAGTGGAAAACTTTATCTGGTTGATTTAGCTG

GTAGTGAAAAGGTTAGTAAAACTGGAGCTGAAGGTGCTGTGCTGGATGAAGCTAAAAACATCAACAAGTC

ACTTTCTGCTCTTGGAAATGTTATTTCTGCTTTGGCTGAGGGTAGTACATATGTTCCATATCGAGATAGT

AAAATGACAAGAATCCTTCAAGATTCATTAGGTGGCAACTGTAGAACCACTATTGTAATTTGCTGCTCTC

CATCATCATACAATGAGTCTGAAACAAAATCTACACTCTTATTTGGCCAAAGGGCCAAAACAATTAAGAA

CACAGTTTGTGTCAATGTGGAGTTAACTGCAGAACAGTGGAAAAAGAAGTATGAAAAAGAAAAAGAAAAA

AATAAGATCCTGCGGAACACTATTCAGTGGCTTGAAAATGAGCTCAACAGATGGCGTAATGGGGAGACGG

TGCCTATTGATGAACAGTTTGACAAAGAGAAAGCCAACTTGGAAGCTTTCACAGTGGATAAAGATATTAC

TCTTACCAATGATAAACCAGCAACCGCAATTGGAGTTATAGGAAATTTTACTGATGCTGAAAGAAGAAAG

TGTGAAGAAGAAATTGCTAAATTATACAAACAGCTTGATGACAAGGATGAAGAAATTAACCAGCAAAGTC

AACTGGTAGAGAAACTGAAGACGCAAATGTTGGATCAGGAGGAGCTTTTGGCATCTACCAGAAGGGATCA

AGACAATATGCAAGCTGAGCTGAATCGCCTTCAAGCAGAAAATGATGCCTCTAAAGAAGAAGTGAAAGAA

GTTTTACAGGCCCTAGAAGAACTTGCTGTCAATTATGATCAGAAGTCTCAGGAAGTTGAAGACAAAACTA

AGGAATATGAATTGCTTAGTGATGAATTGAATCAGAAATCGGCAACTTTAGCGAGTATAGATGCTGAGCT

TCAGAAACTTAAGGAAATGACCAACCACCAGAAAAAACGAGCAGCTGAGATGATGGCATCTTTACTAAAA

GACCTTGCAGAAATAGGAATTGCTGTGGGAAATAATGATGTAAAGCAGCCTGAGGGAACTGGCATGATAG

ATGAAGAGTTCACTGTTGCAAGACTCTACATTAGCAAAATGAAGTCAGAAGTAAAAACCATGGTGAAACG

TTGCAAGCAGTTAGAAAGCACACAAACTGAGAGCAACAAAAAAATGGAAGAAAATGAAAAGGAGTTAGCA

GCATGTCAGCTTCGTATCTCTCAACATGAAGCCAAAATCAAGTCATTGACTGAATACCTTCAAAATGTGG

AACAAAAGAAAAGACAGTTGGAGGAATCTGTCGATGCCCTCAGTGAAGAACTAGTCCAGCTTCGAGCACA

AGAGAAAGTCCATGAAATGGAAAAGGAGCACTTAAATAAGGTTCAGACTGCAAATGAAGTTAAGCAAGCT

GTTGAACAGCAGATCCAGAGCCATAGAGAAACTCATCAAAAACAGATCAGTAGTTTGAGAGATGAAGTAG

AAGCAAAAGCAAAACTTATTACTGATCTTCAAGACCAAAACCAGAAAATGATGTTAGAGCAGGAACGTCT

AAGAGTAGAACATGAGAAGTTGAAAGCCACAGATCAGGAAAAGAGCAGAAAACTACATGAACTTACGGTT

ATGCAAGATAGACGAGAACAAGCAAGACAAGACTTGAAGGGTTTGGAAGAGACAGTGGCAAAAGAACTTC

AGACTTTACACAACCTGCGCAAACTCTTTGTTCAGGACCTGGCTACAAGAGTTAAAAAGAGTGCTGAGAT

TGATTCTGATGACACCGGAGGCAGCGCTGCTCAGAAGCAAAAAATCTCCTTTCTTGAAAATAATCTTGAA

CAGCTCACTAAAGTGCACAAACAGTTGGTACGTGATAATGCAGATCTCCGCTGTGAACTTCCTAAGTTGG

AAAAGCGACTTCGAGCTACAGCTGAGAGAGTGAAAGCTTTGGAATCAGCACTGAAAGAAGCTAAAGAAAA

TGCATCTCGTGATCGCAAACGCTATCAGCAAGAAGTAGATCGCATAAAGGAAGCAGTCAGGTCAAAGAAT

ATGGCCAGAAGAGGGCATTCTGCACAGATTGCTAAACCTATTCGTCCCGGGCAACATCCAGCAGCTTCTC

CAACTCACCCAAGTGCAATTCGTGGAGGAGGTGCATTTGTTCAGAACAGCCAGCCAGTGGCAGTGCGAGG

TGGAGGAGGCAAACAAGTGTAA

>lcl|NM_017644.3_cds_NP_060114.2_1 [gene=KLHL24] [db_xref=CCDS:CCDS3246.1] [protein=kelch-like protein 24 isoform b] [protein_id=NP_060114.2] [location=296..2098] [gbkey=CDS]

ATGGTACTAATATTGGGACGCAGACTAAACAGAGAGGATCTTGGGGTGCGTGATTCCCCAGCAACTAAGC

GAAAAGTTTTTGAAATGGACCCCAAATCTCTGACAGGTCATGAGTTTTTTGACTTCTCTTCAGGATCATC

CCATGCCGAAAACATACTCCAGATATTTAATGAATTTCGTGATAGCCGCTTATTCACAGATGTTATCATT

TGTGTGGAAGGAAAAGAATTTCCTTGCCATAGAGCTGTGCTCTCAGCCTGTAGCAGCTACTTCAGAGCTA

TGTTTTGTAATGACCACAGGGAAAGCCGAGAAATGTTGGTTGAGATCAATGGTATTTTAGCTGAAGCTAT

GGAATGTTTTTTGCAGTATGTTTATACTGGAAAGGTGAAGATCACTACAGAGAATGTACAGTATCTCTTT

GAGACATCAAGCCTCTTTCAGATTAGTGTTCTCCGTGATGCATGTGCCAAGTTCTTGGAGGAGCAACTTG

ATCCTTGTAATTGCTTAGGAATCCAGCGCTTTGCTGATACCCATTCACTCAAAACACTCTTCACAAAATG

CAAAAATTTTGCGTTACAGACTTTTGAGGATGTATCCCAGCACGAAGAATTTCTTGAGCTTGACAAAGAT

GAACTTATTGATTATATTTGTAGTGATGAACTTGTTATTGGTAAAGAGGAGATGGTTTTTGAAGCCGTCA

TGCGTTGGGTCTATCGTGCCGTTGATCTGAGAAGACCACTGTTACACGAGCTCCTGACACATGTGAGACT

CCCTCTGTTGCATCCCAACTACTTTGTTCAAACAGTTGAAGTGGACCAATTGATCCAGAATTCTCCTGAG

TGTTATCAGTTGTTGCATGAAGCAAGACGGTACCACATACTTGGGAATGAAATGATGTCCCCAAGGACTA

GGCCACGCAGGTCCACTGGCTATTCTGAGGTGATAGTTGTCGTTGGAGGATGTGAGCGAGTTGGAGGATT

TAATCTTCCATACACTGAGTGCTACGATCCTGTAACAGGAGAATGGAAGTCTTTGGCTAAGCTTCCAGAA

TTTACCAAATCAGAGTATGCAGTCTGTGCTCTAAGGAATGACATTCTTGTTTCAGGTGGAAGAATCAACA

GCCGTGATGTCTGGATTTATAACTCACAGTTAAATATTTGGATCAGAGTTGCCTCTCTCAATAAAGGCAG

ATGGCGTCACAAAATGGCTGTCCTCCTTGGTAAAGTATATGTTGTCGGTGGCTATGATGGGCAAAACAGA

CTTAGCAGCGTAGAATGTTATGATTCCTTTTCAAATCGATGGACTGAAGTTGCTCCCCTTAAGGAAGCCG

TGAGTTCTCCTGCAGTGACTAGCTGTGTAGGCAAACTGTTTGTGATTGGTGGAGGACCTGATGATAATAC

TTGTTCTGATAAGGTTCAATCTTATGATCCAGAAACCAATTCTTGGCTACTTCGTGCAGCTATCCCAATT

GCCAAAAGGTGTATAACAGCTGTATCCCTAAACAACCTGATCTATGTTGCCGGTGGACTGACCAAGGCAA

TATACTGTTACGATCCAGTTGAAGATTACTGGATGCACGTACAGAATACATTCAGCCGTCAGGAAAACTG

TGGTATGTCTGTGTGTAATGGTAAAATATATATCCTGGGCGGAAGACGGGAAAATGGAGAAGCCACAGAC

ACTATTCTCTGTTATGATCCTGCAACAAGTATCATCACAGGGGTAGCTGCAATGCCCAGGCCAGTGTCCT

ATCATGGCTGTGTGACTATTCATAGATACAATGAGAAATGCTTTAAACTCTGA

>lcl|NM_005561.4_cds_NP_005552.3_1 [gene=LAMP1] [db_xref=CCDS:CCDS41909.1] [protein=lysosome-associated membrane glycoprotein 1 precursor] [protein_id=NP_005552.3] [location=197..1450] [gbkey=CDS]

ATGGCGGCCCCCGGCAGCGCCCGGCGACCCCTGCTGCTGCTACTGCTGTTGCTGCTGCTCGGCCTCATGC

ATTGTGCGTCAGCAGCAATGTTTATGGTGAAAAATGGCAACGGGACCGCGTGCATAATGGCCAACTTCTC

TGCTGCCTTCTCAGTGAACTACGACACCAAGAGTGGCCCTAAGAACATGACCTTTGACCTGCCATCAGAT

GCCACAGTGGTGCTCAACCGCAGCTCCTGTGGAAAAGAGAACACTTCTGACCCCAGTCTCGTGATTGCTT

TTGGAAGAGGACATACACTCACTCTCAATTTCACGAGAAATGCAACACGTTACAGCGTCCAGCTCATGAG

TTTTGTTTATAACTTGTCAGACACACACCTTTTCCCCAATGCGAGCTCCAAAGAAATCAAGACTGTGGAA

TCTATAACTGACATCAGGGCAGATATAGATAAAAAATACAGATGTGTTAGTGGCACCCAGGTCCACATGA

ACAACGTGACCGTAACGCTCCATGATGCCACCATCCAGGCGTACCTTTCCAACAGCAGCTTCAGCCGGGG

AGAGACACGCTGTGAACAAGACAGGCCTTCCCCAACCACAGCGCCCCCTGCGCCACCCAGCCCCTCGCCC

TCACCCGTGCCCAAGAGCCCCTCTGTGGACAAGTACAACGTGAGCGGCACCAACGGGACCTGCCTGCTGG

CCAGCATGGGGCTGCAGCTGAACCTCACCTATGAGAGGAAGGACAACACGACGGTGACAAGGCTTCTCAA

CATCAACCCCAACAAGACCTCGGCCAGCGGGAGCTGCGGCGCCCACCTGGTGACTCTGGAGCTGCACAGC

GAGGGCACCACCGTCCTGCTCTTCCAGTTCGGGATGAATGCAAGTTCTAGCCGGTTTTTCCTACAAGGAA

TCCAGTTGAATACAATTCTTCCTGACGCCAGAGACCCTGCCTTTAAAGCTGCCAACGGCTCCCTGCGAGC

GCTGCAGGCCACAGTCGGCAATTCCTACAAGTGCAACGCGGAGGAGCACGTCCGTGTCACGAAGGCGTTT

TCAGTCAATATATTCAAAGTGTGGGTCCAGGCTTTCAAGGTGGAAGGTGGCCAGTTTGGCTCTGTGGAGG

AGTGTCTGCTGGACGAGAACAGCATGCTGATCCCCATCGCTGTGGGTGGTGCCCTGGCGGGGCTGGTCCT

CATCGTCCTCATCGCCTACCTCGTCGGCAGGAAGAGGAGTCACGCAGGCTACCAGACTATCTAG

>lcl|NM_001122606.1_cds_NP_001116078.1_1 [gene=LAMP2] [db_xref=CCDS:CCDS48159.1] [protein=lysosome-associated membrane glycoprotein 2 isoform C precursor] [protein_id=NP_001116078.1] [location=181..1416] [gbkey=CDS]

ATGGTGTGCTTCCGCCTCTTCCCGGTTCCGGGCTCAGGGCTCGTTCTGGTCTGCCTAGTCCTGGGAGCTG

TGCGGTCTTATGCATTGGAACTTAATTTGACAGATTCAGAAAATGCCACTTGCCTTTATGCAAAATGGCA

GATGAATTTCACAGTACGCTATGAAACTACAAATAAAACTTATAAAACTGTAACCATTTCAGACCATGGC

ACTGTGACATATAATGGAAGCATTTGTGGGGATGATCAGAATGGTCCCAAAATAGCAGTGCAGTTCGGAC

CTGGCTTTTCCTGGATTGCGAATTTTACCAAGGCAGCATCTACTTATTCAATTGACAGCGTCTCATTTTC

CTACAACACTGGTGATAACACAACATTTCCTGATGCTGAAGATAAAGGAATTCTTACTGTTGATGAACTT

TTGGCCATCAGAATTCCATTGAATGACCTTTTTAGATGCAATAGTTTATCAACTTTGGAAAAGAATGATG

TTGTCCAACACTACTGGGATGTTCTTGTACAAGCTTTTGTCCAAAATGGCACAGTGAGCACAAATGAGTT

CCTGTGTGATAAAGACAAAACTTCAACAGTGGCACCCACCATACACACCACTGTGCCATCTCCTACTACA

ACACCTACTCCAAAGGAAAAACCAGAAGCTGGAACCTATTCAGTTAATAATGGCAATGATACTTGTCTGC

TGGCTACCATGGGGCTGCAGCTGAACATCACTCAGGATAAGGTTGCTTCAGTTATTAACATCAACCCCAA

TACAACTCACTCCACAGGCAGCTGCCGTTCTCACACTGCTCTACTTAGACTCAATAGCAGCACCATTAAG

TATCTAGACTTTGTCTTTGCTGTGAAAAATGAAAACCGATTTTATCTGAAGGAAGTGAACATCAGCATGT

ATTTGGTTAATGGCTCCGTTTTCAGCATTGCAAATAACAATCTCAGCTACTGGGATGCCCCCCTGGGAAG

TTCTTATATGTGCAACAAAGAGCAGACTGTTTCAGTGTCTGGAGCATTTCAGATAAATACCTTTGATCTA

AGGGTTCAGCCTTTCAATGTGACACAAGGAAAGTATTCTACAGCTGAAGAATGTTCTGCTGACTCTGACC

TCAACTTTCTTATTCCTGTTGCAGTGGGTGTGGCCTTGGGCTTCCTTATAATTGTTGTCTTTATCTCTTA

TATGATTGGAAGAAGGAAAAGTCGTACTGGTTATCAGTCTGTGTAA

>lcl|NM_181509.3_cds_NP_852610.1_1 [gene=MAP1LC3A] [db_xref=CCDS:CCDS13237.1] [protein=microtubule-associated proteins 1A/1B light chain 3A isoform b] [protein_id=NP_852610.1] [location=147..524] [gbkey=CDS]

ATGAAGATGAGATTCTTCAGTTCTCCATGTGGAAAAGCAGCTGTGGACCCAGCCGACCGCTGTAAGGAGG

TACAGCAGATCCGCGACCAGCACCCCAGCAAAATCCCGGTGATCATCGAGCGCTACAAGGGTGAGAAGCA

GCTGCCCGTCCTGGACAAGACCAAGTTTTTGGTCCCGGACCATGTCAACATGAGCGAGTTGGTCAAGATC

ATCCGGCGCCGCCTGCAGCTGAACCCCACGCAGGCCTTCTTCCTGCTGGTGAACCAGCACAGCATGGTGA

GTGTGTCCACGCCCATCGCGGACATCTACGAGCAGGAGAAAGACGAGGACGGCTTCCTCTATATGGTCTA

CGCCTCCCAGGAAACCTTCGGCTTCTGA

>lcl|NM_022818.5_cds_NP_073729.1_1 [gene=MAP1LC3B] [db_xref=CCDS:CCDS10960.1] [protein=microtubule-associated proteins 1A/1B light chain 3B] [protein_id=NP_073729.1] [location=93..470] [gbkey=CDS]

ATGCCGTCGGAGAAGACCTTCAAGCAGCGCCGCACCTTCGAACAAAGAGTAGAAGATGTCCGACTTATTC

GAGAGCAGCATCCAACCAAAATCCCGGTGATAATAGAACGATACAAGGGTGAGAAGCAGCTTCCTGTTCT

GGATAAAACAAAGTTCCTTGTACCTGACCATGTCAACATGAGTGAGCTCATCAAGATAATTAGAAGGCGC

TTACAGCTCAATGCTAATCAGGCCTTCTTCCTGTTGGTGAACGGACACAGCATGGTCAGCGTCTCCACAC

CAATCTCAGAGGTGTATGAGAGTGAGAAAGATGAAGATGGATTCCTGTACATGGTCTATGCCTCCCAGGA

GACGTTCGGGATGAAATTGTCAGTGTAA

>lcl|NM_001004343.3_cds_NP_001004343.1_1 [gene=MAP1LC3C] [db_xref=CCDS:CCDS31074.1] [protein=microtubule-associated proteins 1A/1B light chain 3C] [protein_id=NP_001004343.1] [location=91..534] [gbkey=CDS]

ATGCCGCCTCCACAGAAAATCCCAAGCGTCAGACCCTTCAAGCAGAGGAAAAGCTTGGCAATCAGACAAG

AGGAAGTTGCTGGAATCCGGGCAAAGTTCCCCAACAAAATCCCGGTGGTAGTGGAGCGCTACCCCAGGGA

GACGTTCCTGCCCCCGCTGGACAAAACCAAGTTCCTGGTCCCGCAGGAGCTGACCATGACCCAGTTCCTC

AGCATCATCCGGAGCCGCATGGTCCTGAGAGCCACGGAAGCCTTTTACTTGCTGGTGAACAACAAGAGCC

TGGTCAGCATGAGCGCAACCATGGCAGAGATCTACAGAGACTACAAGGATGAGGATGGCTTCGTGTACAT

GACCTACGCCTCCCAGGAGACATTTGGCTGCCTGGAGTCAGCAGCCCCCAGGGATGGGAGCAGCCTTGAG

GACAGACCCTGCAATCCTCTCTAG

>lcl|NM_001297555.2_cds_NP_001284484.1_1 [gene=MAP2K7] [db_xref=CCDS:CCDS74277.1] [protein=dual specificity mitogen-activated protein kinase kinase 7 isoform 1] [protein_id=NP_001284484.1] [location=69..1376] [gbkey=CDS]

ATGGCGGCGTCCTCCCTGGAACAGAAGCTGTCCCGCCTGGAAGCAAAGCTGAAGCAGGAGAACCGGGAGG

CCCGGCGGAGGATCGACCTCAACCTGGATATCAGCCCCCAGCGGCCCAGGCCCATTATTGTGATCACTCT

AAGCCCTGCTCCTGCCCCGTCCCAACGAGCAGCCCTGCAGCTCCCGCTGGCCAACGATGGGGGCAGCCGC

TCGCCATCCTCAGAGAGCTCCCCGCAGCACCCCACGCCCCCCGCCCGGCCCCGCCACATGCTGGGGCTCC

CGTCAACCCTGTTCACACCCCGCAGCATGGAGAGCATTGAGATTGACCAGAAGCTGCAGGAGATCATGAA

GCAGACGGGCTACCTGACCATCGGGGGCCAGCGCTACCAGGCAGAAATCAACGACCTGGAGAACTTGGGC

GAGATGGGCAGCGGCACCTGCGGCCAGGTGTGGAAGATGCGCTTCCGGAAGACCGGCCACGTCATTGCCG

TTAAGCAAATGCGGCGCTCCGGGAACAAGGAGGAGAACAAGCGCATCCTCATGGACCTGGATGTGGTGCT

GAAGAGCCACGACTGCCCCTACATCGTGCAGTGCTTTGGGACGTTCATCACCAACACGGACGTCTTCATC

GCCATGGAGCTCATGGGCACCTGCGCTGAGAAGCTCAAGAAGCGGATGCAGGGCCCCATCCCCGAGCGCA

TTCTGGGCAAGATGACAGTGGCGATTGTGAAGGCGCTGTACTACCTGAAGGAGAAGCACGGTGTCATCCA

CCGCGACGTCAAGCCCTCCAACATCCTGCTGGACGAGCGGGGCCAGATCAAGCTCTGCGACTTCGGCATC

AGCGGCCGCCTGGTGGACTCCAAAGCCAAGACGCGGAGCGCCGGCTGTGCCGCCTACATGGCACCCGAGC

GCATTGACCCCCCAGACCCCACCAAGCCGGACTATGACATCCGGGCCGACGTATGGAGCCTGGGCATCTC

GTTGGTGGAGCTGGCAACAGGACAGTTTCCCTACAAGAACTGCAAGACGGACTTTGAGGTCCTCACCAAA

GTCCTACAGGAAGAGCCCCCGCTTCTGCCCGGACACATGGGCTTCTCGGGGGACTTCCAGTCCTTCGTCA

AAGACTGCCTTACTAAAGATCACAGGAAGAGACCAAAGTATAATAAGCTACTTGAACACAGCTTCATCAA

GCGCTACGAGACGCTGGAGGTGGACGTGGCGTCCTGGTTCAAGGATGTCATGGCGAAGACTGAGTCACCG

CGGACTAGCGGCGTCCTGAGCCAGCCCCACCTGCCCTTCTTCAGGTAG

>lcl|NM_002745.5_cds_NP_002736.3_1 [gene=MAPK1] [db_xref=CCDS:CCDS13795.1] [protein=mitogen-activated protein kinase 1] [protein_id=NP_002736.3] [location=206..1288] [gbkey=CDS]

ATGGCGGCGGCGGCGGCGGCGGGCGCGGGCCCGGAGATGGTCCGCGGGCAGGTGTTCGACGTGGGGCCGC

GCTACACCAACCTCTCGTACATCGGCGAGGGCGCCTACGGCATGGTGTGCTCTGCTTATGATAATGTCAA

CAAAGTTCGAGTAGCTATCAAGAAAATCAGCCCCTTTGAGCACCAGACCTACTGCCAGAGAACCCTGAGG

GAGATAAAAATCTTACTGCGCTTCAGACATGAGAACATCATTGGAATCAATGACATTATTCGAGCACCAA

CCATCGAGCAAATGAAAGATGTATATATAGTACAGGACCTCATGGAAACAGATCTTTACAAGCTCTTGAA

GACACAACACCTCAGCAATGACCATATCTGCTATTTTCTCTACCAGATCCTCAGAGGGTTAAAATATATC

CATTCAGCTAACGTTCTGCACCGTGACCTCAAGCCTTCCAACCTGCTGCTCAACACCACCTGTGATCTCA

AGATCTGTGACTTTGGCCTGGCCCGTGTTGCAGATCCAGACCATGATCACACAGGGTTCCTGACAGAATA

TGTGGCCACACGTTGGTACAGGGCTCCAGAAATTATGTTGAATTCCAAGGGCTACACCAAGTCCATTGAT

ATTTGGTCTGTAGGCTGCATTCTGGCAGAAATGCTTTCTAACAGGCCCATCTTTCCAGGGAAGCATTATC

TTGACCAGCTGAACCACATTTTGGGTATTCTTGGATCCCCATCACAAGAAGACCTGAATTGTATAATAAA

TTTAAAAGCTAGGAACTATTTGCTTTCTCTTCCACACAAAAATAAGGTGCCATGGAACAGGCTGTTCCCA

AATGCTGACTCCAAAGCTCTGGACTTATTGGACAAAATGTTGACATTCAACCCACACAAGAGGATTGAAG

TAGAACAGGCTCTGGCCCACCCATATCTGGAGCAGTATTACGACCCGAGTGACGAGCCCATCGCCGAAGC

ACCATTCAAGTTCGACATGGAATTGGATGACTTGCCTAAGGAAAAGCTCAAAGAACTAATTTTTGAAGAG

ACTGCTAGATTCCAGCCAGGATACAGATCTTAA

>lcl|NM_001040056.3_cds_NP_001035145.1_1 [gene=MAPK3] [db_xref=CCDS:CCDS42149.1] [protein=mitogen-activated protein kinase 3 isoform 2] [protein_id=NP_001035145.1] [location=12..1085] [gbkey=CDS]

ATGGCGGCGGCGGCGGCTCAGGGGGGCGGGGGCGGGGAGCCCCGTAGAACCGAGGGGGTCGGCCCGGGGG

TCCCGGGGGAGGTGGAGATGGTGAAGGGGCAGCCGTTCGACGTGGGCCCGCGCTACACGCAGTTGCAGTA

CATCGGCGAGGGCGCGTACGGCATGGTCAGCTCGGCCTATGACCACGTGCGCAAGACTCGCGTGGCCATC

AAGAAGATCAGCCCCTTCGAACATCAGACCTACTGCCAGCGCACGCTCCGGGAGATCCAGATCCTGCTGC

GCTTCCGCCATGAGAATGTCATCGGCATCCGAGACATTCTGCGGGCGTCCACCCTGGAAGCCATGAGAGA

TGTCTACATTGTGCAGGACCTGATGGAGACTGACCTGTACAAGTTGCTGAAAAGCCAGCAGCTGAGCAAT

GACCATATCTGCTACTTCCTCTACCAGATCCTGCGGGGCCTCAAGTACATCCACTCCGCCAACGTGCTCC

ACCGAGATCTAAAGCCCTCCAACCTGCTCATCAACACCACCTGCGACCTTAAGATTTGTGATTTCGGCCT

GGCCCGGATTGCCGATCCTGAGCATGACCACACCGGCTTCCTGACGGAGTATGTGGCTACGCGCTGGTAC

CGGGCCCCAGAGATCATGCTGAACTCCAAGGGCTATACCAAGTCCATCGACATCTGGTCTGTGGGCTGCA

TTCTGGCTGAGATGCTCTCTAACCGGCCCATCTTCCCTGGCAAGCACTACCTGGATCAGCTCAACCACAT

TCTGGGCATCCTGGGCTCCCCATCCCAGGAGGACCTGAATTGTATCATCAACATGAAGGCCCGAAACTAC

CTACAGTCTCTGCCCTCCAAGACCAAGGTGGCTTGGGCCAAGCTTTTCCCCAAGTCAGACTCCAAAGCCC

TTGACCTGCTGGACCGGATGTTAACCTTTAACCCCAATAAACGGATCACAGTGGAGGAAGCGCTGGCTCA

CCCCTACCTGGAGCAGTACTATGACCCGACGGATGAGGTGGGCCAGTCCCCAGCAGCAGTGGGGCTGGGG

GCAGGGGAGCAGGGGGGCACGTAG

>lcl|NM_001278547.2_cds_NP_001265476.1_1 [gene=MAPK8] [db_xref=CCDS:CCDS7223.1] [protein=mitogen-activated protein kinase 8 isoform beta2] [protein_id=NP_001265476.1] [location=195..1478] [gbkey=CDS]

ATGAGCAGAAGCAAGCGTGACAACAATTTTTATAGTGTAGAGATTGGAGATTCTACATTCACAGTCCTGA

AACGATATCAGAATTTAAAACCTATAGGCTCAGGAGCTCAAGGAATAGTATGCGCAGCTTATGATGCCAT

TCTTGAAAGAAATGTTGCAATCAAGAAGCTAAGCCGACCATTTCAGAATCAGACTCATGCCAAGCGGGCC

TACAGAGAGCTAGTTCTTATGAAATGTGTTAATCACAAAAATATAATTGGCCTTTTGAATGTTTTCACAC

CACAGAAATCCCTAGAAGAATTTCAAGATGTTTACATAGTCATGGAGCTCATGGATGCAAATCTTTGCCA

AGTGATTCAGATGGAGCTAGATCATGAAAGAATGTCCTACCTTCTCTATCAGATGCTGTGTGGAATCAAG

CACCTTCATTCTGCTGGAATTATTCATCGGGACTTAAAGCCCAGTAATATAGTAGTAAAATCTGATTGCA

CTTTGAAGATTCTTGACTTCGGTCTGGCCAGGACTGCAGGAACGAGTTTTATGATGACGCCTTATGTAGT

GACTCGCTACTACAGAGCACCCGAGGTCATCCTTGGCATGGGCTACAAGGAAAACGTTGACATTTGGTCA

GTTGGGTGCATCATGGGAGAAATGATCAAAGGTGGTGTTTTGTTCCCAGGTACAGATCATATTGATCAGT

GGAATAAAGTTATTGAACAGCTTGGAACACCATGTCCTGAATTCATGAAGAAACTGCAACCAACAGTAAG

GACTTACGTTGAAAACAGACCTAAATATGCTGGATATAGCTTTGAGAAACTCTTCCCTGATGTCCTTTTC

CCAGCTGACTCAGAACACAACAAACTTAAAGCCAGTCAGGCAAGGGATTTGTTATCCAAAATGCTGGTAA

TAGATGCATCTAAAAGGATCTCTGTAGATGAAGCTCTCCAACACCCGTACATCAATGTCTGGTATGATCC

TTCTGAAGCAGAAGCTCCACCACCAAAGATCCCTGACAAGCAGTTAGATGAAAGGGAACACACAATAGAA

GAGTGGAAAGAATTGATATATAAGGAAGTTATGGACTTGGAGGAGAGAACCAAGAATGGAGTTATACGGG

GGCAGCCCTCTCCTTTAGGTGCAGCAGTGATCAATGGCTCTCAGCATCCATCATCATCGTCGTCTGTCAA

TGATGTGTCTTCAATGTCAACAGATCCGACTTTGGCCTCTGATACAGACAGCAGTCTAGAAGCAGCAGCT

GGGCCTCTGGGCTGCTGTAGATGA

>lcl|NM_005456.4_cds_NP_005447.1_1 [gene=MAPK8IP1] [db_xref=CCDS:CCDS7916.1] [protein=C-Jun-amino-terminal kinase-interacting protein 1] [protein_id=NP_005447.1] [location=171..2306] [gbkey=CDS]

ATGGCGGAGCGAGAAAGCGGCGGCCTGGGAGGGGGGGCCGCGTCCCCGCCCGCCGCCTCCCCGTTCCTGG

GGCTGCACATCGCTTCGCCTCCCAATTTCAGGCTCACCCATGACATCAGCCTGGAGGAGTTTGAGGATGA

AGACCTCTCGGAGATCACTGATGAGTGTGGCATCAGCTTACAGTGCAAAGACACCCTGTCCTTACGGCCC

CCGCGCGCCGGGCTGCTCTCTGCGGGCGGCGGCGGCGCGGGGAGCCGGTTGCAGGCCGAGATGCTGCAGA

TGGACCTGATCGACGCGACGGGGGACACTCCCGGGGCCGAGGACGACGAGGAGGACGACGACGAGGAGCG

CGCGGCCCGGCGGCCGGGAGCGGGGCCGCCCAAGGCCGAGTCCGGCCAGGAGCCGGCGTCCCGCGGCCAG

GGCCAGAGCCAAGGCCAGAGCCAGGGCCCGGGCAGCGGGGACACGTACCGGCCCAAGCGGCCCACCACGC

TCAACCTCTTTCCGCAGGTGCCGCGGTCTCAGGACACACTGAATAATAATTCTCTGGGCAAAAAGCACAG

TTGGCAGGATCGGGTGTCTCGATCATCCTCACCCCTGAAGACAGGGGAGCAGACACCACCGCATGAACAC

ATCTGCCTGAGCGATGAGCTGCCCCCCCAGAGCGGCCCCGCCCCCACCACAGATCGAGGCACCTCCACCG

ACAGCCCTTGCCGCCGCAGCACAGCCACCCAGATGGCACCTCCGGGTGGTCCCCCTGCTGCCCCGCCTGG

GGGTCGGGGCCACTCGCATCGAGACCGAATCCACTACCAGGCCGATGTGCGACTAGAGGCCACTGAGGAG

ATCTACCTGACCCCAGTGCAGAGGCCCCCAGACGCTGCAGAGCCCACCTCCGCCTTCCTGCCGCCCACTG

AGAGCCGGATGTCAGTCAGCTCCGATCCAGACCCTGCCGCCTACCCCTCCACGGCAGGGCGGCCGCACCC

CTCCATCAGTGAAGAGGAAGAGGGCTTCGACTGCCTGTCGTCCCCAGAGCGGGCTGAGCCCCCAGGCGGA

GGGTGGCGGGGGAGCCTGGGGGAGCCGCCGCCACCTCCACGGGCCTCTCTGAGCTCGGACACCAGCGCCC

TGTCCTATGACTCTGTCAAGTACACGCTGGTGGTAGATGAGCATGCACAGCTGGAGCTGGTGAGCCTGCG

GCCGTGCTTCGGAGACTACAGTGACGAGAGTGACTCTGCCACCGTCTATGACAACTGTGCCTCCGTCTCC

TCGCCCTATGAGTCGGCCATCGGAGAGGAATATGAGGAGGCCCCGCGGCCCCAGCCCCCTGCCTGCCTCT

CCGAGGACTCCACGCCTGATGAACCCGACGTCCATTTCTCCAAGAAATTCCTGAACGTCTTCATGAGTGG

CCGCTCCCGCTCCTCCAGTGCTGAGTCCTTCGGGCTGTTCTCCTGCATCATCAACGGGGAGGAGCAGGAG

CAGACCCACCGGGCCATATTCAGGTTTGTGCCTCGACACGAAGACGAACTTGAGCTGGAAGTGGATGACC

CTCTGCTAGTGGAGCTCCAGGCTGAAGACTACTGGTACGAGGCCTACAACATGCGCACTGGTGCCCGGGG

TGTCTTTCCTGCCTATTACGCCATCGAGGTCACCAAGGAGCCCGAGCACATGGCAGCCCTGGCCAAAAAC

AGTGACTGGGTGGACCAGTTCCGGGTGAAGTTCCTGGGCTCAGTCCAGGTTCCCTATCACAAGGGCAATG

ACGTCCTCTGTGCTGCTATGCAAAAGATTGCCACCACCCGCCGGCTCACCGTGCACTTTAACCCGCCCTC

CAGCTGTGTCCTGGAGATCAGCGTGCGGGGTGTGAAGATAGGCGTCAAGGCCGATGACTCCCAGGAGGCC

AAGGGGAATAAATGTAGCCACTTTTTCCAGTTAAAAAACATCTCTTTCTGCGGATATCATCCAAAGAACA

ACAAGTACTTTGGGTTCATCACCAAGCACCCCGCCGACCACCGGTTTGCCTGCCACGTCTTTGTGTCTGA

AGACTCCACCAAAGCCCTGGCAGAGTCCGTGGGGAGAGCATTCCAGCAGTTCTACAAGCAGTTTGTGGAG

TACACCTGCCCCACAGAAGATATCTACCTGGAGTAG

>lcl|NM_001135044.2_cds_NP_001128516.1_1 [gene=MAPK9] [db_xref=CCDS:CCDS47356.1] [protein=mitogen-activated protein kinase 9 isoform JNK2 gamma] [protein_id=NP_001128516.1] [location=284..1012] [gbkey=CDS]

ATGAGCGACAGTAAATGTGACAGTCAGTTTTATAGTGTGCAAGTGGCAGACTCAACCTTCACTGTCCTAA

AACGTTACCAGCAGCTGAAACCAATTGGCTCTGGGGCCCAAGGGATTGTTTGTGCTGCATTTGATACAGT

TCTTGGGATAAATGTTGCAGTCAAGAAACTAAGCCGTCCTTTTCAGAACCAAACTCATGCAAAGAGAGCT

TATCGTGAACTTGTCCTCTTAAAATGTGTCAATCATAAAAATATAATTAGTTTGTTAAATGTGTTTACAC

CACAAAAAACTCTAGAAGAATTTCAAGATGTGTATTTGGTTATGGAATTAATGGATGCTAACTTATGTCA

GGTTATTCACATGGAGCTGGATCATGAAAGAATGTCCTACCTTCTTTACCAGATGCTTTGTGGTATTAAA

CATCTGCATTCAGCTGGTATAATTCATAGAGATTTGAAGCCTAGCAACATTGTTGTGAAATCAGACTGCA

CCCTGAAGATCCTTGACTTTGGCCTGGCCCGGACAGCGTGCACTAACTTCATGATGACCCCTTACGTGGT

GACACGGTACTACCGGGCGCCCGAAGTCATCCTGGGTATGGGCTACAAAGAGAACGTTGATATCTGGTCA

GTGGGTTGCATCATGGGAGAGCTGGTGAAAGGTTGTGTGATATTCCAAGGCACTGACCGTATCCTTCCCC

GCGACCTTGGCCCCGCCATGCTTTCTTAA

>lcl|NM_015884.4_cds_NP_056968.1_1 [gene=MBTPS2] [db_xref=CCDS:CCDS14201.1] [protein=membrane-bound transcription factor site-2 protease] [protein_id=NP_056968.1] [location=119..1678] [gbkey=CDS]

ATGATTCCGGTGTCGCTGGTGGTGGTGGTGGTGGGTGGCTGGACTGTCGTCTACCTGACCGACTTGGTGC

TGAAGTCATCTGTCTATTTTAAACATTCTTATGAAGACTGGCTGGAAAACAACGGACTGAGCATCTCCCC

TTTCCACATAAGATGGCAAACTGCTGTTTTCAATCGTGCCTTTTACAGTTGGGGACGGCGGAAAGCAAGG

ATGCTTTACCAATGGTTCAATTTTGGAATGGTGTTTGGCGTAATTGCCATGTTTAGCTCATTTTTTCTCC

TTGGAAAAACGCTGATGCAGACTTTGGCACAAATGATGGCTGACTCTCCCTCTTCTTATTCTTCCTCCTC

TTCTTCCTCTTCCTCCTCTTCTTCCTCTTCCTCTTCTTCATCTTCTTCCTCTTCCTCGCTTCACAATGAA

CAGGTGTTACAAGTTGTGGTTCCTGGTATAAATTTACCCGTCAATCAACTGACCTATTTCTTCACGGCAG

TTCTCATTAGTGGTGTTGTACATGAAATTGGACATGGGATAGCAGCTATTAGGGAACAAGTTCGATTTAA

TGGCTTTGGGATTTTTCTCTTCATTATTTATCCTGGAGCATTTGTTGATCTGTTCACCACTCATTTGCAA

CTTATATCGCCAGTCCAGCAGCTAAGGATATTTTGTGCAGGTATCTGGCATAATTTTGTCCTTGCACTCT

TGGGTATTTTAGCTCTTGTTCTCCTCCCAGTAATTCTCTTGCCATTTTACTACACTGGAGTTGGGGTGCT

CATCACTGAAGTTGCTGAGGACTCTCCTGCCATTGGACCCAGAGGCCTTTTTGTGGGAGACCTTGTCACC

CATCTACAGGATTGTCCTGTTACTAATGTGCAAGATTGGAATGAATGTTTAGATACCATCGCCTATGAGC

CCCAAATTGGTTACTGTATAAGTGCATCAACTTTACAGCAGTTAAGTTTCCCAGTTAGAGCATACAAACG

ACTAGATGGTTCAACTGAATGCTGTAACAATCACAGCCTCACAGATGTGTGCTTTTCCTACAGAAATAAT

TTTAATAAGCGTTTGCATACATGTCTTCCTGCCCGGAAAGCAGTTGAAGCAACTCAAGTTTGCAGAACCA

ATAAAGACTGTAAAAAAAGCTCAAGTTCAAGTTTCTGTATAATACCTTCTTTGGAAACTCACACTCGCTT

AATAAAAGTAAAACACCCACCTCAGATTGATATGTTATACGTAGGACATCCTCTGCATCTTCACTACACA

GTGAGCATCACCAGTTTTATCCCACGTTTTAACTTTCTAAGCATAGATCTGCCAGTGGTTGTGGAGACAT

TTGTCAAGTACCTGATTTCCCTCTCAGGAGCTCTGGCTATTGTTAATGCAGTACCCTGCTTTGCTTTGGA

TGGACAATGGATTCTAAACTCTTTCTTGGATGCCACCCTTACCTCAGTGATTGGAGACAATGATGTCAAA

GATCTAATAGGGTTTTTCATCTTGCTGGGTGGCAGTGTACTTTTGGCTGCCAATGTGACCCTGGGACTCT

GGATGGTTACAGCACGGTAA

>lcl|NM_022372.6_cds_NP_071767.3_1 [gene=MLST8] [db_xref=CCDS:CCDS10462.2] [protein=target of rapamycin complex subunit LST8 isoform a] [protein_id=NP_071767.3] [location=115..1095] [gbkey=CDS]

ATGAACACCTCCCCAGGCACGGTGGGCAGTGACCCGGTCATCCTGGCCACTGCAGGCTACGACCACACCG

TGCGCTTCTGGCAGGCCCACAGCGGCATCTGCACCCGGACGGTGCAGCACCAGGACTCCCAGGTGAATGC

CTTGGAGGTCACACCGGACCGCAGCATGATTGCTGCTGCAGGTTACCAGCACATCCGCATGTATGATCTC

AACTCCAATAACCCTAACCCCATCATCAGCTACGACGGCGTCAACAAGAACATCGCGTCTGTGGGCTTCC

ACGAAGACGGCCGCTGGATGTACACGGGCGGCGAGGACTGCACAGCCAGGATCTGGGACCTCAGGTCCCG

GAACCTGCAGTGCCAGCGGATCTTCCAGGTGAACGCACCCATTAACTGCGTGTGCCTGCACCCCAACCAG

GCAGAGCTCATCGTGGGTGACCAGAGCGGGGCTATCCACATCTGGGACTTGAAAACAGACCACAACGAGC

AGCTGATCCCTGAGCCCGAGGTCTCCATCACGTCCGCCCACATCGATCCCGACGCCAGCTACATGGCAGC

TGTCAATAGCACCGGAAACTGCTATGTCTGGAATCTGACGGGGGGCATTGGTGACGAGGTGACCCAGCTC

ATCCCCAAGACTAAGATCCCTGCCCACACGCGCTACGCCCTGCAGTGTCGCTTCAGCCCCGACTCCACGC

TCCTCGCCACCTGCTCGGCTGATCAGACGTGCAAGATCTGGAGGACGTCCAACTTCTCCCTGATGACGGA

GCTGAGCATCAAGAGCGGCAACCCCGGGGAGTCCTCCCGCGGCTGGATGTGGGGCTGCGCCTTCTCGGGG

GACTCCCAGTACATCGTCACTGCTTCCTCGGACAACCTGGCCCGGCTCTGGTGTGTGGAGACTGGAGAGA

TCAAGAGAGAGTATGGCGGCCACCAGAAGGCTGTTGTCTGCCTGGCCTTCAATGACAGTGTGCTGGGCTA

G

>lcl|NM_022485.5_cds_NP_071930.2_1 [gene=MTMR14] [db_xref=CCDS:CCDS43045.1] [protein=myotubularin-related protein 14 isoform 3] [protein_id=NP_071930.2] [location=80..1696] [gbkey=CDS]

ATGGCCGGCGCTCGGGCCGCCGCCGCCGCTGCCTCGGCGGGGTCCTCGGCCTCTTCAGGCAACCAGCCGC

CTCAGGAGCTGGGGCTTGGGGAGCTGCTGGAGGAGTTCTCCCGGACTCAGTACCGGGCCAAGGATGGCAG

CGGGACCGGCGGCTCTAAGGTTGAGCGCATTGAGAAGAGATGTCTGGAGCTGTTTGGCCGAGACTACTGT

TTCAGCGTGATTCCAAACACGAATGGGGATATCTGTGGCCACTATCCCCGGCACATCGTGTTCCTGGAGT

ATGAGAGTTCTGAGAAGGAGAAAGACACGTTTGAGAGTACCGTACAGGTGAGCAAGTTGCAAGACCTCAT

CCACCGCAGCAAGATGGCCCGGTGCAGAGGACGGTTTGTCTGCCCAGTAATCCTGTTCAAGGGCAAGCAC

ATTTGCAGGTCGGCCACACTGGCTGGATGGGGAGAGCTGTATGGACGCTCAGGCTACAACTATTTTTTCT

CAGGGGGTGCAGATGATGCCTGGGCAGATGTGGAGGACGTCACGGAGGAGGACTGTGCTCTTCGAAGTGG

TGACACGCATCTTTTTGATAAGGTCAGAGGCTATGACATCAAGCTGCTTCGATACCTGTCAGTCAAATAC

ATCTGTGACCTGATGGTGGAGAACAAGAAGGTGAAGTTTGGCATGAATGTAACCTCCTCTGAGAAGGTGG

ACAAAGCCCAGCGCTATGCCGACTTCACTCTCCTCTCCATCCCGTATCCAGGCTGTGAATTTTTCAAGGA

ATATAAAGATCGGGATTACATGGCAGAAGGGCTCATATTTAACTGGAAGCAGGACTACGTTGATGCCCCA

TTGAGCATCCCCGACTTCCTGACTCACTCTCTGAACATTGACTGGAGCCAGTATCAGTGTTGGGATCTGG

TGCAACAAACACAAAACTACCTGAAGCTGCTGCTTTCCTTAGTTAACAGTGATGATGACAGCGGGCTGCT

GGTACACTGTATCTCAGGCTGGGATCGGACCCCCCTCTTCATCTCCCTCCTGCGCCTTTCCTTGTGGGCT

GATGGGCTCATCCACACGTCCCTGAAGCCCACTGAGATCCTCTACCTCACTGTGGCCTATGACTGGTTCC

TCTTCGGGCACATGTTGGTAGATCGGCTCAGCAAAGGGGAGGAGATTTTCTTCTTCTGCTTCAATTTTTT

GAAGCATATTACCTCCGAGGAGTTCTCTGCTCTGAAGACCCAGAGGAGGAAGAGTTTGCCAGCCCGGGAT

GGAGGCTTCACCCTGGAAGACATCTGCATGCTGAGACGAAAGGACCGTGGCAGCACCACCAGCCTTGGCA

GCGACTTCTCCCTGGTCATGGAGAGTTCCCCAGGAGCCACTGGGAGCTTCACCTATGAGGCCGTGGAGCT

GGTCCCAGCAGGAGCGCCAACTCAGGCAGCTTGGCTTGCAGCCCTGAGTGATCGAGAGACTCGGCTGCAG

GAGGTGCGCTCAGCCTTCTTGGCTGCGTACAGCAGCACAGTGGGGCTTCGGGCAGTAGCCCCCAGTCCTT

CCGGTGCCATCGGGGGCCTGCTGGAGCAATTTGCCCGTGGTGTTGGACTCCGGAGCATCAGCAGCAATGC

CTTGTGA

>lcl|NM_004958.4_cds_NP_004949.1_1 [gene=MTOR] [db_xref=CCDS:CCDS127.1] [protein=serine/threonine-protein kinase mTOR isoform 1] [protein_id=NP_004949.1] [location=122..7771] [gbkey=CDS]

ATGCTTGGAACCGGACCTGCCGCCGCCACCACCGCTGCCACCACATCTAGCAATGTGAGCGTCCTGCAGC

AGTTTGCCAGTGGCCTAAAGAGCCGGAATGAGGAAACCAGGGCCAAAGCCGCCAAGGAGCTCCAGCACTA

TGTCACCATGGAACTCCGAGAGATGAGTCAAGAGGAGTCTACTCGCTTCTATGACCAACTGAACCATCAC

ATTTTTGAATTGGTTTCCAGCTCAGATGCCAATGAGAGGAAAGGTGGCATCTTGGCCATAGCTAGCCTCA

TAGGAGTGGAAGGTGGGAATGCCACCCGAATTGGCAGATTTGCCAACTATCTTCGGAACCTCCTCCCCTC

CAATGACCCAGTTGTCATGGAAATGGCATCCAAGGCCATTGGCCGTCTTGCCATGGCAGGGGACACTTTT

ACCGCTGAGTACGTGGAATTTGAGGTGAAGCGAGCCCTGGAATGGCTGGGTGCTGACCGCAATGAGGGCC

GGAGACATGCAGCTGTCCTGGTTCTCCGTGAGCTGGCCATCAGCGTCCCTACCTTCTTCTTCCAGCAAGT

GCAACCCTTCTTTGACAACATTTTTGTGGCCGTGTGGGACCCCAAACAGGCCATCCGTGAGGGAGCTGTA

GCCGCCCTTCGTGCCTGTCTGATTCTCACAACCCAGCGTGAGCCGAAGGAGATGCAGAAGCCTCAGTGGT

ACAGGCACACATTTGAAGAAGCAGAGAAGGGATTTGATGAGACCTTGGCCAAAGAGAAGGGCATGAATCG

GGATGATCGGATCCATGGAGCCTTGTTGATCCTTAACGAGCTGGTCCGAATCAGCAGCATGGAGGGAGAG

CGTCTGAGAGAAGAAATGGAAGAAATCACACAGCAGCAGCTGGTACACGACAAGTACTGCAAAGATCTCA

TGGGCTTCGGAACAAAACCTCGTCACATTACCCCCTTCACCAGTTTCCAGGCTGTACAGCCCCAGCAGTC

AAATGCCTTGGTGGGGCTGCTGGGGTACAGCTCTCACCAAGGCCTCATGGGATTTGGGACCTCCCCCAGT

CCAGCTAAGTCCACCCTGGTGGAGAGCCGGTGTTGCAGAGACTTGATGGAGGAGAAATTTGATCAGGTGT

GCCAGTGGGTGCTGAAATGCAGGAATAGCAAGAACTCGCTGATCCAAATGACAATCCTTAATTTGTTGCC

CCGCTTGGCTGCATTCCGACCTTCTGCCTTCACAGATACCCAGTATCTCCAAGATACCATGAACCATGTC

CTAAGCTGTGTCAAGAAGGAGAAGGAACGTACAGCGGCCTTCCAAGCCCTGGGGCTACTTTCTGTGGCTG

TGAGGTCTGAGTTTAAGGTCTATTTGCCTCGCGTGCTGGACATCATCCGAGCGGCCCTGCCCCCAAAGGA

CTTCGCCCATAAGAGGCAGAAGGCAATGCAGGTGGATGCCACAGTCTTCACTTGCATCAGCATGCTGGCT

CGAGCAATGGGGCCAGGCATCCAGCAGGATATCAAGGAGCTGCTGGAGCCCATGCTGGCAGTGGGACTAA

GCCCTGCCCTCACTGCAGTGCTCTACGACCTGAGCCGTCAGATTCCACAGCTAAAGAAGGACATTCAAGA

TGGGCTACTGAAAATGCTGTCCCTGGTCCTTATGCACAAACCCCTTCGCCACCCAGGCATGCCCAAGGGC

CTGGCCCATCAGCTGGCCTCTCCTGGCCTCACGACCCTCCCTGAGGCCAGCGATGTGGGCAGCATCACTC

TTGCCCTCCGAACGCTTGGCAGCTTTGAATTTGAAGGCCACTCTCTGACCCAATTTGTTCGCCACTGTGC

GGATCATTTCCTGAACAGTGAGCACAAGGAGATCCGCATGGAGGCTGCCCGCACCTGCTCCCGCCTGCTC

ACACCCTCCATCCACCTCATCAGTGGCCATGCTCATGTGGTTAGCCAGACCGCAGTGCAAGTGGTGGCAG

ATGTGCTTAGCAAACTGCTCGTAGTTGGGATAACAGATCCTGACCCTGACATTCGCTACTGTGTCTTGGC

GTCCCTGGACGAGCGCTTTGATGCACACCTGGCCCAGGCGGAGAACTTGCAGGCCTTGTTTGTGGCTCTG

AATGACCAGGTGTTTGAGATCCGGGAGCTGGCCATCTGCACTGTGGGCCGACTCAGTAGCATGAACCCTG

CCTTTGTCATGCCTTTCCTGCGCAAGATGCTCATCCAGATTTTGACAGAGTTGGAGCACAGTGGGATTGG

AAGAATCAAAGAGCAGAGTGCCCGCATGCTGGGGCACCTGGTCTCCAATGCCCCCCGACTCATCCGCCCC

TACATGGAGCCTATTCTGAAGGCATTAATTTTGAAACTGAAAGATCCAGACCCTGATCCAAACCCAGGTG

TGATCAATAATGTCCTGGCAACAATAGGAGAATTGGCACAGGTTAGTGGCCTGGAAATGAGGAAATGGGT

TGATGAACTTTTTATTATCATCATGGACATGCTCCAGGATTCCTCTTTGTTGGCCAAAAGGCAGGTGGCT

CTGTGGACCCTGGGACAGTTGGTGGCCAGCACTGGCTATGTAGTAGAGCCCTACAGGAAGTACCCTACTT

TGCTTGAGGTGCTACTGAATTTTCTGAAGACTGAGCAGAACCAGGGTACACGCAGAGAGGCCATCCGTGT

GTTAGGGCTTTTAGGGGCTTTGGATCCTTACAAGCACAAAGTGAACATTGGCATGATAGACCAGTCCCGG

GATGCCTCTGCTGTCAGCCTGTCAGAATCCAAGTCAAGTCAGGATTCCTCTGACTATAGCACTAGTGAAA

TGCTGGTCAACATGGGAAACTTGCCTCTGGATGAGTTCTACCCAGCTGTGTCCATGGTGGCCCTGATGCG

GATCTTCCGAGACCAGTCACTCTCTCATCATCACACCATGGTTGTCCAGGCCATCACCTTCATCTTCAAG

TCCCTGGGACTCAAATGTGTGCAGTTCCTGCCCCAGGTCATGCCCACGTTCCTTAACGTCATTCGAGTCT

GTGATGGGGCCATCCGGGAATTTTTGTTCCAGCAGCTGGGAATGTTGGTGTCCTTTGTGAAGAGCCACAT

CAGACCTTATATGGATGAAATAGTCACCCTCATGAGAGAATTCTGGGTCATGAACACCTCAATTCAGAGC

ACGATCATTCTTCTCATTGAGCAAATTGTGGTAGCTCTTGGGGGTGAATTTAAGCTCTACCTGCCCCAGC

TGATCCCACACATGCTGCGTGTCTTCATGCATGACAACAGCCCAGGCCGCATTGTCTCTATCAAGTTACT

GGCTGCAATCCAGCTGTTTGGCGCCAACCTGGATGACTACCTGCATTTACTGCTGCCTCCTATTGTTAAG

TTGTTTGATGCCCCTGAAGCTCCACTGCCATCTCGAAAGGCAGCGCTAGAGACTGTGGACCGCCTGACGG

AGTCCCTGGATTTCACTGACTATGCCTCCCGGATCATTCACCCTATTGTTCGAACACTGGACCAGAGCCC

AGAACTGCGCTCCACAGCCATGGACACGCTGTCTTCACTTGTTTTTCAGCTGGGGAAGAAGTACCAAATT

TTCATTCCAATGGTGAATAAAGTTCTGGTGCGACACCGAATCAATCATCAGCGCTATGATGTGCTCATCT

GCAGAATTGTCAAGGGATACACACTTGCTGATGAAGAGGAGGATCCTTTGATTTACCAGCATCGGATGCT

TAGGAGTGGCCAAGGGGATGCATTGGCTAGTGGACCAGTGGAAACAGGACCCATGAAGAAACTGCACGTC

AGCACCATCAACCTCCAAAAGGCCTGGGGCGCTGCCAGGAGGGTCTCCAAAGATGACTGGCTGGAATGGC

TGAGACGGCTGAGCCTGGAGCTGCTGAAGGACTCATCATCGCCCTCCCTGCGCTCCTGCTGGGCCCTGGC

ACAGGCCTACAACCCGATGGCCAGGGATCTCTTCAATGCTGCATTTGTGTCCTGCTGGTCTGAACTGAAT

GAAGATCAACAGGATGAGCTCATCAGAAGCATCGAGTTGGCCCTCACCTCACAAGACATCGCTGAAGTCA

CACAGACCCTCTTAAACTTGGCTGAATTCATGGAACACAGTGACAAGGGCCCCCTGCCACTGAGAGATGA

CAATGGCATTGTTCTGCTGGGTGAGAGAGCTGCCAAGTGCCGAGCATATGCCAAAGCACTACACTACAAA

GAACTGGAGTTCCAGAAAGGCCCCACCCCTGCCATTCTAGAATCTCTCATCAGCATTAATAATAAGCTAC

AGCAGCCGGAGGCAGCGGCCGGAGTGTTAGAATATGCCATGAAACACTTTGGAGAGCTGGAGATCCAGGC

TACCTGGTATGAGAAACTGCACGAGTGGGAGGATGCCCTTGTGGCCTATGACAAGAAAATGGACACCAAC

AAGGACGACCCAGAGCTGATGCTGGGCCGCATGCGCTGCCTCGAGGCCTTGGGGGAATGGGGTCAACTCC

ACCAGCAGTGCTGTGAAAAGTGGACCCTGGTTAATGATGAGACCCAAGCCAAGATGGCCCGGATGGCTGC

TGCAGCTGCATGGGGTTTAGGTCAGTGGGACAGCATGGAAGAATACACCTGTATGATCCCTCGGGACACC

CATGATGGGGCATTTTATAGAGCTGTGCTGGCACTGCATCAGGACCTCTTCTCCTTGGCACAACAGTGCA

TTGACAAGGCCAGGGACCTGCTGGATGCTGAATTAACTGCGATGGCAGGAGAGAGTTACAGTCGGGCATA

TGGGGCCATGGTTTCTTGCCACATGCTGTCCGAGCTGGAGGAGGTTATCCAGTACAAACTTGTCCCCGAG

CGACGAGAGATCATCCGCCAGATCTGGTGGGAGAGACTGCAGGGCTGCCAGCGTATCGTAGAGGACTGGC

AGAAAATCCTTATGGTGCGGTCCCTTGTGGTCAGCCCTCATGAAGACATGAGAACCTGGCTCAAGTATGC

AAGCCTGTGCGGCAAGAGTGGCAGGCTGGCTCTTGCTCATAAAACTTTAGTGTTGCTCCTGGGAGTTGAT

CCGTCTCGGCAACTTGACCATCCTCTGCCAACAGTTCACCCTCAGGTGACCTATGCCTACATGAAAAACA

TGTGGAAGAGTGCCCGCAAGATCGATGCCTTCCAGCACATGCAGCATTTTGTCCAGACCATGCAGCAACA

GGCCCAGCATGCCATCGCTACTGAGGACCAGCAGCATAAGCAGGAACTGCACAAGCTCATGGCCCGATGC

TTCCTGAAACTTGGAGAGTGGCAGCTGAATCTACAGGGCATCAATGAGAGCACAATCCCCAAAGTGCTGC

AGTACTACAGCGCCGCCACAGAGCACGACCGCAGCTGGTACAAGGCCTGGCATGCGTGGGCAGTGATGAA

CTTCGAAGCTGTGCTACACTACAAACATCAGAACCAAGCCCGCGATGAGAAGAAGAAACTGCGTCATGCC

AGCGGGGCCAACATCACCAACGCCACCACTGCCGCCACCACGGCCGCCACTGCCACCACCACTGCCAGCA

CCGAGGGCAGCAACAGTGAGAGCGAGGCCGAGAGCACCGAGAACAGCCCCACCCCATCGCCGCTGCAGAA

GAAGGTCACTGAGGATCTGTCCAAAACCCTCCTGATGTACACGGTGCCTGCCGTCCAGGGCTTCTTCCGT

TCCATCTCCTTGTCACGAGGCAACAACCTCCAGGATACACTCAGAGTTCTCACCTTATGGTTTGATTATG

GTCACTGGCCAGATGTCAATGAGGCCTTAGTGGAGGGGGTGAAAGCCATCCAGATTGATACCTGGCTACA

GGTTATACCTCAGCTCATTGCAAGAATTGATACGCCCAGACCCTTGGTGGGACGTCTCATTCACCAGCTT

CTCACAGACATTGGTCGGTACCACCCCCAGGCCCTCATCTACCCACTGACAGTGGCTTCTAAGTCTACCA

CGACAGCCCGGCACAATGCAGCCAACAAGATTCTGAAGAACATGTGTGAGCACAGCAACACCCTGGTCCA

GCAGGCCATGATGGTGAGCGAGGAGCTGATCCGAGTGGCCATCCTCTGGCATGAGATGTGGCATGAAGGC

CTGGAAGAGGCATCTCGTTTGTACTTTGGGGAAAGGAACGTGAAAGGCATGTTTGAGGTGCTGGAGCCCT

TGCATGCTATGATGGAACGGGGCCCCCAGACTCTGAAGGAAACATCCTTTAATCAGGCCTATGGTCGAGA

TTTAATGGAGGCCCAAGAGTGGTGCAGGAAGTACATGAAATCAGGGAATGTCAAGGACCTCACCCAAGCC

TGGGACCTCTATTATCATGTGTTCCGACGAATCTCAAAGCAGCTGCCTCAGCTCACATCCTTAGAGCTGC

AATATGTTTCCCCAAAACTTCTGATGTGCCGGGACCTTGAATTGGCTGTGCCAGGAACATATGACCCCAA

CCAGCCAATCATTCGCATTCAGTCCATAGCACCGTCTTTGCAAGTCATCACATCCAAGCAGAGGCCCCGG

AAATTGACACTTATGGGCAGCAACGGACATGAGTTTGTTTTCCTTCTAAAAGGCCATGAAGATCTGCGCC

AGGATGAGCGTGTGATGCAGCTCTTCGGCCTGGTTAACACCCTTCTGGCCAATGACCCAACATCTCTTCG

GAAAAACCTCAGCATCCAGAGATACGCTGTCATCCCTTTATCGACCAACTCGGGCCTCATTGGCTGGGTT

CCCCACTGTGACACACTGCACGCCCTCATCCGGGACTACAGGGAGAAGAAGATCCTTCTCAACATCG

AGCATCGCATCATGTTGCGGATGGCTCCGGACTATGACCACTTGACTCTGATGCAGAAGGTGGAGGTGTT

TGAGCATGCCGTCAATAATACAGCTGGGGACGACCTGGCCAAGCTGCTGTGGCTGAAAAGCCCCAGCTCC

GAGGTGTGGTTTGACCGAAGAACCAATTATACCCGTTCTTTAGCGGTCATGTCAATGGTTGGGTATATTT

TAGGCCTGGGAGATAGACACCCATCCAACCTGATGCTGGACCGTCTGAGTGGGAAGATCCTGCACATTGA

CTTTGGGGACTGCTTTGAGGTTGCTATGACCCGAGAGAAGTTTCCAGAGAAGATTCCATTTAGACTAACA

AGAATGTTGACCAATGCTATGGAGGTTACAGGCCTGGATGGCAACTACAGAATCACATGCCACACAGTGA

TGGAGGTGCTGCGAGAGCACAAGGACAGTGTCATGGCCGTGCTGGAAGCCTTTGTCTATGACCCCTTGCT

GAACTGGAGGCTGATGGACACAAATACCAAAGGCAACAAGCGATCCCGAACGAGGACGGATTCCTACTCT

GCTGGCCAGTCAGTCGAAATTTTGGACGGTGTGGAACTTGGAGAGCCAGCCCATAAGAAAACGGGGACCA

CAGTGCCAGAATCTATTCATTCTTTCATTGGAGACGGTTTGGTGAAACCAGAGGCCCTAAATAAGAAAGC

TATCCAGATTATTAACAGGGTTCGAGATAAGCTCACTGGTCGGGACTTCTCTCATGATGACACTTTGGAT

GTTCCAACGCAAGTTGAGCTGCTCATCAAACAAGCGACATCCCATGAAAACCTCTGCCAGTGCTATATTG

GCTGGTGCCCTTTCTGGTAA

>lcl|NM_001354870.1_cds_NP_001341799.1_1 [gene=MYC] [db_xref=CCDS:CCDS87627.1] [protein=myc proto-oncogene protein isoform 2] [protein_id=NP_001341799.1] [location=1161..2522] [gbkey=CDS]

CTGGATTTTTTTCGGGTAGTGGAAAACCAGCCTCCCGCGACGATGCCCCTCAACGTTAGCTTCACCAACA

GGAACTATGACCTCGACTACGACTCGGTGCAGCCGTATTTCTACTGCGACGAGGAGGAGAACTTCTACCA

GCAGCAGCAGCAGAGCGAGCTGCAGCCCCCGGCGCCCAGCGAGGATATCTGGAAGAAATTCGAGCTGCTG

CCCACCCCGCCCCTGTCCCCTAGCCGCCGCTCCGGGCTCTGCTCGCCCTCCTACGTTGCGGTCACACCCT

TCTCCCTTCGGGGAGACAACGACGGCGGTGGCGGGAGCTTCTCCACGGCCGACCAGCTGGAGATGGTGAC

CGAGCTGCTGGGAGGAGACATGGTGAACCAGAGTTTCATCTGCGACCCGGACGACGAGACCTTCATCAAA

AACATCATCATCCAGGACTGTATGTGGAGCGGCTTCTCGGCCGCCGCCAAGCTCGTCTCAGAGAAGCTGG

CCTCCTACCAGGCTGCGCGCAAAGACAGCGGCAGCCCGAACCCCGCCCGCGGCCACAGCGTCTGCTCCAC

CTCCAGCTTGTACCTGCAGGATCTGAGCGCCGCCGCCTCAGAGTGCATCGACCCCTCGGTGGTCTTCCCC

TACCCTCTCAACGACAGCAGCTCGCCCAAGTCCTGCGCCTCGCAAGACTCCAGCGCCTTCTCTCCGTCCT

CGGATTCTCTGCTCTCCTCGACGGAGTCCTCCCCGCAGGGCAGCCCCGAGCCCCTGGTGCTCCATGAGGA

GACACCGCCCACCACCAGCAGCGACTCTGAGGAGGAACAAGAAGATGAGGAAGAAATCGATGTTGTTTCT

GTGGAAAAGAGGCAGGCTCCTGGCAAAAGGTCAGAGTCTGGATCACCTTCTGCTGGAGGCCACAGCAAAC

CTCCTCACAGCCCACTGGTCCTCAAGAGGTGCCACGTCTCCACACATCAGCACAACTACGCAGCGCCTCC

CTCCACTCGGAAGGACTATCCTGCTGCCAAGAGGGTCAAGTTGGACAGTGTCAGAGTCCTGAGACAGATC

AGCAACAACCGAAAATGCACCAGCCCCAGGTCCTCGGACACCGAGGAGAATGTCAAGAGGCGAACACACA

ACGTCTTGGAGCGCCAGAGGAGGAACGAGCTAAAACGGAGCTTTTTGCCCTGCGTGACCAGATCCCGGA

GTTGGAAAACAATGAAAAGGCCCCCAAGGTAGTTATCCTTAAAAAAGCCACAGCATACATCCTGTCCGTC

CAAGCAGAGGAGCAAAAGCTCATTTCTGAAGAGGACTTGTTGCGGAAACGACGAGAACAGTTGAAACACA

AACTTGAACAGCTACGGAACTCTTGTGCGTAA

>lcl|NM_138386.3_cds_NP_612395.2_1 [gene=NAF1] [db_xref=CCDS:CCDS3803.1] [protein=H/ACA ribonucleoprotein complex non-core subunit NAF1 isoform a] [protein_id=NP_612395.2] [location=164..1648] [gbkey=CDS]

ATGGAGGTAGTGGAGGCCGCCGCCGCTCAGCTGGAAACTCTGAAATTCAATGGCACCGACTTTGGAGTTG

GGGAAGGTCCGGCGGCTCCGTCTCCGGGCTCTGCCCCTGTGCCAGGGACACAGCCGCCGCTACAGTCGTT

TGAGGGGTCCCCGGACGCTGGGCAGACCGTGGAGGTTAAGCCTGCCGGGGAGCAGCCTCTGCAGCCCGTT

CTGAACGCCGTCGCGGCCGGGACCCCGGCGCCGCAGCCACAGCCACCGGCTGAATCGCCGGCCTGCGGAG

ACTGCGTCACCTCCCCAGGAGCCGCAGAGCCTGCGCGGGCGCCGGACTCCTTGGAGACCTCGGACTCGGA

TTCGGACTCGGACAGTGAAACAGATTCAGATAGTTCAAGTTCATCGTCTTCCTCTTCATCTTCCTCATCG

TCGTCTTCTTCCTCTTGTATATCACTTCCTCCAGTGCTGTCAGATGGAGATGATGATTTACAAATTGAGA

AGGAAAATAAGAATTTTCCTCTTAAAACAAAAGATGAATTACTTCTTAATGAACTGCCTTCTGTTGAAGA

ACTCACTATTATTCTGCCTGAAGATATTGAGTTAAAGCCTCTTGGGATGGTTTCAAGTATTATTGAACAA

CTAGTAATAATTGAATCTATGACTAACCTACCTCCAGTTAATGAGGAGACTGTAATTTTTAAAAGTGATC

GACAAGCAGCAGGAAAGATATTCGAGATATTTGGACCTGTTGCACATCCATTTTATGTGTTACGGTTTAA

TTCTTCAGATCACATTGAGAGTAAAGGTATTAAAATAAAGGAGACTATGTATTTTGCTCCATCAATGAAA

GATTTCACTCAATATATATTCACAGAAAAACTAAAACAGGATAAGGGATCAGATGCATCATGGAAGAATG

ATCAGGAACCACCACCAGAGGCCTTAGATTTTAGTGATGATGAAAAAGAGAAGGAAGCCAAACAGAGGAA

AAAATCTCAGATTCAAGGCCGGAAAAAACTCAAATCTGAATTTAATGAGCCTGGTGAAGATTTTACTGAA

GTACATCAGAATTGGAATGCTCATAGCTCTGCTTCAGAGCATGCAAAAGGATATCGTAACAGAGAATTCA

CACGAGGATTTTCCAGGGCCAGATACCCTCGATCTTGCCATGGCAGGCCTCCACCTCAGCATTTCTATAA

CTCAGAACATATGGTATCTCAGGAGACTTCAGGATTTCCTTCTCAGAGACAAAATAATCCCATTATGCCA

CAATACCCCTTTCCTCTTCCAGTGTTTGACATGCATAATTTTCCCCTCCGCCCTCCACCCCCACCACCAC

CCCCACCTGTAAACATGGGTTGGGCTACACCAAACATGGCTGCTCATCCATTACTTAACTTACCATACTC

CCTACCCCCACCCCCTCCCCCTCCACCACTGCCTCCTCCACCCTCTTCTGGAGATAGTAATTCTCATTTT

GGACCTTACTATTAG

>lcl|NM_005746.3_cds_NP_005737.1_1 [gene=NAMPT] [db_xref=CCDS:CCDS5737.1] [protein=nicotinamide phosphoribosyltransferase precursor] [protein_id=NP_005737.1] [location=100..1575] [gbkey=CDS]

ATGAATCCTGCGGCAGAAGCCGAGTTCAACATCCTCCTGGCCACCGACTCCTACAAGGTTACTCACTATA

AACAATATCCACCCAACACAAGCAAAGTTTATTCCTACTTTGAATGCCGTGAAAAGAAGACAGAAAACTC

CAAATTAAGGAAGGTGAAATATGAGGAAACAGTATTTTATGGGTTGCAGTACATTCTTAATAAGTACTTA

AAAGGTAAAGTAGTAACCAAAGAGAAAATCCAGGAAGCCAAAGATGTCTACAAAGAACATTTCCAAGATG

ATGTCTTTAATGAAAAGGGATGGAACTACATTCTTGAGAAGTATGATGGGCATCTTCCAATAGAAATAAA

AGCTGTTCCTGAGGGCTTTGTCATTCCCAGAGGAAATGTTCTCTTCACGGTGGAAAACACAGATCCAGAG

TGTTACTGGCTTACAAATTGGATTGAGACTATTCTTGTTCAGTCCTGGTATCCAATCACAGTGGCCACAA

ATTCTAGAGAGCAGAAGAAAATATTGGCCAAATATTTGTTAGAAACTTCTGGTAACTTAGATGGTCTGGA

ATACAAGTTACATGATTTTGGCTACAGAGGAGTCTCTTCCCAAGAGACTGCTGGCATAGGAGCATCTGCT

CACTTGGTTAACTTCAAAGGAACAGATACAGTAGCAGGACTTGCTCTAATTAAAAAATATTATGGAACGA

AAGATCCTGTTCCAGGCTATTCTGTTCCAGCAGCAGAACACAGTACCATAACAGCTTGGGGGAAAGACCA

TGAAAAAGATGCTTTTGAACATATTGTAACACAGTTTTCATCAGTGCCTGTATCTGTGGTCAGCGATAGC

TATGACATTTATAATGCGTGTGAGAAAATATGGGGTGAAGATCTAAGACATTTAATAGTATCAAGAAGTA

CACAGGCACCACTAATAATCAGACCTGATTCTGGAAACCCTCTTGACACTGTGTTAAAGGTTTTGGAGAT

TTTAGGTAAGAAGTTTCCTGTTACTGAGAACTCAAAGGGTTACAAGTTGCTGCCACCTTATCTTAGAGTT

ATTCAAGGGGATGGAGTAGATATTAATACCTTACAAGAGATTGTAGAAGGCATGAAACAAAAAATGTGGA

GTATTGAAAATATTGCCTTCGGTTCTGGTGGAGGTTTGCTACAGAAGTTGACAAGAGATCTCTTGAATTG

TTCCTTCAAGTGTAGCTATGTTGTAACTAATGGCCTTGGGATTAACGTCTTCAAGGACCCAGTTGCTGAT

CCCAACAAAAGGTCCAAAAAGGGCCGATTATCTTTACATAGGACGCCAGCAGGGAATTTTGTTACACTGG

AGGAAGGAAAAGGAGACCTTGAGGAATATGGTCAGGATCTTCTCCATACTGTCTTCAAGAATGGCAAGGT

GACAAAAAGCTATTCATTTGATGAAATAAGAAAAAATGCACAGCTGAATATTGAACTGGAAGCAGCACAT

CATTAG

>lcl|NM_005899.5_cds_NP_005890.2_1 [gene=NBR1] [db_xref=CCDS:CCDS45694.1] [protein=next to BRCA1 gene 1 protein isoform a] [protein_id=NP_005890.2] [location=99..2999] [gbkey=CDS]

ATGGAACCACAGGTTACTCTAAATGTGACTTTTAAAAATGAAATTCAAAGCTTTCTGGTTTCTGATCCAG

AAAATACAACTTGGGCTGATATCGAAGCTATGGTAAAAGTTTCATTTGATCTGAATACTATTCAAATAAA

ATACCTGGATGAGGAAAATGAAGAGGTATCCATCAACAGTCAAGGAGAATATGAAGAAGCGCTTAAGATG

GCAGTTAAACAGGGAAACCAACTGCAGATGCAAGTCCACGAAGGGCACCATGTCGTTGATGAAGCCCCAC

CCCCAGTTGTAGGAGCAAAACGACTAGCTGCCAGGGCAGGGAAGAAGCCACTTGCACATTACTCTTCACT

GGTGAGAGTCTTGGGATCAGACATGAAGACCCCAGAGGATCCTGCAGTGCAGTCGTTTCCACTTGTTCCA

TGTGACACAGACCAGCCTCAAGACAAGCCCCCAGACTGGTTCACAAGCTACCTGGAGACGTTCAGAGAAC

AAGTGGTTAACGAAACGGTTGAGAAGCTTGAACAGAAATTACATGAAAAGCTTGTCCTCCAGAACCCATC

CTTGGGTTCTTGTCCCTCAGAAGTCTCAATGCCTACTTCAGAAGAAACATTGTTTTTGCCAGAAAACCAG

TTCAGCTGGCATATTGCTTGCAACAACTGCCAAAGAAGGATTGTTGGTGTCCGCTACCAGTGTAGCCTAT

GCCCATCCTACAATATCTGTGAAGATTGTGAAGCAGGGCCATATGGCCATGACACTAACCACGTCCTGCT

GAAGTTGCGGAGACCTGTTGTGGGCTCCTCTGAACCGTTCTGTCACTCAAAGTACTCTACTCCTCGTCTT

CCTGCTGCTCTGGAACAAGTCAGGCTCCAGAAACAGGTTGATAAGAACTTTCTTAAAGCAGAAAAGCAAA

GGTTGCGAGCTGAGAAGAAACAACGTAAAGCAGAGGTCAAGGAACTTAAAAAGCAGCTTAAACTCCATAG

GAAAATTCACCTGTGGAATTCAATCCATGGACTCCAGAGCCCCAAGTCTCCTTTAGGCCGACCTGAGAGC

TTGCTCCAGTCTAATACCCTGATGCTCCCTTTGCAGCCCTGTACCTCCGTTATGCCAATGCTCAGTGCAG

CATTTGTGGATGAGAATTTGCCTGATGGGACTCACCTTCAGCCAGGAACCAAGTTTATCAAACACTGGAG

GATGAAAAATACAGGAAATGTAAAGTGGAGTGCAGACACAAAGCTCAAGTTCATGTGGGGAAACCTGACT

TTGGCTTCCACAGAAAAGAAGGATGTTTTGGTTCCCTGCCTCAAGGCCGGCCATGTGGGAGTTGTATCTG

TGGAGTTCATTGCCCCAGCCTTGGAGGGAACGTATACTTCCCATTGGCGTCTTTCTCACAAAGGCCAGCA

ATTTGGGCCTCGGGTCTGGTGCAGTATCATAGTAGATCCTTTCCCCTCCGAAGAGAGCCCTGATAACATT

GAAAAGGGCATGATCAGCTCAAGCAAAACTGATGATCTCACCTGCCAGCAAGAGGAAACTTTTCTTCTGG

CTAAAGAAGAAAGACAGCTTGGTGAAGTGACTGAGCAGACAGAAGGGACAGCAGCCTGCATCCCACAGAA

GGCAAAAAATGTTGCCAGTGAGAGGGAGCTCTACATCCCATCTGTGGATCTTCTGACTGCCCAGGACCTG

CTGTCCTTTGAGCTGTTGGATATAAACATTGTTCAAGAGTTGGAGAGAGTGCCCCACAACACCCCTGTGG

ATGTGACTCCCTGCATGTCTCCTCTGCCACATGACAGTCCTTTAATAGAGAAGCCAGGCTTGGGGCAGAT

AGAGGAAGAGAATGAAGGGGCAGGATTTAAAGCACTTCCTGATTCTATGGTGTCAGTAAAGAGGAAGGCT

GAGAACATTGCTTCTGTGGAGGAAGCAGAAGAAGACCTGAGTGGGACCCAGTTTGTGTGTGAGACAGTAA

TCCGATCCCTTACCTTGGATGCTGCCCCAGACCACAACCCTCCTTGCAGACAGAAGTCCTTGCAGATGAC

ATTTGCCTTGCCTGAAGGACCACTTGGAAATGAGAAGGAGGAGATTATCCATATCGCTGAGGAAGAAGCT

GTCATGGAGGAGGAGGAGGATGAGGAGGATGAGGAGGAGGAGGATGAGCTCAAAGATGAAGTTCAAAGTC

AGTCCTCTGCTTCCTCAGAGGATTACATCATCATCCTGCCTGAGTGCTTTGATACCAGCCGCCCCCTGGG

GGATTCTATGTACAGCTCTGCGCTCTCACAGCCAGGCCTGGAGCGAGGTGCTGAAGGCAAGCCTGGGGTT

GAGGCTGGGCAGGAACCAGCTGAGGCTGGGGAAAGACTCCCTGGAGGGGAGAACCAGCCACAGGAGCACA

GCATAAGTGACATCCTCACGACCTCACAGACTCTGGAAACAGTGCCCCTAATCCCAGAGGTAGTGGAGCT

TCCACCGTCACTGCCCAGGAGCTCTCCTTGTGTACATCATCATGGTTCCCCAGGAGTGGATTTACCAGTT

ACCATACCAGAAGTTTCTTCAGTCCCTGATCAGATCAGAGGAGAGCCCAGAGGCTCATCAGGACTTGTAA

ACAGCAGACAGAAGAGCTATGACCACTCAAGGCACCATCATGGGAGCAGCATTGCTGGAGGACTGGTGAA

GGGGGCTTTGTCTGTTGCTGCCTCTGCATACAAGGCCCTGTTTGCTGGGCCACCAGTCACTGCACAGCCA

ATAATTTCTGAAGATCAGACAGCAGCCCTGATGGCCCATCTCTTTGAAATGGGATTCTGTGACAGGCAGC

TGAACCTACGGCTGCTGAAGAAACACAATTACAATATCCTGCAGGTTGTGACAGAACTTCTTCAGTTAAA

CAACAACGACTGGTACAGCCAACGCTATTGA

>lcl|NM_205842.3_cds_NP_995314.1_1 [gene=NCKAP1] [db_xref=CCDS:CCDS2288.1] [protein=nck-associated protein 1 isoform 2] [protein_id=NP_995314.1] [location=359..3763] [gbkey=CDS]

ATGTCGCGCTCAGTGCTGCAGCCCAGTCAGCAGAAGCTGGCGGAGAAGCTCACCATCCTCAACGACCGGG

GCGTCGGCATGCTCACCCGCCTCTACAACATCAAGAAGCAAGGACAAGTTTGGAAGGCATGTGGAGACCC

CAAGGCAAAACCATCCTATCTTATCGACAAAAACCTGGAATCTGCTGTGAAATTCATAGTCAGAAAATTC

CCTGCTGTAGAAACCCGCAACAACAATCAACAGCTTGCACAACTACAGAAAGAAAAATCAGAGATTCTGA

AAAATCTGGCATTATATTACTTCACATTTGTAGATGTTATGGAATTTAAGGACCATGTTTGTGAATTGCT

GAATACTATTGACGTTTGCCAAGTCTTCTTTGATATTACTGTAAACTTTGATTTAACAAAGAACTACTTA

GATTTAATTATAACCTATACAACACTAATGATACTGCTGTCTCGAATTGAAGAAAGGAAGGCAATCATTG

GATTATACAACTATGCCCATGAAATGACTCATGGAGCAAGTGACAGAGAATACCCACGCCTTGGCCAGAT

GATTGTGGATTATGAAAACCCCTTTAAAGAAGATGATGGAAGAATTTGTACCCCATAGCAAGTCTCTTTCA

GATGCACTAATTTCTCTTCAAATGGTATATCCTCGAAGGAATCTTTCAGCTGACCAGTGGAGAAATGCCC

AGTTATTGAGCCTCATCAGTGCACCTAGTACAATGCTTAATCCAGCACAGTCCGACACTATGCCTTGTGA

ATACCTCTCTTTGGATGCAATGGAAAAGTGGATTATCTTTGGCTTTATTTTGTGCCATGGGATCCTAAAT

ACTGACGCTACAGCACTGAACCTTTGGAAACTAGCTCTTCAAAGTAGCTCTTGCCTCTCTCTCTTTCGGG

ATGAAGTTTTCCACATTCACAAAGCTGCAGAAGACTTATTTGTAAACATACGAGGCTATAATAAACGTAT

TAATGACATAAGAGAATGCAAGGAGGCAGCCGTGTCACATGCTGGTTCAATGCACAGAGAAAGACGCAAG

TTTTTAAGATCTGCACTGAAGGAATTGGCTACTGTCCTCTCTGATCAACCTGGATTGCTAGGTCCCAAGG

CACTTTTTGTTTTTATGGCATTATCCTTTGCCCGTGATGAAATCATCTGGCTACTTCGTCATGCAGATAA

CATGCCAAAGAAGAGTGCAGACGACTTTATAGATAAGCACATTGCTGAATTAATATTTTACATGGAAGAA

CTTAGAGCACATGTGAGGAAATACGGACCTGTAATGCAGAGGTATTACGTGCAGTACCTTTCTGGCTTTG

ATGCTGTTGTCCTCAATGAACTCGTGCAGAATCTTTCTGTTTGCCCTGAAGATGAATCAATCATCATGTC

CTCTTTTGTTAACACTATGACTTCCCTAAGTGTAAAACAAGTTGAAGATGGGGAAGTATTTGATTTCAGA

GGAATGAGATTAGATTGGTTTAGGTTACAGGCATATACTAGTGTCTCAAAGGCTTCACTTGGCCTTGCAG

ATCACAGAGAACTTGGAAAGATGATGAATACAATAATTTTTCATACAAAAATGGTAGATTCCTTGGTGGA

AATGTTGGTGGAAACATCAGATCTCTCCATATTTTGTTTTTATAGTCGTGCTTTTGAGAAGATGTTTCAA

CAGTGTTTGGAGTTACCCTCTCAATCAAGATACTCAATTGCATTTCCACTACTTTGCACTCATTTTATGA

GTTGCACGCATGAACTATGTCCAGAAGAGCGACATCATATTGGAGATCGCAGTCTTTCCTTATGTAATAT

GTTCCTAGATGAAATGGCCAAACAAGCTCGAAATCTCATCACTGATATTTGCACAGAACAGTGTACCCTT

AGTGACCAGTTGCTACCCAAGCATTGTGCCAAAACTATCAGTCAAGCAGTGAATAAGAAATCAAAAAAGC

AGACTGGTAAGAAAGGGGAACCTGAAAGGGAGAAACCAGGTGTTGAGAGCATGAGGAAAAACAGGCTGGT

TGTGACCAACCTTGATAAATTGCACACTGCACTTTCTGAGTTATGCTTCTCTATAAATTATGTACCAAAC

ATGGTGGTATGGGAACATACCTTTACCCCACGAGAATATTTGACTTCTCATCTGGAAATACGCTTTACCA

AGTCAATTGTTGGGATGACTATGTATAATCAAGCCACACAGGAAATTGCAAAACCTTCAGAACTTCTAAC

AAGTGTAAGAGCATACATGACCGTACTCCAGTCAATAGAAAACTATGTGCAGATTGATATTACAAGAGTA

TTTAATAATGTGCTTCTTCAACAAACACAACATTTAGACAGTCATGGAGAGCCAACCATTACAAGTCTAT

ACACAAATTGGTATTTGGAAACTTTGTTACGACAAGTCAGCAATGGCCATATAGCATATTTTCCTGCAAT

GAAAGCGTTTGTGAACTTACCTACAGAAAATGAATTAACATTCAATGCAGAGGAATATTCTGACATATCA

GAAATGAGGTCATTATCAGAACTACTAGGCCCATATGGTATGAAGTTTCTAAGTGAAAGCCTTATGTGGC

ATATTTCATCACAAGTTGCTGAACTTAAGAAACTTGTGGTGGAGAATGTTGATGTGTTAACACAAATGAG

GACCAGCTTTGACAAACCAGACCAGATGGCTGCACTGTTTAAAAGATTATCATCTGTTGACAGTGTCTTG

AAGAGGATGACAATAATTGGTGTAATTTTATCCTTCCGATCATTGGCACAAGAAGCACTTAGAGATGTCT

TATCCTACCACATTCCTTTTCTTGTAAGTTCAATTGAAGATTTTAAGGATCACATTCCAAGGGAAACTGA

TATGAAGGTTGCAATGAATGTGTATGAGTTATCATCAGCTGCCGGATTACCTTGTGAGATTGATCCTGCA

TTGGTCGTAGCTCTTTCTTCACAAAAATCGGAAAACATTAGTCCAGAAGAAGAGTATAAAATTGCCTGCC

TTCTCATGGTGTTTGTGGCAGTTTCTTTGCCAACACTGGCCAGTAATGTGATGTCTCAGTACAGCCCTGC

TATAGAAGGGCATTGCAACAACATACATTGCTTGGCCAAAGCCATCAACCAGATTGCTGCAGCTTTGTTT

ACAATTCACAAAGGAAGCATTGAAGACCGTCTTAAAGAATTTCTGGCGCTTGCATCCTCCAGTCTACTGA

AAATTGGCCAGGAGACAGATAAAACTACAACAAGAAATAGAGAATCTGTTTATTTACTGCTAGATATGAT

TGTACAAGAATCTCCATTCCTTACAATGGATCTTTTGGAATCTTGTTTTCCTTATGTCTTGCTGAGAAAT

GCATACCATGCTGTCTACAAACAAAGTGTTACATCTTCTGCATAA

>lcl|NM_006164.5_cds_NP_006155.2_1 [gene=NFE2L2] [db_xref=CCDS:CCDS42782.1] [protein=nuclear factor erythroid 2-related factor 2 isoform 1] [protein_id=NP_006155.2] [location=152..1969] [gbkey=CDS]

ATGATGGACTTGGAGCTGCCGCCGCCGGGACTCCCGTCCCAGCAGGACATGGATTTGATTGACATACTTT

GGAGGCAAGATATAGATCTTGGAGTAAGTCGAGAAGTATTTGACTTCAGTCAGCGACGGAAAGAGTATGA

GCTGGAAAAACAGAAAAAACTTGAAAAGGAAAGACAAGAACAACTCCAAAAGGAGCAAGAGAAAGCCTTT

TTCGCTCAGTTACAACTAGATGAAGAGACAGGTGAATTTCTCCCAATTCAGCCAGCCCAGCACATCCAGT

CAGAAACCAGTGGATCTGCCAACTACTCCCAGGTTGCCCACATTCCCAAATCAGATGCTTTGTACTTTGA

TGACTGCATGCAGCTTTTGGCGCAGACATTCCCGTTTGTAGATGACAATGAGGTTTCTTCGGCTACGTTT

CAGTCACTTGTTCCTGATATTCCCGGTCACATCGAGAGCCCAGTCTTCATTGCTACTAATCAGGCTCAGT

CACCTGAAACTTCTGTTGCTCAGGTAGCCCCTGTTGATTTAGACGGTATGCAACAGGACATTGAGCAAGT

TTGGGAGGAGCTATTATCCATTCCTGAGTTACAGTGTCTTAATATTGAAAATGACAAGCTGGTTGAGACT

ACCATGGTTCCAAGTCCAGAAGCCAAACTGACAGAAGTTGACAATTATCATTTTTACTCATCTATACCCT

CAATGGAAAAAGAAGTAGGTAACTGTAGTCCACATTTTCTTAATGCTTTTGAGGATTCCTTCAGCAGCAT

CCTCTCCACAGAAGACCCCAACCAGTTGACAGTGAACTCATTAAATTCAGATGCCACAGTCAACACAGAT

TTTGGTGATGAATTTTATTCTGCTTTCATAGCTGAGCCCAGTATCAGCAACAGCATGCCCTCACCTGCTA

CTTTAAGCCATTCACTCTCTGAACTTCTAAATGGGCCCATTGATGTTTCTGATCTATCACTTTGCAAAGC

TTTCAACCAAAACCACCCTGAAAGCACAGCAGAATTCAATGATTCTGACTCCGGCATTTCACTAAACACA

AGTCCCAGTGTGGCATCACCAGAACACTCAGTGGAATCTTCCAGCTATGGAGACACACTACTTGGCCTCA

GTGATTCTGAAGTGGAAGAGCTAGATAGTGCCCCTGGAAGTGTCAAACAGAATGGTCCTAAAACACCAGT

ACATTCTTCTGGGGATATGGTACAACCCTTGTCACCATCTCAGGGGCAGAGCACTCACGTGCATGATGCC

CAATGTGAGAACACACCAGAGAAAGAATTGCCTGTAAGTCCTGGTCATCGGAAAACCCCATTCACAAAAG

ACAAACATTCAAGCCGCTTGGAGGCTCATCTCACAAGAGATGAACTTAGGGCAAAAGCTCTCCATATCCC

ATTCCCTGTAGAAAAAATCATTAACCTCCCTGTTGTTGACTTCAACGAAATGATGTCCAAAGAGCAGTTC

AATGAAGCTCAACTTGCATTAATTCGGGATATACGTAGGAGGGGTAAGAATAAAGTGGCTGCTCAGAATT

GCAGAAAAAGAAAACTGGAAAATATAGTAGAACTAGAGCAAGATTTAGATCATTTGAAAGATGAAAAAGA

AAAATTGCTCAAAGAAAAAGGAGAAAATGACAAAAGCCTTCACCTACTGAAAAAACAACTCAGCACCTTA

TATCTCGAAGTTTTCAGCATGCTACGTGATGAAGATGGAAAACCTTATTCTCCTAGTGAATACTCCCTGC

AGCAAACAAGAGATGGCAATGTTTTCCTTGTTCCCAAAAGTAAGAAGCCAGATGTTAAGAAAAACTAG

>lcl|NM_003998.4_cds_NP_003989.2_1 [gene=NFKB1] [db_xref=CCDS:CCDS3657.1] [protein=nuclear factor NF-kappa-B p105 subunit isoform 1] [protein_id=NP_003989.2] [location=438..3347] [gbkey=CDS]

ATGGCAGAAGATGATCCATATTTGGGAAGGCCTGAACAAATGTTTCATTTGGATCCTTCTTTGACTCATA

CAATATTTAATCCAGAAGTATTTCAACCACAGATGGCACTGCCAACAGCAGATGGCCCATACCTTCAAAT

ATTAGAGCAACCTAAACAGAGAGGATTTCGTTTCCGTTATGTATGTGAAGGCCCATCCCATGGTGGACTA

CCTGGTGCCTCTAGTGAAAAGAACAAGAAGTCTTACCCTCAGGTCAAAATCTGCAACTATGTGGGACCAG

CAAAGGTTATTGTTCAGTTGGTCACAAATGGAAAAAATATCCACCTGCATGCCCACAGCCTGGTGGGAAA

ACACTGTGAGGATGGGATCTGCACTGTAACTGCTGGACCCAAGGACATGGTGGTCGGCTTCGCAAACCTG

GGTATACTTCATGTGACAAAGAAAAAAGTATTTGAAACACTGGAAGCACGAATGACAGAGGCGTGTATAA

GGGGCTATAATCCTGGACTCTTGGTGCACCCTGACCTTGCCTATTTGCAAGCAGAAGGTGGAGGGGACCG

GCAGCTGGGAGATCGGGAAAAAGAGCTAATCCGCCAAGCAGCTCTGCAGCAGACCAAGGAGATGGACCTC

AGCGTGGTGCGGCTCATGTTTACAGCTTTTCTTCCGGATAGCACTGGCAGCTTCACAAGGCGCCTGGAAC

CCGTGGTATCAGACGCCATCTATGACAGTAAAGCCCCCAATGCATCCAACTTGAAAATTGTAAGAATGGA

CAGGACAGCTGGATGTGTGACTGGAGGGGAGGAAATTTATCTTCTTTGTGACAAAGTTCAGAAAGATGAC

ATCCAGATTCGATTTTATGAAGAGGAAGAAAATGGTGGAGTCTGGGAAGGATTTGGAGATTTTTCCCCCA

CAGATGTTCATAGACAATTTGCCATTGTCTTCAAAACTCCAAAGTATAAAGATATTAATATTACAAAACC

AGCCTCTGTGTTTGTCCAGCTTCGGAGGAAATCTGACTTGGAAACTAGTGAACCAAAACCTTTCCTCTAC

TATCCTGAAATCAAAGATAAAGAAGAAGTGCAGAGGAAACGTCAGAAGCTCATGCCCAATTTTTCGGATA

GTTTCGGCGGTGGTAGTGGTGCTGGAGCTGGAGGCGGAGGCATGTTTGGTAGTGGCGGTGGAGGAGGGGG

CACTGGAAGTACAGGTCCAGGGTATAGCTTCCCACACTATGGATTTCCTACTTATGGTGGGATTACTTTC

CATCCTGGAACTACTAAATCTAATGCTGGGATGAAGCATGGAACCATGGACACTGAATCTAAAAAGGACC

CTGAAGGTTGTGACAAAAGTGATGACAAAAACACTGTAAACCTCTTTGGGAAAGTTATTGAAACCACAGA

GCAAGATCAGGAGCCCAGCGAGGCCACCGTTGGGAATGGTGAGGTCACTCTAACGTATGCAACAGGAACA

AAAGAAGAGAGTGCTGGAGTTCAGGATAACCTCTTTCTAGAGAAGGCTATGCAGCTTGCAAAGAGGCATG

CCAATGCCCTTTTCGACTACGCGGTGACAGGAGACGTGAAGATGCTGCTGGCCGTCCAGCGCCATCTCAC

TGCTGTGCAGGATGAGAATGGGGACAGTGTCTTACACTTAGCAATCATCCACCTTCATTCTCAACTTGTG

AGGGATCTACTAGAAGTCACATCTGGTTTGATTTCTGATGACATTATCAACATGAGAAATGATCTGTACC

AGACGCCCTTGCACTTGGCAGTGATCACTAAGCAGGAAGATGTGGTGGAGGATTTGCTGAGGGCTGGGGC

CGACCTGAGCCTTCTGGACCGCTTGGGTAACTCTGTTTTGCACCTAGCTGCCAAAGAAGGACATGATAAA

GTTCTCAGTATCTTACTCAAGCACAAAAAGGCAGCACTACTTCTTGACCACCCCAACGGGGACGGTCTGA

ATGCCATTCATCTAGCCATGATGAGCAATAGCCTGCCATGTTTGCTGCTGCTGGTGGCCGCTGGGGCTGA

CGTCAATGCTCAGGAGCAGAAGTCCGGGCGCACAGCACTGCACCTGGCTGTGGAGCACGACAACATCTCA

TTGGCAGGCTGCCTGCTCCTGGAGGGTGATGCCCATGTGGACAGTACTACCTACGATGGAACCACACCCC

TGCATATAGCAGCTGGGAGAGGGTCCACCAGGCTGGCAGCTCTTCTCAAAGCAGCAGGAGCAGATCCCCT

GGTGGAGAACTTTGAGCCTCTCTATGACCTGGATGACTCTTGGGAAAATGCAGGAGAGGATGAAGGAGTT

GTGCCTGGAACCACGCCTCTAGATATGGCCACCAGCTGGCAGGTATTTGACATATTAAATGGGAAACCAT

ATGAGCCAGAGTTTACATCTGATGATTTACTAGCACAAGGAGACATGAAACAGCTGGCTGAAGATGTGAA

GCTGCAGCTGTATAAGTTACTAGAAATTCCTGATCCAGACAAAAACTGGGCTACTCTGGCGCAGAAATTA

GGTCTGGGGATACTTAATAATGCCTTCCGGCTGAGTCCTGCTCCTTCCAAAACACTTATGGACAACTATG

AGGTCTCTGGGGGTACAGTCAGAGAGCTGGTGGAGGCCCTGAGACAAATGGGCTACACCGAAGCAATTGA

AGTGATCCAGGCAGCCTCCAGCCCAGTGAAGACCACCTCTCAGGCCCACTCGCTGCCTCTCTCGCCTGCC

TCCACAAGGCAGCAAATAGACGAGCTCCGAGACAGTGACAGTGTCTGCGACAGCGGCGTGGAGACATCCT

TCCGCAAACTCAGCTTTACCGAGTCTCTGACCAGTGGTGCCTCACTGCTAACTCTCAACAAAATGCCCCA

TGATTATGGGCAGGAAGGACCTCTAGAAGGCAAAATTTAG

>lcl|NM_145285.3_cds_NP_660328.2_1 [gene=NKX2-3] [db_xref=CCDS:CCDS41558.1] [protein=homeobox protein Nkx-2.3] [protein_id=NP_660328.2] [location=191..1285] [gbkey=CDS]

ATGATGTTACCAAGCCCGGTCACCTCCACCCCTTTCTCAGTCAAAGACATTTTGAATCTGGAGCAGCAGC

ACCAGCACTTCCATGGTGCGCACTTGCAGGCGGACTTGGAGCACCACTTCCACTCTGCGCCCTGCATGCT

GGCCGCCGCTGAGGGGACGCAATTTTCTGACGGAGGGGAGGAGGACGAGGAAGACGAGGGCGAGAAATTG

TCCTATTTGAACTCACTAGCCGCAGCAGACGGCCACGGGGATTCAGGGCTGTGTCCCCAGGGCTATGTCC

ACACGGTCCTGCGAGACTCGTGCAGCGAGCCCAAGGAACATGAAGAGGAGCCCGAGGTCGTGAGGGACCG

GAGCCAAAAAAGCTGCCAGCTGAAGAAGTCTCTAGAGACGGCCGGAGACTGCAAGGCGGCGGAGGAGAGC

GAGAGGCCGAAGCCACGCAGCCGCCGGAAGCCCCGGGTCCTCTTCTCGCAAGCCCAGGTCTTCGAGCTGG

AACGCAGGTTCAAGCAGCAGCGGTACCTGTCGGCACCCGAGCGCGAGCACCTCGCCAGCAGCCTGAAGCT

CACATCCACTCAGGTGAAAATCTGGTTCCAGAATCGCAGGTACAAGTGCAAGAGACAGCGGCAGGACAAG

TCTCTGGAGCTTGGCGCACACGCGCCCCCGCCGCCGCCGCGCCGCGTGGCTGTCCCGGTGCTGGTGCGGG

ACGGCAAGCCGTGCGTCACGCCCAGCGCGCAGGCCTACGGCGCGCCCTACAGCGTGGGCGCCAGCGCCTA

CTCCTACAACAGCTTCCCCGCCTACGGCTATGGGAACTCGGCCGCGGCCGCCGCCGCCGCCGCCGCCGCC

GCCGCAGCAGCGGCGGCCTACAGCAGCAGCTATGGCTGTGCGTACCCGGCGGGCGGCGGCGGCGGCGGCG

GCGGGACCTCCGCGGCGACCACTGCCATGCAGCCCGCCTGCAGCGCGGCCGGAGGCGGCCCCTTTGTGAA

CGTGAGCAACCTAGGAGGCTTCGGCAGCGGCGGCAGCGCACAGCCGTTGCACCAGGGTACTGCAGCCGGG

GCCGCGTGCGCTCAGGGCACCTTGCAGGGCATCCGGGCCTGGTAG

>lcl|NM_021209.4_cds_NP_067032.3_1 [gene=NLRC4] [db_xref=CCDS:CCDS33174.1] [protein=NLR family CARD domain-containing protein 4 isoform a] [protein_id=NP_067032.3] [location=276..3350] [gbkey=CDS]

ATGAATTTCATAAAGGACAATAGCCGAGCCCTTATTCAAAGAATGGGAATGACTGTTATAAAGCAAATCA

CAGATGACCTATTTGTATGGAATGTTCTGAATCGCGAAGAAGTAAACATCATTTGCTGCGAGAAGGTGGA

GCAGGATGCTGCTAGAGGGATCATTCACATGATTTTGAAAAAGGGTTCAGAGTCCTGTAACCTCTTTCTT

AAATCCCTTAAGGAGTGGAACTATCCTCTATTTCAGGACTTGAATGGACAAAGTCTTTTTCATCAGACAT

CAGAAGGAGACTTGGACGATTTGGCTCAGGATTTAAAGGACTTGTACCATACCCCATCTTTTCTGAACTT

TTATCCCCTTGGTGAAGATATTGACATTATTTTTAACTTGAAAAGCACCTTCACAGAACCTGTCCTGTGG

AGGAAGGACCAACACCATCACCGCGTGGAGCAGCTGACCCTGAATGGCCTCCTGCAGGCTCTTCAGAGCC

CCTGCATCATTGAAGGGGAATCTGGCAAAGGCAAGTCCACTCTGCTGCAGCGAATTGCCATGCTCTGGGG

CTCCGGAAAGTGCAAGGCTCTGACCAAGTTCAAATTCGTCTTCTTCCTCCGTCTCAGCAGGGCCCAGGGT

GGACTTTTTGAAACCCTCTGTGATCAACTCCTGGATATACCTGGCACAATCAGGAAGCAGACATTCATGG

CCATGCTGCTGAAGCTGCGGCAGAGGGTTCTTTTCCTTCTTGATGGCTACAATGAATTCAAGCCCCAGAA

CTGCCCAGAAATCGAAGCCCTGATAAAGGAAAACCACCGCTTCAAGAACATGGTCATCGTCACCACTACC

ACTGAGTGCCTGAGGCACATACGGCAGTTTGGTGCCCTGACTGCTGAGGTGGGGGATATGACAGAAGACA

GCGCCCAGGCTCTCATCCGAGAAGTGCTGATCAAGGAGCTTGCTGAAGGCTTGTTGCTCCAAATTCAGAA

ATCCAGGTGCTTGAGGAATCTCATGAAGACCCCTCTCTTTGTGGTCATCACTTGTGCAATCCAGATGGGT

GAAAGTGAGTTCCACTCTCACACACAAACAACGCTGTTCCATACCTTCTATGATCTGTTGATACAGAAAA

ACAAACACAAACATAAAGGTGTGGCTGCAAGTGACTTCATTCGGAGCCTGGACCACTGTGGAGACCTAGC

TCTGGAGGGTGTGTTCTCCCACAAGTTTGATTTCGAACTGCAGGATGTGTCCAGCGTGAATGAGGATGTC

CTGCTGACAACTGGGCTCCTCTGTAAATATACAGCTCAAAGGTTCAAGCCAAAGTATAAATTCTTTCACA

AGTCATTCCAGGAGTACACAGCAGGACGAAGACTCAGCAGTTTATTGACGTCTCATGAGCCAGAGGAGGT

GACCAAGGGGAATGGTTACTTGCAGAAAATGGTTTCCATTTCGGACATTACATCCACTTATAGCAGCCTG

CTCCGGTACACCTGTGGGTCATCTGTGGAAGCCACCAGGGCTGTTATGAAGCACCTCGCAGCAGTGTATC

AACACGGCTGCCTTCTCGGACTTTCCATCGCCAAGAGGCCTCTCTGGAGACAGGAATCTTTGCAAAGTGT

GAAAAACACCACTGAGCAAGAAATTCTGAAAGCCATAAACATCAATTCCTTTGTAGAGTGTGGCATCCAT

TTATATCAAGAGAGTACATCCAAATCAGCCCTGAGCCAAGAATTTGAAGCTTTCTTTCAAGGTAAAAGCT

TATATATCAACTCAGGGAACATCCCCGATTACTTATTTGACTTCTTTGAACATTTGCCCAATTGTGCAAG

TGCCCTGGACTTCATTAAACTGGACTTTTATGGGGGAGCTATGGCTTCATGGGAAAAGGCTGCAGAAGAC

ACAGGTGGAATCCACATGGAAGAGGCCCCAGAAACCTACATTCCCAGCAGGGCTGTATCTTTGTTCTTCA

ACTGGAAGCAGGAATTCAGGACTCTGGAGGTCACACTCCGGGATTTCAGCAAGTTGAATAAGCAAGATAT

CAGATATCTGGGGAAAATATTCAGCTCTGCCACAAGCCTCAGGCTGCAAATAAAGAGATGTGCTGGTGTG

GCTGGAAGCCTCAGTTTGGTCCTCAGCACCTGTAAGAACATTTATTCTCTCATGGTGGAAGCCAGTCCCC

TCACCATAGAAGATGAGAGGCACATCACATCTGTAACAAACCTGAAAACCTTGAGTATTCATGACCTACA

GAATCAACGGCTGCCGGGTGGTCTGACTGACAGCTTGGGTAACTTGAAGAACCTTACAAAGCTCATAATG

GATAACATAAAGATGAATGAAGAAGATGCTATAAAACTAGCTGAAGGCCTGAAAAACCTGAAGAAGATGT

GTTTATTTCATTTGACCCACTTGTCTGACATTGGAGAGGGAATGGATTACATAGTCAAGTCTCTGTCAAG

TGAACCCTGTGACCTTGAAGAAATTCAATTAGTCTCCTGCTGCTTGTCTGCAAATGCAGTGAAAATCCTA

GCTCAGAATCTTCACAATTTGGTCAAACTGAGCATTCTTGATTTATCAGAAAATTACCTGGAAAAAGATG

GAAATGAAGCTCTTCATGAACTGATCGACAGGATGAACGTGCTAGAACAGCTCACCGCACTGATGCTGCC

CTGGGGCTGTGACGTGCAAGGCAGCCTGAGCAGCCTGTTGAAACATTTGGAGGAGGTCCCACAACTCGTC

AAGCTTGGGTTGAAAAACTGGAGACTCACAGATACAGAGATTAGAATTTTAGGTGCATTTTTTGGAAAGA

ACCCTCTGAAAAACTTCCAGCAGTTGAATTTGGCGGGAAATCGTGTGAGCAGTGATGGATGGCTTGCCTT

CATGGGTGTATTTGAGAATCTTAAGCAATTAGTGTTTTTTGACTTTAGTACTAAAGAATTTCTACCTGAT

CCAGCATTAGTCAGAAAACTTAGCCAAGTGTTATCCAAGTTAACTTTTCTGCAAGAAGCTAGGCTTGTTG

GGTGGCAATTTGATGATGATGATCTCAGTGTTATTACAGGTGCTTTTAAACTAGTAACTGCTTAA

>lcl|NM_000271.5_cds_NP_000262.2_1 [gene=NPC1] [db_xref=CCDS:CCDS11878.1] [protein=NPC intracellular cholesterol transporter 1 precursor] [protein_id=NP_000262.2] [location=164..4000] [gbkey=CDS]

ATGACCGCTCGCGGCCTGGCCCTTGGCCTCCTCCTGCTGCTACTGTGTCCAGCGCAGGTGTTTTCACAGT

CCTGTGTTTGGTATGGAGAGTGTGGAATTGCATATGGGGACAAGAGGTACAATTGCGAATATTCTGGCCC

ACCAAAACCATTGCCAAAGGATGGATATGACTTAGTGCAGGAACTCTGTCCAGGATTCTTCTTTGGCAAT

GTCAGTCTCTGTTGTGATGTTCGGCAGCTTCAGACACTAAAAGACAACCTGCAGCTGCCTCTACAGTTTC

TGTCCAGATGTCCATCCTGTTTTTATAACCTACTGAACCTGTTTTGTGAGCTGACATGTAGCCCTCGACA

GAGTCAGTTTTTGAATGTTACAGCTACTGAAGATTATGTTGATCCTGTTACAAACCAGACGAAAACAAAT

GTGAAAGAGTTACAATACTACGTCGGACAGAGTTTTGCCAATGCAATGTACAATGCCTGCCGGGATGTGG

AGGCCCCCTCAAGTAATGACAAGGCCCTGGGACTCCTGTGTGGGAAGGACGCTGACGCCTGTAATGCCAC

CAACTGGATTGAATACATGTTCAATAAGGACAATGGACAGGCACCTTTTACCATCACTCCTGTGTTTTCA

GATTTTCCAGTCCATGGGATGGAGCCCATGAACAATGCCACCAAAGGCTGTGACGAGTCTGTGGATGAGG

TCACAGCACCATGTAGCTGCCAAGACTGCTCTATTGTCTGTGGCCCCAAGCCCCAGCCCCCACCTCCTCC

TGCTCCCTGGACGATCCTTGGCTTGGACGCCATGTATGTCATCATGTGGATCACCTACATGGCGTTTTTG

CTTGTGTTTTTTGGAGCATTTTTTGCAGTGTGGTGCTACAGAAAACGGTATTTTGTCTCCGAGTACACTC

CCATCGATAGCAATATAGCTTTTTCTGTTAATGCAAGTGACAAAGGAGAGGCGTCCTGCTGTGACCCTGT

CAGCGCAGCATTTGAGGGCTGCTTGAGGCGGCTGTTCACACGCTGGGGGTCTTTCTGCGTCCGAAACCCT

GGCTGTGTCATTTTCTTCTCGCTGGTCTTCATTACTGCGTGTTCGTCAGGCCTGGTGTTTGTCCGGGTCA

CAACCAATCCAGTTGACCTCTGGTCAGCCCCCAGCAGCCAGGCTCGCCTGGAAAAAGAGTACTTTGACCA

GCACTTTGGGCCTTTCTTCCGGACGGAGCAGCTCATCATCCGGGCCCCTCTCACTGACAAACACATTTAC

CAGCCATACCCTTCGGGAGCTGATGTACCCTTTGGACCTCCGCTTGACATACAGATACTGCACCAGGTTC

TTGACTTACAAATAGCCATCGAAAACATTACTGCCTCTTATGACAATGAGACTGTGACACTTCAAGACAT

CTGCTTGGCCCCTCTTTCACCGTATAACACGAACTGCACCATTTTGAGTGTGTTAAATTACTTCCAGAAC

AGCCATTCCGTGCTGGACCACAAGAAAGGGGACGACTTCTTTGTGTATGCCGATTACCACACGCACTTTC

TGTACTGCGTACGGGCTCCTGCCTCTCTGAATGATACAAGTTTGCTCCATGACCCTTGTCTGGGTACGTT

TGGTGGACCAGTGTTCCCGTGGCTTGTGTTGGGAGGCTATGATGATCAAAACTACAATAACGCCACTGCC

CTTGTGATTACCTTCCCTGTCAATAATTACTATAATGATACAGAGAAGCTCCAGAGGGCCCAGGCCTGGG

AAAAAGAGTTTATTAATTTTGTGAAAAACTACAAGAATCCCAATCTGACCATTTCCTTCACTGCTGAACG

AAGTATTGAAGATGAACTAAATCGTGAAAGTGACAGTGATGTCTTCACCGTTGTAATTAGCTATGCCATC

ATGTTTCTATATATTTCCCTAGCCTTGGGGCACATGAAAAGCTGTCGCAGGCTTCTGGTGGATTCGAAGG

TCTCACTAGGCATCGCGGGCATCTTGATCGTGCTGAGCTCGGTGGCTTGCTCCTTGGGTGTCTTCAGCTA

CATTGGGTTGCCCTTGACCCTCATTGTGATTGAAGTCATCCCGTTCCTGGTGCTGGCTGTTGGAGTGGAC

AACATCTTCATTCTGGTGCAGGCCTACCAGAGAGATGAACGTCTTCAAGGGGAAACCCTGGATCAGCAGC

TGGGCAGGGTCCTAGGAGAAGTGGCTCCCAGTATGTTCCTGTCATCCTTTTCTGAGACTGTAGCATTTTT

CTTAGGAGCATTGTCCGTGATGCCAGCCGTGCACACCTTCTCTCTCTTTGCGGGATTGGCAGTCTTCATT

GACTTTCTTCTGCAGATTACCTGTTTCGTGAGTCTCTTGGGGTTAGACATTAAACGTCAAGAGAAAAATC

GGCTAGACATCTTTTGCTGTGTCAGAGGTGCTGAAGATGGAACAAGCGTCCAGGCCTCAGAGAGCTGTTT

GTTTCGCTTCTTCAAAAACTCCTATTCTCCACTTCTGCTAAAGGACTGGATGAGACCAATTGTGATAGCA

ATATTTGTGGGTGTTCTGTCATTCAGCATCGCAGTCCTGAACAAAGTAGATATTGGATTGGATCAGTCTC

TTTCGATGCCAGATGACTCCTACATGGTGGATTATTTCAAATCCATCAGTCAGTACCTGCATGCGGGTCC

GCCTGTGTACTTTGTCCTGGAGGAAGGGCACGACTACACTTCTTCCAAGGGGCAGAACATGGTGTGCGGC

GGCATGGGCTGCAACAATGATTCCCTGGTGCAGCAGATATTTAACGCGGCGCAGCTGGACAACTATACCC

GAATAGGCTTCGCCCCCTCGTCCTGGATCGACGATTATTTCGACTGGGTGAAGCCACAGTCGTCTTGCTG

TCGAGTGGACAATATCACTGACCAGTTCTGCAATGCTTCAGTGGTTGACCCTGCCTGCGTTCGCTGCAGG

CCTCTGACTCCGGAAGGCAAACAGAGGCCTCAGGGGGGAGACTTCATGAGATTCCTGCCCATGTTCCTTT

CGGATAACCCTAACCCCAAGTGTGGCAAAGGGGGACATGCTGCCTATAGTTCTGCAGTTAACATCCTCCT

TGGCCATGGCACCAGGGTCGGAGCCACGTACTTCATGACCTACCACACCGTGCTGCAGACCTCTGCTGAC

TTTATTGACGCTCTGAAGAAAGCCCGACTTATAGCCAGTAATGTCACCGAAACCATGGGCATTAACGGCA

GTGCCTACCGAGTATTTCCTTACAGTGTGTTTTATGTCTTCTACGAACAGTACCTGACCATCATTGACGA

CACTATCTTCAACCTCGGTGTGTCCCTGGGCGCGATATTTCTGGTGACCATGGTCCTCCTGGGCTGTGAG

CTCTGGTCTGCAGTCATCATGTGTGCCACCATCGCCATGGTCTTGGTCAACATGTTTGGAGTTATGTGGC

TCTGGGGCATCAGTCTGAACGCTGTATCCTTGGTCAACCTGGTGATGAGCTGTGGCATCTCCGTGGAGTT

CTGCAGCCACATAACCAGAGCGTTCACGGTGAGCATGAAAGGCAGCCGCGTGGAGCGCGCGGAAGAGGCA

CTTGCCCACATGGGCAGCTCCGTGTTCAGTGGAATCACACTTACAAAATTTGGAGGGATTGTGGTGTTGG

CTTTTGCCAAATCTCAAATTTTCCAGATATTCTACTTCAGGATGTATTTGGCCATGGTCTTACTGGGAGC

CACTCACGGATTAATATTTCTCCCTGTCTTACTCAGTTACATAGGGCCATCAGTAAATAAAGCCAAAAGT

TGTGCCACTGAAGAGCGATACAAAGGAACAGAGCGCGAACGGCTTCTAAATTTCTAG

>lcl|NM_030759.5_cds_NP_110386.2_1 [gene=NRBF2] [db_xref=CCDS:CCDS7268.1] [protein=nuclear receptor-binding factor 2 isoform 1] [protein_id=NP_110386.2] [location=144..1007] [gbkey=CDS]

ATGGAAGTAATGGAAGGACCCCTCAACCTGGCTCATCAACAGAGCAGACGAGCAGACCGTTTATTAGCTG

CAGGCAAATACGAAGAGGCTATTTCTTGTCACAAAAAGGCTGCAGCATATCTTTCTGAAGCCATGAAGCT

GACACAGTCAGAGCAGGCTCATCTTTCACTGGAATTGCAAAGGGATAGCCATATGAAACAGCTCCTCCTC

ATCCAAGAGAGATGGAAAAGGGCCCAGCGTGAAGAAAGATTGAAAGCCCAGCAGAACACAGACAAGGATG

CAGCTGCCCATCTTCAGACATCTCACAAACCCTCTGCAGAGGATGCAGAGGGCCAGAGTCCCCTTTCTCA

GAAGTACAGCCCTTCCACAGAGAAATGCCTGCCTGAGATTCAGGGGATCTTTGACAGGGATCCAGACACA

CTACTTTATTTACTTCAGCAAAAGAGTGAGCCAGCAGAGCCATGTATTGGAAGCAAAGCCCCAAAAGATG

ATAAAACAATTATAGAGGAGCAGGCAACCAAAATTGCAGATTTGAAGAGGCATGTGGAATTCCTTGTGGC

TGAGAATGAAAGATTAAGGAAAGAAAATAAACAACTAAAGGCTGAAAAGGCCAGACTTCTAAAAGGTCCA

ATAGAAAAGGAGCTGGATGTAGATGCTGATTTTGTAGAAACGTCAGAGTTATGGAGCTTGCCACCACATG

CAGAAACTGCTACAGCCTCCTCAACCTGGCAGAAGTTCGCAGCAAATACTGGGAAAGCCAAGGACATTCC

AATCCCCAATCTTCCTCCCTTGGATTTTCCATCTCCAGAACTTCCTCTTATGGAGCTCTCTGAGGATATT

CTGAAAGGATTTATGAATAATTAA

>lcl|NM_001159995.3_cds_NP_001153467.1_1 [gene=NRG1] [db_xref=GeneID:3084,HGNC:HGNC:7997,MIM:142445] [protein=pro-neuregulin-1, membrane-bound isoform isoform HRG-beta1c] [protein_id=NP_001153467.1] [location=151..1974] [gbkey=CDS]

ATGGGGAAAGGACGCGCGGGCCGAGTTGGCACCACAGCCTTGCCTCCCCGATTGAAAGAGATGAAAAGCC

AGGAATCGGCTGCAGGTTCCAAACTAGTCCTTCGGTGTGAAACCAGTTCTGAATACTCCTCTCTCAGATT

CAAGTGGTTCAAGAATGGGAATGAATTGAATCGAAAAAACAAACCACAAAATATCAAGATACAAAAAAAG

CCAGGGAAGTCAGAACTTCGCATTAACAAAGCATCACTGGCTGATTCTGGAGAGTATATGTGCAAAGTGA

TCAGCAAATTAGGAAATGACAGTGCCTCTGCCAATATCACCATCGTGGAATCAAACGAGATCATCACTGG

TATGCCAGCCTCAACTGAAGGAGCATATGTGTCTTCAGCTACATCTACATCCACCACTGGGACAAGCCAT

CTTGTAAAATGTGCGGAGAAGGAGAAAACTTTCTGTGTGAATGGAGGGGAGTGCTTCATGGTGAAAGACC

TTTCAAACCCCTCGAGATACTTGTGCAAGTGCCCAAATGAGTTTACTGGTGATCGCTGCCAAAACTACGT

AATGGCCAGCTTCTACAAGCATCTTGGGATTGAATTTATGGAGGCGGAGGAGCTGTACCAGAAGAGAGTG

CTGACCATAACCGGCATCTGCATCGCCCTCCTTGTGGTCGGCATCATGTGTGTGGTGGCCTACTGCAAAA

CCAAGAAACAGCGGAAAAAGCTGCATGACCGTCTTCGGCAGAGCCTTCGGTCTGAACGAAACAATATGAT

GAACATTGCCAATGGGCCTCACCATCCTAACCCACCCCCCGAGAATGTCCAGCTGGTGAATCAATACGTA

TCTAAAAACGTCATCTCCAGTGAGCATATTGTTGAGAGAGAAGCAGAGACATCCTTTTCCACCAGTCACT

ATACTTCCACAGCCCATCACTCCACTACTGTCACCCAGACTCCTAGCCACAGCTGGAGCAACGGACACAC

TGAAAGCATCCTTTCCGAAAGCCACTCTGTAATCGTGATGTCATCCGTAGAAAACAGTAGGCACAGCAGC

CCAACTGGGGGCCCAAGAGGACGTCTTAATGGCACAGGAGGCCCTCGTGAATGTAACAGCTTCCTCAGGC

ATGCCAGAGAAACCCCTGATTCCTACCGAGACTCTCCTCATAGTGAAAGGTATGTGTCAGCCATGACCAC

CCCGGCTCGTATGTCACCTGTAGATTTCCACACGCCAAGCTCCCCCAAATCGCCCCCTTCGGAAATGTCT

CCACCCGTGTCCAGCATGACGGTGTCCATGCCTTCCATGGCGGTCAGCCCCTTCATGGAAGAAGAGAGAC

CTCTACTTCTCGTGACACCACCAAGGCTGCGGGAGAAGAAGTTTGACCATCACCCTCAGCAGTTCAGCTC

CTTCCACCACAACCCCGCGCATGACAGTAACAGCCTCCCTGCTAGCCCCTTGAGGATAGTGGAGGATGAG

GAGTATGAAACGACCCAAGAGTACGAGCCAGCCCAAGAGCCTGTTAAGAAACTCGCCAATAGCCGGCGGG

CCAAAAGAACCAAGCCCAATGGCCACATTGCTAACAGATTGGAAGTGGACAGCAACACAAGCTCCCAGAG

CAGTAACTCAGAGAGTGAAACAGAAGATGAAAGAGTAGGTGAAGATACGCCTTTCCTGGGCATACAGAAC

CCCCTGGCAGCCAGTCTTGAGGCAACACCTGCCTTCCGCCTGGCTGACAGCAGGACTAACCCAGCAGGCC

GCTTCTCGACACAGGAAGAAATCCAGGCCAGGCTGTCTAGTGTAATTGCTAACCAAGACCCTATTGCTGT

ATAA

>lcl|NM_013982.3_cds_NP_053585.1_1 [gene=NRG2] [db_xref=GeneID:9542,HGNC:HGNC:7998,MIM:603818] [protein=pro-neuregulin-2, membrane-bound isoform isoform 3] [protein_id=NP_053585.1] [location=231..2807] [gbkey=CDS]

ATGCGGCAGGTTTGCTGCTCAGCGCTGCCGCCGCCGCCACTGGAGAAGGGTCGGTGCAGCAGCTACAGCG

ACAGCAGCAGCAGCAGCAGCGAGAGGAGCAGCAGCAGCAGCAGCAGCAGCGAGAGCGGCAGCAGCAG

CAGGAGCAGCAGCAACAACAGCAGCATCTCTCGTCCCGCTGCGCCCCCAGAGCCGCGGCCGCAGCAACAG

CCGCAGCCCCGCAGCCCCGCAGCCCGGAGAGCCGCCGCCCGTTCGCGAGCCGCAGCCGCCGGCGGCATGA

GGCGCGACCCGGCCCCCGGCTTCTCCATGCTGCTCTTCGGTGTGTCGCTCGCCTGCTACTCGCCCAGCCT

CAAGTCAGTGCAGGACCAGGCGTACAAGGCACCCGTGGTGGTGGAGGGCAAGGTACAGGGGCTGGTCCCA

GCCGGCGGCTCCAGCTCCAACAGCACCCGAGAGCCGCCCGCCTCGGGTCGGGTGGCGTTGGTAAAGGTGC

TGGACAAGTGGCCGCTCCGGAGCGGGGGGCTGCAGCGCGAGCAGGTGATCAGCGTGGGCTCCTGTGTGCC

GCTCGAAAGGAACCAGCGCTACATCTTTTTCCTGGAGCCCACGGAACAGCCCTTAGTCTTTAAGACGGCC

TTTGCCCCCCTCGATACCAACGGCAAAAATCTCAAGAAAGAGGTGGGCAAGATCCTGTGCACTGACTGCG

CCACCCGGCCCAAGTTGAAGAAGATGAAGAGCCAGACGGGACAGGTGGGTGAGAAGCAATCGCTGAAGTG

TGAGGCAGCAGCCGGTAATCCCCAGCCTTCCTACCGTTGGTTCAAGGATGGCAAGGAGCTCAACCGCAGC

CGAGACATTCGCATCAAATATGGCAACGGCAGAAAGAACTCACGACTACAGTTCAACAAGGTGAAGGTGG

AGGACGCTGGGGAGTATGTCTGCGAGGCCGAGAACATCCTGGGGAAGGACACCGTCCGGGGCCGGCTTTA

CGTCAACAGCGTGAGCACCACCCTGTCATCCTGGTCGGGGCACGCCCGGAAGTGCAACGAGACAGCCAAG

TCCTATTGCGTCAATGGAGGCGTCTGCTACTACATCGAGGGCATCAACCAGCTCTCCTGCAAATGTCCAA

ATGGATTCTTCGGACAGAGATGTTTGGAGAAACTGCCTTTGCGATTGTACATGCCAGATCCTAAGCAAAA

GCACCTTGGATTTGAATTAAAGGAAGCCGAGGAGCTGTACCAGAAGAGGGTCCTGACCATCACGGGCATC

TGCGTGGCTCTGCTGGTCGTGGGCATCGTCTGTGTGGTGGCCTACTGCAAGACCAAAAAACAGCGGAAGC

AGATGCACAACCACCTCCGGCAGAACATGTGCCCGGCCCATCAGAACCGGAGCTTGGCCAATGGGCCCAG

CCACCCCCGGCTGGACCCAGAGGAGATCCAGATGGCAGATTATATTTCCAAGAACGTGCCAGCCACAGAC

CATGTCATCAGGAGAGAAACTGAGACCACCTTCTCTGGGAGCCACTCCTGTTCTCCTTCTCACCACTGCT

CCACAGCCACACCCACCTCCAGCCACAGACACGAGAGCCACACGTGGAGCCTGGAACGTTCTGAGAGCCT

GACTTCTGACTCCCAGTCGGGGATCATGCTATCATCAGTGGGTACCAGCAAATGCAACAGCCCAGCATGT

GTGGAGGCCCGGGCAAGGCGGGCAGCAGCCTACAACCTGGAGGAGCGGCGCAGGGCCACCGCGCCACCCT

ATCACGATTCCGTGGACTCCCTTCGCGACTCCCCACACAGCGAGAGGTACGTGTCGGCCCTGACCACGCC

CGCGCGCCTCTCGCCCGTGGACTTCCACTACTCGCTGGCCACGCAGGTGCCAACTTTCGAGATCACGTCC

CCCAACTCGGCGCACGCCGTGTCGCTGCCGCCGGCGGCGCCCATCAGTTACCGCCTGGCCGAGCAGCAGC

CGTTACTGCGGCACCCGGCGCCCCCCGGCCCGGGACCCGGACCCGGGCCCGGGCCCGGGCCCGGCGCAGA

CATGCAGCGCAGCTATGACAGCTACTATTACCCCGCGGCGGGGCCCGGACCGCGGCGCGGGACCTGCGCG

CTCGGCGGCAGCCTGGGCAGCCTGCCTGCCAGCCCCTTCCGCATCCCCGAGGACGACGAGTACGAGACCA

CGCAGGAGTGCGCGCCCCCGCCGCCGCCGCGGCCGCGCGCGCGCGGTGCGTCCCGCAGGACGTCGGCGGG

GCCCCGGCGCTGGCGCCGCTCGCGCCTCAACGGGCTGGCGGCGCAGCGCGCACGGGCGGCGAGGGACTCG

CTGTCGCTGAGCAGCGGCTCGGGCGGCGGCTCAGCCTCGGCGTCGGACGACGACGCGGACGACGCGGACG

GGGCGCTGGCGGCCGAGAGCACACCTTTCCTGGGCCTGCGTGGGGCGCACGACGCGCTGCGCTCGGACTC

GCCGCCACTGTGCCCGGCGGCCGACAGCAGGACTTACTACTCACTGGACAGCCACAGCACGCGGGCCAGC

AGCAGACACAGCCGCGGGCCGCCCCCGCGGGCCAAGCAGGACTCGGCGCCACTCTAG

>lcl|NM_001010848.4_cds_NP_001010848.2_1 [gene=NRG3] [db_xref=CCDS:CCDS31233.1] [protein=pro-neuregulin-3, membrane-bound isoform isoform 1] [protein_id=NP_001010848.2] [location=148..2238] [gbkey=CDS]

ATGAGTGAAGGGGCGGCCGCTGCCTCGCCACCTGGTGCCGCTTCGGCAGCCGCCGCCTCGGCCGAGGAGG

GCACCGCGGCGGCTGCGGCGGCGGCAGCGGCGGGCGGGGGCCCGGACGGCGGCGGCGAAGGGGCGGCCGA

GCCCCCCCGGGAGTTACGCTGTAGCGACTGCATCGTGTGGAACCGGCAGCAGACGTGGCTGTGCGTGGTA

CCTCTGTTCATCGGCTTCATCGGCCTGGGGCTCAGCCTCATGCTTCTCAAATGGATCGTGGTGGGCTCCG

TCAAGGAGTACGTGCCCACCGACCTAGTGGACTCCAAGGGGATGGGCCAGGACCCCTTCTTCCTCTCCAA

GCCCAGCTCTTTCCCCAAGGCCATGGAGACCACCACCACTACCACTTCCACCACGTCCCCCGCCACCCCC

TCCGCCGGGGGTGCCGCCTCCTCCAGGACGCCCAACCGGATTAGCACTCGCCTGACCACCATCACGCGGG

CGCCCACTCGCTTCCCCGGGCACCGGGTGCCCATCCGGGCCAGCCCGCGCTCCACCACAGCACGGAACAC

TGCGGCCCCTGCGACGGTCCCGTCCACCACGGCCCCGTTCTTCAGTAGCAGCACGCTGGGCTCCCGACCC

CCGGTGCCAGGAACTCCAAGTACCCAGGCAATGCCCTCCTGGCCTACTGCGGCATACGCTACCTCCTCCT

ACCTTCACGATTCTACTCCCTCCTGGACCCTGTCTCCCTTTCAGGATGCTGCCTCCTCTTCTTCCTCTTC

TTCCTCCTCCGCTACCACCACCACACCAGAAACTAGCACCAGCCCCAAATTTCATACGACGACATATTCC

ACAGAGCGATCCGAGCACTTCAAACCCTGCCGAGACAAGGACCTTGCATACTGTCTCAATGATGGCGAGT

GCTTTGTGATCGAAACCCTGACCGGATCCCATAAACACTGTCGGTGCAAAGAAGGCTACCAAGGAGTCCG

TTGTGATCAATTTCTGCCGAAAACTGATTCCATCTTATCGGATCCAACAGACCACTTGGGGATTGAATTC

ATGGAGAGTGAAGAAGTTTATCAAAGGCAGGTGCTGTCAATTTCATGTATCATCTTTGGAATTGTCATCG

TGGGCATGTTCTGTGCAGCATTCTACTTCAAAAGCAAGAAACAAGCTAAACAAATCCAAGAGCAGCTGAA

AGTGCCACAAAATGGTAAAAGCTACAGTCTCAAAGCATCCAGCACAATGGCAAAGTCAGAGAACTTGGTG

AAGAGCCATGTCCAGCTGCAAAATTATTCAAAGGTGGAAAGGCATCCTGTGACTGCATTGGAGAAAATGA

TGGAGTCAAGTTTTGTCGGCCCCCAGTCATTCCCTGAGGTCCCTTCTCCTGACAGAGGAAGCCAGTCTGT

CAAACACCACAGGAGTCTATCCTCTTGCTGCAGCCCAGGGCAAAGAAGTGGCATGCTCCATAGGAATGCC

TTCAGAAGGACACCCCCGTCACCCCGAAGTAGGCTAGGTGGAATTGTGGGACCAGCATATCAGCAACTCG

AAGAATCAAGGATCCCAGACCAGGATACGATACCTTGCCAAGGGTATTCATCCAGTGGTTTAAAAACCCA

ACGAAATACATCAATAAATATGCAACTGCCTTCAAGAGAGACAAACCCCTATTTTAATAGCTTGGAGCAA

AAGGACCTGGTGGGCTATTCATCCACAAGGGCCAGTTCTGTGCCCATCATCCCTTCAGTGGGTTTAGAGG

AAACCTGCCTGCAAATGCCAGGGATTTCTGAAGTCAAAAGCATCAAATGGTGCAAAAACTCCTATTCAGC

TGACGTTGTCAATGTGAGTATTCCAGTCAGCGATTGTCTTATAGCAGAACAACAAGAAGTGAAAATATTG

CTAGAAACTGTCCAGGAGCAGATCCGAATTCTGACTGATGCCAGACGGTCAGAAGACTACGAACTGGCCA

GCGTAGAAACCGAGGACAGTGCAAGCGAAAACACAGCCTTTCTCCCCCTGAGTCCCACAGCCAAATCAGA

ACGAGAGGCGCAATTTGTCTTAAGAAATGAAATACAAAGAGACTCTGCATTGACCAAGTGA

>lcl|NM_000918.4_cds_NP_000909.2_1 [gene=P4HB] [db_xref=CCDS:CCDS11787.1] [protein=protein disulfide-isomerase precursor] [protein_id=NP_000909.2] [location=65..1591] [gbkey=CDS]

ATGCTGCGCCGCGCTCTGCTGTGCCTGGCCGTGGCCGCCCTGGTGCGCGCCGACGCCCCCGAGGAGGAGG

ACCACGTCCTGGTGCTGCGGAAAAGCAACTTCGCGGAGGCGCTGGCGGCCCACAAGTACCTGCTGGTGGA

GTTCTATGCCCCTTGGTGTGGCCACTGCAAGGCTCTGGCCCCTGAGTATGCCAAAGCCGCTGGGAAGCTG

AAGGCAGAAGGTTCCGAGATCAGGTTGGCCAAGGTGGACGCCACGGAGGAGTCTGACCTGGCCCAGCAGT

ACGGCGTGCGCGGCTATCCCACCATCAAGTTCTTCAGGAATGGAGACACGGCTTCCCCCAAGGAATATAC

AGCTGGCAGAGAGGCTGATGACATCGTGAACTGGCTGAAGAAGCGCACGGGCCCGGCTGCCACCACCCTG

CCTGACGGCGCAGCTGCAGAGTCCTTGGTGGAGTCCAGCGAGGTGGCTGTCATCGGCTTCTTCAAGGACG

TGGAGTCGGACTCTGCCAAGCAGTTTTTGCAGGCAGCAGAGGCCATCGATGACATACCATTTGGGATCAC

TTCCAACAGTGACGTGTTCTCCAAATACCAGCTCGACAAAGATGGGGTTGTCCTCTTTAAGAAGTTTGAT

GAAGGCCGGAACAACTTTGAAGGGGAGGTCACCAAGGAGAACCTGCTGGACTTTATCAAACACAACCAGC

TGCCCCTTGTCATCGAGTTCACCGAGCAGACAGCCCCGAAGATTTTTGGAGGTGAAATCAAGACTCACAT

CCTGCTGTTCTTGCCCAAGAGTGTGTCTGACTATGACGGCAAACTGAGCAACTTCAAAACAGCAGCCGAG

AGCTTCAAGGGCAAGATCCTGTTCATCTTCATCGACAGCGACCACACCGACAACCAGCGCATCCTCGAGT

TCTTTGGCCTGAAGAAGGAAGAGTGCCCGGCCGTGCGCCTCATCACCCTGGAGGAGGAGATGACCAAGTA

CAAGCCCGAATCGGAGGAGCTGACGGCAGAGAGGATCACAGAGTTCTGCCACCGCTTCCTGGAGGGCAAA

ATCAAGCCCCACCTGATGAGCCAGGAGCTGCCGGAGGACTGGGACAAGCAGCCTGTCAAGGTGCTTGTTG

GGAAGAACTTTGAAGACGTGGCTTTTGATGAGAAAAAAAACGTCTTTGTGGAGTTCTATGCCCCATGGTG

TGGTCACTGCAAACAGTTGGCTCCCATTTGGGATAAACTGGGAGAGACGTACAAGGACCATGAGAACATC

GTCATCGCCAAGATGGACTCGACTGCCAACGAGGTGGAGGCCGTCAAAGTGCACAGCTTCCCCACACTCA

AGTTCTTTCCTGCCAGTGCCGACAGGACGGTCATTGATTACAACGGGGAACGCACGCTGGATGGTTTTAA

GAAATTCCTGGAGAGCGGTGGCCAGGATGGGGCAGGGGATGATGACGATCTCGAGGACCTGGAAGAAGCA

GAGGAGCCAGACATGGAGGAAGACGATGATCAGAAAGCTGTGAAAGATGAACTGTAA

>lcl|NM_001618.4_cds_NP_001609.2_1 [gene=PARP1] [db_xref=CCDS:CCDS1554.1] [protein=poly [ADP-ribose] polymerase 1] [protein_id=NP_001609.2] [location=165..3209] [gbkey=CDS]

ATGGCGGAGTCTTCGGATAAGCTCTATCGAGTCGAGTACGCCAAGAGCGGGCGCGCCTCTTGCAAGAAAT

GCAGCGAGAGCATCCCCAAGGACTCGCTCCGGATGGCCATCATGGTGCAGTCGCCCATGTTTGATGGAAA

AGTCCCACACTGGTACCACTTCTCCTGCTTCTGGAAGGTGGGCCACTCCATCCGGCACCCTGACGTTGAG

GTGGATGGGTTCTCTGAGCTTCGGTGGGATGACCAGCAGAAAGTCAAGAAGACAGCGGAAGCTGGAGGAG

TGACAGGCAAAGGCCAGGATGGAATTGGTAGCAAGGCAGAGAAGACTCTGGGTGACTTTGCAGCAGAGTA

TGCCAAGTCCAACAGAAGTACGTGCAAGGGGTGTATGGAGAAGATAGAAAAGGGCCAGGTGCGCCTGTCC

AAGAAGATGGTGGACCCGGAGAAGCCACAGCTAGGCATGATTGACCGCTGGTACCATCCAGGCTGCTTTG

TCAAGAACAGGGAGGAGCTGGGTTTCCGGCCCGAGTACAGTGCGAGTCAGCTCAAGGGCTTCAGCCTCCT

TGCTACAGAGGATAAAGAAGCCCTGAAGAAGCAGCTCCCAGGAGTCAAGAGTGAAGGAAAGAGAAAAGGC

GATGAGGTGGATGGAGTGGATGAAGTGGCGAAGAAGAAATCTAAAAAAGAAAAAGACAAGGATAGTAAGC

TTGAAAAAGCCCTAAAGGCTCAGAACGACCTGATCTGGAACATCAAGGACGAGCTAAAGAAAGTGTGTTC

AACTAATGACCTGAAGGAGCTACTCATCTTCAACAAGCAGCAAGTGCCTTCTGGGGAGTCGGCGATCTTG

GACCGAGTAGCTGATGGCATGGTGTTCGGTGCCCTCCTTCCCTGCGAGGAATGCTCGGGTCAGCTGGTCT

TCAAGAGCGATGCCTATTACTGCACTGGGGACGTCACTGCCTGGACCAAGTGTATGGTCAAGACACAGAC

ACCCAACCGGAAGGAGTGGGTAACCCCAAAGGAATTCCGAGAAATCTCTTACCTCAAGAAATTGAAGGTT

AAAAAACAGGACCGTATATTCCCCCCAGAAACCAGCGCCTCCGTGGCGGCCACGCCTCCGCCCTCCACAG

CCTCGGCTCCTGCTGCTGTGAACTCCTCTGCTTCAGCAGATAAGCCATTATCCAACATGAAGATCCTGAC

TCTCGGGAAGCTGTCCCGGAACAAGGATGAAGTGAAGGCCATGATTGAGAAACTCGGGGGGAAGTTGACG

GGGACGGCCAACAAGGCTTCCCTGTGCATCAGCACCAAAAAGGAGGTGGAAAAGATGAATAAGAAGATGG

AGGAAGTAAAGGAAGCCAACATCCGAGTTGTGTCTGAGGACTTCCTCCAGGACGTCTCCGCCTCCACCAA

GAGCCTTCAGGAGTTGTTCTTAGCGCACATCTTGTCCCCTTGGGGGGCAGAGGTGAAGGCAGAGCCTGTT

GAAGTTGTGGCCCCAAGAGGGAAGTCAGGGGCTGCGCTCTCCAAAAAAAGCAAGGGCCAGGTCAAGGAGG

AAGGTATCAACAAATCTGAAAAGAGAATGAAATTAACTCTTAAAGGAGGAGCAGCTGTGGATCCTGATTC

TGGACTGGAACACTCTGCGCATGTCCTGGAGAAAGGTGGGAAGGTCTTCAGTGCCACCCTTGGCCTGGTG

GACATCGTTAAAGGAACCAACTCCTACTACAAGCTGCAGCTTCTGGAGGACGACAAGGAAAACAGGTATT

GGATATTCAGGTCCTGGGGCCGTGTGGGTACGGTGATCGGTAGCAACAAACTGGAACAGATGCCGTCCAA

GGAGGATGCCATTGAGCACTTCATGAAATTATATGAAGAAAAAACCGGGAACGCTTGGCACTCCAAAAAT

TTCACGAAGTATCCCAAAAAGTTCTACCCCCTGGAGATTGACTATGGCCAGGATGAAGAGGCAGTGAAGA

AGCTGACAGTAAATCCTGGCACCAAGTCCAAGCTCCCCAAGCCAGTTCAGGACCTCATCAAGATGATCTT

TGATGTGGAAAGTATGAAGAAAGCCATGGTGGAGTATGAGATCGACCTTCAGAAGATGCCCTTGGGGAAG

CTGAGCAAAAGGCAGATCCAGGCCGCATACTCCATCCTCAGTGAGGTCCAGCAGGCGGTGTCTCAGGGCA

GCAGCGACTCTCAGATCCTGGATCTCTCAAATCGCTTTTACACCCTGATCCCCCACGACTTTGGGATGAA

GAAGCCTCCGCTCCTGAACAATGCAGACAGTGTGCAGGCCAAGGTGGAAATGCTTGACAACCTGCTGGAC

ATCGAGGTGGCCTACAGTCTGCTCAGGGGAGGGTCTGATGATAGCAGCAAGGATCCCATCGATGTCAACT

ATGAGAAGCTCAAAACTGACATTAAGGTGGTTGACAGAGATTCTGAAGAAGCCGAGATCATCAGGAAGTA

TGTTAAGAACACTCATGCAACCACACACAATGCGTATGACTTGGAAGTCATCGATATCTTTAAGATAGAG

CGTGAAGGCGAATGCCAGCGTTACAAGCCCTTTAAGCAGCTTCATAACCGAAGATTGCTGTGGCACGGGT

CCAGGACCACCAACTTTGCTGGGATCCTGTCCCAGGGTCTTCGGATAGCCCCGCCTGAAGCGCCCGTGAC

AGGCTACATGTTTGGTAAAGGGATCTATTTCGCTGACATGGTCTCCAAGAGTGCCAACTACTGCCATACG

TCTCAGGGAGACCCAATAGGCTTAATCCTGTTGGGAGAAGTTGCCCTTGGAAACATGTATGAACTGAAGC

ACGCTTCACATATCAGCAAGTTACCCAAGGGCAAGCACAGTGTCAAAGGTTTGGGCAAAACTACCCCTGA

TCCTTCAGCTAACATTAGTCTGGATGGTGTAGACGTTCCTCTTGGGACCGGGATTTCATCTGGTGTGAAT

GACACCTCTCTACTATATAACGAGTACATTGTCTATGATATTGCTCAGGTAAATCTGAAGTATCTGCTGA

AACTGAAATTCAATTTTAAGACCTCCCTGTGGTAA

>lcl|NM_003768.5_cds_NP_003759.1_1 [gene=PEA15] [db_xref=CCDS:CCDS1199.1] [protein=astrocytic phosphoprotein PEA-15 isoform b] [protein_id=NP_003759.1] [location=142..534] [gbkey=CDS]

ATGGCTGAGTACGGGACCCTCCTGCAAGACCTGACCAACAACATCACCCTTGAAGATCTAGAACAGCTCA

AGTCGGCCTGCAAGGAAGACATCCCCAGCGAAAAGAGTGAGGAGATCACTACTGGCAGTGCCTGGTTTAG

CTTCCTGGAGAGCCACAACAAGCTGGACAAAGACAACCTCTCCTACATTGAGCACATCTTTGAGATCTCC

CGCCGTCCTGACCTACTCACTATGGTGGTTGACTACAGAACCCGTGTGCTGAAGATCTCTGAGGAGGATG

AGCTGGACACCAAGCTAACCCGTATCCCCAGTGCCAAGAAGTACAAAGACATTATCCGGCAGCCCTCTGA

GGAAGAGATCATCAAATTGGCTCCCCCACCGAAGAAGGCCTGA

>lcl|NM_014389.3_cds_NP_055204.4_1 [gene=PELP1] [db_xref=CCDS:CCDS58503.2] [protein=proline-, glutamic acid- and leucine-rich protein 1 isoform 1] [protein_id=NP_055204.4] [location=27..3419] [gbkey=CDS]

ATGGCGGCAGCCGTTCTGAGTGGGCCCTCTGCGGGCTCCGCGGCTGGGGTTCCTGGCGGGACCGGGGGTC

TCTCGGCAGTGAGCTCGGGCCCGCGGCTCCGCCTGCTGCTGCTGGAGAGTGTTTCTGGTTTGCTGCAACC

TCGAACGGGGTCTGCCGTTGCTCCGGTGCATCCCCCAAACCGCTCGGCCCCACATTTGCCCGGGCTCATG

TGCCTATTGCGGCTGCATGGGTCGGTGGGCGGGGCCCAGAACCTTTCAGCTCTTGGGGCATTGGTGAGTC

TCAGTAATGCACGTCTCAGTTCCATCAAAACTCGGTTTGAGGGCCTGTGTCTGCTGTCCCTGCTGGTAGG

GGAGAGCCCCACAGAGCTATTCCAGCAGCACTGTGTGTCTTGGCTTCGGAGCATTCAGCAGGTGTTACAG

ACCCAGGACCCGCCTGCCACAATGGAGCTGGCCGTGGCTGTCCTGAGGGACCTCCTCCGATATGCAGCCC

AGCTGCCTGCACTGTTCCGGGACATCTCCATGAACCACCTCCCTGGCCTTCTCACCTCCCTGCTGGGCCT

CAGGCCAGAGTGTGAGCAGTCAGCATTGGAAGGAATGAAGGCTTGTATGACCTATTTCCCTCGGGCTTGT

GGTTCTCTCAAAGGCAAGCTGGCCTCATTTTTTCTGTCTAGGGTGGATGCCTTGAGCCCTCAGCTCCAAC

AGTTGGCCTGTGAGTGTTATTCCCGGCTGCCCTCTTTAGGGGCTGGCTTTTCCCAAGGCCTGAAGCACAC

CGAGAGCTGGGAGCAGGAGCTACACAGTCTGCTGGCCTCACTGCACACCCTGCTGGGGGCCCTGTACGAG

GGAGCAGAGACTGCTCCTGTGCAGAATGAAGGCCCTGGGGTGGAGATGCTGCTGTCCTCAGAAGATGGTG

ATGCCCATGTCCTTCTCCAGCTTCGGCAGAGGTTTTCGGGACTGGCCCGCTGCCTAGGGCTCATGCTCAG

CTCTGAGTTTGGAGCTCCCGTGTCCGTCCCTGTGCAGGAAATCCTGGATTTCATCTGCCGGACCCTCAGC

GTCAGTAGCAAGAATATTAGCTTGCATGGAGATGGTCCCCTGCGGCTGCTGCTGCTGCCCTCTATCCACC

TTGAGGCCTTGGACCTGCTGTCTGCACTCATCCTCGCGTGTGGAAGCCGGCTCTTGCGCTTTGGGATCCT

GATCGGCCGCCTGCTTCCCCAGGTCCTCAATTCCTGGAGCATCGGTAGAGATTCCCTCTCTCCAGGCCAG

GAGAGGCCTTACAGCACGGTTCGGACCAAGGTGTATGCGATATTAGAGCTGTGGGTGCAGGTTTGTGGGG

CCTCGGCGGGAATGCTTCAGGGAGGAGCCTCTGGAGAGGCCCTGCTCACCCACCTGCTCAGCGACATCTC

CCCGCCAGCTGATGCCCTTAAGCTGCGTAGCCCGCGGGGGAGCCCTGATGGGAGTTTGCAGACTGGGAAG

CCTAGCGCCCCCAAGAAGCTAAAGCTGGATGTGGGGGAAGCTATGGCCCCGCCAAGCCACCGGAAAGGGG

ATAGCAATGCCAACAGCGACGTGTGTGCGGCTGCACTCAGAGGCCTCAGCCGGACCATCCTCATGTGTGG

GCCTCTCATCAAGGAGGAGACTCACAGGAGACTGCATGACCTGGTCCTCCCCCTGGTCATGGGTGTACAG

CAGGGTGAGGTCCTAGGCAGCTCCCCGTACACGAGCTCCCGCTGCCGCCGTGAACTCTACTGCCTGCTGC

TGGCGCTGCTGCTGGCCCCGTCTCCTCGCTGCCCACCTCCTCTTGCCTGTGCCCTGCAAGCCTTCTCCCT

CGGCCAGCGAGAAGATAGCCTTGAGGTCTCCTCTTTCTGCTCAGAAGCACTGGTGACCTGTGCTGCTCTG

ACCCACCCCCGGGTTCCTCCCCTGCAGCCCATGGGCCCCACCTGCCCCACACCTGCTCCAGTTCCCCCTC

CTGAGGCCCCATCGCCCTTCAGGGCCCCACCGTTCCATCCTCCGGGCCCCATGCCCTCAGTGGGCTCCAT

GCCCTCAGCAGGCCCCATGCCTTCAGCAGGCCCCATGCCCTCAGCAGGCCCTGTGCCCTCGGCACGCCCT

GGACCTCCCACCACAGCCAACCACCTAGGCCTTTCTGTCCCAGGCCTAGTGTCTGTCCCTCCCCGGCTTC

TTCCTGGCCCTGAGAACCACCGGGCAGGCTCAAATGAGGACCCCATCCTTGCCCCTAGTGGGACTCCCCC

ACCTACTATACCCCCAGATGAAACTTTTGGGGGGAGAGTGCCCAGACCAGCCTTTGTCCACTATGACAAG

GAGGAGGCATCTGATGTGGAGATCTCCTTGGAAAGTGACTCTGATGACAGCGTGGTGATCGTGCCCGAGG

GGCTTCCCCCCCTGCCACCCCCACCACCCTCAGGTGCCACACCACCCCCTATAGCCCCCACTGGGCCACC

AACAGCCTCCCCTCCTGTGCCAGCGAAGGAGGAGCCTGAAGAACTTCCTGCAGCCCCAGGGCCTCTCCCG

CCACCCCCACCTCCGCCGCCGCCTGTTCCTGGTCCTGTGACGCTCCCTCCACCCCAGTTGGTCCCTGAAG

GGACTCCTGGTGGGGGAGGACCCCCAGCCCTGGAAGAGGATTTGACAGTTATTAATATCAACAGCAGTGA

TGAAGAGGAGGAGGAAGAGGAAGAAGAGGAAGAAGAAGAAGAGGAAGAAGAGGAAGAGGAGGAAGACTTT

GAGGAAGAGGAAGAGGATGAAGAGGAATATTTTGAAGAGGAAGAAGAGGAGGAAGAAGAGTTTGAGGAAG

AATTTGAGGAAGAAGAAGGTGAGTTAGAGGAAGAAGAAGAAGAGGAGGATGAGGAGGAGGAAGAAGAACT

GGAAGAGGTGGAAGACCTGGAGTTTGGCACAGCAGGAGGGGAGGTAGAAGAAGGTGCACCTCCACCCCCA

ACCCTGCCTCCAGCTCTGCCTCCCCCTGAGTCTCCCCCAAAGGTGCAGCCAGAACCCGAACCTGAACCCG

GGCTGCTTTTGGAAGTGGAGGAGCCAGGGACGGAGGAGGAGCGTGGGGCTGACACAGCTCCCACCCTGGC

CCCTGAAGCGCTCCCCTCCCAGGGAGAGGTGGAGAGGGAAGGGGAAAGCCCTGCGGCAGGGCCCCCTCCC

CAGGAGCTTGTTGAAGAAGAGCCCTCTGCTCCCCCAACCCTGTTGGAAGAGGAGACTGAGGATGGGAGTG

ACAAGGTGCAGCCCCCACCAGAGACACCTGCAGAAGAAGAGATGGAGACAGAGACAGAGGCCGAAGCTCT

CCAGGAAAAGGAGCAGGATGACACAGCTGCCATGCTGGCCGACTTCATCGATTGTCCCCCTGATGATGAG

AAGCCACCACCTCCCACAGAGCCTGACTCCTAG

>lcl|NM_004565.3_cds_NP_004556.1_1 [gene=PEX14] [db_xref=CCDS:CCDS30582.1] [protein=peroxisomal membrane protein PEX14] [protein_id=NP_004556.1] [location=18..1151] [gbkey=CDS]

ATGGCGTCCTCGGAGCAGGCAGAGCAGCCGAGCCAGCCAAGCTCTACTCCAGGAAGTGAAAATGTGCTGC

CTCGAGAGCCGCTGATTGCCACGGCAGTGAAGTTTCTACAGAATTCCCGGGTCCGCCAGAGCCCACTTGC

AACCAGGAGAGCATTCCTAAAGAAGAAAGGGCTGACAGATGAAGAGATTGATATGGCCTTCCAGCAGTCG

GGCACTGCTGCCGATGAGCCTTCGTCCTTGGGCCCAGCCACACAGGTGGTTCCTGTCCAGCCCCCTCACC

TCATATCTCAGCCATACAGTCCCGCAGGCTCCCGATGGCGAGATTACGGCGCCCTGGCCATCATCATGGC

AGGCATTGCATTTGGCTTTCACCAGCTCTACAAGAAATACCTGCTCCCCCTCATCCTGGGCGGCCGAGAG

GACAGAAAGCAGCTGGAGAGGATGGAGGCCGGTCTCTCTGAGCTGAGTGGCAGCGTGGCCCAGACAGTGA

CTCAGTTACAGACGACCCTCGCCTCCGTCCAGGAGCTGCTGATTCAGCAGCAGCAGAAGATCCAGGAGCT

TGCCCACGAGCTGGCCGCTGCCAAGGCCACCACATCCACCAACTGGATCCTGGAGTCCCAGAATATCAAC

GAACTCAAGTCCGAAATTAACTCCTTGAAAGGGCTTCTTTTAAATCGGAGGCAGTTCCCTCCATCCCCAT

CAGCCCCGAAGATCCCCTCCTGGCAGATCCCAGTCAAGTCACCGTCACCCTCCAGCCCTGCGGCCGTGAA

CCACCACAGCAGCAGCGACATCTCACCTGTCAGCAACGAGTCCACGTCGTCCTCGCCTGGGAAGGAGGGC

CACAGCCCCGAGGGCTCCACGGTCACCTACCACTTGCTGGGCCCCCAGGAGGAAGGCGAGGGGGTGGTGG

ACGTCAAGGGCCAGGTGCGGATGGAGGTGCAAGGCGAGGAGGAGAAGAGGGAGGACAAGGAGGACGAGGA

GGATGAGGAGGATGATGATGTGAGCCATGTGGACGAGGAGGACTGCCTGGGGGTGCAGAGGGAGGACCGC

CGGGGCGGGGATGGGCAGATCAACGAGCAGGTGGAGAAGCTGCGGCGGCCCGAGGGCGCCAGCAACGAGA

GTGAGCGGGACTAG

>lcl|NM_003630.3_cds_NP_003621.1_1 [gene=PEX3] [db_xref=CCDS:CCDS5199.1] [protein=peroxisomal biogenesis factor 3] [protein_id=NP_003621.1] [location=239..1360] [gbkey=CDS]

ATGCTGAGGTCTGTATGGAATTTTCTGAAACGCCACAAAAAGAAATGCATCTTCCTGGGCACGGTCCTTG

GAGGAGTATATATTCTGGGGAAATATGGACAGAAGAAAATCAGAGAAATACAGGAAAGGGAGGCTGCAGA

ATACATTGCCCAAGCACGACGACAATATCATTTTGAAAGTAACCAGAGGACTTGCAATATGACAGTGCTG

TCCATGCTTCCAACACTGAGAGAGGCCTTAATGCAGCAACTGAATTCCGAGAGCCTCACAGCTCTGCTAA

AAAACAGGCCTTCAAACAAGCTAGAAATATGGGAGGATCTGAAGATAATAAGTTTCACAAGAAGTACTGT

GGCTGTATACAGTACCTGTATGCTGGTTGTTCTTTTGCGGGTCCAGTTAAACATAATTGGTGGATATATT

TACCTGGATAATGCAGCAGTTGGCAAAAATGGCACTACAATTCTTGCTCCCCCAGATGTCCAACAGCAGT

ATTTATCAAGTATTCAGCACCTACTTGGAGATGGCCTGACAGAATTGATCACTGTCATTAAACAAGCTGT

GCAGAAGGTTTTAGGAAGTGTTTCTCTTAAACATTCTTTGTCCCTTTTGGACTTGGAGCAAAAACTAAAA

GAAATCAGAAATCTCGTTGAGCAGCATAAGTCTTCTTCTTGGATTAATAAAGATGGATCCAAACCTTTAT

TATGCCATTATATGATGCCAGATGAAGAAACTCCATTAGCAGTGCAGGCCTGTGGACTTTCTCCTCGAGA

CATTACCACTATTAAACTTCTCAATGAAACTAGAGACATGTTGGAAAGCCCAGATTTTAGTACAGTTTTG

AATACCTGTTTAAACCGAGGTTTTAGTAGACTTCTAGACAATATGGCTGAGTTCTTTCGACCTACTGAAC

AGGACCTGCAACATGGTAACTCTATGAATAGTCTTTCCAGTGTCAGCCTGCCTTTAGCTAAGATAATTCC

AATAGTAAACGGACAGATCCATTCAGTTTGCAGTGAAACACCTAGTCATTTTGTTCAGGATCTGTTGACA

ATGGAGCAAGTGAAAGACTTTGCTGCTAATGTGTATGAAGCTTTTAGTACCCCTCAGCAACTGGAGAAAT

GA

>lcl|NM_002647.4_cds_NP_002638.2_1 [gene=PIK3C3] [db_xref=CCDS:CCDS11920.1] [protein=phosphatidylinositol 3-kinase catalytic subunit type 3 isoform 1] [protein_id=NP_002638.2] [location=59..2722] [gbkey=CDS]

ATGGGGGAAGCAGAGAAGTTTCACTACATCTATAGTTGTGACCTGGATATCAACGTCCAGCTTAAGATAG

GAAGCTTGGAAGGGAAGAGAGAACAAAAGAGTTATAAAGCTGTCCTGGAAGACCCAATGTTGAAGTTCTC

AGGACTATATCAAGAGACATGCTCTGATCTTTATGTTACTTGTCAAGTTTTTGCAGAAGGGAAGCCTTTG

GCCTTGCCAGTGAGAACATCCTACAAAGCATTTAGTACAAGATGGAACTGGAATGAATGGCTGAAACTAC

CAGTAAAATACCCTGACCTGCCCAGGAATGCCCAAGTGGCCCTCACCATATGGGATGTGTATGGTCCCGG

AAAAGCAGTGCCTGTAGGAGGAACAACGGTTTCGCTCTTTGGAAAATACGGCATGTTTCGCCAAGGGATG

CATGACTTGAAAGTCTGGCCTAATGTAGAAGCAGATGGATCAGAACCCACAAAAACTCCTGGCAGAACAA

GTAGCACTCTCTCAGAAGATCAGATGAGCCGTCTTGCCAAGCTCACCAAAGCTCATCGACAAGGACACAT

GGTGAAAGTAGATTGGCTGGATAGATTGACATTTAGAGAAATAGAAATGATAAATGAGAGTGAAAAACGA

AGTTCTAATTTCATGTACCTGATGGTTGAATTTCGATGTGTCAAGTGTGATGATAAGGAATATGGTATTG

TTTATTATGAAAAGGACGGTGATGAATCATCTCCAATTTTAACAAGTTTTGAATTAGTGAAAGTTCCTGA

CCCCCAGATGTCTATGGAGAATTTAGTTGAGAGCAAACACCACAAGCTTGCCCGGAGTTTAAGAAGTGGA

CCTTCTGACCACGATCTGAAACCCAATGCTGCCACGAGAGATCAGTTAAATATTATTGTGAGTTATCCAC

CAACCAAGCAACTTACATATGAAGAACAAGATCTTGTTTGGAAGTTTAGATATTATCTTACGAATCAAGA

AAAAGCCTTGACAAAATTCTTGAAATGTGTTAATTGGGATCTACCTCAAGAGGCCAAACAGGCCTTGGAA

CTTCTGGGAAAATGGAAGCCGATGGATGTAGAGGACTCCTTGGAGCTGTTATCCTCTCATTACACCAACC

CAACTGTGAGGCGTTATGCTGTTGCCCGGTTGCGACAGGCCGATGATGAGGATTTGTTGATGTACCTATT

ACAATTGGTCCAGGCTCTCAAATATGAAAATTTTGATGATATAAAGAATGGATTGGAACCTACCAAGAAG

GATAGTCAGAGTTCAGTGTCAGAAAATGTGTCAAATTCTGGAATAAATTCTGCAGAAATAGATAGCTCCC

AAATTATAACCAGCCCCCTTCCTTCAGTCTCTTCACCTCCTCCTGCATCAAAAACAAAAGAAGTTCCAGA

TGGCGAAAATCTGGAACAAGATCTCTGTACCTTCTTGATATCGAGAGCCTGCAAAAACTCAACACTGGCT

AATTATTTATACTGGTATGTGATAGTGGAATGTGAAGATCAAGATACTCAGCAGAGAGATCCAAAGACCC

ATGAGATGTACTTGAACGTAATGAGAAGATTCAGCCAAGCATTGTTGAAGGGTGATAAGTCTGTCAGAGT

TATGCGTTCTTTGCTGGCTGCACAACAGACATTTGTAGATCGGTTGGTGCATCTAATGAAGGCAGTACAA

CGCGAAAGTGGAAATCGTAAGAAAAAGAATGAGAGACTACAGGCATTGCTTGGAGATAATGAAAAGATGA

ATTTGTCAGATGTGGAACTTATCCCGTTGCCTTTAGAACCCCAAGTGAAAATTAGAGGAATAATTCCGGA

AACAGCTACACTGTTTAAAAGTGCCCTTATGCCTGCACAGTTGTTTTTTAAGACGGAAGATGGAGGCAAA

TATCCAGTTATATTTAAGCATGGAGATGATTTACGTCAAGATCAACTTATTCTTCAAATCATTTCACTCA

TGGACAAGCTGTTACGGAAAGAAAATCTGGACTTGAAATTGACACCTTATAAGGTGTTAGCCACCAGTAC

AAAACATGGCTTCATGCAGTTTATCCAGTCAGTTCCTGTGGCTGAAGTTCTTGATACAGAGGGAAGCATT

CAGAACTTTTTTAGAAAATATGCACCAAGTGAGAATGGGCCAAATGGGATTAGTGCTGAGGTCATGGACA

CTTACGTTAAAAGCTGTGCTGGATATTGCGTGATCACCTATATACTTGGAGTTGGAGACAGGCACCTGGA

TAACCTTTTGCTAACAAAAACAGGCAAACTCTTCCACATAGACTTTGGATATATTTTGGGTCGGGATCCA

AAGCCTCTTCCTCCACCAATGAAGCTGAATAAAGAAATGGTAGAAGGAATGGGGGGCACACAGAGTGAGC

AGTACCAAGAGTTCCGTAAACAGTGTTACACGGCTTTCCTCCACCTGCGAAGGTATTCTAATCTGATTTT

GAACTTGTTTTCCTTGATGGTTGATGCAAACATTCCAGATATTGCACTTGAACCAGATAAAACTGTGAAA

AAGGTTCAGGATAAATTCCGCTTAGACCTGTCGGATGAAGAGGCTGTGCATTACATGCAGAGTCTGATTG

ATGAGAGTGTCCATGCTCTTTTTGCTGCAGTGGTGGAACAGATTCACAAGTTTGCCCAGTACTGGAGAAA

ATGA

>lcl|NM_014602.3_cds_NP_055417.1_1 [gene=PIK3R4] [db_xref=CCDS:CCDS3067.1] [protein=phosphoinositide 3-kinase regulatory subunit 4] [protein_id=NP_055417.1] [location=559..4635] [gbkey=CDS]

ATGGGAAATCAGCTTGCTGGCATTGCTCCCTCCCAGATCCTTTCTGTAGAGAGTTATTTTTCAGATATTC

ATGACTTTGAATATGATAAAAGCCTGGGGAGTACTCGGTTTTTTAAAGTTGCTCGAGCCAAGCACCGAGA

AGGCCTGGTCGTTGTGAAGGTTTTTGCAATTCAGGATCCCACATTGCCTTTAACCAGCTATAAACAAGAG

CTGGAGGAACTGAAAATCAGGCTTAATTCTGCACAGAATTGTCTACCTTTCCAGAAAGCATCAGAAAAAG

CATCTGAGAAAGCAGCTATGCTCTTTAGGCAGTATGTGCGAGACAATCTCTATGATCGCATCAGTACCCG

TCCATTCTTGAATAACATTGAGAAGCGCTGGATTGCTTTCCAGATCCTGACAGCTGTGGACCAAGCACAC

AAATCTGGAGTTCGTCATGGGGACATCAAGACTGAGAATGTGATGGTCACCAGTTGGAATTGGGTTCTTC

TAACTGATTTTGCCAGTTTTAAGCCCACTTATCTTCCAGAAGACAACCCGGCAGATTTCAATTATTTCTT

TGACACATCACGGAGGAGAACTTGCTATATTGCTCCTGAACGTTTTGTTGATGGTGGGATGTTTGCCACT

GAGTTAGAATATATGAGAGATCCTTCAACTCCGCTTGTAGACTTAAATAGCAATCAGAGAACAAGAGGAG

AGTTGAAGAGAGCAATGGACATCTTTTCAGCAGGTTGTGTGATAGCTGAGCTTTTTACAGAAGGTGTACC

ATTATTTGATCTCTCTCAACTTTTGGCTTATAGAAATGGACATTTTTTCCCTGAACAAGTGCTAAATAAA

ATTGAAGATCACAGTATCAGAGAATTGGTAACTCAGATGATTCACCGTGAGCCAGATAAACGTTTAGAGG

CAGAAGATTACTTAAAACAGCAGCGTGGCAATGCCTTTCCTGAAATATTTTACACTTTTCTTCAGCCCTA

CATGGCCCAGTTTGCCAAGGAAACGTTTCTTTCTGCAGATGAGCGTATTCTGGTTATACGGAAGGATTTG

GGCAACATTATTCACAATCTCTGTGGACATGATCTGCCAGAAAAAGCCGAAGGAGAGCCTAAGGAAAATG

GGCTGGTTATCTTGGTATCTGTTATAACATCCTGCCTACAGACCCTTAAATACTGTGATTCCAAACTAGC

TGCTTTGGAACTGATTCTTCATTTGGCTCCAAGATTAAGTGTTGAAATCCTTTTGGATCGTATTACTCCA

TATCTTTTGCATTTCAGCAATGACTCTGTTCCTAGGGTGAGGGCTGAAGCCTTGAGGACGTTGACCAAAG

TTCTTGCTCTCGTCAAAGAGGTTCCTCGTAATGATATCAATATTTATCCGGAATACATTCTGCCAGGCAT

AGCCCACTTAGCCCAAGATGATGCTACTATCGTTAGACTAGCCTATGCTGAAAACATAGCTCTGCTGGCA

GAAACAGCTCTGAGATTCCTGGAATTAGTACAGTTAAAAAATCTTAATATGGAAAATGACCCCAATAATG

AAGAAATAGATGAGGTTACACATCCAAATGGAAATTATGACACAGAGCTCCAAGCCTTACATGAAATGGT

CCAGCAGAAAGTTGTTACTTTGCTAAGTGACCCTGAAAATATTGTAAAACAAACCTTGATGGAAAATGGA

ATAACACGGCTGTGTGTATTCTTTGGACGTCAGAAAGCCAACGATGTTTTGTTGTCCCACATGATTACTT

TCCTAAATGATAAGAATGATTGGCATCTACGTGGAGCATTTTTTGATAGTATAGTTGGTGTTGCTGCCTA

TGTTGGCTGGCAAAGCTCCTCAATTCTCAAGCCTCTGCTGCAACAAGGTCTTAGTGATGCTGAGGAATTT

GTCATTGTGAAAGCTCTTTATGCCCTTACTTGTATGTGCCAGTTAGGACTGCTACAAAAACCCCATGTTT

ACGAATTTGCCAGTGATATTGCCCCCTTCCTGTGTCATCCCAATTTATGGATACGTTATGGTGCCGTGGG

ATTTATCACAGTGGTAGCTCGTCAAATAAGTACAGCTGATGTCTACTGTAAACTGATGCCTTATCTTGAC

CCATATATTACCCAACCAATAATACAGATTGAAAGAAAACTTGTTCTGCTCAGTGTTTTAAAGGAACCAG

TAAGTCGTTCTATATTTGATTATGCTTTGAGGTCTAAAGATATTACTAGCTTGTTCAGACATCTTCACAT

GCGTCAGAAGAAACGAAATGGTTCTCTTCCCGACTGCCCTCCGCCAGAGGATCCTGCCATAGCACAGCTT

CTGAAGAAGTTGCTCTCACAGGGAATGACAGAGGAAGAGGAAGACAAACTTCTGGCACTGAAAGACTTCA

TGATGAAATCTAATAAAGCAAAGGCCAATATAGTGGACCAGAGCCATCTTCATGATAGTAGTCAGAAAGG

TGTAATTGACTTGGCAGCTTTAGGCATAACTGGGAGACAAGTTGATCTTGTTAAAACCAAACAAGAACCA

GATGACAAACGGGCCAGAAAACATGTAAAACAAGACTCAAATGTAAATGAAGAATGGAAAAGCATGTTTG

GGTCACTGGACCCACCAAACATGCCACAGGCCCTACCTAAAGGGAGTGATCAGGAGGTGATTCAGACTGG

GAAACCTCCTCGTTCCGAGTCCTCTGCTGGCATTTGTGTCCCTTTGTCAACTTCTTCACAGGTTCCAGAA

GTGACAACTGTCCAAAATAAAAAACCAGTAATACCGGTTTTAAGTAGTACAATCTTACCATCCACCTATC

AGATTCGAATTACAACTTGTAAAACTGAACTTCAGCAACTCATCCAGCAAAAGCGGGAGCAGTGCAATGC

TGAGAGAATAGCTAAGCAGATGATGGAAAATGCTGAATGGGAGAGTAAACCACCACCACCTGGATGGCGT

CCTAAAGGGCTGTTAGTTGCCCATCTTCATGAGCATAAATCTGCTGTGAATCGAATTAGAGTCTCTGATG

AACACTCACTTTTTGCAACATGTTCAAATGATGGCACAGTGAAAATCTGGAACAGTCAAAAGATGGAGGG

GAAGACCACCACTACCAGATCTATTCTTACATACAGCCGAATTGGAGGACGAGTCAAGACGCTCACATTC

TGCCAAGGCTCCCACTATTTAGCCATAGCATCTGATAATGGTGCTGTCCAGCTTCTTGGAATTGAGGCTT

CTAAGCTGCCCAAGTCTCCTAAAATCCATCCTCTACAAAGCAGAATTCTAGATCAGAAGGAGGACGGTTG

TGTTGTGGATATGCATCACTTCAACTCTGGAGCACAGTCTGTTCTTGCCTATGCCACTGTGAATGGCTCT

CTGGTTGGCTGGGACCTTAGGTCTTCAAGCAATGCGTGGACTTTAAAGCATGATTTAAAGTCGGGCCTCA

TCACTTCCTTTGCTGTGGACATCCACCAATGCTGGCTCTGCATTGGTACAAGCAGTGGTACCATGGCTTG

TTGGGACATGAGGTTCCAGTTGCCAATTTCAAGTCACTGTCATCCTTCCAGGGCTCGAATCAGACGCCTC

TCAATGCACCCTCTGTATCAGTCCTGGGTGATTGCAGCTGTTCAGGGCAACAACGAAGTGTCCATGTGGG

ACATGGAGACTGGTGACAGAAGATTTACTCTCTGGGCCAGCAGTGCACCACCACTTTCTGAATTACAGCC

TTCTCCTCATAGCGTCCATGGTATCTACTGTAGTCCTGCAGATGGAAATCCTATCCTACTAACAGCTGGC

TCAGATATGAAAATAAGGTTTTGGGACTTGGCTTACCCAGAAAGGTCCTATGTTGTTGCAGGAAGTACTA

GTTCCCCATCTGTGTCCTACTACAGGAAAATAATTGAAGGCACTGAAGTTGTCCAGGAAATTCAGAATAA

GCAGAAAGTAGGACCAAGTGATGACACCCCTCGAAGGGGCCCAGAGTCCCTGCCCGTGGGACATCATGAC

ATCATCACTGATGTCGCCACATTCCAGACCACACAGGGCTTCATCGTAACTGCTTCTAGAGATGGGATTG

TGAAGGTGTGGAAATAA

>lcl|NM_032409.3_cds_NP_115785.1_1 [gene=PINK1] [db_xref=CCDS:CCDS211.1] [protein=serine/threonine-protein kinase PINK1, mitochondrial precursor] [protein_id=NP_115785.1] [location=92..1837] [gbkey=CDS]

ATGGCGGTGCGACAGGCGCTGGGCCGCGGCCTGCAGCTGGGTCGAGCGCTGCTGCTGCGCTTCACGGGCA

AGCCCGGCCGGGCCTACGGCTTGGGGCGGCCGGGCCCGGCGGCGGGCTGTGTCCGCGGGGAGCGTCCAGG

CTGGGCCGCAGGACCGGGCGCGGAGCCTCGCAGGGTCGGGCTCGGGCTCCCTAACCGTCTCCGCTTCTTC

CGCCAGTCGGTGGCCGGGCTGGCGGCGCGGTTGCAGCGGCAGTTCGTGGTGCGGGCCTGGGGCTGCGCGG

GCCCTTGCGGCCGGGCAGTCTTTCTGGCCTTCGGGCTAGGGCTGGGCCTCATCGAGGAAAAACAGGCGGA

GAGCCGGCGGGCGGTCTCGGCCTGTCAGGAGATCCAGGCAATTTTTACCCAGAAAAGCAAGCCGGGGCCT

GACCCGTTGGACACGAGACGCTTGCAGGGCTTTCGGCTGGAGGAGTATCTGATAGGGCAGTCCATTGGTA

AGGGCTGCAGTGCTGCTGTGTATGAAGCCACCATGCCTACATTGCCCCAGAACCTGGAGGTGACAAAGAG

CACCGGGTTGCTTCCAGGGAGAGGCCCAGGTACCAGTGCACCAGGAGAAGGGCAGGAGCGAGCTCCGGGG

GCCCCTGCCTTCCCCTTGGCCATCAAGATGATGTGGAACATCTCGGCAGGTTCCTCCAGCGAAGCCATCT

TGAACACAATGAGCCAGGAGCTGGTCCCAGCGAGCCGAGTGGCCTTGGCTGGGGAGTATGGAGCAGTCAC

TTACAGAAAATCCAAGAGAGGTCCCAAGCAACTAGCCCCTCACCCCAACATCATCCGGGTTCTCCGCGCC

TTCACCTCTTCCGTGCCGCTGCTGCCAGGGGCCCTGGTCGACTACCCTGATGTGCTGCCCTCACGCCTCC

ACCCTGAAGGCCTGGGCCATGGCCGGACGCTGTTCCTCGTTATGAAGAACTATCCCTGTACCCTGCGCCA

GTACCTTTGTGTGAACACACCCAGCCCCCGCCTCGCCGCCATGATGCTGCTGCAGCTGCTGGAAGGCGTG

GACCATCTGGTTCAACAGGGCATCGCGCACAGAGACCTGAAATCCGACAACATCCTTGTGGAGCTGGACC

CAGACGGCTGCCCCTGGCTGGTGATCGCAGATTTTGGCTGCTGCCTGGCTGATGAGAGCATCGGCCTGCA

GTTGCCCTTCAGCAGCTGGTACGTGGATCGGGGCGGAAACGGCTGTCTGATGGCCCCAGAGGTGTCCACG

GCCCGTCCTGGCCCCAGGGCAGTGATTGACTACAGCAAGGCTGATGCCTGGGCAGTGGGAGCCATCGCCT

ATGAAATCTTCGGGCTTGTCAATCCCTTCTACGGCCAGGGCAAGGCCCACCTTGAAAGCCGCAGCTACCA

AGAGGCTCAGCTACCTGCACTGCCCGAGTCAGTGCCTCCAGACGTGAGACAGTTGGTGAGGGCACTGCTC

CAGCGAGAGGCCAGCAAGAGACCATCTGCCCGAGTAGCCGCAAATGTGCTTCATCTAAGCCTCTGGGGTG

AACATATTCTAGCCCTGAAGAATCTGAAGTTAGACAAGATGGTTGGCTGGCTCCTCCAACAATCGGCCGC

CACTTTGTTGGCCAACAGGCTCACAGAGAAGTGTTGTGTGGAAACAAAAATGAAGATGCTCTTTCTGGCT

AACCTGGAGTGTGAAACGCTCTGCCAGGCAGCCCTCCTCCTCTGCTCATGGAGGGCAGCCCTGTGA

>lcl|NM_014330.5_cds_NP_055145.3_1 [gene=PPP1R15A] [db_xref=CCDS:CCDS12738.1] [protein=protein phosphatase 1 regulatory subunit 15A] [protein_id=NP_055145.3] [location=241..2265] [gbkey=CDS]

ATGGCCCCAGGCCAAGCACCCCATCAGGCTACCCCGTGGAGGGATGCCCACCCTTTCTTCCTCCTGTCCC

CAGTGATGGGCCTCCTCAGCCGCGCCTGGAGCCGCCTGAGGGGCCTGGGACCTCTAGAGCCCTGGCTGGT

GGAAGCAGTAAAAGGAGCAGCTCTGGTAGAAGCTGGCCTGGAGGGAGAAGCTAGGACTCCTCTGGCAATC

CCCCATACCCCTTGGGGCAGACGCCCTGAAGAGGAGGCTGAAGACAGTGGAGGCCCTGGAGAGGACAGAG

AAACACTGGGGCTGAAAACCAGCAGTTCCCTTCCTGAAGCCTGGGGACTTTTGGATGATGATGATGGCAT

GTATGGTGAGCGAGAGGCAACCAGTGTCCCTAGAGGGCAGGGAAGTCAATTTGCAGATGGCCAGCGTGCT

CCCCTGTCTCCCAGCCTTCTGATAAGGACACTGCAAGGTTCTGATAAGAACCCAGGGGAGGAGAAAGCCG

AGGAAGAGGGAGTTGCTGAAGAGGAGGGAGTTAACAAGTTCTCTTATCCACCATCACACCGGGAGTGTTG

TCCAGCCGTGGAGGAGGAGGACGATGAAGAAGCTGTAAAGAAAGAAGCTCACAGAACCTCTACTTCTGCC

TTGTCTCCAGGATCCAAGCCCAGCACTTGGGTGTCTTGCCCAGGGGAGGAAGAGAATCAAGCCACGGAGG

ATAAAAGAACAGAAAGAAGTAAAGGAGCCAGGAAGACCTCCGTGTCCCCCCGATCTTCAGGCTCCGACCC

CAGGTCCTGGGAGTATCGTTCAGGAGAGGCGTCCGAGGAGAAGGAGGAAAAGGCACACAAAGAAACTGGG

AAAGGAGAAGCTGCCCCAGGGCCGCAATCCTCAGCCCCAGCCCAGAGGCCCCAGCTCAAGTCCTGGTGGT

GCCAACCCAGTGATGAAGAGGAGGGTGAGGTCAAGGCTTTGGGGGCAGCTGAGAAGGATGGAGAAGCTGA

GTGTCCTCCCTGCATCCCCCCACCAAGTGCCTTCCTGAAGGCCTGGGTGTATTGGCCAGGAGAGGACACA

GAGGAAGAGGAAGATGAGGAAGAAGATGAGGACAGTGACTCTGGATCAGATGAGGAAGAGGGAGAAGCTG

AGGCTTCCTCTTCCACTCCTGCTACAGGTGTCTTCTTGAAGTCCTGGGTCTATCAGCCAGGAGAGGACAC

AGAGGAGGAGGAAGATGAGGACAGTGATACAGGATCAGCCGAGGATGAAAGAGAAGCTGAGACTTCTGCT

TCCACACCCCCTGCAAGTGCTTTCTTGAAGGCCTGGGTGTATCGGCCAGGAGAGGACACGGAGGAGGAGG

AAGATGAGGATGTGGATAGTGAGGATAAGGAAGATGATTCAGAAGCAGCCTTGGGAGAAGCTGAGTCAGA

CCCACATCCCTCCCACCCGGACCAGAGGGCCCACTTCAGGGGCTGGGGATATCGACCTGGAAAAGAGACA

GAGGAAGAGGAAGCTGCTGAGGACTGGGGAGAAGCTGAGCCCTGCCCCTTCCGAGTGGCCATCTATGTAC

CTGGAGAGAAGCCACCGCCTCCCTGGGCTCCTCCTAGGCTGCCCCTCCGACTGCAAAGGCGGCTCAAGCG

CCCAGAAACCCCTACTCATGATCCGGACCCTGAGACTCCCCTAAAGGCCAGAAAGGTGCGCTTCTCCGAG

AAGGTCACTGTCCATTTCCTGGCTGTCTGGGCAGGGCCGGCCCAGGCCGCCCGCCAGGGCCCCTGGGAGC

AGCTTGCTCGGGATCGCAGCCGCTTCGCACGCCGCATCACCCAGGCCCAGGAGGAGCTGAGCCCCTGCCT

CACCCCTGCTGCCCGGGCCAGAGCCTGGGCACGCCTCAGGAACCCACCTTTAGCCCCCATCCCTGCCCTC

ACCCAGACCTTGCCTTCCTCCTCTGTCCCTTCGTCCCCAGTCCAGACCACGCCCTTGAGCCAAGCTGTGG

CCACACCTTCCCGCTCGTCTGCTGCTGCAGCGGCTGCCCTGGACCTCAGTGGGAGGCGTGGCTGA

>lcl|NM_006253.5_cds_NP_006244.2_1 [gene=PRKAB1] [db_xref=CCDS:CCDS9191.1] [protein=5'-AMP-activated protein kinase subunit beta-1] [protein_id=NP_006244.2] [location=113..925] [gbkey=CDS]

ATGGGCAATACCAGCAGTGAGCGCGCCGCGCTGGAGCGGCATGGTGGCCATAAGACGCCCCGGAGGGACA

GCTCGGGGGGCACCAAGGACGGGGACAGGCCCAAGATCCTGATGGACAGCCCCGAAGACGCCGACCTCTT

CCACTCCGAGGAAATCAAGGCACCAGAGAAGGAGGAATTCCTGGCCTGGCAGCATGATCTGGAAGTGAAT

GATAAAGCTCCCGCCCAGGCTCGGCCAACGGTGTTTCGATGGACGGGGGGCGGAAAGGAAGTTTACTTAT

CTGGGTCCTTCAACAACTGGAGTAAACTTCCCCTCACCAGAAGCCACAATAACTTTGTAGCCATCCTGGA

TCTGCCGGAAGGAGAGCATCAGTACAAGTTCTTTGTGGATGGTCAGTGGACGCACGACCCTTCCGAGCCC

ATAGTAACCAGCCAGCTTGGCACAGTTAACAACATCATTCAAGTGAAGAAAACTGACTTTGAAGTATTTG

ATGCTTTAATGGTGGATTCCCAAAAGTGCTCCGATGTGTCTGAGCTGTCCAGTTCTCCCCCAGGACCCTA

CCATCAGGAGCCCTACGTCTGCAAACCCGAAGAGCGCTTTCGGGCACCCCCTATTCTCCCCCCACATCTC

CTCCAGGTCATCCTGAACAAGGACACGGGGATTTCCTGTGATCCAGCTTTGCTTCCTGAGCCCAATCACG

TCATGCTGAACCACCTATACGCGCTGTCTATCAAGGATGGAGTGATGGTGCTCAGCGCAACCCACCGGTA

CAAGAAGAAGTACGTCACCACCTTGTTATACAAGCCCATATGA

>lcl|NM_001276289.2_cds_NP_001263218.1_1 [gene=PRKAR1A] [db_xref=CCDS:CCDS11678.1] [protein=cAMP-dependent protein kinase type I-alpha regulatory subunit isoform a] [protein_id=NP_001263218.1] [location=259..1404] [gbkey=CDS]

ATGGAGTCTGGCAGTACCGCCGCCAGTGAGGAGGCACGCAGCCTTCGAGAATGTGAGCTCTACGTCCAGA

AGCATAACATTCAAGCGCTGCTCAAAGATTCTATTGTGCAGTTGTGCACTGCTCGACCTGAGAGACCCAT

GGCATTCCTCAGGGAATACTTTGAGAGGTTGGAGAAGGAGGAGGCAAAACAGATTCAGAATCTGCAGAAA

GCAGGCACTCGTACAGACTCAAGGGAGGATGAGATTTCTCCTCCTCCACCCAACCCAGTGGTTAAAGGTA

GGAGGCGACGAGGTGCTATCAGCGCTGAGGTCTACACGGAGGAAGATGCGGCATCCTATGTTAGAAAGGT

TATACCAAAAGATTACAAGACAATGGCCGCTTTAGCCAAAGCCATTGAAAAGAATGTGCTGTTTTCACAT

CTTGATGATAATGAGAGAAGTGATATTTTTGATGCCATGTTTTCGGTCTCCTTTATCGCAGGAGAGACTG

TGATTCAGCAAGGTGATGAAGGGGATAACTTCTATGTGATTGATCAAGGAGAGACGGATGTCTATGTTAA

CAATGAATGGGCAACCAGTGTTGGGGAAGGAGGGAGCTTTGGAGAACTTGCTTTGATTTATGGAACACCG

AGAGCAGCCACTGTCAAAGCAAAGACAAATGTGAAATTGTGGGGCATCGACCGAGACAGCTATAGAAGAA

TCCTCATGGGAAGCACACTGAGAAAGCGGAAGATGTATGAGGAATTCCTTAGTAAAGTCTCTATTTTAGA

GTCTCTGGACAAGTGGGAACGTCTTACGGTAGCTGATGCATTGGAACCAGTGCAGTTTGAAGATGGGCAG

AAGATTGTGGTGCAGGGAGAACCAGGGGATGAGTTCTTCATTATTTTAGAGGGGTCAGCTGCTGTGCTAC

AACGTCGGTCAGAAAATGAAGAGTTTGTTGAAGTGGGAAGATTGGGGCCTTCTGATTATTTTGGTGAAAT

TGCACTACTGATGAATCGTCCTCGTGCTGCCACAGTTGTTGCTCGTGGCCCCTTGAAGTGCGTTAAGCTG

GACCGACCTAGATTTGAACGTGTTCTTGGCCCATGCTCAGACATCCTCAAACGAAACATCCAGCAGTACA

ACAGTTTTGTGTCACTGTCTGTCTGA

>lcl|NM_001316327.2_cds_NP_001303256.1_1 [gene=PRKCD] [db_xref=CCDS:CCDS2870.1] [protein=protein kinase C delta type isoform c] [protein_id=NP_001303256.1] [location=201..2231] [gbkey=CDS]

ATGGCGCCGTTCCTGCGCATCGCCTTCAACTCCTATGAGCTGGGCTCCCTGCAGGCCGAGGACGAGGCGA

ACCAGCCCTTCTGTGCCGTGAAGATGAAGGAGGCGCTCAGCACAGAGCGTGGGAAAACACTGGTGCAGAA

GAAGCCGACCATGTATCCTGAGTGGAAGTCGACGTTCGATGCCCACATCTATGAGGGGCGCGTCATCCAG

ATTGTGCTAATGCGGGCAGCAGAGGAGCCAGTGTCTGAGGTGACCGTGGGTGTGTCGGTGCTGGCCGAGC

GCTGCAAGAAGAACAATGGCAAGGCTGAGTTCTGGCTGGACCTGCAGCCTCAGGCCAAGGTGTTGATGTC

TGTTCAGTATTTCCTGGAGGACGTGGATTGCAAACAGTCTATGCGCAGTGAGGACGAGGCCAAGTTCCCA

ACGATGAACCGCCGCGGAGCCATCAAACAGGCCAAAATCCACTACATCAAGAACCATGAGTTTATCGCCA

CCTTCTTTGGGCAACCCACCTTCTGTTCTGTGTGCAAAGACTTTGTCTGGGGCCTCAACAAGCAAGGCTA

CAAATGCAGGCAATGTAACGCTGCCATCCACAAGAAATGCATCGACAAGATCATCGGCAGATGCACTGGC

ACCGCGGCCAACAGCCGGGACACTATATTCCAGAAAGAACGCTTCAACATCGACATGCCGCACCGCTTCA

AGGTTCACAACTACATGAGCCCCACCTTCTGTGACCACTGCGGCAGCCTGCTCTGGGGACTGGTGAAGCA

GGGATTAAAGTGTGAAGACTGCGGCATGAATGTGCACCATAAATGCCGGGAGAAGGTGGCCAACCTCTGC

GGCATCAACCAGAAGCTTTTGGCTGAGGCCTTGAACCAAGTCACCCAGAGAGCCTCCCGGAGATCAGACT

CAGCCTCCTCAGAGCCTGTTGGGATATATCAGGGTTTCGAGAAGAAGACCGGAGTTGCTGGGGAGGACAT

GCAAGACAACAGTGGGACCTACGGCAAGATCTGGGAGGGCAGCAGCAAGTGCAACATCAACAACTTCATC

TTCCACAAGGTCCTGGGCAAAGGCAGCTTCGGGAAGGTGCTGCTTGGAGAGCTGAAGGGCAGAGGAGAGT

ACTTTGCCATCAAGGCCCTCAAGAAGGATGTGGTCCTGATCGACGACGACGTGGAGTGCACCATGGTTGA

GAAGCGGGTGCTGACACTTGCCGCAGAGAATCCCTTTCTCACCCACCTCATCTGCACCTTCCAGACCAAG

GACCACCTGTTCTTTGTGATGGAGTTCCTCAACGGGGGGGACCTGATGTACCACATCCAGGACAAAGGCC

GCTTTGAACTCTACCGTGCCACGTTTTATGCCGCTGAGATAATGTGTGGACTGCAGTTTCTACACAGCAA

GGGCATCATTTACAGGGACCTCAAACTGGACAATGTGCTGCTGGACCGGGATGGCCACATCAAGATTGCC

GACTTTGGGATGTGCAAAGAGAACATATTCGGGGAGAGCCGGGCCAGCACCTTCTGCGGCACCCCTGACT

ATATCGCCCCTGAGATCCTACAGGGCCTGAAGTACACATTCTCTGTGGACTGGTGGTCTTTCGGGGTCCT

TCTGTACGAGATGCTCATTGGCCAGTCCCCCTTCCATGGTGATGATGAGGATGAACTCTTCGAGTCCATC

CGTGTGGACACGCCACATTATCCCCGCTGGATCACCAAGGAGTCCAAGGACATCCTGGAGAAGCTCTTTG

AAAGGGAACCAACCAAGAGGCTGGGAGTGACCGGAAACATCAAAATCCACCCCTTCTTCAAGACCATAAA

CTGGACTCTGCTGGAAAAGCGGAGGTTGGAGCCACCTTTCAGGCCCAAAGTGAAGTCACCCAGAGACTAC

AGTAACTTTGACCAGGAGTTCCTGAACGAGAAGGCGCGCCTCTCCTACAGCGACAAGAACCTCATCGACT

CCATGGACCAGTCTGCATTCGCTGGCTTCTCCTTTGTGAACCCCAAATTCGAGCACCTCCTGGAAGATTG

A

>lcl|NM_006257.5_cds_NP_006248.1_1 [gene=PRKCQ] [db_xref=CCDS:CCDS7079.1] [protein=protein kinase C theta type isoform 1] [protein_id=NP_006248.1] [location=76..2196] [gbkey=CDS]

ATGTCGCCATTTCTTCGGATTGGCTTGTCCAACTTTGACTGCGGGTCCTGCCAGTCTTGTCAGGGCGAGG

CTGTTAACCCTTACTGTGCTGTGCTCGTCAAAGAGTATGTCGAATCAGAGAACGGGCAGATGTATATCCA

GAAAAAGCCTACCATGTACCCACCCTGGGACAGCACTTTTGATGCCCATATCAACAAGGGAAGAGTCATG

CAGATCATTGTGAAAGGCAAAAACGTGGACCTCATCTCTGAAACCACCGTGGAGCTCTACTCGCTGGCTG

AGAGGTGCAGGAAGAACAACGGGAAGACAGAAATATGGTTAGAGCTGAAACCTCAAGGCCGAATGCTAAT

GAATGCAAGATACTTTCTGGAAATGAGTGACACAAAGGACATGAATGAATTTGAGACGGAAGGCTTCTTT

GCTTTGCATCAGCGCCGGGGTGCCATCAAGCAGGCAAAGGTCCACCACGTCAAGTGCCACGAGTTCACTG

CCACCTTCTTCCCACAGCCCACATTTTGCTCTGTCTGCCACGAGTTTGTCTGGGGCCTGAACAAACAGGG

CTACCAGTGCCGACAATGCAATGCAGCAATTCACAAGAAGTGTATTGATAAAGTTATAGCAAAGTGCACA

GGATCAGCTATCAATAGCCGAGAAACCATGTTCCACAAGGAGAGATTCAAAATTGACATGCCACACAGAT

TTAAAGTCTACAATTACAAGAGCCCGACCTTCTGTGAACACTGTGGGACCCTGCTGTGGGGACTGGCACG

GCAAGGACTCAAGTGTGATGCATGTGGCATGAATGTGCATCATAGATGCCAGACAAAGGTGGCCAACCTT

TGTGGCATAAACCAGAAGCTAATGGCTGAAGCGCTGGCCATGATTGAGAGCACTCAACAGGCTCGCTGCT

TAAGAGATACTGAACAGATCTTCAGAGAAGGTCCGGTTGAAATTGGTCTCCCATGCTCCATCAAAAATGA

AGCAAGGCCGCCATGTTTACCGACACCGGGAAAAAGAGAGCCTCAGGGCATTTCCTGGGAGTCTCCGTTG

GATGAGGTGGATAAAATGTGCCATCTTCCAGAACCTGAACTGAACAAAGAAAGACCATCTCTGCAGATTA

AACTAAAAATTGAGGATTTTATCTTGCACAAAATGTTGGGGAAAGGAAGTTTTGGCAAGGTCTTCCTGGC

AGAATTCAAGAAAACCAATCAATTTTTCGCAATAAAGGCCTTAAAGAAAGATGTGGTCTTGATGGACGAT

GATGTTGAGTGCACGATGGTAGAGAAGAGAGTTCTTTCCTTGGCCTGGGAGCATCCGTTTCTGACGCACA

TGTTTTGTACATTCCAGACCAAGGAAAACCTCTTTTTTGTGATGGAGTACCTCAACGGAGGGGACTTAAT

GTACCACATCCAAAGCTGCCACAAGTTCGACCTTTCCAGAGCGACGTTTTATGCTGCTGAAATCATTCTT

GGTCTGCAGTTCCTTCATTCCAAAGGAATAGTCTACAGGGACCTGAAGCTAGATAACATCCTGTTAGACA

AAGATGGACATATCAAGATCGCGGATTTTGGAATGTGCAAGGAGAACATGTTAGGAGATGCCAAGACGAA

TACCTTCTGTGGGACACCTGACTACATCGCCCCAGAGATCTTGCTGGGTCAGAAATACAACCACTCTGTG

GACTGGTGGTCCTTCGGGGTTCTCCTTTATGAAATGCTGATTGGTCAGTCGCCTTTCCACGGGCAGGATG

AGGAGGAGCTCTTCCACTCCATCCGCATGGACAATCCCTTTTACCCACGGTGGCTGGAGAAGGAAGCAAA

GGACCTTCTGGTGAAGCTCTTCGTGCGAGAACCTGAGAAGAGGCTGGGCGTGAGGGGAGACATCCGCCAG

CACCCTTTGTTTCGGGAGATCAACTGGGAGGAACTTGAACGGAAGGAGATTGACCCACCGTTCCGGCCGA

AAGTGAAATCACCATTTGACTGCAGCAATTTCGACAAAGAATTCTTAAACGAGAAGCCCCGGCTGTCATT

TGCCGACAGAGCACTGATCAACAGCATGGACCAGAATATGTTCAGGAACTTTTCCTTCATGAACCCCGGG

ATGGAGCGGCTGATATCCTGA

>lcl|NM_004562.3_cds_NP_004553.2_1 [gene=PRKN] [db_xref=CCDS:CCDS5281.1] [protein=E3 ubiquitin-protein ligase parkin isoform 1] [protein_id=NP_004553.2] [location=99..1496] [gbkey=CDS]

ATGATAGTGTTTGTCAGGTTCAACTCCAGCCATGGTTTCCCAGTGGAGGTCGATTCTGACACCAGCATCT

TCCAGCTCAAGGAGGTGGTTGCTAAGCGACAGGGGGTTCCGGCTGACCAGTTGCGTGTGATTTTCGCAGG

GAAGGAGCTGAGGAATGACTGGACTGTGCAGAATTGTGACCTGGATCAGCAGAGCATTGTTCACATTGTG

CAGAGACCGTGGAGAAAAGGTCAAGAAATGAATGCAACTGGAGGCGACGACCCCAGAAACGCGGCGGGAG

GCTGTGAGCGGGAGCCCCAGAGCTTGACTCGGGTGGACCTCAGCAGCTCAGTCCTCCCAGGAGACTCTGT

GGGGCTGGCTGTCATTCTGCACACTGACAGCAGGAAGGACTCACCACCAGCTGGAAGTCCAGCAGGTAGA

TCAATCTACAACAGCTTTTATGTGTATTGCAAAGGCCCCTGTCAAAGAGTGCAGCCGGGAAAACTCAGGG

TACAGTGCAGCACCTGCAGGCAGGCAACGCTCACCTTGACCCAGGGTCCATCTTGCTGGGATGATGTTTT

AATTCCAAACCGGATGAGTGGTGAATGCCAATCCCCACACTGCCCTGGGACTAGTGCAGAATTTTTCTTT

AAATGTGGAGCACACCCCACCTCTGACAAGGAAACATCAGTAGCTTTGCACCTGATCGCAACAAATAGTC

GGAACATCACTTGCATTACGTGCACAGACGTCAGGAGCCCCGTCCTGGTTTTCCAGTGCAACTCCCGCCA

CGTGATTTGCTTAGACTGTTTCCACTTATACTGTGTGACAAGACTCAATGATCGGCAGTTTGTTCACGAC

CCTCAACTTGGCTACTCCCTGCCTTGTGTGGCTGGCTGTCCCAACTCCTTGATTAAAGAGCTCCATCACT

TCAGGATTCTGGGAGAAGAGCAGTACAACCGGTACCAGCAGTATGGTGCAGAGGAGTGTGTCCTGCAGAT

GGGGGGCGTGTTATGCCCCCGCCCTGGCTGTGGAGCGGGGCTGCTGCCGGAGCCTGACCAGAGGAAAGTC

ACCTGCGAAGGGGGCAATGGCCTGGGCTGTGGGTTTGCCTTCTGCCGGGAATGTAAAGAAGCGTACCATG

AAGGGGAGTGCAGTGCCGTATTTGAAGCCTCAGGAACAACTACTCAGGCCTACAGAGTCGATGAAAGAGC

CGCCGAGCAGGCTCGTTGGGAAGCAGCCTCCAAAGAAACCATCAAGAAAACCACCAAGCCCTGTCCCCGC

TGCCATGTACCAGTGGAAAAAAATGGAGGCTGCATGCACATGAAGTGTCCGCAGCCCCAGTGCAGGCTCG

AGTGGTGCTGGAACTGTGGCTGCGAGTGGAACCGCGTCTGCATGGGGGACCACTGGTTCGACGTGTAG

>lcl|NM_000314.8_cds_NP_000305.3_1 [gene=PTEN] [db_xref=CCDS:CCDS31238.1] [protein=phosphatidylinositol 3,4,5-trisphosphate 3-phosphatase and dual-specificity protein phosphatase PTEN isoform PTEN] [protein_id=NP_000305.3] [location=846..2057] [gbkey=CDS]

ATGACAGCCATCATCAAAGAGATCGTTAGCAGAAACAAAAGGAGATATCAAGAGGATGGATTCGACTTAG

ACTTGACCTATATTTATCCAAACATTATTGCTATGGGATTTCCTGCAGAAAGACTTGAAGGCGTATACAG

GAACAATATTGATGATGTAGTAAGGTTTTTGGATTCAAAGCATAAAAACCATTACAAGATATACAATCTT

TGTGCTGAAAGACATTATGACACCGCCAAATTTAATTGCAGAGTTGCACAATATCCTTTTGAAGACCATA

ACCCACCACAGCTAGAACTTATCAAACCCTTTTGTGAAGATCTTGACCAATGGCTAAGTGAAGATGACAA

TCATGTTGCAGCAATTCACTGTAAAGCTGGAAAGGGACGAACTGGTGTAATGATATGTGCATATTTATTA

CATCGGGGCAAATTTTTAAAGGCACAAGAGGCCCTAGATTTCTATGGGGAAGTAAGGACCAGAGACAAAA

AGGGAGTAACTATTCCCAGTCAGAGGCGCTATGTGTATTATTATAGCTACCTGTTAAAGAATCATCTGGA

TTATAGACCAGTGGCACTGTTGTTTCACAAGATGATGTTTGAAACTATTCCAATGTTCAGTGGCGGAACT

TGCAATCCTCAGTTTGTGGTCTGCCAGCTAAAGGTGAAGATATATTCCTCCAATTCAGGACCCACACGAC

GGGAAGACAAGTTCATGTACTTTGAGTTCCCTCAGCCGTTACCTGTGTGTGGTGATATCAAAGTAGAGTT

CTTCCACAAACAGAACAAGATGCTAAAAAAGGACAAAATGTTTCACTTTTGGGTAAATACATTCTTCATA

CCAGGACCAGAGGAAACCTCAGAAAAAGTAGAAAATGGAAGTCTATGTGATCAAGAAATCGATAGCATTT

GCAGTATAGAGCGTGCAGATAATGACAAGGAATATCTAGTACTTACTTTAACAAAAAATGATCTTGACAA

AGCAAATAAAGACAAAGCCAACCGATACTTTTCTCCAAATTTTAAGGTGAAGCTGTACTTCACAAAAACA

GTAGAGGAGCCGTCAAATCCAGAGGCTAGCAGTTCAACTTCTGTAACACCAGATGTTAGTGACAATGAAC

CTGATCATTATAGATATTCTGACACCACTGACTCTGATCCAGAGAATGAACCTTTTGATGAAGATCAGCA

TACACAAATTACAAAAGTCTGA

>lcl|NM_001256358.2_cds_NP_001243287.1_1 [gene=PTK6] [db_xref=CCDS:CCDS74750.1] [protein=protein-tyrosine kinase 6 isoform 2] [protein_id=NP_001243287.1] [location=63..467] [gbkey=CDS]

ATGGTGTCCCGGGACCAGGCTCACCTGGGCCCCAAGTATGTGGGCCTCTGGGACTTCAAGTCCCGGACGG

ACGAGGAGCTGAGCTTCCGCGCGGGGGACGTCTTCCACGTGGCCAGGAAGGAGGAGCAGTGGTGGTGGGC

CACGCTGCTGGACGAGGCGGGTGGGGCCGTGGCCCAGGGCTATGTGCCCCACAACTACCTGGCCGAGAGG

GAGACGGTGGAGTCGGAACCTGCGGGACACGCAGGCTGTGCGGCACTACAAGATCTGGCGGCGTGCCGGG

GGCCGGCTGCACCTGAACGAGGCGGTGTCCTTCCTCAGCCTGCCCGAGCTTGTGAACTACCACAGGGCCC

AGAGCCTGTCCCACGGCCTGCGGCTGGCCGCGCCCTGCCGGAAGCACGAGCCTGA

>lcl|NM_001206836.2_cds_NP_001193765.1_1 [gene=RAB11A] [db_xref=CCDS:CCDS58373.1] [protein=ras-related protein Rab-11A isoform 2] [protein_id=NP_001193765.1] [location=96..563] [gbkey=CDS]

ATGGGCACCCGCGACGACGAGTACGACTACCTCTTTAAAGTTGTCCTTATTGGAGATTCTGGTGTTGGAA

AGAGTAATCTCCTGTCTCGATTTACTCGAAATGAGTTTAATCTGGAAAGCAAGAGCACCATTGGAGTAGA

GTTTGCAACAAGAAGCATCCAGGTTGATGGAAAAACAATAAAGGCACAGATATGGGACACAGCAGGGCAA

GAGCGATATCGAGCTATAACATCAGCATATTATCGTGGAGCTGTAGGTGCCTTATTGGTTTATGACATTG

CTAAACATCTCACATATGAAAATGTAGAGCGATGGCTGAAAGAACTGAGAGATCATGCTGATAGTAACAT

TGTTATCATGCTTGTGGGCAATAAGAGTGATCTACGTCATCTCAGGGCAGTTCCTACAGATGAAGCAAGA

GCTTTTGCAGAAAAGAATGAAGCAAATGTCAGACAGACGCGAAAATGA

>lcl|NM_004161.5_cds_NP_004152.1_1 [gene=RAB1A] [db_xref=CCDS:CCDS46306.1] [protein=ras-related protein Rab-1A isoform 1] [protein_id=NP_004152.1] [location=192..809] [gbkey=CDS]

ATGTCCAGCATGAATCCCGAATATGATTATTTATTCAAGTTACTTCTGATTGGCGACTCAGGGGTTGGAA

AGTCTTGCCTTCTTCTTAGGTTTGCAGATGATACATATACAGAAAGCTACATCAGCACAATTGGTGTGGA

TTTCAAAATAAGAACTATAGAGTTAGACGGGAAAACAATCAAGCTTCAAATATGGGACACAGCAGGCCAG

GAAAGATTTCGAACAATCACCTCCAGTTATTACAGAGGAGCCCATGGCATCATAGTTGTGTATGATGTGA

CAGATCAGGAGTCCTTCAATAATGTTAAACAGTGGCTGCAGGAAATAGATCGTTATGCCAGTGAAAATGT

CAACAAATTGTTGGTAGGGAACAAATGTGATCTGACCACAAAGAAAGTAGTAGACTACACAACAGCGAAG

GAATTTGCTGATTCCCTTGGAATTCCGTTTTTGGAAACCAGTGCTAAGAATGCAACGAATGTAGAACAGT

CTTTCATGACGATGGCAGCTGAGATTAAAAAGCGAATGGGTCCCGGAGCAACAGCTGGTGGTGCTGAGAA

GTCCAATGTTAAAATTCAGAGCACTCCAGTCAAGCAGTCAGGTGGAGGTTGCTGCTAA

>lcl|NM_130781.4_cds_NP_570137.2_1 [gene=RAB24] [db_xref=CCDS:CCDS34300.1] [protein=ras-related protein Rab-24] [protein_id=NP_570137.2] [location=346..957] [gbkey=CDS]

ATGAGCGGGCAGCGCGTGGACGTCAAGGTGGTGATGCTGGGCAAGGAGTACGTGGGCAAGACTAGCCTGG

TGGAGCGCTACGTGCACGACCGCTTTCTGGTGGGGCCTTATCAGAACACCATCGGGGCCGCCTTCGTGGC

CAAGGTGATGTCGGTCGGAGACCGGACTGTGACATTAGGTATTTGGGACACAGCAGGCTCTGAGCGCTAT

GAGGCCATGAGTAGAATCTACTATCGGGGTGCCAAGGCTGCCATCGTCTGCTATGACCTCACAGACAGCA

GCAGCTTTGAGCGAGCAAAGTTCTGGGTGAAGGAACTGCGCAGCCTAGAGGAGGGCTGCCAAATCTACTT

ATGTGGCACCAAGAGTGACCTGCTGGAAGAAGACCGGAGGCGTCGACGTGTGGACTTCCACGACGTCCAG

GACTATGCAGACAATATCAAAGCTCAGCTCTTTGAAACATCCAGCAAGACAGGCCAGAGTGTGGACGAGC

TCTTCCAGAAAGTGGCAGAGGATTACGTCAGTGTGGCTGCCTTCCAGGTGATGACAGAGGACAAGGGCGT

GGATCTGGGCCAGAAGCCAAACCCCTACTTCTACAGCTGTTGTCATCACTGA

>lcl|NM_031296.3_cds_NP_112586.1_1 [gene=RAB33B] [db_xref=CCDS:CCDS3747.1] [protein=ras-related protein Rab-33B] [protein_id=NP_112586.1] [location=76..765] [gbkey=CDS]

ATGGCTGAGGAGATGGAGTCGTCGCTCGAGGCAAGCTTTTCGTCCAGCGGGGCAGTGTCAGGGGCCTCAG

GGTTTTTGCCTCCTGCCCGCTCCCGCATCTTCAAGATAATCGTGATCGGCGACTCCAATGTGGGCAAGAC

ATGCCTGACCTACCGCTTCTGCGCTGGCCGCTTCCCCGACCGCACCGAGGCCACGATAGGGGTGGATTTC

CGAGAACGAGCGGTGGAGATTGATGGGGAGCGCATCAAGATCCAGCTATGGGACACAGCAGGACAAGAAC

GATTCAGAAAGAGCATGGTTCAGCACTACTACAGAAATGTACATGCTGTTGTCTTCGTGTATGATATGAC

CAACATGGCTAGTTTTCATAGCCTACCATCTTGGATAGAAGAATGCAAACAACATTTGCTAGCCAATGAT

ATACCACGGATTCTTGTTGGAAATAAATGTGACTTGAGAAGTGCCATACAGGTACCCACAGACTTGGCAC

AAAAATTTGCTGACACACACAGTATGCCTTTGTTTGAAACGTCTGCTAAAAACCCCAATGATAATGACCA

TGTGGAAGCTATATTTATGACCTTGGCTCATAAGCTTAAGAGCCACAAACCATTAATGCTTAGTCAGCCC

CCTGATAATGGAATTATCCTGAAGCCTGAACCAAAGCCTGCAATGACGTGCTGGTGCTAA

>lcl|NM_004162.5_cds_NP_004153.2_1 [gene=RAB5A] [db_xref=CCDS:CCDS2633.1] [protein=ras-related protein Rab-5A isoform 1] [protein_id=NP_004153.2] [location=519..1166] [gbkey=CDS]

ATGGCTAGTCGAGGCGCAACAAGACCCAACGGGCCAAATACGGGAAATAAAATATGCCAGTTCAAACTAG

TACTTCTGGGAGAGTCCGCTGTTGGCAAATCAAGCCTAGTGCTTCGTTTTGTGAAAGGCCAATTTCATGA

ATTTCAAGAGAGTACCATTGGGGCTGCTTTTCTAACCCAAACTGTATGTCTTGATGACACTACAGTAAAG

TTTGAAATATGGGATACAGCTGGTCAAGAACGATACCATAGCCTAGCACCAATGTACTACAGAGGAGCAC

AAGCAGCCATAGTTGTATATGATATCACAAATGAGGAGTCCTTTGCAAGAGCAAAAAATTGGGTTAAAGA

ACTTCAGAGGCAAGCAAGTCCTAACATTGTAATAGCTTTATCGGGAAACAAGGCCGACCTAGCAAATAAA

AGAGCAGTAGATTTCCAGGAAGCACAGTCCTATGCAGATGACAATAGTTTATTATTCATGGAGACATCCG

CTAAAACATCAATGAATGTAAATGAAATATTCATGGCAATAGCTAAAAAATTGCCAAAGAATGAACCACA

AAATCCAGGAGCAAATTCTGCCAGAGGAAGAGGAGTAGACCTTACCGAACCCACACAACCAACCAGGAAT

CAGTGTTGTAGTAACTAA

>lcl|NM_004637.6_cds_NP_004628.4_1 [gene=RAB7A] [db_xref=CCDS:CCDS3052.1] [protein=ras-related protein Rab-7a] [protein_id=NP_004628.4] [location=186..809] [gbkey=CDS]

ATGACCTCTAGGAAGAAAGTGTTGCTGAAGGTTATCATCCTGGGAGATTCTGGAGTCGGGAAGACATCAC

TCATGAACCAGTATGTGAATAAGAAATTCAGCAATCAGTACAAAGCCACAATAGGAGCTGACTTTCTGAC

CAAGGAGGTGATGGTGGATGACAGGCTAGTCACAATGCAGATATGGGACACAGCAGGACAGGAACGGTTC

CAGTCTCTCGGTGTGGCCTTCTACAGAGGTGCAGACTGCTGCGTTCTGGTATTTGATGTGACTGCCCCCA

ACACATTCAAAACCCTAGATAGCTGGAGAGATGAGTTTCTCATCCAGGCCAGTCCCCGAGATCCTGAAAA

CTTCCCATTTGTTGTGTTGGGAAACAAGATTGACCTCGAAAACAGACAAGTGGCCACAAAGCGGGCACAG

GCCTGGTGCTACAGCAAAAACAACATTCCCTACTTTGAGACCAGTGCCAAGGAGGCCATCAACGTGGAGC

AGGCGTTCCAGACGATTGCACGGAATGCACTTAAGCAGGAAACGGAGGTGGAGCTGTACAACGAATTTCC

TGAACCTATCAAACTGGACAAGAATGACCGGGCCAAGGCCTCGGCAGAAAGCTGCAGTTGCTGA

>lcl|NM_018890.4_cds_NP_061485.1_1 [gene=RAC1] [db_xref=CCDS:CCDS5349.1] [protein=ras-related C3 botulinum toxin substrate 1 isoform Rac1b] [protein_id=NP_061485.1] [location=210..845] [gbkey=CDS]

ATGCAGGCCATCAAGTGTGTGGTGGTGGGAGACGGAGCTGTAGGTAAAACTTGCCTACTGATCAGTTACA

CAACCAATGCATTTCCTGGAGAATATATCCCTACTGTCTTTGACAATTATTCTGCCAATGTTATGGTAGA

TGGAAAACCGGTGAATCTGGGCTTATGGGATACAGCTGGACAAGAAGATTATGACAGATTACGCCCCCTA

TCCTATCCGCAAACAGTTGGAGAAACGTACGGTAAGGATATAACCTCCCGGGGCAAAGACAAGCCGATTG

CCGATGTGTTCTTAATTTGCTTTTCCCTTGTGAGTCCTGCATCATTTGAAAATGTCCGTGCAAAGTGGTA

TCCTGAGGTGCGGCACCACTGTCCCAACACTCCCATCATCCTAGTGGGAACTAAACTTGATCTTAGGGAT

GATAAAGACACGATCGAGAAACTGAAGGAGAAGAAGCTGACTCCCATCACCTATCCGCAGGGTCTAGCCA

TGGCTAAGGAGATTGGTGCTGTAAAATACCTGGAGTGCTCGGCGCTCACACAGCGAGGCCTCAAGACAGT

GTTTGACGAAGCGATCCGAGCAGTCCTCTGCCCGCCTCCCGTGAAGAAGAGGAAGAGAAAATGCCTGCTG

TTGTAA

>lcl|NM_006098.5_cds_NP_006089.1_1 [gene=RACK1] [db_xref=CCDS:CCDS34324.1] [protein=receptor of activated protein C kinase 1] [protein_id=NP_006089.1] [location=107..1060] [gbkey=CDS]

ATGACTGAGCAGATGACCCTTCGTGGCACCCTCAAGGGCCACAACGGCTGGGTAACCCAGATCGCTACTA

CCCCGCAGTTCCCGGACATGATCCTCTCCGCCTCTCGAGATAAGACCATCATCATGTGGAAACTGACCAG

GGATGAGACCAACTATGGAATTCCACAGCGTGCTCTGCGGGGTCACTCCCACTTTGTTAGTGATGTGGTT

ATCTCCTCAGATGGCCAGTTTGCCCTCTCAGGCTCCTGGGATGGAACCCTGCGCCTCTGGGATCTCACAA

CGGGCACCACCACGAGGCGATTTGTGGGCCATACCAAGGATGTGCTGAGTGTGGCCTTCTCCTCTGACAA

CCGGCAGATTGTCTCTGGATCTCGAGATAAAACCATCAAGCTATGGAATACCCTGGGTGTGTGCAAATAC

ACTGTCCAGGATGAGAGCCACTCAGAGTGGGTGTCTTGTGTCCGCTTCTCGCCCAACAGCAGCAACCCTA

TCATCGTCTCCTGTGGCTGGGACAAGCTGGTCAAGGTATGGAACCTGGCTAACTGCAAGCTGAAGACCAA

CCACATTGGCCACACAGGCTATCTGAACACGGTGACTGTCTCTCCAGATGGATCCCTCTGTGCTTCTGGA

GGCAAGGATGGCCAGGCCATGTTATGGGATCTCAACGAAGGCAAACACCTTTACACGCTAGATGGTGGGG

ACATCATCAACGCCCTGTGCTTCAGCCCTAACCGCTACTGGCTGTGTGCTGCCACAGGCCCCAGCATCAA

GATCTGGGATTTAGAGGGAAAGATCATTGTAGATGAACTGAAGCAAGAAGTTATCAGTACCAGCAGCAAG

GCAGAACCACCCCAGTGCACCTCCCTGGCCTGGTCTGCTGATGGCCAGACTCTGTTTGCTGGCTACACGG

ACAACCTGGTGCGAGTGTGGCAGGTGACCATTGGCACACGCTAG

>lcl|NM_002880.4_cds_NP_002871.1_1 [gene=RAF1] [db_xref=CCDS:CCDS2612.1] [protein=RAF proto-oncogene serine/threonine-protein kinase isoform b] [protein_id=NP_002871.1] [location=332..2278] [gbkey=CDS]

ATGGAGCACATACAGGGAGCTTGGAAGACGATCAGCAATGGTTTTGGATTCAAAGATGCCGTGTTTGATG

GCTCCAGCTGCATCTCTCCTACAATAGTTCAGCAGTTTGGCTATCAGCGCCGGGCATCAGATGATGGCAA

ACTCACAGATCCTTCTAAGACAAGCAACACTATCCGTGTTTTCTTGCCGAACAAGCAAAGAACAGTGGTC

AATGTGCGAAATGGAATGAGCTTGCATGACTGCCTTATGAAAGCACTCAAGGTGAGGGGCCTGCAACCAG

AGTGCTGTGCAGTGTTCAGACTTCTCCACGAACACAAAGGTAAAAAAGCACGCTTAGATTGGAATACTGA

TGCTGCGTCTTTGATTGGAGAAGAACTTCAAGTAGATTTCCTGGATCATGTTCCCCTCACAACACACAAC

TTTGCTCGGAAGACGTTCCTGAAGCTTGCCTTCTGTGACATCTGTCAGAAATTCCTGCTCAATGGATTTC

GATGTCAGACTTGTGGCTACAAATTTCATGAGCACTGTAGCACCAAAGTACCTACTATGTGTGTGGACTG

GAGTAACATCAGACAACTCTTATTGTTTCCAAATTCCACTATTGGTGATAGTGGAGTCCCAGCACTACCT

TCTTTGACTATGCGTCGTATGCGAGAGTCTGTTTCCAGGATGCCTGTTAGTTCTCAGCACAGATATTCTA

CACCTCACGCCTTCACCTTTAACACCTCCAGTCCCTCATCTGAAGGTTCCCTCTCCCAGAGGCAGAGGTC

GACATCCACACCTAATGTCCACATGGTCAGCACCACCCTGCCTGTGGACAGCAGGATGATTGAGGATGCA

ATTCGAAGTCACAGCGAATCAGCCTCACCTTCAGCCCTGTCCAGTAGCCCCAACAATCTGAGCCCAACAG

GCTGGTCACAGCCGAAAACCCCCGTGCCAGCACAAAGAGAGCGGGCACCAGTATCTGGGACCCAGGAGAA

AAACAAAATTAGGCCTCGTGGACAGAGAGATTCAAGCTATTATTGGGAAATAGAAGCCAGTGAAGTGATG

CTGTCCACTCGGATTGGGTCAGGCTCTTTTGGAACTGTTTATAAGGGTAAATGGCACGGAGATGTTGCAG

TAAAGATCCTAAAGGTTGTCGACCCAACCCCAGAGCAATTCCAGGCCTTCAGGAATGAGGTGGCTGTTCT

GCGCAAAACACGGCATGTGAACATTCTGCTTTTCATGGGGTACATGACAAAGGACAACCTGGCAATTGTG

ACCCAGTGGTGCGAGGGCAGCAGCCTCTACAAACACCTGCATGTCCAGGAGACCAAGTTTCAGATGTTCC

AGCTAATTGACATTGCCCGGCAGACGGCTCAGGGAATGGACTATTTGCATGCAAAGAACATCATCCATAG

AGACATGAAATCCAACAATATATTTCTCCATGAAGGCTTAACAGTGAAAATTGGAGATTTTGGTTTGGCA

ACAGTAAAGTCACGCTGGAGTGGTTCTCAGCAGGTTGAACAACCTACTGGCTCTGTCCTCTGGATGGCCC

CAGAGGTGATCCGAATGCAGGATAACAACCCATTCAGTTTCCAGTCGGATGTCTACTCCTATGGCATCGT

ATTGTATGAACTGATGACGGGGGAGCTTCCTTATTCTCACATCAACAACCGAGATCAGATCATCTTCATG

GTGGGCCGAGGATATGCCTCCCCAGATCTTAGTAAGCTATATAAGAACTGCCCCAAAGCAATGAAGAGGC

TGGTAGCTGACTGTGTGAAGAAAGTAAAGGAAGAGAGGCCTCTTTTTCCCCAGATCCTGTCTTCCATTGA

GCTGCTCCAACACTCTCTACCGAAGATCAACCGGAGCGCTTCCGAGCCATCCTTGCATCGGGCAGCCCAC

ACTGAGGATATCAATGCTTGCACGCTGACCACGTCCCCGAGGCTGCCTGTCTTCTAG

>lcl|NM_000321.3_cds_NP_000312.2_1 [gene=RB1] [db_xref=CCDS:CCDS31973.1] [protein=retinoblastoma-associated protein isoform 1] [protein_id=NP_000312.2] [location=163..2949] [gbkey=CDS]

ATGCCGCCCAAAACCCCCCGAAAAACGGCCGCCACCGCCGCCGCTGCCGCCGCGGAACCCCCGGCACCGC

CGCCGCCGCCCCCTCCTGAGGAGGACCCAGAGCAGGACAGCGGCCCGGAGGACCTGCCTCTCGTCAGGCT

TGAGTTTGAAGAAACAGAAGAACCTGATTTTACTGCATTATGTCAGAAATTAAAGATACCAGATCATGTC

AGAGAGAGAGCTTGGTTAACTTGGGAGAAAGTTTCATCTGTGGATGGAGTATTGGGAGGTTATATTCAAA

AGAAAAAGGAACTGTGGGGAATCTGTATCTTTATTGCAGCAGTTGACCTAGATGAGATGTCGTTCACTTT

TACTGAGCTACAGAAAAACATAGAAATCAGTGTCCATAAATTCTTTAACTTACTAAAAGAAATTGATACC

AGTACCAAAGTTGATAATGCTATGTCAAGACTGTTGAAGAAGTATGATGTATTGTTTGCACTCTTCAGCA

AATTGGAAAGGACATGTGAACTTATATATTTGACACAACCCAGCAGTTCGATATCTACTGAAATAAATTC

TGCATTGGTGCTAAAAGTTTCTTGGATCACATTTTTATTAGCTAAAGGGGAAGTATTACAAATGGAAGAT

GATCTGGTGATTTCATTTCAGTTAATGCTATGTGTCCTTGACTATTTTATTAAACTCTCACCTCCCATGT

TGCTCAAAGAACCATATAAAACAGCTGTTATACCCATTAATGGTTCACCTCGAACACCCAGGCGAGGTCA

GAACAGGAGTGCACGGATAGCAAAACAACTAGAAAATGATACAAGAATTATTGAAGTTCTCTGTAAAGAA

CATGAATGTAATATAGATGAGGTGAAAAATGTTTATTTCAAAAATTTTATACCTTTTATGAATTCTCTTG

GACTTGTAACATCTAATGGACTTCCAGAGGTTGAAAATCTTTCTAAACGATACGAAGAAATTTATCTTAA

AAATAAAGATCTAGATGCAAGATTATTTTTGGATCATGATAAAACTCTTCAGACTGATTCTATAGACAGT

TTTGAAACACAGAGAACACCACGAAAAAGTAACCTTGATGAAGAGGTGAATGTAATTCCTCCACACACTC

CAGTTAGGACTGTTATGAACACTATCCAACAATTAATGATGATTTTAAATTCAGCAAGTGATCAACCTTC

AGAAAATCTGATTTCCTATTTTAACAACTGCACAGTGAATCCAAAAGAAAGTATACTGAAAAGAGTGAAG

GATATAGGATACATCTTTAAAGAGAAATTTGCTAAAGCTGTGGGACAGGGTTGTGTCGAAATTGGATCAC

AGCGATACAAACTTGGAGTTCGCTTGTATTACCGAGTAATGGAATCCATGCTTAAATCAGAAGAAGAACG

ATTATCCATTCAAAATTTTAGCAAACTTCTGAATGACAACATTTTTCATATGTCTTTATTGGCGTGCGCT

CTTGAGGTTGTAATGGCCACATATAGCAGAAGTACATCTCAGAATCTTGATTCTGGAACAGATTTGTCTT

TCCCATGGATTCTGAATGTGCTTAATTTAAAAGCCTTTGATTTTTACAAAGTGATCGAAAGTTTTATCAA

AGCAGAAGGCAACTTGACAAGAGAAATGATAAAACATTTAGAACGATGTGAACATCGAATCATGGAATCC

CTTGCATGGCTCTCAGATTCACCTTTATTTGATCTTATTAAACAATCAAAGGACCGAGAAGGACCAACTG

ATCACCTTGAATCTGCTTGTCCTCTTAATCTTCCTCTCCAGAATAATCACACTGCAGCAGATATGTATCT

TTCTCCTGTAAGATCTCCAAAGAAAAAAGGTTCAACTACGCGTGTAAATTCTACTGCAAATGCAGAGACA

CAAGCAACCTCAGCCTTCCAGACCCAGAAGCCATTGAAATCTACCTCTCTTTCACTGTTTTATAAAAAAG

TGTATCGGCTAGCCTATCTCCGGCTAAATACACTTTGTGAACGCCTTCTGTCTGAGCACCCAGAATTAGA

ACATATCATCTGGACCCTTTTCCAGCACACCCTGCAGAATGAGTATGAACTCATGAGAGACAGGCATTTG

GACCAAATTATGATGTGTTCCATGTATGGCATATGCAAAGTGAAGAATATAGACCTTAAATTCAAAATCA

TTGTAACAGCATACAAGGATCTTCCTCATGCTGTTCAGGAGACATTCAAACGTGTTTTGATCAAAGAAGA

GGAGTATGATTCTATTATAGTATTCTATAACTCGGTCTTCATGCAGAGACTGAAAACAAATATTTTGCAG

TATGCTTCCACCAGGCCCCCTACCTTGTCACCAATACCTCACATTCCTCGAAGCCCTTACAAGTTTCCTA

GTTCACCCTTACGGATTCCTGGAGGGAACATCTATATTTCACCCCTGAAGAGTCCATATAAAATTTCAGA

AGGTCTGCCAACACCAACAAAAATGACTCCAAGATCAAGAATCTTAGTATCAATTGGTGAATCATTCGGG

ACTTCTGAGAAGTTCCAGAAAATAAATCAGATGGTATGTAACAGCGACCGTGTGCTCAAAAGAAGTGCTG

AAGGAAGCAACCCTCCTAAACCACTGAAAAAACTACGCTTTGATATTGAAGGATCAGATGAAGCAGATGG

AAGTAAACATCTCCCAGGAGAGTCCAAATTTCAGCAGAAACTGGCAGAAATGACTTCTACTCGAACACGA

ATGCAAAAGCAGAAAATGAATGATAGCATGGATACCTCAAACAAGGAAGAGAAATGA

>lcl|NM_014781.5_cds_NP_055596.3_1 [gene=RB1CC1] [db_xref=CCDS:CCDS34892.1] [protein=RB1-inducible coiled-coil protein 1 isoform 1] [protein_id=NP_055596.3] [location=528..5312] [gbkey=CDS]

ATGAAGTTATATGTATTTCTGGTTAACACTGGAACTACTCTAACATTTGACACTGAACTTACAGTGCAAA

CTGTGGCAGACCTTAAGCATGCCATTCAAAGCAAATACAAGATTGCTATTCAACACCAGGTGCTGGTGGT

CAATGGAGGAGAATGCATGGCTGCAGATCGAAGAGTGTGTACCTACAGTGCTGGGACGGATACAAATCCA

ATTTTTCTTTTTAACAAAGAAATGATCTTATGTGATCGTCCACCTGCTATTCCTAAAACTACCTTTTCGA

CAGAAAATGACATGGAAATAAAAGTTGAAGAATCTCTTATGATGCCTGCAGTTTTTCATACTGTTGCTTC

AAGGACACAGCTTGCATTGGAAATGTATGAAGTTGCCAAGAAACTTTGTTCTTTTTGTGAAGGTCTTGTA

CATGATGAACATCTTCAACACCAAGGCTGGGCTGCAATCATGGCCAACCTGGAGGACTGTTCAAATTCAT

ACCAAAAGCTACTTTTCAAGTTTGAAAGTATTTATTCAAATTATCTGCAGTCCATAGAAGACATCAAGTT

AAAACTTACTCATTTAGGAACTGCAGTTTCAGTAATGGCCAAGATTCCACTGTTGGAGTGCCTAACCAGA

CATAGTTACAGAGAATGTTTGGGAAGACTGGATTCTTTACCTGAACATGAAGACTCAGAAAAAGCTGAGA

TGAAAAGATCCACTGAACTGGTGCTCTCTCCTGATATGCCTAGAACAACTAACGAATCTTTGTTAACCTC

ATTTCCCAAGTCAGTGGAACATGTGTCCCCAGATACCGCAGATGCTGAAAGTGGCAAAGAAATTAGGGAA

TCTTGTCAAAGTACTGTTCATCAGCAAGATGAAACTACGATTGACACTAAAGATGGTGATCTGCCCTTTT

TTAATGTCTCTTTGTTAGACTGGATAAATGTTCAAGATAGACCTAATGATGTGGAATCTTTGGTCAGGAA

GTGCTTTGATTCTATGAGCAGGCTTGATCCAAGGATTATTCGACCATTTATAGCAGAATGCCGTCAAACT

ATTGCCAAACTTGATAATCAGAATATGAAAGCCATTAAAGGACTTGAAGATCGGCTCTACGCCCTGGACC

AGATGATTGCTAGCTGTGGCCGACTGGTGAATGAACAGAAAGAGCTTGCTCAGGGATTTTTAGCTAATCA

GAAGAGAGCTGAAAACTTAAAGGATGCATCTGTATTACCTGATTTATGCCTGAGTCACGCAAATCAGTTG

ATGATTATGTTGCAAAATCATAGAAAACTGTTAGATATTAAGCAGAAGTGTACCACTGCCAAACAAGAAC

TAGCAAATAACCTACATGTCAGACTGAAGTGGTGTTGCTTTGTAATGCTTCATGCTGATCAAGATGGAGA

GAAGTTACAAGCTTTGCTCCGCCTCGTAATAGAGCTGTTAGAAAGAGTCAAAATTGTTGAAGCTCTTAGT

ACAGTTCCTCAGATGTACTGCTTAGCTGTTGTTGAGGTTGTAAGAAGAAAAATGTTCATAAAACACTACA

GGGAGTGGGCTGGTGCTTTAGTCAAAGATGGAAAGAGATTATATGAAGCAGAAAAATCAAAAAGGGAATC

CTTTGGGAAATTATTTAGGAAGTCTTTTTTAAGAAATCGTCTGTTTAGGGGACTGGACTCCTGGCCCCCT

TCCTTTTGTACTCAAAAGCCTCGAAAGTTTGACTGTGAACTTCCAGATATTTCATTAAAAGATTTACAGT

TTCTGCAATCATTTTGTCCTTCGGAAGTTCAGCCATTCCTCAGGGTTCCCTTACTTTGTGACTTTGAACC

TCTACACCAGCATGTACTTGCTCTACATAATTTGGTAAAAGCAGCACAAAGTTTGGATGAAATGTCACAG

ACCATTACAGATCTACTGAGTGAACAAAAGGCATCTGTGAGTCAGACATCCCCACAGTCTGCTTCTTCAC

CAAGGATGGAAAGTACAGCAGGAATTACAACTACTACCTCACCGAGAACTCCTCCACCACTGACTGTTCA

GGATCCCTTATGTCCTGCAGTTTGTCCCTTAGAAGAATTATCTCCAGATAGTATTGATGCACATACGTTT

GATTTTGAAACTATTCCCCATCCAAACATAGAACAGACTATTCACCAAGTTTCTTTAGACTTGGATTCAT

TAGCAGAAAGTCCTGAATCAGATTTTATGTCTGCTGTGAATGAGTTTGTAATAGAAGAAAATTTGTCGTC

TCCTAATCCTATAAGTGATCCACAAAGCCCAGAAATGATGGTGGAATCACTTTATTCATCAGTTATCAAT

GCGATAGACAGTAGACGAATGCAGGATACAAATGTATGTGGTAAGGAGGATTTTGGAGATCATACTTCTC

TGAATGTCCAGTTGGAAAGATGTAGAGTTGTTGCCCAAGACTCTCACTTCAGTATACAAACCATTAAGGA

AGACCTTTGCCACTTTAGAACATTTGTACAAAAAGAACAGTGTGACTTCTCAAATTCATTAAAATGTACA

GCAGTAGAAATAAGAAACATTATTGAAAAAGTAAAATGTTCTCTGGAAATAACACTAAAAGAAAAACATC

AAAAAGAACTACTGTCTTTAAAAAATGAATATGAAGGTAAACTTGACGGACTAATAAAGGAAACTGAAGA

GAATGAAAACAAAATTAAAAAATTGAAGGGAGAGTTAGTATGCCTTGAGGAGGTTTTACAAAATAAAGAT

AATGAATTTGCTTTGGTTAAACATGAAAAAGAAGCTGTAATCTGCCTGCAGAATGAAAAGGATCAGAAGT

TGTTAGAGATGGAAAATATAATGCACTCTCAAAATTGTGAAATTAAAGAACTGAAGCAGTCACGAGAAAT

AGTGTTAGAAGACTTAAAAAAGCTCCATGTTGAAAATGATGAGAAGTTACAGTTATTGAGGGCAGAACTT

CAGTCCTTGGAGCAAAGTCATCTAAAGGAATTAGAGGACACACTTCAGGTTAGGCACATACAAGAGTTTG

AGAAGGTTATGACAGACCACAGAGTTTCTTTGGAGGAATTAAAAAAGGAAAACCAACAAATAATTAATCA

AATACAAGAATCTCATGCTGAAATTATCCAGGAAAAAGAAAAACAGTTACAGGAATTAAAACTCAAGGTT

TCTGATTTGTCAGACACGAGATGCAAGTTAGAGGTTGAACTTGCGTTGAAGGAAGCAGAAACTGATGAAA

TAAAAATTTTGCTGGAAGAAAGCAGAGCCCAGCAGAAGGAGACCTTGAAATCTCTTCTTGAACAAGAGAC

AGAAAATTTGAGAACAGAAATTAGTAAACTCAACCAAAAGATTCAGGATAATAATGAAAATTATCAGGTG

GGCTTAGCAGAGCTAAGAACTTTAATGACAATTGAAAAAGATCAGTGTATTTCCGAGTTAATTAGTAGAC

ATGAAGAAGAATCTAATATACTTAAAGCTGAATTAAACAAAGTAACATCTTTGCATAACCAAGCATTTGA

AATAGAAAAAAACCTAAAAGAACAAATAATTGAACTGCAGAGTAAATTGGATTCAGAATTGAGTGCTCTT

GAAAGACAAAAAGATGAAAAAATTACCCAACAAGAAGAGAAATACGAAGCTATTATCCAGAACCTTGAGA

AAGACAGACAAAAATTGGTCAGCAGCCAGGAGCAAGACAGAGAACAGTTAATTCAGAAGCTTAATTGTGA

AAAAGATGAAGCTATTCAGACTGCCCTAAAAGAATTTAAATTGGAGAGAGAAGTTGTTGAGAAAGAGTTA

TTAGAAAAAGTTAAACATCTTGAGAATCAAATAGCAAAAAGTCCTGCCATTGACTCTACCAGAGGAGATT

CTTCAAGCTTAGTTGCTGAACTTCAAGAAAAGCTTCAGGAAGAAAAAGCTAAGTTTCTAGAACAACTTGA

AGAGCAAGAAAAAAGAAAGAATGAAGAAATGCAAAATGTTCGAACATCTTTGATTGCGGAACAACAGACC

AATTTTAACACTGTTTTAACAAGAGAGAAAATGAGAAAAGAAAACATAATAAATGATCTTAGTGATAAGT

TGAAAAGTACAATGCAGCAACAAGAACGGGATAAAGATTTGATAGAGTCACTTTCTGAAGATCGAGCTCG

TTTGCTTGAGGAAAAGAAAAAGCTTGAAGAAGAAGTCAGTAAGTTGCGTAGTAGCAGTTTTGTTCCTTCA

CCATATGTAGCTACAGCCCCAGAACTTTATGGAGCTTGTGCACCTGAACTCCCAGGTGAATCAGATAGAT

CCGCTGTGGAAACAGCAGATGAAGGAAGAGTGGATTCAGCAATGGAGACAAGCATGATGTCTGTACAAGA

AAATATTCATATGTTGTCTGAAGAAAAACAGCGGATAATGCTGTTAGAACGAACATTGCAATTGAAAGAA

GAAGAAAATAAACGGTTAAATCAAAGACTGATGTCTCAGAGCATGTCTTCAGTATCTTCAAGGCATTCTG

AAAAGATAGCTATTAGAGATTTTCAGGTGGGAGATTTGGTACTCATCATCCTAGACGAACGCCATGACAA

TTATGTGTTATTTACTGTTAGTCCTACTTTATATTTTCTACATTCAGAGTCTCTACCTGCCCTGGATCTC

AAACCAGGTGAGGGTGCTTCAGGTGCATCTAGAAGACCCTGGGTACTTGGAAAAGTAATGGAAAAAGAAT

ACTGTCAAGCCAAAAAGGCACAAAACAGATTTAAAGTTCCTTTGGGGACAAAGTTTTACAGAGTGAAAGC

CGTATCATGGAATAAGAAAGTATAA

>lcl|NM_021975.4_cds_NP_068810.3_1 [gene=RELA] [db_xref=CCDS:CCDS31609.1] [protein=transcription factor p65 isoform 1] [protein_id=NP_068810.3] [location=85..1740] [gbkey=CDS]

ATGGACGAACTGTTCCCCCTCATCTTCCCGGCAGAGCCAGCCCAGGCCTCTGGCCCCTATGTGGAGATCA

TTGAGCAGCCCAAGCAGCGGGGCATGCGCTTCCGCTACAAGTGCGAGGGGCGCTCCGCGGGCAGCATCCC

AGGCGAGAGGAGCACAGATACCACCAAGACCCACCCCACCATCAAGATCAATGGCTACACAGGACCAGGG

ACAGTGCGCATCTCCCTGGTCACCAAGGACCCTCCTCACCGGCCTCACCCCCACGAGCTTGTAGGAAAGG

ACTGCCGGGATGGCTTCTATGAGGCTGAGCTCTGCCCGGACCGCTGCATCCACAGTTTCCAGAACCTGGG

AATCCAGTGTGTGAAGAAGCGGGACCTGGAGCAGGCTATCAGTCAGCGCATCCAGACCAACAACAACCCC

TTCCAAGTTCCTATAGAAGAGCAGCGTGGGGACTACGACCTGAATGCTGTGCGGCTCTGCTTCCAGGTGA

CAGTGCGGGACCCATCAGGCAGGCCCCTCCGCCTGCCGCCTGTCCTTTCTCATCCCATCTTTGACAATCG

TGCCCCCAACACTGCCGAGCTCAAGATCTGCCGAGTGAACCGAAACTCTGGCAGCTGCCTCGGTGGGGAT

GAGATCTTCCTACTGTGTGACAAGGTGCAGAAAGAGGACATTGAGGTGTATTTCACGGGACCAGGCTGGG

AGGCCCGAGGCTCCTTTTCGCAAGCTGATGTGCACCGACAAGTGGCCATTGTGTTCCGGACCCCTCCCTA

CGCAGACCCCAGCCTGCAGGCTCCTGTGCGTGTCTCCATGCAGCTGCGGCGGCCTTCCGACCGGGAGCTC

AGTGAGCCCATGGAATTCCAGTACCTGCCAGATACAGACGATCGTCACCGGATTGAGGAGAAACGTAAAA

GGACATATGAGACCTTCAAGAGCATCATGAAGAAGAGTCCTTTCAGCGGACCCACCGACCCCCGGCCTCC

ACCTCGACGCATTGCTGTGCCTTCCCGCAGCTCAGCTTCTGTCCCCAAGCCAGCACCCCAGCCCTATCCC

TTTACGTCATCCCTGAGCACCATCAACTATGATGAGTTTCCCACCATGGTGTTTCCTTCTGGGCAGATCA

GCCAGGCCTCGGCCTTGGCCCCGGCCCCTCCCCAAGTCCTGCCCCAGGCTCCAGCCCCTGCCCCTGCTCC

AGCCATGGTATCAGCTCTGGCCCAGGCCCCAGCCCCTGTCCCAGTCCTAGCCCCAGGCCCTCCTCAGGCT

GTGGCCCCACCTGCCCCCAAGCCCACCCAGGCTGGGGAAGGAACGCTGTCAGAGGCCCTGCTGCAGCTGC

AGTTTGATGATGAAGACCTGGGGGCCTTGCTTGGCAACAGCACAGACCCAGCTGTGTTCACAGACCTGGC

ATCCGTCGACAACTCCGAGTTTCAGCAGCTGCTGAACCAGGGCATACCTGTGGCCCCCCACACAACTGAG

CCCATGCTGATGGAGTACCCTGAGGCTATAACTCGCCTAGTGACAGGGGCCCAGAGGCCCCCCGACCCAG

CTCCTGCTCCACTGGGGGCCCCGGGGCTCCCCAATGGCCTCCTTTCAGGAGATGAAGACTTCTCCTCCAT

TGCGGACATGGACTTCTCAGCCCTGCTGAGTCAGATCAGCTCCTAA

>lcl|NM_005873.3_cds_NP_005864.1_1 [gene=RGS19] [db_xref=CCDS:CCDS13555.1] [protein=regulator of G-protein signaling 19] [protein_id=NP_005864.1] [location=294..947] [gbkey=CDS]

ATGCCCACCCCGCATGAGGCTGAGAAGCAGATCACAGGGCCAGAGGAGGCGGACCGGCCCCCTTCAATGT

CCAGTCATGATACAGCCTCTCCAGCGGCCCCCAGCCGCAACCCCTGCTGCCTGTGCTGGTGCTGCTGCTG

TAGCTGCTCCTGGAACCAAGAGCGGCGGCGCGCGTGGCAGGCCTCCCGGGAGAGCAAGCTGCAGCCCCTC

CCCAGCTGTGAAGTATGTGCCACGCCAAGTCCTGAGGAGGTGCAGAGCTGGGCGCAGTCTTTTGACAAGC

TGATGCACAGCCCAGCGGGACGCAGCGTGTTCCGGGCGTTCCTGCGGACAGAGTACAGCGAGGAGAACAT

GCTCTTCTGGTTGGCCTGCGAGGAGCTGAAGGCCGAGGCCAACCAGCATGTGGTAGACGAGAAGGCGAGG

CTCATCTACGAGGACTACGTATCCATCCTGTCCCCCAAGGAGGTGAGCCTGGACTCCCGTGTGCGGGAGG

GCATCAACAAGAAGATGCAGGAGCCGTCCGCACACACGTTCGACGACGCGCAGCTGCAGATCTACACGCT

CATGCACCGGGACTCCTACCCCCGCTTCCTCAGCTCTCCCACCTACCGTGCCCTGCTGCTGCAGGGGCCA

TCACAGTCCTCCTCCGAGGCCTAG

>lcl|NM_005614.4_cds_NP_005605.1_1 [gene=RHEB] [db_xref=CCDS:CCDS5927.1] [protein=GTP-binding protein Rheb] [protein_id=NP_005605.1] [location=385..939] [gbkey=CDS]

ATGCCGCAGTCCAAGTCCCGGAAGATCGCGATCCTGGGCTACCGGTCTGTGGGGAAATCCTCATTGACGA

TTCAATTTGTTGAAGGCCAATTTGTGGACTCCTACGATCCAACCATAGAAAACACTTTTACAAAGTTGAT

CACAGTAAATGGACAAGAATATCATCTTCAACTTGTAGACACAGCCGGGCAAGATGAATATTCTATCTTT

CCTCAGACATACTCCATAGATATTAATGGCTATATTCTTGTGTATTCTGTTACATCAATCAAAAGTTTTG

AAGTGATTAAAGTTATCCATGGCAAATTGTTGGATATGGTGGGGAAAGTACAAATACCTATTATGTTGGT

TGGGAATAAGAAAGACCTGCATATGGAAAGGGTGATCAGTTATGAAGAAGGGAAAGCTTTGGCAGAATCT

TGGAATGCAGCTTTTTTGGAATCTTCTGCTAAAGAAAATCAGACTGCTGTGGATGTTTTTCGAAGGATAA

TTTTGGAGGCAGAAAAAATGGACGGGGCAGCTTCACAAGGCAAGTCTTCATGCTCGGTGATGTGA

>lcl|NM_003161.4_cds_NP_003152.1_1 [gene=RPS6KB1] [db_xref=CCDS:CCDS11621.1] [protein=ribosomal protein S6 kinase beta-1 isoform a] [protein_id=NP_003152.1] [location=65..1642] [gbkey=CDS]

ATGAGGCGACGAAGGAGGCGGGACGGCTTTTACCCAGCCCCGGACTTCCGAGACAGGGAAGCTGAGGACA

TGGCAGGAGTGTTTGACATAGACCTGGACCAGCCAGAGGACGCGGGCTCTGAGGATGAGCTGGAGGAGGG

GGGTCAGTTAAATGAAAGCATGGACCATGGGGGAGTTGGACCATATGAACTTGGCATGGAACATTGTGAG

AAATTTGAAATCTCAGAAACTAGTGTGAACAGAGGGCCAGAAAAAATCAGACCAGAATGTTTTGAGCTAC

TTCGGGTACTTGGTAAAGGGGGCTATGGAAAGGTTTTTCAAGTACGAAAAGTAACAGGAGCAAATACTGG

GAAAATATTTGCCATGAAGGTGCTTAAAAAGGCAATGATAGTAAGAAATGCTAAAGATACAGCTCATACA

AAAGCAGAACGGAATATTCTGGAGGAAGTAAAGCATCCCTTCATCGTGGATTTAATTTATGCCTTTCAGA

CTGGTGGAAAACTCTACCTCATCCTTGAGTATCTCAGTGGAGGAGAACTATTTATGCAGTTAGAAAGAGA

GGGAATATTTATGGAAGACACTGCCTGCTTTTACTTGGCAGAAATCTCCATGGCTTTGGGGCATTTACAT

CAAAAGGGGATCATCTACAGAGACCTGAAGCCGGAGAATATCATGCTTAATCACCAAGGTCATGTGAAAC

TAACAGACTTTGGACTATGCAAAGAATCTATTCATGATGGAACAGTCACACACACATTTTGTGGAACAAT

AGAATACATGGCCCCTGAAATCTTGATGAGAAGTGGCCACAATCGTGCTGTGGATTGGTGGAGTTTGGGA

GCATTAATGTATGACATGCTGACTGGAGCACCCCCATTCACTGGGGAGAATAGAAAGAAAACAATTGACA

AAATCCTCAAATGTAAACTCAATTTGCCTCCCTACCTCACACAAGAAGCCAGAGATCTGCTTAAAAAGCT

GCTGAAAAGAAATGCTGCTTCTCGTCTGGGAGCTGGTCCTGGGGACGCTGGAGAAGTTCAAGCTCATCCA

TTCTTTAGACACATTAACTGGGAAGAACTTCTGGCTCGAAAGGTGGAGCCCCCCTTTAAACCTCTGTTGC

AATCTGAAGAGGATGTAAGTCAGTTTGATTCCAAGTTTACACGTCAGACACCTGTCGACAGCCCAGATGA

CTCAACTCTCAGTGAAAGTGCCAATCAGGTCTTTCTGGGTTTTACATATGTGGCTCCATCTGTACTTGAA

AGTGTGAAAGAAAAGTTTTCCTTTGAACCAAAAATCCGATCACCTCGAAGATTTATTGGCAGCCCACGAA

CACCTGTCAGCCCAGTCAAATTTTCTCCTGGGGATTTCTGGGGAAGAGGTGCTTCGGCCAGCACAGCAAA

TCCTCAGACACCTGTGGAATACCCAATGGAAACAAGTGGCATAGAGCAGATGGATGTGACAATGAGTGGG

GAAGCATCGGCACCACTTCCAATACGACAGCCGAACTCTGGGCCATACAAAAAACAAGCTTTTCCCATGA

TCTCCAAACGGCCAGAGCACCTGCGTATGAATCTATGA

>lcl|NM_020761.3_cds_NP_065812.1_1 [gene=RPTOR] [db_xref=CCDS:CCDS11773.1] [protein=regulatory-associated protein of mTOR isoform 1] [protein_id=NP_065812.1] [location=793..4800] [gbkey=CDS]

ATGGAGTCCGAAATGCTGCAATCGCCTCTTCTGGGCCTGGGGGAGGAAGATGAGGCTGATCTTACAGACT

GGAACCTACCTTTGGCTTTTATGAAAAAGAGGCACTGTGAGAAAATTGAAGGCTCCAAATCCTTAGCTCA

GAGCTGGAGGATGAAGGATCGGATGAAGACAGTCAGTGTTGCCTTAGTTTTGTGCCTGAATGTTGGTGTG

GACCCTCCCGATGTGGTGAAGACCACGCCCTGTGCACGCTTGGAATGCTGGATCGATCCTCTGTCGATGG

GTCCTCAGAAAGCTCTGGAAACCATCGGTGCAAATTTACAGAAGCAGTACGAGAACTGGCAGCCAAGGGC

CCGGTACAAGCAGAGCCTTGACCCAACTGTGGATGAAGTCAAGAAGCTCTGCACGTCCTTACGTCGCAAC

GCCAAGGAGGAGCGAGTCCTCTTTCACTACAATGGCCACGGGGTGCCCCGGCCCACAGTCAACGGGGAGG

TCTGGGTCTTCAACAAGAACTACACGCAGTACATCCCTCTGTCCATATATGACCTGCAGACGTGGATGGG

CAGCCCGTCGATCTTCGTCTACGACTGCTCCAATGCTGGCTTGATCGTCAAGTCCTTCAAGCAGTTCGCA

CTACAGCGGGAGCAGGAGCTGGAGGTAGCTGCAATCAACCCAAATCACCCTCTTGCTCAGATGCCTTTGC

CTCCGTCGATGAAAAACTGCATCCAGCTGGCAGCCTGCGAGGCCACCGAGCTGCTGCCCATGATCCCCGA

CCTCCCGGCTGACCTATTCACCTCCTGCCTCACCACCCCCATCAAGATCGCCCTGCGCTGGTTTTGCATG

CAGAAATGTGTCAGTCTGGTGCCTGGCGTCACACTGGATTTGATAGAAAAGATCCCTGGCCGCCTGAACG

ACAGGAGGACGCCCCTGGGTGAACTGAACTGGATCTTCACAGCCATCACAGACACCATCGCGTGGAACGT

GCTCCCCCGGGATCTCTTCCAAAAGCTCTTCAGACAGGACTTGCTGGTGGCTAGTCTGTTTCGAAATTTT

TTATTGGCGGAAAGGATTATGAGGTCGTATAACTGCACTCCCGTCAGCAGCCCGCGTCTGCCGCCCACGT

ACATGCACGCCATGTGGCAAGCCTGGGACCTGGCTGTTGACATCTGTCTGTCTCAGCTGCCGACGATCAT

CGAGGAAGGCACTGCGTTTCGGCACAGCCCGTTCTTCGCCGAGCAGCTGACCGCATTCCAGGTGTGGCTC

ACCATGGGCGTGGAGAACCGAAACCCACCCGAACAGCTGCCCATCGTCCTGCAGGTGCTGTTAAGCCAAG

TGCACCGGCTGAGAGCATTGGACTTGCTTGGAAGATTTTTGGACCTGGGTCCCTGGGCAGTGAGCCTGGC

CTTGTCTGTCGGCATCTTCCCCTACGTGCTGAAGCTGCTCCAGAGCTCGGCCCGAGAGCTGCGGCCACTT

CTCGTTTTCATCTGGGCCAAGATCCTCGCAGTGGACAGCTCGTGCCAAGCGGACCTCGTGAAGGACAACG

GCCACAAGTACTTCCTGTCGGTCCTGGCGGACCCCTACATGCCAGCTGAACACCGGACCATGACGGCTTT

CATTCTCGCCGTGATCGTCAACAGCTATCACACGGGGCAGGAAGCCTGCCTTCAGGGAAACCTCATTGCC

ATCTGCCTGGAGCAGCTCAACGACCCGCACCCCTTGCTGCGCCAGTGGGTGGCCATCTGCCTCGGCAGGA

TCTGGCAGAACTTCGACTCGGCGAGGTGGTGCGGCGTGAGGGACAGCGCTCATGAGAAGCTCTACAGCCT

CCTCTCCGACCCCATTCCCGAGGTCCGCTGCGCAGCGGTCTTCGCCCTTGGCACGTTCGTGGGCAACTCT

GCAGAGAGGACGGACCACTCCACCACCATCGACCACAACGTGGCCATGATGCTGGCCCAGCTGGTCAGCG

ACGGGAGCCCCATGGTCCGGAAGGAGCTGGTGGTGGCTCTGAGTCATCTTGTGGTTCAGTATGAAAGCAA

TTTCTGCACCGTGGCCCTGCAGTTCATAGAAGAGGAAAAGAACTACGCCTTGCCTTCTCCAGCAACCACA

GAGGGAGGGAGTTTGACCCCAGTGCGAGACAGCCCGTGCACCCCCAGACTTCGTTCTGTGAGCTCCTATG

GAAACATCCGTGCTGTCGCCACAGCCAGGAGCCTCAACAAATCTTTGCAGAACCTGAGTTTGACAGAGGA

ATCTGGTGGCGCGGTGGCGTTCTCCCCCGGAAACCTCAGCACCAGCAGCAGCGCCAGCAGCACCCTGGGC

AGCCCCGAGAATGAGGAGCATATCCTGTCCTTCGAGACCATCGACAAGATGCGCCGCGCCAGCTCCTACT

CCTCCCTCAACTCCCTCATCGGAGTTTCCTTTAACAGTGTTTACACTCAGATTTGGAGAGTCCTGCTGCA

CCTGGCTGCTGACCCCTATCCAGAGGTCTCGGACGTGGCCATGAAAGTACTCAACAGCATCGCCTACAAG

GCCACCGTGAACGCCCGGCCGCAGCGCGTCCTGGACACCTCCTCCCTCACGCAGTCGGCCCCCGCCAGCC

CCACCAACAAGGGCGTGCACATCCACCAGGCGGGGGGCTCCCCTCCGGCGTCCAGCACCAGCAGCTCCAG

CCTGACCAACGATGTGGCCAAGCAGCCGGTCAGCCGAGACTTGCCTTCTGGCCGGCCGGGCACCACAGGC

CCCGCTGGGGCGCAGTACACCCCTCACTCCCACCAGTTCCCCCGGACACGGAAGATGTTCGACAAGGGCC

CAGAGCAGACTGCGGACGACGCGGACGATGCTGCTGGACACAAAAGTTTCATCTCCGCCACGGTGCAGAC

GGGGTTCTGCGACTGGAGCGCCCGCTATTTTGCCCAGCCCGTCATGAAGATCCCAGAAGAGCACGACCTG

GAGAGTCAGATCCGCAAGGAGCGGGAGTGGCGGTTCCTGCGAAACAGCCGTGTCAGGAGGCAGGCCCAGC

AAGTCATTCAGAAGGGCATTACGAGATTGGACGACCAAATATTTCTGAACAGGAACCCCGGCGTCCCCTC

TGTGGTGAAATTCCACCCCTTCACGCCGTGCATCGCCGTAGCCGACAAGGACAGCATCTGCTTTTGGGAC

TGGGAGAAAGGGGAGAAGCTGGATTATTTCCACAATGGGAACCCTCGGTACACGAGGGTCACTGCCATGG

AGTATCTGAACGGCCAGGACTGCTCGCTTCTGCTGACGGCCACAGACGATGGTGCCATCAGGGTCTGGAA

GAATTTTGCTGATTTGGAAAAGAACCCAGAGATGGTGACCGCGTGGCAGGGGCTCTCGGACATGCTGCCA

ACGACGCGAGGAGCTGGGATGGTGGTGGACTGGGAGCAGGAGACCGGCCTCCTCATGAGCTCAGGAGACG

TGCGGATCGTCCGGATCTGGGACACAGACCGTGAGATGAAGGTGCAGGACATCCCTACGGGCGCAGACAG

CTGTGTGACGAGTCTGTCCTGTGATTCCCACCGCTCACTCATCGTGGCTGGCCTCGGTGACGGCTCCATC

CGCGTCTACGACAGAAGGATGGCACTCAGCGAATGCCGCGTCATGACGTACCGGGAGCACACAGCCTGGG

TGGTGAAGGCCTCCCTGCAGAAGCGTCCCGACGGCCACATCGTGAGTGTGAGCGTCAATGGAGATGTGCG

CATCTTTGATCCCCGGATGCCTGAGTCGGTAAATGTGCTTCAGATCGTGAAGGGGCTGACGGCCCTGGAC

ATCCACCCCCAGGCGGACCTGATCGCATGTGGCTCCGTCAATCAGTTCACCGCCATCTACAACAGCAGCG

GAGAGCTCATCAACAACATCAAGTACTACGACGGCTTCATGGGCCAGCGGGTCGGCGCCATCAGCTGCCT

GGCCTTCCACCCGCACTGGCCTCACCTGGCCGTGGGAAGCAACGACTACTACATCTCCGTGTACTCGGTG

GAGAAGCGTGTCAGATAG

>lcl|NM_001145642.5_cds_NP_001139114.1_1 [gene=RUBCN] [db_xref=CCDS:CCDS46987.1] [protein=run domain Beclin-1-interacting and cysteine-rich domain-containing protein isoform 1] [protein_id=NP_001139114.1] [location=668..3451] [gbkey=CDS]

ATGCAGAGCATCCTCTATCACGGGCTTATCCGTGACCAGGCGTGCCGCCGCCAGACGGATTACTGGCAGT

TCGTGAAAGACATCCGGTGGCTCAGTCCCCACTCAGCCCTTCACGTGGAGAAGTTCATCAGCGTGCACGA

GAACGACCAGAGCAGTGCTGATGGTGCCAGTGAACGTGCTGTTGCCGAGCTGTGGCTGCAGCACAGCCTG

CAGTACCACTGCCTCTCAGCCCAGCTCCGGCCCCTGCTCGGGGATAGACAGTATATCAGAAAATTCTACA

CAGATGCTGCCTTCCTGCTAAGTGACGCTCATGTCACGGCCATGCTGCAGTGCCTGGAAGCAGTGGAACA

GAACAACCCCCGCCTCCTGGCTCAGATCGATGCGTCCATGTTTGCCAGAAAGCACGAGAGCCCGCTCCTG

GTGACAAAGAGCCAGAGCCTGACAGCCCTGCCCAGTTCCACATACACCCCTCCAAACAGCTATGCTCAGC

ATTCCTACTTTGGGTCCTTCTCTAGCCTCCACCAATCCGTGCCCAACAATGGCTCAGAGAGAAGATCTAC

TTCCTTTCCACTCTCTGGCCCTCCCCGGAAACCTCAAGAAAGCAGAGGGCACGTCTCACCAGCAGAGGAT

CAAACCATCCAAGCCCCCCCAGTTTCAGTCTCTGCACTAGCCAGGGATTCCCCTTTGACCCCAAATGAAA

TGAGCTCCAGTACTCTGACCAGCCCCATAGAGGCATCCTGGGTCAGCAGCCAGAATGATTCCCCAGGTGA

TGCCAGTGAGGGGCCTGAGTACCTGGCCATTGGCAACTTGGACCCCCGAGGCCGGACTGCCAGCTGTCAG

AGTCACAGCAGCAATGCCGAGAGCAGCAGTTCCAATTTGTTCTCCTCCAGCAGCTCCCAGAAGCCAGATT

CTGCTGCCTCTTCCTTAGGGGACCAGGAAGGAGGTGGGGAGAGCCAGCTGTCCAGTGTCCTCCGCAGGTC

CAGCTTCTCAGAGGGGCAGACACTCACTGTCACCAGTGGGGCAAAGAAAAGCCACATTCGCTCCCATTCG

GATACCAGCATTGCCTCCAGGGGAGCTCCAGGAGGCCCCAGGAATATCACCATTATAGTTGAAGATCCCA

TTGCAGAATCCTGCAATGATAAGGCGAAGTTGAGAGGCCCTTTGCCCTACTCTGGTCAAAGCAGTGAAGT

CAGCACACCCAGCTCTCTGTACATGGAATATGAAGGTGGTCGGTACCTGTGCTCAGGGGAAGGCATGTTC

CGAAGACCATCAGAAGGACAGTCCCTCATCAGCTACCTCTCTGAGCAAGACTTCGGCAGCTGTGCCGACC

TGGAAAAGGAGAATGCCCACTTCAGCATCTCAGAGTCCTTAATTGCTGCCATCGAGCTAATGAAGTGCAA

CATGATGAGCCAGTGCCTAGAGGAGGAGGAAGTGGAAGAGGAAGACAGTGATAGAGAGATCCAGGAGCTG

AAGCAGAAGATCCGCCTTCGGCGCCAGCAAATCCGCACCAAGAACCTGCTCCCCATGTACCAGGAGGCTG

AGCACGGAAGCTTTCGGGTCACCTCCAGCAGCTCCCAGTTCAGCTCACGTGATTCGGCACAGCTCTCTGA

CTCTGGCTCTGCTGATGAGGTTGATGAATTTGAAATCCAAGATGCTGACATCAGAAGGAACACAGCCTCA

AGCAGCAAATCCTTCGTTTCCTCCCAGTCCTTCTCCCACTGCTTCCTGCACTCCACGTCTGCTGAGGCGG

TGGCCATGGGGCTCCTGAAGCAGTTTGAGGGGATGCAGCTTCCAGCCGCCTCGGAGCTGGAGTGGCTTGT

CCCGGAGCATGATGCCCCTCAGAAGCTCCTGCCCATTCCTGACTCACTGCCCATCTCACCGGATGACGGG

CAGCACGCTGACATCTACAAGCTGCGGATTCGTGTTCGTGGCAACTTGGAGTGGGCCCCGCCCCGGCCTC

AGATAATTTTTAATGTTCATCCAGCCCCAACGAGGAAAATTGCCGTGGCCAAGCAGAATTACCGCTGTGC

AGGATGTGGCATCCGGACTGACCCTGATTACATCAAGCGACTGCGGTACTGTGAGTACCTGGGCAAGTAC

TTCTGCCAGTGCTGCCACGAGAATGCCCAGATGGCCATCCCCAGCCGGGTTCTGCGCAAGTGGGACTTCA

GCAAGTACTACGTCAGCAACTTCTCCAAGGACCTGCTCATTAAGATCTGGAATGATCCTCTCTTCAACGT

GCAGGACATAAACAGTGCCCTCTATAGGAAGGTCAAGCTGCTCAATCAAGTCCGGCTGCTGCGGGTCCAG

CTGTGTCACATGAAGAACATGTTCAAGACTTGCCGACTGGCCAAGGAGCTTCTGGATTCCTTTGACACAG

TCCCAGGCCACCTGACAGAGGACCTCCACCTGTACTCACTGAATGACCTGACTGCGACCAGGAAGGGGGA

GCTGGGGCCCCGGCTTGCTGAGCTCACCAGGGCAGGGGCTACCCATGTGGAGAGATGCATGCTCTGCCAA

GCCAAAGGCTTCATCTGTGAGTTCTGTCAGAATGAGGATGACATCATCTTTCCCTTTGAGCTCCATAAGT

GCCGGACCTGTGAAGAGTGTAAAGCGTGTTACCATAAAGCCTGCTTCAAGTCTGGAAGCTGTCCGCGCTG

CGAGCGGCTGCAGGCCCGGCGGGAGGCACTGGCCAGGCAGAGCCTGGAGTCTTACCTGTCAGACTACGAG

GAGGAGCCCGCGGAAGCGCTGGCCCTGGAAGCCGCCGTCCTGGAGGCCACCTGA

>lcl|NM_001142648.2_cds_NP_001136120.1_1 [gene=SAR1A] [db_xref=CCDS:CCDS7298.1] [protein=GTP-binding protein SAR1a] [protein_id=NP_001136120.1] [location=189..785] [gbkey=CDS]

ATGTCTTTCATCTTTGAGTGGATCTACAATGGCTTCAGCAGTGTGCTCCAGTTCCTAGGACTGTACAAGA

AATCTGGAAAACTTGTATTCTTAGGTTTGGATAATGCAGGCAAAACCACTCTTCTTCACATGCTCAAAGA

TGACAGATTGGGCCAACATGTTCCAACACTACATCCGACATCAGAAGAGCTAACAATTGCTGGAATGACC

TTTACAACTTTTGATCTTGGTGGGCACGAGCAAGCACGTCGCGTTTGGAAAAATTATCTCCCAGCAATTA

ATGGGATTGTCTTTCTGGTGGACTGTGCAGATCATTCTCGCCTCGTGGAATCCAAAGTTGAGCTTAATGC

TTTAATGACTGATGAAACAATATCCAATGTGCCAATCCTTATCTTGGGTAACAAAATTGACAGAACAGAT

GCAATCAGTGAAGAAAAACTCCGTGAGATATTTGGGCTTTATGGACAGACCACAGGAAAGGGGAATGTGA

CCCTGAAGGAGCTGAATGCTCGCCCCATGGAAGTGTTCATGTGCAGTGTGCTCAAGAGGCAAGGTTACGG

CGAGGGTTTCCGCTGGCTCTCCCAGTATATTGACTGA

>lcl|NM_001002235.3_cds_NP_001002235.1_1 [gene=SERPINA1] [db_xref=CCDS:CCDS9925.1] [protein=alpha-1-antitrypsin precursor] [protein_id=NP_001002235.1] [location=184..1440] [gbkey=CDS]

ATGCCGTCTTCTGTCTCGTGGGGCATCCTCCTGCTGGCAGGCCTGTGCTGCCTGGTCCCTGTCTCCCTGG

CTGAGGATCCCCAGGGAGATGCTGCCCAGAAGACAGATACATCCCACCATGATCAGGATCACCCAACCTT

CAACAAGATCACCCCCAACCTGGCTGAGTTCGCCTTCAGCCTATACCGCCAGCTGGCACACCAGTCCAAC

AGCACCAATATCTTCTTCTCCCCAGTGAGCATCGCTACAGCCTTTGCAATGCTCTCCCTGGGGACCAAGG

CTGACACTCACGATGAAATCCTGGAGGGCCTGAATTTCAACCTCACGGAGATTCCGGAGGCTCAGATCCA

TGAAGGCTTCCAGGAACTCCTCCGTACCCTCAACCAGCCAGACAGCCAGCTCCAGCTGACCACCGGCAAT

GGCCTGTTCCTCAGCGAGGGCCTGAAGCTAGTGGATAAGTTTTTGGAGGATGTTAAAAAGTTGTACCACT

CAGAAGCCTTCACTGTCAACTTCGGGGACACCGAAGAGGCCAAGAAACAGATCAACGATTACGTGGAGAA

GGGTACTCAAGGGAAAATTGTGGATTTGGTCAAGGAGCTTGACAGAGACACAGTTTTTGCTCTGGTGAAT

TACATCTTCTTTAAAGGCAAATGGGAGAGACCCTTTGAAGTCAAGGACACCGAGGAAGAGGACTTCCACG

TGGACCAGGTGACCACCGTGAAGGTGCCTATGATGAAGCGTTTAGGCATGTTTAACATCCAGCACTGTAA

GAAGCTGTCCAGCTGGGTGCTGCTGATGAAATACCTGGGCAATGCCACCGCCATCTTCTTCCTGCCTGAT

GAGGGGAAACTACAGCACCTGGAAAATGAACTCACCCACGATATCATCACCAAGTTCCTGGAAAATGAAG

ACAGAAGGTCTGCCAGCTTACATTTACCCAAACTGTCCATTACTGGAACCTATGATCTGAAGAGCGTCCT

GGGTCAACTGGGCATCACTAAGGTCTTCAGCAATGGGGCTGACCTCTCCGGGGTCACAGAGGAGGCACCC

CTGAAGCTCTCCAAGGCCGTGCATAAGGCTGTGCTGACCATCGACGAGAAAGGGACTGAAGCTGCTGGGG

CCATGTTTTTAGAGGCCATACCCATGTCTATCCCCCCCGAGGTCAAGTTCAACAAACCCTTTGTCTTCTT

AATGATTGAACAAAATACCAAGTCTCCCCTCTTCATGGGAAAAGTGGTGAATCCCACCCAAAAATAA

>lcl|NM_031459.5_cds_NP_113647.1_1 [gene=SESN2] [db_xref=CCDS:CCDS321.1] [protein=sestrin-2] [protein_id=NP_113647.1] [location=331..1773] [gbkey=CDS]

ATGATCGTGGCGGACTCCGAGTGCCGCGCAGAGCTCAAGGACTACCTGCGGTTCGCCCCGGGCGGCGTCG

GCGACTCGGGCCCCGGAGAGGAGCAGAGGGAGAGCCGGGCTCGGCGAGGCCCTCGAGGGCCCAGCGCCTT

CATCCCCGTGGAGGAGGTCCTTCGGGAGGGGGCTGAGAGCCTCGAGCAGCACCTGGGGCTGGAGGCACTG

ATGTCCTCTGGGCGAGTAGACAACCTGGCAGTGGTGATGGGCCTGCACCCTGACTACTTTACCAGCTTCT

GGCGCCTGCACTACCTGCTGCTGCACACGGATGGTCCCTTGGCCAGCTCCTGGCGCCACTACATTGCCAT

CATGGCTGCCGCCCGCCATCAGTGTTCTTACCTGGTAGGCTCCCACATGGCCGAGTTTCTGCAGACTGGT

GGTGACCCTGAGTGGCTGCTGGGCCTCCACCGGGCCCCCGAGAAGCTGCGCAAACTCAGCGAGATCAACA

AGTTGCTGGCGCATCGGCCATGGCTCATCACCAAGGAACACATCCAGGCCTTGCTGAAGACCGGCGAGCA

CACTTGGTCCCTGGCCGAGCTCATTCAGGCTCTGGTCCTGCTCACCCACTGCCACTCGCTCTCCTCCTTC

GTGTTTGGCTGTGGCATCCTCCCTGAGGGGGATGCAGATGGCAGCCCTGCCCCCCAGGCACCTACACCCC

CTAGTGAACAGAGCAGCCCCCCAAGCAGGGACCCGTTGAACAACTCTGGGGGCTTTGAGTCTGCCCGCGA

CGTGGAGGCGCTGATGGAGCGCATGCAGCAGCTGCAGGAGAGCCTGCTGCGGGATGAGGGGACGTCCCAG

GAGGAGATGGAGAGCCGCTTTGAGCTGGAGAAGTCAGAGAGCCTGCTGGTGACCCCCTCAGCTGACATCC

TGGAGCCCTCTCCACACCCAGACATGCTGTGCTTTGTGGAAGACCCTACTTTCGGATATGAGGACTTCAC

TCGGAGAGGGGCTCAGGCACCCCCTACCTTCCGGGCCCAGGATTATACCTGGGAAGACCATGGCTACTCG

CTGATCCAGCGGCTTTACCCTGAGGGTGGGCAGCTGCTGGATGAGAAGTTCCAGGCAGCCTATAGCCTCA

CCTACAATACCATCGCCATGCACAGTGGTGTGGACACCTCCGTGCTCCGCAGGGCCATCTGGAACTATAT

CCACTGCGTCTTTGGCATCAGATATGATGACTATGATTATGGGGAGGTGAACCAGCTCCTGGAGCGGAAC

CTCAAGGTCTATATCAAGACAGTGGCCTGCTACCCAGAGAAGACCACCCGAAGAATGTACAACCTCTTCT

GGAGGCACTTCCGCCACTCAGAGAAGGTCCACGTGAACTTGCTGCTCCTGGAGGCGCGCATGCAAGCCGC

TCTGCTGTACGCCCTCCGTGCCATCACCCGCTACATGACCTGA

>lcl|NM_016009.5_cds_NP_057093.1_1 [gene=SH3GLB1] [db_xref=CCDS:CCDS710.1] [protein=endophilin-B1 isoform 1] [protein_id=NP_057093.1] [location=325..1422] [gbkey=CDS]

ATGAATATCATGGACTTCAACGTGAAGAAGCTGGCGGCCGACGCAGGCACCTTCCTCAGTCGCGCCGTGC

AGTTCACAGAAGAAAAGCTTGGCCAGGCTGAGAAGACAGAATTGGATGCTCACTTAGAGAACCTCCTTAG

CAAAGCTGAATGTACCAAAATATGGACAGAAAAAATAATGAAACAAACTGAAGTGTTATTGCAGCCAAAT

CCAAATGCCAGGATAGAAGAATTTGTTTATGAGAAACTGGATAGAAAAGCTCCAAGTCGTATAAACAACC

CAGAACTTTTGGGACAATATATGATTGATGCAGGGACTGAGTTTGGCCCAGGAACAGCTTATGGTAATGC

CCTTATTAAATGTGGAGAAACCCAAAAAAGAATTGGAACAGCAGACAGAGAACTGATTCAAACGTCAGCC

TTAAATTTTCTTACTCCTTTAAGAAACTTTATAGAAGGAGATTACAAAACAATTGCTAAAGAAAGGAAAC

TATTGCAAAATAAGAGACTGGATTTGGATGCTGCAAAAACGAGACTAAAAAAGGCAAAAGCTGCAGAAAC

TAGAAATTCATCTGAACAGGAATTAAGAATAACTCAAAGTGAATTTGATCGTCAAGCAGAGATTACCAGA

CTTCTGCTAGAGGGAATCAGCAGTACACATGCCCATCACCTTCGCTGTCTGAATGACTTTGTAGAAGCCC

AGATGACTTACTATGCACAGTGTTACCAGTATATGTTGGACCTCCAGAAACAACTGGGAAGTTTTCCATC

CAATTATCTTAGTAACAACAATCAGACTTCTGTGACACCTGTACCATCAGTTTTACCAAATGCGATTGGT

TCTTCTGCCATGGCTTCAACAAGTGGCCTAGTAATCACCTCTCCTTCCAACCTCAGTGACCTTAAGGAGT

GTAGTGGCAGCAGAAAGGCCAGGGTTCTCTATGATTATGATGCAGCAAACAGTACTGAATTATCACTTCT

GGCAGATGAGGTGATCACTGTGTTCAGTGTTGTTGGAATGGATTCAGACTGGCTAATGGGGGAAAGGGGA

AACCAGAAGGGCAAGGTGCCAATTACCTACTTAGAACTGCTCAATTAA

>lcl|NM_012238.5_cds_NP_036370.2_1 [gene=SIRT1] [db_xref=CCDS:CCDS7273.1] [protein=NAD-dependent protein deacetylase sirtuin-1 isoform a] [protein_id=NP_036370.2] [location=67..2310] [gbkey=CDS]

ATGGCGGACGAGGCGGCCCTCGCCCTTCAGCCCGGCGGCTCCCCCTCGGCGGCGGGGGCCGACAGGGAGG

CCGCGTCGTCCCCCGCCGGGGAGCCGCTCCGCAAGAGGCCGCGGAGAGATGGTCCCGGCCTCGAGCGGAG

CCCGGGCGAGCCCGGTGGGGCGGCCCCAGAGCGTGAGGTGCCGGCGGCGGCCAGGGGCTGCCCGGGTGCG

GCGGCGGCGGCGCTGTGGCGGGAGGCGGAGGCAGAGGCGGCGGCGGCAGGCGGGGAGCAAGAGGCCCAGG

CGACTGCGGCGGCTGGGGAAGGAGACAATGGGCCGGGCCTGCAGGGCCCATCTCGGGAGCCACCGCTGGC

CGACAACTTGTACGACGAAGACGACGACGACGAGGGCGAGGAGGAGGAAGAGGCGGCGGCGGCGGCGATT

GGGTACCGAGATAACCTTCTGTTCGGTGATGAAATTATCACTAATGGTTTTCATTCCTGTGAAAGTGATG

AGGAGGATAGAGCCTCACATGCAAGCTCTAGTGACTGGACTCCAAGGCCACGGATAGGTCCATATACTTT

TGTTCAGCAACATCTTATGATTGGCACAGATCCTCGAACAATTCTTAAAGATTTATTGCCGGAAACAATA

CCTCCACCTGAGTTGGATGATATGACACTGTGGCAGATTGTTATTAATATCCTTTCAGAACCACCAAAAA

GGAAAAAAAGAAAAGATATTAATACAATTGAAGATGCTGTGAAATTACTGCAAGAGTGCAAAAAAATTAT

AGTTCTAACTGGAGCTGGGGTGTCTGTTTCATGTGGAATACCTGACTTCAGGTCAAGGGATGGTATTTAT

GCTCGCCTTGCTGTAGACTTCCCAGATCTTCCAGATCCTCAAGCGATGTTTGATATTGAATATTTCAGAA

AAGATCCAAGACCATTCTTCAAGTTTGCAAAGGAAATATATCCTGGACAATTCCAGCCATCTCTCTGTCA

CAAATTCATAGCCTTGTCAGATAAGGAAGGAAAACTACTTCGCAACTATACCCAGAACATAGACACGCTG

GAACAGGTTGCGGGAATCCAAAGGATAATTCAGTGTCATGGTTCCTTTGCAACAGCATCTTGCCTGATTT

GTAAATACAAAGTTGACTGTGAAGCTGTACGAGGAGATATTTTTAATCAGGTAGTTCCTCGATGTCCTAG

GTGCCCAGCTGATGAACCGCTTGCTATCATGAAACCAGAGATTGTGTTTTTTGGTGAAAATTTACCAGAA

CAGTTTCATAGAGCCATGAAGTATGACAAAGATGAAGTTGACCTCCTCATTGTTATTGGGTCTTCCCTCA

AAGTAAGACCAGTAGCACTAATTCCAAGTTCCATACCCCATGAAGTGCCTCAGATATTAATTAATAGAGA

ACCTTTGCCTCATCTGCATTTTGATGTAGAGCTTCTTGGAGACTGTGATGTCATAATTAATGAATTGTGT

CATAGGTTAGGTGGTGAATATGCCAAACTTTGCTGTAACCCTGTAAAGCTTTCAGAAATTACTGAAAAAC

CTCCACGAACACAAAAAGAATTGGCTTATTTGTCAGAGTTGCCACCCACACCTCTTCATGTTTCAGAAGA

CTCAAGTTCACCAGAAAGAACTTCACCACCAGATTCTTCAGTGATTGTCACACTTTTAGACCAAGCAGCT

AAGAGTAATGATGATTTAGATGTGTCTGAATCAAAAGGTTGTATGGAAGAAAAACCACAGGAAGTACAAA

CTTCTAGGAATGTTGAAAGTATTGCTGAACAGATGGAAAATCCGGATTTGAAGAATGTTGGTTCTAGTAC

TGGGGAGAAAAATGAAAGAACTTCAGTGGCTGGAACAGTGAGAAAATGCTGGCCTAATAGAGTGGCAAAG

GAGCAGATTAGTAGGCGGCTTGATGGTAATCAGTATCTGTTTTTGCCACCAAATCGTTACATTTTCCATG

GCGCTGAGGTATATTCAGACTCTGAAGATGACGTCTTATCCTCTAGTTCTTGTGGCAGTAACAGTGATAG

TGGGACATGCCAGAGTCCAAGTTTAGAAGAACCCATGGAGGATGAAAGTGAAATTGAAGAATTCTACAAT

GGCTTAGAAGATGAGCCTGATGTTCCAGAGAGAGCTGGAGGAGCTGGATTTGGGACTGATGGAGATGATC

AAGAGGCAATTAATGAAGCTATATCTGTGAAACAGGAAGTAACAGACATGAACTATCCATCAAACAAATC

ATAG

>lcl|NM_001193286.2_cds_NP_001180215.1_1 [gene=SIRT2] [db_xref=CCDS:CCDS74361.1] [protein=NAD-dependent protein deacetylase sirtuin-2 isoform 3] [protein_id=NP_001180215.1] [location=162..866] [gbkey=CDS]

ATGGACTTCCTGCGGAACTTATTCTCCCAGACGCTCAGCCTGGGCAGCCAGAAGGAGCGTCTGCTGGACG

AGCTGACCTTGGAAGGGGTGGCCCGGTACATGCAGAGCGAACGCTGTCGCAGAGTCATCTGTTTGGTGGG

AGCTGGAATCTCCACATCCGCAGGCATCCCCGACTTTCGCTCTCCATCCACCGGCCTCTATGACAACCTA

GAGAAGTACCATCTTCCCTACCCAGAGGCCATCTTTGAGATCAGCTATTTCAAGAAACATCCGGAACCCT

TCTTCGCCCTCGCCAAGGAACTCTATCCTGGGCAGTTCAAGCCAACCATCTGTCACTACTTCATGCGCCT

GCTGAAGGACAAGGGGCTACTCCTGCGCTGCTACACGCAGAACATAGATACCCTGGAGCGAATAGCCGGG

CTGGAACAGGAGGACTTGGTGGAGGCGCACGGCACCTTCTACACATCACACTGCGTCAGCGCCAGCTGCC

GGCACGAATACCCGCTAAGCTGGATGAAAGAGAAGATCTTCTCTGAGGTGACGCCCAAGTGTGAAGACTG

TCAGAGCCTGGTGAAGCCTGATATCGTCTTTTTTGGTGAGAGCCTCCCAGCGCGTTTCTTCTCCTGTATG

CAGTCAGACTTCCTGAAGGTGGACCTCCTCCTGGTCATGGGTACCTCCTTGCAGGGACGTGGCCTGGCTG

GGTGA

>lcl|NM_182965.3_cds_NP_892010.2_1 [gene=SPHK1] [db_xref=CCDS:CCDS11744.1] [protein=sphingosine kinase 1 isoform 2] [protein_id=NP_892010.2] [location=498..1910] [gbkey=CDS]

ATGTCCGCTCAAGTTCTGGGATTTTTACGCAGCTGGACTCCCCTCCCCCTGGCAGCCCCGAGGGGTCCAG

CCGCCGCAGGGAATGACGCCGGTGCTCCTGCAGCCACGGCTCCGGGCGGGGAAGGCGAGCCCCACAGCCG

GCCCTGCGACGCCCGCCTGGGCAGCACCGATAAGGAGCTGAAGGCAGGAGCCGCCGCCACGGGCAGCGCC

CCCACAGCGCCAGGGACCCCCTGGCAGCGGGAGCCGCGGGTCGAGGTTATGGATCCAGCGGGCGGCCCCC

GGGGCGTGCTCCCGCGGCCCTGCCGCGTGCTGGTGCTGCTGAACCCGCGCGGCGGCAAGGGCAAGGCCTT

GCAGCTCTTCCGGAGTCACGTGCAGCCCCTTTTGGCTGAGGCTGAAATCTCCTTCACGCTGATGCTCACT

GAGCGGCGGAACCACGCGCGGGAGCTGGTGCGGTCGGAGGAGCTGGGCCGCTGGGACGCTCTGGTGGTCA

TGTCTGGAGACGGGCTGATGCACGAGGTGGTGAACGGGCTCATGGAGCGGCCTGACTGGGAGACCGCCAT

CCAGAAGCCCCTGTGTAGCCTCCCAGCAGGCTCTGGCAACGCGCTGGCAGCTTCCTTGAACCATTATGCT

GGCTATGAGCAGGTCACCAATGAAGACCTCCTGACCAACTGCACGCTATTGCTGTGCCGCCGGCTGCTGT

CACCCATGAACCTGCTGTCTCTGCACACGGCTTCGGGGCTGCGCCTCTTCTCTGTGCTCAGCCTGGCCTG

GGGCTTCATTGCTGATGTGGACCTAGAGAGTGAGAAGTATCGGCGTCTGGGGGAGATGCGCTTCACTCTG

GGCACCTTCCTGCGTCTGGCAGCCCTGCGCACCTACCGCGGCCGACTGGCCTACCTCCCTGTAGGAAGAG

TGGGTTCCAAGACACCTGCCTCCCCCGTTGTGGTCCAGCAGGGCCCGGTAGATGCACACCTTGTGCCACT

GGAGGAGCCAGTGCCCTCTCACTGGACAGTGGTGCCCGACGAGGACTTTGTGCTAGTCCTGGCACTGCTG

CACTCGCACCTGGGCAGTGAGATGTTTGCTGCACCCATGGGCCGCTGTGCAGCTGGCGTCATGCATCTGT

TCTACGTGCGGGCGGGAGTGTCTCGTGCCATGCTGCTGCGCCTCTTCCTGGCCATGGAGAAGGGCAGGCA

TATGGAGTATGAATGCCCCTACTTGGTATATGTGCCCGTGGTCGCCTTCCGCTTGGAGCCCAAGGATGGG

AAAGGTGTGTTTGCAGTGGATGGGGAATTGATGGTTAGCGAGGCCGTGCAGGGCCAGGTGCACCCAAACT

ACTTCTGGATGGTCAGCGGTTGCGTGGAGCCCCCGCCCAGCTGGAAGCCCCAGCAGATGCCACCGCCAGA

AGAGCCCTTATGA

>lcl|NM_032038.3_cds_NP_114427.1_1 [gene=SPNS1] [db_xref=CCDS:CCDS10646.1] [protein=protein spinster homolog 1 isoform 1] [protein_id=NP_114427.1] [location=375..1961] [gbkey=CDS]

ATGGCCGGGTCCGACACCGCGCCCTTCCTCAGCCAGGCGGATGACCCGGACGACGGGCCAGTGCCTGGCA

CCCCGGGGTTGCCAGGGTCCACGGGGAACCCGAAGTCCGAGGAGCCCGAGGTCCCGGACCAGGAGGGGCT

GCAGCGCATCACCGGCCTGTCTCCCGGCCGTTCGGCTCTCATAGTGGCGGTGCTGTGCTACATCAATCTC

CTGAACTACATGGACCGCTTCACCGTGGCTGGCGTCCTTCCCGACATCGAGCAGTTCTTCAACATCGGGG

ACAGTAGCTCTGGGCTCATCCAGACCGTGTTCATCTCCAGTTACATGGTGTTGGCACCTGTGTTTGGCTA

CCTGGGTGACAGGTACAATCGGAAGTATCTCATGTGCGGGGGCATTGCCTTCTGGTCCCTGGTGACACTG

GGGTCATCCTTCATCCCCGGAGAGCATTTCTGGCTGCTCCTCCTGACCCGGGGCCTGGTGGGGGTCGGGG

AGGCCAGTTATTCCACCATCGCGCCCACTCTCATTGCCGACCTCTTTGTGGCCGACCAGCGGAGCCGGAT

GCTCAGCATCTTCTACTTTGCCATTCCGGTGGGCAGTGGTCTGGGCTACATTGCAGGCTCCAAAGTGAAG

GATATGGCTGGAGACTGGCACTGGGCTCTGAGGGTGACACCGGGTCTAGGAGTGGTGGCCGTTCTGCTGC

TGTTCCTGGTAGTGCGGGAGCCGCCAAGGGGAGCCGTGGAGCGCCACTCAGATTTGCCACCCCTGAACCC

CACCTCGTGGTGGGCAGATCTGAGGGCTCTGGCAAGAAATCCTAGTTTCGTCCTGTCTTCCCTGGGCTTC

ACTGCTGTGGCCTTTGTCACGGGCTCCCTGGCTCTGTGGGCTCCGGCATTCCTGCTGCGTTCCCGCGTGG

TCCTTGGGGAGACCCCACCCTGCCTTCCCGGAGACTCCTGCTCTTCCTCTGACAGTCTCATCTTTGGACT

CATCACCTGCCTGACCGGAGTCCTGGGTGTGGGCCTGGGTGTGGAGATCAGCCGCCGGCTCCGCCACTCC

AACCCCCGGGCTGATCCCCTGGTCTGTGCCACTGGCCTCCTGGGCTCTGCACCCTTCCTCTTCCTGTCCC

TTGCCTGCGCCCGTGGTAGCATCGTGGCCACTTATATTTTCATCTTCATTGGAGAGACCCTCCTGTCCAT

GAACTGGGCCATCGTGGCCGACATTCTGCTGTACGTGGTGATCCCTACCCGACGCTCCACCGCCGAGGCC

TTCCAGATCGTGCTGTCCCACCTGCTGGGTGATGCTGGGAGCCCCTACCTCATTGGCCTGATCTCTGACC

GCCTGCGCCGGAACTGGCCCCCCTCCTTCTTGTCCGAGTTCCGGGCTCTGCAGTTCTCGCTCATGCTCTG

CGCGTTTGTTGGGGCACTGGGCGGCGCAGCCTTCCTGGGCACCGCCATCTTCATTGAGGCCGACCGCCGG

CGGGCACAGCTGCACGTGCAGGGCCTGCTGCACGAAGCAGGGTCCACAGACGACCGGATTGTGGTGCCCC

AGCGGGGCCGCTCCACCCGCGTGCCCGTGGCCAGTGTGCTCATCTGA

>lcl|NM_001142298.2_cds_NP_001135770.1_1 [gene=SQSTM1] [db_xref=CCDS:CCDS47355.1] [protein=sequestosome-1 isoform 2] [protein_id=NP_001135770.1] [location=356..1426] [gbkey=CDS]

ATGGCCATGTCCTACGTGAAGGATGACATCTTCCGAATCTACATTAAAGAGAAAAAAGAGTGCCGGCGGG

ACCACCGCCCACCGTGTGCTCAGGAGGCGCCCCGCAACATGGTGCACCCCAATGTGATCTGCGATGGCTG

CAATGGGCCTGTGGTAGGAACCCGCTACAAGTGCAGCGTCTGCCCAGACTACGACTTGTGTAGCGTCTGC

GAGGGAAAGGGCTTGCACCGGGGGCACACCAAGCTCGCATTCCCCAGCCCCTTCGGGCACCTGTCTGAGG

GCTTCTCGCACAGCCGCTGGCTCCGGAAGGTGAAACACGGACACTTCGGGTGGCCAGGATGGGAAATGGG

TCCACCAGGAAACTGGAGCCCACGTCCTCCTCGTGCAGGGGAGGCCCGCCCTGGCCCCACGGCAGAATCA

GCTTCTGGTCCATCGGAGGATCCGAGTGTGAATTTCCTGAAGAACGTTGGGGAGAGTGTGGCAGCTGCCC

TTAGCCCTCTGGGCATTGAAGTTGATATCGATGTGGAGCACGGAGGGAAAAGAAGCCGCCTGACCCCCGT

CTCTCCAGAGAGTTCCAGCACAGAGGAGAAGAGCAGCTCACAGCCAAGCAGCTGCTGCTCTGACCCCAGC

AAGCCGGGTGGGAATGTTGAGGGCGCCACGCAGTCTCTGGCGGAGCAGATGAGGAAGATCGCCTTGGAGT

CCGAGGGGCGCCCTGAGGAACAGATGGAGTCGGATAACTGTTCAGGAGGAGATGATGACTGGACCCATCT

GTCTTCAAAAGAAGTGGACCCGTCTACAGGTGAACTCCAGTCCCTACAGATGCCAGAATCCGAAGGGCCA

AGCTCTCTGGACCCCTCCCAGGAGGGACCCACAGGGCTGAAGGAAGCTGCCTTGTACCCACATCTCCCGC

CAGAGGCTGACCCGCGGCTGATTGAGTCCCTCTCCCAGATGCTGTCCATGGGCTTCTCTGATGAAGGCGG

CTGGCTCACCAGGCTCCTGCAGACCAAGAACTATGACATCGGAGCGGCTCTGGACACCATCCAGTATTCA

AAGCATCCCCCGCCGTTGTGA

>lcl|NM_003932.5_cds_NP_003923.2_1 [gene=ST13] [db_xref=CCDS:CCDS14006.1] [protein=hsc70-interacting protein isoform 1] [protein_id=NP_003923.2] [location=100..1209] [gbkey=CDS]

ATGGACCCCCGCAAAGTGAACGAGCTTCGGGCCTTTGTGAAAATGTGTAAGCAGGATCCGAGCGTTCTGC

ACACCGAGGAAATGCGCTTCCTGAGGGAGTGGGTGGAGAGCATGGGTGGTAAAGTACCACCTGCTACTCA

GAAAGCTAAATCAGAAGAAAATACCAAGGAAGAAAAACCTGATAGTAAGAAGGTGGAGGAAGACTTAAAG

GCAGACGAACCATCAAGTGAGGAAAGTGATCTAGAAATTGATAAAGAAGGTGTGATTGAACCAGACACTG

ATGCTCCTCAAGAAATGGGAGATGAAAATGCGGAGATAACGGAGGAGATGATGGATCAGGCAAATGATAA

AAAAGTGGCTGCTATTGAAGCCCTAAATGATGGTGAACTCCAGAAAGCCATTGACTTATTCACAGATGCC

ATCAAGCTGAATCCTCGCTTGGCCATTTTGTATGCCAAGAGGGCCAGTGTCTTCGTCAAATTACAGAAGC

CAAATGCTGCCATCCGAGACTGTGACAGAGCCATTGAAATAAATCCTGATTCAGCTCAGCCTTACAAGTG

GCGGGGGAAAGCACACAGACTTCTAGGCCACTGGGAAGAAGCAGCCCATGATCTTGCCCTTGCCTGTAAA

TTGGATTATGATGAAGATGCTAGTGCAATGCTGAAAGAAGTTCAACCTAGGGCACAGAAAATTGCAGAAC

ATCGGAGAAAGTATGAGCGAAAACGTGAAGAGCGAGAGATCAAAGAAAGAATAGAACGAGTTAAGAAGGC

TCGAGAAGAGCATGAGAGAGCCCAGAGGGAGGAAGAAGCCAGACGACAGTCAGGAGCTCAGTATGGCTCT

TTTCCAGGTGGCTTTCCTGGGGGAATGCCTGGTAATTTTCCCGGAGGAATGCCTGGAATGGGAGGGGGCA

TGCCTGGAATGGCTGGAATGCCTGGACTCAATGAAATTCTTAGTGATCCAGAGGTTCTTGCAGCCATGCA

GGATCCAGAAGTTATGGTGGCTTTCCAGGATGTGGCTCAGAACCCAGCAAATATGTCAAAATACCAGAGC

AACCCAAAGGTTATGAATCTCATCAGTAAATTGTCAGCCAAATTTGGAGGTCAAGCGTAA

>lcl|NM_000455.5_cds_NP_000446.1_1 [gene=STK11] [db_xref=CCDS:CCDS45896.1] [protein=serine/threonine-protein kinase STK11 isoform 1] [protein_id=NP_000446.1] [location=1137..2438] [gbkey=CDS]

ATGGAGGTGGTGGACCCGCAGCAGCTGGGCATGTTCACGGAGGGCGAGCTGATGTCGGTGGGTATGGACA

CGTTCATCCACCGCATCGACTCCACCGAGGTCATCTACCAGCCGCGCCGCAAGCGGGCCAAGCTCATCGG

CAAGTACCTGATGGGGGACCTGCTGGGGGAAGGCTCTTACGGCAAGGTGAAGGAGGTGCTGGACTCGGAG

ACGCTGTGCAGGAGGGCCGTCAAGATCCTCAAGAAGAAGAAGTTGCGAAGGATCCCCAACGGGGAGGCCA

ACGTGAAGAAGGAAATTCAACTACTGAGGAGGTTACGGCACAAAAATGTCATCCAGCTGGTGGATGTGTT

ATACAACGAAGAGAAGCAGAAAATGTATATGGTGATGGAGTACTGCGTGTGTGGCATGCAGGAAATGCTG

GACAGCGTGCCGGAGAAGCGTTTCCCAGTGTGCCAGGCCCACGGGTACTTCTGTCAGCTGATTGACGGCC

TGGAGTACCTGCATAGCCAGGGCATTGTGCACAAGGACATCAAGCCGGGGAACCTGCTGCTCACCACCGG

TGGCACCCTCAAAATCTCCGACCTGGGCGTGGCCGAGGCACTGCACCCGTTCGCGGCGGACGACACCTGC

CGGACCAGCCAGGGCTCCCCGGCTTTCCAGCCGCCCGAGATTGCCAACGGCCTGGACACCTTCTCCGGCT

TCAAGGTGGACATCTGGTCGGCTGGGGTCACCCTCTACAACATCACCACGGGTCTGTACCCCTTCGAAGG

GGACAACATCTACAAGTTGTTTGAGAACATCGGGAAGGGGAGCTACGCCATCCCGGGCGACTGTGGCCCC

CCGCTCTCTGACCTGCTGAAAGGGATGCTTGAGTACGAACCGGCCAAGAGGTTCTCCATCCGGCAGATCC

GGCAGCACAGCTGGTTCCGGAAGAAACATCCTCCGGCTGAAGCACCAGTGCCCATCCCACCGAGCCCAGA

CACCAAGGACCGGTGGCGCAGCATGACTGTGGTGCCGTACTTGGAGGACCTGCACGGCGCGGACGAGGAC

GAGGACCTCTTCGACATCGAGGATGACATCATCTACACTCAGGACTTCACGGTGCCCGGACAGGTCCCAG

AAGAGGAGGCCAGTCACAATGGACAGCGCCGGGGCCTCCCCAAGGCCGTGTGTATGAACGGCACAGAGGC

GGCGCAGCTGAGCACCAAATCCAGGGCGGAGGGCCGGGCCCCCAACCCTGCCCGCAAGGCCTGCTCCGCC

AGCAGCAAGATCCGCCGGCTGTCGGCCTGCAAGCAGCAGTGA

>lcl|NM_017569.4_cds_NP_060039.1_1 [gene=SUPT20H] [db_xref=CCDS:CCDS9362.1] [protein=transcription factor SPT20 homolog isoform b] [protein_id=NP_060039.1] [location=303..2504] [gbkey=CDS]

ATGCAACAAGCTTTAGAACTAGCTTTGGATCGTGCAGAGTATGTCATTGAAAGTGCCCGACAGAGACCTC

CTAAAAGGAAATACCTATCAAGTGGAAGAAAATCTGTATTTCAAAAACTTTATGACTTGTATATTGAAGA

ATGTGAAAAAGAACCTGAAGTTAAGCAGAAATTAAGAAGAAATGTGAACTTGTTAGAGAAGCTTGTTATG

CAAGAGACTTTGTCATGTTTAGTGGTCAATCTATACCCAGGAAATGAGGGATATTCTCTGATGCTCAGGG

GAAAAAACGGATCAGATTCCGAGACCATTCGACTGCCCTATGAAGAAGGAGAGTTGCTTGAATATTTGGA

TGCAGAAGAATTACCTCCTATTTTGGTTGATCTCCTAGAAAAATCTCAGGTTAATATTTTTCATTGCGGA

TGTGTCATAGCAGAAATACGTGACTACAGGCAGTCCAGTAACATGAAATCTCCTGGTTACCAAAGTCGGC

ACATTCTCTTACGTCCAACAATGCAGACTTTAATTTGTGATGTACATTCAATAACAAGTGATAACCACAA

ATGGACCCAGGAAGACAAACTTTTGCTTGAGAGCCAGCTCATCCTAGCTACAGCTGAACCACTCTGTCTT

GATCCTTCTATAGCAGTCACCTGCACTGCAAACAGACTGCTCTATAACAAGCAAAAGATGAACACTCGCC

CAATGAAACGGTGTTTCAAGAGGTATTCCAGATCCTCTCTGAATCGGCAGCAAGATCTATCTCATTGTCC

ACCTCCTCCTCAGCTGAGGTTACTTGATTTCTTACAAAAAAGAAAGGAAAGAAAAGCAGGTCAGCATTAT

GACCTCAAAATTTCTAAGGCAGGAAATTGTGTAGATATGTGGAAACGGAGTCCCTGTAATTTGGCCATAC

CTTCTGAAGTAGATGTGGAGAAATATGCTAAAGTGGAAAAGTCTATCAAATCTGATGACTCACAGCCAAC

AGTCTGGCCAGCCCATGATGTAAAAGATGATTATGTATTTGAATGTGAAGCTGGTACTCAGTATCAGAAA

ACAAAGCTGACCATCTTGCAGTCGCTTGGAGATCCACTTTACTATGGTAAAATACAGCCATGTAAAGCAG

ATGAAGAAAGTGACAGCCAGATGTCTCCATCACACTCGTCCACAGATGATCATTCAAATTGGTTCATTAT

TGGATCAAAGACCGATGCTGAGAGGGTAGTCAATCAGTACCAAGAATTAGTCCAGAATGAAGCCAAATGT

CCGGTCAAGATGTCACACAGCTCCAGTGGCTCAGCCAGTCTGAGTCAGGTTTCTCCAGGGAAAGAAACAG

ATCAAACTGAAACCGTGTCAGTTCAGTCTTCGGTATTGGGGAAGGGTGTAAAACATCGACCCCCACCAAT

CAAACTTCCCTCAAGCTCAGGAAATAGTTCCTCAGGTAACTATTTTACACCACAACAGACAAGCAGCTTT

CTCAAATCTCCAACTCCTCCTCCTTCTTCTAAGCCATCAAGTATTCCTCGGAAATCATCTGTGGATCTCA

ATCAAGTTAGCATGCTTTCTCCAGCTGCCCTATCACCTGCCAGCTCATCACAAAGAACCACGGCCACCCA

GGTCATGGCAAACTCTGCTGGACTTAACTTCATCAATGTAGTGGGCTCTGTTTGTGGGGCCCAGGCTTTG

ATGAGTGGTTCAAACCCCATGCTGGGCTGTAACACTGGTGCCATAACTCCTGCAGGAATAAACCTGAGCG

GCCTTCTACCCTCAGGAGGTCTGCTACCAAATGCACTGCCCAGTGCAATGCAGGCAGCTTCTCAAGCAGG

TGTTCCATTTGGTTTAAAAAATACTTCAAGTCTCAGGCCCTTAAATCTACTCCAGCTTCCAGGTGGTTCA

CTTATTTTTAACACTCTGCAGCAGCAGCAACAGCAGCTCTCCCAGTTTACACCACAACAACCTCAGCAGC

CCACAACTTGTAGTCCTCAACAGCCAGGGGAGCAGGGTTCTGAGCAAGGTTCAACCAGTCAAGAACAGGC

CTTATCTGCTCAGCAAGCTGCTGTTATTAACCTTACTGGAGTAGGAAGTTTTATGCAGTCACAGGCAGCT

GCAGTTGCGATTCTTGCAGCATCAAATGGCTATGGCAGCAGCAGCAGCACAAACAGCTCAGCTACATCAT

CATCGGCATACAGGCAGCCAGTCAAAAAGTAA

>lcl|NM_013254.4_cds_NP_037386.1_1 [gene=TBK1] [db_xref=CCDS:CCDS8968.1] [protein=serine/threonine-protein kinase TBK1] [protein_id=NP_037386.1] [location=100..2289] [gbkey=CDS]

ATGCAGAGCACTTCTAATCATCTGTGGCTTTTATCTGATATTTTAGGCCAAGGAGCTACTGCAAATGTCT

TTCGTGGAAGACATAAGAAAACTGGTGATTTATTTGCTATCAAAGTATTTAATAACATAAGCTTCCTTCG

TCCAGTGGATGTTCAAATGAGAGAATTTGAAGTGTTGAAAAAACTCAATCACAAAAATATTGTCAAATTA

TTTGCTATTGAAGAGGAGACAACAACAAGACATAAAGTACTTATTATGGAATTTTGTCCATGTGGGAGTT

TATACACTGTTTTAGAAGAACCTTCTAATGCCTATGGACTACCAGAATCTGAATTCTTAATTGTTTTGCG

AGATGTGGTGGGTGGAATGAATCATCTACGAGAGAATGGTATAGTGCACCGTGATATCAAGCCAGGAAAT

ATCATGCGTGTTATAGGGGAAGATGGACAGTCTGTGTACAAACTCACAGATTTTGGTGCAGCTAGAGAAT

TAGAAGATGATGAGCAGTTTGTTTCTCTGTATGGCACAGAAGAATATTTGCACCCTGATATGTATGAGAG
AGCAGTGCTAAGAAAAGATCATCAGAAGAAATATGGAGCAACAGTTGATCTTTGGAGCATTGGGGTAACA
TTTTACCATGCAGCTACTGGATCACTGCCATTTAGACCCTTTGAAGGGCCTCGTAGGAATAAAGAAGTGA
TGTATAAAATAATTACAGGAAAGCCTTCTGGTGCAATATCTGGAGTACAGAAAGCAGAAAATGGACCAAT
TGACTGGAGTGGAGACATGCCTGTTTCTTGCAGTCTTTCTCGGGGTCTTCAGGTTCTACTTACCCCTGTT
CTTGCAAACATCCTTGAAGCAGATCAGGAAAAGTGTTGGGGTTTTGACCAGTTTTTTGCAGAAACTAGTG
ATATACTTCACCGAATGGTAATTCATGTTTTTTCGCTACAACAAATGACAGCTCATAAGATTTATATTCA
TAGCTATAATACTGCTACTATATTTCATGAACTGGTATATAAACAAACCAAAATTATTTCTTCAAATCAA
GAACTTATCTACGAAGGGCGACGCTTAGTCTTAGAACCTGGAAGGCTGGCACAACATTTCCCTAAAACTA
CTGAGGAAAACCCTATATTTGTAGTAAGCCGGGAACCTCTGAATACCATAGGATTAATATATGAAAAAAT
TTCCCTCCCTAAAGTACATCCACGTTATGATTTAGACGGGGATGCTAGCATGGCTAAGGCAATAACAGGG
GTTGTGTGTTATGCCTGCAGAATTGCCAGTACCTTACTGCTTTATCAGGAATTAATGCGAAAGGGGATAC
GATGGCTGATTGAATTAATTAAAGATGATTACAATGAAACTGTTCACAAAAAGACAGAAGTTGTGATCAC
ATTGGATTTCTGTATCAGAAACATTGAAAAAACTGTGAAAGTATATGAAAAGTTGATGAAGATCAACCTG
GAAGCGGCAGAGTTAGGTGAAATTTCAGACATACACACCAAATTGTTGAGACTTTCCAGTTCTCAGGGAA
CAATAGAAACCAGTCTTCAGGATATCGACAGCAGATTATCTCCAGGTGGATCACTGGCAGACGCATGGGC
ACATCAAGAAGGCACTCATCCGAAAGACAGAAATGTAGAAAAACTACAAGTCCTGTTAAATTGCATGACA
GAGATTTACTATCAGTTCAAAAAAGACAAAGCAGAACGTAGATTAGCTTATAATGAAGAACAAATCCACA
AATTTGATAAGCAAAAACTGTATTACCATGCCACAAAAGCTATGACGCACTTTACAGATGAATGTGTTAA
AAAGTATGAGGCATTTTTGAATAAGTCAGAAGAATGGATAAGAAAGATGCTTCATCTTAGGAAACAGTTA
TTATCGCTGACTAATCAGTGTTTTGATATTGAAGAAGAAGTATCAAAATATCAAGAATATACTAATGAGT
TACAAGAAACTCTGCCTCAGAAAATGTTTACAGCTTCCAGTGGAATCAAACATACCATGACCCCAATTTA
TCCAAGTTCTAACACATTAGTAGAAATGACTCTTGGTATGAAGAAATTAAAGGAAGAGATGGAAGGGGTG
GTTAAAGAACTTGCTGAAAATAACCACATTTTAGAAAGGTTTGGCTCTTTAACCATGGATGGTGGCCTTC
GCAACGTTGACTGTCTTTAG

>lcl|NM_006405.7_cds_NP_006396.2_1 [gene=TM9SF1] [db_xref=CCDS:CCDS9617.1] [protein=transmembrane 9 superfamily member 1 isoform a precursor] [protein_id=NP_006396.2] [location=114..1934] [gbkey=CDS]

ATGACAGTCGTAGGGAACCCTCGAAGTTGGAGCTGCCAGTGGTTGCCAATCCTGATACTGTTGCTGGGCA

CAGGCCATGGGCCAGGGGTGGAAGGCGTGACACACTACAAGGCCGGCGACCCTGTTATTCTGTATGTCAA

CAAAGTGGGACCCTACCATAACCCTCAGGAAACTTACCACTACTATCAGCTTCCAGTCTGCTGCCCTGAG

AAGATACGTCACAAAAGCCTTAGCCTGGGTGAAGTGCTGGATGGGGACCGAATGGCTGAGTCTTTGTATG

AGATCCGCTTTCGGGAAAACGTGGAGAAGAGAATTCTGTGCCACATGCAGCTCAGTTCTGCACAGGTGGA

GCAGCTGCGCCAGGCCATTGAAGAACTGTACTACTTTGAATTTGTGGTAGATGACTTGCCAATCCGGGGC

TTTGTGGGCTACATGGAGGAGAGTGGTTTCCTGCCACACAGCCACAAGATAGGACTCTGGACCCATTTGG

ACTTCCACCTAGAATTCCATGGAGACCGAATTATATTTGCCAATGTTTCAGTGCGGGACGTCAAGCCCCA

CAGCTTGGATGGGTTACGACCTGACGAGTTCCTAGGCCTTACCCACACTTATAGCGTGCGCTGGTCTGAG

ACTTCAGTGGAGCGTCGGAGTGACAGGCGCCGTGGTGACGATGGTGGTTTCTTTCCTCGAACACTGGAAA

TCCATTGGTTGTCCATCATCAACTCCATGGTGCTTGTGTTTTTACTGGTGGGTTTTGTGGCTGTCATTCT

AATGCGTGTGCTTCGGAATGACCTGGCTCGGTACAACTTAGATGAGGAGACCACCTCTGCAGGTTCTGGT

GATGACTTTGACCAGGGTGACAATGGCTGGAAAATTATCCATACAGATGTCTTCCGCTTCCCCCCATACC

GTGGTCTGCTCTGTGCTGTGCTTGGCGTGGGTGCCCAGTTCCTGGCCCTTGGCACTGGCATTATTGTCAT

GGCACTGCTGGGCATGTTCAATGTGCACCGTCATGGGGCCATTAACTCAGCAGCCATCTTGTTGTATGCC

CTGACCTGCTGCATCTCTGGCTACGTGTCCAGCCACTTCTACCGGCAGATTGGAGGCGAGCGTTGGGTGT

GGAACATCATTCTCACCACCAGTCTCTTCTCTGTGCCTTTCTTCCTGACGTGGAGTGTGGTGAACTCAGT

GCATTGGGCCAATGGTTCGACACAGGCTCTGCCAGCCACAACCATCCTGCTGCTTCTGACGGTTTGGCTG

CTGGTGGGCTTTCCCCTCACTGTCATTGGAGGCATCTTTGGGAAGAACAACGCCAGCCCCTTTGATGCAC

CCTGTCGCACCAAGAACATCGCCCGGGAGATTCCACCCCAGCCCTGGTACAAGTCTACTGTCATCCACAT

GACTGTTGGAGGCTTCCTGCCTTTCAGTGCCATCTCTGTGGAGCTGTACTACATCTTTGCCACAGTATGG

GGTCGGGAGCAGTACACTTTGTACGGCATCCTCTTCTTTGTCTTCGCCATCCTGCTGAGTGTGGGGGCTT

GCATCTCCATTGCACTCACCTACTTCCAGTTGTCTGGGGAGGATTACCGCTGGTGGTGGCGATCTGTGCT

GAGTGTTGGCTCCACCGGCCTCTTCATCTTCCTCTACTCAGTTTTCTATTATGCCCGGCGCTCCAACATG

TCTGGGGCAGTACAGACAGTAGAGTTCTTCGGCTACTCCTTACTCACTGGTTATGTCTTCTTCCTCATGC

TGGGCACCATCTCCTTTTTTTCTTCCCTAAAGTTCATCCGGTATATCTATGTTAACCTCAAGATGGACTG

A

>lcl|NM_001165030.3_cds_NP_001158502.1_1 [gene=TMEM41B] [db_xref=CCDS:CCDS53600.1] [protein=transmembrane protein 41B isoform 2] [protein_id=NP_001158502.1] [location=153..536] [gbkey=CDS]

ATGGCGAAAGGCAGAGTCGCCGAACGATCGCAGTTGGGCGCTCACCACACGACCCCCGTGGGGGACGGGG

CAGCGGGGACGCGGGGTCTCGCGGCGCCTGGCAGCAGAGACCACCAGAAGGAAAAATCCTGGGTAGAAGC

TGGATCAGCAAGAATGTCACTCCTTATATTGGTGTCCATTTTCTTATCTGCAGCTTTTGTTATGTTTTTG

GTATATAAAAATTTTCCTCAGCTTAGTGAAGAAGAAAGAGTGAATATGAAGGTTCCCAGAGATATGGATG

ATGCCAAGGCTCTAGGAAAAGTTTTATCCAAATACAAGGACACCTTTTATGTTCAAGTACTTGTAGCTTA

TTTTGCTACATATATTTTGTATCCTTTTAACTGA

>lcl|NM_153015.3_cds_NP_694560.1_1 [gene=TMEM74] [db_xref=CCDS:CCDS6310.1] [protein=transmembrane protein 74] [protein_id=NP_694560.1] [location=159..1076] [gbkey=CDS]

ATGGAGCTCCACTACCTTGCTAAGAAGAGCAACCAGGCAGACCTCTGTGATGCCAGGGACTGGAGTTCAA

GAGGGCTGCCTGGTGACCAGGCAGATACAGCAGCCACAAGAGCTGCTCTCTGCTGTCAGAAACAGTGTGC

ATCCACCCCAAGAGCAACCGAGATGGAAGGGTCTAAACTTAGTTCTTCTCCAGCATCCCCCTCCTCCTCT

CTGCAAAACAGTACTCTTCAGCCAGATGCCTTTCCACCAGGACTTCTCCACTCAGGGAACAACCAAATAA

CAGCGGAACGGAAAGTCTGTAACTGCTGCAGCCAGGAATTAGAAACTTCTTTTACCTATGTGGACAAAAA

CATCAACTTGGAGCAGCGGAACCGGAGCTCGCCATCAGCAAAAGGGCATAATCACCCTGGGGAGCTTGGC

TGGGAAAATCCAAATGAGTGGTCCCAAGAGGCTGCCATATCTTTGATATCTGAAGAGGAGGATGATACAA

GTTCAGAAGCCACGTCTTCAGGGAAGTCTATAGACTATGGTTTCATCAGCGCCATCTTGTTCTTGGTCAC

TGGGATCCTGCTCGTGATCATCTCTTACATCGTCCCACGGGAAGTGACTGTGGACCCCAACACTGTGGCA

GCCCGGGAGATGGAGCGCCTGGAGAAGGAGAGTGCGAGGCTGGGGGCTCACCTGGACCGCTGTGTGATTG

CGGGGCTCTGCCTCCTCACGCTGGGGGGCGTCATCCTGTCCTGCTTGTTAATGATGTCCATGTGGAAGGG

GGAGCTCTATCGTCGAAACAGATTTGCCTCTTCCAAAGAGTCTGCAAAACTCTATGGTTCTTTCAACTTC

AGGATGAAAACCAGCACGAATGAAAACACTCTGGAACTGTCCTTGGTAGAGGAAGATGCGCTTGCTGTAC

AGAGTTAA

>lcl|NM_001190942.2_cds_NP_001177871.1_1 [gene=TNFSF10] [db_xref=CCDS:CCDS54680.1] [protein=tumor necrosis factor ligand superfamily member 10 isoform 2] [protein_id=NP_001177871.1] [location=47..352] [gbkey=CDS]

ATGGCTATGATGGAGGTCCAGGGGGGACCCAGCCTGGGACAGACCTGCGTGCTGATCGTGATCTTCACAG

TGCTCCTGCAGTCTCTCTGTGTGGCTGTAACTTACGTGTACTTTACCAACGAGCTGAAGCAGATGCAGGA

CAAGTACTCCAAAAGTGGCATTGCTTGTTTCTTAAAAGAAGATGACAGTTATTGGGACCCCAATGACGAA

GAGAGTATGAACAGCCCCTGCTGGCAAGTCAAGTGGCAACTCCGTCAGCTCGTTAGAAAGACTCCAAGAA

TGAAAAGGCTCTGGGCCGCAAAATAA

>lcl|NM_000546.6_cds_NP_000537.3_1 [gene=TP53] [db_xref=CCDS:CCDS11118.1] [protein=cellular tumor antigen p53 isoform a] [protein_id=NP_000537.3] [location=143..1324] [gbkey=CDS]

ATGGAGGAGCCGCAGTCAGATCCTAGCGTCGAGCCCCCTCTGAGTCAGGAAACATTTTCAGACCTATGGA

AACTACTTCCTGAAAACAACGTTCTGTCCCCCTTGCCGTCCCAAGCAATGGATGATTTGATGCTGTCCCC

GGACGATATTGAACAATGGTTCACTGAAGACCCAGGTCCAGATGAAGCTCCCAGAATGCCAGAGGCTGCT

CCCCCCGTGGCCCCTGCACCAGCAGCTCCTACACCGGCGGCCCCTGCACCAGCCCCCTCCTGGCCCCTGT

CATCTTCTGTCCCTTCCCAGAAAACCTACCAGGGCAGCTACGGTTTCCGTCTGGGCTTCTTGCATTCTGG

GACAGCCAAGTCTGTGACTTGCACGTACTCCCCTGCCCTCAACAAGATGTTTTGCCAACTGGCCAAGACC

TGCCCTGTGCAGCTGTGGGTTGATTCCACACCCCCGCCCGGCACCCGCGTCCGCGCCATGGCCATCTACA

AGCAGTCACAGCACATGACGGAGGTTGTGAGGCGCTGCCCCCACCATGAGCGCTGCTCAGATAGCGATGG

TCTGGCCCCTCCTCAGCATCTTATCCGAGTGGAAGGAAATTTGCGTGTGGAGTATTTGGATGACAGAAAC

ACTTTTCGACATAGTGTGGTGGTGCCCTATGAGCCGCCTGAGGTTGGCTCTGACTGTACCACCATCCACT

ACAACTACATGTGTAACAGTTCCTGCATGGGCGGCATGAACCGGAGGCCCATCCTCACCATCATCACACT

GGAAGACTCCAGTGGTAATCTACTGGGACGGAACAGCTTTGAGGTGCGTGTTTGTGCCTGTCCTGGGAGA

GACCGGCGCACAGAGGAAGAGAATCTCCGCAAGAAAGGGGAGCCTCACCACGAGCTGCCCCCAGGGAGCA

CTAAGCGAGCACTGCCCAACAACACCAGCTCCTCTCCCCAGCCAAAGAAGAAACCACTGGATGGAGAATA

TTTCACCCTTCAGATCCGTGGGCGTGAGCGCTTCGAGATGTTCCGAGAGCTGAATGAGGCCTTGGAACTC

AAGGATGCCCAGGCTGGGAAGGAGCCAGGGGGGAGCAGGGCTCACTCCAGCCACCTGAAGTCCAAAAAGG

GTCAGTCTACCTCCCGCCATAAAAAACTCATGTTCAAGACAGAAGGGCCTGACTCAGACTGA

>lcl|NM_021202.3_cds_NP_067025.1_1 [gene=TP53INP2] [db_xref=CCDS:CCDS13240.1] [protein=tumor protein p53-inducible nuclear protein 2] [protein_id=NP_067025.1] [location=336..998] [gbkey=CDS]

ATGTTCCAGCGCCTCTCCAGCCTCTTCTTCAGCACCCCCTCGCCCCCCGAAGACCCCGACTGCCCCCGCG

CCTTCGTGTCGGAGGAGGATGAAGTGGACGGCTGGCTCATCATTGACCTGCCGGACAGCTACGCGGCTCC

ACCCAGCCCCGGGGCCGCCCCTGCCCCCGCGGGCCGCCCTCCGCCCGCGCCCTCCTTGATGGACGAGAGC

TGGTTTGTTACCCCTCCCGCCTGTTTTACGGCAGAGGGGCCTGGACTCGGTCCCGCCCGCCTCCAGAGCA

GTCCCCTGGAGGACCTCCTCATCGAGCACCCCAGCATGTCCGTTTACGTCACCGGCAGCACCATAGTGCT

AGAGCCCGGGTCCCCTTCCCCGCTCCCGGACGCGGCCCTGCCTGACGGCGACCTCAGCGAAGGGGAATTG

ACGCCCGCCCGCCGCGAGCCGCGGGCCGCGCCACGCCGCTCCTCTCCCAGCGCGGGCGGCGCTGCTGG

AGAAGGCGGGCCAGGTGCGGCGGCTGCAGCGGGCCCGGCAGCGGGCAGAGCGCCACGCGCTGAGCGCCAA

GGCGGTGCAGCGGCAGAACCGAGCCCGCGAGAGCCGTCCGCGCCGGTCCAAGAACCAGAGCAGCTTCATC

TACCAGCCGTGCCAGCGCCAGTTCAACTACTGA

>lcl|NM_003722.5_cds_NP_003713.3_1 [gene=TP63] [db_xref=CCDS:CCDS3293.1] [protein=tumor protein 63 isoform 1] [protein_id=NP_003713.3] [location=128..2170] [gbkey=CDS]

ATGAATTTTGAAACTTCACGGTGTGCCACCCTACAGTACTGCCCTGACCCTTACATCCAGCGTTTCGTAG

AAACCCCAGCTCATTTCTCTTGGAAAGAAAGTTATTACCGATCCACCATGTCCCAGAGCACACAGACAAA

TGAATTCCTCAGTCCAGAGGTTTTCCAGCATATCTGGGATTTTCTGGAACAGCCTATATGTTCAGTTCAG

CCCATTGACTTGAACTTTGTGGATGAACCATCAGAAGATGGTGCGACAAACAAGATTGAGATTAGCATGG

ACTGTATCCGCATGCAGGACTCGGACCTGAGTGACCCCATGTGGCCACAGTACACGAACCTGGGGCTCCT

GAACAGCATGGACCAGCAGATTCAGAACGGCTCCTCGTCCACCAGTCCCTATAACACAGACCACGCGCAG

AACAGCGTCACGGCGCCCTCGCCCTACGCACAGCCCAGCTCCACCTTCGATGCTCTCTCTCCATCACCCG

CCATCCCCTCCAACACCGACTACCCAGGCCCGCACAGTTTCGACGTGTCCTTCCAGCAGTCGAGCACCGC

CAAGTCGGCCACCTGGACGTATTCCACTGAACTGAAGAAACTCTACTGCCAAATTGCAAAGACATGCCCC

ATCCAGATCAAGGTGATGACCCCACCTCCTCAGGGAGCTGTTATCCGCGCCATGCCTGTCTACAAAAAAG

CTGAGCACGTCACGGAGGTGGTGAAGCGGTGCCCCAACCATGAGCTGAGCCGTGAATTCAACGAGGGACA

GATTGCCCCTCCTAGTCATTTGATTCGAGTAGAGGGGAACAGCCATGCCCAGTATGTAGAAGATCCCATC

ACAGGAAGACAGAGTGTGCTGGTACCTTATGAGCCACCCCAGGTTGGCACTGAATTCACGACAGTCTTGT

ACAATTTCATGTGTAACAGCAGTTGTGTTGGAGGGATGAACCGCCGTCCAATTTTAATCATTGTTACTCT

GGAAACCAGAGATGGGCAAGTCCTGGGCCGACGCTGCTTTGAGGCCCGGATCTGTGCTTGCCCAGGAAGA

GACAGGAAGGCGGATGAAGATAGCATCAGAAAGCAGCAAGTTTCGGACAGTACAAAGAACGGTGATGGTA

CGAAGCGCCCGTTTCGTCAGAACACACATGGTATCCAGATGACATCCATCAAGAAACGAAGATCCCCAGA

TGATGAACTGTTATACTTACCAGTGAGGGGCCGTGAGACTTATGAAATGCTGTTGAAGATCAAAGAGTCC

CTGGAACTCATGCAGTACCTTCCTCAGCACACAATTGAAACGTACAGGCAACAGCAACAGCAGCAGCACC

AGCACTTACTTCAGAAACAGACCTCAATACAGTCTCCATCTTCATATGGTAACAGCTCCCCACCTCTGAA

CAAAATGAACAGCATGAACAAGCTGCCTTCTGTGAGCCAGCTTATCAACCCTCAGCAGCGCAACGCCCTC

ACTCCTACAACCATTCCTGATGGCATGGGAGCCAACATTCCCATGATGGGCACCCACATGCCAATGGCTG

GAGACATGAATGGACTCAGCCCCACCCAGGCACTCCCTCCCCCACTCTCCATGCCATCCACCTCCCACTG

CACACCCCCACCTCCGTATCCCACAGATTGCAGCATTGTCAGTTTCTTAGCGAGGTTGGGCTGTTCATCA

TGTCTGGACTATTTCACGACCCAGGGGCTGACCACCATCTATCAGATTGAGCATTACTCCATGGATGATC

TGGCAAGTCTGAAAATCCCTGAGCAATTTCGACATGCGATCTGGAAGGGCATCCTGGACCACCGGCAGCT

CCACGAATTCTCCTCCCCTTCTCATCTCCTGCGGACCCCAAGCAGTGCCTCTACAGTCAGTGTGGGCTCC

AGTGAGACCCGGGGTGAGCGTGTTATTGATGCTGTGCGATTCACCCTCCGCCAGACCATCTCTTTCCCAC

CCCGAGATGAGTGGAATGACTTCAACTTTGACATGGATGCTCGCCGCAATAAGCAACAGCGCATCAAAGA

GGAGGGGGAGTGA

>lcl|NM_005427.4_cds_NP_005418.1_1 [gene=TP73] [db_xref=CCDS:CCDS49.1] [protein=tumor protein p73 isoform a] [protein_id=NP_005418.1] [location=160..2070] [gbkey=CDS]

ATGGCCCAGTCCACCGCCACCTCCCCTGATGGGGGCACCACGTTTGAGCACCTCTGGAGCTCTCTGGAAC

CAGACAGCACCTACTTCGACCTTCCCCAGTCAAGCCGGGGGAATAATGAGGTGGTGGGCGGAACGGATTC

CAGCATGGACGTCTTCCACCTGGAGGGCATGACTACATCTGTCATGGCCCAGTTCAATCTGCTGAGCAGC

ACCATGGACCAGATGAGCAGCCGCGCGGCCTCGGCCAGCCCCTACACCCCAGAGCACGCCGCCAGCGTGC

CCACCCACTCGCCCTACGCACAACCCAGCTCCACCTTCGACACCATGTCGCCGGCGCCTGTCATCCCCTC

CAACACCGACTACCCCGGACCCCACCACTTTGAGGTCACTTTCCAGCAGTCCAGCACGGCCAAGTCAGCC

ACCTGGACGTACTCCCCGCTCTTGAAGAAACTCTACTGCCAGATCGCCAAGACATGCCCCATCCAGATCA

AGGTGTCCACCCCGCCACCCCCAGGCACCGCCATCCGGGCCATGCCTGTTTACAAGAAAGCGGAGCACGT

GACCGACGTCGTGAAACGCTGCCCCAACCACGAGCTCGGGAGGGACTTCAACGAAGGACAGTCTGCTCCA

GCCAGCCACCTCATCCGCGTGGAAGGCAATAATCTCTCGCAGTATGTGGATGACCCTGTCACCGGCAGGC

AGAGCGTCGTGGTGCCCTATGAGCCACCACAGGTGGGGACGGAATTCACCACCATCCTGTACAACTTCAT

GTGTAACAGCAGCTGTGTAGGGGGCATGAACCGGCGGCCCATCCTCATCATCATCACCCTGGAGATGCGG

GATGGGCAGGTGCTGGGCCGCCGGTCCTTTGAGGGCCGCATCTGCGCCTGTCCTGGCCGCGACCGAAAAG

CTGATGAGGACCACTACCGGGAGCAGCAGGCCCTGAACGAGAGCTCCGCCAAGAACGGGGCCGCCAGCAA

GCGTGCCTTCAAGCAGAGCCCCCCTGCCGTCCCCGCCCTTGGTGCCGGTGTGAAGAAGCGGCGGCATGGA

GACGAGGACACGTACTACCTTCAGGTGCGAGGCCGGGAGAACTTTGAGATCCTGATGAAGCTGAAAGAGA

GCCTGGAGCTGATGGAGTTGGTGCCGCAGCCACTGGTGGACTCCTATCGGCAGCAGCAGCAGCTCCTACA

GAGGCCGAGTCACCTACAGCCCCCGTCCTACGGGCCGGTCCTCTCGCCCATGAACAAGGTGCACGGGGGC

ATGAACAAGCTGCCCTCCGTCAACCAGCTGGTGGGCCAGCCTCCCCCGCACAGTTCGGCAGCTACACCCA

ACCTGGGGCCCGTGGGCCCCGGGATGCTCAACAACCATGGCCACGCAGTGCCAGCCAACGGCGAGATGAG

CAGCAGCCACAGCGCCCAGTCCATGGTCTCGGGGTCCCACTGCACTCCGCCACCCCCCTACCACGCCGAC

CCCAGCCTCGTCAGTTTTTTAACAGGATTGGGGTGTCCAAACTGCATCGAGTATTTCACCTCCCAAGGGT

TACAGAGCATTTACCACCTGCAGAACCTGACCATTGAGGACCTGGGGGCCCTGAAGATCCCCGAGCAGTA

CCGCATGACCATCTGGCGGGGCCTGCAGGACCTGAAGCAGGGCCACGACTACAGCACCGCGCAGCAGCTG

CTCCGCTCTAGCAACGCGGCCACCATCTCCATCGGCGGCTCAGGGGAACTGCAGCGCCAGCGGGTCATGG

AGGCCGTGCACTTCCGCGTGCGCCACACCATCACCATCCCCAACCGCGGCGGCCCAGGCGGCGGCCCTGA

CGAGTGGGCGGACTTCGGCTTCGACCTGCCCGACTGCAAGGCCCGCAAGCAGCCCATCAAGGAGGAGTTC

ACGGAGGCCGAGATCCACTGA

>lcl|NM_000368.5_cds_NP_000359.1_1 [gene=TSC1] [db_xref=CCDS:CCDS6956.1] [protein=hamartin isoform 1] [protein_id=NP_000359.1] [location=218..3712] [gbkey=CDS]

ATGGCCCAACAAGCAAATGTCGGGGAGCTTCTTGCCATGCTGGACTCCCCCATGCTGGGTGTGCGGGACG

ACGTGACAGCTGTCTTTAAAGAGAACCTCAATTCTGACCGTGGCCCTATGCTTGTAAACACCTTGGTGGA

TTATTACCTGGAAACCAGCTCTCAGCCGGCATTGCACATCCTGACCACCTTGCAAGAGCCACATGACAAG

CACCTCTTGGACAGGATTAACGAATATGTGGGCAAAGCCGCCACTCGTTTATCCATCCTCTCGTTACTGG

GTCATGTCATAAGACTGCAGCCATCTTGGAAGCATAAGCTCTCTCAAGCACCTCTTTTGCCTTCTTTACT

AAAATGTCTCAAGATGGACACTGACGTCGTTGTCCTCACAACAGGCGTCTTGGTGTTGATAACCATGCTA

CCAATGATTCCACAGTCTGGGAAACAGCATCTTCTTGATTTCTTTGACATTTTTGGCCGTCTGTCATCAT

GGTGCCTGAAGAAACCAGGCCACGTGGCGGAAGTCTATCTCGTCCATCTCCATGCCAGTGTGTACGCACT

CTTTCATCGCCTTTATGGAATGTACCCTTGCAACTTCGTCTCCTTTTTGCGTTCTCATTACAGTATGAAA

GAAAACCTGGAGACTTTTGAAGAAGTGGTCAAGCCAATGATGGAGCATGTGCGAATTCATCCGGAATTAG

TGACTGGATCCAAGGACCATGAACTGGACCCTCGAAGGTGGAAGAGATTAGAAACTCATGATGTTGTGAT

CGAGTGTGCCAAAATCTCTCTGGATCCCACAGAAGCCTCATATGAAGATGGCTATTCTGTGTCTCACCAA

ATCTCAGCCCGCTTTCCTCATCGTTCAGCCGATGTCACCACCAGCCCTTATGCTGACACACAGAATAGCT

ATGGGTGTGCTACTTCTACCCCTTACTCCACGTCTCGGCTGATGTTGTTAAATATGCCAGGGCAGCTACC

TCAGACTCTGAGTTCCCCATCGACACGGCTGATAACTGAACCACCACAAGCTACTCTTTGGAGCCCATCT

ATGGTTTGTGGTATGACCACTCCTCCAACTTCTCCTGGAAATGTCCCACCTGATCTGTCACACCCTTACA

GTAAAGTCTTTGGTACAACTGCAGGTGGAAAAGGAACTCCTCTGGGAACCCCAGCAACCTCTCCTCCTCC

AGCCCCACTCTGTCATTCGGATGACTACGTGCACATTTCACTCCCCCAGGCCACAGTCACACCCCCCAGG

AAGGAAGAGAGAATGGATTCTGCAAGACCATGTCTACACAGACAACACCATCTTCTGAATGACAGAGGAT

CAGAAGAGCCACCTGGCAGCAAAGGTTCTGTCACTCTAAGTGATCTTCCAGGGTTTTTAGGTGATCTGGC

CTCTGAAGAAGATAGTATTGAAAAAGATAAAGAAGAAGCTGCAATATCTAGAGAACTTTCTGAGATCACC

ACAGCAGAGGCAGAGCCTGTGGTTCCTCGAGGAGGCTTTGACTCTCCCTTTTACCGAGACAGTCTCCCAG

GTTCTCAGCGGAAGACCCACTCGGCAGCCTCCAGTTCTCAGGGCGCCAGCGTGAACCCTGAGCCTTTACA

CTCCTCCCTGGACAAGCTTGGGCCTGACACACCAAAGCAAGCCTTTACTCCCATAGACCTGCCCTGCGGC

AGTGCTGATGAAAGCCCTGCGGGAGACAGGGAATGCCAGACTTCTTTGGAGACCAGTATCTTCACTCCCA

GTCCTTGTAAAATTCCACCTCCGACGAGAGTGGGCTTTGGAAGCGGGCAGCCTCCCCCGTATGATCATCT

TTTTGAGGTGGCATTGCCAAAGACAGCCCATCATTTTGTCATCAGGAAGACTGAGGAGCTGTTAAAGAAA

GCAAAAGGAAACACAGAGGAAGATGGTGTGCCCTCTACCTCCCCAATGGAAGTGCTGGACAGACTGATAC

AGCAGGGAGCAGACGCGCACAGCAAGGAGCTGAACAAGTTGCCTTTACCCAGCAAGTCTGTCGACTGGAC

CCACTTTGGAGGCTCTCCTCCTTCAGATGAGATCCGCACCCTCCGAGACCAGTTGCTTTTACTGCACAAC

CAGTTACTCTATGAGCGTTTTAAGAGGCAGCAGCATGCCCTCCGGAACAGGCGGCTCCTCCGCAAGGTGA

TCAAAGCAGCAGCTCTGGAGGAACATAATGCTGCCATGAAAGATCAGTTGAAGTTACAAGAGAAGGACAT

CCAGATGTGGAAGGTTAGTCTGCAGAAAGAACAAGCTAGATACAATCAGCTCCAGGAGCAGCGTGACACT

ATGGTAACCAAGCTCCACAGCCAGATCAGACAGCTGCAGCATGACCGAGAGGAATTCTACAACCAGAGCC

AGGAATTACAGACGAAGCTGGAGGACTGCAGGAACATGATTGCGGAGCTGCGGATAGAACTGAAGAAGGC

CAACAACAAGGTGTGTCACACTGAGCTGCTGCTCAGTCAGGTTTCCCAAAAGCTCTCAAACAGTGAGTCG

GTCCAGCAGCAGATGGAGTTCTTGAACAGGCAGCTGTTGGTTCTTGGGGAGGTCAACGAGCTCTATTTGG

AACAACTGCAGAACAAGCACTCAGATACCACAAAGGAAGTAGAAATGATGAAAGCCGCCTATCGGAAAGA

GCTAGAAAAAAACAGAAGCCATGTTCTCCAGCAGACTCAGAGGCTTGATACCTCCCAAAAACGGATTTTG

GAACTGGAATCTCACCTGGCCAAGAAAGACCACCTTCTTTTGGAACAGAAGAAATATCTAGAGGATGTCA

AACTCCAGGCAAGAGGACAGCTGCAGGCCGCAGAGAGCAGGTATGAGGCTCAGAAAAGGATAACCCAGGT

GTTTGAATTGGAGATCTTAGATTTATATGGCAGGTTGGAGAAAGATGGCCTCCTGAAAAAACTTGAAGAA

GAAAAAGCAGAAGCAGCTGAAGCAGCAGAAGAAAGGCTTGACTGTTGTAATGACGGGTGCTCAGATTCCA

TGGTAGGGCACAATGAAGAGGCATCTGGCCACAACGGTGAGACCAAGACCCCCAGGCCCAGCAGCGCCCG

GGGCAGTAGTGGAAGCAGAGGTGGTGGAGGCAGCAGCAGCAGCAGCAGCGAGCTTTCTACCCCAGAGAAA

CCCCCACACCAGAGGGCAGGCCCATTCAGCAGTCGGTGGGAGACGACTATGGGAGAAGCGTCTGCCAGCA

TCCCCACCACTGTGGGCTCACTTCCCAGTTCAAAAAGCTTCCTGGGTATGAAGGCTCGAGAGTTATTTCG

TAATAAGAGCGAGAGCCAGTGTGATGAGGACGGCATGACCAGTAGCCTTTCTGAGAGCCTAAAGACAGAA

CTGGGCAAAGACTTGGGTGTGGAAGCCAAGATTCCCCTGAACCTAGATGGCCCTCACCCGTCTCCCCCGA

CCCCGGACAGTGTTGGACAGCTACATATCATGGACTACAATGAGACTCATCATGAACACAGCTAA

>lcl|NM_000548.5_cds_NP_000539.2_1 [gene=TSC2] [db_xref=CCDS:CCDS10458.1] [protein=tuberin isoform 1] [protein_id=NP_000539.2] [location=111..5534] [gbkey=CDS]

ATGGCCAAACCAACAAGCAAAGATTCAGGCTTGAAGGAGAAGTTTAAGATTCTGTTGGGACTGGGAACAC

CGAGGCCAAATCCCAGGTCTGCAGAGGGTAAACAGACGGAGTTTATCATCACCGCGGAAATACTGAGAGA

ACTGAGCATGGAATGTGGCCTCAACAATCGCATCCGGATGATAGGGCAGATTTGTGAAGTCGCAAAAACC

AAGAAATTTGAAGAGCACGCAGTGGAAGCACTCTGGAAGGCGGTCGCGGATCTGTTGCAGCCGGAGCGGC

CGCTGGAGGCCCGGCACGCGGTGCTGGCTCTGCTGAAGGCCATCGTGCAGGGGCAGGGCGAGCGTTTGGG

GGTCCTCAGAGCCCTCTTCTTTAAGGTCATCAAGGATTACCCTTCCAACGAAGACCTTCACGAAAGGCTG

GAGGTTTTCAAGGCCCTCACAGACAATGGGAGACACATCACCTACTTGGAGGAAGAGCTGGCTGACTTTG

TCCTGCAGTGGATGGATGTTGGCTTGTCCTCGGAATTCCTTCTGGTGCTGGTGAACTTGGTCAAATTCAA

TAGCTGTTACCTCGACGAGTACATCGCAAGGATGGTTCAGATGATCTGTCTGCTGTGCGTCCGGACCGCG

TCCTCTGTGGACATAGAGGTCTCCCTGCAGGTGCTGGACGCCGTGGTCTGCTACAACTGCCTGCCGGCTG

AGAGCCTCCCGCTGTTCATCGTTACCCTCTGTCGCACCATCAACGTCAAGGAGCTCTGCGAGCCTTGCTG

GAAGCTGATGCGGAACCTCCTTGGCACCCACCTGGGCCACAGCGCCATCTACAACATGTGCCACCTCATG

GAGGACAGAGCCTACATGGAGGACGCGCCCCTGCTGAGAGGAGCCGTGTTTTTTGTGGGCATGGCTCTCT

GGGGAGCCCACCGGCTCTATTCTCTCAGGAACTCGCCGACATCTGTGTTGCCATCATTTTACCAGGCCAT

GGCATGTCCGAACGAGGTGGTGTCCTATGAGATCGTCCTGTCCATCACCAGGCTCATCAAGAAGTATAGG

AAGGAGCTCCAGGTGGTGGCGTGGGACATTCTGCTGAACATCATCGAACGGCTCCTTCAGCAGCTCCAGA

CCTTGGACAGCCCGGAGCTCAGGACCATCGTCCATGACCTGTTGACCACGGTGGAGGAGCTGTGTGACCA

GAACGAGTTCCACGGGTCTCAGGAGAGATACTTTGAACTGGTGGAGAGATGTGCGGACCAGAGGCCTGAG

TCCTCCCTCCTGAACCTGATCTCCTATAGAGCGCAGTCCATCCACCCGGCCAAGGACGGCTGGATTCAGA

ACCTGCAGGCGCTGATGGAGAGATTCTTCAGGAGCGAGTCCCGAGGCGCCGTGCGCATCAAGGTGCTGGA

CGTGCTGTCCTTTGTGCTGCTCATCAACAGGCAGTTCTATGAGGAGGAGCTGATTAACTCAGTGGTCATC

TCGCAGCTCTCCCACATCCCCGAGGATAAAGACCACCAGGTCCGAAAGCTGGCCACCCAGTTGCTGGTGG

ACCTGGCAGAGGGCTGCCACACACACCACTTCAACAGCCTGCTGGACATCATCGAGAAGGTGATGGCCCG

CTCCCTCTCCCCACCCCCGGAGCTGGAAGAAAGGGATGTGGCCGCATACTCGGCCTCCTTGGAGGATGTG

AAGACAGCCGTCCTGGGGCTTCTGGTCATCCTTCAGACCAAGCTGTACACCCTGCCTGCAAGCCACGCCA

CGCGTGTGTATGAGATGCTGGTCAGCCACATTCAGCTCCACTACAAGCACAGCTACACCCTGCCAATCGC

GAGCAGCATCCGGCTGCAGGCCTTTGACTTCCTGTTGCTGCTGCGGGCCGACTCACTGCACCGCCTGGGC

CTGCCCAACAAGGATGGAGTCGTGCGGTTCAGCCCCTACTGCGTCTGCGACTACATGGAGCCAGAGAGAG

GCTCTGAGAAGAAGACCAGCGGCCCCCTTTCTCCTCCCACAGGGCCTCCTGGCCCGGCGCCTGCAGGCCC

CGCCGTGCGGCTGGGGTCCGTGCCCTACTCCCTGCTCTTCCGCGTCCTGCTGCAGTGCTTGAAGCAGGAG

TCTGACTGGAAGGTGCTGAAGCTGGTTCTGGGCAGGCTGCCTGAGTCCCTGCGCTATAAAGTGCTCATCT

TTACTTCCCCTTGCAGTGTGGACCAGCTGTGCTCTGCTCTCTGCTCCATGCTTTCAGGCCCAAAGACACT

GGAGCGGCTCCGAGGCGCCCCAGAAGGCTTCTCCAGAACTGACTTGCACCTGGCCGTGGTTCCAGTGCTG

ACAGCATTAATCTCTTACCATAACTACCTGGACAAAACCAAACAGCGCGAGATGGTCTACTGCCTGGAGC

AGGGCCTCATCCACCGCTGTGCCAGCCAGTGCGTCGTGGCCTTGTCCATCTGCAGCGTGGAGATGCCTGA

CATCATCATCAAGGCGCTGCCTGTTCTGGTGGTGAAGCTCACGCACATCTCAGCCACAGCCAGCATGGCC

GTCCCACTGCTGGAGTTCCTGTCCACTCTGGCCAGGCTGCCGCACCTCTACAGGAACTTTGCCGCGGAGC

AGTATGCCAGTGTGTTCGCCATCTCCCTGCCGTACACCAACCCCTCCAAGTTTAATCAGTACATCGTGTG

TCTGGCCCATCACGTCATAGCCATGTGGTTCATCAGGTGCCGCCTGCCCTTCCGGAAGGATTTTGTCCCT

TTCATCACTAAGGGCCTGCGGTCCAATGTCCTCTTGTCTTTTGATGACACCCCCGAGAAGGACAGCTTCA

GGGCCCGGAGTACTAGTCTCAACGAGAGACCCAAGAGTCTGAGGATAGCCAGACCCCCCAAACAAGGCTT

GAATAACTCTCCACCCGTGAAAGAATTCAAGGAGAGCTCTGCAGCCGAGGCCTTCCGGTGCCGCAGCATC

AGTGTGTCTGAACATGTGGTCCGCAGCAGGATACAGACGTCCCTCACCAGTGCCAGCTTGGGGTCTGCAG

ATGAGAACTCCGTGGCCCAGGCTGACGATAGCCTGAAAAACCTCCACCTGGAGCTCACGGAAACCTGTCT

GGACATGATGGCTCGATACGTCTTCTCCAACTTCACGGCTGTCCCGAAGAGGTCTCCTGTGGGCGAGTTC

CTCCTAGCGGGTGGCAGGACCAAAACCTGGCTGGTTGGGAACAAGCTTGTCACTGTGACGACAAGCGTGG

GAACCGGGACCCGGTCGTTACTAGGCCTGGACTCGGGGGAGCTGCAGTCCGGCCCGGAGTCGAGCTCCAG

CCCCGGGGTGCATGTGAGACAGACCAAGGAGGCGCCGGCCAAGCTGGAGTCCCAGGCTGGGCAGCAGGTG

TCCCGTGGGGCCCGGGATCGGGTCCGTTCCATGTCGGGGGGCCATGGTCTTCGAGTTGGCGCCCTGGACG

TGCCGGCCTCCCAGTTCCTGGGCAGTGCCACTTCTCCAGGACCACGGACTGCACCAGCCGCGAAACCTGA

GAAGGCCTCAGCTGGCACCCGGGTTCCTGTGCAGGAGAAGACGAACCTGGCGGCCTATGTGCCCCTGCTG

ACCCAGGGCTGGGCGGAGATCCTGGTCCGGAGGCCCACAGGGAACACCAGCTGGCTGATGAGCCTGGAGA

ACCCGCTCAGCCCTTTCTCCTCGGACATCAACAACATGCCCCTGCAGGAGCTGTCTAACGCCCTCATGGC

GGCTGAGCGCTTCAAGGAGCACCGGGACACAGCCCTGTACAAGTCACTGTCGGTGCCGGCAGCCAGCACG

GCCAAACCCCCTCCTCTGCCTCGCTCCAACACAGTGGCCTCTTTCTCCTCCCTGTACCAGTCCAGCTGCC

AAGGACAGCTGCACAGGAGCGTTTCCTGGGCAGACTCCGCCGTGGTCATGGAGGAGGGAAGTCCGGGCGA

GGTTCCTGTGCTGGTGGAGCCCCCAGGGTTGGAGGACGTTGAGGCAGCGCTAGGCATGGACAGGCGCACG

GATGCCTACAGCAGGTCGTCCTCAGTCTCCAGCCAGGAGGAGAAGTCGCTCCACGCGGAGGAGCTGGTTG

GCAGGGGCATCCCCATCGAGCGAGTCGTCTCCTCGGAGGGTGGCCGGCCCTCTGTGGACCTCTCCTTCCA

GCCCTCGCAGCCCCTGAGCAAGTCCAGCTCCTCTCCCGAGCTGCAGACTCTGCAGGACATCCTCGGGGAC

CCTGGGGACAAGGCCGACGTGGGCCGGCTGAGCCCTGAGGTTAAGGCCCGGTCACAGTCAGGGACCCTGG

ACGGGGAAAGTGCTGCCTGGTCGGCCTCGGGCGAAGACAGTCGGGGCCAGCCCGAGGGTCCCTTGCCTTC

CAGCTCCCCCCGCTCGCCCAGTGGCCTCCGGCCCCGAGGTTACACCATCTCCGACTCGGCCCCATCACGC

AGGGGCAAGAGAGTAGAGAGGGACGCCTTAAAGAGCAGAGCCACAGCCTCCAATGCAGAGAAAGTGCCAG

GCATCAACCCCAGTTTCGTGTTCCTGCAGCTCTACCATTCCCCCTTCTTTGGCGACGAGTCAAACAAGCC

AATCCTGCTGCCCAATGAGTCACAGTCCTTTGAGCGGTCGGTGCAGCTCCTCGACCAGATCCCATCATAC

GACACCCACAAGATCGCCGTCCTGTATGTTGGAGAAGGCCAGAGCAACAGCGAGCTCGCCATCCTGTCCA

ATGAGCATGGCTCCTACAGGTACACGGAGTTCCTGACGGGCCTGGGCCGGCTCATCGAGCTGAAGGACTG

CCAGCCGGACAAGGTGTACCTGGGAGGCCTGGACGTGTGTGGTGAGGACGGCCAGTTCACCTACTGCTGG

CACGATGACATCATGCAAGCCGTCTTCCACATCGCCACCCTGATGCCCACCAAGGACGTGGACAAGCACC

GCTGCGACAAGAAGCGCCACCTGGGCAACGACTTTGTGTCCATTGTCTACAATGACTCCGGTGAGGACTT

CAAGCTTGGCACCATCAAGGGCCAGTTCAACTTTGTCCACGTGATCGTCACCCCGCTGGACTACGAGTGC

AACCTGGTGTCCCTGCAGTGCAGGAAAGACATGGAGGGCCTTGTGGACACCAGCGTGGCCAAGATCGTGT

CTGACCGCAACCTGCCCTTCGTGGCCCGCCAGATGGCCCTGCACGCAAATATGGCCTCACAGGTGCATCA

TAGCCGCTCCAACCCCACCGATATCTACCCCTCCAAGTGGATTGCCCGGCTCCGCCACATCAAGCGGCTC

CGCCAGCGGATCTGCGAGGAAGCCGCCTACTCCAACCCCAGCCTACCTCTGGTGCACCCTCCGTCCCATA

GCAAAGCCCCTGCACAGACTCCAGCCGAGCCCACACCTGGCTATGAGGTGGGCCAGCGGAAGCGCCTCAT

CTCCTCGGTGGAGGACTTCACCGAGTTTGTGTGA

>lcl|NM_001004125.3_cds_NP_001004125.2_1 [gene=TUSC1] [db_xref=CCDS:CCDS34999.1] [protein=tumor suppressor candidate gene 1 protein] [protein_id=NP_001004125.2] [location=133..762] [gbkey=CDS]

ATGTGGCGCATGCGTGGTGGCGCCACTAGGCGCGGGAGTTGCTGCGGCGGGGACGGTGCTGCGGACGGCC

GAGGGCCAGGCCGCTCCGGCCGGGCTCGTGGTGGGGGCAGCCCCAGCGGCGGCGGCGGCGTGGGCTG

GCGAGGCCGCGCGGACGGCGCCCGACAGCAGCTGGAGGAGCGGTTTGCCGACCTGGCGGCGAGCCACTTG

GAGGCCATCCGTGCGCGGGACGAGTGGGACCGGCAGAACGCGCGGCTGCGTCAGGAGAACGCCCGGCTGC

GGCTCGAGAACCGGCGGCTGAAGCGCGAGAACCGCAGCCTCTTCCGTCAGGCTTTGCGGCTCCCCGGCGA

AGGCGGCAACGGGACGCCCGCGGAGGCACGCCGGGTCCCTGAAGAGGCCAGCACGAACCGGAGGGCTAGA

GACAGCGGCCGAGAGGACGAGCCGGGCAGCCCCAGGGCCCTGAGGGCCCGACTTGAGAAGCTGGAAGCCA

TGTACCGCCGGGCCCTGCTGCAGCTGCACCTCGAGCAGCGGGGACCACGTCCGAGCGGGGACAAGGAGGA

GCAGCCTCTACAGGAACCCGACTCCGGCCTCCGCTCCCGGGACTCGGAGCCCTCTGGGCCCTGGCTGTAG

>lcl|NM_003565.4_cds_NP_003556.2_1 [gene=ULK1] [db_xref=CCDS:CCDS9274.1] [protein=serine/threonine-protein kinase ULK1] [protein_id=NP_003556.2] [location=381..3533] [gbkey=CDS]

ATGGAGCCCGGCCGCGGCGGCACAGAGACCGTGGGCAAGTTCGAGTTCTCCCGCAAGGACCTGATCGGCC

ACGGCGCCTTCGCGGTGGTCTTCAAGGGCCGCCACCGCGAGAAGCACGATTTGGAGGTCGCCGTCAAGTG

CATTAACAAGAAGAACCTCGCCAAGTCTCAGACGCTGCTGGGGAAGGAAATCAAAATCCTGAAGGAACTG

AAACATGAAAACATCGTGGCCCTGTACGACTTCCAGGAAATGGCTAATTCTGTCTACCTGGTTATGGAGT

ACTGCAACGGTGGGGACCTGGCCGACTACCTGCACGCCATGCGCACGCTGAGCGAGGACACCATCAGGCT

CTTCCTGCAGCAGATCGCGGGCGCCATGCGGCTTCTGCACAGCAAAGGCATCATCCACCGCGACCTGAAA

CCGCAGAACATCCTGCTGTCCAACCCCGCCGGCCGCCGCGCCAACCCCAACAGCATCCGCGTCAAGATCG

CTGACTTCGGCTTCGCGCGGTACCTCCAGAGCAACATGATGGCGGCCACACTCTGCGGCTCCCCCATGTA

CATGGCCCCCGAGGTCATCATGTCCCAGCACTACGACGGGAAGGCGGACCTGTGGAGCATCGGCACCATC

GTCTACCAGTGCCTGACGGGGAAGGCGCCCTTCCAGGCCAGCAGCCCCCAGGACCTGCGCCTGTTCTACG

AGAAGAACAAGACGTTGGTCCCCACCATCCCCCGGGAGACCTCGGCCCCGCTGCGGCAGCTGCTCCTGGC

CCTACTGCAACGCAACCACAAGGACCGCATGGACTTCGATGAGTTTTTCATCACCCTTTCCTCGATGCC

AGCCCCTCGGTCAGGAAATCCCCACCCGTGCCTGTGCCCTCGTACCCAAGCTCGGGGTCCGGCAGCAGCT

CCAGCAGCAGCTCCACCTCCCACCTGGCCTCCCCGCCGTCCCTGGGCGAGATGCAGCAGCTGCAGAAGAC

CCTGGCCTCCCCGGCTGACACCGCTGGCTTCCTGCACAGCTCCCGGGACTCTGGTGGCAGCAAGGACTCT

TCCTGTGACACAGACGACTTCGTCATGGTCCCCGCGCAGTTTCCAGGTGACCTGGTGGCTGAGGCGCCCA

GTGCCAAACCCCCGCCAGACAGCCTGATGTGCAGTGGGAGCTCACTGGTGGCCTCTGCGGGCTTGGAGAG

CCACGGCCGGACCCCATCTCCATCCCCACCCTGCAGCAGCTCCCCCAGTCCCTCAGGCCGGGCTGGCCCG

TTCTCCAGCAGCAGGTGCGGCGCCTCTGTCCCCATCCCAGTCCCCACGCAGGTGCAGAACTACCAGCGCA

TTGAGCGAAACCTGCAGTCACCCACCCAGTTCCAAACACCTCGGTCCTCTGCCATCCGCAGGTCAGGCAG

CACCAGCCCCCTGGGCTTTGCAAGGGCCAGCCCCTCGCCCCCTGCCCACGCTGAGCATGGAGGCGTCCTG

GCCAGGAAGATGTCTCTGGGTGGAGGCCGGCCCTACACGCCATCTCCTCAAGTTGGAACCATCCCTGAGC

GGCCAGGCTGGAGCGGGACGCCCTCCCCACAGGGAGCTGAGATGCGGGGTGGCAGGTCCCCTCGTCCAGG

CTCCTCTGCACCCGAGCACTCTCCCCGCACTTCCGGGCTGGGCTGCCGCCTGCACAGCGCCCCCAACCTG

TCTGACTTGCACGTCGTCCGCCCCAAGCTGCCCAAACCCCCCACGGACCCCCTGGGAGCTGTGTTCAGCC

CACCACAGGCCAGCCCTCCCCAGCCGTCCCACGGCCTGCAGTCCTGCCGGAACCTGCGGGGCTCACCCAA

GCTGCCCGACTTCCTGCAGCGAAACCCCCTGCCCCCCATCCTGGGCTCCCCCACCAAGGCTGTGCCCTCC

TTTGACTTCCCGAAGACCCCCAGCTCCCAGAACCTGCTGGCCCTCCTAGCCCGGCAGGGCGTGGTGATGA

CGCCCCCTCGAAACCGGACGCTGCCCGACCTCTCGGAGGTGGGACCCTTCCATGGTCAGCCGTTGGGCCC

TGGCCTGCGGCCAGGCGAGGACCCCAAGGGCCCCTTTGGCCGGTCTTTCAGCACCAGCCGCCTCACTGAC

CTGCTCCTTAAGGCGGCGTTTGGGACACAAGCCCCGGACCCGGGCAGCACGGAGAGCCTGCAGGAGAAGC

CCATGGAGATCGCACCCTCAGCTGGCTTTGGAGGGAGCCTGCACCCAGGAGCCCGTGCTGGGGGCACCAG

CAGCCCTTCCCCGGTGGTCTTCACCGTGGGCTCTCCCCCGAGCGGGAGCACGCCCCCCCAGGGCCCCCGC

ACCAGGATGTTCTCAGCGGGCCCCACTGGCTCTGCCAGCTCTTCTGCCCGCCACCTGGTGCCTGGGCCCT

GCAGCGAGGCCCCAGCCCCTGAGCTCCCTGCTCCAGGACACGGCTGCAGCTTTGCCGACCCCATTACTGC

GAACCTGGAGGGGGCTGTGACCTTCGAGGCCCCCGACCTCCCTGAGGAGACCCTCATGGAGCAAGAGCAC

ACGGAGATCCTGCGTGGCCTGCGCTTCACGCTGCTGTTCGTGCAGCACGTCCTGGAGATCGCAGCCCTGA

AGGGCAGCGCCAGTGAGGCGGCGGGGGGCCCTGAGTACCAGCTGCAGGAGAGTGTGGTGGCCGACCAGAT

CAGCCTGCTGAGCCGAGAATGGGGCTTCGCGGAACAGCTGGTGCTGTACCTGAAGGTGGCCGAGCTACTG

TCCTCCGGCCTGCAAAGTGCCATCGACCAGATCCGGGCCGGCAAGCTCTGCCTGTCGTCCACTGTGAAGC

AGGTGGTGCGCAGGCTGAATGAGCTGTACAAGGCCAGCGTGGTGTCCTGCCAGGGCCTGAGCCTGCGGCT

GCAGCGCTTCTTCCTGGACAAGCAGCGGCTCCTGGACCGCATTCACAGCATCACTGCCGAGAGGCTCATC

TTCAGCCACGCTGTGCAGATGGTGCAGTCGGCTGCCCTGGACGAGATGTTCCAGCACCGTGAGGGCTGCG

TCCCACGCTACCACAAGGCCCTGCTGCTCCTGGAGGGGCTGCAGCACATGCTCTCGGACCAGGCCGACAT

CGAGAACGTCACCAAGTGCAAGCTGTGCATTGAGCGGAGACTCTCGGCGCTGCTGACTGGCATCTGTGCC

TGA

>lcl|NM_014683.4_cds_NP_055498.3_1 [gene=ULK2] [db_xref=CCDS:CCDS11213.1] [protein=serine/threonine-protein kinase ULK2] [protein_id=NP_055498.3] [location=520..3630] [gbkey=CDS]

ATGGAGGTGGTGGGTGACTTCGAGTACAGCAAGAGGGATCTCGTGGGACACGGGGCCTTCGCCGTGGTCT

TCCGGGGGCGGCACCGCCAGAAAACTGATTGGGAGGTAGCTATTAAAAGTATTAATAAAAAGAACTTGTC

AAAATCACAAATACTGCTTGGAAAGGAAATTAAAATCTTAAAGGAACTTCAGCATGAAAATATTGTAGCA

CTCTATGATGTTCAGGAATTACCCAACTCTGTCTTTTTGGTGATGGAGTATTGCAATGGTGGAGACCTCG

CAGATTATTTGCAAGCGAAAGGGACTCTCAGTGAAGACACGATCAGAGTGTTTCTGCATCAGATTGCTGC

TGCCATGCGAATCCTGCACAGCAAAGGAATCATCCACAGAGATCTCAAACCACAGAACATCTTGCTGTCC

TATGCCAATCGCAGAAAATCAAGTGTCAGTGGTATTCGCATCAAAATAGCGGATTTTGGTTTTGCTCGTT

ACCTACATAGTAACATGATGGCTGCAACACTGTGTGGATCCCCGATGTACATGGCTCCTGAGGTTATTAT

GTCTCAACATTATGATGCTAAGGCTGACTTGTGGAGCATAGGAACAGTGATATACCAATGCCTAGTTGGA

AAACCACCTTTTCAGGCCAATAGTCCTCAAGACTTAAGGATGTTTTATGAAAAAAACAGGAGCTTAATGC

CTAGTATTCCCAGAGAAACATCACCTTATTTGGCTAATCTCCTTTTGGGTTTGCTTCAGAGAAACCAAAA

AGATAGAATGGACTTTGAAGCATTTTTTAGCCATCCTTTTCTTGAGCAAGGTCCAGTAAAAAAATCTTGC

CCAGTTCCAGTGCCCATGTATTCTGGTTCTGTCTCTGGAAGCTCCTGTGGCAGCTCTCCATCTTGTCGTT

TTGCTTCTCCACCATCCCTTCCAGATATGCAGCATATTCAGGAAGAAAACTTATCTTCCCCACCATTGGG

TCCTCCCAACTATCTACAAGTTTCCAAAGATTCTGCCAGTACTAGTAGCAAGAACTCTTCTTGTGACACG

GATGACTTTGTTTTGGTGCCACACAACATCTCGTCAGACCACTCATGTGATATGCCAGTGGGGACTGCTG

GCAGACGTGCTTCAAATGAATTCTTGGTGTGTGGAGGGCAGTGTCAGCCTACTGTGTCACCTCACAGCGA

AACAGCACCAATTCCAGTTCCTACTCAAATAAGGAATTATCAGCGCATAGAGCAGAATCTTACATCTACT

GCCAGCTCAGGCACAAATGTACATGGTTCTCCAAGATCTGCAGTGGTACGAAGGTCCAACACCAGCCCCA

TGGGCTTCCTCCGGCCGGGATCATGCTCCCCAGTACCAGCAGACACAGCACAGACAGTTGGACGAAGGCT

CTCCACTGGGTCTTCTAGGCCTTACTCACCTTCCCCTTTGGTTGGTACCATTCCTGAGCAATTCAGTCAG

TGCTGCTGTGGGCATCCTCAGGGCCATGACTCCAGGAGTAGAAACTCCTCAGGTTCTCCAGTGCCACAAG

CTCAGTCCCCACAGTCTCTCTTATCGGGTGCTAGACTGCAGAGCGCCCCCACCCTCACTGACATCTATCA

GAACAAGCAGAAGCTCAGAAAACAGCACTCTGACCCCGTGTGCCCATCCCATACTGGGGCTGGGTACAGC

TACTCGCCTCAGCCCAGTCGGCCTGGCAGCCTTGGAACTTCTCCCACCAAGCACTTGGGGTCCTCTCCAC

GGAGTTCTGACTGGTTCTTTAAAACTCCTTTGCCAACAATCATTGGCTCTCCTACTAAGACCACAGCTCC

TTTCAAAATCCCTAAAACTCAAGCATCTTCCAACCTGTTAGCCTTGGTTACTCGTCATGGGCCTGCTGAA

GAACAGTCGAAAGATGGGAATGAGCCACGGGAATGTGCCCATTGCCTCTTAGTGCAAGGAAGTGAGAGGC

AGCGGGCCGAGCAGCAGAGCAAGGCAGTGTTTGGCAGATCTGTCAGTACCGGGAAGTTATCAGATCAACA

AGGAAAGACTCCTATATGTCGACATCAGGGCAGCACAGACAGTTTAAATACAGAACGACCAATGGATATA

GCTCCGGCAGGAGCCTGTGGTGGTGTTCTGGCACCTCCTGCAGGTACAGCAGCAAGTTCCAAGGCTGTCC

TCTTCACTGTAGGGTCTCCTCCACACAGTGCGGCAGCCCCCACTTGTACCCACATGTTCCTTCGAACAAG

AACAACCTCAGTGGGGCCCAGCAACTCCGGGGGCTCTCTTTGTGCCATGAGTGGCCGCGTGTGCGTGGGG

TCCCCGCCTGGCCCAGGCTTCGGCTCTTCCCCTCCAGGAGCAGAGGCAGCTCCCAGCCTGAGATACGTGC

CTTACGGTGCTTCACCCCCCAGCCTAGAGGGGCTCATCACCTTTGAAGCCCCTGAACTGCCGGAGGAGAC

GCTGATGGAGCGGGAACACACAGACACCTTACGCCATCTGAATGTGATGCTGATGTTCACTGAGTGTGTG

CTGGACCTGACAGCCATGAGGGGAGGAAACCCTGAGCTGTGCACATCTGCTGTGTCCTTGTACCAGATCC

AGGAGAGTGTGGTGGTGGACCAGATCAGTCAGCTGAGCAAAGACTGGGGGCGGGTGGAGCAGCTGGTGTT

GTACATGAAAGCAGCACAGCTGCTTGCGGCTTCTCTGCATCTTGCCAAAGCCCAGATCAAGTCCGGGAAA

CTGAGCCCATCCACAGCTGTGAAACAAGTTGTCAAGAATCTGAACGAACGATATAAATTCTGCATCACCA

TGTGCAAGAAACTTACAGAAAAGCTGAATCGATTCTTCTCTGACAAACAGAGGTTTATTGATGAAATCAA

CAGTGTGACTGCAGAGAAACTCATCTATAATTGTGCTGTAGAAATGGTTCAGTCTGCAGCCCTGGATGAG

ATGTTTCAGCAGACCGAAGATATTGTTTATCGCTATCATAAGGCAGCCCTTCTTTTGGAAGGCCTAAGTA

GGATTCTACAGGACCCTGCAGATATTGAAAATGTGCATAAATATAAATGTAGTATTGAGAGAAGACTGTC

GGCGCTCTGCCATAGCACCGCAACCGTGTGA

>lcl|NM_001099436.4_cds_NP_001092906.3_1 [gene=ULK3] [db_xref=CCDS:CCDS45305.1] [protein=serine/threonine-protein kinase ULK3 isoform a] [protein_id=NP_001092906.3] [location=52..1470] [gbkey=CDS]

ATGGCGGGGCCCGGCTGGGGTCCCCCGCGCCTGGACGGCTTCATCCTCACCGAGCGCCTGGGCAGCGGCA

CGTACGCCACGGTGTACAAGGCCTACGCCAAGAAGGACACTCGTGAAGTGGTAGCCATAAAGTGTGTAGC

CAAGAAAAGTCTGAACAAGGCATCGGTGGAGAACCTCCTCACGGAGATTGAGATCCTCAAGGGCATTCGA

CATCCCCACATTGTGCAGCTGAAAGACTTTCAGTGGGACAGTGACAATATCTACCTCATCATGGAGTTTT

GCGCAGGGGGCGACCTGTCTCGCTTCATCCATACCCGCAGGATTCTGCCTGAGAAGGTGGCGCGTGTCTT

CATGCAGCAATTAGCTAGCGCCCTGCAATTCCTGCATGAACGGAATATCTCTCACCTGGATCTGAAGCCA

CAGAACATTCTACTGAGCTCCTTGGAGAAGCCCCACCTAAAACTGGCAGACTTTGGTTTCGCACAACACA

TGTCCCCGTGGGATGAGAAGCACGTGCTCCGTGGCTCCCCCCTCTACATGGCCCCCGAGATGGTGTGCCA

GCGGCAGTATGACGCCCGCGTGGACCTCTGGTCCATGGGGGTCATCCTGTATGAAGCCCTCTTCGGGCAG

CCCCCCTTTGCCTCCAGGTCGTTCTCGGAGCTGGAAGAGAAGATCCGTAGCAACCGGGTCATCGAGCTCC

CCTTGCGGCCCCTGCTCTCCCGAGACTGCCGGGACCTACTGCAGCGGCTCCTGGAGCGGGACCCCAGCCG

TCGCATCTCCTTCCAGGACTTTTTTGCGCACCCCTGGGTGGACCTGGAGCACATGCCCAGTGGGGAGAGT

CTGGGGCGAGCAACCGCCCTGGTGGTGCAGGCTGTGAAGAAAGACCAGGAGGGGGATTCAGCAGCTGCCT

TATCACTCTACTGCAAGGCTCTGGACTTCTTTGTACCTGCCCTGCACTATGAAGTGGATGCCCAGCGGAA

GGAGGCAATTAAGGCAAAGGTGGGGCAGTACGTGTCCCGGGCTGAGGAGCTCAAGGCCATCGTCTCCTCT

TCCAATCAGGCCCTGCTGAGGCAGGGGACCTCTGCCCGAGACCTGCTCAGAGAGATGGCCCGGGACAAGC

CACGCCTCCTAGCTGCCCTGGAAGTGGCTTCAGCTGCCATGGCCAAGGAGGAGGCCGCCGGCGGGGAGCA

GGATGCCCTGGACCTGTACCAGCACAGCCTGGGGGAGCTACTGCTGTTGCTGGCAGCGGAGCCCCCGGGC

CGGAGGCGGGAGCTGCTTCACACTGAGGTTCAGAACCTCATGGCCCGAGCTGAATACTTGAAGGAGCAGG

TCAAGATGAGGGAATCTCGCTGGGAAGCTGACACCCTGGACAAAGAGGGACTGTCGGAATCTGTTCGTAG

CTCTTGCACCCTTCAGTGA

>lcl|NM_001272075.2_cds_NP_001259004.1_1 [gene=USP10] [db_xref=CCDS:CCDS62004.1] [protein=ubiquitin carboxyl-terminal hydrolase 10 isoform 1] [protein_id=NP_001259004.1] [location=201..2609] [gbkey=CDS]

ATGCCCTGGTTGCCCTCTCCTGGAATAGGGCAGTATATTTTTGGAGATTTTAGCCCTGATGAATTCAATC

AATTCTTTGTGACTCCTCGATCTTCAGTTGAGCTTCCTCCATACAGTGGAACAGTTCTGTGTGGCACACA

GGCTGTGGATAAACTACCTGATGGACAAGAATATCAGAGAATTGAGTTTGGTGTCGATGAAGTCATTGAA

CCCAGTGACACTTTGCCGAGAACCCCCAGCTACAGTATTTCAAGCACACTGAACCCTCAGGCCCCTGAAT

TTATTCTCGGTTGTACAGCTTCCAAAATAACCCCTGATGGTATCACTAAAGAAGCAAGCTATGGCTCCAT

CGACTGCCAGTACCCAGGCTCTGCCCTCGCTTTGGATGGAAGTTCTAATGTGGAGGCGGAAGTTTTGGAA

AATGATGGTGTCTCAGGTGGTCTTGGACAAAGGGAGCGTAAAAAGAAGAAAAAGCGGCCACCTGGATATT

ACAGCTATTTGAAAGATGGTGGCGATGATAGTATCTCCACAGAAGCCCTGGTCAATGGCCATGCCAATTC

AGCAGTCCCGAACAGTGTCAGTGCAGAGGATGCAGAATTTATGGGTGACATGCCCCCGTCAGTTACGCCC

AGGACTTGTAACAGCCCCCAGAACTCCACAGACTCTGTCAGTGACATTGTGCCTGACAGTCCTTTCCCCG

GAGCACTCGGCAGTGACACCAGGACTGCAGGGCAGCCAGAGGGGGGCCCCGGGGCTGATTTTGGTCAGTC

CTGCTTCCCTGCAGAGGCTGGCAGAGACACCCTGTCAAGGACAGCTGGGGCTCAGCCCTGCGTTGGTACC

GATACTACTGAAAACCTTGGAGTTGCTAATGGACAAATACTTGAATCCTCGGGTGAGGGCACAGCTACCA

ACGGGGTGGAGTTGCACACCACGGAAAGCATAGACTTGGACCCAACCAAACCCGAGAGTGCATCACCTCC

TGCTGACGGCACGGGCTCTGCATCAGGCACCCTTCCTGTCAGCCAGCCCAAGTCCTGGGCCAGCCTCTTT

CATGATTCTAAGCCCTCTTCCTCCTCGCCGGTGGCCTATGTGGAAACTAAGTATTCCCCTCCCGCCATAT

CTCCCCTGGTTTCTGAAAAGCAGGTTGAAGTCAAAGAAGGGCTTGTTCCGGTTTCAGAGGATCCTGTAGC

CATAAAGATTGCAGAGTTGCTGGAGAATGTAACCCTAATCCATAAACCAGTGTCGTTGCAACCCCGTGGG

CTGATCAATAAAGGGAACTGGTGCTACATTAATGCTACACTGCAGGCATTGGTTGCTTGCCCGCCGATGT

ACCACCTGATGAAGTTCATTCCTCTGTATTCCAAAGTGCAAAGGCCTTGTACGTCAACACCCATGATAGA

CAGCTTTGTTCGGCTAATGAATGAGTTCACTAATATGCCAGTACCTCCAAAACCCCGACAAGCTCTTGGA

GATAAAATCGTGAGGGATATTCGCCCTGGAGCTGCCTTTGAGCCCACATATATTTACAGACTCCTGACAG

TTAACAAGTCAAGCCTGTCTGAAAAGGGTCGACAAGAAGATGCTGAGGAATACTTAGGCTTCATTCTAAA

TGGACTTCATGAGGAAATGTTGAACCTAAAGAAGCTTCTCTCACCAAGTAATGAAAAACTTACGATTTCC

AACGGCCCCAAAAACCACTCGGTCAATGAAGAAGAGCAGGAAGAACAAGGTGAAGGAAGCGAGGATGAAT

GGGAACAAGTGGGCCCCCGGAACAAGACTTCCGTCACCCGCCAGGCGGATTTTGTTCAGACTCCAATCAC

CGGCATTTTTGGTGGACACATCAGGTCTGTGGTTTACCAGCAGAGTTCAAAAGAATCTGCCACTTTGCAG

CCATTTTTCACGTTGCAGTTGGATATCCAGTCAGACAAGATACGCACAGTCCAGGATGCACTGGAGAGCT

TGGTGGCAAGAGAATCTGTCCAAGGTTATACCACAAAAACCAAACAAGAGGTTGAGATAAGTCGAAGAGT

GACTCTGGAAAAACTCCCTCCTGTCCTCGTGCTGCACCTGAAACGATTCGTTTATGAGAAGACTGGTGGG

TGCCAGAAGCTTATCAAAAATATTGAATATCCTGTGGACTTGGAAATTAGTAAAGAACTGCTTTCTCCAG

GGGTTAAAAATAAGAATTTTAAATGCCACCGAACCTATCGGCTCTTTGCAGTGGTCTACCATCACGGCAA

CAGTGCGACGGGCGGCCATTACACTACAGACGTCTTCCAGATCGGTCTGAATGGCTGGCTGCGCATCGAT

GACCAGACAGTCAAGGTGATCAACCAGTACCAGGTGGTGAAACCAACTGCTGAACGCACAGCCTACCTCC

TGTATTACCGCCGAGTGGACCTGCTGTAA

>lcl|NM_003369.4_cds_NP_003360.2_1 [gene=UVRAG] [db_xref=CCDS:CCDS8241.1] [protein=UV radiation resistance-associated gene protein isoform 2] [protein_id=NP_003360.2] [location=199..2298] [gbkey=CDS]

ATGAGCGCCTCCGCGTCGGTCGGGGGCCCCGTCCCCCAGCCACCCCCGGGCCCGGCCGCTGCTCTGCCTC

CCGGTTCTGCCGCGCGGGCCCTGCATGTGGAGCTGCCGTCTCAGCAGCGGCGTCTTCGACATCTTCGGAA

CATTGCTGCCCGGAACATTGTTAATAGAAATGGCCATCAGCTCCTTGATACCTACTTTACACTTCACTTG

TGTAGTACTGAAAAGATATATAAAGAATTTTATAGAAGTGAAGTGATTAAGAATTCCTTGAATCCCACGT

GGCGAAGTCTCGATTTTGGAATTATGCCAGACCGTCTTGATACATCTGTGTCTTGTTTCGTGGTGAAGAT

ATGGGGTGGAAAGGAGAACATCTACCAGCTGTTGATTGAATGGAAAGTCTGTTTGGATGGGCTGAAATAC

TTGGGTCAGCAGATTCATGCCCGAAACCAAAATGAAATAATTTTTGGGCTGAATGATGGATACTATGGTG

CTCCATTTGAACATAAGGGTTATTCAAATGCTCAGAAGACTATTCTTCTGCAGGTGGATCAGAACTGTGT

TCGCAATTCTTACGATGTCTTCTCTTTGCTACGGCTTCATAGAGCCCAGTGTGCAATTAAACAGACTCAG

GTAACTGTTCAGAAAATTGGAAAGGAAATTGAAGAAAAACTAAGACTCACATCTACAAGCAATGAACTGA

AAAAAAAAAGTGAATGCCTGCAGTTAAAAATTTTGGTGCTTCAGAATGAACTGGAACGGCAGAAGAAAGC

TTTGGGACGGGAGGTGGCATTACTGCATAAGCAACAAATTGCATTACAAGACAAAGGAAGTGCATTTTCA

GCTGAGCACCTCAAACTTCAACTCCAGAAGGAATCCCTAAATGAGCTGAGGAAGGAGTGCACTGCAAAAA

GAGAACTCTTCTTGAAGACTAATGCTCAGTTGACAATTCGTTGCAGGCAGTTACTCTCTGAGCTTTCCTA

CATTTACCCTATTGATTTGAATGAACATAAGGATTACTTTGTATGCGGTGTCAAGTTGCCTAATTCTGAG

GACTTCCAAGCAAAAGATGATGGAAGCATTGCTGTTGCCCTTGGTTATACTGCACATCTGGTCTCCATGA

TTTCCTTTTTCCTACAAGTGCCCCTCAGATATCCTATAATTCATAAGGGGTCTAGATCAACAATCAAAGA

CAATATCAATGACAAACTGACGGAAAAGGAGAGAGAGTTTCCACTGTATCCAAAAGGAGGGGAGAAGTTG

CAGTTTGATTATGGTGTCTATCTTCTGAACAAAAATATAGCACAGCTAAGATATCAACATGGACTAGGGA

CTCCAGACTTGCGGCAAACCCTTCCCAACCTGAAAAACTTCATGGAGCATGGACTAATGGTCAGGTGTGA

CAGACATCACACCTCCAGTGCAATCCCTGTTCCTAAGAGACAAAGCTCCATATTTGGGGGTGCAGATGTA

GGCTTCTCTGGGGGGATCCCTTCACCAGACAAAGGACATCGAAAACGGGCCAGCTCTGAGAATGAGAGAC

TTCAGTACAAAACCCCTCCTCCCAGTTACAACTCAGCATTAGCCCAGCCTGTGACCACCGTCCCCTCCAT

GGGAGAGACCGAGAGAAAGATAACATCTCTATCCTCCTCCTTGGATACCTCCTTGGACTTCTCCAAAGAA

AACAAGAAAAAAGGAGAGGATCTAGTTGGCAGCTTAAACGGAGGCCACGCGAATGTGCACCCTAGCCAAG

AACAAGGAGAAGCCCTCTCCGGGCACCGGGCCACAGTCAATGGCACTCTCCTACCCAGCGAGCAGGCCGG

GTCCGCCAGTGTCCAGCTTCCAGGCGAGTTCCACCCAGTCTCAGAAGCTGAGCTCTGCTGTACTGTGGAG

CAAGCAGAAGAAATCATCGGGCTGGAAGCCACAGGTTTCGCCTCAGGTGATCAGCTAGAAGCATTTAACT

GCATCCCAGTGGACAGTGCTGTGGCAGTAGAGTGTGACGAACAAGTTCTGGGAGAATTTGAAGAGTTCTC

CCGAAGGATCTATGCACTGAATGAAAACGTATCCAGCTTCCGCCGGCCGCGCAGGAGTTCCGATAAGTGA

>lcl|NM_004781.4_cds_NP_004772.1_1 [gene=VAMP3] [db_xref=CCDS:CCDS88.1] [protein=vesicle-associated membrane protein 3] [protein_id=NP_004772.1] [location=89..391] [gbkey=CDS]

ATGTCTACAGGTCCAACTGCTGCCACTGGCAGTAATCGAAGACTTCAGCAGACACAAAATCAAGTAGATG

AGGTGGTGGACATAATGCGAGTTAACGTGGACAAGGTTCTGGAAAGAGACCAGAAGCTCTCTGAGTTAGA

CGACCGTGCAGACGCACTGCAGGCAGGCGCTTCTCAATTTGAAACGAGCGCAGCCAAGTTGAAGAGGAAA

TATTGGTGGAAGAATTGCAAGATGTGGGCAATCGGGATTACTGTTCTGGTTATCTTCATCATCATCATCA

TCGTGTGGGTTGTCTCTTCATGA

>lcl|NM_005638.6_cds_NP_005629.1_1 [gene=VAMP7] [db_xref=CCDS:CCDS14770.4] [protein=vesicle-associated membrane protein 7 isoform 1] [protein_id=NP_005629.1] [location=114..776] [gbkey=CDS]

ATGGCGATTCTTTTTGCTGTTGTTGCCAGGGGGACCACTATCCTTGCCAAACATGCTTGGTGTGGAGGAA

ACTTCCTGGAGGTGACAGAGCAGATTCTGGCTAAGATACCTTCTGAAAATAACAAACTAACGTACTCACA

TGGCAATTATTTGTTTCATTACATCTGCCAAGACAGGATTGTATATCTTTGTATCACTGATGATGATTTT

GAACGTTCCCGAGCCTTTAATTTTCTGAATGAGATAAAGAAGAGGTTCCAGACTACTTACGGTTCAAGAG

CACAGACAGCACTTCCATATGCCATGAATAGCGAGTTCTCAAGTGTCTTAGCTGCACAGCTGAAGCATCA

CTCTGAGAATAAGGGCCTAGACAAAGTGATGGAGACTCAAGCCCAAGTGGATGAACTGAAAGGAATCATG

GTCAGAAACATAGATCTGGTAGCTCAGCGAGGAGAAAGATTGGAATTATTGATTGACAAAACAGAAAATC

TTGTGGATTCTTCTGTCACCTTCAAAACTACCAGCAGAAATCTTGCTCGAGCCATGTGTATGAAGAACCT

CAAGCTCACTATTATCATCATCATCGTATCAATTGTGTTCATCTATATCATTGTTTCACCTCTCTGTGGT

GGATTTACATGGCCAAGCTGTGTGAAGAAATAG

>lcl|NM_001025366.3_cds_NP_001020537.2_1 [gene=VEGFA] [db_xref=CCDS:CCDS34457.1] [protein=vascular endothelial growth factor A isoform a] [protein_id=NP_001020537.2] [location=497..1735] [gbkey=CDS]

CTGACGGACAGACAGACAGACACCGCCCCCAGCCCCAGCTACCACCTCCTCCCCGGCCGGCGGCGGACAG

TGGACGCGGCGGCGAGCCGCGGGCAGGGGCCGGAGCCCGCGCCCGGAGGCGGGGTGGAGGGGGTCGGGGC

TCGCGGCGTCGCACTGAAACTTTTCGTCCAACTTCTGGGCTGTTCTCGCTTCGGAGGAGCCGTGGTCCGC

GCGGGGGAAGCCGAGCCGAGCGGAGCCGCGAGAAGTGCTAGCTCGGGCCGGGAGGAGCCGCAGCCGGAGG

AGGGGGAGGAGGAAGAAGAGAAGGAAGAGGAGAGGGGGCCGCAGTGGCGACTCGGCGCTCGGAAGCCGGG

CTCATGGACGGGTGAGGCGGCGGTGTGCGCAGACAGTGCTCCAGCCGCGCGCGCTCCCCAGGCCCTGGCC

CGGGCCTCGGGCCGGGGAGGAAGAGTAGCTCGCCGAGGCGCCGAGGAGAGCGGGCCGCCCCACAGCCCGA

GCCGGAGAGGGAGCGCGAGCCGCGCCGGCCCCGGTCGGGCCTCCGAAACCATGAACTTTCTGCTGTCTTG

GGTGCATTGGAGCCTTGCCTTGCTGCTCTACCTCCACCATGCCAAGTGGTCCCAGGCTGCACCCATGGCA

GAAGGAGGAGGGCAGAATCATCACGAAGTGGTGAAGTTCATGGATGTCTATCAGCGCAGCTACTGCCATC

CAATCGAGACCCTGGTGGACATCTTCCAGGAGTACCCTGATGAGATCGAGTACATCTTCAAGCCATCCTG

TGTGCCCCTGATGCGATGCGGGGGCTGCTGCAATGACGAGGGCCTGGAGTGTGTGCCCACTGAGGAGTCC

AACATCACCATGCAGATTATGCGGATCAAACCTCACCAAGGCCAGCACATAGGAGAGATGAGCTTCCTAC

AGCACAACAAATGTGAATGCAGACCAAAGAAAGATAGAGCAAGACAAGAAAAAAAATCAGTTCGAGGAAA

GGGAAAGGGGCAAAAACGAAAGCGCAAGAAATCCCGGTATAAGTCCTGGAGCGTGTACGTTGGTGCCCGC

TGCTGTCTAATGCCCTGGAGCCTCCCTGGCCCCCATCCCTGTGGGCCTTGCTCAGAGCGGAGAAAGCATT

TGTTTGTACAAGATCCGCAGACGTGTAAATGTTCCTGCAAAAACACAGACTCGCGTTGCAAGGCGAGGCA

GCTTGAGTTAAACGAACGTACTTGCAGATGTGACAAGCCGAGGCGGTGA

>lcl|NM_030938.5_cds_NP_112200.2_1 [gene=VMP1] [db_xref=CCDS:CCDS11619.1] [protein=vacuole membrane protein 1 isoform 1] [protein_id=NP_112200.2] [location=122..1342] [gbkey=CDS]

ATGGCAGAGAATGGAAAAAATTGTGACCAGAGACGTGTAGCAATGAACAAGGAACATCATAATGGAAATT

TCACAGACCCCTCTTCAGTGAATGAAAAGAAGAGGAGGGAGCGGGAAGAAAGGCAGAATATTGTCCTGTG

GAGACAGCCGCTCATTACCTTGCAGTATTTTTCTCTGGAAATCCTTGTAATCTTGAAGGAATGGACCTCA

AAATTATGGCATCGTCAAAGCATTGTGGTGTCTTTTTTACTGCTGCTTGCTGTGCTTATAGCTACGTATT

ATGTTGAAGGAGTGCATCAACAGTATGTGCAACGTATAGAGAAACAGTTTCTTTTGTATGCCTACTGGAT

AGGCTTAGGAATTTTGTCTTCTGTTGGGCTTGGAACAGGGCTGCACACCTTTCTGCTTTATCTGGGTCCA

CATATAGCCTCAGTTACATTAGCTGCTTATGAATGCAATTCAGTTAATTTTCCCGAACCACCCTATCCTG

ATCAGATTATTTGTCCAGATGAAGAGGGCACTGAAGGAACCATTTCTTTGTGGAGTATCATCTCAAAAGT

TAGGATTGAAGCCTGCATGTGGGGTATCGGTACAGCAATCGGAGAGCTGCCTCCATATTTCATGGCCAGA

GCAGCTCGCCTCTCAGGTGCTGAACCAGATGATGAAGAGTATCAGGAATTTGAAGAGATGCTGGAACATG

CAGAGTCTGCACAAGACTTTGCCTCCCGGGCCAAACTGGCAGTTCAAAAACTAGTACAGAAAGTTGGATT

TTTTGGAATTTTGGCCTGTGCTTCAATTCCAAATCCTTTATTTGATCTGGCTGGAATAACGTGTGGACAC

TTTCTGGTACCTTTTTGGACCTTCTTTGGTGCAACCCTAATTGGAAAAGCAATAATAAAAATGCATATCC

AGAAAATTTTTGTTATAATAACATTCAGCAAGCACATAGTGGAGCAAATGGTGGCTTTCATTGGTGCTGT

CCCCGGCATAGGTCCATCTCTGCAGAAGCCATTTCAGGAGTACCTGGAGGCTCAACGGCAGAAGCTTCAC

CACAAAAGCGAAATGGGCACACCACAGGGAGAAAACTGGTTGTCCTGGATGTTTGAAAAGTTGGTCGTTG

TCATGGTGTGTTACTTCATCCTATCTATCATTAACTCCATGGCACAAAGTTATGCCAAACGAATCCAGCA

GCGGTTGAACTCAGAGGAGAAAACTAAATAA

>lcl|NM_014991.6_cds_NP_055806.2_1 [gene=WDFY3] [db_xref=CCDS:CCDS3609.1] [protein=WD repeat and FYVE domain-containing protein 3] [protein_id=NP_055806.2] [location=708..11288] [gbkey=CDS]

ATGAACATGGTGAAGAGGATCATGGGGCGGCCGAGGCAGGAGGAGTGCAGCCCACAAGACAACGCCTTAG

GACTGATGCACCTCCGCCGGCTCTTCACGGAGTTGTGCCATCCTCCCCGGCACATGACTCAGAAGGAACA

AGAAGAGAAACTGTATATGATGCTGCCAGTGTTTAACAGGGTTTTTGGAAATGCTCCGCCGAATACAATG

ACAGAAAAAATTTTCTGATCTTCTGCAGTTCACAACACAAGTCTCACGACTAATGGTGACAGAAATTCGAA

GGAGAGCATCAAACAAATCCACAGAGGCTGCAAGTCGGGCCATAGTTCAGTTCCTAGAGATTAATCAGAG

TGAAGAAGCCAGTAGAGGCTGGATGCTTCTAACGACAATTAATTTGTTAGCTTCCTCTGGTCAGAAAACC

GTGGACTGCATGACAACAATGTCAGTGCCTTCCACCCTGGTTAAATGTTTATATCTGTTTTTTGACCTTC

CACATGTGCCTGAGGCAGTTGGAGGTGCACAGAATGAGCTACCTCTAGCAGAACGTCGAGGACTACTCCA

GAAAGTTTTTGTACAGATCTTAGTGAAACTGTGCAGTTTTGTTTCCCCTGCGGAGGAGCTGGCTCAGAAA

GATGATCTCCAGCTTCTATTCAGTGCAATAACCTCTTGGTGCCCTCCCTATAACCTGCCTTGGAGAAAGA

GTGCTGGAGAAGTCCTCATGACCATATCTCGTCATGGTCTTAGTGTCAATGTAGTGAAGTATATTCATGA

GAAAGAGTGTTTATCTACATGTGTTCAGAATATGCAGCAATCAGATGACCTGTCTCCCCTAGAAATTGTC

GAAATGTTTGCTGGGCTTTCTTGTTTCCTCAAAGATTCCAGCGATGTTTCCCAAACACTTCTGGATGATT

TTCGGATATGGCAAGGATATAATTTTCTTTGTGATCTCTTGCTTAGATTGGAACAAGCAAAAGAGGCAGA

ATCCAAAGATGCCTTGAAAGATCTGGTTAATCTGATAACTTCCCTAACAACATATGGTGTCAGTGAACTA

AAACCAGCTGGTATTACCACAGGGGCACCCTTTTTATTGCCTGGATTTGCAGTACCTCAGCCTGCAGGCA

AAGGTCACAGTGTGAGAAACGTCCAGGCCTTTGCAGTTCTTCAGAATGCATTTTTAAAAGCAAAAACCAG

CTTCCTTGCCCAAATCATCCTTGATGCTATCACAAATATTTACATGGCTGACAATGCCAATTACTTCATC

CTAGAGTCACAGCACACATTGTCACAGTTTGCAGAGAAGATTTCTAAACTCCCAGAAGTACAAAACAAAT

ACTTTGAGATGCTGGAGTTTGTTGTTTTTAGCTTAAATTATATACCTTGTAAAGAACTTATTAGTGTCAG

TATCCTCTTAAAATCTAGCTCTTCTTATCACTGTAGCATTATTGCAATGAAAACACTTCTTAAGTTTACA

AGACATGACTACATATTTAAAGACGTGTTCAGGGAGGTTGGCCTTTTGGAGGTCATGGTAAACCTTTTGC

ATAAATATGCTGCCCTGTTGAAGGATCCAACTCAGGCACTAAATGAACAAGGGGACTCAAGAAATAATAG

TTCAGTTGAAGACCAAAAACACCTGGCTTTATTGGTTATGGAGACCTTGACAGTGCTTCTTCAAGGATCA

AACACAAATGCAGGAATTTTTCGAGAATTTGGAGGTGCAAGATGTGCACATAATATAGTAAAGTACCCTC

AATGCCGGCAGCATGCCTTGATGACTATCCAACAGCTGGTGCTCTCCCCAAATGGGGACGATGACATGGG

CACTCTCCTGGGGCTAATGCATTCAGCCCCACCGACGGAATTGCAGTTGAAGACTGATATTTTAAGGGCC

CTCCTGTCGGTCCTTCGAGAAAGCCATCGTTCAAGAACAGTTTTTAGGAAAGTTGGAGGATTTGTGTACA

TTACATCCTTGCTCGTTGCTATGGAAAGATCTTTGAGCTGTCCACCCAAGAATGGCTGGGAGAAAGTGAA

CCAGAATCAAGTGTTTGAACTTCTTCACACTGTGTTCTGCACGTTGACTGCAGCAATGCGCTATGAGCCA

GCCAACTCTCATTTCTTCAAAACAGAGATTCAGTATGAGAAGTTGGCAGATGCTGTTCGATTTCTTGGCT

GCTTCTCAGACCTAAGAAAAATAAGCGCCATGAATGTCTTCCCCTCAAATACACAGCCATTTCAAAGACT

TTTAGAGGAAGATGTAATCTCAATAGAATCAGTGTCACCCACGTTACGGCACTGCAGTAAACTTTTTATT

TATCTTTACAAAGTAGCCACAGATTCTTTTGACAGTCGTGCAGAACAGATCCCTCCTTGCCTGACAAGTG

AGTCTTCTCTCCCCTCTCCTTGGGGTACACCAGCTTTGTCCAGGAAAAGGCATGCATATCATTCTGTTTC

AACTCCCCCTGTTTACCCTCCTAAAAATGTTGCCGACCTGAAACTACATGTGACAACTTCATCTCTGCAG

AGTTCTGATGCAGTCATCATTCATCCTGGAGCCATGCTTGCCATGCTGGACCTACTGGCCTCTGTTGGGT

CAGTGACACAGCCAGAACATGCTTTGGATCTTCAACTTGCCGTGGCAAATATTTTACAATCCCTGGTGCA

CACAGAAAGGAACCAGCAAGTCATGTGTGAAGCTGGTCTTCATGCACGACTGCTGCAGAGGTGCAGTGCT

GCATTGGCTGATGAGGACCACTCACTGCACCCGCCCCTGCAGCGGATGTTTGAACGATTAGCCTCTCAGG

CTCTGGAACCCATGGTGTTGAGGGAGTTTTTACGTTTGGCAAGTCCTTTAAATTGTGGTGCCTGGGACAA

AAAACTGCTAAAACAATATAGGGTCCACAAACCAAGTTCACTGAGTTATGAACCAGAAATGAGAAGTAGT

ATGATCACATCTCTGGAAGGTCTGGGTACTGATAATGTTTTTAGCTTACATGAAGATAACCATTACCGGA

TAAGCAAGAGCCTGGTAAAATCTGCGGAAGGAAGTACTGTACCCCTGACCAGGGTGAAGTGTCTGGTCTC

CATGACAACCCCACATGACATCAGACTTCATGGGTCATCAGTTACTCCAGCTTTTGTTGAATTTGACACA

TCACTTGAAGGGTTTGGATGTCTTTTTTTGCCCAGTTTGGCCCCTCATAATGCTCCTACAAATAATACCG

TCACAACAGGTCTTATTGATGGGGCTGTGGTCAGTGGCATTGGTTCTGGTGAAAGATTCTTCCCTCCTCC

CTCCGGCTTAAGTTACTCTAGCTGGTTTTGTATTGAACATTTTAGTTCTCCTCCAAATAACCACCCTGTC

AGACTTCTTACTGTTGTGCGCCGAGCAAATTCTTCTGAGCAACATTACGTGTGCCTTGCAATAGTTCTAT

CAGCAAAAGACCGATCTCTGATTGTTTCCACCAAAGAGGAACTCCTCCAAAATTATGTTGATGATTTTAG

TGAAGAGTCCTCATTTTATGAAATTCTCCCATGCTGTGCTCGCTTTCGATGTGGAGAGCTTATCATTGAG

GGACAGTGGCATCATTTGGTCCTGGTAATGAGCAAAGGCATGTTGAAAAACAGTACTGCAGCCCTTTATA

TTGATGGACAGCTTGTTAACACTGTAAAGCTTCATTATGTCCACAGTACTCCAGGGGGTTCAGGTTCGGC

AAATCCACCAGTGGTGAGCACGGTCTATGCCTACATTGGTACTCCACCTGCCCAACGCCAAATTGCCTCA

TTGGTTTGGCGCCTGGGACCCACACATTTTCTAGAAGAAGTTTTACCTTCTTCAAATGTTACTACCATTT

ATGAACTTGGACCAAATTATGTTGGAAGCTTTCAGGCTGTATGTATGCCATGTAAAGATGCAAAATCCGA

AGGGGTGGTGCCATCCCCTGTGTCATTAGTACCAGAGGAGAAAGTGTCATTTGGCCTCTATGCACTCTCT

GTGTCGTCTCTAACAGTGGCAAGAATCCGGAAAGTGTATAACAAATTGGATAGCAAAGCCATTGCTAAGC

AGTTAGGCATTTCCTCACATGAGAATGCCACTCCTGTGAAGTTGATACACAATTCAGCAGGACATCTTAA

TGGATCTGCACGGACAATTGGGGCCGCTCTGATTGGATACTTGGGAGTAAGAACATTTGTCCCTAAGCCT

GTTGCCACTACTTTGCAGTACGTTGGTGGAGCTGCAGCCATCCTGGGCCTGGTGGCCATGGCCTCTGATG

TGGAAGGGTTATATGCAGCAGTCAAGGCCCTGGTTTGTGTGGTCAAGAGTAACCCACTAGCCAGCAAAGA

AATGGAAAGAATCAAGGGCTACCAGTTGCTGGCAATGTTGCTTAAGAAGAAACGTTCCCTTCTTAACAGC

CACATCCTCCATCTAACTTTTTCTTTGGTGGGAACTGTTGATAGTGGACATGAGACCTCCATTATTCCAA

ATTCAACTGCTTTCCAGGACCTCCTCTGTGATTTTGAAGTCTGGCTCCATGCACCATATGAACTTCATCT

TTCCTTATTTGAACACTTTATTGAACTGCTCACAGAGTCCAGTGAAGCCTCAAAGAATGCCAAATTAATG

AGAGAATTCCAGTTAATCCCAAAGCTGCTCCTGACTCTTCGAGATATGTCTTTATCCCAGCCTACTATTG

CTGCTATTAGTAATGTCCTGAGCTTCTTACTGCAAGGTTTTCCTAGCAGCAATGATCTGCTCAGATTTGG

GCAGTTTATTTCTTCTACTTTGCCAACCTTTGCGGTTTGTGAGAAATTTGTAGTAATGGAAATAAATAAT

GAAGAGAAGCTTGACACTGGAACTGAAGAGGAGTTTGGAGGTCTTGTATCAGCTAATCTTATACTTTTGA

GGAACAGACTTCTGGATATCTTGCTAAAACTAATTTATACATCTAAAGAAAAGACAAGCATTAATTTGCA

AGCTTGTGAAGAACTGGTGAAGACACTGGGTTTTGACTGGATCATGATGTTTATGGAGGAACACTTACAT

TCCACCACAGTTACAGCAGCCATGAGGATTCTTGTTGTCCTACTAAGTAATCAGTCTATTCTCATCAAGT

TTAAAGAAGGACTCAGTGGTGGAGGATGGCTTGAACAGACAGATTCTGTCTTAACTAATAAGATTGGAAC

TGTATTAGGATTCAACGTGGGCAGAAGTGCTGGTGGGAGATCGACGGTCAGGGAGATTAACCGAGATGCT

TGTCATTTTCCTGGTTTTCCAGTCCTTCAGTCATTCCTTCCTAAACACACTAATGTCCCTGCCCTCTATT

TTCTCCTCATGGCCTTGTTTCTGCAGCAGCCAGTTAGTGAGCTGCCTGAGAACCTGCAGGTCAGTGTGCC

TGTCATCAGCTGCCGGAGTAAGCAGGGTTGCCAGTTTGATTTGGATTCCATTTGGACATTCATCTTTGGA

GTTCCTGCCTCCAGCGGAACTGTGGTCTCTTCTATCCATAACGTATGCACAGAAGCTGTTTTTTTATTAT

TGGGAATGCTCCGCAGCATGCTGACTTCACCTTGGCAATCAGAAGAAGAGGGATCTTGGCTCCGAGAATA

TCCTGTGACCCTGATGCAGTTCTTCAGATATTTGTATCACAACGTGCCAGACCTTGCCTCCATGTGGATG

AGCCCTGACTTCCTGTGTGCATTAGCAGCCACCGTCTTCCCCTTCAATATTCGCCCTTACTCAGAGATGG

TGACTGACCTTGATGATGAAGTTGGATCTCCAGCAGAAGAGTTTAAAGCGTTTGCAGCAGACACAGGGAT

GAACAGGAGCCAATCAGAGTACTGCAATGTGGGCACCAAGACATATCTGACCAATCACCCGGCTAAAAAG

TTCGTTTTTGACTTCATGCGGGTCTTAATCATAGACAACCTCTGTCTCACTCCTGCCAGCAAGCAAACTC

CACTAATTGATCTTTTGTTGGAGGCTTCCCCTGAAAGGTCTACAAGAACTCAGCAAAAAGAATTTCAAAC

TTACATTTTGGATAGCGTGATGGACCATTTGCTTGCAGCTGATGTGTTATTAGGGGAAGATGCATCTCTG

CCTATTACCAGTGGAGGAAGCTACCAGGTATTGGTGAACAATGTGTTTTATTTCACACAGCGTGTGGTGG

ACAAGCTTTGGCAAGGCATGTTCAACAAAGAATCTAAACTTCTTATAGATTTTATAATTCAACTAATTGC

ACAGTCAAAGAGAAGATCACAGGGATTGTCACTGGATGCAGTGTATCATTGCCTCAATAGGACCATCTTG

TACCAGTTCTCACGGGCACACAAAACCGTTCCTCAGCAAGTAGCTCTGCTTGATTCACTCAGGGTCCTCA

CTGTAAACAGAAACTTGATCCTGGGACCTGGGAACCATGACCAAGAATTCATTAGCTGTCTGGCCCACTG

CTTGATAAATCTACATGTTGGAAGCAACGTGGATGGATTTGGACTGGAAGCAGAAGCCCGCATGACCACA

TGGCACATTATGATCCCCTCGGACATTGAACCAGATGGTAGTTACAGCCAAGATATTAGTGAAGGGCGTC

AGCTTCTCATAAAAGCTGTCAACAGAGTTTGGACTGAACTGATACATAGTAAGAAACAAGTCTTAGAGGA

ACTTTTCAAAGTAACTCTACCTGTGAATGAAAGGGGCCACGTGGACATAGCTACAGCAAGGCCACTCATT

GAAGAAGCTGCCCTGAAGTGCTGGCAGAATCATTTGGCCCATGAAAAGAAATGCATAAGTCGAGGAGAAG

CTTTAGCGCCCACCACACAGTCCAAATTATCCCGTGTCAGCAGTGGCTTTGGTCTTTCCAAGTTAACAGG

ATCAAGAAGGAATCGAAAAGAAAGTGGTCTTAATAAACACAGTCTTTCCACCCAGGAGATTTCGCAGTGG

ATGTTTACTCACATTGCTGTTGTTCGTGACTTAGTAGATACACAATATAAAGAATATCAGGAGCGTCAGC

AGAATGCCCTGAAGTACGTGACAGAAGAGTGGTGTCAGATCGAGTGCGAGCTGTTGAGGGAGCGGGGGCT

GTGGGGCCCTCCCATCGGCTCCCACCTCGACAAGTGGATGCTGGAGATGACAGAAGGGCCCTGCAGGATG

AGGAAAAAGATGGTGCGAAATGATATGTTTTATAACCATTACCCTTACGTGCCAGAAACTGAGCAAGAGA

CAAATGTGGCGTCTGAGATCCCAAGTAAACAGCCTGAGACACCCGATGATATTCCTCAAAAGAAACCTGC

TCGATATAGAAGAGCCGTAAGTTATGACAGTAAAGAGTACTACATGCGACTGGCCTCTGGCAATCCCGCC

ATTGTCCAAGACGCCATTGTGGAGAGTTCAGAAGGTGAAGCTGCTCAGCAAGAACCAGAGCATGGGGAAG

ACACTATTGCTAAAGTCAAAGGTTTGGTCAAGCCTCCTCTAAAACGCTCCCGATCTGCACCTGATGGAGG

AGATGAGGAGAACCAGGAGCAGCTACAAGACCAGATTGCTGAGGGCAGCTCCATAGAAGAGGAGGAGAAA

ACAGATAATGCTACCTTACTGCGCCTGTTAGAGGAAGGAGAAAAGATCCAACACATGTACCGCTGTGCTC

GAGTCCAGGGCCTAGATACCAGTGAGGGGCTCCTTCTTTTTGGTAAAGAGCATTTTTATGTGATTGATGG

ATTTACCATGACAGCAACCAGGGAAATAAGAGATATTGAAACCTTACCTCCAAATATGCATGAGCCTATT

ATTCCTAGAGGAGCCAGGCAAGGCCCTAGTCAACTCAAGAGAACATGCAGCATTTTTGCATATGAAGATA

TCAAGGAAGTTCATAAAAGGAGATATCTCCTGCAGCCTATTGCTGTGGAAGTTTTCTCTGGAGATGGACG

GAATTACCTCCTTGCTTTTCAGAAAGGAATCAGAAACAAAGTCTATCAAAGGTTTTTGGCTGTAGTGCCA

TCTCTAACGGACAGTTCAGAATCTGTATCTGGGCAACGACCAAACACGAGTGTGGAGCAGGGATCTGGGT

TACTTAGCACTTTGGTTGGAGAGAAGTCTGTGACTCAGAGATGGGAGAGAGGTGAAATCAGCAACTTCCA

ATATTTGATGCATTTGAACACTTTGGCTGGCAGATCATATAATGATCTCATGCAGTATCCTGTCTTCCCC

TGGATCCTTGCAGATTATGACTCAGAGGAGGTGGATCTTACTAATCCCAAGACGTTTAGAAACCTGGCTA

AGCCAATGGGAGCACAAACAGATGAACGATTAGCTCAGTATAAGAAGCGGTATAAAGACTGGGAGGATCC

TAATGGAGAAACTCCTGCATACCACTATGGGACCCACTATTCATCTGCAATGATTGTGGCCTCATACCTT

GTAAGGATGGAGCCTTTCACACAGATATTCTTAAGGCTACAGGGTGGCCACTTTGACCTGGCTGACCGGA

TGTTTCACAGTGTGCGCGAGGCCTGGTATTCAGCGTCAAAGCACAATATGGCAGATGTAAAAGAACTTAT

CCCAGAGTTCTTTTATTTACCAGAATTCCTGTTCAATTCCAACAACTTTGATCTAGGCTGTAAACAAAAT

GGCACCAAGCTTGGAGATGTTATCCTTCCACCCTGGGCAAAAGGGGACCCACGAGAATTCATCAGAGTCC

ATCGTGAGGCTTTGGAGTGTGATTACGTGAGTGCCCATCTACATGAGTGGATTGACTTAATCTTCGGTTA

TAAACAGCAAGGCCCTGCTGCAGTAGAAGCTGTAAATGTCTTCCATCATCTTTTTTATGAGGGTCAAGTG

GATATCTACAACATCAATGACCCACTAAAGGAGACAGCCACAATTGGGTTCATTAATAACTTCGGTCAGA

TCCCTAAACAGTTATTTAAAAAACCTCATCCACCAAAGCGAGTGAGAAGTCGACTCAATGGAGACAATGC

AGGAATCTCTGTCCTACCAGGATCTACAAGTGACAAGATCTTTTTTCATCATCTAGACAACTTGAGGCCT

TCTCTAACACCTGTAAAAGAACTCAAAGAACCTGTAGGACAAATCGTATGTACAGATAAAGGTATTCTTG

CGGTGGAACAGAATAAGGTTCTTATCCCACCAACCTGGAATAAAACTTTTGCTTGGGGCTATGCAGACCT

CAGTTGCAGACTGGGAACCTATGAGTCAGACAAGGCCATGACTGTTTATGAATGCTTGTCTGAGTGGGGC

CAGATTCTCTGTGCAATCTGCCCCAACCCCAAGCTGGTCATCACGGGTGGAACAAGCACGGTTGTGTGTG

TGTGGGAGATGGGCACCTCCAAAGAAAAGGCCAAGACCGTCACCCTCAAACAGGCCTTACTGGGCCACAC

TGATACCGTCACCTGCGCCACAGCATCATTAGCCTATCACATAATTGTCAGTGGGTCCCGTGATCGAACC

TGTATCATTTGGGATTTGAACAAACTGTCATTTCTAACCCAGCTTCGAGGGCATCGAGCTCCAGTTTCTG

CTCTTTGTATCAATGAATTAACAGGGGACATTGTGTCCTGCGCTGGCACATATATCCATGTGTGGAGCAT

CAATGGGAACCCTATCGTGAGTGTCAACACGTTCACAGGTAGGAGCCAGCAGATCATCTGCTGCTGCATG

TCGGAGATGAACGAATGGGACACGCAGAACGTCATAGTGACAGGACACTCAGATGGAGTGGTTCGGTTTT

GGAGAATGGAATTTTTGCAAGTTCCTGAAACACCAGCTCCTGAGCCTGCTGAAGTCCTAGAAATGCAGGA

AGACTGTCCAGAAGCACAAATAGGGCAGGAAGCCCAAGACGAGGACAGCAGTGATTCAGAAGCAGATGAG

CAGAGCATCAGCCAGGACCCTAAGGACACTCCAAGCCAACCCAGCAGCACCAGCCACAGGCCCCGGGCAG

CCTCCTGCCGCGCAACAGCCGCCTGGTGTACTGACAGTGGCTCTGACGACTCCAGACGCTGGTCCGACCA

GCTCAGTCTAGATGAGAAAGACGGCTTCATATTTGTGAACTATTCAGAGGGCCAGACCAGAGCCCATCTG

CAGGGCCCCCTTAGCCACCCCCACCCCAATCCCATTGAGGTGCGGAATTACAGCAGATTGAAACCTGGGT

ACCGATGGGAACGGCAGCTGGTGTTCAGGAGTAAGCTGACTATGCACACAGCCTTTGATCGAAAGGACAA

TGCACACCCAGCTGAGGTCACTGCCTTGGGCATCTCCAAGGATCACAGTAGGATCCTCGTTGGTGACAGT

CGAGGCCGAGTTTTCAGCTGGTCTGTGAGTGACCAGCCAGGCCGTTCTGCTGCTGATCACTGGGTGAAGG

ATGAAGGTGGTGACAGCTGCTCAGGCTGCTCGGTGAGGTTTTCACTCACAGAAAGACGACACCATTGCAG

GAACTGTGGTCAGCTCTTCTGCCAGAAGTGCAGTCGCTTTCAATCTGAAATCAAACGCTTGAAAATCTCA

TCCCCGGTGCGTGTTTGTCAGAACTGTTATTATAACTTACAGCATGAGAGAGGTTCAGAAGATGGGCCTC

GAAATTGTTGA

>lcl|NM_007075.4_cds_NP_009006.2_1 [gene=WDR45] [db_xref=CCDS:CCDS14318.1] [protein=WD repeat domain phosphoinositide-interacting protein 4 isoform 1] [protein_id=NP_009006.2] [location=497..1582] [gbkey=CDS]

ATGACTCAACAGCCACTTCGAGGAGTGACCAGCCTGCGTTTCAACCAAGACCAAAGCTGCTTTTGCTGCG

CCATGGAGACAGGTGTGCGCATCTACAACGTGGAGCCCTTGATGGAGAAGGGGCATCTGGACCACGAGCA

GGTGGGCAGCATGGGCTTGGTGGAGATGCTGCACCGCTCCAACCTTCTGGCCTTGGTGGGCGGTGGTAGT

AGTCCCAAGTTCTCAGAGATCTCAGCAGTGCTGATCTGGGACGATGCCCGGGAGGGCAAGGACTCCAAGG

AGAAGCTGGTGCTGGAGTTCACCTTCACCAAGCCAGTGCTTTCTGTGCGCATGCGCCATGACAAGATCGT

GATCGTGCTGAAGAACCGCATCTATGTGTACTCCTTCCCCGACAATCCCCGAAAGCTGTTTGAGTTTGAT

ACCCGGGACAACCCCAAGGGGCTCTGTGACCTCTGCCCCAGCCTGGAGAAGCAACTGCTAGTGTTCCCGG

GACACAAGTGTGGGAGTCTGCAACTTGTGGACCTGGCGAGCACAAAGCCTGGCACCTCGTCTGCTCCATT

CACGATCAATGCACATCAGAGTGACATAGCCTGTGTGTCTCTAAACCAGCCAGGCACTGTAGTGGCCTCA

GCCTCCCAGAAGGGTACCCTTATTCGCCTCTTTGACACACAATCCAAGGAGAAACTGGTGGAGCTGCGCC

GAGGCACTGACCCTGCCACCCTCTACTGCATTAACTTCAGCCACGACTCCTCCTTCCTCTGCGCTTCCAG

TGATAAGGGTACTGTCCATATCTTTGCTCTCAAGGATACCCGCCTCAACCGCCGCTCCGCGCTGGCTCGC

GTGGGCAAGGTGGGGCCTATGATTGGGCAGTACGTGGACTCTCAGTGGAGCCTGGCGAGCTTCACTGTGC

CTGCTGAGTCAGCTTGCATCTGCGCCTTCGGTCGCAATACTTCCAAGAACGTCAACTCTGTCATTGCCAT

CTGCGTAGATGGGACCTTCCACAAATATGTCTTCACTCCTGATGGAAACTGCAACAGAGAGGCTTTCGAC

GTGTACCTTGACATCTGTGATGATGATGACTTTTAA

>lcl|NM_019613.4_cds_NP_062559.2_1 [gene=WDR45B] [db_xref=CCDS:CCDS11815.2] [protein=WD repeat domain phosphoinositide-interacting protein 3] [protein_id=NP_062559.2] [location=105..1139] [gbkey=CDS]

ATGAACCTCCTGCCGTGTAACCCTCACGGCAACGGGCTGCTCTACGCCGGCTTCAACCAGGACCACGGAT

GCTTTGCGTGTGGGATGGAAAATGGATTCCGAGTCTATAACACTGATCCACTAAAAGAAAAAGAGAAACA

AGAATTTCTAGAAGGAGGAGTTGGCCATGTTGAAATGTTATTTCGCTGCAACTATTTAGCTTTAGTTGGT

GGTGGAAAAAAGCCGAAATACCCTCCCAACAAAGTAATGATCTGGGATGACCTGAAGAAGAAGACTGTTA

TTGAAATAGAATTTTCTACAGAAGTCAAGGCAGTCAAGCTGCGGCGAGATAGAATTGTGGTGGTTTTGGA

CTCCATGATTAAGGTGTTCACATTCACACACAATCCCCATCAGTTGCACGTCTTCGAAACCTGCTATAAC

CCCAAAGGCCTCTGTGTCCTTTGTCCCAATAGTAACAACTCCCTCCTGGCCTTTCCGGGCACGCACACGG

GCCATGTGCAGCTTGTGGACCTGGCCAGCACGGAGAAGCCACCCGTGGACATTCCTGCACACGAGGGTGT

CCTGAGCTGCATTGCACTCAACCTGCAGGGAACAAGAATTGCAACTGCATCCGAGAAAGGGACGCTTATA

AGAATATTTGATACTTCATCAGGGCATTTAATCCAGGAACTGCGAAGAGGATCTCAAGCAGCCAATATTT

ACTGCATCAACTTCAATCAGGATGCGTCCCTCATCTGCGTATCCAGCGACCACGGCACAGTGCATATTTT

TGCAGCTGAAGATCCAAAAAGGAATAAACAGTCCAGTTTGGCCTCAGCCAGTTTCCTTCCAAAATACTTC

AGTTCCAAGTGGAGTTTCTCCAAGTTTCAGGTTCCCTCAGGCTCTCCGTGCATTTGTGCCTTTGGAACAG

AGCCAAACGCCGTCATTGCAATTTGTGCAGACGGCAGCTACTACAAATTCCTGTTCAACCCCAAGGGGGA

GTGCATCCGAGATGTCTACGCGCAGTTTCTAGAGATGACCGATGACAAGCTGTGA

>lcl|NM_017983.7_cds_NP_060453.3_1 [gene=WIPI1] [db_xref=CCDS:CCDS11677.1] [protein=WD repeat domain phosphoinositide-interacting protein 1 isoform a] [protein_id=NP_060453.3] [location=76..1416] [gbkey=CDS]

ATGGAGGCCGAGGCCGCGGACGCTCCCCCGGGCGGGGTTGAGTCGGCGCTCAGCTGCTTCTCTTTCAACC

AGGACTGCACATCCCTAGCAACTGGAACTAAAGCCGGGTATAAGCTGTTTTCTCTGAGTTCTGTGGAGCA

GCTGGATCAAGTCCACGGAAGCAATGAAATCCCGGACGTCTACATCGTGGAGCGCCTCTTCTCCAGCAGC

CTGGTGGTGGTAGTCAGTCACACAAAACCACGGCAGATGAACGTGTATCACTTCAAGAAAGGCACAGAGA

TCTGTAATTACAGCTACTCCAGCAACATCTTGTCCATAAGGCTGAACCGGCAAAGGCTGCTGGTTTGCCT

AGAAGAGTCCATTTATATTCACAACATTAAAGACATGAAGCTGTTGAAGACCCTCCTGGATATTCCTGCA

AACCCAACAGGTCTATGTGCTCTCTCTATCAACCATTCCAATTCTTACCTGGCCTATCCTGGAAGCCTGA

CTTCAGGGGAGATTGTGCTTTATGATGGAAACTCCCTGAAAACAGTCTGCACTATTGCTGCCCATGAGGG

AACACTAGCTGCCATCACCTTCAATGCCTCAGGCTCCAAACTAGCAAGTGCGTCTGAAAAAGGCACAGTC

ATCCGGGTGTTCTCTGTCCCTGATGGGCAAAAGCTCTATGAGTTCCGGAGAGGGATGAAAAGGTATGTGA

CAATCAGCTCTCTAGTGTTCAGTATGGATTCACAATTCCTCTGCGCCTCCAGTAACACCGAGACGGTACA

CATCTTCAAGCTGGAACAGGTCACCAACAGTCGACCAGAAGAGCCTTCGACCTGGAGTGGCTACATGGGA

AAGATGTTTATGGCTGCTACCAACTACCTCCCTACCCAGGTGTCAGACATGATGCATCAGGACAGGGCTT

TTGCCACTGCACGCTTGAACTTCTCCGGACAGAGGAACATCTGTACCCTCTCAACGATCCAGAAGTTGCC

ACGGCTGCTAGTTGCGTCATCCAGTGGACACCTTTATATGTACAATTTGGATCCTCAGGATGGAGGAGAG

TGTGTCTTAATCAAAACCCACAGCTTGCTTGGCTCAGGAACAACAGAAGAGAATAAAGAAAATGACCTCA

GACCTTCCTTACCTCAGTCTTATGCAGCGACCGTAGCCAGACCAAGTGCATCTTCAGCCTCCACGGTGCC

AGGTTATTCTGAGGACGGCGGGGCGCTGCGAGGAGAAGTTATTCCTGAACATGAGTTTGCGACGGGACCA

GTGTGTCTTGATGATGAGAATGAGTTTCCTCCTATAATCTTGTGCCGTGGAAATCAGAAGGGCAAAACGA

AGCAGTCATGA

>lcl|NM_015610.4_cds_NP_056425.1_1 [gene=WIPI2] [db_xref=CCDS:CCDS5339.1] [protein=WD repeat domain phosphoinositide-interacting protein 2 isoform a] [protein_id=NP_056425.1] [location=188..1552] [gbkey=CDS]

ATGAACCTGGCGAGCCAGAGCGGGGAGGCCGGCGCCGGCCAGCTGCTCTTCGCCAACTTCAACCAGGACA

ACACAGAAGTGAAAGGGGCATCAAGAGCAGCTGGTCTTGGCCGTCGCGCTGTTGTCTGGTCCCTAGCTGT

TGGTAGTAAGTCCGGTTATAAATTTTTCTCCCTTTCTTCTGTGGATAAGCTGGAACAGATCTATGAATGC

ACCGATACGGAAGATGTGTGCATTGTAGAGAGATTGTTCTCCAGCAGCCTAGTGGCCATCGTCAGCCTTA

AAGCACCAAGGAAGCTAAAGGTTTGCCACTTTAAGAAGGGAACTGAGATCTGCAACTACAGCTACTCCAA

CACGATTCTGGCTGTGAAGCTCAACAGGCAGAGGCTGATAGTATGCCTGGAGGAGTCCCTGTACATCCAC

AACATTCGGGACATGAAGGTGCTGCATACGATCAGGGAGACGCCTCCAAACCCTGCAGGCCTGTGTGCGC

TGTCAATCAACAACGACAACTGCTACTTGGCGTACCCAGGGAGCGCGACCATCGGAGAGGTGCAGGTCTT

CGATACCATTAATTTGAGAGCTGCAAACATGATTCCGGCTCACGACAGTCCTTTAGCGGCACTGGCCTTT

GACGCAAGTGGAACTAAACTTGCCACGGCTTCGGAGAAGGGGACCGTGATTAGGGTATTTTCCATTCCAG

AAGGACAAAAACTCTTTGAGTTTCGGAGAGGAGTAAAGAGGTGCGTGAGCATCTGCTCCCTGGCCTTCAG

CATGGACGGCATGTTCCTCTCCGCCTCCAGCAACACTGAGACCGTGCACATCTTCAAACTCGAGACTGTG

AAAGAAAAACCCCCAGAGGAGCCCACCACCTGGACCGGGTACTTCGGGAAAGTGCTCATGGCCTCCACCA

GCTACCTGCCTTCCCAAGTGACAGAAATGTTCAACCAGGGCAGAGCCTTCGCCACGGTCCGCCTGCCATT

CTGCGGCCACAAAAACATCTGCTCGCTAGCCACAATTCAGAAGATCCCGCGGTTGTTGGTGGGTGCCGCC

GACGGGTACCTGTACATGTACAACCTGGACCCCCAGGAGGGCGGCGAGTGTGCCCTGATGAAGCAGCACC

GGCTGGACGGCAGTCTGGAAACGACCAATGAGATCTTGGACTCTGCCTCTCACGACTGCCCCTTAGTCAC

TCAGACATACGGCGCAGCTGCAGGAAAAGGTACTTACGTGCCTTCATCCCCAACGAGACTTGCCTACACA

GACGACCTGGGTGCTGTGGGTGGCGCCTGCCTGGAGGACGAGGCCAGCGCCCTGCGCCTGGATGAGGACA

GCGAGCACCCGCCCATGATTCTTCGGACTGACTGA

>lcl|NM_021260.4_cds_NP_067083.1_1 [gene=ZFYVE1] [db_xref=CCDS:CCDS9811.1] [protein=zinc finger FYVE domain-containing protein 1 isoform 1] [protein_id=NP_067083.1] [location=616..2949] [gbkey=CDS]

ATGAGTGCCCAGACTTCCCCAGCAGAGAAGGGCCTGAATCCGGGGCTGATGTGCCAGGAAAGTTACGCTT

GCAGCGGGACTGATGAAGCTATCTTTGAGTGTGATGAGTGCTGCAGTCTGCAGTGTCTCCGCTGCGAGGA

GGAGCTCCATCGGCAGGAGCGCCTGAGAAACCATGAGCGGATAAGACTCAAACCTGGCCATGTCCCTTAC

TGTGACCTCTGCAAGGGTCTCAGTGGGCATTTACCAGGTGTTAGGCAGAGGGCAATAGTGAGGTGCCAGA

CCTGCAAAATTAACTTGTGCCTGGAGTGCCAGAAGAGGACTCATTCTGGGGGTAACAAAAGGAGACACCC

TGTTACTGTGTACAATGTCAGTAATCTCCAGGAGTCACTGGAGGCAGAAGAGATGGATGAGGAGACCAAG

AGGAAGAAGATGACTGAGAAGGTTGTGAGTTTCCTCCTAGTAGACGAAAATGAAGAAATTCAGGTAACAA

ATGAAGAAGACTTTATTAGAAAATTGGACTGCAAACCTGATCAGCATCTGAAAGTGGTTTCCATTTTTGG

AAATACTGGTGATGGAAAGTCTCATACTCTCAACCACACTTTCTTTTATGGTCGTGAAGTCTTTAAAACC

TCCCCGACCCAGGAGTCCTGCACTGTGGGAGTGTGGGCAGCCTATGACCCAGTTCACAAAGTAGCAGTGA

TCGATACGGAAGGGCTCCTGGGGGCCACCGTGAATCTAAGCCAGAGAACACGGCTGCTGCTTAAGGTCCT

GGCCATCTCAGACCTCGTCATCTATCGAACTCATGCAGACCGGCTGCATAACGACCTCTTCAAATTCCTT

GGGGATGCCTCAGAAGCTTATCTGAAGCACTTCACCAAGGAGCTCAAGGCCACCACTGCTCGCTGTGGCC

TGGATGTCCCTTTATCCACACTGGGCCCTGCAGTTATCATCTTCCATGAGACCGTGCACACCCAGCTACT

GGGCTCTGATCATCCCTCAGAGGTGCCAGAGAAGCTCATCCAGGACCGGTTCCGGAAGCTGGGCCGTTTC

CCTGAAGCCTTTAGTTCCATTCACTACAAGGGAACGAGGACTTACAACCCTCCCACGGACTTTTCTGGGC

TTCGGCGTGCTTTGGAGCAGCTACTAGAGAATAACACCACCCGTTCTCCCCGGCACCCGGGAGTCATCTT

CAAAGCCCTGAAGGCACTAAGTGACCGCTTCAGCGGTGAGATCCCCGATGACCAGATGGCGCACAGCTCC

TTTTTTCCAGATGAGTATTTCACCTGCTCCTCCTTGTGCCTCAGCTGTGGGGTTGGATGTAAGAAAAGCA

TGAATCATGGGAAGGAAGGAGTGCCTCATGAAGCCAAGAGCCGCTGCAGATACTCCCACCAGTATGACAA

CCGAGTGTATACCTGCAAGGCCTGCTATGAGAGAGGCGAGGAAGTCAGTGTAGTGCCCAAAACATCTGCT

TCCACTGACTCCCCCTGGATGGGTCTCGCAAAATATGCCTGGTCTGGGTATGTGATCGAATGTCCTAACT

GTGGCGTGGTCTATCGTAGTCGGCAGTACTGGTTTGGAAACCAAGATCCTGTGGATACGGTGGTGCGGAC

AGAGATTGTGCATGTGTGGCCTGGAACTGATGGGTTTCTGAAGGACAACAACAATGCTGCCCAGCGCCTG

TTGGACGGGATGAACTTCATGGCTCAGTCGGTGTCCGAGCTTAGCCTTGGACCCACCAAGGCTGTGACTT

CCTGGCTGACAGACCAGATCGCCCCTGCCTACTGGAGGCCCAACTCCCAGATTCTGAGCTGCAACAAGTG

TGCGACGTCCTTTAAAGATAACGACACTAAGCATCACTGCCGAGCCTGTGGGGAGGGCTTCTGTGACAGC

TGTTCATCAAAGACTCGGCCAGTGCCTGAGCGGGGCTGGGGCCCTGCGCCAGTGCGGGTCTGTGACAACT

GCTACGAAGCCAGGAACGTCCAGTTAGCTGTTACCGAGGCACAAGTGGACGATGAAGGTGGAACGCTCAT

TGCTCGGAAGGTGGGCGAGGCCGTGCAGAACACTCTGGGAGCCGTGGTGACAGCCATTGACATACCACTA

GGTCTGGTAAAGGACGCGGCCAGGCCTGCGTACTGGGTGCCTGACCACGAAATCCTCCACTGCCACAACT

GCCGGAAGGAGTTCAGCATCAAGCTCTCCAAGCACCACTGCCGGGCCTGCGGACAGGGCTTCTGTGATGA

GTGCTCCCATGACCGCCGGGCTGTTCCTTCTCGTGGCTGGGACCATCCCGTCCGAGTCTGCTTCAACTGC

AATAAAAAGCCCGGTGACCTTTAA
