# Supplementary material for: Analysis of the Compositional Features and Codon Usage Pattern of Genes Involved in Human Autophagy
Source: Cells. 2022 Oct 12;11(20):3203. doi: 10.3390/cells11203203 (PMC9601114; doi:10.3390/cells11203203)
Supplement: Supplementary file 1 [file cells-11-03203-s001.zip › Supplementary Table S3.pdf]

Supplementary Table S3. Complete mRNA sequences of 224 human autophagy genes.

>NM_017749.3 Homo sapiens autophagy and beclin 1 regulator 1 (AMBRA1), transcript variant 2, mRNA

AGGGGATTAATGAAGAGCCACCAGACTATCTTTATGGAGAATCTAGATTGGGAAGTTAAGCTAAGAATGT

GCATTTTTGAAAAAGTTTTTATGGTAGAAGAGAATGTATATCAGAGGAAAGAATGTAGAGAAGAAAGCAA

CATTGTCCACTGGGAATTGATAAGTTCTGCGTAGAATTTAGAGTTATAAGGACTATCTCATTGGCTCCTA

ATGAATGCTAAATAACAAATGGAAATTGCTATTTCCATGAGGACAACTTACAAGGACCTGTATGGCCTCA

TTTTAGCTGCTTCAGTGTGTTGGAGCTGTTACTTGCTCCTTCATTTCCTGGGCCTGGTGTGACAGTGGCT

ACTGAGCGCCATGAAGGTTGTCCCAGAAAAGAATGCTGTCCGGATACTCTGGGGGCGAGAACGGGGTGCT

CGGGCCATGGGAGCTCAGCGGCTTCTGCAGGAGCTGGTAGAAGATAAAACCCGGTGGATGAAATGGGAGG

GCAAGAGAGTAGAACTGCCGGATAGTCCACGCTCTACCTTCTTATTGGCCTTCAGCCCAGACAGGACTCT

CTTAGCCTCCACCCATGTGAACCATAATATCTATATTACGGAGGTGAAGACTGGCAAGTGTGTTCATTCC

CTGATTGGACACCGCCGTACTCCATGGTGTGTCACTTTTCATCCCACCATCTCAGGCCTTATTGCTTCTG

GCTGCCTAGATGGGGAGGTTAGGATTTGGGATTTACACGGTGGCAGTGAAAGCTGGTTCACAGATAGCAA

CAATGCCATTGCCTCCCTGGCTTTCCACCCTACGGCTCAGCTCCTGCTGATTGCCACTGCCAATGAGATC

CACTTCTGGGACTGGAGTCGACGGGAACCCTTTGCTGTGGTGAAGACAGCTAGTGAGATGGAACGGGTCC

GTCTGGTGAGATTTGATCCACTTGGACACTACTTACTCACAGCAATTGTTAACCCCTCTAATCAACAGGG

TGATGACGAACCAGAGATCCCCATAGATGGAACAGAATTATCCCACTACCGTCAGCGTGCCCTCCTGCAA

TCACAGCCAGTTCGCCGGACGCCTCTCCTCCACAATTTCCTGCACATGCTGTCCTCCCGCTCTTCTGGCA

TCCAGACCGAGCCCTTCCATCCCCCGGAGCAGGCCTCGTCAACGCAGCAGGACCAGGGCCTCCTGAACCG

GCCGTCTGCCTTCAGTACAGTCCAGAGCAGCACTGCCGGCAACACGCTCCGCAACCTCAGTCTGGGTCCT

ACCCGCCGCTCTTTGGGAGGGCCTCTGTCTAGCCACCCTTCTAGGTATCACCGAGAAATAGCTCCTGGGT

TGACAGGATCTGAGTGGACCCGGACAGTACTCAGTCTGAACTCCCGCTCTGAGGCGGAATCCATGCCCCC

GCCCAGAACCAGTGCCTCTTCGGTGAGTTTGCTGTCTGTGCTGAGACAGCAGGAAGGTGGCTCTCAGGCA

TCTGTGTACACTTCAGCCACAGAAGGGAGGGGTTTTCCGGCATCAGGGTTGGCAACTGAGTCAGATGGAG

GGAATGGCTCCAGCCAAAACAACTCGGGCAGCATTCGCCATGAGCTTCAGTGTGACCTGAGACGCTTCTT

TCTGGAGTATGACCGGCTTCAGGAGCTGGATCAGAGCCTGAGTGGGGAAGCTCCCCAGACCCAACAGGCC

CAGGAAATGCTCAACAATAACATTGAATCTGAGAGGCCAGGCCCTTCCCACCAGCCCACCCCACACAGCA

GTGAGAACAACTCCAACCTGTCCCGTGGCCACCTGAATCGCTGTCGTGCTTGCCACAATCTCCTGACCTT

CAACAACGATACCCTGCGCTGGGAAAGAACCACACCTAACTACTCCTCTGGCGAGGCTAGTTCCTCTTGG

CAGGTCCCCAGCTCCTTTGAGAGTGTGCCATCAAGTGGCAGCCAGTTGCCACCTCTCGAGCGGACTGAGG

GCCAAACGCCCAGCTCCAGCAGGCTGGAGTTGAGCAGCTCTGCTAGTCCGCAGGAGGAGAGGACTGTGGG

GGTGGCCTTTAACCAGGAGACAGGCCACTGGGAAAGAATTTACACCCAGTCCAGCAGATCTGGAACTGTG

TCACAGGAGGCCTTACATCAGGATATGCCTGAGGAGAGCTCTGAGGAGGATTCACTCAGGAGGAGGCTGC

TGGAATCTTCCCTCATTTCATTATCCCGTTATGATGGAGCAGGATCCAGAGAGCACCCAATTTACCCAGA

CCCAGCGAGATTATCTCCTGCTGCATACTACGCCCAGAGGATGATCCAGTATCTCTCACGGAGAGACAGT

ATTCGCCAGCGCTCCATGCGCTACCAACAGAACCGTCTCCGTTCTTCCACCTCCTCCTCTTCCTCAGACA

ACCAGGGTCCATCAGTAGAGGGAACCGACTTGGAATTTGAGGACTTTGAGGACAATGGTGACAGATCCAG

GCACCGAGCTCCACGCAATGCCCGGATGTCTGCACCTTCGCTTGGACGCTTTGTCCCAAGGCGTTTCTTG

CTGCCTGAGTACTTGCCTTATGCTGGGATTTTTCATGAACGTGGACAGCCTGGCTTGGCTACTCACTCTT

CTGTTAACAGGGTCCTGGCAGGGGCAGTGATCGGTGATGGACAGTCTGCTGTGGCCAGTAACATTGCCAA

TACTACCTACCGGCTCCAGTGGTGGGACTTCACTAAGTTTGACCTCCCTGAAATCAGTAATGCTTCCGTG

AATGTGCTGGTGCAGAACTGCAAGATCTACAATGATGCCAGCTGTGACATTTCTGCAGATGGCCAGCTCC

TGGCAGCTTTCATCCCCAGCAGCCAGAGGGGCTTTCCTGATGAAGGCATCCTGGCAGTGTACTCCCTGGC

CCCCCATAACCTGGGCGAAATGCTCTACACCAAGCGATTTGGTCCCAATGCCATTTCGGTGAGCCTGTCC

CCAATGGGCAGATATGTAATGGTGGGCTTGGCCTCACGAAGGATCCTGCTGCACCCCTCCACAGAGCACA

TGGTGGCCCAGGTCTTCAGGCTGCAACAGGCCCATGGTGGAGAGACCTCCATGAGGAGAGTTTTCAACGT

CCTTTATCCCATGCCTGCCGACCAGCGGAGACATGTCAGTATCAACTCTGCCCGTTGGCTGCCTGAGCCA

GGGCTTGGCTTGGCCTATGGTACTAACAAAGGAGACCTGGTGATCTGCCGACCAGAGGCCTTAAACTCTG

GTGTTGAGTACTACTGGGACCAGCTGAACGAGACGGTCTTCACTGTCCATTCCAACAGCAGGAGCAGCGA

GCGGCCTGGAACCAGCAGAGCCACATGGAGGACAGACAGAGACATGGGGCTGATGAATGCCATTGGGCTT

CAGCCCCGGAACCCTGCCACCTCAGTGACATCTCAGGGCACCCAGACTCTGGCCCTTCAGCTGCAGAATG

CCGAAACACAGACTGAGAGGGAGGTGCCGGAGCCAGGGACAGCCGCCTCAGGTCCTGGTGAAGGTGAGGG

TTCAGAGTATGGTGCCAGTGGAGAAGATGCGCTCAGCAGGATCCAGAGGCTGATGGCGGAGGGCGGCATG

ACAGCCGTGGTGCAGCGGGAGCAGAGCACCACCATGGCCTCCATGGGCGGCTTCGGCAACAACATCATCG

TCAGCCACCGCATTCACCGCAGCTCTCAGACGGGCACTGAACCTGGTGCCGCCCACACCTCCTCACCCCA

GCCCTCCACCTCTCGGGGACTGCTCCCAGAGGCCGGGCAACTGGCAGAGCGAGGCCTAAGCCCCCGGACA

GCTTCCTGGGACCAGCCTGGTACCCCTGGGCGGGAGCCAACCCAGCCAACCCTGCCCTCTTCCTCCCCTG

TCCCCATTCCTGTTTCCCTTCCCAGCGCTGAGGGACCAACCCTCCACTGCGAGTTGACCAATAACAACCA

CCTTCTGGATGGTGGCAGCAGCAGGGGGGACGCTGCAGGCCCTAGGGGAGAACCACGGAACAGGTAGAGA

CAAACGTTGCACTGGTGCCTCCCCTCGAACCGCCAAGCAGAAACCGGACCTCACAGCTGACTGGGAACTG

GACATGTGGAAGAGCTGCTGGCTGCATCAGGGAACAGGAGGAGGAAGAGGGTCAGGGTGGAGAGGAAGAT

CAGTCAGTGGGCACAAGACAGTCAAATGGGCAAGGCCTGCCTCGGGGAACTAGAACCTTCCAGGATCTGG

AGCCCGGGAGAGCCACACTGTGGGCTTAATGTGAATAGAGGAACAAGTGGGTATCTCTGCCAGGCACCCC

ACTTTCTCCTAGTAACATGGGCTCAGGGGACTCAGCCCTGGACAGAGAGCCTCCAGAGAGTGAACAGTCT

TCCAGATCTGGGCCAATCATCCTGGACAGAGGCCCGCGAGGCAGCTTTGCCCTGTCCACCTGTTGGGTGG

GCAGAGCCACCAGGAACCCAGACACCACCTCCAACTCTGAGCCTTCCAGAGCTTCAGCCTCTCTTCGTCG

TCTTACCCCACTGAAACCAACAGGGATCGGGCCAGGCTCCCAGATTCTTGAGGACAGGGACTTCGGCATT

TACTAATGGGGGACTACTGTGGGGTAAGGGGGCGCCTGCTTGCCTGATACAGGATGGGGTCAAGGGACAG

TGGGCAGGTCCTCACTCAGGAGTGGGGGGTGTAGGCTGGCCAGCCCCCAGGGCTTGTCCACCAGTCTTCT

CCCCGCAAGGCCCTCAGAGCAGCGCCTGTGGGTGTCAGTATTACCTGAGCCTAGGCCAAAGCTAGCCCAA

GGCTGGGGAAGGGGAGGAGACTCCAGGTCAGAATGTGAGGTCTCAGTCTGTGATTTAAGGTGTTGCATGT

GGACTCTTAACTGTACGTGTAGTTTCTAGTGGAGAAATCAAGGCTCTGATCATTTTGTTTTTAGTATGAA

AATGTGATTTCCTTTCTGTTTGTAACTCATCATAGAAACATTGTGGTGGGAGGAGAGGGGATAGTCTACA

GCTAATGAGGGAAACACCAAAGATCACATCATTAAAATGATGACATGCCCCTCGCAAAAAAAAA

>NM_145343.3 Homo sapiens apolipoprotein L1 (APOL1), transcript variant 2, mRNA

ATTATACAGACGCATAACTGGAGGTGGGATCCACACAGCTCAGAACAGCTGGATCTTGCTCAGTCTCTGC

CAGGGGAAGATTCCTTGACTTCTGGGGTGATGGAGAAGAAACAGGCTGTGCTGTGTCCCTAATGGGAAAC

GTGGCTGAGACAGGGGAGTGAGAAGGGTGCGTTGCAGAATGGTGCCTGTGGCATGATGCCAGCTTTGCAA

TCATGAGATTCAAAAGCCACACTGTGGAATTGAGGAGGCCCTGCAGCGACATGGAGGGAGCTGCTTTGCT

GAGAGTCTCTGTCCTCTGCATCTGGATGAGTGCACTTTTCCTTGGTGTGGGAGTGAGGGCAGAGGAAGCT

GGAGCGAGGGTGCAACAAAACGTTCCAAGTGGGACAGATACTGGAGATCCTCAAAGTAAGCCCCTCGGTG

ACTGGGCTGCTGGCACCATGGACCCAGAGAGCAGTATCTTTATTGAGGATGCCATTAAGTATTTCAAGGA

AAAAGTGAGCACACAGAATCTGCTACTCCTGCTGACTGATAATGAGGCCTGGAACGGATTCGTGGCTGCT

GCTGAACTGCCCAGGAATGAGGCAGATGAGCTCCGTAAAGCTCTGGACAACCTTGCAAGACAAATGATCA

TGAAAGACAAAAACTGGCACGATAAAGGCCAGCAGTACAGAAACTGGTTTCTGAAAGAGTTTCCTCGGTT

GAAAAGTGAGCTTGAGGATAACATAAGAAGGCTCCGTGCCCTTGCAGATGGGGTTCAGAAGGTCCACAAA

GGCACCACCATCGCCAATGTGGTGTCTGGCTCTCTCAGCATTTCCTCTGGCATCCTGACCCTCGTCGGCA

TGGGTCTGGCACCCTTCACAGAGGGAGGCAGCCTTGTACTCTTGGAACCTGGGATGGAGTTGGGAATCAC

AGCCGCTTTGACCGGGATTACCAGCAGTACCATGGACTACGGAAAGAAGTGGTGGACACAAGCCCAAGCC

CACGACCTGGTCATCAAAAGCCTTGACAAATTGAAGGAGGTGAGGGAGTTTTTGGGTGAGAACATATCCA

ACTTTCTTTCCTTAGCTGGCAATACTTACCAACTCACACGAGGCATTGGGAAGGACATCCGTGCCCTCAG

ACGAGCCAGAGCCAATCTTCAGTCAGTACCGCATGCCTCAGCCTCACGCCCCCGGGTCACTGAGCCAATC

TCAGCTGAAAGCGGTGAACAGGTGGAGAGGGTTAATGAACCCAGCATCCTGGAAATGAGCAGAGGAGTCA

AGCTCACGGATGTGGCCCCTGTAAGCTTCTTTCTTGTGCTGGATGTAGTCTACCTCGTGTACGAATCAAA

GCACTTACATGAGGGGGCAAAGTCAGAGACAGCTGAGGAGCTGAAGAAGGTGGCTCAGGAGCTGGAGGAG

AAGCTAAACATTCTCAACAATAATTATAAGATTCTGCAGGCGGACCAAGAACTGTGACCACAGGGCAGGG

CAGCCACCAGGAGAGATATGCCTGGCAGGGGCCAGGACAAAATGCAAACTTTTTTTTTTTTCTGAGACAG

AGTCTTGCTCTGTCGCCAAGTTGGAGTGCAATGGTGCGATCTCAGCTCACTGCAAGCTCTGCCTCCCGTG

TTCAAGCGATTCTCCTGCCTTGGCCTCCCAAGTAGCTGGGACTACAGGCGCCTACCACCATGCCCAGCTA

ATTTTTGTATTTTTAATAGAGATGGGGTTTCACCATGTTGGCCAGGATGGTCTCGATCTCCTGACCTCTT

GATCTGCCCACCTTGGCCTCCCAAAGTGCTGGGATTACAGGCGTGAGCCATCGCTTTTGACCCAAATGCA

AACATTTTATTAGGGGGATAAAGAGGGTGAGGTAAAGTTTATGGAACTGAGTGTTAGGGACTTTGGCATT

TCCATAGCTGAGCACAGCAGGGGAGGGGTTAATGCAGATGGCAGTGCAGCAAGGAGAAGGCAGGAACATT

GGAGCCTGCAATAAGGGAAAAATGGGAACTGGAGAGTGTGGGGAATGGGAAGAAGCAGTTTACTTTAGAC

TAAAGAATATATTGGGGGGCCGGGTGTAGTGGCTCATGCCTGTAATCCGAGCACTTTGGGAGGCCAAGGC

GGGCGGATCACGAGGTCAGGAGATCGAGACCATCCTGGCTAACACAGTGAAACCCCGTCTCTACTAAAAA

TACAAAAAATTAGCCGGGCATGGTGGCGGGCGCCTGTAGTTCCAGCTAACTGGGCGGCTGAGGCAGGAGA

ATGGCGTGAACCTGGGAGGTGGAGCTTGCAGTGAGCCGAGATATCGCCACTGCACTCCAGCCTGGGTGAC

AGAGCGAGACTCCATCTCAAAAAAAAAAAAAAAAAGAATATATTGACGGAAGAATAGAGAGGAGGCTTGA

AGGAACCAGCAATGAGAAGGCCAGGAAAAGAAAGAGCTGAAAATGGAGAAAGCCCAAGAGTTAGAACAGT

TGGATACAGGAGAAGAAACAGCGGCTCCACTACAGACCCAGCCCCAGGTTCAATGTCCTCCGAAGAATGA

AGTCTTTCCCTGGTGATGGTCCCCTGCCCTGTCTTTCCAGCATCCACTCTCCCTTGTCCTCCTGGGGGCA

TATCTCAGTCAGGCAGCGGCTTCCTGATGATGGTCATTGGGGTGGTTGTCATGTGATGGGTCCCCTCCAG

GTTACTAAAGGGTGCATGTCCCCTGCTTGAACACTGAAGGGCAGGTGGTGGGCCATGGCCATGGTCCCCA

GCTGAGGAGCAGGTGTCCCTGAGAACCCAAACTTCCCAGAGAGTATGTGAGAACCAACCAATGAAAACAG

TCCCATCGCTCTTACCCGGTAAGTAAACAGTCAGAAAATTAGCATGAAAGCAGTTTAGCATTGGGAGGAA

GCTCAGATCTCTAGAGCTGTCTTGTCGCCGCCCAGGATTGACCTGTGTGTAAGTCCCAATAAACTCACCT

ACTCATCAA

>NM_001197325.2 Homo sapiens aryl hydrocarbon receptor nuclear translocator (ARNT), transcript variant 4, mRNA

GGGGTGGCGCGGCGGCGGTGGCATCTGCGGCCATGGCGGCGACTACTGCCAACCCCGAAATGACATCAGA

TGTACCATCACTGGGTCCAGCCATTGCCTCTGGAAACTCTGGACCTGGAATTCAAGGTGGAGGAGCCATT

GTCCAGAGGGCTATTAAGCGGCGACCAGGGCTGGATTTTGATGATGATGGAGAAGGGAACAGTAAATTTT

TGAGGTGTGATGATGATCAGATGTCTAACGATAAGGAGCGGTTTGCCAGGGAAAATCACAGTGAAATTGA

ACGGCGGCGACGGAACAAGATGACAGCCTACATCACAGAACTGTCAGATATGGTACCCACCTGTAGTGCC

CTGGCTCGAAAACCAGACAAGCTAACCATCTTACGCATGGCAGTTTCTCACATGAAGTCCTTGCGGGGAA

CTGGCAACACATCCACTGATGGCTCCTATAAGCCGTCTTTCCTCACTGATCAGGAACTGAAACATTTGAT

CTTGGAGGCAGCAGATGGCTTTCTGTTTATTGTCTCATGTGAGACAGGCAGGGTGGTGTATGTGTCTGAC

TCCGTGACTCCTGTTTTGAACCAGCCACAGTCTGAATGGTTTGGCAGCACACTCTATGATCAGGTGCACC

CAGATGATGTGGATAAACTTCGTGAGCAGCTTTCCACTTCAGAAAATGCCCTGACAGGGCGTATCCTGGA

TCTAAAGACTGGAACAGTGAAAAAGGAAGGTCAGCAGTCTTCCATGAGAATGTGTATGGGCTCAAGGAGA

TCGTTTATTTGCCGAATGAGGTGTGGCAGTAGCTCTGTGGACCCAGTTTCTGTGAATAGGCTGAGCTTTG

TGAGGAACAGATGCAGGAATGGACTTGGCTCTGTAAAGGATGGGGAACCTCACTTCGTGGTGGTCCACTG

CACAGGCTACATCAAGGCCTGGCCCCCAGCAGGTGTTTCCCTCCCAGATGATGACCCAGAGGCTGGCCAG

GGAAGCAAGTTTTGCCTAGTGGCCATTGGCAGATTGCAGGTAACTAGTTCTCCCAACTGTACAGACATGA

GTAATGTTTGTCAACCAACAGAGTTCATCTCCCGACACAACATTGAGGGTATCTTCACTTTTGTGGATCA

CCGCTGTGTGGCTACTGTTGGCTACCAGCCACAGGAACTCTTAGGAAAGAATATTGTAGAATTCTGTCAT

CCTGAAGACCAGCAGCTTCTAAGAGACAGCTTCCAACAGGTAGTGAAATTAAAAGGCCAAGTGCTGTCTG

TCATGTTCCGGTTCCGGTCTAAGAACCAAGAATGGCTCTGGATGAGAACCAGCTCCTTTACTTTCCAGAA

CCCTTACTCAGATGAAATTGAGTACATCATCTGTACCAACACCAATGTGAAGAACTCTAGCCAAGAACCA

CGGCCTACACTCTCCAACACAATCCAGAGGCCACAACTAGGTCCCACAGCTAATTTACCCCTGGAGATGG

GCTCAGGACAGCTGGCACCCAGGCAGCAGCAACAGCAAACAGAATTGGACATGGTACCAGGAAGAGATGG

ACTGGCCAGCTACAATCATTCCCAGGTGGTTCAGCCTGTGACAACCACAGGACCAGAACACAGCAAGCCC

CTTGAGAAGTCAGATGGTTTATTTGCCCAGGATAGAGATCCAAGATTTTCAGAAATCTATCACAACATCA

ATGCGGATCAGAGTAAAGGCATCTCCTCCAGCACTGTCCCTGCCACCCAACAGCTATTCTCCCAGGGCAA

CACATTCCCTCCTACCCCCCGGCCGGCAGAGAATTTCAGGAATAGTGGCCTAGCCCCTCCTGTAACCATT

GTCCAGCCATCAGCTTCTGCAGGACAGATGTTGGCCCAGATTTCCCGCCACTCCAACCCCACCCAAGGAG

CAACCCCAACTTGGACCCCTACTACCCGCTCAGGCTTTTCTGCCCAGGTGGCTACCCAGGCTACTGCTAA

GACTCGTACTTCCCAGTTTGGTGTGGGCAGCTTTCAGACTCCATCCTCCTTCAGCTCCATGTCCCTCCCT

GGTGCCCCAACTGCATCGCCTGGTGCTGCTGCCTACCCTAGTCTCACCAATCGTGGATCTAACTTTGCTC

CTGAGACTGGACAGACTGCAGGACAATTCCAGACACGGACAGCAGAGGGTGTGGGTGTCTGGCCACAGTG

GCAGGGCCAGCAGCCTCATCATCGTTCAAGTTCTAGTGAGCAACATGTTCAACAACCGCCAGCACAGCAA

CCTGGCCAGCCTGAGGTCTTCCAGGAGATGCTGTCCATGCTGGGAGATCAGAGCAACAGCTACAACAATG

AAGAATTCCCTGATCTAACTATGTTTCCCCCCTTTTCAGAATAGAACTATTGGGGTGAGGATAAGGGGTG

GGGGAGAAAAAATCACTGTTTGTTTTTAAAAAGCAAATCTTTCTGTAAACAGAATAAAAGTTCCTCTCCC

TTCCCTTCCCTCACCCCTGACATGTACCCCCTTTCCCTTCTGGCTGTTCCCCTGCTCTGTTGCCTCTCTA

AGGTAACATTTATAGAAGAAATGGAATGAATCTCCAAGGCTTTTAGGACTGTCTGAAAATTTGAGGCTGG

GTGAAGTTAAAACACCTTTCCTTATGTCTCCTGACCTGAAATTGTATAGTGTTGATTTGTGCTGAGATCA

AGAGGCAGGTTAGAAGAACCTGACATCCACTGTTTGCCTTGGATAGTATGGCTTGTTTTTGGAAAGAAAT

TCTGAAGAGAGTGGAGGAGAGGAGAAATGTCCTCATATTTGAGGACCATGAAACATTGTAGGTATATATG

GGGCTTTAGCAAGTTTGAGCATAGGCTCTTTTTGCTGCCTGTGAGCAGTCCCTCTGGAAAGAAACATGTG

AGTAAGTGAGAGAGAGTGTGTGTGTATGTGTGTGTGTGTGTGTGCGCACACATGCTTCTGTATTTCAC

TCTTTCTCCCTATTAGGGAGTTATGCAAAATTTGTCCCCGATTTTACCTTTGTCTTTCTGTGTACTTTTC

AAAGAGTCCTAAGGAGTTAAATCTTCCAGGTATTTTCCACTTAGTATTGCAGCCAAAGAATATTTAAATA

AACGTCTTTGCTGCGCTTGCATCCATGCCCAGCCAATATACAACTGTAAAGCAAATATAGAAAGTCGGCT

GTTGATACGATTGTCTGTTATCGAACACATTCAGTGATAAAGCTGGGTTACTGCTGCTTTTGGTGCTCTC

ACCTTATCTGGAAGATCTGCAAACATTACCTAAATAGGCTGGCAAGATAAACACTTTCTGGAACCCGAGA

CTTGGCCATAAAGATAATGCTGCATTTTTCTGTCAGAATCACATATGATGTGTGTTCTGTAGAGGTTATT

TCTGCATGGAAACTCAACTTCTTGGATTAGCCGTCCCAGTGAAAATCCTCATTGTTGGAGTGTAAACCAA

ATACGAAGCCCTCTTGCAAAGTAGCCTCTTTCATCCCATACTCAAAATACCCAGTTTAGCAAGCAACTGA

GATTTAAGTCTCTCTGGCCCTAAGAGGTTTTTCCTCTTTGCTCCCTCCAATCTTGAGATTGGGTTTTGCT

TTAGAGTGCAAGTATCATAATTCCGTATGATAGATGGGGCCTGGACACCCATCTCAACAGGGTCACTTGG

TAATTAACAATAGCCATATAAATGCGGATACAGGTTACTACCCTCACCCTTTACCTTCCTCAGGTAACAG

TCGTAGATACCAGCTTTTTTTTTTTTTTTTTTAAATTGGCTTTGGCCAGTAGCTAAAGTGCAAGACTGAA

TTAATGAGAAGATATATTAAATGTAGTCATAGGGGACTGAGGAGCAAGGGTGGCCTTGAAGAGGCCAAAG

GAATGTCCATTTGCTGAGTTTCCCTTCCTTATGTCTCCAGTCTGGTGCCAGGTAGTGGAGTAAAAAAGGA

GACAGTTTATTTTTTTATTCTATGTGCACACTTACAGTATACATATATATTTATATCACAATTTACGAAA

CCAAAAAGTTGAGTTTCCAATGGAACCCTTGTTTTTTAATAATCGACTTTTTAAATGTGATCAGGACTAT

AATATTGTACAGTTATTATAGGGCTTTTGGGGAAGGGGAGGATAGCGAGAAGATGCTCTGGGGGTTTTGT

TTTTGCTTTTCCTTCAGGGTTTTATTTTTGACTGTTTTGTTTTCTTGTTGGCCATTTCTGTATTGCTGGC

ATCTGTGCTAAGCTTTACAGTGGCAAAAATAATGACATGTAGCAAAGATTTTCAAACAAAATATTTTTTC

CTTTTGTAAAATTTCTTGTGTTGTGTGATCTTGATTGCGGCTTTATCATTCCTTTCCAGTTCATAAACAA

CAGGCACCCACAACCAGAGGAATCTATAGTTTAAGCTCCAGACATACAAACATAAGGCACATTGTGTCTT

TAATTTCAGGAATCAGAAATCATAGGGTTCTGATCACATTGCACGCCTCCCCCCTCACTTGTCCTCCTGA

TCCTGACACATTCTGAGTAACATCAGCAGGAATGCTCTGACCATGAGGTGGGGGTTTTGGGGTGGGCGTT

GCCTGGGTTCTTGGGAGAGAGGGGAAGAGTCGGGACTTGAAAACCACTAGGGCACATCTGGATGCCTTCC

CCCAGTATGTCCTTTTCTGGATTAAAATGAGTGAAATTTAAA

>NM_000487.6 Homo sapiens arylsulfatase A (ARSA), transcript variant 1, mRNA

GAGGCGGAAGCGCCCGCAGCCCGGTACCGGCTCCTCCTGGGCTCCCTCTAGCGCCTTCCCCCCGGCCCGA

CTCCGCTGGTCAGCGCCAAGTGACTTACGCCCCCGACCCTGAGCCCGGACCGCTAGGCGAGGAGGATCAG

ATCTCCGCTCGAGAATCTGAAGGTGCCCTGGTCCTGGAGGAGTTCCGTCCCAGCCCGCGGTCTCCCGGTA

CTGTCGGGCCCCGGCCCTCTGGAGCTTCAGGAGGCGGCCGTCAGGGTCGGGGAGTATTTGGGTCCGGGGT

CTCAGGGAAGGGCGGCGCCTGGGTCTGCGGTATCGGAAAGAGCCTGCTGGAGCCAAGTAGCCCTCCCTCT

CTTGGGACAGACCCCTCGGTCCCATGTCCATGGGGGCACCGCGGTCCCTCCTCCTGGCCCTGGCTGCTGG

CCTGGCCGTTGCCCGTCCGCCCAACATCGTGCTGATCTTTGCCGACGACCTCGGCTATGGGGACCTGGGC

TGCTATGGGCACCCCAGCTCTACCACTCCCAACCTGGACCAGCTGGCGGCGGGAGGGCTGCGGTTCACAG

ACTTCTACGTGCCTGTGTCTCTGTGCACACCCTCTAGGGCCGCCCTCCTGACCGGCCGGCTCCCGGTTCG

GATGGGCATGTACCCTGGCGTCCTGGTGCCCAGCTCCCGGGGGGGCCTGCCCCTGGAGGAGGTGACCGTG

GCCGAAGTCCTGGCTGCCCGAGGCTACCTCACAGGAATGGCCGGCAAGTGGCACCTTGGGGTGGGGCCTG

AGGGGGCCTTCCTGCCCCCCCATCAGGGCTTCCATCGATTTCTAGGCATCCCGTACTCCCACGACCAGGG

CCCCTGCCAGAACCTGACCTGCTTCCCGCCGGCCACTCCTTGCGACGGTGGCTGTGACCAGGGCCTGGTC

CCCATCCCACTGTTGGCCAACCTGTCCGTGGAGGCGCAGCCCCCCTGGCTGCCCGGACTAGAGGCCCGCT

ACATGGCTTTCGCCCATGACCTCATGGCCGACGCCCAGCGCCAGGATCGCCCCTTCTTCCTGTACTATGC

CTCTCACCACACCCACTACCCTCAGTTCAGTGGGCAGAGCTTTGCAGAGCGTTCAGGCCGCGGGCCATTT

GGGGACTCCCTGATGGAGCTGGATGCAGCTGTGGGGACCCTGATGACAGCCATAGGGGACCTGGGGCTGC

TTGAAGAGACGCTGGTCATCTTCACTGCAGACAATGGACCTGAGACCATGCGTATGTCCCGAGGCGGCTG

CTCCGGTCTCTTGCGGTGTGGAAAGGGAACGACCTACGAGGGCGGTGTCCGAGAGCCTGCCTTGGCCTTC

TGGCCAGGTCATATCGCTCCCGGCGTGACCCACGAGCTGGCCAGCTCCCTGGACCTGCTGCCTACCCTGG

CAGCCCTGGCTGGGGCCCCACTGCCCAATGTCACCTTGGATGGCTTTGACCTCAGCCCCCTGCTGCTGGG

CACAGGCAAGAGCCCTCGGCAGTCTCTCTTCTTCTACCCGTCCTACCCAGACGAGGTCCGTGGGGTTTTT

GCTGTGCGGACTGGAAAGTACAAGGCTCACTTCTTCACCCAGGGCTCTGCCCACAGTGATACCACTGCAG

ACCCTGCCTGCCACGCCTCCAGCTCTCTGACTGCTCATGAGCCCCCGCTGCTCTATGACCTGTCCAAGGA

CCCTGGTGAGAACTACAACCTGCTGGGGGGTGTGGCCGGGGCCACCCCAGAGGTGCTGCAAGCCCTGAAA

CAGCTTCAGCTGCTCAAGGCCCAGTTAGACGCAGCTGTGACCTTCGGCCCCAGCCAGGTGGCCCGGGGCG

AGGACCCCGCCCTGCAGATCTGCTGTCATCCTGGCTGCACCCCCCGCCCAGCTTGCTGCCATTGCCCAGA

TCCCCATGCCTGAGGGCCCCTCGGCTGGCCTGGGCATGTGATGGCTCCTCACTGGGAGCCTGTGGGGGAG

GCTCAGGTGTCTGGAGGGGGTTTGTGCCTGATAACGTAATAACACCAGTGGAGACTTGCAGATGTGACAA

TTCGTCCAATCCTGGGGTAATGCTGTGTGCTGGTGCCGGTCCCCTGTGGTACGAATGAGGAAACTGAGGT

GCAGAGAGGTTCAGGACTTGTACAAGATCACCCAGCCAGAAAGAGGTTGGGCTGGGATTTGAACCCTGGT

GTCGTGGCTCTGGAAGCTGCCCTGGCGCCTTGGTGATCTGCGTGGGTCAGTGCACACAGGCACACGTCAG

CCTCAAGGACATGGGCACATCTGTTCACAGGAGCAGCGCCACGTGCCTTTGAGTGCCAGGAACGGGGTGG

GAGGGTGGGAGGGTGTGAGGGCCAGAAGACTCAGAAGATGCAAAGTGCCTGAGAGAGACGGGATATTCCC

CCAGAAGAAGCATTCTTAGAGACACAGGCACTGGACCTCCTTGGTTCTTATAAGAAACCTGTCTGAAGCT

GGGTGATGAGTTGCACACTCCAGGTGGGGCTAAGGGGCCTGGAGCCCCTGCTGGCTCCTAGGAAGGCACA

GCAGCAGGCCCTGAGACGGCTCCTCTGGGGCCCCTCCACCCTCCCAGGCCTCTGCATTTCACCTGTGCCC

ACACTTCTGTCTCCTGCCTTCACCTTTTGACCCACTACTAACGATTCTCCACCCAGCAGACAAAGTGATC

TCTTAAAAATATCTGTTGGCTGGGCACGGTGGCTCACGCCTGTAATCCCAGCACTTTAGGAAGCCGAGGC

GGGTGGATCACCTGAGGTCGGGAGTTCGAGACCAGCCTGACCAACATGGAGAAACCCCATCTCTACTAAA

AATACAAAATTAGCCAGGTGTAGTGGTGCATCCCTGTAATCCCAGCTACTTGGGAGTCTGAGGCTGGAGA

ATCACTTGAACCTGGGAGGCGGTGGTTGCAGTGAGCCGAGATCGCACCATTGCACTCCAGCCTGGGCAAC

AAGAGAAAAACTCTGTCTCAAAAAACAAAAAATCTGTTAGGCTGCACACGGCGATTCACTCCTGTATTCC

CAGTGCTTTGGGAGGCTGAGGTGAGAGGATGCCTGAGGCCAGGAATTCAGACCAGCCTGGGCAACATAGT

GAGACCCCAGCTCTAAAGATTTGTTTTTGTTTTTTTTTTTTTTTTTTTTTTTTTTTTTTTTTTTTTTTTTTG

AGACGGAGTCTCGCTCTGTCGCCCAGGCTAGAGTGCAGTGGTACCATCTCCGCTCACTGCAACCTCCGCC

TCCCGGGTTCCAGGGATTCTCCTGCCTCAGCCTCCCTAGTAGCTGGAACTACAGGTGTGTGCTGCCATGC

CCAGCTAATTTTTTTTTATTTAATAGAGACAAGATTTCACCATGTTGGCCAGGCTGGTCTCAAACTCCTG

ACCTCAGGTGATCCACCCGCCTCAGCCTCCCAAAGTGCTGGGATTACAGGTGTGAACCACCACACCTGGC

CAACAATATTTGTTTTAATTAGCCAGGCGTGGTAGCATTTGTCCTAGCAATTTGGGAGGTTGAGGTGGGA

GAATCACTTCAGCCCACTAGGTCGAGGCTGTAGTGAGCTATAATTGTACCACTGCACTCCAGCCTCGGGG

ACAGAGTGAGACCCTGTCTGCAAATAAACAAATAAAACATCAGGCTGGGCTTGAGCATCTATTCCTGCTC

AAAATTTCGCAGGCTTCTCAGAAGAAAATCCAAACCCCTTACAGTGACCCAGTTTGCCCTTGAGGCCTCC

ACCCACACCCCCTTCCCCCCAGTCTTAGGGGGTGGCCTGGCTGTTCCCTTCAACGGCAACGCTCTGCCTC

CATTGTTGGCCTCCTCTGCAGGGAGGGACTGTCTGAGCACCTGCCCGTGTCTGTGCAGCATGGCACACTG

ACGTCAGGCCCACGTGCATGCCCAGGTGGCCAGTCACACGCCAGGTGCTCCCTCAGTGTTGGCCAAGTGA

GAGGAGCACACCTTCCGGGCGTTCAGACACCTCCCCGTGGCAGACACCGTTCGTTGCTACCAAACAGCCA

CCTCCTTCCTAATGGGCTCCCATTTTTCAGTGCTGGGCAAAGGTCCCTTGATCTTGGAGTTGCAGCCTCT

TTCTCTCCAAGGAGGGCGGTGACCAGCCTGAGCCAGTCAATCCAGTGATTGGTTCAGGAGTAGCCTGTGA

CCAGGAGTCCTGGTAGTGAACGACTGGGGCAGCCCTGGGGGTGAGGACCTTGCGCAGCCGTCACAGGCCC

TGATTGGACACTGGGCAGCTGCTAACCCAGTGTCTCCAGCTGCCTACCTGGAGAGCTCCAAGCGTAAGAA

AATAAACCCTGCCTGTTGAAGCCA

>NM_000046.5 Homo sapiens arylsulfatase B (ARSB), transcript variant 1, mRNA

ATTCTATCAGCGGTACAAGGGGCTGGTGGCGCCACAGGCGCTGGGACCGCGGGCGGACAAGGATGGGTCC

GCGCGGCGCGGCGAGCTTGCCCCGAGGCCCCGGACCTCGGCGGCTGCTCCTCCCCGTCGTCCTCCCGCTG

CTGCTGCTGCTGTTGTTGGCGCCGCCGGGCTCGGGCGCCGGGGCCAGCCGGCCGCCCCACCTGGTCTTCT

TGCTGGCAGACGACCTAGGCTGGAACGACGTCGGCTTCCACGGCTCCCGCATCCGCACGCCGCACCTGGA

CGCGCTGGCGGCCGGCGGGGTGCTCCTGGACAACTACTACACGCAGCCGCTGTGCACGCCGTCGCGGAGC

CAGCTGCTCACTGGCCGCTACCAGATCCGTACAGGTTTACAGCACCAAATAATCTGGCCCTGTCAGCCCA

GCTGTGTTCCTCTGGATGAAAAACTCCTGCCCCAGCTCCTAAAAGAAGCAGGTTATACTACCCATATGGT

CGGAAAATGGCACCTGGGAATGTACCGGAAAGAATGCCTTCCAACCCGCCGAGGATTTGATACCTACTTT

GGATATCTCCTGGGTAGTGAAGATTATTATTCCCATGAACGCTGTACATTAATTGACGCTCTGAATGTCA

CACGATGTGCTCTTGATTTTCGAGATGGCGAAGAAGTTGCAACAGGATATAAAAATATGTATTCAACAAA

CATATTCACCAAAAGGGCTATAGCCCTCATAACTAACCATCCACCAGAGAAGCCTCTGTTTCTCTACCTT

GCTCTCCAGTCTGTGCATGAGCCCCTTCAGGTCCCTGAGGAATACTTGAAGCCATATGACTTTATCCAAG

ACAAGAACAGGCATCACTATGCAGGAATGGTGTCCCTTATGGATGAAGCAGTAGGAAATGTCACTGCAGC

TTTAAAAAGCAGTGGGCTCTGGAACAACACGGTGTTCATCTTTTCTACAGATAACGGAGGGCAGACTTTG

GCAGGGGGTAATAACTGGCCCCTTCGAGGAAGAAAATGGAGCCTGTGGGAAGGAGGCGTCCGAGGGGTGG

GCTTTGTGGCAAGCCCCTTGCTGAAGCAGAAGGGCGTGAAGAACCGGGAGCTCATCCACATCTCTGACTG

GCTGCCAACACTCGTGAAGCTGGCCAGGGGACACACCAATGGCACAAAGCCTCTGGATGGCTTCGACGTG

TGGAAAACCATCAGTGAAGGAAGCCCATCCCCCAGAATTGAGCTGCTGCATAATATTGACCCGAACTTCG

TGGACTCTTCACCGTGTCCCAGGAACAGCATGGCTCCAGCAAAGGATGACTCTTCTCTTCCAGAATATTC

AGCCTTTAACACATCTGTCCATGCTGCAATTAGACATGGAAATTGGAAACTCCTCACGGGCTACCCAGGC

TGTGGTTACTGGTTCCCTCCACCGTCTCAATACAATGTTTCTGAGATACCCTCATCAGACCCACCAACCA

AGACCCTCTGGCTCTTTGATATTGATCGGGACCCTGAAGAAAGACATGACCTGTCCAGAGAATATCCTCA

CATCGTCACAAAGCTCCTGTCCCGCCTACAGTTCTACCATAAACACTCAGTCCCCGTGTACTTCCCTGCA

CAGGACCCCCGCTGTGATCCCAAGGCCACTGGGGTGTGGGGCCCTTGGATGTAGGATTTCAGGGAGGCTA

GAAAACCTTTCAATTGGAAGTTGGACCTCAGGCCTTTTCTCACGACTCTTGTCTCATTTGTTATCCCAAC

CTGGGTTCACTTGGCCCTTCTCTTGCTCTTAAACCACACCGAGGTGTCTAATTTCAACCCCTAATGCATT

TAAGAAGCTGATAAAATCTGCAACACTCCTGCTGTTGGCTGGAGCATGTGTCTAGAGGTGGGGGTGGCTG

GGTTTATCCCCCTTTCCTAAGCCTTGGGACAGCTGGGAACTTAACTTGAAATAGGAAGTTCTCACTGAAT

CCTGGAGGCTGGAACAGCTGGCTCTTTTAGACTCACAAGTCAGACGTTCGATTCCCCTCTGCCAATAGCC

AGTTTTATTGGAGTGAATCACATTTCTTACGCAAATGAAGGGAGCAGACAGTGATTAATGGTTCTGTTGG

CCAAGGCTTCTCCCTGTCGGTGAAGGATCATGTTCAGGCACTCCAAGTGAACCACCCCTCTTGGTTCACC

CCTTACTCACTTATCTCATCACAGAGCATAAGGCCCATTTTGTTGTTCAGGTCAACAGCAAAATGCCTGC

ACCATGACTGTGGCTTTTAAAATAAAGAAATGTGTTTTTATCGTAATTTATTTCCCCCCAGCCATTGCTC

ACTCTGTCTAGACTTCCTGCCACTTCCAATTCTTCTGTGGCTTTTCCTGCCTTTCCTTTTGACCTCAGTA

GTCCTATCCCTGGGAAGGCCACTTTGCTTCTCTACCTGAGCACCCCTGATTTCTGGAACGCTGCTGAGCC

CTGCCTTACTTTTGCCCCTAGGGCTGAAGCTAGAGGCCTCCCCGTAATAGGCGGTGGAGTTGCTCTGTGA

GGATGTTCATGGTAGACACTAAGAGGGCTGGGTGGGAGATGCTTGGCTCTGTGGCATCTGTTCAGCGAGG

CTTTTCCTATATTGCATGGAGTTAGTCATTGTGATTGTAGCTTTATTTCATAATATATTAAGACTTGCAC

TGCTATTTACTAGCAGTGAGAAGAAACCTCAGGAAAGGATATGAAAAAGCAAGTGGCCAGTGTCTGGGAT

ACTGGGCCTTGGTAAAGCAGAGGAGGGCACACCCACAGTCCTCTTATTCTCTGTTTTACTGCTTGTTTTG

AGGTTCTGGGGTCTGGCAAAGAGGATGCAGTTTGACACCTGCAGCCCTTTCTCAATCCCACTAATGTCTT

ACTAATGTGGAACAGTCCATATTAGCTCCAGAGAGTGTCAAACCCAGAGAAATGTGTGCAAAAATGATAC

TCTTTTCTGCATTAGCCCCACCATTGTGTTCACCAATGCTTGGAACACTGCCTGAAGGCACTCATTTTTT

AATTTTTATTTTATTTTTAATTTTTTATATCTTTATGAGACGATCTCACTCTGTCACCAGGTTGGAGTAC

AGTGGTACAATCACAACTCACCGTAGCCTCAAACTCCTGGGCTCAAGTGATTCTCCCACCTCAGGCACCC

AAATAGCTGGAACTACAGGCATATACCGCCACACCCAGCTAATTTTATTTTTTGAAAAGACAAGGTTCCC

TATGTTGCCCAGCTGGTCTTAAACTCCTGGGCTCCAGCAATTATCCCAGCTTGGGCTCCAAAAGTGCTGG

GATTACAGGCATGAGTCACCATGCCTGGCCTCATTTTTTAAAACAAATGAATAAATGGACAAATGAGTAA

ATGAGAAAGTCTCACACCATGAAAGATGCTAGTCCAATGAGCTGAATACAGAGGTAATATAAATGTCTTC

CAGCTGTTGCTTTTCTGTTCTCAAGCTGCCCCTCCTGGGGTAGGAGCATAATCTACATCACTGGGCAGTC

ACAGGACACTCTATAGCAAGGTTGTAGCGTCCTCTCCAGTGGGGGGAGAAAAGGAACTGTGCCTACCAAA

GGTACTCTCTTGTCAGCAATTTCCATTTCTATACTTTATGGGACACTAGAAACTAAAAGCAACAAATAAT

CTGATATAAGTCCTTGTATAGTCATCCTTCAATTCAGTAGCAATATTTTCTGGTCACTACTAACCTGTAT

TGTATTAAAATGAGACTATTGGAAGGAAATGGTGCTAAAACTAATAACATCTCTTACCAACCTTTACCCA

ACTCCTGGGTTGGCAAACAGCTGACCAAACTGCCATCACCTCCCACTTGGAAGTGTATGGCCGACAGCAT

GAAATAGCTGAGCCCAGATGTTCCTTCTGCATCCTCCGAATCCCAGGGCTGGGTGTAGGTAGCCGTTGGA

GGCCATCGCTACAGGGCACCTATCTGTTATCGCTGCTGTCCTCCCAACAGCTGTCTCCAGTTCTAGTTCC

TTGGTTTTCAGGCACAGTGGGGGATGTTCTGCACCCAGTGGACTTCAAAAGAGTTTTGAAGACTTAATTT

TTTGTAAAACAAGTACTTGAGATTTTGGTTTATCCATAATAGAATGTATTTCATTAGATTCTCTGATTCT

ATATAAGAATGTGAAAAGATTGATATATTGTTGTTAGAAATAATGTTATTTCTTTCCAATTTTTTTTTTT

TTTTTTTTTGAGATGGAGTCTCGCTCTGTCACCCAGGCTGGAGTGCAGTGGTGTGATCTCGGCTCACTGC

AGCCTCTAACTCCCAGGTTCAAGCTATTCTCCTGCCTCAGCCTCCCAAGTAGCTGGATTACAGGCATACA

CCACCACGCCTGGCTATGTTTTGTATTTTTCGTAGAGATAGGGTTTCACCATGTTGGCCAGGCTGGTCTC

AAACTCCTGACCTCAAGTGATCCACCCACTTCAGCTTCCCAAAGCACTGGGATTACAGGTGTGAGCCACT

GTGCCCGGCAAATTTTTTTACCTTTACAGAAGGTTTTGCTTATTTAATTGTGAGCTCATTTTTCTTTGTT

ACTTTTGTCCCCCCAGATTTGGGGGACAAAATAAAATTAATCTTTTAAAATGTGTCAGCCATATGTATGG

GGCTTCCATTTGGGGTGAGGAGAAAGTTCTGGAACTAGATAGTGGTCATGGTTATACAACATCATAAATG

CAATTACTGCCACTGAATTGTATGTTTTAAAGTGGTTAAAATGTTAAGTTTTATGTTTTATTACAATTTT

TAAATGTGTCAACCAACTTTATAGTACATAAATTATATCTCAGTAAAGCTGTTAAATAAATAAATATAGT

AAAAATTTTAGAACTAAAAAAA

>NM_001675.4 Homo sapiens activating transcription factor 4 (ATF4), transcript variant 1, mRNA

AGCCATTTCTACTTTGCCCGCCCACAGATGTAGTTTTCTCTGCGCGTGTGCGTTTTCCCTCCTCCCCGCC

CTCAGGGTCCACGGCCACCATGGCGTATTAGGGGCAGCAGTGCCTGCGGCAGCATTGGCCTTTGCAGCGG

CGGCAGCAGCACCAGGCTCTGCAGCGGCAACCCCCAGCGGCTTAAGCCATGGCGTGAGTACCGGGGCGGG

TCGTCCAGCTGTGCTCCTGGGGCCGGCGCGGGTTTTGGATTGGTGGGGTGCGGCCTGGGGCCAGGGCGGT

GCCGCCAAGGGGGAAGCGATTTAACGAGCGCCCGGGACGCGTGGTCTTTGCTTGGGTGTCCCCGAGACGC

TCGCGTGCCTGGGATCGGGAAAGCGTAGTCGGGTGCCCGGACTGCTTCCCCAGGAGCCCTACAGCCCTCG

GACCCCGAGCCCCGCAAGGGTCCCAGGGGTCTTGGCTGTTGCCCCACGAAACGTGGCAGGAACCAAGATG

GCGGCGGCAGGGCGGCGGCGCGGGCGTGAGTCAAGGGCGGGCGGTGGGCGGGGCGCGGCCGCCCTGGCCG

TATTTGGACGTGGGGACGGAGCGCTTTCCTCTTGGCGGCCGGTGGAAGAATCCCCTGGTCTCCGTGAGCG

TCCATTTTGTGGAACCTGAGTTGCAAGCAGGGAGGGGCAAATACAACTGCCCTGTTCCCGATTCTCTAGA

TGGCCGATCTAGAGAAGTCCCGCCTCATAAGTGGAAGGATGAAATTCTCAGAACAGCTAACCTCTAATGG

GAGTTGGCTTCTGATTCTCATTCAGGCTTCTCACGGCATTCAGCAGCAGCGTTGCTGTAACCGACAAAGA

CACCTTCGAATTAAGCACATTCCTCGATTCCAGCAAAGCACCGCAACATGACCGAAATGAGCTTCCTGAG

CAGCGAGGTGTTGGTGGGGGACTTGATGTCCCCCTTCGACCAGTCGGGTTTGGGGGCTGAAGAAAGCCTA

GGTCTCTTAGATGATTACCTGGAGGTGGCCAAGCACTTCAAACCTCATGGGTTCTCCAGCGACAAGGCTA

AGGCGGGCTCCTCCGAATGGCTGGCTGTGGATGGGTTGGTCAGTCCCTCCAACAACAGCAAGGAGGATGC

CTTCTCCGGGACAGATTGGATGTTGGAGAAAATGGATTTGAAGGAGTTCGACTTGGATGCCCTGTTGGGT

ATAGATGACCTGGAAACCATGCCAGATGACCTTCTGACCACGTTGGATGACACTTGTGATCTCTTTGCCC

CCCTAGTCCAGGAGACTAATAAGCAGCCCCCCCAGACGGTGAACCCAATTGGCCATCTCCCAGAAAGTTT

AACAAAACCCGACCAGGTTGCCCCCTTCACCTTCTTACAACCTCTTCCCCTTTCCCCAGGGGTCCTGTCC

TCCACTCCAGATCATTCCTTTAGTTTAGAGCTGGGCAGTGAAGTGGATATCACTGAAGGAGATAGGAAGC

CAGACTACACTGCTTACGTTGCCATGATCCCTCAGTGCATAAAGGAGGAAGACACCCCTTCAGATAATGA

TAGTGGCATCTGTATGAGCCCAGAGTCCTATCTGGGGTCTCCTCAGCACAGCCCCTCTACCAGGGGCTCT

CCAAATAGGAGCCTCCCATCTCCAGGTGTTCTCTGTGGGTCTGCCCGTCCCAAACCTTACGATCCTCCTG

GAGAGAAGATGGTAGCAGCAAAAGTAAAGGGTGAGAAACTGGATAAGAAGCTGAAAAAAATGGAGCAAAA

CAAGACAGCAGCCACTAGGTACCGCCAGAAGAAGAGGGCGGAGCAGGAGGCTCTTACTGGTGAGTGCAAA

GAGCTGGAAAAGAAGAACGAGGCTCTAAAAGAGAGGGCGGATTCCCTGGCCAAGGAGATCCAGTACCTGA

AAGATTTGATAGAAGAGGTCCGCAAGGCAAGGGGGAAGAAAAGGGTCCCCTAGTTGAGGATAGTCAGGAG

CGTCAATGTGCTTGTACATAGAGTGCTGTAGCTGTGTGTTCCAATAAATTATTTTGTAGGGAAAGTAAAA

AAAAAAAAAAA

>NM_007348.4 Homo sapiens activating transcription factor 6 (ATF6), mRNA

AGATATTAATCACGGAGTTCCAGGGAGAAGGAACTTGTGAAATGGGGGAGCCGGCTGGGGTTGCCGGCAC

CATGGAGTCACCTTTTAGCCCGGGACTCTTTCACAGGCTGGATGAAGATTGGGATTCTGCTCTCTTTGCT

GAACTCGGTTATTTCACAGACACTGATGAGCTGCAATTGGAAGCAGCAAATGAGACGTATGAAAACAATT

TTGATAATCTTGATTTTGATTTGGATTTGATGCCTTGGGAGTCAGACATTTGGGACATCAACAACCAAAT

CTGTACAGTTAAAGATATTAAGGCAGAACCTCAGCCACTTTCTCCAGCCTCCTCAAGTTATTCAGTCTCG

TCTCCTCGGTCAGTGGACTCTTATTCTTCAACTCAGCATGTTCCTGAGGAGTTGGATTTGTCTTCTAGTT

CTCAGATGTCTCCCCTTTCCTTATATGGTGAAAACTCTAATAGTCTCTCTTCAGCGGAGCCACTGAAGGA

AGATAAGCCTGTCACTGGTCCTAGGAACAAGACTGAAAATGGACTGACTCCAAAGAAAAAAATTCAGGTG

AATTCAAAACCTTCAATTCAGCCCAAGCCTTTATTGCTTCCAGCAGCACCCAAGACTCAAACAAACTCCA

GTGTTCCAGCAAAAACCATCATTATTCAGACAGTACCAACGCTTATGCCATTGGCAAAGCAGCAACCAAT

TATCAGTTTACAACCTGCACCCACTAAAGGCCAGACGGTTTTGCTGTCTCAGCCTACTGTGGTACAACTT

CAAGCACCTGGAGTTCTGCCCTCTGCTCAGCCAGTCCTTGCTGTTGCTGGGGGAGTCACACAGCTCCCTA

ATCACGTGGTGAATGTGGTACCAGCCCCTTCAGCGAATAGCCCAGTGAATGGAAAACTTTCCGTGACTAA

ACCTGTCCTACAAAGTACCATGAGAAATGTCGGTTCAGATATTGCTGTGCTAAGGAGACAGCAACGTATG

ATAAAAAATCGAGAATCCGCTTGTCAGTCTCGCAAGAAGAAGAAAGAATATATGCTAGGGTTAGAGGCGA

GATTAAAGGCTGCCCTCTCAGAAAACGAGCAACTGAAGAAAGAAAATGGAACACTGAAGCGGCAGCTGGA

TGAAGTTGTGTCAGAGAACCAGAGGCTTAAAGTCCCTAGTCCAAAGCGAAGAGTTGTCTGTGTGATGATA

GTATTGGCATTTATAATACTGAACTATGGACCTATGAGCATGTTGGAACAGGATTCCAGGAGAATGAACC

CTAGTGTGAGCCCTGCAAATCAAAGGAGGCACCTTCTAGGATTTTCTGCTAAAGAGGCACAGGACACATC

AGATGGTATTATCCAGAAAAACAGCTACAGATATGATCATTCTGTTTCAAATGACAAAGCCCTGATGGTG

CTAACTGAAGAACCATTGCTTTACATTCCTCCACCTCCTTGTCAGCCCCTAATTAACACAACAGAGTCTC

TCAGGTTAAATCATGAACTTCGAGGATGGGTTCATAGACATGAAGTAGAAAGGACCAAGTCAAGAAGAAT

GACAAATAATCAACAGAAAACCCGTATTCTTCAGGGTGCTCTGGAACAGGGCTCAAATTCTCAGCTGATG

GCTGTTCAATACACAGAAACCACTAGTAGTATCAGCAGGAACTCAGGGAGTGAGCTACAAGTGTATTATG

CTTCACCCAGAAGTTATCAAGACTTTTTTGAAGCCATCCGCAGAAGGGGAGACACATTTTATGTTGTGTC

ATTTCGAAGGGATCACCTGCTGTTACCAGCTACCACCCATAACAAGACCACAAGACCAAAAATGTCAATT

GTGTTACCAGCAATAAACATAAATGAGAATGTGATCAATGGGCAGGACTACGAAGTGATGATGCAGATTG

ACTGTCAGGTGATGGACACCAGGATCCTCCATATCAAAAGTTCGTCAGTTCCTCCTTACCTCCGAGATCA

GCAGAGGAATCAAACCAACACCTTCTTTGGCTCCCCTCCCGCAGCCACAGAGGCAACCCACGTTGTCAGC

ACCATCCCTGAGTCATTACAATAGCACCCTGCAGCTATGCTGGAAAACTGAGCGTGGGACCCTGCCAGAC

TGAAGAGCAGGTGAGCAAAATGCTGCTTTCTGCCTTGGTGGCAGGCAGAGAACTGTCTCGTACTAGAATT

CAAGGAGGAAAGAAGAAGAAATAAAAGAAGCTGCTCCATTTTTCATCATCTACCCATCTATTTGGAAAGC

ACTGGAATTCAGATGCAAGAGAACAATGTTTCTTCAGTGGCAAATGTAGCCCTGCATCCTCCAGTGTTAC

CTGGTGTAGATTTTTTTTTCTGTACCTTTCTAAACCTCTCTTCCCTCTGTGATGGTTTTGTGTTTAAACA

GTCATCTTCTTTTAAATAATATCCACCTCTCCTTTTTGCCATTTCACTTATTGATTCATAAAGTGAATTT

TATTTAAAGCTATGCCACACATGCATGTTCAAATGGTTTCCACTGATTCGATTTTTCATTCATTTAATGC

AAACCCATTCTGGATATTGTGCTTATTTGAGAAAACACATTTCAAAACCAGAAAAGCCAAAAACACTCCA

AAAACAAGCAAAACAATTTGGAGCTTTAGATAAAAGGAAAAACTCCCAGTTGGTAAAGTTTATCTTTACT

TAGGATTTGTGGCTCACACCTAAACAAAGGGGGTCAGGGAGTGGGTACAAATTTGAGAAAATAGAAGGGT

AAGGGAAGGGCCAGTGGTGGGGTTTGGAGAGAGGAGATAGCTCCATTAATACACATGTTTAAAAGATGGA

AAGTTCACGCCTGTAATCCCAGCACTTTGGGAGGCCGAGGCGGGTGGATCACGAGGTCAGGAGATCAAGA

CCATCCCGGCTAAAACGGTGAAACCCCGTCTCTACTAAAAATACAAAAAATTAGCCGGGCGTAGTGACGG

GCGCCTGTAGTCCCAGCTACTTGGGAGGCTGAGGCAGGAGAATGGCGTGAACCCGGGAGGCGGAGCTTGC

AGTGAGCCGAGATCCCGCCACTGCACTCCAGCCTGGGCGACAGAGCGAGACTCCGTCTCAAAAAAAAAAA

AAAAAAAAAAAAAAGATGGAAAGTTCGATGTGACTGCAGTATGAGATTAAAGCCACAACTATTGTTTATT

TTGGGGACTCTAGGCCACCAAGTATTAGCACACATACTTATGTTTTCTCTACTAATCTGGTCCAGGTCCT

CATGGACCACAGGACAAAGCTTTCATTTTCATTCATTCTTCTATTGAAATTATACCAAATTCAGCTGAGG

AATATGGAAGTAACTTTAGACTTAAACAAGACAAAAGTTTTTTCACTGAAGAATTGACAAGTATTTGCTC

CTTAAAACAACGCAGATTAGTGAACGTGGATTCCTGCTGAGGGAGTGCATCCCATAATATGGCAATAATT

TTCAGTTTCTCCAACGAAAAGATAGTGAAGGAATTAAATCTTTTGTCCTCCCATGGTTAAAAAAAAAAAA

AAAGCTGTGTTCATTTTTACTGTACTATGCCTCTTTTTTCACCATAGTAGACAATTATGTTTCATTTGAT

GAATTCATAGAACTGGATCTCATACAGCGATGTCCTCTCTAATGTTCTACCTTTCAGTTTCTAAAGTGAG

TCTTCCTCCCTCTCCTACAAAACTTTTCAATTTTTTGATGTAACTCATCTACAAATACTGTTTCTTACCC

CAGTTGACTTGCCTTTGTCAGATTTCTTCTTGTTCCACACTATAGCAATCAATTTCTCTTCTTCCTTACA

AGAAAGGGAACGAGAAATTGTAGCAACCTCTCAAGGATTATATGCAGCTAGTTAGTTTTCTGCCTGTGAA

ATTAGGTCTGGCTCCTAAATAATTTTAAAGAACCATCAGCACTTCTAACTCTCTGGACAGGTGCCTCTTT

GTCCAAGCTAGTTAAATGCTTTCCAAGGAAATCAGTTCAACTTTTGTGAGCGGGGAAAAGCAGGGCTTTA

TTGTTGTGTTACCTGGGAGTCTGGAGTTTGAAAAGTGCTAATTAACCTTCCTCTTTTTCCACATTACAAA

CCTTTTTAAGCAGCGCAGCACTCCCCTTAGATTTGGCTATCCTGGGTGATTTTCAGACAAGAACCATTTT

CTCTGGGGACCATTCTTCTGCTGGGTGCCAAGGAATATAAGGCAAATGCCCAGAAGACCTTCAGGTGACT

GGGCAGTCTTATCATGGGATATTTCTTCTGGCCCTGCCCCTTCCCATTCTGTAATGTGAATTAGCCACAC

CAGAGGCTGTGACCATGGCTAGTAGACAGTGGCAACATAGTCATCCCCAAGATGCTAATCTTCTGCTGGA

ACTGTCATACGTTATCATGGTCAATGTAAACCTGGTTTGTGTGGGGTGATTATAAATAGAGTTTCCCTCC

TCTCTGTGACAGAATCACAGGAGAAGGACCCATCTCGTGGCCTTCTTGTTCTTAGCGCTTCACTTTTACT

TCATCCCTCGATTCCCAGCTTTTTCTATCATCATTTTGCCAACTCCTCAGATGCAAGACTTTGGTTATGT

CATACTCACCAACGTTAGTCCCTCTCTTCCAGGTGAAAAGGTGGGTAGCGGTTGGGAGGGAGTCTCCACT

GAAGAGCAGGAAGGTGGTAGCAGGGCCGGCAGCTCTGCCACAGAGCTAGGGGTGCCTGTAAGGTGCCGCC

TAGAGCAGCCTGGGAGCTTTGCCTTCTTTTGTCTCTCACTAGCCCTTCTACTCTTTGTCATTGCCTGTTC

TTGAGTGGATCTTTGAAATGAGGGGACAGGATTCTCCTAAGGGTAGAGTTTCAGGAAATGAGTGAAAGGC

AATTGACAAATGCAAAGAAGTAGTCACTTTTTAAATTGCTGGCAAAGCTATAATTAATCCCTAGGCACAA

TTGTAGTTTTTATTTTAATGTTTGTATGCACAAGGCCCTTTAGGAAATGAGAAGTTGCCATGCCAGATTA

ATTTTTTTTTTTTTTTTTGGTGGGATTGCCTTTTGGGGGTTGCAGCCAGAAATTGTGGGTAATGTGTGTA

TTTTTTTATTTATTAAATTTTAAACAGGATTGTGCAAGCTTATGAGACAATTAGATAAACTCATGGAGGA

GGCAGGTCCTCCTGTTATTAGATGATTTTGTGCTCTTGGGGCTGACAATAATACACTCTTGGGAAGTGAT

GGTAGAGACTGATGGGAATAGTCTTTCTGCCTGGTTGCAAGTCCCAAATTTTTAAGGGTTAATGGAAGTA

AGTGGATGTTTCCTCATGTTAACTACTGAATCAGATGTTAGGAGCTTGTCCCTTTGGGGTTGACTTATGC

CCAGCAGTACAGGGACACAGCTTCATTAGAGTGTTAGTGTAAACTAACTCCAAAGTTAGGAGTTAATGTG

AAAGGATCATCCTTGAAACAAATCTGCTGTTTGCCATGCTTGTAGTACAGAAACTTCACATGGAGTTTTG

GGTGGGATTTGTGTTTTCACAAGTAAAAAATCCCTCACGATTATAAAACTCAGAGCATCATCTAATTTTT

TTTTTTAATGACTACAAGTTCCAGCACAAAACTGGCATTTCTTTGCCATTTCTTGCCAGTAAGAAGTTGA

CACGGAGGTATTTGAAAGCAATGTTATGTGAGTCATTCTTAAGTGTTCCAAGTAAGTTTAGAAACAGAAA

AGGAACTTGGGATTCAAATTGATTTTTCAAATCATTTTTAAAGAGACATCATCCTGACTAAATCTTAGCC

TGAACCTTCCTCCCCTGTGTGTATTCCCCGGTAGTCACCGCAGCGAGATGCTGGTGAGACTGCCGTGGTG

GCATTTAGCATCGTTAAAACTGGAAAACTCTCAAGCTCTTTGCCACTTTCCTACTATTTTTTGATTCTTG

CCATTTTACCAAGCTTAGGTTGTGAAACTTGACAGAAATGTATTACAGGAAAAACTTATAATTGTATTTG

ACTTTCTAACACATTGCAAAGTTTCAAAGTGACTTTCACTTTCAACAACATATTAGAAGTAACCACTTTT

GCTTTCACAGCCTGAAGAGTTAGAGCCTGATCTGATGCCCCCTTTCACTCTGAAGTCATGGGAAATTTTC

CAGCCATGAAAGCCCTCTTTCCACTGCATACTGATGGGCTGACTCAGCTTCCTTCAGCCGACTGAGATCT

TTTCATACTATTGGCTATTTCATACCAATTAACCTCTTAAATAAGATTGTGAATTGCCAAAATTGATAGA

CACTTATTACCACCTGTGGACTCCATATTCCTTACCACAAATGTTATTTTCATCAGTCCTGAGTCATTTT

AACTTACAGAAATTAGGATTGTTGCTGCTAATATGAATACCAATTATAACTTTTAGAAACAAGAATAAAG

CCTAAAAGAGAATGAAATATAAGAAATGTTCGTTCCCACCCCTAATAACATTTGGAAGTGAATATTCCCA

TTTTCTTCCACCCACAGGGATTGGGATTGATTTTTAATTTCCTAGGAAACAATACTAGACTACCCAAAAA

GATGTTGCCAGAATCCAAAAGGAACTATGCTCGTAAAAGAAATGCAGTTTTCTCCTACCTAAAAAAAAGA

AAGTAAAGTGTGTTCTGTTCTTATCTTTTTAATGACTAAGCTTTAAACAGTTTATTTTGGGTAAGACTAG

AACTTTCGGCCATTTGTTCTAATATGTGTGTTATTAGATGCAATAGAATTTATGAAAAGAAGAATGACAA

AGGTATCTGATTAGAAAATTTGATCTTACGCATGAATCCATGTCATGGCCAGCCACTGTCACATAGTGGG

TGCCATTCTCAACATATTGGTTTGCTAACTTTAAGCATTAGGGATTTAGCACACTAAAATACTTTTAATT

ATATTAGGTTTGGTAACTAAGGAGTAAATAAATCATAATTTATCATTTGCCAAGGCCAACAAACAACACT

ATTGTGCTGTTTGCTCTCAATGAAGTTGAATAAACCAGGAGGCTTGGCATATCCCCTTTATGTTAATCCC

AGCTAGAGATTAGTAGGTTGACTTTCACAGCAATTGTATATTGATCCATTTTAACTCATCCTTGCCATAA

TTTCCAGGCCAGTCACCAGGACAGAGGAGATGATGGGGAAACAGAGCTTTAGATGAAAACTACTATGCAC

TACTAGCCTTAGAGGCACTGGTTTCCTGTTACCACTTTGGCAAGTATGGATGGTCTAAGTCCAGTAGGGC

TTCATCCATGGAGCCATTAGAACTGAGGGGGGAGTGTTAGAGATGCCATTTCACCAGGATCTTTTTGCTC

AGGTTGTACCCATGCCAATTGAAGAACGTGTTAAAGATGAGGAGGAGAGATGTACCATTCTCTCCCTTAA

TAATGATGTTGGTTTGCAAAACCTAAAGAAATAATAACAACAGACTATTTCATACTTTCAAGCAAGTCTT

TATACTACCTGTTATTTCTCTAAAATTCAAATAAAGAATTTTTAAACTTA

>NM_001131028.2 Homo sapiens autophagy related 10 (ATG10), transcript variant 3, mRNA

GCAGTGCGCACGCTCCGACTCGGCCGTGGCGGACCTGACTGAAGGAGGCCGCGGACCTGACTGAAGGAGG

CCACGGCCACTTCTGGTTGGCCTCGGGGCGCGCTGGCTCGGCTCTTCCTCCGCCCTCGAGGCCCCCGCAG

TCCCATCATTCAGTTCCGTAGGGTCACCGGCGCGGCAGTGGCCTCGCAGGGCGCTGGGTCCCTCTCCCCA

GCTCTCCTCCCCCTGGCCCCGTCGCCCCGCCCTCGCCGGGCTGGGCTGCGGGGTCAGGGGCCGAGCGGAG

AGGGTAGAGACGGGGTTTCACCGTGTTAGCCAAGATGGTCTCGATCTCCTGACCTCGTGATCCGCCCGCC

TCGGCCTCCCAAAGTGCTGGGATTACAGGCGTGAGCCACTGCGCCCGGCCTGTTGTACAGTTATTAAAGT

TATCATTTAACATGGAAGAAGATGAGTTCATTGGAGAAAAAACATTCCAACGTTATTGTGCAGAATTCAT

TAAACATTCACAACAGATAGGTGATAGTTGGGAATGGAGACCATCAAAGGACTGTTCTGATGGCTACATG

TGCAAAATACACTTTCAAATTAAGAATGGGTCTGTGATGTCACATCTAGGAGCATCTACCCATGGACAGA

CATGTCTTCCCATGGAGGAGGCTTTCGAGCTACCCTTGGATGATTGTGAAGTGATTGAAACTGCAGCAGC

GTCCGAAGTGATTAAATATGAGTATCATGTCTTATATTCCTGTAGCTACCAAGTGCCTGTACTTTACTTT

AGGGCAAGCTTTTTAGATGGGAGACCTTTAACTCTGAAGGACATATGGGAAGGAGTTCATGAGTGCTATA

AGATGCGACTGCTACAGGGACCATGGGACACTATTACGCAACAGGAACATCCAATACTTGGGCAACCCTT

TTTTGTACTTCATCCCTGCAAGACGAATGAATTCATGACTCCTGTATTAAAGAATTCTCAGAAAATCAAT

AAGAATGTCAACTATATCACATCATGGCTGAGCATTGTAGGGCCAGTTGTTGGGCTGAATCTACCTCTGA

GTTATGCCAAAGCAACGTCTCAGGATGAACGAAATGTCCCTTAACAAGATTCTTCTATTGAGTTTAGGAA

TTGCGGCACGAAGAATGCCAAGAGTTTACCTGGCCAGCCCTGGCTTTAATAGGACTGATACCATGGAATA

TTTCATCTCACCAAGATGTGACATGGATTATTTTTCCCTTGGACACAAATGTCTACAGCAACTGGTGTTT

GATAGGCTGAATGTTTAGAAGAAACACTTCAAAGGGATACATCATGGCCAGGCATGGTGGCTCACACCTG

TAATCCAAGCACTTTGGGAGGCCAAGGTGGGAGCATCACTTGATCCTGGGAGTTCGAGACCAGCCTGGGC

AACATGGTGAAACCCTGTCGGTACAAAAAAATACAAAAATTTGCCTGTTTATGGTGGTGTGTTCCTGTAG

TCCCAGCTCCCCAGGAGGCTGAGGTGGGAGGTTGGCTTTAACCCAGGAGGCAGAGGTTGCAGTGAGCTGA

GACTGTGCCACTGCAGTCCAGCCTGGGTGACAGAGCCAGACACTGTCTCGGGGAAAAAAAAAAAAAAAA

AAGACACATCACTATAAATAGCAAAAAAACAAATCTAACTTATTAATACTAGGAATACCAACATTATTAG

GGCACTTGCAGGTTATTCTTTTCTAGGCCAAGTACTTCACTTCCATTTGTCTGACATGGAGATTGAGGGA

GAAATGTATTTGTGTGTTCATTTTAATGTAAGATATATAAAAATTAAATTACTGGATTTACCTGTCCCTG

AAACTGGTGTTATAAACATGACCTATCTTAAGTGATTTTCCCACAATCAAACTCAGGAACAATAGATTAT

TTCTGTTTTACTCCAAAAGAGAGAGAGAGAGTGAGTGTGAGTGTGTGTGTGTGTGTGTGTATGTGTGG

GTGTTTGTGTAGATAGTTGTAAAACAAAGAAAAAACACAATATTTTACTGTGAGATAATATGTTTTACCA

GCAAAGTGTGGCATAGTAATTAGAAGTTTTCTAAAAAGCTATAGGAGATATTTAAACATTAAAATTTCTT

TTTGACCTATAGTAATAAAACAATGGTCATTTTACCCCTCTGCTTCTCAACCCCACAGCTGCTCTGCTGT

ACTCTTTGAGGGCTCTTGAGCGAGTCTTCATGTCCCTGAGACTTATTTTCCTCATCTTTAATTTGAAACT

AACAAGCTACCTCATAGGGTTGCTGTGAGAACCACATGAGATCATTAATGCATGATAAGATATTGTAAAG

TATTATACGAATATTCATTAAATGCTCACCTTTCTTGTATATAATTGGTATTCACTAAGGCTGTAAATAA

GTTTCATAGCCAGTTAAGTATTAAGATAAACCTAACCTGGAACAATTTTTTTTTTGGTTGCTTGTTTTGT

TTTTTGCTTTCATTTGCTAGGAGCTGAAACGAAGATTAAAATGTCATTGGCTTTTTAGAGAGTCTAGATT

AGTGTGTCTTTGAATGAGGGATATACTTAAAATCACCTGAGGATCGTTTACAGCTACAGATGCTGGGGCC

ACCTGACACAGTCTCTCTGGGGGTGATTCTGCTCTGGAGAACCTCTGGTCTGGATATACCTTTATTGTGA

CATTGATTTGTATTGTTTTGCTCTACTGACAGGCCTTTTCCAATCCATACAAGCTGAACTTGCTTCCTAA

TGTTTGTCTAGGCCTTACATGGATAGTAAGGAAGCTTAGGAGACATGTACACAAGGCAGCCCACTTGTAG

GAAACTGCTTCAGGTGAGGACATCATTCACCAGTGGATCACCGAGCACCGTGACAGCACCCTCAGTCCAC

GTCTGCAGATTAAGAGCTGAACTCTCACAGAGAAAGTGGAAGTAGAGAAAGGGTTAATTCAAACTGCTAG

AAAAGCTAAAAGTAAATTTTAAAATGCTGAGCTGCCCATATTCATGCTAGGATGAGAGCCGGCTTAAATG

CTTGCTGGGCATTTTGGTATTTACCACTTAGCATGTTTTCAACTTCTGTGAGGGATTTTCCCCCCAATCT

CCCTAATGTAAACACAGATTTCTCAGCAAGTCATTTCTCAAGCCATGTCATTTCTAGTGTAAAATAAATT

ATAATAATGTGTCA

>NM_021934.5 Homo sapiens autophagy related 101 (ATG101), transcript variant 1, mRNA

CCGGTCCCGGCTCAGCCTCCGACCCAGGTGGTCTGGAGCCTGCCGGGAGAGTGGTGGCATCTGAGAGGCT

GGTCGTGGACTGTGGTTGGGGGAGGTGGGAGCTGTTTTAACCGTGTGCCCCCTCTCCTGTGCCGGCGTGG

GCATCCCCCGGGGCAGTGGAACGCGGGCGCTCCTCCAGCTTCCGAGTCCAGCCAGCCTGGGCGCGGGGCG

CCGCCCCCGAGACACCCGAGGAGTCCGTTCCTCCCTGGTTACGTGGACTGTGGAGCTGGTCTCTTGTGGC

TCAGCGCCGTGCGGAGGTTGAAGCGTACCTGCGGAGGTCGCACCAGGGCGTGAGGAGGAGGAGGAAGGGC

ATGAGCCGAGCTTGAGGAATCCGTGCTCCAAACTCTACACTCAAGGGTGGCCCTTGGGTAGGGTGAAGAT

CCCCTGTCTTTATCCTAGTTCCACACCTTGGTGTGGGTTACTGGGTGCAGGATGAACTGTCGCTCGGAGG

TGCTGGAGGTGTCGGTGGAGGGGCGGCAGGTGGAGGAGGCCATGCTGGCTGTGCTGCACACGGTGCTTCT

GCACCGCAGCACAGGCAAGTTCCACTACAAGAAGGAGGGCACCTACTCCATTGGCACCGTGGGCACCCAG

GATGTTGACTGTGACTTCATCGACTTCACTTATGTGCGTGTCTCTTCTGAGGAACTGGATCGTGCCCTGC

GCAAGGTTGTTGGGGAGTTCAAGGATGCACTGCGCAACTCTGGTGGCGATGGGCTGGGGCAGATGTCCTT

GGAGTTCTACCAGAAGAAGAAGTCTCGCTGGCCATTCTCAGACGAGTGCATCCCATGGGAAGTGTGGACG

GTCAAGGTGCATGTGGTAGCCCTGGCCACGGAGCAGGAGCGGCAGATCTGCCGGGAGAAGGTGGGTGAGA

AACTCTGCGAGAAGATCATCAACATCGTGGAGGTGATGAATCGGCATGAGTACTTGCCCAAGATGCCCAC

ACAGTCGGAGGTGGATAACGTGTTTGACACAGGCTTGCGGGACGTGCAGCCCTACCTGTACAAGATCTCC

TTCCAGATCACTGATGCCCTGGGCACCTCAGTCACCACCACCATGCGCAGGCTCATCAAAGACACCCTTG

CCCTCTGAGCGTCGCTGGATCTCTGGGAGCTCCTTGATGGCTCCCAGACCTTGGCTTTTGGGAATTGCAC

TTTTGGGCCTTTGGGCTCTGGAACCTGCTCTGGGTCATTGGTGAGACTTGGAAGGGGCAGCCCCCGCTGG

CTTCTTGGTTTTGTGGTTGCCAGCCTCAGGTCATCCTTTTAATCTTTGCTGACGGTTCAGTCCTGCCTCT

ACTGTCTCTCCATAGCCCTGGTGGGGTCCCCCTTCTTTCTCCACTGTACAGAAGAGCCACCACTGGGATG

GGGAATAAAGTTGAGAACATGAGTTTGGGCTGA

>NM_004707.4 Homo sapiens autophagy related 12 (ATG12), transcript variant 1, mRNA

GTCTCCAAGCAAGATGGCGGAGGAGCCGCAGTCTGTGTTGCAGCTTCCTACTTCAATTGCTGCTGGAGGG

GAAGGACTTACGGATGTCTCCCCAGAAACAACCACCCCGGAGCCCCCGTCTTCCGCTGCAGTTTCCCCGG

GAACAGAGGAACCTGCTGGCGACACCAAGAAAAAAATTGACATTTTGCTAAAGGCTGTGGGAGACACTCC

TATTATGAAAACAAAGAAGTGGGCAGTAGAGCGAACACGAACCATCCAAGGACTCATTGACTTCATCAAA

AAGTTTCTTAAACTTGTGGCCTCAGAACAGTTGTTTATTTATGTGAATCAGTCCTTTGCTCCTTCCCCAG

ACCAAGAAGTTGGAACTCTCTATGAGTGTTTTGGCAGTGATGGTAAACTGGTTTTACATTACTGCAAGTC

TCAGGCGTGGGGATGAACCACAAAGAAAATCAACTTGCTACTACATGAAATGGATTTTCACGGAAGAGAC

AGCTCTGAAAAGTTTTGATGCTTGTGGCAAGAGACTTAACAGATGTGATCTATTTAGTATGTGTCTACTC

TATGTTTATGCATAAGAAAACATCCATAGCATGAATGGACTCAGAAAAATGTGATTTGTATTAATGCACC

AGTCATCATAAAAGATGGTCATGATAGTACACCCATTGCTCCTACTTGTTACTATTATTGCTGCAGATCT

GCCTCCAAGGTTGAAAAGGAGACTAAGACTGTATAAACATCTTCATTGTCAGTTCTCAAAATGACTGAAA

TTGTTTTCATGGTAAAAGTTAATATACTAAAGGGTTCCTTTTTTTTTAATGTTTACATTTATCTCTATGT

TTACCTTTTTAGTCACATTGACCTGCTGGCTGAATACCTCAAATAGTCCAGTAGAGGGCAGTCCACCAGG

CAGAAAAGGTTAGGCGTTTTGGTTTCACATCTTTGCTGGGGAATAATAGGGGAAATGGCTGTTTTTGCTA

ATTTTTAGCTAATATCTAGCCAGGAGAGCAAGCACATAGGACAGACTGAAAGACTGTAATTTTACACAAT

ACACATGGCTTAATTATTTTATTGGGATACAGAAAAATATAAATTCTGGACAAATAAGTCATATACCTGT

TTTCAGTCCTAACATTTAAGGATTCTTGAGTCCCAATCACATAACTGTGGTGTTACTCTGTCATTTATAT

GGTGTCAAAAGCACTTGATGAGTAAACCCAGTAGCATCTTTTTGAGTGTTTCATAATGCATTTTCCAACT

TGAAAACAATAATTGAAAAATAGCCTTATTGTATATTTTATGCCATGACTAAAAGTGCCATTTTTACTGA

TGCTATTAGACTGATAATTTCTTGAAGTGAAATTTAACCTTTTTTTCTCTTTAGTATTATGTTTATAATG

CCATATTTTTAGAAAGCATTCCAGATCAGGCATGGTGGCTTACACCTGTAATCCCAGCACTTTGGAAGGC

TGAGGTGTGGGGATTGCGTGAAGCCACAAGTTTGAGACCAGCCTGGTTAGCAAGGCAAGATCCCCAACTC

TACAAAAAAATAAAAATTAAAAAAAAATTATTAGGCTGCAGAGGCAAGAGGATCCCCTGAGCCCAGAAGT

TCAAGGGTATAGTGAGTCGTGATTGTACCACTGCATTCCTGCTGAGCAACAGAGTGAGACCCCATCTCAA

AAAAGAAAAAAAAAGGCATTCTAGTAAATCGAATGTAATGTGAATGGAATTTCAAAACAGGATCTAAGAT

GGTATGTAGTAGAATTCAAAGTAATATCATTTTAAAGTTAAATGAGTATGGAAAAGGTCTGTTCTCTAGT

TTTGTCCAGTTCAGTTTACTGAAGGAATATATTTAATTATATTCATATATTTAACAAATAAAAATATGTT

GAATTTTCGTATTGTTTGCCACTGAGGGTTCAGATGATAGACCTCAAAAAATCGAAAATACTGGTTGAAA

TTTGTAGCATCCATTTAGTTATTCTTTTTGACCTAAATAACTTAATAGTTTATTAAATCTAAGGTTAGCT

AAATATGTAGCTAACCTTATTTGTTTTCTTTCCTAACAACTCTGAAGAATACATAGGACTTTGCACTTTT

TTTTTTTTTTTTTTTTTTTAAAGAGACAGCGTTTCGGTCTTGTTGCCCAGGCTGGACTGTAATGGCACAA

TCTTGGCTCATTGCAACCTCTACCTCCCGGGTTCAAGTGATTCTCCTGCCTCAGCCTCCTGAGTAGCTGG

GATTACAGGCACCTGCCACCACACCCGCCTAATTTTTTGTTATTTTTAGTGGAGACAGGGTTTTACCATT

TTGGCCAGGCTGGTCTCAAACTCCTGACTGACCTCAGGTGATCCACCCGCCTTGGCCTCCCAAAGTGCTG

GGATTATAGGTGTGAGCCACCGTGCCGGGCCTGGACTTTGTACTTTTTCAAATCATATTTAATTATTTCT

TGATCTTTACTCAAAGAATTATTCTGCGCTAAATTTGCAACATTAAACAATAATTAAGCCTGGATCCGAT

AAGTTATTGGGTGACTAGAACCTACAACACAATTTATTTTTAATTTAAACTCTCAAGTCTGATTAAACTT

GGGGGAAAAACTGAACATTCAGGTGATATTTTAAATTTTCTTTGTGACTAAATAGATGTCATTAATATCA

TGCTTCATCTTACTGTGCATAGATGACCCATGGCACACAAAGTATATGTATAATATTGCAATATTGTACT

TCCATCTTTAAAAGTATGCCAAAGACTTCATAATGAATTCACCATATTTAAGGAATAAGGCAAATTAATG

TTTATCCGAAATCAAAATGAGATTGGATAATTCCTGGAATCTGATTTCTGTAAGTTTCAGTCCTAAAGGG

ACCTTAAATATCTACTAGAACGAAGTACAGTGTATTTCTCAGATTACATGACATTAGTCAACCAATTTAG

TTGTAACACAAAGTGAGAAAGCCTTAGGTGTTGAAGGAGTGTGAAAATAAAAATCTCTTTTCATGCAGTT

TCTGCTTCATTTTCCAAGGATTATAATATGCTGAGTAAACTTTTGGCACTAAGGAAGCCAGCTACAGGCC

ACGTAATGAAAACTATTCAGAAAACAGTTCAGCAAATACTACTATTTGAATACAGTTCAAATCGTATTTA

TATAAATACTCTGCCTACATTATTTAACCCAAACTGGATTATTCACCATTCTTTGAAGATGCCTTGTGTT

TTCTGTTATCTACTTCTGCTCGTGCAGTTTACTTACACCTTCACCCTTTCAAATCCTAACTCTTCTTCAA

GGCCTGATTCAGATTTTAACTTTTTAAAGGCTATCTGAATCATTCAAGGAGAAGATACCCTTTCTCTCAT

AAAAACACTTAGAGCAAACTACCACTATTAAATCACTTATTGCATACTGCCTCAGAGCCATGTGTCCAGT

TATTCTGGTTTTTATCTGGTACCCACACACACATAATACTTAGTACATTTCCTTATATGTAAATAATTAG

TAATGTAACACATCGAGTGAATGAAATTGTGATTAGCAAAGTATGACAGCTAAAAACAAAGGAAATTTTG

TATAGTTTAAGTTTTTGTACAGCAGAAGGTAATGGAAAATATTTGCAACATATGACAGTGTTTTTAATGT

CTTAAGTATATGAAGAATTTGTTTCAATTGGTAAGAAAAGACCAACTGAAAAATAGGCAAAACATACCAA

ATTTCGATTCACAAAAAGATACAAATGGCCAACAAAACATGAAACACCTTGAACCCCATTGGTTACAAAA

AATACAAATAAAAACAATGCTGACTGACAATAAAAATAATGTACATCTTGCTATTAGCAGAGTTGTGGGG

AAATCCTGTCATATTTTGGTGAATAGAATATGAGTTGGTATATCACTTTTAGGGGGCCATTTGGCAATAT

CAAAAACCTTGAAACGTAAGTATAAACTTCAGTTTGTTCGTATAACCTGTCATTCACATATTGAGTGCCT

ACTATGTCCTAAATATTGGAATAACACTAATAAAGCTCATAAGAGACTAA

>NM_014741.5 Homo sapiens autophagy related 13 (ATG13), transcript variant 2, mRNA

GGCCATTACCGAAGCGGATGAAAACAAACACTAACGATGGCGGCGCCGGGAAGCGACCGGCTGCTGGGCT

TAAGGCGGGAGTGACCGCTTAACCAGTGAGGGAAGCACTGAAGAGCGCCAGTCGACGTGGGTGCGACAAC

TCGCGGAGTCTTAGGAGCAAAACGTCTGGGGCCTGCGAGCCAGGACCCTTCTGAAGCCTTAGGTGTCTAT

CGGCGACGTGTACGGTCACTGCAGCTCCGGAGCGCGGAACCCTCAGCCAGGAGGCGCGGCTGGTCGGTCC

CAGGTCCCGGCCTCCGTAATGAGAGCCCGGAACCACTCTTTGTGCCGCAGCTTCGCAGCATCTTGGACTC

AAGTGATTCTCCTGCCTCAGCCTCCTGAGTAGCTGGGACTACAGATTCCTATAGGCAATGGAAACTGATC

TCAATTCCCAGGACAGAAAGGACCTGGACAAGTTTATTAAATTTTTTGCCCTCAAGACTGTCCAAGTGAT

TGTCCAGGCTCGGCTTGGTGAAAAGATTTGCACTCGTTCATCATCTTCTCCAACGGGTTCAGATTGGTTC

AACTTAGCAATCAAAGACATCCCAGAGGTTACACATGAAGCAAAGAAGGCACTGGCAGGACAGCTGCCTG

CAGTCGGGAGGTCCATGTGTGTGGAGATTTCACTTAAGACTTCTGAGGGAGATTCCATGGAGCTGGAAAT

ATGGTGTCTTGAAATGAATGAAAAGTGTGATAAAGAAATCAAAGTTTCCTACACGGTGTACAACAGACTG

TCATTGCTGCTGAAGTCCCTTCTTGCTATAACTAGGGTGACACCAGCCTATAGGCTCTCCAGGAAACAAG

GGCATGAATATGTCATATTATACAGGATATATTTGGAGAAGTTCAGCTGAGTGGCTTAGGAGAAGGCTT

CCAGACAGTTCGTGTTGGGACAGTGGGCACCCCTGTGGGCACCATCACTCTTTCTTGTGCTTACAGAATT

AACTTGGCATTCATGTCTACCAGGCAATTTGAGAGGACCCCACCTATCATGGGGATTATTATTGATCACT

TTGTGGACCGTCCCTATCCCAGCTCCTCTCCCATGCACCCCTGCAATTACAGAACTGCTGGTGAGGACAC

TGGAGTAATATACCCGTCTGTAGAAGACTCTCAAGAAGTGTGTACCACCTCTTTTTCCACCTCCCCACCA

TCCCAGCTGATGGTTCCTGGGAAGGAAGGTGGGGTACCCCTTGCTCCCAACCAGCCTGTCCATGGTACCC

AGGCTGACCAGGAGAGACTGGCAACCTGCACCCCTTCTGACAGAACCCACTGTGCTGCCACACCCTCCAG

TAGTGAGGATACTGAAACCGTATCAAACAGCAGTGAGGGACGGGCCTCCCCTCACGATGTCTTGGAGACC

ATCTTTGTCCGAAAAGTGGGGGCTTTTGTCAACAAACCCATTAACCAGGTGACCCTGACGAGTTTGGATA

TACCCTTTGCCATGTTTGCTCCCAAGAATTTGGAGCTGGAGGATACCGATCCAATGGTGAATCCTCCAGA

TTCCCCAGAGACTGAATCTCCTCTCCAGGGCAGCCTGCACTCAGATGGCTCCAGCGGGGGCAGCAGTGGC

AATACCCATGATGACTTTGTTATGATAGACTTTAAACCAGCTTTTTCTAAAGATGACATTCTTCCGATGG

ACCTGGGGACCTTCTATCGGGAGTTTCAGAACCCACCTCAGCTGAGCAGCCTCTCCATAGATATTGGAGC

ACAGTCCATGGCTGAAGACTTGGACTCATTACCAGAGAAGCTGGCTGTGCATGAGAAGAATGTCCGCGAG

TTTGATGCCTTTGTGGAAACCCTGCAGTAAAAGTATCCTTGAGTCCCAGCAGCACCCCCTTTTTGTGGCC

CCAGGGCATAAGCAGCCTCCCATGCATCAGCTGCTCCCACCCCTCATCCTGCTCTGAGCCAGGTGGAAGG

GAGGCTGGCTTCTCCCATGGGGACCCAGAAGTCCCTACTCTTGGACCTCCTGGAGACTCCGTGGCGGCAG

TCAAGCCCAGTGCCCAGTTGGAGAAGACTCACGTGCTGGCCTTGGAGATGGGAAGAACCTTCGTACGAAA

AAGCCCTCAGCAGGGCCATCTGTGTGCCCTGCCCATCACCAACTGCTTCCCAAGGGTGTCATCCTGTTCC

TCCTGCTGCCGGCCTCCTGCCTGGGCCTGCCTTGCAGCTGGCCCCTTCCCTGCCTGCTGTCACCATCCAC

TGTTTGACATTCCAGCTGGTGGCCAAGAGATTGGTGTGGAGGCAGAAAGAGGAAGGAGACAGTGCCAGGA

GGAAGAAGGAAGGAGTCCCTTAGCTCTCTTCATTGTCCCCTTTACTTCCTGCTATCTTCTTCTCCTCTTC

TTCTCTCTCTTGCCTCTATGCCTGTATTTCTGGCAATATGACAGGCCTGCCTACCCAAGATCAGAACTCC

AAAACCACTCCCACCCCTGAAGGTCGGGAGGGTCTGAGCAGCCCTGGTGGCTGCCTGTGCTCAGGTCCTC

AGCTCCATGGGAAATAAAAATGGCACCCTGAATCTCTAGGATTTTGTCACTTGGAGTCACAGCAAAGTTC

TCTTCCTCTTGTCCCCCCGTTGCTGCTCCTTGGTTATAGAACATGGTAAATATTTATTACTTTCAGAGAA

ACCAGATATTTTATAGAGGAAATATGTTTGAGGTGAGTTGTTTTTCACTTGGAGAAGGCGGAGGGCTCTT

CCTGGGACGGAGACCTCCTCCTCCGGAGGTTATTGAGAATCCGGGCTGCTGCTTTGAGGATCTTCCCACC

ATACAGACAGCGAGATCCAAGAAGAGGGCTGGCCGGGGGCAAAGTCACCTCCCAGTGTGGCTGCACTGGA

ACTGACTAAAGGCTTTACCTTGGATAGTTGCGTATTCCTGGTGAGAGCCTTACATCTCCCACAGTTTCTG

CAGAGTGACTGACTCCATTCTGGCAGCCCAGGAAGTCCTGGGTGCTAAATGTGATGGCCACATGTAGTGG

TTAGGGGATGTTGTGTGTGTCCCCCAACTGCCTGGGTACTTGTTCCTGATCCCTGGGGCTGTCCTGTGGA

GCTTTTCCTCCTGCTTGGGCCTAGCTACCATCTCCCTCTAATCCCAGGTTCTCTACACTGCCCTGGGGTT

TACCAGCTGGATTGGCTTCTGGTTGAGAAATCAAAGCTGGGCGTATGATTGACTTAACCCTTCAGGTATT

GTTACTTGAATAAGTCAAGTGCCTAGCCTCACCCACCTATGATCTGTCCTTTCCCAGCCTCGCTGGTAGT

CCTGGTCAAGGAGATCTAGGTCTACTCCATTCCTCCTGGCCCACCTGGGGCATTCACTGGCAGCAGCTGT

GCTTCAGTGGAGCAGGTGGTTCTCAGCTGCTTGTTAGTATACTGCATGTGACACTGTTCCCACATACAAG

GCTGACTTCTGAGGATTGGAGCAGGCTCTGGCGGGGACCAGAGCTCTGCGTGCTGCTGCTGCCACCAAGA

AGTGTTAGCAGAAGCAGTAGCAGCCAACTGGCCCTCCTGACTTTGGCCCAGAGCACATGCGTGGCTTGCT

GAACCCAGGCTCAGGTTTATCCCCAAGGCCCCAGCTTTGAGAAGGGGGAAGGCCCCTGGTAAGTTATTGA

TGCCCCCATATTTCAGCTACTGCTCTCTTTCCAAGGCCTTGCATGGAAAGGCCTAGCCATTGTCTGAGGC

AGCAATCTTTGGCATCTACAGGTGGCAGCAGCCTTTCACCAGGGCTCCATCTGTGAAGAGTCTCAGCCAT

GACTTTGAGCTGAGCTTGGGAGAAGTAAAGCAACTGTTAAGGCCAGCCCTTGCCCCTCAGACCTGCCATG

AAAGGAATGAGCCCTAGACTGACTCCTGCAGCACCCCCGGGACAGGCTGGGACCAGCTGTTTGTCTCCAG

GTGTCAGAGTCCCTCCTCCTCCTCCAACCTCTCCAACCTACTTTGTTTGGAAATACCGAGCTACACTTCA

AAATGTATTCAAGGGATTTCCAATAAATTTTTTTCTGTACTTTA

>NM_014924.5 Homo sapiens autophagy related 14 (ATG14), mRNA

AGGCCATCATGGCGTCTCCCAGTGGGAAGGGAGCCCGGGCGCTGGAGGCTCCTGGCTGCGGGCCCCGGCC

GCTCGCCCGGGACCTGGTGGACTCCGTGGACGATGCGGAGGGGCTGTACGTGGCTGTGGAGCGCTGCCCG

CTGTGCAACACTACCCGCCGGCGGCTGACCTGCGCCAAATGCGTTCAGAGCGGCGATTTCGTCTACTTCG

ACGGCCGCGACCGGGAGAGGTTTATCGACAAGAAGGAAAGGTTAAGCCGACTTAAGAGCAAGCAAGAAGA

ATTTCAGAAAGAAGTGTTAAAAGCTATGGAAGGAAAATGGATAACAGATCAGTTGAGATGGAAAATAATG

TCCTGCAAGATGAGGATTGAACAGTTAAAACAAACAATATGTAAAGGAAATGAAGAAATGGAGAAAAATT

CTGAAGGCCTTCTCAAAACCAAGGAAAAGAATCAGAAGCTTTACAGTCGAGCACAACGGCACCAAGAGAA

AAAGGAGAAGATTCAGAGGCATAATCGCAAACTTGGTGACCTGGTAGAAAAAAAGACCATTGACTTAAGA

AGTCATTATGAGCGTCTGGCAAATCTTCGACGATCCCATATATTAGAGCTCACCTCTGTCATTTTTCCAA

TCGAGGAAGTAAAGACGGGTGTGAGAGACCCCGCAGATGTGTCTTCAGAGAGTGACAGTGCCATGACCTC

CAGCACTGTGAGCAAGCTTGCTGAAGCCCGGAGGACAACTTACCTCTCAGGACGATGGGTCTGTGACGAT

CACAACGGAGACACCAGCATTAGCATTACAGGGCCTTGGATTAGCCTCCCTAACAATGGGGACTACTCTG

CCTACTACAGCTGGGTGGAGGAGAAGAAAACAACCCAGGGGCCTGACATGGAGCAGAGTAACCCTGCCTA

CACCATCAGTGCTGCGCTGTGCTATGCAACTCAGCTGGTCAACATTCTGTCTCATATACTTGATGTAAAT

CTTCCCAAAAAGCTCTGCAACAGTGAATTTTGTGGCGAAAATCTAAGCAAGCAGAAATTTACTCGAGCAG

TGAAGAAACTGAATGCAAATATTCTTTACCTTTGTTTTTCTCAGCATGTAAATTTAGATCAATTACAACC

ACTGCATACCCTCAGGAATCTAATGTACCTGGTCAGTCCAAGCTCTGAACACCTAGGCAGGTCAGGGCCC

TTTGAAGTACGAGCAGACCTTGAGGAGTCCATGGAATTTGTGGATCCCGGAGTTGCTGGAGAATCAGATG

AGAGCGGAGATGAGCGCGTCAGCGATGAAGAAACCGACCTGGGCACAGACTGGGAGAACTTGCCTAGTCC

CCGGTTTTGTGATATCCCTTCCCAGTCTGTGGAAGTCTCCCAGAGTCAGAGCACCCAGGCGTCCCCACCC

ATCGCGAGCAGCAGTGCAGGTGGGATGATCTCCTCTGCAGCAGCCTCGGTGACCTCCTGGTTTAAAGCTT

ACACTGGACACCGTTAACGAGCATGGACCAAAACATACCAAATCTGCATCAAGAAAGTTCTTCTCCCACT

ACACTCTAGTAAACATTTTCTGTTTAAGTTAAGATAGTGTCTGGAACAAAGAGGTTAAAGTGTTGTTTTG

TTTTGTCTTTTTAAGCAGGGAGACAAACATTTCTATTTGCCAAGTGGCCTGTGATGGTGACCAACATGCT

TATGATAATTAAGAGAACAGGGGTCGAAGGTCTTTCTACCCAGACCAGTGCTGGTGGAAGGAGGACCTGT

GCGTGTGGCCAGTTCTGCCAAGGAAGCAGTTGATTTGGGTTCCCTCTGGGCCCGGGCCACCGGGCCCACA

GATATGGGTCAGTGTGCTGGTCCTTGCGGTGCTGAGACTGTTCCTGACACTTTAAGTTTTAGAGGTTGGT

TGAATCACAAGAGGTGATTCTTGATTATTAGGACATGAAAGATAAAAGCTCTTTAATAAGAGTTTTTCTG

CCATTGTTTTTTGTATGAGAACCAGCAGGCAATTTAAAATTTCTAATTTGGTCCTTTGATTTTGTTTGGG

AGGGGTGAGTTACACGTATTTTATTCATGCTGCTCTGTCGTAGTTTGTCAGACATTCCTGTTTTTCTTTC

CCCCACACACCAAAGAAAATGAAAGTCTTTTTCTTTAGGACCCACATCCATAAATGGAAGAAATCCTGGC

TGCAATAATGTCTAGAGAGTTTTTAACTATTTTCTTGTATTCTGAGGGGAATTAAGCTTATTCTTACCTA

GTTGAATTCCTGCCATCCACACTATGAGCATTTTGAAATTGAACTTATATTTTCTGGGTGAAAATAAGTC

ATGAAGGTCATTCCCTTATGTAAGCTCAATGCCTGCCTGGGCACAGGGGAAAAGCCACTTAGTTAAGTGG

CCTCTGGTCATTCTTGTGGTGTCCACTTTCTTTCTATGGGATTGAGTAGGTGGCAGGTGTTTTCAGGGGA

AACCATCCTACTTGTTTCCCCGAACTCTTTGTTGCTCTGAGGACACAGCTTTGCTCAGAAATGCAGCGCA

GATCCTTACGGCTGATGCTACTCTGCTCTGTTCTGGGGAAAGCACAATATAAAGAAAGAATTTCCCAGCC

AGGCGCAGTGGCTCACGCCTGTAATCCCAGCACTTTAGGAGGCCGAGGCAGGCGGATCACTTGAGGTCAG

GAGTTTGAGAGCAGCCTGGCTAACATGGTGAAACCCTGTTTCTACTAAAAATACAAAAAATTACCGGGTG

TGGTGGCGCACGCCTGTAATCCCAGCTACTCGGGAGGCTGCGGCAGGAGAATCGCTTGAACCGGGAGGCA

GAGGTTGCAGTGAGCCGAGATTGTGCCATTGCACTCCAGCCTGGGCAACAAGAGCGAAACTCCGTCTCAA

AAAAAAAAAGAATTTCCCTCAGCAGGAGATCATTTTCAGCTCACGTGTCTTGTCATTCTTTTAGTGACAA

TCTTACAAGAAAACTATAATGAGAGAGGCATTATGTACAAATATGTAAGTAGTTTATTTTTAATAACTGC

AAAAAAATCCTATGTAACAACTACCAAAAGAAATCCTATGAAAGAGTCCTAACAGGCATTATTACCATAT

CTTATGTGATTGGCATGATAGCACCTCTGATAAATCATTCAGAGGTTTGCCATGCCCCAGCTTCTTTTCT

CATCATAATAATTGTAGTTGATACTTTGCCTCCAAGTCCGAGGTGCTATATAGCTTTTGCTAATGGTATA

TTTGGTGTTTTGTATAGTTTTGGGTAGAGTTGCAGAACGGAGTTTATTTCTATCCGGTAGTCACAAATTC

CTTGGCTCTATGAATTTTCCATGAAAGGAGGAAGTAGGCTTTTCTCGTTGTGGGTGGTCTTTTTTTTTT

TGGAGACGGAGTCTCACTCAGCTGCCCAGGCTGGAGTGTAGTGGCACCATCTCCGCTCACTGCAACCACC

ATCTCCTGGGTTCAAGCAATTCTTCCATCTCAACCTCCCGAGTAGCTGGGATTATAGGCACCTGCCATCA

TGCCCAGCTAATTTTTGTATTTTAGTAAAGACGGGGGTTTTCACCATGTTGGCCAGGCTGGTCTTGAACT

CCTGACTTCAGGTGATCCGCTTGCCTTGGCCTCCTAAAGTGCTAGGATTACAGGCCTGAGCCACCGCGCC

CGGCCCCTTATGGGTTCTTCTACACTGCTGGGATCTCTGTTTTAAGTGCTCAGCTTCATGATTGATTGCT

GGGCTTCCATTTTCCCATCCAGTTCTGGAGTTCGTAGAGAGTGAAGATGGTAGACTTGAACAGATAAATA

AACTTAACGATCTTGTAAGAGTTGTCTAGCTACTTAAAACCCTCAGAAGTAAGAGCTTAGTCTCACGAGT

TGTAAGAGTGGGATTTGGAGCTTGGTGGTGGAGACTGACTTCAGCTGAGAGATGCACAACAGTCATGGTT

TTCTTAAGCCTCTTATGAAACCATGAATGAGAGATGAAGCTAAAGAATAGAATCCAGAGATCACAAACTC

ATCTAGAGTACTTCCACAAAATTTACAAAGATGTGGGAACTTTATGGATAGGATATATTTTGTTTGTTGT

TGTTAATATCAACTAGAGGCACTTTACATAGGGTTAAGTGATCGAACCCTTTTGTGGTTTTGAACACCAA

CATACTGGCTTACACTGCTGAAATATTTTGGGTTTCATTATTTTGCACTGGATCCACCCTGTAAATACTC

TTAAGTATACATTTCAACCACTGTTTTTTCTACTCTTTTTGCTGCTCATTAAAATCTTTCATGTAGGTGC

CAGAACCATATGTAAACAGCTTTTTAAAAAAATTGAAGCTGGTATTTTGTTTAAACAAAAAGCCATAGAAC

TTGGTCATGTTTTCCATTTTAAAATGATTTACTGAAACAAAGTAATACTAATAAAAACCCACAGGCACCA

AACAGGCTGCTTAAAATGGTCTGTTAAAGACATTTTTTGGTTATGGAATATAAGAAAAGTTTTGCACATC

TGTAAGGGGGAAAAACAGTATATCACCATTGGGTAGAGTGGACGGGACTCATGTAAGGACTCAATTTGGG

GAAGAGCATTCAGTGGCATGCTGTTAGAGGACTAGTGTCCGAGAATCTCCTCACAGTATCATGTTGCAGG

AATTCCCCATTGCTCTGCAACTTCCAAACCAGTTTGAGTCATACAAATGTTTTCTAAACTTTTATTGTAT

TACTGCAATAAATCTTTTAACAGTA

>NM_017974.4 Homo sapiens autophagy related 16 like 1 (ATG16L1), transcript variant 2, mRNA

AGACCGTCCCGGATGGCCTCGGGGACTGCCAGTGTGTGGAGGTGAGCTCCGGGATTGCCGGCATTCCCGC

TTCTGCTGGTTGCTTCATGCTGCAGGCTGCGGCCGTCAGCCCTCGCTCGCATTGGTGGCGCTGAGGTGCC

GGGGCAGCAAGTGACATGTCGTCGGGCCTCCGCGCCGCTGACTTCCCCCGCTGGAAGCGCCACATCTCGG

AGCAACTGAGGCGCCGGGACCGGCTGCAGAGACAGGCGTTCGAGGAGATCATCCTGCAGTATAACAAATT

GCTGGAAAAGTCAGATCTTCATTCAGTGTTGGCCCAGAAACTACAGGCTGAAAAGCATGACGTACCAAAC

AGGCACGAGATAAGTCCCGGACATGATGGCACATGGAATGACAATCAGCTACAAGAAATGGCCCAACTGA

GGATTAAGCACCAAGAGGAACTGACTGAATTACACAAGAAACGTGGGGAGTTAGCTCAACTGGTGATTGA

CCTGAATAACCAAATGCAGCGGAAGGACAGGGAGATGCAGATGAATGAAGCAAAAATTGCAGAATGTTTG

CAGACTATCTCTGACCTGGAGACGGAGTGCCTAGACCTGCGCACTAAGCTTTGTGACCTTGAAAGAGCCA

ACCAGACCCTGAAGGATGAATATGATGCCCTGCAGATCACTTTTACTGCCTTGGAGGGAAAACTGAGGAA

AACTACGGAAGAGAACCAGGAGCTGGTCACCAGATGGATGGCTGAGAAAGCCCAGGAAGCCAATCGGCTT

AATGCAGAGAATGAAAAAGACTCCAGGAGGCGGCAAGCCCGGCTGCAGAAAGAGCTTGCAGAAGCAGCAA

AGGAACCTCTACCAGTCGAACAGGATGATGACATTGAGGTCATTGTGGATGAAACTTCTGATCACACAGA

AGAGACCTCTCCTGTGCGAGCCATCAGCAGAGCAGCCACGAGACGCTCTGTCTCTTCCTTCCCAGTCCCC

CAGGACAATGTGGATACTCATCCTGGTTCTGGTAAAGAAGTGAGGGTACCAGCTACTGCCTTGTGTGTCT

TCGATGCACATGATGGGGAAGTCAACGCTGTGCAGTTCAGTCCAGGTTCCCGGTTACTGGCCACTGGAGG

CATGGACCGCAGGGTTAAGCTTTGGGAAGTATTTGGAGAAAAATGTGAGTTCAAGGGTTCCCTATCTGGC

AGTAATGCAGGAATTACAAGCATTGAATTTGATAGTGCTGGATCTTACCTCTTAGCAGCTTCAAATGATT

TTGCAAGCCGAATCTGGACTGTGGATGATTATCGATTACGGCACACACTCACGGGACACAGTGGGAAAGT

GCTGTCTGCTAAGTTCCTGCTGGACAATGCGCGGATTGTCTCAGGAAGTCACGACCGGACTCTCAAACTC

TGGGATCTACGCAGCAAAGTCTGCATAAAGACAGTGTTTGCAGGATCCAGTTGCAATGATATTGTCTGCA

CAGAGCAATGTGTAATGAGTGGACATTTTGACAAGAAAATTCGTTTCTGGGACATTCGATCAGAGAGCAT

AGTTCGAGAGATGGAGCTGTTGGGAAAGATTACTGCCCTGGACTTAAACCCAGAAAGGACTGAGCTCCTG

AGCTGCTCCCGTGATGACTTGCTAAAAGTTATTGATCTCCGAACAAATGCTATCAAGCAGACATTCAGTG

CACCTGGGTTCAAGTGCGGCTCTGACTGGACCAGAGTTGTCTTCAGCCCTGATGGCAGTTACGTGGCGGC

AGGCTCTGCTGAGGGCTCTCTGTATATCTGGAGTGTGCTCACAGGGAAAGTGGAAAAGGTTCTTTCAAAG

CAGCACAGCTCATCCATCAATGCGGTGGCGTGGTCGCCCTCTGGCTCGCACGTTGTCAGTGTGGACAAAG

GATGCAAAGCTGTGCTGTGGGCACAGTACTGACGGGGCTCTCAGGGCTGGGAGGACCCCAGTGCCCTCCT

CAGAAGAAGCACATGGGCTCCTGCAGCCCTGTCCTGGCAGGTGATGTGCTGGGTATAGCATGGACCTCCC

AGAGAAGCTCAAGCTATGTGGCACTGTAGCTTTGCCGTGAATGGGATTTCTGAAGATTTGACTGAGGTCT

CTCTTGGCCTGGAAGAATAACACTGAAAAAACCTGACGCTGCGGTCACTTAGCAGAGGCTCAGGTTCTTG

CCTTGGGAAACACTACTAGCTCTGACCTTCCATACCTCACTTGGGGGAGCACAGGGCCCCGCTGGGCCTC

CTCACCAACGGCAGTGCCAAAATCAGCCCCCACATCAAGGTGGTGTTCTCTGTGCTTTCTCTCGTCCTTC

CAAAGTCGGTTCTGGCCTAACGCATGTCCCAACACCTTGGGTTCATTTGCCCGGTGAACTCACTTTAAGC

ATTGGATTAACGGAAACTCCCGAACTACAGACCCCTCCCTGGTGGGTTGCATGAATGTGTCTCATTACTG

CTGAAATGTCCTCACATCTCTTTCACTGTTCTTCAGAGCTTTCTGGCTCTCTTTCCCCCACAAAATTCGA

CATATTTAAAAATCTCCGTGTGGCTTTAAAAAATGGTTTTTTGTTTTTTTGTTTTTTTGAGGTGGGAGAG

GATGTGTGAAAATCTTTTCCAGGGAAATGGGTTCGCTGCAGAGGTAAGGATGTGTTCCTGTATCGATCTG

CAGACACCCAGAAGGTGGGTGCACACTGCATGCTTGGGGGTGCCAAGGGATTCGAGACCTCCAACATACT

TGTCTGAAGGTGGTGATTCTGGCCATGGCCCCTCTGCCAAGCCTGTGTGCGATGCCCTTGGTGCTTTAGT

GCAAGAAGCCTAGGCTCAGAAGCACAGCAGCGCCATCTTTCCGTTTCAGGGGTTGTGATGAAGGCCAAGG

AAAAACATTTATCTTTACTATTTTACCTACGTATAAAGTTTTAGTTCATTGGGTGTGCGAAACACCCTTT

TTATCACTTTTAAATTTGCACTTTATTTTTTTTCTTCCATGCTTGTTCTCTGGACATTTGGGGATGTGAG

TGTTAGAGCTGGTGAGAGAGGAGTCAGGTGGCCTTCCCACCGATGGTCCTGGCCTCCACCTGCCCTCTCT

TCCCTGCCTGATCACCGCTTTCCAATTTGCCCTTCAGAGAACTTAAGTCAAGGAGAGTTGAAATTCACAG

GCCAGGGCACATCTTTTATTTATTTCATTATGTTGGCCAACAGAACTTGATTGTAAATAATAATAAAGAA

ATCTGTTATATACTTTTCAAA

>NM_033388.2 Homo sapiens autophagy related 16 like 2 (ATG16L2), transcript variant 1, mRNA

GGGAGGAACGCGCCGCTAGGCGGGAGAGCGCGGCCATGGCGGGGCCGGGCGTCCCCGGTGCCCCCGCAGC

GCGCTGGAAACGCCACATCGTGCGGCAGCTGCGGCTTCGGGACCGTACGCAAAAGGCGCTTTTCCTGGAG

CTGGTGCCGGCCTATAACCATCTCTTAGAGAAGGCTGAGCTGCTGGACAAGTTCTCAAAGAAGCTGCAGC

CGGAGCCAAACAGTGTCACTCCCACCACCCACCAGGGCCCCTGGGAGGAGTCAGAGCTTGACTCAGACCA

AGTCCCATCACTGGTCGCACTGAGGGTGAAGTGGCAGGAGGAGGAGGAGGGGCTCCGGCTGGTCTGTGGT

GAGATGGCCTACCAGGTGGTGGAGAAGGGCGCGGCCCTGGGCACGCTGGAGTCGGAGCTGCAGCAGAGGC

AAAGCAGGCTGGCAGCCCTGGAGGCCCGCGTGGCGCAGCTGCGAGAGGCGCGGGCGCAGCAGGCCCAGCA

GGTGGAGGAGTGGCGGGCGCAGAATGCGGTGCAGCGGGCAGCCTACGAGGCGCTGCGCGCGCACGTCGGG

CTCCGGGAGGCGGCACTGCGCAGGCTCCAGGAAGAGGCGCGCGACCTGCTGGAGAGGCTCGTGCAGCGCA

AGGCGCGCGCCGCGGCCGAGCGCAACCTGCGCAACGAGCGCCGGGAGCGGGCCAAGCAGGCGCGGGTGTC

CCAGGAGCTGAAGAAGGCTGCCAAGCGGACCGTGAGCATCAGCGAGGGCCCGGACACCCTAGGCGATGGG

ATGAGGGAGAGAAGGGAGACTCTGGCTCTGGCCCCTGAGCCAGAGCCCCTGGAGAAGGAAGCTTGTGAGA

AGTGGAAGAGGCCCTTCAGGTCTGCCTCAGCCACCTCCCTGACGCTGTCCCACTGTGTGGATGTGGTGAA

GGGGCTTCTGGATTTTAAGAAGAGGAGAGGTCACTCAATTGGGGGAGCCCCTGAGCAGCGATACCAGATC

ATCCCTGTGTGTGTGGCTGCCCGACTTCCTACCCGGGCTCAGGATGTGCTGGATGCCCACCTCTCTGAGG

TCAATGCTGTTCGTTTTGGCCCCAACAGCAGCCTCCTGGCCACTGGAGGGGCTGACCGCCTGATCCACCT

CTGGAATGTTGTGGGAAGTCGCCTGGAGGCCAACCAGACCCTGGAGGGAGCTGGTGGCAGCATCACCAGT

GTGGACTTTGACCCCTCGGGCTACCAGGTTTTAGCAGCAACTTACAACCAGGCTGCCCAGCTCTGGAAGG

TGGGGGAGGCACAGTCCAAGGAGACACTGTCTGGACACAAGGATAAGGTGACAGCTGCCAAATTCAAGCT

AACGAGGCACCAGGCAGTGACTGGGAGCCGCGACCGGACAGTGAAGGAGTGGGACCTCGGCCGTGCCTAT

TGCTCCAGGACCATCAATGTCCTTTCCTACTGTAATGACGTGGTGTGTGGGGACCATATCATCATTAGTG

GCCACAATGACCAGAAGATCCGGTTCTGGGACAGCAGGGGGCCCCACTGCACCCAGGTCATCCCTGTGCA

GGGCCGGGTCACCTCCCTGAGCCTCAGCCACGACCAACTGCACCTGCTCAGCTGTTCCCGAGACAACACA

CTCAAGGTCATCGACCTGCGTGTCAGCAACATCCGCCAGGTGTTCAGGGCCGATGGCTTCAAGTGTGGTT

CTGACTGGACCAAAGCTGTGTTCAGCCCGGACAGAAGCTATGCACTGGCAGGCTCCTGTGATGGGGCCCT

TTACATCTGGGATGTGGACACCGGGAAACTGGAGAGCAGACTACAGGGACCCCATTGCGCTGCCGTCAAC

GCCGTGGCCTGGTGCTACTCCGGGAGCCACATGGTGAGCGTGGACCAGGGCAGGAAGGTTGTGCTCTGGC

AGTAGGGCCACGACCTGCCTGCCTGGGCTGGAGCTCTTGCCCGAAGCCTGAAGCTTCCTTCGGCGCCATG

CAGGGGTTGGGGTTGGGACTGGAGCTGGCCTTGGGATTTAATGGGGAAGAAGGCCTGGCAGGACCTGGCC

TGTTTGTTTAAAAATGAAGTATGGGTTGGGGGATTACGCTAGTTTTTCTTTGTATTTTTATCTCTATCTC

CTCACTTTTTCTCCCAAAGTAGAAAAAAATGATATCTGAA

>NM_015104.3 Homo sapiens autophagy related 2A (ATG2A), transcript variant 1, mRNA

AGTGAACAGCGGAGCCGGACGGGGATCGCCGGCGGGCGGCAAGCGGAGGCGGCCCAGGCCCGGCGGTCTC

CGAGATGTCACGATGGCTGTGGCCATGGTCAAACTGTGTGAAAGAGCGGGTCTGCCGCTACTTGCTGCAC

CACTACTTAGGTCACTTCTTCCAAGAGCACCTCAGCCTGGACCAGCTCAGCCTCGATCTGTACAAGGGCA

GCGTTGCCCTGCGAGACATCCACCTGGAAATCTGGTCTGTGAACGAGGTGCTGGAGTCAATGGAGTCACC

GCTGGAGCTGGTGGAAGGCTTCGTGGGCTCCATCGAGGTGGCCGTGCCCTGGGCTGCTCTGCTCACCGAC

CACTGCACAGTGCGCGTGTCCGGCCTCCAGCTCACCTTGCAGCCCCGCCGGGGTCCAGCGCCAGGGGCTG

CCGACTCACAGAGCTGGGCCTCATGCATGACCACAAGCCTGCAGCTGGCCCAGGAGTGTCTGCGGGATGG

GCTACCGGAGCCCTCTGAGCCACCACAGCCCCTGGAGGGGCTGGAGATGTTTGCCCAGACCATTGAGACT

GTGCTTCGGAGGATCAAAGTGACCTTCCTGGACACTGTCGTGAGGGTGGAGCACTCTCCGGGTGATGGGG

AACGTGGTGTGGCCGTCGAGGTCCGTGTGCAGAGACTGGAGTACTGTGATGAGGCAGTGCGGGACCCAAG

CCAGGCGCCGCCGGTGGACGTGCATCAGCCGCCTGCCTTCCTGCACAAGCTGCTGCAGCTGGCAGGGGTC

CGCCTGCACTACGAGGAGCTCCCGGCACAGGAAGAGCCTCCAGAGCCCCCCTTGCAGATCGGCAGCTGCT

CAGGGTACATGGAGCTGATGGTGAAGTTGAAGCAAAATGAGGCCTTCCCTGGCCCCAAGTTGGAGGTGGC

GGGACAGCTGGGCTCCCTGCACCTGCTCCTGACCCCGAGGCAGCTCCAGCAACTTCAGGAACTGCTCAGC

GCCGTGAGCCTTACAGACCACGAGGGCCTGGCTGACAAGCTGAACAAGAGCCGCCCGCTAGGTGCCGAAG

ACCTGTGGCTGATTGAGCAGGACCTGAACCAGCAGCTGCAGGCAGGGGCAGTGGCTGAGCCCCTCAGCCC

AGACCCCCTTACCAACCCCCTTCTCAACCTGGATAACACTGACCTCTTCTTCTCCATGGCTGGCCTCACA

AGCAGTGTGGCCTCAGCCCTCTCTGAGCTCTCCCTCTCCGATGTAGACCTGGCCTCCTCTGTGCGCAGCG

ACATGGCCTCCCGCCGGCTCTCTGCCCAGGCCCACCCAGCTGGCAAGATGGCCCCCAACCCCCTCCTGGA

CACCATGCGCCCTGACTCGCTGCTGAAGATGACCTTGGGGGGTGTGACCCTGACCTTGCTTCAGACGTCT

GCCCCATCTTCCGGACCACCTGACCTCGCCACGCACTTTTTCACCGAGTTTGATGCCACCAAGGATGGGC

CCTTCGGTTCCCGAGACTTCCATCACCTTCGACCACGCTTCCAGAGGGCCTGTCCCTGTAGCCATGTTCG

GCTAACGGGCACAGCCGTGCAGCTGTCCTGGGAGCTGCGGACGGGCAGTCGGGGCCGGCGGACAACCAGC

ATGGAAGTGCACTTCGGGCAGCTGGAGGTGCTGGAGTGTCTGTGGCCCCGGGGCACCTCTGAGCCTGAGT

ACACGGAGATCCTGACCTTTCCTGGTACGCTGGGCTCCCAGGCCTCAGCTCGGCCCTGCGCCCATCTGCG

CCACACACAGATCCTGCGCCGTGTGCCTAAGAGCCGACCCCGGCGCTCAGTTGCCTGCCATTGCCACTCA

GAACTGGCCCTGGACCTGGCCAACTTCCAGGCGGACGTGGAGCTGGGGGCCCTGGACCGGCTGGCCGCCC

TACTGCGCCTGGCCACCGTACCTGCTGAGCCTCCAGCCGGCCTGCTGACAGAGCCCCTGCCGGCGATGGA

GCAGCAGACGGTATTTCGGCTCTCTGCACCCCGGGCCACGCTGCGGCTGCGCTTCCCCATTGCCGACCTG

CGGCCTGAGCCGGACCCCTGGGCGGGCCAGGCCGTGCGGGCTGAGCAGCTTCGGCTGGAGCTGAGTGAGC

CCCAGTTCCGGTCAGAGCTTAGCAGTGGGCCTGGTCCCCCAGTCCCCACCCACCTGGAACTCACCTGCTC

CGACCTACATGGTATCTATGAAGATGGAGGGAAGCCACCTGTCCCTTGCCTGCGTGTCTCCAAAGCCCTG

GACCCCAAGAGCACTGGGCGCAAGTACTTCCTGCCCCAGGTAGTGGTGACTGTGAACCCCCAGTCCAGCA

GCACACAGTGGGAGGTGGCCCCGGAGAAGGGAGAGGAACTGGAGCTGTCAGTGGAGAGTCCCTGTGAGCT

GCGGGAACCTGAGCCCTCGCCCTTCTCCTCTAAGAGGACCATGTATGAGACAGAGGAGATGGTGATCCCT

GGAGACCCTGAGGAGATGAGGACGTTCCAGAGCCGGACCCTGGCACTGTCCCGCTGCAGCCTGGAAGTGA

TCCTGCCCAGTGTCCACATCTTTCTGCCCAGCAAGGAGGTCTACGAGAGCATCTACAACAGGATCAACAA

CGACCTGCTCATGTGGGAGCCTGCAGATCTGCTTCCCACCCCCGACCCCGCCGCCCAGCCCTCGGGCTTC

CCCGGCCCCTCAGGCTTCTGGCACGACAGCTTTAAGATGTGCAAGTCAGCCTTCAAGCTGGCCAACTGCT

TTGATCTCACCCCAGACTCGGACTCGGATGACGAGGATGCCCACTTCTTCTCAGTGGGGGCATCAGGTGG

CCCACAGGCCGCTGCCCCTGAGGCCCCAAGTCTTCACTTGCAGAGCACCTTCTCTACACTGGTGACAGTG

CTGAAGGGGCGGATCACAGCCCTCTGTGAGACCAAGGATGAGGGTGGGAAGCGGCTGGAGGCTGTGCACG

GGGAGCTGGTGCTGGACATGGAGCACGGTACCCTCTTCAGCGTCTCCCAGTACTGTGGCCAGCCAGGACT

TGGCTACTTCTGTCTGGAAGCTGAAAAGGCAACACTCTACCACCGAGCGGCCGTGGATGACTACCCGCTG

CCCAGTCACCTGGACCTTCCCAGTTTCGCTCCCCCGGCTCAGCTGGCCCCAACCATCTACCCATCGGAGG

AAGGGGTGACCGAGCGGGGAGCCTCGGGCCGCAAGGGCCAGGGCCGGGGACCCCACATGTTGTCCACTGC

TGTGCGCATCCACCTGGACCCCCACAAGAATGTGAAGGAGTTCCTGGTGACACTGCGGTTGCACAAAGCC

ACCTTGCGCCACTACATGGCCCTGCCCGAGCAGAGCTGGCATTCCCAGTTGTTGGAGTTCCTAGACGTGC

TGGATGACCCTGTGCTGGGCTACCTGCCCCCGACGGTCATCACCATCCTGCACACACACCTGTTCTCCTG

CTCTGTGGACTATAGGCCACTCTACCTCCCAGTGCGTGTCCTCATCACCGCGGAGACCTTCACTCTCTCC

AGCAACATCATCATGGACACCTCCACCTTCCTGCTCAGGTTCATCCTCGATGACTCCGCCTTGTACCTGT

CCGACAAGTGTGAGGTGGAGACCCTGGACCTGCGGCGAGATTATGTCTGTGTTTTGGATGTTGACCTCTT

GGAACTTGTGATTAAAACCTGGAAAGGGAGCACCGAGGGCAAACTGAGCCAGCCACTATTCGAGCTGCGC

TGCTCCAACAATGTGGTACACGTGCACAGCTGTGCCGACTCCTGTGCCCTGCTGGTCAACCTGCTCCAGT

ACGTAATGAGCACAGGCGATCTGCACCCCCCACCCCGGCCCCCCAGCCCCACGGAGATCGCCGGCCAGAA

GCTCTCGGAGAGTCCTGCCTCTCTGCCCTCGTGCCCCCCAGTGGAGACGGCCCTCATCAACCAGCGTGAC

CTGGCCGACGCCCTCCTGGACACCGAGCGCAGCCTACGGGAGCTGGCCCAGCCTTCAGGTGGCCACCTCC

CTCAGGCGTCGCCCATCTCCGTCTACCTATTCCCAGGTGAACGGAGTGGGGCCCCACCCCCTTCACCACC

TGTCGGGGGCCCTGCTGGCAGCTTAGGGTCATGCTCAGAGGAGAAGGAAGATGAAAGGGAAGAGGAGGGC

GATGGAGACACCCTGGACAGTGATGAGTTCTGCATCCTTGATGCTCCCGGCCTGGGCATCCCGCCCCGAG

ATGGGGAGCCTGTGGTGACACAGCTGCATCCCGGCCCCATCGTTGTGAGGGACGGTTACTTCTCACGGCC

GATCGGCAGCACGGACTTGCTGCGGGCACCTGCCCATTTCCCAGTGCCCAGCACTCGGGTGGTGCTACGT

GAGGTCTCCCTCGTCTGGCACCTCTATGGGGGCCGAGACTTTGGCCCCCACCCCGGCCACAGGGCAAGAA

CTGGCCTCTCAGGTCCCAGGAGCTCCCCTTCCCGCTGCTCTGGCCCCAACCGGCCCCAGAACTCATGGCG

CACGCAGGGGGGCAGCGGGCGGCAGCACCATGTCCTCATGGAGATCCAGCTGAGCAAGGTAAGCTTCCAG

CACGAGGTGTACCCAGCGGAGCCAGCCACAGGCCCTGCGGCCCCCAGCCAGGAGCTGGAGGAGCGACCGC

TGTCCCGTCAGGTGTTCATCGTGCAGGAGCTGGAGGTCCGAGACCGGCTCGCCTCCTCCCAGATCAACAA

GTTCCTGTACCTACACACGAGTGAGCGGATGCCGCGACGTGCCCACTCTAACATGCTCACCATCAAAGCG

CTGCATGTGGCCCCCACTACCAACCTGGGTGGGCCTGAGTGCTGTCTCCGCGTCTCGCTGATGCCCCTGC

GGCTCAATGTGGACCAGGATGCCCTCTTCTTCCTCAAGGACTTCTTCACTAGTCTGGTGGCCGGCATCAA

CCCCGTGGTCCCAGGGGAGACCTCCGCTGAGGCTCGCCCCGAGACTCGAGCCCAGCCCAGCAGCCCCCTG

GAAGGGCAGGCCGAAGGCGTAGAGACCACTGGTTCGCAGGAGGCCCCAGGAGGTGGACACAGCCCCTCCC

CTCCTGACCAGCAGCCCATCTACTTCAGAGAGTTCCGCTTCACGTCTGAGGTCCCCATCTGGCTGGATTA

CCATGGCAAGCACGTCACGATGGACCAGGTGGGCACTTTTGCTGGCCTCCTCATCGGCCTGGCCCAACTC

AACTGCTCCGAGCTGAAGCTAAAGCGGCTCTGTTGCAGGCACGGGCTCCTGGGTGTGGACAAGGTGCTGG

GCTATGCCCTCAACGAGTGGCTGCAGGACATCCGCAAGAACCAGCTGCCCGGCCTGCTGGGAGGCGTGGG

CCCCATGCACTCGGTTGTCCAGCTCTTCCAAGGGTTCCGGGACCTGCTGTGGCTGCCCATTGAGCAGTAC

AGGAAGGATGGCCGCCTCATGCGGGGGCTGCAGCGAGGGGCTGCCTCCTTTGGCTCATCCACAGCCTCTG

CCGCCCTGGAACTCAGCAACCGGTTGGTACAGGCTATCCAGGCCACAGCTGAGACCGTGTATGACATCCT

GTCCCCGGCAGCCCCCGTCTCCCGCTCCCTGCAGGATAAGCGCTCTGCGCGGAGGCTGCGCAGGGGCCAG

CAGCCTGCCGACCTGCGGGAGGGTGTGGCCAAGGCCTACGACACAGTGCGAGAGGGCATCTTGGATACAG

CTCAGACCATCTGTGACGTGGCATCGCGGGGCCATGAGCAGAAGGGGCTGACGGGCGCCGTGGGGGGCGT

GATCCGCCAGCTGCCCCCGACTGTGGTGAAGCCGCTCATCCTGGCCACGGAGGCCACGTCCAGCCTGCTC

GGGGGCATGCGCAACCAGATTGTCCCCGACGCCCACAAGGACCACGCCCTCAAGTGGCGCTCGGACAGTG

CCCAAGACTGAGCCTGGGGTGCCCGGCACCCAGAGGGTGCTGCCCACCATGCTCCTGAGCCTCCCAAGAG

CTGCAGCCCACGGGCCCGGCCCGGCCTGGCCCTTCAGGGGATGGCCACTGTGAAGGACGCCTTCCCAGCC

TGCCCGTTGCCAATCTGCTGTGAGAGGGGGGCCTCCCTGCCTTGGGGCCTTAGCCCTGGCTCTGCACTTT

TCCTCCGGGGAGAAAGGACACTGCCCCTCCCCCGACCTGGGCCCACACTGCTGCCTTCTCCCAGGACGGA

GGCTTTTGGACCCTCGGACCCCATCCCACTCAGCCAAGTGTCTTTCTGTGTCTGGGGGGAGGAGGGGATG

ATATCCGTGTGGTTCGATGTATTATTTTTAAGCTCCGTGAGTGCGTGGGTCAGTGTCTGCATGAAGTGGA

ATAAACTGCCCACCGCCA

>NM_018036.7 Homo sapiens autophagy related 2B (ATG2B), mRNA

ACAGCCGGGAACGCGCCTCGCGGATGACAACAAATTCGTCGCGCGGCGGCAGTGGCGGTGGCTGCCTCAG

CCCCTGCCGCCTCCTCTCGCGCTCTCTTGCACTCTCTCCGAGCCGGTTGGGACCCTCCTCGGAAGTCCCC

TGGCCTGGGGCCAGGAGGACCCCTCTGCGTCTTGCCTCCGCCAGGCCCAGCGCGAGCGGTGCGGCGCCGG

CGATGGGGTCTCCCTGAGGGACTCCCGGCACCAAATAGGGATGGAGGGGCCGCCCCAGGCTTAGGCCCCG

CCGCGGCCTGGAGCCGGAGTCGCTACCCCGAGGCCGGAGCCGTCGCAACCCGCAGCCGCAGTCAGCCCAG

CTGCCAGCAGTCACTATGCCTTGGCCGTTTTCGGAGTCCATCAAGAAGAGGGCCTGCCGGTACCTCCTGC

AGAGGTACCTGGGCCACTTTCTGCAGGAGAAGCTGAGCCTGGAGCAGCTCAGCCTGGACCTGTACCAAGG

CACCGGGTCCCTCGCCCAGGTCCCCTTGGACAAATGGTGTCTCAATGAGATCTTGGAGTCAGCAGATGCA

CCCTTAGAAGTCACTGAAGGATTCATTCAGTCAATTTCCCTGTCAGTTCCATGGGGCTCTTTACTGCAGG

ATAATTGTGCACTGGAAGTGAGAGGATTAGAAATGGTCTTCCGGCCTAGACCTCGCCCAGCAACTGGTTC

TGAGCCTATGTATTGGTCAAGTTTTATGACCAGCAGTATGCAATTGGCAAAAGAATGTCTTAGCCAGAAA

CTAACAGATGAACAAGGAGAAGGATCCCAGCCTTTTGAAGGACTTGAAAAGTTTGCTGAAACCATTGAAA

CAGTACTAAGAAGAGTAAAAGTCACTTTTATAGATACTGTTTTGAGAATTGAACATGTGCCAGAAAATTC

CAAAACTGGAACTGCACTTGAAATTCGAATAGAAAGAACTGTGTACTGTGATGAAACTGCTGACGAATCC

TCAGGAATTAATGTGCATCAACCCACTGCTTTTGCTCACAAGTTACTTCAGCTCTCTGGAGTGTCTCTCT

TCTGGGATGAGTTTTCTGCATCAGCCAAATCTTCCCCAGTGTGTTCAACTGCACCAGTGGAAACTGAGCC

AAAGCTCTCACCTAGCTGGAACCCCAAAATTATTTATGAGCCACACCCACAGCTAACTAGAAATTTACCA

GAGATAGCACCTTCTGACCCAGTGCAGATTGGACGGTTAATTGGTAGGTTGGAGTTGAGTCTCACGTTGA

AACAGAATGAAGTGCTTCCTGGAGCTAAGTTGGATGTTGATGGACAGATAGACTCTATTCATCTACTCCT

GTCACCAAGACAGGTGCACTTGCTTTTGGATATGTTGGCAGCTATTGCTGGACCAGAAAATTCTAGCAAA

ATAGGGTTAGCTAATAAAGATAGGAAAAATCGACCCATGCAGCAGGAAGACGAGTATCGAATTCAGATGG

AATTAAACCGGTATTATTTGAGAAAAGATTCCCTCTCTGTGGGTGTATCTTCAGAGCAAAGCTTTTATGA

GACAGAAACAGCTCGTACACCTTCTAGCCGTGAAGAAGAAGTATTCTTCTCCATGGCTGATATGGACATG

TCTCATAGTCTCTCTTCTCTTCCACCCCTTGGGGACCCCCCAAACATGGACCTTGAGTTATCATTAACTA

GTACATATACAAATACCCCAGCAGGATCTCCATTAAGTGCTACTGTGCTTCAGCCCACTTGGGGAGAGTT

CCTTGATCATCATAAAGAACAGCCAGTAAGAGGGTCAACATTTCCATCCAACCTAGTTCACCCAACACCT

TTACAGAAGACATCTCTCCCATCTAGATCTGTTTCAGTGGATGAATCAAGGCCTGAACTTATTTTTAGAC

TAGCTGTGGGAACTTTTTCAATCTCTGTGCTTCACATTGATCCTTTATCTCCACCTGAAACGTCACAGAA

CCTTAATCCATTGACACCTATGGCAGTAGCTTTCTTTACTTGTATAGAAAAGATTGATCCAGCAAGATTT

TCAACAGAAGATTTTAAGTCTTTCCGAGCAGTGTTTGCAGAAGCTTGCTCACACGATCACCTTAGATTTA

TAGGTACTGGCATTAAAGTATCCTATGAACAAAGACAAAGATCAGCTTCCCGATATTTTAGTACTGATAT

GTCCATTGGGCAAATGGAATTTTTGGAGTGCCTATTTCCAACTGATTTTCATTCTGTTCCTCCTCACTAT

ACAGAGCTTTTAACGTTCCATTCCAAAGAAGAAACTGGTTCCCATTCCCCTGTGTGTCTTCAGCTTCATT

ATAAGCATTCTGAGAATAGAGGGCCCCAGGGTAATCAAGCAAGACTTAGTTCAGTTCCTCACAAGGCAGA

ATTGCAAATTAAATTAAATCCAGTGTGTTGTGAGCTGGATATCAGTATTGTGGACAGGTTAAATTCCTTG

CTTCAACCACAGAAACTTGCCACAGTAGAGATGATGGCATCCCACATGTATACTTCATATAATAAACATA

TTAGTCTGCACAAGGCTTTCACTGAAGTGTTTCTAGATGATTCACATAGTCCTGCAAATTGTCGGATATC

AGTACAAGTTGCCACACCAGCATTAAACCTTTCTGTTCGCTTCCCAATACCTGATCTTCGATCTGATCAA

GAAAGAGGACCATGGTTTAAGAAGTCACTTCAGAAGGAGATCCTTTATTTAGCCTTCACAGATCTAGAAT

TTAAGACTGAATTTATAGGAGGATCAACCCCAGAACAAATTAAATTGGAACTTACCTTTAGAGAACTAAT

TGGATCGTTCCAGGAAGAGAAAGGAGATCCATCTATTAAGTTTTTCCATGTGTCTAGTGGAGTAGATGGA

GATACAACATCGTCAGATGACTTTGACTGGCCACGAATTGTACTGAAAATAAATCCACCAGCCATGCATT

CCATTTTGGAGAGAATTGCAGCTGAAGAAGAAGAGGAGAATGATGGTCACTACCAGGAGGAAGAGGAAGG

AGGTGCTCATTCCTTGAAAGATGTTTGTGATCTAAGAAGACCAGCCCCATCTCCTTTTTCTTCTCGTAGA

GTAATGTTTGAAAATGAACAGATGGTGATGCCAGGAGACCCTGTAGAAATGACAGAATTTCAGGATAAAG

CAATCAGCAATTCTCACTATGTGCTGGAACTTACGTTACCAAATATTTATGTAACACTACCTAATAAGAG

CTTTTATGAGAAGCTTTATAATAGGATCTTTAATGACTTGCTACTGTGGGAACCAACAGCTCCTTCACCA

GTGGAGACATTCGAGAATATTTCCTATGGCATTGGGCTTTCAGTAGCCAGTCAGCTCATTAATACTTTCA

ACAAAGATAGTTTTAGTGCATTTAAATCTGCAGTTCACTATGATGAGGAAAGTGGATCTGAGGAGGAGAC

TTTGCAGTATTTTTCCACTGTTGATCCCAACTATCGTTCTCGCAGGAAAAAAAAATTAGACTCTCAGAAC

AAGAACTCTCAGAGTTTTCTCTCAGTTCTTCTGAATATTAATCATGGATTAATAGCAGTGTTCACAGATG

TGAAGCAAGATAATGGAGATCTGTTGGAAAACAAGCATGGTGAATTCTGGTTAGAGTTCAATAGTGGTTC

ATTATTTTGTGTGACAAAATATGAAGGTTTTGATGACAAGCACTACATTTGCCTTCATTCTAGCAGTTTC

AGTCTGTACCATAAAGGCATAGTGAATGGAGTGATTCTCCCGACAGAAACACGACTTCCCAGCTCAACCC

GCCCACACTGGTTGGAACCTACTATTTATTCCTCTGAAGAAGATGGCCTCAGTAAAACTTCTTCAGATGG

AGTTGGAGGAGACAGTTTGAATATGCTGTCTGTTGCCGTTAAAATATTGTCTGATAAATCAGAGTCCAAT

ACAAAGGAATTTCTCATTGCCGTAGGACTGAAAGGAGCCACTCTCCAGCATAGAATGCTTCCTTCTGGGC

TTAGCTGGCATGAGCAGATTTTATACTTCTTGAATATTGCTGATGAACCTGTTTTGGGATATAATCCTCC

AACTTCATTTACAACTTTTCATGTTCATCTTTGGAGCTGTGCACTTGATTATAGACCCCTTTATTTGCCA

ATCCGATCTCTTCTTACCGTGGAAACATTCAGTGTTTCCAGTAGCGTTGCATTGGATAAATCTTCCTCTA

CTCTCAGAATAATCTTGGATGAAGCTGCTTTACATCTATCTGACAAATGCAATACTGTCACTATAAATCT

GAGTAGAGATTATGTTCGTGTGATGGATATGGGGCTTTTGGAGTTAACCATAACTGCAGTGAAGTCTGAT

TCTGATGGAGAGCAAACTGAGCCCCGCTTTGAGTTACACTGTTCCAGCGATGTTGTCCATATCAGAACGT

GCTCAGACTCTTGTGCTGCGTTAATGAATCTCATTCAGTACATTGCAAGCTATGGTGACTTGCAGACACC

TAACAAGGCAGATATGAAGCCTGGAGCCTTTCAAAGAAGGTCTAAGGTAGATTCCAGTGGTCGATCATCC

TCACGTGGTCCAGTACTTCCTGAAGCAGATCAACAAATGTTACGAGATCTGATGAGTGATGCTATGGAGG

AGATCGACATGCAACAAGGCACCTCGTCAGTAAAACCACAGGCTAATGGTGTTTTGGATGAAAAATCTCA

AATTCAGGAGCCATGTTGTTCAGACCTCTTCCTGTTTCCTGACGAGAGTGGGAATGTATCCCAGGAGTCC

GGCCCCACCTATGCCTCATTCTCTCACCATTTCATCAGTGATGCAATGACAGGTGTGCCCACTGAGAATG

ATGACTTTTGCATTCTTTTTGCACCAAAAGCAGCCATGCAGGAGAAGGAAGAAGAACCAGTTATAAAAAT

CATGGTTGATGATGCAATTGTGATAAGAGACAATTATTTCAGTCTGCCCGTTAATAAGACCGATACGAGC

AAAGCCCCCTTACACTTTCCCATTCCTGTGATTCGCTATGTGGTGAAGGAGGTCTCTCTTGTCTGGCATC

TTTATGGAGGAAAGGATTTTGGAATAGTCCCTCCCACTTCTCCGGCTAAAAGTTATATTAGTCCCCACAG

TTCGCCTTCTCACACACCCACGAGACATGGACGTAATACAGTATGTGGGGGAAAAGGAAGGAACCATGAC

TTTTTAATGGAAATACAGCTAAGCAAGGTGAAGTTTCAGCATGAAGTCTACCCGCCATGCAAACCTGATT

GTGATTCCAGCCTCTCAGAACACCCAGTCTCCCGGCAGGTGTTCATTGTTCAGGATCTTGAGATTCGAGA

TCGTTTGGCAACATCACAAATGAATAAATTTTTATACCTGTATTGCAGTAAAGAAATGCCTCGAAAAGCT

CACTCCAACATGTTGACAGTGAAAGCCTTACACGTGTGTCCAGAATCTGGCAGGTCCCCACAGGAGTGCT

GCTTGAGAGTGTCGCTGATGCCGCTCCGCCTCAATATTGACCAGGATGCTTTGTTCTTCCTGAAGGATTT

CTTCACAAGTCTTTCTGCAGAAGTAGAGCTTCAAATGACTCCAGATCCAGAAGTTAAAAAGTCTCCTGGA

GCTGATGTCACCTGCAGTTTGCCAAGGCATTTGAGTACCTCAAAGGAGCCAAATCTGGTTATTTCTTTCT

CTGGGCCAAAACAGCCTTCCCAAAATGATAGTGCCAATTCAGTGGAAGTGGTTAATGGCATGGAAGAGAA

GAACTTCTCTGCTGAAGAAGCATCTTTTAGGGATCAGCCTGTGTTTTTTAGAGAATTTAGATTCACGTCA

GAAGTTCCCATTCGACTTGATTATCATGGCAAACATGTATCAATGGATCAGGGTACGCTAGCTGGGATTT

TGATTGGTCTGGCTCAGTTAAACTGCTCTGAACTAAAGCTCAAGAGGCTTTCCTATCGACATGGTTTACT

AGGCGTTGACAAATTATTCTCATATGCAATCACTGAGTGGCTTAATGACATTAAGAAGAACCAGCTACCA

GGAATCCTGGGAGGTGTTGGACCTATGCATTCACTAGTACAATTAGTACAAGGCCTAAAGGACTTGGTCT

GGCTCCCAATAGAGCAGTACCGGAAGGATGGCCGCATTGTCAGAGGGTTTCAGAGAGGCGCTGCTTCCTT

TGGTACCTCGACAGCGATGGCTGCTCTAGAACTCACAAACAGAATGGTTCAAACCATACAGGCAGCTGCA

GAGACTGCTTATGATATGGTGTCTCCTGGTACCCTTTCTATCGAGCCCAAGAAGACCAAAAGGTTTCCTC

ATCACCGGTTAGCCCACCAGCCAGTAGACCTGAGGGAAGGTGTGGCCAAGGCCTACAGTGTTGTGAAAGA

GGGAATCACAGACACGGCTCAGACCATTTATGAAACTGCGGCTCGAGAACACGAGAGCAGAGGGGTGACT

GGTGCCGTGGGCGAGGTTCTGCGCCAGATTCCTCCGGCAGTGGTGAAACCTCTGATTGTTGCCACAGAAG

CAACGTCAAACGTGCTGGGTGGCATGAGAAACCAAATTAGGCCAGATGTCCGGCAAGACGAGTCACAGAA

ATGGCGCCACGGGGATGACTGATGGCTTGGAACTGACAGTGTGAAGATAAGGAGAGTGGAACCAGGAGCT

CAGAGTCCTGACAGCAGCTTCAGAGGAAGCTCGTTTAATTTTATTGTGCTCATCTCAGGAACAAAAGCAT

TTTTTAGTTAAATAATTTAACATCAAAACAACATGCAACCAAAAACTTCTGACATTTATAGTTGATACTT

GCCTATAGAAATGTTTGGTGGCTGGTGTCAAAGGTCCTTAAAGCATTTGCTGCCAAGTTAGTGGAAGGCT

CACTTTTGTTAAGATGACTGTAATTCTCCTTGTTACCGACGAGAGATCATTGGAAGCTGCCTTCTAACAC

TTTGTGTAGCTCTGTGGAGTTGGATTTTCTTAAGGTTTAAAAAGAATCACAGCTTCGGAACTTTTAACTG

AAAATGAGAGACAGAAGCCACAGGGGAAGCAAAGCAAATAGGATTTTCAATATAAATATCAGTGTGGAAA

AATAACCTATTCTGTTGAATTTAGTGTTCATGCACTTGAGAACAACATTATTTCCATTTACTCCGAAAAT

CCTTCTGTGGGGGTTTGAGAAAGTGAATGTTGCAGACATGTTCTGTTGTGTTGCACTTTATCCTGTGTTT

ATGTGTATGTGTTTTTAGATTAATTCAAGTTGTGTGCTATATTTCTTGTATAATTTACAAAGTTACACAA

AATATAAAGAGCAGTAAACTTGTCTGAAAGTTTTTGGCAAAGGAAGGTAACTTCAATGTAATAGCTTCCT

TTAAGAGTACAGGAAAATGCATTCTGTAATGAAGTGGGGCCCATGTAATTGTTTATATTTTCAGTTTTAA

GCAGGTATAGTGCAGGCTTGTTAGGAATGTGTGGAAGGGAAGATTGGAAGTGATTTTTCCTCTTTTAAAA

GTAAACAAAATTCTTCAAATATGCCCTAGTTAACTATTTCAGCATACCATTTTTACTTGGTTAACAGTGT

ACATTTTGATAACCTATCAGGAATGAATAAAGTATTTTTATTTAAAGGTGATATTGTTTTATGCTTCCAT

GAATATTTTGCTTTTCTTAGAGCATATTCATAGATGCAGTACTGTGATTTACCACAAGATAATTGGGAAC

ACTTTCTCAAAATCGATTTTGGTTAATATCAGGTAAATCATTACCTAGGAGTAAGAGGGAAATTGTTGTC

AATTCCTGACTTTAGATTGATCCAGCAGAAAGAAAAGGTGGAACTTTGTATATATCCACTGTTGAAAGTT

GCATGACCTGACTTCCTGTTTTCTAAAGTTGGGGGAATATTATTGAACTTCAATTTCAGAACTGCATTTC

AATTTGGGGCTTGGAAAATGTAGTTAAATCAATTTCGTTCTCTAAATTTTCTAAGCAGGAAAGCAATGTA

AACCAAAAATCAAAATGTTTAGAATTTATAAAAATGGGGTTATATAGCTTTTAATTTTAGTTTCCTTTTT

ACTGCCAAAAAACTTAAAAAGACAACAGCGTAACCACCTGTTTAAGCACCGTGGGACCATCTCTTATTCG

GGTAGAGCCTGGTCCTGGCGACACACTAACAGGAAGGCGTGCTGTGTTACCGGGAAGGCGCCTCTGGCTG

TGGGGTGCGTTCTGACCACCCCGCTCAGATGCCAGCATTTTCCATTTGCCCTTAAGGAAACTTGCTATGT

GTGTTAACCCAGTTATGATTAGTCAAACTGGAAAACACTTGATCAAACTTAAAATTGTAAAATCTGTCTA

ACTTTTAAATATACTTTCCACAGTTGTACTTAAGTTCAATTAACTACAGTATAGAGAAAAATAATTATTC

TCTTCTTTGCACCCGGAGTTGCAAACAAATTAAGTTTACAAATCTCATTTAAACATATATCTTTGTAATG

TAATCTCCGCGGTTCTTTCTTTGTTCTACCCTCCTGCACCTGTTGTCGATGTAATCATTTTGGGAACAGA

AACGTTGTGTCTCAAAAAGATTCGTTGTCAGTTCAGCCAAGATGTTCTTTACCTGAGATTCTGGAAAGAT

GTTTATCTATATCGGAACACTTCTACATTATTAAATGTCCTTAAGCTTTTTCTTTTTTACAAGTGTCTTT

TCAGACTTTGGTGTTGTGATGACACAGTAGAAAGAGCGTGGCTAGAATCCGTGGTTTGCCACATCTGCAG

CTTACCAAACGGATGACTTGTCCAGGACATCTGATCCCTGTAAGCCTCAGCATCCCTCCTGGTAAATGGA

GGGTTTTTTACGACAAAGCCCAAAGGGAGGTTTTGGATGATTAGGCTGTGTGTGCGTGTGAGAATGATCA

CATGTGGCGTCATGCTTTGTACAGAGCCTCAGACCACTGGGCCTCGTCCAGTGAGAGTCCTCTCTGGCGA

CATCACACGCGGAGCAGCCAGGGGCCACCTTAGATCTCAGATCTCTCAGAGCAATACTTTTCTGAACTGC

CACTGTGCCTGGGTGGTTGGGTTGGTGTCATGCTTCTGACTAGAGTAGATCGCGCATGTCCACCAGTGAT

ACGTTGAGTCCTTACAGTTCCCCCCATGGAGTCCCATAAGCAGCTCCATCGAGATCTGTCAGCAAGTTGC

AGGACCCCACAATGTTCTGACATGTTAAGACCCCCTTACATGACGAGTAGAGAGGCAGCTGAGGCCACAA

CCGTGTCTTCCTCTTGAATGGAGCTAACTCGGAACCCCCCGTTTTCTCTTCCTTTCTGCCCACCACTGAA

CATTGCCTTTTAGATAACTCAGTGTTTCTTCTAGATGTCATAGCAATAGACTTTCACTTTCATGAAGTTT

GGGTACGATTTGGATTCTCGCTTAAGTACATATATTTATCAATATTTTTATAAGGCAAAGTTCACTTAAA

AAATCTTTCCAAGTAGCAGTGTGCCTAAGATGGCAAAATACTAAAAACTGGTGTTTCCTGCTCCTGTTGT

GTGTCACTTTTCAAGCCGATTGAAATATTTCTGGCTTTTAGGGCATTACTTTTTAACTATCTCCTTTAAA

AACGATGTTCTGTAGGTTTAGTGTCTTTGTTCATTTCCAAAAGAGTCCAGACAACTGTGTCTGCCCCTGC

AGAGGCTGTTTGTCCAAAGGCAGCATGCCGCTTCCACCGGAATGCAGACAGCAGGGGAGCGGAATTCTAA

AGCAGCGACTTAAAATGAGGAATCCCCAATTGCACTAAATGGTTTCAGGATTGACTAATCATTGTCTTAA

CATTAACTCAGATTTTCAATGTGTAAAGAGCTGTGTGACTTGGCGTCTGAGAGATCCCTCTGCTTTGCTT

TGCTTCAGAGTCCTCGCACCCGCATCCTCAGAACTGTGGGGCATGGTGGGCTCTAACGAGCACTCCCCTT

CTGTTTTCCTTCATTACTTTTGACCTCCTTAAGACTTCAGAGAGAATGTCCGTCAAGTTCTTTTCTCCAT

CAAGTTCTTTAAGTTCCTTGAAAGGAAGGGACTGTGCAAACACAAAGCAATATTCTTTTGTATCTGCAAA

TGCATCAGCTGGACATACCAATTGGTATCAAATAGAATAAAATCAAATATAAATGTTTGAGTCTTAGGTT

AAAAAGGAAGGTTATTTGTATAGTTTATAGATAATGAAGGAAAAATTTCTTTTTCATTGCAGGAAATCTT

GTTTACTGGAAGATAGAGTCACTCTTTTCATATAAGACAAATAGTGCTTTAATGCCAACTTCTTTTTATC

TCAACATTTCAGGATCATGCTAGGCACACTGCCCCCTTGAATAGACATTATATGCACAGTTGCAAGTCAG

CCAATGTTTTTATTCAGAAGTATTTCCCCCCATTATAGTGCCTGCATATCAGAGATACAAAAAGCATCCA

ACACACTACCGTAATAGGCTTCTTTGGGGATGAGAAATTTGAGTCTCAACAACTCAGAGTTTGAGATGTC

AGCTTTTTTGGTAAACGTAGGTGTTAGAGGTATATTTTGCTTTCCTACAACAATTGTTGGCCCTTGATTT

CAAGCATGTTGCTTCATAGGAAGCACCAGAGTGCCATCTGCTGCATTTCAAGAGATTGTAAATGTCATCT

CAGCTGGCTCAGTTATATCTCTAATGTCCCGGGTAGCAGCACCTCCCTCTAAAAATATGTTTACTTCACT

GTTTCACTTGTATTTTGTGTATACGAAATGGCAGCTTCCGATTTCTAGTTGGATTTGTCTTGCATTGTTT

GTATAACTTGCTGGTCACCCAGGGCTATTTGCTTTTTCATTGAGAAATTTGGTAGGGGTGTCTAGTTCAG

CTTTTATGTTGATCCATCCTGACTTATTTTAGACATTGAATTTATCTCACCACAAGTAAAAGAACATGTG

TATTGACTGTCTTTGCTAAGTTTCCTAATTTTTCCTAATTATGGCAATTATGGATGTGAATAAGAATACT

GATGCTGTACAAATATTTTTGTGGAAATGTACCTTGTTAATGTGACTATTTAAATAATATGAAAATAAGA

ATACTCTTGAAGAAAAAATTAAAATATTTACTCTTTGGATTTTGCTTTAAACACTTGTCTTTCACCAAGA

AGTGTTCCCAAAACTTGTGTAAAATACTTATTGGGGTGGGGTGTACATCTGCCTCAGACCTTGTTATTGG

GAAATGATGTTCCAGTGCCCCTGATGCTTCTGATAGCAGATCTTGGGTCTGTCTGCTACTGATTGCTGAG

CTGTATGGAGTTTTAACAGTTCTTCCCTCCCTGCTTTGGATTTTAGTCCCTCTATTAGTTATCCTATCCC

TGCAATAAGGACTTGCAGCAAACACACCTCCTAGTTTGATATCACGAAACTGATAACTATTTGGAGGAGT

ATTTTTACTTATGAAGGGAATATTTAAAAGACATACTTATTTTTATTTTATTTATTTATTTTTTCAGACG

GAGCTTTGCTCTTGTTGCCCAGGCTGGAGTACAATGGAGCAATCTTAGCTCACTGCAGCCTCCACCTCCT

GGGTTCAAGCGATTCTCTTGCCTCAGCCTCCTGAGTAGTTGGGATTACAGGCACCTGCCACCACGCCCCA

CAAATTTTTTGTTTTTTTAGTAGAGACGAGGTTTCACCGTGTTGGCCAGGTTAACCTCAGCCTGGTCTCG

AACTCTTGACCTCAGCCTCCAAAAATGCTGGGATTACAGGCATGAGCCACCGTGCCTGGCCAGACATACT

TATTTTTAAGGGCAATTTACTTTAAAAGATTTTGTTGTGACAGATGTGACTATATAACATCCACTTTGAA

CAATTAGGGAAAAAATGACAAGTCACACTTCACCCATGGCTCTTGTTGAAAAAAGGGATGGGGACAATTG

TGAGATGCTTACTGTGTGCCAAGTATCCTACTGTCTTTTTATAATTTCACAGCCCCCACTCAGAGGCAGT

TACTAGAGTCCCTGTTTTCCAGATGAGGAGACAGGTTCAGAGAGGTCAAGAAACTTGCTCACCGTCACAT

GGTTTGACTTCCAGACCTCATGCTCTTCCCAGGACTTCATTTAAGAAAAGCTTGGAGCAGTTACCTAACT

TAGCAAAAAGTCTTCACTAATTGGGAAACCACAGAATTTTGCTAGAAGATTGAGATTAAAGGCTCTTAGA

ATATTTGAGCAAGAAAATGTGCTTGATCTCTTCCTGGCGTCTCCAGCCCCTCTCCCCTCTTCTGGCTGTG

CTTAGCCACCCCAGCGGTTCAGTTCTGCATGCCCGTGAGTAGGGGTTAGCACCACCTGGAAGATTTCTTT

CTATAAATGCTGCACCACAGGCCACAGTCAACACTGATTAAAGCAAAATCTCAGAATGGGGCTAAAGTCG

GGTTTCTCCACCTCAGCAGTTGACATTTGGGACCAGCCTGTGCATTGTAGGATGCAGGGGATGTTGCCAC

CCTCCCACCCCCCGCTGGCCTCTGGCCACTGGATGCCAGTAACAAACTCCCCTCCCCCAGTTGTGACAGC

CAAAGACGTCTCCAGACATGGCCAAATATGCCAAATATGACAAAAATCCCCTGTTAAAAATCACTGGGCT

AAGAGCTCTCCTGGGGAAAATCATGCACAGCCTGGGGCAAGAACGCGCTATGGCCGCCCCAACTCGGATG

CTCTGTCGGGATCTCTGGCTGCTCCTGCTGTCTCAGCCCGGAGGCTCCACTTCAGGCAGCCTGGCCACCC

TCCCTTCCTCTCTGTTTCCCACTATAGCATGCTTTCCTTTTTTTTGTTTGTTAAACTTTGCGGCACTCCC

CGCATACTGTAGATCCACGTCTGTTTCCCTGTTTGACTTCCTTCAGGAGGGCAAGGGACTTAGGGACCCG

GTGCCTAGCACAGTGCCTTGCCCAGAGTCAGGGATCAGCAAATGGTGAATGAGTGAATGAATGAATGAAT

GAGTGAGTGAACCCGAGTGGTCATCTAGTCCTGTCTGCAGGTGAGGAAATCGAAGCCCGGAGAGGGGAAA

TTCTTTGCCCAAGATCACATGATTACTACTTACAGATGTCCTTTATTCAATGTAAGCTCACGTTTTCTTG

ATTTTAGCAATACTTGTAATAATTTTTAAAATATAAATATATATGAAATAAAAGTTCATCATTGATTGAC

TA

>NM_022488.5 Homo sapiens autophagy related 3 (ATG3), transcript variant 1, mRNA

AGTGTCACGTGAGGCCCCGGTGGCGGCGCAGCTACGGCAAGAGAGTGAGAAGGAAGGGAAGCCGGAAGGG

GCGCGAGTGAAGCAAAGCGAGGACAGACAGCTCGCAGAGGGCGAGGGGTGCGTGTGCGTCCGCTTCTCAC

CTCAGGTCTCCCTTCGGCCCCGCTGCCCTCCCTCGCGGCTGGGTGACAGCTGGGTCCGGTCCGTCGCGGG

CTGCCTGGGGTGCGAGGATCGCGCACCCCGTCTTCGCGCGCTGTGCCTGCCGCCCCGCCCCCTCGTCCCG

CCCGTCCCGTCGCGTCGCGTCCCGTCCCCTCGGGTGCTGCCAGCCGGGTGCTGATGCGAGTCGGTGGCAG

CGAGGACATTTTCTGACTCCCTGGCCCCTGACACGGCTGCACTTTCCATCCCGTCGCGGGGCCGGCCGCT

ACTCCGGCCCCAGGATGCAGAATGTGATTAATACTGTGAAGGGAAAGGCACTGGAAGTGGCTGAGTACCT

GACCCCGGTCCTCAAGGAATCAAAGTTTAAGGAAACAGGTGTAATTACCCCAGAAGAGTTTGTGGCAGCT

GGAGATCACCTAGTCCACCACTGTCCAACATGGCAATGGGCTACAGGGGAAGAATTGAAAGTGAAGGCAT

ACCTACCAACAGGCAAACAATTTTTGGTAACCAAAAATGTGCCGTGCTATAAGCGGTGCAAACAGATGGA

ATATTCAGATGAATTGGAAGCTATCATTGAAGAAGATGATGGTGATGGCGGATGGGTAGATACATATCAC

AACACAGGTATTACAGGAATAACGGAAGCCGTTAAAGAGATCACACTGGAAAATAAGGACAATATAAGGC

TTCAAGATTGCTCAGCACTATGTGAAGAGGAAGAAGATGAAGATGAAGGAGAAGCTGCAGATATGGAAGA

ATATGAAGAGAGTGGATTGTTGGAAACAGATGAGGCTACCCTAGATACAAGGAAAATAGTAGAAGCTTGT

AAAGCCAAAACTGATGCTGGCGGTGAAGATGCTATTTTGCAAACCAGAACTTATGACCTTTACATCACTT

ATGATAAATATTACCAGACTCCACGATTATGGTTGTTTGGCTATGATGAGCAACGGCAGCCTTTAACAGT

TGAGCACATGTATGAAGACATCAGTCAGGATCATGTGAAGAAAACAGTGACCATTGAAAATCACCCTCAT

CTGCCACCACCTCCCATGTGTTCAGTTCACCCATGCAGGCATGCTGAGGTGATGAAGAAAATCATTGAGA

CTGTTGCAGAAGGAGGGGGAGAACTTGGAGTTCATATGTATCTTCTTATTTTCTTGAAATTTGTACAAGC

TGTCATTCCAACAATAGAATATGACTACACAAGACACTTCACAATGTAATGAAGAGAGCATAAAATCTAT

CCTAATTATTGGTTCTGATTTTTAAAGAATTAACCCATAGATGTGACCATTGACCATATTCATCAATATA

TACAGTTTCTCTAATAAGGGACTTATATGTTTATGCATTAAATAAAAATATGTTCCACTACCAGCCTTAC

TTGTTTAATAAAAATCAGTGCAAAGAGA

>NM_052936.5 Homo sapiens autophagy related 4A cysteine peptidase (ATG4A), transcript variant 1, mRNA

GTCCGTAGTCAAGTTGCCGGTGGAATTGGCCCAGGATGACAGCTGGAGAATGGAGTCAGTTTTATCCAAG

TATGAAGATCAGATTACTATTTTCACTGACTACCTAGAAGAATATCCAGATACAGATGAGCTGGTATGGA

TCTTAGGGAAGCAGCATCTCCTTAAAACAGAAAAATCTAAGCTGTTGTCTGATATAAGTGCTCGTCTATG

GTTTACATACAGAAGGAAATTTTCACCAATTGGTGGAACGGGCCCTTCATCAGATGCTGGTTGGGGATGT

ATGCTACGCTGTGGACAGATGATGCTGGCTCAAGCCCTTATCTGTAGACACTTGGGAAGGGACTGGAGCT

GGGAGAAACAAAAAGAACAACCCAAAGAATACCAACGCATCCTACAGTGCTTCTTAGATAGAAAAGATTG

TTGCTACTCTATCCATCAAATGGCACAAATGGGTGTAGGAGAAGGGAAATCAATTGGAGAATGGTTTGGA

CCAAATACAGTTGCACAGGTGTTAAAAAAACTTGCTTTATTTGACGAATGGAATTCCTTGGCTGTTTATG

TTTCAATGGATAACACAGTGGTCATTGAAGATATCAAAAAAATGTGCCGTGTCCTTCCCTTGAGTGCTGA

CACAGCTGGTGACAGGCCTCCCGATTCTTTAACTGCTTCAAACCAGAGTAAGGGCACCTCTGCCTACTGC

TCAGCCTGGAAACCCCTGCTGCTCATTGTGCCCCTTCGCCTGGGCATAAACCAAATCAATCCTGTCTATG

TTGATGCATTCAAAGAGTGTTTTAAGATGCCACAGTCTTTAGGGGCATTAGGAGGAAAACCAAATAACGC

GTATTATTTCATAGGATTCTTAGGTGACGAGCTCATCTTCTTGGACCCTCATACAACCCAGACCTTTGTT

GACACTGAAGAGAATGGAACGGTTAATGACCAGACTTTCCATTGCCTGCAGTCCCCACAGCGAATGAACA

TCCTAAACCTGGATCCTTCAGTTGCATTGGGATTTTTCTGCAAAGAAGAAAAAGACTTTGATAACTGGTG

TAGCCTTGTTCAGAAGGAAATTCTAAAGGAGAATTTAAGGATGTTTGAATTAGTTCAGAAACATCCATCA

CACTGGCCTCCCTTTGTACCTCCAGCCAAGCCAGAAGTGACAACCACTGGGGCAGAATTCATTGACTCTA

CTGAGCAACTGGAGGAGTTTGATCTGGAGGAAGATTTTGAGATTCTGAGTGTGTAGAATCCTGGGAACTC

AACTTGAAGGTCTGTCTTCCATCTGGCACCATAAAAACATGAACTTATTGCATAAAACTTTTCTAGTCAG

CAAGTGCCTGATATGCCAATAGCATACAAACTCAATAGCAATCATGACTGAGCCAATCACTGTTTCTCAG

AAAAACAAAACAAAACAAAACAAATGACAGTAACCCTTCCCCGGAAAGAAATAGAACAATCATGGAGCCT

AGGAGCAGAGAGATGAGGAGGAGTTCATTGCTTCCCAGCTTGTGTTATATGGCTACAGCAAGTCTTCAGC

TGCTGCAATGAGGAAATGGGCATCTGGAAGACAAACAGCAACTCTCAGCTTGCTTCAAGAACCAGCAGAT

AAGAGATGGTTAAGCTGTTCTTCACCCTTTCAGATGTGACCTCTTTTGGACTAAGCAGCAATCTGTTCTC

TTGCTCAAATAATAAAGTGACTGAATCAGGGAGGAAAAGGTTCTTGTTAAATTATTTGATTGTGTAGTTG

AAGTAATTATAATTTATATCAAAACGTTTGTCAAAGAAACGATGTCAAATATACACTTCTTGATCTCCCT

TCTGTTTGCGGGGATCTTACTATTTGATGGGTCACTGTCCCCATTCTTACTGATACTTTTGTCAGATATC

ACCCTGTCCTTAAATCATGATCACTTAAATCAGGGGTCAGCAAACTTTTTCTGTAAAGGGCCAGACGGGA

AATATTTTGGGCTTTGCAGGCCATGCGGCCTCTGTCACATCTACTCAACTCTGCTGTTGACATGCAAAAG

CAGCAATAGACAATATGCGTGTAAATGAGTGTGGCTGTAATCCAAGAAAACTTTATTTACAAAAGCAGGT

GGAGGGCTGGGTTTGGCCTGCAGGCTGTAGCTTGCCAATCAGTGACTTAAATTGTTGATTTTTGTTTGAT

AAATTAAAAATAAATTGTGTTTGAAGTATACCCTA

>NM_013325.5 Homo sapiens autophagy related 4B cysteine peptidase (ATG4B), transcript variant 1, mRNA

AGTCGGCGGCCGGACTGGGAAGATGGACGCAGCTACTCTGACCTACGACACTCTCCGGTTTGCTGAGTTT

GAAGATTTTCCTGAGACCTCAGAGCCCGTTTGGATACTGGGTAGAAAATACAGCATTTTCACAGAAAAGG

ACGAGATCTTGTCTGATGTGGCATCTAGACTTTGGTTTACATACAGGAAAAACTTTCCAGCCATTGGGGG

GACAGGCCCCACCTCGGACACAGGCTGGGGCTGCATGCTGCGGTGTGGACAGATGATCTTTGCCCAAGCC

CTGGTGTGCCGGCACCTAGGCCGAGATTGGAGGTGGACACAAAGGAAGAGGCAGCCAGACAGCTACTTCA

GCGTCCTCAACGCATTCATCGACAGGAAGGACAGTTACTACTCCATTCACCAGATAGCGCAAATGGGAGT

TGGCGAAGGCAAGTCCATAGGCCAGTGGTACGGGCCCAACACTGTCGCCCAGGTCCTGAAGAAGCTTGCT

GTCTTCGATACGTGGAGCTCCTTGGCGGTCCACATTGCAATGGACAACACTGTTGTGATGGAGGAAATCA

GAAGGTTGTGCAGGACCAGCGTTCCCTGTGCAGGCGCCACTGCGTTTCCTGCAGATTCCGACCGGCACTG

CAACGGATTCCCTGCCGGAGCTGAGGTCACCAACAGGCCGTCGCCATGGAGACCCCTGGTACTTCTCATT

CCCCTGCGCCTGGGGCTCACGGACATCAACGAGGCCTACGTGGAGACGCTGAAGCACTGCTTCATGATGC

CCCAGTCCCTGGGCGTCATCGGAGGGAAGCCCAACAGCGCCCACTACTTCATCGGCTACGTTGGTGAGGA

GCTCATCTACCTGGACCCCCACACCACGCAGCCAGCCGTGGAGCCCACTGATGGCTGCTTCATCCCGGAC

GAGAGCTTCCACTGCCAGCACCCGCCGTGCCGCATGAGCATCGCGGAGCTTGACCCGTCCATCGCTGTGG

GGTTTTTCTGTAAGACTGAAGATGACTTCAATGATTGGTGCCAGCAAGTCAAAAAGCTGTCTCTGCTTGG

AGGTGCCCTGCCCATGTTTGAGCTGGTGGAGCTGCAGCCTTCACATCTGGCCTGCCCCGACGTCCTGAAC

CTGTCCCTAGATTCTTCTGATGTAGAGCGACTGGAAAGATTCTTCGACTCAGAAGATGAAGACTTTGAAA

TCCTGTCCCTTTGAAAATCCTGGGGTCGGGGGTGGCACCTGTGAGAGCCTGGGGCTCCTGGTGCCGCTGC

GTTTCATCCATCCCGCCCGCTCGCCTGCCGAGGGCTGCGCCCCGTGCTGCCTCCCCCCAGAGGGCCACCC

GCTGTGCTCGTGGACTGAGGCTGCGCTGCCCGGGAGGCCTTACTGCTTGGTGTCAGACTGCCCAGCTCAG

AGTGCCCGTCAGGGCCTGTGCATCCGCACGCGGAGCCGTCTGTTAGGAGCTTCCAGAGTGTTCTCTCGAC

ACTGCCAGCCCCGTGTTAGCACCTGGGCCTCAGTCCCACTTGCTCCCAGGCGCCGGTTCTGTGGTTGGTT

TGGAATTAAAGTCCTGTTTGAAGTTGTCAGACACAGACATGAATTTCTGGGCGCTCCCTGAGTCAGAGTC

TCAGAAGACCTGTGCAGGCTGGCGTGAGAGGAGCGGCAGCCACACTGCGGCCCCACGCCCAAGGACTGGG

CTGCTCTCGAGGGGGGCGCGCCCACCGCTGTGTCCTCTCTGCCCAGCCTGGCTTACCAAGGGCTACCTCA

GTGGGAGATGAGGTTGGAGGAACGAAGGCGAGGTTCCTCCTTGCTTTGGGGAGAAAAGTATTCAGGAAGT

GGGTGTGTGGGAAACCTGAAGATGGCGTGCACAGGACACAGCGTGGGCGGCCTGGGCAGAAGGGCGGCTG

GCTGTCCTGGAGCTGCTGCTGGAGCCTGCCCTCAGAGTGTCCCTTTCCAGTGCTGTGGCATTCTGTGGCA

GCTTCCCCAGGTGTGGTGACGGGGGGGGGGCGGGGCCTCCACCTGTGACAGCCAGGCTTGAGGGTGGACG

GCGTGCCTCTCCCAGGAGCCTTCCCCATGTCCTTGCCTTGCTGAGAATTGCCCTCCCATGCCGCTGAGGT

GTTAGGTGGTTTAGGGCCAAAAGGGGAAAACCACTTGAGTCTTGTGGTGTGTGGTGGGCAGACACCACAG

GGTGGCATCACCTGGTGGCATTTCCAGAACCTCAGCCCCGATTCCAGCACCCACCACCGCCTGACCCTGT

GTAACCTGCTGTCCCGGGTCCCAGAGTGCACTCTGCCCCGCTGCTCTGCTGCCTGTCCTGGGAAAGTATC

TTTGCCCCACTAGGAAATGTAAACAGGAGGGCTTGGGGAGCGTGGGCACTTTTCTCATGAGCAGCTACTG

CGGCGTTGGCAGGACTCGCTGCTGCTGCTGCTGCTTGTGTAGGTCGGGGAGCCAGAGATCCCCGAGGACG

CGCGCCGGACAGTCGGCACTGACCGGCCCACCTGGTAGCAGAGGACACCCCCAGCCCCCCAAGCATTGAA

GACATAGTGTATTTCCTCGTATCCTTTCTCCCTTGGGTGTAGTTGGGGTGGGGAAGCAGGGAAGGCTGGT

GCGATCTCCATTCCTTGGGCTCCACGTCCGAGTTCATGGTGCGCCGCTGTGCTGGGAGCTGCAGTGGTAA

TGTGTGGGACACCTTGACCAAAGGGGAGCTTTGTCTCGTGTGTTTTGAAAAAGGCTTAATGAAGAGAATG

TTGTTCATTCTTAGTAGTATAGTTTGCAATTCTTAATGGCAAATAATAAGTTTCAGTAGAAAACAAA

>NM_032852.4 Homo sapiens autophagy related 4C cysteine peptidase (ATG4C), transcript variant 1, mRNA

AAAGTACCTGTAGCTGCGGCGCTGAGGTCGGAACGTCTGCGTGTGTGCGGGCTGGTTTTGTGGCGGCTGC

TGCTAGAGCTGGAGCATTTGCCGGGTTGGTGGCTCCTGCACATTTTTACAGTTCTCCAGTCCTTCTCTTT

CGTCAGTATAAAAGATTAAACTCTACAGAAGAATGCAATCAAGTGATGGCTTTTCCTTTAGAATTTGAAT

ATGGAGGCTACAGGAACAGATGAAGTTGACAAGCTAAAAACCAAATTTATATCTGCTTGGAACAACATGA

AATATAGTTGGGTGTTGAAAACAAAGACGTATTTTAGTAGAAATTCTCCTGTATTATTGCTTGGAAAATG

TTACCATTTTAAATATGAAGATGAAGATAAAACGTTACCTGCAGAGTCGGGATGTACAATAGAGGATCAC

GTAATTGCAGGAAATGTAGAAGAATTTCGTAAAGATTTCATTTCTAGAATATGGCTGACCTACAGGGAAG

AATTCCCTCAAATAGAAGGCTCAGCTTTGACAACAGACTGTGGGTGGGGCTGCACATTGAGAACTGGCCA

GATGCTCTTGGCTCAAGGACTCATACTACACTTTCTTGGTAGAGCTTGGACCTGGCCTGATGCTTTGAAT

ATTGAAAATTCAGACTCTGAATCATGGACTTCCCACACTGTCAAAAAATTTACTGCATCATTTGAAGCAT

CACTTTCAGGGGAAAGAGAATTCAAAACCCCAACAATTTCTCTGAAGGAAACAATTGGGAAATATTCTGA

TGATCATGAAATGCGAAATGAAGTTTATCATAGGAAAATCATCTCTTGGTTTGGTGATTCCCCCTTGGCT

CTTTTTGGCTTACATCAACTAATAGAATATGGAAAGAAGTCTGGGAAAAAAGCAGGAGATTGGTATGGAC

CAGCTGTGGTTGCTCACATTTTAAGAAAAGCAGTTGAAGAAGCAAGGCATCCTGATTTACAAGGAATAAC

TATTTATGTTGCACAAGATTGTACAGTTTACAATTCTGATGTAATTGATAAACAGAGTGCTTCCATGACT

TCTGATAATGCAGATGACAAAGCTGTTATTATTCTAGTTCCTGTTAGACTTGGTGGAGAAAGAACCAACA

CCGACTACTTAGAATTTGTGAAGGGTATTTTAAGCCTGGAATATTGTGTGGGTATTATTGGTGGCAAACC

TAAACAGTCATATTACTTTGCTGGATTTCAAGATGACAGTTTGATTTACATGGATCCTCATTACTGCCAA

TCTTTTGTAGATGTCAGCATAAAGGATTTCCCTCTTGAGACATTCCACTGCCCTTCTCCCAAAAAGATGT

CTTTTCGAAAAATGGATCCCAGCTGTACAATAGGATTTTACTGTCGAAATGTTCAGGACTTCAAACGAGC

TTCTGAAGAAATCACCAAGATGCTGAAATTTTCTTCTAAGGAGAAATATCCCTTATTTACTTTTGTAAAT

GGTCATTCCAGAGACTATGATTTTACATCTACTACAACCAATGAAGAAGACCTTTTTTCAGAGGATGAAA

AGAAACAATTAAAAAGATTTAGCACGGAAGAGTTTGTCTTGCTTTAAAGATTAGCACATTTGTGCTTGAT

AAGAAGAATTCCATTGAAAGGGGAAAAATGAAGAGAAACAAGTATATCTGAAATGTTTATTTTCACAAAT

ATCTTAATTTTATATGTTCTTTAAAAAAGAACATTTGAAAATATAACAGTTAAAGATATTTTTCTAAAAG

AGAAATGATTTAATGAATCTTGCTTTCTAATAAATAAATTGAGTGATTCTGGTTGCATTCCTATTTCCCT

AAGATCTACTAGTGATAATTCTACCTTAACTGTAAGCCTTTTAGTCTTCAAAGTCTTCCACCTGAGCCCA

TTGTTCTCATGGAGGTTTTGTGATATTAACCCTCCCCCAAAGACTGGGATCACCAAATAGTTTCAAAATT

CTCAGTTTGTACTGAAGACCAGAAGATCAGAGAAGGAAACTTTAATGCTGTCTAGCCTCCTGCTATTAAT

GCAATCAAAGAATACTTTTGCATATGTCTTGATAATTAAATAGTATTTGTTAACTGTGATATGCATACAC

TTATATAAGCAGAATTATGAGTTAAAGTAATACTTAGCAATATGATTTTATAATGGCTCCTCATTATGCT

TGCTGTTGAACCTTTTATGAGGAGTGAATATAAAGTATTGGTTTTCCCTCACAAATTTAAAGATTATGTT

ATTAATACTATTATAACTGCATCAATCAAGTCAGATAAAGGCAACTATAAAATAGTAGTAGTGTTTGTTT

CCTATCTCAAGGGCGAAATTTTATGGGAACTCAATTTATTATGCAGTTTTTAAGTTTAAAATACCAAGAA

AGATGTCACTAGATTCTCTTCTATGTGATTTTTGTTTTTTATATAAAGCAGTGTAGTGGTGTTTAGAAGC

TGAGGCCACCTGTAAGGCAAATCTGCCTTAAGTGTATTATGTGTTACTTAAAGGCAAATTTGTGATCTAA

AAGTACAAGAGTGATTTTTGAGCTAGGATTATAAAATACATAATAAAGATGTGAGAAGATAAAATGCTTT

TGTTTTGGTTTTAATGTTGGGATTATTTTAATCCTTTCATTTGAAAAATCAGTGTCTCAAATGAATTCTG

TTCATTTATAATAAATGCATATATTGCTCTGAAAACAATATTAATTTTTTTAAGTGGAGCTTTCATGTTT

CAGTGTAGAAACGTGACTTATAAAGACATAAAATTGTGTATCATCTAAAGTCATTGATCTCTTCCATCAG

AAAACCTGTTTGTTTAGAGTTGCATGTAAAGCTGAAAGAGAGGGTGGGCGGATTGTTTTAGTATACCTGA

TGAGAAAAATCAGGTTTGACATTTCTCAGTGAAGATTACTTACTGAGTTGAAATAAATAGTCTGAAATCT

AAAA

>NM_032885.6 Homo sapiens autophagy related 4D cysteine peptidase (ATG4D), transcript variant 1, mRNA

GTCCTGGCCCGCTAAGATGGCGATGGCTGCGGTAGCAGCGGCGGCGGCTGTTGCCTGGCCCGGTACCCTG

GGGACGGGGGCCGAGTAGCGCCTTCCCCGGGCCCCGTGAACCGGCTGCGGGTCGCCCTTGGGGGGCAGCG

GCCGCAGCCCCCCACCTGGGCCCTCGGTCCGCCCTCCCGGCGCGTCCATGAACTCAGTGTCGCCGGCCGC

CGCGCAGTACCGGAGCAGCAGCCCGGAGGACGCGCGCCGCCGGCCCGAGGCCCGCAGGCCGCGGGGTCCC

AGAGGCCCAGACCCCAACGGCCTGGGGCCTTCCGGAGCCAGCGGCCCCGCTCTTGGCTCTCCCGGGGCTG

GCCCGAGTGAGCCGGACGAAGTGGACAAGTTCAAGGCCAAGTTCCTGACAGCCTGGAACAACGTCAAGTA

CGGTTGGGTGGTTAAAAGCCGGACCAGCTTTAGCAAGATCTCCAGCATCCACCTCTGTGGCCGCCGCTAC

CGTTTCGAGGGCGAGGGTGACATACAGCGTTTCCAGCGGGACTTTGTGTCCCGCCTGTGGCTCACATACC

GCCGGGACTTCCCGCCCCTTCCTGGGGGCTGCCTGACCTCGGACTGTGGCTGGGGGTGCATGTTACGCAG

CGGCCAGATGATGCTGGCACAGGGCCTTCTGCTGCATTTCCTGCCCAGAGACTGGACATGGGCCGAGGGC

ATGGGCCTGGGCCCCCCTGAGCTGTCAGGGTCAGCCTCTCCCAGCCGGTACCATGGGCCTGCCCGCTGGA

TGCCCCCACGCTGGGCCCAGGGTGCCCCTGAGCTGGAGCAGGAACGCCGGCACCGGCAGATTGTGTCCTG

GTTCGCCGACCACCCCCGGGCCCCCTTTGGCCTACACCGGCTGGTGGAGCTTGGGCAGAGCTCAGGCAAG

AAGGCAGGTGACTGGTATGGGCCATCGCTAGTGGCACACATCCTCAGGAAAGCCGTGGAGAGCTGCTCCG

ACGTCACCCGCCTGGTGGTGTACGTTTCTCAGGACTGCACAGTGTACAAGGCGGATGTGGCACGCCTGGT

GGCCAGGCCAGACCCCACAGCCGAGTGGAAGTCTGTGGTCATCCTGGTGCCCGTGCGACTGGGTGGCGAG

ACTCTCAACCCCGTGTATGTGCCCTGCGTGAAGGAACTCCTGCGTTGCGAGCTGTGCCTGGGCATCATGG

GTGGGAAACCGCGACACTCACTGTACTTCATTGGCTACCAAGATGACTTCCTGCTGTACCTGGACCCTCA

CTACTGCCAGCCCACTGTGGATGTCAGCCAGGCCGACTTCCCCCTGGAGTCCTTCCACTGCACCTCGCCC

CGCAAGATGGCCTTTGCCAAGATGGACCCAAGCTGTACCGTGGGCTTCTATGCTGGAGACAGGAAGGAGT

TTGAGACACTCTGCTCAGAGCTGACCAGGGTCCTCAGCTCCTCCTCAGCCACAGAGCGGTACCCCATGTT

CACCCTGGCCGAGGGCCATGCTCAGGACCACAGCCTGGACGACCTCTGCTCCCAGCTCGCCCAGCCCACA

CTCCGGCTCCCTCGCACAGGGCGGCTCCTCAGGGCCAAACGCCCCAGCTCTGAGGACTTTGTGTTTTTAT

AAAGGGAGGGGATGAGGGGAAAGATACAACACTATTTATTTTTTTATTTATGTCATGTCGGGTGTGGGAT

CTTGAGCTCTGGCAGTGATGATGGTACTTCCTGTTGTCAGCCCCTCAAGCCCAGCTGCAACCAGTCTGGG

GCCATTCAGCCAGGGACAGAGCCCACAGAGCCCATACACCTGTCTCCCACCAGCGGGGCCCTCCTGGCAG

GGTAGGGAAGGAGGACCCCGGGCACCCCCCTCAGGGCCTGACTCACGTACTGTAGTTTGCACTGGACGCC

CGGGCCCTCCCTGTCCCAAAGCCCCCTTGGGGGAACTGTGGCTGCTGGGGGCCAATAAAGCTGTGTAACT

TGA

>NM_004849.4 Homo sapiens autophagy related 5 (ATG5), transcript variant 1, mRNA

AGCGTTGCCGGTTGTATTCGCTGGATACCAGAGGGCGGAAGTGCAGCAGGGTTCAGCTCCGACCTCCGCG

CCGGTGCTTTTTGCGGCTGCGCGGGCTTCCTGGAGTCCTGCTACCGCGTCCCCGCAGGACAGTGTGTCAG

GCGGGCAGCTTGCCCCGCCGCCCCACCGGAGCGCGGAATCTGGGCGTCCCCACCAGTGCGGGGAGCCGGA

AGGAGGAGCCATAGCTTGGAGTAGGTTTGGCTTTGGTTGAAATAAGAATTTAGCCTGTATGTACTGCTTT

AACTCCTGGAAGAATGACAGATGACAAAGATGTGCTTCGAGATGTGTGGTTTGGACGAATTCCAACTTGT

TTCACGCTATATCAGGATGAGATAACTGAAAGGGAAGCAGAACCATACTATTTGCTTTTGCCAAGAGTAA

GTTATTTGACGTTGGTAACTGACAAAGTGAAAAAGCACTTTCAGAAGGTTATGAGACAAGAAGACATTAG

TGAGATATGGTTTGAATATGAAGGCACACCACTGAAATGGCATTATCCAATTGGTTTGCTATTTGATCTT

CTTGCATCAAGTTCAGCTCTTCCTTGGAACATCACAGTACATTTTAAGAGTTTTCCAGAAAAAGACCTTC

TGCACTGTCCATCTAAGGATGCAATTGAAGCTCATTTTATGTCATGTATGAAAGAAGCTGATGCTTTAAA

ACATAAAAGTCAAGTAATCAATGAAATGCAGAAAAAAGATCACAAGCAACTCTGGATGGGATTGCAAAAT

GACAGATTTGACCAGTTTTGGGCCATCAATCGGAAACTCATGGAATATCCTGCAGAAGAAAATGGATTTC

GTTATATCCCCTTTAGAATATATCAGACAACGACTGAAAGACCTTTCATTCAGAAGCTGTTTCGTCCTGT

GGCTGCAGATGGACAGTTGCACACACTAGGAGATCTCCTCAAAGAAGTTTGTCCTTCTGCTATTGATCCT

GAAGATGGGGAAAAAAAGAATCAAGTGATGATTCATGGAATTGAGCCAATGTTGGAAACACCTCTGCAGT

GGCTGAGTGAACATCTGAGCTACCCGGATAATTTTCTTCATATTAGTATCATCCCACAGCCAACAGATTG

AAGGATCAACTATTTGCCTGAACAGAATCATCCTTAAATGGGATTTATCAGAGCATGTCACCCTTTTGCT

TCAATCAGGTTTGGTGGAGGCAACCTGACCAGAAACACTTCGCTGCTGCAAGCCAGACAGGAAAAAGATT

CCATGTCAGATAAGGCAACTGGGCTGGTCTTACTTTGCATCACCTCTGCTTTCCTCCACTGCCATCATTA

AACCTCAGCTGTGACATGAAAGACTTACCGGACCACTGAAGGTCTTCTGTAAAATATAATGAAGCTGAAA

CCTTTGGCCTAAGAAGAAAATGGAAGTATGTGCCACTCGATTTGTATTTCTGATTAACAAATAAACAGGG

GTATTTCCTAAGGTGACCATGGTTGAACTTTAGCTCATGAAAGTGGAAACATTGGTTTAATTTTCAAGAG

AATTAAGAAAGTAAAAGAGAAATTCTGTTATCAATAACTTGCAAGTAATTTTTTGTAAAAGATTGAATTA

CAGTAAACCCATCTTTCCTTAACGAAAATTTCCTATGTTTACAGTCTGTCTATTGGTATGCAATCTTGTA

ACTTTGATAATGAACAGTGAGAGATTTTTAAATAAAGCCTCTAAATATGTTTTGTCATTTAATAACATAC

AGTTTTGTCACTTTTCAAGTACTTTCTGACTCACATACAGTAGATCACTTTTTACTCTGTGTTACCATTT

TGACTGGTCGTCATTGGCATGGGGTGGATATAGGGCATAGGATTACTTGTCTCAGAAGCTGTCATAGAAT

TTCTTGCTGCCAATTAAAAAACCTGTGTTCTTTACACACTACACGTATAAATATTGTAACTGTTCATCTT

TGTTGTTTTATCACTGTAAGCCTGTCAAATCATAGTATCCTAAGCATCTGTAAATGCTAATTTTGCATTT

TTGGAAAAACCCATTCCTTCCAAGCTAGTGTTTTTCATTGGCTCCAGGTCTAATTTTTCACTGTGGTCCC

TGGCAGCCAGTCTTTTGAAGTTTAAAGATTACCTGTCTCTTGACTGCAGTACCTTTTCTTTAATTTTTAC

CAAAAATATCCAGAGGTTACTGGAGTTCTTATTCAATATAAGGAAAGTTTGCTGCACTTTATTACCAAGC

CTCTGGGATTTTACCAGTCAAACATATTTGTGCATTACATTTCATTTCTTGTGAGCTAGCTGGCTGTCCA

TATTGAATGTTGACCCATTTGAGTACGCTAAAAGGCTTACAGTATCAGACACGATCATGGTTTTAGATCC

CATAATAAAAATGAATGTTTTTCTTATAAAAAATTATACAAATGCTGAAGTGAGATTCTACTATTGTTCA

TTGCTTCCTTTTCTTTTTCCTTTTGCGATTTTCACTGATTAATAGCACATTTCTTCACAAAATTAGATAA

AGTTGGTCAAAGACCAGATATTCTGGAATGGAAATTGTAAAGCTTAATCAAAAAGAATAGCCAGTACAGC

ATACAATCTCAGAAACTTAGAAGCAAGTAGAAAATAATTGGTTGATGTAAACGAAAGTGCCATTTTAGTA

AAGGCAGGAAAAAAATAGCAATATTTGAGTTATGTAAGGATAAAAAATCCACTGACTTGTATTTTTGCAC

AAGAGGCTGGTCTGAATATGATTGTTCACATTAAGAGTGTTTATTCGTCGGTTCATTTTGGGGATTTTCC

CCCTTGATGTTTTGACAGATTGAAGTGAGCTTTAGTGAGCAAAAGGATCAGAATGCAGGGAACACTAAGC

TGTGATGAAGAAAGTGTGGTAAAAAGCCAGAGTAGTTTTATACAGACAAAACCAGTGTCAGGCCTTTGCA

GTAGGCTTGAGTGAACTTCTGATCTAGATTTGAAAGTAAATTTTATGAAGACATTGCCCATTTTTACTTC

CTCATTCATTATTGTACCAGCATCATAGCTTTATTACTCTAATCCCAGGTAAGTCAAGCCTACAATGCCC

TAGAGGAAGAGTAAAACCAGAAATTCATGCTGGCTTAAATAATCTATTTTTGTTTCTTTTCATTTGAATA

TTTAAATTTTATGGTTTATTAAAAAATTAAATAAA

>NM_004849.4 Homo sapiens autophagy related 5 (ATG5), transcript variant 1, mRNA

AGCGTTGCCGGTTGTATTCGCTGGATACCAGAGGGCGGAAGTGCAGCAGGGTTCAGCTCCGACCTCCGCG

CCGGTGCTTTTTGCGGCTGCGCGGGCTTCCTGGAGTCCTGCTACCGCGTCCCCGCAGGACAGTGTGTCAG

GCGGGCAGCTTGCCCCGCCGCCCCACCGGAGCGCGGAATCTGGGCGTCCCCACCAGTGCGGGGAGCCGGA

AGGAGGAGCCATAGCTTGGAGTAGGTTTGGCTTTGGTTGAAATAAGAATTTAGCCTGTATGTACTGCTTT

AACTCCTGGAAGAATGACAGATGACAAAGATGTGCTTCGAGATGTGTGGTTTGGACGAATTCCAACTTGT

TTCACGCTATATCAGGATGAGATAACTGAAAGGGAAGCAGAACCATACTATTTGCTTTTGCCAAGAGTAA

GTTATTTGACGTTGGTAACTGACAAAGTGAAAAAGCACTTTCAGAAGGTTATGAGACAAGAAGACATTAG

TGAGATATGGTTTGAATATGAAGGCACACCACTGAAATGGCATTATCCAATTGGTTTGCTATTTGATCTT

CTTGCATCAAGTTCAGCTCTTCCTTGGAACATCACAGTACATTTTAAGAGTTTTCCAGAAAAAGACCTTC

TGCACTGTCCATCTAAGGATGCAATTGAAGCTCATTTTATGTCATGTATGAAAGAAGCTGATGCTTTAAA

ACATAAAAGTCAAGTAATCAATGAAATGCAGAAAAAAGATCACAAGCAACTCTGGATGGGATTGCAAAAT

GACAGATTTGACCAGTTTTGGGCCATCAATCGGAAACTCATGGAATATCCTGCAGAAGAAAATGGATTTC

GTTATATCCCCTTTAGAATATATCAGACAACGACTGAAAGACCTTTCATTCAGAAGCTGTTTCGTCCTGT

GGCTGCAGATGGACAGTTGCACACACTAGGAGATCTCCTCAAAGAAGTTTGTCCTTCTGCTATTGATCCT

GAAGATGGGGAAAAAAAGAATCAAGTGATGATTCATGGAATTGAGCCAATGTTGGAAACACCTCTGCAGT

GGCTGAGTGAACATCTGAGCTACCCGGATAATTTTCTTCATATTAGTATCATCCCACAGCCAACAGATTG

AAGGATCAACTATTTGCCTGAACAGAATCATCCTTAAATGGGATTTATCAGAGCATGTCACCCTTTTGCT

TCAATCAGGTTTGGTGGAGGCAACCTGACCAGAAACACTTCGCTGCTGCAAGCCAGACAGGAAAAAGATT

CCATGTCAGATAAGGCAACTGGGCTGGTCTTACTTTGCATCACCTCTGCTTTCCTCCACTGCCATCATTA

AACCTCAGCTGTGACATGAAAGACTTACCGGACCACTGAAGGTCTTCTGTAAAATATAATGAAGCTGAAA

CCTTTGGCCTAAGAAGAAAATGGAAGTATGTGCCACTCGATTTGTATTTCTGATTAACAAATAAACAGGG

GTATTTCCTAAGGTGACCATGGTTGAACTTTAGCTCATGAAAGTGGAAACATTGGTTTAATTTTCAAGAG

AATTAAGAAAGTAAAAGAGAAATTCTGTTATCAATAACTTGCAAGTAATTTTTTGTAAAAGATTGAATTA

CAGTAAACCCATCTTTCCTTAACGAAAATTTCCTATGTTTACAGTCTGTCTATTGGTATGCAATCTTGTA

ACTTTGATAATGAACAGTGAGAGATTTTTAAATAAAGCCTCTAAATATGTTTTGTCATTTAATAACATAC

AGTTTTGTCACTTTTCAAGTACTTTCTGACTCACATACAGTAGATCACTTTTTACTCTGTGTTACCATTT

TGACTGGTCGTCATTGGCATGGGGTGGATATAGGGCATAGGATTACTTGTCTCAGAAGCTGTCATAGAAT

TTCTTGCTGCCAATTAAAAAACCTGTGTTCTTTACACACTACACGTATAAATATTGTAACTGTTCATCTT

TGTTGTTTTATCACTGTAAGCCTGTCAAATCATAGTATCCTAAGCATCTGTAAATGCTAATTTTGCATTT

TTGGAAAAACCCATTCCTTCCAAGCTAGTGTTTTTCATTGGCTCCAGGTCTAATTTTTCACTGTGGTCCC

TGGCAGCCAGTCTTTTGAAGTTTAAAGATTACCTGTCTCTTGACTGCAGTACCTTTTCTTTAATTTTTAC

CAAAAATATCCAGAGGTTACTGGAGTTCTTATTCAATATAAGGAAAGTTTGCTGCACTTTATTACCAAGC

CTCTGGGATTTTACCAGTCAAACATATTTGTGCATTACATTTCATTTCTTGTGAGCTAGCTGGCTGTCCA

TATTGAATGTTGACCCATTTGAGTACGCTAAAAGGCTTACAGTATCAGACACGATCATGGTTTTAGATCC

CATAATAAAAATGAATGTTTTTCTTATAAAAAATTATACAAATGCTGAAGTGAGATTCTACTATTGTTCA

TTGCTTCCTTTTCTTTTTCCTTTTGCGATTTTCACTGATTAATAGCACATTTCTTCACAAAATTAGATAA

AGTTGGTCAAAGACCAGATATTCTGGAATGGAAATTGTAAAGCTTAATCAAAAAGAATAGCCAGTACAGC

ATACAATCTCAGAAACTTAGAAGCAAGTAGAAAATAATTGGTTGATGTAAACGAAAGTGCCATTTTAGTA

AAGGCAGGAAAAAAATAGCAATATTTGAGTTATGTAAGGATAAAAAATCCACTGACTTGTATTTTTGCAC

AAGAGGCTGGTCTGAATATGATTGTTCACATTAAGAGTGTTTATTCGTCGGTTCATTTTGGGGATTTTCC

CCCTTGATGTTTTGACAGATTGAAGTGAGCTTTAGTGAGCAAAAGGATCAGAATGCAGGGAACACTAAGC

TGTGATGAAGAAAGTGTGGTAAAAAGCCAGAGTAGTTTTATACAGACAAAACCAGTGTCAGGCCTTTGCA

GTAGGCTTGAGTGAACTTCTGATCTAGATTTGAAAGTAAATTTTATGAAGACATTGCCCATTTTTACTTC

CTCATTCATTATTGTACCAGCATCATAGCTTTATTACTCTAATCCCAGGTAAGTCAAGCCTACAATGCCC

TAGAGGAAGAGTAAAACCAGAAATTCATGCTGGCTTAAATAATCTATTTTTGTTTCTTTTCATTTGAATA

TTTAAATTTTATGGTTTATTAAAAAAATTAAATAAA

>NM_006395.3 Homo sapiens autophagy related 7 (ATG7), transcript variant 1, mRNA

AGAGAGAGCTGTGGTTGCCGGAAGTTGAGCGGCGGCAAGAAATAATGGCGGCAGCTACGGGGGATCCTGG

ACTCTCTAAACTGCAGTTTGCCCCTTTTAGTAGTGCCTTGGATGTTGGGTTTTGGCATGAGTTGACCCAG

AAGAAGCTGAACGAGTATCGGCTGGATGAAGCTCCCAAGGACATTAAGGGTTATTACTACAATGGTGACT

CTGCTGGGCTGCCAGCTCGCTTAACATTGGAGTTCAGTGCTTTTGACATGAGTGCTCCCACCCCAGCCCG

TTGCTGCCCAGCTATTGGAACACTGTATAACACCAACACACTCGAGTCTTTCAAGACTGCAGATAAGAAG

CTCCTTTTGGAACAAGCAGCAAATGAGATATGGGAATCCATAAAATCAGGCACTGCTCTTGAAAACCCTG

TACTCCTCAACAAGTTCCTCCTCTTGACATTTGCAGATCTAAAGAAGTACCACTTCTACTATTGGTTTTG

CTATCCTGCCCTCTGTCTTCCAGAGAGTTTACCTCTCATTCAGGGGCCAGTGGGTTTGGATCAAAGGTTT

TCACTAAAACAGATTGAAGCACTAGAGTGTGCATATGATAATCTTTGTCAAACAGAAGGAGTCACAGCTC

TTCCTTACTTCTTAATCAAGTATGATGAGAACATGGTGCTGGTTTCCTTGCTTAAACACTACAGTGATTT

CTTCCAAGGTCAAAGGACGAAGATAACAATTGGTGTATATGATCCCTGTAACTTAGCCCAGTACCCTGGA

TGGCCTTTGAGGAATTTTTTGGTCCTAGCAGCCCACAGATGGAGTAGCAGTTTCCAGTCTGTTGAAGTTG

TTTGCTTCCGTGACCGTACCATGCAGGGGGCGAGAGACGTTGCCCACAGCATCATCTTCGAAGTGAAGCT

TCCAGAAATGGCATTTAGCCCAGATTGTCCTAAAGCAGTTGGATGGGAAAAGAACCAGAAAGGAGGCATG

GGACCAAGGATGGTGAACCTCAGTGAATGTATGGACCCTAAAAGGTTAGCTGAGTCATCAGTGGATCTAA

ATCTCAAACTGATGTGTTGGAGATTGGTTCCTACTTTAGACTTGGACAAGGTTGTGTCTGTCAAATGTCT

GCTGCTTGGAGCCGGCACCTTGGGTTGCAATGTAGCTAGGACGTTGATGGGTTGGGGCGTGAGACACATC

ACATTTGTGGACAATGCCAAGATCTCCTACTCCAATCCTGTGAGGCAGCCTCTCTATGAGTTTGAAGATT

GCCTAGGGGGTGGTAAGCCCAAGGCTCTGGCAGCAGCGGACCGGCTCCAGAAAATATTCCCCGGTGTGAA

TGCCAGAGGATTCAACATGAGCATACCTATGCCTGGGCATCCAGTGAACTTCTCCAGTGTCACTCTGGAG

CAAGCCCGCAGAGATGTGGAGCAACTGGAGCAGCTCATCGAAAGCCATGATGTCGTCTTCCTATTGATGG

ACACCAGGGAGAGCCGGTGGCTTCCTGCCGTCATTGCTGCAAGCAAGAGAAAGCTGGTCATCAATGCTGC

TTTGGGATTTGACACATTTGTTGTCATGAGACATGGTCTGAAGAAACCAAAGCAGCAAGGAGCTGGGGAC

TTGTGTCCAAACCACCCTGTGGCATCTGCTGACCTCCTGGGCTCATCGCTTTTTGCCAACATCCCTGGTT

ACAAGCTTGGCTGCTACTTCTGCAATGATGTGGTGGCCCCAGGAGATTCAACCAGAGACCGGACCTTGGA

CCAGCAGTGCACTGTGAGTCGTCCAGGACTGGCCGTGATTGCAGGAGCCCTGGCCGTGGAATTGATGGTA

TCTGTTTTGCAGCATCCAGAAGGGGGCTATGCCATTGCCAGCAGCAGTGACGATCGGATGAATGAGCCTC

CAACCTCTCTTGGGCTTGTGCCTCACCAGATCCGGGGATTTCTTTCACGGTTTGATAATGTCCTTCCCGT

CAGCCTGGCATTTGACAAATGTACAGCTTGTTCTTCCAAAGTTCTTGATCAATATGAACGAGAAGGATTT

AACTTCCTAGCCAAGGTGTTTAATTCTTCACATTCCTTCTTAGAAGACTTGACTGGTCTTACATTGCTGC

ATCAAGAAACCCAAGCTGCTGAGATCTGGGACATGAGCGATGATGAGACCATCTGAGATGGCCCCGCTGT

GGGGCTGACTTCTCCCCGGCCGCCTGCTGAGGAGCTCTCCATCGCCAGAGCAGGACTGCTGACCCCAGGC

CTGGTGATTCTGGGCCCCTCCTCCATACCCCGAGGTCTGGGATTCCCCCCTCTGCTGCCCAGGAGTGGCC

AGTGTTCGGCGTTGCTCGGGATTCAAGATACCACCAGTTCAGAGCTAAATAATAACCTTGGCCTTGGCCT

TGCTATTGACCTGGGACTTGGTCCTCCATGCAGTTTTTATTTCTTGTCACAGTGACTGATAGCCATCCCC

CAGGATCCTTTCCCCTTGGCCCTGAGGGGGTGACCCAACACAGACCAAATGGGGAAATGAGCAACCAGCT

CCTGCCCAGAGCCACTGCGGGAGGTGGCACCCTCATCCCCGGAATGTGCTGCCCACCGCACCGCAGGCTC

CTCCTGTGGGGGCCCTGGGCATGGGTGAGGGTGGGACCCCGTGAGCGCACTGCACCCTGGCCCTGGTGGA

GCGGGAGGAGGAGGAGAGCCGAGCTGGGTACGAGACTAAAGGGCCCACATGACCCAGTGACGCCAGATTT

CCACCAAGGACTGAGTGAGCTGCTCAGACATGGCTTTCTGCCTCCCAGCCTGTCCTCCACTGTGGGCATA

GCATCTGTGCCTGCCTGCCTGCTTGAGGGAGAGGAGTTTCTGCTGCTGCCTTGAGCTGGGGGGAAGAGCC

CAGGGGCAGATCCTGGCAGCTGCCTGGATGGGGCTCCTCCCTGCCCTTATGAGCAGGCCAGGCCCAGAAA

GGCCGAGCCTGGGCTGCCTTCCTGCCCCAGCCGAGGGAGGGGTCAGACGGCTCTACCATGGGTAACTCAG

GCAAGAGCTGGTTTTCCTCTTTATTCTGGGTGTGTGCAGCTGTGAGGCCCCAACCCAGGAGAGGCCATGG

CCTAGGTACCTGTGACCACCCTGCCCCCGTGTAGAGGGCATCGTCTTTCCTGCTATTTTATTCTTTCAGC

TTTTGTCTTAGGCCCAGAATCAAAGTGAAAATTGAGTCGAGCTGACCCTTACAACAGTAGGATTTAGTAG

GGTAGATTTCAAATGAGGCTTCGCTTCTCCCAAAGTAGCCAGTCCAAGTTCCAGTGGCTGTCGTTCAGCT

CATGGGAGCTTCATGGGGACACAGCCGGCACAGGTGCAGGGCCCGAGTCCGCCCACCCAGCCTGGCGCTG

AAACTGCACACGTACACTATGTGGTTTAAGAGCACTTTATTATTGTTCTTAAGGCTACTTTTAAGTACAA

AAAAAGATGGCCTGCCAAACCTTTTTTTTTCTTCTTCCAGGAAAAACAGGCCACAGAGAATGGTATATTA

CAGATTTACACACATGAAGAGAAGGTCAGAGCGCACTGCAGGCAGCGCGGCTCTGGGAAGAACTTCACGG

AGCCCCTTCTTAGAGCAGGGAGGGGGCTTTCTCAGTGAAATGTTTGGTTTTCTGCTGCCTCCTCTGCCCC

AGGCCCCCCTCCAGGGTACTGCCTATCCCAGATAGGTCAGTGCACCAGGGACCCGGCCGCCAGCACCGCC

GACCCCTCCCAGAGTGACGCCCTTGTTCACTGACAAAGAGACCTGTCCCAGGAGTGTCCTCCACCGAGCC

GGTCAGCTGTGGGTGGTTTTCCTGTTACGACGCTCAGTAGCCTGTAGCAATAACAAACTCGTGGCTATGA

ATGCAGATGCAGTGTTCTCATAGAATAACTGTTCCTGCACTTTTACAGACAAATCTACGACAAAAAAAAA

GATCAACTTTTTTTTTCCGAACAACAAAAAAAAATGAATGATTACAATAGGAAAGGGAAAAATTAAATAGC

TACATATCATTAACAAATTAATGTTCTTCAAAAAATACCTACAAATTTCTCTGTACATTCTTTACGCACA

GCGTAACGATGGTCTCAAAATCACCCATATAGAAAAGTGTTCTCAACGATTTTTCCTACAGAAAATATAG

GGGCCTGAATGCCAAAGCTTGGAAGCCCAGTACAGTGGGAGTGAAATGTGTGCGGGGCAAGGAGAAGGGC

TTTTCTTTCCTCCACTTTTCAAAGGCCTGCAGCCACTCTGTGACTACAAGAGCCAGTCCTCCGACCTTTT

CACCCAGTGCCAATTTCCAAAATTCAACAGCTAAAAACTGTAAAACCGGGGGTCATACGGTGTGCAGAGT

CCACAAAGCCTTGCAGGTGAGGTGACCACGCCCACGTCACCTGGTCAGGTGCCATCGTCGTGAGCCTCTG

GTGGGCCAGGTGGGACACAGCACACCCCAGGGGGAGGGGATAGAAACGCTCATTGACCAAAAAGGAGCAG

CTGTGACCTCCACAGCTGTGTCTGTCATGCTTGCTTCATCTAATTTCTAGTTAGTAGCTATTAATATAGC

AAATAATAAATGCAGTAATAACAGTATAAAGTCAGAGGAATGTATACTGCCTTGGCCCCAGCGTACGAGG

AAGCGTATAAAACACCATATCACAGATTGTCTGTCAGTAATCTGCTGTTCAGCCAAGAGAGTTCAAAGGG

AGCAGTTTCTGCATGTAGGGAAGTTGGAAGACACAAACCCCACCTCCCCTGGGAGCTTGTAACAAAGCAG

ACAGGGATGCAAAAATAAATGATGTCAGCCTGCAGCCAAACTCCAGCATCCCACACCGCAGCTGACCCAC

TGCTCATCGCGAGGGCCTGCCAGGAGCTGGCCTCCCGCACTACTTGTGAGTAAAGTGAATATCAAATACC

AATCTTAGAGTACAACTGTACCAGCAGTAAGTATATCTAGGACTGTAACTGACAAAAATAAACTAATTCT

GAAAAGAA

>NM_024085.5 Homo sapiens autophagy related 9A (ATG9A), transcript variant 2, mRNA

GAGTGGCAGACACCCGGCCTAGCGCCGCGGGTCGCGCCGAGCCGAGCCGAGCCGAGCGGAGCCGGCGGAG

CCTCTGGAATCACCCGGGTCGCTGTTCCTGAGGTGGTCAAGGTGGACAGGGGGCGGTGGTGATGGCGCAG

TTTGACACTGAATACCAGCGCCTAGAGGCCTCCTATAGTGATTCACCCCCAGGGGAGGAGGACCTGTTGG

TGCACGTCGCCGAGGGGAGCAAGTCACCTTGGCACCATATTGAAAACCTTGACCTCTTCTTCTCTCGAGT

TTATAATCTGCACCAGAAGAATGGCTTCACATGTATGCTCATCGGGGAGATCTTTGAGCTCATGCAGTTC

CTCTTTGTGGTTGCCTTCACTACCTTCCTGGTCAGCTGCGTGGACTATGACATCCTATTTGCCAACAAGA

TGGTGAACCACAGTCTTCACCCTACTGAACCCGTCAAGGTCACTCTGCCAGACGCCTTTTTGCCTGCTCA

AGTCTGTAGTGCCAGGATTCAGGAAAATGGCTCCCTTATCACCATCCTGGTCATTGCTGGTGTCTTCTGG

ATCCACCGGCTTATCAAGTTCATCTATAACATTTGCTGCTACTGGGAGATCCACTCCTTCTACCTGCACG

CTCTGCGCATCCCTATGTCTGCCCTTCCGTATTGCACGTGGCAAGAAGTGCAGGCCCGGATCGTGCAGAC

GCAGAAGGAGCACCAGATCTGCATCCACAAACGTGAGCTGACAGAACTGGACATCTACCACCGCATCCTC

CGTTTCCAGAACTACATGGTGGCACTGGTTAACAAATCCCTCCTGCCTCTGCGCTTCCGCCTGCCTGGCC

TCGGGGAAGCTGTCTTCTTCACCCGTGGTCTCAAGTACAACTTTGAGCTGATCCTCTTCTGGGGACCTGG

CTCTCTGTTTCTCAATGAATGGAGCCTCAAGGCCGAGTACAAACGTGGGGGGCAACGGCTAGAGCTGGCC

CAGCGCCTCAGCAACCGCATCCTGTGGATTGGCATCGCTAACTTCCTGCTGTGCCCCCTCATCCTCATAT

GGCAAATCCTCTATGCCTTCTTCAGCTATGCTGAGGTGCTGAAGCGGGAGCCGGGGGCCCTGGGAGCACG

CTGCTGGTCACTCTATGGCCGCTGCTACCTCCGCCACTTCAACGAGCTGGAGCACGAGCTGCAGTCCCGC

CTCAACCGTGGCTACAAGCCCGCCTCCAAGTACATGAATTGCTTCTTGTCACCTCTTTTGACACTGCTGG

CCAAGAATGGAGCCTTCTTCGCTGGCTCCATCCTGGCTGTGCTTATTGCCCTCACCATTTATGACGAAGA

TGTGTTGGCTGTGGAACATGTGCTGACCACCGTCACACTCCTGGGGGTCACCGTGACCGTGTGCAGGTCC

TTTATCCCGGACCAGCACATGGTGTTCTGCCCTGAGCAGCTGCTCCGCGTGATCCTCGCTCACATCCACT

ACATGCCTGACCACTGGCAGGGTAATGCCCACCGCTCGCAGACCCGGGACGAGTTTGCCCAGCTCTTCCA

GTACAAGGCAGTGTTCATTTTGGAAGAGTTGCTGAGCCCCATTGTCACACCCCTCATCCTCATCTTCTGC

CTGCGCCCACGGGCCCTGGAGATTATAGACTTCTTCCGAAACTTCACCGTGGAGGTCGTTGGTGTGGGAG

ATACCTGCTCCTTTGCTCAGATGGATGTTCGCCAGCATGGTCATCCCCAGTGGCTATCTGCTGGGCAGAC

AGAGGCCTCAGTGTACCAGCAAGCTGAGGATGGAAAGACAGAGTTGTCACTCATGCACTTTGCCATCACC

AACCCTGGCTGGCAGCCACCACGTGAGAGCACAGCCTTCCTAGGCTTCCTCAAGGAGCAGGTTCAGCGGG

ATGGAGCAGCTGCTAGCCTCGCCCAAGGGGGTCTGCTCCCTGAAAATGCCCTCTTTACGTCTATCCAGTC

CTTACAATCTGAGTCTGAGCCCCTGAGCCTTATCGCAAATGTGGTAGCTGGCTCATCCTGCCGGGGCCCT

CCACTGCCCAGAGACCTGCAGGGCTCCAGGCACAGGGCTGAAGTCGCCTCTGCCCTGCGCTCCTTCTCCC

CGCTGCAACCCGGGCAGGCGCCCACAGGCCGGGCTCACAGCACCATGACAGGCTCTGGGGTGGATGCCAG

GACAGCCAGCTCCGGGAGCAGCGTGTGGGAAGGACAGCTGCAGAGCCTGGTGCTGTCAGAATATGCATCC

ACAGAGATGAGCCTGCATGCCCTCTATATGCACCAGCTCCACAAGCAGCAGGCCCAGGCTGAACCTGAGC

GGCATGTATGGCACCGCCGGGAGAGTGATGAGAGTGGAGAAAGCGCCCCTGATGAAGGGGGAGAGGGCGC

CCGGGCCCCCAGTCTATCCCTCGCTCTGCTAGCTATCCCTGTGCAGCACCCCGGCCTGGAGCTCCTGAG

ACCACTGCCCTGCATGGGGGCTTCCAGAGGCGCTACGGTGGCATCACAGATCCTGGCACAGTGCCCAGGG

TTCCCTCTCATTTCTCTCGGCTGCCTCTTGGAGGGTGGGCAGAAGATGGGCAGTCGGCATCAAGGCACCC

TGAGCCCGTGCCCGAAGAGGGCTCGGAGGATGAGCTACCCCCTCAGGTGCACAAGGTATAGACAAGGCTG

AGCAGGGTTCCTGTGGCCCAGGATGGAGGCCACCGCTGCCCTGCCATCCCGTCTGCCTGCCATGGGACGG

CTCCTCTGAGTGTTCCCTGGCCCCACGTGTGTGGTGTTTGTGTGTCTGTGCCTGGCCAAGGGAGGTGCCA

ACACTGGGCTTGCCACAGCCCCAGGAGAGGAATTTGGGGCCTAGGAACCGAGGGCACACGGGACTCTAGC

CTCATCCCCAGGACCCCCTTGGCTCAGAGTGTGGTGCTAGAAACTGGCCCCCAGCCCAGCCCCAGTACTG

CCACCTTTACACCTACCCCTGCAAGTCCCCAGAGGGCTGCCCACGATAGAAGCTGCCAAGCAGGGAGAAC

CTGTGCCAACTGTGGAGTGGGGAGGTTGGGCCTGGACCCTCAACCCCTGCAACCTTCCCTAGCCCCCTCA

ATAGATGAGCAGGTCAGGCTGTGGCCCTTACCTCACCCGCAGTTCTCGCCCAGTGCTGCAGCCGGCTCAC

CTCTCTCCGCTTCTTGCACATCACTGGCCTGTGTGTGCTGCTTGCTCCTGTTCTGTTCGCTTGCTCCCGT

TCCGTTCGGCTTTTGCTTTGCGTTAGGGTGAAGACCCTAGCGTCCAGCTCCCCTCAACGCTATATTTTGA

CACTAAAAAAGAAGGTTTCTAAATTGTAGGAGCAGGATGGAAATACTTTGCTGCCCTTGCCATCTTTTAG

GATGGGCCCCCAGGAGACTGAGGTCTTCCTGGGCCCTCATTGCTGCTTATCGTACCCCCCATCACCTGCA

CATGGGACAGACCGGGCTGGAGGGTGACCTTGGCTGTGTGCGTCCCAGCAAAAGAGCTCTGGCCCGCATC

TCGCTGTGCCCTGAAGGGGGATGAAGGGCGATGCCTCGCCCGAGGCTTTGGGCTGCTGCACTGCATGCTG

GGACTGCTCCTACTCTCTGTCCCACCCCTCACCCAGCTGTGGTCCGGCTTTGGGAGAGTGGTGAATTGCG

CTGCCCGAACTCGGAGCGGAGCAGGGTAGGGACCGTGTACAGCTTGATAACCCTTAATAAAAAGGGAGTT

TGACCAGA

>NM_173681.5 Homo sapiens autophagy related 9B (ATG9B), transcript variant 1, mRNA

AGCTCAGTGTCACAGACCTTTGTGGAAGCAACAACAGCTCCCATCCTTCCAACCTTTCTGGAGAAGCCGT

GGCCTGATGGTGAGCCGAATGGGCTGGGGGGGGAGAAGAAGGCGGCTGGGGCGGTGGGGAGATCTGGGGC

CCGGATCGGTGCCCCTCCTCCCCATGCCACTGCCACCTCCTCCTCCTTCATGCCGGGGACCTGGGGG

AGGGAGGATCTCCATCTTCTCTCTGTCCCCTGCCCCTCATACAAGAAGCTCCCCCTCCTCATTTTCCCCT

CCCACCGCAGGGCCCCCTTGCTCAGTGCTACAGGGGACAGGGGCTTCTCAGTCTTGCCACAGTGCTCTCC

CTATCCCAGCCACCCCCCCAACACAGGCTCAACCTGCAATGACACCTGCCTCTGCATCTCCCTCCTGGGG

ATCCCACTCCACCCCACCCCTGGCCCCGGCAACCCCCACTCCCTCACAGCAGTGCCCCCAGGACTCTCCT

GGGCTGCGGGTAGGCCCTTTGATCCCTGAACAGGATTATGAGCGGCTGGAGGACTGTGACCCTGAGGGGT

CCCAAGACTCACCCATCCACGGGGAGGAGCAGCAACCCCTGCTTCATGTCCCTGAAGGGCTCCGAGGCTC

CTGGCATCACATCCAGAACCTGGACAGTTTCTTCACCAAGATCTACAGCTACCACCAGCGGAATGGCTTT

GCCTGCATCTTGCTGGAGGATGTCTTCCAGCTGGGACAATTTATTTTCATTGTCACCTTCACAACCTTCC

TCCTTCGATGCGTGGATTACAATGTTCTCTTTGCCAACCAACCAAGTAACCATACCAGACCTGGGCCGTT

CCACAGCAAAGTGACCCTGTCAGATGCCATCCTACCCTCAGCCCAGTGTGCTGAGAGGATCCGCTCCAGC

CCGCTGCTGGTCCTCCTCCTGGTCCTGGCTGCCGGCTTCTGGCTGGTCCAACTGCTTCGCTCAGTCTGCA

ACCTCTTCAGCTACTGGGACATCCAGGTGTTTTACAGGGAGGCCCTGCACATCCCCCCGGAGGAGCTGAG

CTCGGTTCCCTGGGCAGAGGTGCAGTCCCGCCTCTTGGCACTGCAGCGGAGCGGGGGCCTGTGCGTGCAG

CCGCGGCCCCTGACGGAGCTGGACATCCACCACCGCATCCTGCGCTACACCAACTACCAGGTGGCGCTGG

CCAACAAAGGCCTGCTGCCGGCCCGCTGCCCGCTGCCCTGGGGAGGCAGTGCGGCTTTCCTCAGCCGCGG

CCTGGCGCTCAATGTCGACCTGCTGCTCTTCCGCGGTCCCTTCTCGCTCTTCCGCGGGGGCTGGGAGCTG

CCGCACGCCTACAAGCGCAGCGACCAGCGGGGCGCCCTAGCAGCGCGCTGGGGGCGCACAGTGCTGCTGC

TGGCCGCCCTGAACCTGGCGCTGAGCCCGCTGGTGCTGGCCTGGCAGGTTCTGCACGTCTTCTATAGCCA

CGTGGAGCTGCTGCGGCGCGAGCCTGGCGCGCTGGGGGCGCGCGGCTGGTCCCGCCTGGCGCGCTTGCAG

CTGCGCCACTTCAACGAGCTGCCGCACGAGCTGCGCGCGCGCCTGGCCCGCGCCTACCGCCCCGCCGCCG

CCTTCCTGCGCACCGCTGCGCCCCCCGCGCCCCTGCGCACGCTGCTGGCCCGCCAGCTCGTTTTCTTCGC

GGGTGCACTCTTCGCCGCGCTGCTTGTGCTCACCGTCTACGACGAGGACGTGCTAGCCGTGGAGCACGTG

CTCACCGCCATGACCGCGCTCGGGGTCACCGCCACCGTCGCCAGGTCTTTCATTCCGGAAGAGCAGTGCC

AGGGTCGTGCGCCGCAGCTCCTGCTGCAGACAGCCCTGGCCCACATGCACTACCTCCCGGAGGAGCCCGG

CCCCGGCGGCAGGGACCGCGCCTACCGGCAGATGGCGCAGCTGCTGCAGTACCGAGCGGTCTCCCTCCTG

GAGGAGCTCCTGTCCCCGCTCCTCACCCCGCTGTTTCTGCTTTTCTGGTTCCGCCCTCGTGCCCTGGAGA

TTATCGACTTTTTTCATCACTTCACTGTGGATGTGGCTGGGGTTGGGGACATCTGTTCCTTTGCCCTTAT

GGATGTGAAGCGCCACGGACACCCTCAGTGGCTCTCGGCGGGACAGACTGAGGCCTCGCTGTCTCAGCGT

GCGGAGGACGGCAAGACTGAGCTTTCTTTGATGCGGTTCTCCCTGGCGCATCCACTCTGGCGCCCCCCAG

GGCACAGCTCTAAGTTTCTTGGGCACCTCTGGGGCCGAGTACAACAAGATGCAGCTGCCTGGGGTGCCAC

CTCGGCTCGCGGCCCCTCCACCCCGGGGGTGCTCAGCAACTGCACCTCGCCCCTGCCTGAGGCCTTCCTG

GCCAACCTCTTCGTGCACCCTCTCCTGCCTCCGAGAGATCTGAGCCCGACAGCCCCCTGTCCAGCTGCGG

CCACAGCCAGCCTCCTTGCCTCCATTTCCCGAATTGCCCAGGACCCCAGCTCTGTGTCCCCAGGAGGCAC

TGGGGGCCAGAAGCTGGCCCAGCTCCCAGAACTTGCTTCTGCCGAGATGAGTCTCCATGTCATCTACCTG

CACCAGCTTCACCAGCAGCAGCAGCAGCAGGAGCCGTGGGGTGAGGCTGCAGCCTCCATCCTGTCCAGGC

CCTGCTCCAGCCCCTCACAGCCACCCTCGCCTGATGAGGAGAAGCCATCCTGGTCAAGTGACGGCTCCAG

TCCTGCCTCTAGCCCCAGACAACAGTGGGGAACCCAGAAGGCCCGGAATCTGTTCCCCGGAGGGTTTCAG

GTGACCACAGACACCCAGAAGGAGCCTGACCGGGCCTCTTGCACTGACTGAGACGACTCCTCAGGGCCCC

ACTCCTGGCAGCTGAGTGCTGCACCTGCCTTCTCGATCACGAGCCTGAGAACTGCACGGGGGAGCAGCCC

AGACTGGCTGGAAACCATGTTTTCAGAGTGCAGGGTCAAGTGGCGTCCTCTGGACAGATGTGAGAAGGCA

CCACCTCAAGAGGGGAGGAGCGGGGCTGATCCTGGTCGGGCGGACCTGAGTCGGGCAGCCGCTCTCCCGG

AACTGGAAGGGAAAGCCAGGCGGCTCTCAGGGGCTGTTGGTGTCTGAGCCGGGAGGGTCGAACGCCCAGG

GCACTGCGCCCCGCAACTGACGCTCCTGCAAGGAAAAGCTCTGGCACGCCGATGACGTCGCCGGCCTCGT

CCAGCTCCATGTCGCCCTCCGTCGCCAGGATGCGCTGCACGGTCTGCAGGACGTTGGTTGCCATGGTAAC

ATCGCCGCAGACAAACATGTGGCCCCGCTCGAGGCACAGCACGCGGTGCACCTCCGCAGCCAGCTCCGTC

CTCAGGATGTCCTGCACGTTACCATTGCGCCCTCAGGGTCTCACACCTTGGGGTTGTCAGGTTCCCGGGA

GAAGGCGGTGAGGACTCGGCCAAACACCCCGCGCTGCTGGGCGTTCTGCACCTCGTCGCGGTAGAGATGG

TCAAGTTGGGAGCATCGGCAGCCGAACACCAAAGTCATGGGAGTGGGCTGCAGCCCTGCAAGCAACCCCG

CGCTTGGGAAGGCTCTTCTCCGGGGCACAGTGCTAGTGGGGCCTCCCCACAGGCCACCGTCGCCCAGCCT

GGCAGCCCAGCGTTTTGGGCTGAAACCACCATTACTTTGGCTGTTTCATCTCTAAGAGAGTGTAGAATGC

TTTCACAGAGACCCTGCACAGCCCTGATTTACAGAGGGAAGGGAAGCGCGGGTGAGGTGGTGGCCCCTGG

TGGAGCTGCGCTAGAATGCGCGGCTTCGGACTCAGAATTCCATGCCCTTTGCATTGTGTGACATCGCAGT

CCCCTTCCCCGCTCCCACTTCATATTCTCCCATACTTATAACTCTCCTCCCAGTCACAGCCCTCTTCTTT

TGATTGACAGACCACCCTGGGCACCCCATGCTCAATATGAACACCTTGTAGCATCCTTAGGTCTTGGACA

GACCTCGGTCAGCTCTGGCACAGTCAAGAATCCACCTTGGGCAGTTCTTCTTTGAGTCTTAGCCCTAATC

CCTTTGAAAATTCTATTTTCCTTTGTCCTCAATCCAGTCAACACCTACTATGTACCAGGCACCAGGAAGC

CAGAAGCTCAGGGCCCAGCCCTTGCCTTTGGTACCTGCCCTCCCCTTGGTCTCATGAGCGTCTTTTCAGC

TTTGTGGACTTTGTTTCCTGCCCTCTCTTTCCTATTTCCTGCCTTCCAGCCCCACCCCTCTGTCCCTAGA

TTGTGTGACTCCCTTCAGGCAGTCCTTTAGTCCCCAGCCTCACCTTTGCTCTCAATGTCATGCAGCCGCT

CCTGCCAGAATCCCCGGAAGGGGGCAATGCCAGTGCCTGGACCCACCAGGATGCAGGGCAAGCTGGGATC

GGGTGGCAGCCGGAAGGAGGGAGCCCTGGCAGGAGAGACACTCCATCAGGTCCCCTTTGCCTTTCCTGCT

CCACCATTCTCACCATGCAGGGTGGGTGGAGCAAGGAGGACCCAAGTTCTTCCCATCTCAAAAAAAAATT

AAAAAAAAAACAAAAAACAAAAAACAA

>NM_004044.7 Homo sapiens 5-aminoimidazole-4-carboxamide ribonucleotide formyltransferase/IMP cyclohydrolase (ATIC), mRNA

GCCACATCCCGGCAGCCCTCCTACCTGCGCACGTGGTGCCGCCGCTGCTGCCTCCCGCTCGCCCTGAACC

CAGTGCCTGCAGCCATGGCTCCCGGCCAGCTCGCCTTATTTAGTGTCTCTGACAAAACCGGCCTTGTGGA

ATTTGCAAGAAACCTGACCGCTCTTGGTTTGAATCTGGTCGCTTCCGGAGGGACTGCAAAAGCTCTCAGG

GATGCTGGTCTGGCAGTCAGAGATGTCTCTGAGTTGACGGGATTTCCTGAAATGTTGGGGGGACGTGTGA

AAACTTTGCATCCTGCAGTCCATGCTGGAATCCTAGCTCGTAATATTCCAGAAGATAATGCTGACATGGC

CAGACTTGATTTCAATCTTATAAGAGTTGTTGCCTGCAATCTCTATCCCTTTGTAAAGACAGTGGCTTCT

CCAGGTGTAACTGTTGAGGAGGCTGTGGAGCAAATTGACATTGGTGGAGTAACCTTACTGAGAGCTGCAG

CCAAAAACCACGCTCGAGTGACAGTGGTGTGTGAACCAGAGGACTATGTGGTGGTGTCCACGGAGATGCA

GAGCTCCGAGAGTAAGGACACCTCCTTGGAGACTAGACGCCAGTTAGCCTTGAAGGCATTCACTCATACG

GCACAATATGATGAAGCAATTTCAGATTATTTCAGGAAACAGTACAGCAAAGGCGTATCTCAGATGCCCT

TGAGATATGGAATGAACCCACATCAGACCCCTGCCCAGCTGTACACACTGCAGCCCAAGCTTCCCATCAC

AGTTCTAAATGGAGCCCCTGGATTTATAAACTTGTGCGATGCTTTGAACGCCTGGCAGCTGGTGAAGGAA

CTCAAGGAGGCTTTAGGTATTCCAGCCGCTGCCTCTTTCAAACATGTCAGCCCAGCAGGTGCTGCTGTTG

GAATTCCACTCAGTGAAGATGAGGCCAAAGTCTGCATGGTTTATGATCTCTATAAAACCCTCACACCCAT

CTCAGCGGCATATGCAAGAGCAAGAGGGGCTGATAGGATGTCTTCATTTGGTGATTTTGTTGCATTGTCC

GATGTTTGTGATGTACCAACTGCAAAAATTATTTCCAGAGAAGTATCTGATGGTATAATTGCCCCAGGAT

ATGAAGAAGAAGCCTTGACAATACTTTCCAAAAAGAAAAATGGAAACTATTGTGTCCTTCAGATGGACCA

ATCTTACAAACCAGATGAAAATGAAGTTCGAACTCTCTTTGGTCTTCATTTAAGCCAGAAGAGAAATAAT

GGTGTCGTCGACAAGTCATTATTTAGCAATGTTGTTACCAAAAATAAAGATTTGCCAGAGTCTGCCCTCC

GAGACCTCATCGTAGCCACCATTGCTGTCAAGTACACTCAGTCTAACTCTGTGTGCTACGCCAAGAACGG

GCAGGTTATCGGCATTGGAGCAGGACAGCAGTCTCGTATACACTGCACTCGCCTTGCAGGAGATAAGGCA

AACTATTGGTGGCTTAGACACCATCCACAAGTGCTTTCGATGAAGTTTAAAACAGGAGTGAAGAGAGCAG

AAATCTCCAATGCCATCGATCAATATGTGACTGGAACCATTGGCGAGGATGAAGATTTGATAAAGTGGAA

GGCACTGTTTGAGGAAGTCCCTGAGTTACTCACTGAGGCAGAGAAGAAGGAATGGGTTGAGAAACTGACT

GAAGTTTCTATCAGCTCTGATGCCTTCTTCCCTTTCCGAGATAACGTAGACAGAGCTAAAAGGAGTGGTG

TGGCGTACATTGCGGCTCCCTCCGGTTCTGCTGCTGACAAAGTTGTGATTGAGGCCTGCGACGAACTGGG

AATCATCCTCGCTCATACGAACCTTCGGCTCTTCCACCACTGATTTTACCACACACTGTTTTTTGGCTTG

CTTATGTGTAGGTGAACAGTCACGCCTGAAACTTTGAGGATAACTTTTTAAAAAAATAAAACAGTATCTC

TTAATCACTGGA

>NM_004323.6 Homo sapiens BAG cochaperone 1 (BAG1), transcript variant 1, mRNA

AGCTTCCATCGCTGGGCGGTCAACAAGTGCGGGCCTGGCTCAGCGCGGGGGGGCGCGGAGACCGCGAGGC

GACCGGGAGCGGCTGGGTTCCCGGCTGCGCGCCCTTCGGCCAGGCCGGGAGCCGCGCCAGTCGGAGCCCC

CGGCCCAGCGTGGTCCGCCTCCCTCTGGGCGTCCACCTGCCCGGAGTACTGCCAGCGGGCATGACCGACC

CACCAGGGGCGCCGCCGCCGGCGCTCGCAGGCCGCGGATGAAGAAGAAAACCCGGCGCCGCTCGACCCGG

AGCGAGGAGTTGACCCGGAGCGAGGAGTTGACCCTGAGTGAGGAAGCGACCTGGAGTGAAGAGGCGACCC

AGAGTGAGGAGGCGACCCAGGGCGAAGAGATGAATCGGAGCCAGGAGGTGACCCGGGACGAGGAGTCGAC

CCGGAGCGAGGAGGTGACCAGGGAGGAAATGGCGGCAGCTGGGCTCACCGTGACTGTCACCCACAGCAAT

GAGAAGCACGACCTTCATGTTACCTCCCAGCAGGGCAGCAGTGAACCAGTTGTCCAAGACCTGGCCCAGG

TTGTTGAAGAGGTCATAGGGGTTCCACAGTCTTTTCAGAAACTCATATTTAAGGGAAAATCTCTGAAGGA

AATGGAAACACCGTTGTCAGCACTTGGAATACAAGATGGTTGCCGGGTCATGTTAATTGGGAAAAAGAAC

AGTCCACAGGAAGAGGTTGAACTAAAGAAGTTGAAACATTTGGAGAAGTCTGTGGAGAAGATAGCTGACC

AGCTGGAAGAGTTGAATAAAGAGCTTACTGGAATCCAGCAGGGTTTTCTGCCCAAGGATTTGCAAGCTGA

AGCTCTCTGCAAACTTGATAGGAGAGTAAAAGCCACAATAGAGCAGTTTATGAAGATCTTGGAGGAGATT

GACACACTGATCCTGCCAGAAAATTTCAAAGACAGTAGATTGAAAAGGAAAGGCTTGGTAAAAAAGGTTC

AGGCATTCCTAGCCGAGTGTGACACAGTGGAGCAGAACATCTGCCAGGAGACTGAGCGGCTGCAGTCTAC

AAACTTTGCCCTGGCCGAGTGAGGTGTAGCAGAAAAAGGCTGTGCTGCCCTGAAGAATGGCGCCACCAGC

TCTGCCGTCTCTGGAGCGGAATTTACCTGATTTCTTCAGGGCTGCTGGGGGCAACTGGCCATTTGCCAAT

TTTCCTACTCTCACACTGGTTCTCAATGAAAAATAGTGTCTTTGTGATTTTGAGTAAAGCTCCTATCTGT

TTTCTCCTTCTGTCTCTGTGGTTGTACTGTCCAGCAATCCACCTTTTCTGGAGAGGGCCACCTCTGCCCA

AATTTTCCCAGCTGTTTGGACCTCTGGGTGCTTTCTTTGGGCTGGTGAGAGCTCTAATTTGCCTTGGGCC

AGTTTCAGGTTTATAGGCCCCCTCAGTCTTCAGATACATGAGGGCTTCTTTGCTCTTGTGATCGTGTAGT

CCCATAGCTGTAAAACCAGAATCACCAGGAGGTTGCACCTAGTCAGGAATATTGGGAATGGCCTAGAACA

AGGTGTTTGGCACATAAGTAGACCACTTATCCCTCATTGTGACCTAATTCCAGAGCATCTGGCTGGGTTG

TTGGGTTCTAGACTTTGTCCTCACCTCCCAGTGACCCTGACTAGCCACAGGCCATGAGATACCAGGGGGC

CGTTCCTTGGATGGAGCCTGTGGTTGATGCAAGGCTTCCTTGTCCCCAAGCAAGTCTTCAGAAGGTTAGA

ACCCAGTGTTGACTGAGTCTGTGCTTGAAACCAGGCCAGAGCCATGGATTAGGAAGGGCAAAGAGAAGGC

ACCAGAATGAGTAAAGCAGGCAGGTGGTGAAGCCAACCATAAACTTCTCAGGAGTGACATGTGCTTCCTT

CAAAGGCATTTTTGTTAACCATATCCTTCTGAGTTCTATGTTTCCTTCACAGCTGTTCTATCCATTTTGT

GGACTGTCCCCCACCCCCACCCCATCATTGTTTTTAAAAAATTAAGGCCTGGCGCAGCAGCTCATGCCTA

TAATCCCAGCACTTTGGGAGGCTGAGGCGGGCGGATCACTTGAGGCCAGGAGTTTGAGACCAGCCCAGGC

AACATAGCAAAACCCCATTCTGCTTTAAAAAAAAAAAAAAAAAAAAATTAGCTTGGCGTAGTGGCATGTGC

CTATAATCCCAGCTACTGGGGAGGCTGAGGCACAAGAATCATTTGAACCTGGGAGGTAGAGGTTGCTGTG

AGCCGAGATTACGCCCCTGCACTCCAGCCTGGGTCACAGAGTGAGACTCCATCTCAGAAAAAAAAAAAAT

TGAGTCAGGTGCAGTAGCTCCTTCCTGTAGTCCCAGCTACTTGGGAGGCTGAGGCTAGAGGATCACTTGA

GCCCAGGAGTTTGAGTCTAGTCTGGGCAACATAGCAAGACCCCATCTCTAAAATTTAAGTAAGTAAAAGT

AGATAAATAAAAAGAAAAAAAAAACTGTTTATGTGCTCATCATAAAGTAGAAGAGTGGTTTGCTTTTTTTT

TTTTTTTTGGATTAATGAGGAAATCATTCTGTGGCTCTAGTCATAATTTATGCTTAATAACATTGATAGT

AGCCCTTTGCGCTATAACTCTACCTAAAGACTCACATCATTTGGCAGAGAGAGAGTCGTTGAAGTCCCAG

GAATTCAGGACTGGGCAGGTTAAGACCTCAGACAAGGTAGTAGAGGTAGACTTGTGGACAAGGCTCGGGT

CCCAGCCCACCGCACCCCAACTTTAATCAGAGTGGTTCACTATTGATCTATTTTTGTGTGATAGCTGTGT

GGCGTGGGCCACAACATTTAATGAGAAGTTACTGTGCACCAAACTGCCGAACACCATTCTAAACTATTCA

TATATATTAGTCATTTAATTCTTACATAACTTGAGAGGTAGACAGATATCCTTATTTTAGAGATGAGGAA

ACCAAGAGAACTTAGGTCATTAGCGCAAGGTTGTAGAGTAAGCGGCAAAGCCAAGACACAAAGCTGGGTG

GTTTGGTTTCAGAGCCAGTGCTTTTCCCCTCTACTGTACTGCCTCTCAACCAACACAGGGTTGCACAGGC

CCATTCTCTGATTTTTTTCCTCTTGTCCTCTGCCTCTCCCTCTAGCTCCCACTTCCTCTCTGCTCTAGTT

CATTTTCTTTAGAGCAGCCCGAGTGATCATGAAGTGCAAATCTTGCCATGTCAGTCCCCTGCTTAGAACC

CTCCAATGGCTCACTTTCTCTTTAGGCAAAAGTCTTTACCCCATGCCTTCTCCCATCTCATCTCAACCCC

CTCATTTGTTGGCTGTCTGCTGTCAGCCACTCTTCTTTCAGGTCCTCAGATGCACTGCACCCTCTCCTGC

CTGGGGGTCTTTGCTCCTGCTACTACCTCTGCTTGAACAGCTCCTCACCTTCCTTCCTCCAACCCTACCC

TTGTATAGGTGACTTTTGTTCATCCTTCAGAATTCAACTCACATGTCTCTTGCATGGAGAACCCTCACCT

ACTGTGTTGAGACCCTGTCCAGCCCCCAGGTGGGATCCTCTCTCGACTTCCCATACATTTCTTTCACAGC

ATTTACATAGTCCATGATAGTTTACTTGTGGGATTATTTGGTTAATCTTTGCCTTTAACACCAGGGTTCC

TTGGGTGAAGGAGCTTCTTTATCTTGGTAACAGCATTATTTCAAGCATAACTTGTAATATAGTTATATTA

CATATATAACATATATATATATATAACATAACATATATAACATATATAACAAGCATAACTTGTTATATAGTC

TTGTATATAGTAAGACCTCAATAAATATTTGGAGAACAAA

>NM_004281.4 Homo sapiens BAG cochaperone 3 (BAG3), mRNA

GCATCCAACCCCGGGCCGCGGCCAACTTCTCTGGACTGGACCAGAAGTTTCTAGCCGGCCAGTTGCTACC

TCCCTTTATCTCCTCCTTCCCCTCTGGCAGCGAGGAGGCTATTTCCAGACACTTCCACCCCTCTCTGGCC

ACGTCACCCCCGCCTTTAATTCATAAAGGTGCCCGGCGCCGGCTTCCCGGACACGTCGGCGGCGGAGAGG

GGCCCACGGCGGCGGCCCGGCCAGAGACTCGGCGCCCGGAGCCAGCGCCCCGCACCCGCGCCCCAGCGGG

CAGACCCCAACCCAGCATGAGCGCCGCCACCCACTCGCCCATGATGCAGGTGGCGTCCGGCAACGGTGAC

CGCGACCCTTTGCCCCCCGGATGGGAGATCAAGATCGACCCGCAGACCGGCTGGCCCTTCTTCGTGGACC

ACAACAGCCGCACCACTACGTGGAACGACCCGCGCGTGCCCTCTGAGGGCCCCAAGGAGACTCCATCCTC

TGCCAATGGCCCTTCCCGGGAGGGCTCTAGGCTGCCGCCTGCTAGGGAAGGCCACCCTGTGTACCCCCAG

CTCCGACCAGGCTACATTCCCATTCCTGTGCTCCATGAAGGCGCTGAGAACCGGCAGGTGCACCCTTTCC

ATGTCTATCCCCAGCCTGGGATGCAGCGATTCCGAACTGAGGCGGCAGCAGCGGCTCCTCAGAGGTCCCA

GTCACCTCTGCGGGGCATGCCAGAAACCACTCAGCCAGATAAACAGTGTGGACAGGTGGCAGCGGCGGCG

GCAGCCCAGCCCCCAGCCTCCCACGGACCTGAGCGGTCCCAGTCTCCAGCTGCCTCTGACTGCTCATCCT

CATCCTCCTCGGCCAGCCTGCCTTCCTCCGGCAGGAGCAGCCTGGGCAGTCACCAGCTCCCGCGGGGGTA

CATCTCCATTCCGGTGATACACGAGCAGAACGTTACCCGGCCAGCAGCCCAGCCCTCCTTCCACCAAGCC

CAGAAGACGCACTACCCAGCGCAGCAGGGGGAGTACCAGACCCACCAGCCTGTGTACCACAAGATCCAGG

GGGATGACTGGGAGCCCCGGCCCCTGCGGGCGGCATCCCCGTTCAGGTCATCTGTCCAGGGTGCATCGAG

CCGGGAGGGCTCACCAGCCAGGAGCAGCACGCCACTCCACTCCCCCTCGCCCATCCGTGTGCACACCGTG

GTCGACAGGCCTCAGCAGCCCATGACCCATCGAGAAACTGCACCTGTTTCCCAGCCTGAAAACAAACCAG

AAAGTAAGCCAGGCCCAGTTGGACCAGAACTCCCTCCTGGACACATCCCAATTCAAGTGATCCGCAAAGA

GGTGGATTCTAAACCTGTTTCCCAGAAGCCCCCACCTCCCTCTGAGAAGGTAGAGGTGAAAGTTCCCCCT

GCTCCAGTTCCTTGTCCTCCTCCCAGCCCTGGCCCTTCTGCTGTCCCCTCTTCCCCCAAGAGTGTGGCTA

CAGAAGAGAGGGCAGCCCCCAGCACTGCCCCTGCAGAAGCTACACCTCCAAAACCAGGAGAAGCCGAGGC

TCCCCCAAAACATCCAGGAGTGCTGAAAGTGGAAGCCATCCTGGAGAAGGTACAGGGGCTGGAGCAGGCT

GTAGACAACTTTGAAGGCAAGAAGACTGACAAAAAGTACCTGATGATCGAAGAGTATTTGACCAAAGAGC

TGCTGGCCCTGGATTCAGTGGACCCCGAGGGACGAGCCGATGTGCGTCAGGCCAGGAGAGACGGTGTCAG

GAAGGTTCAGACCATCTTGGAAAAACTTGAACAGAAAGCCATTGATGTCCCAGGTCAAGTCCAGGTCTAT

GAACTCCAGCCCAGCAACCTTGAAGCAGATCAGCCACTGCAGGCAATCATGGAGATGGGTGCCGTGGCAG

CAGACAAGGGCAAGAAAAATGCTGGAAATGCAGAAGATCCCCACACAGAAACCCAGCAGCCAGAAGCCAC

AGCAGCAGCGACTTCAAACCCCAGCAGCATGACAGACACCCCTGGTAACCCAGCAGCACCGTAGCCTCTG

CCCTGTAAAAATCAGACTCGGAACCGATGTGTGCTTTAGGGAATTTTAAGTTGCATGCATTTCAGAGACT

TTAAGTCAGTTGGTTTTTATTAGCTGCTTGGTATGCAGTAACTTGGGTGGAGGCAAAACACTAATAAAAG

GGCTAAAAAGGAAAATGATGCTTTTCTTCTATATTCTTACTCTGTACAAATAAAGAAGTTGCTTGTTGTT

TGAGAAGTTTAACCCCGTTGCTTGTTGTTCTGCAGCCCTGTCTACTTGGGCACCCCCACCACCTGTTAGC

TGTGGTTGTGCACTGTCTTTTGTAGCTCTGGACTGGAGGGGTAGATGGGGAGTCAATTACCCATCACATA

AATATGAAACATTTATCAGAAATGTTGCCATTTTAATGAGATGATTTTCTTCATCTCATAATTAAAATAC

CTGACTTTAGAGAGAGTAAAATGTGCCAGGAGCCATAGGAATATCTGTATGTTGGATGACTTTAATGCTA

CATTTTAAAAAAAGAAAATAAAGTAATAATATAACTCAAAA

>NM_001188.4 Homo sapiens BCL2 antagonist/killer 1 (BAK1), mRNA

ACAGAGCAACTTCCTCTAGAGGGAGCTGATTGGAGCCGGGTGCCGCTGGCACCTCTATGATCACTGGAGT

CTCGCGGGTCCCTCGGGCTGCACAGGGACAAGTAAAGGCTACATCCAGATGCCGGGAATGCACTGACGCC

CATTCCTGGAAACTGGGCTCCCACTCAGCCCCTGGGAGCAGCAGCCGCCAGCCCCTCGGGACCTCCATCT

CCACCCTGCTGAGCCACCCGGGTTGGGCCAGGATCCCGGCAGGCTGATCCCGTCCTCCACTGAGACCTGA

AAAATGGCTTCGGGGCAAGGCCCAGGTCCTCCCAGGCAGGAGTGCGGAGAGCCTGCCCTGCCCTCTGCTT

CTGAGGAGCAGGTAGCCCAGGACACAGAGGAGGTTTTCCGCAGCTACGTTTTTTACCGCCATCAGCAGGA

ACAGGAGGCTGAAGGGGTGGCTGCCCCTGCCGACCCAGAGATGGTCACCTTACCTCTGCAACCTAGCAGC

ACCATGGGGCAGGTGGGACGGCAGCTCGCCATCATCGGGGACGACATCAACCGACGCTATGACTCAGAGT

TCCAGACCATGTTGCAGCACCTGCAGCCCACGGCAGAGAATGCCTATGAGTACTTCACCAAGATTGCCAC

CAGCCTGTTTGAGAGTGGCATCAATTGGGGCCGTGTGGTGGCTCTTCTGGGCTTCGGCTACCGTCTGGCC

CTACACGTCTACCAGCATGGCCTGACTGGCTTCCTAGGCCAGGTGACCCGCTTCGTGGTCGACTTCATGC

TGCATCACTGCATTGCCCGGTGGATTGCACAGAGGGGTGGCTGGGTGGCAGCCCTGAACTTGGGCAATGG

TCCCATCCTGAACGTGCTGGTGGTTCTGGGTGTGGTTCTGTTGGGCCAGTTTGTGGTACGAAGATTCTTC

AAATCATGACTCCCAAGGGTGCCCTTTGGGGTCCCGGTTCAGACCCCTGCCTGGACTTAAGCGAAGTCTT

TGCCTTCTCTGTTCCCTTGCAGGGGTCCCCCCTCAAGAGTACAGAAGCTTTAGCAAGTGTGCACTCCAGC

TTCGGAGGGCCCCTGCGTGGGGGCCAGTCAGGCTGCAGAGGCACCTCAACATTGCATGGTGCTAGTGGGC

CCTCTCTCTGGGCCCAGGGGCTGTGGCCGTCTCCTCCCTCAGCTCTCTGGGACCTCCTTAGCCCTGTCTG

CTAGGCGCTGGGGAGACTGATAACTTGGGGAGGCAAGAGACTGGGAGCCACTTCTCCCCAGAAAGTGTTT

AACGGTTTTAGCTTTTTATAATACCCTTGTGAGAGCCCATTCCCACCATTCTACCTGAGGCCAGGACGTC

TGGGGTGTGGGGATTGGTGGGTCTATGTTCCCCAGGATTCAGCTATTCTGGAAGATCAGCACCCTAAGAG

ATGGGACTAGGACCTGAGCCTGGTCCTGGCCGTCCCTAAGCATGTGTCCCAGGAGCAGGACCTACTAGGA

GAGGGGGGCCAAGGTCCTGCTCAACTCTACCCCTGCTCCCATTCCTCCCTCCGGCCATACTGCCTTTGCA

GTTGGACTCTCAGGGATTCTGGGCTTGGGGTGTGGGGTGGGGTGGAGTCGCAGACCAGAGCTGTCTGAAC

TCACGTGTCAGAAGCCTCCAAGCCTGCCTCCCAAGGTCCTCTCAGTTCTCTCCCTTCCTCTCTCCTTATA

GACACTTGCTCCCAACCCATTCACTACAGGTGAAGGCTCTCACCCCCATCCCTGGGGGCCTTGGGTGAGT

GGCCTGCTAAGGCTCCTCCTTGCCCAGACTACAGGGCTTAGGACTTGGTTTGTTATATCAGGGAAAAGGA

GTAGGGAGTTCATCTGGAGGGTTCTAAGTGGGAGAAGGACTATCAACACCACTAGGAATCCCAGAGGTGG

GATCCTCCCTCATGGCTCTGGCACAGTGTAATCCAGGGGTGTAGATGGGGGAACTGTGAATACTTGAACT

CTGTTCCCCCACCCTCCATGCTCCTCACCTGTCTAGGTCTCCTCAGGGTGGGGGGTGACAGTGCCTTCTC

TATTGGGCACAGCCTAGGGTCTTGGGGGTCAGGGGGGAGAAGTTCTTGATTCAGCCAAATGCAGGGAGGG

GAGGCAGATGGAGCCCATAGGCCACCCCCTATCCTCTGAGTGTTTGGAAATAAACTGTGCAATCCCCTCA

>NM_138763.4 Homo sapiens BCL2 associated X, apoptosis regulator (BAX), transcript variant delta, mRNA

GCGCTGCGGCCGCCCGCGCGGACCCGGCGAGAGGCGGCGGCGGGAGCGGCGGTGATGGACGGGTCCGGGG

AGCAGCCCAGAGGCGGGGGGCCCACCAGCTCTGAGCAGATCATGAAGACAGGGGCCCTTTTGCTTCAGGG

GATGATTGCCGCCGTGGACACAGACTCCCCCCGAGAGGTCTTTTTCCGAGTGGCAGCTGACATGTTTTCT

GACGGCAACTTCAACTGGGGCCGGGTTGTCGCCCTTTTCTACTTTGCCAGCAAACTGGTGCTCAAGGCCC

TGTGCACCAAGGTGCCGGAACTGATCAGAACCATCATGGGCTGGACATTGGACTTCCTCCGGGAGCGGCT

GTTGGGCTGGATCCAAGACCAGGGTGGTTGGGACGGCCTCCTCTCCTACTTTGGGACGCCCACGTGGCAG

ACCGTGACCATCTTTGTGGCGGGAGTGCTCACCGCCTCACTCACCATCTGGAAGAAGATGGGCTGAGGCC

CCCAGCTGCCTTGGACTGTGTTTTTCCTCCATAAATTATGGCATTTTTCTGGGAGGGGTGGGGATTGGGG

GACGTGGGCATTTTTCTTACTTTTGTAATTATTGGGGGGTGTGGGGAAGAGTGGTCTTGAGGGGGTAATA

AACCTCCTTCGGGACACA

>NM_000633.3 Homo sapiens BCL2 apoptosis regulator (BCL2), transcript variant alpha, mRNA

ACCCCTCGCCGCACCACACACAGCGCGGGCTTCTAGCGCTCGGCACCGGCGGGCCAGGCGCGTCCTGCCT

TCATTTATCCAGCAGCTTTTCGGAAAATGCATTTGCTGTTCGGAGTTTAATCAGAAGAGGATTCCTGCCT

CCGTCCCCGGCTCCTTCATCGTCCCCTCTCCCCTGTCTCTCTCCTGGGGAGGCGTGAAGCGGTCCCGTGG

ATAGAGATTCATGCCTGTGCCCGCGCGTGTGTGCGCGCGTGTAAATTGCCGAGAAGGGGAAAACATCACA

GGACTTCTGCGAATACCGGACTGAAAATTGTAATTCATCTGCCGCCGCCGCTGCCTTTTTTTTTTCTCGA

GCTCTTGAGATCTCCGGTTGGGATTCCTGCGGATTGACATTTCTGTGAAGCAGAAGTCTGGGAATCGATC

TGGAAATCCTCCTAATTTTTACTCCCTCTCCCCGCGACTCCTGATTCATTGGGAAGTTTCAAATCAGCTA

TAACTGGAGAGTGCTGAAGATTGATGGGATCGTTGCCTTATGCATTTGTTTTGGTTTTACAAAAAGGAAA

CTTGACAGAGGATCATGCTGTACTTAAAAAAATACAACATCACAGAGGAAGTAGACTGATATTAACAATAC

TTACTAATAATAACGTGCCTCATGAAATAAAGATCCGAAAGGAATTGGAATAAAAATTTCCTGCATCTCA

TGCCAAGGGGGAAACACCAGAATCAAGTGTTCCGCGTGATTGAAGACACCCCCTCGTCCAAGAATGCAAA

GCACATCCAATAAAATAGCTGGATTATAACTCCTCTTCTTTCTCTGGGGGCCGTGGGGTGGGAGCTGGGG

CGAGAGGTGCCGTTGGCCCCCGTTGCTTTTCCTCTGGGAAGGATGGCGCACGCTGGGAGAACAGGGTACG

ATAACCGGGAGATAGTGATGAAGTACATCCATTATAAGCTGTCGCAGAGGGGCTACGAGTGGGATGCGGG

AGATGTGGGCGCCGCGCCCCCGGGGGCCGCCCCCGCACCGGGCATCTTCTCCTCCCAGCCCGGGCACACG

CCCCATCCAGCCGCATCCCGGGACCCGGTCGCCAGGACCTCGCCGCTGCAGACCCCGGCTGCCCCCGGCG

CCGCCGCGGGGCCTGCGCTCAGCCCGGTGCCACCTGTGGTCCACCTGACCCTCCGCCAGGCCGGCGACGA

CTTCTCCCGCCGCTACCGCCGCGACTTCGCCGAGATGTCCAGCCAGCTGCACCTGACGCCCTTCACCGCG

CGGGGACGCTTTGCCACGGTGGTGGAGGAGCTCTTCAGGGACGGGGTGAACTGGGGGAGGATTGTGGCCT

TCTTTGAGTTCGGTGGGGTCATGTGTGTGGAGAGCGTCAACCGGGAGATGTCGCCCCTGGTGGACAACAT

CGCCCTGTGGATGACTGAGTACCTGAACCGGCACCTGCACACCTGGATCCAGGATAACGGAGGCTGGGAT

GCCTTTGTGGAACTGTACGGCCCCAGCATGCGGCCTCTGTTTGATTTCTCCTGGCTGTCTCTGAAGACTC

TGCTCAGTTTGGCCCTGGTGGGAGCTTGCATCACCCTGGGTGCCTATCTGGGCCACAAGTGAAGTCAACA

TGCCTGCCCCAAACAAATATGCAAAAGGTTCACTAAAGCAGTAGAAATAATATGCATTGTCAGTGATGTA

CCATGAAACAAAGCTGCAGGCTGTTTAAGAAAAAATAACACACATATAAACATCACACACACAGACAGAC

ACACACACACACAACAATTAACAGTCTTCAGGCAAAACGTCGAATCAGCTATTTACTGCCAAAGGGAAAT

ATCATTTATTTTTTACATTATTAAGAAAAAAAGATTTATTTATTTAAGACAGTCCCATCAAAACTCCTGT

CTTTGGAAATCCGACCACTAATTGCCAAGCACCGCTTCGTGTGGCTCCACCTGGATGTTCTGTGCCTGTA

AACATAGATTCGCTTTCCATGTTGTTGGCCGGATCACCATCTGAAGAGCAGACGGATGGAAAAAGGACCT

GATCATTGGGGAAGCTGGCTTTCTGGCTGCTGGAGGCTGGGGAGAAGGTGTTCATTCACTTGCATTTCTT

TGCCCTGGGGGCTGTGATATTAACAGAGGGAGGGTTCCTGTGGGGGGAAGTCCATGCCTCCCTGGCCTGA

AGAAGAGACTCTTTGCATATGACTCACATGATGCATACCTGGTGGGAGGAAAAGAGTTGGGAACTTCAGA

TGGACCTAGTACCCACTGAGATTTCCACGCCGAAGGACAGCGATGGGAAAAATGCCCTTAAATCATAGGA

AAGTATTTTTTTAAGCTACCAATTGTGCCGAGAAAAGCATTTTAGCAATTTATACAATATCATCCAGTAC

CTTAAGCCCTGATTGTGTATATTCATATATTTTGGATACGCACCCCCCAACTCCCAATACTGGCTCTGTC

TGAGTAAGAAACAGAATCCTCTGGAACTTGAGGAAGTGAACATTTCGGTGACTTCCGCATCAGGAAGGCT

AGAGTTACCCAGAGCATCAGGCCGCCACAAGTGCCTGCTTTTAGGAGACCGAAGTCCGCAGAACCTGCCT

GTGTCCCAGCTTGGAGGCCTGGTCCTGGAACTGAGCCGGGGCCCTCACTGGCCTCCTCCAGGGATGATCA

ACAGGGCAGTGTGGTCTCCGAATGTCTGGAAGCTGATGGAGCTCAGAATTCCACTGTCAAGAAAGAGCAG

TAGAGGGGTGTGGCTGGGCCTGTCACCCTGGGGCCCTCCAGGTAGGCCCGTTTTCACGTGGAGCATGGGA

GCCACGACCCTTCTTAAGACATGTATCACTGTAGAGGGAAGGAACAGAGGCCCTGGGCCCTTCCTATCAG

AAGGACATGGTGAAGGCTGGGAACGTGAGGAGAGGCAATGGCCACGGCCCATTTTGGCTGTAGCACATGG

CACGTTGGCTGTGTGGCCTTGGCCCACCTGTGAGTTTAAAGCAAGGCTTTAAATGACTTTGGAGAGGGTC

ACAAATCCTAAAAGAAGCATTGAAGTGAGGTGTCATGGATTAATTGACCCCTGTCTATGGAATTACATGT

AAAACATTATCTTGTCACTGTAGTTTGGTTTTATTTGAAAACCTGACAAAAAAAAAGTTCCAGGTGTGGA

ATATGGGGGTTATCTGTACATCCTGGGGCATTAAAAAAAAAAATCAATGGTGGGGAACTATAAAGAAGTAA

CAAAAGAAGTGACATCTTCAGCAAATAAACTAGGAAATTTTTTTTTCTTCCAGTTTAGAATCAGCCTTGA

AACATTGATGGAATAACTCTGTGGCATTATTGCATTATATACCATTTATCTGTATTAACTTTGGAATGTA

CTCTGTTCAATGTTTAATGCTGTGGTTGATATTTCGAAAGCTGCTTTAAAAAAATACATGCATCTCAGCG

TTTTTTTGTTTTTAATTGTATTTAGTTATGGCCTATACACTATTTGTGAGCAAAGGTGATCGTTTTCTGT

TTGAGATTTTTATCTCTTGATTCTTCAAAAGCATTCTGAGAAGGTGAGATAAGCCCTGAGTCTCAGCTAC

CTAAGAAAAACCTGGATGTCACTGGCCACTGAGGAGCTTTGTTTCAACCAAGTCATGTGCATTTCCACGT

CAACAGAATTGTTTATTGTGACAGTTATATCTGTTGTCCCTTTGACCTTGTTTCTTGAAGGTTTCCTCGT

CCCTGGGCAATTCCGCATTTAATTCATGGTATTCAGGATTACATGCATGTTTGGTTAAACCCATGAGATT

CATTCAGTTAAAAATCCAGATGGCAAATGACCAGCAGATTCAAATCTATGGTGGTTTGACCTTTAGAGAG

TTGCTTTACGTGGCCTGTTTCAACACAGACCCACCCAGAGCCCTCCTGCCCTCCTTCCGCGGGGGCTTTC

TCATGGCTGTCCTTCAGGGTCTTCCTGAAATGCAGTGGTGCTTACGCTCCACCAAGAAAGCAGGAAACCT

GTGGTATGAAGCCAGACCTCCCCGGCGGGCCTCAGGGAACAGAATGATCAGACCTTTGAATGATTCTAAT

TTTTAAGCAAAATATTATTTTATGAAAGGTTTACATTGTCAAAGTGATGAATATGGAATATCCAATCCTG

TGCTGCTATCCTGCCAAAATCATTTTAATGGAGTCAGTTTGCAGTATGCTCCACGTGGTAAGATCCTCCA

AGCTGCTTTAGAAGTAACAATGAAGAACGTGGACGTTTTTAATATAAAGCCTGTTTTGTCTTTTGTTGTT

GTTCAAACGGGATTCACAGAGTATTTGAAAAATGTATATATATTAAGAGGTCACGGGGGCTAATTGCTGG

CTGGCTGCCTTTTGCTGTGGGGTTTTGTTACCTGGTTTTAATAACAGTAAATGTGCCCAGCCTCTTGGCC

CCAGAACTGTACAGTATTGTGGCTGCACTTGCTCTAAGAGTAGTTGATGTTGCATTTTCCTTATTGTTAA

AAACATGTTAGAAGCAATGAATGTATATAAAAGCCTCAACTAGTCATTTTTTTCTCCTCTTCTTTTTTTT

CATTATATCTAATTATTTTGCAGTTGGGCAACAGAGAACCATCCCTATTTTGTATTGAAGAGGGATTCAC

ATCTGCATCTTAACTGCTCTTTATGAATGAAAAAACAGTCCTCTGTATGTACTCCTCTTTACACTGGCCA

GGGTCAGAGTTAAATAGAGTATATGCACTTTCCAAATTGGGGACAAGGGCTCTAAAAAAAGCCCCAAAAG

GAGAAGAACATCTGAGAACCTCCTCGGCCCTCCCAGTCCCTCGCTGCACAAATACTCCGCAAGAGAGGCC

AGAATGACAGCTGACAGGGTCTATGGCCATCGGGTCGTCTCCGAAGATTTGGCAGGGGCAGAAAACTCTG

GCAGGCTTAAGATTTGGAATAAAGTCACAGAATTAAGGAAGCACCTCAATTTAGTTCAAACAAGACGCCA

ACATTCTCTCCACAGCTCACTTACCTCTCTGTGTTCAGATGTGGCCTTCCATTTATATGTGATCTTTGTT

TTATTAGTAAATGCTTATCATCTAAAGATGTAGCTCTGGCCCAGTGGGAAAAATTAGGAAGTGATTATAA

ATCGAGAGGAGTTATAATAATCAAGATTAAATGTAAATAATCAGGGCAATCCCAACACATGTCTAGCTTT

CACCTCCAGGATCTATTGAGTGAACAGAATTGCAAATAGTCTCTATTTGTAATTGAACTTATCCTAAAAC

AAATAGTTTATAAATGTGAACTTAAACTCTAATTAATTCCAACTGTACTTTTAAGGCAGTGGCTGTTTTT

AGACTTTCTTATCACTTATAGTTAGTAATGTACACCTACTCTATCAGAGAAAAACAGGAAAGGCTCGAAA

TACAAGCCATTCTAAGGAAATTAGGGAGTCAGTTGAAATTCTATTCTGATCTTATTCTGTGGTGTCTTTT

GCAGCCCAGACAAATGTGGTTACACACTTTTTAAGAAATACAATTCTACATTGTCAAGCTTATGAAGGTT

CCAATCAGATCTTTATTGTTATTCAATTTGGATCTTTCAGGGATTTTTTTTTAAATTATTATGGGACAA

AGGACATTTGTTGGAGGGGTGGGAGGGAGGAAGAATTTTTAAATGTAAAACATTCCCAAGTTTGGATCAG

GGAGTTGGAAGTTTTCAGAATAACCAGAACTAAGGGTATGAAGGACCTGTATTGGGGTCGATGTGATGCC

TCTGCGAAGAACCTTGTGTGACAAATGAGAAACATTTTGAAGTTTGTGGTACGACCTTTAGATTCCAGAG

ACATCAGCATGGCTCAAAGTGCAGCTCCGTTTGGCAGTGCAATGGTATAAATTTCAAGCTGGATATGTCT

AATGGGTATTTAAACAATAAATGTGCAGTTTTAACTAACAGGATATTTAATGACAACCTTCTGGTTGGTA

GGGACATCTGTTTCTAAATGTTTATTATGTACAATACAGAAAAAAATTTTATAAAATTAAGCAATGTGAA

ACTGAATTGGAGAGTGATAATACAAGTCCTTTAGTCTTACCCAGTGAATCATTCTGTTCCATGTCTTTGG

ACAACCATGACCTTGGACAATCATGAAATATGCATCTCACTGGATGCAAAGAAAATCAGATGGAGCATGA

ATGGTACTGTACCGGTTCATCTGGACTGCCCCAGAAAAATAACTTCAAGCAAACATCCTATCAACAACAA

GGTTGTTCTGCATACCAAGCTGAGCACAGAAGATGGGAACACTGGTGGAGGATGGAAAGGCTCGCTCAAT

CAAGAAAATTCTGAGACTATTAATAAATAAGACTGTAGTGTAGATACTGAGTAAATCCATGCACCTAAAC

CTTTTGGAAAATCTGCCGTGGGCCCTCCAGATAGCTCATTTCATTAAGTTTTTCCCTCCAAGGTAGAATT

TGCAAGAGTGACAGTGGATTGCATTTCTTTTGGGGAAGCTTTCTTTTGGTGGTTTTGTTTATTATACCTT

CTTAAGTTTTCAACCAAGGTTTGCTTTTGTTTTGAGTTACTGGGGTTATTTTTGTTTTAAATAAAAATAA

GTGTACAATAAGTGTTTTTGTATTGAAAGCTTTTGTTATCAAGATTTTCATACTTTTACCTTCCATGGCT

CTTTTTAAGATTGATACTTTTAAGAGGTGGCTGATATTCTGCAACACTGTACACATAAAAAATACGGTAA

GGATACTTTACATGGTTAAGGTAAAGTAAGTCTCCAGTTGGCCACCATTAGCTATAATGGCACTTTGTTT

GTGTTGTTGGAAAAAGTCACATTGCCATTAAACTTTCCTTGTCTGTCTAGTTAATATTGTGAAGAAAAAT

AAAGTACAGTGTGAGATACTG

>NM_138578.3 Homo sapiens BCL2 like 1 (BCL2L1), transcript variant 1, mRNA

CTCGATCCGGGCGATGGAGGAGGAAGCAAGCGAGGGGGCTGGTTCCTGAGCTTCGCAATTCCTGTGTCGC

CTTCTGGGCTCCCAGCCTGCCGGGTCGCATGATCCCTCCGGCCGGAGCTGGTTTTTTTGCCAGCCACCGC

GAGGCCGGCTGAGTTACCGGCATCCCCGCAGCCACCTCCTCTCCCGACCTGTGATACAAAAGATCTTCCG

GGGGCTGCACCTGCCTGCCTTTGCCTAAGGCGGATTTGAATCTCTTTCTCTCCCTTCAGAATCTTATCTT

GGCTTTGGATCTTAGAAGAGAATCACTAACCAGAGACGAGACTCAGTGAGTGAGCAGGTGTTTTGGACAA

TGGACTGGTTGAGCCCATCCCTATTATAAAAATGTCTCAGAGCAACCGGGAGCTGGTGGTTGACTTTCTC

TCCTACAAGCTTTCCCAGAAAGGATACAGCTGGAGTCAGTTTAGTGATGTGGAAGAGAACAGGACTGAGG

CCCCAGAAGGGACTGAATCGGAGATGGAGACCCCCAGTGCCATCAATGGCAACCCATCCTGGCACCTGGC

AGACAGCCCCGCGGTGAATGGAGCCACTGGCCACAGCAGCAGTTTGGATGCCCGGGAGGTGATCCCCATG

GCAGCAGTAAAGCAAGCGCTGAGGGAGGCAGGCGACGAGTTTGAACTGCGGTACCGGCGGGCATTCAGTG

ACCTGACATCCCAGCTCCACATCACCCCAGGGACAGCATATCAGAGCTTTGAACAGGTAGTGAATGAACT

CTTCCGGGATGGGGTAAACTGGGGTCGCATTGTGGCCTTTTTCTCCTTCGGCGGGGCACTGTGCGTGGAA

AGCGTAGACAAGGAGATGCAGGTATTGGTGAGTCGGATCGCAGCTTGGATGGCCACTTACCTGAATGACC

ACCTAGAGCCTTGGATCCAGGAGAACGGCGGCTGGGATACTTTTGTGGAACTCTATGGGAACAATGCAGC

AGCCGAGAGCCGAAAGGGCCAGGAACGCTTCAACCGCTGGTTCCTGACGGGCATGACTGTGGCCGGCGTG

GTTCTGCTGGGCTCACTCTTCAGTCGGAAATGACCAGACACTGACCATCCACTCTACCCTCCCACCCCCT

TCTCTGCTCCACCACATCCTCCGTCCAGCCGCCATTGCCACCAGGAGAACCACTACATGCAGCCCATGCC

CACCTGCCCATCACAGGGTTGGGCCCAGATCTGGTCCCTTGCAGCTAGTTTTCTAGAATTTATCACACTT

CTGTGAGACCCCCACACCTCAGTTCCCTTGGCCTCAGAATTCACAAAATTTCCACAAAATCTGTCCAAAG

GAGGCTGGCAGGTATGGAAGGGTTTGTGGCTGGGGGCAGGAGGGCCCTACCTGATTGGTGCAACCCTTAC

CCCTTAGCCTCCCTGAAAATGTTTTTCTGCCAGGGAGCTTGAAAGTTTTCAGAACCTCTTCCCCAGAAAG

GAGACTAGATTGCCTTTGTTTTGATGTTTGTGGCCTCAGAATTGATCATTTTCCCCCCACTCTCCCCACA

CTAACCTGGGTTCCCTTTCCTTCCATCCCTACCCCCTAAGAGCCATTTAGGGGCCACTTTTGACTAGGGA

TTCAGGCTGCTTGGGATAAAGATGCAAGGACCAGGACTCCCTCCTCACCTCTGGACTGGCTAGAGTCCTC

ACTCCCAGTCCAAATGTCCTCCAGAAGCCTCTGGCTAGAGGCCAGCCCCACCCAGGAGGGAGGGGGCTAT

AGCTACAGGAAGCACCCCATGCCAAAGCTAGGGTGGCCCTTGCAGTTCAGCACCACCCTAGTCCCTTCCC

CTCCCTGGCTCCCATGACCATACTGAGGGACCAACTGGGCCCAAGACAGATGCCCCAGAGCTGTTTATGG

CCTCAGCTGCCTCACTTCCTACAAGAGCAGCCTGTGGCATCTTTGCCTTGGGCTGCTCCTCATGGTGGGT

TCAGGGGACTCAGCCCTGAGGTGAAAGGGAGCTATCAGGAACAGCTATGGGAGCCCCAGGGTCTTCCCTA

CCTCAGGCAGGAAGGGCAGGAAGGAGAGCCTGCTGCATGGGGTGGGGTAGGGCTGACTAGAAGGGCCAGT

CCTGCCTGGCCAGGCAGATCTGTGCCCCATGCCTGTCCAGCCTGGGCAGCCAGGCTGCCAAGGCCAGAGT

GGCCTGGCCAGGAGCTCTTCAGGCCTCCCTCTCTCTTCTGCTCCACCCTTGGCCTGTCTCATCCCCAGGG

GTCCCAGCCACCCCGGGCTCTCTGCTGTACATATTTGAGACTAGTTTTTATTCCTTGTGAAGATGATATA

CTATTTTTGTTAAGCGTGTCTGTATTTATGTGTGAGGAGCTGCTGGCTTGCAGTGCGCGTGCACGTGGAG

AGCTGGTGCCCGGAGATTGGACGGCCTGATGCTCCCTCCCCTGCCCTGGTCCAGGGAAGCTGGCCGAGGG

TCCTGGCTCCTGAGGGGCATCTGCCCCTCCCCCAACCCCCACCCCACACTTGTTCCAGCTCTTTGAAATA

GTCTGTGTGAAGGTGAAAGTGCAGTTCAGTAATAAACTGTGTTTACTCAGTGAA

>NM_003766.5 Homo sapiens beclin 1 (BECN1), transcript variant 1, mRNA

CGGCGGCTACCGGGAAGTCGCTGAAGACAGAGCGATGGTAGTTCTGGAGGCCTCGCTCCGGGGCCGACCC

GAGGCCACAGTGCCTCCGCGGTAGACCGGACTTGGGTGACGGGCTCCGGGCTCCCGAGGTGAAGAGCATC

GGGGGCTGAGGGATGGAAGGGTCTAAGACGTCCAACAACAGCACCATGCAGGTGAGCTTCGTGTGCCAGC

GCTGCAGCCAGCCCCTGAAACTGGACACGAGTTTCAAGATCCTGGACCGTGTCACCATCCAGGAACTCAC

AGCTCCATTACTTACCACAGCCCAGGCGAAACCAGGAGAGACCCAGGAGGAAGAGACTAACTCAGGAGAG

GAGCCATTTATTGAAACTCCTCGCCAGGATGGTGTCTCTCGCAGATTCATCCCCCCAGCCAGGATGATGT

CCACAGAAAGTGCCAACAGCTTCACTCTGATTGGGGAGGCATCTGATGGCGGCACCATGGAGAACCTCAG

CCGAAGACTGAAGGTCACTGGGGACCTTTTTGACATCATGTCGGGCCAGACAGATGTGGATCACCCACTC

TGTGAGGAATGCACAGATACTCTTTTAGACCAGCTGGACACTCAGCTCAACGTCACTGAAAATGAGTGTC

AGAACTACAAACGCTGTTTGGAGATCTTAGAGCAAATGAATGAGGATGACAGTGAACAGTTACAGATGGA

GCTAAAGGAGCTGGCACTAGAGGAGGAGAGGCTGATCCAGGAGCTGGAAGACGTGGAAAAGAACCGCAAG

ATAGTGGCAGAAAATCTCGAGAAGGTCCAGGCTGAGGCTGAGAGACTGGATCAGGAGGAAGCTCAGTATC

AGAGAGAATACAGTGAATTTAAACGACAGCAGCTGGAGCTGGATGATGAGCTGAAGAGTGTTGAAAACCA

GATGCGTTATGCCCAGACGCAGCTGGATAAGCTGAAGAAAACCAACGTCTTTAATGCAACCTTCCACATC

TGGCACAGTGGACAGTTTGGCACAATCAATAACTTCAGGCTGGGTCGCCTGCCCAGTGTTCCCGTGGAAT

GGAATGAGATTAATGCTGCTTGGGGCCAGACTGTGTTGCTGCTCCATGCTCTGGCCAATAAGATGGGTCT

GAAATTTCAGAGATACCGACTTGTTCCTTACGGAAACCATTCATATCTGGAGTCTCTGACAGACAAATCT

AAGGAGCTGCCGTTATACTGTTCTGGGGGGTTGCGGTTTTTCTGGGACAACAAGTTTGACCATGCAATGG

TGGCTTTCCTGGACTGTGTGCAGCAGTTCAAAGAAGAGGTTGAGAAAGGCGAGACACGTTTTTGTCTTCC

CTACAGGATGGATGTGGAGAAAGGCAAGATTGAAGACACAGGAGGCAGTGGCGGCTCCTATTCCATCAAA

ACCCAGTTTAACTCTGAGGAGCAGTGGACAAAAGCTCTCAAGTTCATGCTGACGAATCTTAAGTGGGGTC

TTGCTTGGGTGTCCTCACAATTTTATAACAAATGACTTTTTTCCTTAGGGGGAGGTTTGCCTTAAAGGCT

TTTAATTTTGTTTTGTTTGCAAACATGTTTTAAATTAAATTCGGGTAATATTAAACAGTACATGTTTACA

ATACCAAAAAAGAAAAAATCCACAAAAGCCACTTTATTTTAAAATATCATGTGACAGATACTTTCCAGAG

CTACAACATGCCATCTATAGTTGCCAGCCCTGGTCAGTTTTGATTCTTAACCCCATGGACTCCTTTCCCT

TTCTTCTCTGAAAAAAACTAATTTAAATTTGCTTTTCTTTTTTTTAACTGAGTTGAATTGAGATTGATGT

GTTTTCACTGGATTTTTATCTCTCTCAACTTCCTGCACTTAACAATATGAAATAGAAACTTTTGTCTTTA

CTGAGATGAGGATATGTTTGAGATGCACAGTTGGATAATGTGGGAAAATGACATCTAAGCTTTACCTGGT

CACCATGTGATGTGATCAGATGCTTGAAATTTAACACTTTTCACTTGGTTCTTATACTGAATGCCGACTC

TGCTCTGTGTTAGAGATATGAAATGGTGTTTGATACTGTTTGAGACATTATGGAGAGATTTAATTATTTG

TAATAAAAGATTTGCTGCAGTCTGAAAACTG

>NM_001290693.1 Homo sapiens beclin 2 (BECN2), mRNA

ATGTCTTCCATCCGCTTCCTGTGCCAGCGCTGCCACCAGGCCCTGAAGCTGAGCGGCTCCTCGGAGTCTA

GGAGCCTCCCTGCAGCCCCGGCGCCCACCTCTGGGCAGGCTGAGCCCGGAGACACCCGGGAGCCCGGCGT

CACCACCAGGGAGGTGACAGACGCTGAGGAGCAACAGGACGGTGCCTCTAGCAGATCCCCTCCAGGCGAT

GGCAGTGTGTCCAAGGGCCATGCCAACATCTTCACCCTGCTGGGGGAGCTTGGCGCCATGCACATGCTCA

GTAGCATCCAGAAGGCAGCTGGTGACATTTTTGACATAGTCTCTGGCCAAGCAGTTGTGGACCATCCCCT

GTGTGAAGAATGCACCGACAGTCTTTTAGAGCAGCTGGACATCCAGCTCGCTCTCACAGAAGCTGACAGT

CAGAACTACCAACGCTGCCTGGAGACCGGGGAGCTGGCGACCAGCGAGGACGAGGCGGCGGCGCTGCGGG

CGGAGCTGCGGGACCTGGAGCTGGAGGAGGCCAGGCTGGTGCAGGAGCTGGAGGATGTGGACAGGAACAA

TGCAAGAGCAGCGGCGGATCTCCAGGCAGCCCAGGCAGAGGCTGCGGAGCTGGACCAGCAGGAGAGGCAG

CACTACAGGGACTACAGTGCCTTGAAGCGGCAGCAGCTGGAACTGCTTGATCAGCTGGGGAACGTGGAGA

ACCAGCTGCAGTATGCCAGGGTCCAGAGGGACCGGCTGAAGGAAATCAACTGTTTCACCGCCACGTTTGA

GATCTGGGTGGAGGGCCCCTTGGGCGTCATCAATAACTTCAGGTTGGGCCGCCTCCCCACTGTCCGTGTG

GGCTGGAATGAGATTAACACTGCCTGGGGACAGGCGGCCTTGCTGCTCCTTACCCTGGCCAATACAATTG

GACTGCAGTTTCAGAGGTATCGACTCATCCCCTGCGGAAACCATTCGTATCTGAAGTCTTTAACAGATGA

CCGCACTGAGCTGCCGTTGTTCTGTTATGGGGGGCAGGATGTTTTCCTCAATAACAAGTATGACCGCGCG

ATGGTGGCCTTCCTGGACTGCATGCAGCAGTTCAAGGAAGAGGCTGAGAAGGGTGAGCTGGGCCTCTCTC

TGCCCTACGGGATCCAGGTGGAGACAGGCCTGATGGAGGACGTTGGCGGCCGAGGGGAATGCTATTCCAT

CAGAACCCATCTGAACACGCAGGAGCTGTGGACAAAGGCACTCAAGTTCATGCTTATAAATTTCAAGTGG

AGTCTCATCTGGGTTGCCTCAAGGTATCAAAAGTAG

>NM_197966.3 Homo sapiens BH3 interacting domain death agonist (BID), transcript variant 1, mRNA

ACCCTTGCCGGCGGATCGGAATCCCCGCCCACACCGTGGTCTTTCCAGCACCGCAGACACCTGCCGGCTC

CTCCCGAGCGGAGCTCAGGGCTGACAAAGCGCGGTCAGAGCGGCCGCTTACTGGGGCTCGCCCGCTCCTT

AGAGCACTGGCAATGATGTGCGGATCCTCGCTGCTGCTGCTGGGAAACTGTTGAGTGGCTGAATGACCCC

AGGGGACCCTGGGAGAGCTCTGAAGCCCTCAGCCACCAAGTGGCTGGGCTGGCAAGGGTTCATTCATTCA

TTCAACAAATACGAATGTGCAGCGGTGCTGGGGTCATGATGGCTCGGTGGGCAGCGAGGGGCCGGGCCGG

CTGGAGGAGCACAGTGCGGATTCTGTCGCCACTGGGACACTGTGAACCAGGAGTGAGTCGGAGCTGCCGC

GCTGCCCAGGCCATGGACTGTGAGGTCAACAACGGTTCCAGCCTCAGGGATGAGTGCATCACAAACCTAC

TGGTGTTTGGCTTCCTCCAAAGCTGTTCTGACAACAGCTTCCGCAGAGAGCTGGACGCACTGGGCCACGA

GCTGCCAGTGCTGGCTCCCCAGTGGGAGGGCTACGATGAGCTGCAGACTGATGGCAACCGCAGCAGCCAC

TCCCGCTTGGGAAGAATAGAGGCAGATTCTGAAAGTCAAGAAGACATCATCCGGAATATTGCCAGGCACC

TCGCCCAGGTCGGGGACAGCATGGACCGTAGCATCCCTCCGGGCCTGGTGAACGGCCTGGCCCTGCAGCT

CAGGAACACCAGCCGGTCGGAGGAGGACCGGAACAGGGACCTGGCCACTGCCCTGGAGCAGCTGCTGCAG

GCCTACCCTAGAGACATGGAGAAGGAGAAGACCATGCTGGTGCTGGCCCTGCTGCTGGCCAAGAAGGTGG

CCAGTCACACGCCGTCCTTGCTCCGTGATGTCTTTCACACAACAGTGAATTTTATTAACCAGAACCTACG

CACCTACGTGAGGAGCTTAGCCAGAAATGGGATGGACTGAACGGACAGTTCCAGAAGTGTGACTGGCTAA

AGCTCGATGTGGTCACAGCTGTATAGCTGCTTCCAGTGTAGACGGAGCCCTGGCATGTCAACAGCGTTCC

TAGAGAAGACAGGCTGGAAGATAGCTGTGACTTCTATTTTAAAGACAATGTTAAACTTATAACCCACTTT

AAAATATCTACATTAATATACTTGAATGAAAATGTCCATTTACACGTATTTGAATGGCCTTCATATCATC

CACACATGAATCTGCACATCTGTAAATCTACACACGGTGCCTTTATTTCCACTGTGCAGGTTCCCACTTA

AAAATTAAATTGGAAAGCAGGTTTCAAGGAAGTAGAAACAAAATACAATTTTTTTGGTAAAAAAAAATTA

CTGTTTATTAAAGTACAACCATAGAGGATGGTCTTACAGCAGGCAGTATCCTGTTTGAGGAAAGCAAGAA

TCAGAGAAGGAACATACCCCTTACAAATGAAAAATTCCACTCAAAATAGGGACTATCTATCTTAATACTA

AGGAACCAACAATCTTCCTGTTTAAAAAACCACATGGCACAGAGATTCTGAACTAAAGTGCTGCACTCAA

ATGATGGGAAGTCCGGCCCCAGTACACAGGGGCTTGACTTTTTCAACTTCGTTTCCTTTGTTGGAGTCAA

AAAGAACCACTTGTGGTTCTAAAAGGTGTGAAGGTGATTTAAGGGCCCAGGTCAGCCACTGTTTGTTTAC

AAAATCAGGTAACTAACTGCATACACTTTTTCTCTTTCCATGACATCAAGACTTTGCTAAAGACATGAAG

CCACGGGTGCCAGAAGCTACTGCGATGCCCCGGGAGTTAGCCCCCTGGTAATAGCTGTAAACTTCCAATT

TCTAGCCATACGCTCAGCTCATCCATGCCTCAGAAGTGCATCTGGAGAGAACAGGTTTCTAAGCATAAAA

GATGAAAGAGCAGTTGGACTTTTTAAAAATTCAGCAAAGTGGTTCCCTCTCTTAGGGACAGTCAAAACCA

AGTCACTTAGGTAGTACCAAAATAAATAAGGAAAAGCTTAGCTTTAGAAACAGTGCAACACTGGTCTGCT

GTTCCAGTGGTAAGCTATGTCCCAGGAATCAGTTTAAAAGCACGACAGTGGATGCTGGGTCCATATCACA

CACATTGCTGTGAACAGGAAACTCCTGTGACCACAACATGAGGCCACTGGAGACGCATATGAGTAAGGGC

ACTGACGGACTCATGATTTCTTCTTACCAGATGCTTTCCTGTTCTTTAAGAGTTTAAAATCATCAGAAAG

GAAAAACAAACTCTATATTGTTCAGCATGCAATACATACCACGCTAGGGCTGGCTCAATTGAAAGTGGGC

AAAAGCTTACAAATACTAAAAAGAAGTGCTGCCGCGCAGTGTGGAGGCCACTGTTTGGAAATAAATCTTC

CTAACACTACGA

>NM_001168.3 Homo sapiens baculoviral IAP repeat containing 5 (BIRC5), transcript variant 1, mRNA

GCGGCGCGCCATTAACCGCCAGATTTGAATCGCGGGACCCGTTGGCAGAGGTGGCGGCGGCGGCATGGGT

GCCCCGACGTTGCCCCCTGCCTGGCAGCCCTTTCTCAAGGACCACCGCATCTCTACATTCAAGAACTGGC

CCTTCTTGGAGGGCTGCGCCTGCACCCCGGAGCGGATGGCCGAGGCTGGCTTCATCCACTGCCCCACTGA

GAACGAGCCAGACTTGGCCCAGTGTTTCTTCTGCTTCAAGGAGCTGGAAGGCTGGGAGCCAGATGACGAC

CCCATAGAGGAACATAAAAAGCATTCGTCCGGTTGCGCTTTCCTTTCTGTCAAGAAGCAGTTTGAAGAAT

TAACCCTTGGTGAATTTTTGAAACTGGACAGAGAAAGAGCCAAGAACAAAATTGCAAAGGAAACCAACAA

TAAGAAGAAAGAATTTGAGGAAACTGCGGAGAAAGTGCGCCGTGCCATCGAGCAGCTGGCTGCCATGGAT

TGAGGCCTCTGGCCGGAGCTGCCTGGTCCCAGAGTGGCTGCACCACTTCCAGGGTTTATTCCCTGGTGCC

ACCAGCCTTCCTGTGGGCCCCTTAGCAATGTCTTAGGAAAGGAGATCAACATTTTCAAATTAGATGTTTC

AACTGTGCTCTTGTTTTGTCTTGAAAGTGGCACCAGAGGTGCTTCTGCCTGTGCAGCGGGTGCTGCTGGT

AACAGTGGCTGCTTCTCTCTCTCTCTCTCTTTTTTGGGGGCTCATTTTTGCTGTTTTGATTCCCGGGCTT

ACCAGGTGAGAAGTGAGGGAGGAAGAAGGCAGTGTCCCTTTTGCTAGAGCTGACAGCTTTGTTCGCGTGG

GCAGAGCCTTCCACAGTGAATGTGTCTGGACCTCATGTTGTTGAGGCTGTCACAGTCCTGAGTGTGGACT

TGGCAGGTGCCTGTTGAATCTGAGCTGCAGGTTCCTTATCTGTCACACCTGTGCCTCCTCAGAGGACAGT

TTTTTTGTTGTTGTGTTTTTTTGTTTTTTTTTTTTTGGTAGATGCATGACTTGTGTGTGATGAGAGAATG

GAGACAGAGTCCCTGGCTCCTCTACTGTTTAACAACATGGCTTTCTTATTTTGTTTGAATTGTTAATTCA

CAGAATAGCACAAACTACAATTAAAACTAAGCACAAAGCCATTCTAAGTCATTGGGGAAACGGGGTGAAC

TTCAGGTGGATGAGGAGACAGAATAGAGTGATAGGAAGCGTCTGGCAGATACTCCTTTTGCCACTGCTGT

GTGATTAGACAGGCCCAGTGAGCCGCGGGGCACATGCTGGCCGCTCCTCCCTCAGAAAAAGGCAGTGGCC

TAAATCCTTTTTAAATGACTTGGCTCGATGCTGTGGGGGACTGGCTGGGCTGCTGCAGGCCGTGTGTCTG

TCAGCCCAACCTTCACATCTGTCACGTTCTCCACACGGGGGAGAGACGCAGTCCGCCCAGGTCCCCGCTT

TCTTTGGAGGCAGCAGCTCCCGCAGGGCTGAAGTCTGGCGTAAGATGATGGATTTGATTCGCCCTCCTCC

CTGTCATAGAGCTGCAGGGTGGATTGTTACAGCTTCGCTGGAAACCTCTGGAGGTCATCTCGGCTGTTCC

TGAGAAATAAAAAGCCTGTCATTTCAAACACTGCTGTGGACCCTACTGGGTTTTTAAAATATTGTCAGTT

TTTCATCGTCGTCCCTAGCCTGCCAACAGCCATCTGCCCAGACAGCCGCAGTGAGGATGAGCGTCCTGGC

AGAGACGCAGTTGTCTCTGGGCGCTTGCCAGAGCCACGAACCCCAGACCTGTTTGTATCATCCGGGCTCC

TTCCGGGCAGAAACAACTGAAAATGCACTTCAGACCCACTTATTTCTGCCACATCTGAGTCGGCCTGAGA

TAGACTTTTCCCTCTAAACTGGGAGAATATCACAGTGGTTTTTGTTAGCAGAAAATGCACTCCAGCCTCT

GTACTCATCTAAGCTGCTTATTTTTGATATTTGTGTCAGTCTGTAAATGGATACTTCACTTTAATAACTG

TTGCTTAGTAATTGGCTTTGTAGAGAAGCTGGAAAAAAATGGTTTTGTCTTCAACTCCTTTGCATGCCAG

GCGGTGATGTGGATCTCGGCTTCTGTGAGCCTGTGCTGTGGGCAGGGCTGAGCTGGAGCCGCCCCTCTCA

GCCCGCCTGCCACGGCCTTTCCTTAAAGGCCATCCTTAAAACCAGACCCTCATGGCTACCAGCACCTGAA

AGCTTCCTCGACATCTGTTAATAAAGCCGTAGGCCCTTGTCTAAGTGCAACCGCCTAGACTTTCTTTCAG

ATACATGTCCACATGTCCATTTTTCAGGTTCTCTAAGTTGGAGTGGAGTCTGGGAAGGGTTGTGAATGAG

GCTTCTGGGCTATGGGTGAGGTTCCAATGGCAGGTTAGAGCCCCTCGGGCCAACTGCCATCCTGGAAAGT

AGAGACAGCAGTGCCCGCTGCCCAGAAGAGACCAGCAAGCCAAACTGGAGCCCCCATTGCAGGCTGTCGC

CATGTGGAAAGAGTAACTCACAATTGCCAATAAAGTCTCATGTGGTTTTATCTA

>NM_016252.4 Homo sapiens baculoviral IAP repeat containing 6 (BIRC6), transcript variant 1, mRNA

CTCCCTCCCTGCTTCTCCCCCTCTCCCGTCAGCCTCCCTCCGAGTTTGGCCCCTCCGGCCGGGCGATCGA

CGTTCCGCGTGCGTGCGGGCGCCTGACTTCACTTCCGGCTAACGCGCTCGGCTTGCCCCCTGGCCCCGGA

TGGTGACTGGTGGTGGTGCTGCACCTCCCGGGACTGTCACTGAGCCGCTTCCCAGTGTGATTGTGCTGAG

CGCAGGCCGGAAGATGGCGGCTGCGGCTGCGGCGGCCTCGGGCCCCGGCTGCTCCTCGGCGGCGGGGGCG

GGGGCGGCCGGGGTCTCAGAGTGGCTGGTGCTGCGGGACGGCTGCATGCACTGCGACGCCGACGGGCTGC

ACAGCCTGTCCTACCACCCTGCGCTCAACGCCATCCTGGCCGTCACTAGCCGCGGGACCATCAAAGTCAT

CGACGGCACCTCGGGGGCCACACTGCAGGCCTCCGCGCTCAGTGCTAAACCAGGTGGACAGGTGAAATGT

CAGTATATCTCTGCTGTGGATAAAGTTATATTTGTGGATGATTATGCAGTAGGGTGTAGGAAGGACCTTA

ATGGAATCTTGTTGTTAGACACTGCTCTGCAAACTCCAGTTTCAAAGCAGGATGATGTGGTTCAGCTTGA

ATTACCCGTTACAGAGGCACAGCAGCTCTTATCAGCATGTTTAGAAAAGGTAGATATTTCTAGTACAGAG

GGTTATGATTTGTTCATCACACAGCTCAAAGATGGTTTAAAAAATACATCTCATGAGACTGCAGCAAACC

ACAAAGTTGCTAAGTGGGCCACAGTTACATTTCATCTTCCTCATCATGTGTTGAAGTCCATTGCCAGTGC

CATTGTAAATGAACTCAAGAAAATAAATCAAAATGTTGCTGCCTTACCTGTGGCGTCCTCAGTGATGGAC

AGATTGTCTTACCTCTTACCTAGTGCACGTCCAGAACTCGGAGTGGGGCCAGGCCGTTCTGTAGACAGAT

CACTGATGTATAGTGAAGCTAACAGACGGGAGACATTTACCTCATGGCCTCATGTAGGCTATAGGTGGGC

ACAACCAGATCCCATGGCTCAAGCTGGATTTTATCATCAGCCTGCCTCATCTGGAGATGATAGAGCCATG

TGTTTTACTTGTAGTGTATGCCTCGTTTGTTGGGAACCTACTGATGAACCTTGGTCTGAACACGAAAGAC

ATTCCCCAAACTGCCCATTTGTGAAAGGTGAGCACACACAGAATGTGCCATTGTCAGTCACTCTTGCAAC

AAGTCCTGCACAGTTTCCTTGTACGGATGGAACTGACAGAATATCTTGCTTTGGGTCGGGGAGCTGCCCT

CATTTTCTAGCTGCTGCAACTAAACGAGGAAAGATCTGCATATGGGATGTTTCCAAACTTATGAAGGTGC

ACTTAAAGTTTGAAATTAATGCCTATGATCCAGCAATTGTACAACAGCTTATTCTATCAGGAGACCCAAG

CTCAGGAGTTGATTCAAGGAGACCAACTTTGGCGTGGCTGGAGGACTCCTCTAGTTGCTCAGATATACCA

AAATTGGAAGGAGATAGTGATGATTTACTGGAGGATTCAGACAGTGAAGAGCATTCCAGATCAGATTCTG

TGACAGGGCATACATCACAGAAGGAAGCCATGGAAGTAAGCCTTGATATAACAGCACTCAGCATTCTCCA

ACAGCCAGAAAAACTTCAGTGGGAGATTGTTGCAAATGTGCTTGAAGATACTGTTAAGGATCTTGAAGAA

CTTGGGGCAAATCCTTGTTTAACAAACTCTAAGAGTGAAAAGACAAAGGAAAAGCACCAGGAGCAACACA

ACATTCCTTTTCCATGTTTATTAGCTGGAGGTTTATTAACATATAAATCTCCTGCTACCTCACCCATTAG

TAGTAATTCTCACAGGTCACTGGATGGTTTAAGCAGAACTCAGGGTGAAAGTATATCAGAACAAGGGTCA

ACTGACAATGAATCCTGCACTAATTCAGAACTAAATTCTCCTCTGGTAAGGAGGACTTTACCGGTTTTGC

TTCTTTATAGCATCAAGGAATCTGATGAGAAAGCAGGAAAGATCTTTTCACAGATGAACAATATTATGAG

TAAAAGTTTGCATGATGATGGTTTTACTGTTCCACAGATTATTGAAATGGAGCTGGATAGTCAGGAGCAG

TTGTTATTGCAGGATCCTCCTGTGACTTACATTCAGCAATTTGCAGATGCAGCAGCCAACCTTACCTCTC

CGGATTCTGAGAAGTGGAACTCTGTGTTTCCCAAGCCTGGGACTTTGGTTCAGTGCTTGAGGCTGCCAAA

GTTTGCAGAGGAGGAGAATCTTTGTATAGACTCAATAACTCCTTGTGCTGACGGAATTCATTTGTTGGTA

GGACTGCGGACATGCCCTGTTGAATCCTTGAGTGCAATAAATCAAGTAGAGGCCTTGAATAATTTAAATA

AATTAAACTCTGCACTATGTAATAGACGGAAAGGTGAGCTGGAATCAAATCTTGCTGTAGTGAATGGTGC

AAATATTAGTGTAATCCAACATGAATCACCAGCAGATGTACAGACTCCTTTAATAATTCAGCCTGAGCAG

AGGAATGTTAGTGGTGGATATTTAGTGCTTTATAAAATGAATTATGCCACTCGGATAGTGACTTTAGAAG

AGGAGCCAATAAAAATACAACATATCAAAGATCCCCAGGACACAATTACCTCGCTCATTTTGCTTCCACC

CGATATATTGGATAATCGAGAGGATGACTGTGAGGAACCTATTGAGGACATGCAGTTAACCTCAAAGAAT

GGTTTTGAGAGAGAAAAAACGTCTGACATTTCTACTCTTGGACACCTGGTAATAACCACTCAGGGAGGAT

ATGTAAAAATACTAGATCTTTCAAACTTTGAAATTTTGGCCAAAGTGGAGCCTCCCAAAAAGGAGGGCAC
TGAGGAACAGGACACATTTGTTTCTGTGATTTACTGTTCTGGCACAGACAGGCTGTGTGCATGCACCAAA
GGTGGTGAGCTTCATTTTCTCCAAATTGGAGGAACCTGTGATGATATTGATGAAGCTGATATACTAGTGG
ATGGATCTCTTTCTAAAGGAATAGAACCATCTTCAGAAGGTTCCAAACCTTTATCAAATCCTTCAAGTCC
TGGCATTTCAGGAGTTGATTTATTGGTGGACCAGCCATTCACCCTTGAAATCTTGACATCCCTAGTGGAG
CTAACCCGCTTTGAGACTTTGACTCCAAGGTTTTCAGCGACTGTTCCTCCATGCTGGGTAGAAGTTCAAC
AAGAACAGCAGCAAAGGAGGCATCCTCAACATTTGCATCAGCAACACCATGGTGATGCTGCTCAGCATAC
TCGAACTTGGAAACTACAGACCGACAGCAACAGCTGGGATGAACATGTATTTGAATTAGTACTACCTAAA
GCTTGTATGGTTGGACATGTGGACTTCAAATTCGTTTTGAACTCAAACATCACCAATATTCCACAGATAC
AAGTGACACTGCTGAAAAATAAAGCTCCAGGATTAGGGAAAGTCAATGCTCTTAACATTGAAGTGGAACA
AAATGGGAAACCGTCCCTGGTTGATTTGAATGAAGAAATGCAGCACATGGATGTAGAGGAATCACAGTGT
CTTAGATTATGTCCATTTTTGGAGGATCATAAAGAAGACATTCTATGTGGGCCAGTATGGCTTGCTAGTG
GCCTTGATCTATCAGGGCATGCTGGAATGTTGACGTTAACAAGCCCCAAACTTGTTAAAGGTATGGCAGG
AGGAAAATATCGTTCGTTTTTAATCCATGTCAAGGCAGTGAATGAAAGAGGAACAGAAGAGATTTGTAAT
GGTGGTATGCGTCCTGTAGTAAGGCTTCCATCCCTAAAACACCAGAGTAACAAGGGTTATTCACTTGCTT
CACTTTTGGCTAAAGTTGCAGCAGGCAAGGAAAAATCATCTAATGTTAAGAATGAAAATACAAGTGGCAC
CCGTAAATCTGAAAACCTCCGGGGCTGTGATTTACTTCAAGAGGTCTCAGTCACCATTCGAAGATTTAAG
AAAACCTCAATTTCTAAGGAAAGAGTGCAACGATGTGCCATGTTACAGTTTTCAGAATTTCATGAGAAGC
TTCTTAATACTCTTTGCAGAAAAACAGATGATGGCCAGATCACAGAACATGCCCAGAGCCTTGTGTTGGA
TACTCTCTGTTGGTTAGCTGGAGTTCATTCAAATGGACCCGGAAGCTCAAAGGAAGGAAATGAGAACCTA
CTTTCAAAAACACGAAAATTTCTGTCAGACATCGTACGTGTTTGCTTCTTTGAGGCAGGACGAAGTATAG
CCCATAAGTGTGCCCGATTTCTAGCCTTGTGCATTAGTAATGGCAAATGTGACCCATGTCAACCAGCATT
TGGACCTGTTCTGTTGAAGGCTTTACTTGATAATATGTCATTTTTACCTGCAGCAACAACTGGTGGTTCT
GTCTATTGGTATTTTGTCTTACTGAATTATGTGAAAGATGAAGATCTGGCTGGATGCAGTACAGCTTGTG
CATCTTTGCTTACTGCAGTGTCCAGACAGTTACAGGACAGGCTAACACCAATGGAGGCTTTACTTCAGAC

AAGATATGGATTATATAGCTCACCATTTGATCCAGTCCTCTTTGATTTGGAGATGAGTGGCTCTTCTTGT

AAAAATGTTTATAACAGCAGCATTGGTGTCCAGTCAGATGAAATTGATTTATCAGATGTCCTTTCAGGAA

ATGGAAAGGTCAGTAGTTGCACAGCTGCTGAGGGTAGTTTCACATCTCTCACTGGACTTTTGGAAGTTGA

ACCTCTGCACTTTACTTGTGTGTCAACTAGTGATGGAACCAGAATAGAAAGGGATGATGCAATGAGTTCC

TTCGGGGTTACTCCTGCAGTAGGTGGACTATCATCTGGGACAGTTGGGGAAGCCTCGACAGCCCTGAGTT

CAGCAGCCCAGGTAGCTTTGCAGTCTCTCTCTCATGCAATGGCTTCAGCCGAGCAACAGCTACAGGTGCT

GCAAGAGAAACAGCAGCAGCTTTTGAAGCTTCAGCAACAGAAAGCAAAGCTGGAAGCCAAGTTACATCAG

ACAACAGCTGCAGCAGCTGCAGCAGCATCAGCAGTAGGTCCTGTTCACAACTCTGTGCCTTCCAACCCAG

TGGCTGCCCCTGGATTCTTCATTCATCCATCTGATGTTATTCCACCCACTCCAAAAACAACACCTCTTTT

TATGACTCCACCACTCACTCCACCCAATGAAGCAGTTTCCGTTGTGATTAATGCCGAACTTGCACAGCTT

TTCCCAGGCTCAGTCATTGATCCCCCAGCAGTCAATCTTGCTGCACATAACAAAAATTCCAACAAGTCCA

GAATGAATCCACTTGGTTCTGGTCTAGCCCTTGCAATTTCTCATGCTTCACATTTTCTTCAACCTCCGCC

TCACCAGTCCATTATTATAGAGCGAATGCATTCAGGAGCAAGAAGATTTGTGACCTTGGATTTTGGGAGG

CCTATATTGTTGACTGATGTATTGATTCCCACTTGTGGAGACTTGGCCTCTTTGTCAATTGACATTTGGA

CATTAGGAGAAGAGGTGGATGGAAGGCGGTTGGTAGTGGCAACTGATATAAGCACTCATTCACTAATTCT

TCATGACTTAATACCACCTCCCGTGTGCAGATTCATGAAGATCACTGTTATTGGACGTTACGGGAGTACA

AATGCCAGAGCCAAAATCCCATTAGGATTTTACTATGGTCATACCTACATCTTGCCTTGGGAAAGTGAAC

TGAAGTTAATGCATGATCCTCTAAAGGGAGAGGGAGAATCTGCAAACCAGCCAGAAATTGACCAGCATTT

AGCAATGATGGTTGCTTTGCAGGAGGATATACAGTGCAGGTATAACTTGGCTTGTCATCGTCTGGAAACC

CTTTTGCAAAGTATTGATCTTCCTCCTCTAAACAGTGCTAACAATGCACAGTACTTTTTACGAAAACCAG

ATAAGGCAGTTGAGGAAGACAGTAGGGTTTTTTCTGCTTATCAAGATTGTATTCAGCTACAGCTTCAACT

AAATTTGGCTCATAATGCAGTGCAGAGGCTCAAAGTGGCGCTAGGTGCAAGCCGGAAGATGTTGAGTGAA

ACATCAAATCCAGAAGATTTAATTCAGACATCTTCCACAGAGCAGTTACGTACTATCATCAGATATTTAC

TGGACACTTTGCTCAGCCTGCTTCATGCTTCTAATGGACACTCTGTTCCTGCAGTTTTGCAGAGCACATT

TCATGCCCAGGCCTGTGAAGAGCTCTTTAAACACTTGTGCATCAGTGGAACCCCAAAGATACGGTTACAT

ACTGGTCTTCTTCTTGTTCAACTGTGTGGTGGTGAAAGGTGGTGGGGTCAATTTCTTTCTAATGTCCTTC

AGGAATTGTACAATTCGGAACAGCTTCTCATCTTTCCACAGGATAGGGTCTTCATGTTACTTTCCTGCAT

TGGTCAAAGATCACTTAGTAATAGTGGAGTATTAGAAAGCTTACTTAATCTCTTGGATAATTTATTGTCA

CCTCTTCAGCCACAGTTACCCATGCATAGGAGGACAGAAGGAGTACTAGATATTCCCATGATCAGTTGGG

TTGTTATGCTGGTGTCCAGGTTGCTGGATTATGTGGCAACTGTTGAAGATGAAGCAGCAGCTGCAAAGAA

ACCTTTGAATGGTAATCAGTGGAGTTTTATTAACAATAATCTACACACTCAGAGCTTAAATAGATCTTCT

AAAGGCAGCAGTAGCCTTGATAGATTATATTCCAGAAAAATCAGAAAGCAGCTTGTTCATCATAAACAGC

AACTTAACCTACTAAAAGCAAAGCAGAAGGCATTGGTAGAACAGATGGAAAAAGAAAAAATACAAAGTAA

CAAAGGATCATCATATAAACTCCTGGTAGAACAAGCAAAACTAAAGCAGGCCACTTCAAAGCACTTTAAG

GATTTAATTCGTTTACGTCGGACAGCAGAATGGTCCCGTTCTAATTTAGACACAGAAGTTACAACAGCAA

AAGAAAGTCCTGAGATAGAACCACTTCCATTTACTCTGGCCCATGAGCGTTGTATCTCAGTAGTCCAGAA

ACTTGTTCTGTTTCTTCTCTCCATGGACTTTACATGTCATGCAGATCTCTTATTGTTTGTTTGTAAGGTT

CTTGCACGCATTGCAAATGCCACGAGGCCAACTATTCATCTGTGTGAGATTGTGAACGAACCCCAGCTGG

AAAGACTGCTGTTACTTTTGGTTGGAACTGACTTCAATAGAGGAGATATATCTTGGGGTGGTGCTTGGGC

TCAGTATTCCTTAACTTGCATGCTACAAGATATTTTAGCAGGAGAATTACTGGCTCCAGTAGCCGCAGAA

GCCATGGAGGAAGGAACAGTGGGTGATGATGTAGGTGCGACAGCTGGTGACTCTGATGACTCCCTTCAAC

AGTCCTCAGTTCAGTTGCTGGAAACTATAGATGAACCTTTGACACATGACATAACAGGTGCACCTCCTCT

GTCCTCTTTGGAAAAAGATAAAGAAATTGACCTTGAGTTACTTCAGGATCTAATGGAAGTTGACATTGAT

CCTTTAGATATTGATTTGGAAAAGGACCCTCTTGCAGCCAAGGTTTTTAAGCCAATAAGCAGTACATGGT

ATGATTATTGGGGTGCTGATTATGGGACCTACAATTACAACCCTTACATTGGAGGTCTGGGAATTCCTGT

AGCAAAGCCACCAGCAAACACGGAGAAGAACGGATCACAGACAGTTAGCGTTTCAGTCTCTCAGGCCCTG

GATGCTCGCCTAGAAGTTGGACTTGAACAGCAAGCAGAACTGATGTTGAAAATGATGTCTACTCTGGAGG

CAGATTCCATTTTACAGGCATTAACAAATACATCTCCTACATTATCACAGTCTCCCACTGGAACAGATGA

TTCACTTCTAGGGGGTTTACAAGCAGCAAACCAAACCAGCCAGCTTATTATACAGTTATCATCTGTCCCA

ATGTTAAATGTTTGTTTCAACAAACTTTTTTCCATGCTTCAAGTCCATCATGTTCAGTTGGAGTCACTTC

TCCAATTGTGGCTCACACTGAGCCTGAATTCTAGTTCAACTGGAAACAAAGAAAATGGAGCAGACATATT

TTTATATAATGCTAATAGGATACCTGTTATTTCATTAAATCAAGCATCAATAACTAGCTTTCTCACAGTG

TTAGCTTGGTATCCCAATACTTTGCTCCGGACATGGTGCCTTGTGCTTCATAGCCTAACACTCATGACAA

ACATGCAGCTTAATTCTGGTTCCAGCAGTGCCATTGGAACTCAGGAGAGTACTGCTCATTTGTTGGTTTC

AGATCCAAACCTAATTCATGTATTAGTGAAATTTCTTTCTGGCACCAGTCCACATGGAACAAATCAACAC

AGTCCACAGGTTGGTCCTACAGCTACACAAGCTATGCAAGAATTTCTTACTCGATTACAAGTGCATCTTT

CTTCAACATGTCCTCAGATATTCAGTGAATTTTTGCTCAAGCTAATTCATATACTTTCAACTGAAAGGGG

TGCTTTCCAGACAGGCCAAGGACCTCTCGATGCCCAAGTGAAGCTCTTAGAATTCACTCTGGAGCAGAAT

TTTGAAGTCGTTTCAGTTAGTACTATTTCTGCCGTGATAGAATCGGTTACATTTTTAGTGCACCACTATA

TCACTTGCTCAGACAAAGTAATGTCAAGAAGTGGATCAGATAGCTCCGTGGGTGCTCGAGCATGCTTTGG

GGGACTCTTTGCCAATCTTATTCGTCCGGGTGATGCAAAAGCAGTTTGTGGCGAAATGACAAGAGATCAA

CTCATGTTTGATTTGTTAAAACTTGTTAACATTTTAGTGCAGCTGCCTCTTTCAGGCAATAGGGAATACA

GTGCAAGAGTGTCTGTGACCACAAATACAACAGATAGTGTTTCAGATGAAGAAAAAGTCTCAGGAGGCAA

AGATGGCAATGGAAGCAGTACCAGTGTTCAAGGATCGCCTGCATATGTTGCTGACTTAGTCTTAGCCAAC

CAACAAATTATGAGCCAGATTTTGTCTGCTCTGGGCCTGTGTAATAGCAGTGCCATGGCAATGATAATTG

GAGCAAGTGGATTACATCTCACTAAACATGAAAACTTTCATGGTGGGTTGGATGCCATATCAGTTGGGGA

TGGATTATTTACCATACTGACAACCCTTAGTAAAAAAGCTTCTACAGTCCACATGATGCTGCAGCCAATT

TTAACATACATGGCCTGTGGATATATGGGCAGACAAGGCTCTCTTGCTACTTGCCAGTTATCTGAGCCAT

TATTGTGGTTCATTTTGAGAGTATTGGATACTAGTGATGCCTTGAAAGCATTTCATGATATGGGTGGTGT

TCAGCTCATATGCAATAATATGGTTACTAGTACAAGGGCTATTGTGAACACTGCAAGAAGTATGGTATCA

ACTATTATGAAATTTCTTGACTCTGGTCCAAATAAAGCTGTTGACAGCACATTGAAAACAAGAATACTAG

CTTCTGAGCCTGACAATGCTGAAGGGATTCATAACTTTGCACCCCTCGGTACAATCACATCTAGCAGTCC

TACTGCCCAACCAGCTGAAGTGCTATTGCAGGCCACACCTCCTCACAGAAGAGCTCGCTCTGCTGCTTGG

TCCTACATCTTTCTTCCAGAGGAGGCTTGGTGTGACCTTACCATTCACCTTCCTGCAGCAGTGCTGCTTA

AGGAGATACATATCCAGCCTCATCTTGCATCTCTTGCAACCTGCCCTTCCTCAGTGTCTGTTGAAGTAAG

TGCAGATGGGGTAAATATGCTACCTTTGTCCACTCCTGTTGTCACAAGTGGCCTCACCTACATAAAAATT

CAGCTTGTAAAAGCCGAAGTAGCTTCTGCTGTCTGCCTTAGACTACATCGTCCACGGGATGCCAGCACAT

TAGGCCTTTCACAAATTAAATTATTGGGGCTCACTGCTTTTGGTACCACCTCTTCTGCAACAGTTAATAA

TCCATTCCTTCCATCTGAAGATCAGGTATCCAAAACAAGTATTGGATGGTTACGGTTATTACATCATTGC

CTTACTCACATAAGTGATCTAGAAGGAATGATGGCAAGTGCAGCTGCACCTACTGCTAATCTGCTGCAGA

CTTGTGCGGCCTTATTGATGTCACCTTACTGTGGAATGCATTCACCCAACATCGAGGTTGTGCTTGTAAA

GATAGGACTGCAGTCTACTAGAATTGGCCTGAAGCTCATAGACATTCTCCTGAGAAATTGTGCAGCATCA

GGCAGTGATCCTACAGATTTGAATAGTCCTTTACTTTTTGGAAGACTAAATGGACTCTCTTCTGACTCTA

CGATAGATATTCTTTACCAGCTTGGAACAACTCAGGATCCTGGTACAAAAGACAGAATTCAGGCCTTGTT

AAAATGGGTTAGTGATTCTGCAAGAGTGGCTGCTATGAAGAGAAGTGGCAGGATGAACTACATGTGTCCT

AACTCCTCAACAGTAGAGTATGGTCTTCTGATGCCATCTCCTTCTCATTTGCACTGTGTAGCAGCCATTC

TGTGGCATAGTTATGAGCTGCTTGTAGAATATGACTTACCAGCACTCCTGGACCAAGAGCTCTTTGAGTT

ACTTTTTAATTGGTCCATGTCTCTTCCCTGCAATATGGTTTTGAAGAAAGCTGTTGACAGTCTACTTTGC

TCAATGTGTCACGTACACCCAAACTATTTTTCTTTGCTCATGGGCTGGATGGGAATTACCCCTCCTCCAG

TGCAATGTCATCATAGACTGTCCATGACAGATGATAGCAAAAAGCAGGATCTTAGTTCATCTTTAACAGA

TGACTCTAAAAATGCACAAGCACCTCTCGCATTAACTGAATCACATTTGGCTACCCTTGCTTCCTCTTCT

CAATCTCCTGAAGCTATTAAACAATTACTAGACTCAGGTTTGCCTTCTCTTCTTGTGAGGAGTCTGGCTA

GTTTCTGCTTTAGCCACATTTCTAGCTCAGAAAGCATTGCCCAGTCAATAGATATTTCCCAGGACAAACT

CAGGCGCCATCATGTCCCACAACAATGTAATAAGATGCCTATCACAGCCGACCTAGTTGCTCCTATTCTT

AGGTTTTTGACAGAAGTTGGCAATAGCCATATTATGAAAGATTGGCTTGGTGGTTCTGAAGTCAATCCAC

TATGGACAGCACTTCTGTTTTTATTGTGTCACTCTGGGTCCACTTCTGGAAGCCATAATTTAGGTGCACA

ACAGACCAGTGCAAGATCAGCTTCTCTTTCTTCAGCTGCTACAACAGGACTGACTACTCAACAGCGCACA

GCAATTGAGAATGCAACTGTTGCGTTCTTTCTACAGTGCATTTCATGCCATCCTAATAATCAAAAGCTGA

TGGCACAGGTTCTTTGTGAACTATTTCAGACATCTCCTCAAAGAGGGAACCTTCCAACATCTGGGAACAT

TTCAGGGTTTATACGAAGATTATTTTTACAGTTGATGCTGGAAGATGAGAAAGTGACAATGTTTCTTCAG

TCTCCATGTCCACTGTACAAAGGTAGAATTAATGCTACTAGCCACGTCATCCAGCATCCAATGTATGGAG

CAGGCCACAAATTCCGTACTCTTCATTTGCCAGTCTCAACAACATTATCAGATGTTCTTGACAGAGTGTC

AGATACTCCAAGTATCACAGCTAAATTAATTAGTGAACAAAAAGATGACAAAGAAAAGAAAAACCATGAA

GAGAAAGAAAAAGTTAAAGCGGAAAATGGATTTCAAGACAATTACAGTGTTGTTGTTGCCTCTGGGCTGA

AGTCTCAATCTAAACGTGCTGTGTCAGCTACACCACCTCGCCCACCATCCAGGAGGGGGAGGACAATACC

TGATAAAATAGGAAGTACTTCAGGAGCAGAGGCTGCCAACAAAATAATTACTGTCCCAGTGTTTCACCTG

TTTCACAAACTCTTGGCAGGCCAGCCATTGCCAGCTGAAATGACACTTGCCCAGCTTTTAACTCTCCTAT

ATGACCGAAAACTTCCTCAGGGTTACCGCTCAATAGATCTGACTGTTAAATTGGGATCAAGAGTTATAAC

AGACCCCAGTCTATCAAAAACAGATTCTTATAAAAGACTACACCCTGAAAAAGATCATGGAGACTTACTT

GCTAGCTGTCCAGAAGATGAGGCTCTCACTCCAGGTGATGAATGCATGGATGGGATACTGGATGAATCTT

TGCTTGAAACCTGTCCAATTCAGTCACCATTACAAGTTTTTGCAGGAATGGGTGGACTGGCTCTTATTGC

TGAAAGACTACCCATGCTATATCCAGAAGTAATTCAACAGGTGAGTGCTCCAGTTGTAACATCTACCACT

CAGGAAAAGCCGAAGGATAGCGATCAGTTTGAATGGGTGACCATTGAACAGTCAGGGGAGTTAGTTTATG

AAGCACCAGAAACTGTTGCGGCTGAACCTCCACCTATCAAGTCAGCAGTACAGACCATGTCTCCCATACC

TGCCCATTCTTTGGCTGCTTTTGGATTATTTCTTCGTCTTCCGGGCTATGCGGAAGTGCTACTGAAAGAG

AGAAAACATGCCCAGTGCCTTCTTCGATTGGTATTGGGAGTGACAGATGATGGAGAAGGAAGTCATATTC

TTCAATCTCCATCAGCCAATGTGCTTCCAACCCTTCCTTTCCACGTCCTTCGTAGCTTGTTTAGCACTAC

ACCTTTGACAACTGATGATGGTGTACTTCTAAGGCGGATGGCATTGGAAATTGGAGCCTTACACCTCATT

CTTGTCTGTCTCTCTGCTTTGAGCCACCATTCCCCACGAGTTCCAAACTCTAGCGTGAATCAAACTGAGC

CACAGGTGTCAAGCTCTCATAACCCTACATCAACAGAAGAACAACAGTTATATTGGGCCAAAGGGACTGG

CTTTGGAACAGGCTCTACAGCTTCTGGGTGGGATGTGGAACAAGCCTTAACTAAGCAAAGGCTGGAAGAG

GAACATGTTACCTGCCTTCTGCAGGTTCTTGCCAGTTACATAAATCCCGTCAGTAGTGCGGTAAATGGAG

AAGCTCAGTCATCTCATGAGACTAGAGGGCAGAACAGTAATGCCCTTCCTTCTGTACTTCTCGAGCTTCT

CAGTCAGTCCTGCCTCATCCCAGCCATGTCATCTTATCTACGAAATGATTCAGTTCTGGACATGGCAAGA

CATGTGCCACTCTATCGGGCACTGCTGGAATTGCTTCGGGCCATTGCTTCTTGTGCTGCCATGGTGCCCC

TATTGTTGCCCCTTTCTACAGAGAACGGTGAAGAGGAAGAAGAACAGTCAGAATGTCAAACTTCTGTTGG

TACATTGTTAGCCAAAATGAAGACCTGTGTTGATACCTATACCAACCGTTTAAGATCTAAAAGGGAAAAT

GTTAAAACAGGAGTAAAACCAGATGCGTCTGATCAAGAACCAGAAGGACTTACTCTTTTGGTACCAGACA

TCCAAAAGACTGCTGAGATAGTTTATGCAGCCACCACCAGTTTGCGGCAAGCAAATCAGGAAAAAAAACT

GGGTGAATACTCCAAGAAGGCGGCTATGAAACCCAAACCTTTGTCAGTATTAAAGTCACTTGAAGAAAAA

TATGTGGCTGTTATGAAGAAATTACAGTTTGATACGTTTGAAATGGTTTCTGAAGATGAAGATGGGAAAT

TGGGATTTAAAGTAAATTACCACTACATGTCTCAGGTGAAAAATGCTAATGATGCGAACAGTGCTGCCAG

AGCTCGCCGCCTTGCCCAGGAAGCTGTGACGCTTTCAACCTCACTGCCTCTGTCTTCATCCTCTAGTGTG

TTTGTACGCTGTGATGAGGAGCGACTTGATATCATGAAGGTTCTAATAACTGGTCCAGCGGACACCCCTT

ATGCAAATGGCTGCTTTGAGTTTGATGTGTATTTTCCTCAAGATTATCCCAGTTCACCCCCTCTTGTGAA

TCTAGAGACAACTGGTGGTCATAGCGTGCGATTCAATCCAAACCTTTATAATGATGGCAAGGTTTGTTTA

AGCATCTTAAACACGTGGCATGGAAGACCAGAAGAGAAGTGGAATCCTCAGACCTCAAGCTTTTTGCAAG

TGTTGGTGTCTGTCCAGTCCCTTATATTAGTAGCTGAGCCTTATTTTAATGAACCGGGATATGAACGGTC

TAGAGGCACTCCCAGTGGCACACAGAGTTCTCGAGAATATGATGGAAACATTCGACAAGCAACAGTTAAG

TGGGCAATGCTAGAACAAATCAGAAACCCTTCACCATGTTTTAAAGAGGTAATACACAAACATTTTTACT

TGAAAAGAGTTGAGATAATGGCCCAATGTGAGGAGTGGATTGCGGATATCCAGCAGTACAGCAGTGATAA

GCGGGTAGGCAGGACTATGTCTCACCATGCAGCAGCTCTCAAGCGTCACACTGCTCAGCTCCGCGAAGAG

TTGCTGAAACTTCCCTGCCCTGAAGGCTTGGATCCTGACACTGACGATGCCCCAGAGGTGTGCAGAGCCA

CAACAGGTGCTGAGGAGACTCTAATGCATGATCAGGTTAAACCCAGCAGCAGCAAAGAACTCCCCAGTGA

CTTCCAGTTATGAGCTGCATTGATGTGGACTTCATAGACACAAAGGCTTCGAAGCACAAGCCAAATATGT

CAATATTTGTATGTAAGAAACTAATTATGTAATAGGTAATGAAACTGAAACTATACTATGCCCTTAAGGA

GATCCAGTTTAATTCAAGGTGATCTTTTATTTACCTGTACAGGAGTGTAAACTTTTTTGTGCTTTTATTT

TTCAATTGTGAGAACCACTGATTGGTATGTTCAACAAATTTGTGTATACAAAGAAATGGATAAATCACTG

CTATATAAGGGAAACTACCTTAGGAAAGAATGTTTACTGAATGTTTATTTTATTTTATTTTTTTTTTACT

ATAGAGTGAGGGGTTGTTAACAAAGAATATATATTGGTCATTCTTACAACTACTATTTAAAGTCAGCAAC

TTTTCACTGAATTTGATAGATTTTATGTTTGGCCATATCTTCATGCTCACATTTGATTTCTGAAGACCTC

CTACATACACTTCAATAAAAGTTAAATGGACATACTCCCTCTTTTTTGATTTACTGGTACATTTTTAAAA

TAATAAATCTGCCATAAAATGCATTATATCTGGAGACTTGCACTTGTATGGATGAATTTATTACATTCAA

CATATTTAATTTTATGCCTTCTAATTCTAAGATGCAGAAAAAAATAAATGAACATGATTTTATTCTATGC

CAACATTTGGGCCTCTGAATGTATCTGTTATTTGAATTTAAGTATTTGAAAAGGAATGGTCAATTTGAAA

GTCATTCTAAACTGATTTTTTTTTTCTAAAGGGCTCCTTTTTTCCTGGACTATGTGGTTTTATGACTAAA

GTCACATGTATGTATTAAACATTGAGGCTCTGTAGAGGAGAGAGGATGTACCTCTCTGGTGCTGTTACAG

TACATTCTGTACCTGCCATACAGGCTCATTTTCATGCAAATTCTTCCTAGAGCCAAATAAATAAAGACTT

AGGTGAA

>NM_013979.3 Homo sapiens BCL2 interacting protein 1 (BNIP1), transcript variant BNIP1-b, mRNA

GCCGGCGTCCCCAACATGGCGGCTCCCCAAGACGTCCACGTCCGGATCTGTAACCAAGAGATTGTCAAAT

TTGACCTGGAGGTGAAGGCGCTTATTCAGGATATCCGTGATTGTTCAGGACCCTTAAGTGCTCTTACTGA

ACTGAATACTAAAGTAAAAGAGAAATTTCAACAGTTGCGTCACAGAATACAGCCAGTTCTCTATCAAAGG

GCATTTATTTGGACTGCTTCCACATTTTTTTTTAAGCTAACTTATTCCCTGACAGACTTTTCTTCAACTC

AGCATGACTTCAACTCTCCAACTACACCTGTTACCTTCAGTGACCTGGAGCAGTTGGCTAAAGAGCAAGA

CAAAGAATCAGAGAAACAACTTCTACTCCAGGAAGTGGAGAATCACAAAAAGCAGATGCTCAGCAATCAG

GCCTCATGGAGGAAAGCTAATCTCACCTGCAAAATTGCAATCGACAATCTAGAGAAAGCAGAACTTCTTC

AGGGAGGAGATCTCTTAAGGCAAAGGAAAACCACCAAAGAGAGCCTGGCCCAGACATCCAGTACCATCAC

TGAGAGCCTCATGGGGATCAGCAGGATGATGGCCCAGCAGGTCCAGCAGAGCGAGGAGGCCATGCAGTCT

CTAGTCACTTCTTCACGAACGATCCTGGATGCAAATGAAGAATTTAAGTCCATGTCGGGCACCATCCAGC

TGGGCCGGAAGCTTATCACAAAATACAATCGCCGGGAGCTGACGGACAAGCTTCTCATCTTCCTTGCGCT

AGCCCTGTTTCTTGCTACGGTCCTCTATATTGTGAAAAAGCGGCTCTTTCCATTTTTGTGAGATCCCAAA

GGTGCCAGTTCTGGCCCTTTCAGCTCCTGTTTCAGGATCTGTCCTGGTTCCTGAGCTCTAGGCTGCTAAG

CTGAGCCACACACCCCTCCGTTTGCACCAGTTGCCTGCAGGTTGGATGGAACACAGTGCCCCACTTTTCT

GCAAGTAGCTGGCTTGTAAAGGGTGAACAGAGCCATGGGAGGAAGGTCTGGCATTGGGATGCCGCCCTGG

GGACATACGAACCGCCTCCTTCCACCATTGTGCACTATGGGAGGCCGCTGCTGCGTGGAGCACTTAAAGT

CCAGCCTCCAGGACCGGATGCCCCTCCTGTCTCCCGCTCCCATCGTGCCCTTAAATGCCAGATCTGGTGG

AGGGAAGAGAGAAGAGGTAGGAAGAAAGGTGATGAAAACTCCTGGTCATTCCTTGAATAAACTTGATTTT

ATTTAATAGAATAAAATGTATTTGATTTTGTAAGATA

>NM_004052.4 Homo sapiens BCL2 interacting protein 3 (BNIP3), mRNA

AGTCCGGGAGCGCAGCTGGGCCGCGGCGCTCCGACCTCCGCTTTCCCACCGCCCGCAGCTGAAGCACATC

CCGCAGCCCGGCGCGGACTCCGATCGCCGCAGTTGCCCTCTGGCGCCATGTCGCAGAACGGAGCGCCCGG

GATGCAGGAGGAGAGCCTGCAGGGCTCCTGGGTAGAACTGCACTTCAGCAATAATGGGAACGGGGGCAGC

GTTCCAGCCTCGGTTTCTATTTATAATGGAGACATGGAAAAAATACTGCTGGACGCACAGCATGAGTCTG

GACGGAGTAGCTCCAAGAGCTCTCACTGTGACAGCCCACCTCGCTCGCAGACACCACAAGATACCAACAG

AGCTTCTGAAACAGATACCCATAGCATTGGAGAGAAAAACAGCTCACAGTCTGAGGAAGATGATATTGAA

AGAAGGAAAGAAGTTGAAAGCATCTTGAAGAAAAACTCAGATTGGATATGGGATTGGTCAAGTCGGCCGG

AAAATATTCCCCCCAAGGAGTTCCTCTTTAAACACCCGAAGCGCACGGCCACCCTCAGCATGAGGAACAC

GAGCGTCATGAAGAAAGGGGGCATATTCTCTGCAGAATTTCTGAAAGTTTTCCTTCCATCTCTGCTGCTC

TCTCATTTGCTGGCCATCGGATTGGGGATCTATATTGGAAGGCGTCTGACAACCTCCACCAGCACCTTTT

GATGAAGAACTGGAGTCTGACTTGGTTCGTTAGTGGATTACTTCTGAGCTTGCAACATAGCTCACTGAAG

AGCTGTTAGATCCTGGGGTGGCCACGTCACTTGTGTTTATTTGTTCTGTAAATGCTGCGTTCCTAATTTA

GTAAAATAAAAGAATAGACACTAAAATCATGTTGATCTATAATTACACCTATGGGATCAATAAGCATGTC

AGACTGATTAATGTCTACTGTGAAAATTTGGTAGTAAATTTTCATTTGATATTAGATATAAATATCTGAA

TATAAATAATTTTAATATACTAGTCATGATGTGTGTTGTATTTTAAAAATTATCTGCAACCTTAATTCAG

CTGAAGTACTTTATATTTCAAAAGAATGAATAACATTGATAATAAAATCGCTACTTTAAGGGGTTTGTCC

AAAATAAATATTGTGGCCTTATATATCACACTATTGTAGAAAGTATTATTTAATTTAAATGGATGCAGGT

TGTCTACTAAAGAAAGATTATATATAACTATGCTAATTGTTCATAATCAACAGAAACCAAGATAGAGCTA

CAAACTCAGCTGTACAGTTCGTACACTAAACTCTTCTTGCTTTTGCATTATAAGGAATTAAGTCTCCGAT

TATTAGGTGATCACCCTGGATGATCAGTTTTCTGCTGAAGGCACCTACTCAGTATCTTTTCCTCTTTATC

ACTCTGCATTGGTGAATTTAATCCTCTCCTTTGTGTTCAACTTTTGTGTGCTTTTAAAATCAGCTTTATT

CTAAGCAAATCTGTGTCTACTTTAAAAAACTGGAAATGGAAAAAAAAATAAATCTTTGCCAAATCCTTCA

GA

>NM_004331.3 Homo sapiens BCL2 interacting protein 3 like (BNIP3L), transcript variant 1, mRNA

GGACTCGGCTTGTTGTGTTGCTGCCTGAGTGCCGGAGACGGTCCTGCTGCTGCCGCAGTCCTGCCAGCTG

TCCGACAATGTCGTCCCACCTAGTCGAGCCGCCGCCGCCCCTGCACAACAACAACAACAACTGCGAGGAA

AATGAGCAGTCTCTGCCCCCGCCGGCCGGCCTCAACAGTTCCTGGGTGGAGCTACCCATGAACAGCAGCA

ATGGCAATGATAATGGCAATGGGAAAAATGGGGGGCTGGAACACGTACCATCCTCATCCTCCATCCACAA

TGGAGACATGGAGAAGATTCTTTTGGATGCACAACATGAATCAGGACAGAGTAGTTCCAGAGGCAGTTCT

CACTGTGACAGCCCTTCGCCACAAGAAGATGGGCAGATCATGTTTGATGTGGAAATGCACACCAGCAGGG

ACCATAGCTCTCAGTCAGAAGAAGAAGTTGTAGAAGGAGAGAAGGAAGTCGAGGCTTTGAAGAAAAGTGC

GGACTGGGTATCAGACTGGTCCAGTAGACCCGAAAACATTCCACCCAAGGAGTTCCACTTCAGACACCCT

AAACGTTCTGTGTCTTTAAGCATGAGGAAAAGTGGAGCCATGAAGAAAGGGGGTATTTTCTCCGCAGAAT

TTCTGAAGGTGTTCATTCCATCTCTCTTCCTTTCTCATGTTTTGGCTTTGGGGCTAGGCATCTATATTGG

AAAGCGACTGAGCACACCCTCTGCCAGCACCTACTGAGGGAAAGGAAAAGCCCCTGGAAATGCGTGTGAC

CTGTGAAGTGGTGTATTGTCACAGTAGCTTATTTGAACTTGAGACCATTGTAAGCATGACCCAACCTACC

ACCCTGTTTTTACATATCCAATTCCAGTAACTCTCAAATTCAATATTTTATTCAAACTCTGTTGAGGCAT

TTTACTAACCTTATACCCTTTTTGGCCTGAAGACATTTTAGAATTTCCTAACAGAGTTTACTGTTGTTTA

GAAATTTGCAAGGGCTTCTTTTCCGCAAATGCCACCAGCAGATTATAATTTTGTCAGCAATGCTATTATC

TCTAATTAGTGCCACCAGACTAGACCTGTATCATTCATGGTATAAATTTTACTCTTGCAACATAACTACC

ATCTCTCTCTTAAAACGAGATCAGGTTAGCAAATGATGTAAAAGAAGCTTTATTGTCTAGTTGTTTTTTT

TCCCCCAAGACAAAGGCAAGTTTCCCTAAGTTTGAGTTGATAGTTATTAAAAAGAAAACAAAACAAAAAA

AAAAGGCAAGGCACAACAAAAAAATATCCTGGGCAATAAAAAAAATATTTTAAACCAGCTTTGGAGCCAC

TTTTTTGTCTAAGCCTCCTAATAGCGTCTTTTAATTTATAGGAGGCAAACTGTATAAATGATAGGTATGA

AATAGAATAAGAAGTAAAATACATCAGCAGATTTTCATACTAGTATGTTGTAATGCTGTCTTTTCTATGG

TGTAGAATCTTTCTTTCTGATAAGGAACGTCTCAGGCTTAGAAATATATGAAATTGCTTTTTGAGATTTT

TGCGTGTGTGTTTGATATTTTTTACGATAATTAGCTGCATGTGAATTTTTCATGACCTTCTTTACATTTT

TTATTTTTTATTTCTTTATTTTTTTTTCTCTAAGAAGAGGCTTTGGAATGAGTTCCAATTTGTGATGTTA

ATACAGGCTTCTTGTTTTAGGAAGCATCACCTATACTCTGAAGCCTTTAAACTCTGAAGAGAATTGTTTC

AGAGTTATTCCAAGCACTTGTGCAACTTGGAAAAACAGACTTGGGTTGTGGGAACAGTTGACAGCGTTCT

GAAAAGATGCCATTTGTTTCCTTCTGATCTCTCACTGAATAATGTTTACTGTACAGTCTTCCCAAGGTGA

TTCCTGCGACTGCAGGCACTGGTCATTTTCTCATGTAGCTGTCTTTTCAGTTATGGTAAACTCTTAAAGT

TCAGAACACTCAACAGATTCCTTCAGTGATATACTTGTTCGTTCATTTCTAAAATGTGAAGCTTTAGGAC

CAAATTGTTAGAAAGCATCAGGATGACCAGTTATCTCGAGTAGATTTTCTTGGATTTCAGAACATCTAGC

ATGACTCTGAAGGATACCACATGTTTTATATATAAATAATTACTGTTTATGATATAGACATTGATATTGA

CTATTTAGAGAACCGTTGTTAATTTTAAAACTAGCAATCTATAAAGTGCACCAGGTCAACTTGAATAAAA

ACACTATGACAGACAGGTTTGCCAGTTTGCAGAAACTAACTCTTTTCTCACATCAACATTTGTAAAATTG

ATGTGTTATAGTGGAAAATAACATATAGATTAAACAAAATTTTTATCTTTTTTCAAGAATATAGCTGGCT

ATCTTTAAGAAAGATGATATATCCTAGTTTTGAAAGTAATTTTCTTTTTTCTTTCTAGCATTTGATGTCT

AAATAATTTTGGACATCTTTTTCCTAGACCATGTTTCTGTCTTACTCTTAAACCTGGTAACACTTGATTT

GCCTTCTATAACCTATTTATTTCAAGTGTTCATATTTGAATTTCTTTGGGAAGAAAGTAAATCTGATGGC

TCACTGATTTTTGAAAAGCCTGAATAAAATTGGAAAGACTGGAAAGTTAGGAGAACTGACTAGCTAAACT

GCTACAGTATGCAATTTCTATTACAATTGGTATTACAGGGGGGAAAAGTAAAATTACACTTTACCTGAAA

GTGACTTCTTACAGCTAGTGCATTGTGCTCTTTCCAAGTTCAGCAGCAGTTCTATCAGTGGTGCCACTGA

AACTGGGTATATTTATGATTTCTTTCAGCGTTAAAAAGAAACATAGTGTTGCCCTTTTTCTTAAAGCATC

AGTGAAATTATGGAAAATTACTTAAAACGTGAATACATCATCACAGTAGAATTTATTATGAGAGCATGTA

GTATGTATCTGTAGCCCTAACACATGGGATGAACGTTTTACTGCTACACCCAGATTTGTGTTGAACGAAA

ACATTGTGGTTTGGAAAGGAGAATTCAACAATTAATAGTTGAAATTGTGAGGTTAATGTTTAAAAAGCTT

TACACCTGTTTACAATTTGGGGACAAAAAGGCAGGCTTCATTTTTCATATGTTTGATGAAAACTGGCTCA

AGATGTTTGTAAATAGAATCAAGAGCAAAACTGCACAAACTTGCACATTGGAAAGTGCAACAAGTTCCCG

TGATTGCAGTAAAAATATTTACTATTCTAAAAAAATGAGAATTGAAGACTTAGCCAGTCAGATAAGTTTT

TTCATGAACCCGTTGTGGAAATTATTGGAATTAACTGAGCCAAAGTGATTATGCATTCTTCATCTATTTT

AGTTAGCACTTTGTATCGTTATATACAGTTTACAATACATGTATAACTTGTAGCTATAAACATTTTGTGC

CATTAAAGCTCTCACAAAACTT

>NM_005831.5 Homo sapiens calcium binding and coiled-coil domain 2 (CALCOCO2), transcript variant 3, mRNA

ACTCTGCCCTGTTGCTGTCGCGCCGCTGCTGGTTGCTGTCCCTGGACCCCTACCATGGAGGAGACCATCA

AAGATCCCCCCACATCAGCTGTCTTGCTGGATCACTGTCATTTCTCTCAGGTCATCTTTAACAGTGTGGA

GAAGTTCTACATCCCTGGAGGGGACGTCACATGTCATTATACCTTCACCCAGCATTTCATCCCTCGTCGA

AAGGATTGGATTGGCATCTTTAGAGTGGGGTGGAAGACAACCCGTGAGTATTACACCTTCATGTGGGTTA

CTTTGCCCATTGACCTAAACAACAAATCAGCTAAACAGCAGGAAGTCCAATTCAAAGCTTACTACCTGCC

CAAGGATGATGAGTATTACCAGTTCTGCTATGTGGATGAGGATGGTGTGGTCCGGGGAGCAAGTATTCCT

TTCCAATTCCGTCCAGAAAATGAGGAAGACATCCTGGTTGTTACCACTCAGGGAGAGGTGGAAGAGATTG

AGCAGCACAACAAGGAGCTTTGCAAAGAAAACCAGGAGCTGAAGGACAGCTGTATCAGCCTCCAGAAGCA

GAACTCAGACATGCAGGCTGAGCTCCAAAAGAAGCAGGAGGAGCTAGAAACCCTACAGAGCATCAATAAG

AAGTTGGAACTGAAAGTGAAAGAACAGAAGGACTATTGGGAGACAGAGCTGCTTCAACTGAAAGAACAAA

ACCAGAAGATGTCCTCAGAAAATGAGAAGATGGGAATCAGAGTGGATCAGCTTCAGGCCCAGCTGTCAAC

TCAAGAGAAAGAAATGGAGAAGCTTGTTCAGGGAGATCAAGATAAGACAGAGCAGTTAGAGCAGCTGAAA

AAGGAAAATGACCACCTCTTTCTCAGTTTAACTGAACAGAGGAAGGACCAGAAGAAGCTCGAGCAGACAG

TGGAGCAAATGAAGCAGAATGAAACTACTGCAATGAAGAAACAACAGGAATTAATGGATGAAAACTTTGA

CCTGTCAAAAAGACTGAGTGAGAACGAAATTATATGTAATGCTCTGCAGAGACAGAAAGAGAGATTGGAA

GGAGAAAATGATCTTTTGAAGAGGGAGAACAGCAGATTGCTCAGTTACATGGGTCTGGATTTTAATTCTT

TGCCGTATCAAGTACCTACTTCAGATGAAGGAGGCGCAAGACAAAATCCAGGACTTGCCTATGGAAACCC

ATATTCTGGTATCCAAGAAAGTTCTTCCCCCAGCCCGCTCTCCATCAAGAAATGCCCTATCTGCAAAGCA

GATGATATTTGTGATCACACCTTGGAGCAACAGCAGATGCAGCCCCTTTGTTTCAATTGTCCAATTTGTG

ACAAGATCTTCCCAGCTACAGAGAAGCAGATCTTTGAAGACCACGTGTTCTGCCACTCTCTCTGAGTATC

CCAACCTCTTGGATGTATACAGAGATTTTATAGAATAGAACCTATAGCTTCTACCATGAGTTATATGAGT

CAAGATCCTGCCTAACCTGAAATTATTAGGGATTTACTCAGCCCTGCTGCCGCTAACAGTGGAGTTATGT

CACTGATCTGAAGGTCACTGTTAAGGGCTTCTGCTGCCATCCTTGTGGGTTGCTACCTTTAAGTCGCATA

ACTCTAGCTGTATCATCCTCTCACCTGTCATTCTTCTGAGGGTCTCAGTACAAGGGCCCTGGGATGGAGC

CAACCTGGGTATTCACAACAGGCCTGACTTGATACTAAGTGATTAGTTTTCCAAGTTGTCCCACTGCCAT

TCAAAGTCAGCCCTTGAGTGTATTTGTTCTCAGTCCTAACCCTGGGGCCAGAGATTGGTCCGAGGTTGAG

AATTCCTTCCTCCTCATCCTTGGTGTTGCTTTCTCCAAATGATTGTTTTAGACTAGCCAAAAATGCCGTG

GCAAAGAGCTCAGAAATCCAATTTGGATACCAAAGGTTTCTCATGTTAATTTCTCAGCCCCCAAAGAAGC

ATCTTACTCCTGAACCTTAGACAGGAAGTATTGTTTCAGTCACAGAAAGCTTTTCTGGGTACCTCTGGTT

AGCACTTTCTACTCTCTGATATTTCCTATGTACATAGCTTTTATTGTTGTAAATCCTTTCTTAATGGTTA

AATAGGATTGTTAGCAACTATGGGTTTGCAGTTTTCTGAGTAGGTGAGTTTTGAATATGGGTAAATCAGA

ATAATGAGACAACTTGTTAATCTCTTTAATACTAAAAATAAATTACTCTTCTATTTCAGGGACTTAGGTA

ATTTAAAATAAACCTTCAATTTATGGTCTTCTGTTTTGAAGCTCATGGGAAAATTGTGATCAAAAGGGCT

ATGGGAAGGGCAGACCCCGCCAATGATTTCTCTTCACCTGTCTTAAGATTAAATAAAAAAGAGTGTCCTG

GCAGTTATCTTGAGGTGGGGAAGGAGGTGATGAAACATTAGTTTGTGAAATCCAAGGCCCTGGCTTGCTT

TCTTTCTTTTTTTTTTTTTTTTTTGAAACAGTCTCTCTCTGTCACCCAGGCTGGCGTGCAATGGCGCAG

TTGACTCACTACAGCCTCTGCCTCCCAGGTTCAAGCGATTCTCATGCCTTAGCCTCCCAAGTAGCTGGGA

TTACAGGTGTGTGCCGCAATGCCCAGCTAATTTTTGTGTTTTTAGTAGAGACAGGGTTTCACTATGTTGG

CCAGGCTGGTCTCGAACTCCTGGCCTCACGTGATCTGTCCACTTCAGCCGTCCAAAGTGCTGGGATTACA

AGCGTGAGCCACTGTGCTGGGCCCGAGGCCCTGACTTCTTGCTGTAACTTTCCATGCATTTTTTTTAAAA

GGAGCAGTGTGGATTTTCGCACCCTTTGTGAACTAAGTTCAATGCGCTCTATCCAAATTTGCCTAATTGA

ACTATAAGAAAGTAATAATTCCATTTTCTATCCCCTCAGGGACTGAACAAATGGAAATAACTCCCAGGCA

GTATCAGGTGGTCACTACAGAGACTTCCACAAAAACTTTTGAATGATGTGAAACACGATGTCATGAATAA

GGGTTGAGCCAACTATAGCTCTGTGTTCCTACTGGGCTTTCCCTAATGTGGTTGGGAGTTATGCCCTAGA

CTAACTGTATTGTCCTAGTCACAGCTCCTTGCTTTGATTTCATCCTTGATAAAATGAAGATGAAACTTAC

ACTACTTCTCCAAGCCTTTTGCTGTCTTAAGAATAAGACCTGAGATTAACACTAACCCTAGAATAGAAAT

GTAATAGGGAGATGGTAATAAAGGAGTTTTTCTGGCACATACCCTCCCTACAGAATTTCTGTTGCTCCCC

AGATCCAGTGAAGAATTGCAGTTTCATTTATTTTGTACCAGTCAGCTCTTAATTAAGTACATGAATGGAG

AGGAACAGTGGTGCACATAATCCAAATCAGTGAATACCATTTTCTGGTGAATTACCCACCCCTTTGCCCC

TGCTACCCCGAGGGTTACCATGATTGTCAACAGCAGCAGGAGCCCTTCCACAGGGCTTGGTAAAAAAACC

AGTTGAGGTGTTAATGACCCTTTTTGCTGGGTGTAAAACAAAGCATCTTTAACCACTGTTCATTATCCCC

AGCTGCTCTTACCAAGGCTTTGAAGGGGGAAATTATGCTCTAGGCAGCCACTAGTAGTAAACAAT

>NM_172226.3 Homo sapiens calcium/calmodulin dependent protein kinase kinase 2 (CAMKK2), transcript variant 7, mRNA

AGAGCAAGCTGAGCCGAGCCGAGCCGAGCTGGGGGCGCAGAGCGCGGGAGGCGGCGGCGCGGAGCCC

AGGTGGCTCCGCTGCCGGATGGGAGTGCCCCAGTGTGCTGGATGAAGCTGGCGCATGCACCATGTCATCA

TGTGTCTCTAGCCAGCCCAGCAGCAACCGGGCCGCCCCCCAGGATGAGCTGGGGGGCAGGGGCAGCAGCA

GCAGCGAAAGCCAGAAGCCCTGTGAGGCCCTGCGGGGCCTCTCATCCTTGAGCATCCACCTGGGCATGGA

GTCCTTCATTGTGGTCACCGAGTGTGAGCCGGGCTGTGCTGTGGACCTCGGCTTGGCGCGGGACCGGCCC

CTGGAGGCCGATGGCCAAGAGGTCCCCCTTGACACCTCCGGGTCCCAGGCCCGGCCCCACCTCTCCGGTC

GCAAGCTGTCTCTGCAAGAGCGGTCCCAGGGTGGGCTGGCAGCCGGTGGCAGCCTGGACATGAACGGACG

CTGCATCTGCCCGTCCCTGCCCTACTCACCCGTCAGCTCCCCGCAGTCCTCGCCTCGGCTGCCCCGGCGG

CCGACAGTGGAGTCTCACCACGTCTCCATCACGGGTATGCAGGACTGTGTGCAGCTGAATCAGTATACCC

TGAAGGATGAAATTGGAAAGGGCTCCTATGGTGTCGTCAAGTTGGCCTACAATGAAAATGACAATACCTA

CTATGCAATGAAGGTGCTGTCCAAAAAGAAGCTGATCCGGCAGGCCGGCTTTCCACGTCGCCCTCCACCC

CGAGGCACCCGGCCAGCTCCTGGAGGCTGCATCCAGCCCAGGGGCCCCATTGAGCAGGTGTACCAGGAAA

TTGCCATCCTCAAGAAGCTGGACCACCCCAATGTGGTGAAGCTGGTGGAGGTCCTGGATGACCCCAATGA

GGACCATCTGTACATGGTGTTCGAACTGGTCAACCAAGGGCCCGTGATGGAAGTGCCCACCCTCAAACCA

CTCTCTGAAGACCAGGCCCGTTTCTACTTCCAGGATCTGATCAAAGGCATCGAGTACTTACACTACCAGA

AGATCATCCACCGTGACATCAAACCTTCCAACCTCCTGGTCGGAGAAGATGGGCACATCAAGATCGCTGA

CTTTGGTGTGAGCAATGAATTCAAGGGCAGTGACGCGCTCCTCTCCAACACCGTGGGCACGCCCGCCTTC

ATGGCACCCGAGTCGCTCTCTGAGACCCGCAAGATCTTCTCTGGGAAGGCCTTGGATGTTTGGGCCATGG

GTGTGACACTATACTGCTTTGTCTTTGGCCAGTGCCCATTCATGGACGAGCGGATCATGTGTTTACACAG

TAAGATCAAGAGTCAGGCCCTGGAATTTCCAGACCAGCCCGACATAGCTGAGGACTTGAAGGACCTGATC

ACCCGTATGCTGGACAAGAACCCCGAGTCGAGGATCGTGGTGCCGGAAATCAAGCTGCACCCCTGGGTCA

CGAGGCATGGGGCGGAGCCGTTGCCGTCGGAGGATGAGAACTGCACGCTGGTCGAAGTGACTGAAGAGGA

GGTCGAGAACTCAGTCAAACACATTCCCAGCTTGGCAACCGTGATCCTGGTGAAGACCATGATACGTAAA

CGCTCCTTTGGGAACCCATTCGAGGGCAGCCGGCGGGAGGAACGCTCACTGTCAGCGCCTGGAAACTTGC

TCACGAAGCAAGGCAGCGAAGACAACCTCCAGGGCACCGACCCGCCCCCCGTGGGGGAGGAGGAAGTGCT

CTTGTGAGAGGCAGTCCCTGCGTGGAAAGTTGCTGGGCCCCCGCCCCCGGCTCCCCCGCACGCATGCATC

CACTGCGGCCGGAGGAGGCCATGGAGCCCGAGTAGCTGCCTGGATCGCTCGACCTCGCATGCGCGCCGCG

TCGCCTCTGGGGGGCTGCTGCACCGCGTTTCCATAGCAGCATGTCCTACGGAAACCCAGCACGTGTGTAG

AGCCTCGATCGTCATCTCTGGTTATTTGTTTTTTCCTTTGTTGTTTTAAAGGGGACAAAAAAAAAAAAAG

GACTTGACTCCATGACGTCGACCGTGGCCGCTGGCTGGCTGGACAGGCGGGTGTGAGGAGTTGCAGACCC

AAACCCACGTGCATTTTGGGACAATTGCTTTTTAAAACGTTTTTATGCCAAAAATCCTTCATTGTGATTT

TCAGAACCACGTCAGATATACCAAGTGACTGTGTGTGGGGTTTGACAACTGTGGAAAGGCGAGCAGAAAA

CTCCGGCGGTCTGAGGCCATGGAGGTGGTTGCTGCATTTGAGAGGGAGTAGGGGGCTAGATGTGGCTCCT

AGTGCAAACCGGAAACCATGGCACCTTCCAGAGCCGTGGTCTCAAGGAGTCAGAGCAGGGCTGGCCCTCA

GTAGCTGCAGGGAGCTTTGATGCAACTTATTTGTAAGAAGGATTTTTAAATTTTTTATGGGTAGAATTGT

AGTCAGGAAAACAGAAAGGGCTTGAAATTTAATAAGTGCTGCTGGAAGGGGATTTTCCAAGCCTGGAAGG

GTATTCAGCAGCTGTGGTGGGGAAACATTTCTCCTGAAAGACTGAACGTGTTTCTTCATGACAGCTGCTC

AAAGCAGGTTTCTGAGATAGCTGACCGAGCTCTGGTAAATCTCTTTGTCAAATTACGAAAACTTCAGGGT

GAAATCCTATGCTTCCATGTACATTACATGGCTTAAGATTAAACAAAAACATTTTTCAAGTCTCTAACTA

GAGTGAACTCTAGAGCACAGTAGTTCAGAAACTATTTAGAGCTTCCAGGATATATTTCACAGCTTCAGGC

ATGTGATCAGTTAGAGCCGATGAAACCTATGCCCGCCTGTATATATATTAGCAGCTTAGCTAGTTCATAA

CCTGTATATTCTAAAGACTGCTAAGGTTTTGTTTTCATTTTAAATCCTAGCTGATTGTTGTGGTCAATGA

AATACCCAGTTTCTGGAGGGCCAGGTGGGAAATGCTTTCACTGGACCAACACACAAATGATCATCCTGAG

GATCTGAGCTTCCCTAGACTCCACACAATAACCTTGGGGCACCCTTTTAGAGAAGACTGTTGAAACCCAC

AGCACTCGTTGGGGTATGAGGAAACCAGGGCTTGGCACAGGAAGTTCCCCTTTGTAGCTAAAAGTCCAGA

AAGAAAGGGTTCATCTTTTTGACTTCCAACTGATATTGGGAAGTTTGGTTGAGGTTCAAGTGTGACTCCT

TCCAGAGCCACAGGTAGGGGAGTGTGAAGTTGAGGGGGAGGAAAGCTGGAAGGACTCTGCCTTGGGAGAT

TCCCAGCTCTGCTTTCCAGCGCTTGGTGGAATCTGGGCTGGGGAAAGACGGCACCGGGAAACTCTGCTTC

CCCATTGTTTCCATCTGATCAGCTGTGGTGTGAGGACTTCTCAGACAAAGGCAAGGCCTCGTGCCCCTGC

CCAGCCCATTCATGGAGCCCTGGGCCTTCTTGGCTTCCATAGATCCTAAGCTCTTGACTGTAGTTTAGCC

AGACTTGTTTTGCTATCTTATAAGCAGTTCAGAATTAGGGAATGCTGGTTTTGAAGAGCAAAGGACAGGT

AGTCTAGAGAGGGTCGTCTGGCCTGCTTGCTGGGTCTTTGTAACCCAGCACTTCCTCTTGCCCTCCTGGC

TTTATGTTTATGGGGAGAGGACTCAATAGCTCCACCCCTTCTGGCACCAGATGGGGCTTGGTTAGTTTGC

AATAAGCACCTTGCAGAGGTTAAAGCCAGCGGGTCCCTAGTCTTAGGCCCAGCCTGCTTGTGTGGGCTCT

GGCCTGGCCTGGTGGCTGGCCCAGGGGGCAGCAGTGCTTAGAGCTTCTGCAGGGCTTCTCTTGTTTACAC

AGCTGCATCAGACAATGCCATTTCTCCCCACCACGGAACCTTCCATCTAAGATTTCTTCCAGGGAATGCC

AGCAATCAGGCAGCACCCAGCTGTGGGGGCAGTGGGGTGGGGGAGACCCACATTGATGACTTTTTTTTTT

TCTTTTAATGAAGAAACACCAAAGAAAGCTGTGGAAAGGACCTGCCCCACATGAAAAGGATAAGCCAAGA

TGGCTGTAAACACAGAGCATTTGAGCTGCCACTCTTGGAGCACATTGATTTTTCAAAAGCCAGCTCTGTC

AGGAAAGGAGGTGCTGTTATGAGCAGCTCTTCCAGTGGGCAAAGAGGACGCCCATAATTTCTTCCATTGC

TAGCTCATCTGTGGGACCAATTTGGTGTAAGCAACCTGTGGCCTGCACTTGTGGCCTCGAAGGAAGCACA

AACCCTCCATCCACTTCCCATTTCCTCTGCCCTTTTCCACCTCCCCCTTCCATCCCACCAGCTGCCAGTG

GCTCCCAGAAAGCCTTATTGAGCCCCTTGTTGACACTTGGGGCTGCGGAGGCCTCTCCCTACTGGTCTGG

CCTTTCCTGAGAGGCAGGTCTTCCGTCCTCAGAGCCTTTCTGGAACAAGGAGAATGCCTGTGCAGGTGGA

CACACAGGCCTGGCCTGTCGCTCTCACTTGTCTTCCAGCGGGGAGCTTCACGTTGCCGAGTGGAAGAACC

ATGACCTCCACTTGCTTCCAAGGTGCTAGGGAAGTTTCAGGGTACGCTGGTTCCCCTCTCCAGCTGGAGG

CCGAGTTTCTGGGGACTGCAGATTTTTCTACTCTGTGATCGATTCAATGCCCGATGCTTCTGTTTCATTC

CCGACCCTTTCTACTATGCATTTTCCTTTTATCAGGTGTATAAAGTTAAATACTGTGTATTTATCACTAA

AAAGTACATGAACTTAAGAGACAACTAAGCCTTTCGTGTTTTTCCACAGGTGTTTAAGCTTCTCTGTACA

GTTGAAATAAACAGACAGCAAAATGGTGCCAA

>NM_001024649.2 Homo sapiens calnexin (CANX), transcript variant 2, mRNA

AGCGGTGGCCGAGGCCTCTTGGTTCTGCGGCACGTGACGGTCGGGCCGCCTCCGCCTCTCTCTTTACTGC

GGCGCGGGGCAAGATCATGGAAGGGAAGTGGTTGCTGTGTATGTTACTGGTGCTTGGAACTGCTATTGTT

GAGGCTCATGATGGACATGATGATGATGTGATTGATATTGAGGATGACCTTGACGATGTCATTGAAGAGG

TAGAAGACTCAAAACCAGATACCACTGCTCCTCCTTCATCTCCCAAGGTTACTTACAAAGCTCCAGTTCC

AACAGGGGAAGTATATTTTGCTGATTCTTTTGACAGAGGAACTCTGTCAGGGTGGATTTTATCCAAAGCC

AAGAAAGACGATACCGATGATGAAATTGCCAAATATGATGGAAAGTGGGAGGTAGAGGAAATGAAGGAGT

CAAAGCTTCCAGGTGATAAAGGACTTGTGTTGATGTCTCGGGCCAAGCATCATGCCATCTCTGCTAAACT

GAACAAGCCCTTCCTGTTTGACACCAAGCCTCTCATTGTTCAGTATGAGGTTAATTTCCAAAATGGAATA

GAATGTGGTGGTGCCTATGTGAAACTGCTTTCTAAAACACCAGAACTCAACCTGGATCAGTTCCATGACA

AGACCCCTTATACGATTATGTTTGGTCCAGATAAATGTGGAGAGGACTATAAACTGCACTTCATCTTCCG

ACACAAAAACCCCAAAACGGGTATCTATGAAGAAAAACATGCTAAGAGGCCAGATGCAGATCTGAAGACC

TATTTTACTGATAAGAAAACACATCTTTACACACTAATCTTGAATCCAGATAATAGTTTTGAAATACTGG

TTGACCAATCTGTGGTGAATAGTGGAAATCTGCTCAATGACATGACTCCTCCTGTAAATCCTTCACGTGA

AATTGAGGACCCAGAAGACCGGAAGCCCGAGGATTGGGATGAAAGACCAAAAATCCCAGATCCAGAAGCT

GTCAAGCCAGATGACTGGGATGAAGATGCCCCTGCTAAGATTCCAGATGAAGAGGCCACAAAACCCGAAG

GCTGGTTAGATGATGAGCCTGAGTACGTACCTGATCCAGACGCAGAGAAACCTGAGGATTGGGATGAAGA

CATGGATGGAGAATGGGAGGCTCCTCAGATTGCCAACCCTAGATGTGAGTCAGCTCCTGGATGTGGTGTC

TGGCAGCGACCTGTGATTGACAACCCCAATTATAAAGGCAAATGGAAGCCTCCTATGATTGACAATCCCA

GTTACCAGGGAATCTGGAAACCCAGGAAAATACCAAATCCAGATTTCTTTGAAGATCTGGAACCTTTCAG

AATGACTCCTTTTAGTGCTATTGGTTTGGAGCTGTGGTCCATGACCTCTGACATTTTTTTTGACAACTTT

ATCATTTGTGCTGATCGAAGAATAGTTGATGATTGGGCCAATGATGGATGGGGCCTGAAGAAAGCTGCTG

ATGGGGCTGCTGAGCCAGGCGTTGTGGGGCAGATGATCGAGGCAGCTGAAGAGCGCCCGTGGCTGTGGGT

AGTCTATATTCTAACTGTAGCCCTTCCTGTGTTCCTGGTTATCCTCTTCTGCTGTTCTGGAAAGAAACAG

ACCAGTGGTATGGAGTATAAGAAAACTGATGCACCTCAACCGGATGTGAAGGAAGAGGAAGAAGAGAAGG

AAGAGGAAAAGGACAAGGGAGATGAGGAGGAGGAAGGAGAAGAGAAACTTGAAGAGAAACAGAAAAGTGA

TGCTGAAGAAGATGGTGGCACTGTCAGTCAAGAGGAGGAAGACAGAAAACCTAAAGCAGAGGAGGATGAA

ATTTTGAACAGATCACCAAGAAACAGAAAGCCACGAAGAGAGTGAAACAATCTTAAGAGCTTGATCTGTG

ATTTCTTCTCCCTCCTCCCCTGCAAGAGTGGTCCTAGGAGAGGACCTGGCACACCTTAGGTTGACATTCA

GAAAACTTCAAGACATCACCATCAGCAGGCTCCAGTTGAACACTAGTCTGTGTAACTTTAAACATCTAGC

AGTAAATACTTGCAGTTGTGATATAAAGGACCCTGTTTCTGTAGAAAAGAAAACATTTAACATAATGGTT

GTGAAATGTAACATGAAGCAAACTAACTTTTTTTTTTTTAACATCTTTGTTTTTAAAATAGAATGATAGA

ACTTTGCCAGTCTTTAAGATCTTGGCTTAATTTAATGTATTAATCTGTTTGTGCAAACATAATACCACCA

TTTAAAAATGTTAGGGAGATGAGTTGCAGTTTTTATAATAGATTTTTTTTAAAGTTTGGTATTGTAAAAC

ATTCACACCTCTGTCCCTCAAAATTGATAATTACGTTTAAAGTGCAGTCATTTGTGGTTAGAATCTTGTT

TTGTTTGCTTCCATTATTGAGTTCCTCCTAAGGAAATTGAGGAGAGGGACTGAATAGAAGCCCAAATTCA

TATAAAAGTTGCGTTTAAGTTGTATTAAAAATAGATATATAAGAAAAAATTCTTTCACTTGATGTTTGTT

AGACCAGAAAGTGTGTGTTCTGTAGCTCAGTTCCCAGACAGCTTTTTAGGTAGTGGAGGAGGTGGCTT

CATGTGGCACTTGGGCATTTATATTCCACTTGGGAGGGTCAGGCTGTGGCCTTCTGGAGCAGGTGGCTTG

TTAAGGAATGCTAGCAGGGCATGGCACGTGAGCTCCGGAATAGATGTCTTCATCACTTCTTCCACTGTGT

GTTGACACTGTTTTCCTTACCTATTTCCTCAGATCCCCAGCTTTCTCCTCTGCTATGCATTTTCTTCACA

GTGCAGCTTGCAGTCCGTTGCTGAAAATGATTATAAGCCCTGCATAATGTTAAGCTTTATTGTGATTACG

TGTATGTTTCTTCTTTCTTTTAAGCAGACCCATACCTTTCCAGGGTCAAAGTACAGAATAGAATACATTG

ATACAAAGTACAGAAAAATACTTTGATTTTTATCCATTTCTTTTACTCTGTGTAAAGACTTGAGAAGTCT

AATTCACAGGCAAACCAATACAGAATTGACTGCAGTTGAACAGACTAGAAGTATTTGTGGGAGGAGTGAC

ATGAAGCATGAGTTATCTGATTTTTTTTGTAGCTGCTATATATTTTAAGCCTTCATTTGCAATTCATGTA

ACAGTTGTGTCATAAATTACACAATAAAGCAGTCCTGTTCAAATTTTTTTTTAACGTGGCTTGTAGAATT

TTTAAAAAAGTGATCTTAGGTTTGTTTTTTCATGCGGATGCAGATGGGTGCTATCAGAGCCTCTCCCAC

ACCACTATAGTGTAATAATGTTATTATTACTCTACACTGAAACGTATTCAGAGTTAGATATTATTTTAGC

TTCAGTTGTTCTTTAGAGGCTTTCAAATGTACCGATGATACTGTTTCTTGCACTGAATATATAAACACTC

CACAGTGTTTATATTGGGAAGATATTGGGAAGGAAATATATTTGTAAAAGATGAAGGCTGTATCTATTTT

TTTTTCTTTTTAAAGTTTGTTCACTTAAATTCTTTTGAGGATGGGATGTATTTTTCTTGCTGTTCAGTGC

TTTTTCCTTTTCATCTGTTGTTCTGTGGTCACAGTGACCTTAGCTACATAGCAGACTTTCCCAAATGTAT

TGATTACAAATAAACAGTTGTTACTTAGCAAGACCTGAAAATATGTCTGCAGGTTTCTCCTTGAAGCAAA

TGTGTGGGATCATTGCATTTCCAGAAATCTGCCTCCTTCACCCTCCGTTGACAGTATATGTCATGCCTCA

CTTTCTTCTAGCTGAGCTTTAAATCATTAGAGCTTAAATTGTCAGATCGTTCATTGCCTTTCCAGGGTTA

TTTAGTAAAGTTTGTTGAAAACAAAAACGCCTTTTCTTGGTTCTTTTTCAGTTATTTTGAAGGTCCAGC

ATCCTGATTAAATGTCTGACACATTAATGAATGACCAGCAGCAGCTTTCAGCTCTTAAAAAGACACTTAT

ATTTTGATTTTACATGCTGGTTACCTGTTCCATTGTTGTCAAATGCCCACTCTCCATCAGATGTGTTCCT

CCATTTTCTTATCCACAAAGTACTCCTCACTTTTCAATTTGTCATGTTACTAAATGGTGTTACATTAAAG

CCCTGTGTTAAGTGTCTGCTTTTGACTGAATTTCTTCATAGTAACCTTCAGTGTGTGTGTGTGACTATGT

TCAATTAGTGGGTTGATCTTCGTATAATTGGCCACTATGTGAGAGTTCACTACTAGGCAGAAACTATTAT

GGACAGTGAAATAATGACTTTTATCTCACCACGTGAGTTTGATGCAGTCTTTTCTGTCTAGCCCTTGCCT

CTTCCTGCCCATGTGATTGCGGTGCAGTAGTTTCTGTTGTATAATAGTGTGGACAGCAGCTCAGAAAAGG

AGGGAATGCTACTGATAATTTGTAGATAATATTCTTTAAGACTTAGGGGAACCATTGAACTTTGAAATTT

TTATTAGAAAATTATTTGTTCAGAATCAGACTCCATTATTTTACATATACTTAAATACTTTAGGGGTATT

TTTGAAAGTTAGCCCAGTTTTTTATGTGCTATTAAATTTTTAGATTACATACTAAAGAAAAGTATGTACA

CAGAATGTAGTGCTCCTAGTTACTATTTTTTCTATTAAGAAATAGTTTAGTTCTGGTGTAAAATTTGTTT

GAATGCTAAAAAAAAAAAAAGCAGGACTGCATTATGGCACTTTTGCCTTGGTGGCCCACTTTCTCCATTTA

AAACATTAGGATTTGTATTTTTCTGCTGCGTTTGTATGAAGACATATTTGATTGTTGTTTTCTCTTGATT

TTAAAATAAAACCTCATGAGCCCTAGTAAAAA

>NM_001198868.2 Homo sapiens calpain 1 (CAPN1), transcript variant 1, mRNA

AGCCCGGCCCCTCCTCAGAGCAGCTGCCGCAGCCCGAGGATGTCGGAGGAGATCATCACGCCGGTGTACT

GCACTGGGGTGTCAGCCCAAGTGCAGAAGCAGCGGGCCAGGGAGCTGGGCCTGGGCCGCCATGAGAATGC

CATCAAGTACCTGGGCCAGGATTATGAGCAGCTGCGGGTGCGATGCCTGCAGAGTGGGACCCTCTTCCGT

GATGAGGCCTTCCCCCCGGTACCCCAGAGCCTGGGTTACAAGGACCTGGGTCCCAATTCCTCCAAGACCT

ATGGCATCAAGTGGAAGCGTCCCACGGAACTGCTGTCAAACCCCCAGTTCATTGTGGATGGAGCTACCCG

CACAGACATCTGCCAGGGAGCACTGGGGGACTGCTGGCTCTTGGCGGCCATCGCCTCCCTCACTCTCAAC

GACACCCTCCTGCACCGAGTGGTTCCGCACGGCCAGAGCTTCCAGAATGGCTATGCCGGCATCTTCCATT

TCCAGCTGTGGCAATTTGGGGAGTGGGTGGACGTGGTCGTGGATGACCTGCTGCCCATCAAGGACGGGAA

GCTAGTGTTCGTGCACTCTGCCGAAGGCAACGAGTTCTGGAGCGCCCTGCTTGAGAAGGCCTATGCCAAG

GTAAATGGCAGCTACGAGGCCCTGTCAGGGGGCAGCACCTCAGAGGGCTTTGAGGACTTCACAGGCGGGG

TTACCGAGTGGTACGAGTTGCGCAAGGCTCCCAGTGACCTCTACCAGATCATCCTCAAGGCGCTGGAGCG

GGGCTCCCTGCTGGGCTGCTCCATAGACATCTCCAGCGTTCTAGACATGGAGGCCATCACTTTCAAGAAG

TTGGTGAAGGGCCATGCCTACTCTGTGACCGGGGCCAAGCAGGTGAACTACCGAGGCCAGGTGGTGAGCC

TGATCCGGATGCGGAACCCCTGGGGCGAGGTGGAGTGGACGGGAGCCTGGAGCGACAGCTCCTCAGAGTG

GAACAACGTGGACCCATATGAACGGGACCAGCTCCGGGTCAAGATGGAGGACGGGGAGTTCTGGATGTCA

TTCCGAGACTTCATGCGGGAGTTCACCCGCCTGGAGATCTGCAACCTCACACCCGACGCCCTCAAGAGCC

GGACCATCCGCAAATGGAACACCACACTCTACGAAGGCACCTGGCGGCGGGGGAGCACCGCGGGGGGCTG

CCGAAACTACCCAGCCACCTTCTGGGTGAACCCTCAGTTCAAGATCCGGCTGGATGAGACGGATGACCCG

GACGACTACGGGGACCGCGAGTCAGGCTGCAGCTTCGTGCTCGCCCTTATGCAGAAGCACCGTCGCCGCG

AGCGCCGCTTCGGCCGCGACATGGAGACTATTGGCTTCGCGGTCTACGAGGTCCCTCCGGAGCTGGTGGG

CCAGCCGGCCGTACACTTGAAGCGTGACTTCTTCCTGGCCAATGCGTCTCGGGCGCGCTCAGAGCAGTTC

ATCAACCTGCGAGAGGTCAGCACCCGCTTCCGCCTGCCACCCGGGGAGTATGTGGTGGTGCCCTCCACCT

TCGAGCCCAACAAGGAGGGCGACTTCGTGCTGCGCTTCTTCTCAGAGAAGAGTGCTGGGACTGTGGAGCT

GGATGACCAGATCCAGGCCAATCTCCCCGATGAGCAAGTGCTCTCAGAAGAGGAGATTGACGAGAACTTC

AAGGCCCTCTTCAGGCAGCTGGCAGGGGAGGACATGGAGATCAGCGTGAAGGAGTTGCGGACAATCCTCA

ATAGGATCATCAGCAAACACAAAGACCTGCGGACCAAGGGCTTCAGCCTAGAGTCGTGCCGCAGCATGGT

GAACCTCATGGATCGTGATGGCAATGGGAAGCTGGGCCTGGTGGAGTTCAACATCCTGTGGAACCGCATC

CGGAATTACCTGTCCATCTTCCGGAAGTTTGACCTGGACAAGTCGGGCAGCATGAGTGCCTACGAGATGC

GGATGGCCATTGAGTCGGCAGGCTTCAAGCTCAACAAGAAGCTGTACGAGCTCATCATCACCCGCTACTC

GGAGCCCGACCTGGCGGTCGACTTTGACAATTTCGTTTGCTGCCTGGTGCGGCTAGAGACCATGTTCCGA

TTTTTCAAAACTCTGGACACAGATCTGGATGGAGTTGTGACCTTTGACTTGTTTAAGTGGTTGCAGCTGA

CCATGTTTGCATGAGGCAGGGACTCGGTCCCCCTTGCCGTGCTCCCCTCCCTCCTCGTCTGCCAAGCCTC

GCCTCCTACCACACCACACCAGGCCACCCCAGCTGCAAGTGCCTTCCTTGGAGCAGAGAGGCAGCCTCGT

CCTCCTGTCCCCTCTCCTCCCAGCCACCATCGTTCATCTGCTCCGGGCAGAACTGTGTGGCCCCTGCCTG

TGCCAGCCATGGGCTCGGGATGGACTCCCTGGGCCCCACCCATTGCCAAGCCAGGAAGGCAGCTTTCGCT

TGTTCCTGCCTCGGGACAGCCCCGGGTTTCCCCAGCATCCTGATGTGTCCCCTCTCCCCACTTCAGAGGC

CACCCACTCAGCACCACCGGCCTGGCCTTGCCTGCAGACTATAAACTATAACCACTAGCTCGACACAGTC

TGCAGTCCAGGCGTGTGGAGCCGCCTCCCGGCTCGGGGAGGCCCCGGGGCTGGGAACGCCTGTGCCTTCC

TGCGCCGAAGCCAACGCCCCCTCTGTCCTTCCCTGGCCCTGCTGCCGACCAGGAGCTGCCCAGCCTGTGG

GCGGTCGGCCTTCCCTCCTTCGCTCCTTTTTTATATTAGTGATTTTAAAGGGGACTCTTCAGGGACTTGT

GTACTGGTTATGGGGGTGCCAGAGGCACTAGGCTTGGGGTGGGGAGGTCCCGTGTTCCATATAGAGGAAC

CCCAAATAATAAAAGGCCCCACATCTGTCTGTGA

>NM_023083.4 Homo sapiens calpain 10 (CAPN10), transcript variant 1, mRNA

AGACTAGCGGGCCGGCGTACTGGCCTGGTCCAGCACCTGCGGGGCCCTCGGGCTTGGAGGGCTGGGCCGG

GCGGGGAACGGGCGGGGCGGGCCGGAGGCGGCGGCTGACTCGCCTTCTCTCCGGGGCTGCGACCCCG

AGGCAACCGGCTGCAGATGGGAGCCCGCGGAGCCGAGGATGCGGGCGGGCCGGGGCGCGACGCCGGCGAG

GGAGCTGTTCCGGGACGCCGCCTTCCCCGCCGCGGACTCCTCGCTCTTCTGCGACTTGTCTACGCCGCTG

GCCCAGTTCCGCGAGGACATCACGTGGAGGCGGCCCCAGGAGATTTGTGCCACACCCCGGCTGTTTCCAG

ATGACCCACGGGAAGGGCAGGTGAAGCAGGGGCTGCTGGGGGATTGCTGGTTCCTGTGTGCCTGCGCCGC

GCTGCAGAAGAGCAGGCACCTCCTGGACCAGGTCATTCCTCCGGGACAGCCGAGCTGGGCCGACCAGGAG

TACCGGGGCTCCTTCACCTGTCGCATTTGGCAGTTTGGACGCTGGGTGGAGGTGACCACAGATGACCGCC

TGCCGTGCCTTGCAGGGAGACTCTGTTTCTCCCGCTGCCAGAGGGAGGATGTGTTCTGGCTCCCCTTACT

GGAAAAGGTCTACGCCAAGGTCCATGGGTCCTACGAGCACCTGTGGGCCGGGCAGGTGGCGGATGCCCTG

GTGGACCTGACCGGCGGCCTGGCAGAAAGATGGAACCTGAAGGGCGTAGCAGGAAGCGGAGGCCAGCAGG

ACAGGCCAGGCCGCTGGGAGCACAGGACTTGTCGGCAGCTGCTCCACCTGAAGGACCAGTGTCTGATCAG

CTGCTGCGTGCTCAGCCCCAGAGCAGGTGCCCGGGAGCTGGGGGAGTTCCATGCCTTCATTGTCTCGGAC

CTGCGGGAGCTCCAGGGTCAGGCGGGCCAGTGCATCCTGCTGCTGCGGATCCAGAACCCCTGGGGCCGGC

GGTGCTGGCAGGGGCTCTGGAGAGAGGGGGGTGAAGGGTGGAGCCAGGTAGATGCAGCGGTAGCATCTGA

GCTCCTGTCCCAGCTCCAGGAAGGGGAGTTCTGGGTGGAGGAGGAGGAGTTCCTCAGGGAGTTTGACGAG

CTCACCGTTGGCTACCCGGTCACGGAGGCCGGCCACCTGCAGAGCCTCTACACAGAGAGGCTGCTCTGCC

ATACGCGGGCGCTGCCTGGGGCCTGGGTCAAGGGCCAGTCAGCAGGAGGCTGCCGGAACAACAGCGGCTT

TCCCAGCAACCCCAAATTCTGGCTGCGGGTCTCAGAACCGAGTGAGGTGTACATTGCCGTCCTGCAGAGA

TCCAGGCTGCACGCGGCGGACTGGGCAGGCCGGGCCCGGGCACTGGTGGGTGACAGTCATACTTCGTGGA

GCCCAGCGAGCATCCCGGGCAAGCACTACCAGGCTGTGGGTCTGCACCTCTGGAAGGTAGAGAAGCGGCG

GGTCAATCTGCCTAGGGTCCTGTCCATGCCCCCCGTGGCTGGCACCGCGTGCCATGCATACGACCGGGAG

GTCCACCTGCGTTGTGAGCTCTCACCGGGCTACTACCTGGCTGTCCCCAGCACCTTCCTGAAGGACGCGC

CAGGGGAGTTCCTGCTCCGAGTCTTCTCTACCGGGCGAGTCTCCCTTAGCGCCATCAGGGCAGTGGCCAA

GAACACCACCCCCGGGGCAGCCCTGCCTGCGGGGGAGTGGGGGACCGTGCAGCTACGGGGTTCTTGGAGA

GTCGGCCAGACGGCGGGGGGCAGCAGGAACTTTGCCTCATACCCCACCAACCCCTGCTTCCCCTTCTCGG

TCCCCGAGGGCCCTGGCCCCCGCTGCGTCCGCATCACTCTGCATCAGCACTGCCGGCCCAGTGACACCGA

GTTCCACCCCATCGGCTTCCATATCTTCCAGGTCCCAGAGGGTGGAAGGAGCCAGGACGCACCCCCACTG

CTGCTGCAGGAGCCGCTGCTGAGCTGCGTGCCACATCGCTACGCCCAGGAGGTGAGCCGGCTCTGCCTCC

TGCCTGCGGGCACCTACAAGGTTGTGCCCTCCACCTACCTGCCGGACACAGAGGGGGCCTTCACAGTGAC

CATCGCAACCAGGATTGACAGGCCATCCATTCACAGCCAGGAGATGCTGGGCCAGTTCCTCCAAGAGGTC

TCCATCATGGCAGTGATGAAAACCTAACAGGGTGGCCCCCTGTGCCAGCTCAGGTGACTGGAGCCCGAGG

GCCTGACAGGTTCCCAGCAGCTGGGCCGGCCAGCCTTGCACTGTGGGGGCTGGTCCTGAGTCTTGGCCTG

CCTCCCAGCCCTGCCAGGAGGCTGCGGCCTAGGGGTCCACGGGAAGCCTCCGTCAGGAGAGACGCAGCCC

TGGGGGCCAGCTGGTGCTGCAAGGAAGGGTGGGAAGCTTGCTGGCTTCTGTTGCGCCACTGAGACGGCAG

AGACCCCAGGATCCCAGAGCTTCCCAGGATCCCTCCCAGATCCTCTGCTGACTCCATATGGAGGCCTCAC

ACCCAGAGGGTAGGGCAGCAGATCTTCTTTATAACTATTTATTGTTCGAATCACTTTTAGGATGTAACTT

TATAAATAAACATGAGCGCTGATGATTTGCA

>NM_001748.5 Homo sapiens calpain 2 (CAPN2), transcript variant 1, mRNA

AGTGGCCGCAGCAGCGCGCCGGGCCCTGGCCGCGCCCCAGCCGAGCGCAGCGCGGAGTCGCCCCGACCTT

TCTCTGCGCAGTACGGCCGCCGGGACCGCAGCATGGCGGGCATCGCGGCCAAGCTGGCGAAGGACCGGGA

GGCGGCCGAGGGGCTGGGCTCCCACGACAGGGCCATCAAGTACCTCAACCAGGACTACGAGGCGCTGCGG

AACGAGTGCCTGGAGGCCGGGACGCTCTTCCAGGACCCGTCCTTCCCGGCCATCCCCTCGGCCCTGGGCT

TCAAGGAGTTGGGGCCCTACTCCAGCAAAACCCGGGGCATCGAGTGGAAGCGCCCCACGGAGATCTGCGC

TGACCCCCAGTTTATCATTGGAGGAGCCACCCGCACAGACATCTGCCAAGGAGCCCTGGGTGACTGCTGG

CTGCTGGCAGCCATTGCCTCCCTCACCTTGAATGAAGAAATCCTGGCTCGAGTCGTCCCCCTAAACCAGA

GCTTCCAGGAAAACTATGCAGGGATCTTTCACTTCCAGTTCTGGCAATACGGCGAGTGGGTGGAGGTGGT

GGTGGATGACAGGCTGCCCACCAAGGACGGGGAGCTGCTCTTTGTGCATTCAGCCGAAGGGAGCGAGTTC

TGGAGCGCCCTGCTGGAGAAGGCATACGCCAAGATCAACGGATGCTATGAAGCGCTATCAGGGGGTGCCA

CCACTGAGGGCTTCGAAGACTTCACCGGAGGCATTGCTGAGTGGTATGAGTTGAAGAAGCCCCCTCCCAA

CCTGTTCAAGATCATCCAGAAAGCTCTGCAAAAAGGCTCTCTCCTTGGCTGCTCCATCGACATCACCAGC

GCCGCGGACTCGGAGGCCATCACGTTTCAGAAGCTGGTGAAGGGGCACGCGTACTCGGTCACCGGAGCCG

AGGAGGTTGAAAGTAACGGAAGCCTACAGAAACTGATCCGCATCCGAAATCCCTGGGGAGAAGTGGAGTG

GACAGGGCGGTGGAATGACAACTGCCCAAGCTGGAACACTATAGACCCAGAGGAGAGGGAAAGGCTGACC

AGACGGCATGAAGATGGAGAATTCTGGATGTCTTTCAGTGACTTCCTGAGGCACTATTCCCGCCTGGAGA

TCTGTAACCTGACCCCAGACACTCTCACCAGCGATACCTACAAGAAGTGGAAACTCACCAAAATGGATGG

GAACTGGAGGCGGGGCTCCACCGCGGGAGGTTGCAGGAACTACCCGAACACATTCTGGATGAACCCTCAG

TACCTGATCAAGCTGGAGGAGGAGGATGAGGACGAGGAGGATGGGGAGAGCGGCTGCACCTTCCTGGTGG

GGCTCATTCAGAAGCACCGACGGCGGCAGAGGAAGATGGGCGAGGACATGCACACCATCGGCTTTGGCAT

CTATGAGGTTCCAGAGGAGTTAAGTGGGCAGACCAACATCCACCTCAGCAAAAACTTCTTCCTGACGAAT

CGCGCCAGGGAGCGCTCAGACACCTTCATCAACCTCCGGGAGGTGCTCAACCGCTTCAAGCTGCCGCCAG

GAGAGTACATTCTCGTGCCTTCCACCTTCGAACCCAACAAGGATGGGGATTTCTGCATCCGGGTCTTTTC

TGAAAAGAAAGCTGACTACCAAGCTGTCGATGATGAAATCGAGGCCAATCTTGAAGAGTTCGACATCAGC

GAGGATGACATTGATGATGGATTCAGGAGACTGTTTGCCCAGTTGGCAGGAGAGGATGCGGAGATCTCTG

CCTTTGAGCTGCAGACCATCCTGAGAAGGGTTCTAGCAAAGCGCCAAGATATCAAGTCAGATGGCTTCAG

CATCGAGACATGCAAAATTATGGTTGACATGCTAGATTCGGACGGGAGTGGCAAGCTGGGGCTGAAGGAG

TTCTACATTCTCTGGACGAAGATTCAAAAATACCAAAAAATTTACCGAGAAATCGACGTTGACAGGTCTG

GTACCATGAATTCCTATGAAATGCGGAAGGCATTAGAAGAAGCAGGTTTCAAGATGCCCTGTCAACTCCA

CCAAGTCATCGTTGCTCGGTTTGCAGATGACCAGCTCATCATCGATTTTGATAATTTTGTTCGGTGTTTG

GTTCGGCTGGAAACGCTATTCAAGATATTTAAGCAGCTGGATCCCGAGAATACTGGAACAATAGAGCTCG

ACCTTATCTCTTGGCTCTGTTTCTCAGTACTTTGAAGTTATAACTAATCTGCCTGAAGACTTCTCATGAT

GGAAAATCAGCCAAGGACTAAGCTTCCATAGAAATACACTTTGTATCTGGACCTCAAAATTATGGGAACA

TTTACTTAAACGGATGATCATAGCTGAAAATAATGATACTGTCAATTTGAGATAGCAGAAGTTTCACACA

TCAAAGTAAAAGATTTGCATATCATTATACTAAATGCAAATGAGTCGCTTAACCCTTGACAAGGTCAAAG

AAAGCTTTAAATCTGTAAATAGTATACACTTTTTACTTTTACACACTTTCCTGTTCATAGCAATATTAAA

TCAGGAAAAAAAAATGCAGGGAGGTATTTAACAGCTGAGCAAAAACATTGAGTCGCTCTCAAAGGACACG

AGGCCCTTGGCAGGGAATATTTAAAGCAACTTCAAGTTTAAAATGCAGCTGTTGATTCTACCAAACAACA

GTCCAAGATTACCATTTCCCATGAGCCAACTGGGAAACATGGTATATCATGAAGTAATCTTGTCAAGGCA

TCTGGAGAGTCCAGGAGAGAAGACTCACCTCTGTCGCTTGGGTTAAACAAGAGACAGGTTTTGTAGAATA

TTGATTGGTAATAGTAAATCGTTCTCCTTACAATCAAGTTCTTGACCCTATTCGGCCTTATACATCTGGT

CTTACAAAGACCAAAGGGATCCTGCGCTTGATCAACTGAACCAGTATGCCAAAACCAGGCATCCAATTTG

TAAACCAATTATGATAAAGGACAAAATAAGCTGTTTGCCACCTCAAAACTTTATGAACTTCACCACCACT

AGTGTCTGTCCATGGAGTTAGAGGGGACATCACTTAGAAGTTCTTATAGAAAGGACACAAGTTTGTTTCC

TGGCTTTACCTTGGGAAAATGCTAGCAACATTATAGAAATTTTGCCTTGTTGCCTTATCTTCTTCCAAAT

GTACTGTTAAATAAAAATAAAGGGTTACCCCATGCAATCACACCATGCCATGTTTTCCTTCCTGGAGGGC

AGCCCCACAGGACGGTTTATGAGCACACAATTATAGCTTGTTTCTACTTTAACAAGGTATGCTGCCTCTG

TAAATTCATGTATTCAAAGGAAAAGACACCTTGCCTATAATTAAAATGTGGAACTATAAAATTTTTTAAA

ATCCAA

>NM_001003962.3 Homo sapiens calpain small subunit 1 (CAPNS1), transcript variant 2, mRNA

GAGTCACCGGCCCCGCCCTCCGGAGCCGGACGCTGCGGGAGGCCCGGGAGCGGCAGTGGAACCGACTCCC

AGAACTCCGGACGTGTGCGGCGTGAGTCGCAGCCATGTTCCTGGTTAACTCGTTCTTGAAGGGCGGCGGC

GGCGGCGGCGGGGGAGGCGGGGGCCTGGGTGGGGGCCTGGGAAATGTGCTTGGAGGCCTGATCAGCGGGG

CCGGGGGCGGCGGCGGCGGCGGCGGCGGCGGCGGTGGTGGAGGCGGCGGTGGCGGTGGAACGGCCAT

GCGCATCCTAGGCGGAGTCATCAGCGCCATCAGCGAGGCGGCTGCGCAGTACAACCCGGAGCCCCCGCCC

CCACGCACACATTACTCCAACATTGAGGCCAACGAGAGTGAGGAGGTCCGGCAGTTCCGGAGACTCTTTG

CCCAGCTGGCTGGAGATGACATGGAGGTCAGCGCCACAGAACTCATGAACATTCTCAATAAGGTTGTGAC

ACGACACCCTGATCTGAAGACTGATGGTTTTGGCATTGACACATGTCGCAGCATGGTGGCCGTGATGGAT

AGCGACACCACAGGCAAGCTGGGCTTTGAGGAATTCAAGTACTTGTGGAACAACATCAAAAGGTGGCAGG

CCATATACAAACAGTTCGACACTGACCGATCAGGGACCATTTGCAGTAGTGAACTCCCAGGTGCCTTTGA

GGCAGCAGGGTTCCACCTGAATGAGCATCTCTATAACATGATCATCCGACGCTACTCAGATGAAAGTGGG

AACATGGATTTTGACAACTTCATCAGCTGCTTGGTCAGGCTGGACGCCATGTTCCGTGCCTTCAAATCTC

TTGACAAAGATGGCACTGGACAAATCCAGGTGAACATCCAGGAGTGGCTGCAGCTGACTATGTATTCCTG

AACTGGAGCCCCAGACCCGCCCCCTCACTGCCTTGCTATAGGAGTCACCTGGAGCCTCGGTCTCTCCCAG

GGCCGATCCTGTCTGCAGTCACATCTTTGTGGGGCCTGCTGACCCACAAGCTTTTGTTCTCTCAGTACTT

GTTACCCAGCTTCTCAACATCCAGGGCCCAATTTGCCCTGCCTGGAGTTCCCCCTGGCTCTAGGACACTC

TAACAAGCTCTGTCCACGGGTCTCCCCATTCCCACCAGGCCCTGCACACACCCACTCCGTAACCTCTCCC

CTGTACCTGTGCCAAGCCTAGCACTTGTGATGCCTCCATGCCCCGAGGGCCCTCTCTCAGTTCTGGGAGG

ATGACTCCAGTCCCTGCACGCCCTGGCACACCCTTCACGGTTGCTACCCAGGCGGCCAAGCTCCAGACCG

TGCCAGACCCAGGTGCCCCAGTGCCTTTGTCTATATTCTGCTCCCAGCCTGCCAGGCCCAGGAGGAAATA

AACATGCCCCAGTTGCTGATCTCTA

>NM_033292.4 Homo sapiens caspase 1 (CASP1), transcript variant alpha, mRNA

CAGTCACACAAGAAGGGAGGAGAGAAAAGCCATGGCCGACAAGGTCCTGAAGGAGAAGAGAAAGCTGTTT

ATCCGTTCCATGGGTGAAGGTACAATAAATGGCTTACTGGATGAATTATTACAGACAAGGGTGCTGAACA

AGGAAGAGATGGAGAAAGTAAAACGTGAAAATGCTACAGTTATGGATAAGACCCGAGCTTTGATTGACTC

CGTTATTCCGAAAGGGGCACAGGCATGCCAAATTTGCATCACATACATTTGTGAAGAAGACAGTTACCTG

GCAGGGACGCTGGGACTCTCAGCAGATCAAACATCTGGAAATTACCTTAATATGCAAGACTCTCAAGGAG

TACTTTCTTCCTTTCCAGCTCCTCAGGCAGTGCAGGACAACCCAGCTATGCCCACATCCTCAGGCTCAGA

AGGGAATGTCAAGCTTTGCTCCCTAGAAGAAGCTCAAAGGATATGGAAACAAAAGTCGGCAGAGATTTAT

CCAATAATGGACAAGTCAAGCCGCACACGTCTTGCTCTCATTATCTGCAATGAAGAATTTGACAGTATTC

CTAGAAGAACTGGAGCTGAGGTTGACATCACAGGCATGACAATGCTGCTACAAAATCTGGGGTACAGCGT

AGATGTGAAAAAAAATCTCACTGCTTCGGACATGACTACAGAGCTGGAGGCATTTGCACACCGCCCAGAG

CACAAGACCTCTGACAGCACGTTCCTGGTGTTCATGTCTCATGGTATTCGGGAAGGCATTTGTGGGAAGA

AACACTCTGAGCAAGTCCCAGATATACTACAACTCAATGCAATCTTTAACATGTTGAATACCAAGAACTG

CCCAAGTTTGAAGGACAAACCGAAGGTGATCATCATCCAGGCCTGCCGTGGTGACAGCCCTGGTGTGGTG

TGGTTTAAAGATTCAGTAGGAGTTTCTGGAAACCTATCTTTACCAACTACAGAAGAGTTTGAGGATGATG

CTATTAAGAAAGCCCACATAGAGAAGGATTTTATCGCTTTCTGCTCTTCCACACCAGATAATGTTTCTTG

GAGACATCCCACAATGGGCTCTGTTTTTATTGGAAGACTCATTGAACATATGCAAGAATATGCCTGTTCC

TGTGATGTGGAGGAAATTTTCCGCAAGGTTCGATTTTCATTTGAGCAGCCAGATGGTAGAGCGCAGATGC

CCACCACTGAAAGAGTGACTTTGACAAGATGTTTCTACCTCTTCCCAGGACATTAAAATAAGGAAACTGT

ATGAATGTCTGTGGGCAGGAAGTGAAGAGATCCTTCTGTAAAGGTTTTTGGAATTATGTCTGCTGAATAA

TAAACTTTTTTGAAATAATAAATCTGGTAGAAAAATGAAAACTTGTCCTCATTTTTCTCCCACACTGAAG

AAACAGGGACTGGAACTTAGAGTGACTAAGGAATTT

>NM_004346.4 Homo sapiens caspase 3 (CASP3), transcript variant 1, mRNA

AGTGCAGACGCGGCTCCTAGCGGATGGGTGCTATTGTGAGGCGGTTGTAGAAGAGTTTCGTGAGTGCTCG

CAGCTCATACCTGTGGCTGTGTATCCGTGGCCACAGCTGGTTGGCGTCGCCTTGAAATCCCAGGCCGTGA

GGAGTTAGCGAGCCCTGCTCACACTCGGCGCTCTGGTTTTCGGTGGGTGTGCCCTGCACCTGCCTCTTCC

CCCATTCTCATTAATAAAGGTATCCATGGAGAACACTGAAAACTCAGTGGATTCAAAATCCATTAAAAAT

TTGGAACCAAAGATCATACATGGAAGCGAATCAATGGACTCTGGAATATCCCTGGACAACAGTTATAAAA

TGGATTATCCTGAGATGGGTTTATGTATAATAATTAATAATAAGAATTTTCATAAAAGCACTGGAATGAC

ATCTCGGTCTGGTACAGATGTCGATGCAGCAAACCTCAGGGAAACATTCAGAAACTTGAAATATGAAGTC

AGGAATAAAAATGATCTTACACGTGAAGAAATTGTGGAATTGATGCGTGATGTTTCTAAAGAAGATCACA

GCAAAAGGAGCAGTTTTGTTTGTGTGCTTCTGAGCCATGGTGAAGAAGGAATAATTTTTGGAACAAATGG

ACCTGTTGACCTGAAAAAAATAACAAACTTTTTCAGAGGGGATCGTTGTAGAAGTCTAACTGGAAAACCC

AAACTTTTCATTATTCAGGCCTGCCGTGGTACAGAACTGGACTGTGGCATTGAGACAGACAGTGGTGTTG

ATGATGACATGGCGTGTCATAAAATACCAGTGGAGGCCGACTTCTTGTATGCATACTCCACAGCACCTGG

TTATTATTCTTGGCGAAATTCAAAGGATGGCTCCTGGTTCATCCAGTCGCTTTGTGCCATGCTGAAACAG

TATGCCGACAAGCTTGAATTTATGCACATTCTTACCCGGGTTAACCGAAAGGTGGCAACAGAATTTGAGT

CCTTTTCCTTTGACGCTACTTTTCATGCAAAGAAACAGATTCCATGTATTGTTTCCATGCTCACAAAAGA

ACTCTATTTTTATCACTAAAGAAATGGTTGGTTGGTGGTTTTTTTTAGTTTGTATGCCAAGTGAGAAGAT

GGTATATTTGGTACTGTATTTCCCTCTCATTTTGACCTACTCTCATGCTGCAGAGGGTACTTTAAGACAT

ACTCCTTCCATCAAATAGAACCACTATGAAGCTACCTCAAACTTCCAGTCAGGTAGTTGCAATTGAATTA

AATTAGGAATAAATAAAAATGGATACTGGTGCAGTCATTATGAGAGGCAATGATTGTTAATTTACAGCTT

TCATGATTAGCAAGTTACAGTGATGCTGTGCTATGAATTTTCAAGTAATTGTGAAAAAGTTAAACATTGA

AGTAATGAATTTTTATGATATTCCCCCCACTTAAGACTGTGTATTCTAGTTTTGTCAAACTGTAGAAATG

ATGATGTGGAAGAACTTAGGCATCTGTGGGCATGGTCAAAGGCTCAAACCTTTATTTTAGAATTGATATA

CACGGATGACTTAACTGCATTTTTAGACCATTTATCTGGGATTATGGTTTTGTGATGTTTGTCCTGAACA

CTTTTGTTGTAAAAAAATAATAATAATGTTTAATATTGAGAAAGAAACTAATATTTTATGTGAGAGAAAG

TGTGAGCAAACTAACTTGACTTTTAAGGCTAAAACTTAACATTCATAGAGGGGTGGAGTTTTAACTGTAA

GGTGCTACAATGCCCCTGGATCTACCAGCATAAATATCTTCTGATTTGTCCCTATGCATATCAGTTGAGC

TTCATATACCAGCAATATATCTGAAGAGCTATTATATAAAAACCCCAAACTGTTGATTATTAGCCAGGTA

ATGTGAATAAATTCTATAGGAACATATGAAAATACAACTTAAATAATAAACAGTGGAATATAAGGAAAGC

AATAAATGAATGGGCTGAGCTGCCTGTAACTTGAGAGTAGATGGTTTGAGCCTGAGCAGAGACATGACTC

AGCCTGTTCCATGAAGGCAGAGCCATGGACCACGCAGGAAGGGCCTACAGCCCATTTCTCCATACGCACT

GGTATGTGTGGATGATGCTGCCAGGGCGCCATCGCCAAGTAAGAAAGTGAAGCAAATCAGAAACTTGTGA

AGTGGAAATGTTCTAAAGGTGGTGAGGCAATAAAAATCATAGTACTCTTTGTAGCAAAATTCTTAAGTAT

GTTATTTTCTGTTGAAGTTTACAATCAAAGGAAAATAGTAATGTTTTATACTGTTTACTGAAAGAAAAAG

ACCTATGAGCACATAGGACTCTAGACGGCATCCAGCCGGAGGCCAGAGCTGAGCCCTCAGCCCGGGAGGC

AGGCTCCAGGCCTCAGCAGGTGCGGAGCCGTCACTGCACCAAGTCTCACTGGCTGTCAGTATGACATTTC

ACGGGAGATTTCTTGTTGCTCAAAAAATGAGCTCGCATTTGTCAATGACAGTTTCTTTTTTCTTACTAGA

CCTGTAACTTTTGTAAATACACATAGCATGTAATGGTATCTTAAAGTGTGTTTCTATGTGACAATTTTGT

ACAAATTTGTTATTTTCCATTTTTATTTCAAAATATACATTCAAACTTAAAATTA

>NM_001225.4 Homo sapiens caspase 4 (CASP4), transcript variant alpha, mRNA

ACTCTGAGGCTCTTTCCAACGCTGTAAAAAAGGACAGAGGCTGTTCCCTATGGCAGAAGGCAACCACAGA

AAAAAGCCACTTAAGGTGTTGGAATCCCTGGGCAAAGATTTCCTCACTGGTGTTTTGGATAACTTGGTGG

AACAAAATGTACTGAACTGGAAGGAAGAGGAAAAAAAGAAATATTACGATGCTAAAACTGAAGACAAAGT

TCGGGTCATGGCAGACTCTATGCAAGAGAAGCAACGTATGGCAGGACAAATGCTTCTTCAAACCTTTTTT

AACATAGACCAAATATCCCCCAATAAAAAAGCTCATCCGAATATGGAGGCTGGACCACCTGAGTCAGGAG

AATCTACAGATGCCCTCAAGCTTTGTCCTCATGAAGAATTCCTGAGACTATGTAAAGAAAGAGCTGAAGA

GATCTATCCAATAAAGGAGAGAAACAACCGCACACGCCTGGCTCTCATCATATGCAATACAGAGTTTGAC

CATCTGCCTCCGAGGAATGGAGCTGACTTTGACATCACAGGGATGAAGGAGCTACTTGAGGGTCTGGACT

ATAGTGTAGATGTAGAAGAGAATCTGACAGCCAGGGATATGGAGTCAGCGCTGAGGGCATTTGCTACCAG

ACCAGAGCACAAGTCCTCTGACAGCACATTCTTGGTACTCATGTCTCATGGCATCCTGGAGGGAATCTGC

GGAACTGTGCATGATGAGAAAAAACCAGATGTGCTGCTTTATGACACCATCTTCCAGATATTCAACAACC

GCAACTGCCTCAGTCTGAAGGACAAACCCAAGGTCATCATTGTCCAGGCCTGCAGAGGTGCAAACCGTGG

GGAACTGTGGGTCAGAGACTCTCCAGCATCCTTGGAAGTGGCCTCTTCACAGTCATCTGAGAACCTAGAG

GAAGATGCTGTTTACAAGACCCACGTGGAGAAGGACTTCATTGCTTTCTGCTCTTCAACGCCACACAACG

TGTCCTGGAGAGACAGCACAATGGGCTCTATCTTCATCACACAACTCATCACATGCTTCCAGAAATATTC

TTGGTGCTGCCACCTAGAGGAAGTATTTCGGAAGGTACAGCAATCATTTGAAACTCCAAGGGCCAAAGCT

CAAATGCCCACCATAGAACGACTGTCCATGACAAGATATTTCTACCTCTTTCCTGGCAATTGAAAATGGA

AGCCACAAGCAGCCCAGCCCTCCTTAATCAACTTCAAGGAGCACCTTCATTAGTACAGCTTGCATATTTA

ACATTTTGTATTTCAATAAAAGTGAAGACAAACGAA

>NM_001228.4 Homo sapiens caspase 8 (CASP8), transcript variant 1, mRNA

GTGCTCTGAGTTTTTGGTTTCTGTTTCACCTTGTGTCTGAGCTGGTCTGAAGGCTGGTTGTTCAGACTGA

GCTTCCTGCCTGCCTGTACCCCGCCAACAGCTTCAGAAGAAGGTGACTGGTGGCTGCCTGAGGAATACCA

GTGGGCAAGAGAATTAGCATTTCTGGAGCATCTGCTGTCTGAGCAGCCCCTGGGTGCGTCCACTTTCTGG

GCACGTGAGGTTGGGCCTTGGCCGCCTGAGCCCTTGAGTTGGTCACTTGAACCTTGGGAATATTGAGATT

ATATTCTCCTGCCTTTTAAAAAGATGGACTTCAGCAGAAATCTTTATGATATTGGGGAACAACTGGACAG

TGAAGATCTGGCCTCCCTCAAGTTCCTGAGCCTGGACTACATTCCGCAAAGGAAGCAAGAACCCATCAAG

GATGCCTTGATGTTATTCCAGAGACTCCAGGAAAAGAGAATGTTGGAGGAAAGCAATCTGTCCTTCCTGA

AGGAGCTGCTCTTCCGAATTAATAGACTGGATTTGCTGATTACCTACCTAAACACTAGAAAGGAGGAGAT

GGAAAGGGAACTTCAGACACCAGGCAGGGCTCAAATTTCTGCCTACAGGTTCCACTTCTGCCGCATGAGC

TGGGCTGAAGCAAACAGCCAGTGCCAGACACAGTCTGTACCTTTCTGGCGGAGGGTCGATCATCTATTAA

TAAGGGTCATGCTCTATCAGATTTCAGAAGAAGTGAGCAGATCAGAATTGAGGTCTTTTAAGTTTCTTTT

GCAAGAGGAAATCTCCAAATGCAAACTGGATGATGACATGAACCTGCTGGATATTTTCATAGAGATGGAG

AAGAGGGTCATCCTGGGAGAAGGAAAGTTGGACATCCTGAAAAGAGTCTGTGCCCAAATCAACAAGAGCC

TGCTGAAGATAATCAACGACTATGAAGAATTCAGCAAAGGGGAGGAGTTGTGTGGGGTAATGACAATCTC

GGACTCTCCAAGAGAACAGGATAGTGAATCACAGACTTTGGACAAAGTTTACCAAATGAAAAGCAAACCT

CGGGGATACTGTCTGATCATCAACAATCACAATTTTGCAAAAGCACGGGAGAAAGTGCCCAAACTTCACA

GCATTAGGGACAGGAATGGAACACACTTGGATGCAGGGGCTTTGACCACGACCTTTGAAGAGCTTCATTT

TGAGATCAAGCCCCACGATGACTGCACAGTAGAGCAAATCTATGAGATTTTGAAAATCTACCAACTCATG

GACCACAGTAACATGGACTGCTTCATCTGCTGTATCCTCTCCCATGGAGACAAGGGCATCATCTATGGCA

CTGATGGACAGGAGGCCCCCATCTATGAGCTGACATCTCAGTTCACTGGTTTGAAGTGCCCTTCCCTTGC

TGGAAAACCCAAAGTGTTTTTTATTCAGGCTTGTCAGGGGGATAACTACCAGAAAGGTATACCTGTTGAG

ACTGATTCAGAGGAGCAACCCTATTTAGAAATGGATTTATCATCACCTCAAACGAGATATATCCCGGATG

AGGCTGACTTTCTGCTGGGGATGGCCACTGTGAATAACTGTGTTTCCTACCGAAACCCTGCAGAGGGAAC

CTGGTACATCCAGTCACTTTGCCAGAGCCTGAGAGAGCGATGTCCTCGAGGCGATGATATTCTCACCATC

CTGACTGAAGTGAACTATGAAGTAAGCAACAAGGATGACAAGAAAAACATGGGGAAACAGATGCCTCAGC

CTACTTTCACACTAAGAAAAAAACTTGTCTTCCCTTCTGATTGATGGTGCTATTTTGTTTGTTTTGTTTT

GTTTTGTTTTTTTGAGACAGAATCTCGCTCTGTCGCCCAGGCTGGAGTGCAGTGGCGTGATCTCGGCTCA

CCGCAAGCTCCGCCTCCCGGGTTCAGGCCATTCTCCTGCCTCAGCCTCCCGAGTAGCTGGGACTACAGGG

GCCCGCCACCACACCTGGCTAATTTTTTAAAAATATTTTTAGTAGAGACAGGGTTTCACTGTGTTAGCCA

GGGTGGTCTTGATCTCCTGACCTCGTGATCCACCCACCTCGGCCTCCCAAAGTGCTGGGATTACAGGCGT

GAGCCACCGCGCCTGGCCGATGGTACTATTTAGATATAACACTATGTTTATTTACTAATTTTCTAGATTT

TCTACTTTATTAATTGTTTTGCACTTTTTTATAAGAGCTAAAGTTAAATAGGATATTAACAACAATAACA

CTGTCTCCTTTCTCTTATGCTTAAGGCTTTGGGAATGTTTTTAGCTGGTGGCAATAAATACCAGACACGT

ACAAAATCCAGCTATGAATATAGAGGGCTTATGATTCAGATTGTTATCTATCAACTATAAGCCCACTGTT

AATATTCTATTAACTTTAATTCTCTTTCAAAGCTAAATTCCACACTACCACATTAAAAAAATTAGAAAGT

AGCCACGTATGGTGGCTCATGTCTATAATCCCAGCACTTTGGGAGGTTGAGGTGGGAGGATTGCTTGAAC

CCAAGAGGTCAAGGCTGCAGTGAGCCATGTTCACACCGCTGCACTCAAGCTTGGGTGACAGAACAAGACC

CCGTCTCAAAAAAAATTTTTTTTTTAATAAAACAAAATTTGTTTGAAATCTTTTAAAAATTCAAATGATT

TTTACAAGTTTTAAATAAGCTCTCCCCAAACTTGCTTTATGCCTTCTTATTGCTTTTATGATATATATAT

GCTTGGCTAACTATATTTGCTTTTTGCTAACAATGCTCTGGGGTCTTTTTATGCATTTGCATTTGCTCTT

TCATCTCTGCTTGGATTATTTTAAATCATTAGGAATTAAGTTATCTTTAAAATTTAAGTATCTTTTTTCA

AAAACATTTTTTAATAGAATAAAATATAATTTGATCTTATTAAA

>NM_002982.4 Homo sapiens C-C motif chemokine ligand 2 (CCL2), mRNA

GAGAGGCTGAGACTAACCCAGAAACATCCAATTCTCAAACTGAAGCTCGCACTCTCGCCTCCAGCATGAA

AGTCTCTGCCGCCCTTCTGTGCCTGCTGCTCATAGCAGCCACCTTCATTCCCCAAGGGCTCGCTCAGCCA

GATGCAATCAATGCCCCAGTCACCTGCTGTTATAACTTCACCAATAGGAAGATCTCAGTGCAGAGGCTCG

CGAGCTATAGAAGAATCACCAGCAGCAAGTGTCCCAAAGAAGCTGTGATCTTCAAGACCATTGTGGCCAA

GGAGATCTGTGCTGACCCCAAGCAGAAGTGGGTTCAGGATTCCATGGACCACCTGGACAAGCAAACCCAA

ACTCCGAAGACTTGAACACTCACTCCACAACCCAAGAATCTGCAGCTAACTTATTTTCCCCTAGCTTTCC

CCAGACACCCTGTTTTATTTTATTATAATGAATTTTGTTTGTTGATGTGAAACATTATGCCTTAAGTAAT

GTTAATTCTTATTTAAGTTATTGATGTTTTAAGTTTATCTTTCATGGTACTAGTGTTTTTTAGATACAGA

GACTTGGGGAAATTGCTTTTCCTCTTGAACCACAGTTCTACCCCTGGGATGTTTTGAGGGTCTTTGCAAG

AATCATTAATACAAAGAATTTTTTTTAACATTCCAATGCATTGCTAAAATATTATTGTGGAAATGAATAT

TTTGTAACTATTACACCAAATAAATATATTTTTGTACAAAA

>NM_001123041.3 Homo sapiens C-C motif chemokine receptor 2 (CCR2), transcript variant A, mRNA

AGATCTGTTTGGTTCAGTTGCTGAGAAGCCTGACATACCAGGACTGCCTGAGACAAGCCACAAGCTGAAC

AGAGAAAGTGGATTGAACAAGGACGCATTTCCCCAGTACATCCACAACATGCTGTCCACATCTCGTTCTC

GGTTTATCAGAAATACCAACGAGAGCGGTGAAGAAGTCACCACCTTTTTTGATTATGATTACGGTGCTCC

CTGTCATAAATTTGACGTGAAGCAAATTGGGGCCCAACTCCTGCCTCCGCTCTACTCGCTGGTGTTCATC

TTTGGTTTTGTGGGCAACATGCTGGTCGTCCTCATCTTAATAAACTGCAAAAAGCTGAAGTGCTTGACTG

ACATTTACCTGCTCAACCTGGCCATCTCTGATCTGCTTTTTCTTATTACTCTCCCATTGTGGGCTCACTC

TGCTGCAAATGAGTGGGTCTTTGGGAATGCAATGTGCAAATTATTCACAGGGCTGTATCACATCGGTTAT

TTTGGCGGAATCTTCTTCATCATCCTCCTGACAATCGATAGATACCTGGCTATTGTCCATGCTGTGTTTG

CTTTAAAAGCCAGGACGGTCACCTTTGGGGTGGTGACAAGTGTGATCACCTGGTTGGTGGCTGTGTTTGC

TTCTGTCCCAGGAATCATCTTTACTAAATGCCAGAAAGAAGATTCTGTTTATGTCTGTGGCCCTTATTTT

CCACGAGGATGGAATAATTTCCACACAATAATGAGGAACATTTTGGGGCTGGTCCTGCCGCTGCTCATCA

TGGTCATCTGCTACTCGGGAATCCTGAAAACCCTGCTTCGGTGTCGAAACGAGAAGAAGAGGCATAGGGC

AGTGAGAGTCATCTTCACCATCATGATTGTTTACTTTCTCTTCTGGACTCCCTATAATATTGTCATTCTC

CTGAACACCTTCCAGGAATTCTTCGGCCTGAGTAACTGTGAAAGCACCAGTCAACTGGACCAAGCCACGC

AGGTGACAGAGACTCTTGGGATGACTCACTGCTGCATCAATCCCATCATCTATGCCTTCGTTGGGGAGAA

GTTCAGAAGCCTTTTTCACATAGCTCTTGGCTGTAGGATTGCCCCACTCCAAAAACCAGTGTGTGGAGGT

CCAGGAGTGAGACCAGGAAAGAATGTGAAAGTGACTACACAAGGACTCCTCGATGGTCGTGGAAAAGGAA

AGTCAATTGGCAGAGCCCCTGAAGCCAGTCTTCAGGACAAAGAAGGAGCCTAGAGACAGAAATGACAGAT

CTCTGCTTTGGAAATCACACGTCTGGCTTCACAGATGTGTGATTCACAGTGTGAATCTTGGTGTCTACGT

TACCAGGCAGGAAGGCTGAGAGGAGAGAGACTCCAGCTGGGTTGGAAAACAGTATTTTCCAAACTACCTT

CCAGTTCCTCATTTTTGAATACAGGCATAGAGTTCAGACTTTTTTTAAATAGTAAAAATAAAATTAAAGC

TGAAAACTGCAACTTGTAAATGTGGTAAAGAGTTAGTTTGAGTTACTATCATGTCAAACGTGAAAATGCT

GTATTAGTCACAGAGATAATTCTAGCTTTGAGCTTAAGAATTTTGAGCAGGTGGTATGTTTGGGAGACTG

CTGAGTCAACCCAATAGTTGTTGATTGGCAGGAGTTGGAAGTGTGTGATCTGTGGGCACATTAGCCTATG

TGCATGCAGCATCTAAGTAATGATGTCGTTTGAATCACAGTATACGCTCCATCGCTGTCATCTCAGCTGG

ATCTCCATTCTCTCAGGCTTGCTGCCAAAAGCCTTTTGTGTTTTGTTTTGTATCATTATGAAGTCATGCG

TTTAATCACATTCGAGTGTTTCAGTGCTTCGCAGATGTCCTTGATGCTCATATTGTTCCCTATTTTGCCA

GTGGGAACTCCTAAATCAAGTTGGCTTCTAATCAAAGCTTTTAAACCCTATTGGTAAAGAATGGAAGGTG

GAGAAGCTCCCTGAAGTAAGCAAAGACTTTCCTCTTAGTCGAGCCAAGTTAAGAATGTTCTTATGTTGCC

CAGTGTGTTTCTGATCTGATGCAAGCAAGAAACACTGGGCTTCTAGAACCAGGCAACTTGGGAACTAGAC

TCCCAAGCTGGACTATGGCTCTACTTTCAGGCCACATGGCTAAAGAAGGTTTCAGAAAGAAGTGGGGACA

GAGCAGAACTTTCACCTTCATATATTTGTATGATCCTAATGAATGCATAAAATGTTAAGTTGATGGTGAT

GAAATGTAAATACTGTTTTTAACAACTATGATTTGGAAAATAAATCAATGCTATAACTATGTTGATAAAA

GATTTAAAAACAA

>NM_172361.3 Homo sapiens CD46 molecule (CD46), transcript variant l, mRNA

GCGGCTCGGGCCACGCCCACCTGTCCTGCAGCACTGGATGCTTTGTGAGTTGGGGATTGTTGCGTCCCAT

ATCTGGACCCAGAAGGGACTTCCCTGCTCGGCTGGCTCTCGGTTTCTCTGCTTTCCTCCGGAGAAATAAC

AGCGTCTTCCGCGCCGCGCATGGAGCCTCCCGGCCGCCGCGAGTGTCCCTTTCCTTCCTGGCGCTTTCCT

GGGTTGCTTCTGGCGGCCATGGTGTTGCTGCTGTACTCCTTCTCCGATGCCTGTGAGGAGCCACCAACAT

TTGAAGCTATGGAGCTCATTGGTAAACCAAAACCCTACTATGAGATTGGTGAACGAGTAGATTATAAGTG

TAAAAAAGGATACTTCTATATACCTCCTCTTGCCACCCATACTATTTGTGATCGGAATCATACATGGCTA

CCTGTCTCAGATGACGCCTGTTATAGAGAAACATGTCCATATATACGGGATCCTTTAAATGGCCAAGCAG

TCCCTGCAAATGGGACTTACGAGTTTGGTTATCAGATGCACTTTATTTGTAATGAGGGTTATTACTTAAT

TGGTGAAGAAATTCTATATTGTGAACTTAAAGGATCAGTAGCAATTTGGAGCGGTAAGCCCCCAATATGT

GAAAAGGTTTTGTGTACACCACCTCCAAAAATAAAAAATGGAAAACACACCTTTAGTGAAGTAGAAGTAT

TTGAGTATCTTGATGCAGTAACTTATAGTTGTGATCCTGCACCTGGACCAGATCCATTTTCACTTATTGG

AGAGAGCACGATTTATTGTGGTGACAATTCAGTGTGGAGTCGTGCTGCTCCAGAGTGTAAAGTGGTCAAA

TGTCGATTTCCAGTAGTCGAAAATGGAAAACAGATATCAGGATTTGGAAAAAAATTTTACTACAAAGCAA

CAGTTATGTTTGAATGCGATAAGGGTTTTTACCTCGATGGCAGCGACACAATTGTCTGTGACAGTAACAG

TACTTGGGATCCCCCAGTTCCAAAGTGTCTTAAAGGATATCCTAAACCTGAGGAAGGAATACTTGACAGT

TTGGATGTTTGGGTCATTGCTGTGATTGTTATTGCCATAGTTGTTGGAGTTGCAGTAATTTGTGTTGTCC

CGTACAGATATCTTCAAAGGAGGAAGAAGAAAGGGAAAGCAGATGGTGGAGCTGAATATGCCACTTACCA

GACTAAATCAACCACTCCAGCAGAGCAGAGAGGCTGAATAGATTCCACAACCTGGTTTGCCAGTTCATCT

TTTGACTCTATTAAAATCTTCAATAGTTGTTATTCTGTAGTTTCACTCTCATGAGTGCAACTGTGGCTTA

GCTAATATTGCAATGTGGCTTGAATGTAGGTAGCATCCTTTGATGCTTCTTTGAAACTTGTATGAATTTG

GGTATGAACAGATTGCCTGCTTTCCCTTAAATAACACTTAGATTTATTGGACCAGTCAGCACAGCATGCC

TGGTTGTATTAAAGCAGGGATATGCTGTATTTTATAAAATTGGCAAAATTAGAGAAATATAGTTCACAAT

GAAATTATATTTTCTTTGTAAAGAAAGTGGCTTGAAATCTTTTTTGTTCAAAGATTAATGCCAACTCTTA

AGATTATTCTTTCACCAACTATAGAATGTATTTTATATATCGTTCATTGTAAAAAGCCCTTAAAAATATG

TGTATACTACTTTGGCTCTTGTGCATAAAAACAAGAACACTGAAAATTGGGAATATGCACAAACTTGGCT

TCTTTAACCAAGAATATTATTGGAAAATTCTCTAAAAGTTAATAGGGTAAATTCTCTATTTTTTGTAATG

TGTTCGGTGATTTCAGAAAGCTAGAAAGTGTATGTGTGGCATTTGTTTTCACTTTTTAAAACATCCCTAA

CTGATCGAATATATCAGTAATTTCAGAATCAGATGCATCCTTTCATAAGAAGTGAGAGGACTCTGACAGC

CATAACAGGAGTGCCACTTCATGGTGCGAAGTGAACACTGTAGTCTTGTTGTTTTCCCAAAGAGAACTCC

GTATGTTCTCTTAGGTTGAGTAACCCACTCTGAATTCTGGTTACATGTGTTTTTCTCTCCCTCCTTAAAT

AAAGAGAGGGGTTAAACATGCCCTCTAAAAGTAGGTGGTTTTGAAGAGAATAAATTCATCAGATAACCTC

AAGTCACATGAGAATCTTAGTCCATTTACATTGCCTTGGCTAGTAAAAGCCATCTATGTATATGTCTTAC

CTCATCTCCTAAAAGGCAGAGTACAAAGTAAGCCATGTATCTCAGGAAGGTAACTTCATTTTGTCTATTT

GCTGTTGATTGTACCAAGGGATGGAAGAAGTAAATATAGCTCAGGTAGCACTTTATACTCAGGCAGATCT

CAGCCCTCTACTGAGTCCCTTAGCCAAGCAGTTTCTTTCAAAGAAGCCAGCAGGCGAAAAGCAGGGACTG

CCACTGCATTTCATATCACACTGTTAAAAGTTGTGTTTTGAAATTTTATGTTTAGTTGCACAAATTGGGC

CAAAGAAACATTGCCTTGAGGAAGATATGATTGGAAAATCAAGAGTGTAGAAGAATAAATACTGTTTTAC

TGTCCAAAGACATGTTTATAGTGCTCTGTAAATGTTCCTTTCCTTTGTAGTCTCTGGCAAGATGCTTTAG

GAAGATAAAAGTTTGAGGAGAACAAACAGGAATTCTGAATTAAGCACAGAGTTGAAGTTTATACCCGTTT

CACATGCTTTTCAAGAATGTCGCAATTACTAAGAAGCAGATAATGGTGTTTTTTAGAAACCTAATTGAAG

TATATTCAACCAAATACTTTAATGTATAAAATAAATATTATACAATATACTTGTATAGCAGTTTCTGCTT

CACATTTGATTTTTTCAAATTTAATATTTATATTAGAGATCTATATATGTATAAATATGTATTTTGTCAA

ATTTGTTACTTAAATATATAGAGACCAGTTTTCTCTGGAAGTTTGTTTAAATGACAGAAGCGTATATGAA

TTCAAGAAAATTTAAGCTGCAAAAATGTATTTGCTATAAAATGAGAAGTCTCACTGATAGAGGTTCTTTA

TTGCTCATTTTTTAAAAAATGGACTCTTGAAATCTGTTAAAATAAAATTGTACATTTGGA

>NM_078467.3 Homo sapiens cyclin dependent kinase inhibitor 1A (CDKN1A), transcript variant 2, mRNA

ATGTTGAGCTCTGGCATAGAAGAGGCTGGTGGCTATTTTGTCCTTGGGCTGCCTGTTTTCAGCTGCTGCA

ACCACAGGGATTTCTTCTGTTCAGGCGCCATGTCAGAACCGGCTGGGGATGTCCGTCAGAACCCATGCGG

CAGCAAGGCCTGCCGCCGCCTCTTCGGCCCAGTGGACAGCGAGCAGCTGAGCCGCGACTGTGATGCGCTA

ATGGCGGGCTGCATCCAGGAGGCCCGTGAGCGATGGAACTTCGACTTTGTCACCGAGACACCACTGGAGG

GTGACTTCGCCTGGGAGCGTGTGCGGGGCCTTGGCCTGCCCAAGCTCTACCTTCCCACGGGGCCCCGGCG

AGGCCGGGATGAGTTGGGAGGAGGCAGGCGGCCTGGCACCTCACCTGCTCTGCTGCAGGGGACAGCAGAG

GAAGACCATGTGGACCTGTCACTGTCTTGTACCCTTGTGCCTCGCTCAGGGGAGCAGGCTGAAGGGTCCC

CAGGTGGACCTGGAGACTCTCAGGGTCGAAAACGGCGGCAGACCAGCATGACAGATTTCTACCACTCCAA

ACGCCGGCTGATCTTCTCCAAGAGGAAGCCCTAATCCGCCCACAGGAAGCCTGCAGTCCTGGAAGCGCGA

GGGCCTCAAAGGCCCGCTCTACATCTTCTGCCTTAGTCTCAGTTTGTGTGTCTTAATTATTATTTGTGTT

TTAATTTAAACACCTCCTCATGTACATACCCTGGCCGCCCCCTGCCCCCCAGCCTCTGGCATTAGAATTA

TTTAAACAAAAACTAGGCGGTTGAATGAGAGGTTCCTAAGAGTGCTGGGCATTTTTATTTTATGAAATAC

TATTTAAAGCCTCCTCATCCCGTGTTCTCCTTTTCCTCTCTCCCGGAGGTTGGGTGGGCCGGCTTCATGC

CAGCTACTTCCTCCTCCCCACTTGTCCGCTGGGTGGTACCCTCTGGAGGGGTGTGGCTCCTTCCCATCGC

TGTCACAGGCGGTTATGAAATTCACCCCCTTTCCTGGACACTCAGACCTGAATTCTTTTTCATTTGAGAA

GTAAACAGATGGCACTTTGAAGGGGCCTCACCGAGTGGGGGCATCATCAAAAACTTTGGAGTCCCCTCAC

CTCCTCTAAGGTTGGGCAGGGTGACCCTGAAGTGAGCACAGCCTAGGGCTGAGCTGGGGACCTGGTACCC

TCCTGGCTCTTGATACCCCCCTCTGTCTTGTGAAGGCAGGGGGAAGGTGGGGTCCTGGAGCAGACCACCC

CGCCTGCCCTCATGGCCCCTCTGACCTGCACTGGGGAGCCCGTCTCAGTGTTGAGCCTTTTCCCTCTTTG

GCTCCCCTGTACCTTTTGAGGAGCCCCAGCTACCCTTTTTCTCCAGCTGGGCTCTGCAATTCCCCTCTGC

TGCTGTCCCTCCCCCTTGTCCTTTCCCTTCAGTACCCTCTCAGCTCCAGGTGGCTCTGAGGTGCCTGTCC

CACCCCCACCCCCAGCTCAATGGACTGGAAGGGGAAGGGACACACAAGAAGAAGGGCACCCTAGTTCTAC

CTCAGGCAGCTCAAGCAGCGACCGCCCCCTCCTCTAGCTGTGGGGGTGAGGGTCCCATGTGGTGGCACAG

GCCCCCTTGAGTGGGGTTATCTCTGTGTTAGGGGTATATGATGGGGGAGTAGATCTTTCTAGGAGGGAGA

CACTGGCCCCTCAAATCGTCCAGCGACCTTCCTCATCCACCCCATCCCTCCCCAGTTCATTGCACTTTGA

TTAGCAGCGGAACAAGGAGTCAGACATTTTAAGATGGTGGCAGTAGAGGCTATGGACAGGGCATGCCACG

TGGGCTCATATGGGGCTGGGAGTAGTTGTCTTTCCTGGCACTAACGTTGAGCCCCTGGAGGCACTGAAGT

GCTTAGTGTACTTGGAGTATTGGGGTCTGACCCCAAACACCTTCCAGCTCCTGTAACATACTGGCCTGGA

CTGTTTTCTCTCGGCTCCCCATGTGTCCTGGTTCCCGTTTCTCCACCTAGACTGTAAACCTCTCGAGGGC

AGGGACCACACCCTGTACTGTTCTGTGTCTTTCACAGCTCCTCCCACAATGCTGAATATACAGCAGGTGC

TCAATAAATGATTCTTAGTGACTTTA

>NM_004064.5 Homo sapiens cyclin dependent kinase inhibitor 1B (CDKN1B), mRNA

CTTCTTCGTCAGCCTCCCTTCCACCGCCATATTGGGCCACTAAAAAAAGGGGGCTCGTCTTTTCGGGGTG

TTTTTCTCCCCCTCCCCTGTCCCCGCTTGCTCACGGCTCTGCGACTCCGACGCCGGCAAGGTTTGGAGAG

CGGCTGGGTTCGCGGGACCCGCGGGCTTGCACCCGCCCAGACTCGGACGGGCTTTGCCACCCTCTCCGCT

TGCCTGGTCCCCTCTCCTCTCCGCCCTCCCGCTCGCCAGTCCATTTGATCAGCGGAGACTCGGCGGCCGG

GCCGGGGCTTCCCCGCAGCCCCTGCGCGCTCCTAGAGCTCGGGCCGTGGCTCGTCGGGGTCTGTGTCTTT

TGGCTCCGAGGGCAGTCGCTGGGCTTCCGAGAGGGGTTCGGGCTGCGTAGGGGCGCTTTGTTTTGTTCGG

TTTTGTTTTTTTGAGAGTGCGAGAGAGGCGGTCGTGCAGACCCGGGAGAAAGATGTCAAACGTGCGAGTG

TCTAACGGGAGCCCTAGCCTGGAGCGGATGGACGCCAGGCAGGCGGAGCACCCCAAGCCCTCGGCCTGCA

GGAACCTCTTCGGCCCGGTGGACCACGAAGAGTTAACCCGGGACTTGGAGAAGCACTGCAGAGACATGGA

AGAGGCGAGCCAGCGCAAGTGGAATTTCGATTTTCAGAATCACAAACCCCTAGAGGGCAAGTACGAGTGG

CAAGAGGTGGAGAAGGGCAGCTTGCCCGAGTTCTACTACAGACCCCCGCGGCCCCCCAAAGGTGCCTGCA

AGGTGCCGGCGCAGGAGAGCCAGGATGTCAGCGGGAGCCGCCCGGCGGCGCCTTTAATTGGGGCTCCGGC

TAACTCTGAGGACACGCATTTGGTGGACCCAAAGACTGATCCGTCGGACAGCCAGACGGGGTTAGCGGAG

CAATGCGCAGGAATAAGGAAGCGACCTGCAACCGACGATTCTTCTACTCAAAACAAAAGAGCCAACAGAA

CAGAAGAAAATGTTTCAGACGGTTCCCCAAATGCCGGTTCTGTGGAGCAGACGCCCAAGAAGCCTGGCCT

CAGAAGACGTCAAACGTAAACAGCTCGAATTAAGAATATGTTTCCTTGTTTATCAGATACATCACTGCTT

GATGAAGCAAGGAAGATATACATGAAAATTTTAAAAATACATATCGCTGACTTCATGGAATGGACATCCT

GTATAAGCACTGAAAAACAACAACACAATAACACTAAAATTTTAGGCACTCTTAAATGATCTGCCTCTAA

AAGCGTTGGATGTAGCATTATGCAATTAGGTTTTTCCTTATTTGCTTCATTGTACTACCTGTGTATATAG

TTTTTACCTTTTATGTAGCACATAAACTTTGGGGAAGGGAGGGCAGGGTGGGGCTGAGGAACTGACGTGG

AGCGGGGTATGAAGAGCTTGCTTTGATTTACAGCAAGTAGATAAATATTTGACTTGCATGAAGAGAAGCA

ATTTTGGGGAAGGGTTTGAATTGTTTTCTTTAAAGATGTAATGTCCCTTTCAGAGACAGCTGATACTTCA

TTTAAAAAAATCACAAAAATTTGAACACTGGCTAAAGATAATTGCTATTTATTTTTACAAGAAGTTTATT

CTCATTTGGGAGATCTGGTGATCTCCCAAGCTATCTAAAGTTTGTTAGATAGCTGCATGTGGCTTTTTTA

AAAAAGCAACAGAAACCTATCCTCACTGCCCTCCCCAGTCTCTCTTAAAGTTGGAATTTACCAGTTAATT

ACTCAGCAGAATGGTGATCACTCCAGGTAGTTTGGGGCAAAAATCCGAGGTGCTTGGGAGTTTTGAATGT

TAAGAATTGACCATCTGCTTTTATTAAATTTGTTGACAAAATTTTCTCATTTTCTTTTCACTTCGGGCTG

TGTAAACACAGTCAAAATAATTCTAAATCCCTCGATATTTTTAAAGATCTGTAAGTAACTTCACATTAAA

AAATGAAATATTTTTTAATTTAAAGCTTACTCTGTCCATTTATCCACAGGAAAGTGTTATTTTTCAAGGA

AGGTTCATGTAGAGAAAAGCACACTTGTAGGATAAGTGAAATGGATACTACATCTTTAAACAGTATTTCA

TTGCCTGTGTATGGAAAAACCATTTGAAGTGTACCTGTGTACATAACTCTGTAAAAACACTGAAAAATTA

TACTAACTTATTTATGTTAAAAGATTTTTTTTAATCTAGACAATATACAAGCCAAAGTGGCATGTTTTGT

GCATTTGTAAATGCTGTGTTGGGTAGAATAGGTTTTCCCCTCTTTTGTTAAATAATATGGCTATGCTTAA

AAGGTTGCATACTGAGCCAAGTATAATTTTTTGTAATGTGTGAAAAAGATGCCAATTATTGTTACACATT

AAGTAATCAATAAAGAAAACTTCCATAGCTA

>NM_000077.5 Homo sapiens cyclin dependent kinase inhibitor 2A (CDKN2A), transcript variant 1, mRNA

GAGAGCAGGCAGCGGGCGGCGGGGAGCAGCATGGAGCCGGCGGCGGGGAGCAGCATGGAGCCTTCGGCTG

ACTGGCTGGCCACGGCCGCGGCCCGGGGTCGGGTAGAGGAGGTGCGGGCGCTGCTGGAGGCGGGGGCGCT

GCCCAACGCACCGAATAGTTACGGTCGGAGGCCGATCCAGGTCATGATGATGGGCAGCGCCCGAGTGGCG

GAGCTGCTGCTGCTCCACGGCGCGGAGCCCAACTGCGCCGACCCCGCCACTCTCACCCGACCCGTGCACG

ACGCTGCCCGGGAGGGCTTCCTGGACACGCTGGTGGTGCTGCACCGGGCCGGGGCGCGGCTGGACGTGCG

CGATGCCTGGGGCCGTCTGCCCGTGGACCTGGCTGAGGAGCTGGGCCATCGCGATGTCGCACGGTACCTG

CGCGCGGCTGCGGGGGGCACCAGAGGCAGTAACCATGCCCGCATAGATGCCGCGGAAGGTCCCTCAGACA

TCCCCGATTGAAAGAACCAGAGAGGCTCTGAGAAACCTCGGGAAACTTAGATCATCAGTCACCGAAGGTC

CTACAGGGCCACAACTGCCCCCGCCACAACCCACCCCGCTTTCGTAGTTTTCATTTAGAAAATAGAGCTT

TTAAAAATGTCCTGCCTTTTAACGTAGATATATGCCTTCCCCCACTACCGTAAATGTCCATTTATATCAT

TTTTTATATATTCTTATAAAAATGTAAAAAAGAAAAACACCGCTTCTGCCTTTTCACTGTGTTGGAGTTT

TCTGGAGTGAGCACTCACGCCCTAAGCGCACATTCATGTGGGCATTTCTTGCGAGCCTCGCAGCCTCCGG

AAGCTGTCGACTTCATGACAAGCATTTTGTGAACTAGGGAAGCTCAGGGGGGTTACTGGCTTCTCTTGAG

TCACACTGCTAGCAAATGGCAGAACCAAAGCTCAAATAAAAATAAAATAATTTTCATTCATTCACTCA

>NM_003879.7 Homo sapiens CASP8 and FADD like apoptosis regulator (CFLAR), transcript variant 1, mRNA

ACACAAGCCATAGCAGGAAACAGCGAGCTTGCAGCCTCACCGACGAGTCTCAACTAAAAGGGACTCCCGG

AGCTAGGGGTGGGGACTCGGCCTCACACAGTGAGTGCCGGCTATTGGACTTTTGTCCAGTGACAGCTGAG

ACAACAAGGACCACGGGAGGAGGTGTAGGAGAGAAGCGCCGCGAACAGCGATCGCCCAGCACCAAGTCCG

CTTCCAGGCTTTCGGTTTCTTTGCCTCCATCTTGGGTGCGCCTTCCCGGCGTCTAGGGGAGCGAAGGCTG

AGGTGGCAGCGGCAGGAGAGTCCGGCCGCGACAGGACGAACTCCCCCACTGGAAAGGATTCTGAAAGAAA

TGAAGTCAGCCCTCAGAAATGAAGTTGACTGCCTGCTGGCTTTCTGTTGACTGGCCCGGAGCTGTACTGC

AAGACCCTTGTGAGCTTCCCTAGTCTAAGAGTAGGATGTCTGCTGAAGTCATCCATCAGGTTGAAGAAGC

ACTTGATACAGATGAGAAGGAGATGCTGCTCTTTTTGTGCCGGGATGTTGCTATAGATGTGGTTCCACCT

AATGTCAGGGACCTTCTGGATATTTTACGGGAAAGAGGTAAGCTGTCTGTCGGGGACTTGGCTGAACTGC

TCTACAGAGTGAGGCGATTTGACCTGCTCAAACGTATCTTGAAGATGGACAGAAAAGCTGTGGAGACCCA

CCTGCTCAGGAACCCTCACCTTGTTTCGGACTATAGAGTGCTGATGGCAGAGATTGGTGAGGATTTGGAT

AAATCTGATGTGTCCTCATTAATTTTCCTCATGAAGGATTACATGGGCCGAGGCAAGATAAGCAAGGAGA

AGAGTTTCTTGGACCTTGTGGTTGAGTTGGAGAAACTAAATCTGGTTGCCCCAGATCAACTGGATTTATT

AGAAAAATGCCTAAAGAACATCCACAGAATAGACCTGAAGACAAAAATCCAGAAGTACAAGCAGTCTGTT

CAAGGAGCAGGGACAAGTTACAGGAATGTTCTCCAAGCAGCAATCCAAAAGAGTCTCAAGGATCCTTCAA

ATAACTTCAGGCTCCATAATGGGAGAAGTAAAGAACAAAGACTTAAGGAACAGCTTGGCGCTCAACAAGA

ACCAGTGAAGAAATCCATTCAGGAATCAGAAGCTTTTTTGCCTCAGAGCATACCTGAAGAGAGATACAAG

ATGAAGAGCAAGCCCCTAGGAATCTGCCTGATAATCGATTGCATTGGCAATGAGACAGAGCTTCTTCGAG

ACACCTTCACTTCCCTGGGCTATGAAGTCCAGAAATTCTTGCATCTCAGTATGCATGGTATATCCCAGAT

TCTTGGCCAATTTGCCTGTATGCCCGAGCACCGAGACTACGACAGCTTTGTGTGTGTCCTGGTGAGCCGA

GGAGGCTCCCAGAGTGTGTATGGTGTGGATCAGACTCACTCAGGGCTCCCCCTGCATCACATCAGGAGGA

TGTTCATGGGAGATTCATGCCCTTATCTAGCAGGGAAGCCAAAGATGTTTTTTATTCAGAACTATGTGGT

GTCAGAGGGCCAGCTGGAGGACAGCAGCCTCTTGGAGGTGGATGGGCCAGCGATGAAGAATGTGGAATTC

AAGGCTCAGAAGCGAGGGCTGTGCACAGTTCACCGAGAAGCTGACTTCTTCTGGAGCCTGTGTACTGCGG

ACATGTCCCTGCTGGAGCAGTCTCACAGCTCACCATCCCTGTACCTGCAGTGCCTCTCCCAGAAACTGAG

ACAAGAAAGAAAACGCCCACTCCTGGATCTTCACATTGAACTCAATGGCTACATGTATGATTGGAACAGC

AGAGTTTCTGCCAAGGAGAAATATTATGTCTGGCTGCAGCACACTCTGAGAAAGAAACTTATCCTCTCCT

ACACATAAGAAACCAAAAGGCTGGGCGTAGTGGCTCACACCTGTAATCCCAGCACTTTGGGAGGCCAAGG

AGGGCAGATCACTTCAGGTCAGGAGTTCGAGACCAGCCTGGCCAACATGGTAAACGCTGTCCCTAGTAAA

AATACAAAAATTAGCTGGGTGTGGGTGTGGGTACCTGTATTCCCAGTTACTTGGGAGGCTGAGGTGGGAG

GATCTTTTGAACCCAGGAGTTCAGGGTCATAGCATGCTGTGATTGTGCCTACGAATAGCCACTGCATACC

AACCTGGGCAATATAGCAAGATCCCATCTCTTTAAAAAAAAAAAAAAAAGGACAGGAACTATCTTACTCAA

TGTATTAGTCATGTTTCTCTAGAGGGACAGAACTAATAGGATACATGTATATAAAAAGGGGAGTTTATTA

AGGAGTATTGACTCACATGATCACAGGGTTAGGTCCCACAATAGGTCATCTGCAAGCAAGGAAGCCAATT

CAAGTCCCAAAGCTGAAGAACTTGGAGTCCAATGTTTGAGGGCAGGAAGCATTCAGCATGAGAGAAAGAT

GGAGGCCAGAAGACTACACCAGTCTAGTCTTTCCATGTTTTGCCTGCTTTTATTCTGGCAGTGCTGGCAG

CTGATTAGATGGTGCCCACCCAGATTGAGGATGGTCTGCCTTTCCCAGTCCACTGACTCAAATGTTAAAT

CTCCTTTGGCAGCACCCTCACAGATGTACCCGGGAACACTTTGCATCCTTCTATTCAATCAAGTTGATAC

TCAGTATTAACCATCACAGTCCATTTGGGCAACTATACCAAATTACCATAGACCAGGTGACTTAAACAGC

AGTTATTTCTCACAGTTCCGGAGGCTGGGAAATCCAACATCTAAGTGGTAGCATATCTGGTGTCTGGTAA

GGCATGCTTCCAGATCTTACCAGATGTCAGTCTTTTGATGTTCTCACATGGCAGAAAAAGAGGATGCAAA

CTCTCAAGTATATCTTTAAGGGCACAAATTCCATTCATGAGGGCTCTACCCTCATCACCTAATTACCTCC

CAAAGGCCCCACCTTCTGATACTGTCACTTTGGGGATACTGTCTCCCCTTTGAATTCTGGGGGGAATACA

AACATTCAGTTTGTAACAATAGCCTTATGATTTAGAGGTTACTTGTTCATTCACCTAGACCTCAAATTGC

ATTTTACAGCTAGTCAAGTATATCTTTCTCTGATTTGATAGTGTGACCTAAAAGGGGACCATTGTTTGAA

ATATCATTAGAGTTGCTTATTATTATTATTATTATTATTATTATTATTATTATTATTATTATTGAGACAG

AGTTTCATTCTGCTGCCCAGGCTGGAGTGCAGTGGCATCATCTTGGCTCATTGCAACCTCTGCCTTCTGG

GTTCAAGCGATTCTCCTGCCTCAGCCTCCCGAGTAGCTGGGATTACAGGCTCCTGCCACCACACCCGGCT

AATTTTTGTATTTTTAGTGGAGACAGGGTTTCCACCATGTTGGCCAGCGTGGTCTTGAACTCCTGACCTC

AGGTGATTCACCAGCCTCGGCCTCCCAAAGTGCTGGGATTACAGGTGTGAGCCACTGCACCTGGCCTATT

ATTATTTTTAAATTTTTTTTTTTTTAATTGATCATTCTTGGGTGTTTCTCACAGAGGGTGATTTGGCAGGG

TCACAGGACAATAGTGGAGGGAAGGTCAGCAGATAAACAAGTGAACAAAGGTCTCTGGTTTTCCTAGGCA

GAGGACCCTGCGGCCTTCCGCAGTGTTTGTGTCCCTGGGTACTTGAGATTAGGGAGTGGTGATGACTCTT

AAGGAGCATGCTGCCTTCAAGCATCTGTTTAACAAAGCACATCTTGCACTGCCCTTAATCCATTTAACCC

TGAGTGGACACAGCACATGTTTCAGAGAGCACAGGGTTGGGGGTAAGGTCATAGATCAACAGCATCCTAA

GGCAGAAGAATTTTTCTTAGTACAGAACAAAATGAAGTCTCCCATGTCTACTTCTTTCTACACAGACACA

GCAACAATCTGATTTCTCTATCTTTTCCCCACCTTTCCCCCTTTTCTATTCCACAAAACCGCCATCGTCA

TCATGGCCTGTTCTCAATGAGCTGTTGGGTACACCTCCCAGACGGGGTGGCGGCTGGGCAGAGGGGCTCC

TCACTTCCCAGATGGGGCGGCCAGGCGGACGCGCCCCCCACCTCCCTCCCGGACGGGATAGCTGGCCGGG

CGGGGGCTGACCCCCCACCTCCCTCCCCGACGGGGCGGCTGGCCGGGCGGGGGCTGACCCCCACGCCTCC

CTCCCGGACGGGGCGGCTGCCAGGCGGAGGGGCTCCTCACTTCTCAGACGGGGTGGCTGCTGGGCGGAGA

CGCTCCTCACTTCCCAGACAGGGTGGCTGTCGGGCGGAGGGGCTCCTCACTTCTCAGACGGGGCAGCTGC

GGGCGGAGGGGCTCCTCACTTCTCAGACGGGGTGGCCGGGCAGAGAAGCTCCTCACATCCCAGACGGGGG

GGCGGGGCAGAGGCGCTCCCCACATCTCAGACGATGGGCGGCCGGGCAGAGACGCTCCTCACTTCATCCC

AGACGGGGTGGCGGCCGGGCAGAAGCTGTAATCTCGGCACCCTGGGGGGCCAAGGCAGGCGGCTGGGAGG

CGGAGGCCGTAGCCAGCTGAGATCACACCACTGCACTCCAGCCTGGGCAACATTGAGCACTGAGTGGACG

AGACTCTGCCCGCAATCCCGGCACCTCGGGAGGCCGAGGCTGGCAGATCACTCGCAGTCAGGAGCTGGAG

ACCAGCCCGGCCAACACAGTGAAACCCTGTCTCCACCAAAAAAATACGAAAACCAGTCAGGCGTGGCGGC

GCCCGCAATGGCAGGCACGCGGCAGGCCGAGGCGGGAGAATCAGGCAGGGAGGCTGCAGTGAGCCGAGAT

GGCAGCAGTACAGTCCAGCTTCGGCTCGGCATCAGAGGGAGACCGTGGGGAGAGGGAGAAGAGAGGGAGG

GGGAGAGGGCTATTTTTAAAATTTTTTAAAATTGCTGAACAGGGGTACCTCTGGGCAGTGTGTCAGAATA

CCACTTTTTAAATATTTTATGATTTATTTATTTTTCTATTTCTTGAGGTTTTAACTGATGTGTATCTGTA

TGTCTATTTGTGTATATTTTGTCATGATCATGTAACAGAGTCTGAAAAGTGTCGAAGAGACAGTTTTCAG

GAACAACAAGCAATTATTCCTACTTTCCAAGTTATTTTGATGCCATGGTGGCTCATACCTATAATCTGAG

TACTTTGGGAGGCTGAGGTGGACTGATCACTTGAGCCCAGGAGTTTGAGACCAGCCTGGGCAACATAGCA

AGACTCCATCTCTACAAAAAAAGACAAAATTTAGCTGAGCGTGGTGGCGTGTTCCTGTAGTCCCAGCTAC

TTGGGAGGCTGAAGTGAGTGGATCCCCTGAGCCCAGAGAGGTCAAGGTTGTGATGAGCTGTGATCACACC

ACTGCACTTCAGCATGGGAGACAGAGTGAGACCCTGTTTCAGAAAAAATAAATAAATAAAACCACCAGCA

CCACAAACAACAACAAAAAGTTATTTTGTACTTGTTTTGAGCACAGGACTCCTGAGGGTATCTTTGCATT

TAATATTACATAGGGGTGCCAGTGGGAAGTAATGTGTATGCTTGGCCTCATGAGCTAAAACCCTGTGTTA

ATTATGACAGAAGGAAAGTGTGTGAGAGAGATCTTAACTACCTAGCAGCTCTAGCTGCCATCTTGAACCA

TGAAGATACGGGCCACACGTAGGGGTAGCTGGGTAGTGAGCAGCAAGAAGCCTTGTTGGATGAGGGCACG

AAGGAGCAGAATCACTGGAATCACTGTGTCAGCCCTAATTACCTACCTCTGGACTTTTATGTGAGGGGAA

AAAAAATTGACAGTTTATATTTATCTCAACCTAGTTAACCCAAGTGATGCATTGTTATGAGATTAAAATG

TTTGGAGGCCGGGTGCGGTGGCTCACGCCTATAATCCCAGCCCTTTGGGAGGCCAAGGCGGGCGGATCAC

GAGGTCAGGAGATCAAGACCATCCTGGCTAACATGTAAAACCCCGTCTCTACTAAAAATACAAAAAATTA

GCCAGGCGTTGTGGCGGTCGCCTGTAGTCCCTGCTATTTGGGAGGCCGAGGCAAGAGAACGGCATGAACC

TGGGAGGTGGAGCTTGCAGCGAGCTGAGATCTTGCCACTGCACTCCAGCCTGGGCGACAGTGCGAGACTC

TGTCTCAAAAATAAATAAATAAATAAATAATAAATAAAATGTTTGGAATGTTGGCTTCATCCCTGGGATG

CAAGGCTGGTTCAACATACGCAAATCAAGAAACATAATTCATCACATAAACAGAACTAAAGACAAAAACC

ACATGATTATCTCAATAGATACAGAAAAGGCCTTCAATAAAATTCAACGTTGCTTCATGTTAAAAACTCT

CAATAAACTAGGTATTGATGGAAAATATCTCAAAATAATAACCATTTATGACAAACCCACAGCCATTATC

ATACTGAATGGGCAAAAGCTGGAAGCATTCCCCTTGAAAACTGGCACAAGACAGGGATGCCGTCTCACCA

CTCCTATTTAACATAGTATTGGAAGTTCTGGCCAAGAAAATCAGGCAAGAGAAACAAATAAGGGGTATTC

AAATAGGAAAAGAGGAAGTAAAACTGTGTTTGCAGATGACATGATACTATATCTAGAAAACCCCATTATC

TCCACCCAAAAGTTCCTTAAGCTGATAAGCAACTTCAGCAAAGTCTCAGGATACAAAATCAATGTGCAGA

AATCACAAGCATTCTATACACCAACAATACACAAGCAGAGAGCCAAATCATGAATGAACTCCCATTCACA

GTTGCTAGAAAGAGAATAAAATACCTAGGAATACAGCTAATAAGATGTGAAGGATCTCTTCAAGGAGAAC

TACAAACCACTGCTCAAGGAAATAAGAGAGGACACAAATGAAAAAACATTCCATTCTCGTGGATAGGAAG

AATCAATATCATGAAAATGGCCATACTACCCAAAGTAATTTATAGGTTCATTGCTATTCCCATTAAACTA

CTATTGACATTCTTCACAGAATTAGAAAAAAACTACTTTAAAATTCAAATGGAACCAAAAAAGAGCCCGT

ATAACCAAGACAACAATAAGCAAAAAGAACAAAGCTGGAAGCATCACACTACCCAACTTCAAAGTATACT

GCAAGGCTACAGTAGCCAAAATGGCATGGTACTGGTACAAAAACAGACACATAGACCAATGGAACAGAAT

AGAGACCAGAGAAAGAAGACCACACATCTACAGCCATCTGATCATCGACAAACCTGACAAAAACAAGCAA

TGGGGAAAAGATTCCCTATTTAATAAATGGTGCTGGGAAAACTGGCTAGCCATATGCAGAAAATTGAAAC

TGACCCCTTCCTTACACCTTATACAAAAATTAACTCAAGATTAAAGACTTAATGTAAAACCTAAAACTAT

AAAAACCCTAGAAGAAAATCTATTTAATACCATTCAAGACATAGGCACAAGCAAAGGTTTCATGACAAAA

ACATCAAAAGCAATTGCAACAAAAGCAAAAATTACAAATGGGATCTAATTAAACTAAAGAGCTCCTGCAC

AGCAAAAGAAACTATCATTAGAGTGAACAGGCAACCTACAGAATGGGAGAACATTTTTGCAATCTATCCA

TCTGACAAAGGTCTAATATCCAGAACCTACAAGGAACTTAAAACAAATTTACAAGGAAAAAAACAACCCC

ATCAAAAAGTGGACAAAGGACATGAACAGACACTTCTCAAAAGAAGACATTTATGTGGCCAACAAACATA

TAAAAAAAAGCTCAACCTTACTGATCATTAGAGAAATGCAAAGGAGAACCACAATGAGATACCATCTCAT

GCCGGTCAGAATGGTGATTATTAAAAAGTCAAAAAACAACAGATGCTGGCGAGGCTGTGGAGAAGTAGGA

ACACTTTTACATTGTTGGTGGGAATGTAAATTAGTTCAACCGTTGTGGAAGTGTGTGTGGCTATTCCTCA

AAGATCTAGAACTAGAAATACTATTTGTCCCAGCAATCCCATTACTGGGTATATACCCAAAGGAATATAA

ACCATTTTATTATAAAGATACATGCACATTTTTGTTCATTGCAGCACTCTTCACAATAGCAAAGACACAA

TAGCAAATGCCCATCAAAGATAGACTGGATAAAGAAAATGTGGTACATATACACCATGGAATACTGTGCA

GTGCAGCCATTACAGCTTTTGGTGATACAGTGAATCAGATTTTTCATTAATTCTTTTAATTGGTTATTAC

TGAACGTGAAAAAGTAATGTTTGTATTGAAATCTTGAGTCTGGCCATGTTTCTATTTTAAATTCATAAAG

AATTCTAACAAGAGGAATTCCAAGAATGTCATAAATGGATGTTTCTCCATGGATGAAGGAACTGTTTTAT

TCACTTGCTGATAATTCAGCCTAATCCAGTTTGACATCATATAGATAAGTAGTTGAATTATGGATTTAAA

ATACATATCATTTTCTAACTCCAAAGGTAATACTTATTTAAATGGTTTTGAAAATATAGAAAGGCACAAT

TTCTTTTTAAATCTGTTATTCTCCACCACCACTCAATCTGTCTATCATCTATCTCTCCATTCATTCTTCC

ATTTGTTTATATCTGTTAATCTTTGTATGTGTTCATGTATAGCTTTTACATGATTGGAATCATAATGCAT

ATTCCATTTTGAAGTCTGCTTTTTTTTACACAAAAATATGTTGTGAATATTTTCCTATATTATGAAATAT

CATTAGCTGAGCTTTTAGAATTGACTGCATGTTTTGGTACCATTTAGATATAGTTTAAGATACTTAGAAG

TTATGTGGCTTTGCCACTATGGATGAATCTTATTTACTCAATATTAATTACTTACAAATAACCTCACCTA

AACACTACTCAGCCATAAAAAGGAATGAATTAATGACATTCACAGCAACCTGGAGACTATTACTCTAAAG

GAAGTAACTGAGGAATGGAAAACCAAACATTGTATGTTCTCACTCATAAGTGGGAGATAAGCTATGAGGA

TGCAAAGGCATAAGAAGGATACAATGGACTTTGGGGACTTAGGGGAAAGGGTGGGAGGGGGGTGAAGGAT

AAAAGAATACAAATTGGGTTCAGTGTATACTGCTCAGGTGATGGGTGCACCAGAATCTCACAAGTAACCA

CTTAATTACTTACGCATGTAACCAGATACCACCTGTTCCCCAAACACCTATGGAAATAATTTTGTTTTTT

TTTTTAAAAAAGGAATGAGATCATGTCCTTTGCAGGGACATGGATGAAGCTGGAAGCCATTATCCTCAGC

AAACTAACAGAGGAGCAGGAAACCAAACACCACATGTTCTCACTTGTAAGCGGAAGCTGAACAATGAGAA

CACACGGACACAGGGATGAGATCAACACACACTGGGGCCTGATGCAGGGGCCGTAGCGGGGAGAGCATCA

GGATAACTAGCTAATGCATGTGGGGCTTAATACCTAGGTGATAGGTTGATAGGTGCAGCAAACCACCATG

GGACACGTTTACCTATGTAACAAACCCGCACATCCTGCACTTGTATCCAGAACTTAAAATATTTTAAAAA

TCTTTAGAGAATACAAAAAAAAAAAAAAAAGATTCTTCAATGCATACACAATAAAATTGCAGTTCAGTCAA

ACATTGGAAGTCTTTCTCTGACTGTCTAGTTGGTATCTTCATTTTCAGCTTCTTCAAGATCCCACTCCAA

ACACTGTTAGCTCAGCCAAATTGAACAGCTCATATCTCCTACCTCTGGATCTTTGGTTCTGGTGATTGTA

TATTTCTGGACCATCTGGAACCCCAGCATATCACCCTACCCCACATCTCCACATCCCCAAAATATAACCA

TACTTCAAGGGCAGTTCAAATACCATCTCCTTCTATCCTCCATGAAGTCAGTTATCTCTTCCATTGGAAT

TATCGCCCCCTCTCCTGAACAGTACTATTTCGTGTGAATCTCCTCCAAGCCTTCTTTTCATTTTATATCT

CATGCTGTAATTCTTGGAAAGTATGCTGTAGCTCAAGTGCAGAATTCTCATCAGTTTTATCTTTATATCT

CTCCTAAACACTTTACCTGATGAAGAGCCTGGCATACACATAAATATATATTGAATGAATCAGTGATGGA

TTGAAAAGAGAAATGATGGATCTCCTAAATTTTAACTTTTATAAAATATTTTGATACATTCATGACCTTA

CTTTAGCAAGCAATGAACGTGATGTAAACTATTGTTGATATAGTTTTTATATTGGAAGTGTAAGTAGTTT

GTGGCATGGGATTGTGACATATCCTAGGTTTCCTCATCTTCTTTTTATTGAAATGTAATTCACAAGCCAT

AAAATTTGCCCCTTTAAAGTAAATGATGCAGTGGATTTTAGTATATTTACAGAGTTGTGCAATCATCACC

ACTATCTAATTCCAGAACATTTCCATCTACCTAGAAACTCCATACCAGTGAGCTGCCACTCTAATCCTCC

TCTTCCCCCAGCCTCTAGAAACAATAATCCATTTTCTGTCTCTATGATTTGCCTGTTCTAGATATTTTAT

AAAAATAAACATGTGGCCTTTCGTGTCTGACTTCCTTCACTTAGCATGTTTCAAGGTTCATCTATGCTAT

AGAGTGTACCAGTGCTTCATTACATTTTATGGCTGAATAATACCCTATTGTGTGGATATACCGCATTTAG

TTTATTCATCAGTTGATCGAAATTTAGACTGTTTCCACTTTTTTGCTATTATGGATGATGCTGCTATGAA

TATTCGTATATGAATTTTTATATGGATATATGTTTTCAGTTCTTCTGGGCATATACCTTAGAAGTGAGAG

TGCTCATCATATAGTAACTCTATGTTTAGCCTTTTGAGGAACTGCCAAACTGCTTTTCAAAGTAGTAGCA

TCATTTTATATTCCCACCAGCAGTGTATGAGGTTTTTTTTATTTTTTTGTTTTTGAGACGGAGTCTCACT

CTGTCGCCCAGGGTGGAGTGCAGTGGCGTTATCTTGATTCACTGTAACCTCTGCCTTCTGGGTTCAAGCC

ATTCTCCTGCCTCAGCCTCCCTAGTAGTTGGGAGTAGCTGGGTTTACAGGAGCTCGCCACCATGCCCAAC

TAATTTTTGTATTGTTTTTTGAGACGGAGTCTCGCTCTGTGGCCCAGGCTGGAGTGCAGTGGTGCAATCT

CAGCTCACTGCAAGCTCCGCCTCCTGGGTTCATGCCATTCTCCTGCCTCAGCCTCCCAAGTATCTGGGAC

TACAGCCGCCCTCCACCACGCCCGGCTAATTTTTTGTATTTTTAGTAGAGACAGGGTTTCACCGTGTTAG

CCAGGATGGTCTCTATCTCCTGACCTCGTGATCCGCCTGCCTTGGCCTCCCAAAGTGCTGGGATTACAGG

CGTGAGCCACCGCGCCCAGCCAATTTTTGTATTTTTAGTAAAGACAGGGTTTCACCATGTTGGCCAGGCT

GGTCTCGAACTCCTGGCCTCAGGTGATCTGACTGCCTTGGCTTCCCAAAGTGCTGGCATTACAGACATTA

GCTACTGTGCCTGGCCTCAAGTCCTTTCTCATTTTAAAACTGCGTTATTTTATTTTATTTTTCTGAGATG

GAATCTCACTCTGTCGCCCAGGTTGGAGTGCAGTGGTGCAATCTCGGCTCACTGCAACCTCCACCTCCTG

AGTTCAAGCGACTCTCCTGCCTCGGCCTCCCTAGTAACTGGGATTACAGGCACCCACCATCACGCCTGGC

TAATTTTTGTATTTTTAGTAGAGACAGGGTTTCGCCATGTTGGCCAGGCTGGTCTTGAACTCCTGAACTC

AGGTGATCTGCCTGCCTCGGCCTCCCAAAATGCTAGGATTACAGGCGTGAGCCACCACACCCGGTGGGTT

GTCTTTTTATTGTTGAGTTGTAAGCATTCTTTTTAATATTCTGGATACTAGTTTCTTATCAGGTACAATC

TTTGTGAATATATTCTCCCATTCCATGGATTGTCTTCTCACTTTCTGGATACTATACAGTACAAGTTTTA

AATTTTGATGAAGTTCACTTGATTTCTGTTTTTTATGTTATTGCTTATGCTTTTGGTGTCATACCTAAAA

AACCATTGTCTAAATCAATTGCCTATGAGGTGTTACCCCTATGTTTTCTTCTAAAGTTTTATGATTTTAG

CTCTTTGACCCATTTTGAGTTATTTTTGTGTATGCAGTGCGAGATAAGCATCCAGCCTCATTCTTTTGCA

CATAAATGTTCACTTGTCTTAGCATTACTTATTGAAAATACTATTTTTTCCCTATAGAATTATTTTATAC

TTATCACAAATCAACTAACTATAAGGGTTTATTTCTGGACTCAATTCTGTTGCTCTATCTTATGGTAGTA

CCACACTGTCTTGATTATTGTAACTTCGTAGTAAGTTTTGGAATCCAGAAATGTAGTCCTCTAACTTTGT

TGTTGTTGTTGTTCTTCTTTTTTCTTTTGTAGAGATGAGGCCTTGCTATGTTGCCCAGGCTGGTCTCAAA

CTCCTGGCCTCAAACAATCCTCCCACCTCCACCTCCCAAAGTGCTGAGGGGTGAGCCACTGCAACTGGCC

TGTTCTTTTTCAAAATTGTTAGGGCTGTATTGGGTTTACCTTTCCCTAAATTTTCATATAACTGTTAGGA

TTCACTTGCAAATTTATGTAAAAAGGGCCTCTGGAATTTTGATAGTGATTACACTGAATGAGAGGAGTTT

GGCAGGACTGGTTTCCCAAGACCACAAGACCCTGTAAGACCCTGCTGATAAGACAGGATGTGGTAGAAAG

GCCAACCCAAACCAGCCAAAACCATGATGGTGACAGAAGAGACCTCTGGTTACCTTTGCTGCCCATTCTA

TGGTAATTATAATACATTAGCACGCTAAAAGAAACTTCTACAGCACCATGATAGTTTACAACTGCCATAG

CAACTTTCAGAAGTTACCCTGTATGGTCTAAAAGGTCCAGACCTCCCCACTTCTTTCCTGGAAAACTCAT

GAATAAGCCACCTCTTGTTTAGCGTATAGTCAAGAAGCAACCATAAATATAGCCAGTCAGCAGCCCATCA

GGGCTACTCTTCCTATGGGGTAGCCATTCTTTTATTCCTTTACTTTCTTAATGAACTTGCTTTCACTTTA

TTCTGTCAGCATGCTCTTGAATTCTTTCCTGAACCCACTTGACCTCTTGGGCTGAGCCTTAACCTTGGGG

TACTTTTGCCTATAACAAACATACAAAGATAAGTTTGGCTGGGCGCAGTGGCTCATGCCTGTAATCCCAG

GACTTTGGGAGGCTGAGGATGGTGGATCGCTTGAGCCAAGGAATTTGAGGCCACCTTGGGCAACACGGCA

AAACCCTGTCTCTACTAAAAATACAAAAATTAGCTGGGTATGGTAGCATGCGCCTGTAGTACCAGCTACT

CGGAAGGCTGAGGTGGGAGGATCACTTGAGCCCGGGAGGTGGAGGTTTCAGTGAGCTGAGATTGTGCCAC

TGTACTCCAGCCTGGGGAACAGAGTGAGACCCTGTCTCAAAATAAAAATAAATTTGAGGTGTATTGCCAT

CTTAACGGAAGTTGTTACTGGTGGACGGTATCCAAGTTACTGGTGGTAAATCTGCAGCAACCTCAATTCT

TTCCTCCTCAGAAGAAAGAATTTGACTGAGGGGCATAAGGCAGAAGGAGGGACCGAGGCAAGTTTCAGAG

CAACAGTGAAAGTTTATTACGCTTTAGGCTGGGCACAGTGGCTCATGCCTGTAATCCCAGCATTTTGGGA

GGCTGAGGCAGGTGGATTACTTGAGGTCAGTAGTTCGAGACCAGCCTGGCCATCATGATGATGAAACCTC

GTCTCTACTAAAAATAGAAAAATCAGCTGGGCATGGTGGTCCATGCCTCTAATCCCAGCTACTCGGGAGG

CTGAGGCAGGCGAATCAGTTGAACCTGGGAGGCGGAGGTTGCAGTGAGCCAAGAAAGTTTATTAAGCTTT

AGAACAGTAAGGAAAGGAAAGAAAAGAAGGAAAGTATAACTTGGAAGAGGGCCAAGCAGGTGACCTGAGA

AACCAGGTGCAGGGCTTGCCCTCTTGACTTGGGGTTTTATATGCTGGCATACTTCCGGGATCTTGTGTTA

CTTTTCCCCACTCCTGAGATCTTTTTGAGAAGCTGATGATCAGTCTCAGGTGTTTTCTATTGCGGGGGGG

GGGGGCGGGCGGGGGAGCTGCCTGTCCCTGGCACCACCTGTGACCAATTATTACTTTACAGAAACATTTA

ACAACCGCCTGACCATCACCTGATGGTTGCCCAACACTCTTGGTGTGTGTCGGGGGAGCCCTCTCCTGCC

TTGCTCAGACCTGAATGTACCCATTGTAACAAACCCCTTCTTTGTATACTTTGATATGGTGACATTTCTT

GTCACTTGCCTCATTGTGAATTTTTATAGATGTGGCTAAGTCATATGCTTCCTTCTGAAAAATTATGTCA

AATACTAGCAGTTTCTCACTGTTTTTTGCCCTAGAAACAACAAACTAACCTTGAAGTGAAAGGCCTTTCT

GCGTTTATCTTACCTTTTTTGAGCAGCATTAAAACTGTTTTACTCAGACCAA

>NM_014043.4 Homo sapiens charged multivesicular body protein 2B (CHMP2B), transcript variant 1, mRNA

GTTAGTTCCCGGTCACCTGAGCTCCGGGTGACGCGGCTGCGGTAGCTGCGGATACAAGCCTTCCGCGGGT

CCTGCCTGGCGACCCCGACCTCCTCCTGCTGTCTCTCCGCTCCGCCACCCCGAACCCGCCAAGGTCCTGT

CCTTTTCCTCCTGTCCTTTGCCAGCGTTGGGCCGGACCGGGCCGAGCCGGGCCGCCCGGGCGCAGTCTTT

AACCATGGCGTCCCTCTTCAAGAAGAAAACCGTGGATGATGTAATAAAGGAACAGAATCGAGAGTTACGA

GGTACACAGAGGGCTATAATCAGAGATCGAGCAGCTTTAGAGAAACAAGAAAAACAGCTGGAATTAGAAA

TTAAGAAAATGGCCAAGATTGGTAATAAGGAAGCTTGCAAAGTTTTAGCCAAACAACTTGTGCATCTACG

GAAACAGAAGACGAGAACTTTTGCTGTAAGTTCAAAAGTTACTTCTATGTCTACACAAACAAAAGTGATG

AATTCCCAAATGAAGATGGCTGGAGCAATGTCTACTACAGCAAAAACAATGCAGGCAGTTAACAAGAAGA

TGGATCCACAAAAGACATTACAAACAATGCAGAATTTCCAGAAGGAAAACATGAAAATGGAAATGACTGA

AGAAATGATCAATGATACACTTGATGACATCTTTGACGGTTCTGATGACGAAGAAGAAAGCCAGGATATT

GTGAATCAAGTTCTTGATGAAATTGGAATTGAAATTTCTGGAAAGATGGCCAAAGCTCCATCAGCTGCTC

GAAGCTTACCATCTGCCTCTACTTCAAAGGCTACAATCTCAGATGAAGAGATTGAACGGCAACTCAAGGC

TTTAGGAGTAGATTAGTCAAAAGAAGTCATACTATTTTGCTTACTTATAATTATGTAGTATAAACCAAGC

ACAGTGCAGATTTCTTTTACAAAACACATGTATTTTGCAAAAAAAAAAAAAAATGAAGACCATGAGTGAAC

AGTTGTTTCCTAACCCATGGCTATTTAGAATCTTTTGCCAAAGAATGACAATGATGCAAAAATGGGAACA

GTTTGGATTTTAATTAGAACTGTTTAGGAGTGATGATGTGTAAAAAGTTGACTTCTCTTTTGCATGGCAC

AGAGAAATTATATTCCTTACTTCATGTCAGTTTATGTTCTAAATCTTTTTCACTGAATATAAAAATCTTG

TTAAATGCCATTAGGCACCAACTTAAAGAGGGTTGTAAAAATATTAAAAGTATATCGTTAATTCTGTATC

TGTTGCTTGTCTTTTGTAAGTGATTATGTGTTATGACCATAGGTGGTTACAGCTGCCAAATTATTTTTAA

ATGGTCAAAAAGAAGAGTGCTATTTAAACATCTGTCTTAAACAAAAACTGTCATAACTTTTCTTTTTTCT

TTTTCCATTAGGAGAACATTCTAGTTGGTAAATTTCAAAATGTGCTTGACACCTGCCTTAAATAGCACAG

ACCTATTGTGCACATCTTTAAATTATTTCAGCTGGCAGAAAAGAATTACATTTAAAACTGAAATCAAGGC

CTCAATACAAAGATTATCCTGGCTCTTTTCTATCTCTGTGGGCCTAATTGAAATATGTACTCTTATTTTA

GACACGCCTCTGTTAAAACAGACCAGGTTTTCCTGGTCTCAGACCTATGATGACTTGTCCCTTTGATGTC

ACTACTGTGAATTGAATATAATTAGTAAAAATAGACGATGAATAAATAACACTTTATAGTAAGAAAACAA

TATATTTTGGCCATCTAAAAATGAGAATTATAATTATATGAATTATAATTTAAACTGTTTAATTTTGTTT

AATGTGTATATTGAATCTTCCAAATTGAAGCCATTATTCTCAATTAAGTACTACAACTATGACAATGCTT

GACCTACATTTCTAAAATAAAAATTCACATTTTTTGATAAATAAACTACAGTTTTACCAGAAATTACTAT

CTAAATGTGTATTAGCAGTATTTTTTAAGGTGAAATTGCCTTGGTATCTAATGAATGTGTAGACAGGGAG

ATAAAATGAAGGATTGCCAGACTAGTTAGAATAGAATTTAGGATTAGGTTAGTTTTGAAAAATGATGTTG

TAATATATGGGTTCTAACACATCCTACCATAAAAACTGGAGGAGATATGTGTAACCTGGTTAATTTGGGA

TGGTGGACATTTTGGGCTAATACTGACAAAATACATCTTAGGACTAGTATACATGTGACACGGATTGCTA

GGAGGAATGAAAAACTAAACTGTATAGTTTATATTCCGTAAACCATTTTATAATGTTCAAAGATTAGGTT

TTGTTATTGATAGTATTAAATACACAGTTTCTCTTAACAGTGATGGGTGAAAACATTTTACCGGATTATG

GAATGTTTACCAGAACATGTTTTGATTCTTGAATGTACATAATAATGCCATCTAACTTATTTACGTTCTT

GTTTACATGTGGGAGCTTTTGTTTTCAAAAATTATTTTGTTAAAAAATCTCAATAAAGATTTATTATTGT

TGTTCTTTTCTTACCTTTTTTGCTCTTTTTGGTTCCTGCTAAAATTAAAAATTTTATGCATATTTGGTAA

>NM_176812.5 Homo sapiens charged multivesicular body protein 4B (CHMP4B), mRNA

GGAGAGGCCTGCGGCGGCAGGGAGCGGCGGGACTGGGAGCGGGCGCCGGAGCCGACCCGAGCCGAGCCGA
GCCGAGCCGAGCCGGAGCGGGCGGCGAAGGCCGGCGCGGCGAGCAGCAACCATGTCGGTGTTCGGGAAGC
TGTTCGGGGCTGGAGGGGGTAAGGCCGGCAAGGGCGGCCCGACCCCCCAGGAGGCCATCCAGCGGCTGCG
GGACACGGAAGAGATGTTAAGCAAGAAACAGGAGTTCCTGGAGAAGAAAATCGAGCAGGAGCTGACGGCC
GCCAAGAAGCACGGCACCAAAAACAAGCGCGCGGCCCTCCAGGCACTGAAGCGTAAGAAGAGGTATGAGA
AGCAGCTGGCGCAGATCGACGGCACATTATCAACCATCGAGTTCCAGCGGGAGGCCCTGGAGAATGCCAA
CACCAACACCGAGGTGCTCAAGAACATGGGCTATGCCGCCAAGGCCATGAAGGCGGCCCATGACAACATG
GACATCGATAAAGTTGATGAGTTAATGCAGGACATTGCTGACCAGCAAGAACTTGCAGAGGAGATTTCAA
CAGCAATTTCGAAACCTGTAGGGTTTGGAGAAGAGTTTGACGAGGATGAGCTCATGGCGGAATTAGAAGA
ACTAGAACAGGAGGAACTAGACAAGAATTTGCTGGAAATCAGTGGACCCGAAACAGTCCCTCTACCAAAT
GTTCCCTCTATAGCCCTACCATCAAAACCCGCCAAGAAGAAAGAAGAGGAGGACGACGACATGAAGGAAT
TGGAGAACTGGGCTGGATCCATGTAATGGGGTCCAGCGCTGGCTGGGCCCAGACAGACTGTGGTGGCCTG
CGCAGCGAGCAGGCGTGTGCGTGTGTGGGGCAGGCAGGATGTGGTGCAGGCAGGTTCCATCGCTTTCGAC
TCTCACTCCAAAGCAGTAGGGCCGCGTTGCTGCTCACTCTCTGCATAGCATGGTCTGCACCTGGGAGATG
GGCGGGGGGAGGGGGGCGGGCGGGGTGGGAAGTGCCTGCTGTTTATAATGTTGAATTTCTGTAAAATAAA
CTGTATTTGCAAATCCAACATTGAGCTTCTGGACTACGCTGACTCCACTGCTGAATCCTCAATGGAAAGG
GTCGACTGGTTGCAGTTGAAATGACCTGAAATGTAGCCTCTGTCCTTGTAAGTCAGTTGACTTGCCGCAC
ATCTCTTTGTGTACTTGTACGGTACTGGCAGAAAAGTCATTTTTCAAAAGCCATAGGCTTTTCCTTGCCC
TTAGCTGTAATAATGCATCTGATTTTGATTTCCTCCAGAGCTGTGTTTCTGTCCATCACCTGTGTATTGG
CCCTGTGTTTACCACTCTGGCCCACTCCTCACCCCCTTGCTCCCCTGGTCTTCTGGAGTTTGTGACATTG
ATTTGAAATGGATGGTGTTCTCTTGAGAGCAAGTGAGATTGTTAGAATTAAGTTCCAACTATACAGTTTT
CTAACATAGCTATAAGGTCCTTGTTGCTGTTTGTGATAACTGATAGATAACTCATTGGAAACGTGCATAC
ATTTATATTCAGATGAAATTATGGTTTGCACTGTCTATTAAATATCTCGATTAATTTTCATA

>NM_000086.2 Homo sapiens CLN3 lysosomal/endosomal transmembrane protein, battenin (CLN3), transcript variant 2, mRNA

GTGCTGTCACGTGATCCGACAAACGGCCTCTGCATAGTGCAGAACATTCTGCTGCTCTTAAAGGTACAGG

CCTCAGGGTCCCTGCTGTAGACGGGGCGGGGGAGAGTACGATGGGTGGGGCGTGGTGGGTCGTAGGGCGC

TCGAGATGGAGCCCCCAGCTTCCTTGATGGATCGCGGGGCGCGAGTGCCCTAGACAAGCCGGAGCTGGGA

CCGGCAATCGGGCGTTGATCCTTGTCACCTGTCGCAGACCCTCATCCCTCCCGTGGGAGCCCCCTTTGGA

CACTCTATGACCCTGGACCCTCGGGGGACCTGAACTTGATGCGATGGGAGGCTGTGCAGGCTCGCGGCGG

CGCTTTTCGGATTCCGAGGGGGAGGAGACCGTCCCGGAGCCCCGGCTCCCTCTGTTGGACCATCAGGGCG

CGCATTGGAAGAACGCGGTGGGCTTCTGGCTGCTGGGCCTTTGCAACAACTTCTCTTATGTGGTGATGCT

GAGTGCCGCCCACGACATCCTTAGCCACAAGAGGACATCGGGAAACCAGAGCCATGTGGACCCAGGCCCA

ACGCCGATCCCCCACAACAGCTCATCACGATTTGACTGCAACTCTGTCTCTACGGCTGCTGTGCTCCTGG

CGGACATCCTCCCCACACTCGTCATCAAATTGTTGGCTCCTCTTGGCCTTCACCTGCTGCCCTACAGCCC

CCGGGTTCTCGTCAGTGGGATTTGTGCTGCTGGAAGCTTCGTCCTGGTTGCCTTTTCTCATTCTGTGGGG

ACCAGCCTGTGTGGTGTGGTCTTCGCTAGCATCTCATCAGGCCTTGGGGAGGTCACCTTCCTCTCCCTCA

CTGCCTTCTACCCCAGGGCCGTGATCTCCTGGTGGTCCTCAGGGACTGGGGGAGCTGGGCTGCTGGGGGC

CCTGTCCTACCTGGGCCTCACCCAGGCCGGCCTCTCCCCTCAGCAGACCCTGCTGTCCATGCTGGGTATC

CCTGCCCTGCTGCTGGCCAGCTATTTCTTGTTGCTCACATCTCCTGAGGCCCAGGACCCTGGAGGGGAAG

AAGAAGCAGAGAGCGCAGCCCGGCAGCCCCTCATAAGAACCGAGGCCCCGGAGTCGAAGCCAGGCTCCAG

CTCCAGCCTCTCCCTTCGGGAAAGGTGGACAGTGTTCAAGGGTCTGCTGTGGTACATTGTTCCCTTGGTC

GTAGTTTACTTTGCCGAGTATTTCATTAACCAGGGACTTTTTGAACTCCTCTTTTTCTGGAACACTTCCC

TGAGTCACGCTCAGCAATACCGCTGGTACCAGATGCTGTACCAGGCTGGCGTCTTTGCCTCCCGCTCTTC

TCTCCGCTGCTGTCGCATCCGTTTCACCTGGGCCCTGGCCCTGCTGCAGTGCCTCAACCTGGTGTTCCTG

CTGGCAGACGTGTGGTTCGGCTTTCTGCCAAGCATCTACCTCGTCTTCCTGATCATTCTGTATGAGGGGC

TCCTGGGAGGCGCAGCCTACGTGAACACCTTCCACAACATCGCCCTGGAGACCAGTGATGAGCACCGGGA

GTTTGCAATGGCGGCCACCTGCATCTCTGACACACTGGGGATCTCCCTGTCGGGGCTCCTGGCTTTGCCT

CTGCATGACTTCCTCTGCCAGCTCTCCTGATACTCGGGATCCTCAGGACGCAGGTCACATTCACCTGTGG

GCAGAGGGACAGGTCAGACACCCAGGCCCACCCCAGAGACCCTCCATGAACTGTGCTCCCAGCCTTCCCG

GCAGGTCTGGGAGTAGGGAAGGGCTGAAGCCTTGTTTCCCTTGCAGGGGGGCCAGCCATTGTCTCCCACT

TGGGGAGTTTCTTCCTGGCATCATGCCTTCTGAATAAATGCCGATTTTATCCATGGAAA

>NM_147780.4 Homo sapiens cathepsin B (CTSB), transcript variant 2, mRNA

GGCTGCAGGGCTCCGGCAACCGCTCCGGCAACGCCAACCGCTCCGCTGCGCGCAGGCTGGGCTGCAGGCT

CTCGGCTGCAGCGCTGGGCTGGTGTGCAGTGGTGCGACCACGGCTCACGGCAGCCTCAGCCACCCAGATG

TAAGCGATCTGGTTCCCACCTCAGCCTCCCGAGTAGTGTCTTCAGGCCTATGGAGAGCAGCTTGCGTGGG

CTGGGCCTGCAGTACCTGGTTTGCATAGATGATTGGCAGGTGGATCTAGGATCCGGCTTCCAACATGTGG

CAGCTCTGGGCCTCCCTCTGCTGCCTGCTGGTGTTGGCCAATGCCCGGAGCAGGCCCTCTTTCCATCCCC

TGTCGGATGAGCTGGTCAACTATGTCAACAAACGGAATACCACGTGGCAGGCCGGGCACAACTTCTACAA

CGTGGACATGAGCTACTTGAAGAGGCTATGTGGTACCTTCCTGGGTGGGCCCAAGCCACCCCAGAGAGTT

ATGTTTACCGAGGACCTGAAGCTGCCTGCAAGCTTCGATGCACGGGAACAATGGCCACAGTGTCCCACCA

TCAAAGAGATCAGAGACCAGGGCTCCTGTGGCTCCTGCTGGGCCTTCGGGGCTGTGGAAGCCATCTCTGA

CCGGATCTGCATCCACACCAATGCGCACGTCAGCGTGGAGGTGTCGGCGGAGGACCTGCTCACATGCTGT

GGCAGCATGTGTGGGGACGGCTGTAATGGTGGCTATCCTGCTGAAGCTTGGAACTTCTGGACAAGAAAAG

GCCTGGTTTCTGGTGGCCTCTATGAATCCCATGTAGGGTGCAGACCGTACTCCATCCCTCCCTGTGAGCA

CCACGTCAACGGCTCCCGGCCCCCATGCACGGGGGAGGGAGATACCCCCAAGTGTAGCAAGATCTGTGAG

CCTGGCTACAGCCCGACCTACAAACAGGACAAGCACTACGGATACAATTCCTACAGCGTCTCCAATAGCG

AGAAGGACATCATGGCCGAGATCTACAAAAACGGCCCCGTGGAGGGAGCTTTCTCTGTGTATTCGGACTT

CCTGCTCTACAAGTCAGGAGTGTACCAACACGTCACCGGAGAGATGATGGGTGGCCATGCCATCCGCATC

CTGGGCTGGGGAGTGGAGAATGGCACACCCTACTGGCTGGTTGCCAACTCCTGGAACACTGACTGGGGTG

ACAATGGCTTCTTTAAAATACTCAGAGGACAGGATCACTGTGGAATCGAATCAGAAGTGGTGGCTGGAAT

TCCACGCACCGATCAGTACTGGGAAAAGATCTAATCTGCCGTGGGCCTGTCGTGCCAGTCCTGGGGGCGA

GATCGGGGTAGAAATGCATTTTATTCTTTAAGTTCACGTAAGATACAAGTTTCAGACAGGGTCTGAAGGA

CTGGATTGGCCAAACATCAGACCTGTCTTCCAAGGAGACCAAGTCCTGGCTACATCCCAGCCTGTGGTTA

CAGTGCAGACAGGCCATGTGAGCCACCGCTGCCAGCACAGAGCGTCCTTCCCCCTGTAGACTAGTGCCGT

AGGGAGTACCTGCTGCCCCAGCTGACTGTGGCCCCCTCCGTGATCCATCCATCTCCAGGGAGCAAGACAG

AGACGCAGGAATGGAAAGCGGAGTTCCTAACAGGATGAAAGTTCCCCCATCAGTTCCCCCAGTACCTCCA

AGCAAGTAGCTTTCCACATTTGTCACAGAAATCAGAGGAGAGACGGTGTTGGGAGCCCTTTGGAGAACGC

CAGTCTCCCAGGCCCCCTGCATCTATCGAGTTTGCAATGTCACAACCTCTCTGATCTTGTGCTCAGCATG

ATTCTTTAATAGAAGTTTTATTTTTTCGTGCACTCTGCTAATCATGTGGGTGAGCCAGTGGAACAGCGGG

AGACCTGTGCTAGTTTTACAGATTGCCTCCTTATGACGCGGCTCAAAAGGAAACCAAGTGGTCAGGAGTT

GTTTCTGACCCACTGATCTCTACTACCACAAGGAAAATAGTTTAGGAGAAACCAGCTTTTACTGTTTTTG

AAAAATTACAGCTTCACCCTGTCAAGTTAACAAGGAATGCCTGTGCCAATAAAAGTTTTCTCCAACTTGA

AGTCTACTCTGATGGGATCTCAGATCCTTTGTCACTGCCTATAGACTTGTAGCTGCTGTCTCTCTTTGTC

CCTGCAGAGAATCACGTCCTGGAACTGCATGTTCTTGCGACTCTTGGGACTTCATCTTAACTTCTCGCTG

CCCCAGCCATGTTTTCAACCATGGCATCCCTCCCCCAATTAGTTCCCTGTCATCCTCGTCAACCTTCTCT

GTAAGTGCCTGGTAAGCTTGCCCTTGCTTAAGAACTCAAAACATAGCTGTGCTCTATTTTTTTGTTGTTG

TTGTGACTGACAGAGTGAGATTCCGTCTCCCAGGCTGGAGTGCAGTGGCGCCTTCTCAGCTCACTGCAAC

CTGCAGCCTCCTAGATTCAAGCGATTCTCCTGCTTCAGCCTTCCGAGTAGCTGGGATGACAGGCACTCAC

CAATATGCCTGGGTAATTTTTGTATTTTTAAGTACATACAGGATTTCACCATGTTGGCCAGGCTAGTTTC

AAACTCCCGGCCTCAGGTGGTCTGCCTGCCTCAGCCTCCCAAAGTGTTGGGATTACAGGCGTGAGCCACT

GGGCCCTGCCTGTATTTTTTATCAGCCACAAATCCAGCAACAAGCTGAGGATTCAGCTCATAAAACAGGC

TTGGTGTCTTGGTGATCTCACATAACCAAGATGCTACCCCGTGGGGAACCACATCCCCCTGGATGCCCTC

CAGCCTTGGTTTGGGCTGGAGTCAGGGCCTGTATACAGTATTTTGAATTTGTATGCCACTGGTTTGCATT

GCTGGTCAGGAACTCTAGTGCTTTGCATAGCCCTGGTTTAGAAACATGTTATAGCAGTTCTTGGTATAGA

GCAAACTAGAAGAACCAGCAATCATTCCACTGTCCTGCCAAGGTACACCTCAGTACTCCCCTTCCCAACT

GAAGTGGTATGAGGCTAGCTCTTTCCAAAAGCATTCAAGTTTGGCTTCTGATGTGACTCAGAATTTAGGA

ACCAGATGCTAGATCAAATAAGCTCTGAAAATCTGAGGAACATTGTAGGAAAGGTTTGTTAAGCATCTCT

TAAGTGCCATGATGAGCATAACAGCCGGCCGTCGTGGCTCACGCCTGTAATCCCAGCACTTTGGGAGGCC

AAGGTGGGAGGATGACAAGGTCAGGAGTTCAAGACCAGCCTGGCCAACATGCTGAAACCTCACCTCTACT

AAAAATACAAAAATTAGCTGGGCATGGTGGCACATGCCTGTAATCCCAGCTACTTGGGAGGCTGAGGCAG

GAGAATCGCTTGAACCCGGGAGGCGGAGGTTGCAGTGAGCCAAGACAGTGCCAGTGCACTCCAGCCTCGG

TGACAGCGCAAGGCTCCGTCTCAATAATTAAAAAAAAAAAAAAAAAAAAAAAAGGCCGGGCGCAGTGGCTC

AAGCCTGTAATCCCAGCACTTTGGGAGGCTGAGGCGGGCAGATCACCTGAGGTCAGGAGTTTTGAGATCA

GCCTTGGCAACACGGTGAAACCCCATCTCTACTAAAAATACAAAATTAGCCAAGCATGCTGGCACATGCC

TGTAATCCCAGCTACTCGGGAGGCTGAGGTACGAGAATCGCTTGAACCTGGGAGGCAGAGGATGCAGTGA

GCCGAGATCACGCCATTGCACTCCAGCCTGGGGGACAAGAGTGAATCTGTGTCTCACCAAAAAAAAAAAG

AAAAAGAAAGATGCTTAACAAAGGTTACCATAAGCCACAAATTCATAACCACTTATCCTTCCAGTTTCAA

GTAGAATATATTCATAACCTCAATAAAGTTCTCCCTGCTCCCAAA

>NM_001909.5 Homo sapiens cathepsin D (CTSD), mRNA

AACTGCGGCGTCATCCCGGCTATAAGCGCACGGCCTCGGCGACCCTCTCCGACCCGGCCGCCGCCGCCAT

GCAGCCCTCCAGCCTTCTGCCGCTCGCCCTCTGCCTGCTGGCTGCACCCGCCTCCGCGCTCGTCAGGATC

CCGCTGCACAAGTTCACGTCCATCCGCCGGACCATGTCGGAGGTTGGGGGCTCTGTGGAGGACCTGATTG

CCAAAGGCCCCGTCTCAAAGTACTCCCAGGCGGTGCCAGCCGTGACCGAGGGGCCCATTCCCGAGGTGCT

CAAGAACTACATGGACGCCCAGTACTACGGGGAGATTGGCATCGGGACGCCCCCCCAGTGCTTCACAGTC

GTCTTCGACACGGGCTCCTCCAACCTGTGGGTCCCCTCCATCCACTGCAAACTGCTGGACATCGCTTGCT

GGATCCACCACAAGTACAACAGCGACAAGTCCAGCACCTACGTGAAGAATGGTACCTCGTTTGACATCCA

CTATGGCTCGGGCAGCCTCTCCGGGTACCTGAGCCAGGACACTGTGTCGGTGCCCTGCCAGTCAGCGTCG

TCAGCCTCTGCCCTGGGCGGTGTCAAAGTGGAGAGGCAGGTCTTTGGGGAGGCCACCAAGCAGCCAGGCA

TCACCTTCATCGCAGCCAAGTTCGATGGCATCCTGGGCATGGCCTACCCCCGCATCTCCGTCAACAACGT

GCTGCCCGTCTTCGACAACCTGATGCAGCAGAAGCTGGTGGACCAGAACATCTTCTCCTTCTACCTGAGC

AGGGACCCAGATGCGCAGCCTGGGGGTGAGCTGATGCTGGGTGGCACAGACTCCAAGTATTACAAGGGTT

CTCTGTCCTACCTGAATGTCACCCGCAAGGCCTACTGGCAGGTCCACCTGGACCAGGTGGAGGTGGCCAG

CGGGCTGACCCTGTGCAAGGAGGGCTGTGAGGCCATTGTGGACACAGGCACTTCCCTCATGGTGGGCCCG

GTGGATGAGGTGCGCGAGCTGCAGAAGGCCATCGGGGCCGTGCCGCTGATTCAGGGCGAGTACATGATCC

CCTGTGAGAAGGTGTCCACCCTGCCCGCGATCACACTGAAGCTGGGAGGCAAAGGCTACAAGCTGTCCCC

AGAGGACTACACGCTCAAGGTGTCGCAGGCCGGGAAGACCCTCTGCCTGAGCGGCTTCATGGGCATGGAC

ATCCCGCCACCCAGCGGGCCACTCTGGATCCTGGGCGACGTCTTCATCGGCCGCTACTACACTGTGTTTG

ACCGTGACAACAACAGGGTGGGCTTCGCCGAGGCTGCCCGCCTCTAGTTCCCAAGGCGTCCGCGCGCCAG

CACAGAAACAGAGGAGAGTCCCAGAGCAGGAGGCCCCTGGCCCAGCGGCCCCTCCCACACACACCCACAC

ACTCGCCCGCCCACTGTCCTGGGCGCCCTGGAAGCCGGCGGCCCAAGCCCGACTTGCTGTTTTGTTCTGT

GGTTTTCCCCTCCCTGGGTTCAGAAATGCTGCCTGCCTGTCTGTCTCTCCATCTGTTTGGTGGGGGTAGA

GCTGATCCAGAGCACAGATCTGTTTCGTGCATTGGAAGACCCCACCCAAGCTTGGCAGCCGAGCTCGTGT

ATCCTGGGGCTCCCTTCATCTCCAGGGAGTCCCCTCCCCGGCCCTACCAGCGCCCGCTGGGCTGAGCCCC

TACCCCACACCAGGCCGTCCTCCCGGGCCCTCCCTTGGAAACCTGCCCTGCCTGAGGGCCCCTCTGCCCA

GCTTGGGCCCAGCTGGGCTCTGCCACCCTACCTGTTCAGTGTCCCGGGCCCGTTGAGGATGAGGCCGCTA

GAGGCCTGAGGATGAGCTGGAAGGAGTGAGAGGGGACAAAACCCACCTTGTTGGAGCCTGCAGGGTGGTG

CTGGGACTGAGCCAGTCCCAGGGGCATGTATTGGCCTGGAGGTGGGGTTGGGATTGGGGGCTGGTGCCAG

CCTTCCTCTGCAGCTGACCTCTGTTGTCCTCCCCTTGGGCGGCTGAGAGCCCCAGCTGACATGGAAATAC

AGTTGTTGGCCTCCGGCCTCCCCTC

>NM_001912.5 Homo sapiens cathepsin L (CTSL), transcript variant 1, mRNA

AGAACCGCGACCTCCGCAACCTTGAGCGGCATCCGTGGAGTGCGCCTGCGCAGCTACGACCGCAGCAGGA

AAGCGCCGCCGGCCAGGCCCAGCTGTGGCCGGACAGGGACTGGAAGAGAGGACGCGGTCGAGTAGGTGTG

CACCAGCCCTGGCAACGAGAGCGTCTACCCCGAACTCTGCTGGCCTTGAGGTGGGGAAGCCGGGGAGGGC

AGTTGAGGACCCCGCGGAGGCGCGTGACTGGTTGAGCGGGCAGGCCAGCCTCCGAGCCGGGTGGACACAG

GTTTTAAAACATGAATCCTACACTCATCCTTGCTGCCTTTTGCCTGGGAATTGCCTCAGCTACTCTAACA

TTTGATCACAGTTTAGAGGCACAGTGGACCAAGTGGAAGGCGATGCACAACAGATTATACGGCATGAATG

AAGAAGGATGGAGGAGAGCAGTGTGGGAGAAGAACATGAAGATGATTGAACTGCACAATCAGGAATACAG

GGAAGGGAAACACAGCTTCACAATGGCCATGAACGCCTTTGGAGACATGACCAGTGAAGAATTCAGGCAG

GTGATGAATGGCTTTCAAAACCGTAAGCCCAGGAAGGGGAAAGTGTTCCAGGAACCTCTGTTTTATGAGG

CCCCCAGATCTGTGGATTGGAGAGAGAAAGGCTACGTGACTCCTGTGAAGAATCAGGGTCAGTGTGGTTC

TTGTTGGGCTTTTAGTGCTACTGGTGCTCTTGAAGGACAGATGTTCCGGAAAACTGGGAGGCTTATCTCA

CTGAGTGAGCAGAATCTGGTAGACTGCTCTGGGCCTCAAGGCAATGAAGGCTGCAATGGTGGCCTAATGG

ATTATGCTTTCCAGTATGTTCAGGATAATGGAGGCCTGGACTCTGAGGAATCCTATCCATATGAGGCAAC

AGAAGAATCCTGTAAGTACAATCCCAAGTATTCTGTTGCTAATGACACCGGCTTTGTGGACATCCCTAAG

CAGGAGAAGGCCCTGATGAAGGCAGTTGCAACTGTGGGGCCCATTTCTGTTGCTATTGATGCAGGTCATG

AGTCCTTCCTGTTCTATAAAGAAGGCATTTATTTTGAGCCAGACTGTAGCAGTGAAGACATGGATCATGG

TGTGCTGGTGGTTGGCTACGGATTTGAAAGCACAGAATCAGATAACAATAAATATTGGCTGGTGAAGAAC

AGCTGGGGTGAAGAATGGGGCATGGGTGGCTACGTAAAGATGGCCAAAGACCGGAGAAACCATTGTGGAA

TTGCCTCAGCAGCCAGCTACCCCACTGTGTGAGCTGGTGGACGGTGATGAGGAAGGACTTGACTGGGGAT

GGCGCATGCATGGGAGGAATTCATCTTCAGTCTACCAGCCCCCGCTGTGTCGGATACACACTCGAATCAT

TGAAGATCCGAGTGTGATTTGAATTCTGTGATATTTTCACACTGGTAAATGTTACCTCTATTTTAATTAC

TGCTATAAATAGGTTTATATTATTGATTCACTTACTGACTTTGCATTTTCGTTTTTAAAAGGATGTATAA

ATTTTTACCTGTTTAAATAAAATTTAATTTCAAATGTAGTGGTGGGGCTTCTTTCTATTTTTGATGCACT

GAATTTTTGTGTAATAAAGAACATAATTGGGCTCTAAGCCATAA

>NM_002996.6 Homo sapiens C-X3-C motif chemokine ligand 1 (CX3CL1), transcript variant 1, mRNA

AAGGGGACAGCACTGAGCTCTGCCGCCTGGCTCTAGCCGCCTGCCTGGCCCCCGCCGGGACTCTTGCCCA

CCCTCAGCCATGGCTCCGATATCTCTGTCGTGGCTGCTCCGCTTGGCCACCTTCTGCCATCTGACTGTCC

TGCTGGCTGGACAGCACCACGGTGTGACGAAATGCAACATCACGTGCAGCAAGATGACATCAAAGATACC

TGTAGCTTTGCTCATCCACTATCAACAGAACCAGGCATCATGCGGCAAACGCGCAATCATCTTGGAGACG

AGACAGCACAGGCTGTTCTGTGCCGACCCGAAGGAGCAATGGGTCAAGGACGCGATGCAGCATCTGGACC

GCCAGGCTGCTGCCCTAACTCGAAATGGCGGCACCTTCGAGAAGCAGATCGGCGAGGTGAAGCCCAGGAC

CACCCCTGCCGCCGGGGGAATGGACGAGTCTGTGGTCCTGGAGCCCGAAGCCACAGGCGAAAGCAGTAGC

CTGGAGCCGACTCCTTCTTCCCAGGAAGCACAGAGGGCCCTGGGGACCTCCCCAGAGCTGCCGACGGGCG

TGACTGGTTCCTCAGGGACCAGGCTCCCCCCGACGCCAAAGGCTCAGGATGGAGGGCCTGTGGGCACGGA

GCTTTTCCGAGTGCCTCCCGTCTCCACTGCCGCCACGTGGCAGAGTTCTGCTCCCCACCAACCTGGGCCC

AGCCTCTGGGCTGAGGCAAAGACCTCTGAGGCCCCGTCCACCCAGGACCCCTCCACCCAGGCCTCCACTG

CGTCCTCCCCAGCCCCAGAGGAGAATGCTCCGTCTGAAGGCCAGCGTGTGTGGGGTCAGGGACAGAGCCC

CAGGCCAGAGAACTCTCTGGAGCGGGAGGAGATGGGTCCCGTGCCAGCGCACACGGATGCCTTCCAGGAC

TGGGGGCCTGGCAGCATGGCCCACGTCTCTGTGGTCCCTGTCTCCTCAGAAGGGACCCCCAGCAGGGAGC

CAGTGGCTTCAGGCAGCTGGACCCCTAAGGCTGAGGAACCCATCCATGCCACCATGGACCCCCAGAGGCT

GGGCGTCCTTATCACTCCTGTCCCTGACGCCCAGGCTGCCACCCGGAGGCAGGCGGTGGGGCTGCTGGCC

TTCCTTGGCCTCCTCTTCTGCCTGGGGGTGGCCATGTTCACCTACCAGAGCCTCCAGGGCTGCCCTCGAA

AGATGGCAGGAGAGATGGCGGAGGGCCTTCGCTACATCCCCCGGAGCTGTGGTAGTAATTCATATGTCCT

GGTGCCCGTGTGAACTCCTCTGGCCTGTGTCTAGTTGTTTGATTCAGACAGCTGCCTGGGATCCCTCATC

CTCATACCCACCCCCACCCAAGGGCCTGGCCTGAGCTGGGATGATTGGAGGGGGGAGGTGGGATCCTCCA

GGTGCACAAGCTCCAAGCTCCCAGGCATTCCCCAGGAGGCCAGCCTTGACCATTCTCCACCTGCCAGGGA

CAGAGGGGGTGGCCTCCCAACTCACCCCAGCCCCAAAACTCTCCTCTGCTGCTGGCTGGTTAGAGGTTCC

CTTTGACGCCATCCCAGCCCCAATGAACAATTATTTATTAAATGCCCAGCCCCTTCTGACCCATGCTGCC

CTGTGAGTACTACAGTCCTCCCATCTCACACATGAGCATCAGGCCAGGCCCTCTGCCCACTCCCTGCAAC

CTGATTGTGTCTCTTGGTCCTGCTGCAGTTGCCAGTCACCCCGGCCACCTGCGGTGCTATCTCCCCCAGC

CCCATCCTCTGTACAGAGCCCACGCCCCCACTGGTGACATGTCTTTTCTTGCATGAGGCTAGTGTGGTGT

TTCCTGGCACTGCTTCCAGTGAGGCTCTGCCCTTGGTTAGGCATTGTGGGAAGGGGAGATAAGGGTATCT

GGTGACTTTCCTCTTTGGTCTACACTGTGCTGAGTCTGAAGGCTGGGTTCTGATCCTAGTTCCACCATCA

AGCCACCAACATACTCCCATCTGTGAAAGGAAAGAGGGAGGTAAGGAATACCTGTCCCCCTGACAACACT

CATTGACCTGAGGCCCTTCTCTCCAGCCCCTGGATGCAGCCTCACAGTCCTTACCAGCAGAGCACCTTAG

ACAGTCCCTGCCAATGGACTAACTTGTCTTTGGACCCTGAGGCCCAGAGGGCCTGCAAGGGAGTGAGTTG

ATAGCACAGACCCTGCCCTGTGGGCCCCCAAATGGAAATGGGCAGAGCAGAGACCATCCCTGAAGGCCCC

GCCCAGGCTTAGTCACTGAGACAGCCCGGGCTCTGCCTCCCATCACCCGCTAAGAGGGAGGGAGGGCTCC

AGACACATGTCCAAGAAGCCCAGGAAAGGCTCCAGGAGCAGCCACATTCCTGATGCTTCTTCAGAGACTC

CTGCAGGCAGCCAGGCCACAAGACCCTTGTGGTCCCACCCCACACACGCCAGATTCTTTCCTGAGGCTGG

GCTCCCTTCCCACCTCTCTCACTCCTTGAAAACACTGTTCTCTGCCCTCCAAGACCTTCTCCTTCACCTT

TGTCCCCACCGCAGACAGGACCAGGGATTTCCATGATGTTTTCCATGAGTCCCCTGTTTGTTTCTGAAAG

GGACGCTACCCGGGAAGGGGGCTGGGACATGGGAAAGGGGAAGTTGTAGGCATAAAGTCAGGGGTTCCCT

TTTTTGGCTGCTGAAGGCTCGAGCATGCCTGGATGGGGCTGCACCGGCTGGCCTGGCCCCTCAGGGTCCC

TGGTGGCAGCTCACCTCTCCCTTGGATTGTCCCCGACCCTTGCCGTCTACCTGAGGGGCCTCTTATGGGC

TGGGTTCTACCCAGGTGCTAGGAACACTCCTTCACAGATGGGTGCTTGGAGGAAGGAAACCCAGCTCTGG

TCCATAGAGAGCAAGACGCTGTGCTGCCCTGCCCACCTGGCCTCTGCACTCCCCTGCTGGGTGTGGCGCA

GCATATTCAGGAAGCTCAGGGCCTGGCTCAGGTGGGGTCACTCTGGCAGCTCAGAGAGGGTGGGAGTGGG

CCCAATGCACTTTGTTCTGGCTCTTCCAGGCTGGGAGAGCCTTCCAGGGGTGGGACACCCTGTGATGGGG

CCCTGCCTCCTTTGTGAGGAAGCCGCTGGGGCCAGTTGGTCCCCCTTCCATGGACTTTGTTAGTTTCTCC

AAGCAGGACATGGACAAGGATGATCTAGGAAGACTTTGGAAAGAGTAGGAAGACTTTGGAAAGACTTTTC

CAACCCTCATCACCAACGTCTGTGCCATTTTGTATTTTACTAATAAAATTTAAAAGTCTTGTGAA

>NM_001008540.2 Homo sapiens C-X-C motif chemokine receptor 4 (CXCR4), transcript variant 1, mRNA

CTTCCCTCTAGTGGGCGGGGCAGAGGAGTTAGCCAAGATGTGACTTTGAAACCCTCAGCGTCTCAGTGCC

CTTTTGTTCTAAACAAAGAATTTTGTAATTGGTTCTACCAAAGAAGGATATAATGAAGTCACTATGGGAA

AAGATGGGGAGGAGAGTTGTAGGATTCTACATTAATTCTCTTGTGCCCTTAGCCCACTACTTCAGAATTT

CCTGAAGAAAGCAAGCCTGAATTGGTTTTTTAAATTGCTTTAAAAATTTTTTTTAACTGGGTTAATGCTT

GCTGAATTGGAAGTGAATGTCCATTCCTTTGCCTCTTTTGCAGATATACACTTCAGATAACTACACCGAG

GAAATGGGCTCAGGGGACTATGACTCCATGAAGGAACCCTGTTTCCGTGAAGAAAATGCTAATTTCAATA

AAATCTTCCTGCCCACCATCTACTCCATCATCTTCTTAACTGGCATTGTGGGCAATGGATTGGTCATCCT

GGTCATGGGTTACCAGAAGAAACTGAGAAGCATGACGGACAAGTACAGGCTGCACCTGTCAGTGGCCGAC

CTCCTCTTTGTCATCACGCTTCCCTTCTGGGCAGTTGATGCCGTGGCAAACTGGTACTTTGGGAACTTCC

TATGCAAGGCAGTCCATGTCATCTACACAGTCAACCTCTACAGCAGTGTCCTCATCCTGGCCTTCATCAG

TCTGGACCGCTACCTGGCCATCGTCCACGCCACCAACAGTCAGAGGCCAAGGAAGCTGTTGGCTGAAAAG

GTGGTCTATGTTGGCGTCTGGATCCCTGCCCTCCTGCTGACTATTCCCGACTTCATCTTTGCCAACGTCA

GTGAGGCAGATGACAGATATATCTGTGACCGCTTCTACCCCAATGACTTGTGGGTGGTTGTGTTCCAGTT

TCAGCACATCATGGTTGGCCTTATCCTGCCTGGTATTGTCATCCTGTCCTGCTATTGCATTATCATCTCC

AAGCTGTCACACTCCAAGGGCCACCAGAAGCGCAAGGCCCTCAAGACCACAGTCATCCTCATCCTGGCTT

TCTTCGCCTGTTGGCTGCCTTACTACATTGGGATCAGCATCGACTCCTTCATCCTCCTGGAAATCATCAA

GCAAGGGTGTGAGTTTGAGAACACTGTGCACAAGTGGATTTCCATCACCGAGGCCCTAGCTTTCTTCCAC

TGTTGTCTGAACCCCATCCTCTATGCTTTCCTTGGAGCCAAATTTAAAACCTCTGCCCAGCACGCACTCA

CCTCTGTGAGCAGAGGGTCCAGCCTCAAGATCCTCTCCAAAGGAAAGCGAGGTGGACATTCATCTGTTTC

CACTGAGTCTGAGTCTTCAAGTTTTCACTCCAGCTAACACAGATGTAAAAGACTTTTTTTTATACGATAA

ATAACTTTTTTTTAAGTTACACATTTTTCAGATATAAAAGACTGACCAATATTGTACAGTTTTTATTGCT

TGTTGGATTTTTGTCTTGTGTTTCTTTAGTTTTTGTGAAGTTTAATTGACTTATTTATATAAATTTTTTT

TGTTTCATATTGATGTGTGTCTAGGCAGGACCTGTGGCCAAGTTCTTAGTTGCTGTATGTCTCGTGGTAG

GACTGTAGAAAAGGGAACTGAACATTCCAGAGCGTGTAGTGAATCACGTAAAGCTAGAAATGATCCCCAG

CTGTTTATGCATAGATAATCTCTCCATTCCCGTGGAACGTTTTTCCTGTTCTTAAGACGTGATTTTGCTG

TAGAAGATGGCACTTATAACCAAAGCCCAAAGTGGTATAGAAATGCTGGTTTTTCAGTTTTCAGGAGTGG

GTTGATTTCAGCACCTACAGTGTACAGTCTTGTATTAAGTTGTTAATAAAAGTACATGTTAAACTTAAAA

AAAAAAAAAAAAAA

>NM_004938.4 Homo sapiens death associated protein kinase 1 (DAPK1), transcript variant 1, mRNA

GCTTCGGAGTGTGAGGAGGACAGCCGGACCGAGCCAACGCCGGGGACTTTGTTCCCTCCGCGGAGGGGAC

TCGGCAACTCGCAGCGGCAGGGTCTGGGGCCGGCGCCTGGGAGGGATCTGCGCCCCCCACTCACTCCCTA

GCTGTGTTCCCGCCGCCGCCCCGGCTAGTCTCCGGCGCTGGCGCCTATGGTCGGCCTCCGACAGCGCTCC

GGAGGGACCGGGGGAGCTCCCAGGCGCCCGGGACTGGAGACTGATGCATGAGGGGGCTACGGAGGCGCAG

GAGCGGTGGTGATGGTCTGGGAAGCGGAGCTGAAGTGCCCTGGGCTTTGGTGAGGCGTGACAGTTTATCA

TGACCGTGTTCAGGCAGGAAAACGTGGATGATTACTACGACACCGGCGAGGAACTTGGCAGTGGACAGTT

TGCGGTTGTGAAGAAATGCCGTGAGAAAAGCACCGGCCTCCAGTATGCCGCCAAATTCATCAAGAAAAGG

AGGACTAAGTCCAGCCGGCGGGGTGTGAGCCGCGAGGACATCGAGCGGGAGGTCAGCATCCTGAAGGAGA

TCCAGCACCCCAATGTCATCACCCTGCACGAGGTCTATGAGAACAAGACGGACGTCATCCTGATCTTGGA

ACTCGTTGCAGGTGGCGAGCTGTTTGACTTCTTAGCTGAAAAGGAATCTTTAACTGAAGAGGAAGCAACT

GAATTTCTCAAACAAATTCTTAATGGTGTTTACTACCTGCACTCCCTTCAAATCGCCCACTTTGATCTTA

AGCCTGAGAACATAATGCTTTTGGATAGAAATGTCCCCAAACCTCGGATCAAGATCATTGACTTTGGGTT

GGCCCATAAAATTGACTTTGGAAATGAATTTAAAAACATATTTGGGACTCCAGAGTTTGTCGCTCCTGAG

ATAGTCAACTATGAACCTCTTGGTCTTGAGGCAGATATGTGGAGTATCGGGGTAATAACCTATATCCTCC

TAAGTGGGGCCTCCCCATTTCTTGGAGACACTAAGCAAGAAACGTTAGCAAATGTATCCGCTGTCAACTA

CGAATTTGAGGATGAATACTTCAGTAATACCAGTGCCCTAGCCAAAGATTTCATAAGAAGACTTCTGGTC

AAGGATCCAAAGAAGAGAATGACAATTCAAGATAGTTTGCAGCATCCCTGGATCAAGCCTAAAGATACAC

AACAGGCACTTAGTAGAAAAGCATCAGCAGTAAACATGGAGAAATTCAAGAAGTTTGCAGCCCGGAAAAA

ATGGAAACAATCCGTTCGCTTGATATCACTGTGCCAAAGATTATCCAGGTCATTCCTGTCCAGAAGTAAC

ATGAGTGTTGCCAGAAGCGATGATACTCTGGATGAGGAAGACTCCTTTGTGATGAAAGCCATCATCCATG

CCATCAACGATGACAATGTCCCAGGCCTGCAGCACCTTCTGGGCTCATTATCCAACTATGATGTTAACCA

ACCCAACAAGCACGGGACACCTCCATTACTCATTGCTGCTGGCTGTGGGAATATTCAAATACTACAGTTG

CTCATTAAAAGAGGCTCGAGAATCGATGTCCAGGATAAGGGCGGGTCCAATGCCGTCTACTGGGCTGCTC

GGCATGGCCACGTCGATACCTTGAAATTTCTCAGTGAGAACAAATGCCCTTTGGATGTGAAAGACAAGTC

TGGAGAGATGGCCCTCCACGTGGCAGCTCGCTATGGCCATGCTGACGTGGCTCAGTTACTGTGCAGCTTC

GGCTCAAATCCCAATATCCAGGACAAGGAAGAAGAAACCCCCCTGCACTGTGCTGCTTGGCACGGCTATT

ACTCTGTGGCCAAAGCCCTTTGTGAAGCCGGCTGTAACGTGAACATCAAGAACCGAGAAGGAGAGACGCC

CCTCCTGACAGCCTCTGCCAGGGGCTACCACGACATCGTGGAGTGTCTGGCCGAACATGGAGCCGACCTT

AATGCTTGCGACAAGGACGGACACATTGCCCTTCATCTGGCTGTAAGACGGTGTCAGATGGAGGTAATCA

AGACTCTCCTCAGCCAAGGGTGTTTCGTCGATTATCAAGACAGGCACGGCAATACTCCCCTCCATGTGGC

ATGTAAAGATGGCAACATGCCTATCGTGGTGGCCCTCTGTGAAGCAAACTGCAATTTGGACATCTCCAAC

AAGTATGGGCGAACGCCTCTGCACCTTGCGGCCAACAACGGAATCCTAGACGTGGTCCGGTATCTCTGTC

TGATGGGAGCCAGCGTTGAGGCGCTGACCACGGACGGAAAGACGGCAGAAGATCTTGCTAGATCGGAACA

GCACGAGCACGTAGCAGGTCTCCTTGCAAGACTTCGAAAGGATACGCACCGAGGACTCTTCATCCAGCAG

CTCCGACCCACACAGAACCTGCAGCCAAGAATTAAGCTCAAGCTGTTTGGCCACTCGGGATCCGGGAAAA

CCACCCTTGTAGAATCTCTCAAGTGTGGGCTGCTGAGGAGCTTTTTCAGAAGGCGTCGGCCCAGACTGTC

TTCCACCAACTCCAGCAGGTTCCCACCTTCACCCCTGGCTTCTAAGCCCACAGTCTCAGTGAGCATCAAC

AACCTGTACCCAGGCTGCGAGAACGTGAGTGTGAGGAGCCGCAGCATGATGTTCGAGCCGGGTCTTACCA

AAGGGATGCTGGAGGTGTTTGTGGCCCCGACCCACCACCCGCACTGCTCGGCCGATGACCAGTCCACCAA

GGCCATCGACATCCAGAACGCTTATTTGAATGGAGTTGGCGATTTCAGCGTGTGGGAGTTCTCTGGAAAT

CCTGTGTATTTCTGCTGTTATGACTATTTTGCTGCAAATGATCCCACGTCAATCCATGTTGTTGTCTTTA

GTCTAGAAGAGCCCTATGAGATCCAGCTGAACCAAGTGATTTTCTGGCTCAGTTTCCTGAAGTCCCTTGT

CCCAGTTGAAGAACCCATAGCCTTCGGTGGCAAGCTGAAGAACCCACTCCAAGTTGTCCTGGTGGCCACC

CACGCTGACATCATGAATGTTCCTCGACCGGCTGGAGGCGAGTTTGGATATGACAAAGACACATCGTTGC

TGAAAGAGATTAGGAACAGGTTTGGAAATGATCTTCACATTTCAAATAAGCTGTTTGTTCTGGATGCTGG

GGCTTCTGGGTCAAAGGACATGAAGGTACTTCGAAATCATCTGCAAGAAATACGAAGCCAGATTGTTTCG

GTCTGTCCTCCCATGACTCACCTGTGTGAGAAAATCATCTCCACGCTGCCTTCCTGGAGGAAGCTCAATG

GACCCAACCAGCTGATGTCGCTGCAGCAGTTTGTGTACGACGTGCAGGACCAGCTGAACCCCCTGGCCAG

CGAGGAGGACCTCAGGCGCATTGCTCAGCAGCTCCACAGCACAGGCGAGATCAACATCATGCAAAGTGAA

ACAGTTCAGGACGTGCTGCTCCTGGACCCCCGCTGGCTCTGCACAAACGTCCTGGGGAAGTTGCTGTCCG

TGGAGACCCCACGGGCGCTGCACCACTACCGGGGCCGCTACACCGTGGAGGACATCCAGCGCCTGGTGCC

CGACAGCGACGTGGAGGAGCTGCTGCAGATCCTCGATGCCATGGACATCTGCGCCCGGGACCTGAGCAGC

GGGACCATGGTGGACGTCCCAGCCCTGATCAAGACAGACAACCTGCACCGCTCCTGGGCTGATGAGGAGG

ACGAGGTGATGGTGTATGGTGGCGTGCGCATCGTGCCCGTGGAACACCTCACCCCCTTCCCATGTGGCAT

CTTTCACAAGGTCCAGGTGAACCTGTGCCGGTGGATCCACCAGCAAAGCACAGAGGGCGACGCGGACATC

CGCCTGTGGGTGAATGGCTGCAAGCTGGCCAACCGTGGGGCCGAGCTGCTGGTGCTGCTGGTCAACCACG

GCCAGGGCATTGAGGTCCAGGTCCGCGGCCTGGAGACGGAGAAGATCAAGTGCTGCCTGCTGCTGGACTC

GGTGTGCAGCACCATTGAGAACGTCATGGCCACCACGCTGCCAGGGCTCCTGACCGTGAAGCATTACCTG

AGCCCCCAGCAGCTGCGGGAGCACCATGAGCCCGTCATGATCTACCAGCCACGGGACTTCTTCCGGGCAC

AGACTCTGAAGGAAACCTCACTGACCAACACCATGGGGGGGTACAAGGAAAGCTTCAGCAGCATCATGTG

CTTCGGGTGTCACGACGTCTACTCACAGGCCAGCCTCGGCATGGACATCCATGCATCAGACCTGAACCTC

CTCACTCGGAGGAAACTGAGTCGCCTGCTGGACCCGCCCGACCCCCTGGGGAAGGACTGGTGCCTTCTCG

CCATGAACTTAGGCCTCCCTGACCTCGTGGCAAAGTACAACACCAGTAACGGGGCTCCCAAGGATTTCCT

CCCCAGCCCCCTCCACGCCCTGCTGCGGGAATGGACCACCTACCCTGAGAGCACAGTGGGCACCCTCATG

TCCAAACTGAGGGAGCTGGGTCGCCGGGATGCCGCAGACTTTTTGCTGAAGGCATCCTCTGTGTTCAAAA

TCAACCTGGATGGCAATGGCCAGGAGGCCTATGCCTCGAGCTGCAACAGCGGCACCTCTTACAATTCCAT

TAGCTCTGTTGTATCCCGGTGAGGGCAGCCTCTGGCTTGGGCAGGGTCTGTTTGGACTGCAGAAGCAAGG

GGGTGATGTAGCCCATCCTTCCCTTTGGAGATGCTGAGGGTGTTTCTTCCTGCACCCACAGCCAGGGGGA

TGCCACTCCTCCCTCCGGCTTGACCTGTTTCTCTGCCGCTACCTCCCTCCCCGTCTCATTCCGTTGTCTG

TGGATGGTCATTGCAGTTTAAGAGCAGAACAGATCTTTTACTTTGGCCGCTTGAAAAGCTAGTGTACCTC

CTCTCAGTGTTTTGGACTCCATCTCTCATCCTCCAGTACCTTGCTTCTTACTGATAATTTTGCTGGAATT

CCTAACTTTTCAATGACATTTTTTTTAACTACTATATTGATTGTCCTTTAAAAAAGAAAAGTGCATATTT

ATCCAAAATGTGTATTTCTTATACGCTTTTCTTTGTTATACCATTTCCTCAGCTTATCTCTTTTATATTT

GTAGGAGAAACTCCCATGTATGGAATCCCACTGTATGATTTATAAACAGACAATATGTGAGTGCCTTTTG

CAGAAGAGGGTGTGTTTGAAATCATCGGAGTCAGCCAGGAGCTGTCACCAAGGAAACGCTACCTCTCTGT

CCCTTGCTGTATGCTGATCATCGCCAGAGGTGCTTCACCCTGAGTTTTGTTTTGTATTGTTTTCTGACAG

TTTTTCTGTTTTGTTTGGCAAGGAAAGGGGAGAAGGGAATCCTCCTCCAGGGTGATTTTATGATCAGTGT

TGTTGCTCTAGGAAGACATTTTTCCGTTTGCTTTTGTTCCAATGTCAATGTGAACGTCCACATGAAACCT

ACACACTGTCATGCTTCATCATTCCCTCTCATCTCAGGTAGAAGGTTGACACAGTTGTAGGGTTACAGAG

ACCTATGTAAGAATTCAGAAGACCCCTGACTCATCATTTGTGGCAGTCCCTTATAATTGGTGCATAGCAG

ATGGTTTCCACATTTAGATCCTGGTTTCATAACTTCCTGTACTTGAAGTCTAAAAGCAGAAAATAAAGGA

AGCAAGTTTTCTTCCATGATTTTAAATTGTGATCGAGTTTTAAATTGATAGGAGGGAACATGTCCTAATT

CTTCTGTCCTGAGAAGCATGTAATGTTAATGTTATATCATATGTATATATATATATGCACTATGTATATA

CATATATATTAATACTGGTATTTTTACTTAATCTATAAAATGTCGTTAAAAAGTTGTTTGTTTTTTTCTT

TTTTTATAAATAAACTGTTGCTCGTTGCATTA

>NM_014326.5 Homo sapiens death associated protein kinase 2 (DAPK2), transcript variant 1, mRNA

CTCGGCTCCTAGGCTGCTCGGCGGGCGGGTGCCGGGCGCTCTGGGAGGAGAGGACTGCAGGGCCGAGCCC

AGCGCACCCCGCCGCGTCCCCCGTTCCCGCCGCGCCCGCCGCGCCCGCCGCGCCCGCCGCGCCCGCCGCT

CCCGCCGCGCCTGCCGCGGCCGCGACCGCGGCAGCTCAGCCTCCCGCCGATTGTATGTTCCAGGCCTCAA

TGAGGAGTCCAAACATGGAGCCATTCAAGCAGCAGAAGGTGGAGGACTTTTATGACATCGGAGAGGAGCT

GGGGAGTGGCCAGTTTGCCATCGTGAAGAAGTGCCGGGAGAAGAGCACGGGGCTTGAGTATGCAGCCAAG

TTCATCAAGAAGCGGCAGAGCCGGGCGAGCCGGCGCGGTGTGAGCCGGGAGGAGATCGAGCGGGAGGTGA

GCATCCTGCGGCAGGTGCTGCACCACAATGTCATCACGCTGCACGACGTCTATGAGAACCGCACCGACGT

GGTGCTCATCCTTGAGCTAGTGTCTGGAGGAGAGCTCTTCGATTTCCTGGCCCAGAAGGAGTCACTGAGT

GAGGAGGAGGCCACCAGCTTCATTAAGCAGATCCTGGATGGGGTGAACTACCTTCACACAAAGAAAATTG

CTCACTTTGATCTCAAGCCAGAAAACATTATGTTGTTAGACAAGAATATTCCCATTCCACACATCAAGCT

GATTGACTTTGGTCTGGCTCACGAAATAGAAGATGGAGTTGAATTTAAGAATATTTTTGGGACGCCGGAA

TTTGTTGCTCCAGAAATTGTGAACTACGAGCCCCTGGGTCTGGAGGCTGACATGTGGAGCATAGGCGTCA

TCACCTACATCCTCTTAAGTGGAGCATCCCCTTTCCTGGGAGACACGAAGCAGGAAACACTGGCAAATAT

CACAGCAGTGAGTTACGACTTTGATGAGGAATTCTTCAGCCAGACGAGCGAGCTGGCCAAGGACTTTATT

CGGAAGCTTCTGGTTAAAGAGACCCGGAAACGGCTCACAATCCAAGAGGCTCTCAGACACCCCTGGATCA

CGCCGGTGGACAACCAGCAAGCCATGGTGCGCAGGGAGTCTGTGGTCAATCTGGAGAACTTCAGGAAGCA

GTATGTCCGCAGGCGGTGGAAGCTTTCCTTCAGCATCGTGTCCCTGTGCAACCACCTCACCCGCTCGCTG

ATGAAGAAGGTGCACCTGAGGCCGGATGAGGACCTGAGGAACTGTGAGAGTGACACTGAGGAGGACATCG

CCAGGAGGAAAGCCCTCCACCCACGGAGGAGGAGCAGCACCTCCTAACTGGCCTGACCTGCAGTGGCCGC

CAGGGAGGTCTGGGCCCAGCGGGGCTCCCTTCTGTGCAGACTTTTGGACCCAGCTCAGCACCAGCACCCG

GGCGTCCTGAGCACTTTGCAAGAGAGATGGGCCCAAGGAATTCAGAAGAGCTTGCAGGCAAGCCAGGAGA

CCCTGGGAGCTGTGGCTGTCTTCTGTGGAGGAGGCTCCAGCATTCCCAAAGCTCTTAATTCTCCATAAAA

TGGGCTTTCCTCTGTCTGCCATCCTCAGAGTCTGGGGTGGGAGTGTGGACTTAGGAAAACAATATAAAGG

ACATCCTCATCATCACGGGGTGAAGGTCAGACTAAGGCAGCCTTCTTCACAGGCTGAGGGGGTTCAGAAC

CAGCCTGGCCAAAAATTACACCAGAGAGACAGAGTCCTCCCCATTGGGAACAGGGTGATTGAGGAAAGTG

AACCTTGGGTGTGAGGGACCAATCCTGTGACCTCCCAGAACCATGGAAGCCAGGACGTCAGGCTGACCAA

CACCTCAGACCTTCTGAAGCAGCCCATTGCTGGCCCGCCATGTTGTAATTTTGCTCATTTTTATTAAACT

TCTGGTTTACCTGATGCTTGGCTTCTTTTAGGGCTACCCCCATCTCATTTCCTTTAGCCCGTGTGCCTGT

AACTCTGAGGGGGGGCACCCAGTGGGGTGCTGAGTGGGCAGAATCTCAGAAGGTCCTCCTGAACCGTCCG

CGCAGGCCTGCAGTGGGCCTGCCTCCTCCTTGCTTCCCTAACAGGAAGGTGTCCAGTTCAAGAGAACCCA

CCCAGAGACTGGGAGTGGTGGCTCACGCCTATAATCCCTGCGCTTTGGCAGTCCGAGGCAGGGGAATTGC

TTGAACTCAGGAGTTGGAGACCAGCCTGGGCAACATGGCAAAACGCAGTCTGTACAAAAAATACAAAAAA

TTAGCCAGGTGTAGGGGTAGGCACCTGGCATCCCAGCTACTCCAGGGGCTGAGGTGACAGCATTGCTTAA

GCCCAGAAGGTCGAGGCTGCAGTGAGCTGAGATCACGCCACTGCACTCCAGTCTGGGTGACAGAGAGAGA

CCATATCCAAAAAAAAAAAAAAGTTGCCAGAGACGAGTATGCCCATGCTCCCTCTACCTCACTGCCACCAC

TCCTGCTGTTAGGAGCTGAGTGTGTCTCCCTAAAATTTCTATGTTGAAGTCTTAACCCTTGGTACCACAG

AATATCACTGTATTTGGAGATGGGGTCTTTAGAAAGGCACTTAAATTAAAATGAGCTCACTGATATGGGC

CCCGATGCAATATAATTGGTGTCCTTATAAGAAGGGGAGGTTAGGACACGCAGGAAAGACCACATGAAGG

CCCAGGAGTGGGAGGGGGAATAGCCATCGACAAACTAAGGGGGCCTCAGAGGAAACCAACCCTGCTGACA

CCTCAATCTTAGACTCTGGCCTCAAAAATTGTAAGAAAATAAACTTCTGTCTTTTAAGCCA

>NM_004083.6 Homo sapiens DNA damage inducible transcript 3 (DDIT3), transcript variant 5, mRNA

GTCAGAGACTTAAGTCTAAGGCACTGAGCGTATCATGTTAAAGATGAGCGGGTGGCAGCGACAGAGCCAA

AATCAGAGCTGGAACCTGAGGAGAGAGTGTTCAAGAAGGAAGTGTATCTTCATACATCACCACACCTGAA

AGCAGATGTGCTTTTCCAGACTGATCCAACTGCAGAGATGGCAGCTGAGTCATTGCCTTTCTCCTTCGGG

ACACTGTCCAGCTGGGAGCTGGAAGCCTGGTATGAGGACCTGCAAGAGGTCCTGTCTTCAGATGAAAATG

GGGGTACCTATGTTTCACCTCCTGGAAATGAAGAGGAAGAATCAAAAATCTTCACCACTCTTGACCCTGC

TTCTCTGGCTTGGCTGACTGAGGAGGAGCCAGAACCAGCAGAGGTCACAAGCACCTCCCAGAGCCCTCAC

TCTCCAGATTCCAGTCAGAGCTCCCTGGCTCAGGAGGAAGAGGAGGAAGACCAAGGGAGAACCAGGAAAC

GGAAACAGAGTGGTCATTCCCCAGCCCGGGCTGGAAAGCAGCGCATGAAGGAGAAAGAACAGGAGAATGA

AAGGAAAGTGGCACAGCTAGCTGAAGAGAATGAACGGCTCAAGCAGGAAATCGAGCGCCTGACCAGGGAA

GTAGAGGCGACTCGCCGAGCTCTGATTGACCGAATGGTGAATCTGCACCAAGCATGAACAATTGGGAGCA

TCAGTCCCCCACTTGGGCCACACTACCCACCTTTCCCAGAAGTGGCTACTGACTACCCTCTCACTAGTGC

CAATGATGTGACCCTCAATCCCACATACGCAGGGGGAAGGCTTGGAGTAGACAAAAGGAAAGGTCTCAGC

TTGTATATAGAGATTGTACATTTATTTATTACTGTCCCTATCTATTAAAGTGACTTTCTATGA

>NM_004675.5 Homo sapiens DIRAS family GTPase 3 (DIRAS3), mRNA

GTGTCCAGTTGGTTGCCGCGGCAGTCTCTCCGAGCAGCGCATTTGTCTTCTAGGCTGCTTGGTTCGTGCC

TCCGAGAAAGGGGTCTCCTGCTGCCAGCTAAGTGTGGGAGAACTTGTGCACGTATCTCCCCTCCGAATCC

CAACGATGGGTAACGCCAGCTTTGGCTCCAAGGAACAGAAGCTGCTGAAGCGGTTGCGGCTTCTGCCCGC

CCTGCTTATCCTCCGCGCCTTCAAGCCCCACAGGAAGATCAGAGATTACCGCGTCGTGGTAGTCGGCACC

GCTGGTGTGGGGAAAAGTACGCTGCTGCACAAGTGGGCGAGCGGCAACTTCCGTCATGAGTACCTGCCGA

CCATTGAAAATACCTACTGCCAGTTGCTGGGCTGCAGCCACGGTGTGCTTTCCCTGCACATCACCGACAG

CAAGAGTGGCGACGGCAACCGCGCTCTGCAGCGCCACGTTATAGCCCGGGGCCACGCCTTCGTCCTGGTC

TACTCAGTCACCAAGAAGGAAACCCTGGAAGAGCTGAAGGCCTTCTATGAGCTGATCTGCAAGATCAAAG

GTAACAACCTGCATAAGTTCCCCATCGTGCTGGTGGGCAATAAAAGTGATGACACCCACCGGGAGGTGGC

CCTGAATGATGGTGCCACCTGTGCGATGGAGTGGAATTGCGCCTTCATGGAGATTTCAGCCAAGACCGAT

GTGAATGTGCAGGAGCTGTTCCACATGCTGCTGAATTACAAGAAAAAGCCCACCACCGGCCTCCAGGAGC

CCGAGAAGAAATCCCAGATGCCCAACACCACTGAGAAGCTGCTTGACAAGTGCATAATCATGTGAGCCCT

GGGCCTTAAGAGCCAGCTCTTCCTATCCTGTAGCGTGTAGAAAACGTGGACTCATTTCACTATGTTACAT

GTACATGGTTGATTTTGTGCTGTTGTTTGGACTGTAACATCCATGTTGTCAATACGTATACCTTGTAAGT

GGATAACTTTTCTTTTTCCCAGGCCAGAGAATTCAAATTGTTAAAACATTGGCATTTGAAGAGGAGAACA

AAATGTAGCATGATGTATTTAAAGTAAGGCCTTTAGTAATGAATGTAATGAGAGAAAATGTTTTGAAAAG

AACAAAACATCAAAATGAATAGAAAGAAAAATTGGAAGGCGTCCTTTTGGTAACCCGATTATTGTGTATT

ACCTTTAAATATTTCACATCCTGTAAGTGCTTAATCATATCTTTTAATTGTGTATTTAAGAAAAGTGTTT

TCACAACAAAAGCTTTTGATAAATTGCTGCGTGACATATACTAAATAAAAAAATGAATATGTTGATCATT

AGGGGTGTGGGAGCAGAGAAAATTGTGAAAGTGACTCTCACTAAAGATGTTAGTAGTTTCTCATGTCATT

TAAAAATGTTTGAGTATTCTGCATAGCAGTTTGTAAAAGTGTAACAGCTTATTGACTTAATAAAGCTTTT

CCTGCATGCAA

>NM_182643.3 Homo sapiens DLC1 Rho GTPase activating protein (DLC1), transcript variant 1, mRNA

AGTCTGAATTTAGTAGACAGAAGTCACTAGGAATGCCTTGACAGGATCCTGCCTTAGCTAAGGCTCCCTC

CAGCTGCAGAGGGTGTTTTTGTTAGACTCACACACTGCGTGAAACTGCTCAGAATAGAGCCATGATCTCA

ACCACGAAATGGGAACTTAGATTTTGGAGAAACTAACGGGGACGGACTTCTTTCCTAGCCTGAGTGTTGA

GCAGTGTCATGCCTTGGCGTTTCAGCTCCTCGTTGTCTAGGTGGTGAAATGACAGAACTCATTCGCTTCT

TTGATTGGTGATTTTGAAATAATCTTTCATCAAGTTCCATCTCCTTTACCCTCATATGGAATATATCTCT

CTGTCTGTTGTTAAACTACGATGACATGTCTGTAGCTATCAGAAAGAGAAGCTGGGAAGAACATGTGACC

CACTGGATGGGACAGCCTTTTAATTCTGATGATCGTAACACAGCATGTCATCATGGACTAGTAGCTGACA

GCTTGCAGGCAAGTATGGAAAAAGATGCAACTCTAAATGTGGACCGCAAAGAGAAGTGTGTTTCACTACC

TGACTGCTGTCATGGATCAGAGCTGAGAGATTTTCCTGGGAGGCCAATGGGTCATCTTTCAAAGGATGTG

GACGAAAATGACAGCCATGAAGGTGAAGATCAGTTTCTTTCTCTGGAAGCCAGCACAGAAACACTAGTGC

ATGTTTCTGATGAGGATAACAATGCTGATTTATGCCTTACAGATGATAAACAGGTTTTAAATACCCAAGG

GCAGAAAACATCAGGCCAACATATGATCCAAGGAGCAGGCTCCTTAGAAAAGGCACTGCCCATCATACAA

AGTAACCAAGTTTCTTCTAACTCCTGGGGAATAGCTGGTGAAACTGAATTAGCACTGGTAAAAGAAAGTG

GGGAGAGAAAAGTTACTGACTCTATAAGTAAAAGCCTGGAGCTTTGCAATGAAATAAGCTTAAGTGAAAT

AAAAGATGCACCCAAAGTAAATGCAGTGGATACTTTGAACGTGAAAGATATTGCACCTGAGAAACAATTG

CTTAACTCTGCTGTAATTGCTCAGCAACGAAGGAAACCTGACCCCCCTAAAGATGAAAATGAAAGAAGCA

CCTGCAATGTAGTACAAAATGAGTTCTTGGATACTCCTTGCACAAACAGAGGACTGCCATTATTAAAAAC

AGATTTTGGAAGCTGCCTTCTGCAGCCTCCTTCCTGCCCCAATGGAATGTCAGCTGAAAATGGCCTGGAG

AAGAGTGGTTTTTCACAACATCAAAACAAAAGTCCACCAAAGGTCAAGGCAGAAGATGGCATGCAGTGTT

TACAATTAAAGGAGACCCTGGCCACCCAGGAACCCACAGATAACCAAGTCAGACTTCGTAAGAGAAAGGA

AATAAGAGAAGATCGAGATAGGGCGCGGCTGGACTCCATGGTGCTGCTGATTATGAAACTGGACCAGCTT

GATCAGGACATAGAAAATGCCCTCAGCACCAGCTCCTCTCCATCAGGCACACCAACAAACCTGCGGCGGC

ACGTTCCTGATCTGGAATCAGGATCTGAAAGTGGAGCAGATACCATTTCAGTAAATCAGACACGAGTAAA

TTTGTCTTCTGACACTGAGTCCACGGACCTCCCATCTTCCACTCCAGTAGCCAATTCTGGAACCAAACCC

AAGACTACGGCTATTCAAGGTATTTCAGAGAAGGAAAAGGCTGAAATTGAAGCCAAGGAAGCTTGTGATT

GGCTACGGGCAACTGGTTTCCCCCAGTATGCACAGCTTTATGAAGATTTCCTGTTCCCCATCGATATTTC

CTTGGTCAAGAGAGAGCATGATTTTTTGGACAGAGATGCCATTGAGGCTCTATGCAGGCGTCTAAATACT

TTAAACAAATGTGCGGTGATGAAGCTAGAAATTAGTCCTCATCGGAAACGAAGTGACGATTCAGACGAGG

ATGAGCCTTGTGCCATCAGTGGCAAATGGACTTTCCAAAGGGACAGCAAGAGGTGGTCCCGGCTTGAAGA

GTTTGATGTCTTTTCTCCAAAACAAGACCTGGTCCCTGGGTCCCCAGACGACTCCCACCCGAAGGACGGC

CCCAGCCCCGGAGGCACGCTGATGGACCTCAGCGAGCGCCAGGAGGTGTCTTCCGTCCGCAGCCTCAGCA

GCACTGGCAGCCTCCCCAGCCACGCGCCCCCCAGCGAGGATGCTGCCACCCCCCGGACTAACTCCGTCAT

CAGCGTTTGCTCCTCCAGCAACTTGGCAGGCAATGACGACTCTTTCGGCAGCCTGCCCTCTCCCAAGGAA

CTGTCCAGCTTCAGCTTCAGCATGAAAGGCCACGAAAAAACTGCCAAGTCCAAGACGCGCAGTCTGCTGA

AACGGATGGAGAGCCTGAAGCTCAAGAGCTCCCATCACAGCAAGCACAAAGCGCCCTCAAAGCTGGGGTT

GATCATCAGCGGGCCCATCTTGCAAGAGGGGATGGATGAGGAGAAGCTGAAGCAGCTCAACTGCGTGGAG

ATCTCCGCCCTCAATGGCAACCGCATCAACGTCCCCATGGTACGAAAGAGGAGCGTTTCCAACTCCACGC

AGACCAGCAGCAGCAGCAGCCAGTCGGAGACCAGCAGCGCGGTCAGCACGCCCAGCCCTGTTACGAGGAC

CCGGAGCCTCAGTGCGTGCAACAAGCGGGTGGGCATGTACTTAGAGGGCTTCGATCCTTTCAATCAGTCA

ACATTTAACAACGTGGTGGAGCAGAACTTTAAGAACCGCGAGAGCTACCCAGAGGACACGGTGTTCTACA

TCCCTGAAGATCACAAGCCTGGCACTTTCCCCAAAGCTCTCACCAATGGCAGTTTCTCCCCCTCGGGGAA

TAACGGCTCTGTGAACTGGAGGACGGGAAGCTTCCACGGCCCTGGCCACATCAGCCTCAGGAGGGAAAAC

AGTAGCGACAGCCCCAAGGAACTGAAGAGACGCAATTCTTCCAGCTCCATGAGCAGCCGCCTGAGCATCT

ACGACAACGTGCCGGGCTCCATCCTCTACTCCAGTTCAGGGGACCTGGCGGATCTGGAGAACGAGGACAT

CTTCCCCGAGCTGGACGACATCCTCTACCACGTGAAGGGGATGCAGCGGATAGTCAATCAGTGGTCGGAG

AAGTTTTCTGATGAGGGAGATTCGGACTCAGCCCTGGACTCGGTCTCTCCCTGCCCGTCCTCTCCAAAAC

AGATACACCTGGATGTGGACAACGACCGAACCACACCCAGCGACCTGGACAGCACAGGCAACTCCCTGAA

TGAACCGGAAGAGCCCTCCGAGATCCCGGAAAGAAGGGATTCTGGGGTTGGGGCTTCCCTAACCAGGTCC

AACAGGCACCGACTGAGATGGCACAGTTTCCAGAGCTCACATCGGCCAAGCCTCAACTCTGTATCACTAC

AGATTAACTGCCAGTCTGTGGCCCAGATGAACCTGCTGCAGAAATACTCACTCCTAAAGCTAACGGCCCT

GCTGGAGAAATACACACCTTCTAACAAGCATGGTTTTAGCTGGGCCGTGCCCAAGTTCATGAAGAGGATC

AAGGTTCCAGACTACAAGGACCGGAGTGTGTTTGGGGTCCCACTGACGGTCAACGTGCAGCGCACAGGAC

AACCGTTGCCTCAGAGCATCCAGCAGGCCATGCGATACCTCCGGAACCATTGTTTGGATCAGGTTGGGCT

CTTCAGAAAATCGGGGGTCAAGTCCCGGATTCAGGCTCTGCGCCAGATGAATGAAGGTGCCATAGACTGT

GTCAACTACGAAGGACAGTCTGCTTATGACGTGGCAGACATGCTGAAGCAGTATTTTCGAGATCTTCCTG

AGCCACTAATGACGAACAAACTCTCGGAAACCTTTCTACAGATCTACCAATATGTGCCCAAGGACCAGCG

CCTGCAGGCCATCAAGGCTGCCATCATGCTGCTGCCTGACGAGAACCGGGAGGTTCTGCAGACCCTGCTT

TATTTCCTGAGCGATGTCACAGCAGCCGTAAAAGAAAACCAGATGACCCCAACCAACCTGGCCGTGTGCT

TAGCGCCTTCCCTCTTCCATCTCAACACCCTGAAGAGAGAGAATTCCTCTCCCAGGGTAATGCAAAGAAA

ACAAAGTTTGGGCAAACCAGATCAGAAAGATTTGAATGAAAACCTAGCTGCCACTCAAGGGCTGGCCCAT

ATGATCGCCGAGTGCAAGAAGCTTTTCCAGGTTCCCGAGGAAATGAGCCGATGTCGTAATTCCTATACCG

AACAAGAGCTGAAGCCCCTCACTCTGGAAGCACTCGGGCACCTGGGTAATGATGACTCAGCTGACTACCA

ACACTTCCTCCAGGACTGTGTGGATGGCCTGTTTAAAGAAGTCAAAGAGAAGTTTAAAGGCTGGGTCAGC

TACTCCACTTCGGAGCAGGCTGAGCTGTCCTATAAGAAGGTGAGCGAAGGACCCCCTCTGAGGCTTTGGA

GGTCAGTCATTGAAGTCCCTGCTGTGCCAGAGGAAATCTTAAAGCGCCTACTTAAAGAACAGCACCTCTG

GGATGTAGACCTGTTGGATTCAAAAGTGATCGAAATTCTGGACAGCCAAACTGAAATTTACCAGTATGTC

CAAAACAGTATGGCACCTCATCCTGCTCGAGACTACGTTGTTTTAAGAACCTGGAGGACTAATTTACCCA

AAGGAGCCTGTGCCCTTTTACTAACCTCTGTGGATCACGATCGCGCACCTGTGGTGGGTGTGAGGGTTAA

TGTGCTCTTGTCCAGGTATTTGATTGAACCCTGTGGGCCAGGAAAATCCAAACTCACCTACATGTGCAGA

GTTGACTTAAGGGGCCACATGCCAGAATGGTACACAAAATCTTTTGGACATTTGTGTGCAGCTGAAGTTG

TAAAGATCCGGGATTCCTTCAGTAACCAGAACACTGAAACCAAAGACACCAAATCTAGGTGATCACTGAA

GCAACGCAACCGCTTCCACCACCATGGTGTTTGTTTCTAGAACTTTTGCCAGTCCTTGAAGAATGGGTTC

TGTGTCTAATCCTGAAACAAAGAAAACTACAAGCTGGAGTGTAGGAATTGACTATAGCAATTTGATACAT

TTTTAAAGCTGCTTCCTGTTTGTTGAGGGTCTGTATTCATAGACCTTGACTGGAATATGTAAGACTGTGC

AAAAAAAAAAAAAAAATTTATGTGTATTCTTATTCAAATTGCTTCTGAGAAGCAAAACTCTTTAAATACA

TTATGGAAGATAATGAAGATACTTCATTCTCTTGTGATATCAGTGTATGCGTACCTGTGTCGCTTTATTT

GCAGTGTGTTGAGGGACTGGTGTATCCACTGGAATAGTTGGTACTCTTGGATGTGTTTTCTCACCAAGAT

GAGCAAAGAAAGGTTTGCACAGAGGAGTGTGAATGTGTGTTTGTTGCTGGCTGAATGGCAATAGATGTCT

AAGGTGGATTCAGTGTCTGGCACACTGAGACACCTCCAAGAAGGAGATTGATGCATCAGGTTCAGTTTAA

CCTGGAATATCTGACTACCCCTGAATCCACCCAGAAAGGGGGCCCAACACCCTTGTCCATTTATGGGTAT

TTTTTTTCGAAGTTATTAAGCATATTCCTTTTCCACGAACCTCTTCTGTACTTTGATTGTAATAGGTTGG

CTCTTACACCCATTCCAAATGCAGTTTATTTTTAGACCCGATTGCAAATAGTGATGTAGTTTTAACCAGT

ATGGATTAGTTCAGGGATGAACTGCTCCCTCTAGCCTTACTGGCTCTGATCCACAGGGTTTTGTTTTGTT

TTGTTTTGTTTTTTGTTTAAGTCGAGATATAAAAACTGAACACGATAACACTTACTCTTAAATCAAGCAT

CAACACTTTTTCCCTGTTAGAATTCTTTGCATTTTTGTGTTTGTAACAGAAACGCCTTAAGACACTATGT

TTGGGAATATAGGAAACTATGTGTGTCCCAAGGAAATCCCTGTAAATTTAACTCACCTACAAAAGGCTTT

TTCCCCGCCTTTGGTTGTTAACGGCATTCCTGAAAGCCACATGTGTTTATTCATTGGGCTTGTTCTTATC

AGCAAATAGGTTTTCTGGTTTTATGACTTTTTGTCTTATTTTATTTTTCCTACATTTCTTTTTTTTTTTT

TTTCCTTTAGAATGCCCTGGAAATATATTTAAGTGGTAATGAAAAATAGTAATCATAGTAAAACGCAACA

AGAAGAAAACCAACCCAAACCAGTGAAGTTTTTTAGAACCTTTAGAAGGGTGGTCTTTATTCAGGTTTTA

CTGTAATGGTAAGGATTGACTCAAGAGACAGTATTAGTAAATTTATTGTGTATGGATCAAAAGTGAATAA

TGTATGAATGAGAGCTGTAAGAAGGATTTTTATTTTGTTATAATTTAGTTACCATTTTCAGTGTTATTTC

AAAGGTTCTTTGAAGAATTTTGGGGCAGGGCATCAGATTAGAGTTTTAAAATTTGAGTATTTTGGATATC

AGTGTTCCTCATGAAGATATACATGGATATTCAATTTTGATGGCTTCCAGATTTGTAAGATTGTATGTTG

TATATACCATTCTATTAAGAAACATGTCCACTGTGCTTTCAAACATAGATAAAGCATGATAAAGATTATT

ATTTAAGATATACTTGTATTTATACCTCAGATATTCTTTTGGGTTTTGTACCTCAAGGCTTTTTTCTTCT

TATTGTAAATACACTTTACGTGAATACAGTCTAAGTGAAGAAAATAAATAAAAGGAAGAGGTTTATAACT

TGCTCTATATCTGTACAGATTATAATCAATAAGTGCACTATTATTAAATGTTTAAAGTAAGGGAAAAGTC

TGGGCTGCCTTCCTTAATATTGCATCTCACTCCCACCCTTAAAACCACAGATTGCAAAGCATAGCATTTT

AGCATCAACTACAATCAAAAGAGCGATTTGCTGAAGGAAAAATCGGACTGCAAATCATTCCAAGGCCAAA

CTGCAACTGAGCCACCCACTCCCAAACAGGAAACCCTGGTGAAGGTTCAGGAAGCACGGAGATTCTCTCC

AACAAAGGTCCAGTTAGGAAACGACGCTGAGAGGATGACGACAACGTGCAACAGCAGAAAGATGCTTGCA

AGCAGAGTCAGGGTCACCAGTGAATGCCACAAAAGTTCTCTTTCCCACTGTTTAATTTGACAAGAGAAGA

ATTTGAAGGATATGAACATTTTCAAGAACTCTGCTGAGGTCACTTAGAGCGCCATCACAACTTATTTGTG

TGACTAATTGCCTAGATTGTAAGCTCTTTGAGGGCAGGGCTTGTCTCTTACACATCTTTATAATCCCCTG

CAGCGGCTTTCAGTATTTTGTACTTGTAGGCACCTAATAAATTTATTATTTGCTATACTGAA

>NM_006145.3 Homo sapiens DnaJ heat shock protein family (Hsp40) member B1 (DNAJB1), transcript variant 1, mRNA

GGAGCCGGGGGACGGCGACAGCGGGTCGGCGGGCCGCAGGAGGGGGTCATGGGTAAAGACTACTACCAGA

CGTTGGGCCTGGCCCGCGGCGCGTCGGACGAGGAGATCAAGCGGGCCTACCGCCGCCAGGCGCTGCGCTA

CCACCCGGACAAGAACAAGGAGCCCGGCGCCGAGGAGAAGTTCAAGGAGATCGCTGAGGCCTACGACGTG

CTCAGCGACCCGCGCAAGCGCGAGATCTTCGACCGCTACGGGGAGGAAGGCCTAAAGGGGAGTGGCCCCA

GTGGCGGTAGCGGCGGTGGTGCCAATGGTACCTCTTTCAGCTACACATTCCATGGAGACCCTCATGCCAT

GTTTGCTGAGTTCTTCGGTGGCAGAAATCCCTTTGACACCTTTTTTGGGCAGCGGAACGGGGAGGAAGGC

ATGGACATTGATGACCCATTCTCTGGCTTCCCTATGGGCATGGGTGGCTTCACCAACGTGAACTTTGGCC

GCTCCCGCTCTGCCCAAGAGCCCGCCCGAAAGAAGCAAGATCCCCCAGTCACCCACGACCTTCGAGTCTC

CCTTGAAGAGATCTACAGCGGCTGTACCAAGAAGATGAAAATCTCCCACAAGCGGCTAAACCCCGACGGA

AAGAGCATTCGAAACGAAGACAAAATATTGACCATCGAAGTGAAGAAGGGGTGGAAAGAAGGAACCAAAA

TCACTTTCCCCAAGGAAGGAGACCAGACCTCCAACAACATTCCAGCTGATATCGTCTTTGTTTTAAAGGA

CAAGCCCCACAATATCTTTAAGAGAGATGGCTCTGATGTCATTTATCCTGCCAGGATCAGCCTCCGGGAG

GCTCTGTGTGGCTGCACAGTGAACGTCCCCACTCTGGACGGCAGGACGATACCCGTCGTATTCAAAGATG

TTATCAGGCCTGGCATGCGGCGAAAAGTTCCTGGAGAAGGCCTCCCCCTCCCCAAAACACCCGAGAAACG

TGGGGACCTCATTATTGAGTTTGAAGTGATCTTCCCCGAAAGGATTCCCCAGACATCAAGAACCGTACTT

GAGCAGGTTCTTCCAATATAGCTATCTGAGCTCCCCAAGGACTGACCAGGGACCTTTCCAGAGCTCAAGG

ATTTCTGGACCTTTCTACCAGTTGTGGACCATGAGAGGGTGGGAGGGCCCAGGGAGGGCTTTCGTACTGC

TGAATGTTTTCCAGAGCATATATTACAATCTTTCAAAGTCGCACACTAGACTTCAGTGGTTTTTCGAGCT

ATAGGGCATCAGGTGGTGGGAACAGCAGGAAAAGGCATTCCAGTCTGCCCCACTGGGTCTGGCAGCCCTC

CCGGGATGGGCCCACATCCACCTCCAGTCCCTGGCCAGGGGTGAGAGGCAGACCAGCAGATGGACTTGAT

CCCTCTGTGTCTTTTTGCTTCTGGCTGGTAGATAATGTCAACCTGCAGTCTTGATTCCCAGACCCTGTAC

ACTCCTCCTTTTCTGTTGTGTGATCAGTTTGTGCTTTATTCTGTATTTGTCTCCCATGTCTTGCTCTTCT

CCTGGAGAATTCTGTCTTCTCTTTGGCCATCTCAAATTGAGAACCTAAACTATTCCTGCAGAACTGCCTG

GTTGGCGTCCACAAGCAATACCTCTCGTTCCAGCAGGACCAAGGGAGCCAGCCTCCAGTGAGTGACTCCA

GCAAGTGCAGCCACCTCTCCCTTGATGGTCTGGGAGCCTGGCCTCAGCAAGGGGCCTTCCTGACCTCTGG

CTCCAGTGAAGCTGAATGTCCTCACTTTGTGGGTCACACTCTTTACATTTCTGTAAGGCAATCTTGGCAC

ACGTGGGGCTTACCAGTGGCCCAGGTAATTTTTTGTTTCATGGACTATGGACTCTTTCAAAGGGATCTGA

TCCTTTTGAATTTTGCACAGCCCTAGATACAATCCCTTTTGATAAAAGGGTCTTTGCTTCTGATTACAGG

AGCACTGTGGAACGTCTGTAAATATGTTTTTATAATTCCATGTATAGTTGGTGTACACTCAAAACCTGTC

CCCGGCAGCCAGTGCTCTCTGTATAGGGCCATAATGGAATTCTGAAGAAATCTTGGGGAGGGAAGGGGAG

TTGGAACAAATGTCTGTTCCCTGGAGGCCAGTCCAGTGCTCAGACCTTTAGACTCATTGTAAGTTGCCAC

TGCCAACATGAGACCAAAGTGTGTGACTAGTCAATGAAGTGCGACAGCATTAAAGACTGATGCTAAACCT

CA

>NM_012328.3 Homo sapiens DnaJ heat shock protein family (Hsp40) member B9 (DNAJB9), mRNA

GAGGTGGTGGCGCCAGCGGCTACCTCCTGCCTGTGAGGAGCTGGCTGAGAGGGGACTGGGCGCCGGCGGG

GAAGGAGGAGCGCTAGGTCGGTGTACGACCGAGATTAGGGTGCGTGCCAGCTCCGGGAGGCCGCGGTGAG

GGGCCGGGCCCAAGCTGCCGACCCGAGCCGATCGTCAGGGTCGCCAGCGCCTCAGCTCTGTGGAGGAGCA

GCAGTAGTCGGAGGGTGCAGGATATTAGAAATGGCTACTCCCCAGTCAATTTTCATCTTTGCAATCTGCA

TTTTAATGATAACAGAATTAATTCTGGCCTCAAAAAGCTACTATGATATCTTAGGTGTGCCAAAATCGGC

ATCAGAGCGCCAAATCAAGAAGGCCTTTCACAAGTTGGCCATGAAGTACCACCCTGACAAAAATAAGAGC

CCGGATGCTGAAGCAAAATTCAGAGAGATTGCAGAAGCATATGAAACACTCTCAGATGCTAATAGACGAA

AAGAGTATGATACACTTGGACACAGTGCTTTTACTAGTGGTAAAGGACAAAGAGGTAGTGGAAGTTCTTT

TGAGCAGTCATTTAACTTCAATTTTGATGACTTATTTAAAGACTTTGGCTTTTTTGGTCAAAACCAAAAC

ACTGGATCCAAGAAGCGTTTTGAAAATCATTTCCAGACACGCCAGGATGGTGGTTCCAGTAGACAAAGGC

ATCATTTCCAAGAATTTTCTTTTGGAGGTGGATTATTTGATGACATGTTTGAAGATATGGAGAAAATGTT

TTCTTTTAGTGGTTTTGACTCTACCAATCAGCATACAGTACAGACTGAAAATAGATTTCATGGATCTAGC

AAGCACTGCAGGACTGTCACTCAACGAAGAGGAAATATGGTTACTACATACACTGACTGTTCAGGACAGT

AGTTCTTATTCTATTCTCACTAAATCCAACTGGTTGACTCTTCCTCATTATCTTTGATGCTAAACAATTT

TCTGTGAACTATTTTGACAAGTGCATGATTTCACTTTAAACAATTTGATATAGCTATTAAATATATTTAA

GGGTTTTTTTTTTTTGACAAATTCAACATTCAACGAGTAGACAAAATGCTAATTATTTCCCTGATTAGGA

AAGTTTCTTTAAAAAACACGTAATTTTGCCTAGTGCTTTTTCTCTACCTGCCCTTGGGCTCACTAATATC

ACCAGTATTATTACCAAGAAAATATTGAGTTTACCTGATTAAACTTTAAAAGTTAATTGTAGATTTAAAT

TGTGTGAACCTAATGATTTTTGCAGTGAAACCTTTACTAATTCAAAGTTGCATGTTCTATGACATCTGTG

ACTTGCGTTGCAGAGTGTACATGAAACTGTATAATTGAGTCATTCAGTAAAGGAGAACAGTATCTTGGTT

AATTGCTACTGAAAGGTTGAGAAAGGAATGGTTTGATATTTACCACAGCGCTGTGCCTTTCTACAGTAGA

ACTGGGGTAAAGGAAATGGTTTTATTGCCCATAGTCATTTAGGCTGGAAAAAAGTTGAAAACTTAACGAA

ATATTGCCAAGAGATTGTTATGTGTTTGGTTCCAGCCTAAAAATGATTTTGTAGTGTTGAAATCATAGCT

ACTTACATAGCTTTTTCATATTTCTTTCTTAGTTGTTGGCACTCTTAGGTCTTAGTATGGATTTATGTGT

TTGTGTGTGTGTAGTTTATCCTCTCTCTCATCTTTATCTAGAGATTGACTGATACCTCATTCTGTTTGTA

AAACCAGCCAGTAATTTCTGTGCAACCTTACTATGTGCAATATTTTTAAATCCTGAGAAATGTGTGCTTT

TGTTTTCGGATAGACTTATTTCTTTAGTTCTGCACTTTTCCACATTATACTCCATATGAGTATTAATCCT

ATGGATACATATTAAAACAAGTGTCTCATACAACATTGTATGTGAGAGAAATATAAATATTTACAACCTG

ATATTCGTTGTTGTTTTATTGTTAAAAGTTTATTATGCAACTCTGGAGGTATAGAGGGCATATAAGCTAT

GGGACATATGCTGATCACAGGCTATATTCATGAAGTTACTTTTGACCAACCTGAAAACTGATAGGATTTT

GTTTGTCATTTGGTAATTTCTACTGCATTCTTACCATCCTTCTCTCACAAATTTTGATAGCTTGAAGATC

TTTTTAATTATAATTTTGTTGTATTTGTTTCCTAGGAGCAAGTGTTCCTGCTGCCAGTTCTTTCCTCTTT

AGGCGTGGTTGAGAAAAAGCAGAAACTTTACATAAAGCTGTATTTCTTAATCATCTTTAATTTGAAACTT

AAGAAAATGAATTTATTCTGTTATATTTATGTAACTTATTTCCTGGAAGTTATATCTACTAGTTTTGTTT

GATAATAATAAAATTAGCTATACCTTGAA

>NM_018370.3 Homo sapiens DNA damage regulated autophagy modulator 1 (DRAM1), mRNA

ACTCTGGCCCGGCAGCCTCGCCGCCCGCAGCCTCGCTCCGCTCCTCGCGCTTCCCCTCCCTCCGGGGCTG

GGCCTGCCCCGGCCGTCGCGGAGCCTCCCCTCCCACCGTCCGTGAGTGTACGCGCCCGGCCGCCGCCTCC

AGGCAGCCCGGAGCAACCCGGCGCCCGGCCCCGCTGGGCGCAGCACTCCGTCGGCGGCGGCGGCGGCGCG

ATGCTGTGCTTCCTGAGGGGAATGGCTTTCGTCCCCTTCCTCTTGGTGACCTGGTCGTCAGCCGCCTTCA

TTATCTCCTACGTGGTCGCCGTGCTCTCCGGGCACGTCAACCCCTTCCTCCCGTATATCAGTGATACGGG

AACAACACCTCCAGAGAGTGGTATTTTTGGATTTATGATAAACTTCTCTGCATTTCTTGGTGCAGCCACG

ATGTATACAAGATACAAAATAGTACAGAAGCAAAATCAAACCTGCTATTTCAGCACTCCTGTTTTTAACT

TGGTGTCTTTAGTGCTTGGATTGGTGGGATGTTTCGGAATGGGCATTGTCGCCAATTTTCAGGAGTTAGC

TGTGCCAGTGGTTCATGACGGGGGCGCTCTTTTGGCCTTTGTCTGTGGTGTCGTGTACACGCTCCTACAG

TCCATCATCTCTTACAAATCATGTCCCCAGTGGAACAGTCTCTCGACATGCCACATACGGATGGTCATCT

CTGCCGTTTCTTGCGCAGCTGTCATCCCCATGATTGTCTGTGCTTCACTAATTTCCATAACCAAGCTGGA

GTGGAATCCAAGAGAAAAGGATTATGTATATCACGTAGTGAGTGCGATCTGTGAATGGACAGTGGCCTTT

GGTTTTATTTTCTACTTCCTAACTTTCATCCAAGATTTCCAGAGTGTCACCCTAAGGATATCCACAGAAA

TCAATGGTGATATTTGAAGAAAGAAGAATTCAGTCTCACTCAGTGAATGTCGCAGGCCATTTCTAAAAGT

GCTACAGAGGACAGACAGGGTTTTGAGGCCACCCTGATTATTGGGATGCATCTGCAGCACATCCAGGACT

TGAATTTCATTACGAGTTCCTAATAGTTGTATTTCTAAAGATGTGTTTCCTAGAGAATGTACAGCCTTAT

GACACTGTAGTGATGTTTTTATAATTTTCTAAGTAGATTTTTTTATATTAACAAATTCATATACAGAAAA

AATAAGGTGTTACAAAAAATGGAGAGCTCTTATTTTTGTACAGATTCTGTCGTTTTTGTTTTATTTGTGT

GAGATTTATGGAAATACACTAAATGAGTAATTCAGGTTCAGTACATTTATTACAAAGTGAAATCAGGGGA

TATTCATTTGTAAATTTTATTCTTAGTGAATGAACTGTATAATTTTTTTTATCAGGAGAGCACTTATAAA

ATTCAATTTATAAAGATCATATACCCAAATCATAAAGATTTAGTTGATACATTAACACTAAGATACTCTG

ATTTTTAGCCGAACTAAACAAAGTGCTTCTACTGAGAGGCCTTTATACCACCATGTACAGTAACTCTAAG

TGAATACGGAAGACCTTGGTTTTGAAATTCTGCCACCTTGTTTCTCCCTGCTCATGAGGTCGCACCTTTT

GCTCTTGCTGCTAATTGCCCATTCGTAGTGGGTGTAATGCCAGGTGGAATGGTTTCAACAAGTCAGGTGA

AAACCATCCTTTATTGTTGCTGGCACAACTTGATATATAGTCTGACTCAGAACTGAAGCTCACATCTCAA

ATTCATTTCATGCCAGTAAATGTGGCAAAGAGAAGAAAGGCCCAAGAGCGAGACAAGAAGAATGGAGAAG

GGGGCAGCCAAGAAGAACTTCTGGGTTCAGGGTACTGTTTATTTGCTCCTTCTCTTCATGCCTGTGGCTG

GATGTCCCACAACACTATAAGAAATATAAGTCAAGCCCTTTGTGTTAAGCAAGAACTACAGACTCCATCT

TTTCACCCAAATCATGAATGACCAATAAAAAGCAAGTTATTCCAGAGGAAGAAGCAGCCCTTGAAATGTT

AAGGCTTAGGCTTGAAAGGTGAAGAGCAGGAATTCTCTCTTTCAAATCCTAGAGCATAAACCCATGTGTG

GCCAAGTGAGATCAGCCCTCAAGGGCACATGCCAAGGGCAGAGCAGCCCATGTAGACAGCTTCGGAGGGC

ATGGGGGTGTAGGGAGTTCGGGGTAGCTCCTCATTAACTATTTGTTGGGTGAGTAAAGGGGTGAGGCTCA

GTGGCAGGTACCTCTGCAATGACAAGCTGCCTCCCCTCTATGTGTTTAGCATATGTTATTAGAACATGTC

CGACACCCCTACCGCTGCCATTTGGGCCCTTTAATAAAGCCAAGTAGAGAAATCTGGCAATAAAAGGCAA

ATGTAAGCATGCTTTCTTTAAGACGCATCATAAATGGTTTTCTTTAAGTGAATGGAAGAGTTTGACAGAG

ATACACCTTTGTAAGAAAACATTAAGAATGCTGGCTGGCTGTGGTGGCTCACACCTGTATTCCCAGCACT

TTGGGAGGCCTAGGCAGGAGGATTGCTTGAGCCTGGGACTTCGAGACCAGACTGGGAAACATGGCAAAAT

CCCATCTCTACAACAAAAATACAAAAATTAGCCAAGTGCGGTGGTGTGCCTGTAGTCCTAGTTACTTGGG

AGGCTGAGGTGGGAGAATCACCTGAGCCCAGGAGGTGGAGGCTGCAGTGAGCCATGCCAATGCACTCCAG

TCTGGGCAACAGAGTGAGACCCTGTCTCAAAAATAAATAAATAAATAAATGAATAAAGAGAATGCTAATC

ATTTCTGGGTTCACTGCGACTCACTGTAGTGCTGGGGATCCCCCTTGTAACACTGGAACTGAAAGACAGT

GATGAAAGCTATGTCAAGCATTCATTATTCTGAAGAGGAGGAGAAATGCCACATACCTTTCCCATGGGAC

CTGTGGTGGAATGAATCCATACTTCTGCCTCACTTCGAGCAGACTTTTGTTCTCGGCGCTCCTCACGATG

GAGTTTCATGCTTCATTTTCACATCTCTCTGCACAATTAGATTGGGAGCTCCTTGAGGGCAGAGTACGTG

CCTTAATCTTTATCTTTGTAATGCCACAATGAACAGAGTGCCTCCTGGTACACTGTAGGAGCTTAAGAAA

TACTCACTGAATGCATGAATGAATGAATGAACAAATGAAGGAATGACTAAGGATGTTTGTAGTGCTATAA

TATAGAATGGGATTTACTCTGCTTTACCAGTTAGTTTCATAATAAACAAATAGTCTGTA

>NM_014674.3 Homo sapiens ER degradation enhancing alpha-mannosidase like protein 1 (EDEM1), mRNA

AAAGGGGAAGCGAGCCGGGCTACGGGGCGAGCGCGGGGTGCGGTGGTCGGCGGGGAGGCCCCCGCGCTTT

AAAATAATGCCCGCGGCGCCCGCGCGACCATGCAATGGCGAGCGCTCGTCCTGGGGCTGGTGCTCCTCCG

GCTTGGCCTCCATGGAGTATTGTGGCTCGTCTTCGGGCTGGGGCCCAGCATGGGCTTCTACCAGCGCTTT

CCGCTCAGCTTCGGCTTCCAGCGTCTGAGGAGCCCCGACGGCCCCGCGTCGCCCACCTCGGGGCCCGTGG

GCCGGCCTGGGGGGGTATCCGGGCCGTCGTGGCTGCAGCCGCCGGGGACCGGGGCAGCGCAGAGCCCGCG

CAAGGCTCCGCGGCGTCCTGGGCCGGGGATGTGCGGCCCAGCCAACTGGGGCTACGTGCTGGGCGGCCGG

GGCCGCGGCCCGGACGAGTACGAGAAGCGCTACAGCGGCGCCTTCCCTCCGCAGCTGCGTGCCCAGATGC

GCGACCTGGCACGGGGCATGTTCGTCTTTGGCTACGACAACTACATGGCTCACGCCTTCCCCCAGGACGA

GCTCAACCCCATCCACTGCCGCGGCCGTGGGCCCGACCGCGGGGACCCTTCAAATCTGAACATCAATGAT

GTACTAGGGAACTACTCATTGACTCTTGTTGATGCATTGGATACACTTGCAATAATGGGAAATTCATCCG

AGTTCCAGAAAGCCGTCAAGTTAGTGATCAACACAGTTTCATTTGACAAAGATTCCACCGTCCAAGTCTT

TGAGGCCACGATAAGGGTCCTGGGAAGCCTCCTTTCTGCTCACAGAATAATAACTGACTCCAAGCAGCCC

TTTGGTGACATGACAATTAAGGACTATGATAATGAGTTGTTATACATGGCCCATGACCTGGCGGTGCGGC

TCCTCCCTGCTTTTGAAAACACCAAGACAGGGATTCCATATCCTCGGGTGAATCTAAAGACAGGAGTTCC

TCCTGACACCAATAATGAGACATGCACAGCGGGAGCCGGTTCCCTCCTGGTGGAATTTGGGATTCTGAGT

CGACTCCTGGGGGACTCCACATTTGAGTGGGTGGCCAGACGAGCAGTGAAAGCCCTTTGGAACCTCCGGA

GCAATGATACAGGATTACTAGGCAATGTCGTGAACATTCAGACGGGCCACTGGGTTGGAAAGCAGAGTGG

CCTGGGTGCCGGGCTGGACTCCTTCTATGAATACCTCTTGAAATCTTACATTCTCTTTGGAGAAAAAGAA

GACCTAGAAATGTTTAATGCTGCATATCAGAGTATTCAGAACTACTTAAGAAGAGGGCGGGAAGCCTGCA

ATGAAGGAGAAGGAGACCCTCCACTCTATGTCAACGTGAACATGTTCAGTGGGCAGCTGATGAACACCTG

GATTGACTCTCTGCAGGCCTTTTTCCCTGGACTGCAGGTGCTGATAGGAGATGTGGAAGATGCCATCTGC

CTTCATGCCTTCTACTATGCCATATGGAAACGATATGGTGCCCTCCCTGAGAGATATAACTGGCAGCTGC

AGGCCCCTGACGTTCTCTTCTACCCACTGAGACCAGAGTTAGTGGAATCCACATATCTCCTCTACCAGGC

AACCAAGAATCCCTTCTACCTCCATGTAGGAATGGATATTCTGCAGAGTCTGGAAAAGTACACAAAAGTC

AAGTGTGGGTACGCCACGCTGCATCACGTCATTGACAAGTCCACAGAAGACCGGATGGAGAGCTTCTTTC

TCAGTGAGACCTGTAAATATTTGTATCTGCTGTTTGATGAAGACAATCCAGTACACAAGTCTGGAACCAG

ATACATGTTCACAACAGAGGGACACATTGTATCTGTGGATGAGCATCTTCGGGAATTGCCATGGAAGGAA

TTCTTCTCTGAAGAGGGAGGGCAGGACCAAGGGGGAAAGTCTGTGCACAGGCCGAAACCTCATGAGTTAA

AAGTCATCAACTCCAGCTCCAACTGCAATCGTGTACCTGATGAGAGGAGGTACTCCCTGCCCTTAAAGAG

CATCTACATGCGACAGATTGACCAGATGGTTGGTTTGATTTGATCTGCTCTCTGTGAGGCCTCATCTTGA

ACCAGACCTTAACGACCAAACCCAGACCATGCCAAAGTCCAGTCTGAAATGAAAGGGGACAGAAGTCTTG

CTGTCCATGGTGGTGTAGGAATTTCTGTGCAACACCTCACCACGTCTGGTTAATCCTTGCACACTTCAGT

GTTTCTCTCCTGTTCAATAAAATGCCCTGTTAAGGATATAATTTGAAGTGAGAAGATACATGGAAATTGC

CCTCTTATGACATGTTGATGTTATAAGCACAATAGATGGGGCATCTTTGGATTGATGTTCACAGCTTTAT

ACTTCAGAACCTAAGTCTCTTCACTTTGCTGGCACCTGCTATACTGGAGTATTGCTATGTCTTTAAAAAA

TTTTTTTTATTATATTTTATTTTTTTGAGACAGGGTCTTGATATTTTTTTGGGACAGGGTTACCTGGGCT

CAAGTGATCCTTCTGCCTCAGCCTCCCGAGTAGCTGGGATTACAGGTGAGCACCACTGTACCTGGCTAGC

TACTTCTTTGTTAGAGGATTGAGAATGAAATTTCTGCAAAAGGGCCCATGGTTCATTTGGTATCCCTATT

TAATTGCATTGAAAATGTCATCCTTTCTGTTGTTAGATAATTGGGGTCTTCCCCTGATATCCAACCGTGA

TTTTGGATCACATGGGAGAAAAAGTCATCCAGTTTTTCATGTTTGCCTCAAGTAATCTTTACAGTGTTAC

AAATTATTTGCTTAAGAAGAATGGTCTTAACCAGAATTCTTAACAGATAGTCTCTTAGGTTATTATGTTA

TGGTCTAAGAGGTTAACTGACATCTTTTGGGTGGTATTTTGCATTTTGAATATGAACTTACCTGAGGAAC

TCCCATAGTTCCAGAATCAGGTGCCTTTTAGGGAGAGAACAATACCTAAGATTGTCTGAGCTTCCATCTT

TCTCATATTTCCTAAGCAAGGATTCTCACTTCTGACCATATTTGGGTTAGAGTTCTGTTTTGTTTCTGTT

TTCTGTGTCTAGTGCCAATTAGCTAAATCAGGGAGAAAGAAATGATCACATGACTTTTAGCATCCTTGAG

CCATTTCTCTGTGTAATACAGGCTTTAGATTAGTGCCTTATATTGGTTTTGGTTTGGGGCACTGGATGTC

GCAGCTACTGCTATGGTTTCAGGAGGCCTGTTTAGCCACATGGTGAGACCGTGGTGAAAGGGGGATGGAA

ATTGCTTGGCCAGTCTTTGCCTTTCATCCTGTAAAAGTAAGCATGTAGAAGGAGGAAGTTGTGCTAAAAT

GCCTTTGTTTTTTTGTTATTATTTTCTTAGCCAGAACATCTCTCTTTGAACTCACACTGATACACACCTG

CTACTCTTACACAGTGCAGCAGGGTTGACTCTTAGTCTGGCTTCCATGAAGCGTCATGGGTGGAAACGCA

TTCTAGTAAAAAAGGTAGGAAATCCCTAAAACTTCCAGCCTCACATAGCACGGTTCTCACCTGTCACTGT

TTTCCCACCTCTAAGGATTTCATGTACATCTTTTCAAAGCTAGAAATAAGCACTGTCTAAGTTTATGTTG

CATTTTTAGTCAAAAGGGAGAAATCTTATTCCTTCTTGAAAATTTTAAGTGTTATGGTTTTATATAGTTC

AGTTCTTTGAGATTTTTGAAAAGAGTATTTTCAGTAATAAACGTGCCATCTCTATCTCTTAAACATTTAT

TACAACAATTGTTTTAAAATAGAAAAAATAAAATGCTTCTATTTTACCTTTTTTCATTTCAGAAGCATTA

TTCTGTTTATTAACAGTGTCCCATCTACTGAATAGAAAACTTTGAGAATAATATATATATATATTTTAAA

TGTTTTCACTGACTCATTGAAAATGTTAATTACACACACATGCATGCATGCACACACGAGCATACTTGTA

CCTTTGTCTCTGGGCAAACAGGTGGGACTGTTAGTGACCCATTTGGGAAAATAGAGCATCTCAGAGAAGG

AGGTGAGTTCTTCCTGCCTGTGATTTCTCTTGGCGCTCCCCTCCTCTCCCGCTCTGGCTTCTGTGGCGGC

AGTGGTGGGTAAGCACTCCAGTGTTCTCTTAATGAGGCACTTTGCCTGTCACTCGAGCAAGCCTGGGTGT

TCCTTCCTCCTCATGCTCCTGGAATAGGGAATAGGGATCTCATGCTTGCAAACTACACAATGCTGCAGGT

GCTTCCCAGGGGCCACAGGCTGTCAGGAAACGTGTTTTATGTTAAGTCACAAACCCACTTGACTTCTGGG

TACTGGAATTAATACCAGTGGGTGAGACTGAGGGTGAGTGAGTTAGTACATATTAATCCTGGTTGTTGAG

CTTCCAGACTACCCCGTCCAAAGTTTGATGCTATGTAGTCAGTGGTTTGTGGGGCTGGATGCCAGAAGGT

TCTTTGAGCCAGTTTCAAAGGTTACTTGTTTTTTTTTTTTTTTTTTAAAGTCAGAATGTTAACAGCTGTG

ATATATCCTGCAGGGCTTTTGCAGTTTCTTCTGTTCTGTGTTCTGAAATACTGGGTAGAGAATGGCTGAG

GAGGAGATTACCAGAGAAGTTGCTTTGCTCAGTGCTTTGCCCCAGGATTGCCTCAAATCTGAGTGGACTT

CATCCTTTGCGGCGGCTCTGAGCCTGGCCCATCTTCCTATTCCCACGTGTAGCTAGTGTCTAGTGTCAGC

TTTGCTCAGTGTGGTGGAAACATTTTGCAGAACTGTTGTAGAAAGCTGCCTTATAGTTGGCTTGACAAAG

CATAATTCTCTCATAACAAACTTTCAAATCATTACAGTAGCTTAGCTACTTTAGTTGATGTGACCGAGGA

ATCCCTTCTAGAATCATAGGTGGCAAGGGAGGGTTTGCTAGCTCTCCATTTGCACTGGCCATTGTGAAAA

ACCAGCTTCTGTATTCAAATCTTTCCTTCATTTTTTTAAATTTTTTTTTTGGCAGCGCTTGTGCTGGAAC

TTACTCATTGTAACTGAATCCTCAGGGCTTTTCTTGTTTTAGATCATGGACTGTGCACGTGACACTTAAA

TAATTTTCTATGTATTTAAAGAAAAATGCACCAGGATGGTGTCTGTGCACGTGACTATTAGAGGAGCGTC

TGTAGAAGTACCTGGTTTGGTCAGTGCAGTTGTGCAATCTGAGGGCCTTGTTTCCTCCTCCCCTTTCCCC

TTCTCCCCACCAAAGGAAAATATCCCTCTTAATGATTTCGTAGTTCAGTTTACTGAATGATTACCACCTG

TAATTCCTCTTTGGATTGTGTAGACTCAACATGAGACATTCCTTTCTGCTTTCTGGAGGGCACCAGGGGC

CTTTCTCTTTGATAAATTTTTTTTGTCTGTTGACAAAAACAAAAATCTTTTTTCAAATGTAGTGCTGGTG

AAAAGGTAGGGCTGAGTGATTACCTTAGCCACAGGGTGGCTGAGCAGGAACTTTAGAAGAAAATCCTGAG

CTTTCCTGTCCATTCCCAGCATCCAGCTCCTATTCTAGTGCCTCTTCCCTGCAGGGCAGGGACCCCTTGG

GAAATCGAGGAGGTGGGACGGGCTGGGCCCTGTGTCCCAGGTTTCACAGGGCTCAGGGTTATGCTCCCGC

TTGAATCTGGACGTGAATCTGGTAAAAATATCAAGTACCTGTGGAACTCCCTGATTCTATACCCTCTTCC

TTCTTTCTGCAAGGCAGAGGAATAATATTTTTAAAGGTTATTTTGTTTTAGTTTTAAATAGCAAAACACA

AGCTGCATTTTTATTTATTTTGCATAAGAAAGGTAAATCTTTTTACAAAAAAAAGTATAGAGTTGGAAAC

TCTGGGAAAACTTACGGAAATACACAAATGCTTCTCTGTAATGTGCAATATGCTTTGCAACTGTAGATGA

TATTTTATGTTTAATCTGTAAATAAGAAATGTATTTAAATTAAAAGGGATCTTTTTGTAAAAGGACCAAA

TGTTCTTTTATAAATGTAATAAGGAATATCTTGCTCTTTAAAATTTATTAGGATTTTTATGAGTAATTTT

TATTAAAAGATTTCTTTTTTTGA

>NM_001961.4 Homo sapiens eukaryotic translation elongation factor 2 (EEF2), mRNA

CTCTTCCGCCGTCGTCGCCGCCATCCTCGGCGCGACTCGCTTCTTTCGGTTCTACCTGGGAGAATCCACC

GCCATCCGCCACCATGGTGAACTTCACGGTAGACCAGATCCGCGCCATCATGGACAAGAAGGCCAACATC

CGCAACATGTCTGTCATCGCCCACGTGGACCATGGCAAGTCCACGCTGACAGACTCCCTGGTGTGCAAGG

CGGGCATCATCGCCTCGGCCCGGGCCGGGGAGACACGCTTCACTGATACCCGGAAGGACGAGCAGGAGCG

TTGCATCACCATCAAGTCAACTGCCATCTCCCTCTTCTACGAGCTCTCGGAGAATGACTTGAACTTCATC

AAGCAGAGCAAGGACGGTGCCGGCTTCCTCATCAACCTCATTGACTCCCCCGGGCATGTCGACTTCTCCT

CGGAGGTGACTGCTGCCCTCCGAGTCACCGATGGCGCATTGGTGGTGGTGGACTGCGTGTCAGGCGTGTG

CGTGCAGACGGAGACAGTGCTGCGGCAGGCCATTGCCGAGCGCATCAAGCCTGTGCTGATGATGAACAAG

ATGGACCGCGCCCTGCTGGAGCTGCAGCTGGAGCCCGAGGAGCTCTACCAGACTTTCCAGCGCATCGTGG

AGAACGTGAACGTCATCATCTCCACCTACGGCGAGGGCGAGAGCGGCCCCATGGGCAACATCATGATCGA

TCCTGTCCTCGGTACCGTGGGCTTTGGGTCTGGCCTCCACGGGTGGGCCTTCACCCTGAAGCAGTTTGCC

GAGATGTATGTGGCCAAGTTCGCCGCCAAGGGGGAGGGCCAGTTGGGGCCTGCCGAGCGGGCCAAGAAAG

TAGAGGACATGATGAAGAAGCTGTGGGGTGACAGGTACTTTGACCCAGCCAACGGCAAGTTCAGCAAGTC

AGCCACCAGCCCCGAAGGGAAGAAGCTGCCACGCACCTTCTGCCAGCTGATCCTGGACCCCATCTTCAAG

GTGTTTGATGCGATCATGAATTTCAAGAAAGAGGAGACAGCAAAACTGATAGAGAAACTGGACATCAAAC

TGGACAGCGAGGACAAGGACAAAGAAGGCAAACCCCTGCTGAAGGCTGTGATGCGCCGCTGGCTGCCTGC

CGGAGACGCCTTGTTGCAGATGATCACCATCCACCTGCCCTCCCCTGTGACGGCCCAGAAGTACCGCTGC

GAGCTCCTGTACGAGGGGCCCCCGGACGACGAGGCTGCCATGGGCATTAAAAGCTGTGACCCCAAAGGCC

CTCTTATGATGTATATTTCCAAAATGGTGCCAACCTCCGACAAAGGTCGGTTCTACGCCTTTGGACGAGT

CTTCTCGGGGCTGGTCTCCACTGGCCTGAAGGTCAGGATCATGGGGCCCAACTATACCCCTGGGAAGAAG

GAGGACCTCTACCTGAAGCCAATCCAGAGAACAATCTTGATGATGGGCCGCTACGTGGAGCCCATCGAGG

ATGTGCCTTGTGGGAACATTGTGGGCCTCGTGGGCGTGGACCAGTTCCTGGTGAAGACGGGCACCATCAC

CACCTTCGAGCACGCGCACAACATGCGGGTGATGAAGTTCAGCGTCAGCCCTGTTGTCAGAGTGGCCGTG

GAGGCCAAGAACCCGGCTGACCTGCCCAAGCTGGTGGAGGGGCTGAAGCGGCTGGCCAAGTCCGACCCCA

TGGTGCAGTGCATCATCGAGGAGTCGGGAGAGCATATCATCGCGGGCGCCGGCGAGCTGCACCTGGAGAT

CTGCCTGAAGGACCTGGAGGAGGACCACGCCTGCATCCCCATCAAGAAATCTGACCCGGTCGTCTCGTAC

CGCGAGACGGTCAGTGAAGAGTCGAACGTGCTCTGCCTCTCCAAGTCCCCCAACAAGCACAACCGGCTGT

ACATGAAGGCGCGGCCCTTCCCCGACGGCCTGGCCGAGGACATCGATAAAGGCGAGGTGTCCGCCCGTCA

GGAGCTCAAGCAGCGGGCGCGCTACCTGGCCGAGAAGTACGAGTGGGACGTGGCTGAGGCCCGCAAGATC

TGGTGCTTTGGGCCCGACGGCACCGGCCCCAACATCCTCACCGACATCACCAAGGGTGTGCAGTACCTCA

ACGAGATCAAGGACAGTGTGGTGGCCGGCTTCCAGTGGGCCACCAAGGAGGGCGCACTGTGTGAGGAGAA

CATGCGGGGTGTGCGCTTCGACGTCCACGACGTCACCCTGCACGCCGACGCCATCCACCGCGGAGGGGGC

CAGATCATCCCCACAGCACGGCGCTGCCTCTATGCCAGTGTGCTGACCGCCCAGCCACGCCTCATGGAGC

CCATCTACCTTGTGGAGATCCAGTGTCCAGAGCAGGTGGTCGGTGGCATCTACGGGGTTTTGAACAGGAA

GCGGGGCCACGTGTTCGAGGAGTCCCAGGTGGCCGGCACCCCCATGTTTGTGGTCAAGGCCTATCTGCCC

GTCAACGAGTCCTTTGGCTTCACCGCTGACCTGAGGTCCAACACGGGCGGCCAGGCGTTCCCCCAGTGTG

TGTTTGACCACTGGCAGATCCTGCCCGGAGACCCCTTCGACAACAGCAGCCGCCCCAGCCAGGTGGTGGC

GGAGACCCGCAAGCGCAAGGGCCTGAAAGAAGGCATCCCTGCCCTGGACAACTTCCTGGACAAATTGTAG

GCGGCCCTTCCTGCAGCGCCTGCCGCCCCGGGGACTCGCAGCACCCACAGCACCACGTCCTCGAATTCTC

AGACGACACCTGGAGACTGTCCCGACACAGCGACGCTCCCCTGAGAGGTTTCTGGGGCCCGCTGCGTGCC

ATCACTCAACCATAACACTTGATGCCGTTTCTTTCAATATTTATTTCCAGAGTCCGGAGGCAGCAGACAC

GCCCTCTTAGTAGGGACTTAATGGGCCGGTCGGGGAGGGGGAGGCGGGATGGGACACCCAACACTTTTTC

CATTTCTTCAGAGGGAAACTCAGATGTCCAAACTAATTTTAACAAACGCATTAAGAGGTTTATTTGGGTA

CATGGCCCGCAGTGGCTTTTGCCCCAGAAAGGGGAAAGGAACACGCGGGTAGATGATTTCTAGCAGGCAG

GAAGTCCTGTGCGGTGTCACCATGAGCACCTCCAGCTGTACTAGTGCCATTGGAATAATAAATTTGATAA

GGTGGTGA

>NM_013302.5 Homo sapiens eukaryotic elongation factor 2 kinase (EEF2K), mRNA

GCTCAACCTAGACCAGCCCCAGCTTCAGCCTCAGCTCCCCTCCTTCCTGGATCGAGCGCCCGCACTCCCG

GCCCTGCAGCCACCCGAGTCCCGCTCGCTGTCGCCTGCACGCGAGTCCCCCCTGGCACGCGCTCCCACAT

CCCGGGATCGTCCCAACGGCCCCTGCGCCCTTCCTGGGATCACTCCGACTGCCCCGCGCGCCCTGGGATC

GGTCCATCTACCCCGCGTGGCCCCAGCTGCTTGCCCGGAGCGCCAGCTAGCGCTCCCCGCTCTCCGCTCC

CCGGCACTCTCGGGGGGCCCGCCCGCCCTGCACCCTGGAGCTCCGGGCCGCGAGCCTCTGCCAACTCCTC

TGGACCCTCGCGGCCGTGGGCAGCGGCTGCCGCGCCTGTCTGCCCGAGGGAGGACCTTCGCCTCTGCATT

TGTCCAGTAACTCTGGCTGTGCCGGATACTGCTTGGGTAAAACGGGCACCCCAGGAACATGGCAGACGAA

GATCTCATCTTCCGCCTGGAAGGCGTTGATGGCGGCCAGTCCCCCCGAGCTGGCCATGATGGTGATTCTG

ATGGGGACAGCGACGATGAGGAAGGTTACTTCATCTGCCCCATCACGGATGACCCAAGCTCGAACCAGAA

TGTCAATTCCAAGGTTAATAAGTACTACAGCAACCTAACAAAAAGTGAGCGGTATAGCTCCAGCGGGTCC

CCGGCAAACTCCTTCCACTTCAAGGAAGCCTGGAAGCACGCAATCCAGAAGGCCAAGCACATGCCCGACC

CCTGGGCTGAGTTCCACCTGGAAGATATTGCCACCGAACGTGCTACTCGACACAGGTACAACGCCGTCAC

CGGGGAATGGCTGGATGATGAAGTTCTGATCAAGATGGCATCTCAGCCCTTCGGCCGAGGAGCAATGAGG

GAGTGCTTCCGGACGAAGAAGCTCTCCAACTTCTTGCATGCCCAGCAGTGGAAGGGCGCCTCCAACTACG

TGGCGAAGCGCTACATCGAGCCCGTAGACCGGGATGTGTACTTTGAGGACGTGCGTCTACAGATGGAGGC

CAAGCTCTGGGGGGAGGAGTATAATCGGCACAAGCCCCCCAAGCAGGTGGACATCATGCAGATGTGCATC

ATCGAGCTGAAGGACAGACCGGGCAAGCCCCTCTTCCACCTGGAGCACTACATCGAGGGCAAGTACATCA

AGTACAACTCCAACTCTGGCTTTGTCCGCGATGACAACATCCGCCTGACGCCGCAGGCCTTCAGCCACTT

CACTTTTGAGCGTTCCGGCCATCAGCTGATAGTGGTGGACATCCAGGGAGTTGGGGATCTCTACACTGAC

CCACAGATCCACACGGAGACGGGCACTGACTTTGGAGACGGCAACCTAGGTGTCCGCGGGATGGCGCTCT

TCTTCTACTCTCATGCCTGCAACCGGATTTGCGAGAGCATGGGCCTTGCTCCCTTTGACCTCTCGCCCCG

GGAGAGGGATGCAGTGAATCAGAACACCAAGCTGCTGCAATCAGCCAAGACCATCTTGAGAGGAACAGAG

GAAAAATGTGGGAGCCCCCAAGTAAGGACCCTCTCTGGGAGCCGGCCACCCCTGCTCCGTCCCCTTTCAG

AGAACTCTGGAGACGAGAACATGAGCGACGTGACCTTCGACTCTCTCCCTTCTTCCCCATCTTCGGCCAC

ACCACACAGCCAGAAGCTAGACCACCTCCATTGGCCAGTGTTCAGTGACCTCGATAACATGGCATCCAGA

GACCATGATCATCTAGACAACCACCGGGAGTCTGAGAATAGTGGGGACAGCGGATACCCCAGTGAGAAGC

GGGGTGAGCTGGATGACCCTGAGCCCCGAGAACATGGCCACTCATACAGTAATCGGAAGTACGAGTCTGA

CGAAGACAGCCTGGGCAGCTCTGGACGGGTATGTGTAGAGAAGTGGAATCTCCTCAACTCCTCCCGCCTC

CACCTGCCGAGGGCTTCGGCCGTGGCCCTGGAAGTGCAAAGGCTTAATGCTCTGGACCTCGAAAAGAAAA

TCGGGAAGTCCATTTTGGGGAAGGTCCATCTGGCCATGGTGCGCTACCACGAGGGTGGGCGCTTCTGCGA

GAAGGGCGAGGAGTGGGACCAGGAGTCGGCTGTCTTCCACCTGGAGCACGCAGCCAACCTGGGCGAGCTG

GAGGCCATCGTGGGCCTGGGACTCATGTACTCGCAGTTGCCTCATCACATCCTAGCCGATGTCTCTCTGA

AGGAGACAGAAGAGAACAAAACCAAAGGATTTGATTACTTACTAAAGGCCGCTGAAGCTGGCGACAGGCA

GTCCATGATCCTAGTGGCGCGAGCTTTTGACTCTGGCCAGAACCTCAGCCCGGACAGGTGCCAAGACTGG

CTAGAGGCCCTGCACTGGTACAACACTGCCCTGGAGATGACGGACTGTGATGAGGGCGGTGAGTACGACG

GAATGCAGGACGAGCCCCGGTACATGATGCTGGCCAGGGAGGCCGAGATGCTGTTCACAGGAGGCTACGG

GCTGGAGAAGGACCCGCAGAGATCAGGGGACTTGTATACCCAGGCAGCAGAGGCAGCGATGGAAGCCATG

AAGGGCCGACTGGCCAACCAGTACTACCAAAAGGCTGAAGAGGCCTGGGCCCAGATGGAGGAGTAACCAG

GAAAATCACTGCCGGCTAGTCCCAAGCAAACGGGCTAGGAGGAAAGATTAAAAAAACAACAACAACAACT

TATTTAGTTTGGGGAGGGGAAGCATTTTTAAGTGTGTTGTAAAATCAAATTTTATATTTCATTTTTTGAC

TCTTGAAAAATGTCTTTGCTCCTTGGCAGCTACCAGCAGAGACTCTATAGCTGTCTCTTAGGGCAGTATT

TTGGGGAAGTGGGGCTTGAAGAAGCAGCCTAATGAACCAACATACCGTTTTGTGTGTGGTTTTTTTTGTT

TGTTTGTTTGTTTGTTTTGAGACAGAGTCTTGCTCTGTCACCCAGGCTGGAGTGCAGTGACATGATCTTA

GCTCACTGCAACCTCCGCCTCCTGGGTTCAAGTGATTCTCCTGCCTCAGCCTCCCAAGTAGCTGGGATTA

CTGGTGCACACCACCACACTCAGCTAATTTTTGCATTTTTAGTAGAGATGGGGTTTCACCATGTTGGCCA

GGCTGGTCTCGAACTCCTAACCTCAGGTGATCCACCTGCCTCAGCCTCCCAAAGTGCTGGGATTACAGGT

GTGAGCCACCATGCCTGCCCATTTTGTGGTTCTATTTTCATTTTTATTTCTTTTTTTTTTTTTTGTCACGA

GATATAAGAAAGTGCTTTTTGCCTTGAATGGACAATTTTAGGGCTGTGCTCACTAGTCTTTTCAGGCTGG

ACTGAAATGTCGGGCCCATGGAGCCCTGTGTTTTGTGCATCGGGATGAGAAATGAAGCACTTCACGCTGG

CTTTCCTAAGTCACGGGGGCGTGTATTGCCGTGGCTTAGTGCAAAGCATTCTTTCTCAGAGCATTTAGAGG

CATGCGTGGCATTTTTTCAGTGGGTGTGAGATTGCACAATACCCAGGCTCCCTTCTACTGTGGGGAAGGG

CCTGCATGTTGGCTGTTTTTTAAACTTCTAGTTCAATTTCCTTCCATAATGCTACTGATTTTCTGGCATA

CAGCCGAATTCCATCTTTTAAGCATGCTTTCTACGGTGGGCTTTTCAAAACAGGTTTGAGTTTTGTATGC

ACACGTTTACTACCTCTAACTCCTACATCAGCTAGTGTGGAAGAGGGTGCACCTCAAAGCTTTTACACGT

AAGGACAGCGGCTTGGAATGTGAGAGCCTTTTCTCCAAGCAGACCCACACTCTGCATCTCAGTGGCAGCT

CCCACAACGTGACTGCAATGTCTCTTATACAGTATTCCTTGGTGTTTTCTTAGTGTCTGGATGTTCTTAC

GTGAAATCTGCTCCCCAGCCCTGGTCCTTGGCATTTTCTGCTTGAAGCTGGGCTGATTTTCTTGTAATTT

ACAGCAGGACGCTTTCAGCAGCAGTCTCTTGGGATTTTATCTAAGATGTTTGAGGATGAGAGGGCAAGAA

CTATAAACACTCATTAATTCTAGTAGTCTCCCCATGGCCAGACAATGGCGATTGTTATTTAATGAGCTTT

TCCTTTCAATGGAATTCAGCTCTCACATTAGTATGATTTCATTTGATGTTTCAAATAGCAAAGATGCTAG

GTGCGGTGGCTCCCGCCTGTAATCTCAGCACTTTGAGGAGGGCCAAGGTGGGAGGATTGCTTGAGCTCAG

GACTTCAAGACCAGCCTGGGTAAACATGGCGAGACCCTGTCTCTACCAAAACAACAAAAAAAAGACAGAC

TGCTTTGATCAACCCTAAATGCAAAAGCAGCCTATTTTTCTTTGTTTAAAAGTCAAAACATAAAAAAGCA

GAGTATAACATACAAACCATTCTTAACTATTCATTAAAATGGGTCCTTCAACACCTTAGTGGGGTTTGTT

GTTGTTGCTTATGCAGAGAGATTATTTTCTTTTTATTATTTTATAATTTTTGAAATAGAGATGGGGTCTC

ACTGTGTTGCCCAGGCTGGTCTCGAACTCCTGGACTTAAGTGAGCCTCCCGCCTCAGTCTCCCAAAGCGC

TGGGATTACAGGCAGGAGCCACTGAGCCCAGCCAAGACTTCAGTGTTGACTGCTTTGGAGGCACAAACCC

ATGCAAGCGTTAGTTCCAAAGTTCAGTGTGTACCCTTAAATGAACAATGAAGCAGGTAAAATTACCCTTG

AAAAAAATCCCTTGGACCACCCATAAATGACAGTGACTTTTTCAATATGGACTCATCATAGCCAGTTTTC

CTTTTGAAGTTGGAACTGATCACCCTTTTGTCATCTGTACCAGATCAGTAGTTGGCTTGTGTTACATTTT

GTGTGTGTGTGTGCGTGTTTTAAACCAGTGCATATAAATTGTATGTTAAATGTAAGTAACTTTAAGTTGA

CTTATCTCTTCACAGTAATCAAGCCTCACGTAATTCATGCTTTTTAAATTCAGCCAGCCCCCCCTCTCTG

AAATTTTATTATGTAAATAATTTGTGTTCCCTGATCACTCGTTTAAGTTCTTAGTTGTATGTCATCTCTT

CTCTAGCAGGAATTGGCAAACTTTTTTGTAAAGGGGTAGAAAGTGAAGATTTTAGGCTTTGCAGGCCATA

TAGCCTCTGCTGCAAATGCTCAGCCCTGCTGTTGTAATGTAAAAGCTGCCACAGACACTACATGAACACG

AATGAGTGTGGCTGGTGTTCCAATAAAACTTTATTTACACAAACGGGTGGCCCTTTTGGGCTGTAGTTTG

TCAACCCTTGCTCTAACCCTTGACTGAGAGCTACTTTATTAAGCCCTGAGGGCAGGAGCTATCCCAATTT

TGTGTTCCCCAGGGCACCAAAACACAGTGCTTTGGCATAGAGTAGGCACTCAACAAGTGTGTGAACAGAT

GGAGAGCCAGCCCTAGTCAGTGCACTCACCCTTTGAGGCTCTGGTTCCTCCAAACAATGATTCGTTGTCT

GGATTGGCTGGAACTGTCACCCCCGCAATTCTACTCCCCACCCACCCATGTGACCTTAATGTCAGTTGCT

GGTCTGTTGCTCTTCGGGGAGGGAGAGATGGCCTGGATACAGAGCTAAGCAAATGCTTCTTTAAGGGCCC

TTAAAAGTGAAAAGTAACTTGCAAGAGGTTGAGATCCTTCTGAGCTAGGAGAACTTATTCCACCTTCAAA

ACCTAGTTTGGGCTGGGTGCAGTGGCTCACGCCACTTTGGGAGGCTGAGGTGGGCGGATCACCTGAAGTC

AGCTACTCGGGAGGCTGAGGCAGGAGAATCGCTTGAACCCAGGAGGCAGAGGTTGCAGTGAGCCGAGATT

GCGTCACTGCACTCCAGCCTGGGCGACAGAGTGAGACTCCGTCTCGAAACCAAAAACAAGAAAAACCCCT

AGTTTTGTGCCTTCTGAAGATAGTTAGGACATCTTCTTTTTCCGCAAATGCTGGACATGCCAAAAACCTA

ACGCAAAACCCAGGACATTCCAGCCCAACTGGGACGATATCAGAGACCATCTTCAAGTGCAAGAAGCAGG

TGAAAGCCCAGGAAATTGGCAGAGCCAGGATTATCATCCAGTACTTTCATTTCACAAATGGAGAAACCGA

GGTTCTGCCAGCTAGATTTTTTTTCACAGTGTCACTGCTAGCAAGCCACTAAGCTGGAGCTGGGATTTGA

GAGCTGCTGCTATTTAGAGGATCTTGGGAATAAAATTTAAACTGGAGTTTAATGGCCCTTTCAGTTTTGC

TATAGGCAAGAGAATAAAATGAATGAATGGATAGGTGGCTTTATGGGTGTAAGAAAGAAGCGAAAAAAAC

TCCCAAACCCCAGTGTTCCTGAATATCTGTTCTCCCCCTGACCACTCTGGGAATTTATCAAATGCAGCTT

TGACCTCCAAGCCAAGTAAACTGCTCTTGTTGCTATTTTAGTGGTTTTTGTTTTTTTAAGACACAAGGTC

TCACTTTGTTACTCAGGCTGGAGTGCAGTGGCATGATCATAACTCATTGTAGGCTCAACCTCCTGGGCCC

CAGTGATCCTCCTGCCTCAGCCTCTCAAGTAGCTAAAACTACAGATGTGCACCATCACATCTGGCTAATT

TTTTTTTTTTTTTTTTTGAGGTGGAGTCTCGCTCTGTTGCCCAGATTCAAGTGCAATGGCACGTTTTGGC

TCACTGCAACCTCTGCCTCCCAGGTTCAAGCGATTCTCCCTGTCTCGGCCTCCCGAGTAGCTGGGACTAC

AGGCACCTGCCACCACGCGCAGCTAATGTTTGTATTTTTAGTAGAGACGGGGGTTTCACCATGTTGGCCA

GGCTGGTTTCAAACTCCTGACATTAGGTGATCCACCTGCCTCGGCCTCCCAAAGTGCTGGGATTACAGGC

ATGAGCCACCGTGCCAAGCCACGCCTGGCTAATTTTTTAAAATTATTTTTTGCAGAGACAGAGTTTCACT

ATGTTGCCCAGGCTGGTCTTGAACTCCTTGGCCTCAAGTGATCCTCCCACCTCAGCCTCCCAAAGCATTG

GGGTTACAGGCGTGAGCCACTGCACCTGGCCCTGTTTTCATGGCTTTTATTTCCTTTCATCCCACACATT

TGTCTGCAGTACTAGACATGTTTTACAAATCAACAAGTTTACACAAGTATATGCAGCTTGTTTTGGGGGG

AAAGGAGACAAAATATGCATATTTTCTGTGTGGATTTGTGCCTCTTAATTGTGTTTCTAGTCTCTAAGGT

GACCCTTTAACCTACTCAAGATGGGGCCCAGAGAAGTGGCCTGCGTTACAGATTTATTTTGGCATATGTA

CTAAGTTCCATTTTCTCTTTACAAATAAAGTGTTTTCTTTCTTTTCTG

>NM_005228.5 Homo sapiens epidermal growth factor receptor (EGFR), transcript variant 1, mRNA

AGACGTCCGGGCAGCCCCCGGCGCAGCGCGGCCGCAGCAGCCTCCGCCCCCCGCACGGTGTGAGCGCCCG

ACGCGGCCGAGGCGGCCGGAGTCCCGAGCTAGCCCCGGCGGCCGCCGCCGCCCAGACCGGACGACAGGCC

ACCTCGTCGGCGTCCGCCCGAGTCCCCGCCTCGCCGCCAACGCCACAACCACCGCGCACGGCCCCCTGAC

TCCGTCCAGTATTGATCGGGAGAGCCGGAGCGAGCTCTTCGGGGAGCAGCGATGCGACCCTCCGGGACGG

CCGGGGCAGCGCTCCTGGCGCTGCTGGCTGCGCTCTGCCCGGCGAGTCGGGCTCTGGAGGAAAAGAAAGT

TTGCCAAGGCACGAGTAACAAGCTCACGCAGTTGGGCACTTTTGAAGATCATTTTCTCAGCCTCCAGAGG

ATGTTCAATAACTGTGAGGTGGTCCTTGGGAATTTGGAAATTACCTATGTGCAGAGGAATTATGATCTTT

CCTTCTTAAAGACCATCCAGGAGGTGGCTGGTTATGTCCTCATTGCCCTCAACACAGTGGAGCGAATTCC

TTTGGAAAACCTGCAGATCATCAGAGGAAATATGTACTACGAAAATTCCTATGCCTTAGCAGTCTTATCT

AACTATGATGCAAATAAAACCGGACTGAAGGAGCTGCCCATGAGAAATTTACAGGAAATCCTGCATGGCG

CCGTGCGGTTCAGCAACAACCCTGCCCTGTGCAACGTGGAGAGCATCCAGTGGCGGGACATAGTCAGCAG

TGACTTTCTCAGCAACATGTCGATGGACTTCCAGAACCACCTGGGCAGCTGCCAAAAGTGTGATCCAAGC

TGTCCCAATGGGAGCTGCTGGGGTGCAGGAGAGGAGAACTGCCAGAAACTGACCAAAATCATCTGTGCCC

AGCAGTGCTCCGGGCGCTGCCGTGGCAAGTCCCCCAGTGACTGCTGCCACAACCAGTGTGCTGCAGGCTG

CACAGGCCCCCGGGAGAGCGACTGCCTGGTCTGCCGCAAATTCCGAGACGAAGCCACGTGCAAGGACACC

TGCCCCCCACTCATGCTCTACAACCCCACCACGTACCAGATGGATGTGAACCCCGAGGGCAAATACAGCT

TTGGTGCCACCTGCGTGAAGAAGTGTCCCCGTAATTATGTGGTGACAGATCACGGCTCGTGCGTCCGAGC

CTGTGGGGCCGACAGCTATGAGATGGAGGAAGACGGCGTCCGCAAGTGTAAGAAGTGCGAAGGGCCTTGC

CGCAAAGTGTGTAACGGAATAGGTATTGGTGAATTTAAAGACTCACTCTCCATAAATGCTACGAATATTA

AACACTTCAAAAACTGCACCTCCATCAGTGGCGATCTCCACATCCTGCCGGTGGCATTTAGGGGTGACTC

CTTCACACATACTCCTCCTCTGGATCCACAGGAACTGGATATTCTGAAAACCGTAAAGGAAATCACAGGG

TTTTTGCTGATTCAGGCTTGGCCTGAAAACAGGACGGACCTCCATGCCTTTGAGAACCTAGAAATCATAC

GCGGCAGGACCAAGCAACATGGTCAGTTTTCTCTTGCAGTCGTCAGCCTGAACATAACATCCTTGGGATT

ACGCTCCCTCAAGGAGATAAGTGATGGAGATGTGATAATTTCAGGAAACAAAAATTTGTGCTATGCAAAT

ACAATAAACTGGAAAAAACTGTTTGGGACCTCCGGTCAGAAAACCAAAATTATAAGCAACAGAGGTGAAA

ACAGCTGCAAGGCCACAGGCCAGGTCTGCCATGCCTTGTGCTCCCCCGAGGGCTGCTGGGGCCCGGAGCC

CAGGGACTGCGTCTCTTGCCGGAATGTCAGCCGAGGCAGGGAATGCGTGGACAAGTGCAACCTTCTGGAG

GGTGAGCCAAGGGAGTTTGTGGAGAACTCTGAGTGCATACAGTGCCACCCAGAGTGCCTGCCTCAGGCCA

TGAACATCACCTGCACAGGACGGGGACCAGACAACTGTATCCAGTGTGCCCACTACATTGACGGCCCCCA

CTGCGTCAAGACCTGCCCGGCAGGAGTCATGGGAGAAAACAACACCCTGGTCTGGAAGTACGCAGACGCC

GGCCATGTGTGCCACCTGTGCCATCCAAACTGCACCTACGGATGCACTGGGCCAGGTCTTGAAGGCTGTC

CAACGAATGGGCCTAAGATCCCGTCCATCGCCACTGGGATGGTGGGGGCCCTCCTCTTGCTGCTGGTGGT

GGCCCTGGGGATCGGCCTCTTCATGCGAAGGCGCCACATCGTTCGGAAGCGCACGCTGCGGAGGCTGCTG

CAGGAGAGGGAGCTTGTGGAGCCTCTTACACCCAGTGGAGAAGCTCCCAACCAAGCTCTCTTGAGGATCT

TGAAGGAAACTGAATTCAAAAAGATCAAAGTGCTGGGCTCCGGTGCGTTCGGCACGGTGTATAAGGGACT

CTGGATCCCAGAAGGTGAGAAAGTTAAAATTCCCGTCGCTATCAAGGAATTAAGAGAAGCAACATCTCCG

AAAGCCAACAAGGAAATCCTCGATGAAGCCTACGTGATGGCCAGCGTGGACAACCCCCACGTGTGCCGCC

TGCTGGGCATCTGCCTCACCTCCACCGTGCAGCTCATCACGCAGCTCATGCCCTTCGGCTGCCTCCTGGA

CTATGTCCGGGAACACAAAGACAATATTGGCTCCCAGTACCTGCTCAACTGGTGTGTGCAGATCGCAAAG

GGCATGAACTACTTGGAGGACCGTCGCTTGGTGCACCGCGACCTGGCAGCCAGGAACGTACTGGTGAAAA

CACCGCAGCATGTCAAGATCACAGATTTTGGGCTGGCCAAACTGCTGGGTGCGGAAGAGAAAGAATACCA

TGCAGAAGGAGGCAAAGTGCCTATCAAGTGGATGGCATTGGAATCAATTTTACACAGAATCTATACCCAC

CAGAGTGATGTCTGGAGCTACGGGGTGACTGTTTGGGAGTTGATGACCTTTGGATCCAAGCCATATGACG

GAATCCCTGCCAGCGAGATCTCCTCCATCCTGGAGAAAGGAGAACGCCTCCCTCAGCCACCCATATGTAC

CATCGATGTCTACATGATCATGGTCAAGTGCTGGATGATAGACGCAGATAGTCGCCCAAAGTTCCGTGAG

TTGATCATCGAATTCTCCAAAATGGCCCGAGACCCCCAGCGCTACCTTGTCATTCAGGGGGATGAAAGAA

TGCATTTGCCAAGTCCTACAGACTCCAACTTCTACCGTGCCCTGATGGATGAAGAAGACATGGACGACGT

GGTGGATGCCGACGAGTACCTCATCCCACAGCAGGGCTTCTTCAGCAGCCCCTCCACGTCACGGACTCCC

CTCCTGAGCTCTCTGAGTGCAACCAGCAACAATTCCACCGTGGCTTGCATTGATAGAAATGGGCTGCAAA

GCTGTCCCATCAAGGAAGACAGCTTCTTGCAGCGATACAGCTCAGACCCCACAGGCGCCTTGACTGAGGA

CAGCATAGACGACACCTTCCTCCCAGTGCCTGAATACATAAACCAGTCCGTTCCCAAAAGGCCCGCTGGC

TCTGTGCAGAATCCTGTCTATCACAATCAGCCTCTGAACCCCGCGCCCAGCAGAGACCCACACTACCAGG

ACCCCCACAGCACTGCAGTGGGCAACCCCGAGTATCTCAACACTGTCCAGCCCACCTGTGTCAACAGCAC

ATTCGACAGCCCTGCCCACTGGGCCCAGAAAGGCAGCCACCAAATTAGCCTGGACAACCCTGACTACCAG

CAGGACTTCTTTCCCAAGGAAGCCAAGCCAAATGGCATCTTTAAGGGCTCCACAGCTGAAAATGCAGAAT

ACCTAAGGGTCGCGCCACAAAGCAGTGAATTTATTGGAGCATGACCACGGAGGATAGTATGAGCCCTAAA

AATCCAGACTCTTTCGATACCCAGGACCAAGCCACAGCAGGTCCTCCATCCCAACAGCCATGCCCGCATT

AGCTCTTAGACCCACAGACTGGTTTTGCAACGTTTACACCGACTAGCCAGGAAGTACTTCCACCTCGGGC

ACATTTTGGGAAGTTGCATTCCTTTGTCTTCAAACTGTGAAGCATTTACAGAAACGCATCCAGCAAGAAT

ATTGTCCCTTTGAGCAGAAATTTATCTTTCAAAGAGGTATATTTGAAAAAAAAAAAAAGTATATGTGAGG

ATTTTTATTGATTGGGGATCTTGGAGTTTTTCATTGTCGCTATTGATTTTTACTTCAATGGGCTCTTCCA

ACAAGGAAGAAGCTTGCTGGTAGCACTTGCTACCCTGAGTTCATCCAGGCCCAACTGTGAGCAAGGAGCA

CAAGCCACAAGTCTTCCAGAGGATGCTTGATTCCAGTGGTTCTGCTTCAAGGCTTCCACTGCAAAACACT

AAAGATCCAAGAAGGCCTTCATGGCCCCAGCAGGCCGGATCGGTACTGTATCAAGTCATGGCAGGTACAG

TAGGATAAGCCACTCTGTCCCTTCCTGGGCAAAGAAGAAACGGAGGGGATGGAATTCTTCCTTAGACTTA

CTTTTGTAAAAATGTCCCCACGGTACTTACTCCCCACTGATGGACCAGTGGTTTCCAGTCATGAGCGTTA

GACTGACTTGTTTGTCTTCCATTCCATTGTTTTGAAACTCAGTATGCTGCCCCTGTCTTGCTGTCATGAA

ATCAGCAAGAGAGGATGACACATCAAATAATAACTCGGATTCCAGCCCACATTGGATTCATCAGCATTTG

GACCAATAGCCCACAGCTGAGAATGTGGAATACCTAAGGATAGCACCGCTTTTGTTCTCGCAAAAACGTA

TCTCCTAATTTGAGGCTCAGATGAAATGCATCAGGTCCTTTGGGGCATAGATCAGAAGACTACAAAAATG

AAGCTGCTCTGAAATCTCCTTTAGCCATCACCCCAACCCCCCAAAATTAGTTTGTGTTACTTATGGAAGA

TAGTTTTCTCCTTTTACTTCACTTCAAAAGCTTTTTACTCAAAGAGTATATGTTCCCTCCAGGTCAGCTG

CCCCCAAACCCCCTCCTTACGCTTTGTCACACAAAAAGTGTCTCTGCCTTGAGTCATCTATTCAAGCACT

TACAGCTCTGGCCACAACAGGGCATTTTACAGGTGCGAATGACAGTAGCATTATGAGTAGTGTGGAATTC

AGGTAGTAAATATGAAACTAGGGTTTGAAATTGATAATGCTTTCACAACATTTGCAGATGTTTTAGAAGG

AAAAAAGTTCCTTCCTAAAATAATTTCTCTACAATTGGAAGATTGGAAGATTCAGCTAGTTAGGAGCCCA

CCTTTTTTCCTAATCTGTGTGTGCCCTGTAACCTGACTGGTTAACAGCAGTCCTTTGTAAACAGTGTTTT

AAACTCTCCTAGTCAATATCCACCCCATCCAATTTATCAAGGAAGAAATGGTTCAGAAAATATTTTCAGC

CTACAGTTATGTTCAGTCACACACACATACAAAATGTTCCTTTTGCTTTTAAAGTAATTTTTGACTCCCA

GATCAGTCAGAGCCCCTACAGCATTGTTAAGAAAGTATTTGATTTTTGTCTCAATGAAAATAAAACTATA

TTCATTTCCACTCTATTATGCTCTCAAATACCCCTAAGCATCTATACTAGCCTGGTATGGGTATGAAAGA

TACAAAGATAAATAAAACATAGTCCCTGATTCTAAGAAATTCACAATTTAGCAAAGGAAATGGACTCATA

GATGCTAACCTTAAAACAACGTGACAAATGCCAGACAGGACCCATCAGCCAGGCACTGTGAGAGCACAGA

GCAGGGAGGTTGGGTCCTGCCTGAGGAGACCTGGAAGGGAGGCCTCACAGGAGGATGACCAGGTCTCAGT

CAGCGGGGAGGTGGAAAGTGCAGGTGCATCAGGGGCACCCTGACCGAGGAAACAGCTGCCAGAGGCCTCC

ACTGCTAAAGTCCACATAAGGCTGAGGTCAGTCACCCTAAACAACCTGCTCCCTCTAAGCCAGGGGATGA

GCTTGGAGCATCCCACAAGTTCCCTAAAAGTTGCAGCCCCCAGGGGGATTTTGAGCTATCATCTCTGCAC

ATGCTTAGTGAGAAGACTACACAACATTTCTAAGAATCTGAGATTTTATATTGTCAGTTAACCACTTTCA

TTATTCATTCACCTCAGGACATGCAGAAATATTTCAGTCAGAACTGGGAAACAGAAGGACCTACATTCTG

CTGTCACTTATGTGTCAAGAAGCAGATGATCGATGAGGCAGGTCAGTTGTAAGTGAGTCACATTGTAGCA

TTAAATTCTAGTATTTTTGTAGTTTGAAACAGTAACTTAATAAAAGAGCAAAAGCTATTCTAGCTTTCTT

CTTCATATTTTAATTTTCCACCATAAAGTTTAGTTGCTAAATTCTATTAATTTTAAGATTGTGCTTCCCA

AAATAGTTCTCACTTCATCTGTCCAGGGAGGCACAGTTCTGTCTGGTAGAAGCCGCAAAGCCCTTAGCCT

CTTCACGGATCTGGCGACTGTGATGGGCAGGTCAGGAGAGGAGCTGCCCAAAGTCCCATGATTTTCACCT

AACAGCCCTGATCAGTCAGTACTCAAAGCTTGGACTCCATCCCTGAAGGTCTTCCTGATTGATAGCCTGG

CCTTAATACCCTACAGAAAGCCTGTCCATTGGCTGTTTCTTCCTCAGTCAGTTCCTGGAAGACCTTACCC

CATGACCCCAGCTTCAGATGTGGTCTTTGGAAACAGAGGTCGAAGGAAAGTAAGGAGCTGAGAGCTCACA

TTCATAGGTGCCGCCAGCCTTCGTGCATCTTCTTGCATCATCTCTAAGGAGCTCCTCTAATTACACCATG

CCCGTCACCCCATGAGGGATCAGAGAAGGGATGAGTCTTCTAAACTCTATATTCGCTGTGAGTCCAGGTT

GTAAGGGGGAGCACTGTGGATGCATCCTATTGCACTCCAGCTGATGACACCAAAGCTTAGGTGTTTGCTG

AAAGTTCTTGATGTTGTGACTTACCACCCCTGCCTCACAACTGCAGACATAAGGGGACTATGGATTGCTT

AGCAGGAAAGGCACTGGTTCTCAAGGGCGGCTGCCCTTGGGAATCTTCTGGTCCCAACCAGAAAGACTGT

GGCTTGATTTTCTCAGGTGCAGCCCAGCCGTAGGGCCTTTTCAGAGCACCCCCTGGTTATTGCAACATTC

ATCAAAGTTTCTAGAACCTCTGGCCTAAAGGAAGGGCCTGGTGGGATCTACTTGGCACTCGCTGGGGGGC

CACCCCCCAGTGCCACTCTCACTAGGCCTCTGATTGCACTTGTGTAGGATGAAGCTGGTGGGTGATGGGA

ACTCAGCACCTCCCCTCAGGCAGAAAAGAATCATCTGTGGAGCTTCAAAAGAAGGGGCCTGGAGTCTCTG

CAGACCAATTCAACCCAAATCTCGGGGGCTCTTTCATGATTCTAATGGGCAACCAGGGTTGAAACCCTTA

TTTCTAGGGTCTTCAGTTGTACAAGACTGTGGGTCTGTACCAGAGCCCCCGTCAGAGTAGAATAAAAGGC

TGGGTAGGGTAGAGATTCCCATGTGCAGTGGAGAGAACAATCTGCAGTCACTGATAAGCCTGAGACTTGG

CTCATTTCAAAAGCGTTCAATTCATCCTCACCAGCAGTTCAGCTGGAAAGGGGCAAATACCCCCACCTGA

GCTTTGAAAACGCCCTGGGACCCTCTGCATTCTCTAAGTAAGTTATAGAAACCAGTCTCTTCCCTCCTTT

GTGAGTGAGCTGCTATTCCACGTAGGCAACACCTGTTGAAATTGCCCTCAATGTCTACTCTGCATTTCTT

TCTTGTGATAAGCACACACTTTTATTGCAACATAATGATCTGCTCACATTTCCTTGCCTGGGGGCTGTAA

AACCTTACAGAACAGAAATCCTTGCCTCTTTCACCAGCCACACCTGCCATACCAGGGGTACAGCTTTGTA

CTATTGAAGACACAGACAGGATTTTTAAATGTAAATCTATTTTTGTAACTTTGTTGCGGGATATAGTTCT

CTTTATGTAGCACTGAACTTTGTACAATATATTTTTAGAAACTCATTTTTCTACTAAAACAAACACAGTT

TACTTTAGAGAGACTGCAATAGAATCAAAATTTGAAACTGAAATCTTTGTTTAAAAGGGTTAAGTTGAGG

CAAGAGGAAAGCCCTTTCTCTCTCTTATAAAAAGGCACAACCTCATTGGGGAGCTAAGCTAGGTCATTGT

CATGGTGAAGAAGAGAAGCATCGTTTTTATATTTAGGAAATTTTAAAAGATGATGGAAAGCACATTTAGC

TTGGTCTGAGGCAGGTTCTGTTGGGGCAGTGTTAATGGAAAGGGCTCACTGTTGTTACTACTAGAAAAAT

CCAGTTGCATGCCATACTCTCATCATCTGCCAGTGTAACCCTGTACATGTAAGAAAAGCAATAACATAGC

ACTTTGTTGGTTTATATATATAATGTGACTTCAATGCAAATTTTATTTTTATATTTACAATTGATATGCA

TTTACCAGTATAAACTAGACATGTCTGGAGAGCCTAATAATGTTCAGCACACTTTGGTTAGTTCACCAAC

AGTCTTACCAAGCCTGGGCCCAGCCACCCTAGAGAAGTTATTCAGCCCTGGCTGCAGTGACATCACCTGA

GGAGCTTTTAAAAGCTTGAAGCCCAGCTACACCTCAGACCGATTAAACGCAAATCTCTGGGGCTGAAACC

CAAGCATTCGTAGTTTTTAAAGCTCCTGAGGTCATTCCAATGTGCGGCCAAAGTTGAGAACTACTGGCCT

AGGGATTAGCCACAAGGACATGGACTTGGAGGCAAATTCTGCAGGTGTATGTGATTCTCAGGCCTAGAGA

GCTAAGACACAAAGACCTCCACATCTGTCGCTGAGAGTCAAGAACCTGAACAGAGTTTCCATGAAGGTTC

TCCAAGCACTAGAAGGGAGAGTGTCTAAACAATGGTTGAAAAGCAAAGGAAATATAAAACAGACACCTCT

TTCCATTTCCTAAGGTTTCTCTCTTTATTAAGGGTGGACTAGTAATAAAATATAATATTCTTGCTGCTTA

TGCAGCTGACATTGTTGCCCTCCCTAAAGCAACCAAGTAGCCTTTATTTCCCACAGTGAAAGAAAACGCT

GGCCTATCAGTTACATTACAAAAGGCAGATTTCAAGAGGATTGAGTAAGTAGTTGGATGGCTTTCATAAA

AACAAGAATTCAAGAAGAGGATTCATGCTTTAAGAAACATTTGTTATACATTCCTCACAAATTATACCTG

GGATAAAAACTATGTAGCAGGCAGTGTGTTTTCCTTCCATGTCTCTCTGCACTACCTGCAGTGTGTCCTC

TGAGGCTGCAAGTCTGTCCTATCTGAATTCCCAGCAGAAGCACTAAGAAGCTCCACCCTATCACCTAGCA

GATAAAACTATGGGGAAAACTTAAATCTGTGCATACATTTCTGGATGCATTTACTTATCTTTAAAAAAAA

AGGAATCCTATGACCTGATTTGGCCACAAAAATAATCTTGCTGTACAATACAATCTCTTGGAAATTAAGA

GATCCTATGGATTTGATGACTGGTATTAGAGGTGACAATGTAACCGATTAACAACAGACAGCAATAACTT

CGTTTTAGAAACATTCAAGCAATAGCTTTATAGCTTCAACATATGGTACGTTTTAACCTTGAAAGTTTTG

CAATGATGAAAGCAGTATTTGTACAAATGAAAAGCAGAATTCTCTTTTATATGGTTTATACTGTTGATCA

GAAATGTTGATTGTGCATTGAGTATTAAAAAATTAGATGTATATTATTCATTGTTCTTTACTCCTGAGTA

CCTTATAATAATAATAATGTATTCTTTGTTAACAA

>NM_002759.4 Homo sapiens eukaryotic translation initiation factor 2 alpha kinase 2 (EIF2AK2), transcript variant 1, mRNA

GAAACTGAGAACCAGCTCTCCCGAAGCCGCGGGTCTCCGGCCGGCGGCGGCGGCGGCGGCGGCGGCG

CAGTTTGCTCATACTTTGTGACTTGCGGTCACAGTGGCATTCAGCTCCACACTTGGTAGAACCACAGGCA

CGACAAGCATAGAAACATCCTAAACAATCTTCATCGAGGCATCGAGGTCCATCCCAATAAAAATCAGGAG

ACCCTGGCTATCATAGACCTTAGTCTTCGCTGGTATCACTCGTCTGTCTGAACCAGCGGTTGCATTTTTT

TAAGCCTTCTTTTTTCTCTTTTACCAGTTTCTGGAGCAAATTCAGTTTGCCTTCCTGGATTTGTAAATTG

TAATGACCTCAAAACTTTAGCAGTTCTTCCATCTGACTCAGGTTTGCTTCTCTGGCGGTCTTCAGAATCA

ACATCCACACTTCCGTGATTATCTGCGTGCATTTTGGACAAAGCTTCCAACCAGGATACGGGAAGAAGAA

ATGGCTGGTGATCTTTCAGCAGGTTTCTTCATGGAGGAACTTAATACATACCGTCAGAAGCAGGGAGTAG

TACTTAAATATCAAGAACTGCCTAATTCAGGACCTCCACATGATAGGAGGTTTACATTTCAAGTTATAAT

AGATGGAAGAGAATTTCCAGAAGGTGAAGGTAGATCAAAGAAGGAAGCAAAAAATGCCGCAGCCAAATTA

GCTGTTGAGATACTTAATAAGGAAAAGAAGGCAGTTAGTCCTTTATTATTGACAACAACGAATTCTTCAG

AAGGATTATCCATGGGGAATTACATAGGCCTTATCAATAGAATTGCCCAGAAGAAAAGACTAACTGTAAA

TTATGAACAGTGTGCATCGGGGGTGCATGGGCCAGAAGGATTTCATTATAAATGCAAAATGGGACAGAAA

GAATATAGTATTGGTACAGGTTCTACTAAACAGGAAGCAAAACAATTGGCCGCTAAACTTGCATATCTTC

AGATATTATCAGAAGAAACCTCAGTGAAATCTGACTACCTGTCCTCTGGTTCTTTTGCTACTACGTGTGA

GTCCCAAAGCAACTCTTTAGTGACCAGCACACTCGCTTCTGAATCATCATCTGAAGGTGACTTCTCAGCA

GATACATCAGAGATAAATTCTAACAGTGACAGTTTAAACAGTTCTTCGTTGCTTATGAATGGTCTCAGAA

ATAATCAAAGGAAGGCAAAAAGATCTTTGGCACCCAGATTTGACCTTCCTGACATGAAAGAAACAAAGTA

TACTGTGGACAAGAGGTTTGGCATGGATTTTAAAGAAATAGAATTAATTGGCTCAGGTGGATTTGGCCAA

GTTTTCAAAGCAAAACACAGAATTGACGGAAAGACTTACGTTATTAAACGTGTTAAATATAATAACGAGA

AGGCGGAGCGTGAAGTAAAAGCATTGGCAAAACTTGATCATGTAAATATTGTTCACTACAATGGCTGTTG

GGATGGATTTGATTATGATCCTGAGACCAGTGATGATTCTCTTGAGAGCAGTGATTATGATCCTGAGAAC

AGCAAAAATAGTTCAAGGTCAAAGACTAAGTGCCTTTTCATCCAAATGGAATTCTGTGATAAAGGGACCT

TGGAACAATGGATTGAAAAAAGAAGAGGCGAGAAACTAGACAAAGTTTTGGCTTTGGAACTCTTTGAACA

AATAACAAAAGGGGTGGATTATATACATTCAAAAAAATTAATTCATAGAGATCTTAAGCCAAGTAATATA

TTCTTAGTAGATACAAAACAAGTAAAGATTGGAGACTTTGGACTTGTAACATCTCTGAAAAATGATGGAA

AGCGAACAAGGAGTAAGGGAACTTTGCGATACATGAGCCCAGAACAGATTTCTTCGCAAGACTATGGAAA

GGAAGTGGACCTCTACGCTTTGGGGCTAATTCTTGCTGAACTTCTTCATGTATGTGACACTGCTTTTGAA

ACATCAAAGTTTTTCACAGACCTACGGGATGGCATCATCTCAGATATATTTGATAAAAAAGAAAAAACTC

TTCTACAGAAATTACTCTCAAAGAAACCTGAGGATCGACCTAACACATCTGAAATACTAAGGACCTTGAC

TGTGTGGAAGAAAAGCCCAGAGAAAAATGAACGACACACATGTTAGAGCCCTTCTGAAAAAGTATCCTGC

TTCTGATATGCAGTTTTCCTTAAATTATCTAAAATCTGCTAGGGAATATCAATAGATATTTACCTTTTAT

TTTAATGTTTCCTTTAATTTTTTACTATTTTTACTAATCTTTCTGCAGAAACAGAAAGGTTTTCTTCTTT

TTGCTTCAAAAACATTCTTACATTTTACTTTTTCCTGGCTCATCTCTTTATTCTTTTTTTTTTTTTAAAG

ACAGAGTCTCGCTCTGTTGCCCAGGCTGGAGTGCAATGACACAGTCTTGGCTCACTGCAACTTCTGCCTC

TTGGGTTCAAGTGATTCTCCTGCCTCAGCCTCCTGAGTAGCTGGATTACAGGCATGTGCCACCCACCCAA

CTAATTTTTGTGTTTTTAATAAAGACAGGGTTTCACCATGTTGGCCAGGCTGGTCTCAAACTCCTGACCT

CAAGTAATCCACCTGCCTCGGCCTCCCAAAGTGCTGGGATTACAGGGATGAGCCACCGCGCCCAGCCTCA

TCTCTTTGTTCTAAAGATGGAAAAACCACCCCCAAATTTTCTTTTTATACTATTAATGAATCAATCAATT

CATATCTATTTATTAAATTTCTACCGCTTTTAGGCCAAAAAAATGTAAGATCGTTCTCTGCCTCACATAG

CTTACAAGCCAGCTGGAGAAATATGGTACTCATTAAAAAAAAAAAAAAAAAGTGATGTACAACCACTTCGG

AAAACAATTTGGCATTATCTAGTAAAGTTGAATCCATGTATACCCACATAGCTATCAATTCTATTCCTAC

ATACGTGCTTACAAGAATGTCCATAAAACCCTGTTTATAATAGCCAAAAGAACAGGGAACAACCATAATG

CACATCAAAAGAAGAATGGATTAAAAAAATTATATTCACACACAGGAGTACTATATAGTATTGAAAACAA

TTGAAGTACAGCTAAATGTAATAACGTAACACAATACAACTCTCAGAAACATAATGTTAAGCGAACAAAG

CAGGTTTTCAGAAAATATATGCAGAATAATTCCATTTATATAAAGTTCCAGAGCATGCAAAACTAAATCA

TTTTGTATAAAAAAACCCAACAAATGTGATGAGACAATAATGGGAAGGAAGGGAATGAGAAATATTAAATT

CTGGATGGTGGTTATCTTTGAGGGAGGGGAATGATGTGATTGGGGAAATGGACTTTCAAAGGTAATGGTA

ACTTCCTTAAGCTGGATGGTAGGTCCACTAGTGTTTGCTGCATAGTTATACCTTTTATCTTAAATACATT

TTGTATCTATTGTAACAACCACTTTAAAGACAACCGTGCTGTAAGGCAGTAGCTAAAAACAGAAAATAGT

CCATCGGGAAGGGTAAGATGGCTTTCTGCTGAGCACAGGGCTAGAAGTGACAGCCCAGTGGGCCTTCCAA

CTATATGCCAGGGTGTTAGATGAGTAGAGAGGAGACCACCCAGGAAGTCTGGACAAGGGGTCTGGCATGA

GCTCTGGAGAAGATATATTTGAGGAACATGGGGTATGCTAGTTTGTTGTCCTGAATTGCTGTAGAGAAGA

TAATTTAAATTGCATCTTAGAAGACGACCCTGAGGGTGAATTTCAACTTAGGGCAATTGTTTTAGTTTGT

TTCTTATTGGTTTAAATGGATACTTGAAGCTGGATAATTTATAAGGAAAAGAGATTTATATGACTTACAG

TTCTGCAGGCTGTACAAGAAACATGGCACCAGCATCTGCTTCTTCCCCGGCTGCTTCCACTCATGGTGGA

AGGTGAAGGGGAGCCGGATGTGCAGAGATCATATGGCAAGAGAGGAAGCAAGAGAGCGAGGGAGAAGGTG

CCAGGCTCTTTTTAAATAACCGGCTCTTGAGGGAACTAATAGATTGAGAACTCCTTGCTTCTCCTCCCCA

GCACACCCCACCCCCAGGGACGGCATTAATGTATTCATGAGGGGTCTTCCCCCATGACCCAAACACCTCC

CATCAGGCCCCACCTCCAACACTGGGATCAAATTTCAACATGAGATTTTGGGGGACAAACATGCAAACTA

TAGCAGCAACCAGCTACCATTCTAAAACTGCCATATGATTTTAGGATTTTTAAAAAGGGCCAAATTTAGG

TTAAGCAAAAAGTGAAAATACAGTAAGAGAAAATAGTTTTATAAAGGCTTTAAAAAGTGTTCATAAAGAG

CAGTTCATAGATTTTTCACGTAACGTTCATCAATAGATGCTAAAACTATTGGATGAAAAGCTATTGTGGA

ATAGGATCTTCTACAAGGATTTAAATATAATCCTACAGAAGAGGTATCAATTATACAGGGGAAAATGTAC

CTTTACAATGGTCACCACAATAACCAAGTGATTCAATTTAGCATCACCCAAGGTGGAACACCCTAACATT

ATGCCCCTCCTGATGTGACTTAAGAATTTTACCTATTTAGTTTTCTTGCACAAAGTGTTAAACTGGAATT

TAATCATGAGGAAATTATTAGACAAATCCTGAATGTAGTCATTCTATAAGATGATTGACTTGGGCTTCTC

AAAAAGTCAGTGTCATGAAAAAAAAAAAAAAGGGGAGAGACTTGTAAGATACAATGACCAAATGCAATGTT

TGAATCTTTATTGTATTCTGGATATTTAAAAAGACAAAAGCTGGACGCAGTAGCTAATGCCTGTTATCCC

AGCATTTTTGGGAGGTCAAAAGGGGAGGATGGCTTGAGCATAGGATTTTGAGACTAGCCTGGGCAACACA

GTGAGTTCTCATCTCTACAAAAAATCAGAAAATTAGCTGGGCATGGTAGTGTGCATCTGTGGTCCCAGCT

ACTTGGGGGGGTGAGGTGGGAGGATCACTTGAGCCTAGGAGGTCAAGGCCGCAGTGAGCCATGATCATGT

TACTGCATTCCAGCCTGGGTGACAGAGTGAGATCATATCTAAATAATAATAATAATAAAAGAAAGCAACA

ACCCAGCAAACAGCTATAAAGATATCTTCGGGACAATTGAGAACATTTGAATATAGACTGCATATTAGAT

AACAATGCCAAATAACTGTTTTCTCAGGTGTGATAATAGTATATGTAGAAAATGTCTTTTTGTTTTGTTT

TATTTTGTTTTTGTTTGTTTGTTTGTTTTGAGACAGAGTCTCACTGTGTCGCCCAGGCTGGAGTGCAGTG

GCACGATCTCGGCTCACTGCAACCTCTGCCTCCCAGGTTCAAGTGATTCTCCTGCCTCTGCTTCCTGAGT

AGCTGGGATTACAGGTATCTGCCACCACTCCAGGTTAATTTTTTCTATTTTTAGTAGAGACGGAATTTCA

CCATGTTGACCCAGCTGGTTTTGAACTCCTGACCTCAGGTGATTCACTTGCCTTGGCCTCCCAAAGTGTT

GAGATTACAGGCATGAGTCACTGCACCCAGCCAAAAATGTTTTTTTAGGCAATGCATGCACAAACATTTA

GAGGCCAAGTGTGTCATGATGTCTGCAACTTTCAATGTTCAGCAAAAATCAACATGGAGAGAGAAAGAGG

GCACACATGTGGCAAAATATTAGCCATTGTACAATTGGTAAATCTAGATGACAGTACACAGGTGTTTGTT

GTATTATTCTTTGAACTTTTCTGTAGATTTGGAAATGTTCTGCGTAGAGAGAAATCTTTTTTTAAAAAAA

CAATATCTGGTTTTGATTAATGAGGCTATATTCAAAAGAAACACTAAATGATGTGGCCAGTTAGTGAGAA

TGTAGCCTTATCTTTACTGACCTCATTTTAATATTCCTACTCTGGCTGGGCATGGTGGCTCACGCCTATA

ATCCCAGCACTCTGGGAGGCCGAGACGGGTGGATCACCTGAGGTCAGGGGTTCGAGACCAGCCTGGCCAA

CATGGTGAAACCCCATCTCTACTAAAAATACAAAAATTAGCTGGGTGTGGTGGTAGGCGCCTGTAATCCC

AGCTACTCGGGAGTCCGTGGCAGGAGAATCGCTTGAACCCAGGAGGTGGAGGTTGCAGTGAGCTGAGATC

ACGCCATTGCACTCCAGCCTGGGTGACAAGAGCAAAACTCAGTCTCAAAAAAAAAAAAGAAAAAAAAAGA

TATATATATTCCTACTCTTATTAAATGAATATAGCATTTCTTAGCAACTTCTTTTGATTTGTGAGTATAT

ATTTTCTTGGCCTCAAAATTAAAGTAAAAAGTATCCTCTTACTCAGAGGAGGACAGGGGCAAGAGCCTGG

GACCTTATTTGCTAATTAAAATGCACACACACACACACACACACAGAAATTTTGAGAGCCATTTTAATAT

AATTGCCTCCCTAGAAACATACCTTTTAGGGAATTTTTATCACTAAACCACATGTTATTTAAATACGTAC

ATGTTTAACATAAATACATACATAAAATTCACATGCATACTTAACACTTATGTTAAATATATTCAATGTA

TATACATATGTACACAATATATGCATATATACATGTGGGTATGTGGTATGTGTGCATGTGTGTGTATGGC

CAGCTACATAATTTGTGGGACTAAGGGCAAAATGAAACTGTACGGCCCTCGTTCAAAAATTAGGTGTGGG

GTGCTTCTAAGCACAGTCTTGTGCAACTGCACAGGTTGCATGTCCATGAAGCCATCCTGTGTGTGTGCAC

ATATGTGTATGAATATGTATTTTATATAATACATATATATGCATGTATGTATCTGAGAAGAAATGTTTAA

AAGGCTACATACAAGCTTTTCCAGGTCTCTACTATCTGTTAACTAACTAGAGACATAACTCACAATATGC

AGTCCCCACTGAGTGTTCACCATAATTTGAGATTCTTGGCATGTTAACTTTTCATTATGGAATATTGAAT

AATTTCAATATTATTCATACATTTCTTTATGTTCAAACATACACAAAAATAGAATAATGAAACTCTACCC

ATCACCCAGCTGCAACAAATATCAATACTTTACCGTTCTTAATACATCTAACCCCTTACTTTTTGTTTGT

TTCTTTTGGTGAAGTATTTAATTGTAATTTTTTTTTTAAGAGACAGGATCTCACTCTGTCACCCAGGCCA

GAGTACAGTGGTACGATCACAGCTCCTTGTAACCTCGAACTCCTAGGCTCAAGTGATCCTCCTGCCTCAG

CCGCTTGAATAGCTGGGACTACATGCATACACCAACACACCCAACTAATTTTTAAATTTTTTGTAGAAAT

ATGGTCTTGCTGTGTTTCCCAGGCTGGTCTCAAACTCCTGGCCTTGAGAGATTCTCCTGCCTTGGTCTTC

CAAACTGCTGGGATTACAATATCCGGCCCTACTGGAATATTTTAAAGTAAATCTCAGGTGTTACATCATT

GCACTTGTAAGTACTTTAAAATGTATCTCTAGCAGATAAATCTTTTTAACAAAACCACAGCATCATTGTC

ACACTCAAAAAATTGACAAGATTTCCTTAATTATCATATAATAGTCTGTATTGTTTCTATCTCAAAAATA

CAGTTTTCTTGGTTGTTGAAATCTAGATCCAACATTGCATTTGGTTGATGTGTCTCCTTTACTCTGTAAA

ATTTACCCCTGCGTTTTAGTTCTTTTCGGCTTTTATTTGTCCTGTGGAATTTCCTCCACTCAGGATTTGA

CTGAGAGTATCCCCAGAGTGTCCAGTTAGACCTAGAAGCTGGATTTGATCCTGGTTCATTTATTTTCCCC

CAAGAATACTCCATAGGTGATGCTATACACTTTCTCTTACATCATTTCAGGAGGAACATGATGTCTGATT

GTTCCACTTTCAGTGATTTTAATATTGATCAATGAGTTCTGGTGGTACCAACTACTACAAAGTTTCTCAT

CAATCTTTCACCTGATGGTTTTAGCAGCTATTGATAATTGTTGCCTGGATCCATTATTTCATTTCAGGGG

TTGTAAAATAGTGATTTTCTGGTTCTATCAAACCTCTTGCATTTATCATCTATGATTCTTCTTTAAGGAA

CTTTCTCTAAATCTGAATGGGAAAGATGCGATAAATATTTATCAAATTTTAGAGTGAGTTAGTGCCCTAG

CAACCTCCACAGTTGACAAATGAGTATTTCTTTAAAGCAAGGTATGGCCCAACCAATACTTTGAAACTAG

CATGTTTAGAATAGAGCAGGAGGAAACTATATTTGAAAGGTCAGAAGTGGAAAGACTAAGAGGGCCTGAA

CAATAAGAGGACAGAACCAAGAGGAGTTGGCAACTAATTGGATGTGGGGATTAAGGAAAGGTAAGCATCA

AAGATTACCTCCAAGTTTGTTAGAAGGTTAGTAGCAGGATTCTGATGCCATTCAAGTAAATACAAGTCTC

AGTCAGATGAACCCCAAGAGCCACATGTATTTGAGGGGTACTTTGTCTCACACTTTTACCTGTTACATGG

TTTTCAGTAATTTAGAATTTAAGCCAGTAGTGGGGCGACTGTACATCTATCGACATGGTGAGGTAGAGCA

TGTTTGGGAGGAAAGACGTTGAATCCCATTTGGTGACAGTGAGCTTGAGGTGCTGCCAGAACACTGCACT

GAAGATAGGAGGAGACTGTAGGAAATACAAGATAGGAAAGGTCTCCACTGAAATGTTAACTCTTTCTCTC

TAAACGGCCATCCAGGCCTCAATGTCTGCAGTTTCTGATCTGTGATTATGACTTATCCAAATCTTACATT

TCTTAAAAATAGTCATAGATGAAGGGAATCACAGTTGATAGTTATATGGTGACATTATTGGCTTAAATTC

TAAATAACTAGAAACTGTATAATAGGCAAAACTGTGAGGCAAATAAAATGCTTCTCAAATCTGTGTGGCT

CTTATGGGGTTAATTTGATTTGGACCTGTATTAATTTCTTATGGCTGCTATAACTAACAAATTACCACAA

ACTTGGTGGTTTAAAACAACACACATTTATTCTCTTTCTGTTCTGGAGGCCAGAAGTCTAAAATGAGATT

CACTGGGCTGCAGTTCACTGGGCAAGGCCATGCTCCTCTGGAGGCTTCCATGATGCATCCCATATTCAGT

GTTTCCCGAGTAAGCCCCACCCATGCAGGTCTGCAGTTTTACCTCAACAGGCTTTTGCACTCAGTGGCTC

TCTCCTGTGGTTTCTATCTGAAATTCTCTTCATTTTTTTTTTAATAACTGCTTTATTGAGATATAATTCA

CATGCCATACAATTCACCTATACAGTATACAATTCAGTAGTGTTTACTATATTCAGAGTTGTGCAACTAT

CATTTTTCTCACCCCCAAAAGAAACCCTATGCCCGTTAGTATTCACTCTGTTTTCTCACAACTCTAGGTA

ACCACTAATCTACTCTCCATCTCTATAGATTTGCCCATGCTAGACATTTCATATAAATGGAATCATACAT

GTGGTCTTTTTCACTGAGTATATTTTCAAGGTGCATCCCAGCTGTGGCATGAATCAGCATCTCATTCCTT

TTTATTGCCAAATAATATTCTATCATATGGATATGTCGCATTTTATGTATCCATTCATCAGCTGATGGAC

ATTTGGGTTGTTTGTACTTTTTGGCTACCATGAATAATGCTACTATGAACTTTTGTGTACAAATCTTATG

TGGACTTGTTTTCATTTCTCTTGGATGTATATTCCTAGGAGAATGGCTGGATTATACAGTAACTCTTTCC

TCATAACTTTGCATATCAAAATTATATCCATCTTTCAGAACCCAAATTCAGTGTTATTTCCAAAGTGTTC

CAACATCTCCCACTCAAAATGAATCTTCTCCCTAGTACTCCCATTGTTTAGTACCTAATTGTTCTTACCA

GTTTGTCTTAGAGCTCAGTCATTCCTACACAGCACTTTTCTTCATTATACTGCTAATTTCATTACACTGT

AAGAGTGGGGACAAGCTGTTCTTCTTGTATACCAGAAAGTAGTGAGTGCACTGCTGTGAAGAAAGCAGGA

ACTCAAAAGTTAAGAATTTTGAATGAGCCTATAAAATGTGTCTAATAAATAATAATGTTCATTTTCAAAT

CTCAACAATTAAATGTTTAACAAATAACACTTGAAATAAATGTTTATTAGAAAAACCAA

>NM_004836.7 Homo sapiens eukaryotic translation initiation factor 2 alpha kinase 3 (EIF2AK3), transcript variant 1, mRNA

AGGCTGTCACTCAGGTGGCAGCGGCAGAGGCCGGGCTGAGACGTGGCCAGGGGAACACGGCTGGCTGTCC

AGGCCGTCGGGGCGGCAGTAGGGTCCCTAGCACGTCCTTGCCTTCTTGGGAGCTCCAAGCGGCGGGAGAG

GCAGGCGTCAGTGGCTGCGCCTCCATGCCTGCGCGCGGGGCGGGACGCTGATGGAGCGCGCCATCAGCCC

GGGGCTGCTGGTACGGGCGCTGCTGCTGCTGCTGCTGCTGGGGCTCGCGGCAAGGACGGTGGCCGCG

GGGCGCGCCCGTGGCCTCCCAGCGCCGACGGCGGAGGCGGCGTTCGGCCTCGGGGCGGCCGCTGCTCCCA

CCTCAGCGACGCGAGTACCGGCGGCGGGCGCCGTGGCTGCGGCCGAGGTGACTGTGGAGGACGCTGAGGC

GCTGCCGGCAGCCGCGGGAGAGCAGGAGCCTCGGGGTCCGGAACCAGACGATGAGACAGAGTTGCGACCG

CGCGGCAGGTCATTAGTAATTATCAGCACTTTAGATGGGAGAATTGCTGCCTTGGATCCTGAAAATCATG

GTAAAAAGCAGTGGGATTTGGATGTGGGATCCGGTTCCTTGGTGTCATCCAGCCTTAGCAAACCAGAGGT

ATTTGGGAATAAGATGATCATTCCTTCCCTGGATGGAGCCCTCTTCCAGTGGGACCAAGACCGTGAAAGC

ATGGAAACAGTTCCTTTCACAGTTGAATCACTTCTTGAATCTTCTTATAAATTTGGAGATGATGTTGTTT

TGGTTGGAGGAAAATCTCTGACTACATATGGACTCAGTGCATATAGTGGAAAGGTGAGGTATATCTGTTC

AGCTCTGGGTTGTCGCCAATGGGATAGTGACGAAATGGAACAAGAGGAAGACATCCTGCTTCTACAGCGT

ACCCAAAAAACTGTTAGAGCTGTCGGACCTCGCAGTGGCAATGAGAAGTGGAATTTCAGTGTTGGCCACT

TTGAACTTCGGTATATTCCAGACATGGAAACGAGAGCCGGATTTATTGAAAGCACCTTTAAGCCCAATGA

GAACACAGAAGAGTCTAAAATTATTTCAGATGTGGAAGAACAGGAAGCTGCCATAATGGACATAGTGATA

AAGGTTTCGGTTGCTGACTGGAAAGTTATGGCATTCAGTAAGAAGGGAGGACATCTGGAATGGGAGTACC

AGTTTTGTACTCCAATTGCATCTGCCTGGTTACTTAAGGATGGGAAAGTCATTCCCATCAGTCTTTTTGA

TGATACAAGTTATACATCTAATGATGATGTTTTAGAAGATGAAGAAGACATTGTAGAAGCTGCCAGAGGA

GCCACAGAAAACAGTGTTTACTTGGGAATGTATAGAGGCCAGCTGTATCTGCAGTCATCAGTCAGAATTT

CAGAAAAGTTTCCTTCAAGTCCCAAGGCTTTGGAATCTGTCACTAATGAAAACGCAATTATTCCTTTACC

AACAATCAAATGGAAACCCTTAATTCATTCTCCTTCCAGAACTCCTGTCTTGGTAGGATCTGATGAATTT

GACAAATGTCTCAGTAATGATAAGTTTTCTCATGAAGAATATAGTAATGGTGCACTTTCAATCTTGCAGT

ATCCATATGATAATGGTTATTATCTACCATACTACAAGAGGGAGAGGAACAAACGAAGCACACAGATTAC

AGTCAGATTCCTCGACAACCCACATTACAACAAGAATATCCGCAAAAAGGATCCTGTTCTTCTTTTACAC

TGGTGGAAAGAAATAGTTGCAACGATTTTGTTTTGTATCATAGCAACAACGTTTATTGTGCGCAGGCTTT

TCCATCCTCATCCTCACAGGCAAAGGAAGGAGTCTGAAACTCAGTGTCAAACTGAAAATAAATATGATTC

TGTAAGTGGTGAAGCCAATGACAGTAGCTGGAATGACATAAAAAACTCTGGATATATATCACGATATCTA

ACTGATTTTGAGCCAATTCAATGCCTGGGACGTGGTGGCTTTGGAGTTGTTTTTGAAGCTAAAAACAAAG

TAGATGACTGCAATTATGCTATCAAGAGGATCCGTCTCCCCAATAGGGAATTGGCTCGGGAAAAGGTAAT

GCGAGAAGTTAAAGCCTTAGCCAAGCTTGAACACCCGGGCATTGTTAGATATTTCAATGCCTGGCTCGAA

GCACCACCAGAGAAGTGGCAAGAAAAGATGGATGAAATTTGGCTGAAAGATGAAAGCACAGACTGGCCAC

TCAGCTCTCCTAGCCCAATGGATGCACCATCAGTTAAAATACGCAGAATGGATCCTTTCGCTACAAAAGA

ACATATTGAAATCATAGCTCCTTCACCACAAAGAAGCAGGTCTTTTTCAGTAGGGATTTCCTGTGACCAG

ACAAGTTCATCTGAGAGCCAGTTCTCACCACTGGAATTCTCAGGAATGGACCATGAGGACATCAGTGAGT

CAGTGGATGCAGCATACAACCTCCAGGACAGTTGCCTTACAGACTGTGATGTGGAAGATGGGACTATGGA

TGGCAATGATGAGGGGCACTCCTTTGAACTTTGTCCTTCTGAAGCTTCTCCTTATGTAAGGTCAAGGGAG

AGAACCTCCTCTTCAATAGTATTTGAAGATTCTGGCTGTGATAATGCTTCCAGTAAAGAAGAGCCGAAAA

CTAATCGATTGCATATTGGCAACCATTGTGCTAATAAACTAACTGCTTTCAAGCCCACCAGTAGCAAATC

TTCTTCTGAAGCTACATTGTCTATTTCTCCTCCAAGACCAACCACTTTAAGTTTAGATCTCACTAAAAAC

ACCACAGAAAAACTCCAGCCCAGTTCACCAAAGGTGTATCTTTACATTCAAATGCAGCTGTGCAGAAAAG

AAAACCTCAAAGACTGGATGAATGGACGATGTACCATAGAGGAGAGAGAGAGGAGCGTGTGTCTGCACAT

CTTCCTGCAGATCGCAGAGGCAGTGGAGTTTCTTCACAGTAAAGGACTGATGCACAGGGACCTCAAGCCA

TCCAACATATTCTTTACAATGGATGATGTGGTCAAGGTTGGAGACTTTGGGTTAGTGACTGCAATGGACC

AGGATGAGGAAGAGCAGACGGTTCTGACCCCAATGCCAGCTTATGCCAGACACACAGGACAAGTAGGGAC

CAAACTGTATATGAGCCCAGAGCAGATTCATGGAAACAGCTATTCTCATAAAGTGGACATCTTTTCTTTA

GGCCTGATTCTATTTGAATTGCTGTATCCATTCAGCACTCAGATGGAGAGAGTCAGGACCTTAACTGATG

TAAGAAATCTCAAATTTCCACCATTATTTACTCAGAAATATCCTTGTGAGTACGTGATGGTTCAAGACAT

GCTCTCTCCATCCCCCATGGAACGACCTGAAGCTATAAACATCATTGAAAATGCTGTATTTGAGGACTTG

GACTTTCCAGGAAAAACAGTGCTCAGACAGAGGTCTCGCTCCTTGAGTTCATCGGGAACAAAACATTCAA

GACAGTCCAACAACTCCCATAGCCCTTTGCCAAGCAATTAGCCTTAAGTTGTGCTAGCAACCCTAATAGG

TGATGCAGATAATAGCCTACTTCTTAGAATATGCCTGTCCAAAATTGCAGACTTGAAAAGTTTGTTCTTC

GCTCAATTTTTTTGTGGACTACTTTTTTTATATCAAATTTAAGCTGGATTTGGGGGCATAACCTAATTTG

AGCCAACTCCTGAGTTTTGCTATACTTAAGGAAAGGGCTATCTTTGTTCTTTGTTAGTCTCTTGAAACTG

GCTGCTGGCCAAGCTTTATAGCCCTCACCATTTGCCTAAGGAGGTAGCAGCAATCCCTAATATATATATA

TAGTGAGAACTAAAATGGATATATTTTTATAATGCAGAAGAAGGAAAGTCCCCCTGTGTGGTAACTGTAT

TGTTCTAGAAATATGCTTTCTAGAGATATGATGATTTTGAAACTGATTTCTAGAAAAAGCTGACTCCATT

TTTGTCCCTGGCGGGTAAATTAGGAATCTGCACTATTTTGGAGGACAAGTAGCACAAACTGTATAACGGT

TTATGTCCGTAGTTTTATAGTCCTATTTGTAGCATTCAATAGCTTTATTCCTTAGATGGTTCTAGGGTGG

GTTTACAGCTTTTTGTACTTTTACCTCCAATAAAGGGAAAATGAAGCTTTTTATGTAAATTGGTTGAAAG

GTCTAGTTTTGGGAGGAAAAAAGCCGTAGTAAGAAATGGATCATATATATTACAACTAACTTCTTCAACT

ATGGACTTTTTAAGCCTAATGAAATCTTAAGTGTCTTATATGTAATCCTGTAGGTTGGTACTTCCCCCAA

ACTGATTATAGGTAACAGTTTAATCATCTCACTTGCTAACATGTTTTTATTTTTCACTGTAAATATGTTT

ATGTTTTATTTATAAAAATTCTGAAATCAATCCATTTGGGTTGGTGGTGTACAGAACACACTTAAGTGTG

TTAACTTGTGACTTCTTTCAAGTCTAAATGATTTAATAAAACTTTTTTTAAATTAA

>NM_004094.5 Homo sapiens eukaryotic translation initiation factor 2 subunit alpha (EIF2S1), mRNA

ATGCGCGGTGGAGTGAGCGAAGCGCACGCTGAGGAGGATCGGCGGCCGGTGAGGGGGAAGCAAGTCTGGT

CTCTGTGATTGAAGAAGTCGGCTCTGGGCTCCAGTGCGGGAATCACACACATACCTCAGAATGCCGGGTC

TAAGTTGTAGATTTTATCAACACAAATTTCCTGAGGTGGAAGATGTAGTGATGGTGAATGTCAGATCCAT

TGCTGAAATGGGGGCTTATGTCAGCTTGCTGGAATACAACAACATTGAAGGCATGATTCTTCTTAGTGAA

TTATCCAGAAGGCGTATCCGTTCTATCAACAAACTCATCCGAATTGGCAGGAATGAGTGTGTGGTTGTCA

TTAGGGTGGACAAAGAAAAAGGATATATTGATTTGTCAAAAAGAAGAGTTTCTCCAGAGGAAGCAATCAA

ATGTGAAGACAAATTCACAAAATCCAAAACTGTTTATAGCATTCTTCGTCATGTTGCTGAGGTGTTAGAA

TACACCAAGGATGAGCAGCTGGAAAGCCTATTCCAGAGGACTGCCTGGGTCTTTGATGACAAGTACAAGA

GACCTGGATATGGTGCCTATGATGCATTTAAGCATGCAGTCTCAGACCCATCTATTTTGGATAGTTTAGA

TTTGAATGAAGATGAACGGGAAGTACTCATTAATAATATTAATAGGCGCTTGACCCCACAGGCTGTCAAA

ATTCGAGCAGATATTGAAGTGGCTTGTTATGGTTATGAAGGCATTGATGCTGTAAAAGAAGCCCTAAGAG

CAGGTTTGAATTGTTCTACAGAAAACATGCCCATTAAGATTAATCTAATAGCTCCTCCTCGGTATGTAAT

GACTACGACAACCCTGGAGAGAACAGAAGGCCTTTCTGTCCTCAGTCAAGCTATGGCTGTTATCAAAGAG

AAGATTGAGGAAAAGAGGGGTGTGTTCAATGTTCAAATGGAGCCCAAAGTGGTCACAGATACAGATGAGA

CTGAACTTGCGAGGCAGATGGAGAGGCTTGAAAGAGAAAATGCCGAAGTGGATGGAGATGATGATGCAGA

AGAAATGGAAGCCAAAGCTGAAGATTAACTTTGTGGGAAACAGAGTCCAATTTAAGGAACACAGAGCAGC

GCTTCCTGGCTGTAAATCCTAGACTTGAAAGTTTTCCAGTATTGAAAACTTCAAAGCTGAATATTTTTTA

TTTCTAAGTATTTAAATGTTCTAACAGATCAGAACATGAAATGCCCTCCTAAATGTCAGCTGTTGTCACA

CAGTAGCTCCAACACTTTGAGCATTTTTAAGGGAGTGGCCTCATTTCACTAGAGACAAATCTTTAAGAAT

AGTTCTAAAATTGGGCTTGTGATTTCCATTTCTGATGTCTCCAGATTGGCACCCCTTTCTAGTTCAATGC

CTCACGAGATTTGCCAGGGGCATCCAAGGCAAACAATCCCAATCTTTCTATATAAAATGTATTCAAGCAA

ACATCAAATAAATTTCTGGGATATTTAACTATAGGCTTCTTCCTTCTTGTCACCAGTTAAAAGCATTTTA

ATACTAAGACCCTAATTCTTTTATCTTTATTTTAGTCTTGATGTGGAACTGTAGGAGCAGGTGAATAAAG

GATCTCTATAACAGATCCTTTCAAAAGAAGAGTTTTAGAGAAAATAAATTTAACTTTAACCACAGTGAAA

GTTGACCCTTAGCGGGACAAAGCCTTAAAATGCATTGAAAGAATTAGATCGGTTCTGTGCCTTTTATCTA

TTTGAGATTGATGACAACCTGTGTGAGAGAATTTATCACACCACGTCCTTATTGGAATAATAAGCTACTT

GCCTTGAGTTTATAATTCAGGGTGGTAAAGTATGTTTTTAAATTTTAAAAAGCAGCTGCATTTTTTATTT

AGTTGGAATATCACCCAATTTTTTATTTTTATTGCTATTAAAATATCCACTAGATGCCACCTAGAGCTCC

AGTTCTTTATAACAAAACAGGGATCTGTTTGAACACTTACTGTTGTTTTTTTTTTTTTACATGTTTCCAT

CATTTCTGTCTTTAAGAACTAATTCGTACATAATAAGTTTCATAGGTAACACATTATTTGAAGTTACAGC

TACAGTAGTCAGGTGTAGAAAATCTTGAGTTGCTTTGGTCTGCTTGTCTTCATAAATTTATTCTCTTATG

ATACTTGAGGTACCAGTTGTATTTAATTTTATTCATTATCCCTAGATAGCTATTAAGATACTTAGATTAG

ACCTAACCCACCATAGTCAATCCAAGACTAGACTACTCAATATTAAAGGGTCTGGAAAATAGAAGAGTGT

GTTGGGCAGGTAGTTTGTACCATTTATGAAGGTTTGTTCCTTTGTTAAATTTAGCAGCCTGTACTAGCTT

TTGAAATCCAGAAGTTTTAACTTCCAGTGGCTGGTTTCTGAGAGAGTGCCATGATTGCTAGCCAGCATTC

CATATTGGGAATATGTAGAGGAGAACCTGGATGTACTTAAGAGTGGCATATAATTTTCACTTCTGTCTGT

TGAGGCATCAAAAAAACAAGTTTAGAAAGCTGGCAACATGAAGAATGCATTCAAAATATAACAGGTGCTT

CTTTGTTGTACGCAGAGGAATTTTTTCTTTTGATTTTGTTTACTGAAATTTGTTATACTTCAAAAGCCAT

AACTTGAAAAATACTGGTGGCGTCGATGGTGAGTGATTTTTATCCCACGTGGGCCTTTTGCTCAGTTCCA

TGGCAATTTTGTAAATTGACCCTAGCCAGAGAATAGATCAGTATTTCACTGATACCACGAGGAAAAGAAC

AAACAAAACTTAAAAGTATTCATACACCTTGCTAACCTAAAAGACAGCAGGGACAAGATACATATGAGGA

CAAGGTATACACCCAGTTCTGAATAACTTGAAGAACAGGCTTGGCAAAGAAGTAAGTTTATCCAAATCTT

GAATTTCTGCCAGGCATGGTAGCTCATGCCTGTAACCCTGGCACTTAAGGGGCCAAGGCAGGAGGATCAC

TTGAGGCCAGTGAGCTGTGATCACTCCAGTGCACTCGAGCCTGGGTGACAGGGCAAGACCCTGTCTCAAA

AAAAAAAAAAAAAACCAAAAATCTTGAATCTCCCATCAAAGCCTTTTCATTAAAAATACAATTTCATCTA

CTCCCTACTGTACCTTTCACTGATGTCAAGTGGCTATCAATTCATATAGCTGAAAATTAGTGATAGTTTG

GGTCTCATTTATGCATAAATTAATTGAAAATCAGTAATGGCCAGGAAGAAATATGAATATTCAAACCACT

TTTTTCTTTTTTTTTTTTTTTTTTTTGGCAAATGGTGTTATATTGCCTGGGCAGGTCTCGAAATCCTGGGC

TCAAGCTATCCTCCTGCCTCTGCCTCTGTAAGAGTGTGAGCCACCATGCCCCACCCAAACCATTTTTCAT

ATCAGTTATAGCAAAACAAGACAAACCAGTTCTTAATAATGTTCAAAGTTGGACCCATAGAGACATGCTA

AGCACTACATCATGCTAAGAAGAACAGTTATTACTCAGCTGCCATACTTCAGCCATTCTACTTTTACACC

AAGAACAAAATGGCTAGAGATTTTTTTAAAAATACAAATGCCAGTACCTTGCCGAGACTTGTTCACTGAT

TTGCCCCAACCCCAAGGTAATGGTCATCTACTTTGCAACCTATGGAGATCCTGATAGCTCCCATACAAAG

TGAGGTACACTGTTGCCAATTTGGAGGCAGGAAGCCATCAAACTAAACGTAGTTTAATTCAGATGAACAC

CTATCGATATTCAAAAGGCAAAACAAAAAAACTAATTCCAGGGACATTAGACCTTGGTGAAATGCCAAAT

GGAAAATGGTGCTTTAAAAGGTAGTCTCTTACCATATAAGGAATAAGTAAAACATTAGCTTGTGATTTCT

CATTATTCAGGTCAATGTTTTAGACACGACTAACAAGGGACAGTTCAAACTATTAAAGAGAACAAATTTT

TACTTAAGCACTTTGAGGTATCCACTCCCCTTAAATGTAAGTTATGTATTTTATCTGTTGATTAAGGGGA

GGTCCAAGCATTTGTACTTGGCAGGAATGCTGGCAATAGCTTGTGCCAGATTACTGTATAAACTTGAAAG

TAATAAAAATGTTCTTTAAAAGTA

>NM_004095.4 Homo sapiens eukaryotic translation initiation factor 4E binding protein 1 (EIF4EBP1), mRNA

AGGGCAGCGAGAGGTTCGCGGGTGCAGCGCACAGGAGACCATGTCCGGGGGCAGCAGCTGCAGCCAGACC

CCAAGCCGGGCCATCCCCGCCACTCGCCGGGTGGTGCTCGGCGACGGCGTGCAGCTCCCGCCCGGGGACT

ACAGCACGACCCCCGGCGGCACGCTCTTCAGCACCACCCCGGGAGGTACCAGGATCATCTATGACCGGAA

ATTCCTGATGGAGTGTCGGAACTCACCTGTGACCAAAACACCCCCAAGGGATCTGCCCACCATTCCGGGG

GTCACCAGCCCTTCCAGTGATGAGCCCCCCATGGAAGCCAGCCAGAGCCACCTGCGCAATAGCCCAGAAG

ATAAGCGGGCGGGCGGTGAAGAGTCACAGTTTGAGATGGACATTTAAAGCACCAGCCATCGTGTGGAGCA

CTACCAAGGGGCCCCTCAGGGCCTTCCTGGGAGGAGTCCCACCAGCCAGGCCTTATGAAAGTGATCATAC

TGGGCAGGCGTTGGCGTGGGGTCGGACACCCCAGCCCTTTCTCCCTCACTCAGGGCACCTGCCCCCTCCT

CTTCGTGAACACCAGCAGATACCTCCTTGTGCCTCCACTGATGCAGGAGCTGCCACCCCAAGGGGAGTGA

CCCCTGCCAGCACACCCTGCAGCCAAGGGCCAGGAAGTGGACAAGAACGAACCCTTCCTTCCGAATGATC

AGCAGTTCCAGCCCCTCGCTGCTGGGGGCGCAACCACCCCTTCCTTAGGTTGATGTGCTTGGGAAAGCTC

CCTCCCCCTCCTTCCCCAAGAGAGGAAATAAAAGCCACCTTCGCCCTAGGGCCAAGA

>NM_182917.4 Homo sapiens eukaryotic translation initiation factor 4 gamma 1 (EIF4G1), transcript variant 1, mRNA

TCACTTGCCTGAAACCGGCTCCTCGACGGCCGCCGCCCGCCTGGCCTTTTAGGGCCTGACTCCCGCCCTT

CCTGGCCTACACTCCTGGGCGGCGGCAGGCCTAGCTTCTGGCCCAGTGCGGGTTCCCCGGCGGCAGGCGT

ATCCTGTGTGCCCCTGGGCCAGGCCCGAACCCGGTGTCCCCGGGTGGGGGGTGGGGACGCCACGGCCGAA

GCAGCTAGCTCCGTTCGTGATCCGGGAGCCTGGTGCCAGCGAGACCTGGAATTTCCGGTCTGGTTGGTCT

GGGGCCCCGCGGAGCCAGGTTGATACCCTCACCTCCCAACCCCAGGCCCTCGGATGCCCAGAACCTGTAG

GCCGCACCGTGGACTTGTTCTTAATCGAGGGGGTGCTGGGGGGACCCTGATGTGGCACCAAATGAAATGA

ACAAAGCTCCACAGTCCACAGGCCCCCCACCCGCCCCATCCCCCGGACTCCCACAGCCAGCGTTTCCCCC

GGGGCAGACAGCGCCGGTGGTGTTCAGTACGCCACAAGCGACACAAATGAACACGCCTTCTCAGCCCCGC

CAGCACTTCTACCCTAGCCGGGCCCAGCCCCCGAGCAGTGCAGCCTCCCGAGTGCAGAGTGCAGCCCCTG

CCCGCCCTGGCCCAGCTGCCCATGTCTACCCTGCTGGATCCCAAGTAATGATGATCCCTTCCCAGATCTC

CTACCCAGCCTCCCAGGGGGCCTACTACATCCCTGGACAGGGGCGTTCCACATACGTTGTCCCGACACAG

CAGTACCCTGTGCAGCCAGGAGCCCCAGGCTTCTATCCAGGTGCAAGCCCTACAGAATTTGGGACCTACG

CTGGCGCCTACTATCCAGCCCAAGGGGTGCAGCAGTTTCCCACTGGCGTGGCCCCCGCCCCAGTTTTGAT

GAACCAGCCACCCCAGATTGCTCCCAAGAGGGAGCGTAAGACGATCCGAATTCGAGATCCAAACCAAGGA

GGAAAGGATATCACAGAGGAGATCATGTCTGGGGCCCGCACTGCCTCCACACCCACCCCTCCCCAGACGG

GAGGCGGTCTGGAGCCTCAAGCTAATGGGGAGACGCCCCAGGTTGCTGTCATTGTCCGGCCAGATGACCG

GTCACAGGGAGCAATCATTGCTGACCGGCCAGGGCTGCCTGGCCCAGAGCATAGCCCTTCAGAATCCCAG

CCTTCGTCGCCTTCTCCGACCCCATCACCATCCCCAGTCTTGGAACCGGGGTCTGAGCCTAATCTCGCAG

TCCTCTCTATTCCTGGGGACACTATGACAACTATACAAATGTCTGTAGAAGAATCAACCCCCATCTCCCG

TGAAACTGGGGAGCCATATCGCCTCTCTCCAGAACCCACTCCTCTCGCCGAACCCATACTGGAAGTAGAA

GTGACACTTAGCAAACCGGTTCCAGAATCTGAGTTTTCTTCCAGTCCTCTCCAGGCTCCCACCCCTTTGG

CATCTCACACAGTGGAAATTCATGAGCCTAATGGCATGGTCCCATCTGAAGATCTGGAACCAGAGGTGGA

GTCAAGCCCAGAGCTTGCTCCTCCCCCAGCTTGCCCCTCCGAATCCCCTGTGCCCATTGCTCCAACTGCC

CAACCTGAGGAACTGCTCAACGGAGCCCCCTCGCCACCAGCTGTGGACTTAAGCCCAGTCAGTGAGCCAG

AGGAGCAGGCCAAGGAGGTGACAGCATCAATGGCGCCCCCCACCATCCCCTCTGCTACTCCAGCTACGGC

TCCTTCAGCTACTTCCCCAGCTCAGGAGGAGGAAATGGAAGAAGAAGAAGAAGAGGAAGAAGGAGAAGCA

GGAGAAGCAGGAGAAGCTGAGAGTGAGAAAGGAGGAGAGGAACTGCTCCCCCCAGAGAGTACCCCTATTC

CAGCCAACTTGTCTCAGAATTTGGAGGCAGCAGCAGCCACTCAAGTGGCAGTATCTGTGCCAAAGAGGAG

ACGGAAAATTAAGGAGCTAAATAAGAAGGAGGCTGTTGGAGACCTTCTGGATGCCTTCAAGGAGGCGAAC

CCGGCAGTACCAGAGGTGGAAAATCAGCCTCCTGCAGGCAGCAATCCAGGCCCAGAGTCTGAGGGCAGTG

GTGTGCCCCCACGTCCTGAGGAAGCAGATGAGACCTGGGACTCAAAGGAAGACAAAATTCACAATGCTGA

GAACATCCAGCCCGGGGAACAGAAGTATGAATATAAGTCAGATCAGTGGAAGCCTCTAAACCTAGAGGAG

AAAAAACGTTACGACCGTGAGTTCCTGCTTGGTTTTCAGTTCATCTTTGCCAGTATGCAGAAGCCAGAGG

GATTGCCACATATCAGTGACGTGGTGCTGGACAAGGCCAATAAAACACCACTGCGGCCACTGGATCCCAC

TAGACTACAAGGCATAAATTGTGGCCCAGACTTCACTCCATCCTTTGCCAACCTTGGCCGGACAACCCTT

AGCACCCGTGGGCCCCCAAGGGGTGGGCCAGGTGGGGAGCTGCCCCGTGGGCCGCAGGCTGGCCTGGGAC

CCCGGCGCTCTCAGCAGGGACCCCGAAAAGAACCACGCAAGATCATTGCCACAGTGTTAATGACCGAAGA

TATAAAACTGAACAAAGCAGAGAAAGCCTGGAAACCCAGCAGCAAGCGGACGGCGGCTGATAAGGATCGA

GGGGAAGAAGATGCTGATGGCAGCAAAACCCAGGACCTATTCCGCAGGGTGCGCTCCATCCTGAATAAAC

TGACACCCCAGATGTTCCAGCAGCTGATGAAGCAAGTGACGCAGCTGGCCATCGACACCGAGGAACGCCT

CAAAGGGGTCATTGACCTCATTTTTGAGAAGGCCATTTCAGAGCCCAACTTCTCTGTGGCCTATGCCAAC

ATGTGCCGCTGCCTCATGGCGCTGAAAGTGCCCACTACGGAAAAGCCAACAGTGACTGTGAACTTCCGAA

AGCTGTTGTTGAATCGATGTCAGAAGGAGTTTGAGAAAGACAAAGATGATGATGAGGTTTTTGAGAAGAA

GCAAAAAGAGATGGATGAAGCTGCTACGGCAGAGGAACGAGGACGCCTGAAGGAAGAGCTGGAAGAGGCT

CGGGACATAGCCCGGCGGCGCTCTTTAGGGAATATCAAGTTTATTGGAGAGTTGTTCAAACTGAAGATGT

TAACAGAGGCAATAATGCATGACTGTGTGGTCAAACTGCTTAAGAACCATGATGAAGAGTCCCTTGAGTG

CCTTTGTCGTCTGCTCACCACCATTGGCAAAGACCTGGACTTTGAAAAAGCCAAGCCCCGAATGGATCAG

TATTTCAACCAGATGGAAAAAATCATTAAAGAAAAGAAGACGTCATCCCGCATCCGCTTTATGCTGCAGG

ACGTGCTGGATCTGCGAGGGAGCAATTGGGTGCCACGCCGAGGGGATCAGGGTCCCAAGACCATTGACCA

GATCCATAAGGAGGCTGAGATGGAAGAACATCGAGAGCACATCAAAGTGCAGCAGCTCATGGCCAAGGGC

AGTGACAAGCGTCGGGGCGGTCCTCCAGGCCCTCCCATCAGCCGTGGACTTCCCCTTGTGGATGATGGTG

GCTGGAACACAGTTCCCATCAGCAAAGGTAGCCGCCCCATTGACACCTCACGACTCACCAAGATCACCAA

GCCTGGCTCCATCGATTCTAACAACCAGCTCTTTGCACCTGGAGGGCGACTGAGCTGGGGCAAGGGCAGC

AGCGGAGGCTCAGGAGCCAAGCCCTCAGACGCAGCATCAGAAGCTGCTCGCCCAGCTACTAGTACTTTGA

ATCGCTTCTCAGCCCTTCAACAAGCGGTACCCACAGAAAGCACAGATAATAGACGTGTGGTGCAGAGGAG

TAGCTTGAGCCGAGAACGAGGCGAGAAAGCTGGAGACCGAGGAGACCGCCTAGAGCGGAGTGAACGGGGA

GGGGACCGTGGGGACCGGCTTGATCGTGCGCGGACACCTGCTACCAAGCGGAGCTTCAGCAAGGAAGTGG

AGGAGCGGAGTAGAGAACGGCCCTCCCAGCCTGAGGGGCTGCGCAAGGCAGCTAGCCTCACGGAGGATCG

GGACCGTGGGCGGGATGCCGTGAAGCGAGAAGCTGCCCTACCCCCAGTGAGCCCCCTGAAGGCGGCTCTC

TCTGAGGAGGAGTTAGAGAAGAAATCCAAGGCTATCATTGAGGAATATCTCCATCTCAATGACATGAAAG

AGGCAGTCCAGTGCGTGCAGGAGCTGGCCTCACCCTCCTTGCTCTTCATCTTTGTACGGCATGGTGTCGA

GTCTACGCTGGAGCGCAGTGCCATTGCTCGTGAGCATATGGGGCAGCTGCTGCACCAGCTGCTCTGTGCT

GGGCATCTGTCTACTGCTCAGTACTACCAAGGGTTGTATGAAATCTTGGAATTGGCTGAGGACATGGAAA

TTGACATCCCCCACGTGTGGCTCTACCTAGCGGAACTGGTAACACCCATTCTGCAGGAAGGTGGGGTGCC

CATGGGGGAGCTGTTCAGGGAGATTACAAAGCCTCTGAGACCGTTGGGCAAAGCTGCTTCCCTGTTGCTG

GAGATCCTGGGCCTCCTGTGCAAAAGCATGGGTCCTAAAAAGGTGGGGACGCTGTGGCGAGAAGCCGGGC

TTAGCTGGAAGGAATTTCTACCTGAAGGCCAGGACATTGGTGCATTCGTCGCTGAACAGAAGGTGGAGTA

TACCCTGGGAGAGGAGTCGGAAGCCCCTGGCCAGAGGGCACTCCCCTCCGAGGAGCTGAACAGGCAGCTG

GAGAAGCTGCTGAAGGAGGGCAGCAGTAACCAGCGGGTGTTCGACTGGATAGAGGCCAACCTGAGTGAGC

AGCAGATAGTATCCAACACGTTAGTTCGAGCCCTCATGACGGCTGTCTGCTATTCTGCAATTATTTTTGA

GACTCCCCTCCGAGTGGACGTTGCAGTGCTGAAAGCGCGAGCGAAGCTGCTGCAGAAATACCTGTGTGAC

GAGCAGAAGGAGCTACAGGCGCTCTACGCCCTCCAGGCCCTTGTAGTGACCTTAGAACAGCCTCCCAACC

TGCTGCGGATGTTCTTTGACGCACTGTATGACGAGGACGTGGTGAAGGAGGATGCCTTCTACAGTTGGGA

GAGTAGCAAGGACCCCGCTGAGCAGCAGGGCAAGGGTGTGGCCCTTAAATCTGTCACAGCCTTCTTCAAG

TGGCTCCGTGAAGCAGAGGAGGAGTCTGACCACAACTGAGGGCTGGTGGGGCCGGGGACCTGGAGCCCCA

TGGACACACAGATGGCCCGGCTAGCCGCCTGGACTGCAGGGGGGCGGCAGCAGCGGCGGTGGCAGTGGGT

GCCTGTAGTGTGATGTGTCTGAACTAATAAAGTGGCTGAAGAGGCAGGATGGCTTGGGGCTGCCTGGGCC

CCCCTCCAGGATGCCGCCAGGTGTCCCTCTCCTCCCCCTGGGGCACAGAGATATATTATATATAAAGTCT

TGAAATTTGGTGTGTCTTGGGGTGGGGAGGGGCACCAACGCCTGCCCCTGGGGTCCTTTTTTTTATTTTC

TGAAAATCACTCTCGGGACTGCCGTCCTCGCTGCTGGGGGCATATGCCCCAGCCCCTGTACCACCCCTGC

TGTTGCCTGGGCAGGGGGAAGGGGGGGCACGGTGCCTGTAATTATTAAACATGAATTCAATTAAGCTCAA

AAAAAAAAAAAAAAAA

>NM_004448.4 Homo sapiens erb-b2 receptor tyrosine kinase 2 (ERBB2), transcript variant 1, mRNA

CCCCTCCATTGGGACCGGAGAAACCAGGGGAGCCCCCCGGGCAGCCGCGCGCCCCTTCCCACGGGGCCCT

TTACTGCGCCGCGCGCCCGGCCCCCACCCCTCGCAGCACCCCGCGCCCCGCGCCCTCCCAGCCGGGTCCA

GCCGGAGCCATGGGGCCGGAGCCGCAGTGAGCACCATGGAGCTGGCGGCCTTGTGCCGCTGGGGGCTCCT

CCTCGCCCTCTTGCCCCCCGGAGCCGCGAGCACCCAAGTGTGCACCGGCACAGACATGAAGCTGCGGCTC

CCTGCCAGTCCCGAGACCCACCTGGACATGCTCCGCCACCTCTACCAGGGCTGCCAGGTGGTGCAGGGAA

ACCTGGAACTCACCTACCTGCCCACCAATGCCAGCCTGTCCTTCCTGCAGGATATCCAGGAGGTGCAGGG

CTACGTGCTCATCGCTCACAACCAAGTGAGGCAGGTCCCACTGCAGAGGCTGCGGATTGTGCGAGGCACC

CAGCTCTTTGAGGACAACTATGCCCTGGCCGTGCTAGACAATGGAGACCCGCTGAACAATACCACCCCTG

TCACAGGGGCCTCCCCAGGAGGCCTGCGGGAGCTGCAGCTTCGAAGCCTCACAGAGATCTTGAAAGGAGG

GGTCTTGATCCAGCGGAACCCCCAGCTCTGCTACCAGGACACGATTTTGTGGAAGGACATCTTCCACAAG

AACAACCAGCTGGCTCTCACACTGATAGACACCAACCGCTCTCGGGCCTGCCACCCCTGTTCTCCGATGT

GTAAGGGCTCCCGCTGCTGGGGAGAGAGTTCTGAGGATTGTCAGAGCCTGACGCGCACTGTCTGTGCCGG

TGGCTGTGCCCGCTGCAAGGGGCCACTGCCCACTGACTGCTGCCATGAGCAGTGTGCTGCCGGCTGCACG

GGCCCCAAGCACTCTGACTGCCTGGCCTGCCTCCACTTCAACCACAGTGGCATCTGTGAGCTGCACTGCC

CAGCCCTGGTCACCTACAACACAGACACGTTTGAGTCCATGCCCAATCCCGAGGGCCGGTATACATTCGG

CGCCAGCTGTGTGACTGCCTGTCCCTACAACTACCTTTCTACGGACGTGGGATCCTGCACCCTCGTCTGC

CCCCTGCACAACCAAGAGGTGACAGCAGAGGATGGAACACAGCGGTGTGAGAAGTGCAGCAAGCCCTGTG

CCCGAGTGTGCTATGGTCTGGGCATGGAGCACTTGCGAGAGGTGAGGGCAGTTACCAGTGCCAATATCCA

GGAGTTTGCTGGCTGCAAGAAGATCTTTGGGAGCCTGGCATTTCTGCCGGAGAGCTTTGATGGGGACCCA

GCCTCCAACACTGCCCCGCTCCAGCCAGAGCAGCTCCAAGTGTTTGAGACTCTGGAAGAGATCACAGGTT

ACCTATACATCTCAGCATGGCCGGACAGCCTGCCTGACCTCAGCGTCTTCCAGAACCTGCAAGTAATCCG

GGGACGAATTCTGCACAATGGCGCCTACTCGCTGACCCTGCAAGGGCTGGGCATCAGCTGGCTGGGGCTG

CGCTCACTGAGGGAACTGGGCAGTGGACTGGCCCTCATCCACCATAACACCCACCTCTGCTTCGTGCACA

CGGTGCCCTGGGACCAGCTCTTTCGGAACCCGCACCAAGCTCTGCTCCACACTGCCAACCGGCCAGAGGA

CGAGTGTGTGGGCGAGGGCCTGGCCTGCCACCAGCTGTGCGCCCGAGGGCACTGCTGGGGTCCAGGGCCC

ACCCAGTGTGTCAACTGCAGCCAGTTCCTTCGGGGCCAGGAGTGCGTGGAGGAATGCCGAGTACTGCAGG

GGCTCCCCAGGGAGTATGTGAATGCCAGGCACTGTTTGCCGTGCCACCCTGAGTGTCAGCCCCAGAATGG

CTCAGTGACCTGTTTTGGACCGGAGGCTGACCAGTGTGTGGCCTGTGCCCACTATAAGGACCCTCCCTTC

TGCGTGGCCCGCTGCCCCAGCGGTGTGAAACCTGACCTCTCCTACATGCCCATCTGGAAGTTTCCAGATG

AGGAGGGCGCATGCCAGCCTTGCCCCATCAACTGCACCCACTCCTGTGTGGACCTGGATGACAAGGGCTG

CCCCGCCGAGCAGAGAGCCAGCCCTCTGACGTCCATCATCTCTGCGGTGGTTGGCATTCTGCTGGTCGTG

GTCTTGGGGGTGGTCTTTGGGATCCTCATCAAGCGACGGCAGCAGAAGATCCGGAAGTACACGATGCGGA

GACTGCTGCAGGAAACGGAGCTGGTGGAGCCGCTGACACCTAGCGGAGCGATGCCCAACCAGGCGCAGAT

GCGGATCCTGAAAGAGACGGAGCTGAGGAAGGTGAAGGTGCTTGGATCTGGCGCTTTTGGCACAGTCTAC

AAGGGCATCTGGATCCCTGATGGGGAGAATGTGAAAATTCCAGTGGCCATCAAAGTGTTGAGGGAAAACA

CATCCCCCAAAGCCAACAAAGAAATCTTAGACGAAGCATACGTGATGGCTGGTGTGGGCTCCCCATATGT

CTCCCGCCTTCTGGGCATCTGCCTGACATCCACGGTGCAGCTGGTGACACAGCTTATGCCCTATGGCTGC

CTCTTAGACCATGTCCGGGAAAACCGCGGACGCCTGGGCTCCCAGGACCTGCTGAACTGGTGTATGCAGA

TTGCCAAGGGGATGAGCTACCTGGAGGATGTGCGGCTCGTACACAGGGACTTGGCCGCTCGGAACGTGCT

GGTCAAGAGTCCCAACCATGTCAAAATTACAGACTTCGGGCTGGCTCGGCTGCTGGACATTGACGAGACA

GAGTACCATGCAGATGGGGGCAAGGTGCCCATCAAGTGGATGGCGCTGGAGTCCATTCTCCGCCGGCGGT

TCACCCACCAGAGTGATGTGTGGAGTTATGGTGTGACTGTGTGGGAGCTGATGACTTTTGGGGCCAAACC

TTACGATGGGATCCCAGCCCGGGAGATCCCTGACCTGCTGGAAAAGGGGGAGCGGCTGCCCCAGCCCCCC

ATCTGCACCATTGATGTCTACATGATCATGGTCAAATGTTGGATGATTGACTCTGAATGTCGGCCAAGAT

TCCGGGAGTTGGTGTCTGAATTCTCCCGCATGGCCAGGGACCCCCAGCGCTTTGTGGTCATCCAGAATGA

GGACTTGGGCCCAGCCAGTCCCTTGGACAGCACCTTCTACCGCTCACTGCTGGAGGACGATGACATGGGG

GACCTGGTGGATGCTGAGGAGTATCTGGTACCCCAGCAGGGCTTCTTCTGTCCAGACCCTGCCCCGGGCG

CTGGGGGCATGGTCCACCACAGGCACCGCAGCTCATCTACCAGGAGTGGCGGTGGGGACCTGACACTAGG

GCTGGAGCCCTCTGAAGAGGAGGCCCCCAGGTCTCCACTGGCACCCTCCGAAGGGGCTGGCTCCGATGTA

TTTGATGGTGACCTGGGAATGGGGGCAGCCAAGGGGCTGCAAAGCCTCCCCACACATGACCCCAGCCCTC

TACAGCGGTACAGTGAGGACCCCACAGTACCCCTGCCCTCTGAGACTGATGGCTACGTTGCCCCCCTGAC

CTGCAGCCCCCAGCCTGAATATGTGAACCAGCCAGATGTTCGGCCCCAGCCCCCTTCGCCCCGAGAGGGC

CCTCTGCCTGCTGCCCGACCTGCTGGTGCCACTCTGGAAAGGCCCAAGACTCTCTCCCCAGGGAAGAATG

GGGTCGTCAAAGACGTTTTTGCCTTTGGGGGTGCCGTGGAGAACCCCGAGTACTTGACACCCCAGGGAGG

AGCTGCCCCTCAGCCCCACCCTCCTCCTGCCTTCAGCCCAGCCTTCGACAACCTCTATTACTGGGACCAG

GACCCACCAGAGCGGGGGGCTCCACCCAGCACCTTCAAAGGGACACCTACGGCAGAGAACCCAGAGTACC

TGGGTCTGGACGTGCCAGTGTGAACCAGAAGGCCAAGTCCGCAGAAGCCCTGATGTGTCCTCAGGGAGCA

GGGAAGGCCTGACTTCTGCTGGCATCAAGAGGTGGGAGGGCCCTCCGACCACTTCCAGGGGAACCTGCCA

TGCCAGGAACCTGTCCTAAGGAACCTTCCTTCCTGCTTGAGTTCCCAGATGGCTGGAAGGGGTCCAGCCT

CGTTGGAAGAGGAACAGCACTGGGGAGTCTTTGTGGATTCTGAGGCCCTGCCCAATGAGACTCTAGGGTC

CAGTGGATGCCACAGCCCAGCTTGGCCCTTTCCTTCCAGATCCTGGGTACTGAAAGCCTTAGGGAAGCTG

GCCTGAGAGGGGAAGCGGCCCTAAGGGAGTGTCTAAGAACAAAAGCGACCCATTCAGAGACTGTCCCTGA

AACCTAGTACTGCCCCCCATGAGGAAGGAACAGCAATGGTGTCAGTATCCAGGCTTTGTACAGAGTGCTT

TTCTGTTTAGTTTTTACTTTTTTTGTTTTGTTTTTTTAAAGATGAAATAAAGACCCAGGGGGAGAATGGG

TGTTGTATGGGGAGGCAAGTGTGGGGGGTCCTTCTCCACACCCACTTTGTCCATTTGCAAATATATTTTG

GAAAACA

>NM_001433.5 Homo sapiens endoplasmic reticulum to nucleus signaling 1 (ERN1), mRNA

GCTGCCTAGTCAGTTCTGCGTCCGCTGAGGCTCGGTCACCGCCTCGCTGTCGTCGCGGCGCCCCCGCCCC

GTCCTCTGTCCGTACCGCCCCCGGAGCCAGGGCCGAGTCCTCGCCATGCCGGCCCGGCGGCTGCTGCTGC

TGCTGACGCTGCTGCTGCCCGGCCTCGGGATTTTTGGAAGTACCAGCACAGTGACGCTTCCTGAAACCTT

GTTGTTTGTGTCAACGCTGGATGGAAGTTTGCATGCTGTCAGCAAGAGGACAGGCTCAATCAAATGGACT

TTAAAAGAAGATCCAGTCCTGCAGGTCCCAACACATGTGGAAGAGCCTGCCTTTCTCCCAGATCCTAATG

ATGGCAGCCTGTATACGCTTGGAAGCAAGAATAATGAAGGCCTGACGAAACTTCCTTTTACCATCCCAGA

ATTGGTGCAGGCATCCCCATGCCGAAGTTCAGATGGAATCCTCTACATGGGTAAAAAGCAGGACATCTGG

TATGTTATTGACCTCCTGACCGGAGAGAAGCAGCAGACTTTGTCATCGGCCTTTGCAGATAGTCTCTGCC

CATCAACCTCTCTTCTGTATCTTGGGCGAACAGAATACACCATCACCATGTACGACACCAAAACCCGAGA

GCTCCGGTGGAATGCCACCTACTTTGACTATGCGGCCTCACTGCCTGAGGACGACGTGGACTACAAGATG

TCCCACTTTGTGTCCAATGGTGATGGGCTGGTGGTGACTGTGGACAGTGAATCTGGGGACGTCCTGTGGA

TCCAAAACTACGCCTCCCCTGTGGTGGCCTTTTATGTCTGGCAGCGGGAGGGTCTGAGGAAGGTGATGCA

CATCAATGTCGCTGTGGAGACCCTGCGCTATCTGACCTTCATGTCTGGGGAGGTGGGGCGCATCACAAAG

TGGAAGTACCCGTTCCCCAAGGAGACAGAGGCCAAGAGCAAGCTGACGCCCACTCTGTATGTTGGGAAAT

ACTCTACCAGCCTCTATGCCTCTCCCTCAATGGTACACGAGGGGGTTGCTGTCGTGCCCCGCGGCAGCAC

ACTTCCTTTGCTGGAAGGGCCCCAGACTGATGGCGTCACCATTGGGGACAAGGGGGAGTGTGTGATCACG

CCCAGCACGGACGTCAAGTTTGATCCCGGACTCAAAAGCAAGAACAAGCTCAACTACTTGAGGAATTACT

GGCTTCTGATAGGACACCATGAAACCCCACTGTCTGCGTCTACCAAGATGCTGGAGAGATTTCCCAACAA

TCTACCCAAACATCGGGAAAATGTGATTCCTGCTGATTCAGAGAAAAAGAGCTTTGAGGAAGTTATCAAC

CTGGTTGACCAGACTTCAGAAAACGCACCTACCACCGTGTCTCGGGATGTGGAGGAGAAGCCCGCCCATG

CCCCTGCCCGGCCCGAGGCCCCCGTGGACTCCATGCTTAAGGACATGGCTACCATCATCCTGAGCACCTT

CCTGCTGATTGGCTGGGTGGCCTTCATCATCACCTATCCCCTGAGCATGCATCAGCAGCAGCAGCTCCAG

CACCAGCAGTTCCAGAAGGAACTGGAGAAGATCCAGCTCCTGCAGCAGCAGCAGCAGCAGCTGCCCTTCC

ACCCACCTGGAGACACGGCTCAGGACGGCGAGCTCCTGGACACGTCTGGCCCGTACTCAGAGAGCTCGGG

CACCAGCAGCCCCAGCACGTCCCCCAGGGCCTCCAACCACTCGCTCTGCTCCGGCAGCTCTGCCTCCAAG

GCTGGCAGCAGCCCCTCCCTGGAACAAGACGATGGAGATGAGGAAACCAGCGTGGTGATAGTTGGGAAAA

TTTCCTTCTGTCCCAAGGATGTCCTGGGCCATGGAGCTGAGGGCACAATTGTGTACCGGGGCATGTTTGA

CAACCGCGACGTGGCCGTGAAGAGGATCCTCCCCGAGTGTTTTAGCTTCGCAGACCGTGAGGTCCAGCTG

TTGCGAGAATCGGATGAGCACCCGAACGTGATCCGCTACTTCTGCACGGAGAAGGACCGGCAATTCCAGT

ACATTGCCATCGAGCTGTGTGCAGCCACCCTGCAAGAGTATGTGGAGCAGAAGGACTTTGCGCATCTCGG

CCTGGAGCCCATCACCTTGCTGCAGCAGACCACCTCGGGCCTGGCCCACCTCCACTCCCTCAACATCGTT

CACAGAGACCTAAAGCCACACAACATCCTCATATCCATGCCCAATGCACACGGCAAGATCAAGGCCATGA

TCTCCGACTTTGGCCTCTGCAAGAAGCTGGCAGTGGGCAGACACAGTTTCAGCCGCCGATCTGGGGTGCC

TGGCACAGAAGGCTGGATCGCTCCAGAGATGCTGAGCGAAGACTGTAAGGAGAACCCTACCTACACGGTG

GACATCTTTTCTGCAGGCTGCGTCTTTTACTACGTAATCTCTGAGGGCAGCCACCCTTTTGGCAAGTCCC

TGCAGCGGCAGGCCAACATCCTCCTGGGTGCCTGCAGCCTTGACTGCTTGCACCCAGAGAAGCACGAAGA

CGTCATTGCACGTGAATTGATAGAGAAGATGATTGCGATGGATCCTCAGAAACGCCCCTCAGCGAAGCAT

GTGCTCAAACACCCGTTCTTCTGGAGCCTAGAGAAGCAGCTCCAGTTCTTCCAGGACGTGAGCGACAGAA

TAGAAAAGGAATCCCTGGATGGCCCGATCGTGAAGCAGTTAGAGAGAGGCGGGAGAGCCGTGGTGAAGAT

GGACTGGCGGGAGAACATCACTGTCCCCCTCCAGACAGACCTGCGTAAATTCAGGACCTATAAAGGTGGT

TCTGTCAGAGATCTCCTCCGAGCCATGAGAAATAAGAAGCACCACTACCGGGAGCTGCCTGCAGAGGTGC

GGGAGACGCTGGGGTCCCTCCCCGACGACTTCGTGTGCTACTTCACATCTCGCTTCCCCCACCTCCTCGC

ACACACCTACCGGGCCATGGAGCTGTGCAGCCACGAGAGACTCTTCCAGCCCTACTACTTCCACGAGCCC

CCAGAGCCCCAGCCCCCAGTGACTCCAGACGCCCTCTGAGCGAGGGCGGCCCCTCTGTTCTGGTGGCCCC

AGCTGTGACTGAGGGCCTGGTCACCACAATTAGAGCTTGATGCCTCCCGGCTTTGCAGGGAGACCAGGCT

TCCCAAACCAAGTGCCTTGAGCTGCCTGCTCTGCAGCCCACAGAGGACAGTGCTGACCCCAGGAAGTGGG

AGAAGTGGCCCCTCGTGACCTACAGGGAACTGGGAAGATGCTGGCCCCAAAAGCCTTACGGTCATGATGT

CTGCAAAGGAGGGCCTCAGAGACAGCGCGAGTAGCACCCCCAGCCATCTACTGGATAAACTTGCTTCAGA

CTTTTTAAATTCCTGCTTAATGTCAGTCTACAGGCCTTTCAGGAAGGGAGAGGAGGGAATCGTACATTTT

GCTTGCGTGCTGGGACAGCTAGGCTGAGATGCACCAAGTACAGCCTTCACTGGAGACCGGAATTGAGAGG

TGGGGGATGCTGAGGAGGGGGAGGACGGAGTTCAGAGGGTGTCGTCCTGCAGTGTGAGATTTCTCATTGA

TCACAGATGTGCCCAGAGTAGCCCAGGTCACTGTTAACTAGTGTTTCTGCAGAGGCAGCAGGAGCCATGA

GCATGAGGTGTGGCATTAGGGACTGGTCAGCTATGCATGCTGGCAGGTGGGGTTGTGTCTGCAGGTCTCA

GAAATGAAGAGGCTGCTCTGTTCTGGAGGCAGCCGTGGCCCAGTGCCAGTGGCCAGAACAGTGGCCTTTG

GTGGGTGTGTCCCGGGCCATCTCGGGGTGGTGCTCAGGAGCGCCTGGGGCAAGAGGTAAAGAGTTCCCTG

GCCTTCAAGGAGAGCAGCGAAGACCCAGACAGGGGCCAGCCTTCAGGACCAGAGGGAGGCCGCCGAATGG

GACCCTCCTGGTCACCAGGAGAAAGCCCTGGGCCAGCGAGTAGGCAGTCAAACTCCTTCGTCCCCAAGGC

CGGTGGAACAAGAGGCTCGTGGTGAGTCAGGGCCAGGGTGGGTGGCCAAGGCCAGGGTCACCGTGTGCTT

CATGGGCCAGCTTTTTTGTTTTTCTTGGCAAATTTTAATAACTATATTTTGATTATACTGTAGAATGCTA

TGTCAGCATAAGTAAGCTAAACTTGAAGCTTTCTTGTGAAGAATAAATGCAAGATAGAATACATCTTCTA

TTTTTTGTGGTACCAAAAATCACCATCCCCTCAAGAGTGTTCATGTATAGAACATTCTCTAATGCTGAAG

AGTAAAACATTATAGCAACACTATGTAAATGTATTGAACAGTATCAAAGAAATAGTCTCTAAATTGTTTG

TACCATATTTTTTTTTCTAAACTTAACATAATTTTTAGCTTTAGTTTCAGTCAAAACTTTGTCTTTTCTC

TCCCGAGAGCCTTAGAGGTTAAAATGCAATCAGCCTACCGTGTAAGGAGATGTTGTCCATGTACTTTCTC

CAGCCAGTTGGGGGATCATTGCAGCTCAGGCCTGGTGAACTCAGAGATTCCATTCAGTATTAAGAATGGG

ATTGTTGAATTTTACTCACAGAGAAATCACTGTTTCTTCATGTTGTAAGATGTTTTCTGTTTGTGTATTT

GTATCATGGTTACTCATCAAAAGCTCTCATTCTGCCTTTGTAGAATTCAGTTCCCTTCCTTTCATCATAG

CTAAAGTGACTTTTTTCCCTACTATTAACGTGATCCTACATCCTTAAATCTCATCGATTACCTCACTTAG

GCCTTGGAACCTTGGCCCTTGGTCGGTGTCCTTGGCGTCTTCTAAGCAAGGCTGTGCGTTGTTCAGAAAC

GTGGCCAGACCGCATTTCCTGCTGCTCCCATGCCGCATGCCAGGTGGCCTGAGACAGAGCTCCCCATACG

GCTGCAAGGTGCTTTACCTGTGGGCTTTGGCAGTAACCCAAGAGAGGATCAGAAGGTGGAGAAGGTGCCA

CGAGTGATTTAACAGGCCTGCCACAGGGAGTGCCCCCAGCCCAGCTCGTTCTCAGCACAGGTTTTCTCTT

TGGGAGTACCCAGGTGATTTCTAGTGACCCAATTTTGTGTCATCTCCCTGTTTTAGCCCCACTTGCCTAG

AGACAACTGTTTCCGATGCCTTTTCTGCTTATCATACTAGTTTCTAACCACGCAGATTTCTCAAAATCAT

TTATTCAATGTATTTTATTTGAGCACTTAGTGTATTGAGCTAGGCAGGATATAGGGTGCCGGAGATACAG

CGATGAACAAGACAGGCAAAACTTCTGCTTTCCTAAAACTTGTGATGAGAGAGAACAATAAAAAAGTGTT

GCTGCCACAAAGAAACCAAAGTGTGTGGGGAAGGGCGCGTGTTTGCGGTTTACATCTCTTCTGTCCCAGA

ATCACAGGGATCTGGTCCGGTCACCTCTGGTCTTTCCTCTTAGTCGCCTTTAGGAGCCCTGGTTCCCGTC

CATCCTCTGGGGGGTTTGTTTGAAGAGATCTCGTGTGGGTACTTGTCATGAAAACACCTTGGGCATCATC

TGGTGTATCCAGTTCTAGTCTCGAGAATTCTGGTTTCCCACTGTGCTCAGCAAGTGGAAAGTTCTTTTCA

GGCCAGAACAGCTCTGCACCATCACATATCGTGTTGCGGCTTAGCTGTTTGGTCTGTAGTTCAGGTTATG

GGACTTCTCCAATCCTGGAAGGCTGTTGAGCTTTTTAGAAGTACTGTACGCTATCTTCAAGATGGAGCTT

GGTCACATCTGTTAGGAATCCAAAGGACACTATGACTTATTTAAATCTTGTCTTACTAAACCTCTCTTGG

GCACGTGTGCCAGAATTTCTCTTGTTGCTTCTTGAGTCTTTTTAATTTCAGTGTTTTTTCGTTTGTTTTT

TGTTTTTTTGAGACAGAGTCTCGCTCTGTCACCCAGGCTGGAGTGCAGTGGCACGACCTCAGCTCACTGC

AACCTCCGCCTCCTTGGTTCAAGCAATTCTCCTGCCTCAGCCTCCCGAGTAGCTGGGATTACAGGTGTGT

GCCACCACGCTCGGCTAATTTTTTATATTTTTTTTAGTAGAGACGGGGTTTCCCCATGTTAGGCAGGATG

GTCTCGATCTCCTAACCTCGTGATCCGCCTGCCTCAGCCTCCCAAAGTGCTGGGATTACAGGCTTGAGCC

ACCGCACCCGGCCTAATCTCAGTTTTTGAAGTGCTCCACAAGTCATTAGGCACCAAAACATTTTCACCTG

GGGAACACTGGCATTTCCCTGATTAGCTGTGAAGCAATCTAGTGGCTAAGTGTGAAATCCTGGGTGCGCA

GGTGTTCTCACTCCCGCCGTGTTCTCAGTGCAGTGGTGGTCAGAGGCCCTTCCAAGGAGACATCACTCTG

ATCAGTTACAGATAGATGTTCTGGAAGATCTGCAGGTGAGTAGATCCAGCAGAGTTTCTTCCCACCAACT

CTAGAAGAAAGGGCCTTATCAGAGTTGACCCTGAGCCTTTGGTAAGGTTTTGTGTGCATGCGATTCAGTT

ATCTTTGGCAATTCTTTTCTTGCTGCAGTGAGAGATTAATTGGTTGCTGATCAAACCGTTCATGCAGATG

GGGGGACCTTTGGATTGTACGGCTTTCTCCTCTTGGCTGCTTTCTTTTCAGGAAGTTGGACTTTGGCCAG

GTTTGGCTTTCCCAGAGCCGTTCCTTTCTCTGTCCTTTCCTTGGGTCCTCATGGTGTGCCCATTGGATCC

TTGGCCTTGTGATCCTCTGGGAACTGGGGGCCAGTTCCACTTTTGCAGCCTTCTGTGCTGGAAGAGAAGC

CCAGCGCCCTGGAAGGAGCCTCTTTAAGTCCCCCATGTCGCTTTCTCTCTCTGCTCTTTTAGTGTCTGAG

ATTGCCTTTCTTTGAATTTCCCAGTGTTTCTTTTCCTTGTCCCTTCCCTCACCAACCTGGAGTTATTTTG

GTTGACTATGTCCTGGCTTTGGCTTCTCCTGGCAGGAAGTCATCAGGCATCCTCTCCAGGTGAGCCGAAA

TTCCACCCTCCCAGGTTGGACATCATCTTTTAAACCCAATGGTCTACTCCCCTCCTTCTTTATGAAACAG

TGATTTCCCGTGAGTAACTCTGGTTCTGATTTTTTGTACCGGCGCTTAAATTCTTTCTGTAGACGTTGGA

AAGCCACAAAGAACGTGACTGCAGTGAGCCTCCCACTGGAGCAGCCTTAACCAACACTTTGGCCAAAGCC

CCCCCACCTCCCCTGTGTACTGTGTGTGTGTTTGGTGGATACAGTATTCCTTTTCAGTGTCCCTAAAGCT

GTGATGGGGAGTCCCCACTTACCTAGAAAGCATTACCAGTCACCTACTCTGCATTCTCAGATGTAAACCT

TGTGTAGTGTTCTTTTTGCAATGACCTATTTATTTAACCTATTTATATTTATTTAATTTTTACTCTGAAA

TGTATCCAGTTACAATTGTACTTGCTTAAAGCACATCAGATTTGTTTTGGACAACACCCTTGACCATTTT

AAAACTGGAAAAGTGATACTGTATCCTTCCATGGGATGGATGCTTTACAGTAGTCTTATTATTAAAGGGT

GATTAATTTGGTCGGGGTAAAATGTTAATTTTTAGGTGATTTTTAAGAATTCTGTGCCATTATGTCTTCT

GTGTGGATGGTTAATTGTTTAATTAGTACGTGTTAATTGTGTGATACAGTCTTCTTTGTGGAACCCAAAA

TCCTCTTTTTAGCTTTATATTTTATAAACTGCCAGATTGTACAACTTTTATGTGCATTTTTAAAGCTTGA

AGACATGAGGGTCATTATCTAAGTTAAACAGCCTATTTTTGTGCCTCCTGTACAGTTTTATAATTCTGCT

GATGGCGGCATCTTATGTCGAGCCAACCACAATAAAGGTAGTTTTAGATTTTGGA

>NM_014584.3 Homo sapiens endoplasmic reticulum oxidoreductase 1 alpha (ERO1A), transcript variant 2, mRNA

ATCTGAGGGCGCACGGCCCGCGACCGAGCGTGCGGACTGGCCTCCCAAGCGTGGGGCGACAAGCTGCCGG

AGCTGCAATGGGCCGCGGCTGGGGATTCTTGTTTGGCCTCCTGGGCGCCGTGTGGCTGCTCAGCTCGGGC

CACGGAGAGGAGCAGCCCCCGGAGACAGCGGCACAGAGGTGCTTCTGCCAGGTTAGTGGTTACTTGGATG

ATTGTACCTGTGATGTTGAAACCATTGATAGATTTAATAACTACAGGCTTTTCCCAAGACTACAAAAACT

TCTTGAAAGTGACTACTTTAGGTATTACAAGGTAAACCTGAAGAGGCCGTGTCCTTTCTGGAATGACATC

AGCCAGTGTGGAAGAAGGGACTGTGCTGTCAAACCATGTCAATCTGATGAAGTTCCTGATGGAATTAAAT

CTGCGAGCTACAAGTATTCTGAAGAAGCCAATAATCTCATTGAAGAATGTGAACAAGCTGAACGACTTGG

AGCAGTGGATGAATCTCTGAGTGAGGAAACACAGAAGGCTGTTCTTCAGTGGACCAAGCATGATGATTCT

TCAGATAACTTCTGTGAAGCTGATGACATTCAGTCCCCTGAAGCTGAATATGTAGATTTGCTTCTTAATC

CTGAGCGCTACACTGGTTACAAGGGACCAGATGCTTGGAAAATATGGAATGTCATCTACGAAGAAAACTG

TTTTAAGCCACAGACAATTAAAAGACCTTTAAATCCTTTGGCTTCTGGTCAAGGGACAAGTGAAGAGAAC

ACTTTTTACAGTTGGCTAGAAGGTCTCTGTGTAGAAAAAAGAGCATTCTACAGACTTATATCTGGCCTAC

ATGCAAGCATTAATGTGCATTTGAGTGCAAGATATCTTTTACAAGAGACCTGGTTAGAAAAGAAATGGGG

ACACAACATTACAGAATTTCAACAGCGATTTGATGGAATTTTGACTGAAGGAGAAGGTCCAAGAAGGCTT

AAGAACTTGTATTTTCTCTACTTAATAGAACTAAGGGCTTTATCCAAAGTGTTACCATTCTTCGAGCGCC

CAGATTTTCAACTCTTTACTGGAAATAAAATTCAGGATGAGGAAAACAAAATGTTACTTCTGGAAATACT

TCATGAAATCAAGTCATTTCCTTTGCATTTTGATGAGAATTCATTTTTTGCTGGGGATAAAAAAGAAGCA

CACAAACTAAAGGAGGACTTTCGACTGCATTTTAGAAATATTTCAAGAATTATGGATTGTGTTGGTTGTT

TTAAATGTCGTCTGTGGGGAAAGCTTCAGACTCAGGGTTTGGGCACTGCTCTGAAGATCTTATTTTCTGA

GAAATTGATAGCAAATATGCCAGAAAGTGGACCTAGTTATGAATTCCATCTAACCAGACAAGAAATAGTA

TCATTATTCAACGCATTTGGAAGAATTTCTACAAGTGTGAAAGAATTAGAAAACTTCAGGAACTTGTTAC

AGAATATTCATTAAAGAAAACAAGCTGATATGTGCCTGTTTCTGGACAATGGAGGCGAAAGAGTGGAATT

TCATTCAAAGGCATAATAGCAATGACAGTCTTAAGCCAAACATTTTATATAAAGTTGCTTTTGTAAAGGA

GAATTATATTGTTTTAAGTAAACACATTTTTAAAAATTGTGTTAAGTCTATGTATAATACTACTGTGAGT

AAAAGTAATACTTTAATAATGTGGTACAAATTTTAAAGTTTAATATTGAATAAAAGGAGGATTATCAAAT

TCATATATGATAAAAGTGAATGTTCTAAGTCTCTCAAACTAGCGTTTTATGTAATAATATGTAATATAAA

TAAAACTATGGTAAATGTGACAAGCATTTAATAGGAAAATGCTAAGGAGGCCTCATAAATGACCCATAAT

TACCAACGTAGAATTTTTCAGTACATTTAGGGTTGCTGGATTTAGCAAATAAAAATAAAGATTGCCCAGT

TAGATTTGAATTTCAGATAAACAATTAGTTTTTTAATATTTTACATGGAATATTTGGAAAATACTTATAC

TAAAAAATTATTTGTTTGAAATTCAAATTTAACTGGGAGTCTTGTATTTTATCTGGCAATCCTAAAATAC

ATTGGTATGAAACAAATCACTTTTAGAAGTATATTGCTATTTTGATTGGGTTGTTTTTGTGTGTAGAAAC

GTACAATAACAACTCAAAGGCACAGGAGATTTCTAAACATTGTGAAAAGTTGAATAGATTATATATTTAT

TCTCATAATACTTTCACTAATACTAAATAAAATTTGGGGAACACTTTTTATTTTTATATAATTTCCAATT

TACAGAAAAGTTTCAAAAATAGTACAAAGAGCTCTCTTACCCAGATTCACTAATTGTTCATACGTGCTTT

ATCTTTCATGCTTTCTCTGTACACACACACACACACACAAATTTTTCCTCAATCATTTGAAAGTCAGTTA

TAGGCATCATGCCCCTTAAACCCTAAATACTTCAGTGTGTAATACTGAATAATTACTAAAAATGATTTTC

TCAGAAAAAAAAACTCCCACAATTCTGGAACTATAATACTGTAAGCCTTAGAATAAATAATACTTTCAAG

TTCCAATCTAAAGTTCTTTTTGAGTTTTGTTGCCCGTTTTATGCTTGATGTGTATAGTAATAGGGTAGGC

TATTTATTTTATTAAAATTTTTTTTAGAGACAAGGTTTTGCTGTGTTGCCCAAGCTGGAACTTGAACGAC

TGGGCTGAAGTGATCTTCCCACCTCAGCCTCCCAAGTAGCTGGGAATACAGGTGTCTGCCACCATACCCA

GTTTCATTTTTGTTTTTTATACCCGAAGTTCATTTCCTTTGTCTCCCTAAAACTGAACTGTAATTTTGGG

AGGTTTTCATTAGTGGAAGCTCTTCATTTATAAAGCTATTTGAAGGGGTTTAGGAATTTATATCACATGG

TAATTGTAGAGAAAAAGAAGCTATATACCTCAAAATCGTGCCCTCTTTACATATGTCTTATCAGGTATAA

CATGTTGAAATGTCACATTAGTAGTAAAGTGGGGTTTATTTATATAGTGGTTAAGAAATGTCAGTTTACA

CTGCTGTATACTTCTTCTTCTGTGTCCCTAAGGCCTGGTACAGTGCCAAGCACATACTTGGTATCCAATA

AATATTTGTTGGATGAATGTATGCATATGTATTCAGTATATTTTAAATGAATAATCACAGAAGTAAGTTT

AATAATTTGTCCTATTTTTCTCTGTCACTTCCTTTTTCTCTAAGGCAGGAAAGGAAAGACATTAAACCAT

TAATTAAGTCAATCCTCTTGGAGACTCAAAAGACTATGAAGTGATCACTCTATATAAAATATAAATACAG

TGTGGGTTCAAATGGCCATTTTTTGTGTGTCCCTCTCTCTCATCTTATGCTTCCCTTCCTTTTTTTATTT

TTATTTTTTGGAGACGGAGTCTTGCTGTGTCGCCCAGGTTGGAGTGCAGTGGCGTGATCTTAGCTCACTG

CAAGCTCTGCCTTCCAGGTTCACGCCATTCTTCTGCCTCAGCCCCCTGAGTAGCTGGGACTACAGGCGCC

CGCCACCACGCCCGGCTAATTTTTTTTTTTTTTTTTTTGTATTTTTAGTAGAGGCAGTGTTTCACCGTGT

TAGCCAGGATGGTCTTGATCTCCTGACCTCGTGATCCACCCACCTCGACCTCCCAAAGTGCTGGGATTAC

AGGCGTGAGCCACCACGCCCGGCCTCTTTTTTTTTTAGCTGCCAATCTTTTTGAAGGAATATTCTTACCT

CTACTTTGTCACCTTCTACTGGCTCCTTAACTAAAATCTGCCATTTGGCTCTCTGGTTAACAGTCCCTTC

CTGTAAAGTCTAAAATCTTAATTCTAAATCCACAGTTTAATTCACAAGCTAGTACTTGACTTTTTTTCTG

TATTTGACATTTTTGACAACCCCTACTTTAAAGATTTATTCCCTTGACTTCTTACATTTTGCTCACTCCT

GAACCACCCCCCACCTTTTGGCCTCTTCATTTATTCCTTAAATGTTATTCCTCAGACCTCCATTTTTTTT

TTCTCTCTTAATCACAACACCACTTCTCACGCTTGGGTAATTTTAATTCAGCAGTTCCTAAATCCTTATC

TTTAGCCAGACTCCTCAATCCATCTGCCTGTTGCACTTTTCTTGGTTGTCCCAGAGACACCTGTGTGTGT

CTTAAAACATTCATTCTCTGCAAAACCTACTCTAATGCCTGTGTCCCTTACTTTGGTTAATTTTAGAACC

ATTATATTCTAAGTTTTCTAGGCTCATTCCTCTCCTCCACCTTCCCCTATCATTTAGTGTCTAAGTTTTA

CTGATTTTATCTCCACCTCTCTGATACATCACTCTTTCATCTTCATTGCTATTATTAATAAATACCTACA

GTACTAACCTGCCTCCTATACCTAGCTGGTCTCCTCTCTGTTGCTCAATGTTACCACAGCAGGCTTTCTA

GAAGCACTCTGACAGTGTTACTCCCTAATATCCTTCAGTGACTTCAGGAACTTTCAGGAGAAAGCCAAAC

TCCTCTGTTTGGTGTACAAGGTCTTCTGATGTGTTTCCTCCACCGAATGTTCTGGTGAAACAGACTTACA

CTTCTTCAGAAGCCACATTTGGCCAGGCCTCCCGCCTTGGTAAATGCTGTACTCTTTGCATCAAGTATGC

TAGTCATCCTTCCCCACTTGGAAAATTCCTATGCATCTTGCAGGCCTGACATAAGCATTTCCTCTGTGAA

ACCTCCTTTGCTCCACTCAAGGAGAGTCATCTAACTTCCACTTTCGTGTCACCACTGTAATTACAACCTA

CCTCTATTGTATGTCACTTAAATCGTACTGTATTGTTTTATTTTTCAAAAGTCTTTACTAGAATGTGAGC

TCCTTAAGGGCAGGAAAAGGAACCTTTTTATTTTTTGCATCTCCATAGCATAGTTTTTGGCATATGAATG

TTTAATAAATGTTTGTTGAATAAATTGATTTTAAAGTGACATCTTTATTATATTAGAGGTCCTACCTATA

TTCCAAATACTTTCACTCCCTTCACTTTACAGCAAGGGTCAGTAGAGTCCCAAGGATTTGTAGACTTTAG

GGGGTCAATAAAGCTGAAATTGTATTCAA

>NM_003824.4 Homo sapiens Fas associated via death domain (FADD), mRNA

GCTCTTGTCGATTTCCTGTAGTGAATCAGGCACCGGAGTGCAGGTTCGGGGGTGGAATCCTTGGGCCGCT

GGGCAAGCGGCGAGACCTGGCCAGGGCCAGCGAGCCGAGGACAGAGGGCGCACGGAGGGCCGGGCCGCAG

CCCCGGCCGCTTGCAGACCCCGCCATGGACCCGTTCCTGGTGCTGCTGCACTCGGTGTCGTCCAGCCTGT

CGAGCAGCGAGCTGACCGAGCTCAAGTTCCTATGCCTCGGGCGCGTGGGCAAGCGCAAGCTGGAGCGCGT

GCAGAGCGGCCTAGACCTCTTCTCCATGCTGCTGGAGCAGAACGACCTGGAGCCCGGGCACACCGAGCTC

CTGCGCGAGCTGCTCGCCTCCCTGCGGCGCCACGACCTGCTGCGGCGCGTCGACGACTTCGAGGCGGGGG

CGGCGGCCGGGGCCGCGCCTGGGGAAGAAGACCTGTGTGCAGCATTTAACGTCATATGTGATAATGTGGG

GAAAGATTGGAGAAGGCTGGCTCGTCAGCTCAAAGTCTCAGACACCAAGATCGACAGCATCGAGGACAGA

TACCCCCGCAACCTGACAGAGCGTGTGCGGGAGTCACTGAGAATCTGGAAGAACACAGAGAAGGAGAACG

CAACAGTGGCCCACCTGGTGGGGGCTCTCAGGTCCTGCCAGATGAACCTGGTGGCTGACCTGGTACAAGA

GGTTCAGCAGGCCCGTGACCTCCAGAACAGGAGTGGGGCCATGTCCCCGATGTCATGGAACTCAGACGCA

TCTACCTCCGAAGCGTCCTGATGGGCCGCTGCTTTGCGCTGGTGGACCACAGGCATCTACACAGCCTGGA

CTTTGGTTCTCTCCAGGAAGGTAGCCCAGCACTGTGAAGACCCAGCAGGAAGCCAGGCTGAGTGAGCCAC

AGACCACCTGCTTCTGAACTCAAGCTGCGTTTATTAATGCCTCTCCCGCACCAGGCCGGGCTTGGGCCCT

GCACAGATATTTCCATTTCTTCCTCACTATGACACTGAGCAAGATCTTGTCTCCACTAAATGAGCTCCTG

CGGGAGTAGTTGGAAAGTTGGAACCGTGTCCAGCACAGAAGGAATCTGTGCAGATGAGCAGTCACACTGT

TACTCCACAGCGGAGGAGACCAGCTCAGAGGCCCAGGAATCGGAGCGAAGCAGAGAGGTGGAGAACTGGG

ATTTGAACCCCCGCCATCCTTCACCAGAGCCCATGCTCAACCACTGTGGCGTTCTGCTGCCCCTGCAGTT

GGCAGAAAGGATGTTTTGTCCCATTTCCTTGGAGGCCACCGGGACAGACCTGGACACTAGGGTCAGGCGG

GGTGCTGTGGTGGGGAGAGGCATGGCTGGGGTGGGGGTGGGGAGACCTGGTTGGCCGTGGTCCAGCTCTT

GGCCCCTGTGTGAGTTGAGTCTCCTCTCTGAGACTGCTAAGTAGGGGCAGTGATGGTTGCCAGGACGAAT

TGAGATAATATCTGTGAGGTGCTGATGAGTGATTGACACACAGCACTCTCTAAATCTTCCTTGTGAGGAT

TATGGGTCCTGCAATTCTACAGTTTCTTACTGTTTTGTATCAAAATCACTATCTTTCTGATAACAGAATT

GCCAAGGCAGCGGGATCTCGTATCTTTAAAAAGCAGTCCTCTTATTCCTAAGGTAATCCTATTAAAACAC

AGCTTTACAACTTCCATACTACAAAAAA

>NM_000043.6 Homo sapiens Fas cell surface death receptor (FAS), transcript variant 1, mRNA

CTCTTCTCCCGCGGGTTGGTGGACCCGCTCAGTACGGAGTTGGGGAAGCTCTTTCACTTCGGAGGATTGC

TCAACAACCATGCTGGGCATCTGGACCCTCCTACCTCTGGTTCTTACGTCTGTTGCTAGATTATCGTCCA

AAAGTGTTAATGCCCAAGTGACTGACATCAACTCCAAGGGATTGGAATTGAGGAAGACTGTTACTACAGT

TGAGACTCAGAACTTGGAAGGCCTGCATCATGATGGCCAATTCTGCCATAAGCCCTGTCCTCCAGGTGAA

AGGAAAGCTAGGGACTGCACAGTCAATGGGGATGAACCAGACTGCGTGCCCTGCCAAGAAGGGAAGGAGT

ACACAGACAAAGCCCATTTTTCTTCCAAATGCAGAAGATGTAGATTGTGTGATGAAGGACATGGCTTAGA

AGTGGAAATAAACTGCACCCGGACCCAGAATACCAAGTGCAGATGTAAACCAAACTTTTTTTGTAACTCT

ACTGTATGTGAACACTGTGACCCTTGCACCAAATGTGAACATGGAATCATCAAGGAATGCACACTCACCA

GCAACACCAAGTGCAAAGAGGAAGGATCCAGATCTAACTTGGGGTGGCTTTGTCTTCTTCTTTTGCCAAT

TCCACTAATTGTTTGGGTGAAGAGAAAGGAAGTACAGAAAACATGCAGAAAGCACAGAAAGGAAAACCAA

GGTTCTCATGAATCTCCAACTTTAAATCCTGAAACAGTGGCAATAAATTTATCTGATGTTGACTTGAGTA

AATATATCACCACTATTGCTGGAGTCATGACACTAAGTCAAGTTAAAGGCTTTGTTCGAAAGAATGGTGT

CAATGAAGCCAAAATAGATGAGATCAAGAATGACAATGTCCAAGACACAGCAGAACAGAAAGTTCAACTG

CTTCGTAATTGGCATCAACTTCATGGAAAGAAAGAAGCGTATGACACATTGATTAAAGATCTCAAAAAAG

CCAATCTTTGTACTCTTGCAGAGAAAATTCAGACTATCATCCTCAAGGACATTACTAGTGACTCAGAAAA

TTCAAACTTCAGAAATGAAATCCAAAGCTTGGTCTAGAGTGAAAAACAACAAATTCAGTTCTGAGTATAT

GCAATTAGTGTTTGAAAAGATTCTTAATAGCTGGCTGTAAATACTGCTTGGTTTTTTACTGGGTACATTT

TATCATTTATTAGCGCTGAAGAGCCAACATATTTGTAGATTTTTAATATCTCATGATTCTGCCTCCAAGG

ATGTTTAAAATCTAGTTGGGAAAACAAACTTCATCAAGAGTAAATGCAGTGGCATGCTAAGTACCCAAAT

AGGAGTGTATGCAGAGGATGAAAGATTAAGATTATGCTCTGGCATCTAACATATGATTCTGTAGTATGAA

TGTAATCAGTGTATGTTAGTACAAATGTCTATCCACAGGCTAACCCCACTCTATGAATCAATAGAAGAAG

CTATGACCTTTTGCTGAAATATCAGTTACTGAACAGGCAGGCCACTTTGCCTCTAAATTACCTCTGATAA

TTCTAGAGATTTTACCATATTTCTAAACTTTGTTTATAACTCTGAGAAGATCATATTTATGTAAAGTATA

TGTATTTGAGTGCAGAATTTAAATAAGGCTCTACCTCAAAGACCTTTGCACAGTTTATTGGTGTCATATT

ATACAATATTTCAATTGTGAATTCACATAGAAAACATTAAATTATAATGTTTGACTATTATATATGTGTA

TGCATTTTACTGGCTCAAAACTACCTACTTCTTTCTCAGGCATCAAAAGCATTTTGAGCAGGAGAGTATT

ACTAGAGCTTTGCCACCTCTCCATTTTTGCCTTGGTGCTCATCTTAATGGCCTAATGCACCCCCAAACAT

GGAAATATCACCAAAAAATACTTAATAGTCCACCAAAAGGCAAGACTGCCCTTAGAAATTCTAGCCTGGT

TTGGAGATACTAACTGCTCTCAGAGAAAGTAGCTTTGTGACATGTCATGAACCCATGTTTGCAATCAAAG

ATGATAAAATAGATTCTTATTTTTCCCCCACCCCCGAAAATGTTCAATAATGTCCCATGTAAAACCTGCT

ACAAATGGCAGCTTATACATAGCAATGGTAAAATCATCATCTGGATTTAGGAATTGCTCTTGTCATACCC

CCAAGTTTCTAAGATTTAAGATTCTCCTTACTACTATCCTACGTTTAAATATCTTTGAAAGTTTGTATTA

AATGTGAATTTTAAGAAATAATATTTATATTTCTGTAAATGTAAACTGTGAAGATAGTTATAAACTGAAG

CAGATACCTGGAACCACCTAAAGAACTTCCATTTATGGAGGATTTTTTTGCCCCTTGTGTTTGGAATTAT

AAAATATAGGTAAAAGTACGTAATTAAATAATGTTTTTGGTATTTCTGGTTTTCTCTTTTTTGGTAGGGG

CTTGCTTTTTGGTTTTGTCTTCCTTTTCTCTAACTGATGCTAAATATAACTTGTCTTTAATGCTTCTTGG

ATCCCTTAGAAGGTACTTCCTTTTTAACCTTAACCCTTTTAGTAGTTAAATAATTATTTCCATAGGTTGC

TATTGCCAAGAAGACCTCTTCCAAACAGCACATGATTATTCGTCAAACAGTTTCGTATTCCAGATACTGG

AATGTGGATAAGAAAGTATACATTTCAAGGGGTAGGTTTTATTATTAAGAAAGCCAAATGAGGATTTTGA

AATATTCTTTCCTGCATATTATCCATTCTAGCTACATGCTGGCCAGTGGGCCACCTTTCTTTTCTGCAAT

TTAATGCTAGTAATATATTCTATTTAACCCATGAGTCCCAAAGTATTAGCATTTCAACATGTAAGCATGT

CGGTAAGATAGTTGTGCTTTGCTTAGGGTTCCCTCCTGTGTTATGGTCTGGAAAGTGTCTTTAGGCAGAA

AGTCTGAGTGATCACAGGGTTCACTCATTAATTTCTCTTTTCTGAGCCATCATAGTCTGTGCTGTCTGCT

CTCCAGTTTTCTATTTCTAGACAGAAGTAGGGCAAGTTAGGTACTAGTTATTCTTCATGGCCAGAAGTGC

AAGTTCTACTTTGCAAGACAAGATTAAGTTAGAGAACACCCTATTCCACTTTGGTGAACTCAGAGCAAGA

ACTTTGAGTTCCTTTGGGAGGAAGACAGTGGAGAAGTCTTTGTACTTGGTGATGTGGTTTTTTTCCTCAT

GGCTTCACCTAGTGGCCCCAAGCATGACTTCTCCCATGTCAATGAGCACAGCCACATTCCCGAGTTGAGG

TGACCCCACGGTCCAGAATCATCCTCATTCTGGTGAACCTGGTTCTCTTTGTGGTGGGCATACTGGGTAG

GAGAATCACCCAAAGGTCACCCATGAGCTGCAGAAAAAAAGGCTATTTGCAGAAGGAGCTCACAGATCAC

ATTGAAAGCATTGCATATTCAAACATCTTGGTCTTCTTTATTGGCATGCCCACAGGGTCTTCTGACCTCT

GATTAGATCAGACACTTTTTAGATATTGAATCATCAGTTTCTGTACAACTATCTGAATAAGGTATATAAT

CAATGAAATTTAGAATTTTTTTCTATGCTTACTCCTGATTGGTAATTTGTTTGGGTTTAGAATTCTATAC

AAGGCCATTTGTAATTTTCCTCAGCACTTTAAAAATATTAAACCATGTTTTCTTAA

>NM_000801.5 Homo sapiens FKBP prolyl isomerase 1A (FKBP1A), transcript variant 1, mRNA

GCTGTTGGTCCACGCCGCCCGTCGCGCCGCCCGCCCGCTCAGCGTCCGCCGCCGCCATGGGAGTGCAGGT

GGAAACCATCTCCCCAGGAGACGGGCGCACCTTCCCCAAGCGCGGCCAGACCTGCGTGGTGCACTACACC

GGGATGCTTGAAGATGGAAAGAAATTTGATTCCTCCCGGGACAGAAACAAGCCCTTTAAGTTTATGCTAG

GCAAGCAGGAGGTGATCCGAGGCTGGGAAGAAGGGGTTGCCCAGATGAGTGTGGGTCAGAGAGCCAAACT

GACTATATCTCCAGATTATGCCTATGGTGCCACTGGGCACCCAGGCATCATCCCACCACATGCCACTCTC

GTCTTCGATGTGGAGCTTCTAAAACTGGAATGACAGGAATGGCCTCCTCCCTTAGCTCCCTGTTCTTGGA

TCTGCCATGGAGGGATCTGGTGCCTCCAGACATGTGCACATGAATCCATATGGAGCTTTTCCTGATGTTC

CACTCCACTTTGTATAGACATCTGCCCTGACTGAATGTGTTCTGTCACTCAGCTTTGCTTCCGACACCTC

TGTTTCCTCTTCCCCTTTCTCCTCGTATGTGTGTTTACCTAAACTATATGCCATAAACCTCAAGTTATTC

ATTTTATTTTGTTTTCATTTTGGGGTGAAGATTCAGTTTCAGTCTTTTGGATATAGGTTTCCAATTAAGT

ACATGGTCAAGTATTAACAGCACAAGTGGTAGGTTAACATTAGAATAGGAATTGGTGTTGGGGGGGGGGT

TTGCAAGAATATTTTATTTTAATTTTTTGGATGAAATTTTTATCTATTATATATTAAACATTCTTGCTGC

TGCGCTGCAAAGCCATAGCAGATTTGAGGCGCTGTTGAGGACTGAATTACTCTCCAAGTTGAGAGATGTC

TTTGGGTTAAATTAAAAGCCCTACCTAAAACTGAGGTGGGGATGGGGAGAGCCTTTGCCTCCACCATTCC

CACCCACCCTCCCCTTAAACCCTCTGCCTTTGAAAGTAGATCATGTTCACTGCAATGCTGGACACTACAG

GTATCTGTCCCTGGGCCAGCAGGGACCTCTGAAGCCTTCTTTGTGGCCTTTTTTTTTTTTCATCCTGTGG

TTTTTCTAATGGACTTTCAGGAATTTTGTAATCTCATAACTTTCCAAGCTCCACCACTTCCTAAATCTTA

AGAACTTTAATTGACAGTTTCAATTGAAGGTGCTGTTTGTAGACTTAACACCCAGTGAAAGCCCAGCCAT

CATGACAAATCCTTGAATGTTCTCTTAAGAAAATGATGCTGGTCATCGCAGCTTCAGCATCTCCTGTTTT

TTGATGCTTGGCTCCCTCTGCTGATCTCAGTTTCCTGGCTTTTCCTCCCTCAGCCCCTTCTCACCCCTTT

GCTGTCCTGTGTAGTGATTTGGTGAGAAATCGTTGCTGCACCCTTCCCCCAGCACCATTTATGAGTCTCA

AGTTTTATTATTGCAATAAAAGTGCTTTATGCCGGCTTTTCTCA

>NM_004116.5 Homo sapiens FKBP prolyl isomerase 1B (FKBP1B), transcript variant 1, mRNA

GCAGTGGCGGCGAGGAGGCGAGCCGGAGCGACGGCGGGGCTGGGGCCGGAGCCGAGCCGGGGTCGGGCAG

CAGCAGGGACCCCCCAGAGGCGGGGCCTGTGGGACCGCTATGGGCGTGGAGATCGAGACCATCTCCCCCG

GAGACGGAAGGACATTCCCCAAGAAGGGCCAAACGTGTGTGGTGCACTACACAGGAATGCTCCAAAATGG

GAAGAAGTTTGATTCATCCAGAGACAGAAACAAACCTTTCAAGTTCAGAATTGGCAAACAGGAAGTCATC

AAAGGTTTTGAAGAGGGTGCAGCCCAGATGAGCTTGGGGCAGAGGGCGAAGCTGACCTGCACCCCTGATG

TGGCATATGGAGCCACGGGCCACCCCGGTGTCATCCCTCCCAATGCCACCCTCATCTTTGACGTGGAGCT

GCTCAACTTAGAGTGAAGGCAGGAAGGAACTCAAGGTGGCTGGAGATGGCTGCTGCTCACCCTCCTAGCC

TGCTCTGCCACTGGGACGGCTCCTGCTTTTGGGGCTCTTGATCAGTGTGCTAACCTCACTGCCTCATGGC

ATCATCCATTCTCTCTGCCCAAGTTGCTCTGTATGTGTTCGTCAGTGTTCATGCGAATTCTTGCTTGAGG

AAACTTCGGTTGCAGATTGAAGCATTTCAGGTTGTGCATTTTGTGTGATGCATGTAGTAGCCTTTCCTGA

TGACAGAACACAGATCTCTTGTTCGCACAATCTACACTGCCTTACCTTCACTTAAACCACACACACAAGG

TGCTCAGACATGAAATGTACATGGCGTACCGTACACAGAGGGACTTGAGCCAGTTACCTTTGCTGTCACT

TTCTCTCTTATAAATTCTGTTAGCTGCTCACTTAAACAATGTCCTCTTTGAGAAAATGTAAAATAAAGGC

TCTGTGCTTGACAAA

>NM_005252.4 Homo sapiens Fos proto-oncogene, AP-1 transcription factor subunit (FOS), mRNA

AACCGCATCTGCAGCGAGCATCTGAGAAGCCAAGACTGAGCCGGCGGCCGCGGCGCAGCGAACGAGCAGT

GACCGTGCTCCTACCCAGCTCTGCTCCACAGCGCCCACCTGTCTCCGCCCCTCGGCCCCTCGCCCGGCTT

TGCCTAACCGCCACGATGATGTTCTCGGGCTTCAACGCAGACTACGAGGCGTCATCCTCCCGCTGCAGCA

GCGCGTCCCCGGCCGGGGATAGCCTCTCTTACTACCACTCACCCGCAGACTCCTTCTCCAGCATGGGCTC

GCCTGTCAACGCGCAGGACTTCTGCACGGACCTGGCCGTCTCCAGTGCCAACTTCATTCCCACGGTCACT

GCCATCTCGACCAGTCCGGACCTGCAGTGGCTGGTGCAGCCCGCCCTCGTCTCCTCCGTGGCCCCATCGC

AGACCAGAGCCCCTCACCCTTTCGGAGTCCCCGCCCCCTCCGCTGGGGCTTACTCCAGGGCTGGCGTTGT

GAAGACCATGACAGGAGGCCGAGCGCAGAGCATTGGCAGGAGGGGCAAGGTGGAACAGTTATCTCCAGAA

GAAGAAGAGAAAAGGAGAATCCGAAGGGAAAGGAATAAGATGGCTGCAGCCAAATGCCGCAACCGGAGGA

GGGAGCTGACTGATACACTCCAAGCGGAGACAGACCAACTAGAAGATGAGAAGTCTGCTTTGCAGACCGA

GATTGCCAACCTGCTGAAGGAGAAGGAAAAACTAGAGTTCATCCTGGCAGCTCACCGACCTGCCTGCAAG

ATCCCTGATGACCTGGGCTTCCCAGAAGAGATGTCTGTGGCTTCCCTTGATCTGACTGGGGGCCTGCCAG

AGGTTGCCACCCCGGAGTCTGAGGAGGCCTTCACCCTGCCTCTCCTCAATGACCCTGAGCCCAAGCCCTC

AGTGGAACCTGTCAAGAGCATCAGCAGCATGGAGCTGAAGACCGAGCCCTTTGATGACTTCCTGTTCCCA

GCATCATCCAGGCCCAGTGGCTCTGAGACAGCCCGCTCCGTGCCAGACATGGACCTATCTGGGTCCTTCT

ATGCAGCAGACTGGGAGCCTCTGCACAGTGGCTCCCTGGGGATGGGGCCCATGGCCACAGAGCTGGAGCC

CCTGTGCACTCCGGTGGTCACCTGTACTCCCAGCTGCACTGCTTACACGTCTTCCTTCGTCTTCACCTAC

CCCGAGGCTGACTCCTTCCCCAGCTGTGCAGCTGCCCACCGCAAGGGCAGCAGCAGCAATGAGCCTTCCT

CTGACTCGCTCAGCTCACCCACGCTGCTGGCCCTGTGAGGGGGCAGGGAAGGGGAGGCAGCCGGCACCCA

CAAGTGCCACTGCCCGAGCTGGTGCATTACAGAGAGGAGAAACACATCTTCCCTAGAGGGTTCCTGTAGA

CCTAGGGAGGACCTTATCTGTGCGTGAAACACACCAGGCTGTGGGCCTCAAGGACTTGAAAGCATCCATG

TGTGGACTCAAGTCCTTACCTCTTCCGGAGATGTAGCAAAACGCATGGAGTGTGTATTGTTCCCAGTGAC

ACTTCAGAGAGCTGGTAGTTAGTAGCATGTTGAGCCAGGCCTGGGTCTGTGTCTCTTTTCTCTTTCTCCT

TAGTCTTCTCATAGCATTAACTAATCTATTGGGTTCATTATTGGAATTAACCTGGTGCTGGATATTTTCA

AATTGTATCTAGTGCAGCTGATTTTAACAATAACTACTGTGTTCCTGGCAATAGTGTGTTCTGATTAGAA

ATGACCAATATTATACTAAGAAAAGATACGACTTTATTTTCTGGTAGATAGAAATAAATAGCTATATCCA

TGTACTGTAGTTTTTCTTCAACATCAATGTTCATTGTAATGTTACTGATCATGCATTGTTGAGGTGGTCT

GAATGTTCTGACATTAACAGTTTTCCATGAAAACGTTTTATTGTGTTTTTAATTTATTTATTAAGATGGA

TTCTCAGATATTTATATTTTTATTTTATTTTTTTCTACCTTGAGGTCTTTTGACATGTGGAAAGTGAATT

TGAATGAAAAATTTAAGCATTGTTTGCTTATTGTTCCAAGACATTGTCAATAAAAGCATTTAAGTTGAAT

GCGA

>NM_002015.4 Homo sapiens forkhead box O1 (FOXO1), mRNA

GATCCCGTAAGTCGGGCGGCCTGGTAGTCGCAGCAGCCGCTGCCGCAGCCGCCACATTCAACAGGCAGCA

GCGCAGCGGGCGCGCCGCTGGGGAGAGCAAGCGGCCCGCGGCGTCCGTCCGTCCTTCCGTCCGCGGCCCT

GTCAGCTGGAGCGCGGCGCAGGCTCTGCCCCGGCCCGGCGGCTCTGGCCGGCCGTCCAGTCCGTGCGGCG

GACCCCGAGGAGCCTCGATGTGGATGGCCCCGCGAAGTTAAGTTCTGGGCTCGCGCTTCCACTCCGCCGC

GCCTTCCTCCCAGTTTCCGTCCGCTCGCCGCACCGGCTTCGTTCCCCCAAATCTCGGACCGTCCCTTCGC

GCCCCCTCCCCGTCCGCCCCCAGTGCTGCGTTCTCCCCCTCTTGGCTCTCCTGCGGCTGGGGGAGGGGCG

GGGGTCACCATGGCCGAGGCGCCTCAGGTGGTGGAGATCGACCCGGACTTCGAGCCGCTGCCCCGGCCGC

GCTCGTGCACCTGGCCGCTGCCCAGGCCGGAGTTTAGCCAGTCCAACTCGGCCACCTCCAGCCCGGCGCC

GTCGGGCAGCGCGGCTGCCAACCCCGACGCCGCGGCGGGCCTGCCCTCGGCCTCGGCTGCCGCTGTCAGC

GCCGACTTCATGAGCAACCTGAGCTTGCTGGAGGAGAGCGAGGACTTCCCGCAGGCGCCCGGCTCCGTGG

CGGCGGCGGTGGCGGCGGCGGCCGCCGCGGCCGCCACCGGGGGGCTGTGCGGGGACTTCCAGGGCCCGGA

GGCGGGCTGCCTGCACCCAGCGCCACCGCAGCCCCCGCCGCCCGGGCCGCTGTCGCAGCACCCGCCGGTG

CCCCCCGCCGCCGCTGGGCCGCTCGCGGGGCAGCCGCGCAAGAGCAGCTCGTCCCGCCGCAACGCGTGGG

GCAACCTGTCCTACGCCGACCTCATCACCAAGGCCATCGAGAGCTCGGCGGAGAAGCGGCTCACGCTGTC

GCAGATCTACGAGTGGATGGTCAAGAGCGTGCCCTACTTCAAGGATAAGGGTGACAGCAACAGCTCGGCG

GGCTGGAAGAATTCAATTCGTCATAATCTGTCCCTACACAGCAAGTTCATTCGTGTGCAGAATGAAGGAA

CTGGAAAAAGTTCTTGGTGGATGCTCAATCCAGAGGGTGGCAAGAGCGGGAAATCTCCTAGGAGAAGAGC

TGCATCCATGGACAACAACAGTAAATTTGCTAAGAGCCGAAGCCGAGCTGCCAAGAAGAAAGCATCTCTC

CAGTCTGGCCAGGAGGGTGCTGGGGACAGCCCTGGATCACAGTTTTCCAAATGGCCTGCAAGCCCTGGCT

CTCACAGCAATGATGACTTTGATAACTGGAGTACATTTCGCCCTCGAACTAGCTCAAATGCTAGTACTAT

TAGTGGGAGACTCTCACCCATTATGACCGAACAGGATGATCTTGGAGAAGGGGATGTGCATTCTATGGTG

TACCCGCCATCTGCCGCAAAGATGGCCTCTACTTTACCCAGTCTGTCTGAGATAAGCAATCCCGAAAACA

TGGAAAATCTTTTGGATAATCTCAACCTTCTCTCATCACCAACATCATTAACTGTTTCGACCCAGTCCTC

ACCTGGCACCATGATGCAGCAGACGCCGTGCTACTCGTTTGCGCCACCAAACACCAGTTTGAATTCACCC

AGCCCAAACTACCAAAAATATACATATGGCCAATCCAGCATGAGCCCTTTGCCCCAGATGCCTATACAAA

CACTTCAGGACAATAAGTCGAGTTATGGAGGTATGAGTCAGTATAACTGTGCGCCTGGACTCTTGAAGGA

GTTGCTGACTTCTGACTCTCCTCCCCATAATGACATTATGACACCAGTTGATCCTGGGGTAGCCCAGCCC

AACAGCCGGGTTCTGGGCCAGAACGTCATGATGGGCCCTAATTCGGTCATGTCAACCTATGGCAGCCAGG

CATCTCATAACAAAATGATGAATCCCAGCTCCCATACCCACCCTGGACATGCTCAGCAGACATCTGCAGT

TAACGGGCGTCCCCTGCCCCACACGGTAAGCACCATGCCCCACACCTCGGGTATGAACCGCCTGACCCAA

GTGAAGACACCTGTACAAGTGCCTCTGCCCCACCCCATGCAGATGAGTGCCCTGGGGGGCTACTCCTCCG

TGAGCAGCTGCAATGGCTATGGCAGAATGGGCCTTCTCCACCAGGAGAAGCTCCCAAGTGACTTGGATGG

CATGTTCATTGAGCGCTTAGACTGTGACATGGAATCCATCATTCGGAATGACCTCATGGATGGAGATACA

TTGGATTTTAACTTTGACAATGTGTTGCCCAACCAAAGCTTCCCACACAGTGTCAAGACAACGACACATA

GCTGGGTGTCAGGCTGAGGGTTAGTGAGCAGGTTACACTTAAAAGTACTTCAGATTGTCTGACAGCAGGA

ACTGAGAGAAGCAGTCCAAAGATGTCTTTCACCAACTCCCTTTTAGTTTTCTTGGTTAAAAAAAAAAACA

AAAAAAAAAACCCTCCTTTTTTCCTTTCGTCAGACTTGGCAGCAAAGACATTTTTCCTGTACAGGATGTT

TGCCCAATGTGTGCAGGTTATGTGCTGCTGTAGATAAGGACTGTGCCATTGGAAATTTCATTACAATGAA

GTGCCAAACTCACTACACCATATAATTGCAGAAAAGATTTTCAGATCCTGGTGTGCTTTCAAGTTTTGTA

TATAAGCAGTAGATACAGATTGTATTTGTGTGTGTTTTTGGTTTTTCTAAATATCCAATTGGTCCAAGGA

AAGTTTATACTCTTTTTGTAATACTGTGATGGGCCTCATGTCTTGATAAGTTAAACTTTTGTTTGTACTA

CCTGTTTTCTGCGGAACTGACGGATCACAAAGAACTGAATCTCCATTCTGCATCTCCATTGAACAGCCTT

GGACCTGTTCACGTTGCCACAGAATTCACATGAGAACCAAGTAGCCTGTTATCAATCTGCTAAATTAATG

GACTTGTTAAACTTTTGGAAAAAAAAAAGATTAAATGCCAGCTTTGTACAGGTCTTTTCTATTTTTTTTTG

TTTATTTTGTTATTTGCAAATTTGTACAAACATTTAAATGGTTCTAATTTCCAGATAAATGATTTTTGAT

GTTATTGTTGGGACTTAAGAACATTTTTGGAATAGATATTGAACTGTAATAATGTTTTCTTAAAACTAGA

GTCTACTTTGTTACATAGTCAGCTTGTAAATTTTGTGGAACCACAGGTATTTGGGGCAGCATTCATAATT

TTCATTTTGTATTCTAACTGGATTAGTACTAATTTTATACATGCTTAACTGGTTTGTACACTTTGGGATG

CTACTTAGTGATGTTTCTGACTAATCTTAAATCATTGTAATTAGTACTTGCATATTCAACGTTTCAGGCC

CTGGTTGGGCAGGAAAGTGATGTATAGTTATGGACACTTTGCGTTTCTTATTTAGGATAACTTAATATGT

TTTTATGTATGTATTTTAAAGAAATTTCATCTGCTTCTACTGAACTATGCGTACTGCATAGCATCAAGTC

TTCTCTAGAGACCTCTGTAGTCCTGGGAGGCCTCATAATGTTTGTAGATCAGAAAAGGGAGATCTGCATC

TAAAGCAATGGTCCTTTGTCAAACGAGGGATTTTGATCCACTTCACCATTTTGAGTTGAGCTTTAGCAAA

AGTTTCCCCTCATAATTCTTTGCTCTTGTTTCAGTCCAGGTGGAGGTTGGTTTTGTAGTTCTGCCTTGAG

GAATTATGTCAACACTCATACTTCATCTCATTCTCCCTTCTGCCCTGCAGATTAGATTACTTAGCACACT

GTGGAAGTTTAAGTGGAAGGAGGGAATTTAAAAATGGGACTTGAGTGGTTTGTAGAATTTGTGTTCATAA

GTTCAGATGGGTAGCAAATGGAATAGAACTTACTTAAAAATTGGGGAGATTTATTTGAAAACCAGCTGTA

AGTTGTGCATTGAGATTATGTTAAAAGCCTTGGCTTAAGAATTTGAAAATTTCTTTAGCCTGTAGCAACC

TAAACTGTAATTCCTATCATTATGTTTTATTACTTTCCAATTACCTGTAACTGACAGACCAAATTAATTG

GCTTTGTGTCCTATTTAGTCCATCAGTATTTTCAAGTCATGTGGAAAGCCCAAAGTCATCACAATGAAGA

GAACAGGTGCACAGCACTGTTCCTCTTGTGTTCTTGAGAAGGATCTAATTTTTCTGTATATAGCCCACAT

CACACTTGCTTTGTCTTGTATGTTAATTGCATCTTCATTGGCTTGGTATTTCCTAAATGTTTAACAAGAA

CACAAGTGTTCCTGATAAGATTTCCTACAGTAAGCCAGCTCTATTGTAAGCTTCCCACTGTGATGATCAT

TTTTTTGAAGATTCATTGAACAGCCACCACTCTATCATCCTCATTTTGGGGCAGTCCAAGACATAGCTGG

TTTTAGAAACCCAAGTTCCTCTAAGCACAGCCTCCCGGGTATGTAACTGAACTTGGTGCCAAAGTACTTG

TGTACTAATTTCTATTACTACGTACTGTCACTTTCCTCCCGTGCCATTACTGCATCATAATACAAGGAAC

CTCAGAGCCCCCATTTGTTCATTAAAGAGGCAACTACAGCCAAAATCACTGTTAAAATCTTACTACTTCA

TGGAGTAGCTCTTAGGAAAATATATCTTCCTCCTGAGTCTGGGTAATTATACCTCTCCCAAGCCCCCATT

GTGTGTTGAAATCCTGTCATGAATCCTTGGTAGCTCTCTGAGAACAGTGAAGTCCAGGGAAAGGCATCTG

GTCTGTCTGGAAAGCAAACATTATGTGGCCTCTGGTAGTTTTTTTCCTGTAAGAATACTGACTTTCTGGA

GTAATGAGTATATATCAGTTATTGTACATGATTGCTTTGTGAAATGTGCAAATGATATCACCTATGCAGC

CTTGTTTGATTTATTTTCTCTGGTTTGTACTGTTATTAAAAGCATATTGTATTATAGAGCTATTCAGATA

TTTTAAATATAAAGATGTATTGTTTCCGTAATATAGACGTATGGAATATATTTAGGTAATAGATGTATTA

CTTGGAAAGTTCTGCTTTGACAAACTGACAAAGTCTAAATGAGCACATGTATCCCAGTGAGCAGTAAATC

AATGGAACATCCCAAGAAGAGGATAAGGATGCTTAAAATGGAAATCATTCTCCAACGATATACAAATTGG

ACTTGTTCAACTGCTGGATATATGCTACCAATAACCCCAGCCCCAACTTAAAATTCTTACATTCAAGCTC

CTAAGAGTTCTTAATTTATAACTAATTTTAAAAGAGAAGTTTCTTTTCTGGTTTTAGTTTGGGAATAATC

ATTCATTAAAAAAAATGTATTGTGGTTTATGCGAACAGACCAACCTGGCATTACAGTTGGCCTCTCCTTG

AGGTGGGCACAGCCTGGCAGTGTGGCCAGGGGTGGCCATGTAAGTCCCATCAGGACGTAGTCATGCCTCC

TGCATTTCGCTACCCGAGTTTAGTAACAGTGCAGATTCCACGTTCTTGTTCCGATACTCTGAGAAGTGCC

TGATGTTGATGTACTTACAGACACAAGAACAATCTTTGCTATAATTGTATAAAGCCATAAATGTACATAA

ATTATGTTTAAATGGCTTGGTGTCTTTCTTTTCTAATTATGCAGAATAAGCTCTTTATTAGGAATTTTTT

GTGAAGCTATTAAATACTTGAGTTAAGTCTTGTCAGCCA

>NM_001455.4 Homo sapiens forkhead box O3 (FOXO3), transcript variant 1, mRNA

GGTGTCTGCTGCGCCAGGTTCGCTGGCCGCACGTCTTCAGGTCCTCCTGTTCCTGGGAGGCGGGCGCGGC

AGGACTGGGAGGTGGCGGCAGCGGGCGAGGACTCGCCGAGGACGGGGCTCCGGCCCGGGATAACCAACTC

TCCTTCTCTCTTCTTTGGTGCTTCCCCAGGCGGCGGCGGCGGCGCCCGGGAGCCGGAGCCTTCGCGGCGT

CCACGTCCCTCCCCCGCTGCACCCCGCCCCGGCGCGAGAGGAGAGCGCGAGAGCCCCAGCCGCGGGCGGG

CGGGCGGCGAAGATGGCAGAGGCACCGGCTTCCCCGGCCCCGCTCTCTCCGCTCGAAGTGGAGCTGGACC

CGGAGTTCGAGCCCCAGAGCCGTCCGCGATCCTGTACGTGGCCCCTGCAAAGGCCGGAGCTCCAAGCGAG

CCCTGCCAAGCCCTCGGGGGAGACGGCCGCCGACTCCATGATCCCCGAGGAGGAGGACGATGAAGACGAC

GAGGACGGCGGGGGACGGGCCGGCTCGGCCATGGCGATCGGCGGCGGCGGCGGGAGCGGCACGCTGGGCT

CCGGGCTGCTCCTTGAGGACTCGGCCCGGGTGCTGGCACCCGGAGGGCAAGACCCCGGGTCTGGGCCAGC

CACCGCGGCGGGCGGGCTGAGCGGGGGTACACAGGCGCTGCTGCAGCCTCAGCAACCGCTGCCACCGCCG

CAGCCGGGGGCGGCTGGGGGCTCCGGGCAGCCGAGGAAATGTTCGTCGCGGCGGAACGCCTGGGGAAACC

TGTCCTACGCGGACCTGATCACCCGCGCCATCGAGAGCTCCCCGGACAAACGGCTCACTCTGTCCCAGAT

CTACGAGTGGATGGTGCGTTGCGTGCCCTACTTCAAGGATAAGGGCGACAGCAACAGCTCTGCCGGCTGG

AAGAACTCCATCCGGCACAACCTGTCACTGCATAGTCGATTCATGCGGGTCCAGAATGAGGGAACTGGCA

AGAGCTCTTGGTGGATCATCAACCCTGATGGGGGGAAGAGCGGAAAAGCCCCCCGGCGGCGGGCTGTCTC

CATGGACAATAGCAACAAGTATACCAAGAGCCGTGGCCGCGCAGCCAAGAAGAAGGCAGCCCTGCAGACA

GCCCCCGAATCAGCTGACGACAGTCCCTCCCAGCTCTCCAAGTGGCCTGGCAGCCCCACGTCACGCAGCA

GTGATGAGCTGGATGCGTGGACGGACTTCCGTTCACGCACCAATTCTAACGCCAGCACAGTCAGTGGCCG

CCTGTCGCCCATCATGGCAAGCACAGAGTTGGATGAAGTCCAGGACGATGATGCGCCTCTCTCGCCCATG

CTCTACAGCAGCTCAGCCAGCCTGTCACCTTCAGTAAGCAAGCCGTGCACGGTGGAACTGCCACGGCTGA

CTGATATGGCAGGCACCATGAATCTGAATGATGGGCTGACTGAAAACCTCATGGACGACCTGCTGGATAA

CATCACGCTCCCGCCATCCCAGCCATCGCCCACTGGGGGACTCATGCAGCGGAGCTCTAGCTTCCCGTAT

ACCACCAAGGGCTCGGGCCTGGGCTCCCCAACCAGCTCCTTTAACAGCACGGTGTTCGGACCTTCATCTC

TGAACTCCCTACGCCAGTCTCCCATGCAGACCATCCAAGAGAACAAGCCAGCTACCTTCTCTTCCATGTC

ACACTATGGTAACCAGACACTCCAGGACCTGCTCACTTCGGACTCACTTAGCCACAGCGATGTCATGATG

ACACAGTCGGACCCCTTGATGTCTCAGGCCAGCACCGCTGTGTCTGCCCAGAATTCCCGCCGGAACGTGA

TGCTTCGCAATGATCCGATGATGTCCTTTGCTGCCCAGCCTAACCAGGGAAGTTTGGTCAATCAGAACTT

GCTCCACCACCAGCACCAAACCCAGGGCGCTCTTGGTGGCAGCCGTGCCTTGTCGAATTCTGTCAGCAAC

ATGGGCTTGAGTGAGTCCAGCAGCCTTGGGTCAGCCAAACACCAGCAGCAGTCTCCTGTCAGCCAGTCTA

TGCAAACCCTCTCGGACTCTCTCTCAGGCTCCTCCTTGTACTCAACTAGTGCAAACCTGCCCGTCATGGG

CCATGAGAAGTTCCCCAGCGACTTGGACCTGGACATGTTCAATGGGAGCTTGGAATGTGACATGGAGTCC

ATTATCCGTAGTGAACTCATGGATGCTGATGGGTTGGATTTTAACTTTGATTCCCTCATCTCCACACAGA

ATGTTGTTGGTTTGAACGTGGGGAACTTCACTGGTGCTAAGCAGGCCTCATCTCAGAGCTGGGTGCCAGG

CTGAAGGATCACTGAGGAAGGGGAAGTGGGCAAAGCAGACCCTCAAACTGACACAAGACCTACAGAGAAA

ACCCTTTGCCAAATCTGCTCTCAGCAAGTGGACAGTGATACCGTTTACAGCTTAACACCTTTGTGAATCC

CACGCCATTTTCCTAACCCAGCAGAGACTGTTAATGGCCCCTTACCCTGGGTGAAGCACTTACCCTTGGA

ACAGAACTCTAAAAAGTATGCAAAATCTTCCTTGTACAGGGTGGTGAGCCGCCTGCCAGTGGAGGACAGC

ACCCCTCAGCACCACCCACCCTCATTCAGAGCACACCGTGAGCCCCCGTCGGCCATTCTGTGGTGTTTTA

ATATTGCGATGGTTTATGGGACGTTTTAAGTGTTGTTCTTGTGTTTGTTTTCCTTTGACTTTCTGAGTTT

TTCACATGCATTAACTTGCGGTATTTTTCTGTTAAAATGTTAACCGTCCTTCCCCTAGCAAATTTAAAAA

CAGAAAGAAAATGTTGTACCAGTTACCATTCCGGGTTCGAGCATCACAAGCTTTTGAGCGCATGGAACTC

CATAAACTAACAAATTACATAAACTAAAGGGGGATTTTCTTTCTTCTTTTGTTTGGTAGAAAATTATCCT

TTTCTAAAAACTGAACAATGGCACAATTGTTTGCTATGTGCACCCGTCCAGGACAGAACCGTGCATAGGC

AAAAGGAGTGGAGCACAGCGTCCGGCCCAGTGTGTTTCCGGTTCTGAGTCAGGGTGATCTGTGGACGGGA

CCCCAGCACCAAGTCTACGGGTGCCAGATCAGTAGGGCCTGTGATTTCCTGTCAGTGTCCTCAGCTAATG

TGAACAGTGTTGGTCTGCTGGTTAGAAACTAGAATATTGATATTTTCAGGAAAGAAATCAGCTCAGCTCT

CCACTCATTGCCAAATGTCACTAAAGGGTTTAGTTTTAAGGAGAAAGAAAAGGAAAAAAAAAAAAAACAA

AAAAGTCCTGTTTTGCTTTGCAGAACAAATGAACTTACAGGTGAGCATTAAGCTTGCAGTGAGAAATGTG

CGAAGAGTAAAAACCCAAGTCAATGCTGAGGCAGTTCTAACTTCACTGTTTTCCTAAATACACATCCTTG

ATTATTTTCAGCCTTGCTATATAATCTGATCTGCTAGAAGTGTATGAGTGAGAGGCAATAGCATACAAAC

TGATTTTTTAAATATAAGCTTAGGTTGTAATTGTACAAGTGACTCAATGGAAGTACAAAATAGGGCAGTT

TTAACTTTTTTTTCTGCTTCTATGGATTTCATTTTGTTGTGTTTTCAAAAAGTTATGGTGCTGTATAGGT

GCTTTCTGTTTAACCTGGAAAGTGTGATTATATTCGTTACCTTCTTTGGTAGACGGAATAGTTGGGACCA

CCTTTGGTACATAAGAAATTGGTATAACGATGCTCTGATTAGCACAGTATATGCATACTTCTCCAAAGTG

ATATATGAAGACTCTTTTCTTTGCATAAAAAGCATTAGGCATATAAATGTATAAATATATTTTATCATGT

ACAGTACAAAAATGGAACCTTATGCATGGGCCTTAGGAATACAGGCTAGTATTTCAGCACAGACTTCCCT

GCTTGAGTTCTTGCTGATGCTTGCACCGTGACAGTGGGCACCAACACAGACGTGCCACCCAACCCCCTGC

ACACACCACCGGCCACCAGGGGCCCCCTTGTGCGCCTTGGCTTTATAACTCCTCTGGGGGTGATATTGGT

GGTGATCACAGCTCCTAGCATAATGAGAGTTCCATTTGGTATTGTCACACGTCTCCTGCCTCGCTTGGGT

TGCCATGTTTGAGCGATGGCCCTGTTGATTTCACCCTGCCTTTTACTGAATCTGTAAATTGTTGTGCAAT

TGTGGTTATAGTAGACTGTAGCACATTGCCTTTTCTAAACTGCTACATGTTTATAATCTTCATTTTTAAA

GTATGTGTAATTTTTTTAAGTATGTATTCTATTCATATGGTCTGCTTGTCAGTGAGCCAGACTTGCTTAC

TATATTCCTTTATAATAATGCTAGCCACTTCCTGGATTCTTTAGTAATGTGCTGTATGCAAGAACTTTCC

AGTAGCAGTGAAGGAGGGTTGCCTCTCCAAGCTTCCTAAGGGATGCTGCCCTGTGTGGGGATGCATTGCA

GAGGCACTAGTAGCATGGGGGCTAGAGTGGGGAGCGAGATGTAAAAGGGTGGGGGGATAGGAGAATTCCA

GAGTGCTTCCAGCATTAGGGTCCTGAGAACTTCTGAGTTCAGAGAAACATGCAAAGTGACTAACAAAATA

GCTACTTACCTTTGCAGTTTTACAGACCCTGGGAGCTGCTTTGGGAGTGAGAAAGGCAACCCTCCAATGT

GTTTCAACTTTAAAATGTTGAATTCTTTTCAGACATGGTATCTCATTTATTCTCCTTTTCTAGCGTTTGT

TGAATTTCAGGCAGAATGTCTTACAGAATGTCCTAGAACCAGATTATCATTTAATCTGAAACAGCTGAGG

AAGGGACAGAGAAGGTACAAGGGCAAGGCAGCACAAAACAGATCAGGAGAATGAAGAGGGAATGCTTTGG

TTTTTTGTTTTGTTTTGTTTTTTCTTTTTCAAGTAACTAAAACAGCATCTACATGTAGAGTGTTGTGGAG

AGCTGAGACCAGGGTAAAGTCAAGTGCAGCATCAGTACTGCGAGACCCACCAGCCCCTGGAGAGGGTCAG

CCGAGAATCTGGTAGTGAAGCCTGTCTAGGGTCCCGGCACCCTCACCCTCAGCCACCTGCAGAGAGGCCA

GGGCCCCAGAGACTAGCCTGGTTCTGAAGTGGGCAGGGGTGCTGCCAGAGCCCTCTGCCCCTTATGTTGA

GACCCTGCTTTCAGGACAGGCCAGCCGTTGGCCACCATGTCACATTCTGAGTGAGTGTCACAGGTCCCTA

ACAATAATTTTCTGATCTGGAGCATATCAGCAGAATGCTTAGCCTCAAGGGGCCTGGCAGCTGTAATGTT

TGATTTATGATGAGAACTATCCGAGGCCACCCTTGGCCTCTAAATAAGCTGCTCTAGGGAGCCGCCTACT

TTTTGATGAGAAATTAGAAGAGTACCTAATGTTGAAAACATGACATGCGCTCTTGGGATCTGCTGTTCTC

TCCAGGGCTCCAGAACCTGATACCTGTTACCAAAGCTAGGAAAGAGCTTTATCACAAGCCTTCACTGTCC

TGGCATGAGAACTGGCTGCCAGGCTCAGTGTACCCCATTAACTGTGAATGAATCTGAGCTTGGTTTCCTT

TATTGCTTCCTCTGCAATATGATTGCTGAAACACATTTTAAAAATTCAGAAGCTTGTCACTCCTGTTAAT

GGGAGGATCAGTCACACATGTGTAGTACAAGGCGGACTTTGTGTTTGTTTTTGGTGTTAATTTTTAGCAT

TGTGTGTGTTGCTTCCCCACCCTGAGGAGAGGACACCATGGCTTACTACTCAGGACAAGTATGCCCCGCT

CAGGGTGTGATTTCAGGTGGCTTCCAAACTTGTACGCAGTTTAAAGATGGTGGGGACAGACTTTGCCTCT

ACCTAGTGAACCCCACTTAAAGAATAAGGAGCATTTGAATCTCTTGGAAAAGGCCATGAAGAATAAAGCA

GTCAAAAAGAAGTCCTCCATGTTGGTGCCAAGGACTTGCGAGGGGAAATAAAAATGTTATCCAGCCTGAC

CAACATGGAGAAACCCCGTCTCCATTAAAAATACAAAATTAGCCTGGCATGGTGGCGCATGCCTGTAATC

CCAGCTACTCTGGAGGCTGAGGCAGGAGAATCGCTTGAACCCAGGAGGCGGAGGTCGCAGTGAGCCGAGA

TCATGCCAGTGCACTCCAGCCTGGGTAACAAGAGTGAAACTCCGTGTCAAAAAAAAAAAAAAAAATGTTAC

TCATCCTCTCTGAAAGCAAAAAGGAAACCCTAACAGCTCTGAACTCTGGTTTTATTTTTCTTGCTGTATT

TGGGTGAACATTGTATGATTAGGCATAATGTTAAAAAAAAAAAATTTTTTTTTGGTAGAAATGCAATCACC

AGTAAAGAGGTACGAAAAAGCTAGCCTCTCTCAGAGACCGGGGAGGCAGAGTACTACTAGAGGAAGTGAA

GTTCTGATGGAATCATGCCTGTCAAATGAGGTCTTGAAGCGGATGCCCAAATAAAAGAGTATATTTTATC

TAAATCTTAAGTGGGTAACATTTTATGCAGTTTAAATGAATGGAATATTTTCCTCTTGTTTAGTTGTATC

TGTTTGTATTTTTCTTTGATGAATGATTGGTCATGAGGCCTCTTGCCACACTCCAGAAATACGTGTGCGG

CTGCTTTTAAGAACTATGTGTCTGGTCACTTATTTCTCTAAAATTATCTCATTGCCTGGCAATCAGTCTT

CTCTTGTATACTTGTCCTAGCACATTATGTACATGGGAAATGTAAACAAATGTGAAGGAGGACCAGAAAA

ATTAGTTAATATTTAAAAAAATGTATTGTGCATTTTGGCTTCACATGTTTAACTTTTTTTAAGAAAAAAG

TTGCATGAATGGAAAAAAAAAATCTGTATACAGTATCTGTAAAAACTATCTTATCTGTTTCAATTCCTTGC

TCATATCCCATATAATCTAGAACTAAATATGGTGTGTGGCCATATTTAAACACCTGAGAGTCAAGCAGTT

GAGACTTTGATTTGAAGCACCTCATCCTTCTTTCAATGCGAACACTATCATATGGCATTCTTACTGAGGA

TTTTGTCTAACCATATGTTGCCATGAATTAACTCTGCCGCCTTTCTTAAGGATCAAAACCAGTTTGATTT

GGGAATCTTCCCCTTTCCAAATGAAATAGAGATGCAGTACTTAACTTTCCTTGGTGTTTGTAGATATTGC

CTTGTGTATTCCACTTAAAACCGTAATCTAGTTTGTAAAAGAGATGGTGACGCATGTAAATAAAGCATCA

GTGACACTCTATCTTA

>NM_000152.5 Homo sapiens alpha glucosidase (GAA), transcript variant 1, mRNA

CGACCCCGGAGTCTCCGCGGGCGGCCAGGGCGCGCGTGCGCGGAGGTGAGCCGGGCCGGGGCTGCGGGGC

TTCCCTGAGCGCGGGCCGGGTCGGTGGGGCGGTCGGCTGCCCGCGCGGCCTCTCAGTTGGGAAAGCTGAG

GTTGTCGCCGGGGCCGCGGGTGGAGGTCGGGGATGAGGCAGCAGGTAGGACAGTGACCTCGGTGACGCGA

AGGACCCCGGCCACCTCTAGGTTCTCCTCGTCCGCCCGTTGTTCAGCGAGGGAGGCTCTGCGCGTGCCGC

AGCTGACGGGGAAACTGAGGCACGGAGCGGGCCTGTAGGAGCTGTCCAGGCCATCTCCAACCATGGGAGT

GAGGCACCCGCCCTGCTCCCACCGGCTCCTGGCCGTCTGCGCCCTCGTGTCCTTGGCAACCGCTGCACTC

CTGGGGCACATCCTACTCCATGATTTCCTGCTGGTTCCCCGAGAGCTGAGTGGCTCCTCCCCAGTCCTGG

AGGAGACTCACCCAGCTCACCAGCAGGGAGCCAGCAGACCAGGGCCCCGGGATGCCCAGGCACACCCCGG

CCGTCCCAGAGCAGTGCCCACACAGTGCGACGTCCCCCCCAACAGCCGCTTCGATTGCGCCCCTGACAAG

GCCATCACCCAGGAACAGTGCGAGGCCCGCGGCTGTTGCTACATCCCTGCAAAGCAGGGGCTGCAGGGAG

CCCAGATGGGGCAGCCCTGGTGCTTCTTCCCACCCAGCTACCCCAGCTACAAGCTGGAGAACCTGAGCTC

CTCTGAAATGGGCTACACGGCCACCCTGACCCGTACCACCCCCACCTTCTTCCCCAAGGACATCCTGACC

CTGCGGCTGGACGTGATGATGGAGACTGAGAACCGCCTCCACTTCACGATCAAAGATCCAGCTAACAGGC

GCTACGAGGTGCCCTTGGAGACCCCGCATGTCCACAGCCGGGCACCGTCCCCACTCTACAGCGTGGAGTT

CTCCGAGGAGCCCTTCGGGGTGATCGTGCGCCGGCAGCTGGACGGCCGCGTGCTGCTGAACACGACGGTG

GCGCCCCTGTTCTTTGCGGACCAGTTCCTTCAGCTGTCCACCTCGCTGCCCTCGCAGTATATCACAGGCC

TCGCCGAGCACCTCAGTCCCCTGATGCTCAGCACCAGCTGGACCAGGATCACCCTGTGGAACCGGGACCT

TGCGCCCACGCCCGGTGCGAACCTCTACGGGTCTCACCCTTTCTACCTGGCGCTGGAGGACGGCGGGTCG

GCACACGGGGTGTTCCTGCTAAACAGCAATGCCATGGATGTGGTCCTGCAGCCGAGCCCTGCCCTTAGCT

GGAGGTCGACAGGTGGGATCCTGGATGTCTACATCTTCCTGGGCCCAGAGCCCAAGAGCGTGGTGCAGCA

GTACCTGGACGTTGTGGGATACCCGTTCATGCCGCCATACTGGGGCCTGGGCTTCCACCTGTGCCGCTGG

GGCTACTCCTCCACCGCTATCACCCGCCAGGTGGTGGAGAACATGACCAGGGCCCACTTCCCCCTGGACG

TCCAGTGGAACGACCTGGACTACATGGACTCCCGGAGGGACTTCACGTTCAACAAGGATGGCTTCCGGGA

CTTCCCGGCCATGGTGCAGGAGCTGCACCAGGGCGGCCGGCGCTACATGATGATCGTGGATCCTGCCATC

AGCAGCTCGGGCCCTGCCGGGAGCTACAGGCCCTACGACGAGGGTCTGCGGAGGGGGGTTTTCATCACCA

ACGAGACCGGCCAGCCGCTGATTGGGAAGGTATGGCCCGGGTCCACTGCCTTCCCCGACTTCACCAACCC

CACAGCCCTGGCCTGGTGGGAGGACATGGTGGCTGAGTTCCATGACCAGGTGCCCTTCGACGGCATGTGG

ATTGACATGAACGAGCCTTCCAACTTCATCAGGGGCTCTGAGGACGGCTGCCCCAACAATGAGCTGGAGA

ACCCACCCTACGTGCCTGGGGTGGTTGGGGGGACCCTCCAGGCGGCCACCATCTGTGCCTCCAGCCACCA

GTTTCTCTCCACACACTACAACCTGCACAACCTCTACGGCCTGACCGAAGCCATCGCCTCCCACAGGGCG

CTGGTGAAGGCTCGGGGGACACGCCCATTTGTGATCTCCCGCTCGACCTTTGCTGGCCACGGCCGATACG

CCGGCCACTGGACGGGGGACGTGTGGAGCTCCTGGGAGCAGCTCGCCTCCTCCGTGCCAGAAATCCTGCA

GTTTAACCTGCTGGGGGTGCCTCTGGTCGGGGCCGACGTCTGCGGCTTCCTGGGCAACACCTCAGAGGAG

CTGTGTGTGCGCTGGACCCAGCTGGGGGCCTTCTACCCCTTCATGCGGAACCACAACAGCCTGCTCAGTC

TGCCCCAGGAGCCGTACAGCTTCAGCGAGCCGGCCCAGCAGGCCATGAGGAAGGCCCTCACCCTGCGCTA

CGCACTCCTCCCCCACCTCTACACACTGTTCCACCAGGCCCACGTCGCGGGGGAGACCGTGGCCCGGCCC

CTCTTCCTGGAGTTCCCCAAGGACTCTAGCACCTGGACTGTGGACCACCAGCTCCTGTGGGGGGAGGCCC

TGCTCATCACCCCAGTGCTCCAGGCCGGGAAGGCCGAAGTGACTGGCTACTTCCCCTTGGGCACATGGTA

CGACCTGCAGACGGTGCCAGTAGAGGCCCTTGGCAGCCTCCCACCCCCACCTGCAGCTCCCCGTGAGCCA

GCCATCCACAGCGAGGGGCAGTGGGTGACGCTGCCGGCCCCCCTGGACACCATCAACGTCCACCTCCGGG

CTGGGTACATCATCCCCCTGCAGGGCCCTGGCCTCACAACCACAGAGTCCCGCCAGCAGCCCATGGCCCT

GGCTGTGGCCCTGACCAAGGGTGGGGAGGCCCGAGGGGAGCTGTTCTGGGACGATGGAGAGAGCCTGGAA

GTGCTGGAGCGAGGGGCCTACACACAGGTCATCTTCCTGGCCAGGAATAACACGATCGTGAATGAGCTGG

TACGTGTGACCAGTGAGGGAGCTGGCCTGCAGCTGCAGAAGGTGACTGTCCTGGGCGTGGCCACGGCGCC

CCAGCAGGTCCTCTCCAACGGTGTCCCTGTCTCCAACTTCACCTACAGCCCCGACACCAAGGTCCTGGAC

ATCTGTGTCTCGCTGTTGATGGGAGAGCAGTTTCTCGTCAGCTGGTGTTAGCCGGGCGGAGTGTGTTAGT

CTCTCCAGAGGGAGGCTGGTTCCCCAGGGAAGCAGAGCCTGTGTGCGGGCAGCAGCTGTGTGCGGGCCTG

GGGGTTGCATGTGTCACCTGGAGCTGGGCACTAACCATTCCAAGCCGCCGCATCGCTTGTTTCCACCTCC

TGGGCCGGGGCTCTGGCCCCCAACGTGTCTAGGAGAGCTTTCTCCCTAGATCGCACTGTGGGCCGGGGCC

CTGGAGGGCTGCTCTGTGTTAATAAGATTGTAAGGTTTGCCCTCCTCACCTGTTGCCGGCATGCGGGTAG

TATTAGCCACCCCCCTCCATCTGTTCCCAGCACCGGAGAAGGGGGTGCTCAGGTGGAGGTGTGGGGTATG

CACCTGAGCTCCTGCTTCGCGCCTGCTGCTCTGCCCCAACGCGACCGCTGCCCGGCTGCCCAGAGGGCTG

GATGCCTGCCGGTCCCCGAGCAAGCCTGGGAACTCAGGAAAATTCACAGGACTTGGGAGATTCTAAATCT

TAAGTGCAATTATTTTTAATAAAAGGGGCATTTGGAATCAG

>NM_007278.2 Homo sapiens GABA type A receptor-associated protein (GABARAP), mRNA

GCAAATTCGTGGATCGCTCCGCTGAATCCGCCCGCGCGTCGCCGCCGTCGTCGCCGCCCCCCGTCCCGGC

CCCCCTGGGTTCCCTCAGCCCAGCCCTGTCCAGCCCGGTTCCCGGGAGGATGAAGTTCGTGTACAAAGAA

GAGCATCCGTTCGAGAAGCGCCGCTCTGAGGGCGAGAAGATCCGAAAGAAATACCCGGACCGGGTGCCGG

TGATAGTAGAAAAGGCTCCCAAAGCTCGGATAGGAGACCTGGACAAAAAGAAATACCTGGTGCCTTCTGA

TCTCACAGTTGGTCAGTTCTACTTCTTGATCCGGAAGCGAATTCATCTCCGAGCTGAGGATGCCTTGTTT

TTCTTTGTCAACAATGTCATTCCACCCACCAGTGCCACAATGGGTCAGCTGTACCAGGAACACCATGAAG

AAGACTTCTTTCTCTACATTGCCTACAGTGACGAAAGTGTCTACGGTCTGTGAAGCTGCTGCCCCTGAGC

TGGAGGGGGGTCTCATTCTACAAAGAGAGAGGTGGCCCCCCTTTCTTGACCTCCTCCTCCTTCAAGCTCA

AACACCACCTCCCTTATTCAGGACCGGCACTTCTTAATGTTTGTGGCTTTCTCTCCAGCCTCTCTTAGGA

GGGGTAATGGTGGAGTTGGCATCTTGTAACTCTCCTTTCTCCTTTCTTCCCCTTTCTCTGCCCGCCTTTC

CCATCCTGCTGTAGACTTCTTGATTGTCAGTCTGTGTCACATCCAGTGATTGTTTTGGTTTCTGTTCCCT

TTCTGACTGCCCAAGGGGCTCAGAACCCCAGCAATCCCTTCCTTTCACTACCTTCTTTTTTGGGGGTAGT

TGGAAGGGACTGAAATTGTGGGGGGAAGGTAGGAGGCACATCAATAAAGAGGAAACCACCAAGCTGAACT

GAATTTTGCCTTGTGTTGCTCCCCTCGTCCCGCTGATTTTAAGTCTTTCCAAGGTGTCAGTGGGTTTCAG

TGGTGGGGAAAGAAGAGTACTGGGTACAAGCTGGAGGGATAGAAGTATATTTTGGTTTATTCTGTTCATG

TTGGGCTTTTCCCTGTCTGCAAAAAGAGGGTGCTTTTGTTGTGATGGAATGGAATACTGAGGATTATTTC

TTGAAACTTTAGTTTTATAACACGCATGTGAAACTAAATGTTAAAAATGCTCATGTAAAAAAAAATTTTT

TTTTACTGTGGGTTCCTGTGGAGAAAGTTCCGAAGTACCTGCTTTAGGTGAACATCCACATTTGCTAGAA

CATTCTAACTAAGATATTTTCATGTGTGCAAGCTAGTAAAACGGCTGTTCTCAGTTGCA

>NM_001363598.2 Homo sapiens GABA type A receptor associated protein like 1 (GABARAPL1), transcript variant 1, mRNA

GTTTTTGTGCTCCCAGCTCTAGCGAAAAGCCGCCGGTATTTCTCCATCTGGCTCTCCTCTACCTCCAGGC

AGGCTCACCCGAGATCCCCGCCCCGAACCCCCCCTGCACACTCGGCCCAGCGCTGTTGCCCCCGGAGCGG

ACGTTTCTGCAGCTATTCTGAGCACACCTTGACGTCGGCTGAGGGAGCGGGACAGGGTCAGCGGCGAAGG

AGGCAGGCCCCGCGCGGGGATCTCGGAAGCCCTGCGGTGCATCATGAAGTTCCAGTACAAGGAGGACCAT

CCCTTTGAGTATCGGAAAAAGGAAGGAGAAAAGATCCGGAAGAAATATCCGGACAGGGTCCCCGTGATTG

TAGAGAAGGCTCCAAAAGCCAGGGTGCCTGATCTGGACAAGAGGAAGTACCTAGTGCCCTCTGACCTTAC

TGTTGGCCAGTTCTACTTCTTAATCCGGAAGAGAATCCACCTGAGACCTGAGGACGCCTTATTCTTCTTT

GTCAACAACACCATCCCTCCCACCAGTGCTACCATGGGCCAACTGTATGAGGTAATGGTTCTGGTTGCAC

AATACTGGATGCCGTCCAGTGCAGTCTGGCATCCTCTAGCCCTTGTTCTAGATGCGTTGATAACACATCT

GAGAAGTGGGGCAGAAGGTGTTATTTATCCGGATCCTCTTACATATGGCAGTGTAAGGCTCTGAGAGATT

AGATACCTCTTGTTTTTTAGGGATTAGCCACTCTACTAGATTTAATCCTTCTGAAATCTTGTCTGACTAT

AGTCTTGTCTGACTATAGTGTTTTAATGCTGGACTGTTTATTGAAACAGCAGGAAGTGATTCCTGATAGC

AAAAGTTCCAAAGCGTCAGCCCATCGCAGTTCCTGCTATTCTGGTTGGTAGCATGTAATTTGTAATGCAT

TCAGAATCCAGTTCATTATTTTACTCCCATTTAGCAACAGATTGAGAAAGCACGT

>NM_007285.7 Homo sapiens GABA type A receptor associated protein like 2 (GABARAPL2), mRNA

AGTCGCCGCCGTCGCTGCCGCTGCCGCTGCCGCCGTCGTTGTTGTTGTGCTCGGTGCGCTGAGCTCCGCG

GCTCCGCGAGCCGGTTCCGTCCCCTTCCCGCCGCCGCCATGAAGTGGATGTTCAAGGAGGACCACTCGCT

GGAACACAGATGCGTGGAGTCCGCGAAGATTCGAGCGAAATATCCCGACAGGGTTCCGGTGATTGTGGAA

AAGGTCTCAGGCTCTCAGATTGTTGACATTGACAAACGGAAGTACTTGGTTCCATCTGATATCACTGTGG

CTCAGTTCATGTGGATCATCAGGAAAAGGATCCAGCTTCCTTCTGAAAAGGCGATCTTCCTGTTTGTGGA

TAAGACAGTCCCACAGTCCAGCCTAACTATGGGACAGCTTTACGAGAAGGAAAAAGATGAAGATGGATTC

TTATATGTGGCCTACAGCGGAGAGAACACTTTTGGCTTCTGAGGGCCATTGCTGGGCTAGGTGCACCGTA

ACTGCTTGTGTATCTTGTAAATAGCCAGCCATTTTCAGTTATTATACCAGAACCTCTTCACATAGACCTA

TTAGTGCATTTGTAACTGGATTTATTTCTTAATATATTGGAAGGTTTTGTTTCCTTAGACTAGTAAATTA

TCATACAGAGTTTTATTTTGAGTTTTTCTTTTTGTGCATTGTCCTCATGCCTGTATTCTCCAGGAAACTT

GTCCTTCTGGAAATCATATTGAATGATATTTCTATATCGAAGTGAGGTAGGTGCGGTATTAAAGTGAAAG

GGAAGGTGATGCATTTATTCTGGGTTATGCTTGAAGTGTTAGATGGCTAAGTATTAAAATTATCCAAATT

AAATCCTTAGCAGTCAGAACACTTGCTTCACTAGAATATGCCAACTGCCAATCATGTTGGACTGAGCTAA

TTTGTTCCTCTTTCTGAAACTATTAAGGTAAATAATTAACAATAAAAATTCTCTTATAAAGGCA

>NM_002046.7 Homo sapiens glyceraldehyde-3-phosphate dehydrogenase (GAPDH), transcript variant 1, mRNA

GCTCTCTGCTCCTCCTGTTCGACAGTCAGCCGCATCTTCTTTTGCGTCGCCAGCCGAGCCACATCGCTCA

GACACCATGGGGAAGGTGAAGGTCGGAGTCAACGGATTTGGTCGTATTGGGCGCCTGGTCACCAGGGCTG

CTTTTAACTCTGGTAAAGTGGATATTGTTGCCATCAATGACCCCTTCATTGACCTCAACTACATGGTTTA

CATGTTCCAATATGATTCCACCCATGGCAAATTCCATGGCACCGTCAAGGCTGAGAACGGGAAGCTTGTC

ATCAATGGAAATCCCATCACCATCTTCCAGGAGCGAGATCCCTCCAAAATCAAGTGGGGCGATGCTGGCG

CTGAGTACGTCGTGGAGTCCACTGGCGTCTTCACCACCATGGAGAAGGCTGGGGCTCATTTGCAGGGGGG

AGCCAAAAGGGTCATCATCTCTGCCCCCTCTGCTGATGCCCCCATGTTCGTCATGGGTGTGAACCATGAG

AAGTATGACAACAGCCTCAAGATCATCAGCAATGCCTCCTGCACCACCAACTGCTTAGCACCCCTGGCCA

AGGTCATCCATGACAACTTTGGTATCGTGGAAGGACTCATGACCACAGTCCATGCCATCACTGCCACCCA

GAAGACTGTGGATGGCCCCTCCGGGAAACTGTGGCGTGATGGCCGCGGGGCTCTCCAGAACATCATCCCT

GCCTCTACTGGCGCTGCCAAGGCTGTGGGCAAGGTCATCCCTGAGCTGAACGGGAAGCTCACTGGCATGG

CCTTCCGTGTCCCCACTGCCAACGTGTCAGTGGTGGACCTGACCTGCCGTCTAGAAAAACCTGCCAAATA

TGATGACATCAAGAAGGTGGTGAAGCAGGCGTCGGAGGGCCCCCTCAAGGGCATCCTGGGCTACACTGAG

CACCAGGTGGTCTCCTCTGACTTCAACAGCGACACCCACTCCTCCACCTTTGACGCTGGGGCTGGCATTG

CCCTCAACGACCACTTTGTCAAGCTCATTTCCTGGTATGACAACGAATTTGGCTACAGCAACAGGGTGGT

GGACCTCATGGCCCACATGGCCTCCAAGGAGTAAGACCCCTGGACCACCAGCCCCAGCAAGAGCACAAGA

GGAAGAGAGAGACCCTCACTGCTGGGGAGTCCCTGCCACACTCAGTCCCCCACCACACTGAATCTCCCCT

CCTCACAGTTGCCATGTAGACCCCTTGAAGAGGGGAGGGGCCTAGGGAGCCGCACCTTGTCATGTACCAT

CAATAAAGTACCCTGTGCTCAACCA

>NM_006496.4 Homo sapiens G protein subunit alpha i3 (GNAI3), mRNA

GACGGAGAGGGCCACCGCCCAGCAATAGACGGTGCCTCAGCCTGCCGAGCCGCAGTTTCCGTGGTGTGAG

TGAGTCCGGGCCCGTGTCCCCTCTCCCGCCGCCGCCATGGGCTGCACGTTGAGCGCCGAAGACAAGGCGG

CAGTGGAGCGAAGCAAGATGATCGACCGCAACTTACGGGAGGACGGGGAAAAAGCGGCCAAAGAAGTGAA

GCTGCTGCTACTCGGTGCTGGAGAATCTGGTAAAAGCACCATTGTGAAACAGATGAAAATCATTCATGAG

GATGGCTATTCAGAGGATGAATGTAAACAATATAAAGTAGTTGTCTACAGCAATACTATACAGTCCATCA

TTGCAATCATAAGAGCCATGGGACGGCTAAAGATTGACTTTGGGGAAGCTGCCAGGGCAGATGATGCCCG

GCAATTATTTGTTTTAGCTGGCAGTGCTGAAGAAGGAGTCATGACTCCAGAACTAGCAGGAGTGATTAAA

CGGTTATGGCGAGATGGTGGGGTACAAGCTTGCTTCAGCAGATCCAGGGAATATCAGCTCAATGATTCTG

CTTCATATTATCTAAATGATCTGGATAGAATATCCCAGTCTAACTACATTCCAACTCAGCAAGATGTTCT

TCGGACGAGAGTGAAGACCACAGGCATTGTAGAAACACATTTCACCTTCAAAGACCTATACTTCAAGATG

TTTGATGTAGGTGGCCAAAGATCAGAACGAAAAAAGTGGATTCACTGTTTTGAGGGAGTGACAGCAATTA

TCTTCTGTGTGGCCCTCAGTGATTATGACCTTGTTCTGGCTGAGGACGAGGAGATGAACCGAATGCATGA

AAGCATGAAACTGTTTGACAGCATTTGTAATAACAAATGGTTTACAGAAACTTCAATCATTCTCTTCCTT

AACAAGAAAGACCTTTTTGAGGAAAAAATAAAGAGGAGTCCGTTAACTATCTGTTATCCAGAATACACAG

GTTCCAATACATATGAAGAGGCAGCTGCCTATATTCAATGCCAGTTTGAAGATCTGAACAGAAGAAAAGA

TACCAAGGAGATCTATACTCACTTCACCTGTGCCACAGACACGAAGAATGTGCAGTTTGTTTTTGATGCT

GTTACAGATGTCATCATTAAAAACAACTTAAAGGAATGTGGACTTTATTGAGAAGCATGGATGTTAGTGA

AAGTTACTACAGTGTGGAGTGTTGAGACCAGACACCTTTTGCTGTCTCATGGGGCAGCTACAAGCATGAA

CGGGACCAGGGAATGGCAGCAGCATGCAGAATCTTAGCACTCTTTAGCACAATATTTTGTATTAGGGAAC

TTTTAATTGACATGAGATGCTAAAGTCAGACATTGGAATTGGAAGAACTATAAAGTGTGATTCGATCGTC

AAGACATCACTTGGATTTCTTAATCTTAAATGCTTATGGAAGATGTGAAGTTGAGGTGCTGCATTCTAGA

ACTTCAATATGTAGCTTACTCTTTTTTTCCCCCCTTCTTAAACCACCAGTGGTTCATTTTTAAGGTTTTT

TCATCAAGAGAAGAATAACTTTACTAAATTTTATTTCTTTATTTGCAAAAGAATCTTTATTAAAACAAAC

AATCTTAACTATGCACATGATGTGACCAGATCATCTTGAAAATATTCCTCTTTAGTAGGAACTCTTTGTT

TTTAACTCTTGGTATGGTCAGAATATAATACTTCCATAATTACTTATAATTCTTTCCGGTTACTGGGGCT

ATAAATACAACTTTTTAAATGAAATTTATGTTATTATCCTGCTCAAAGTACCATTATGGTTTCCATATGG

TAATTACATTGGAAAGTTCTGCAATAAGATTCTAGTTCTCTCTTTTCTTTAAGCTTATGGTTGAAGGTTA

ACCTTGTTTGTGCAGATTACCAAATTACTGCTGATGTAGTATGATAAAGTCAGCTTCTTGGATACAGACA

CACCCAGGGCAGCTGCCAATCAGAAATGCAAATTGCTAAATTCCAAACAGAACCAGTGACTTTGCTGCTA

CATATTTACTAAATTGACCACTTTGATTCTTGCTTGTTTGTCTCAGTTATAGGGAGTTTTGTACATGGTG

CCTCTTGTTTTCCATCTTCATGGCCTTTTCTGTTGAGAAACATCTAAAGTGTATTAACAGTGCATGCTTT

TACTTTGTAAATGTGGTGCCAAATCCCTGTTTGCCATCGTTTTTACTGTACCGATGTTAACTGTAGTAAT

CCTTAGCCAGTATGTTCTTTTGCTGAAACTGTTGCATTTTGGGACTTTTTTCCACTTTGTCATGTGTACA

TTTTTAATCTGTTATAGTTGGCGGCAGTATTAAAATGGTGAGTAAAGAGCCCAGGTTTGCTCCTGTTTGT

AACTAGTCTTTTCTGGAAATACTGTCTTCTGTTTCCTTTTGCCTTTGCAGAATGTGCTTTAGCCTTTGTC

TGAGAATGGGTTTAGGTAGAAGGGTTTCCTGAAAGTCTAGTCTTTTGGTGTAATAGTATTGTTGAACCTT

AGGTTTTATGTTATATCTGCATATGAGTGATATGTGATCATGATTCATTTTGCAGATTTACATTTTGCTT

GTGTGGAAAGTTACGTTCACTTCAACCTACAGACCCTTTTGTATAATGTACAGCAAATGTCATTAAATAT

TGAATGCTCTATTGGGGGAAGACAAATGAAGAGAATGCATTTTGAGCATTTCAAATCAGTAGTTTGGGCA

GTGCCTTTTGGGGTCCAATCTTTTTGGATTGGATTCTATTTCATCTTTTGATGTGACTTTTCACTAGTTT

ACAAAAAAAAGGTTGGTGGTCAACAAAAAAAGCAAGTCATAGCAGTTCAGTGCCTTTGTTGGTAGTTTTT

AACTTTTTTTGTGATTCTTTTTGGAAAATCATAGCTTACAGATGGTATAACTGTATTATATAATGGAATT

TTCTTTAGGTGTGGGAAAACTTGATCTGAAAGAAAATTATCAGCTTCAATTGGCGATTGATTCAGTGCCC

ACAATGTAAACAGGGTTGGTAGTTGTTACTCATTTTGAATATACCTTTTCCTTATTGTATTCTGTAATAT

AGGATCCTGGAAATGAGACCTGGTGGAATATTATTTTCAACTGTAGTCTTTCTATACTGTGGACTAATGA

AATGGTGTTGTTGATAATCTAAAATAACCAATAATCCAAAAAAGTTCATTTTACTTTGAATTTGTATTTT

CATTTGAATATCTATCTGATATGTTAAATTTTTAGCCCTTCTGTTAAAGAAAAATAGCAGGTCCCCATTT

TTCTGCCTCTGTACCATTTACATTTGCTCTCTGTTTGTTAACATGAAGATCACTCTTATTTCACCATCCC

AAATAACTTATCTAAATGCACATTCCTTGAGGACACAGGGAGACAATTTAAGTCAGTAGTTTTTAATATG

ACTTAAGTAAAAATCACTTGGACCCATCTAAGGGATTGACTTTTGGCTGGTGGGCTGGCAATCGATTTTT

TTTTTTTTTTTTTTTTTTTGAGACGGAGTCTCGCTCTGTCGCCCAGGCTGGAGTGCAGTGGCGCGATCTC

GGCTTATTGCAAGCTCTGCCTCCCGGGTTCATGCTATTCTCCTGCTGGGACTACCAGGTTCCTGCCACCA

CGCCCAGCTAATTTTTTTGTATGTCTTAGTAGAGACGGGGTTTAACCGTGTTAGCCAGGATGGTCTTGAT

CCCCTGACCTCGTGATCTGCCTGCCTCAGCCTCCCAAAGTGCTGGGATTACAGGCGTGAGCCACCGCACC

TGGCTGGCAATTTATAATTTTTAAAAGGTGATTCTGATTACCAGCCAGGCTTGCAAATCACTGGTTTGTA

TTGAAATAAAAGATATTTTCTTGTAGCAGGGCTAAAATGTGGGATGAAACATTAAATCTACTAGCCTGAA

AAAAAACTTTGGCATTTGTAATCAAAGAAGCTGAAAGTTGCCTTGTGTGTTTCCTCTGAAGAATGCTGGA

GATTTGTGATGGATAATGTTTCAGAATTCAGGTGCCTATGCTTTGGACCATCAAAGAAGCCATTAGTCTC

TGTGTAAAGAAGAAGTATTCTTAATGGACAGTCCCCCCTAACCTTATAAATTCAGCTTTGCAACAGATTT

CACCTCCTAGCATGAAAATTCCAAAAACTTTAACAATTACTCATAAGTGGATCTTGAGATGACATTTTCA

TCTTGGACAGTTTGAATTCTAAATCTCTAGTTCACAGTGAATCGTTTTTTAAATTTTTACTGATGGATCG

TTATTTTTCACAAGGTACTACATTCTTACAATGTAAGACTAGATGGACCAAAATTTTAAAAAAAACTTCT

TGCCTTGTTTTTTTAATTTTCATATATATTCTATTTGTTGTATATACACATTAGACTATTTTAAAATAAC

AGCTGGTTTTTGTAATTCATTCATTCACCCTATATTCTTATATTCTGCTCATTATTCTCTCATTTATAGC

TGTGACATTCTTTGGACATTACTTGCCTGGCCTGATTAGTTACTTAATATAATTTATTCATTTGCTTAGC

ATGTGTGACATAGTTCTGGAATTTCCTGTTGCAGAACTCCTTGCTGTATTGCAGTCATATATAAGGTAAA

GTCAGTCTCTTGTTCCTATTAAAAAGCTACCATTGATTATTTTCCCTTTTTACATGTTCAAAAAAAAAAA

AATAAGCAACCCTCTACTCATTTGTATAAACCTAAAACGTCACTGTTTTCCTTGAGCGTTAGTACTTGTG

GGTTCTTTCCTTTTCATGTGTAATACTGATTTTAAGAGCAATGATCTTAGTTTGCCAGTGCCAGGTAATT

ATTGTGTTCTACTTTAGGCTTCGTAGTGCAAATCAGGGATCTGACCTGGATCAGCCTCAGCTCTTTACAT

AGCTTTATGATCATCATATTTTGTTGTATTGCTCCCAACAAGCACAAGAGAAAAAGGCAGGCTTCCTAGA

GACCTCAAGTTGAAAGTTGATTTTGCTGACAACTATCAGGGAGTAAAAGAAAGCCTTTTTCAGGCACTGG

AGAACTAACTTTAATGATGATCAAGGTCTTTATGCCTTTATCTTTTAGTTTTTAAGATCATTTCTCTCCC

TCCTTTCCCCCCAGTTTTCTCAACATTTATGAGTTAGTTCTGCTGAGAACAACATACTTCAGAACATGTC

AGGATAGGGCAGTTTTTTCTTGTTTTTCTTTTTTCTTTTTTTGAGACAGTCTTGCTCTGTTGCCCAGGCT

GGAGTACAGTGGCACGATCTCTGCTCACTGCAACCTTTGCCTCTTAGGTTCAAGCGATTCTTGTGCCTCA

GCCTCCCAAGTAGCTGTGATTACAGGCATGTGCCACCACATCTGGCTAACTTTTGTATTTCTAGTAGAGA

CAGGGTTTCACCATGTTAGCCAGGCTGGTCTCAAACTCCTGACCTCAAGTGATTTGCCTGCCTTGGCCTC

CCAAAGTGCTGAGATTACAGATGTGAACCACTCCACCCACTCAGGGCAGTTTTTTTTCTGATACCATATC

AGGCCTAGGCCTGGGCTGACTCAAATCTTCTCAGGAATAAGAGGTAAGAAGGAATGTTAAACAGCAGTAG

TTACCTGGGGTACATGGTTAATTTGATTTAGCAGATTCTTTGGCTAAACCCTAGTAAATTAAAATGTCTG

AGCCAGGTCATGGGAATATACATGTTTTTAATACATTCCTTAGGTGATTTTGATGTAGCCAATCTGGGCC

AACATTTGGGGACAACTGAGTAGGACCTAATACAGGTTGAATAGCCCTTATCCAAATCTCTTGGGACCAG

AAGTGTTTGAGAGTTCAGATTTTTGTGAGGTTTTGAAATATTTGCATTATATACTTATTGGTTCGGGATT

CCAAATCCAAAAATCTGAAATCCAAAATGCCCCAGTGTGTGCTTCCTTTGAGTGTTATGTTGTTACCCAA

AAAGCTTTTGATTTTGGAGCATTTTAGATTTTGGATTTTCAGATTTGAAATGCTCAATCTATATTAAAGA

TCAACACAGGCTGAGCACAGTGGCTCATGTCTGTAATCCCAGCACTTTGGGAGGCCGAGGTAAGCAGATC

ACTTGAGGTCAGGAGTTTGAGACCAGCTTGGCCAACATGGTGAAACCCCGTCTTTACTAAAAATACAAAA

ATTAGCCAGGCATGGTGGTGGATGCCTGTAATCCTAGCTACTCGGGAGGCTGAGGCAGGAGAATCGCTTG

AACCTGGGAGGCAGAGGTTGCAGTGAGCCGAGATCGCACCACTGAATTCCAGCCTGGGCGACAGAGTGAG

TCTGTTTTTTTTTTGTTTTTTTTTAAAGTCAACACAAATATTGAAAATAAGCAACTTCAGTGCACAAATA

AGCTCAAAATCACTAGTGAGTGATCTATTTTTTGCCAATAAGAAGTACCATTTGTACATGACTTAAAGTG

CCTTCCACCAAGAAAATTATTGAAAACTGTTGGGTCACATAACATTCCTACACTTTGGATACAGAGTATT

TAGTTTTATACAAAATTAAGACAACAAGAAACCTAGGATGTCCAAACATTCATTTCATTTATTTTACTGC

ATTTATTTATGCAGTGAAAATTGAAAGATACACCAAATTAAAGATAAAACCCTGTGACAGATAACAGTAT

ATGTAATAACATGAATATTTATTTACACTTGGTAGAGAAAGCAAAGCTAGCTAGCTCTAAACTAGAATGA

AAGTCGTTTTGAAATGGGTACGGGGGTATATAAGGGGAATGATTGAGTCACATGTAAAAGCCTTTGTATG

CCCAGTGCTAGTGTTTTCCTGAAATCTAGTGAGAATCACAAGGTACCAGAAATAATCAAATAATAAATAT

TAATACACTGCTGGGATAGAAGTATCTCAGTGTACCAGCATTCACTAAGCTATAAAATCTGTGACAGCAG

GAATATTAAATTATGAACAACCTGGTTTATCTGTCTCCTCTTATCTTTTACCTCTCCCTACGCCCAAAAT

TCTCCTTAGGTTATCCAGACATTTTTACTAAGATGCTTATTTATATGTAAGAGAGATTTCCAGTAGATAC

AACAAAACACAAGAAATCACCTGTTCTGTTTATTCTTGCTCATGTAGGATAAAGGTCCTGAGACCTCTCA

GAATCTTACGAGGTTGGAGAGTTGTTTGGAATGCTGGGTTCTGGTCTTGTCTCTGCTTCCAACCAGCTAT

GGAATCTTGTGTATTCTTTCAGTCTTAAATCACTCATCTGTTATCTAAAGATGGACTAAATTATTCCTTC

CTGCTGTGATATTCTGTGGTTCTCATCAGATAGAAATAACATATTTGAAAATGCTTTGTAAATGTTATGA

TACTCAGGATTATACTGCACAGAAAAAAAAAAAATCCTAAGGCAGCACCAACAAAGGTATTCAACGTAGTG

GGTGGTCTTTTACAGCATTACTTTGCCCATATTCATTTTGAAGACCTGAAATGAAGTTGAACTATAAATT

GCTCTAAAATTCGTGAAGCAGGGTGAAAAGGTCTTTGCTTTGTTTTACATTTAAATCCAGCTGTCTATTT

TCTGCTTATACTAGTGCTTTTTCATGGCAAAAAGACAGAAAAGTTCCCTTCTGCAGTCTCCAGTGTCTTC

TGACCTCACAGAAATGTTTTCATGCTTTTACCTAGCAGGACCACCAGAGGCTCTGTCACACTTGCAGTCC

CTGGCCCAACACCGAAATCCTTCTGGAATCTGTGCTCTGGGGGCTGTGCCGGGTAGAGAGGGCAGTGGGA

GGTAAGAGCTCTTCACCCTTCACCACCTTCTCCACCCAGCATGGCCGGCACACTTTGGTCTACGGCACAT

CTCCAAGTATAGAGTGGGTTTTGAATGCTGTTATGTCTCACCGTGGGGATGGGAAGGAATACTCTGTTTT

GGACTATTTGGAGGTACATGTGAGTGGATTTTATTACCAGAGTAGGAACTGTTTCAAGTTAATGTGTCTT

TTATGTTGCTTTTAAGACAACTTTTGTGCCGCTTTTATCCATTCTTCCTTTTATCCACAGAAATACAAAT

TTCTAATTATAGTATATTTTTTCTATTTTTAAAATTGTGATAAAGCATATATAACAAAATTTGCCATCTT

AAGTGTACAGTTCAGTGGCATTAAGTACATTCACACTGTTGCACAGCCATCACCACCATCCATTTCCAGA

ACTTGTTTTCATCTTCCTGAACTGAAACTCTGTACCTGTTAAACAGTAACTCCCCATGCCCCACCTTCTT

TCAGCCCCTGGTAACCAGCATTTTCCTTTGTCTCTATGAATTTGACTATTTTAGATACCTCATATAAGTG

GAATCATAGTATTTGTCCTTTTGTGTCAATCATATTGTATTTTTGTCTAAAAAAGGAAAACTATCAGATT

TATGTGAAATGTAACGTAATTTACATTTGATTTACTGTTAGTAATGTCTCTTTAATCATGCTATGCTAGG

GAAGTACATTATGGTCTCTCCCTCTAAGAATATGAAGAGGAAATTTTACCTTCAACAAACAAGCAAACTT

AGTTATATTTTTGTGATTTTATGCTGTTTTCATATTCTATTTTGAGATCTATTTGTCTTAGCTTGGAGGT

GGTTTTGTTAAATGTCCACGATTTAGAGACGGCATAATGAAAAAATGACCAAGAATTTGGTTGGGAATGG

GAGGAGCTTAGAAGAACAAGAAATTGGCTTTGATTTTATAGTGTATGGTGCTCTGTTGGTAGCTGGACAT

CATCACAGGCTTTGGATTGAGAAGACCTGTTCTGGAGTTCTAGTTCTGCTACTGCCTAGCGTTGTGATCT

TGATGAAGATACTCTTCCTTCCTGGGCTTCAGTTTTTGATCTATAAATTGCTAGTGATGGGGCTGGACTA

TTTTTAAGTTTGTTCTAACTTTAGCAGTCTTAAGAATTAAGGTTGCATGCCAGAAAATAGGGTTAAATAT

TTGTTTCTGATCTGGTTACAAAAAAAAAAAAAAACTTGATGGCTTAAAGTAAATAAAAATTCAACAGTACG

GTGTAAATTTAAAA

>NM_020399.4 Homo sapiens golgi associated PDZ and coiled-coil motif containing (GOPC), transcript variant 1, mRNA

GGGACTGACTGAGTCTTCCGTGTGTCGCCGCCGTTGCCGCCTCAGCTTCAGCCTCGTTACTCCTGCGGTT

CTGTGACTGTGCTGCTGGCGTTAACGGCGCGAGGTGAAGGGAGGTGACGGAGTGTGCCCGCGCGCGCGGG

GGTCCCCTCAGTCCCAGCAGTTCCCTTCGTGCGCGGGGGGCGGCGAGGGTCTTCAGCAGTCGGGAGAGGC

CCTTGACGGCGCCATGTCGGCGGGCGGTCCATGCCCAGCAGCAGCCGGAGGGGGCCCAGGGGGCGCCTCC

TGCTCCGTGGGGGCCCCTGGCGGGGTATCCATGTTCCGGTGGCTGGAGGTGCTGGAGAAGGAGTTCGACA

AAGCTTTTGTGGATGTGGATCTGCTCCTGGGAGAGATCGATCCAGACCAAGCGGACATCACTTATGAGGG

GCGACAGAAGATGACCAGCCTGAGCTCCTGCTTTGCACAGCTTTGCCACAAAGCCCAGTCTGTGTCTCAA

ATCAACCACAAGCTGGAGGCACAGTTGGTGGATCTGAAATCTGAACTGACAGAAACCCAAGCAGAGAAAG

TTGTTTTGGAGAAAGAAGTACATGATCAGCTTTTACAGCTGCACTCTATTCAGCTGCAGCTTCATGCTAA

AACTGGTCAAAGTGCTGACTCTGGTACCATTAAGGCAAAATTGTCTGGCCCCTCTGTGGAGGAGCTGGAA

AGAGAGCTTGAGGCAAACAAAAAAGAAAAAATGAAAGAAGCACAACTTGAAGCTGAAGTGAAATTGTTGA

GAAAAGAGAATGAAGCCCTTCGTAGACATATAGCTGTTCTCCAGGCTGAAGTATATGGGGCGAGACTAGC

TGCCAAGTACTTGGATAAGGAACTGGCAGGAAGGGTCCAACAGATACAATTGCTAGGACGAGATATGAAG

GGACCTGCTCATGATAAGCTTTGGAACCAATTAGAAGCTGAAATACATTTGCATCGTCACAAAACTGTGA

TCCGAGCCTGCAGAGGACGTAATGACTTGAAACGACCAATGCAAGCACCACCAGGCCATGATCAAGATTC

CCTAAAGAAAAGCCAAGGTGTTGGTCCAATTAGAAAAGTTCTCCTCCTTAAGGAAGATCATGAAGGCCTT

GGCATTTCAATTACAGGTGGGAAAGAACATGGTGTTCCAATCCTCATCTCTGAGATCCATCCGGGGCAAC

CTGCTGATAGATGCGGAGGGCTGCACGTTGGGGATGCTATTTTGGCAGTCAACGGAGTTAACCTAAGGGA

CACAAAGCATAAAGAAGCTGTAACTATTCTTTCTCAGCAGAGAGGAGAGATTGAATTTGAAGTAGTTTAT

GTGGCTCCTGAAGTGGATTCTGATGATGAAAACGTAGAGTATGAAGATGAGAGTGGACATCGTTACCGTT

TGTACCTTGATGAGTTAGAAGGAGGTGGTAACCCTGGTGCTAGTTGCAAAGACACAAGTGGGGAAATCAA

AGTATTACAAGGATTTAATAAGAAGGCAGTAACTGACACACATGAAAATGGAGACCTGGGCACTGCAAGT

GAAACTCCGCTAGATGACGGTGCTTCAAAATTAGATGATCTGCACACTCTGTATCATAAAAAATCTTATT

AAATTGACCATATCTCCAGACAAGATTGTTAATCAGACTATTCTGAATTTGGGGCACTGGGGAAGATGGT

GACAAAGACTACAAAATCAGGGGAGGCTGTTTGTTGCCTAAATGAAAGGCGGGTACCTCAGGGCTTCATG

TGAACAATTCTCAATGCATAAAACACCCTGTTTTCTGTGGTAGACCATAATTAGCTATTGCATGTAAAGC

AATTTTGATTTTCTTGAACTTGTAAGCACCAAAGCATGTGTTTCCCAAAGGGGTATTACTGGGCTCTTAA

GTACCAAATGAAACCCTACTGTTTTACTTGGTTCCTTTTCCCTTTAGTAGGAGGACAGTGCTAACTGGAA

TTTTATAAGGAGTACCCTTTAAAATACAGAGAGTAAGTGAATTCATATTTTATATTCTAGAATGTTAACT

GTGGTATTTCAGAAGACAGAGCTTGACTCAATGAATTGTAGAAATATAGGCTTGACTTAATTTTTGTAAC

TGAAAGGCTTTAGACTTAAAAAAATTTATTGTGCATCCTAAAATCTAAACCAATTATTAATGATCATTTA

TTGTAATGAGTTTGCATTTCAGCTTTATTCAATGCAGTACAAAATAGTTTTCTTTAAGCAGTCTTTTATC

AATCAATGCTGCACTAGAAATAGATTCTCAAAAGTTACTAAACATTTTACTTTTTTTGTTTTTTAAAAGA

AATGCAGATGAGTGTAAAACATCTGTTCTCAATTATGTTGATCTGTGTGCGCAGTACTGGAGCATTTACC

CATTCATGTTGAGCCTCAAATGCTTGTTTTCTGGGGTCCACAAAAGACAGTTTTATACATTTTGAGTTGT

TCATAAAGTTTGTCTTGTGATAGTCCTGGCACTTAAAGACAAATTTTTCTGGTAGTAAAAGTTCAGATTT

ATTACTATGTCATGAAACACAGTACATTCAAATCAAACGGCAGTTTTCTTTCTAAGTAAATGATTTCCAG

TCATCTAAAAGGTGGGCAAGATGAGATAAAGACATTTTGATACAGTAATTGTTTTGGTTGGGTTTTCATG

TCAGTTTATGTTTGACTAAAGCTCTCTTCATATGCAGGTTTATAAATTTGTTAGGTCTGTTGTCCCATGA

TTAAACATGGAGTGCCTCCTCTCTGATTTAATATTCTGCAGGTCATTGTAACCTGCTAGGCAAAGTCACA

ACATTGCATTAAAGAGGTGATAGCTTTGCTAATATCACTGTTTTAAAGGACGTACAGTTAAAGGAATATT

AAGTGGGAGAAAGCCTACAAGGCTTTTAGAATATTATCAGTATCTTCATTTCTGGTATTCAGATGTTATG

TGATAAAACACATTTTTTTTGGCTTTCCCAGATACACTATATATTTGTTCAAGGGTAAATCTATAAAATG

TATATACTTTATTTTGTGGTTTTGCTATTTATAAATTTAATGTTTTAACTGTTGCTCATTTATGGTTTGT

TTTGGGTGGTGGTGTTCATCTGTATATCACCATGTTAATTTGTAATGGAAGTGCACTTCGTAGTGTATAT

TGTTACTGACATTAAAATACTTTATAGCATTGTCTCTGAGCAAAAGCTAGTATTTAATTGTACAAATGAA

TAAGCAAGTTACATGTTATTGTTTGCTCTTGACAGGGTAGGCCTCTTAAAAGAAAAAAAACAACTTGTTT

TTTCTTTATGAATCCCCTATGCCAAACACATACCTTCCATGCATGACATGAGATCTGCAAACTGGATTTT

AGCCACCGTATTTATTTAGTCAAAAAAATTGTCCATTGTAGCAGACCCGAAAACCTTTTTGCTGTGACAT

GAAACCATGTTATTCTTATCTTCTTAAAACACAGCCTGGGATGGAATGGCCATGGCATTTTTTTCAGAGA

ACATCCTTTATCTGCTATGACTGAATCCTTAGGAAATGTAAGCTATAACCCTTTGATTTTCAAGAACTAC

CGAATAAGTGTATGAAGAGGTGGTTTTTTAAAACTTCAAGTTGGAATTTTTATGAGGTCACTGTGTAATT

TGAAGAATTGTGTGAGATTGTCATGATATAAATTCCTTTTAAGGACTGATAAATAGAATGAAAAGTTTCC

AGGTAGTTTAAAACTCCACAGGTCAGTTTCCTTTTCATTCCTGCTTCACTGTGGTTTATAAGCCTACGGG

AGAGCACCGTTGCTCAGATGCTACTGTGAGCTTCCTGTCCGGTGTTAGAAAGTAACTAGTTAAAAGTTCA

TTTTAGAATGTATGGTTTTTGGGGATGAACTAAGAATTAGTTATTAGTTCCAAAGGACTGAGAACCAATT

TTAATATTTTCACATTTATAGGAAAGAAATTCATATGTCCCTGAAACTTCTAGGACAAAACCAAACAAGT

AAGGAGGGAACTGTTGCAAAGCCATTTCATCGAGAAGGGGACAGAAGGAGAAATACACACATGTATACAC

AAACAGAATGGTTGAGAAAACGTTTTAATAAAATGTGAGGGTTGTATGTGTGCGTGTATATATTTACACT

TAACCTCTAAAATTCTCTTCTACAGTATCTCTGTTATGAATATGATGGAAAAGCAACATTTTGGTGGTGA

GACTATTGTTAAAATAAATTTGAGAAAGACGAAAATTTTGTGAGTCTTGATAATTACAAGTCAACAGCTA

TCGAAAGTTAGCACAGCTTGTCTGTGGTGCTGTTTTTTTCCCCACTGCAGTGGACTTATGCTGTTTTCAT

GTTTAGAAACAAAAAGGTTTCATGTGATTCATGTGTAAGATGCACAGTATTTGACATCCTGATTATGTAA

TCCCTATTCCATCTATCCAGTCTTACACTTATGGTTGGCCTCAAATCTATTGCATTTATGATAATGTATT

ATATCTAGTTGAGTTTAATATTTTTTTATTAGCCTGTAAATAAAGATGGCATCTTCTACATTAAAATGAT

ATTGATCTCATTTTTTTAAATAAACATTTTGTTTCCTTGACGTTAAA

>NM_017551.3 Homo sapiens glutamate ionotropic receptor delta type subunit 1 (GRID1), mRNA

AGTTCGCTCCTCGCCGGGGCTCAGACACACAGCCAGCCCAGAGCCGCCACGCCGGCCCGGCCGCCGCCGC

CACCGCCTCAGCCTCCCCCGAAGCCGCTCTAGCCGCGGGAAGCGCCGCTGCCACAGCCGCGGGCCGCCCC

CGCCGCGCCGGCCTGGGCTCAAGCTGCCAGCACCGCCGCAGCCGCCACCGGAGCCGGAGCGGGAGCCCGA

CCGCGCTGGGCTGGGCTGGGCTGGGCTGGGGCGGGCGCAGGGCGCAGGGGCGGGCGCGCGGGGGAAGACG

CACGGGCGGGCTCGGCTCTCCCGGGGAGCGGCCCGGGACTGCACCGGGACCAGCGCCTCCCCGCTTCGCG

CTGCCCTCGGCCTCGCCCCGGGCCCGGGTGGATGAGCCGCGCGCCCGGGGGACATGGAAGCGCTGACGCT

GTGGCTTCTCCCCTGGATATGCCAGTGCGTGTCGGTGCGGGCCGACTCCATCATCCACATCGGTGCCATC

TTCGAGGAGAACGCGGCCAAGGACGACAGGGTGTTCCAGTTGGCGGTATCCGACCTGAGCCTCAACGATG

ACATCCTGCAGAGCGAGAAGATCACCTACTCCATCAAGGTCATCGAGGCCAACAACCCATTCCAGGCTGT

GCAGGAAGCCTGTGACCTCATGACCCAGGGGATTTTGGCCTTGGTCACGTCCACTGGCTGTGCATCTGCC

AATGCCCTGCAGTCCCTCACGGATGCCATGCACATCCCACACCTCTTTGTCCAGCGCAACCCGGGAGGGT

CGCCACGCACCGCATGCCACCTGAACCCCAGCCCCGATGGTGAGGCCTACACACTGGCTTCGAGACCACC

CGTCCGCCTCAATGATGTCATGCTCAGGCTGGTGACGGAGCTGCGCTGGCAGAAGTTCGTCATGTTCTAC

GACAGCGAGTATGATATCCGTGGGCTTCAAAGCTTTCTGGACCAGGCCTCGCGGCTGGGCCTTGACGTCT

CTTTACAAAAGGTGGACAAGAACATTAGCCACGTATTCACCAGCCTCTTCACCACGATGAAGACAGAGGA

GCTGAATCGCTACCGGGACACGCTTCGCCGCGCCATCCTGCTGCTCAGCCCACAGGGAGCCCACTCCTTC

ATCAACGAGGCCGTGGAGACCAACCTGGCTTCCAAGGACAGCCACTGGGTCTTTGTGAATGAGGAAATCA

GTGACCCGGAGATCCTGGATCTGGTCCATAGTGCCCTTGGAAGGATGACCGTGGTCCGGCAAATCTTTCC

GTCTGCAAAGGACAATCAGAAATGCACGAGGAACAACCACCGCATCTCCTCCCTGCTCTGCGACCCCCAG

GAAGGCTACCTCCAGATGCTGCAGATCTCCAACCTCTATCTGTATGACAGTGTTCTGATGCTGGCCAACG

CCTTTCACAGGAAGCTGGAGGACCGGAAGTGGCATAGCATGGCGAGCCTCAACTGCATACGGAAATCCAC

TAAGCCATGGAATGGTGGGAGGTCCATGCTGGATACCATCAAAAAGGGCCACATCACTGGCCTCACTGGG

GTGATGGAGTTTCGGGAGGACAGTTCGAATCCCTATGTCCAGTTTGAAATCCTTGGCACTACCTATAGTG

AGACTTTTGGCAAAGACATGCGCAAGTTGGCGACATGGGACTCAGAGAAGGGCTTGAATGGCAGCTTGCA

AGAGAGGCCCATGGGCAGCCGCCTCCAAGGATTGACTCTTAAAGTGGTGACTGTCTTGGAAGAGCCTTTC

GTGATGGTGGCTGAGAACATCCTAGGACAGCCCAAGCGCTACAAAGGGTTCTCCATAGATGTCCTGGATG

CACTGGCCAAGGCTCTGGGCTTTAAATATGAGATTTACCAAGCCCCTGATGGCAGGTACGGTCACCAGCT

CCATAACACCTCCTGGAACGGGATGATCGGGGAGCTCATCAGCAAGAGAGCAGACTTGGCCATCTCTGCC

ATCACCATCACCCCAGAGAGGGAGAGCGTTGTGGACTTCAGCAAGCGGTACATGGACTATTCAGTGGGGA

TTCTAATTAAGAAGCCCGAGGAGAAAATCAGCATCTTCTCCCTCTTTGCTCCATTTGATTTCGCTGTGTG

GGCCTGCATTGCAGCAGCCATCCCTGTGGTTGGTGTGCTGATATTTGTGTTGAACAGGATACAGGCTGTG

AGGGCTCAGAGTGCTGCCCAGCCCAGGCCGTCAGCTTCTGCCACTCTGCACAGCGCCATCTGGATTGTCT

ATGGAGCCTTCGTACAGCAAGGTGGCGAATCTTCCGTGAACTCCATGGCCATGCGCATCGTGATGGGCAG

CTGGTGGCTCTTCACGCTCATTGTGTGCTCCTCCTACACAGCCAACCTTGCTGCCTTCCTCACAGTGTCC

AGGATGGACAACCCCATAAGGACTTTCCAGGACCTGTCCAAACAAGTGGAAATGTCTTATGGCACTGTCC

GGGATTCTGCTGTATATGAGTACTTCCGAGCCAAGGGCACCAACCCCCTGGAGCAGGACAGCACGTTTGC

TGAACTCTGGCGGACCATCAGCAAGAACGGAGGGGCTGACAACTGCGTGTCCAGTCCTTCAGAAGGCATC

AGGAAGGCAAAGAAGGGGAACTACGCCTTCCTGTGGGATGTGGCCGTGGTGGAATACGCAGCCCTGACGG

ATGACGACTGCTCGGTGACTGTCATCGGCAACAGCATCAGCAGCAAGGGTTACGGGATTGCCCTGCAGCA

TGGCAGCCCCTACAGGGACCTCTTCTCCCAGAGGATCCTGGAGCTGCAGGACACAGGGGACCTGGATGTG

CTGAAGCAGAAGTGGTGGCCGCACATGGGCCGCTGTGACCTCACCAGCCATGCCAGCGCCCAGGCCGACG

GCAAATCCCTCAAGCTGCACAGCTTCGCCGGGGTCTTCTGCATCCTGGCCATTGGCCTGCTCCTGGCCTG

CCTGGTGGCTGCCCTGGAGTTGTGGTGGAACAGCAACCGGTGCCACCAGGAGACCCCCAAGGAGGACAAA

GAAGTGAACTTGGAGCAGGTCCACCGGCGCATGAACAGCCTCATGGATGAAGACATTGCTCACAAGCAGA

TTTCCCCAGCGTCGATTGAGCTCTCGGCCCTGGAGATGGGGGGCCTGGCTCCCACCCAGACCTTGGAGCC

GACACGGGAGTACCAGAACACCCAGCTCTCGGTCAGCACCTTTCTGCCAGAGCAGAGCAGCCATGGCACC

AGCCGGACACTCTCATCAGGGCCCAGCAGCAACCTGCCGCTGCCGCTGAGCAGCTCGGCGACCATGCCCT

CCATGCAGTGCAAACACAGGTCACCCAACGGGGGGCTGTTCCGGCAGAGCCCGGTGAAGACCCCCATCCC

CATGTCCTTCCAGCCCGTGCCTGGAGGCGTCCTTCCAGAGGCTCTGGACACCTCCCACGGGACCTCCATC

TGACTGCGCCGCCTGCCCTCCTGCCCACCCTCCCACCCACCCGACCAGCAGAGCTTTTTAATACAAGAAA

ACAACAACACAAACCACACACACTCGCACACACACACATACACAGAGACTCTTTCATTTTTCTTGTACAT

ATGTGTAAATAATGACAGAATGGAGTGGGGTAAAAGTGTATTTTGAATATTCCCAATTTTCGAAGTCAGT

AAAAAAACACAAAAACTGTATGAATGACTTTGTAAATTTTGTTCTATATGAATAAAAAGGCAAATTACTT

GTGATCATTCTGAAGTGCCAAAGGAGCCCCCCATTCCTGGGCCTTTCTGAGGGCAGGAGGGGCGACCAG

ATAAGGAGCCCCTCTCTGCTGGGGGAGAAGGGAAGACGAGGAACCCCACATGCCACTCGCTGCCTTGTCC

CACAGCTTGCTGCCCCATTTCTTTGCTCCTGGCACCTCGTCCCTTTTAGTCCCTCAGCTTGATAAAGAGT

GAGTTTGGAGCCCGCATTGGGCTGGCCCACTGGGTTGCTGTGCTGTAGGGTGATCGGCTGTTCTGGGTAG

CCTGGGGCTGAGGAGGTGCCCTGGACTCAGGGCTATCCTGTCCTGTCTTGGATCTTGCGGGACGAGTTAG

TCACCGCTGTGTGTGTTGCAGTGTGCCTCTGCCCCATGGGCCCGAGAGAAGCTGACAATTACCCATAGTG

AGGTAATTGAGGGCTTTGATACTGAGCTGTTCTCATATCATACTGTTGGCCTTGTTTTTCATTTTCTTTT

CAAAATTAGAAGAATCAAAAAGTGATAGAATATTGGGGGAAGGAGGGCAGACTCCATCGTTTCCTCAGGG

AGACTGGGCGGAGGTGGGGTTGGTCTGGAAGAAGGTCCATGGGGGCAGGGAGTTGCGAGGTGGGGTTAGT

TGCATGGACCAGGTGAGGTGGTGGAACAAAAGGCCAGGTAGAGGAAGAATATTCCCTTGGGTTTGGACCC

ATGGTCCCAGGTGAGAGAAGGAAGTGAGGCCATAGATGCAGGGAGTAGAAGCTTTGTGGTGTCAGGCAAA

CGGACTCTGTTAGTAAGAGTTGGGGGAGGTGCCCAGGTGGGTGGACCAGTACTCCAGGTGATGGGGCATG

CTGAGAAATAGAGAAAAGAGACCATGTTTATTTGGGTAGGAGGAAGCCTTGCCTTGCGCCTTAGGTGAGA

AGCATAAGTGTAACTCCCATCCACCAGGGAAGTTGCTTGTAGCCCCAAAAGTAAAGGCCTATCTCTGGGT

CATAAATCCTGCAGGCAGTCCAACAAACAGGGCTGGCTCCAGCACAAACTCTCCCTTCCACCTTTACGAC

CCTCTCCAGACCAGACCTGGAGTCCTCTTCAAGCAGCAATCCAACCCAGAGCAGGGGCCCTCCCCACTCA

GGCATCTGATAACCTCTGAGATTTCCAGGCCTATCCCTGTGCAGGTACAGCTGCAGGGAAGCCCAGTTTT

CTAAGCCTAGGACCACCTAACAGCGCCCCCTCCTTCAGTTGCCCTTTCTGAGGCAACTAAACTACAGAAT

CAGGGAAAGAACCATTAGGAGTGGAACTGGGGGAATCTGAGTTGTGTCACTTCAGTTCCTCTCCTAAAGA

CAAAGGTTAGTCTGATCTCCAGACCCGTCAGAATGGAATACACAGCCCACATCTGTCACTGAAGGAGGTG

GAGCTCCCACAGCCAGCAGTAATCAGGGAGCTGAGAGCCTGGTTCTGTGATCATGGAGAAATACAAAGTC

CTATTGATTGCTTCTTCTATAGCCTTGTAGAGTTTCTAGAGAGATGTATTTATGAGGGTGATAACTAGCC

CAGGATTGATTTCTTTCCTAAAGTCCCTAGTGACATGATTGAGCAGTAAAGAACGGCCAAATCACACAGT

CAGCTAAAAGCACTGTGGGGAAGAGAGTGTTTATAATTATGTTATTTATTGCTGGATGCTGAGAATGGTC

TGATATTCGTGCTACCTAGGCAATCCATTGACATTTCTCCAATCAGAGCATGTGGACCTTGGAGCCAGGC

ATGCTCAGAGAAGCCTAGGTGGGCTACCATGACCCCGAGGAAGAGCAGGCTTTGTTTTCCATCAGCACGT

TGGGGGCCCTGCCCTGAATGGTCAATTTTTCACATATATATCTCTCTATTTTTTTAATCAAACTCTGGTC

TCACTGCCTTATCTCACACCAACTCTGTTTCCTCATTGCCCCCTGAGATGGCCTGTCTTCTGGGGTATAG

CTTGGATGTCTTCTTGGATGGTTCTGCTTAGAATGAGTGTCAAGGAGGAAAGAGAGGGAGATGGAGGATG

TGTTTGTGCGCCTGTGTGTGTGTGTTTGTGTGTGTGTGTGTGTGAGAGAGAGAGAGAGAGAGAGAGAG

ACCAGCATCTTCAAGAGAAGTATTCTGCTTATACAAAATCCTTAACACCTCATGGTGTTATTCTTCACCA

TGTTTATATATATATATATATATATTTTTTTTTTTTTTTAGAATTTTCTACCCTTGGCATGAGGGGAAATGA

TTGATATTCAAGCAAGTTCTCTAGGAAAAAAAAAAAACTTCCCAACTCAGATTTCTGTGTCAGCTCAGAAT

GTATCTTTTTTTCATGCTTTGCTCTTTGGATTTATAACTCTGTTTAGACTATTCCATACATTTTAGGTAT

ATTTTGTGCCTTCAGACACTGCAAATAATAATCAGCATTTGGATTAAAGTTGTTTAATAATAAA

>NM_001286838.1 Homo sapiens glutamate ionotropic receptor delta type subunit 2 (GRID2), transcript variant 2, mRNA

TGCAAACAACTCTGGCGATGCCAAAATTCCCCTCCAAGTGACACGGCTTTGCGAAGGAGGTTTCCTCAGG

CTGGGCTCTTTCTGTCATTCCCTTCTGCCTTTCTCGGCGACGATAAAAGGCTTTGCTCTGGCAATAGGAA

TTTAGAAAAAAAGAAAAAGCTGCGCTAAACTCCACCGTGACCTCAAACTCTTTGGACTGTTTGAAAAAAA

AAAAATTGGAAGAAAATCCATCCTCCAAGAGAATCGGCATAGGAGGAGATGGAAGTTTTCCCCTTTCTCT

TGGTTTTGTCCGTCTGGTGGTCTCGAACCTGGGACTCGGCGAATGCGGATTCGATCATTCACATCGGAGC

AATTTTTGATGAATCTGCCAAAAAGGATGATGAGGTATTTCGCACTGCGGTTGGTGACCTTAACCAGAAT

GAGGAGATCTTACAGACTGAGAAAATCACATTTTCAGTGACGTTTGTTGATGGCAACAACCCTTTCCAAG

CAGTTCAAGAAGATATCCGTGGAATACAGGAGTTCTTGGACAAAGTCTCTCAGCAGGGAATGGATGTTGC

ACTTCAGAAGGTAGAAAACAACATCAATAAAATGATTACCACTCTCTTTGACACCATGAGAATAGAAGAA

CTGAATCGCTATCGAGACACTCTTAGGCGAGCGATCCTTGTTATGAATCCTGCTACAGCCAAATCCTTCA

TTACTGAGGTTGTGGAGACTAATTTGGTTGCTTTTGACTGTCACTGGATCATTATAAATGAGGAAATAAA

CGATGTGGACGTACAGGAACTTGTAAGAAGGTCAATTGGAAGGTTAACGATTATTCGGCAGACATTTCCA

GTTCCCCAGAACATAAGTCAGCGGTGTTTCCGTGGCAACCATCGAATATCTTCAACATTGTGTGATCCAA

AGGATCCATTTGCTCAGAATATGGAGATTTCCAACCTTTACATATATGACACGGTGCTTCTGCTTGCTAA

TGCTTTTCATAAGAAGCTGGAGGACCGAAAGTGGCACAGCATGGCAAGTCTGTCATGTATCAGAAAGAAC

TCAAAGCCCTGGCAGGGTGGGCGCTCCATGTTGGAGACCATCAAGAAGGGTGGAGTTAGTGGGTTGACTG

GAGAGCTAGAATTTGGAGAAAATGGAGGCAATCCCAATGTCCACTTTGAAATCCTTGGAACCAACTATGG

AGAAGAGCTTGGCAGAGGTGTTCGAAAACTTGGTTGCTGGAATCCTGTCACAGGTCTGAATGGGTCACTG

ACTGACAAGAAATTGGAGAATAACATGCGTGGAGTGGTTCTACGTGTAGTAACTGTTCTGGAAGAACCTT

TTGTGATGGTCTCTGAAAATGTCTTGGGTAAGCCGAAGAAATACCAGGGCTTCTCCATTGATGTTTTGGA

TGCCTTATCTAACTACCTGGGTTTTAACTACGAAATTTACGTAGCACCGGATCACAAATACGGAAGCCCA

CAAGAAGATGGGACATGGAATGGCTTGGTAGGAGAACTTGTCTTTAAGAGAGCCGACATAGGGATTTCTG

CTTTAACCATCACTCCAGATCGTGAAAATGTGGTGGACTTTACGACACGTTACATGGACTACTCAGTGGG

GGTACTACTTCGAAGGGCTGAAAAGACAGTGGATATGTTTGCCTGTCTTGCACCATTTGATCTCTCTCTA

TGGGCTTGCATTGCTGGCACAGTCCTTCTGGTGGGTCTACTGGTCTACCTCTTGAACTGGCTTAATCCCC

CACGATTACAAATGGGATCAATGACGTCTACTACTCTCTACAACTCCATGTGGTTTGTGTATGGATCTTT

TGTACAACAAGGCGGGGAAGTCCCGTACACGACTCTGGCTACCCGAATGATGATGGGGGCTTGGTGGCTA

TTTGCTTTGATTGTTATCTCATCTTACACGGCAAACCTCGCTGCTTTCCTCACTATTACACGCATTGAAA

GTTCCATCCAGTCTCTCCAGGACCTTTCCAAGCAAACAGAAATCCCTTATGGCACAGTCCTAGACTCTGC

GGTATATGAGCATGTCCGCATGAAAGGACTGAATCCTTTTGAGAGGGACAGCATGTATTCCCAAATGTGG

CGGATGATCAACCGAAGCAATGGATCGGAGAACAATGTTCTGGAGTCCCAGGCAGGCATTCAAAAGGTAA

AATATGGAAATTATGCTTTCGTATGGGATGCAGCTGTATTGGAATATGTGGCTATCAATGACCCAGATTG

TTCCTTTTACACCATTGGAAATACTGTTGCTGATCGGGGATATGGAATTGCATTACAACATGGCAGTCCT

TACCGAGATGTTTTTTCACAAAGGATCCTGGAGCTTCAGCAGAATGGTGACATGGACATCCTGAAGCACA

AATGGTGGCCTAAGAATGGCCAGTGTGACCTGTACTCGTCAGTGGACACAAAGCAGAAAGGAGGCGCCCT

GGACATAAAGAGCTTTGCAGGGGTCTTTTGTATCCTGGCTGCTGGAATTGTCCTCTCCTGCTTCATAGCC

ATGCTGGAGACGTGGTGGAACAAGAGGAAAGGCTCCCGGGTTCCATCAAAAGAGGATGACAAGGAAATTG

ACCTGGAGCACCTCCATAGACGTGTAAATAGCTTGTGCACAGATGACGACAGCCCCCATAAACAGTTTTC

CACCTCGTCAATTGATTTGACCCCTCTGGACATTGACACTTTGCCAACACGACAAGCACTGGAGCAAATC

AGTGATTTCAGGAACACTCATATTACCACAACAACCTTTATCCCAGAGCAGATCCAGACTCTTAGCCGCA

CACTGTCAGCTAAAGCTGCTTCTGGTTTCACTTTTGGCAACGTGCCTGAGCACCGAACTGGCCCTTTTAG

GCACAGGGCACCTAATGGGGGCTTTTTCAGGAGTCCTATAAAAACAATGTCATCTATTCCTTATCAACCA

ACTCCTACCCTGGGGCTCAATCTGGGTAATGATCCAGACCGAGGCACCTCCATATGAGCATCAAACAAAT

CTCTTCACTGTTTCTTTTTTAGGACTCCCTTTGCAAGGAGCAACTGTAATATTGTGGGACTAACATGGAT

GTAACGTTTAAAAAAAAGATCAGGATTATTAGTAACAATTCTAGTTTTTTCCTCCCACCTTCTCCCTCTC

CTCTCTCCTCTATGATTTTCTCTCTTTCCCCCTCCCTTCCTGTACATTTTCCTCCACTTTTTTTCATTAC

TCAACTTGTTCCCCAAGAAGGTAGTGTACTAGGATCCTATTAGTCTGCTTTTCATATACTGTACATCAAG

TATAGGAAATGTGCACTGAGTATACTAAAAGTATATGCAGGATTAATTTTACATAAAGGCCTCTTCTGTA

ACATTTCTCTATTTTTTGAAATACAATTGCAATAACTTCAGTCTTTTTACATTCTTCTGCTGCATCCATA

CAATGCTCCACTGCTGTTGAGTGTCCTTTAACTGTGGACCAAAGGCAACCCCTGCAGTGTTTATAAAACA

ATTTTAAGACCATTCAGGCCACATAAGATGAGAATGCAATTTTGTAGGATTACAAAAAAGCCATTAATAT

GCCAGTCAAGACTACAGTTAAAACTACTCTTGCACTGCAACAGTGCATATGACCTTTATGTGATTGTACA

GTATTAAAAGTTTTTTCTTATGTACAGTAAACTTTATCATATGGCAGAAGCCTCTATTGTGTTAAATAAA

TTAGTATTTCATATATATACATATATATGCACCTATATAATGTGTATATATATAAAAAGGCAGCCATTGC

TATTGCAATTCATTTAATAAAGCTTTAGTTTAAACAAAAGTATTGTATACAGGAACTATGTATAGTGCAG

GGGTTCTTTTCAGTTAGAAAAGGCAGGTTGCTTTAATGTTATTCTGACTCGCATTGTTTGCCAACAGAAA

ATATTTTATACTTTTGTTATTAAAGCATCCAGGGCAATTTAATATTTGTTCCTAGATGTAACTCATTTGA

ATAGGTTTTGCATTTGTTATTCAGCAGTGTATAAAGCTAACCTTGATAATTTTACTTTTAATTAATTATC

TAAATGAAAAATGCTATTAGCTGAACCACATCCACAACAGCACATAGAGCTCTAGAAGAGTTGAAACATA

GATCATTGAAGGTTAAAAATCCTCTTCCAAAGCAGCAAGTCAATCAATACAATAATGATATTTGGGGAGG

GGTGGGAGCCCTTAAGAACTATTGATGTGGCCTTAAATTACTGTCATTTATTATCCTAAAGAAGAAAACA

TAATAACCATAATGGGCTCTCAACTCAACTCCCTTTTTTATTTAGTTCTATTTGGAGGAGTTATGTATTT

TTTACATTAGTGCCAAGTATATAGAAGGATGACAGCAAAAACAGTGCCTCTTTGTGCACAATCCTTTTAA

AGTAGCATTCTTACTTGTTAGAAATGTAGAATGAACTTTGTTTGTAATCCTTAAATTAAAATGTGGTAAG

TAGGAAGCAAGAACTTATTCTGTGTTTTTACGCAGCCATACACTTACTTTAATCTAACTCATTCTGTGGC

TAGGATTGATATAGGAATCAACCTATGTGATTTGCTTTAGGAGAAATTAAAAGGTATATTCTTAAGATAT

ATTATATTTTGATGCTAATTCTGTTCTGGGGAGCATCTTGTATTGTCACTGTTTTGTACTAAATCTTCAA

ATATTGTCTACTGTGTAAGGCAGAAAAATTTCTTATCATGCAAAAACCATTTTGTTTCCAGGGTAACAAG

TAACTAAGTGCATGCACAACTTGTTTTCCCTTGTAACATTCATGATCTCATTTACAACTCTGATTTTAAA

ACAAAAAAATCTTTACAACAAGCTATATAGGGACATACCAGATGTTGCAGTGATTTTAGCTATCAACAAA

CTGTTAACCATGTACAATATTTAAAATAGGCTTATTTTGATGTATCGCCTATTATACAAACACACAAAAC

ATTTTTGGAGGCCTCAGTTATGAGTGACAGAGCTTTCTGTGTATAATTTGTCTAAATAATTTGTCTAATA

AAAATGTTTTCTAAAAAAAAAAAAAAAAA

>NM_004964.3 Homo sapiens histone deacetylase 1 (HDAC1), mRNA

CCCCTCCCCCCTGGGTCGGACGCTGAGCGGAGCCGCGGGCGGGAGGGCGGACGGACCGACTGACGGTAGG

GACGGGAGGCGAGCAAGATGGCGCAGACGCAGGGCACCCGGAGGAAAGTCTGTTACTACTACGACGGGGA

TGTTGGAAATTACTATTATGGACAAGGCCACCCAATGAAGCCTCACCGAATCCGCATGACTCATAATTTG

CTGCTCAACTATGGTCTCTACCGAAAAATGGAAATCTATCGCCCTCACAAAGCCAATGCTGAGGAGATGA

CCAAGTACCACAGCGATGACTACATTAAATTCTTGCGCTCCATCCGTCCAGATAACATGTCGGAGTACAG

CAAGCAGATGCAGAGATTCAACGTTGGTGAGGACTGTCCAGTATTCGATGGCCTGTTTGAGTTCTGTCAG

TTGTCTACTGGTGGTTCTGTGGCAAGTGCTGTGAAACTTAATAAGCAGCAGACGGACATCGCTGTGAATT

GGGCTGGGGGCCTGCACCATGCAAAGAAGTCCGAGGCATCTGGCTTCTGTTACGTCAATGATATCGTCTT

GGCCATCCTGGAACTGCTAAAGTATCACCAGAGGGTGCTGTACATTGACATTGATATTCACCATGGTGAC

GGCGTGGAAGAGGCCTTCTACACCACGGACCGGGTCATGACTGTGTCCTTTCATAAGTATGGAGAGTACT

TCCCAGGAACTGGGGACCTACGGGATATCGGGGCTGGCAAAGGCAAGTATTATGCTGTTAACTACCCGCT

CCGAGACGGGATTGATGACGAGTCCTATGAGGCCATTTTCAAGCCGGTCATGTCCAAAGTAATGGAGATG

TTCCAGCCTAGTGCGGTGGTCTTACAGTGTGGCTCAGACTCCCTATCTGGGGATCGGTTAGGTTGCTTCA

ATCTAACTATCAAAGGACACGCCAAGTGTGTGGAATTTGTCAAGAGCTTTAACCTGCCTATGCTGATGCT

GGGAGGCGGTGGTTACACCATTCGTAACGTTGCCCGGTGCTGGACATATGAGACAGCTGTGGCCCTGGAT

ACGGAGATCCCTAATGAGCTTCCATACAATGACTACTTTGAATACTTTGGACCAGATTTCAAGCTCCACA

TCAGTCCTTCCAATATGACTAACCAGAACACGAATGAGTACCTGGAGAAGATCAAACAGCGACTGTTTGA

GAACCTTAGAATGCTGCCGCACGCACCTGGGGTCCAAATGCAGGCGATTCCTGAGGACGCCATCCCTGAG

GAGAGTGGCGATGAGGACGAAGACGACCCTGACAAGCGCATCTCGATCTGCTCCTCTGACAAACGAATTG

CCTGTGAGGAAGAGTTCTCCGATTCTGAAGAGGAGGGAGAGGGGGGCCGCAAGAACTCTTCCAACTTCAA

AAAAGCCAAGAGAGTCAAAACAGAGGATGAAAAAGAGAAAGACCCAGAGGAGAAGAAAGAAGTCACCGAA

GAGGAGAAAACCAAGGAGGAGAAGCCAGAAGCCAAAGGGGTCAAGGAGGAGGTCAAGTTGGCCTGAATGG

ACCTCTCCAGCTCTGGCTTCCTGCTGAGTCCCTCACGTTTCTTCCCCAACCCCTCAGATTTTATATTTTC

TATTTCTCTGTGTATTTATATAAAAATTTATTAAATATAAATATCCCCAGGGACAGAAACCAAGGCCCCG

AGCTCAGGGCAGCTGTGCTGGGTGAGCTCTTCCAGGAGCCACCTTGCCACCCATTCTTCCCGTTCTTAAC

TTTGAACCATAAAGGGTGCCAGGTCTGGGTGAAAGGGATACTTTTATGCAACCATAAGACAAACTCCTGA

AATGCCAAGTGCCTGCTTAGTAGCTTTGGAAAGGTGCCCTTATTGAACATTCTAGAAGGGGTGGCTGGGT

CTTCAAGGATCTCCTGTTTTTTTCAGGCTCCTAAAGTAACATCAGCCATTTTTAGATTGGTTCTGTTTTC

GTACCTTCCCACTGGCCTCAAGTGAGCCAAGAAACACTGCCTGCCCTCTGTCTGTCTTCTCCTAATTCTG

CAGGTGGAGGTTGCTAGTCTAGTTTCCTTTTTGAGATACTATTTTCATTTTTGTGAGCCTCTTTGTAATA

AAATGGTACATTTCTATA

>NM_006044.4 Homo sapiens histone deacetylase 6 (HDAC6), transcript variant 5, mRNA

GGTTGAAGGAACGGGGCAGTCCCCTGAGGAGCGGGGCTGGTTGAAACGCTAGGGGCGGGATCTGGCGGAG

TGGAAGAACCGCGGCAGGGGCCAAGCCTCCTCAACTATGACCTCAACCGGCCAGGATTCCACCACAACCA

GGCAGCGAAGAAGTAGGCAGAACCCCCAGTCGCCCCCTCAGGACTCCAGTGTCACTTCGAAGCGAAATAT

TAAAAAGGGAGCCGTTCCCCGCTCTATCCCCAATCTAGCGGAGGTAAAGAAGAAAGGCAAAATGAAGAAG

CTCGGCCAAGCAATGGAAGAAGACCTAATCGTGGGACTGCAAGGGATGGATCTGAACCTTGAGGCTGAAG

CACTGGCTGGCACTGGCTTGGTGTTGGATGAGCAGTTAAATGAATTCCATTGCCTCTGGGATGACAGCTT

CCCGGAAGGCCCTGAGCGGCTCCATGCCATCAAGGAGCAACTGATCCAGGAGGGCCTCCTAGATCGCTGC

GTGTCCTTTCAGGCCCGGTTTGCTGAAAAGGAAGAGCTGATGTTGGTTCACAGCCTAGAATATATTGATC

TGATGGAAACAACCCAGTACATGAATGAGGGAGAACTCCGTGTCCTAGCAGACACCTACGACTCAGTTTA

TCTGCATCCGAACTCATACTCCTGTGCCTGCCTGGCCTCAGGCTCTGTCCTCAGGCTGGTGGATGCGGTC

CTGGGGGCTGAGATCCGGAATGGCATGGCCATCATTAGGCCTCCTGGACATCACGCCCAGCACAGTCTTA

TGGATGGCTATTGCATGTTCAACCACGTGGCTGTGGCAGCCCGCTATGCTCAACAGAAACACCGCATCCG

GAGGGTCCTTATCGTAGATTGGGATGTGCACCACGGTCAAGGAACACAGTTCACCTTCGACCAGGACCCC

AGTGTCCTCTATTTCTCCATCCACCGCTACGAGCAGGGTAGGTTCTGGCCCCACCTGAAGGCCTCTAACT

GGTCCACCACAGGTTTCGGCCAAGGCCAAGGATATACCATCAATGTGCCTTGGAACCAGGTGGGGATGCG

GGATGCTGACTACATTGCTGCTTTCCTGCACGTCCTGCTGCCAGTCGCCCTCGAGTTCCAGCCTCAGCTG

GTCCTGGTGGCTGCTGGATTTGATGCCCTGCAAGGGGACCCCAAGGGTGAGATGGCCGCCACTCCGGCAG

GGTTCGCCCAGCTAACCCACCTGCTCATGGGTCTGGCAGGAGGCAAGCTGATCCTGTCTCTGGAGGGTGG

CTACAACCTCCGCGCCCTGGCTGAAGGCGTCAGTGCTTCGCTCCACACCCTTCTGGGAGACCCTTGCCCC

ATGCTGGAGTCACCTGGTGCCCCCTGCCGGAGTGCCCAGGCTTCAGTTTCCTGTGCTCTGGAAGCCCTTG

AGCCCTTCTGGGAGGTTCTTGTGAGATCAACTGAGACCGTGGAGAGGGACAACATGGAGGAGGACAATGT

AGAGGAGAGCGAGGAGGAAGGACCCTGGGAGCCCCCTGTGCTCCCAATCCTGACATGGCCAGTGCTACAG

TCTCGCACAGGGCTGGTCTATGACCAAAATATGATGAATCACTGCAACTTGTGGGACAGCCACCACCCTG

AGGTACCCCAGCGCATCTTGCGGATCATGTGCCGTCTGGAGGAGCTGGGCCTTGCCGGGCGCTGCCTCAC

CCTGACACCGCGCCCTGCCACAGAGGCTGAGCTGCTCACCTGTCACAGTGCTGAGTACGTGGGTCATCTC

CGGGCCACAGAGAAAATGAAAACCCGGGAGCTGCACCGTGAGAGTTCCAACTTTGACTCCATCTATATCT

GCCCCAGTACCTTCGCCTGTGCACAGCTTGCCACTGGCGCTGCCTGCCGCCTGGTGGAGGCTGTGCTCTC

AGGAGAGGTTCTGAATGGTGCTGCTGTGGTGCGTCCCCCAGGACACCACGCAGAGCAGGATGCAGCTTGC

GGTTTTTGCTTTTTCAACTCTGTGGCTGTGGCTGCTCGCCATGCCCAGACTATCAGTGGGCATGCCCTAC

GGATCCTGATTGTGGATTGGGATGTCCACCACGGTAATGGAACTCAGCACATGTTTGAGGATGACCCCAG

TGTGCTATATGTGTCCCTGCACCGCTATGATCATGGCACCTTCTTCCCCATGGGGGATGAGGGTGCCAGC

AGCCAGATCGGCCGGGCTGCGGGCACAGGCTTCACCGTCAACGTGGCATGGAACGGGCCCCGCATGGGTG

ATGCTGACTACCTAGCTGCCTGGCATCGCCTGGTGCTTCCCATTGCCTACGAGTTTAACCCAGAACTGGT

GCTGGTCTCAGCTGGCTTTGATGCTGCACGGGGGGATCCGCTGGGGGGCTGCCAGGTGTCACCTGAGGGT

TATGCCCACCTCACCCACCTGCTGATGGGCCTTGCCAGTGGCCGCATTATCCTTATCCTAGAGGGTGGCT

ATAACCTGACATCCATCTCAGAGTCCATGGCTGCCTGCACTCGCTCCCTCCTTGGAGACCCACCACCCCT

GCTGACCCTGCCACGGCCCCCACTATCAGGGGCCCTGGCCTCAATCACTGAGACCATCCAAGTCCATCGC

AGATACTGGCGCAGCTTACGGGTCATGAAGGTAGAAGACAGAGAAGGACCCTCCAGTTCTAAGTTGGTCA

CCAAGAAGGCACCCCAACCAGCCAAACCTAGGTTAGCTGAGCGGATGACCACACGAGAAAAGAAGGTTCT

GGAAGCAGGCATGGGGAAAGTCACCTCGGCATCATTTGGGGAAGAGTCCACTCCAGGCCAGACTAACTCA

GAGACAGCTGTGGTGGCCCTCACTCAGGACCAGCCCTCAGAGGCAGCCACAGGGGGAGCCACTCTGGCCC

AGACCATTTCTGAGGCAGCCATTGGGGGAGCCATGCTGGGCCAGACCACCTCAGAGGAGGCTGTCGGGGG

AGCCACTCCGGACCAGACCACCTCAGAGGAGACTGTGGGAGGAGCCATTCTGGACCAGACCACCTCAGAG

GATGCTGTTGGGGGAGCCACGCTGGGCCAGACTACCTCAGAGGAGGCTGTAGGAGGAGCTACACTGGCCC

AGACCACCTCGGAGGCAGCCATGGAGGGAGCCACACTGGACCAGACTACGTCAGAGGAGGCTCCAGGGGG

CACCGAGCTGATCCAAACTCCTCTAGCCTCGAGCACAGACCACCAGACCCCCCCAACCTCACCTGTGCAG

GGAACTACACCCCAGATATCTCCCAGTACACTGATTGGGAGTCTCAGGACCTTGGAGCTAGGCAGCGAAT

CTCAGGGGGCCTCAGAATCTCAGGCCCCAGGAGAGGAGAACCTACTAGGAGAGGCAGCTGGAGGTCAGGA

CATGGCTGATTCGATGCTGATGCAGGGATCTAGGGGCCTCACTGATCAGGCCATATTTTATGCTGTGACA

CCACTGCCCTGGTGTCCCCATTTGGTGGCAGTATGCCCCATACCTGCAGCAGGCCTAGACGTGACCCAAC

CTTGTGGGGACTGTGGAACAATCCAAGAGAATTGGGTGTGTCTCTCTTGCTATCAGGTCTACTGTGGTCG

TTACATCAATGGCCACATGCTCCAACACCATGGAAATTCTGGACACCCGCTGGTCCTCAGCTACATCGAC

CTGTCAGCCTGGTGTTACTACTGTCAGGCCTATGTCCACCACCAGGCTCTCCTAGATGTGAAGAACATCG

CCCACCAGAACAAGTTTGGGGAGGATATGCCCCACCCACACTAAGCCCCAGAATACGGTCCCTCTTCACC

TTCTGAGGCCCACGATAGACCAGCTGTAGCTCATTCCAGCCTGTACCTTGGATGAGGGGTAGCCTCCCAC

TGCATCCCATCCTGAATATCCTTTGCAACTCCCCAAGAGTGCTTATTTAAGTGTTAATACTTTTAAGAGA

ACTGCGACGATTAATTGTGGATCTCCCCCTGCCCATTGCCTGCTTGAGGGGCACCACTACTCCAGCCCAG

AAGGAAAGGGGGGCAGCTCAGTGGCCCCAAGAGGGAGCTGATATCATGAGGATAACATTGGCGGGAGGGG

AGTTAACTGGCAGGCATGGCAAGGTTGCATATGTAATAAAGTACAAGCTGTTAAAAAA

>NM_004712.5 Homo sapiens hepatocyte growth factor-regulated tyrosine kinase substrate (HGS), mRNA

GCGCCAGCTCGTAGCAGGGGAGCGCCCGCGGCGTCGGGTTTGGGCTGGAGGTCGCCATGGGGCGAGGCAG

CGGCACCTTCGAGCGTCTCCTAGACAAGGCGACCAGCCAGCTCCTGTTGGAGACAGATTGGGAGTCCATT

TTGCAGATCTGCGACCTGATCCGCCAAGGGGACACACAAGCAAAATATGCTGTGAATTCCATCAAGAAGA

AAGTCAACGACAAGAACCCACACGTCGCCTTGTATGCCCTGGAGGTCATGGAATCTGTGGTAAAGAACTG

TGGCCAGACAGTTCATGATGAGGTGGCCAACAAGCAGACCATGGAGGAGCTGAAGGACCTGCTGAAGAGA

CAAGTGGAGGTAAACGTCCGTAACAAGATCCTGTACCTGATCCAGGCCTGGGCGCATGCCTTCCGGAACG

AGCCCAAGTACAAGGTGGTCCAGGACACCTACCAGATCATGAAGGTGGAGGGGCACGTCTTTCCAGAATT

CAAAGAGAGCGATGCCATGTTTGCTGCCGAGAGAGCCCCAGACTGGGTGGACGCTGAGGAATGCCACCGC

TGCAGGGTGCAGTTCGGGGTGATGACCCGTAAGCACCACTGCCGGGCGTGTGGGCAGATATTCTGTGGAA

AGTGTTCTTCCAAGTACTCCACCATCCCCAAGTTTGGCATCGAGAAGGAGGTGCGCGTGTGTGAGCCCTG

CTACGAGCAGCTGAACAGGAAAGCGGAGGGAAAGGCCACTTCCACCACTGAGCTGCCCCCCGAGTACCTG

ACCAGCCCCCTGTCTCAGCAGTCCCAGCTGCCCCCCAAGAGGGACGAGACGGCCCTGCAGGAGGAGGAGG

AGCTGCAGCTGGCCCTGGCGCTGTCACAGTCAGAGGCGGAGGAGAAGGAGAGGCTGAGACAGAAGTCCAC

GTACACTTCGTACCCCAAGGCGGAGCCCATGCCCTCGGCCTCCTCAGCGCCCCCCGCCAGCAGCCTGTAC

TCTTCACCTGTGAACTCGTCGGCGCCTCTGGCTGAGGACATCGACCCTGAGCTCGCACGGTATCTCAACC

GGAACTACTGGGAGAAGAAGCAGGAGGAGGCTCGCAAGAGCCCCACGCCATCTGCGCCCGTGCCCCTGAC

GGAGCCGGCTGCACAGCCTGGGGAAGGGCACGCAGCCCCCACCAACGTGGTGGAGAACCCCCTCCCGGAG

ACAGACTCTCAGCCCATTCCTCCCTCTGGTGGCCCCTTTAGTGAGCCACAGTTCCACAATGGCGAGTCTG

AGGAGAGCCACGAGCAGTTCCTGAAGGCGCTGCAGAACGCCGTCACCACCTTCGTGAACCGCATGAAGAG

TAACCACATGCGGGGCCGCAGCATCACCAATGACTCGGCCGTGCTCTCACTCTTCCAGTCCATCAACGGC

ATGCACCCGCAGCTGCTGGAGCTGCTCAACCAGCTGGACGAGCGCAGGCTGTACTATGAGGGGCTGCAGG

ACAAGCTGGCACAGATCCGCGATGCCCGGGGGGCGCTGAGTGCCCTGCGCGAAGAGCACCGGGAGAAGCT

TCGCCGGGCAGCCGAGGAGGCAGAGCGCCAGCGCCAGATCCAGCTGGCCCAGAAGCTGGAGATAATGCGG

CAGAAGAAGCAGGAGTACCTGGAGGTGCAGAGGCAGCTGGCCATCCAGCGCCTGCAGGAGCAGGAGAAGG

AGCGGCAGATGCGGCTGGAGCAGCAGAAGCAGACGGTCCAGATGCGCGCGCAGATGCCCGCCTTCCCCCT

GCCCTACGCCCAGCTCCAGGCCATGCCCGCAGCCGGAGGTGTGCTCTACCAGCCCTCGGGACCAGCCAGC

TTCCCCAGCACCTTCAGCCCTGCCGGCTCGGTGGAGGGCTCCCCAATGCACGGCGTGTACATGAGCCAGC

CGGCCCCTGCCGCTGGCCCCTACCCCAGCATGCCCAGCACTGCGGCTGATCCCAGCATGGTGAGTGCCTA

CATGTACCCAGCAGGGGCCACTGGGGCGCAGGCGGCCCCCCAGGCCCAGGCCGGACCCACCGCCAGCCCC

GCTTACTCATCCTACCAGCCTACTCCCACAGCGGGCTACCAGAACGTGGCCTCCCAGGCCCCACAGAGCC

TCCCGGCCATCTCTCAGCCTCCGCAGTCCAGCACCATGGGCTACATGGGGAGCCAGTCAGTCTCCATGGG

CTACCAGCCTTACAACATGCAGAATCTCATGACCACCCTCCCAAGCCAGGATGCGTCTCTGCCACCCCAG

CAGCCCTACATCGCGGGGCAGCAGCCCATGTACCAGCAGATGGCACCCTCTGGCGGTCCCCCCCAGCAGC

AGCCCCCCGTGGCCCAGCAACCGCAGGCACAGGGGCCGCCGGCACAGGGCAGCGAGGCCCAGCTCATTTC

ATTCGACTGACCCAGGCCATGCTCACGTCCGGAGTAACACTACATACAGTTCACCTGAAACGCCTCGTCT

CTAACTGCCGTCGTCCTGCCTCCCTGTCCTCTACTGCCGGTAGTGTCCCTTCTCTGCGAGTGAGGGGGGG

CCTTCACCCCAAGCCCACCTCCCTTGTCCTCAGCCTACTGCAGTCCCTGAGTTAGTCTCTGCTTTCTTTC

CCCAGGGCTGGGCCATGGGGAGGGAAGGACTTTCTCCCAGGGGAAGCCCCCAGCCCTGTGGGTCATGGTC

TGTGAGAGGTGGCAGGAATGGGGACCCTCACCCCCCAAGCAGCCTGTGCCCTCTGGCCGCACTGTGAGCT

GGCTGTGGTGTCTGGGTGTGGCCTGGGGCTCCCTCTGCAGGGGCCTCTCTCGGCAGCCACAGCCAAGGGT

GGAGGCTTCAGGTCTCCAGCTTCTCTGCTTCTCAGCTGCCATCTCCAGTGCCCCAGAATGGTACAGCGAT

AATAAAATGTATTTCAGAAAGCA

>NM_001530.4 Homo sapiens hypoxia inducible factor 1 subunit alpha (HIF1A), transcript variant 1, mRNA

AGTGCACAGTGCTGCCTCGTCTGAGGGGACAGGAGGATCACCCTCTTCGTCGCTTCGGCCAGTGTGTCGG

GCTGGGCCCTGACAAGCCACCTGAGGAGAGGCTCGGAGCCGGGCCCGGACCCCGGCGATTGCCGCCCGCT

TCTCTCTAGTCTCACGAGGGGTTTCCCGCCTCGCACCCCCACCTCTGGACTTGCCTTTCCTTCTCTTCTC

CGCGTGTGGAGGGAGCCAGCGCTTAGGCCGGAGCGAGCCTGGGGGCCGCCCGCCGTGAAGACATCGCGGG

GACCGATTCACCATGGAGGGCGCCGGCGGCGCGAACGACAAGAAAAAGATAAGTTCTGAACGTCGAAAAG

AAAAGTCTCGAGATGCAGCCAGATCTCGGCGAAGTAAAGAATCTGAAGTTTTTTATGAGCTTGCTCATCA

GTTGCCACTTCCACATAATGTGAGTTCGCATCTTGATAAGGCCTCTGTGATGAGGCTTACCATCAGCTAT

TTGCGTGTGAGGAAACTTCTGGATGCTGGTGATTTGGATATTGAAGATGACATGAAAGCACAGATGAATT

GCTTTTATTTGAAAGCCTTGGATGGTTTTGTTATGGTTCTCACAGATGATGGTGACATGATTTACATTTC

TGATAATGTGAACAAATACATGGGATTAACTCAGTTTGAACTAACTGGACACAGTGTGTTTGATTTTACT

CATCCATGTGACCATGAGGAAATGAGAGAAATGCTTACACACAGAAATGGCCTTGTGAAAAAGGGTAAAG

AACAAAACACACAGCGAAGCTTTTTTCTCAGAATGAAGTGTACCCTAACTAGCCGAGGAAGAACTATGAA

CATAAAGTCTGCAACATGGAAGGTATTGCACTGCACAGGCCACATTCACGTATATGATACCAACAGTAAC

CAACCTCAGTGTGGGTATAAGAAACCACCTATGACCTGCTTGGTGCTGATTTGTGAACCCATTCCTCACC

CATCAAATATTGAAATTCCTTTAGATAGCAAGACTTTCCTCAGTCGACACAGCCTGGATATGAAATTTTC

TTATTGTGATGAAAGAATTACCGAATTGATGGGATATGAGCCAGAAGAACTTTTAGGCCGCTCAATTTAT

GAATATTATCATGCTTTGGACTCTGATCATCTGACCAAAACTCATCATGATATGTTTACTAAAGGACAAG

TCACCACAGGACAGTACAGGATGCTTGCCAAAAGAGGTGGATATGTCTGGGTTGAAACTCAAGCAACTGT

CATATATAACACCAAGAATTCTCAACCACAGTGCATTGTATGTGTGAATTACGTTGTGAGTGGTATTATT

CAGCACGACTTGATTTTCTCCCTTCAACAAACAGAATGTGTCCTTAAACCGGTTGAATCTTCAGATATGA

AAATGACTCAGCTATTCACCAAAGTTGAATCAGAAGATACAAGTAGCCTCTTTGACAAACTTAAGAAGGA

ACCTGATGCTTTAACTTTGCTGGCCCCAGCCGCTGGAGACACAATCATATCTTTAGATTTTGGCAGCAAC

GACACAGAAACTGATGACCAGCAACTTGAGGAAGTACCATTATATAATGATGTAATGCTCCCCTCACCCA

ACGAAAAATTACAGAATATAAATTTGGCAATGTCTCCATTACCCACCGCTGAAACGCCAAAGCCACTTCG

AAGTAGTGCTGACCCTGCACTCAATCAAGAAGTTGCATTAAAATTAGAACCAAATCCAGAGTCACTGGAA

CTTTCTTTTACCATGCCCCAGATTCAGGATCAGACACCTAGTCCTTCCGATGGAAGCACTAGACAAAGTT

CACCTGAGCCTAATAGTCCCAGTGAATATTGTTTTTATGTGGATAGTGATATGGTCAATGAATTCAAGTT

GGAATTGGTAGAAAAACTTTTTGCTGAAGACACAGAAGCAAAGAACCCATTTTCTACTCAGGACACAGAT

TTAGACTTGGAGATGTTAGCTCCCTATATCCCAATGGATGATGACTTCCAGTTACGTTCCTTCGATCAGT

TGTCACCATTAGAAAGCAGTTCCGCAAGCCCTGAAAGCGCAAGTCCTCAAAGCACAGTTACAGTATTCCA

GCAGACTCAAATACAAGAACCTACTGCTAATGCCACCACTACCACTGCCACCACTGATGAATTAAAAACA

GTGACAAAAGACCGTATGGAAGACATTAAAATATTGATTGCATCTCCATCTCCTACCCACATACATAAAG

AAACTACTAGTGCCACATCATCACCATATAGAGATACTCAAAGTCGGACAGCCTCACCAAACAGAGCAGG

AAAAGGAGTCATAGAACAGACAGAAAAATCTCATCCAAGAAGCCCTAACGTGTTATCTGTCGCTTTGAGT

CAAAGAACTACAGTTCCTGAGGAAGAACTAAATCCAAAGATACTAGCTTTGCAGAATGCTCAGAGAAAGC

GAAAAATGGAACATGATGGTTCACTTTTTCAAGCAGTAGGAATTGGAACATTATTACAGCAGCCAGACGA

TCATGCAGCTACTACATCACTTTCTTGGAAACGTGTAAAAGGATGCAAATCTAGTGAACAGAATGGAATG

GAGCAAAAGACAATTATTTTAATACCCTCTGATTTAGCATGTAGACTGCTGGGGCAATCAATGGATGAAA

GTGGATTACCACAGCTGACCAGTTATGATTGTGAAGTTAATGCTCCTATACAAGGCAGCAGAAACCTACT

GCAGGGTGAAGAATTACTCAGAGCTTTGGATCAAGTTAACTGAGCTTTTTCTTAATTTCATTCCTTTTTT

TGGACACTGGTGGCTCATTACCTAAAGCAGTCTATTTATATTTTCTACATCTAATTTTAGAAGCCTGGCT

ACAATACTGCACAAACTTGGTTAGTTCAATTTTGATCCCCTTTCTACTTAATTTACATTAATGCTCTTTT

TTAGTATGTTCTTTAATGCTGGATCACAGACAGCTCATTTTCTCAGTTTTTTGGTATTTAAACCATTGCA

TTGCAGTAGCATCATTTTAAAAAATGCACCTTTTTATTTATTTATTTTTGGCTAGGGAGTTTATCCCTTT

TTCGAATTATTTTTAAGAAGATGCCAATATAATTTTTGTAAGAAGGCAGTAACCTTTCATCATGATCATA

GGCAGTTGAAAAATTTTTACACCTTTTTTTTCACATTTTACATAAATAATAATGCTTTGCCAGCAGTACG

TGGTAGCCACAATTGCACAATATATTTTCTTAAAAAATACCAGCAGTTACTCATGGAATATATTCTGCGT

TTATAAAACTAGTTTTTAAGAAGAAATTTTTTTTGGCCTATGAAATTGTTAAACCTGGAACATGACATTG

TTAATCATATAATAATGATTCTTAAATGCTGTATGGTTTATTATTTAAATGGGTAAAGCCATTTACATAA

TATAGAAAGATATGCATATATCTAGAAGGTATGTGGCATTTATTTGGATAAAATTCTCAATTCAGAGAAA

TCATCTGATGTTTCTATAGTCACTTTGCCAGCTCAAAAGAAAACAATACCCTATGTAGTTGTGGAAGTTT

ATGCTAATATTGTGTAACTGATATTAAACCTAAATGTTCTGCCTACCCTGTTGGTATAAAGATATTTTGA

GCAGACTGTAAACAAGAAAAAAAAAATCATGCATTCTTAGCAAAATTGCCTAGTATGTTAATTTGCTCAA

AATACAATGTTTGATTTTATGCACTTTGTCGCTATTAACATCCTTTTTTTCATGTAGATTTCAATAATTG

AGTAATTTTAGAAGCATTATTTTAGGAATATATAGTTGTCACAGTAAATATCTTGTTTTTTCTATGTACA

TTGTACAAATTTTTCATTCCTTTTGCTCTTTGTGGTTGGATCTAACACTAACTGTATTGTTTTGTTACAT

CAAATAAACATCTTCTGTGGACCAGG

>NM_007355.4 Homo sapiens heat shock protein 90 alpha family class B member 1 (HSP90AB1), transcript variant 2, mRNA

CTCTCGAGTCACTCCGGCGCAGTGTTGGGACTGTCTGGGTATCGGAAAGCAAGCCTACGTTGCTCACTAT

TACGTATAATCCTTTTCTTTTCAAGATGCCTGAGGAAGTGCACCATGGAGAGGAGGAGGTGGAGACTTTT

GCCTTTCAGGCAGAAATTGCCCAACTCATGTCCCTCATCATCAATACCTTCTATTCCAACAAGGAGATTT

TCCTTCGGGAGTTGATCTCTAATGCTTCTGATGCCTTGGACAAGATTCGCTATGAGAGCCTGACAGACCC

TTCGAAGTTGGACAGTGGTAAAGAGCTGAAAATTGACATCATCCCCAACCCTCAGGAACGTACCCTGACT

TTGGTAGACACAGGCATTGGCATGACCAAAGCTGATCTCATAAATAATTTGGGAACCATTGCCAAGTCTG

GTACTAAAGCATTCATGGAGGCTCTTCAGGCTGGTGCAGACATCTCCATGATTGGGCAGTTTGGTGTTGG

CTTTTATTCTGCCTACTTGGTGGCAGAGAAAGTGGTTGTGATCACAAAGCACAACGATGATGAACAGTAT

GCTTGGGAGTCTTCTGCTGGAGGTTCCTTCACTGTGCGTGCTGACCATGGTGAGCCCATTGGCAGGGGTA

CCAAAGTGATCCTCCATCTTAAAGAAGATCAGACAGAGTACCTAGAAGAGAGGCGGGTCAAAGAAGTAGT

GAAGAAGCATTCTCAGTTCATAGGCTATCCCATCACCCTTTATTTGGAGAAGGAACGAGAGAAGGAAATT

AGTGATGATGAGGCAGAGGAAGAGAAAGGTGAGAAAGAAGAGGAAGATAAAGATGATGAAGAAAAACCCA

AGATCGAAGATGTGGGTTCAGATGAGGAGGATGACAGCGGTAAGGATAAGAAGAAGAAAACTAAGAAGAT

CAAAGAGAAATACATTGATCAGGAAGAACTAAACAAGACCAAGCCTATTTGGACCAGAAACCCTGATGAC

ATCACCCAAGAGGAGTATGGAGAATTCTACAAGAGCCTCACTAATGACTGGGAAGACCACTTGGCAGTCA

AGCACTTTTCTGTAGAAGGTCAGTTGGAATTCAGGGCATTGCTATTTATTCCTCGTCGGGCTCCCTTTGA

CCTTTTTGAGAACAAGAAGAAAAAGAACAACATCAAACTCTATGTCCGCCGTGTGTTCATCATGGACAGC

TGTGATGAGTTGATACCAGAGTATCTCAATTTTATCCGTGGTGTGGTTGACTCTGAGGATCTGCCCCTGA

ACATCTCCCGAGAAATGCTCCAGCAGAGCAAAATCTTGAAAGTCATTCGCAAAAACATTGTTAAGAAGTG

CCTTGAGCTCTTCTCTGAGCTGGCAGAAGACAAGGAGAATTACAAGAAATTCTATGAGGCATTCTCTAAA

AATCTCAAGCTTGGAATCCACGAAGACTCCACTAACCGCCGCCGCCTGTCTGAGCTGCTGCGCTATCATA

CCTCCCAGTCTGGAGATGAGATGACATCTCTGTCAGAGTATGTTTCTCGCATGAAGGAGACACAGAAGTC

CATCTATTACATCACTGGTGAGAGCAAAGAGCAGGTGGCCAACTCAGCTTTTGTGGAGCGAGTGCGGAAA

CGGGGCTTCGAGGTGGTATATATGACCGAGCCCATTGACGAGTACTGTGTGCAGCAGCTCAAGGAATTTG

ATGGGAAGAGCCTGGTCTCAGTTACCAAGGAGGGTCTGGAGCTGCCTGAGGATGAGGAGGAGAAGAAGAA

GATGGAAGAGAGCAAGGCAAAGTTTGAGAACCTCTGCAAGCTCATGAAAGAAATCTTAGATAAGAAGGTT

GAGAAGGTGACAATCTCCAATAGACTTGTGTCTTCACCTTGCTGCATTGTGACCAGCACCTACGGCTGGA

CAGCCAATATGGAGCGGATCATGAAAGCCCAGGCACTTCGGGACAACTCCACCATGGGCTATATGATGGC

CAAAAAGCACCTGGAGATCAACCCTGACCACCCCATTGTGGAGACGCTGCGGCAGAAGGCTGAGGCCGAC

AAGAATGATAAGGCAGTTAAGGACCTGGTGGTGCTGCTGTTTGAAACCGCCCTGCTATCTTCTGGCTTTT

CCCTTGAGGATCCCCAGACCCACTCCAACCGCATCTATCGCATGATCAAGCTAGGTCTAGGTATTGATGA

AGATGAAGTGGCAGCAGAGGAACCCAATGCTGCAGTTCCTGATGAGATCCCCCCTCTCGAGGGCGATGAG

GATGCGTCTCGCATGGAAGAAGTCGATTAGGTTAGGAGTTCATAGTTGGAAAACTTGTGCCCTTGTATAG

TGTCCCCATGGGCTCCCACTGCAGCCTCGAGTGCCCCTGTCCCACCTGGCTCCCCCTGCTGGTGTCTAGT

GTTTTTTTCCCTCTCCTGTCCTTGTGTTGAAGGCAGTAAACTAAGGGTGTCAAGCCCCATTCCCTCTCTA

CTCTTGACAGCAGGATTGGATGTTGTGTATTGTGGTTTATTTTATTTTCTTCATTTTGTTCTGAAATTAA

AGTATGCAAAATAAAGAATATGCCGTTTTTATACA

>NM_005347.5 Homo sapiens heat shock protein family A (Hsp70) member 5 (HSPA5), mRNA

GACGCCGGCCAAGACAGCACAGACAGATTGACCTATTGGGGTGTTTCGCGAGTGTGAGAGGGAAGCGCCG

CGGCCTGTATTTCTAGACCTGCCCTTCGCCTGGTTCGTGGCGCCTTGTGACCCCGGGCCCCTGCCGCCTG

CAAGTCGGAAATTGCGCTGTGCTCCTGTGCTACGGCCTGTGGCTGGACTGCCTGCTGCTGCCCAACTGGC

TGGCAAGATGAAGCTCTCCCTGGTGGCCGCGATGCTGCTGCTGCTCAGCGCGGCGCGGGCCGAGGAGGAG

GACAAGAAGGAGGACGTGGGCACGGTGGTCGGCATCGACCTGGGGACCACCTACTCCTGCGTCGGCGTGT

TCAAGAACGGCCGCGTGGAGATCATCGCCAACGATCAGGGCAACCGCATCACGCCGTCCTATGTCGCCTT

CACTCCTGAAGGGGAACGTCTGATTGGCGATGCCGCCAAGAACCAGCTCACCTCCAACCCCGAGAACACG

GTCTTTGACGCCAAGCGGCTCATCGGCCGCACGTGGAATGACCCGTCTGTGCAGCAGGACATCAAGTTCT

TGCCGTTCAAGGTGGTTGAAAAGAAAACTAAACCATACATTCAAGTTGATATTGGAGGTGGGCAAACAAA

GACATTTGCTCCTGAAGAAATTTCTGCCATGGTTCTCACTAAAATGAAAGAAACCGCTGAGGCTTATTTG

GGAAAGAAGGTTACCCATGCAGTTGTTACTGTACCAGCCTATTTTAATGATGCCCAACGCCAAGCAACCA

AAGACGCTGGAACTATTGCTGGCCTAAATGTTATGAGGATCATCAACGAGCCTACGGCAGCTGCTATTGC

TTATGGCCTGGATAAGAGGGAGGGGGAGAAGAACATCCTGGTGTTTGACCTGGGTGGCGGAACCTTCGAT

GTGTCTCTTCTCACCATTGACAATGGTGTCTTCGAAGTTGTGGCCACTAATGGAGATACTCATCTGGGTG

GAGAAGACTTTGACCAGCGTGTCATGGAACACTTCATCAAACTGTACAAAAAGAAGACGGGCAAAGATGT

CAGGAAAGACAATAGAGCTGTGCAGAAACTCCGGCGCGAGGTAGAAAAGGCCAAACGGGCCCTGTCTTCT

CAGCATCAAGCAAGAATTGAAATTGAGTCCTTCTATGAAGGAGAAGACTTTTCTGAGACCCTGACTCGGG

CCAAATTTGAAGAGCTCAACATGGATCTGTTCCGGTCTACTATGAAGCCCGTCCAGAAAGTGTTGGAAGA

TTCTGATTTGAAGAAGTCTGATATTGATGAAATTGTTCTTGTTGGTGGCTCGACTCGAATTCCAAAGATT

CAGCAACTGGTTAAAGAGTTCTTCAATGGCAAGGAACCATCCCGTGGCATAAACCCAGATGAAGCTGTAG

CGTATGGTGCTGCTGTCCAGGCTGGTGTGCTCTCTGGTGATCAAGATACAGGTGACCTGGTACTGCTTGA

TGTATGTCCCCTTACACTTGGTATTGAAACTGTGGGAGGTGTCATGACCAAACTGATTCCAAGGAACACA

GTGGTGCCTACCAAGAAGTCTCAGATCTTTTCTACAGCTTCTGATAATCAACCAACTGTTACAATCAAGG

TCTATGAAGGTGAAAGACCCCTGACAAAAGACAATCATCTTCTGGGTACATTTGATCTGACTGGAATTCC

TCCTGCTCCTCGTGGGGTCCCACAGATTGAAGTCACCTTTGAGATAGATGTGAATGGTATTCTTCGAGTG

ACAGCTGAAGACAAGGGTACAGGGAACAAAAATAAGATCACAATCACCAATGACCAGAATCGCCTGACAC

CTGAAGAAATCGAAAGGATGGTTAATGATGCTGAGAAGTTTGCTGAGGAAGACAAAAAGCTCAAGGAGCG

CATTGATACTAGAAATGAGTTGGAAAGCTATGCCTATTCTCTAAAGAATCAGATTGGAGATAAAGAAAAG

CTGGGAGGTAAACTTTCCTCTGAAGATAAGGAGACCATGGAAAAAGCTGTAGAAGAAAAGATTGAATGGC

TGGAAAGCCACCAAGATGCTGACATTGAAGACTTCAAAGCTAAGAAGAAGGAACTGGAAGAAATTGTTCA

ACCAATTATCAGCAAACTCTATGGAAGTGCAGGCCCTCCCCCAACTGGTGAAGAGGATACAGCAGAAAAA

GATGAGTTGTAGACACTGATCTGCTAGTGCTGTAATATTGTAAATACTGGACTCAGGAACTTTTGTTAGG

AAAAAATTGAAAGAACTTAAGTCTCGAATGTAATTGGAATCTTCACCTCAGAGTGGAGTTGAAACTGCTA

TAGCCTAAGCGGCTGTTTACTGCTTTTCATTAGCAGTTGCTCACATGTCTTTGGGTGGGGGGGAGAAGAA

GAATTGGCCATCTTAAAAAGCGGGTAAAAAACCTGGGTTAGGGTGTGTGTTCACCTTCAAAATGTTCTAT

TTAACAACTGGGTCATGTGCATCTGGTGTAGGAAGTTTTTTCTACCATAAGTGACACCAATAAATGTTTG

TTATTTACACTGGTCTAATGTTTGTGAGAAGCTTCTAATTAGATCAATTACTTATTTTAGGAAATTTAAG

ACTAGATACTCGTGTGTGGGGTGAGGGGAGGGAGTATTTGGTATGTTGGGATAAGGAAACACTTCTATTT

AATGCTTCCAGGGATTTTTTTTTTTTTTTTTAACCCTCCTGGGCCCAAGTGATCCTTCCACCTCAGTCTC

CCAGCTAATTGAGACCACAGGCTTGTTACCACCATGCTCGGCTTTTGCATTAATCTAAGAAAAGGGGAGA

GAAGTTAATCCACATCTTTACTCAGGCAAGGGGCATTTCACAGTGCCCAAGAGTGGGGTTTTCTTGAACA

TACTTGGTTTCCTATTTCCCCTTATCTTTCTAAAACTGCCTTTCTGGTGGCTTTTTTTAAAATTATTACT

AATGATGCTTTTATAGCTGCTTGGATTCTCTGAGAAATGATGGGGAGTGAGTGATCACTGGTATTAACTT

TATACACTTGGATTTCATTTGTAACTTTAGGATGTAAAGGTATATTGTGAACCCTAGCTGTGTCAGAATC

TCCATCCCTGAAATTTCTCATTAGTGGTACTGGGGTGGGATCTTGGATGGTGACATTGAAACTACACTAA

ATCCCCTCACTATGAATGGGTTGTTAAAGGCAATGGTTTGTGTCAAAACTGGTTTAGGATTACTTAGATT

GTGTTCCTGAAGAAAAGAGTCCAGGTAAATGGTATGATCAATAAAGGACAGGCTGGTGCTAACATAAAAT

CCAATATTGTAATCCTAGCACTTTGGGAGGCCAAGGCGGGTGGATCACAAGGTCAAGAGATAGAGACCAT

CTTTGCCAACATGGTGAAACTCCATCTCTACTGAAAATACAAAAATTAGCTGGGCGTGGTAGTGCAAGCT

GAAGGCTGAGGCAGGAGAATCACTCGAACCCGGGAGGCAGAGGTTGCAGTGAGCCGAGATCACACCACTG

TACTCCAGCCCGGCACTCCAGCCTGGCGACAAGAGTGAGACTCCACCTCAAAAAAAAAAAAAAGAATCCA

ATACTGCCCAAGGATAGGTATTTTATAGATGGGCAACTGGCTGAAAGGTTAATTCTCTAGGGCTAGTAGA

ACTGGATCCCAACACCAAACTCTTAATTAGACCTAGGCCTCAGCTGCACTGCCCGAAAAGCATTTGGGCA

GACCCTGAGCAGAATACTGGTCTCAGGCCAAGCCCAATACAGCCATTAAAGATGACCTACAGTGCTGTGT

ACCCTGGGGCAATAGGGTTAAATGGTAGTTAGCAACTAGGGCTAGTCTTCCCTTACCTCAAAGGCTCTCA

CTACCGTGGACCACCTAGTCTGTAACTCTTTCTGAGGAGCTGTTACTGAATATTAAAAAGATAGACTTCA

A>NM_006597.6 Homo sapiens heat shock protein family A (Hsp70) member 8 (HSPA8), transcript variant 1, mRNA

CTCATTGAACTCGCCTGCAGCTCTTGGGTTTTTTGTGGCTTCCTTCGTTATTGGAGCCAGGCCTACACCC

CAGCAACCATGTCCAAGGGACCTGCAGTTGGTATTGATCTTGGCACCACCTACTCTTGTGTGGGTGTTTT

CCAGCACGGAAAAGTCGAGATAATTGCCAATGATCAGGGAAACCGAACCACTCCAAGCTATGTCGCCTTT

ACGGACACTGAACGGTTGATCGGTGATGCCGCAAAGAATCAAGTTGCAATGAACCCCACCAACACAGTTT

TTGATGCCAAACGTCTGATTGGACGCAGATTTGATGATGCTGTTGTCCAGTCTGATATGAAACATTGGCC

CTTTATGGTGGTGAATGATGCTGGCAGGCCCAAGGTCCAAGTAGAATACAAGGGAGAGACCAAAAGCTTC

TATCCAGAGGAGGTGTCTTCTATGGTTCTGACAAAGATGAAGGAAATTGCAGAAGCCTACCTTGGGAAGA

CTGTTACCAATGCTGTGGTCACAGTGCCAGCTTACTTTAATGACTCTCAGCGTCAGGCTACCAAAGATGC

TGGAACTATTGCTGGTCTCAATGTACTTAGAATTATTAATGAGCCAACTGCTGCTGCTATTGCTTACGGC

TTAGACAAAAAGGTTGGAGCAGAAAGAAACGTGCTCATCTTTGACCTGGGAGGTGGCACTTTTGATGTGT

CAATCCTCACTATTGAGGATGGAATCTTTGAGGTCAAGTCTACAGCTGGAGACACCCACTTGGGTGGAGA

AGATTTTGACAACCGAATGGTCAACCATTTTATTGCTGAGTTTAAGCGCAAGCATAAGAAGGACATCAGT

GAGAACAAGAGAGCTGTAAGACGCCTCCGTACTGCTTGTGAACGTGCTAAGCGTACCCTCTCTTCCAGCA

CCCAGGCCAGTATTGAGATCGATTCTCTCTATGAAGGAATCGACTTCTATACCTCCATTACCCGTGCCCG

ATTTGAAGAACTGAATGCTGACCTGTTCCGTGGCACCCTGGACCCAGTAGAGAAAGCCCTTCGAGATGCC

AAACTAGACAAGTCACAGATTCATGATATTGTCCTGGTTGGTGGTTCTACTCGTATCCCCAAGATTCAGA

AGCTTCTCCAAGACTTCTTCAATGGAAAAGAACTGAATAAGAGCATCAACCCTGATGAAGCTGTTGCTTA

TGGTGCAGCTGTCCAGGCAGCCATCTTGTCTGGAGACAAGTCTGAGAATGTTCAAGATTTGCTGCTCTTG

GATGTCACTCCTCTTTCCCTTGGTATTGAAACTGCTGGTGGAGTCATGACTGTCCTCATCAAGCGTAATA

CCACCATTCCTACCAAGCAGACACAGACCTTCACTACCTATTCTGACAACCAGCCTGGTGTGCTTATTCA

GGTTTATGAAGGCGAGCGTGCCATGACAAAGGATAACAACCTGCTTGGCAAGTTTGAACTCACAGGCATA

CCTCCTGCACCCCGAGGTGTTCCTCAGATTGAAGTCACTTTTGACATTGATGCCAATGGTATACTCAATG

TCTCTGCTGTGGACAAGAGTACGGGAAAAGAGAACAAGATTACTATCACTAATGACAAGGGCCGTTTGAG

CAAGGAAGACATTGAACGTATGGTCCAGGAAGCTGAGAAGTACAAAGCTGAAGATGAGAAGCAGAGGGAC

AAGGTGTCATCCAAGAATTCACTTGAGTCCTATGCCTTCAACATGAAAGCAACTGTTGAAGATGAGAAAC

TTCAAGGCAAGATTAACGATGAGGACAAACAGAAGATTCTGGACAAGTGTAATGAAATTATCAACTGGCT

TGATAAGAATCAGACTGCTGAGAAGGAAGAATTTGAACATCAACAGAAAGAGCTGGAGAAAGTTTGCAAC

CCCATCATCACCAAGCTGTACCAGAGTGCAGGAGGCATGCCAGGAGGAATGCCTGGGGGATTTCCTGGTG

GTGGAGCTCCTCCCTCTGGTGGTGCTTCCTCAGGGCCCACCATTGAAGAGGTTGATTAAGCCAACCAAGT

GTAGATGTAGCATTGTTCCACACATTTAAAACATTTGAAGGACCTAAATTCGTAGCAAATTCTGTGGCAG

TTTTAAAAAGTTAAGCTGCTATAGTAAGTTACTGGGCATTCTCAATACTTGAATATGGAACATATGCACA

GGGGAAGGAAATAACATTGCACTTTATAAACACTGTATTGTAAGTGGAAAATGCAATGTCTTAAATAAAA

CTATTTAAAATTGGCACCATACAA

>NM_014365.3 Homo sapiens heat shock protein family B (small) member 8 (HSPB8), mRNA

AGAAGAAGTTTCTAGGCGCGCGTGCCCTGGGTTTATTAAGCTCCTGGCTCCGCTCTAGACCTCAGCGGTT

CTGGCTGCCAGCCTGGGCAGCCTGGGAAGCCTGGGAGGACGGTGGCTTGCCGGTCTGTCGTGAGGCAGTG

CGGACGGGGACCCTCTGGGATTCTGCTGGATCTGCCCCGGGGGTTACCTTTGGGGGCTGGGACCCCAGTC

GAGGGGACACAACCGTCCCTGGCAGTGGTTGGTTCTGCTTCTCCCTGCAGAAAAGCAGCATTTTCGGAAG

CTGAAGAATAAGCTAGCCCAGCCACACCACCTTGTTGTGTGACCTTGGGCAGGTGGTTCTGTCTCTCTGA

GCCTCTGTTTCTCTCTGAGCTGAGCAGCCACCATGGCTGACGGTCAGATGCCCTTCTCCTGCCACTACCC

AAGCCGCCTGCGCCGAGACCCCTTCCGGGACTCTCCCCTCTCCTCTCGCCTGCTGGATGATGGCTTTGGC

ATGGACCCCTTCCCAGACGACTTGACAGCCTCTTGGCCCGACTGGGCTCTGCCTCGTCTCTCCTCCGCCT

GGCCAGGCACCCTAAGGTCGGGCATGGTGCCCCGGGGCCCCACTGCCACCGCCAGGTTTGGGGTGCCTGC

CGAGGGCAGGACCCCCCCACCCTTCCCTGGGGAGCCCTGGAAAGTGTGTGTGAATGTGCACAGCTTCAAG

CCAGAGGAGTTGATGGTGAAGACCAAAGATGGATACGTGGAGGTGTCTGGCAAACATGAAGAGAAACAGC

AAGAAGGTGGCATTGTTTCTAAGAACTTCACAAAGAAAATCCAGCTTCCTGCAGAGGTGGATCCTGTGAC

AGTATTTGCCTCACTTTCCCCAGAGGGTCTGCTGATCATCGAAGCTCCCCAGGTCCCTCCTTACTCAACA

TTTGGAGAGAGCAGTTTCAACAACGAGCTTCCCCAGGACAGCCAGGAAGTCACCTGTACCTGAGATGCCA

GTACTGGCCCATCCTTGTTTTGTCCCCAACCCTAGGGCTTCTCTGATTCCAGGATACATTACTTTAGCTG

AACTCAGATTTAGTGCAAGTAAAATGTTAGAGGGTGCGGGGGTGAGGACTGACCACAGATTCCCTGGATA

GTGTAGTGGTAGATTTCTCCACAGGATAGCGCAATTGGCAAATCATGCTTGGTTGTGTTAGGCCAAAATA

CTAGTTTTGCTTTCTTTACCTTTTCTATCTTGATGAAAATGTTGCACATTCTATAGTTGCAAAACACATA

AAAGGGGACTTAACATTTCACGTTGTATCTTACTTGCAGTGAATGCAAGGGTTACTTTTCTCTGGGGACC

TCCCCCATCACCCAGGTTCCTACTCTGGGCTCCCGATTCCCATGGCTCCCAAACCATGCCGCATGGTTTG

GTTAATGAAACCCAGTAGCTAACCCCACTGTGCTTCCACATGCCTGGCCTAAAATGGGTGATATACAGGT

CTTATATCCCCATATGGAATTTATCCATCAACCACATAAAAACAAACAGTGCCTTCTGCCCTCTGCCCAG

ATGTGTCCAGCACGTTCTCAAAGTTTCCACATTAGCACTCCCTAAGGACGCTGGGAGCCTGTCAGTTTAT

GATCTGACCTAGGTCCCCCCTTTCTTCTGTCCCCTGTGTTTAAGTCGGGATTTTTACAGAGGGAGCTGTC

TCCAGACAGCTCCATCAGGAACCAAGCAAAGGCCAGATAGCCTGACAGATAGGCTAGTGGTATTGTGTAT

ATGGGCGGGACGTGTGTGTCATTATTATTTGAGTTATGCTGTTGTTTAGGGGTAAATAACAGTAAATAAT

TAATAATAATAATAATAATAATAAAGGAGCTGACGTTCTTA

>NM_000619.3 Homo sapiens interferon gamma (IFNG), mRNA

ACATTGTTCTGATCATCTGAAGATCAGCTATTAGAAGAGAAAGATCAGTTAAGTCCTTTGGACCTGATCA

GCTTGATACAAGAACTACTGATTTCAACTTCTTTGGCTTAATTCTCTCGGAAACGATGAAATATACAAGT

TATATCTTGGCTTTTCAGCTCTGCATCGTTTTGGGTTCTCTTGGCTGTTACTGCCAGGACCCATATGTAA

AAGAAGCAGAAAACCTTAAGAAATATTTTAATGCAGGTCATTCAGATGTAGCGGATAATGGAACTCTTTT

CTTAGGCATTTTGAAGAATTGGAAAGAGGAGAGTGACAGAAAAATAATGCAGAGCCAAATTGTCTCCTTT

TACTTCAAACTTTTTAAAAACTTTAAAGATGACCAGAGCATCCAAAAGAGTGTGGAGACCATCAAGGAAG

ACATGAATGTCAAGTTTTTCAATAGCAACAAAAAGAAACGAGATGACTTCGAAAAGCTGACTAATTATTC

GGTAACTGACTTGAATGTCCAACGCAAAGCAATACATGAACTCATCCAAGTGATGGCTGAACTGTCGCCA

GCAGCTAAAACAGGGAAGCGAAAAAGGAGTCAGATGCTGTTTCGAGGTCGAAGAGCATCCCAGTAATGGT

TGTCCTGCCTGCAATATTTGAATTTTAAATCTAAATCTATTTATTAATATTTAACATTATTTATATGGGG

AATATATTTTTAGACTCATCAATCAAATAAGTATTTATAATAGCAACTTTTGTGTAATGAAAATGAATAT

CTATTAATATATGTATTATTTATAATTCCTATATCCTGTGACTGTCTCACTTAATCCTTTGTTTTCTGAC

TAATTAGGCAAGGCTATGTGATTACAAGGCTTTATCTCAGGGGCCAACTAGGCAGCCAACCTAAGCAAGA

TCCCATGGGTTGTGTGTTTATTTCACTTGATGATACAATGAACACTTATAAGTGAAGTGATACTATCCAG

TTACTGCCGGTTTGAAAATATGCCTGCAATCTGAGCCAGTGCTTTAATGGCATGTCAGACAGAACTTGAA

TGTGTCAGGTGACCCTGATGAAAACATAGCATCTCAGGAGATTTCATGCCTGGTGCTTCCAAATATTGTT

GACAACTGTGACTGTACCCAAATGGAAAGTAACTCATTTGTTAAAATTATCAATATCTAATATATATGAA

TAAAGTGTAAGTTCACAACTA

>NM_001190720.3 Homo sapiens inhibitor of nuclear factor kappa B kinase subunit beta (IKBKB), transcript variant 2, mRNA

GTGACGTCAGAGCAGGAAGTGTTTGAGGAAGTCGCGCCGCGCTGCCCGCGTTAAGATTCCCGCATTTTAA

TGTTTTCAGGGGGGTGTCATAGCCCCGGGTTTGGCCGCCCCAGCCCCGCCTTCCCCGCCCCGGGGAGCCC

GCCCCCTGCCCCGCGTCCCTGCCGACAGGAAACAGGTGAGCAGATTGCCATCAAGCAGTGCCGGCAGGAG

CTCAGCCCCCGGAACCGAGAGCGGTGGTGCCTGGAGATCCAGATCATGAGAAGGCTGACCCACCCCAATG

TGGTGGCTGCCCGAGATGTCCCTGAGGGGATGCAGAACTTGGCGCCCAATGACCTGCCCCTGCTGGCCAT

GGAGTACTGCCAAGGAGGAGATCTCCGGAAGTACCTGAACCAGTTTGAGAACTGCTGTGGTCTGCGGGAA

GGTGCCATCCTCACCTTGCTGAGTGACATTGCCTCTGCGCTTAGATACCTTCATGAAAACAGAATCATCC

ATCGGGATCTAAAGCCAGAAAACATCGTCCTGCAGCAAGGAGAACAGAGGTTAATACACAAAATTATTGA

CCTAGGATATGCCAAGGAGCTGGATCAGGGCAGTCTTTGCACATCATTCGTGGGGACCCTGCAGTACCTG

GCCCCAGAGCTACTGGAGCAGCAGAAGTACACAGTGACCGTCGACTACTGGAGCTTCGGCACCCTGGCCT

TTGAGTGCATCACGGGCTTCCGGCCCTTCCTCCCCAACTGGCAGCCCGTGCAGTGGCATTCAAAAGTGCG

GCAGAAGAGTGAGGTGGACATTGTTGTTAGCGAAGACTTGAATGGAACGGTGAAGTTTTCAAGCTCTTTA

CCCTACCCCAATAATCTTAACAGTGTCCTGGCTGAGCGACTGGAGAAGTGGCTGCAACTGATGCTGATGT

GGCACCCCCGACAGAGGGGCACGGATCCCACGTATGGGCCCAATGGCTGCTTCAAGGCCCTGGATGACAT

CTTAAACTTAAAGCTGGTTCATATCTTGAACATGGTCACGGGCACCATCCACACCTACCCTGTGACAGAG

GATGAGAGTCTGCAGAGCTTGAAGGCCAGAATCCAACAGGACACGGGCATCCCAGAGGAGGACCAGGAGC

TGCTGCAGGAAGCGGGCCTGGCGTTGATCCCCGATAAGCCTGCCACTCAGTGTATTTCAGACGGCAAGTT

AAATGAGGGCCACACATTGGACATGGATCTTGTTTTTCTCTTTGACAACAGTAAAATCACCTATGAGACT

CAGATCTCCCCACGGCCCCAACCTGAAAGTGTCAGCTGTATCCTTCAAGAGCCCAAGAGGAATCTCGCCT

TCTTCCAGCTGAGGAAGGTGTGGGGCCAGGTCTGGCACAGCATCCAGACCCTGAAGGAAGATTGCAACCG

GCTGCAGCAGGGACAGCGAGCCGCCATGATGAATCTCCTCCGAAACAACAGCTGCCTCTCCAAAATGAAG

AATTCCATGGCTTCCATGTCTCAGCAGCTCAAGGCCAAGTTGGATTTCTTCAAAACCAGCATCCAGATTG

ACCTGGAGAAGTACAGCGAGCAAACCGAGTTTGGGATCACATCAGATAAACTGCTGCTGGCCTGGAGGGA

AATGGAGCAGGCTGTGGAGCTCTGTGGGCGGGAGAACGAAGTGAAACTCCTGGTAGAACGGATGATGGCT

CTGCAGACCGACATTGTGGACTTACAGAGGAGCCCCATGGGCCGGAAGCAGGGGGGAACGCTGGACGACC

TAGAGGAGCAAGCAAGGGAGCTGTACAGGAGACTAAGGGAAAAACCTCGAGACCAGCGAACTGAGGGTGA

CAGTCAGGAAATGGTACGGCTGCTGCTTCAGGCAATTCAGAGCTTCGAGAAGAAAGTGCGAGTGATCTAT

ACGCAGCTCAGTAAAACTGTGGTTTGCAAGCAGAAGGCGCTGGAACTGTTGCCCAAGGTGGAAGAGGTGG

TGAGCTTAATGAATGAGGATGAGAAGACTGTTGTCCGGCTGCAGGAGAAGCGGCAGAAGGAGCTCTGGAA

TCTCCTGAAGATTGCTTGTAGCAAGGTCCGTGGTCCTGTCAGTGGAAGCCCGGATAGCATGAATGCCTCT

CGACTTAGCCAGCCTGGGCAGCTGATGTCTCAGCCCTCCACGGCCTCCAACAGCTTACCTGAGCCAGCCA

AGAAGAGTGAAGAACTGGTGGCTGAAGCACATAACCTCTGCACCCTGCTAGAAAATGCCATACAGGACAC

TGTGAGGGAACAAGACCAGAGTTTCACGGCCCTAGACTGGAGCTGGTTACAGACGGAAGAAGAAGAGCAC

AGCTGCCTGGAGCAGGCCTCATGATGTGGGGGGACTCGACCCCCTGACATGGGGCAGCCCATAGCAGGCC

TTGTGCAGTGGGGGGACTCGACCCCCTGACATGGGGCTGCCTGGAGCAGGCCGCGTGACGTGGGGCTGCC

TGGCCGCGGCTCTCACATGGTGGTTCCTGCTGCACTGATGGCCCAGGGGTCTCTGGTATCCAGATGGAGC

TCTCGCTTCCTCAGCAGCTGTGACTTTCACCCAGGACCCAGGACGCAGCCCTCCGTGGGCACTGCCGGCG

CCTTGTCTGCACACTGGAGGTCCTCCATTACAGAGGCCCAGCGCACATCGCTGGCCCCACAAACGTTCAG

GGGTACAGCCATGGCAGCTCCTTCCTCTGCCGTGAGAAAAGTGCTTGGAGTACGGTTTGCCACACACGTG

ACTGGACAGTGTCCAATTCAAATCTTTCAGGGCAGAGTCCGAGCAGCGCTTGGTGACAGCCTGTCCTCTC

CTGCTCTCCAAAGGCCCTGCTCCCTGTCCTCTCTCACTTTACAGCTTGTGTTTCTTCTGGATTCAGCTTC

TCCTAAACAGACAGTTTAATTATAGTTGCGGCCTGGCCCCATCCTCACTTCCTCTTTTTATTTCACTGCT

GCTAAAATTGTGTTTTTACCTACTACTTTGGTGGTTGTCCTCTTTTCGGCAAAGTTGGAGCGAGTGCCAA

GCTCTCCATCTGTGGTCCTTTCTGCCAAGAGCGACTCATAGTAACCAGGATGGGAGAGCAGCTGCCTTAT

TCTGAATCCCAAAAATTACTTGGGGGTGATTGTCACAGAGGAGGGACAGAAAGGGTATCTGCTGACCACC

AGCCTGCCTACCCATGCCCATGTCTCCATTCCTGCTCAAGCGTGTGTGCTGGGCCGGGGAGTCCCTGTCT

CTCACAGCATCTAGCAGTATTATTAAATGGATTCATTTTAAAAATAGCTCCTATATTTTGTAACATGTCT

CAAACACTCATACTGGGTTCCACAATCCACTGTTAGAATACCTATGGTTAGGGCTTCTGAACTAAAATAA

TGGAAAATTTTAACAATTTGTATAGTGCCTGGATCATTACTAGTGCCATAACCCTGCTTCTTCAACATTT

CACAGAACTTCTCTTTTATATAAAGGCAAGAGCACAAAATGAGTTCAGATGATCACAAACAGGTGAGTTT

TGTTGGAGAAGAAAGTTGGAGTAGGAGACTTTCACAAGTGGTTTCCATGGAGATAGAATGAAGCATTCTG

TGGTCAAGTAAGTTTAGGGAGCTATTCATGTTTCACTTGCTTTGTGGAGATTCACACTATGCACTGGGAA

AGTATCTGAAAAGTCTTATAATAAAGAAACAGGCTTAACTTTGTGTAAGAACACTGTTTATCAATGTCAT

TTGGCTATAGAAACATTTTCTCCTGCTGATTGTGTGTGTGAAACATGTATTAACATTCCAATGAACTAGC

ATTTAATAAAGCACAATTTTGGAAA

>NM_001193321.2 Homo sapiens inhibitor of nuclear factor kappa B kinase subunit epsilon (IKBKE), transcript variant 3, mRNA

AGTGCTGAGCTTGGTGTCCCACCGCCACAAGGAGGCAGGGAAGAAACCCACTAGTCCCAGCTCCTGGGGT

GGCACAGACATTGCAACTGGCCCTGCCTGTGGGTCCTAGGGGCCCTTGGCTACCAGGAGGCTAAGAACAC

TGCTCATGAATGACAGTGAGCCCTGAAAGCTCTGGGGGTGTCACCCAGTCCCACAAGCCTGCATCCCCTG

CAGTGGAGATGGGCTCAGCTCCTGGACGTGCCACAGACAGAAAGCATAACATACACTCGCCAGGAAGAGC

CTTTGCCTGACTCAGGGCAGCTCAGAGTGTGGGAAATCCGGAGAGCTGGTTGCTGTGAAGGTCTTCAACA

CTACCAGCTACCTGCGGCCCCGCGAGGTGCAGGTGAGGGAGTTTGAGGTCCTGCGGAAGCTGAACCACCA

GAACATTGTCAAGCTCTTTGCGGTGGAGGAGACGGGCGGAAGCCGGCAGAAGGTACTGGTGATGGAGTAC

TGCTCCAGTGGGAGCCTGCTGAGTGTGCTGGAGAGCCCTGAGAATGCCTTTGGGCTGCCTGAGGATGAGT

TCCTGGTGGTGCTGCGCTGTGTGGTGGCCGGCATGAACCACCTGCGGGAGAACGGCATTGTGCATCGCGA

CATCAAGCCGGGGAACATCATGCGCCTCGTAGGGGAGGAGGGGCAGAGCATCTACAAGCTGACAGACTTC

GGCGCTGCCCGGGAGCTGGATGATGATGAGAAGTTCGTCTCGGTCTATGGGACTGAGGAGTACCTGCATC

CCGACATGTATGAGCGGGCGGTGCTTCGAAAGCCCCAGCAAAAAGCGTTCGGGGTGACTGTGGATCTCTG

GAGCATTGGAGTGACCTTGTACCATGCAGCCACTGGCAGCCTGCCCTTCATCCCCTTTGGTGGGCCACGG

CGGAACAAGGAGATCATGTACCGGATCACCACGGAGAAGCCGGCTGGGGCCATTGCAGGTGCCCAGAGGC

GGGAGAACGGGCCCCTGGAGTGGAGCTACACCCTCCCCATCACCTGCCAGCTGTCACTGGGGCTGCAGAG

CCAGCTGGTGCCCATCCTGGCCAACATCCTGGAGGTGGAGCAGGCCAAGTGCTGGGGCTTCGACCAGTTC

TTTGCGGAGACCAGTGACATCCTGCAGCGAGTTGTCGTCCATGTCTTCTCCCTGTCCCAGGCAGTCCTGC

ACCACATCTATATCCATGCCCACAACACGATAGCCATTTTCCAGGAGGCCGTGCACAAGCAGACCAGTGT

GGCCCCCCGACACCAGGAGTACCTCTTTGAGGGTCACCTCTGTGTCCTCGAGCCCAGCGTCTCAGCACAG

CACATCGCCCACACGACGGCAAGCAGCCCCCTGACCCTCTTCAGCACAGCCATCCCTAAGGGGCTGGCCT

TCAGGGACCCTGCTCTGGACGTCCCCAAGTTCGTCCCCAAAGTGGACCTGCAGGCGGATTACAACACTGC

CAAGGGCGTGTTGGGCGCCGGCTACCAGGCCCTGCGGCTGGCACGGGCCCTGCTGGATGGGCAGGAGCTA

ATGTTTCGGGGGCTGCACTGGGTCATGGAGGTGCTCCAGGCCACATGCAGACGGACTCTGGAAGTGGCAA

GGACATCCCTCCTCTACCTCAGCAGCAGCCTGGGAACTGAGAGGTTCAGCAGCGTGGCTGGAACGCCTGA

GATCCAGGAACTGAAGGCGGCTGCAGAACTGAGGTCCAGGCTGCGGACTCTAGCGGAGGTCCTCTCCAGA

TGCTCCCAAAATATCACGGAGACCCAGGAGAGCCTGAGCAGCCTGAACCGGGAGCTGGTGAAGAGCCGGG

ATCAGGTACATGAGGACAGAAGCATCCAGCAGATTCAGTGCTGTTTGGACAAGATGAACTTCATCTACAA

ACAGTTCAAGAAGTCTAGGATGAGGCCAGGGCTTGGCTACAACGAGGAGCAGATTCACAAGCTGGATAAG

GTGAATTTCAGTCATTTAGCCAAAAGACTCCTGCAGGTGTTCCAGGAGGAGTGCGTGCAGAAGTATCAAG

CGTCCTTAGTCACACACGGCAAGAGGATGAGGGTGGTGCACGAGACCAGGAACCACCTGCGCCTGGTTGG

CTGTTCTGTGGCTGCCTGTAACACAGAAGCCCAGGGGGTCCAGGAGAGTCTCAGCAAGCTCCTGGAAGAG

CTATCTCACCAGCTCCTTCAGGACCGAGCAAAGGGGGCTCAGGCCTCGCCGCCTCCCATAGCTCCTTACC

CCAGCCCTACACGAAAGGACCTGCTTCTCCACATGCAAGAGCTCTGCGAGGGGATGAAGCTGCTGGCATC

TGACCTCCTGGACAACAACCGCATCATCGAACGGCTAAATAGAGTCCCAGCACCTCCTGATGTCTGAGCT

CCATGGGGCACATGAGGCATCCTGAAGCATTAGAATGATTCCAACACTGCTCTTCTGCACCATGAGACCA

ACCCAGGGCAAGATCCCATCCCATCACATCAGCCTACCTCCCTCCTGGCTGCTGGCCAGGATGTCGCCAG

CATTACCTTCCACTGCCTTTCTCCCTGGGAAGCAGCACAGCTGAGACTGGGCACCAGGCCACCTCTGTTG

GGACCCACAGGAAAGAGTGTGGCAGCAACTGCCTGGCTGACCTTTCTATCTTCTCTAGGCTCAGGTACTG

CTCCTCCATGCCCATGGCTGGGCCGTGGGGAGAAGAAGCTCTCATACGCCTTCCCACTCCCTCTGGTTTA

TAGGACTTCACTCCCTAGCCAACAGGAGAGGAGGCCTCCTGGGGTTTCCCCAGGGCAGTAGGTCAAACGA

CCTCATCACAGTCTTCCTTCCTCTTCAAGCGTTTCATGTTGAACACAGCTCTCTCCGCTCCCTTGTGATT

TCTGAGGGTCACCACTGCCAGCCTCAGGCAACATAGAGAGCCTCCTGTTCTTTCTATGCTTGGTCTGACT

GAGCCTAAAGTTGAGAAAATGGGTGGCCAAGGCCAGTGCCAGTGTCTTGGGGCCCCTTTGGCTCTCCCTC

ACTCTCTGAGGCTCCAGCTGGTCCTGGGACATGCAGCCAGGACTGTGAGTCTGGGCAGGTCCAAGGCCTG

CACCTTCAAGAAGTGGAATAAATGTGGCCTTTGCTTCTGTT

>NM_006850.3 Homo sapiens interleukin 24 (IL24), transcript variant 1, mRNA

GCTTGCCTGCAAACCTTTACTTCTGAAATGACTTCCACGGCTGGGACGGGAACCTTCCACCCACAGCTAT

GCCTCTGATTGGTGAATGGTGAAGGTGCCTGTCTAACTTTTCTGTAAAAAGAACCAGCTGCCTCCAGGCA

GCCAGCCCTCAAGCATCACTTACAGGACCAGAGGGACAAGACATGACTGTGATGAGGAGCTGCTTTCGCC

AATTTAACACCAAGAAGAATTGAGGCTGCTTGGGAGGAAGGCCAGGAGGAACACGAGACTGAGAGATGAA

TTTTCAACAGAGGCTGCAAAGCCTGTGGACTTTAGCCAGACCCTTCTGCCCTCCTTTGCTGGCGACAGCC

TCTCAAATGCAGATGGTTGTGCTCCCTTGCCTGGGTTTTACCCTGCTTCTCTGGAGCCAGGTATCAGGGG

CCCAGGGCCAAGAATTCCACTTTGGGCCCTGCCAAGTGAAGGGGGTTGTTCCCCAGAAACTGTGGGAAGC

CTTCTGGGCTGTGAAAGACACTATGCAAGCTCAGGATAACATCACGAGTGCCCGGCTGCTGCAGCAGGAG

GTTCTGCAGAACGTCTCGGATGCTGAGAGCTGTTACCTTGTCCACACCCTGCTGGAGTTCTACTTGAAAA

CTGTTTTCAAAAACTACCACAATAGAACAGTTGAAGTCAGGACTCTGAAGTCATTCTCTACTCTGGCCAA

CAACTTTGTTCTCATCGTGTCACAACTGCAACCCAGTCAAGAAAATGAGATGTTTTCCATCAGAGACAGT

GCACACAGGCGGTTTCTGCTATTCCGGAGAGCATTCAAACAGTTGGACGTAGAAGCAGCTCTGACCAAAG

CCCTTGGGGAAGTGGACATTCTTCTGACCTGGATGCAGAAATTCTACAAGCTCTGAATGTCTAGACCAGG

ACCTCCCTCCCCCTGGCACTGGTTTGTTCCCTGTGTCATTTCAAACAGTCTCCCTTCCTATGCTGTTCAC

TGGACACTTCACGCCCTTGGCCATGGGTCCCATTCTTGGCCCAGGATTATTGTCAAAGAAGTCATTCTTT

AAGCAGCGCCAGTGACAGTCAGGGAAGGTGCCTCTGGATGCTGTGAAGAGTCTACAGAGAAGATTCTTGT

ATTTATTACAACTCTATTTAATTAATGTCAGTATTTCAACTGAAGTTCTATTTATTTGTGAGACTGTAAG

TTACATGAAGGCAGCAGAATATTGTGCCCCATGCTTCTTTACCCCTCACAATCCTTGCCACAGTGTGGGG

CAGTGGATGGGTGCTTAGTAAGTACTTAATAAACTGTGGTGCTTTTTTTGGCCTGTCTTTGGATTGTTAA

AAAACAGAGAGGGATGCTTGGATGTAAAACTGAACTTCAGAGCATGAAAATCACACTGTCTTCTGATATC

TGCAGGGACAGAGCATTGGGGTGGGGGTAAGGTGCATCTGTTTGAAAAGTAAACGATAAAATGTGGATTA

AAGTGCCCAGCACAAAGCAGATCCTCAATAAACATTTCATTTCCCACCCACACTCGCCAGCTCACCCCAT

CATCCCTTTCCCTTGGTGCCCTCCTTTTTTTTTTATCCTAGTCATTCTTCCCTAATCTTCCACTTGAGTG

TCAAGCTGACCTTGCTGATGGTGACATTGCACCTGGATGTACTATCCAATCTGTGATGACATTCCCTGCT

AATAAAAGACAACATAACTCAAGTCTGGCAGACTTTCTTCTCTATTTCTGGATGAATGCCCAGTGAGACT

GTGTTGTACAGCTAGAAAAGGCCTTCTTCCCAATAGCAAGGCTGTGCATCTAGCCTCAAGCTCTGGCTGA

ACTTTGTGGTCGACATCAATCTAAAGATACAGTGTCTGACTATAACCTTGTTCCAAAAACCTAGGCAAAG

AGTATATGTAGGAGGTGGGATATCACTTCCATGACATAAGTGCTATTGCAGAGCCGTGGCCACCCAGGAA

CTCCTGACTGCTTTCC

>NM_001145805.2 Homo sapiens immunity related GTPase M (IRGM), transcript variant 2, mRNA

GGTAACCTGCTCAGGTCCCCTTCCATGCTGTGGAAGCTTTGTTTTTTTGCACTTTGCAGTAAATCTTGCT

GCTGCTCATTCTTTGGGTCCACACTGCCTTTATGAGCTGTAACACTCACCGTGAAGGTCTGCAGCATCAC

TCCTGAAACCTGTGAAACAACGAACCCCCCGGGGAGAAACGAACAACTCCAGACTCGCCGCCTGAAGAGC

TGTAACTCTCACTTCGAAGGTCTGCAGCTTCGCTCCTGAGTCAGTGAAACCACGAACCCACCGGAAGGAA

GAAACTCTGAACACATCCAAACATCAGAAGGAACAAACTCCGGACACGCAGCCTTTAAGAATTGTAACAC

TCACCGCGAGGGTCCGTGGCTTCATTCTTGAAGTCAGTGAGACGAAGAACCCACCAATTCCGGTAGGAGT

AGGAAATCACATCACCTTCTTTTAATCAGTCTCAAATACCTGGCCCCTTGTGGAACCTGCAAGAGCCAGG

TTTGGCGAATGCAATGATAAGCAATTGTCTAATCACTGCCTTGAAAAAGAGCAGAGCATTTGAGTATGCT

GTCTCCTCCTCTCCCTCACTTCAGTTGGGCCCAACATGACAGTCTTAATGGAAGCTCGGGGGTCAAAGGC

TGGTGGCTTACTTCACGTATATTGCAGCATTTCAGTACTGCTCTGCACGTGTGCTCCCCCGCCTGATGAG

CTTACTCCAGTGCCCACAGATACGACAGAGTGTCCCAAGTGCCCCTCACACTCTATTAGCTGCATCCTTA

ACCTCTTTTTGCCACACCATAAGCATTGGGTTTTGTTTGTTTTGAGATGGAGTCTTGCTCTGTTGCCAGG

CTGGAGTGCAATGGCGTGATCTCAGCTCACTGCAATATCTGCGTCCAGGGTTCAAGCGATTCCCCTGCCT

CAGCCTCCTGTATTAGCTGGGACTACAGGCGCACACCACCACGCGCAGCTAATTTTTTTGTATTTTAGTA

GAGAAGGTTTCACGATGTTGGCCAGGATGGTCTCAATCTCCTGACCTCGTGATCTGCTCGCCTCGGCCTT

CCAAAGTGCTGGGATTACAGGCATGAGCCACGGCGCCTGGCCAGCATTGGGGTATTTTATTGAAGATGGA

AGCCATGAATGTTGAGAAAGCCTCAGCAGATGGGAACTTGCCAGAGGTGATCTCTAACATCAAGGAGACT

CTGAAGATAGTGTCCAGGACACCAGTTAACATCACTATGGCAGGGGACTCTGGCAATGGGATGTCCACCT

TCATCAGTGCCCTTCGAAACACAGGACATGAGGGTAAGGCCTCACCTCCTACTGAGCTGGTAAAAGCTAC

CCAAAGATGTGCCTCCTATTTCTCTTCCCACTTTTCAAATGTGGTGTTGTGGGACCTGCCTGGCACAGGG

TCTGCCACCACAACCCTGGAGAACTACCTGATGGAAATGCAGTTCAACCGGTATGACTTCATCATGGTTG

CATCTGCACAATTCAGCATGAATCATGTGATGCTTGCCAAAACCGCTGAGGACATGGGAAAGAAGTTCTA

CATTGTCTGGACCAAGCTAGACATGGACCTCAGCACAGGTGCCCTCCCAGAAGTGCAGCTACTGCAGATC

AGAGAAAATGTCCTGGAAAATCTCCAGAAGGAGCGGGTATGTGAATACTAATTCCTGTCTTCATTAAACA

TTTTCCATCTCCTC

>NM_005501.2 Homo sapiens integrin, alpha 3 (antigen CD49C, alpha 3 subunit of VLA-3 receptor) (ITGA3), transcript variant b, mRNA

TTTCCCCGGAAGGAAAGCGCAGCCCGGGCTGGGCTCGCAAGGTGGGGAGGTGCGGGACTGGGCGTGGGGA

GGCGGGGCGCGCGCCGGGGGACCCCTCCCTCCTGTCCTCCTTGCGGTCGACCGGTGCGCTTGCCAGATCC

GCCGCGAAGCCGGGATCGAAGGCGACAGCGCGGCCAAGGGGGCGCGGCCGGGACAAGCTGGGGGCCGGTT

GCCCGGGGCAGGGACGGCGGCGACCCGGCCGCTGGGGAGGCAGGAAGATAGACCCACGGATCTTAGGAAG

GGATCCGAGAGCGCAGCTGTGAAACTGGCTGGGGCTGGGGGCACGAAACCGATCAGCGCTACGGAGCGCA

GCGGCCGGCGGGTTCCAGTGTCCTCCGGCGGCGCGGGGAGCAGGTGAACAGGTCCTCACGCCCAGCTCCG

CGCCCTCACGCGCTCTCGCCGGGACCCCGCTTCCGCTGGCAGCCATGGGCCCCGGCCCCAGCCGCGCGCC

CCGCGCCCCACGCCTGATGCTCTGTGCGCTCGCCTTGATGGTGGCGGCCGGCGGCTGCGTCGTCTCCGCC

TTCAACCTGGATACCCGATTCCTGGTAGTGAAGGAGGCCGGGAACCCGGGCAGCCTCTTCGGCTACTCGG

TCGCCCTCCATCGGCAGACAGAGCGGCAGCAGCGCTACCTGCTCCTGGCTGGTGCCCCCCGGGAGCTCGC

TGTGCCCGATGGCTACACCAACCGGACTGGTGCTGTGTACCTGTGCCCACTCACTGCCCACAAGGATGAC

TGTGAGCGGATGAACATCACAGTGAAAAATGACCCTGGCCATCACATTATTGAGGACATGTGGCTTGGAG

TGACTGTGGCCAGCCAGGGCCCTGCAGGCAGAGTTCTGGTCTGTGCCCACCGCTACACCCAGGTGCTGTG

GTCAGGGTCAGAAGACCAGCGGCGCATGGTGGGCAAGTGCTACGTGCGAGGCAATGACCTAGAGCTGGAC

TCCAGTGATGACTGGCAGACCTACCACAACGAGATGTGCAATAGCAACACAGACTACCTGGAGACGGGCA

TGTGCCAGCTGGGCACCAGCGGTGGCTTCACCCAGAACACTGTGTACTTCGGCGCCCCCGGTGCCTACAA

CTGGAAAGGAAACAGCTACATGATTCAGCGCAAGGAGTGGGACTTATCTGAGTATAGTTACAAGGACCCA

GAGGACCAAGGAAACCTCTATATTGGGTACACGATGCAGGTAGGCAGCTTCATCCTGCACCCCAAAAACA

TCACCATTGTGACAGGTGCCCCACGGCACCGACATATGGGCGCGGTGTTCTTGCTGAGCCAGGAGGCAGG

CGGAGACCTGCGGAGGAGGCAGGTGCTGGAGGGCTCGCAGGTGGGCGCCTATTTTGGCAGCGCCATTGCC

CTGGCAGACCTGAACAATGATGGGTGGCAGGACCTCCTGGTGGGCGCCCCCTACTACTTCGAGAGGAAAG

AGGAAGTAGGGGGTGCCATCTATGTCTTCATGAACCAGGCGGGAACCTCCTTCCCTGCTCACCCCTCACT

CCTTCTTCATGGCCCCAGTGGCTCTGCCTTTGGTTTATCTGTGGCCAGCATTGGTGACATCAACCAGGAT

GGATTTCAGGATATTGCTGTGGGAGCTCCGTTTGAAGGCTTGGGCAAAGTGTACATCTATCACAGTAGCT

CTAAGGGGCTCCTTAGACAGCCCCAGCAGGTAATCCATGGAGAGAAGCTGGGACTGCCTGGGTTGGCCAC

CTTCGGCTATTCCCTCAGTGGGCAGATGGATGTGGATGAGAACTTCTACCCAGACCTTCTAGTGGGAAGC

CTGTCAGACCACATTGTGCTGCTGCGGGCCCGGCCCGTCATCAACATCGTCCACAAGACCTTGGTGCCCA

GGCCAGCTGTGCTGGACCCTGCACTTTGCACGGCCACCTCTTGTGTGCAAGTGGAGCTGTGCTTTGCTTA

CAACCAGAGTGCCGGGAACCCCAACTACAGGCGAAACATCACCCTGGCCTACACTCTGGAGGCTGACAGG

GACCGCCGGCCGCCCCGGCTCCGCTTTGCCGGCAGTGAGTCCGCTGTCTTCCACGGCTTCTTCTCCATGC

CCGAGATGCGCTGCCAGAAGCTGGAGCTGCTCCTGATGGACAACCTCCGTGACAAACTCCGCCCCATCAT

CATCTCCATGAACTACTCTTTACCTTTGCGGATGCCCGATCGCCCCCGGCTGGGGCTGCGGTCCCTGGAC

GCCTACCCGATCCTCAACCAGGCACAGGCTCTGGAGAACCACACTGAGGTCCAGTTCCAGAAGGAGTGCG

GGCCTGACAACAAGTGTGAGAGCAACTTGCAGATGCGGGCAGCCTTCGTGTCAGAGCAGCAGCAGAAGCT

GAGCAGGCTCCAGTACAGCAGAGACGTCCGGAAATTGCTCCTGAGCATCAACGTGACGAACACCCGGACC

TCGGAGCGCTCCGGGGAGGACGCCCACGAGGCGCTGCTCACCCTGGTGGTGCCTCCCGCCCTGCTGCTGT

CCTCAGTGCGCCCCCCCGGGGCCTGCCAAGCTAATGAGACCATCTTTTGCGAGCTGGGGAACCCCTTCAA

ACGGAACCAGAGGATGGAGCTGCTCATCGCCTTTGAGGTCATCGGGGTGACCCTGCACACAAGGGACCTT

CAGGTGCAGCTGCAGCTCTCCACGTCGAGTCACCAGGACAACCTGTGGCCCATGATCCTCACTCTGCTGG

TGGACTATACACTCCAGACCTCGCTTAGCATGGTAAATCACCGGCTACAAAGCTTCTTTGGGGGGACAGT

GATGGGTGAGTCTGGCATGAAAACTGTGGAGGATGTAGGAAGCCCCCTCAAGTATGAATTCCAGGTGGGC

CCAATGGGGGAGGGGCTGGTGGGCCTGGGGACCCTGGTCCTAGGTCTGGAGTGGCCCTACGAAGTCAGCA

ATGGCAAGTGGCTGCTGTATCCCACGGAGATCACCGTCCATGGCAATGGGTCCTGGCCCTGCCGACCACC

TGGAGACCTTATCAACCCTCTCAACCTCACTCTTTCTGACCCTGGGGACAGGCCATCATCCCCACAGCGC

AGGCGGCGACAGCTGGATCCAGGGGGAGGCCAGGGCCCCCCACCTGTCACTCTGGCTGCTGCCAAAAAAG

CCAAGTCTGAGACTGTGCTGACCTGTGCCACAGGGCGTGCCCACTGTGTGTGGCTAGAGTGCCCCATCCC

TGATGCCCCCGTTGTCACCAACGTGACTGTGAAGGCACGAGTGTGGAACAGCACCTTCATCGAGGATTAC

AGAGACTTTGACCGAGTCCGGGTAAATGGCTGGGCTACCCTATTCCTCCGAACCAGCATCCCCACCATCA

ACATGGAGAACAAGACCACGTGGTTCTCTGTGGACATTGACTCGGAGCTGGTGGAGGAGCTGCCGGCCGA

AATCGAGCTGTGGCTGGTGCTGGTGGCCGTGGGTGCAGGGCTGCTGCTGCTGGGGCTGATCATCCTCCTG

CTGTGGAAGTGTGACTTCTTTAAGCGGACCCGCTATTATCAGATCATGCCCAAGTACCACGCAGTGCGGA

TCCGGGAGGAGGAGCGCTACCCACCTCCAGGGAGCACCCTGCCCACCAAGAAGCACTGGGTGACCAGCTG

GCAGACTCGGGACCAATACTACTGACGTCCTCCCTGATCCCACCCCCTCCTCCCCCAGTGTCCCCTTTCT

TCCTATTTATCATAAGTTATGCCTCTGACAGTCCACAGGGGCCACCACCTTTGGCTGGTAGCAGCAGGCT

CAGGCACATACACCTCGTCAAGAGCATGCACATGCTGTCTGGCCCTGGGGATCTTCCCACAGGAGGGCCA

GCGCTGTGGACCTTACAACGCCGAGTGCACTGCATTCCTGTGCCCTAGATGCACGTGGGGCCCACTGCTC

GTGGACTGTGCTGGTGCATCACGGATGGTGCATGGGCTCGCCGTGTCTCAGCCTCTGCCAGCGCCAAAAC

AAGCCAAAGAGCCTCCCACCAGAGCCGGGAGGAAAAGGCCCCTGCAATGTGGTGACACCTCCCCCTTTCA

CACTGGATCCATCTTGAGCCACAGTCACTGGATTGACTTTGCTGTCAAAACTACTGACAGGGAGCAGCCC

CCGGGCCGCTGGCTGGTGGGCCCCCAATGACACCCATGCCAGAGAGGTGGGGATCCTGCCTAAGGTTGTC

TACGGGGGCACTTGGAGGACCTGGCGTGCTCAGACCCAACAGCAAAGGAACTAGAAAGAAGGACCCAGAA

CGGCTTGCTTTCCTGCATCTCTGTGAAGCCTCTCTCCTTGGCCACAGACTGAACTCGCAGGGAATGCAGC

AGGAAGGAACAAAGACAGGCAAACGGCAACGTAGCCTGGGCTCACTGTGCTGGGGCACGGCGGGATCCTC

CACAGAGAGGAGGGGACCAATTCTGGACAGACAGATGTTGGGAGGATACAGAGGAGATGCCACTTCTCAC

TCACCACTACCAGCCAGCCTCAGAAGGCCCCAGAGAGACCCTGCAAGACCACGGAGGGAGCGACACTTGA

ATGTAGAATAGGCAGGGGGCCCTGCCCCACCCCATCCAGCCAGACCCCACGCTGACCATGCGTCAGGGGC

CTAGAGGTGGAGTTCTTAGCTATCCTTGGCTTTCAGAGCCAGCCTGGCTCTGCCCCCTCCCCCATGGGCT

GTGTCCTAAGGCCCATTTGAGAAGCTGAGGCTAGTTCCAGAAAACCTCTCCTGACCCCTGCCTGTTGGCA

GGCCCACTCCCCAGCCCCAGCCCCTTCCATGGTACTGTAGCAGGGGAATTCCCTCCCCCTCCTTGTGCCT

TCTTTGTATATAGGCTTCTCACGGCGACCAATAAACAGCTCCCAGTTTGTATGCAAAAAAAAAAAAAAAA

AA

>NM_000210.4 Homo sapiens integrin subunit alpha 6 (ITGA6), transcript variant 2, mRNA

AACGGGCTCATTCAGCGGTCGCGAGCTGCCCGCGAGGGGGAGCGGCCGGACGGAGAGCGCGACCCGTCCC

GGGGGTGGGGCCGGGCGCAGCGGCGAGAGGAGGCGAAGGTGGCTGCGGTAGCAGCAGCGCGGCAGCCTCG

GACCCAGCCCGGAGCGCAGGGCGGCCGCTGCAGGTCCCCGCTCCCCTCCCCGTGCGTCCGCCCATGGCCG

CCGCCGGGCAGCTGTGCTTGCTCTACCTGTCGGCGGGGCTCCTGTCCCGGCTCGGCGCAGCCTTCAACTT

GGACACTCGGGAGGACAACGTGATCCGGAAATATGGAGACCCCGGGAGCCTCTTCGGCTTCTCGCTGGCC

ATGCACTGGCAACTGCAGCCCGAGGACAAGCGGCTGTTGCTCGTGGGGGCCCCGCGGGCAGAAGCGCTTC

CACTGCAGAGAGCCAACAGAACGGGAGGGCTGTACAGCTGCGACATCACCGCCCGGGGGCCATGCACGCG

GATCGAGTTTGATAACGATGCTGACCCCACGTCAGAAAGCAAGGAAGATCAGTGGATGGGGGTCACCGTC

CAGAGCCAAGGTCCAGGGGGCAAGGTCGTGACATGTGCTCACCGATATGAAAAAAGGCAGCATGTTAATA

CGAAGCAGGAATCCCGAGACATCTTTGGGCGGTGTTATGTCCTGAGTCAGAATCTCAGGATTGAAGACGA

TATGGATGGGGGAGATTGGAGCTTTTGTGATGGGCGATTGAGAGGCCATGAGAAATTTGGCTCTTGCCAG

CAAGGTGTAGCAGCTACTTTTACTAAAGACTTTCATTACATTGTATTTGGAGCCCCGGGTACTTATAACT

GGAAAGGGATTGTTCGTGTAGAGCAAAAGAATAACACTTTTTTTGACATGAACATCTTTGAAGATGGGCC

TTATGAAGTTGGTGGAGAGACTGAGCATGATGAAAGTCTCGTTCCTGTTCCTGCTAACAGTTACTTAGGT

TTTTCTTTGGACTCAGGGAAAGGTATTGTTTCTAAAGATGAGATCACTTTTGTATCTGGTGCTCCCAGAG

CCAATCACAGTGGAGCCGTGGTTTTGCTGAAGAGAGACATGAAGTCTGCACATCTCCTCCCTGAGCACAT

ATTCGATGGAGAAGGTCTGGCCTCTTCATTTGGCTATGATGTGGCGGTGGTGGACCTCAACAAGGATGGG

TGGCAAGATATAGTTATTGGAGCCCCACAGTATTTTGATAGAGATGGAGAAGTTGGAGGTGCAGTGTATG

TCTACATGAACCAGCAAGGCAGATGGAATAATGTGAAGCCAATTCGTCTTAATGGAACCAAAGATTCTAT

GTTTGGCATTGCAGTAAAAAATATTGGAGATATTAATCAAGATGGCTACCCAGATATTGCAGTTGGAGCT

CCGTATGATGACTTGGGAAAGGTTTTTATCTATCATGGATCTGCAAATGGAATAAATACCAAACCAACAC

AGGTTCTCAAGGGTATATCACCTTATTTTGGATATTCAATTGCTGGAAACATGGACCTTGATCGAAATTC

CTACCCTGATGTTGCTGTTGGTTCCCTCTCAGATTCAGTAACTATTTTCAGATCCCGGCCTGTGATTAAT

ATTCAGAAAACCATCACAGTAACTCCTAACAGAATTGACCTCCGCCAGAAAACAGCGTGTGGGGCGCCTA

GTGGGATATGCCTCCAGGTTAAATCCTGTTTTGAATATACTGCTAACCCCGCTGGTTATAATCCTTCAAT

ATCAATTGTGGGCACACTTGAAGCTGAAAAAGAAAGAAGAAAATCTGGGCTATCCTCAAGAGTTCAGTTT

CGAAACCAAGGTTCTGAGCCCAAATATACTCAAGAACTAACTCTGAAGAGGCAGAAACAGAAAGTGTGCA

TGGAGGAAACCCTGTGGCTACAGGATAATATCAGAGATAAACTGCGTCCCATTCCCATAACTGCCTCAGT

GGAGATCCAAGAGCCAAGCTCTCGTAGGCGAGTGAATTCACTTCCAGAAGTTCTTCCAATTCTGAATTCA

GATGAACCCAAGACAGCTCATATTGATGTTCACTTCTTAAAAGAGGGATGTGGAGACGACAATGTATGTA

ACAGCAACCTTAAACTAGAATATAAATTTTGCACCCGAGAAGGAAATCAAGACAAATTTTCTTATTTACC

AATTCAAAAAGGTGTACCAGAACTAGTTCTAAAAGATCAGAAGGATATTGCTTTAGAAATAACAGTGACA

AACAGCCCTTCCAACCCAAGGAATCCCACAAAAGATGGCGATGACGCCCATGAGGCTAAACTGATTGCAA

CGTTTCCAGACACTTTAACCTATTCTGCATATAGAGAACTGAGGGCTTTCCCTGAGAAACAGTTGAGTTG

TGTTGCCAACCAGAATGGCTCGCAAGCTGACTGTGAGCTCGGAAATCCTTTTAAAAGAAATTCAAATGTC

ACTTTTTATTTGGTTTTAAGTACAACTGAAGTCACCTTTGACACCCCAGATCTGGATATTAATCTGAAGT

TAGAAACAACAAGCAATCAAGATAATTTGGCTCCAATTACAGCTAAAGCAAAAGTGGTTATTGAACTGCT

TTTATCGGTCTCGGGAGTTGCTAAACCTTCCCAGGTGTATTTTGGAGGTACAGTTGTTGGCGAGCAAGCT

ATGAAATCTGAAGATGAAGTGGGAAGTTTAATAGAGTATGAATTCAGGGTAATAAACTTAGGTAAACCTC

TTACAAACCTCGGCACAGCAACCTTGAACATTCAGTGGCCAAAAGAAATTAGCAATGGGAAATGGTTGCT

TTATTTGGTGAAAGTAGAATCCAAAGGATTGGAAAAGGTAACTTGTGAGCCACAAAAGGAGATAAACTCC

CTGAACCTAACGGAGTCTCACAACTCAAGAAAGAAACGGGAAATTACTGAAAAACAGATAGATGATAACA

GAAAATTTTCTTTATTTGCTGAAAGAAAATACCAGACTCTTAACTGTAGCGTGAACGTGAACTGTGTGAA

CATCAGATGCCCGCTGCGGGGGCTGGACAGCAAGGCGTCTCTTATTTTGCGCTCGAGGTTATGGAACAGC

ACATTTCTAGAGGAATATTCCAAACTGAACTACTTGGACATTCTCATGCGAGCCTTCATTGATGTGACTG

CTGCTGCCGAAAATATCAGGCTGCCAAATGCAGGCACTCAGGTTCGAGTGACTGTGTTTCCCTCAAAGAC

TGTAGCTCAGTATTCGGGAGTACCTTGGTGGATCATCCTAGTGGCTATTCTCGCTGGGATCTTGATGCTT

GCTTTATTAGTGTTTATACTATGGAAGTGTGGTTTCTTCAAGAGAAATAAGAAAGATCATTATGATGCCA

CATATCACAAGGCTGAGATCCATGCTCAGCCATCTGATAAAGAGAGGCTTACTTCTGATGCATAGTATTG

ATCTACTTCTGTAATTGTGTGGATTCTTTAAACGCTCTAGGTACGATGACAGTGTTCCCCGATACCATGC

TGTAAGGATCCGGAAAGAAGAGCGAGAGATCAAAGATGAAAAGTATATTGATAACCTTGAAAAAAAACAG

TGGATCACAAAGTGGAACGAAAATGAAAGCTACTCATAGCGGGGGCCTAAAAAAAAAAAGCTTCACAGTA

CCCAAACTGCTTTTTCCAACTCAGAAATTCAATTTGGATTTAAAAGCCTGCTCAATCCCTGAGGACTGAT

TTCAGAGTGACTACACACAGTACGAACCTACAGTTTTAACTGTGGATATTGTTACGTAGCCTAAGGCTCC

TGTTTTGCACAGCCAAATTTAAAACTGTTGGAATGGATTTTTCTTTAACTGCCGTAATTTAACTTTCTGG

GTTGCCTTTATTTTTGGCGTGGCTGACTTACATCATGTGTTGGGGAAGGGCCTGCCCAGTTGCACTCAGG

TGACATCCTCCAGATAGTGTAGCTGAGGAGGCACCTACACTCACCTGCACTAACAGAGTGGCCGTCCTAA

CCTCGGGCCTGCTGCGCAGACGTCCATCACGTTAGCTGTCCCACATCACAAGACTATGCCATTGGGGTAG

TTGTGTTTCAACGGAAAGTGCTGTCTTAAACTAAATGTGCAATAGAAGGTGATGTTGCCATCCTACCGTC

TTTTCCTGTTTCCTAGCTGTGTGAATACCTGCTCACGTCAAATGCATACAAGTTTCATTCTCCCTTTCAC

TAAAACACACAGGTGCAACAGACTTGAATGCTAGTTATACTTATTTGTATATGGTATTTATTTTTTCTTT

TCTTTACAAACCATTTTGTTATTGACTAACAGGCCAAAGAGTCTCCAGTTTACCCTTCAGGTTGGTTTAA

TCAATCAGAATTAGAGCATGGGAGGTCATCACTTTGACCTAAATTATTTACTGCAAAAAGAAAATCTTTA

TAAATGTACCAGAGAGAGTTGTTTTAATAACTTATCTATAAACTATAACCTCTCCTTCATGACAGCCTCC

ACCCCACAACCCAAAAGGTTTAAGAAATAGAATTATAACTGTAAAGATGTTTATTTCAGGCATTGGATAT

TTTTTACTTTAGAAGCCTGCATAATGTTTCTGGATTTCATACTGTAACATTCAGGAATTCTTGGAGAAAA

TGGGTTTATTCACTGAACTCTAGTGCGGTTTACTCACTGCTGCAAATACTGTATATTCAGGACTTGAAAG

AAATGGTGAATGCCTATGGTGGATCCAAACTGATCCAGTATAAGACTACTGAATCTGCTACCAAAACAGT

TAATCAGTGAGTCGATGTTCTATTTTTTGTTTTGTTTCCTCCCCTATCTGTATTCCCAAAAATTACTTTG

GGGCTAATTTAACAAGAACTTTAAATTGTGTTTTAATTGTAAAAATGGCAGGGGGTGGAATTATTACTCT

ATACATTCAACAGAGACTGAATAGATATGAAAGCTGATTTTTTTTAATTACCATGCTTCACAATGTTAAG

TTATATGGGGAGCAACAGCAAACAGGTGCTAATTTGTTTTGGATATAGTATAAGCAGTGTCTGTGTTTTG

AAAGAATAGAACACAGTTTGTAGTGCCACTGTTGTTTTGGGGGGGCTTTTTTCTTTTCGGAAATCTTAAA

CCTTAAGATACTAAGGACGTTGTTTTGGTTGTACTTTGGAATTCTTAGTCACAAAATATATTTTGTTTAC

AAAAATTTCTGTAAAACAGGTTATAACAGTGTTTAAAGTCTCAGTTTCTTGCTTGGGGAACTTGTGTCCC

TAATGTGTTTAGATTGCTAGATTGCTAAGGAGCTGATACTTTGACAGTGTTTTTAGACCTGTGTTACTAA

AAAAAAGATGAATGTCCTGAAAAGGGTGTTGGGAGGGTGGTTCAACAAAGAAACAAAGATGTTATGGTGT

TTAGATTTATGGTTGTTAAAAATGTCATCTCAAGTCAAGTCACTGGTCTGTTTGCATTTGATACATTTTT

GTACTAACTAGCATTGTAAAATTATTTCATGATTAGAAATTACCTGTGGATATTTGTATAAAAGTGTGAA

ATAAATTTTTTATAAAAGTGTTCATTGTTTCGTAACACAGCATTGTATATGTGAAGCAAACTCTAAAATT

ATAAATGACAACCTGAATTATCTATTTCATCAAACCAAAGTTCAGTGTTTTTATTTTTGGTGTCTCATGT

AATCTCAGATCAGCCAAAGATACTAGTGCCAAAGCAATGGGATTCGGGGTTTTTTTCTGTTTTCGCTCTA

TGTAGGTGATCCTCAAGTCTTTCATTTTCCTTCTTTATGATTAAAAGAAACCTACAGGTATTTAACAACC

TACAAA

>NM_002211.4 Homo sapiens integrin subunit beta 1 (ITGB1), transcript variant 1A, mRNA

GCCAGCCCGCGGGAGAGGCCCAGCGGGAGTCGCGGAACAGCAGGCCCGAGCCCACCGCGCCGGGCCCCGG

ACGCCGCGCGGAAAAGATGAATTTACAACCAATTTTCTGGATTGGACTGATCAGTTCAGTTTGCTGTGTG

TTTGCTCAAACAGATGAAAATAGATGTTTAAAAGCAAATGCCAAATCATGTGGAGAATGTATACAAGCAG

GGCCAAATTGTGGGTGGTGCACAAATTCAACATTTTTACAGGAAGGAATGCCTACTTCTGCACGATGTGA

TGATTTAGAAGCCTTAAAAAAGAAGGGTTGCCCTCCAGATGACATAGAAAATCCCAGAGGCTCCAAAGAT

ATAAAGAAAAATAAAAATGTAACCAACCGTAGCAAAGGAACAGCAGAGAAGCTCAAGCCAGAGGATATTA

CTCAGATCCAACCACAGCAGTTGGTTTTGCGATTAAGATCAGGGGAGCCACAGACATTTACATTAAAATT

CAAGAGAGCTGAAGACTATCCCATTGACCTCTACTACCTTATGGACCTGTCTTACTCAATGAAAGACGAT

TTGGAGAATGTAAAAAGTCTTGGAACAGATCTGATGAATGAAATGAGGAGGATTACTTCGGACTTCAGAA

TTGGATTTGGCTCATTTGTGGAAAAGACTGTGATGCCTTACATTAGCACAACACCAGCTAAGCTCAGGAA

CCCTTGCACAAGTGAACAGAACTGCACCAGCCCATTTAGCTACAAAAATGTGCTCAGTCTTACTAATAAA

GGAGAAGTATTTAATGAACTTGTTGGAAAACAGCGCATATCTGGAAATTTGGATTCTCCAGAAGGTGGTT

TCGATGCCATCATGCAAGTTGCAGTTTGTGGATCACTGATTGGCTGGAGGAATGTTACACGGCTGCTGGT

GTTTTCCACAGATGCCGGGTTTCACTTTGCTGGAGATGGGAAACTTGGTGGCATTGTTTTACCAAATGAT

GGACAATGTCACCTGGAAAATAATATGTACACAATGAGCCATTATTATGATTATCCTTCTATTGCTCACC

TTGTCCAGAAACTGAGTGAAAATAATATTCAGACAATTTTTGCAGTTACTGAAGAATTTCAGCCTGTTTA

CAAGGAGCTGAAAAACTTGATCCCTAAGTCAGCAGTAGGAACATTATCTGCAAATTCTAGCAATGTAATT

CAGTTGATCATTGATGCATACAATTCCCTTTCCTCAGAAGTCATTTTGGAAAACGGCAAATTGTCAGAAG

GCGTAACAATAAGTTACAAATCTTACTGCAAGAACGGGGTGAATGGAACAGGGGAAAATGGAAGAAAATG

TTCCAATATTTCCATTGGAGATGAGGTTCAATTTGAAATTAGCATAACTTCAAATAAGTGTCCAAAAAAG

GATTCTGACAGCTTTAAAATTAGGCCTCTGGGCTTTACGGAGGAAGTAGAGGTTATTCTTCAGTACATCT

GTGAATGTGAATGCCAAAGCGAAGGCATCCCTGAAAGTCCCAAGTGTCATGAAGGAAATGGGACATTTGA

GTGTGGCGCGTGCAGGTGCAATGAAGGGCGTGTTGGTAGACATTGTGAATGCAGCACAGATGAAGTTAAC

AGTGAAGACATGGATGCTTACTGCAGGAAAGAAAACAGTTCAGAAATCTGCAGTAACAATGGAGAGTGCG

TCTGCGGACAGTGTGTTTGTAGGAAGAGGGATAATACAAATGAAATTTATTCTGGCAAATTCTGCGAGTG

TGATAATTTCAACTGTGATAGATCCAATGGCTTAATTTGTGGAGGAAATGGTGTTTGCAAGTGTCGTGTG

TGTGAGTGCAACCCCAACTACACTGGCAGTGCATGTGACTGTTCTTTGGATACTAGTACTTGTGAAGCCA

GCAACGGACAGATCTGCAATGGCCGGGGCATCTGCGAGTGTGGTGTCTGTAAGTGTACAGATCCGAAGTT

TCAAGGGCAAACGTGTGAGATGTGTCAGACCTGCCTTGGTGTCTGTGCTGAGCATAAAGAATGTGTTCAG

TGCAGAGCCTTCAATAAAGGAGAAAAGAAAGACACATGCACACAGGAATGTTCCTATTTTAACATTACCA

AGGTAGAAAGTCGGGACAAATTACCCCAGCCGGTCCAACCTGATCCTGTGTCCCATTGTAAGGAGAAGGA

TGTTGACGACTGTTGGTTCTATTTTACGTATTCAGTGAATGGGAACAACGAGGTCATGGTTCATGTTGTG

GAGAATCCAGAGTGTCCCACTGGTCCAGACATCATTCCAATTGTAGCTGGTGTGGTTGCTGGAATTGTTC

TTATTGGCCTTGCATTACTGCTGATATGGAAGCTTTTAATGATAATTCATGACAGAAGGGAGTTTGCTAA

ATTTGAAAAGGAGAAAATGAATGCCAAATGGGACACGGGTGAAAATCCTATTTATAAGAGTGCCGTAACA

ACTGTGGTCAATCCGAAGTATGAGGGAAAATGAGTACTGCCCGTGCAAATCCCACAACACTGAATGCAAA

GTAGCAATTTCCATAGTCACAGTTAGGTAGCTTTAGGGCAATATTGCCATGGTTTTACTCATGTGCAGGT

TTTGAAAATGTACAATATGTATAATTTTTAAAATGTTTTATTATTTTGAAAATAATGTTGTAATTCATGC

CAGGGACTGACAAAAGACTTGAGACAGGATGGTTACTCTTGTCAGCTAAGGTCACATTGTGCCTTTTTGA

CCTTTTCTTCCTGGACTATTGAAATCAAGCTTATTGGATTAAGTGATATTTCTATAGCGATTGAAAGGGC

AATAGTTAAAGTAATGAGCATGATGAGAGTTTCTGTTAATCATGTATTAAAACTGATTTTTAGCTTTACA

AATATGTCAGTTTGCAGTTATGCAGAATCCAAAGTAAATGTCCTGCTAGCTAGTTAAGGATTGTTTTAAA

TCTGTTATTTTGCTATTTGCCTGTTAGACATGACTGATGACATATCTGAAAGACAAGTATGTTGAGAGTT

GCTGGTGTAAAATACGTTTGAAATAGTTGATCTACAAAGGCCATGGGAAAAATTCAGAGAGTTAGGAAGG

AAAAACCAATAGCTTTAAAACCTGTGTGCCATTTTAAGAGTTACTTAATGTTTGGTAACTTTTATGCCTT

CACTTTACAAATTCAAGCCTTAGATAAAAGAACCGAGCAATTTTCTGCTAAAAAGTCCTTGATTTAGCAC

TATTTACATACAGGCCATACTTTACAAAGTATTTGCTGAATGGGGACCTTTTGAGTTGAATTTATTTTAT

TATTTTTATTTTGTTTAATGTCTGGTGCTTTCTGTCACCTCTTCTAATCTTTTAATGTATTTGTTTGCAA

TTTTGGGGTAAGACTTTTTTTATGAGTACTTTTTCTTTGAAGTTTTAGCGGTCAATTTGCCTTTTTAATG

AACATGTGAAGTTATACTGTGGCTATGCAACAGCTCTCACCTACGCGAGTCTTACTTTGAGTTAGTGCCA

TAACAGACCACTGTATGTTTACTTCTCACCATTTGAGTTGCCCATCTTGTTTCACACTAGTCACATTCTT

GTTTTAAGTGCCTTTAGTTTTAACAGTTCACTTTTTACAGTGCTATTTACTGAAGTTATTTATTAAATAT

GCCTAAAATACTTAAATCGGATGTCTTGACTCTGATGTATTTTATCAGGTTGTGTGCATGAAATTTTTAT

AGATTAAAGAAGTTGAGGAAAAGCA

>NM_000213.5 Homo sapiens integrin subunit beta 4 (ITGB4), transcript variant 1, mRNA

CCCCCAACCCCCGCGCCCGCCCTCGGACAGTCCCTGCTCGCCCGCGCGCTGCAGCCCCATCTCCTAGCGG

CAGCCCAGGCGCGGAGGGAGCGAGTCCGCCCCGAGGTAGGTCCAGGACGGGCGCACAGCAGCAGCCGAGG

CTGGCCGGGAGAGGGAGGAAGAGGATGGCAGGGCCACGCCCCAGCCCATGGGCCAGGCTGCTCCTGGCAG

CCTTGATCAGCGTCAGCCTCTCTGGGACCTTGGCAAACCGCTGCAAGAAGGCCCCAGTGAAGAGCTGCAC

GGAGTGTGTCCGTGTGGATAAGGACTGCGCCTACTGCACAGACGAGATGTTCAGGGACCGGCGCTGCAAC

ACCCAGGCGGAGCTGCTGGCCGCGGGCTGCCAGCGGGAGAGCATCGTGGTCATGGAGAGCAGCTTCCAAA

TCACAGAGGAGACCCAGATTGACACCACCCTGCGGCGCAGCCAGATGTCCCCCCAAGGCCTGCGGGTCCG

TCTGCGGCCCGGTGAGGAGCGGCATTTTGAGCTGGAGGTGTTTGAGCCACTGGAGAGCCCCGTGGACCTG

TACATCCTCATGGACTTCTCCAACTCCATGTCCGATGATCTGGACAACCTCAAGAAGATGGGGCAGAACC

TGGCTCGGGTCCTGAGCCAGCTCACCAGCGACTACACTATTGGATTTGGCAAGTTTGTGGACAAAGTCAG

CGTCCCGCAGACGGACATGAGGCCTGAGAAGCTGAAGGAGCCCTGGCCCAACAGTGACCCCCCCTTCTCC

TTCAAGAACGTCATCAGCCTGACAGAAGATGTGGATGAGTTCCGGAATAAACTGCAGGGAGAGCGGATCT

CAGGCAACCTGGATGCTCCTGAGGGCGGCTTCGATGCCATCCTGCAGACAGCTGTGTGCACGAGGGACAT

TGGCTGGCGCCCGGACAGCACCCACCTGCTGGTCTTCTCCACCGAGTCAGCCTTCCACTATGAGGCTGAT

GGCGCCAACGTGCTGGCTGGCATCATGAGCCGCAACGATGAACGGTGCCACCTGGACACCACGGGCACCT

ACACCCAGTACAGGACACAGGACTACCCGTCGGTGCCCACCCTGGTGCGCCTGCTCGCCAAGCACAACAT

CATCCCCATCTTTGCTGTCACCAACTACTCCTATAGCTACTACGAGAAGCTTCACACCTATTTCCCTGTC

TCCTCACTGGGGGTGCTGCAGGAGGACTCGTCCAACATCGTGGAGCTGCTGGAGGAGGCCTTCAATCGGA

TCCGCTCCAACCTGGACATCCGGGCCCTAGACAGCCCCCGAGGCCTTCGGACAGAGGTCACCTCCAAGAT

GTTCCAGAAGACGAGGACTGGGTCCTTTCACATCCGGCGGGGGGAAGTGGGTATATACCAGGTGCAGCTG

CGGGCCCTTGAGCACGTGGATGGGACGCACGTGTGCCAGCTGCCGGAGGACCAGAAGGGCAACATCCATC

TGAAACCTTCCTTCTCCGACGGCCTCAAGATGGACGCGGGCATCATCTGTGATGTGTGCACCTGCGAGCT

GCAAAAAGAGGTGCGGTCAGCTCGCTGCAGCTTCAACGGAGACTTCGTGTGCGGACAGTGTGTGTGCAGC

GAGGGCTGGAGTGGCCAGACCTGCAACTGCTCCACCGGCTCTCTGAGTGACATTCAGCCCTGCCTGCGGG

AGGGCGAGGACAAGCCGTGCTCCGGCCGTGGGGAGTGCCAGTGCGGGCACTGTGTGTGCTACGGCGAAGG

CCGCTACGAGGGTCAGTTCTGCGAGTATGACAACTTCCAGTGTCCCCGCACTTCCGGGTTCCTCTGCAAT

GACCGAGGACGCTGCTCCATGGGCCAGTGTGTGTGTGAGCCTGGTTGGACAGGCCCAAGCTGTGACTGTC

CCCTCAGCAATGCCACCTGCATCGACAGCAATGGGGGCATCTGTAATGGACGTGGCCACTGTGAGTGTGG

CCGCTGCCACTGCCACCAGCAGTCGCTCTACACGGACACCATCTGCGAGATCAACTACTCGGCGATCCAC

CCGGGCCTCTGCGAGGACCTACGCTCCTGCGTGCAGTGCCAGGCGTGGGGCACCGGCGAGAAGAAGGGGC

GCACGTGTGAGGAATGCAACTTCAAGGTCAAGATGGTGGACGAGCTTAAGAGAGCCGAGGAGGTGGTGGT

GCGCTGCTCCTTCCGGGACGAGGATGACGACTGCACCTACAGCTACACCATGGAAGGTGACGGCGCCCCT

GGGCCCAACAGCACTGTCCTGGTGCACAAGAAGAAGGACTGCCCTCCGGGCTCCTTCTGGTGGCTCATCC

CCCTGCTCCTCCTCCTCCTGCCGCTCCTGGCCCTGCTACTGCTGCTATGCTGGAAGTACTGTGCCTGCTG

CAAGGCCTGCCTGGCACTTCTCCCGTGCTGCAACCGAGGTCACATGGTGGGCTTTAAGGAAGACCACTAC

ATGCTGCGGGAGAACCTGATGGCCTCTGACCACTTGGACACGCCCATGCTGCGCAGCGGGAACCTCAAGG

GCCGTGACGTGGTCCGCTGGAAGGTCACCAACAACATGCAGCGGCCTGGCTTTGCCACTCATGCCGCCAG

CATCAACCCCACAGAGCTGGTGCCCTACGGGCTGTCCTTGCGCCTGGCCCGCCTTTGCACCGAGAACCTG

CTGAAGCCTGACACTCGGGAGTGCGCCCAGCTGCGCCAGGAGGTGGAGGAGAACCTGAACGAGGTCTACA

GGCAGATCTCCGGTGTACACAAGCTCCAGCAGACCAAGTTCCGGCAGCAGCCCAATGCCGGGAAAAAGCA

AGACCACACCATTGTGGACACAGTGCTGATGGCGCCCCGCTCGGCCAAGCCGGCCCTGCTGAAGCTTACA

GAGAAGCAGGTGGAACAGAGGGCCTTCCACGACCTCAAGGTGGCCCCCGGCTACTACACCCTCACTGCAG

ACCAGGACGCCCGGGGCATGGTGGAGTTCCAGGAGGGCGTGGAGCTGGTGGACGTACGGGTGCCCCTCTT

TATCCGGCCTGAGGATGACGACGAGAAGCAGCTGCTGGTGGAGGCCATCGACGTGCCCGCAGGCACTGCC

ACCCTCGGCCGCCGCCTGGTAAACATCACCATCATCAAGGAGCAAGCCAGAGACGTGGTGTCCTTTGAGC

AGCCTGAGTTCTCGGTCAGCCGCGGGGACCAGGTGGCCCGCATCCCTGTCATCCGGCGTGTCCTGGACGG

CGGGAAGTCCCAGGTCTCCTACCGCACACAGGATGGCACCGCGCAGGGCAACCGGGACTACATCCCCGTG

GAGGGTGAGCTGCTGTTCCAGCCTGGGGAGGCCTGGAAAGAGCTGCAGGTGAAGCTCCTGGAGCTGCAAG

AAGTTGACTCCCTCCTGCGGGGCCGCCAGGTCCGCCGTTTCCACGTCCAGCTCAGCAACCCTAAGTTTGG

GGCCCACCTGGGCCAGCCCCACTCCACCACCATCATCATCAGGGACCCAGATGAACTGGACCGGAGCTTC

ACGAGTCAGATGTTGTCATCACAGCCACCCCCTCACGGCGACCTGGGCGCCCCGCAGAACCCCAATGCTA

AGGCCGCTGGGTCCAGGAAGATCCATTTCAACTGGCTGCCCCCTTCTGGCAAGCCAATGGGGTACAGGGT

AAAGTACTGGATTCAGGGTGACTCCGAATCCGAAGCCCACCTGCTCGACAGCAAGGTGCCCTCAGTGGAG

CTCACCAACCTGTACCCGTATTGCGACTATGAGATGAAGGTGTGCGCCTACGGGGCTCAGGGCGAGGGAC

CCTACAGCTCCCTGGTGTCCTGCCGCACCCACCAGGAAGTGCCCAGCGAGCCAGGGCGTCTGGCCTTCAA

TGTCGTCTCCTCCACGGTGACCCAGCTGAGCTGGGCTGAGCCGGCTGAGACCAACGGTGAGATCACAGCC

TACGAGGTCTGCTATGGCCTGGTCAACGATGACAACCGACCTATTGGGCCCATGAAGAAAGTGCTGGTTG

ACAACCCTAAGAACCGGATGCTGCTTATTGAGAACCTTCGGGAGTCCCAGCCCTACCGCTACACGGTGAA

GGCGCGCAACGGGGCCGGCTGGGGGCCTGAGCGGGAGGCCATCATCAACCTGGCCACCCAGCCCAAGAGG

CCCATGTCCATCCCCATCATCCCTGACATCCCTATCGTGGACGCCCAGAGCGGGGAGGACTACGACAGCT

TCCTTATGTACAGCGATGACGTTCTACGCTCTCCATCGGGCAGCCAGAGGCCCAGCGTCTCCGATGACAC

TGGCTGCGGCTGGAAGTTCGAGCCCCTGCTGGGGGAGGAGCTGGACCTGCGGCGCGTCACGTGGCGGCTG

CCCCCGGAGCTCATCCCGCGCCTGTCGGCCAGCAGCGGGCGCTCCTCCGACGCCGAGGCGCCCCACGGGC

CCCCGGACGACGGCGGCGCGGGCGGGAAGGGCGGCAGCCTGCCCCGCAGTGCGACACCCGGGCCCCCCGG

AGAGCACCTGGTGAATGGCCGGATGGACTTTGCCTTCCCGGGCAGCACCAACTCCCTGCACAGGATGACC

ACGACCAGTGCTGCTGCCTATGGCACCCACCTGAGCCCACACGTGCCCCACCGCGTGCTAAGCACATCCT

CCACCCTCACACGGGACTACAACTCACTGACCCGCTCAGAACACTCACACTCGACCACACTGCCCAGGGA

CTACTCCACCCTCACCTCCGTCTCCTCCCACGACTCTCGCCTGACTGCTGGTGTGCCCGACACGCCCACC

CGCCTGGTGTTCTCTGCCCTGGGGCCCACATCTCTCAGAGTGAGCTGGCAGGAGCCGCGGTGCGAGCGGC

CGCTGCAGGGCTACAGTGTGGAGTACCAGCTGCTGAACGGCGGTGAGCTGCATCGGCTCAACATCCCCAA

CCCTGCCCAGACCTCGGTGGTGGTGGAAGACCTCCTGCCCAACCACTCCTACGTGTTCCGCGTGCGGGCC

CAGAGCCAGGAAGGCTGGGGCCGAGAGCGTGAGGGTGTCATCACCATTGAATCCCAGGTGCACCCGCAGA

GCCCACTGTGTCCCCTGCCAGGCTCCGCCTTCACTTTGAGCACTCCCAGTGCCCCAGGCCCGCTGGTGTT

CACTGCCCTGAGCCCAGACTCGCTGCAGCTGAGCTGGGAGCGGCCACGGAGGCCCAATGGGGATATCGTC

GGCTACCTGGTGACCTGTGAGATGGCCCAAGGAGGAGGGCCAGCCACCGCATTCCGGGTGGATGGAGACA

GCCCCGAGAGCCGGCTGACCGTGCCGGGCCTCAGCGAGAACGTGCCCTACAAGTTCAAGGTGCAGGCCAG

GACCACTGAGGGCTTCGGGCCAGAGCGCGAGGGCATCATCACCATAGAGTCCCAGGATGGAGGACCCTTC

CCGCAGCTGGGCAGCCGTGCCGGGCTCTTCCAGCACCCGCTGCAAAGCGAGTACAGCAGCATCACCACCA

CCCACACCAGCGCCACCGAGCCCTTCCTAGTGGATGGGCTGACCCTGGGGGCCCAGCACCTGGAGGCAGG

CGGCTCCCTCACCCGGCATGTGACCCAGGAGTTTGTGAGCCGGACACTGACCACCAGCGGAACCCTTAGC

ACCCACATGGACCAACAGTTCTTCCAAACTTGACCGCACCCTGCCCCACCCCCGCCACGTCCCACTAGGC

GTCCTCCCGACTCCTCTCCCGGAGCCTCCTCAGCTACTCCATCCTTGCACCCCTGGGGGCCCAGCCCACC

CGCATGCACAGAGCAGGGGCTAGGTGTCTCCTGGGAGGCATGAAGGGGGCAAGGTCCGTCCTCTGTGGGC

CCAAACCTATTTGTAACCAAAGAGCTGGGAGCAGCACAAGGACCCAGCCTTTGTTCTGCACTTAATAAAT

GGTTTTGCTACTGCTA

>NM_002222.7 Homo sapiens inositol 1,4,5-trisphosphate receptor type 1 (ITPR1), transcript variant 2, mRNA

GCAGTAACCATGTGGATGTGCTGCTGAAGCGTTTCCTCAAGCTCGCTGGGGTGGGAGGAGAGGAGGAGGA

GGAGGTGGTGGTGGAGGAGGAGGCAGGGGGTGGAGAGAGAGAAAGCGCACGCCGAGAGGAGGTGTGGGTG

TTCCGCTTCCATCCTAACGGAACGAGCTCCCTCTTCGCGGACATGGGATTACCCAGCGGCTGCTAACCCC

TCTCCTCGCCCTGCTCCCCCAAACCGGCGTGGCTCCCCGGGCACCAAGGAGCTGACTACAGAGGAGCAGG

ATTTGCACCCCTCGCTGGGCTTGCTTTGGCAACAGAGTGCCTGACCCAGGTCAGGATTTTCAAGAAAGAC

ATGTCTGACAAAATGTCTAGCTTCCTACATATTGGAGACATTTGTTCTCTGTACGCGGAGGGATCGACAA

ATGGATTTATTAGCACCTTGGGCCTGGTTGATGATCGTTGTGTTGTACAGCCAGAAACCGGGGACCTTAA

CAATCCACCTAAGAAATTCAGAGACTGCCTCTTTAAGCTATGTCCCATGAACCGCTACTCTGCCCAAAAG

CAGTTCTGGAAAGCCGCTAAGCCTGGGGCCAACAGCACCACAGACGCAGTGCTACTCAACAAACTGCACC

ACGCTGCAGACTTGGAAAAGAAGCAGAATGAGACAGAAAACAGGAAATTGCTGGGGACCGTAATCCAGTA

TGGCAATGTGATCCAGCTCCTGCATTTGAAAAGTAATAAATACCTAACAGTGAATAAGAGGCTTCCTGCT

CTGTTGGAGAAGAATGCCATGAGAGTCACATTGGACGAGGCTGGAAATGAAGGGTCCTGGTTTTATATTC

AGCCATTCTACAAGCTGCGATCCATTGGAGACAGCGTGGTCATAGGTGACAAGGTGGTTCTGAACCCCGT

CAATGCTGGTCAGCCCCTACATGCTAGCAGCCATCAACTGGTAGATAACCCAGGCTGCAATGAGGTCAAT

TCCGTCAACTGCAATACAAGCTGGAAAATAGTCCTTTTCATGAAATGGAGTGATAACAAAGACGACATAT

TAAAGGGGGGTGACGTGGTGAGGCTGTTTCATGCTGAGCAGGAGAAGTTTCTCACCTGTGACGAACACAG

GAAGAAGCAGCACGTCTTCCTGAGAACCACGGGCCGGCAGTCGGCCACATCTGCCACCAGTTCAAAAGCC

CTGTGGGAGGTGGAGGTGGTCCAGCATGACCCATGTCGGGGCGGAGCAGGGTATTGGAACAGCCTTTTCC

GTTTCAAGCATCTGGCCACGGGGCATTACTTGGCAGCAGAGGTGGACCCTGATCAGGACGCCTCTCGAAG

TAGGTTGCGGAATGCCCAAGAAAAGATGGTATACTCCCTGGTCTCTGTGCCTGAAGGCAATGACATCTCC

TCCATTTTCGAGCTAGATCCCACCACTCTGCGTGGAGGTGACAGCCTTGTCCCAAGGAACTCTTATGTTC

GGCTCAGACACCTATGTACTAATACCTGGGTTCACAGCACAAATATTCCTATTGACAAGGAAGAAGAAAA

GCCCGTGATGCTGAAAATTGGCACCTCTCCTGTGAAGGAGGATAAGGAAGCATTTGCCATAGTTCCGGTT

TCTCCTGCTGAAGTTCGGGACCTGGACTTTGCCAATGATGCCAGCAAGGTGCTGGGCTCCATTGCTGGGA

AGCTAGAGAAGGGCACCATCACCCAGAATGAAAGGAGGTCTGTAACCAAGCTGCTAGAAGATTTGGTTTA

CTTCGTCACTGGTGGAACTAATTCTGGTCAAGATGTTCTCGAAGTTGTCTTCTCCAAGCCCAACAGAGAA

CGGCAGAAACTGATGAGAGAACAGAATATTCTCAAGCAGATCTTCAAGTTGTTACAAGCCCCATTCACAG

ACTGCGGTGATGGCCCAATGCTTCGGCTGGAAGAGCTCGGGGACCAGCGGCACGCTCCTTTCAGACACAT

CTGCCGGCTCTGCTACAGGGTGCTGAGACACTCGCAGCAAGACTACAGGAAGAACCAGGAGTATATAGCC

AAGCAGTTTGGCTTCATGCAGAAGCAGATTGGCTATGATGTGTTGGCTGAAGACACTATCACTGCCCTGC

TCCACAATAATCGGAAACTCCTGGAAAAACACATTACCGCGGCAGAGATTGACACATTTGTCAGCCTGGT

GCGAAAGAACAGGGAGCCCAGATTCTTAGATTACCTCTCCGACCTCTGTGTCTCCATGAACAAATCAATT

CCAGTGACCCAGGAACTGATATGTAAAGCTGTGCTGAACCCCACCAACGCTGACATCCTGATTGAGACCA

AGTTGGTTCTTTCTCGTTTTGAATTTGAAGGTGTCTCTTCCACTGGAGAGAATGCTCTGGAGGCAGGAGA

AGACGAGGAAGAGGTGTGGCTGTTTTGGAGGGACAGCAACAAAGAGATTCGCAGCAAGAGTGTGAGGGAA

TTGGCTCAGGATGCTAAAGAAGGGCAGAAGGAGGACCGAGACGTTCTCAGCTACTACAGATATCAGCTGA

ACCTCTTTGCGAGGATGTGTCTGGACCGCCAATACCTGGCCATCAACGAAATCTCAGGCCAGCTGGATGT

CGATCTCATTCTCCGCTGCATGTCTGACGAGAACCTGCCCTATGACCTCAGGGCGTCCTTCTGCCGCCTC

ATGCTTCACATGCATGTGGACCGAGATCCCCAGGAACAAGTCACCCCCGTGAAATATGCCCGCCTCTGGT

CGGAGATTCCCTCGGAGATCGCCATTGACGACTATGATAGTAGTGGAGCTTCCAAAGATGAAATTAAGGA

GAGATTTGCTCAGACCATGGAGTTTGTGGAGGAGTATTTAAGAGATGTGGTTTGTCAGAGGTTCCCTTTC

TCTGATAAAGAGAAGAATAAGCTTACGTTTGAGGTTGTAAATTTAGCTAGGAATCTCATATACTTTGGTT

TCTACAACTTCTCTGACCTTCTACGATTAACTAAGATCCTTCTGGCCATATTGGACTGTGTACATGTGAC

AACAATCTTCCCCATTAGCAAGATGGCGAAAGGAGAAGAGAATAAAGGCAGTAACGTGATGAGATCTATT

CATGGCGTGGGAGAGCTGATGACCCAGGTGGTGCTCCGGGGAGGAGGCTTTTTGCCCATGACTCCCATGG

CTGCTGCCCCTGAAGGCAATGTGAAGCAGGCAGAGCCTGAGAAGGAGGACATCATGGTCATGGACACCAA

GCTGAAGATCATTGAGATACTCCAGTTTATTTTGAATGTGAGGTTGGATTATAGGATCTCCTGCCTCCTG

TGTATATTTAAGCGAGAGTTTGATGAAAGCAATTCCCAGACTTCAGAAACATCCTCCGGAAACAGCAGCC

AAGAAGGGCCAAGTAATGTACCAGGTGCTCTTGACTTTGAACACATTGAAGAACAAGCAGAAGGCATCTT

TGGAGGAAGTGAGGAGAACACCCCACTGGACTTGGATGACCACGGCGGCAGAACCTTTCTCCGTGTCCTG

CTCCACTTGACGATGCATGACTACCCACCCCTGGTGTCAGGGGCCCTGCAGCTCCTCTTCCGGCACTTCA

GCCAGAGGCAGGAGGTGCTCCAGGCCTTCAAACAGGTTCAACTGCTGGTTACCAGCCAAGATGTGGACAA

CTACAAACAGATCAAACAAGACTTGGATCAACTGAGGTCCATCGTGGAAAAGTCAGAGCTTTGGGTGTAC

AAAGGGCAGGGCCCCGATGAGACTATGGATGGTGCATCTGGAGAAAATGAACATAAGAAAACGGAGGAGG

GAAATAACAAGCCACAAAAGCATGAAAGCACCAGCAGCTACAACTACAGAGTGGTCAAAGAGATTTTGAT

TCGGCTTAGCAAACTCTGTGTTCAAGAGAGTGCCTCAGTGAGAAAGAGCAGGAAGCAGCAACAGCGTCTG

CTCCGGAACATGGGCGCGCACGCCGTGGTGCTGGAGCTGCTGCAGATTCCCTATGAGAAGGCCGAAGATA

CCAAGATGCAAGAGATAATGAGGTTGGCTCATGAATTTTTGCAGAATTTCTGCGCAGGCAACCAGCAGAA

TCAAGCTTTGCTACATAAACACATAAACCTGTTTCTCAACCCAGGGATCCTGGAGGCAGTAACCATGCAG

CACATCTTCATGAACAATTTCCAGCTTTGCAGTGAGATCAACGAGAGAGTTGTTCAGCACTTCGTTCACT

GCATAGAGACTCACGGTCGGAATGTCCAGTATATAAAGTTCTTACAGACAATTGTCAAGGCAGAAGGGAA

ATTTATTAAAAAATGCCAAGACATGGTTATGGCCGAGCTGGTCAATTCGGGAGAGGATGTCCTCGTGTTC

TACAACGACAGAGCCTCTTTCCAGACTCTGATCCAGATGATGCGGTCAGAACGGGATCGGATGGATGAGA

ACAGCCCTCTCATGTACCACATCCACTTGGTCGAGCTCCTGGCTGTGTGCACGGAGGGTAAGAATGTCTA

CACAGAGATCAAGTGCAACTCCCTGCTCCCGCTGGATGACATCGTTCGCGTGGTGACCCACGAGGACTGC

ATCCCTGAGGTTAAAATTGCATACATTAACTTCCTGAATCACTGCTATGTGGATACAGAGGTGGAAATGA

AGGAGATTTATACCAGCAATCACATGTGGAAATTGTTTGAGAATTTCCTTGTAGACATCTGCAGGGCCTG

TAACAACACTAGTGACAGGAAACATGCAGACTCGATTTTGGAGAAGTATGTCACCGAAATCGTCATGAGT

ATTGTTACTACTTTCTTCAGCTCTCCCTTCTCAGACCAGAGTACGACTTTGCAGACTCGCCAGCCTGTCT

TTGTGCAACTGCTGCAAGGCGTGTTCAGGGTTTACCACTGCAACTGGTTAATGCCAAGCCAAAAAGCCTC

CGTGGAGAGCTGTATTCGGGTGCTGTCTGATGTAGCCAAGAGCCGGGCCATTGCCATTCCCGTGGACCTG

GACAGCCAAGTCAACAACCTCTTTCTCAAGTCCCACAGCATTGTGCAGAAAACAGCCATGAACTGGCGGC

TCTCAGCCCGCAATGCCGCACGCAGGGACTCTGTTCTGGCAGCTTCCAGAGACTACCGGAATATCATTGA

GAGATTGCAGGACATCGTCTCCGCGCTGGAGGACCGTCTCAGGCCCCTGGTGCAGGCAGAGTTATCTGTG

CTCGTGGATGTTCTCCACAGACCCGAGCTGCTTTTCCCAGAGAACACAGACGCCAGAAGGAAATGTGAAA

GTGGCGGTTTCATTTGCAAGTTAATAAAGCATACAAAACAGCTGCTAGAAGAAAATGAAGAGAAGCTCTG

CATTAAGGTCCTACAGACCCTGAGGGAAATGATGACCAAAGATAGAGGCTATGGAGAAAAGGGTGAGGCG

CTCAGGCAAGTTCTGGTCAACCGTTACTATGGAAACGTCAGACCTTCGGGACGAAGAGAGAGCCTTACCA

GCTTTGGCAATGGCCCACTGTCAGCAGGAGGACCCGGCAAGCCCGGGGGAGGAGGGGGAGGTTCCGGATC

CAGCTCTATGAGCAGGGGTGAGATGAGTCTGGCCGAGGTTCAGTGTCACCTTGACAAGGAGGGGGCTTCC

AATCTAGTTATCGACCTCATCATGAACGCATCCAGTGACCGAGTGTTCCATGAAAGCATTCTCCTGGCCA

TTGCCCTTCTGGAAGGAGGCAACACCACCATCCAGCACTCCTTTTTCTGTCGCTTGACAGAAGATAAGAA

GTCAGAGAAATTCTTTAAGGTGTTTTATGACCGGATGAAGGTGGCCCAGCAAGAAATCAAAGCAACAGTG

ACAGTGAACACCAGTGACTTGGGAAATAAAAAGAAAGACGATGAGGTAGACAGGGATGCCCCATCACGGA

AAAAAGCTAAAGAGCCCACAACACAGATAACAGAAGAGGTCCGGGATCAGCTCCTGGAGGCCTCCGCTGC

CACCAGGAAAGCCTTCACCACTTTCAGGAGGGAGGCTGATCCCGACGACCACTACCAGCCTGGAGAGGGC

ACCCAGGCCACTGCCGACAAGGCCAAGGACGACCTGGAGATGAGCGCGGTCATCACCATCATGCAGCCCA

TCCTCCGCTTCCTTCAGCTCCTGTGTGAAAACCACAACCGAGACCTGCAGAACTTCCTCCGTTGCCAAAA

TAACAAGACCAACTACAATTTGGTATGTGAGACCCTGCAGTTTCTGGACTGTATTTGTGGAAGCACAACT

GGAGGCCTTGGTCTTCTGGGCTTGTATATAAATGAAAAGAATGTAGCGCTTATCAACCAAACCCTGGAAA

GTCTGACCGAATACTGTCAAGGACCTTGCCATGAGAACCAGAACTGCATAGCCACCCATGAATCCAATGG

CATTGACATCATCACAGCCCTGATCCTCAATGATATCAATCCTTTGGGAAAGAAGAGGATGGACCTTGTG

TTAGAACTGAAGAACAATGCCTCGAAGTTGCTCCTGGCCATCATGGAAAGCAGGCACGACAGTGAAAACG

CAGAGAGGATACTTTATAACATGAGGCCCAAGGAACTGGTGGAAGTGATCAAGAAAGCCTACATGCAAGG

TGAAGTGGAATTTGAGGATGGAGAAAACGGTGAGGATGGGGCGGCGTCCCCCAGGAACGTGGGGCACAAC

ATCTACATATTAGCCCATCAGTTGGCTCGGCATAACAAAGAACTTCAGAGCATGCTGAAACCTGGTGGCC

AAGTGGACGGAGATGAAGCCCTGGAGTTTTATGCCAAGCACACGGCGCAGATAGAGATTGTCAGATTAGA

CCGAACAATGGAACAGATAGTCTTTCCCGTGCCCAGCATATGTGAATTCCTAACCAAGGAGTCAAAACTA

CGAATTTACTATACTACAGAGAGAGACGAACAAGGCAGCAAAATCAATGATTTCTTTCTGCGGTCTGAAG

ACCTCTTCAATGAAATGAATTGGCAGAAGAAACTGAGAGCCCAGCCCGTGTTGTACTGGTGTGCCCGCAA

CATGTCTTTCTGGAGCAGCATTTCGTTTAACCTGGCCGTCCTGATGAACCTGCTGGTGGCGTTTTTCTAC

CCGTTTAAGGGAGTCCGAGGAGGAACCCTGGAGCCCCACTGGTCGGGACTCCTGTGGACAGCCATGCTCA

TCTCTCTGGCCATCGTCATTGCCCTCCCCAAGCCCCATGGCATCCGGGCCTTAATTGCCTCCACAATTCT

ACGACTGATATTTTCAGTCGGGTTACAACCCACGTTGTTTCTTCTGGGCGCTTTCAATGTATGCAATAAA

ATCATCTTTCTAATGAGCTTTGTGGGCAACTGTGGGACATTCACAAGAGGCTACCGAGCCATGGTTCTGG

ATGTTGAGTTCCTCTATCATTTGTTGTATCTGGTGATCTGTGCCATGGGGCTCTTTGTCCATGAATTCTT

CTACAGTCTGCTGCTTTTTGATTTAGTGTACAGAGAAGAGACTTTGCTTAATGTCATTAAAAGTGTCACT

CGCAATGGACGGTCCATCATCCTGACAGCAGTTCTGGCTCTGATCCTCGTTTACCTGTTCTCAATAGTGG

GCTATCTTTTCTTCAAGGATGACTTTATCTTGGAAGTAGATAGGCTGCCCAATGAAACAGCTGTTCCAGA

AACCGGCGAGAGTTTGGCAAGCGAGTTCCTGTTCTCCGATGTGTGTAGGGTGGAGAGTGGGGAGAACTGC

TCCTCTCCTGCACCCAGAGAAGAGCTGGTCCCTGCAGAAGAGACGGAACAGGATAAAGAGCACACATGTG

AGACGCTGCTGATGTGCATTGTCACTGTGCTGAGTCACGGGCTGCGGAGCGGGGGTGGAGTAGGAGATGT

ACTCAGGAAGCCGTCCAAAGAGGAACCCCTGTTTGCTGCTAGAGTTATTTATGACCTCTTGTTCTTCTTC

ATGGTCATCATCATTGTTCTTAACCTGATTTTTGGGGTTATCATTGACACTTTTGCTGACCTGAGGAGTG

AGAAGCAGAAGAAGGAAGAGATCTTGAAGACCACGTGCTTTATCTGTGGCTTGGAAAGAGACAAGTTTGA

CAACAAGACTGTCACCTTTGAAGAGCACATCAAGGAAGAACACAACATGTGGCACTATCTGTGCTTCATC

GTCCTGGTGAAAGTAAAGGACTCCACCGAATATACTGGGCCTGAGAGTTACGTGGCAGAAATGATCAAGG

AAAGAAACCTTGACTGGTTCCCCAGGATGAGAGCCATGTCATTGGTCAGCAGTGATTCTGAAGGAGAACA

GAATGAGCTGAGAAACCTGCAGGAGAAGCTGGAGTCCACCATGAAACTTGTCACGAACCTTTCTGGCCAG

CTGTCGGAATTAAAGGATCAGATGACAGAACAAAGGAAGCAGAAACAAAGAATTGGTCTTCTAGGACATC

CTCCTCACATGAATGTCAACCCACAACAACCAGCATAAGCAAATGAAAGAAAGGAATTGTATTTACCTTT

TATAATTATTATTAGTGTGGGTATGGCTAATGAGTTCTGATTCACCCACGAAGGTTACATTTATGCTGAA

TACATTTGTAAATACTCAGTTTTATACTGTATGTATATGATTGCTACTCTAAAGGTTTGGATATATGTAT

TGTAATTAGAATTGTTGGCATGATGACATTTCATTTGTGCCAAAAATATTAAAAATGCCTTTTTTGGAAG

GACTAACAGAAAGCACCTGATTTGCACTTGAACCAGATTATAGATTTAAAAGTATATGACATGTATTTTG

TATTTAAAACTAGAATAGCCAGTATTTATGTTTTTTATAAAACTGTGCAATACGAATTATGCAATCACAA

TACATTTGTAGCTCCCGAGTGTCCTAAAGGGAGTGCACTTCTTTGAAGCTGGTGTGTTAATACTATGTAA

TAAATGGTTAACTTTCAAATGATGCTGCTGCCAAAATTATATTAATAGTGAGTTTCAGGCCCCTGGGCAT

TTTGTACCATGTAATTATCCTCTGGTGATGCTGTTTCTCGTTAGTGGCAGTAGTGCCTCCGTCTCCTAGT

GATAATGCTCCAAGTCTATGAACTGTTAAATCAGCATTCATTTTAAGAAAAGCAACTTTAGTTTCAAAGA

TACTTTTAAGCTTCTAAATTGATCATTTAAACTATTTCTTTAAATAAGAGAGCCAAATTAGAGGCTCATA

CTTTAGCTTGTGAAGAAGATAATGAATTTTTTAAAGGGAACTTTCTATGCAATGTTCAGGATAAATGCAT

ACTGCTGGCCAATCAGTGTCATCTCCTGGGTAAATTTTGATGTCGCATTATAAAGACATGCATAATTGAT

GGTTTCTAGATTATCTAGTCCAAACAATAGAGTTTATTTTTTCTTCATCTGAACCAACATGCTACAGTAG

CTAAGAAGTATTAAAACTATATACATCCATATAAAGATGAAATATGAACTATCTCATTAGAAGTCATAGT

TGACCACAGACATGTTATTCTTCTGAAAGAGCCACATTTTGGTTTTATTTCTTGTCACATGATTTCTTTT

CTTGATGGATGAAAAATATGAAAGGAAACTTTTATATCTGTTGCCTAGTTTTGTACATGGATCTCATTTT

ACAAGAGAATCTCTCTGCAAAAAAAAAAAAAACAGTTTAAAAATGCATTGAAAGCAGAGTTCTGAAATGA

GTAAAGTTTGTAAATGCATATATAAAAATATTTAATAAATGATGCAGAATATACAGTGA

>NM_004521.3 Homo sapiens kinesin family member 5B (KIF5B), mRNA

GTCGCCCAACGGCGGCCTCAGGAGTGATCGGGCAGCAGTCGGCCGGCCAGCGGACGGCAGAGCGGGCGGA

CGGGTAGGCCCGGCCTGCTCTTCGCGAGGAGGAAGAAGGTGGCCACTCTCCCGGTCCCCAGAACCTCCCC

AGCCCCCGCAGTCCGCCCAGACCGTAAAGGGGGACGCTGAGGAGCCGCGGACGCTCTCCCCGGTGCCGCC

GCCGCTGCCGCCGCCATGGCTGCCATGATGGATCGGAAGTGAGCATTAGGGTTAACGGCTGCCGGCGCCG

GCTCTTCAAGTCCCGGCTCCCCGGCCGCCTCCACCCGGGGAAGCGCAGCGCGGCGCAGCTGACTGCTGCC

TCTCACGGCCCTCGCGACCACAAGCCCTCAGGTCCGGCGCGTTCCCTGCAAGACTGAGCGGCGGGGAGTG

GCTCCCGGCCGCCGGCCCCGGCTGCGAGAAAGATGGCGGACCTGGCCGAGTGCAACATCAAAGTGATGTG

TCGCTTCAGACCTCTCAACGAGTCTGAAGTGAACCGCGGCGACAAGTACATCGCCAAGTTTCAGGGAGAA

GACACGGTCGTGATCGCGTCCAAGCCTTATGCATTTGATCGGGTGTTCCAGTCAAGCACATCTCAAGAGC

AAGTGTATAATGACTGTGCAAAGAAGATTGTTAAAGATGTACTTGAAGGATATAATGGAACAATATTTGC

ATATGGACAAACATCCTCTGGGAAGACACACACAATGGAGGGTAAACTTCATGATCCAGAAGGCATGGGA

ATTATTCCAAGAATAGTGCAAGATATTTTTAATTATATTTACTCCATGGATGAAAATTTGGAATTTCATA

TTAAGGTTTCATATTTTGAAATATATTTGGATAAGATAAGGGACCTGTTAGATGTTTCAAAGACCAACCT

TTCAGTTCATGAAGACAAAAACCGAGTTCCCTATGTAAAGGGGTGCACAGAGCGTTTTGTATGTAGTCCA

GATGAAGTTATGGATACCATAGATGAAGGAAAATCCAACAGACATGTAGCAGTTACAAATATGAATGAAC

ATAGCTCTAGGAGTCACAGTATATTTCTTATTAATGTCAAACAAGAGAACACACAAACGGAACAAAAGCT

GAGTGGAAAACTTTATCTGGTTGATTTAGCTGGTAGTGAAAAGGTTAGTAAAACTGGAGCTGAAGGTGCT

GTGCTGGATGAAGCTAAAAACATCAACAAGTCACTTTCTGCTCTTGGAAATGTTATTTCTGCTTTGGCTG

AGGGTAGTACATATGTTCCATATCGAGATAGTAAAATGACAAGAATCCTTCAAGATTCATTAGGTGGCAA

CTGTAGAACCACTATTGTAATTTGCTGCTCTCCATCATCATACAATGAGTCTGAAACAAAATCTACACTC

TTATTTGGCCAAAGGGCCAAAACAATTAAGAACACAGTTTGTGTCAATGTGGAGTTAACTGCAGAACAGT

GGAAAAAGAAGTATGAAAAAGAAAAAGAAAAAAATAAGATCCTGCGGAACACTATTCAGTGGCTTGAAAA

TGAGCTCAACAGATGGCGTAATGGGGAGACGGTGCCTATTGATGAACAGTTTGACAAAGAGAAAGCCAAC

TTGGAAGCTTTCACAGTGGATAAAGATATTACTCTTACCAATGATAAACCAGCAACCGCAATTGGAGTTA

TAGGAAATTTTACTGATGCTGAAAGAAGAAAGTGTGAAGAAGAAATTGCTAAATTATACAAACAGCTTGA

TGACAAGGATGAAGAAATTAACCAGCAAAGTCAACTGGTAGAGAAACTGAAGACGCAAATGTTGGATCAG

GAGGAGCTTTTGGCATCTACCAGAAGGGATCAAGACAATATGCAAGCTGAGCTGAATCGCCTTCAAGCAG

AAAATGATGCCTCTAAAGAAGAAGTGAAAGAAGTTTTACAGGCCCTAGAAGAACTTGCTGTCAATTATGA

TCAGAAGTCTCAGGAAGTTGAAGACAAAACTAAGGAATATGAATTGCTTAGTGATGAATTGAATCAGAAA

TCGGCAACTTTAGCGAGTATAGATGCTGAGCTTCAGAAACTTAAGGAAATGACCAACCACCAGAAAAAAC

GAGCAGCTGAGATGATGGCATCTTTACTAAAAGACCTTGCAGAAATAGGAATTGCTGTGGGAAATAATGA

TGTAAAGCAGCCTGAGGGAACTGGCATGATAGATGAAGAGTTCACTGTTGCAAGACTCTACATTAGCAAA

ATGAAGTCAGAAGTAAAAACCATGGTGAAACGTTGCAAGCAGTTAGAAAGCACACAAACTGAGAGCAACA

AAAAAATGGAAGAAAATGAAAAGGAGTTAGCAGCATGTCAGCTTCGTATCTCTCAACATGAAGCCAAAAT

CAAGTCATTGACTGAATACCTTCAAAATGTGGAACAAAAGAAAAGACAGTTGGAGGAATCTGTCGATGCC

CTCAGTGAAGAACTAGTCCAGCTTCGAGCACAAGAGAAAGTCCATGAAATGGAAAAGGAGCACTTAAATA

AGGTTCAGACTGCAAATGAAGTTAAGCAAGCTGTTGAACAGCAGATCCAGAGCCATAGAGAAACTCATCA

AAAACAGATCAGTAGTTTGAGAGATGAAGTAGAAGCAAAAGCAAAACTTATTACTGATCTTCAAGACCAA

AACCAGAAAATGATGTTAGAGCAGGAACGTCTAAGAGTAGAACATGAGAAGTTGAAAGCCACAGATCAGG

AAAAGAGCAGAAAACTACATGAACTTACGGTTATGCAAGATAGACGAGAACAAGCAAGACAAGACTTGAA

GGGTTTGGAAGAGACAGTGGCAAAAGAACTTCAGACTTTACACAACCTGCGCAAACTCTTTGTTCAGGAC

CTGGCTACAAGAGTTAAAAAGAGTGCTGAGATTGATTCTGATGACACCGGAGGCAGCGCTGCTCAGAAGC

AAAAAATCTCCTTTCTTGAAAATAATCTTGAACAGCTCACTAAAGTGCACAAACAGTTGGTACGTGATAA

TGCAGATCTCCGCTGTGAACTTCCTAAGTTGGAAAAGCGACTTCGAGCTACAGCTGAGAGAGTGAAAGCT

TTGGAATCAGCACTGAAAGAAGCTAAAGAAAATGCATCTCGTGATCGCAAACGCTATCAGCAAGAAGTAG

ATCGCATAAAGGAAGCAGTCAGGTCAAAGAATATGGCCAGAAGAGGGCATTCTGCACAGATTGCTAAACC

TATTCGTCCCGGGCAACATCCAGCAGCTTCTCCAACTCACCCAAGTGCAATTCGTGGAGGAGGTGCATTT

GTTCAGAACAGCCAGCCAGTGGCAGTGCGAGGTGGAGGAGGCAAACAAGTGTAATCGTTTATACATACCC

ACAGGTGTTAAAAAGTAATCGAAGTACGAAGAGGACATGGTATCAAGCAGTCATTCAATGACTATAACCT

CTACTCCCTTGGGATTGTAGAATTATAACTTTTAAAAAAAATGTATAAATTATACCTGGCCTGTACAGCT

GTTTCCTACCTACTCTTCTTGTAAACTCTGCTGCTTCCCAACACAACTAGAGTGCAATTTTGGCATCTTA

GGAGGGAAAAAGGACAGTTTACAACTGTGGCCCTATTTATTACACAGTTTGTCTATCGTGTCTTAAATTT

AGTCTTTACTGTGCCAAGCTAACTGTACCTTATAGGACTGTACTTTTTGTATTTTTTGTGTATGTTTATT

TTTTAATCTCAGTTTAAATTACCTAGCTGCTACTGCTTCTTGTTTTTCTTTTCCTATTAAAACGTCTTCC

TTTTTTTTTCTTAAGAGAAAATGGAACATTTAGGTTAAATGTCTTTAAATTTTACCACTTAACAACACTA

CATGCCCATAAAATATATCCAGTCAGTACTGTATTTTAAAATCCCTTGAAATGATGATATCAGGGTTAAA

ATTACTTGTATTGTTTCTGAAGTTTGCTCCTGAAAACTACTGTTTGAGCACTGAAACGTTACAAATGCCT

AATAGGCATTTGAGACTGAGCAAGGCTACTTGTTATCTCATGAAATGCCTGTTGCCGAGTTATTTTGAAT

AGAAATATTTTAAAGTATCAAAAGCAGATCTTAGTTTAAGGGAGTTTGGAAAAGGAATTATATTTCTCTT

TTTCCTGATTCTGTACTCAACAAGTCTTGATGGAATTAAAATACTCTGCTTTATTCTGGTGAGCCTGCTA

GCTAATATAAGTATTGGACAGGTAATAATTTGTCATCTTTAATATTAGTAAAATGAATTAAGATATTATA

GGATTAAACATAATTTTATACGGTTAGTACTTTATTGGCCGACCTAAATTTATAGCGTGTGGAAATTGAG

AAAAATGAAGAAACAGGACAGATATATGATGAATTAAAAATATATATAGGTCAATTTTGGTCTGAAATCC

CTGAGGTGTTTTTAACCTGCTACACTAATTTGTACACTAATTTATTTCTTTAGTCTAGAAATAGTAAATT

GTTTGCAAGTCACTAATAATCATTAGATAAATTATTTTCTTGGCCATAGCCGATAATTTTGTAATCAGTA

CTAAGTGTATACGTATTTTTGCCACTTTTTCCTCAGATGATTAAAGTAAGTCAACAGCTTATTTTAGGAA

ACTGTAAAAGTAATAGGGAAAGAGATTTCACTATTTGCTTCATCAGTGGTAGGGGGGCGGTGACTGCAAC

TGTGTTAGCAGAAATTCACAGAGAATGGGGATTTAAGGTTAGCAGAGAAACTTGGAAAGTTCTGTGTTAG

GATCTTGCTGGCAGAATTAACTTTTTGCAAAAGTTTTATACACAGATATTTGTATTAAATTTGGAGCCAT

AGTCAGAAGACTCAGATCATAATTGGCTTATTTTTCTATTTCCGTAACTATTGTAATTTCCACTTTTGTA

ATAATTTTGATTTAAAATATAAATTTATTTATTTATTTTTTTAATAGTCAAAAATCTTTGCTGTTGTAGT

CTGCAACCTCTAAAATGATTGTGTTGCTTTTAGGATTGATCAGAAGAAACACTCCAAAAATTGAGATGAA

ATGTTGGTGCAGCCAGTTATAAGTAATATAGTTAACAAGCAAAAAAAGTGCTGCCACCTTTTATGATGAT

TTTCTAAATGGAGAAACATTTGGCTGCATCCACATAGACCTTTATGTTTTGTTTTCAGTTGAAAACTTGC

CTCCTTTGGCAACATTCGTAAATGAAGCAGAATTTTTTTTTCTCTTTTTTCCAAATATGTTAGTTTTGTT

CTTGTAAGATGTATCATGGGTATTGGTGCTGTGTAATGAACAACGAATTTTAATTAGCATGTGGTTCAGA

ATATACAATGTTAGGTTTTTAAAAAGTATCTTGATGGTTCTTTTCTATTTATAATTTCAGACTTTCATAA

AGTGTACCAAGAATTTCATAAATTTGTTTTCAGTGAACTGCTTTTTGCTATGGTAGGTCATTAAACACAG

CACTTACTCTTAAAAATGAAAATTTCTGATCATCTAGGATATTGACACATTTCAATTTGCAGTGTCTTTT

TGACTGGATATATTAACGTTCCTCTGAATGGCATTGATAGATGGTTCAGAAGAGAAACTCAATGAAATAA

AGAGAATATTTATTCATGGCGATTAATTAAATTATTTGCCTAACTTAAGAAAACTACTGTGCGTAACTCT

CAGTTTGTGCTTAACTCCATTTGACATGAGGTGACAGAAGAGAGTCTGAGTCTACCTGTGGAATATGTTG

GTTTATTTTCAGTGCTTGAAGATACATTCACAAATACTTGGTTTGGGAAGACACCGTTTAATTTTAAGTT

AACTTGCATGTTGTAAATGCGTTTTATGTTTAAATAAAGAGGAAAATTTTTTGAAA

>NM_017644.3 Homo sapiens kelch like family member 24 (KLHL24), transcript variant 7, mRNA

GTTGTGAGCTGCGGCAGAGACTGGTGGCTGGAGGAGACGCCGGCGCTGGAGAGTGCGCTGCGCCGCCCGC

CGCTGAGGGACCGCGGGGTTAGCCACTGCTGGCTGCTTCCAGTGTTCGCCGAGAGGTACCGGGGGTGACA

GCTCCGGGACCGGCCGAAAGGCGAGGAACCGGTGTGGAAATTAAAAGAACACACATATTTTGACTGGGGC

TTTGATCAACCAAATGCTAAAAAGCCACATAAAGAAGATCCCTAATAGTCATTTCTCAACAATTATATAG

TCAACTGATGTAACAATGGTACTAATATTGGGACGCAGACTAAACAGAGAGGATCTTGGGGTGCGTGATT

CCCCAGCAACTAAGCGAAAAGTTTTTGAAATGGACCCCAAATCTCTGACAGGTCATGAGTTTTTTGACTT

CTCTTCAGGATCATCCCATGCCGAAAACATACTCCAGATATTTAATGAATTTCGTGATAGCCGCTTATTC

ACAGATGTTATCATTTGTGTGGAAGGAAAAGAATTTCCTTGCCATAGAGCTGTGCTCTCAGCCTGTAGCA

GCTACTTCAGAGCTATGTTTTGTAATGACCACAGGGAAAGCCGAGAAATGTTGGTTGAGATCAATGGTAT

TTTAGCTGAAGCTATGGAATGTTTTTTGCAGTATGTTTATACTGGAAAGGTGAAGATCACTACAGAGAAT

GTACAGTATCTCTTTGAGACATCAAGCCTCTTTCAGATTAGTGTTCTCCGTGATGCATGTGCCAAGTTCT

TGGAGGAGCAACTTGATCCTTGTAATTGCTTAGGAATCCAGCGCTTTGCTGATACCCATTCACTCAAAAC

ACTCTTCACAAAATGCAAAAATTTTGCGTTACAGACTTTTGAGGATGTATCCCAGCACGAAGAATTTCTT

GAGCTTGACAAAGATGAACTTATTGATTATATTTGTAGTGATGAACTTGTTATTGGTAAAGAGGAGATGG

TTTTTGAAGCCGTCATGCGTTGGGTCTATCGTGCCGTTGATCTGAGAAGACCACTGTTACACGAGCTCCT

GACACATGTGAGACTCCCTCTGTTGCATCCCAACTACTTTGTTCAAACAGTTGAAGTGGACCAATTGATC

CAGAATTCTCCTGAGTGTTATCAGTTGTTGCATGAAGCAAGACGGTACCACATACTTGGGAATGAAATGA

TGTCCCCAAGGACTAGGCCACGCAGGTCCACTGGCTATTCTGAGGTGATAGTTGTCGTTGGAGGATGTGA

GCGAGTTGGAGGATTTAATCTTCCATACACTGAGTGCTACGATCCTGTAACAGGAGAATGGAAGTCTTTG

GCTAAGCTTCCAGAATTTACCAAATCAGAGTATGCAGTCTGTGCTCTAAGGAATGACATTCTTGTTTCAG

GTGGAAGAATCAACAGCCGTGATGTCTGGATTTATAACTCACAGTTAAATATTTGGATCAGAGTTGCCTC

TCTCAATAAAGGCAGATGGCGTCACAAAATGGCTGTCCTCCTTGGTAAAGTATATGTTGTCGGTGGCTAT

GATGGGCAAAACAGACTTAGCAGCGTAGAATGTTATGATTCCTTTTCAAATCGATGGACTGAAGTTGCTC

CCCTTAAGGAAGCCGTGAGTTCTCCTGCAGTGACTAGCTGTGTAGGCAAACTGTTTGTGATTGGTGGAGG

ACCTGATGATAATACTTGTTCTGATAAGGTTCAATCTTATGATCCAGAAACCAATTCTTGGCTACTTCGT

GCAGCTATCCCAATTGCCAAAAGGTGTATAACAGCTGTATCCCTAAACAACCTGATCTATGTTGCCGGTG

GACTGACCAAGGCAATATACTGTTACGATCCAGTTGAAGATTACTGGATGCACGTACAGAATACATTCAG

CCGTCAGGAAAACTGTGGTATGTCTGTGTGTAATGGTAAAATATATATCCTGGGCGGAAGACGGGAAAAT

GGAGAAGCCACAGACACTATTCTCTGTTATGATCCTGCAACAAGTATCATCACAGGGGTAGCTGCAATGC

CCAGGCCAGTGTCCTATCATGGCTGTGTGACTATTCATAGATACAATGAGAAATGCTTTAAACTCTGAAG

ACAGGATACCTCACCGAAGAAGCCACACTGATCCAAGATGGGAGGTTTTAAAAACTCTACAGTGGGAACT

TCACATATCTCCTTTGTGCCATATGCAAAAAATAGTAAAAATAATAATTTGGTGCCTTTCTCCTCAAAAT

ATCAATCTTTCAAACTATAATAAAGCCTTTCCTATAATTGAAAAAAAAAACTTTTTTGTTAAAGGTAATG

GTGGTTGTTACTTGGCCTTTGAAGAGTGTACCTTTGTAAGTATTTGTAAGAAGTCTATGTGAATTAGGAA

ATGTCTGTCTGCATACCTTTTAGGAGCGTGTGAATGGTGTCTTCACTTATTATGTATGTTTATCTGTATG

TATATTCCTTATTTTGTCATATATGTAGAGAAAATTGCATGACTTGAGGCATCATTTAGGTTGAAGAAGT

TAATGCTTAGGATGCATTCTAGGAGAAAAAATCAGTTTTAAAAACCTTTGTTGTTAACAAAGTATATCCA

GATTGGTTAATTTTATTGAAGGGTTTTTTTCTGTAATTGATAAAAATGTAATGACAACAATTCAGGCATC

ATAAAATACTGAACTATTGTGACTTTATTCTTAGAATTGCTGTCTTACATTAAACATGTTTTTAGGGGGA

AGTTAGGTAGGAGATAGAAAAATAAGTGCCCCTACAAGGGGGATTAAAATTACAAGTTAATTCCTAAGAG

AAAAATGGAATGGCCTTTGAAGGAAAAATGACCCACTATGGCTCTCAAAGTTTTTATGCATCATCTCTTC

AATCCTCTAAGAAAGCCTCTTTTCTTAACTTGATAAAGCAGTGGAAACCCATTTTGCAATATTGTTTTGT

GAAAAACAGGGACAGACAGCCAGGTACAGAGACTCACACCTGTACTCCCAACTACTCAGCAGGCTGGGGC

AGGAGGATTGCTTGAGCCCAGGAGTCTGAGGCTACAGTGAGCTATGAACGCACACGGCACCCTAGCCTGG

GCAACAGGTTGCGACACTGTCTCAAGAGAAAAGAAAAAGAAAAATAGGGATAGGTTTTCCTTCCTAGCCC

AGTAGAGTTTGACCTCATTAGTATGGTGCTTTGGGTGAGGACCTCTTCCTTGATTATCCCACTTTCTAGT

GAACAGCTAAAATTCCTGAGAGTCTCTACTGTTAAGGTACCTTTAATAGGATAAAGCAGGGACCACCTAT

CTCAGTGGGTCCATTTTTCTTTTAAAATTAGTTATCTGAAAAAACTTAGCAGTAGTTCCCATCTTTAAGG

TAAGTCTTTCATTTGGTCCCCATTGTGTAAAATACTAATCAACATTTTCAAGCTTCTGTACAACAGACTG

CTTTTGTCTAGATTTCTCAACTCCACTTTATAAAGCTTATCAGTTTTCAGAGAGGAATGTGAATTTTTTT

TCTAATGCAAATAAATGGATATGGCAGGAACTACAGCATAAGTGATTATTGTGATTCTGGGTGGACGGAT

ATAATTTACAACATTTAGGGATGTTCTAGGTAGCCTGCTGTAGTTTGACTTCCAGTCACTGTTGTCTTTC

ACATTATAATTTGTATATTTCTTGTGATAGAAGGGATGATGCAAATATGTAATTAAAGTGTCACCAGATT

TCTGTTAAAACCAAGGTTGAAATAAAAAGCCTAACATTGGTAAGCTACATTGTTTTCTCATTTTAGAATG

ATTCAGAGATTTCAGATAGACATTTTTTAAACTTTAATGCTTAGCTAGAATCTACATTCTGAGGAAAACT

CTAAAAAACTTAAAAATTTTTAGGGAATTTTTATTTTTCAAATCATAATTTTAAAATGATAGATACCATT

TTGTGATAACAACAATTCAGAAAACAATTTTCTATCCTCTTAGTTGAAAGAATGTAGGTACAGTTTGGAT

ACTTGTACTTTAATTTTAGAGTAAACATCTGCATTATACTCTTATAGATAATAGAATTATTTAGTTAAGA

AATTCTTTACAGTAAATGAGATAATGTGTGAAAAAGTATTTTGTAAATGCTGAGGATTCTACAAATGATA

GTTGTTATTTTCATGTGTATTTGTAAGATCATGTCCATTTCATGAATATAGGACTTCACATAAAAAAAGA

CTTTCTCAAGACAACTTTATATTCTAGTATTTTTCTGTTGTAAAAAGTATTAACTATTTACTTTTATTTT

GTTATACATTTATTTTAATATCCATGTGTTTATTATAGTAAATTTGAAATGAAATCCTGAAAAACAGAAT

TTTTTTAAACACAGACCTCACACCAATATTAATTTTTTCTCTACATAATTTAAAACTACATAAATTAAGT

ACTTAAAATTTATATTGAAGGCCACCAAGAACTTAGGTTGAATCTTAGAAAATTTAAATAACTATTTTTA

AAGTTACCCAACTTAATATTTTAATTTTTTAATATTTATCTTCCTTTACTAATTCTTGATAAATAATAGC

ATTAGACTTGATAAAATAAAAAAGAATTTTAGAGTAGAATTAATATATCAAAAGGGGTATATCAACCAAA

TTGGTGTCAGATTGTATTCATTCTCTCATCACATAAAGATTTTTCTTTTGATAGGTGATGCTCATATGAA

CCTTTGGTTTAGAATCTATATATGTACATGTGTATGTATGTAGATAGTATGGTTGTATACACACATATAT

ACCAAACACCATGAATTTTAGCAGTCTGTGATGATCAGCAAAAAAGCACATAAAGTAAAAATTAGTTGAC

CATGCTAAATTCAATTCTGGAATTTTTTTTTATTTGGGCATTTCTAGAACTTTTTACATTTGAAAGTACA

TGATGAGTATTAGTAACGATGACTTATGTATAATCAGAATCTTTATGACAATTTAGTTTTACAAGGTCAG

AAGAGATGAGTTTGCTAAACCCAGCTGTGATACCTCAGTTGGAAAGGGAATTCAAAGGTATGCTTTGTAG

AACAGAAAAGTATAGTTTTTTTTTCATGAACTTTAATCATTTTCTGTTTTTCCTCTATGTGAGTCAGCTA

CAAAAGTGGTCTAATTTTTACAACAGTAGAACTTCCTCCTTTTCTACTGTAATCTTCCCACTGACTTTAC

TGCACAGGTATGAAATACTAGTGTATTGGATCTTCAGTAACCTTTTTATTTCCTAGATGATTGAAATATA

GGTATTTACTCCATTTAAACCAGGTGATAAGATGATGTAAATACTCAGGGAGGGTATTAACTTGTTACTT

TTGCTCGTTTGGGGTGTAAAGTGCCATGACTGAATAATCTTCAATTCATGATTCTAGAGTAAGTTTAATT

TGGAAAAAGGGGCTTCACACATGGTGGTGGTTGAACATTGATTCTTTTATACTTAAAAAGATGAAAATGT

TTTGTGGACTGATACATTTTATCTTACTGAATATGAATTGTTTATGTATCTCTACTGTCAAATAGCCTTT

TTGAAACTCAGGAAAGACAAAGGTTCAATTACACCACTTTTGTCAATAAGCAAACCAGGTATTTTTTTTT

TCTCCTGTTGTCTGGATATGGCAATAGATTTTTTAAATTGCTGTGAGAACCCATATATGAAAAGAGAGGA

GTTGAATTGTGTGTGCCTTTTATGTCTTGAGATTTATATGTGGAAAAGACGACATCTACTTCAAACTGTA

TTTTTTTCGTTTTTTTTTTTTTTTTGGGGAAGGGGGGAGAACGGGGTCTTGCTCTGTCGCCCAGGCTGGAG

TGCAGTGGCGCGATCTCAGCTGACTGCAACCTCCACCTCCCGGGTTCAAGGGATTCTGCCTCAGCCTCCC

GAGTAGCTGAGACCACAGGTGCGTGCCACCACACCCGGCTAATTTTTTTGTATTTTTAGTAGAGACGGGG

TTTAGTAGAGACGGATCACTCCTGACCACGTGATCCGCCCACCTCGGCCTCCCAAAGTGCTGGGATTACA

GGCGTGAGCCACCACCCCCGGCCTGTATTTTCAGAGAGGAGAGCTTGGTGTTTTTGTGGTGCCAAGTGGT

AAGATAATGTCTCTTTGAGGCTTCCTATGGACTGCCTTTATTTTAGTAAACTCAAGACACCAGTTAACCT

CAACAGAGTTTTGGCCTTATTAGAATTTGTTGTGCATCTTATTGAAAGCCAGGTTTACATCACCTCACCC

CATTATTCTTTTTAGTTAAATAAATTTACCATGCCAAGTAACCAGAATGGAGCAAATTGGTTGATCTTTA

AGGCAGTAGGTTTGACTAGCTAGCTATCATTATTGTCACATCTAATGCTAGGCACCAGAAACCATTTGAG

CCAGGAGTGTGAATGAATAATTCCAGAGACACTTTAGACATTTTTTAATGTTTTATATGAACATTTTACA

TTTGTGTGATTGCCTTAGATATTAAATTTTCCTAGTGCTGATAAAAACAGCAACATTCATAACTTATTTT

ATATATTGTTCCAAAGAAAAGAATTTGTTTTAATGGTTTCAAAATAACTGCACCTGAATTTGTTTATGTG

CCTTAAGTTCTCTAGTGCTATTTCAACTTTTTTTTCAATCTAAATGAAGCTTACCTTAGATAAGGTTCAT

ATTTGTTTCCTATAGAGTAAATAAACTTCCCCTTCTTAAATTGTGTAATAAGCACCAACGTGTGGTTGCT

TGGCAGAATGAGAATGTTAAGGGAGATTGTTGGATGTTTGGAGTTTCATTATATTTTTTGTTTTTATTTT

TTGATACCTAGGTGCTTTTTAAAATATTCAGACAAATATCTATCTTACATTGATTAAACCCGTGTAAATT

CATTTGCAGTATCTACATCGAATGTCAAAAAAGTATACTTATTTTTGTTCCATACTTATGTACAATTTTT

TCCCTCTTCAGGCTTTTTCATTTACCTTTTTGAAAAAGCACTTACTCTCCCCTTCCCTATCACCCCTCCC

CCAAGGTTTCTTTATTTAAATTTTTATTGAGAGTTGTTGGAGCTCTAAGACAATACAAATTTAGAGTTGA

ACAAAAGTATAATCTGCTTTACAACTAGTATAGACCTAAGGTCATTTGCTTTCAATTAGAGGCTCCAGAG

TCTTCATAGTGGAAAGAATGCTTTGTATTTAATTGTTCTTAGTTAAGTTGTAGCACGTGAATACTTACTT

ACATGTTTTGTTTAAATATACTTCTTGCATAGTTTAATTTTTTAAAAGTTGTATCTAATAAAATGTCTTT

TAACCATTATTACTTGACTATATGGTTGTATTAAATTTTGTTTACGAAAAA

>NM_005561.4 Homo sapiens lysosomal associated membrane protein 1 (LAMP1), mRNA

ACGTGACAAGCGCTGCCGGCCGCGGTGTCTTCTTCGTGCCGGCGTCGCAGTGGCCGGGCCTCTTGCGTCT

GGTAACGCCGCTGTCTCTAACGCCAGCCCTTGGCGCCCGCGCCCCGCCACCGCAGCGCCCGGCAGTCCGC

GGCCCAACCGCCGCCCGCGCCCCCGCTCCCCGCACCGTACCCGGCCGCCTCGCGCCATGGCGGCCCCCGG

CAGCGCCCGGCGACCCCTGCTGCTGCTACTGCTGTTGCTGCTGCTCGGCCTCATGCATTGTGCGTCAGCA

GCAATGTTTATGGTGAAAAATGGCAACGGGACCGCGTGCATAATGGCCAACTTCTCTGCTGCCTTCTCAG

TGAACTACGACACCAAGAGTGGCCCTAAGAACATGACCTTTGACCTGCCATCAGATGCCACAGTGGTGCT

CAACCGCAGCTCCTGTGGAAAAGAGAACACTTCTGACCCCAGTCTCGTGATTGCTTTTGGAAGAGGACAT

ACACTCACTCTCAATTTCACGAGAAATGCAACACGTTACAGCGTCCAGCTCATGAGTTTTGTTTATAACT

TGTCAGACACACACCTTTTCCCCAATGCGAGCTCCAAAGAAATCAAGACTGTGGAATCTATAACTGACAT

CAGGGCAGATATAGATAAAAAATACAGATGTGTTAGTGGCACCCAGGTCCACATGAACAACGTGACCGTA

ACGCTCCATGATGCCACCATCCAGGCGTACCTTTCCAACAGCAGCTTCAGCCGGGGAGAGACACGCTGTG

AACAAGACAGGCCTTCCCCAACCACAGCGCCCCCTGCGCCACCCAGCCCCTCGCCCTCACCCGTGCCCAA

GAGCCCCTCTGTGGACAAGTACAACGTGAGCGGCACCAACGGGACCTGCCTGCTGGCCAGCATGGGGCTG

CAGCTGAACCTCACCTATGAGAGGAAGGACAACACGACGGTGACAAGGCTTCTCAACATCAACCCCAACA

AGACCTCGGCCAGCGGGAGCTGCGGCGCCCACCTGGTGACTCTGGAGCTGCACAGCGAGGGCACCACCGT

CCTGCTCTTCCAGTTCGGGATGAATGCAAGTTCTAGCCGGTTTTTCCTACAAGGAATCCAGTTGAATACA

ATTCTTCCTGACGCCAGAGACCCTGCCTTTAAAGCTGCCAACGGCTCCCTGCGAGCGCTGCAGGCCACAG

TCGGCAATTCCTACAAGTGCAACGCGGAGGAGCACGTCCGTGTCACGAAGGCGTTTTCAGTCAATATATT

CAAAGTGTGGGTCCAGGCTTTCAAGGTGGAAGGTGGCCAGTTTGGCTCTGTGGAGGAGTGTCTGCTGGAC

GAGAACAGCATGCTGATCCCCATCGCTGTGGGTGGTGCCCTGGCGGGGCTGGTCCTCATCGTCCTCATCG

CCTACCTCGTCGGCAGGAAGAGGAGTCACGCAGGCTACCAGACTATCTAGCCTGGTGCACGCAGGCACAG

CAGCTGCAGGGGCCTCTGTTCCTTTCTCTGGGCTTAGGGTCCTGTCGAAGGGGAGGCACACTTTCTGGCA

AACGTTTCTCAAATCTGCTTCATCCAATGTGAAGTTCATCTTGCAGCATTTACTATGCACAACAGAGTAA

CTATCGAAATGACGGTGTTAATTTTGCTAACTGGGTTAAATATTTTGCTAACTGGTTAAACATTAATATT

TACCAAAGTAGGATTTTGAGGGTGGGGGTGCTCTCTCTGAGGGGGTGGGGGTGCCGCTGTCTCTGAGGGG

TGGGGGTGCCGCTGTCTCTGAGGGGTGGGGGTGCCGCTCTCTCTGAGGGGGTGGGGGTGCCGCTTTCTCT

GAGGGGGTGGGGGTGCCGCTCTCTCTGAGGGGGTGGGGGTGCTGCTCTCTCCGAGGGGTGGAATGCCGCT

GTCTCTGAGGGGTGGGGGTGCCGCTCTAAATTGGCTCCATATCATTTGAGTTTAGGGTTCTGGTGTTTGG

TTTCTTCATTCTTTACTGCACTCAGATTTAAGCCTTACAAAGGGAAAGCCTCTGGCCGTCACACGTAGGA

CGCATGAAGGTCACTCGTGGTGAGGCTGACATGCTCACACATTACAACAGTAGAGAGGGAACATCCTAAG

ACAGAGGAACTCCAGAGATGAGTGTCTGGAGCGCTTCAGTTCAGCTTTAAAGGCCAGGACGGGCCACACG

TGGCTGGCGGCCTCGTTCCAGTGGCGGCACGTCCTTGGGCGTCTCTAATGTCTGCAGCTCAAGGGCTGGC

ACTTTTTTAAATATAAAAATGGGTGTTATTTTTATTTTTATTTGTAAAGTGATTTTTGGTCTTCTGTTGA

CATTCGGGGTGATCCTGTTCTGCGCTGTGTACAATGTGAGATCGGTGCGTTCTCCTGATGTTTTGCCGTG

GCTTGGGGATTGTACACGGGACCAGCTCACGTAATGCATTGCCTGTAACAATGTAATAAAAAGCCTCTTT

CTTTTTGGGGTGGGCCTTGTCCTTCTGTCAGCTAAAATGGGAGCTCATGAGAGAAGGACGTCAGGGAAAC

GGGGTTGAGGGTGGTCTCGGTGCAGAGAGAAGGGTGTCAGGGAAACGGGGGGTGAGGGTGGTCTTGGTGC

CAGACGTAGGGAATGGTGTTGGGAGTGGCCCGAGTGCCTGGCACAGTTGTCTGGTTCATTCATGTAACAT

GATAATTTTTAAATCATTAAAAAAATTACCTTTCATACAGA

>NM_001122606.1 Homo sapiens lysosomal associated membrane protein 2 (LAMP2), transcript variant C, mRNA

AAGAAAGAGCCCCGCCCCTAGTCTTATGACTCGCACTGAAGCGCCGATTCCTGGCTTTTGCAAGGCTGTG

GTCGGTGGTCATCAGTGCTCTTGACCCAGGTCCAGCGAGCCTTTTCCCTGGTGTTGCAGCTGTTGTTGTA

CCGCCGCCGTCGCCGCCGTCGCCGCCTGCTCTGCGGGGTCATGGTGTGCTTCCGCCTCTTCCCGGTTCCG

GGCTCAGGGCTCGTTCTGGTCTGCCTAGTCCTGGGAGCTGTGCGGTCTTATGCATTGGAACTTAATTTGA

CAGATTCAGAAAATGCCACTTGCCTTTATGCAAAATGGCAGATGAATTTCACAGTACGCTATGAAACTAC

AAATAAAACTTATAAAACTGTAACCATTTCAGACCATGGCACTGTGACATATAATGGAAGCATTTGTGGG

GATGATCAGAATGGTCCCAAAATAGCAGTGCAGTTCGGACCTGGCTTTTCCTGGATTGCGAATTTTACCA

AGGCAGCATCTACTTATTCAATTGACAGCGTCTCATTTTCCTACAACACTGGTGATAACACAACATTTCC

TGATGCTGAAGATAAAGGAATTCTTACTGTTGATGAACTTTTGGCCATCAGAATTCCATTGAATGACCTT

TTTAGATGCAATAGTTTATCAACTTTGGAAAAGAATGATGTTGTCCAACACTACTGGGATGTTCTTGTAC

AAGCTTTTGTCCAAAATGGCACAGTGAGCACAAATGAGTTCCTGTGTGATAAAGACAAAACTTCAACAGT

GGCACCCACCATACACACCACTGTGCCATCTCCTACTACAACACCTACTCCAAAGGAAAAACCAGAAGCT

GGAACCTATTCAGTTAATAATGGCAATGATACTTGTCTGCTGGCTACCATGGGGCTGCAGCTGAACATCA

CTCAGGATAAGGTTGCTTCAGTTATTAACATCAACCCCAATACAACTCACTCCACAGGCAGCTGCCGTTC

TCACACTGCTCTACTTAGACTCAATAGCAGCACCATTAAGTATCTAGACTTTGTCTTTGCTGTGAAAAAT

GAAAACCGATTTTATCTGAAGGAAGTGAACATCAGCATGTATTTGGTTAATGGCTCCGTTTTCAGCATTG

CAAATAACAATCTCAGCTACTGGGATGCCCCCCTGGGAAGTTCTTATATGTGCAACAAAGAGCAGACTGT

TTCAGTGTCTGGAGCATTTCAGATAAATACCTTTGATCTAAGGGTTCAGCCTTTCAATGTGACACAAGGA

AAGTATTCTACAGCTGAAGAATGTTCTGCTGACTCTGACCTCAACTTTCTTATTCCTGTTGCAGTGGGTG

TGGCCTTGGGCTTCCTTATAATTGTTGTCTTTATCTCTTATATGATTGGAAGAAGGAAAAGTCGTACTGG

TTATCAGTCTGTGTAATCAGTTAAATCTAGTGTTTGTTTGTTTTTTTCAATTAGAAGTTACGTTTCCATT

GGCTAAAAGCCAGGACATGCTGTGCAATAGATTGTTTAAGATATGCAGACTAACTTCAGTGAGTTCCTAG

CTAACTTGGGCATGAGTACACTTATTTAAGACAAAATATATTAGGACCAATTTTTTTCTGTTTTTTTTCT

TCCTTTGTTAAAGTATAATTAAAAGAAAAATTGTGGCTTAGAATTTTTTAAGTAAATAATGATTTTAAGC

CCCTGGATCCAATTATGAAAGCATTTTTGCTGATGTGTAATTTTATATGTTACAGTTACTTATATTTTAC

TACTTTGATGTTATTTGCAAAATCAAAGGTGTTAAAGAATTTAACTTGCTTCAGGAAATAAATTCAAGAA

CATAGTGGATTCATTTTCATTGGTGGCAGACACGAAATTTGGTTCATGATAAGACTTCCTTTCCCCACCT

CCTGATCAGCATTATTTAAATCTGTATTTTTCTGTTAGTTAAGAAAGAAATGGCTTCATGATATTGTATT

TAATAGCAAAAGTTTGGCTGTCTTCTTCATTACTGTTAATAGCTACTATATTTTAACAAGGAGATTTCTT

TTTTTGTTGTTGTTGTTCTAGAGTTTGGAATATACTGATTATCTCAGACTTGACATTTATACTGAAGGAT

GAAGTAAGACCTCCAGCTTTTTTTAAAAAAGGTGTTGATTTGGAACACCTGTATGGGTTATGGTTTATTA

AGGTTATGGTTTAGAAAGTTTTTTTCCCTCAGAGCCTTAACTTGTTAAGAAGGTTCATTTATCCTGCACT

GAAAACAAAAACTCTATATACTTTGTTTGTGTGCCTCCTGCACTCTCCCATTCCCTATGTGAATATGCTC

TAGTTGATATTTTTAATATATTGATTTCTTTTTTCTCACAGCAACAAGTGCTTACTCTAGAGGTTAGTGG

GCCCTGATATGTCATCAGTCAGATGCCTGCCTAGCCAAAGCTGGACTAAGATTATTCTGTACATTTGTTG

ATCTTGATATAGACTTATATCCCTGTAGGGACTGCTAATGGCTCCGGCTTCTGGAGTAAGGTACTGGAGA

CCACTCATCCCTGTGTCTGCTTGATTGGTTCAGCTGTTGAATTGCCCTTTTATTTGGAAGCAGTGTTGAA

GTTGTCTAGGGTTCAAATGGCTGCTTTGTACACCTGTCATTAGTATAAGGCAGATGTTTATTTTATCAAG

CTATTTTATCTCTACATTTAACTAAAAACAAAAGTTCCCAAAGATCTGCCTTCACTTCAGAAATTTTTTT

TGGATTAAAAAAATTAAGCCTGAACCTTAAATAAAGTGAGTTGGTTATTCATTCCAAGGATTAAGTCCCA

ATCTACCTCTCAGCACAATGCAGAAGCTCACCACTGTATTGCTGCCATTAACTCATGCCAGAACCCTTTG

CCAATAACTGGAATTACAAATTTTTGTTAAAGAAAATTTATCAAGATCTTTCTTTACTGCCTTCTCTATA

TGTACATCTCAAAAACATGTACATCTCAAAAACTGGAGTAGAAAGTTAGATTGCTCAACTACAACTCCTC

TAGAACTCTATAGCTCTGACATACAGATTCACACTCTCCTCTATTTGCTAAGTATGTAAAGAATGTTTTC

TTTTAAAATGTTCTCTTTTGAGAACAACTGCTTATTTGTTATAAAAGCATTTGGTTAAAATGATGTCATC

ATAAAAAACAGTGGCTTTGTTTCAATACATATTTTTGAGATGATTATCTAGAAGCCAGATTAATAAAATC

AGCTTGTGACCTTGCTAAGCATATAAACTGGAAATTCAGATACATTCAAAATTATGGGTTCATTTAAAAG

TGTTCTACCTTTTGGGTATGAGACTAATATCACTAATTCCTCAATAGTTATCATGGCTCTATCTTAATTA

ATTAGAAAATATGTGTGTTTAATTCTTTGAGAATTAAAATAGAGAATATTAACAGAGGGTTAAAAACTGC

TTCAACTCCAATAAGATAAAGGAAGCTCAAAATCTATGAGCTGAGTGTTCAATTAGCTTTGCCTACTGAG

TTCAATTTTATGTCAATACAACAGTGGATCAGACAGTACGACTTTGAACTGGTGAATGTAAACAATTGTT

TTTCACCTAAGCTGCTTTGGAAGAACTGATGCTTGCTGCTAACTAAAGTTTTGGATGTATCGATTTAGAG

AACCAATTAATACCTGCAAAATAAAGCATACTGTGGTACTTCTGTTTGATCTAGTATGTGTGATTTTAGA

TTGATGGATTAAAAATTAATAAAGATCATACATTCCATACCAAAAAAAAAAAAAAAA

>NM_181509.3 Homo sapiens microtubule associated protein 1 light chain 3 alpha (MAP1LC3A), transcript variant 2, mRNA

AACTGAAGTTCAGCCACCTGCCACTCCTGACTGCATGGAAGCCAGGTGCAAGGAGAAAGGATTTTGAGGA

GGGGACTCCATGGCTTCCGAGTTGCTGACTGACCCTCCACCTCAGAGGTAGTTCTGACACTGTCTCAGTT

TTGCAGATGAAGATGAGATTCTTCAGTTCTCCATGTGGAAAAGCAGCTGTGGACCCAGCCGACCGCTGTA

AGGAGGTACAGCAGATCCGCGACCAGCACCCCAGCAAAATCCCGGTGATCATCGAGCGCTACAAGGGTGA

GAAGCAGCTGCCCGTCCTGGACAAGACCAAGTTTTTGGTCCCGGACCATGTCAACATGAGCGAGTTGGTC

AAGATCATCCGGCGCCGCCTGCAGCTGAACCCCACGCAGGCCTTCTTCCTGCTGGTGAACCAGCACAGCA

TGGTGAGTGTGTCCACGCCCATCGCGGACATCTACGAGCAGGAGAAAGACGAGGACGGCTTCCTCTATAT

GGTCTACGCCTCCCAGGAAACCTTCGGCTTCTGAGCCAGCAGTAGGGGGGCTCGGCCTGGGAGTCGGGCG

GCCCCGGTCAGGCCCTGCCCAGAGAGCTCCTGGTTCCTGAACTGAGCTGCCTCTACCGTGGTGGGCTGGG

CAGGCATGTGCCCCCCTAGTCAGAGGGCACCAACCCACCTACTCTGCCCCTGGGTGGATCCTGGGCCGGT

CGTGTTAGGGTTGTCCCTCTGGGTGCTGGCTGGTGGGATGGGGGAGGGTGGGGAGCAGCTCCCAGCACCC

CTGCTGTGTGGTTCATCTTTTTTTTAGGCCCCTGCCTGTCTGCCCATCTGCCCCTCACCCACCCGAGGCT

CTGCCCACCGCCTGGACCTGCCCACCCCTGAAAGACTGGCCCCTGGCTCCCCGCCCCTCGGTCTCCACGT

GGTGTATGGATCTGTGGTCATTGTCCCTCTGCAGAATAAAGATTGCTCAGGCCTGCCTGGC

>NM_022818.5 Homo sapiens microtubule associated protein 1 light chain 3 beta (MAP1LC3B), mRNA

AGAGTCGGATTCGCCGCCGCAGCAGCCGCCGCCCCCGGGAGCCGCCGGGACCCTCGCGTCGTCGCCGCCG

CCGCCGCCCAGATCCCTGCACCATGCCGTCGGAGAAGACCTTCAAGCAGCGCCGCACCTTCGAACAAAGA

GTAGAAGATGTCCGACTTATTCGAGAGCAGCATCCAACCAAAATCCCGGTGATAATAGAACGATACAAGG

GTGAGAAGCAGCTTCCTGTTCTGGATAAAACAAAGTTCCTTGTACCTGACCATGTCAACATGAGTGAGCT

CATCAAGATAATTAGAAGGCGCTTACAGCTCAATGCTAATCAGGCCTTCTTCCTGTTGGTGAACGGACAC

AGCATGGTCAGCGTCTCCACACCAATCTCAGAGGTGTATGAGAGTGAGAAAGATGAAGATGGATTCCTGT

ACATGGTCTATGCCTCCCAGGAGACGTTCGGGATGAAATTGTCAGTGTAAAACCAGAAAAAATGCAGCTC

TTCTAGAATTGTTTAAACCCTTACCAAGGAAAAAAAAGGGATGTTACCAACTGAGATCGATCAGTTCATC

CAATCACAGATCATGAAACAGTAGTGTTCCCACCTAGGAGTGTTAGGAAGTTGTGTTTGTGTTTCAAGCA

GAAAAACTGAGCTCCAAGTGAGCACATTCAGCTTTGGAAACTATATTATTTAATGTAGGCTAGCTTGTTT

TCAAATTTTAAAAGTTTAAAAATAAAATACTTTGCATTCTAAGTTGCCAATAAAATAGACCTTCAAGTTA

TTTTAATGCTCTTTTCTCACTAATAGGAACTTGTAATTCCAGCAGTAATTTAAAGGCTTTCAGAGAGACC

CTGAGTCTTCTCTTCAGGTTCACAAAACCCGCCGCCTTTTTGGGTAGAAGTTTTCTACTCAGCTAGAGAG

ATCTCCCTAAGAGGATCTTTAGGCCTGAGTTGTGAAGCGCAACCCCCGCAAAACGCATTTGCCATCACAG

TTGGCACAAACGCAGGGTAAACGGGCTGTGTGAGAAAACGGCCCTGACTGTAAACTGCTGAAGGTCCCTG

ACTCCTAAGAGAACCACACCCAAAGTCCTCACTCTTGCAGGGGTAGACATTTCTGGTTTGGTTTGTTCTC

TAGATAGTTACACACATAAAGACACCACTCAAAAGGAAACTTGAATAATTTATAATTTTGATCGAGTTTC

TTAAAAGACCCTGGAGAAAGAGTGGCATTTCTTCTGTTTCAGGTTTTGTCTGAGTTCAAACTAGTGCCTG

TGTTGTTACGGAAAGCAGCAGTGTACCAGTGTCACTCTGGAGTACAGCGGGAGAAACACAAAATAGTATA

ACTGAAAACATTAACATTCAGACACACTCCCTTCTGCCTTCCGGCTTAAAGCTGTGGATGATCCACGTTT

TTGTTTTTTTAATGTTAAATGTGTAACTCAGTATTACTGAAAAGGTACCCACATTTTGAATAGTAGTTAT

CACTCTTAGGTCAGACAGCCATCAGAATTCTCCCACACCAAGTGCATGTCAGTTGTGGAGAAAACATAGC

AAAAAGAGCCGTACGCTCTTTACAGATACTAATGTCAAGAGTTAAACCTCCTCAGGTTCAACCTGTGATA

AAAGACTAGTGCTTCCCAGTACTTGCATGGGGTTCACTATTTATAGTTTTCTTGGGAGTATCACAGGAAA

ATCACAATTACACCACTTTAGACCCTATGTGTAGCAGGTCACAACTTACCCTTGTGTGTTTAGATGTGTA

TGAAATACCTGTATACGTTAGTGAAAGCTGTTTACTGTAACGGGGAAAACCAGATTCTTTGCATCTGGGC

CCTCTACTGATTGTTAAAGGAGTTCCTGTCACCTGCTCCCCCCACCCCCGCATGCGTCTGTCCACTTGGC

TAACTTTTAATATGTGTATTTTTACATTATGTATATTCTTAACTGGACTGTCTCGTTTAGACTGTATACA

TCATATCTGACATTATTGTAACTACCGTGTGATCAGTAAGATTCCTGTAAGAAATACTGCTTTTTAAGAA

AAAAAATAACATGCTGAGGGGTGACCTATATCCCATGTGAGTGGTCACTTTATTTATAGGATCTTTAAAA

CATTTTTAATGAACTAAGTTGAATAAAGGCACAATTAAAAACTGTCA

>NM_001004343.3 Homo sapiens microtubule associated protein 1 light chain 3 gamma (MAP1LC3C), mRNA

AGTTTGGAAGCAGCTGGAGGAATGAGTTAGGTTCCCGGTTGCGGGACAGTTTTTTTTTCTTTTTTAAAAC

AGACACAGCTACTGAGTGCAATGCCGCCTCCACAGAAAATCCCAAGCGTCAGACCCTTCAAGCAGAGGAA

AAGCTTGGCAATCAGACAAGAGGAAGTTGCTGGAATCCGGGCAAAGTTCCCCAACAAAATCCCGGTGGTA

GTGGAGCGCTACCCCAGGGAGACGTTCCTGCCCCCGCTGGACAAAACCAAGTTCCTGGTCCCGCAGGAGC

TGACCATGACCCAGTTCCTCAGCATCATCCGGAGCCGCATGGTCCTGAGAGCCACGGAAGCCTTTTACTT

GCTGGTGAACAACAAGAGCCTGGTCAGCATGAGCGCAACCATGGCAGAGATCTACAGAGACTACAAGGAT

GAGGATGGCTTCGTGTACATGACCTACGCCTCCCAGGAGACATTTGGCTGCCTGGAGTCAGCAGCCCCCA

GGGATGGGAGCAGCCTTGAGGACAGACCCTGCAATCCTCTCTAGCCCATGTCGGGAAGGATGTGTGCTCT

GACAGACGTGTCAGATGCTGGCAGAAGGGATTGGTTTTCTCCTGTGTATACAGTGGAGGAGTCTGAGAAG

CAGGGATGCCTGGGGTGATCAGCTCCAACCAGTGGCAGCAGAGTGGTGGCTCTTCCTAGTTTAGTTTTTG

TGCTCCGTGTTCTTGTGTCCTTCTAAGAAAAATGTGGGCTGCTCTTGAAAGTTATATAATTATCATTTTT

TTTTTTTTGAAACAGGGTCTTGCTCTATTGCCTAGGCTGGAGCACAGTGGCATGATCTTGGCTCACTGCA

ACCTCCGCCTCCTGGGTTCAAGTGATTCTTCTGCCTCCACCCCCCGAGTGGCTGGTATTACAAGCACATG

CCACCACACCAGGCTAATTTTTGTATTTTTAGTAGAGACGGGGTTTCACCATGTTGGCCAGGCTGGTCTC

GAACTCCTGACCTCAGGTGATCCACCCACCTCGGCCTCCCAAAGTGCTGGGATTACAGGCGTGAGCCACT

GCATCCAGCCATATAACCGTATTCTAAATAAGAAATGGTTGGCTTGTGTGATGGTTTTGTGTAATGAGCT

AGAGATAATATTTTAAGTGTCTTCTGTGGTATATGTGGGAGGGCCATTAAGGAGTGGGTTTCACTCCCTG

CATGTGGGCAGGTGTCC

>NM_001297555.2 Homo sapiens mitogen-activated protein kinase kinase 7 (MAP2K7), transcript variant 1, mRNA

AGTGCGGTGTTTGTCTGCCGGACTGACGGGCGGCCGGGCGGTGCGCGGCGGCGGTGGCGGCGGGGAAGAT

GGCGGCGTCCTCCCTGGAACAGAAGCTGTCCCGCCTGGAAGCAAAGCTGAAGCAGGAGAACCGGGAGGCC

CGGCGGAGGATCGACCTCAACCTGGATATCAGCCCCCAGCGGCCCAGGCCCATTATTGTGATCACTCTAA

GCCCTGCTCCTGCCCCGTCCCAACGAGCAGCCCTGCAGCTCCCGCTGGCCAACGATGGGGGCAGCCGCTC

GCCATCCTCAGAGAGCTCCCCGCAGCACCCCACGCCCCCCGCCCGGCCCCGCCACATGCTGGGGCTCCCG

TCAACCCTGTTCACACCCCGCAGCATGGAGAGCATTGAGATTGACCAGAAGCTGCAGGAGATCATGAAGC

AGACGGGCTACCTGACCATCGGGGGCCAGCGCTACCAGGCAGAAATCAACGACCTGGAGAACTTGGGCGA

GATGGGCAGCGGCACCTGCGGCCAGGTGTGGAAGATGCGCTTCCGGAAGACCGGCCACGTCATTGCCGTT

AAGCAAATGCGGCGCTCCGGGAACAAGGAGGAGAACAAGCGCATCCTCATGGACCTGGATGTGGTGCTGA

AGAGCCACGACTGCCCCTACATCGTGCAGTGCTTTGGGACGTTCATCACCAACACGGACGTCTTCATCGC

CATGGAGCTCATGGGCACCTGCGCTGAGAAGCTCAAGAAGCGGATGCAGGGCCCCATCCCCGAGCGCATT

CTGGGCAAGATGACAGTGGCGATTGTGAAGGCGCTGTACTACCTGAAGGAGAAGCACGGTGTCATCCACC

GCGACGTCAAGCCCTCCAACATCCTGCTGGACGAGCGGGGCCAGATCAAGCTCTGCGACTTCGGCATCAG

CGGCCGCCTGGTGGACTCCAAAGCCAAGACGCGGAGCGCCGGCTGTGCCGCCTACATGGCACCCGAGCGC

ATTGACCCCCCAGACCCCACCAAGCCGGACTATGACATCCGGGCCGACGTATGGAGCCTGGGCATCTCGT

TGGTGGAGCTGGCAACAGGACAGTTTCCCTACAAGAACTGCAAGACGGACTTTGAGGTCCTCACCAAAGT

CCTACAGGAAGAGCCCCCGCTTCTGCCCGGACACATGGGCTTCTCGGGGGACTTCCAGTCCTTCGTCAAA

GACTGCCTTACTAAAGATCACAGGAAGAGACCAAAGTATAATAAGCTACTTGAACACAGCTTCATCAAGC

GCTACGAGACGCTGGAGGTGGACGTGGCGTCCTGGTTCAAGGATGTCATGGCGAAGACTGAGTCACCGCG

GACTAGCGGCGTCCTGAGCCAGCCCCACCTGCCCTTCTTCAGGTAGCTGCTTGGCGGCGGCCAGCCCCAC

AGGGGGCCAGGGGCATGGCCACAGGCCCCCCTCCCCACTTGGCCACCCAGCTGCCTGCCAGGGGAGACCT

GGGACCTGGACGGCCACCTAGGACTGAGGACAGAGAGTGGGGGGTGCCCACCCACCCCCCCGCCCCGGG

CCTACCAAGCCCCCGCCCTTCCCACCCCGGGGTCAGCCGGCCGTGTGCGTCCCCCGACAGACACTGTGAA

CGGAAGACAGCAGGCCGCGATCAGAGTCGCTGTTCATTCAGCCGCAGCCTCTGGGCCGGGGCGGCCCCCA

GGGGCCAGGAGAGAGCCCTGGAGTCCCGCAGCCACCATGCACGCTCCCAGCGTGCTGTGTCCTTCGCCAC

TCCCACGCGCCCGTTCCTCTTCCGTCGCCCTCTGTCCCCTGCTCTACCTCTCTGTCCTTGTCTGGCTCTC

CCGTCACCCTCCCTGCCTCTGTCTCTCTTCTGGCCTGAGCCTGGGCCCAGCCACCTCCTGACGGGTCCCC

TGGGTCTGCATAGGTCTCCCATGGCGCAATGAGTCAGTGGCCCCCAGCCAGGCAGTGTGGGCATTGCCAC

TGCGGCTGGACGGGGCTGCGCGCTCGCGCTCTCTCTCTCTCTCTCTCTCTCTTTGATCTCAGGGGGTC

CTTTTTGGAGTTTATTGTATTTTATTGTACTTGGTGGGGTGTTTGGGGTGGGGGCGGAGGAGAGCTTGTT

CTCGTGGGGTTGTCGGTACCTTCAGAAACTTTTACCAAAGTCACGATTAGCTGCTTGTGGTGGGGCCCCA

ACCGCCCTCGGGCACTGGGGAGCTGGGCTGGGGCTGCTGCTCTGGGGTCTCCGGGGGCCACAGCTTGGGG

TGAGTTGAAGACCTCAGGGGATGTGGAGGGGTCTGCGGGGCCCTGGCCGCACAGGATGGCCTTCAGGGAA

GGTGGTCTTGGGGCATGGTGCAGAGCAGGTGACCGGAGGGAATCGGTGACGGAGCGGGGCCAAGGGAGGG

GTCCGGAGGGAGTCAGGGATGGAGGGCAGAGGGAGTGGATGTGGGGGTTTGAGGACGTGTGACAAGCTCC

AGCAGGGGTGGGGGCCGGGCTGAGGGTGGGGGTGCGAGGTGGTCACTCCCATCGTGCCCCTGGCCGTCCC

TCCACTCACCCACACCTGGCCCAGTCCACGTTGAGGTCCAGGACTGGGAAGGACCGGGTGAGTGCACCGG

GGACCCAGGCCAGGTGCCCCCCGGAGCCTGCTGGGGTGGCCAGAGCAGGAGGGGGTGTGTTTCCTTTTTG

TGGGTGTTGCATGCAAATCAAGTGGACAAGAAAAAATAACAAAACAAAAAACAAGAAAAAAAAAACACAA

AACCCCGTAAAATCACAAAGAAAATCCAACACCAAAGGCGCAGAAGCCGGCTGGCCGTGGTGGGGGCAGC

GTAGGCGTAGCATCCCTCTCCTCTCACTTAGCCTGTTGACTCTTGTTATTATCATGATATTCACAAAACG

CCGCATGTTTAAAAAGTCATAGATGTCATCTTCTCTCTGCCCCCAGGGAGGAAAGCCACCTTCTCTTGCC

CCTTGGCCCCTTTGTCAGGGGCCAGGGGTCTGCCGGGTGGGGGTGCCAACAGGCCTGGCCCTTTCCTCCC

CTGCATCCAGCCATGGGGGCCTCTGCGATTGCCGGAAGGTTGCATGGCTGGTCCCAGGGCCAGCACAGGC

CCGAGGCCGGGCTGCCTGGTTTTATTTTTATTTAACTTTATTTTCTGTTTTATGAGTGTGTGTCCGCCCA

CCCCCACCCCCTTCAGTGTTAAGTGGGGAGCCCTGGGGGAGTCTCTCCTGCCTCCCAGCCTCTCCCAAGA

CCTCCCCCCTCGTCACCAGCCATCCCTCTGGACCAGGCAGAGGGCGGACCGGGTGGGCAGGGGCCTGAGG

GTGGCTCGGGCCAGCCCACCAGCCAATGGACCCCTCCTCAGGCCGCCAGTGTCGCCCTGCCCCTTTTTAA

AACAAAATGCCCTCGTTTGTAAACCCTTAGACGCTTGAGAATAAACCCCTTCCTTTTCTTCCA

>NM_002745.5 Homo sapiens mitogen-activated protein kinase 1 (MAPK1), transcript variant 1, mRNA

AGTCTGGCAGGCAGGCAGGCAATCGGTCCGAGTGGCTGTCGGCTCTTCAGCTCTCCCGCTCGGCGTCTTC

CTTCCTCCTCCCGGTCAGCGTCGGCGGCTGCACCGGCGGCGGCGCAGTCCCTGCGGGAGGGGCGACAAGA

GCTGAGCGGCGGCCGCCGAGCGTCGAGCTCAGCGCGGCGGAGGCGGCGGCGGCCCGGCAGCCAACATGGC

GGCGGCGGCGGCGGCGGGCGCGGGCCCGGAGATGGTCCGCGGGCAGGTGTTCGACGTGGGGCCGCGCTAC

ACCAACCTCTCGTACATCGGCGAGGGCGCCTACGGCATGGTGTGCTCTGCTTATGATAATGTCAACAAAG

TTCGAGTAGCTATCAAGAAAATCAGCCCCTTTGAGCACCAGACCTACTGCCAGAGAACCCTGAGGGAGAT

AAAAATCTTACTGCGCTTCAGACATGAGAACATCATTGGAATCAATGACATTATTCGAGCACCAACCATC

GAGCAAATGAAAGATGTATATATAGTACAGGACCTCATGGAAACAGATCTTTACAAGCTCTTGAAGACAC

AACACCTCAGCAATGACCATATCTGCTATTTTCTCTACCAGATCCTCAGAGGGTTAAAATATATCCATTC

AGCTAACGTTCTGCACCGTGACCTCAAGCCTTCCAACCTGCTGCTCAACACCACCTGTGATCTCAAGATC

TGTGACTTTGGCCTGGCCCGTGTTGCAGATCCAGACCATGATCACACAGGGTTCCTGACAGAATATGTGG

CCACACGTTGGTACAGGGCTCCAGAAATTATGTTGAATTCCAAGGGCTACACCAAGTCCATTGATATTTG

GTCTGTAGGCTGCATTCTGGCAGAAATGCTTTCTAACAGGCCCATCTTTCCAGGGAAGCATTATCTTGAC

CAGCTGAACCACATTTTGGGTATTCTTGGATCCCCATCACAAGAAGACCTGAATTGTATAATAAATTTAA

AAGCTAGGAACTATTTGCTTTCTCTTCCACACAAAAATAAGGTGCCATGGAACAGGCTGTTCCCAAATGC

TGACTCCAAAGCTCTGGACTTATTGGACAAAATGTTGACATTCAACCCACACAAGAGGATTGAAGTAGAA

CAGGCTCTGGCCCACCCATATCTGGAGCAGTATTACGACCCGAGTGACGAGCCCATCGCCGAAGCACCAT

TCAAGTTCGACATGGAATTGGATGACTTGCCTAAGGAAAAGCTCAAAGAACTAATTTTTGAAGAGACTGC

TAGATTCCAGCCAGGATACAGATCTTAAATTTGTCAGGACAAGGGCTCAGAGGACTGGACGTGCTCAGAC

ATCGGTGTTCTTCTTCCCAGTTCTTGACCCCTGGTCCTGTCTCCAGCCCGTCTTGGCTTATCCACTTTGA

CTCCTTTGAGCCGTTTGGAGGGGCGGTTTCTGGTAGTTGTGGCTTTTATGCTTTCAAAGAATTTCTTCAG

TCCAGAGAATTCCTCCTGGCAGCCCTGTGTGTGTCACCCATTGGTGACCTGCGGCAGTATGTACTTCAGT

GCACCTACTGCTTACTGTTGCTTTAGTCACTAATTGCTTTCTGGTTTGAAAGATGCAGTGGTTCCTCCCT

CTCCTGAATCCTTTTCTACATGATGCCCTGCTGACCATGCAGCCGCACCAGAGAGAGATTCTTCCCCAAT

TGGCTCTAGTCACTGGCATCTCACTTTATGATAGGGAAGGCTACTACCTAGGGCACTTTAAGTCAGTGAC

AGCCCCTTATTTGCACTTCACCTTTTGACCATAACTGTTTCCCCAGAGCAGGAGCTTGTGGAAATACCTT

GGCTGATGTTGCAGCCTGCAGCAAGTGCTTCCGTCTCCGGAATCCTTGGGGAGCACTTGTCCACGTCTTT

TCTCATATCATGGTAGTCACTAACATATATAAGGTATGTGCTATTGGCCCAGCTTTTAGAAAATGCAGTC

ATTTTTCTAAATAAAAAGGAAGTACTGCACCCAGCAGTGTCACTCTGTAGTTACTGTGGTCACTTGTACC

ATATAGAGGTGTAACACTTGTCAAGAAGCGTTATGTGCAGTACTTAATGTTTGTAAGACTTACAAAAAAA

GATTTAAAGTGGCAGCTTCACTCGACATTTGGTGAGAGAAGTACAAAGGTTGCAGTGCTGAGCTGTGGGC

GGTTTCTGGGGATGTCCCAGGGTGGAACTCCACATGCTGGTGCATATACGCCCTTGAGCTACTTCAAATG

TGGGTGTTTCAGTAACCACGTTCCATGCCTGAGGATTTAGCAGAGAGGAACACTGCGTCTTTAAATGAGA

AAGTATACAATTCTTTTTCCTTCTACAGCATGTCAGCATCTCAAGTTCATTTTTCAACCTACAGTATAAC

AATTTGTAATAAAGCCTCCAGGAGCTCATGACGTGAAGCACTGTTCTGTCCTCAAGTACTCAAATATTTC

TGATACTGCTGAGTCAGACTGTCAGAAAAAGCTAGCACTAACTCGTGTTTGGAGCTCTATCCATATTTTA

CTGATCTCTTTAAGTATTTGTTCCTGCCACTGTGTACTGTGGAGTTGACTCGGTGTTCTGTCCCAGTGCG

GTGCCTCCTCTTGACTTCCCCACTGCTCTCTGTGGTGAGAAATTTGCCTTGTTCAATAATTACTGTACCC

TCGCATGACTGTTACAGCTTTCTGTGCAGAGATGACTGTCCAAGTGCCACATGCCTACGATTGAAATGAA

AACTCTATTGTTACCTCTGAGTTGTGTTCCACGGAAAATGCTATCCAGCAGATCATTTAGGAAAAATAAT

TCTATTTTTAGCTTTTCATTTCTCAGCTGTCCTTTTTTCTTGTTTGATTTTTGACAGCAATGGAGAATGG

GTTATATAAAGACTGCCTGCTAATATGAACAGAAATGCATTTGTAATTCATGAAAATAAATGTACATCTT

CTATCTTCACATTCATGTTAAGATTCAGTGTTGCTTTCCTCTGGATCAGCGTGTCTGAATGGACAGTCAG

GTTCAGGTTGTGCTGAACACAGAAATGCTCACAGGCCTCACTTTGCCGCCCAGGCACTGGCCCAGCACTT

GGATTTACATAAGATGAGTTAGAAAGGTACTTCTGTAGGGTCCTTTTTACCTCTGCTCGGCAGAGAATCG

ATGCTGTCATGTTCCTTTATTCACAATCTTAGGTCTCAAATATTCTGTCAAACCCTAACAAAGAAGCCCC

GACATCTCAGGTTGGATTCCCTGGTTCTCTCTAAAGAGGGCCTGCCCTTGTGCCCCAGAGGTGCTGCTGG

GCACAGCCAAGAGTTGGGAAGGGCCGCCCCACAGTACGCAGTCCTCACCACCCAGCCCAGGGTGCTCACG

CTCACCACTCCTGTGGCTGAGGAAGGATAGCTGGCTCATCCTCGGAAAACAGACCCACATCTCTATTCTT

GCCCTGAAATACGCGCTTTTCACTTGCGTGCTCAGAGCTGCCGTCTGAAGGTCCACACAGCATTGACGGG

ACACAGAAATGTGACTGTTACCGGATAACACTGATTAGTCAGTTTTCATTTATAAAAAAGCATTGACAGT

TTTATTACTCTTGTTTCTTTTTAAATGGAAAGTTACTATTATAAGGTTAATTTGGAGTCCTCTTCTAAAT

AGAAAACCATATCCTTGGCTACTAACATCTGGAGACTGTGAGCTCCTTCCCATTCCCCTTCCTGGTACTG

TGGAGTCAGATTGGCATGAAACCACTAACTTCATTCTAGAATCATTGTAGCCATAAGTTGTGTGCTTTTT

ATTAATCATGCCAAACATAATGTAACTGGGCAGAGAATGGTCCTAACCAAGGTACCTATGAAAAGCGCTA

GCTATCATGTGTAGTAGATGCATCATTTTGGCTCTTCTTACATTTGTAAAAATGTACAGATTAGGTCATC

TTAATTCATATTAGTGACACGGAACAGCACCTCCACTATTTGTATGTTCAAATAAGCTTTCAGACTAATA

GCTTTTTTGGTGTCTAAAATGTAAGCAAAAAATTCCTGCTGAAACATTCCAGTCCTTTCATTTAGTATAA

AAGAAATACTGAACAAGCCAGTGGGATGGAATTGAAAGAACTAATCATGAGGACTCTGTCCTGACACAGG

TCCTCAAAGCTAGCAGAGATACGCAGACATTGTGGCATCTGGGTAGAAGAATACTGTATTGTGTGTGCAG

TGCACAGTGTGTGGTGTGTGCACACTCATTCCTTCTGCTCTTGGGCACAGGCAGTGGGTGTAGAGGTAAC

CAGTAGCTTTGAGAAGCTACATGTAGCTCACCAGTGGTTTTCTCTAAGGAATCACAAAAGTAAACTACCC

AACCACATGCCACGTAATATTTCAGCCATTCAGAGGAAACTGTTTTCTCTTTATTTGCTTATATGTTAAT

ATGGTTTTTAAATTGGTAACTTTTATATAGTATGGTAACAGTATGTTAATACACACATACATACGCACAC

ATGCTTTGGGTCCTTCCATAATACTTTTATATTTGTAAATCAATGTTTTGGAGCAATCCCAAGTTTAAGG

GAAATATTTTTGTAAATGTAATGGTTTTGAAAATCTGAGCAATCCTTTTGCTTATACATTTTTAAAGCAT

TTGTGCTTTAAAATTGTTATGCTGGTGTTTGAAACATGATACTCCTGTGGTGCAGATGAGAAGCTATAAC

AGTGAATATGTGGTTTCTCTTACGTCATCCACCTTGACATGATGGGTCAGAAACAAATGGAAATCCAGAG

CAAGTCCTCCAGGGTTGCACCAGGTTTACCTAAAGCTTGTTGCCTTTTCTTGTGCTGTTTATGCGTGTAG

AGCACTCAAGAAAGTTCTGAAACTGCTTTGTATCTGCTTTGTACTGTTGGTGCCTTCTTGGTATTGTACC

CCAAAATTCTGCATAGATTATTTAGTATAATGGTAAGTTAAAAAATGTTAAAGGAAGATTTTATTAAGAA

TCTGAATGTTTATTCATTATATTGTTACAATTTAACATTAACATTTATTTGTGGTATTTGTGATTTGGTT

AATCTGTATAAAAATTGTAAGTAGAAAGGTTTATATTTCATCTTAATTCTTTTGATGTTGTAAACGTACT

TTTTAAAAGATGGATTATTTGAATGTTTATGGCACCTGACTTGTAAAAAAAAAAAACTACAAAAAAATCC

TTAGAATCATTAAATTGTGTCCCTGTATTACCAAAATAACACAGCACCGTGCATGTATAGTTTAATTGCA

GTTTCATCTGTGAAAACGTGAAATTGTCTAGTCCTTCGTTATGTTCCCCAGATGTCTTCCAGATTTGCTC

TGCATGTGGTAACTTGTGTTAGGGCTGTGAGCTGTTCCTCGAGTTGAATGGGGATGTCAGTGCTCCTAGG

GTTCTCCAGGTGGTTCTTCAGACCTTCACCTGTGGGGGGGGGGGGTAGGCGGTGCCCACGCCCATCTCCTC

ATCCTCCTGAACTTCTGCAACCCCACTGCTGGGCAGACATCCTGGGCAACCCCTTTTTTCAGAGCAAGAA

GTCATAAAGATAGGATTTCTTGGACATTTGGTTCTTATCAATATTGGGCATTATGTAATGACTTATTTAC

AAAACAAAGATACTGGAAAATGTTTTGGATGTGGTGTTATGGAAAGAGCACAGGCCTTGGACCCATCCAG

CTGGGTTCAGAACTACCCCCTGCTTATAACTGCGGCTGGCTGTGGGCCAGTCATTCTGCGTCTCTGCTTT

CTTCCTCTGCTTCAGACTGTCAGCTGTAAAGTGGAAGCAATATTACTTGCCTTGTATATGGTAAAGATTA

TAAAAATACATTTCAACTGTTCAGCATAGTACTTCAAAGCAAGTACTCAGTAAATAGCAAGTCTTTTTAA

A

>NM_001040056.3 Homo sapiens mitogen-activated protein kinase 3 (MAPK3), transcript variant 2, mRNA

GAGGAGTGGAGATGGCGGCGGCGGCGGCTCAGGGGGGCGGGGGCGGGGAGCCCCGTAGAACCGAGGGGGT

CGGCCCGGGGGTCCCGGGGGAGGTGGAGATGGTGAAGGGGCAGCCGTTCGACGTGGGCCCGCGCTACACG

CAGTTGCAGTACATCGGCGAGGGCGCGTACGGCATGGTCAGCTCGGCCTATGACCACGTGCGCAAGACTC

GCGTGGCCATCAAGAAGATCAGCCCCTTCGAACATCAGACCTACTGCCAGCGCACGCTCCGGGAGATCCA

GATCCTGCTGCGCTTCCGCCATGAGAATGTCATCGGCATCCGAGACATTCTGCGGGCGTCCACCCTGGAA

GCCATGAGAGATGTCTACATTGTGCAGGACCTGATGGAGACTGACCTGTACAAGTTGCTGAAAAGCCAGC

AGCTGAGCAATGACCATATCTGCTACTTCCTCTACCAGATCCTGCGGGGCCTCAAGTACATCCACTCCGC

CAACGTGCTCCACCGAGATCTAAAGCCCTCCAACCTGCTCATCAACACCACCTGCGACCTTAAGATTTGT

GATTTCGGCCTGGCCCGGATTGCCGATCCTGAGCATGACCACACCGGCTTCCTGACGGAGTATGTGGCTA

CGCGCTGGTACCGGGCCCCAGAGATCATGCTGAACTCCAAGGGCTATACCAAGTCCATCGACATCTGGTC

TGTGGGCTGCATTCTGGCTGAGATGCTCTCTAACCGGCCCATCTTCCCTGGCAAGCACTACCTGGATCAG

CTCAACCACATTCTGGGCATCCTGGGCTCCCCATCCCAGGAGGACCTGAATTGTATCATCAACATGAAGG

CCCGAAACTACCTACAGTCTCTGCCCTCCAAGACCAAGGTGGCTTGGGCCAAGCTTTTCCCCAAGTCAGA

CTCCAAAGCCCTTGACCTGCTGGACCGGATGTTAACCTTTAACCCCAATAAACGGATCACAGTGGAGGAA

GCGCTGGCTCACCCCTACCTGGAGCAGTACTATGACCCGACGGATGAGGTGGGCCAGTCCCCAGCAGCAG

TGGGGCTGGGGGCAGGGGAGCAGGGGGGCACGTAG

>NM_001278547.2 Homo sapiens mitogen-activated protein kinase 8 (MAPK8), transcript variant JNK1-b2, mRNA

GTTACTCAGCCGAGCGGCCGAGGCCGGACGACGCGGCTTGGATTGCGGAGCCGCGAGCAGCGCTGGGTAA

CGGCCGCGGCGACCACCCCGGACGGCCCCTGTCCCCGCTGGCGGGCTTCCCTGTCGCCGTTCGCTGCGCT

GCCGGCTTCTTGGTGAATTTTTGGATGAAGCCATTAAATTAATTGCTTGCCATCATGAGCAGAAGCAAGC

GTGACAACAATTTTTATAGTGTAGAGATTGGAGATTCTACATTCACAGTCCTGAAACGATATCAGAATTT

AAAACCTATAGGCTCAGGAGCTCAAGGAATAGTATGCGCAGCTTATGATGCCATTCTTGAAAGAAATGTT

GCAATCAAGAAGCTAAGCCGACCATTTCAGAATCAGACTCATGCCAAGCGGGCCTACAGAGAGCTAGTTC

TTATGAAATGTGTTAATCACAAAAATATAATTGGCCTTTTGAATGTTTTCACACCACAGAAATCCCTAGA

AGAATTTCAAGATGTTTACATAGTCATGGAGCTCATGGATGCAAATCTTTGCCAAGTGATTCAGATGGAG

CTAGATCATGAAAGAATGTCCTACCTTCTCTATCAGATGCTGTGTGGAATCAAGCACCTTCATTCTGCTG

GAATTATTCATCGGGACTTAAAGCCCAGTAATATAGTAGTAAAATCTGATTGCACTTTGAAGATTCTTGA

CTTCGGTCTGGCCAGGACTGCAGGAACGAGTTTTATGATGACGCCTTATGTAGTGACTCGCTACTACAGA

GCACCCGAGGTCATCCTTGGCATGGGCTACAAGGAAAACGTTGACATTTGGTCAGTTGGGTGCATCATGG

GAGAAATGATCAAAGGTGGTGTTTTGTTCCCAGGTACAGATCATATTGATCAGTGGAATAAAGTTATTGA

ACAGCTTGGAACACCATGTCCTGAATTCATGAAGAAACTGCAACCAACAGTAAGGACTTACGTTGAAAAC

AGACCTAAATATGCTGGATATAGCTTTGAGAAACTCTTCCCTGATGTCCTTTTCCCAGCTGACTCAGAAC

ACAACAAACTTAAAGCCAGTCAGGCAAGGGATTTGTTATCCAAAATGCTGGTAATAGATGCATCTAAAAG

GATCTCTGTAGATGAAGCTCTCCAACACCCGTACATCAATGTCTGGTATGATCCTTCTGAAGCAGAAGCT

CCACCACCAAAGATCCCTGACAAGCAGTTAGATGAAAGGGAACACACAATAGAAGAGTGGAAAGAATTGA

TATATAAGGAAGTTATGGACTTGGAGGAGAGAACCAAGAATGGAGTTATACGGGGGCAGCCCTCTCCTTT

AGGTGCAGCAGTGATCAATGGCTCTCAGCATCCATCATCATCGTCGTCTGTCAATGATGTGTCTTCAATG

TCAACAGATCCGACTTTGGCCTCTGATACAGACAGCAGTCTAGAAGCAGCAGCTGGGCCTCTGGGCTGCT

GTAGATGACTACTTGGGCCATCGGGGGGTGGGAGGGATGGGGAGTCGGTTAGTCATTGATAGAACTACTT

TGAAAACAATTCAGTGGTCTTATTTTTGGGTGATTTTTCAAAAAATGTAGAATTCATTTTGTAGTAAAGT

AGTTTATTTTTTTTAATTTCAAGTGATGTAATTTAAAACCTAAGTTGTGTTTCAAAACAGCAACAAAACT

GTATTGTATTTTTTTTGCTGTAATTAACTGTATAATGTAAACCTAATTATTTTATCATGGTTTAAATTTT

TTGCATATTTGCTTTATCTTATGCTGCTGATTTTTTTAACTGAATTTGTAAGATTTTGTTTATCAAAGCA

ACTATTATGTGGTGACTTGCCTATATCATGAATTATTTAAGATTTTTATAGTTTTTTTTAATTAGAATTT

ATTTCAGATGTTTTGTTCATGATACTATCCTTCAGGGTTATGTGCTTATCAATGAAATAACCCCAGAGGA

GTGAGGGAAAATAACTTGTAGCCAGTTATATTCAGGAATAACTACTGTAAATGATGAACGTGTTAGGAGA

CCTCCAATATTTGCTACTTGCCAATCCTAATTTAGTTACAAGAATTGGTAGGCAATCCTACTTAATTTTG

GCAAAAGCCCCGTCATCTAAATGGCAGAATAACTCAGAGCATGTCTTTGAAGATGCTGGGCGTCTACCAC

CACCTTATGTCCCCACCCTACCCAACAAAAATAAGTAAAAAGAATATGGTGTATTCTACAAATTTGTGGC

ATGCTCAAAGTTTATGATCACATAAAGGCAAGAGGATACTTCATGAATAATACATTTCAATGCAAATAAA

CAGATGGTTCACTTCTACTAGCTATGAGCCTGTTTTTGTATACACTGAGTTAATCTACTCAGGCTGTAGG

TCCCAGCAATGTTCTAGAGTCTGGTCTTTCCCTTTCCTGCAGCTTCGGGTCCTTGGACCTTTCCTGTTTC

CTATTACTTGGAGTGTCTGTCAGTTGAGCACCAGTTGTTCTGGTGTTTCATTTGATTCTACTTGTAGCAT

AATCATTTATACGAGCTATTGGGAGGTTCCAAACCCTACCTAGATTTGTGTAGGTGATGTATCAAATGAG

CAATATACCGTTCATCTGAAAATAGTAGCACACAGCCATATATAGGATATCATTTTCTAAGGACTGTTTC

TTCACATTGAGCAGAGCAGGCATAAATGGTGGTTATTTAGTCTAAGTCTTTTATTTTTTTATACCTGATT

TTCAACATAACACGCAATGTGGATGTCGAGTAGTGTTAAGAATGGTGCTGCTCCTGACAAGTGTATGTTA

ACTGTTTACATTTTCTATCTGTAGAATTATTTCTCTATTACTGAACTTTTCCTAAGTAAAATGTCTTTGA

AGTCTCGTTATTTCTGAAATACGTTGTCTGTAATAGACCCAGGCACCTTTTAAATTATCTCTGGAACAAG

AGGGATTTCATGTAATGAACTAGGAAATGCATACTCACATAAGCAACAAGGTTCTAGGCAGAAAGCCCCT

TGGAATTTGTGACCAACAGGAGCAAGAACAGGTGCGGCTCAACATGCAATGTCTGAAAATTTGCTTGGCA

TTTTATTCATATATTTAGTGCAAAATTATTTTTGAGTGAGATATTTTACATCACTGTTAATGTGCAATAT

TTAAGATTAAAATACATTAGCTTTTTTATATACTTTGAAGTAGCAAGTTTGTTTTCGATGGCTTAGAGTC

ATGATTTCCAGCTTCCCAGCCTTTTTATCAGTCCCTTTTCTAATACAACAAGGTGCATTAATTTGATTAG

GCAAATTAGAGTTCTAAGACACTTCTTGAATTGTAGACAGAAAATATTGGATTCACAATTTCAGCAGAAA

TTTGAGAATGAGTGTGTTTATATTAATTTCACAATTAGCTGTATTTTCTGTAGCATAGATTATGTCACTG

TTGCACTTTCACAGCAGACATGCTTTCAGAAGGTTCTCATATTTTATGTTTGATTGCTGATAAGCCATCT

CTATTGATACAGATTTTGGTTAAGTAAGGAAAACCAGGTGTGTGTCTGTATCATTTATTGTAAATGCCAG

CTGCCACTTGCCAACCATCATGTTCAGTTCAATTCAAAGAAAACAAACTCTCATTACTTAGTGTAAACTA

AAATACTTAACAAATTATATCCTAAAAACAAGGTCTCTTTGTTAAATGTTGCATGCCCTAGGTTTTAAAT

TACTACATCCAAATACAGTTTTCGTCTTAAATTTGTTAAGCTAAATATATGTTGGTTCTTTTTATTTTGG

AATCCTTTAAGCATCTTAAACATTTTTTTTTTGAAGAGAAGTTACAAATAACATTTCTATCAGGTAGTAC

TTGTATGAAACCACCTTTCTTATTCTATAATTTTGATTTTTCAATTTTATATACTTAATATACTCACTGT

CTTACTATCAGAAAGTTATTTTGACCAAGATTTTTATTATCTTCATAGATTCAGAAAGAGATGCTAATTC

TGTACCAATGTCTTCCTGGTTACTATTCTCTTCCCTCTAATATATACTGGCCATTTGTAAAACCATTGTG

TTGTTGGGATCACTTAGTTATACTATACGCAGATAGAGCATCTCAACTCTGTCATAGTGTTTGCTGAACA

GTTTTCAGTGTCATGCACCTTTGGCTGCTAATTGTTCCTGACGTGCACTCTTCCGAGTTGGTAAAGGCAC

AGTGTGTTCATGCCAGACTTCTAAGAGAAACACCAGCCTCTTAAATCAGAAGCCTACACACAACCCCCTT

AACAATCCAAAGAAGCTTGATGGTGTGCAAAGAAGCATCCTGCCAGCCTTGTCATTGTTCTGTTCTATGC

TAATCCTGCTGTGTTGTCTAAAAGATGGAGGGAAGAGGACATCAGTGTCTGATAGTGAAATCATCAGCAG

GAAAGTGAAGCTCTTTCCTTGGTTACAGATAAGACTTGGTTTACACTATTGGCCAGTATCTGCTAAACAT

ATGAAGACTTAACTATTCAGTGTTGCCTAGGCATTCGCCTGCACAACATTTTGAGGTTAGAACATAGAAT

ATTTTCAGAAATACTGTTGTAGTTTGTGAGTGTTGTTCATTAGTTACACATTAGCTATAGAGTGGATGCA

TGAAGCCCCATGACACCAGTAAACTTCTCTTACCAGTAGGTAAACCAAACACCATTCTGTCATTAGCAGC

CCTCTTAAATGTTGCCTCTCCGTATCCTGTTGCATTTTTGTGTGCATTGTGTTTCTACTGATCTCTCTTA

GGTTTTTACGGAATCAAAGGAAACTAATTTTTCCTTAATAGCAAGAAAGATGAAGAGGTAAAGGGCATTG

AAGCAGAAATGTATAGTTTGGGGTACGATTAGAAAACTCGTAAGGAAAACAGAAGTCCTAATTTCAAACT

GACTGCTCTTCGTTAAGTGCTCTTAAGGAGAGTCTAGTAACAGTAACACTTTCTGGCCATTTCTAGTTTA

GATTCTCTTCGTTACTGAAACTTTTGAGAAATATTACCTGTGGATTAATTTTGCACAATGTTCTATTCTC

ATAATGACTTACAAATTAAACTAGGTTTTTATTGAACTACCTCACACTAATTTTCTATGCTTTCCCAAGT

AAGCTGTTGCCCTGTTAGATCTTTACTGAGTGAATTATAAATGTGTGTTAAATACTTTCTAGCCAATGTT

GACACAATACCAGTAAGTATGTAAAGTATATACCTTACATCAGTAAGAGACACGTGTAAAATCTTTGACT

GTATGTCTTGCAAAATTGTGCTCGTTGACATTATTACTGTTTTTGTAAGTAGAAACCTGCTCGTGATATC

GGTCCATTTACATTTTACAAAAGGAGTAAATCTTAGTAAAAATTTTACGAAGAAATAAATTACTTTTGTA

GGCCCAATATTTGGTATATTTTTGAGAAGCTGTTAATCTTTTAGCTGAATAATGAAGTTAGACTGAATTA

CGTGTCTCCCTGGACTGTGACATCTATTTTCTCATTACAGTTTATCCTGGTCAGCAGGGTGTCACACCTG

GAAACCTGAGTATGATAGCTGACATTTGCTTTTCTCCCTCTGCGATGTCATTCCTCCTCCATTCCTCTCC

TTCCCTGTGTTCCGTTCCCTCTCCTTTCCTCTAGACAAAACAAAATGGGGCACTTTTTAGGGAATGCTGA

GATCATTATTGTGGTTTTTCATCATTCATGCCCTAGTCATTAAACATGCACCACTGGAATGTAAACAATG

TTATCTAGTATGTCAATTGGTTATAATATTTTAAATAAAAAAGAAAAAAGTGGTATGAAAATTATGAAA

>NM_005456.4 Homo sapiens mitogen-activated protein kinase 8 interacting protein 1 (MAPK8IP1), mRNA

AGTCCCGCGGGCCGTGCGCGGTGCTGTGCCGCGCCCTGCCAGACACAGGTGCGCCCGCCTAGCCCGAACT

CCGCGGCGGCGGCTGCCCTCTCGCCGCGCCTCCGCCTCCTTCGCAGCCGCCGCCTCCTCCGCGCCGCGCT

CCGCCCGGATGGCCAGGGCTGTGCCCGAGAATGGCGGAGCGAGAAAGCGGCGGCCTGGGAGGGGGGGCCG

CGTCCCCGCCCGCCGCCTCCCCGTTCCTGGGGCTGCACATCGCTTCGCCTCCCAATTTCAGGCTCACCCA

TGACATCAGCCTGGAGGAGTTTGAGGATGAAGACCTCTCGGAGATCACTGATGAGTGTGGCATCAGCTTA

CAGTGCAAAGACACCCTGTCCTTACGGCCCCCGCGCGCCGGGCTGCTCTCTGCGGGCGGCGGCGGCGCGG

GGAGCCGGTTGCAGGCCGAGATGCTGCAGATGGACCTGATCGACGCGACGGGGGACACTCCCGGGGCCGA

GGACGACGAGGAGGACGACGACGAGGAGCGCGCGGCCCGGCGGCCGGGAGCGGGGCCGCCCAAGGCCGAG

TCCGGCCAGGAGCCGGCGTCCCGCGGCCAGGGCCAGAGCCAAGGCCAGAGCCAGGGCCCGGGCAGCGGGG

ACACGTACCGGCCCAAGCGGCCCACCACGCTCAACCTCTTTCCGCAGGTGCCGCGGTCTCAGGACACACT

GAATAATAATTCTCTGGGCAAAAAGCACAGTTGGCAGGATCGGGTGTCTCGATCATCCTCACCCCTGAAG

ACAGGGGAGCAGACACCACCGCATGAACACATCTGCCTGAGCGATGAGCTGCCCCCCCAGAGCGGCCCCG

CCCCCACCACAGATCGAGGCACCTCCACCGACAGCCCTTGCCGCCGCAGCACAGCCACCCAGATGGCACC

TCCGGGTGGTCCCCCTGCTGCCCCGCCTGGGGGTCGGGGCCACTCGCATCGAGACCGAATCCACTACCAG

GCCGATGTGCGACTAGAGGCCACTGAGGAGATCTACCTGACCCCAGTGCAGAGGCCCCCAGACGCTGCAG

AGCCCACCTCCGCCTTCCTGCCGCCCACTGAGAGCCGGATGTCAGTCAGCTCCGATCCAGACCCTGCCGC

CTACCCCTCCACGGCAGGGCGGCCGCACCCCTCCATCAGTGAAGAGGAAGAGGGCTTCGACTGCCTGTCG

TCCCCAGAGCGGGCTGAGCCCCCAGGCGGAGGGTGGCGGGGGAGCCTGGGGGAGCCGCCGCCACCTCCAC

GGGCCTCTCTGAGCTCGGACACCAGCGCCCTGTCCTATGACTCTGTCAAGTACACGCTGGTGGTAGATGA

GCATGCACAGCTGGAGCTGGTGAGCCTGCGGCCGTGCTTCGGAGACTACAGTGACGAGAGTGACTCTGCC

ACCGTCTATGACAACTGTGCCTCCGTCTCCTCGCCCTATGAGTCGGCCATCGGAGAGGAATATGAGGAGG

CCCCGCGGCCCCAGCCCCCTGCCTGCCTCTCCGAGGACTCCACGCCTGATGAACCCGACGTCCATTTCTC

CAAGAAATTCCTGAACGTCTTCATGAGTGGCCGCTCCCGCTCCTCCAGTGCTGAGTCCTTCGGGCTGTTC

TCCTGCATCATCAACGGGGAGGAGCAGGAGCAGACCCACCGGGCCATATTCAGGTTTGTGCCTCGACACG

AAGACGAACTTGAGCTGGAAGTGGATGACCCTCTGCTAGTGGAGCTCCAGGCTGAAGACTACTGGTACGA

GGCCTACAACATGCGCACTGGTGCCCGGGGTGTCTTTCCTGCCTATTACGCCATCGAGGTCACCAAGGAG

CCCGAGCACATGGCAGCCCTGGCCAAAAACAGTGACTGGGTGGACCAGTTCCGGGTGAAGTTCCTGGGCT

CAGTCCAGGTTCCCTATCACAAGGGCAATGACGTCCTCTGTGCTGCTATGCAAAAGATTGCCACCACCCG

CCGGCTCACCGTGCACTTTAACCCGCCCTCCAGCTGTGTCCTGGAGATCAGCGTGCGGGGTGTGAAGATA

GGCGTCAAGGCCGATGACTCCCAGGAGGCCAAGGGGAATAAATGTAGCCACTTTTTCCAGTTAAAAAACA

TCTCTTTCTGCGGATATCATCCAAAGAACAACAAGTACTTTGGGTTCATCACCAAGCACCCCGCCGACCA

CCGGTTTGCCTGCCACGTCTTTGTGTCTGAAGACTCCACCAAAGCCCTGGCAGAGTCCGTGGGGAGAGCA

TTCCAGCAGTTCTACAAGCAGTTTGTGGAGTACACCTGCCCCACAGAAGATATCTACCTGGAGTAGCTGT

GCAGCCCCGCCCTCTGCGTCCCCCAGCCCTCAGGCCAGTGCCAGGACAGCTGGCTGCTGACAGGATGTGG

CACTGCTTGAGGAGGGGCACCTGCCACCGCCAGAGGACAAGGAAGTGGGGGCCGCTGGCCCAGGGTAGGG

GAGGGTGGGGCAATGGGGAGAGGCAAATGCAGTTTATTGTAATATATGGGATTAGATTCATCTATGGAGG

GCAGAGTGGGCTGCCTGGGGATTGGGAGGGACAGGGCTTGGGGAGCAGGTCTCTGGCAGAGAAGGATGTC

CGTTCCAGGAGCACACGGCCCTGCCCCATCCTGGGCCTTACCTCCCCTGCCAGGGCTCGGGCGCTGTGGC

TCCTGCCTTGATGAAGCCCGTGTCCTGCCTTGATGAAGCCTGTGCCACCTGCAAGTGCCCGCCCTGCCCC

TGCCCCAACCCCCACCGAAGAGCCCTGAGCTCAGGCTGAGCCCAGCCACCTCCCAAGGACTTTCCAGTGA

GGAAATGGCAACACGTGGAGGTGAAGTCCCTGTTCTCAGCTCCGTCATCTGCGGGGCTTCTGGGTGGCTC

CTGCCACTGACCTCACCGGCATGCTGGCCTGTGGCAGGCCTAGGACCTCAGGCGGGGAGGAGGAGCTGCC

GCAAGGCCCTGTCCCAGCAGAAGAGGGAGGCTTCCTGACTGACACAGGCCAGCCCCATCTTGGTCCTGTC

ACCCTGGCCCCAACTATTAAAGTGCCATTTCCTGTCTGGA

>NM_001135044.2 Homo sapiens mitogen-activated protein kinase 9 (MAPK9), transcript variant JNK2-g, mRNA

AGACCGGGCGCCGCGGCGGCGGCAGCGGCGGAGAAGGGAGCGGGACCGGAAGGGTCGCAGCGCCCCGGCG

CCCCTCACACCCACTGCGGCGGCGGCGGCGGCGGCGGCGGGGCGGAGCGGAGCGGGGCGGGCCGGCG

GCGGAGCCGGGGCCGCGGAGCCAGGAGTGACTAGCACGCAGCGCCGCCAGTCCGCCCGCCCGCCCTCTCC

CCGTGGCGCGGCGGCGGGGAGGCGAGGGATCTGAAACTTGCCCACCCTTCGGGATATTGCAGGACGCTGC

ATCATGAGCGACAGTAAATGTGACAGTCAGTTTTATAGTGTGCAAGTGGCAGACTCAACCTTCACTGTCC

TAAAACGTTACCAGCAGCTGAAACCAATTGGCTCTGGGGCCCAAGGGATTGTTTGTGCTGCATTTGATAC

AGTTCTTGGGATAAATGTTGCAGTCAAGAAACTAAGCCGTCCTTTTCAGAACCAAACTCATGCAAAGAGA

GCTTATCGTGAACTTGTCCTCTTAAAATGTGTCAATCATAAAAATATAATTAGTTTGTTAAATGTGTTTA

CACCACAAAAAACTCTAGAAGAATTTCAAGATGTGTATTTGGTTATGGAATTAATGGATGCTAACTTATG

TCAGGTTATTCACATGGAGCTGGATCATGAAAGAATGTCCTACCTTCTTTACCAGATGCTTTGTGGTATT

AAACATCTGCATTCAGCTGGTATAATTCATAGAGATTTGAAGCCTAGCAACATTGTTGTGAAATCAGACT

GCACCCTGAAGATCCTTGACTTTGGCCTGGCCCGGACAGCGTGCACTAACTTCATGATGACCCCTTACGT
GGTGACACGGTACTACCGGGCGCCCGAAGTCATCCTGGGTATGGGCTACAAAGAGAACGTTGATATCTGG
TCAGTGGGTTGCATCATGGGAGAGCTGGTGAAAGGTTGTGTGATATTCCAAGGCACTGACCGTATCCTTC
CCCGCGACCTTGGCCCCGCCATGCTTTCTTAATGCCTCAGGCAGCATGACCACCTGCTTACACGAGCGCT
CACGAGGACAAAGTCGTAAAATCCACTCCAGACTGTGTTCTAAGGGGGGCTTTACTGTATTTGTTAATTA
GTGTCACAAGCCAAGTGGTTCAATTTCATTCTTTATTTTTAACTTGACTACCTGTGATGACTTTGAGACT
TAGAGTTCAGTTAAACCGATTATATTAGCTCTTAATTTTGCCAAAAATAGCTGGACACATAGAAGAAATT
ATTTACTCTTTGATAAGTATATTCTGATATTTAAATGAAAATACTGTATTATTTAGTTTATTCCGTTAAT
TTAACTAGGAAAGATGCTATCTAAATTTAATAGGCTAGTGTGCTTGTGTGCTTTAATTAAGTACTATTTT
GTTAGTTACTGGTATGCATTGAAATATTTTCCCCCAAAGTAAGAACTGAAGCACATTACAAAGGACCTAA
GTGAAAGAGATTAAAATGAAAATTTTGTGAGGAACAATATGTTAAACTTTCTTGTTTGTTACTGTGCTGA
CATTTTGTAGAATTTTTTTTAAAAATCTGTCTAGTGGGTTATTTTGTGACACACTGGGGAAGTCAAAATA
AATGTAAAATGAAAGAGATAAACTCATACTGTAGTCAGCAAGTCATGTTAGAGATACAGCAGAAAGGAGA
ATAACTACTCTAATTGTTACAATAGCCTTCTTTCCTCATTTGCCATTCTTCCATCCTCAGTATTTTTTAC
CCTAACATGGTTCTTCACATCTTCCCTTTTTCATTTGTGTTTTAACCACTTAGTTTTTCATTAGTCTTTG
ACTTTGTTTTCGTTATGAAAGTTTAAATTGGTCTCCATCCTTGGGCAGATTGATGGTTGCTTTTAAGGAA
ATCAAGCTCCATAGTCAGTGTTTTGTATGTATCTTAGGAAAATAATTTCAGATTTTAATAATTTTATCAA
AGTGGTCTCCTTATAAATTCTTCACAGTGCAAACTTAAAGTGAAAATATTTTTATTTCTCTCATTTTATT
TACCTTTCTTTACATTTTCTATATCATAGATTATATAATATGTCAAGTTATTTTATTTGTTCTACTAAAT
TTTCTAGCAGTTTCCATAGCCTCATCTCAGATTCACTTGTTGCAACCTATAAATAAGGACGTCTTTATTT
TGCAAGTAAACTTAATTAAAAAGGGAAAAGTTGAAAAATTAAAAGACTAATAATTTAGAAATATCTACTT
AATGTTCAGTTTGTCTCAATTCTTACTCATTTTGTGCCATTCCTTGTTTTATTACAAAATGTTTTCTTCA
AAAAGTACAAAAATGAAAAATGTGAGTACTTTTTGGTGCATTTTAGTAAGTAAAATTAAAATTCCTGCTA
TACATATGAA

>NM_015884.4 Homo sapiens membrane bound transcription factor peptidase, site 2 (MBTPS2), mRNA

GGCGGTAGCTTGGTTCCTGAGCGGATGCTGGGGCTGTAAGGCGCGCGGTCAGCTGTTGGCGGTGCAGG

GAGGAGGACGCCGGGGCTCGCCTTCCCTCCTCTGCCGCCGCTGCCGCCATGATTCCGGTGTCGCTGGTGG

TGGTGGTGGTGGGTGGCTGGACTGTCGTCTACCTGACCGACTTGGTGCTGAAGTCATCTGTCTATTTTAA

ACATTCTTATGAAGACTGGCTGGAAAACAACGGACTGAGCATCTCCCCTTTCCACATAAGATGGCAAACT

GCTGTTTTCAATCGTGCCTTTTACAGTTGGGGACGGCGGAAAGCAAGGATGCTTTACCAATGGTTCAATT

TTGGAATGGTGTTTGGCGTAATTGCCATGTTTAGCTCATTTTTTCTCCTTGGAAAAACGCTGATGCAGAC

TTTGGCACAAATGATGGCTGACTCTCCCTCTTCTTATTCTTCCTCCTCTTCTTCCTCTTCCTCCTCTTCT

TCCTCTTCCTCTTCTTCATCTTCTTCCTCTTCCTCGCTTCACAATGAACAGGTGTTACAAGTTGTGGTTC

CTGGTATAAATTTACCCGTCAATCAACTGACCTATTTCTTCACGGCAGTTCTCATTAGTGGTGTTGTACA

TGAAATTGGACATGGGATAGCAGCTATTAGGGAACAAGTTCGATTTAATGGCTTTGGGATTTTTCTCTTC

ATTATTTATCCTGGAGCATTTGTTGATCTGTTCACCACTCATTTGCAACTTATATCGCCAGTCCAGCAGC

TAAGGATATTTTGTGCAGGTATCTGGCATAATTTTGTCCTTGCACTCTTGGGTATTTTAGCTCTTGTTCT

CCTCCCAGTAATTCTCTTGCCATTTTACTACACTGGAGTTGGGGTGCTCATCACTGAAGTTGCTGAGGAC

TCTCCTGCCATTGGACCCAGAGGCCTTTTTGTGGGAGACCTTGTCACCCATCTACAGGATTGTCCTGTTA

CTAATGTGCAAGATTGGAATGAATGTTTAGATACCATCGCCTATGAGCCCCAAATTGGTTACTGTATAAG

TGCATCAACTTTACAGCAGTTAAGTTTCCCAGTTAGAGCATACAAACGACTAGATGGTTCAACTGAATGC

TGTAACAATCACAGCCTCACAGATGTGTGCTTTTCCTACAGAAATAATTTTAATAAGCGTTTGCATACAT

GTCTTCCTGCCCGGAAAGCAGTTGAAGCAACTCAAGTTTGCAGAACCAATAAAGACTGTAAAAAAAGCTC

AAGTTCAAGTTTCTGTATAATACCTTCTTTGGAAACTCACACTCGCTTAATAAAAGTAAAACACCCACCT

CAGATTGATATGTTATACGTAGGACATCCTCTGCATCTTCACTACACAGTGAGCATCACCAGTTTTATCC

CACGTTTTAACTTTCTAAGCATAGATCTGCCAGTGGTTGTGGAGACATTTGTCAAGTACCTGATTTCCCT

CTCAGGAGCTCTGGCTATTGTTAATGCAGTACCCTGCTTTGCTTTGGATGGACAATGGATTCTAAACTCT

TTCTTGGATGCCACCCTTACCTCAGTGATTGGAGACAATGATGTCAAAGATCTAATAGGGTTTTTCATCT

TGCTGGGTGGCAGTGTACTTTTGGCTGCCAATGTGACCCTGGGACTCTGGATGGTTACAGCACGGTAATG

TTTGCACTCATCTGACAGAATCCCTGAGTTACAGTATACAGCTATGTGGTAATATTCATTGCCATTGAAA

TTCTTACTTGGTATGAAATATAAAGTGTTTCCTTAAAATTACATCTTAGCCAACAACCCTGAGCTCCTCC

CATATCCAGAGTACCCAAACTCTGTGGTAGAAGATAAGCAGAAGAAATGAAAGGCATAGTCCCTGACTAT

ATTCTAATTTAGGACTGAATGTACCCAGATGCTTGTGGAATAATATCTAAGCAAGTGCAAATTCATGTAA

TACCGTTTTGTCTGATTACATATTGTGTTGAAATAGTATAAAGGAGAACAGAACTGGGTGGAATTAATTG

GGAAAAACTTCTTACAAAAGCATCATTAATCAAAATTTGAAGGAGACCAGACTTTGGCCAAGTGGAAGGA

TGACCATTGTCTTGGCCTGTATCTCTTGTCCTTTGTCTTGTTTGAAAAGTATTTTAAAACTTAATGGTGT

ATTATTTTGTGCTCTGAAAGCTGAGGTCTGATTACAATTAGTAAAATTCTATAGAAGAATCTGCTGACGT

GAAAGGGAAAAATCTTTGTCAAATGATACCAAATAAATGAACAAAACAGGAAGTAGGGCCTATGATATCG

TGAGACATGTTTTGCCCCACAAGAGTTGCATCTTTTATAAGGTGTCTCTGCCCCCTCATAAAGCAGCACT

AGCTTTGACATCCACGGTGAGCTGCAGGAAGCATCACACACCAGCAGCATGTGAGCAGAGGGAGGCAGTT

GGGGTTGAACTTCGGAACTAGGCCGGGTCTCCTGACAGATCACAAGACACCCCAGAGGATCTTCAGCAGT

CCTACTTCCCATTCTCTATAGAGCTTTGAAGCTTGGAACCCTTCCAGGGTAAACATTTTCTCTTGTGCTG

CTCAGGACATCTGGGGCCTAGCTCCTGGGTTCCTGTCTCCAAGAAGCAATGACCTTAAACTCTGAGCCAT

ACTCTGTCCTCACCAGCGGCTCCCATGTTTTTCTGTGTCAGGTTATTAAGTACCTAGTCCTTGTTTTCTG

TCTCTCTCCTAAGCTACCTCTCTGGGTCCACAGAAGACTTGGTAGTATAGTGAGAATGGCTATACGTGAG

TACAAACGTGGATTTTCCAGGGCTTGGGAACTGATTCTTGAGCCCAGAAGAGCCACGCCTGCTTTGAGGT

CTTTTGGAGTGGAGATGCAGCCCTGGGAAATTTGGGGAGTCAGCAGGCCAGTGTGAAGCTATTGGTCCTA

GGAGTATATGAGCTTGCTGTTTCTTTGATGGAAAATACATGCTTCTCTTGTATACTCAGAAGTGACTAAG

GGCAATAACTCATTAATAGCCATCTATCCAACTTCTTTACTGAGTGATGTATTCCATGGGGTTACCTTTT

TCAGATTATTGAGTTGCTCTGTAAGCACTAAAACTTTTTAATCATTTTTAAGAAACTTTTTAGATTGTAT

TACAAATTTGCCTTAACAGTAATTAGATGTTGAATATAATTTTAACATTTTATTAATGACTTGGGTCATC

AGTTAATACCAGTACTAAAACCATACGAATTATTGGTTTATTCCAGAAAATACAGTATTTGTTCTATTTT

TAGGTAGACAATCATTTGGGATCAGAGTACATTAGCATAGTAATGCTCAGTCAGACCTGTTCAAGTAGTA

GAGCTTGGAGAATGCCATGAAATACTTATATAATTAATTTGATTGCATGAACTAAGCAATTTTACTAATG

AAAAGGTTGTATATGTGCAAGTCACTTTTTTAAAAACCAAGAAAAAACTTTAATAGAGGAAATCTTATTC

ATTAATTTATTTTTCTGAGTAAAAAAACGAAACCCAAATCTCATTTTATTTCAACTGTTAAACATTTTGA

TCTGTTGACCCATAGGATCAGGATTTGGGAACCACTTTACTAGGAAAGAGCAGATCAGTACCATTTGTAT

AAAACCGGCCTCATTATGTAAGAAAGAAAATGTTACGTGTTTTCTTCTTTAGCTTGGTTGTGGGCACTTC

TACAGCAAGGACCATATCATATTCATCTTTGCATCCCTGGCACAGTGCATGAGACATAAGTACTTAATAA

ATGCAGTTGAATGGATAATGATTAGTGTTATTTATGGATTAGAAAAAGCATGTTTCTATTTAAGTAAGCT

GTAAAAAGTATTATTGAATATTTACTGTAAATATATGTTCACATAAAAAAATAACTTGGAGGGTCTTTGT

GTCCCTGGCATATTATCATCTTCATGGAAAGAATCCACTGTGGTTTCTGTAGAGTGATTGGAAAAATGGA

TTATTTTGAGGATTGAAGAAAGTGTTCTTTCTGCGTTGTCACTTTGTTCAACAGTAAAACTTTATTCTCA

GTGTTCCTACTCTGCATTGTTTACATTTTTGACAGTTTTTTTTAATCACCTACAATCTGTAAAGAATGTA

TATATTCTTTTCAGCATCTCAGTTTGAAAAGACATGCAGTTAAACTTGACCTTTTGATAATCGCTCTTAC

AGGTCATTGTCTGTTCTAACAGCAAATTGTAAACATGTGCTTCATAGATATTGTGGCTCTCAGTCATCAC

TTTGTCCTATGGTATTTATTGAATGTTCACATACTAATGGTGCACAGGTGTTTTTTTCTATAAATCTTCT

GACTGTCCTGTAATTCATTCTTAAGCTTTAACTTGAAGGTATCGTAATTGCCGGCATTTGATGTTTAGCA

ATAAAAGAATAAATGTGTACCAGCATTTTATGTTTA

>NM_022372.6 Homo sapiens MTOR associated protein, LST8 homolog (MLST8), transcript variant 1, mRNA

GACTCGCTCCGGCTCCCCTCGCCGTCCTGGACACGGCGGAGTGCGGAGCCGCCCGTAAGATGCTCTGACC

TTTGACCCCTGCCGTTCAGCTCTAGGGCCCGTGCAGGCCACACCATGAACACCTCCCCAGGCACGGTGGG

CAGTGACCCGGTCATCCTGGCCACTGCAGGCTACGACCACACCGTGCGCTTCTGGCAGGCCCACAGCGGC

ATCTGCACCCGGACGGTGCAGCACCAGGACTCCCAGGTGAATGCCTTGGAGGTCACACCGGACCGCAGCA

TGATTGCTGCTGCAGGTTACCAGCACATCCGCATGTATGATCTCAACTCCAATAACCCTAACCCCATCAT

CAGCTACGACGGCGTCAACAAGAACATCGCGTCTGTGGGCTTCCACGAAGACGGCCGCTGGATGTACACG

GGCGGCGAGGACTGCACAGCCAGGATCTGGGACCTCAGGTCCCGGAACCTGCAGTGCCAGCGGATCTTCC

AGGTGAACGCACCCATTAACTGCGTGTGCCTGCACCCCAACCAGGCAGAGCTCATCGTGGGTGACCAGAG

CGGGGCTATCCACATCTGGGACTTGAAAACAGACCACAACGAGCAGCTGATCCCTGAGCCCGAGGTCTCC

ATCACGTCCGCCCACATCGATCCCGACGCCAGCTACATGGCAGCTGTCAATAGCACCGGAAACTGCTATG

TCTGGAATCTGACGGGGGGCATTGGTGACGAGGTGACCCAGCTCATCCCCAAGACTAAGATCCCTGCCCA

CACGCGCTACGCCCTGCAGTGTCGCTTCAGCCCCGACTCCACGCTCCTCGCCACCTGCTCGGCTGATCAG

ACGTGCAAGATCTGGAGGACGTCCAACTTCTCCCTGATGACGGAGCTGAGCATCAAGAGCGGCAACCCCG

GGGAGTCCTCCCGCGGCTGGATGTGGGGCTGCGCCTTCTCGGGGGACTCCCAGTACATCGTCACTGCTTC

CTCGGACAACCTGGCCCGGCTCTGGTGTGTGGAGACTGGAGAGATCAAGAGAGAGTATGGCGGCCACCAG

AAGGCTGTTGTCTGCCTGGCCTTCAATGACAGTGTGCTGGGCTAGCCTGTGACCCCTCGGGACTGCCTGG

TGCAGGTGGTGGCAGCTGGAGGGACCCATGCAGCACCCAGGTCAGAGCAGACCCTCCCCTGCCGGCCTGC

GCCAGCTGGACCTGATGGCCCCCTGTGGCGCCTTGACCTGCTGGGCCAGGCTGCCCTGGGACTCTCAGCC

CCCAGTTGCTTATCCAGATGTGACAGAGCTCGACCCAAGCCAGGCTGCACACTCCTGGACTGGGCTAGCC

TGCACTGCCTGGGAAAGTCGGCCGAGGGCCCAAAGCTGCTGAGGGGTCTGAGGCTGGTGCCCACCCCCAA

GCTAGTGTGTTCTCTGCCCCTCCCTGCCCGCGTTTCAGGGCCTCGGTCCATAGAGAACACCACCACCATG

GCCAGGTGGAAGGGTTTATTAGTCCCTGCCAGCAGCTGTCCTCCCTGGTGCAGGTGGCCTGGCCAGCCCA

CTGGATTGGGGACGGGCCAGGCTGGGCCAGGTCGGGGGCTCAGTCTGGGAGGTAATAAAAGCAGACCGAC

ACGCAGATGTTGCTCGGGAAGCAGATGTCGATGCAGAGATAAATCAGCCGCTGTCTCCGGG

>NM_022485.5 Homo sapiens myotubularin related protein 14 (MTMR14), transcript variant 3, mRNA

AGGTTCTAGTGTCGGAGTTGGGTGCAGGCAGGTGCCATGGGCCCGCTTGAGGCACACTGAGGGGACGCGG

GGCTGGGCCATGGCCGGCGCTCGGGCCGCCGCCGCCGCTGCCTCGGCGGGGTCCTCGGCCTCTTCAGGCA

ACCAGCCGCCTCAGGAGCTGGGGCTTGGGGAGCTGCTGGAGGAGTTCTCCCGGACTCAGTACCGGGCCAA

GGATGGCAGCGGGACCGGCGGCTCTAAGGTTGAGCGCATTGAGAAGAGATGTCTGGAGCTGTTTGGCCGA

GACTACTGTTTCAGCGTGATTCCAAACACGAATGGGGATATCTGTGGCCACTATCCCCGGCACATCGTGT

TCCTGGAGTATGAGAGTTCTGAGAAGGAGAAAGACACGTTTGAGAGTACCGTACAGGTGAGCAAGTTGCA

AGACCTCATCCACCGCAGCAAGATGGCCCGGTGCAGAGGACGGTTTGTCTGCCCAGTAATCCTGTTCAAG

GGCAAGCACATTTGCAGGTCGGCCACACTGGCTGGATGGGGAGAGCTGTATGGACGCTCAGGCTACAACT

ATTTTTTCTCAGGGGGTGCAGATGATGCCTGGGCAGATGTGGAGGACGTCACGGAGGAGGACTGTGCTCT

TCGAAGTGGTGACACGCATCTTTTTGATAAGGTCAGAGGCTATGACATCAAGCTGCTTCGATACCTGTCA

GTCAAATACATCTGTGACCTGATGGTGGAGAACAAGAAGGTGAAGTTTGGCATGAATGTAACCTCCTCTG

AGAAGGTGGACAAAGCCCAGCGCTATGCCGACTTCACTCTCCTCTCCATCCCGTATCCAGGCTGTGAATT

TTTCAAGGAATATAAAGATCGGGATTACATGGCAGAAGGGCTCATATTTAACTGGAAGCAGGACTACGTT

GATGCCCCATTGAGCATCCCCGACTTCCTGACTCACTCTCTGAACATTGACTGGAGCCAGTATCAGTGTT

GGGATCTGGTGCAACAAACACAAAACTACCTGAAGCTGCTGCTTTCCTTAGTTAACAGTGATGATGACAG

CGGGCTGCTGGTACACTGTATCTCAGGCTGGGATCGGACCCCCCTCTTCATCTCCCTCCTGCGCCTTTCC

TTGTGGGCTGATGGGCTCATCCACACGTCCCTGAAGCCCACTGAGATCCTCTACCTCACTGTGGCCTATG

ACTGGTTCCTCTTCGGGCACATGTTGGTAGATCGGCTCAGCAAAGGGGAGGAGATTTTCTTCTTCTGCTT

CAATTTTTTGAAGCATATTACCTCCGAGGAGTTCTCTGCTCTGAAGACCCAGAGGAGGAAGAGTTTGCCA

GCCCGGGATGGAGGCTTCACCCTGGAAGACATCTGCATGCTGAGACGAAAGGACCGTGGCAGCACCACCA

GCCTTGGCAGCGACTTCTCCCTGGTCATGGAGAGTTCCCCAGGAGCCACTGGGAGCTTCACCTATGAGGC

CGTGGAGCTGGTCCCAGCAGGAGCGCCAACTCAGGCAGCTTGGCTTGCAGCCCTGAGTGATCGAGAGACT

CGGCTGCAGGAGGTGCGCTCAGCCTTCTTGGCTGCGTACAGCAGCACAGTGGGGCTTCGGGCAGTAGCCC

CCAGTCCTTCCGGTGCCATCGGGGGCCTGCTGGAGCAATTTGCCCGTGGTGTTGGACTCCGGAGCATCAG

CAGCAATGCCTTGTGAAGAAGCCAGCCCATGACATTTTCCTGCTCCTCTCTCAGCTGAGCCCTTAGCAGA

GAATCAAAGCCATGCCTGGCCGAAGGGGTACTTCCAGGTCAGGGGAAATTTCAGTCCCCCATCTCCATCA

TGAACATGGCAGCCCCAAAGCTGAGCAAGGCCAAAGACAGGGTTTTCCAACCCCCAGCCTCTTGACTGGT

GACCACCACCCCTTCTTGTCACTGTCTCCCACCCACCCCATCTTTGCTGGGATTCCCATCAACTCTCAGA

ACTGTGTGGGGTTTCCCTGGGGCCTTGTGGAAGCCATGACTTCACAAAGACCCTACCTGTCAGTTCTTGT

TTCTGGGGAGGAGGGATCACCTGCACTGAGAATGAGGCAGTTTGACACAGATCACAAAATAAAATCAAAG

TCTTTTTGAATAGCCA

>NM_004958.4 Homo sapiens mechanistic target of rapamycin kinase (MTOR), transcript variant 1, mRNA

GCTCCCGGCTTAGAGGACAGCGGGGAAGGCGGGCGGTGGGGCAGGGGGCCTGAAGCGGCGGTACCGGTGC

TGGCGGCGGCAGCTGAGGCCTTGGCCGAAGCCGCGCGAACCTCAGGGCAAGATGCTTGGAACCGGACCTG

CCGCCGCCACCACCGCTGCCACCACATCTAGCAATGTGAGCGTCCTGCAGCAGTTTGCCAGTGGCCTAAA

GAGCCGGAATGAGGAAACCAGGGCCAAAGCCGCCAAGGAGCTCCAGCACTATGTCACCATGGAACTCCGA

GAGATGAGTCAAGAGGAGTCTACTCGCTTCTATGACCAACTGAACCATCACATTTTTGAATTGGTTTCCA

GCTCAGATGCCAATGAGAGGAAAGGTGGCATCTTGGCCATAGCTAGCCTCATAGGAGTGGAAGGTGGGAA

TGCCACCCGAATTGGCAGATTTGCCAACTATCTTCGGAACCTCCTCCCCTCCAATGACCCAGTTGTCATG

GAAATGGCATCCAAGGCCATTGGCCGTCTTGCCATGGCAGGGGACACTTTTACCGCTGAGTACGTGGAAT

TTGAGGTGAAGCGAGCCCTGGAATGGCTGGGTGCTGACCGCAATGAGGGCCGGAGACATGCAGCTGTCCT

GGTTCTCCGTGAGCTGGCCATCAGCGTCCCTACCTTCTTCTTCCAGCAAGTGCAACCCTTCTTTGACAAC

ATTTTTGTGGCCGTGTGGGACCCCAAACAGGCCATCCGTGAGGGAGCTGTAGCCGCCCTTCGTGCCTGTC

TGATTCTCACAACCCAGCGTGAGCCGAAGGAGATGCAGAAGCCTCAGTGGTACAGGCACACATTTGAAGA

AGCAGAGAAGGGATTTGATGAGACCTTGGCCAAAGAGAAGGGCATGAATCGGGATGATCGGATCCATGGA

GCCTTGTTGATCCTTAACGAGCTGGTCCGAATCAGCAGCATGGAGGGAGAGCGTCTGAGAGAAGAAATGG

AAGAAATCACACAGCAGCAGCTGGTACACGACAAGTACTGCAAAGATCTCATGGGCTTCGGAACAAAACC

TCGTCACATTACCCCCTTCACCAGTTTCCAGGCTGTACAGCCCCAGCAGTCAAATGCCTTGGTGGGGCTG

CTGGGGTACAGCTCTCACCAAGGCCTCATGGGATTTGGGACCTCCCCCAGTCCAGCTAAGTCCACCCTGG

TGGAGAGCCGGTGTTGCAGAGACTTGATGGAGGAGAAATTTGATCAGGTGTGCCAGTGGGTGCTGAAATG

CAGGAATAGCAAGAACTCGCTGATCCAAATGACAATCCTTAATTTGTTGCCCCGCTTGGCTGCATTCCGA

CCTTCTGCCTTCACAGATACCCAGTATCTCCAAGATACCATGAACCATGTCCTAAGCTGTGTCAAGAAGG

AGAAGGAACGTACAGCGGCCTTCCAAGCCCTGGGGCTACTTTCTGTGGCTGTGAGGTCTGAGTTTAAGGT

CTATTTGCCTCGCGTGCTGGACATCATCCGAGCGGCCCTGCCCCCAAAGGACTTCGCCCATAAGAGGCAG

AAGGCAATGCAGGTGGATGCCACAGTCTTCACTTGCATCAGCATGCTGGCTCGAGCAATGGGGCCAGGCA

TCCAGCAGGATATCAAGGAGCTGCTGGAGCCCATGCTGGCAGTGGGACTAAGCCCTGCCCTCACTGCAGT

GCTCTACGACCTGAGCCGTCAGATTCCACAGCTAAAGAAGGACATTCAAGATGGGCTACTGAAAATGCTG

TCCCTGGTCCTTATGCACAAACCCCTTCGCCACCCAGGCATGCCCAAGGGCCTGGCCCATCAGCTGGCCT

CTCCTGGCCTCACGACCCTCCCTGAGGCCAGCGATGTGGGCAGCATCACTCTTGCCCTCCGAACGCTTGG

CAGCTTTGAATTTGAAGGCCACTCTCTGACCCAATTTGTTCGCCACTGTGCGGATCATTTCCTGAACAGT

GAGCACAAGGAGATCCGCATGGAGGCTGCCCGCACCTGCTCCCGCCTGCTCACACCCTCCATCCACCTCA

TCAGTGGCCATGCTCATGTGGTTAGCCAGACCGCAGTGCAAGTGGTGGCAGATGTGCTTAGCAAACTGCT

CGTAGTTGGGATAACAGATCCTGACCCTGACATTCGCTACTGTGTCTTGGCGTCCCTGGACGAGCGCTTT

GATGCACACCTGGCCCAGGCGGAGAACTTGCAGGCCTTGTTTGTGGCTCTGAATGACCAGGTGTTTGAGA

TCCGGGAGCTGGCCATCTGCACTGTGGGCCGACTCAGTAGCATGAACCCTGCCTTTGTCATGCCTTTCCT

GCGCAAGATGCTCATCCAGATTTTGACAGAGTTGGAGCACAGTGGGATTGGAAGAATCAAAGAGCAGAGT

GCCCGCATGCTGGGGCACCTGGTCTCCAATGCCCCCCGACTCATCCGCCCCTACATGGAGCCTATTCTGA

AGGCATTAATTTTGAAACTGAAAGATCCAGACCCTGATCCAAACCCAGGTGTGATCAATAATGTCCTGGC

AACAATAGGAGAATTGGCACAGGTTAGTGGCCTGGAAATGAGGAAATGGGTTGATGAACTTTTTATTATC

ATCATGGACATGCTCCAGGATTCCTCTTTGTTGGCCAAAAGGCAGGTGGCTCTGTGGACCCTGGGACAGT

TGGTGGCCAGCACTGGCTATGTAGTAGAGCCCTACAGGAAGTACCCTACTTTGCTTGAGGTGCTACTGAA

TTTTCTGAAGACTGAGCAGAACCAGGGTACACGCAGAGAGGCCATCCGTGTGTTAGGGCTTTTAGGGGCT

TTGGATCCTTACAAGCACAAAGTGAACATTGGCATGATAGACCAGTCCCGGGATGCCTCTGCTGTCAGCC

TGTCAGAATCCAAGTCAAGTCAGGATTCCTCTGACTATAGCACTAGTGAAATGCTGGTCAACATGGGAAA

CTTGCCTCTGGATGAGTTCTACCCAGCTGTGTCCATGGTGGCCCTGATGCGGATCTTCCGAGACCAGTCA

CTCTCTCATCATCACACCATGGTTGTCCAGGCCATCACCTTCATCTTCAAGTCCCTGGGACTCAAATGTG

TGCAGTTCCTGCCCCAGGTCATGCCCACGTTCCTTAACGTCATTCGAGTCTGTGATGGGGCCATCCGGGA

ATTTTTGTTCCAGCAGCTGGGAATGTTGGTGTCCTTTGTGAAGAGCCACATCAGACCTTATATGGATGAA

ATAGTCACCCTCATGAGAGAATTCTGGGTCATGAACACCTCAATTCAGAGCACGATCATTCTTCTCATTG

AGCAAATTGTGGTAGCTCTTGGGGGTGAATTTAAGCTCTACCTGCCCCAGCTGATCCCACACATGCTGCG

TGTCTTCATGCATGACAACAGCCCAGGCCGCATTGTCTCTATCAAGTTACTGGCTGCAATCCAGCTGTTT

GGCGCCAACCTGGATGACTACCTGCATTTACTGCTGCCTCCTATTGTTAAGTTGTTTGATGCCCCTGAAG

CTCCACTGCCATCTCGAAAGGCAGCGCTAGAGACTGTGGACCGCCTGACGGAGTCCCTGGATTTCACTGA

CTATGCCTCCCGGATCATTCACCCTATTGTTCGAACACTGGACCAGAGCCCAGAACTGCGCTCCACAGCC

ATGGACACGCTGTCTTCACTTGTTTTTCAGCTGGGGAAGAAGTACCAAATTTTCATTCCAATGGTGAATA

AAGTTCTGGTGCGACACCGAATCAATCATCAGCGCTATGATGTGCTCATCTGCAGAATTGTCAAGGGATA

CACACTTGCTGATGAAGAGGAGGATCCTTTGATTTACCAGCATCGGATGCTTAGGAGTGGCCAAGGGGAT

GCATTGGCTAGTGGACCAGTGGAAACAGGACCCATGAAGAAACTGCACGTCAGCACCATCAACCTCCAAA

AGGCCTGGGGCGCTGCCAGGAGGGTCTCCAAAGATGACTGGCTGGAATGGCTGAGACGGCTGAGCCTGGA

GCTGCTGAAGGACTCATCATCGCCCTCCCTGCGCTCCTGCTGGGCCCTGGCACAGGCCTACAACCCGATG

GCCAGGGATCTCTTCAATGCTGCATTTGTGTCCTGCTGGTCTGAACTGAATGAAGATCAACAGGATGAGC

TCATCAGAAGCATCGAGTTGGCCCTCACCTCACAAGACATCGCTGAAGTCACACAGACCCTCTTAAACTT

GGCTGAATTCATGGAACACAGTGACAAGGGCCCCCTGCCACTGAGAGATGACAATGGCATTGTTCTGCTG

GGTGAGAGAGCTGCCAAGTGCCGAGCATATGCCAAAGCACTACACTACAAAGAACTGGAGTTCCAGAAAG

GCCCCACCCCTGCCATTCTAGAATCTCTCATCAGCATTAATAATAAGCTACAGCAGCCGGAGGCAGCGGC

CGGAGTGTTAGAATATGCCATGAAACACTTTGGAGAGCTGGAGATCCAGGCTACCTGGTATGAGAAACTG

CACGAGTGGGAGGATGCCCTTGTGGCCTATGACAAGAAAATGGACACCAACAAGGACGACCCAGAGCTGA

TGCTGGGCCGCATGCGCTGCCTCGAGGCCTTGGGGGAATGGGGTCAACTCCACCAGCAGTGCTGTGAAAA

GTGGACCCTGGTTAATGATGAGACCCAAGCCAAGATGGCCCGGATGGCTGCTGCAGCTGCATGGGGTTTA

GGTCAGTGGGACAGCATGGAAGAATACACCTGTATGATCCCTCGGGACACCCATGATGGGGCATTTTATA

GAGCTGTGCTGGCACTGCATCAGGACCTCTTCTCCTTGGCACAACAGTGCATTGACAAGGCCAGGGACCT

GCTGGATGCTGAATTAACTGCGATGGCAGGAGAGAGTTACAGTCGGGCATATGGGGCCATGGTTTCTTGC

CACATGCTGTCCGAGCTGGAGGAGGTTATCCAGTACAAACTTGTCCCCGAGCGACGAGAGATCATCCGCC

AGATCTGGTGGGAGAGACTGCAGGGCTGCCAGCGTATCGTAGAGGACTGGCAGAAAATCCTTATGGTGCG

GTCCCTTGTGGTCAGCCCTCATGAAGACATGAGAACCTGGCTCAAGTATGCAAGCCTGTGCGGCAAGAGT

GGCAGGCTGGCTCTTGCTCATAAAACTTTAGTGTTGCTCCTGGGAGTTGATCCGTCTCGGCAACTTGACC

ATCCTCTGCCAACAGTTCACCCTCAGGTGACCTATGCCTACATGAAAAACATGTGGAAGAGTGCCCGCAA

GATCGATGCCTTCCAGCACATGCAGCATTTTGTCCAGACCATGCAGCAACAGGCCCAGCATGCCATCGCT

ACTGAGGACCAGCAGCATAAGCAGGAACTGCACAAGCTCATGGCCCGATGCTTCCTGAAACTTGGAGAGT

GGCAGCTGAATCTACAGGGCATCAATGAGAGCACAATCCCCAAAGTGCTGCAGTACTACAGCGCCGCCAC

AGAGCACGACCGCAGCTGGTACAAGGCCTGGCATGCGTGGGCAGTGATGAACTTCGAAGCTGTGCTACAC

TACAAACATCAGAACCAAGCCCGCGATGAGAAGAAGAAACTGCGTCATGCCAGCGGGGCCAACATCACCA

ACGCCACCACTGCCGCCACCACGGCCGCCACTGCCACCACCACTGCCAGCACCGAGGGCAGCAACAGTGA

GAGCGAGGCCGAGAGCACCGAGAACAGCCCCACCCCATCGCCGCTGCAGAAGAAGGTCACTGAGGATCTG

TCCAAAACCCTCCTGATGTACACGGTGCCTGCCGTCCAGGGCTTCTTCCGTTCCATCTCCTTGTCACGAG

GCAACAACCTCCAGGATACACTCAGAGTTCTCACCTTATGGTTTGATTATGGTCACTGGCCAGATGTCAA

TGAGGCCTTAGTGGAGGGGGTGAAAGCCATCCAGATTGATACCTGGCTACAGGTTATACCTCAGCTCATT

GCAAGAATTGATACGCCCAGACCCTTGGTGGGACGTCTCATTCACCAGCTTCTCACAGACATTGGTCGGT

ACCACCCCCAGGCCCTCATCTACCCACTGACAGTGGCTTCTAAGTCTACCACGACAGCCCGGCACAATGC

AGCCAACAAGATTCTGAAGAACATGTGTGAGCACAGCAACACCCTGGTCCAGCAGGCCATGATGGTGAGC

GAGGAGCTGATCCGAGTGGCCATCCTCTGGCATGAGATGTGGCATGAAGGCCTGGAAGAGGCATCTCGTT

TGTACTTTGGGGAAAGGAACGTGAAAGGCATGTTTGAGGTGCTGGAGCCCTTGCATGCTATGATGGAACG

GGGCCCCCAGACTCTGAAGGAAACATCCTTTAATCAGGCCTATGGTCGAGATTTAATGGAGGCCCAAGAG

TGGTGCAGGAAGTACATGAAATCAGGGAATGTCAAGGACCTCACCCAAGCCTGGGACCTCTATTATCATG

TGTTCCGACGAATCTCAAAGCAGCTGCCTCAGCTCACATCCTTAGAGCTGCAATATGTTTCCCCAAAACT

TCTGATGTGCCGGGACCTTGAATTGGCTGTGCCAGGAACATATGACCCCAACCAGCCAATCATTCGCATT

CAGTCCATAGCACCGTCTTTGCAAGTCATCACATCCAAGCAGAGGCCCCGGAAATTGACACTTATGGGCA

GCAACGGACATGAGTTTGTTTTCCTTCTAAAAGGCCATGAAGATCTGCGCCAGGATGAGCGTGTGATGCA

GCTCTTCGGCCTGGTTAACACCCTTCTGGCCAATGACCCAACATCTCTTCGGAAAAACCTCAGCATCCAG

AGATACGCTGTCATCCCTTTATCGACCAACTCGGGCCTCATTGGCTGGGTTCCCCACTGTGACACACTGC

ACGCCCTCATCCGGGACTACAGGGAGAAGAAGAAGATCCTTCTCAACATCGAGCATCGCATCATGTTGCG

GATGGCTCCGGACTATGACCACTTGACTCTGATGCAGAAGGTGGAGGTGTTTGAGCATGCCGTCAATAAT

ACAGCTGGGGACGACCTGGCCAAGCTGCTGTGGCTGAAAAGCCCCAGCTCCGAGGTGTGGTTTGACCGAA

GAACCAATTATACCCGTTCTTTAGCGGTCATGTCAATGGTTGGGTATATTTTAGGCCTGGGAGATAGACA

CCCATCCAACCTGATGCTGGACCGTCTGAGTGGGAAGATCCTGCACATTGACTTTGGGGACTGCTTTGAG

GTTGCTATGACCCGAGAGAAGTTTCCAGAGAAGATTCCATTTAGACTAACAAGAATGTTGACCAATGCTA

TGGAGGTTACAGGCCTGGATGGCAACTACAGAATCACATGCCACACAGTGATGGAGGTGCTGCGAGAGCA

CAAGGACAGTGTCATGGCCGTGCTGGAAGCCTTTGTCTATGACCCCTTGCTGAACTGGAGGCTGATGGAC

ACAAATACCAAAGGCAACAAGCGATCCCGAACGAGGACGGATTCCTACTCTGCTGGCCAGTCAGTCGAAA

TTTTGGACGGTGTGGAACTTGGAGAGCCAGCCCATAAGAAAACGGGGACCACAGTGCCAGAATCTATTCA

TTCTTTCATTGGAGACGGTTTGGTGAAACCAGAGGCCCTAAATAAGAAAGCTATCCAGATTATTAACAGG

GTTCGAGATAAGCTCACTGGTCGGGACTTCTCTCATGATGACACTTTGGATGTTCCAACGCAAGTTGAGC

TGCTCATCAAACAAGCGACATCCCATGAAAACCTCTGCCAGTGCTATATTGGCTGGTGCCCTTTCTGGTA

ACTGGAGGCCCAGATGTGCCCATCACGTTTTTTCTGAGGCTTTTGTACTTTAGTAAATGCTTCCACTAAA

CTGAAACCATGGTGAGAAAGTTTGACTTTGTTAAATATTTTGAAATGTAAATGAAAAGAACTACTGTATA

TTAAAAGTTGGTTTGAACCAACTTTCTAGCTGCTGTTGAAGAATATATTGTCAGAAACACAAGGCTTGAT

TTGGTTCCCAGGACAGTGAAACATAGTAATACCACGTAAATCAAGCCATTCATTTTGGGGAACAGAAGAT

CCATAACTTTAGAAATACGGGTTTTGACTTAACTCACAAGAGAACTCATCATAAGTACTTGCTGATGGAA

GAATGACCTAGTTGCTCCTCTCAACATGGGTACAGCAAACTCAGCACAGCCAAGAAGCCTCAGGTCGTGG

AGAACATGGATTAGGATCCTAGACTGTAAAGACACAGAAGATGCTGACCTCACCCCTGCCACCTATCCCA

AGACCTCACTGGTCTGTGGACAGCAGCAGAAATGTTTGCAAGATAGGCCAAAATGAGTACAAAAGGTCTG

TCTTCCATCAGACCCAGTGATGCTGCGACTCACACGCTTCAATTCAAGACCTGACCGCTAGTAGGGAGGT

TTATTCAGATCGCTGGCAGCCTCGGCTGAGCAGATGCACAGAGGGGATCACTGTGCAGTGGGACCACCCT

CACTGGCCTTCTGCAGCAGGGTTCTGGGATGTTTTCAGTGGTCAAAATACTCTGTTTAGAGCAAGGGCTC

AGAAAACAGAAATACTGTCATGGAGGTGCTGAACACAGGGAAGGTCTGGTACATATTGGAAATTATGAGC

AGAACAAATACTCAACTAAATGCACAAAGTATAAAGTGTAGCCATGTCTAGACACCATGTTGTATCAGAA

TAATTTTTGTGCCAATAAATGACATCAGAATTTTAAACATA

>NM_001354870.1 Homo sapiens MYC proto-oncogene, bHLH transcription factor (MYC), transcript variant 2, mRNA

GGAGTTTATTCATAACGCGCTCTCCAAGTATACGTGGCAATGCGTTGCTGGGTTATTTTAATCATTCTAG

GCATCGTTTTCCTCCTTATGCCTCTATCATTCCTCCCTATCTACACTAACATCCCACGCTCTGAACGCGC

GCCCATTAATACCCTTCTTTCCTCCACTCTCCCTGGGACTCTTGATCAAAGCGCGGCCCTTTCCCCAGCC

TTAGCGAGGCGCCCTGCAGCCTGGTACGCGCGTGGCGTGGCGGTGGGCGCGCAGTGCGTTCTCGGTGTGG

AGGGCAGCTGTTCCGCCTGCGATGATTTATACTCACAGGACAAGGATGCGGTTTGTCAAACAGTACTGCT

ACGGAGGAGCAGCAGAGAAAGGGAGAGGGTTTGAGAGGGAGCAAAAGAAAATGGTAGGCGCGCGTAGTTA

ATTCATGCGGCTCTCTTACTCTGTTTACATCCTAGAGCTAGAGTGCTCGGCTGCCCGGCTGAGTCTCCTC

CCCACCTTCCCCACCCTCCCCACCCTCCCCATAAGCGCCCCTCCCGGGTTCCCAAAGCAGAGGGCGTGGG

GGAAAAGAAAAAAGATCCTCTCTCGCTAATCTCCGCCCACCGGCCCTTTATAATGCGAGGGTCTGGACGG

CTGAGGACCCCCGAGCTGTGCTGCTCGCGGCCGCCACCGCCGGGCCCCGGCCGTCCCTGGCTCCCCTCCT

GCCTCGAGAAGGGCAGGGCTTCTCAGAGGCTTGGCGGGAAAAAGAACGGAGGGAGGGATCGCGCTGAGTA

TAAAAGCCGGTTTTCGGGGCTTTATCTAACTCGCTGTAGTAATTCCAGCGAGAGGCAGAGGGAGCGAGCG

GGCGGCCGGCTAGGGTGGAAGAGCCGGGCGAGCAGAGCTGCGCTGCGGGCGTCCTGGGAAGGGAGATCCG

GAGCGAATAGGGGGCTTCGCCTCTGGCCCAGCCCTCCCGCTGATCCCCCAGCCAGCGGTCCGCAACCCTT

GCCGCATCCACGAAACTTTGCCCATAGCAGCGGGCGGGCACTTTGCACTGGAACTTACAACACCCGAGCA

AGGACGCGACTCTCCCGACGCGGGGAGGCTATTCTGCCCATTTGGGGACACTTCCCCGCCGCTGCCAGGA

CCCGCTTCTCTGAAAGGCTCTCCTTGCAGCTGCTTAGACGCTGGATTTTTTTCGGGTAGTGGAAAACCAG

CCTCCCGCGACGATGCCCCTCAACGTTAGCTTCACCAACAGGAACTATGACCTCGACTACGACTCGGTGC

AGCCGTATTTCTACTGCGACGAGGAGGAGAACTTCTACCAGCAGCAGCAGCAGAGCGAGCTGCAGCCCCC

GGCGCCCAGCGAGGATATCTGGAAGAAATTCGAGCTGCTGCCCACCCCGCCCCTGTCCCCTAGCCGCCGC

TCCGGGCTCTGCTCGCCCTCCTACGTTGCGGTCACACCCTTCTCCCTTCGGGGAGACAACGACGGCGGTG

GCGGGAGCTTCTCCACGGCCGACCAGCTGGAGATGGTGACCGAGCTGCTGGGAGGAGACATGGTGAACCA

GAGTTTCATCTGCGACCCGGACGACGAGACCTTCATCAAAAACATCATCATCCAGGACTGTATGTGGAGC

GGCTTCTCGGCCGCCGCCAAGCTCGTCTCAGAGAAGCTGGCCTCCTACCAGGCTGCGCGCAAAGACAGCG

GCAGCCCGAACCCCGCCCGCGGCCACAGCGTCTGCTCCACCTCCAGCTTGTACCTGCAGGATCTGAGCGC

CGCCGCCTCAGAGTGCATCGACCCCTCGGTGGTCTTCCCCTACCCTCTCAACGACAGCAGCTCGCCCAAG

TCCTGCGCCTCGCAAGACTCCAGCGCCTTCTCTCCGTCCTCGGATTCTCTGCTCTCCTCGACGGAGTCCT

CCCCGCAGGGCAGCCCCGAGCCCCTGGTGCTCCATGAGGAGACACCGCCCACCACCAGCAGCGACTCTGA

GGAGGAACAAGAAGATGAGGAAGAAATCGATGTTGTTTCTGTGGAAAAGAGGCAGGCTCCTGGCAAAAGG

TCAGAGTCTGGATCACCTTCTGCTGGAGGCCACAGCAAACCTCCTCACAGCCCACTGGTCCTCAAGAGGT

GCCACGTCTCCACACATCAGCACAACTACGCAGCGCCTCCCTCCACTCGGAAGGACTATCCTGCTGCCAA

GAGGGTCAAGTTGGACAGTGTCAGAGTCCTGAGACAGATCAGCAACAACCGAAAATGCACCAGCCCCAGG

TCCTCGGACACCGAGGAGAATGTCAAGAGGCGAACACACAACGTCTTGGAGCGCCAGAGGAGGAACGAGC

TAAAACGGAGCTTTTTTGCCCTGCGTGACCAGATCCCGGAGTTGGAAAACAATGAAAAGGCCCCCAAGGT

AGTTATCCTTAAAAAAGCCACAGCATACATCCTGTCCGTCCAAGCAGAGGAGCAAAAGCTCATTTCTGAA

GAGGACTTGTTGCGGAAACGACGAGAACAGTTGAAACACAAACTTGAACAGCTACGGAACTCTTGTGCGT

AAGGAAAAGTAAGGAAAACGATTCCTTCTAACAGAAATGTCCTGAGCAATCACCTATGAACTTGTTTCAA

ATGCATGATCAAATGCAACCTCACAACCTTGGCTGAGTCTTGAGACTGAAAGATTTAGCCATAATGTAAA

CTGCCTCAAATTGGACTTTGGGCATAAAAGAACTTTTTTATGCTTACCATCTTTTTTTTTTCTTTAACAG

ATTTGTATTTAAGAATTGTTTTTAAAAAATTTTAAGATTTACACAATGTTTCTCTGTAAATATTGCCATT

AAATGTAAATAACTTTAATAAAACGTTTATAGCAGTTACACAGAATTTCAATCCTAGTATATAGTACCTA

GTATTATAGGTACTATAAACCCTAATTTTTTTTATTTAAGTACATTTTGCTTTTTAAAGTTGATTTTTTT

CTATTGTTTTTAGAAAAAATAAAATAACTGGCAAATATATCATTGAGCCAAATCTTAAGTTGTGAATGTT

TTGTTTCGTTTCTTCCCCCTCCCAACCACCACCATCCCTGTTTGTTTTCATCAATTGCCCCTTCAGAGGG

TGGTCTTAAGAAAGGCAAGAGTTTTCCTCTGTTGAAATGGGTCTGGGGGCCTTAAGGTCTTTAAGTTCTT

GGAGGTTCTAAGATGCTTCCTGGAGACTATGATAACAGCCAGAGTTGACAGTTAGAAGGAATGGCAGAAG

GCAGGTGAGAAGGTGAGAGGTAGGCAAAGGAGATACAAGAGGTCAAAGGTAGCAGTTAAGTACACAAAGA

GGCATAAGGACTGGGGAGTTGGGAGGAAGGTGAGGAAGAAACTCCTGTTACTTTAGTTAACCAGTGCCAG

TCCCCTGCTCACTCCAAACCCAGGAATTCTGCCCAGTTGATGGGGACACGGTGGGAACCAGCTTCTGCTG

CCTTCACAACCAGGCGCCAGTCCTGTCCATGGGTTATCTCGCAAACCCCAGAGGATCTCTGGGAGGAATG

CTACTATTAACCCTATTTCACAAACAAGGAAATAGAAGAGCTCAAAGAGGTTATGTAACTTATCTGTAGC

CACGCAGATAATACAAAGCAGCAATCTGGACCCATTCTGTTCAAAACACTTAACCCTTCGCTATCATGCC

TTGGTTCATCTGGGTCTAATGTGCTGAGATCAAGAAGGTTTAGGACCTAATGGACAGACTCAAGTCATAA

CAATGCTAAGCTCTATTTGTGTCCCAAGCACTCCTAAGCATTTTATCCCTAACTCTACATCAACCCCATG

AAGGAGATACTGTTGATTTCCCCATATTAGAAGTAGAGAGGGAAGCTGAGGCACACAAAGACTCATCCAC

ATGCCCAAGATTCACTGATAGGGAAAAGTGGAAGCGAGATTTGAACCCAGGCTGTTTACTCCTAACCTGT

CCAAGCCACCTCTCAGACGACGGTAGGAATCAGCTGGCTGCTTGTGAGTACAGGAGTTACAGTCCAGTGG

GTTATGTTTTTTAAGTCTCAACATCTAAGCCTGGTCAGGCATCAGTTCCCCTTTTTTTGTGATTTATTTT

GTTTTTATTTTGTTGTTCATTGTTTAATTTTTCCTTTTACAATGAGAAGGTCACCATCTTGACTCCTACC

TTAGCCATTTGTTGAATCAGACTCATGACGGCTCCTGGGAAGAAGCCAGTTCAGATCATAAAATAAAACA

TATTTATTCTTTGTCATGGGAGTCATTATTTTAGAAACTACAAACTCTCCTTGCTTCCATCCTTTTTTAC

ATACTCATGACACATGCTCATCCTGAGTCCTTGAAAAGGTATTTTTGAACATGTGTATTAATTATAAGCC

TCTGAAAACCTATGGCCCAAACCAGAAATGATGTTGATTATATAGGTAAATGAAGGATGCTATTGCTGTT

CTAATTACCTCATTGTCTCAGTCTCAAAGTAGGTCTTCAGCTCCCTGTACTTTGGGATTTTAATCTACCA

CCACCCATAAATCAATAAATAATTACTTTCTTTGA

>NM_138386.3 Homo sapiens nuclear assembly factor 1 ribonucleoprotein (NAF1), transcript variant 1, mRNA

GTGTCTTTTGGCTCCTCCGCGTGTAGTTACCTGAGAAACGCGGGAAGTTGGGCCCAGGCAGTGTTGCTGC

GGTTGCCTAAGTTGTTTTTCTATTTCTGGAGAGAGCCGTGAGCTTGTCCAGGGGCCCCAATCCTGAGGCC

GACCCGGTTTCTGGCGCGGTGCGATGGAGGTAGTGGAGGCCGCCGCCGCTCAGCTGGAAACTCTGAAATT

CAATGGCACCGACTTTGGAGTTGGGGAAGGTCCGGCGGCTCCGTCTCCGGGCTCTGCCCCTGTGCCAGGG

ACACAGCCGCCGCTACAGTCGTTTGAGGGGTCCCCGGACGCTGGGCAGACCGTGGAGGTTAAGCCTGCCG

GGGAGCAGCCTCTGCAGCCCGTTCTGAACGCCGTCGCGGCCGGGACCCCGGCGCCGCAGCCACAGCCACC

GGCTGAATCGCCGGCCTGCGGAGACTGCGTCACCTCCCCAGGAGCCGCAGAGCCTGCGCGGGCGCCGGAC

TCCTTGGAGACCTCGGACTCGGATTCGGACTCGGACAGTGAAACAGATTCAGATAGTTCAAGTTCATCGT

CTTCCTCTTCATCTTCCTCATCGTCGTCTTCTTCCTCTTGTATATCACTTCCTCCAGTGCTGTCAGATGG

AGATGATGATTTACAAATTGAGAAGGAAAATAAGAATTTTCCTCTTAAAACAAAAGATGAATTACTTCTT

AATGAACTGCCTTCTGTTGAAGAACTCACTATTATTCTGCCTGAAGATATTGAGTTAAAGCCTCTTGGGA

TGGTTTCAAGTATTATTGAACAACTAGTAATAATTGAATCTATGACTAACCTACCTCCAGTTAATGAGGA

GACTGTAATTTTTAAAAGTGATCGACAAGCAGCAGGAAAGATATTCGAGATATTTGGACCTGTTGCACAT

CCATTTTATGTGTTACGGTTTAATTCTTCAGATCACATTGAGAGTAAAGGTATTAAAATAAAGGAGACTA

TGTATTTTGCTCCATCAATGAAAGATTTCACTCAATATATATTCACAGAAAAACTAAAACAGGATAAGGG

ATCAGATGCATCATGGAAGAATGATCAGGAACCACCACCAGAGGCCTTAGATTTTAGTGATGATGAAAAA

GAGAAGGAAGCCAAACAGAGGAAAAAATCTCAGATTCAAGGCCGGAAAAAACTCAAATCTGAATTTAATG

AGCCTGGTGAAGATTTTACTGAAGTACATCAGAATTGGAATGCTCATAGCTCTGCTTCAGAGCATGCAAA

AGGATATCGTAACAGAGAATTCACACGAGGATTTTCCAGGGCCAGATACCCTCGATCTTGCCATGGCAGG

CCTCCACCTCAGCATTTCTATAACTCAGAACATATGGTATCTCAGGAGACTTCAGGATTTCCTTCTCAGA

GACAAAATAATCCCATTATGCCACAATACCCCTTTCCTCTTCCAGTGTTTGACATGCATAATTTTCCCCT

CCGCCCTCCACCCCCACCACCACCCCCACCTGTAAACATGGGTTGGGCTACACCAAACATGGCTGCTCAT

CCATTACTTAACTTACCATACTCCCTACCCCCACCCCCTCCCCCTCCACCACTGCCTCCTCCACCCTCTT

CTGGAGATAGTAATTCTCATTTTGGACCTTACTATTAGGTGACTATGCATTTCCAGAGAAATGTGGACTT

TTCATATTATGTGGTAAGGATTTTTTTTTTTCAAGAGTGATTATGGAGCTAGATTTTTAAAAACACTGTA

AAATACTAAATGATTCCTTCTGTTGTAAGTTGATATATATTTGTAACCTTTGTGAAATTGTATTCATATG

AAAATGTCAGCTCAAATTCTTGGGAGAACATTAAATTATGTAATATTTAATTAAAA

>NM_005746.3 Homo sapiens nicotinamide phosphoribosyltransferase (NAMPT), mRNA

AGTGACTTAAGCAACGGAGCGCGGTGAAGCTCATTTTTCTCCTTCCTCGCAGCCGCGCCAGGGAGCTCGC

GGCGCGCGGCCCCTGTCCTCCGGCCCGAGATGAATCCTGCGGCAGAAGCCGAGTTCAACATCCTCCTGGC

CACCGACTCCTACAAGGTTACTCACTATAAACAATATCCACCCAACACAAGCAAAGTTTATTCCTACTTT

GAATGCCGTGAAAAGAAGACAGAAAACTCCAAATTAAGGAAGGTGAAATATGAGGAAACAGTATTTTATG

GGTTGCAGTACATTCTTAATAAGTACTTAAAAGGTAAAGTAGTAACCAAAGAGAAAATCCAGGAAGCCAA

AGATGTCTACAAAGAACATTTCCAAGATGATGTCTTTAATGAAAAGGGATGGAACTACATTCTTGAGAAG

TATGATGGGCATCTTCCAATAGAAATAAAAGCTGTTCCTGAGGGCTTTGTCATTCCCAGAGGAAATGTTC

TCTTCACGGTGGAAAACACAGATCCAGAGTGTTACTGGCTTACAAATTGGATTGAGACTATTCTTGTTCA

GTCCTGGTATCCAATCACAGTGGCCACAAATTCTAGAGAGCAGAAGAAAATATTGGCCAAATATTTGTTA

GAAACTTCTGGTAACTTAGATGGTCTGGAATACAAGTTACATGATTTTGGCTACAGAGGAGTCTCTTCCC

AAGAGACTGCTGGCATAGGAGCATCTGCTCACTTGGTTAACTTCAAAGGAACAGATACAGTAGCAGGACT

TGCTCTAATTAAAAAATATTATGGAACGAAAGATCCTGTTCCAGGCTATTCTGTTCCAGCAGCAGAACAC

AGTACCATAACAGCTTGGGGGAAAGACCATGAAAAAGATGCTTTTGAACATATTGTAACACAGTTTTCAT

CAGTGCCTGTATCTGTGGTCAGCGATAGCTATGACATTTATAATGCGTGTGAGAAAATATGGGGTGAAGA

TCTAAGACATTTAATAGTATCAAGAAGTACACAGGCACCACTAATAATCAGACCTGATTCTGGAAACCCT

CTTGACACTGTGTTAAAGGTTTTGGAGATTTTAGGTAAGAAGTTTCCTGTTACTGAGAACTCAAAGGGTT

ACAAGTTGCTGCCACCTTATCTTAGAGTTATTCAAGGGGATGGAGTAGATATTAATACCTTACAAGAGAT

TGTAGAAGGCATGAAACAAAAAATGTGGAGTATTGAAAATATTGCCTTCGGTTCTGGTGGAGGTTTGCTA

CAGAAGTTGACAAGAGATCTCTTGAATTGTTCCTTCAAGTGTAGCTATGTTGTAACTAATGGCCTTGGGA

TTAACGTCTTCAAGGACCCAGTTGCTGATCCCAACAAAAGGTCCAAAAAGGGCCGATTATCTTTACATAG

GACGCCAGCAGGGAATTTTGTTACACTGGAGGAAGGAAAAGGAGACCTTGAGGAATATGGTCAGGATCTT

CTCCATACTGTCTTCAAGAATGGCAAGGTGACAAAAAGCTATTCATTTGATGAAATAAGAAAAAATGCAC

AGCTGAATATTGAACTGGAAGCAGCACATCATTAGGCTTTATGACTGGGTGTGTGTTGTGTGTATGTAAT

ACATAATGTTTATTGTACAGATGTGTGGGGTTTGTGTTTTATGATACATTACAGCCAAATTATTTGTTGG

TTTATGGACATACTGCCCTTTCATTTTTTTTCTTTTCCAGTGTTTAGGTGATCTCAAATTAGGAAATGCA

TTTAACCATGTAAAAGATGAGTGCTAAAGTAAGCTTTTTAGGGCCCTTTGCCAATAGGTAGTCATTCAAT

CTGGTATTGATCTTTTCACAAATAACAGAACTGAGAAACTTTTATATATAACTGATGATCACATAAAACA

GATTTGCATAAAATTACCATGATTGCTTTATGTTTATATTTAACTTGTATTTTTGTACAAACAAGATTGT

GTAAGATATATTTGAAGTTTCAGTGATTTAACAGTCTTTCCAACTTTTCATGATTTTTATGAGCACAGAC

TTTCAAGAAAATACTTGAAAATAAATTACATTGCCTTTTGTCCATTAATCAGCAAATAAAACATGGCCTT

AACAAAGTTGTTTGTGTTATTGTACAATTTGAAAATTATGTCGGGACATACCCTATAGAATTACTAACCT

TACTGCCCCTTGTAGAATATGTATTAATCATTCTACATTAAAGAAAATAATGGTTCTTACTGGAATGTCT

AGGCACTGTACAGTTATTATATATCTTGGTTGTTGTATTGTACCAGTGAAATGCCAAATTTGAAAGGCCT

GTACTGCAATTTTATATGTCAGAGATTGCCTGTGGCTCTAATATGCACCTCAAGATTTTAAGGAGATAAT

GTTTTTAGAGAGAATTTCTGCTTCCACTATAGAATATATACATAAATGTAAAATACTTACAAAAGTGGAA

GTAGTGTATTTTAAAGTAATTACACTTCTGAATTTATTTTTCATATTCTATAGTTGGTATGACTTAAATG

AATTACTGGAGTGGGTAGTGAGTGTACTTAAATGTTTCAATTCTGTTATATTTTTTATTAAGTTTTTAAA

AAATTAAATTGGATATTAAATTGTATGGACATCATTTATTAATTTTAAACTGAATGCCCTCAATAAGTAA

TACTGAAGCACATTCTTAAATGAAGATAAATTATCTCCAATGAAAAGCATGACATGTGTTTCAATAGAAG

AATCTTAAGTTGGCTAAATTCAAAGTGCTTGACATCAAAATGTTCTAGAGTGATTAGCTACTAGATTCTG

AATCATACATCACATCTGACTAGAGACCAGTTTCTTTCGAATGATTCTTTTATGTATGTAGATCTGTTCT

TCTGAGGCAGCGGTTGGCCAACTATAGCCCAAAGGCCAAATTTGGACTTCTTTTTATAAATGCAGATTGT

CTATGGCTGCTTTCCCACTACTCCAGCCTAAGGTAAACAGCTGCAATAGAAGCCAAATGAGAATCGCAAA

GCCCAAAATGTTTATTAACCTGCCCTTTACACAAAATTACACAAAAAGTTTCCTGATCTCTGTTCTAAGA

AAAGGAGTGTGCCTTGCATTTAAAAGGAAATGTTGGTTTCTAGGGAAGGGAGGAGGCTAAATAATTGATA

CGGAATTTTCCTCTTTTGTCTTCTTTTTTCTCACTTAAGAATCCGATACTGGAAGACTGATTTAGAAAAG

TTTTTAACATGACATTAAATGTGAAATTTTAAAAATTGAAAAGCCATAAATCATCTGTTTTAAATAGTTA

CATGAGAAAATGATCACTAGAATAACCTAATTAGAAGTGTTATCTTCATTAAATGTTTTTTGTAAGTGGT

ATTAGAAAGAATATGTTTTTCAGATGGTTCTTTAAACATGTAGTGAGAACAATAAGCATTATTCACTTTT

AGTAAGTCTTCTGTAATCCATGATATAAAATAATTTTAAAATGATTTTTTAATGTATTTGAGTAAAGATG

AGTAGTATTAAGAAAAACACACATTTCTTCACAAAATGTGCTAAGGGGCGTGTAAAGAATCAAAAGAAAC

TATTACCAATAATAGTTTTGATAATCACCCATAATTTTGTGTTTAAACATTGAAATTATAGTACAGACAG

TATTCTCTGTGTTCTGTGAATTTCAGCAGCTTCAGAATAGAGTTTAATTTAGAAATTTGCAGTGAAAAAA

GCTATCTCTTTGTTCACAACCATAAATCAGGAGATGGAGATTAATTCTATTGGCTCTTAGTCACTTGGAA

CTGATTAATTCTGACTTTCTGTCACTAAGCACTTGGTATTTGGCCATCTCCATTCTGAGCACCAAACGGT

TAACACGAATGTCCACTAGAACTCTGCTGTGTGTCACCCTTAAATCAGTCTAAATCTTCCAGACAAAAGC

AAATGGCATTTATGGATTTAAGTCATTAGATTTTCAACTGACATTAATTAATCCCTCTTGATTGATTATA

TCATCAAGTATTTATATCTTAAATAGGAGGTAGGATTTCTGTGTTAAGACTCTTATTTGTACCCTATAAT

TAAAGTAAAATGTTTTTTATGAGTATCCCTTGTTTTCCCTTCTTAAATTGTTATCAAACAATTTTTATAA

TGAAATCTATCTTGGAAAATTAGAAAGAAAAATGGCAAGGTATTTATTGTTCTGTTTGCCATAATTTAGA

ACTCACACTTAAGTATTTTGTAGTTTTACATTCCTTTTTAACCCATTCAGTGGAGAATGTCAGCTTTTCT

CCCAAGTTGTATGTTAAGTCTATTCTAATATGTACTCAACATCAAGTTATAAACATGTAATAAACATGGA

AATAAAGTTTAGCTCTATTA

>NM_005899.5 Homo sapiens NBR1 autophagy cargo receptor (NBR1), transcript variant 1, mRNA

AGATTCACCCCACAGGGATAGCGGCAGAGCCGGTAGCGGACGGTCCTTGCATTGGCCTCCGGCAGGCGCC

CCCCGGGGGCGGGAAGCTGCCTCACAGCATGGAACCACAGGTTACTCTAAATGTGACTTTTAAAAATGAA

ATTCAAAGCTTTCTGGTTTCTGATCCAGAAAATACAACTTGGGCTGATATCGAAGCTATGGTAAAAGTTT

CATTTGATCTGAATACTATTCAAATAAAATACCTGGATGAGGAAAATGAAGAGGTATCCATCAACAGTCA

AGGAGAATATGAAGAAGCGCTTAAGATGGCAGTTAAACAGGGAAACCAACTGCAGATGCAAGTCCACGAA

GGGCACCATGTCGTTGATGAAGCCCCACCCCCAGTTGTAGGAGCAAAACGACTAGCTGCCAGGGCAGGGA

AGAAGCCACTTGCACATTACTCTTCACTGGTGAGAGTCTTGGGATCAGACATGAAGACCCCAGAGGATCC

TGCAGTGCAGTCGTTTCCACTTGTTCCATGTGACACAGACCAGCCTCAAGACAAGCCCCCAGACTGGTTC

ACAAGCTACCTGGAGACGTTCAGAGAACAAGTGGTTAACGAAACGGTTGAGAAGCTTGAACAGAAATTAC

ATGAAAAGCTTGTCCTCCAGAACCCATCCTTGGGTTCTTGTCCCTCAGAAGTCTCAATGCCTACTTCAGA

AGAAACATTGTTTTTGCCAGAAAACCAGTTCAGCTGGCATATTGCTTGCAACAACTGCCAAAGAAGGATT

GTTGGTGTCCGCTACCAGTGTAGCCTATGCCCATCCTACAATATCTGTGAAGATTGTGAAGCAGGGCCAT

ATGGCCATGACACTAACCACGTCCTGCTGAAGTTGCGGAGACCTGTTGTGGGCTCCTCTGAACCGTTCTG

TCACTCAAAGTACTCTACTCCTCGTCTTCCTGCTGCTCTGGAACAAGTCAGGCTCCAGAAACAGGTTGAT

AAGAACTTTCTTAAAGCAGAAAAGCAAAGGTTGCGAGCTGAGAAGAAACAACGTAAAGCAGAGGTCAAGG

AACTTAAAAAGCAGCTTAAACTCCATAGGAAAATTCACCTGTGGAATTCAATCCATGGACTCCAGAGCCC

CAAGTCTCCTTTAGGCCGACCTGAGAGCTTGCTCCAGTCTAATACCCTGATGCTCCCTTTGCAGCCCTGT

ACCTCCGTTATGCCAATGCTCAGTGCAGCATTTGTGGATGAGAATTTGCCTGATGGGACTCACCTTCAGC

CAGGAACCAAGTTTATCAAACACTGGAGGATGAAAAATACAGGAAATGTAAAGTGGAGTGCAGACACAAA

GCTCAAGTTCATGTGGGGAAACCTGACTTTGGCTTCCACAGAAAAGAAGGATGTTTTGGTTCCCTGCCTC

AAGGCCGGCCATGTGGGAGTTGTATCTGTGGAGTTCATTGCCCCAGCCTTGGAGGGAACGTATACTTCCC

ATTGGCGTCTTTCTCACAAAGGCCAGCAATTTGGGCCTCGGGTCTGGTGCAGTATCATAGTAGATCCTTT

CCCCTCCGAAGAGAGCCCTGATAACATTGAAAAGGGCATGATCAGCTCAAGCAAAACTGATGATCTCACC

TGCCAGCAAGAGGAAACTTTTCTTCTGGCTAAAGAAGAAAGACAGCTTGGTGAAGTGACTGAGCAGACAG

AAGGGACAGCAGCCTGCATCCCACAGAAGGCAAAAAATGTTGCCAGTGAGAGGGAGCTCTACATCCCATC

TGTGGATCTTCTGACTGCCCAGGACCTGCTGTCCTTTGAGCTGTTGGATATAAACATTGTTCAAGAGTTG

GAGAGAGTGCCCCACAACACCCCTGTGGATGTGACTCCCTGCATGTCTCCTCTGCCACATGACAGTCCTT

TAATAGAGAAGCCAGGCTTGGGGCAGATAGAGGAAGAGAATGAAGGGGCAGGATTTAAAGCACTTCCTGA

TTCTATGGTGTCAGTAAAGAGGAAGGCTGAGAACATTGCTTCTGTGGAGGAAGCAGAAGAAGACCTGAGT

GGGACCCAGTTTGTGTGTGAGACAGTAATCCGATCCCTTACCTTGGATGCTGCCCCAGACCACAACCCTC

CTTGCAGACAGAAGTCCTTGCAGATGACATTTGCCTTGCCTGAAGGACCACTTGGAAATGAGAAGGAGGA

GATTATCCATATCGCTGAGGAAGAAGCTGTCATGGAGGAGGAGGAGGATGAGGAGGATGAGGAGGAGGAG

GATGAGCTCAAAGATGAAGTTCAAAGTCAGTCCTCTGCTTCCTCAGAGGATTACATCATCATCCTGCCTG

AGTGCTTTGATACCAGCCGCCCCCTGGGGGATTCTATGTACAGCTCTGCGCTCTCACAGCCAGGCCTGGA

GCGAGGTGCTGAAGGCAAGCCTGGGGTTGAGGCTGGGCAGGAACCAGCTGAGGCTGGGGAAAGACTCCCT

GGAGGGGAGAACCAGCCACAGGAGCACAGCATAAGTGACATCCTCACGACCTCACAGACTCTGGAAACAG

TGCCCCTAATCCCAGAGGTAGTGGAGCTTCCACCGTCACTGCCCAGGAGCTCTCCTTGTGTACATCATCA

TGGTTCCCCAGGAGTGGATTTACCAGTTACCATACCAGAAGTTTCTTCAGTCCCTGATCAGATCAGAGGA

GAGCCCAGAGGCTCATCAGGACTTGTAAACAGCAGACAGAAGAGCTATGACCACTCAAGGCACCATCATG

GGAGCAGCATTGCTGGAGGACTGGTGAAGGGGGCTTTGTCTGTTGCTGCCTCTGCATACAAGGCCCTGTT

TGCTGGGCCACCAGTCACTGCACAGCCAATAATTTCTGAAGATCAGACAGCAGCCCTGATGGCCCATCTC

TTTGAAATGGGATTCTGTGACAGGCAGCTGAACCTACGGCTGCTGAAGAAACACAATTACAATATCCTGC

AGGTTGTGACAGAACTTCTTCAGTTAAACAACAACGACTGGTACAGCCAACGCTATTGAGGAGTGACCTT

GTATTAAATAACTGCCTGCTGCTCAGAGATGATCTTTATTCTGTCATTGGGGTATGGGATAGAAGCCCTT

GCTTATTTTTAATCTGATGAATCTGTATAGAGCCCATCGTTGAGTTACCAAGACAATACCTGCTACAGTA

TTTTGGGGAGCAAACTAAAGACCAGAACTTAAATTTTCACTTTAGACATTGGATGAATAGTATGAAGACA

GTTTTTCAGTTGATTTGGATAAAACTATTTTAGTGCATTGACAAGTGTAACTTCAACTTCATATAGAACC

ATTTTTCTTTCTGCTTTTATTGAAACTGAGTATTTTTCTTTGGCTAATGTGGATTTTTTATGGGGATATC

TGTTAATTTTCAGGTTTTGAAAGACATTAACCTCGGAAGTTGTTTTTAAGAATTATTCTCATAATTCTTA

TTCTCATAATTTCTGTAATCCACCTCAAGCTTCATAGTTATTTGGCATTGAAATAACACCCAGAGCATGA

TAGAAATGTTGTTACTCTTCCTCTCTCAAGGAGAAAGTAATTTTCCTGCAATACTTAATAATTGGCACCG

TTGCTTTCTAAAGACTCCATGGTGCATTCAAGAGTATCCAACTTCAAGGGAATCTCCGCATTTCAATGAA

AGGAGGAAGAGTGTGCTGATAAACCTACCAGCACCTATTGAGCAATGTCTATTATAGTAATTTTGCATAC

ATTTTTATTTAAGGGAAAAAATATAGGTATTGTGAAATATTTTGCTAATCTTATAGAAAAGGAAAAAATC

CCGTTATTTAAAGGGAAAAGTAAATTTAACAGTTGCCTTTTTTCTTAATGTCAGGGCAGATCTTATTTTA

CAGTACAGTGGGGGAAATAGAAACATGTGAAAGGCAAAAGGCAGGCTCCTAAATTAATGTCAGTGAAGTT

CAGGGTGGGCAAATGAGTGTGTGTGAGGTATAGGAAATGCTGATGACTTCTTTAATGCTTGAAGTCCGTT

CACAGGTATCTAGCCCTAGAATGCCTAGAACAGGAAGAGGCAGCTGGTGTTCTGCAAAACTTGGACAGGG

GCAAAGTTGCTGAAAAAGTTTTGGTTTAACCCGAAGATAAGTGGAAAAGAGCTTGTCCATGAACCCAGGT

TCTCACTCTGTTTACAGAAGTGTGTTGAGTACAGTTGGTGAAGGAAGAGGTAACAAAAAATGCTAAATAT

TTTATCCATGAAAATGACTTCCAGAAAAGGAAGAATATGAACCCCAGACCGAAGGGGAAAAGATAGTTAA

TAGTATTATCTAACCTGGTTGGTATTTGTAATGAATGGTGATTTTAATTAGTCATTAGCCATAATGATGT

TTATTTACAGTATAACTCCTGAATGCTACTTAAATAAACCAGGATTCAAACTGCAAGCCAGCCAGGCCGT

TCATTATTTAAAACGTTTTAATCGGGGCTTCCGGGTAGAAGGTGGAGCGGCAGGGTGTAATTGGGTTGAT

GGGTGGGACCTGTCTTGACCATCGGAGTTTTATAATCGAGGGCCAGGAGGGCCCGGGTTGCTCTCCTGGT

TATGTATGTACTTGTACATAACCACCTAAAGAATGGTGAAATAAATGTTCTTGGAAATTCCTA

>NM_205842.3 Homo sapiens NCK associated protein 1 (NCKAP1), transcript variant 2, mRNA

CTCAGCCGCCGGAGACGCCGCCGCCGCCGCCGCCGCCGCCACACCTAGTGGAGCAGCCGGGGAAGGCGGC

TCGTTGGGGGCTGGGGCGGAGAGCGCCGGGGGTGGGGGCGGAAGGGCGGCGGGCGGAAGGCAGGAGGCTG

CCGGGGCGCGGGCTGCTGCGGGAGAAGGGGCTCCGAGGAGTCCGCCGCGGCTCGCTCTGTCGCCGGCGCG

GGATTGGGGCGCGAGGGCCATGGGCGCGCTCTCCTAAGGCGGAGGTCGCGGGCGGGAGGGGAGGAGGCCC

GAGAGAGGCTGCTGCGAAGGCCGCGGGCCCGTGACTGGGCGCGAGGCGGCCGGCGGCGGCGGCACCA

GCACCACCATGTCGCGCTCAGTGCTGCAGCCCAGTCAGCAGAAGCTGGCGGAGAAGCTCACCATCCTCAA

CGACCGGGGCGTCGGCATGCTCACCCGCCTCTACAACATCAAGAAGCAAGGACAAGTTTGGAAGGCATGT

GGAGACCCCAAGGCAAAACCATCCTATCTTATCGACAAAAACCTGGAATCTGCTGTGAAATTCATAGTCA

GAAAATTCCCTGCTGTAGAAACCCGCAACAACAATCAACAGCTTGCACAACTACAGAAAGAAAAATCAGA

GATTCTGAAAAATCTGGCATTATATTACTTCACATTTGTAGATGTTATGGAATTTAAGGACCATGTTTGT

GAATTGCTGAATACTATTGACGTTTGCCAAGTCTTCTTTGATATTACTGTAAACTTTGATTTAACAAAGA

ACTACTTAGATTTAATTATAACCTATACAACACTAATGATACTGCTGTCTCGAATTGAAGAAAGGAAGGC

AATCATTGGATTATACAACTATGCCCATGAAATGACTCATGGAGCAAGTGACAGAGAATACCCACGCCTT

GGCCAGATGATTGTGGATTATGAAAACCCTTTAAAGAAGATGATGGAAGAATTTGTACCCCATAGCAAGT

CTCTTTCAGATGCACTAATTTCTCTTCAAATGGTATATCCTCGAAGGAATCTTTCAGCTGACCAGTGGAG

AAATGCCCAGTTATTGAGCCTCATCAGTGCACCTAGTACAATGCTTAATCCAGCACAGTCCGACACTATG

CCTTGTGAATACCTCTCTTTGGATGCAATGGAAAAGTGGATTATCTTTGGCTTTATTTTGTGCCATGGGA

TCCTAAATACTGACGCTACAGCACTGAACCTTTGGAAACTAGCTCTTCAAAGTAGCTCTTGCCTCTCTCT

CTTTCGGGATGAAGTTTTCCACATTCACAAAGCTGCAGAAGACTTATTTGTAAACATACGAGGCTATAAT

AAACGTATTAATGACATAAGAGAATGCAAGGAGGCAGCCGTGTCACATGCTGGTTCAATGCACAGAGAAA

GACGCAAGTTTTTAAGATCTGCACTGAAGGAATTGGCTACTGTCCTCTCTGATCAACCTGGATTGCTAGG

TCCCAAGGCACTTTTTGTTTTTATGGCATTATCCTTTGCCCGTGATGAAATCATCTGGCTACTTCGTCAT

GCAGATAACATGCCAAAGAAGAGTGCAGACGACTTTATAGATAAGCACATTGCTGAATTAATATTTTACA

TGGAAGAACTTAGAGCACATGTGAGGAAATACGGACCTGTAATGCAGAGGTATTACGTGCAGTACCTTTC

TGGCTTTGATGCTGTTGTCCTCAATGAACTCGTGCAGAATCTTTCTGTTTGCCCTGAAGATGAATCAATC

ATCATGTCCTCTTTTGTTAACACTATGACTTCCCTAAGTGTAAAACAAGTTGAAGATGGGGAAGTATTTG

ATTTCAGAGGAATGAGATTAGATTGGTTTAGGTTACAGGCATATACTAGTGTCTCAAAGGCTTCACTTGG

CCTTGCAGATCACAGAGAACTTGGAAAGATGATGAATACAATAATTTTTCATACAAAAATGGTAGATTCC

TTGGTGGAAATGTTGGTGGAAACATCAGATCTCTCCATATTTTGTTTTTATAGTCGTGCTTTTGAGAAGA

TGTTTCAACAGTGTTTGGAGTTACCCTCTCAATCAAGATACTCAATTGCATTTCCACTACTTTGCACTCA

TTTTATGAGTTGCACGCATGAACTATGTCCAGAAGAGCGACATCATATTGGAGATCGCAGTCTTTCCTTA

TGTAATATGTTCCTAGATGAAATGGCCAAACAAGCTCGAAATCTCATCACTGATATTTGCACAGAACAGT

GTACCCTTAGTGACCAGTTGCTACCCAAGCATTGTGCCAAAACTATCAGTCAAGCAGTGAATAAGAAATC

AAAAAAGCAGACTGGTAAGAAAGGGGAACCTGAAAGGGAGAAACCAGGTGTTGAGAGCATGAGGAAAAAC

AGGCTGGTTGTGACCAACCTTGATAAATTGCACACTGCACTTTCTGAGTTATGCTTCTCTATAAATTATG

TACCAAACATGGTGGTATGGGAACATACCTTTACCCCACGAGAATATTTGACTTCTCATCTGGAAATACG

CTTTACCAAGTCAATTGTTGGGATGACTATGTATAATCAAGCCACACAGGAAATTGCAAAACCTTCAGAA

CTTCTAACAAGTGTAAGAGCATACATGACCGTACTCCAGTCAATAGAAAACTATGTGCAGATTGATATTA

CAAGAGTATTTAATAATGTGCTTCTTCAACAAACACAACATTTAGACAGTCATGGAGAGCCAACCATTAC

AAGTCTATACACAAATTGGTATTTGGAAACTTTGTTACGACAAGTCAGCAATGGCCATATAGCATATTTT

CCTGCAATGAAAGCGTTTGTGAACTTACCTACAGAAAATGAATTAACATTCAATGCAGAGGAATATTCTG

ACATATCAGAAATGAGGTCATTATCAGAACTACTAGGCCCATATGGTATGAAGTTTCTAAGTGAAAGCCT

TATGTGGCATATTTCATCACAAGTTGCTGAACTTAAGAAACTTGTGGTGGAGAATGTTGATGTGTTAACA

CAAATGAGGACCAGCTTTGACAAACCAGACCAGATGGCTGCACTGTTTAAAAGATTATCATCTGTTGACA

GTGTCTTGAAGAGGATGACAATAATTGGTGTAATTTTATCCTTCCGATCATTGGCACAAGAAGCACTTAG

AGATGTCTTATCCTACCACATTCCTTTTCTTGTAAGTTCAATTGAAGATTTTAAGGATCACATTCCAAGG

GAAACTGATATGAAGGTTGCAATGAATGTGTATGAGTTATCATCAGCTGCCGGATTACCTTGTGAGATTG

ATCCTGCATTGGTCGTAGCTCTTTCTTCACAAAAATCGGAAAACATTAGTCCAGAAGAAGAGTATAAAAT

TGCCTGCCTTCTCATGGTGTTTGTGGCAGTTTCTTTGCCAACACTGGCCAGTAATGTGATGTCTCAGTAC

AGCCCTGCTATAGAAGGGCATTGCAACAACATACATTGCTTGGCCAAAGCCATCAACCAGATTGCTGCAG

CTTTGTTTACAATTCACAAAGGAAGCATTGAAGACCGTCTTAAAGAATTTCTGGCGCTTGCATCCTCCAG

TCTACTGAAAATTGGCCAGGAGACAGATAAAACTACAACAAGAAATAGAGAATCTGTTTATTTACTGCTA

GATATGATTGTACAAGAATCTCCATTCCTTACAATGGATCTTTTGGAATCTTGTTTTCCTTATGTCTTGC

TGAGAAATGCATACCATGCTGTCTACAAACAAAGTGTTACATCTTCTGCATAAAATTACCTACTTAATCA

AGATAAGCACGCATTTTTGTTGCCTTGGTTTTACCTGTAGACTGTGGAACTATTTTACCTTAAGACCTGA

AAAAGTTTTGTGGATTATAAATTTCTTTCATACGGTTGTATTTTCTGATCATTGGTTTCTTAATATGGTT

GTACTACAGTATACTTGGTTGATTTAGGTTGCACATTCACTGAATTCACTGAGATTATTCCTATAATTTT

AAAGTATCATTTATTTGAAAAACATACATTATCAACATGTTTTTGATATTTGATAATGAAAAAAATCTTT

GCTTGTTTATTTCTGAAAAAGAACTGTATTTAGTGATTATTTTAGATAGTGATATTATAGTATTCATCTG

TGTGTAAATTATTTCATATAGGGAAGAGTTCTGATCTGTACCTATGGTTCTTATTGAAAACAACATTGGA

TGTGCATTTCTGTGATGTTATGAATACATTTCTACTTTATTTTGAAACATTTGCCAAACTAAATACTGTA

ACACTGTATAACATTTAAAAATGTTAAAGAACTGCTTAGTATTAGAAGCAGATCATTTCCCAAAATTCTA

AGAGCAGCAGCATATGTTGTTGCTTGTATAAAGCCTAGCGATAATTTTTAGACTAACTTCCATGGTGCCC

TGTTGGCATTAGCACTACCATTGTACCCTGCTGTATAATAAACAATCTTAGACATTTATCAACTGTTGAT

ACAAATGTTAGTCCCTAACCACTTTTTATATATGTTTTAAATTTTTGAAATTCAAGTGTACCTGCCATAA

CATAAAATAAACACTAGACTGTATCACACTTCGATTGATTTCTTTAAGATCCTTGGATATTCGTTCAATA

ATAATAAAGTTTTTGTTTAAACTTAGTTTGAAATATAAAAAATAATGGTTTAGGAATGTCTAAGTACAGC

ATTCGATACCAGGTCAGCTATTATATGATTTGTTTAAATGTTTTAAAAAACATTTTATTGAAAAAAATAC

CAAAATTAGAAAATAAAATTACACAATACTTCTTAAAAATTAAGAACTTAAAGAAAACTTGTATGCTGTC

AACTTGCTCATTTTATCTGTAACCAAATAGAGTTATTTAAAAGAATAGGGAAAAAAAATTGAGTGATCAT

GGGACTAGTGAGAATGTTGAGAACTGCTGCATTAGTCAGTAATGAATGGGAAAGACATAAAAGGGCCTAT

TGCTTAATAGTTGGCATGGAGGGCAAAGCAGCTCATTTAGTTTGAAAACTAACCCAATGTTTTGCTCTTT

GATTGGCATTGTTGAGGCTGTTTCTCAGCAGCTTTGTATATGAATATCATAAAGAGCTTCTGTTCACAGC

TTCTTATGTATACACTGGTAACTTGTGTTAAGATAAAAGAAGGCTGCTGTGAACTCAGTTATAATGTTTT

TCTTTCTTCAAACATAGAGAGTAGAATGAAATTGAAGTACTATGAGATTATGCTATTATTTTGTGTATGA

TTTGTCCAGCCTATATTTAAAGTAGGGAGTAGTTGTCACTATCTGTCGAAAAATTGTACTTGTTCCATAA

AGATTTTTTAAAAAGGGAAATTTTAAAATGGCATAGTTCAGACCTGAAATAGTCTGATTTACACAGTGAG

CAGTCAAAACTAAGATTTAATGCCGTTTGTTTTGAAATATTAGGATCTTAGAGAGGTAATAACTTTGTTG

GCAGTGATCTCTGGAGAGGTAAAGTAGGAATTTGGATATGTACACTTTGGAATCATATGTGAAAGGGCAT

AAGGATGAAAATAAGGCATTCTCTACCACTGCTAGGCTGAGAAAAAAATCGCTCCTCATAGTCACCTGAA

GTTTTTGTCCAAATTTTTTCTCATACAGCAAAGGCGGGGGAGGAGGCGTGGCAAAGGTAACTTGGAGGAA

AAAGTTAAAAGTAAATTGACAACTACTATTTTTAATATCTTGTTTGTTTTATTTTCTTCTAAACTTACAC

AACAACATACTATTTTTGTTGTTTTCTCTACATTAATGTGATCATACCATACATACTGGCCTGTAATCTG

CTTATTTCACTCAGTAGTAGAAAAAAATGTCTTTAATATTCTTTTTAATACCTTTATAGATACCATAACTA

ATTTAACAATTTCATGGCCAGGCGTAGTGGCACACGCCTATTTCAGCACTTTGGGAGGCCAAGGCGGATG

GATCACTTGAGGTCAGGAGTTCAACACCAGCCTGGCCAACATGGCAAAACCCTGACACTACTAAAAATAC

AAAAATTACCCAGGTATGATGGTGCACACCTATCATCCTGGCTACATGGGAGGCTGAGGTGGAAGAATTG

TTTGAACCCGGAAGGAGGAGGTTGCTGTGAGCTGAGATCACACCACTGCACTCCAGCCTGGATGACAGAA

TGAGACTGTCTCAAAAAAAAAAAGAAAATACTCATTTGGATAGATTGGATAGATTTTTAGACAATTTCTT

TTCATTATTATAAATGTATATCTATATCTTGGTAAAGTTGACCTGTTATTTTTTTAGAATAATTTTTCAG

AAGTAAAATTGCTTCCTTTCTGCTGCTTTTTCATTCACTTATTTAAGCAAGTATTTTTAGATGCCTGCAT

GTAATGGTGAGCAAAAAGGTGACCCTTTTATAGTTGAGCTTGGATTTTAGTGATGGTATACAATAAACAA

ACCAGCTTCTTGAGACGGAGTTTCACTCTTGTTGCCCAGGAAATGGGCAATGGCCTGATCTCGGCTCACT

GTAACCTCCGCCTCCCGGGTTCAAGCAATTCTCCCATCTCAGCCTCCTGAGTAGCTGAGATTACAGGCGC

CCGCCACTACGCCCAGTTAATTTTTGGTATTTTTAGTAGAGATGGGGTTTTACCATGTTGGCCAGGCTGG

TCTCGAACTCCTGACCTCAGGTGATCCACCCACCTCAGCCTCCCAAAGTGCTGGGATTACAGGCATGAGC

CACCACGCCCAGCCATGAGCAAACTTCTGATGGTGACTGGAAAGTATACAGGATAATGTGGGGAGGCAGT

GGTGAAACTACTTTAGAAGAAAGTGTTAGAAGAAAGTGTCATCTGTGTAAGAGAAAACATTTGAGCTGGT

ACGTGAAGGATTAGAAGGAACCACAAGTGGGCTGAGGGGAGGAATAATAAAGGCAAAGACCCAGAAGGCA

AGCACAAACTTGGCATGTTTGAGAAGTTAGAAGTGTGTCATCTGTGTCTCTGTCATGAGGAGTGCTCAAG

TTCCCTCTAGGAGCCTTATGGGCTATCTAGCTCTTTTTCTCTTCTGATCATGTGCTTGCTTGCTTTCTTC

TTTGCTTATATAGCAGAGTTCACAAATTAAATCTAATGTAGGCTGGTTAATAAAGGAAATGAACATGAAT

TCACTGATAGGCTCTTGCATTAATGTGAAAATGTAAAGTCTGTTGTACACCATCAGGCCTTCTAAATGAA

GCCCAAATTCTGTAAGGTAGTGAATTGTAACCTACTGATTAGTACATGATCCATGCCCATTCTGTTATAA

GGACTTGCCTTTCTGAGACTTCACCTGTATTATTCCTAAGACTTTTGTGAATAATCCTTAAAGTAGTGAC

TCCTTACATCTTTCTTTCATTTCTTCTTTGTAGTCACTTGAAATGACAGCATGAACCATCATTTCATTTG

AAGGCCTGAGCCTGAATTTTCAAATAGACAAAATGCTCTCTTTCTCCGAAGATTTGCCATGACCTCTGCC

TTTGGGAAAAAAATATTTGAGATTTTAAAATTTTTATTCTGTAGTACAAAAATTATGTTTTTCGTTAATTG

GTCCTGTGGAATTGGAAGGAAATTTAATATTTATTGACTCTACTTTGGTATTATTTTCAGAGTGAGAAGT

AACTTTAAGTGTATTAAAATTTCATTCATTTTTGCATGAACCAAACTTTCCCTACACATCGCTGTCCTTG

ACTGTCCAGAGTAAACATGTCTAACTCTGGAATGTTTCATTTCCAATAAAAGTATTCAGCCTGTCATTTT

GGGCCTAAGTAAAATGGGATAAATTAGCAATCTGAAGTTAGGATCCTAGCCACAGAGGGCCATGTCTCAT

TTCTGGTTTAAGTGCACCCCTCATCCCCAGCCCTTACCTTCTTCTTTCTCTTAAGGATTGAGACAATCCT

CTTGGTGCTCTTGGTGCAGGTGCATCTTGGTCACCTCTTACATGAGGGCCACAAACTTGGTAAATGGGTG

GCCTGGAGATGGGGTTGTACTAGGTATGTGACCTTGGTGATTGCCTTGTCTCAGTTGTAGTATATGGCTG

AGTCACATTCATAGTACACTACATACACATACTGACCAAATGCCAATGGCTAAGGCTTGAAATTATTTCA

CCAAGACCTAAACTGAAGATTACCATCATAAAATAAGCTTCTAGAATCCATGAATAAGTAGGAAATCATA

TTTAAAATTTACTGTAATTTTATTAAAGCTCATTTTTTAAATTTAATGCAGTGCAGAGTCATTAATTTCT

CAGCAGTATCAGGTGCTAAATTGCTCACTCATTAAACTACTAATTTAAGATAGTTATTTTAAATTATAGA

ACAGGAAGCAACTTACGAGGTTGCAACCAAATTATATTTATTTTTTCTCCATTAGTAACTGACTAAAAGT

GTTTTCTTCAACTCTGGGATTTGAAGATTTTGTGGTCAGTGGTGATATAATTTCACTGAAGCGTGCTCAC

AGACTAAAACTGTGCACTACTCAGTGATGCGTCCTGAATGTTTTCAGAGACAAACCTAAGATAATGTTGC

CCTTGTTTACTTTCATTATTTCTGATTTTTTTTTTATGCATGTGTTTGCTGCATCACACAATTACTTAAG

AAGTTGTCCCTAACCTTTGTGCTGTGGTCTGAATGTTTGTGTCCCCCTGGCCACAAAATTCATATGTTGA

AATCTGATCATCAGTGTGACAGGATGTGGGGCCTTTGGGAGGTGATTAGGTCATGAGAGTGGAGCCTTCA

TGAATGGGATTAATGCCCTCGTAAAAGGGGCCCCAGACAGCCGACTTGCCCCAACCACCACGTGAGGACA

AAGCAAGAAGCCACTGTTCATGAACCAGAAAGCAGACCCTCCACAAACACCAAATATGCCAGCACACTGA

TTGGCTTCCCAGCCTCCAGAACTATAAGCAAGAAGTTTTTTTATAGTAACAAATTTATTGTTCACAGTTC

TTGCTTATAAACTTTATAAACCAGACTAAGGCATTTCGTTATAGCAGCCCACACGGACTCATATTTTGGT

TCTAGAAATGTGACACTGGAATGATGTGGCTCTCAAGATGACAGTGTTGCTTCTGAATGTCTATGTTCAT

GTCATCTTTAATTGCATGAACACCAGTTTTAGCATCTTCCCTTGAGTAGAAAGAGATTCTCTACAGCAAG

TATTACGTAAGAAGTACTGGTGGCCAGGCACAGTGGTTCACGCCTGTAATCCCAACACTTTGAGAGGCGG

AGCCAGAGGTATCATTTGAGCCTAGAAGTTCGAGAGCAGCCTGGGCAATATGTCTAAACCCCATCTCTAC

AAAAAAAATACAAAAATGAAAAAGCCGGGTGTGATTGTGTACACCTGTAGTCCCAGCAACTTGAGAAGCT

GAGGTGGCAGGATTGCTTGAGCCCAAGGGGAGGGTTGGCGGCAGTGGGTGGTTCGAGGCTGCAGTGAGCC

ATGATTATACCAGTGCGCTCCAGCCTAGGTGACAGAGCAAGAGCCGGTCTCGATAATAATTAAGGCCAGA

CGCGGTGGCTCACTCCTGTGATCCCAGCACTTTGGGAGGCCAAGGTGGATGGATCACCTGAGGTCAAGAG

ATTGAGACCAACCTGGCCAACATGGTGAAACCCTGTCTCTACTAAAAATACAAAAATTAGCTGGGCTTTG

TGGCTCGCGCCTGTAATCCCAGCTACTCAGGAGGCTGAGGCGGGAGAATCGCTTGAACCCAGGAGGCGGA

GATTGCAGTGAGCCGAGATCATGCCACTGCACTCCAGCCTGGGCAACAGAACAAGACTCTGTCTCAAAAA

AAAAAAAAATTATAATACATTAAAAAAAAAAAGCACTTGCATTCTGAAGGCATATTCACCCTTGAACTGA

TAGCATTCAATGTATAAAACACATGAGTTTCACTCCAAGTTACATATAGAATGTTTATCTTTATTTGAGA

GGAAATATCCAGTTAATAATCAGAATGTCATACTAATTAGGTATGACCATAACTTACGTTAGTCACATGG

TCATTGATTGCATGCAGATTCTGGCTGCCAGAAAGCACATTGAAGTAGGTAGTCATTTAACCATTTAACT

GTTAGATCTTGCAAAGGTTGAAGCTGAAGCTGAGGAGCAGGTAGTACACAACTTTACAGGTTAATGCTGA

ATGTCAGTTCATTCTGAATTCTTACCTTCAGCCAAAAGGTACAAGGGTCTTTGCAGTCTGAAGCAAGGCC

CAGATGGCAGAACTTGTGATGGCTAGGAGGGGAAAATTTGACCTTAACTGGTAGTTGTGGTTTTATCAAG

CCAAAAATGCTGACTAGAATAATAATGAGAGCTAATGTTTATTGAAAGCCCATTGTGTACCAGATACTCT

GTTTTCTCTAATTCAAACCTCTAAATATTGCTCTTTTAGTCTTCACAGTAATCCTATATCATGATGACCC

CCATTTATTGATATGGAAACTGAAGCTCAGGAAAGACAAAGCAATTTTTACAAACTCACACTACAAGGAA

GTAGCAGAGGCCAACATTTAAATCTGGCCCCAGAGTTGTGTTCTTCTCATTAAACTGTGTCTCAGATCTC

TCAAATGTGAGTGTGGACTTTTCTAACCAATAGCATTTTTTAATTAATGTTTTAAAAATTTGAATAGCTT

TTAGGGTACAGTGGGTTTTTGTTTACATGGATGAATCATACATTGGTGAAGTCTGAGGTTTTAGTGTACC

TGTCACCCAGGTAGTATGCATTGTACCCAATGTGTAGTTTTTTATCCCTAGACCCGTTCCCATCCTCCCA

CTTCTGAGTCTCCAAAGTCCATGATATCACTCTGTATGCCTTTGCATACACATAGCTTAGCTCCCACTTA

CAAGTGAGAACATACGGTATTTGGTTTTCTGTTCCTGAGTTACTTTGCTTAGAATAATGGCCTCTGGCTC

CATCATAGTTGCTGCAAAAAGACATTGTTTCTTTTTTTATGGCTGAACAGTATTCCGTGGTGTGTATATA

TACCACACTTTCTTTGTCCACTCATTGGTCAGTGGGCACTTAGGTTGGTTCCATATCTTTGCAGTTATGA

ATTGTGCTGCAATAAATATACACATGCAGATTTTTTTTGATATATTGACTTCCTTTGGGTAGATACCCAG

TAGTAGGATTGCTGGATCAAATTCTATCCAGTAGCATTTGAAACTGCTTTACATTAACAATGAGGGACAA

AAAAAATAACAATGAGGGCTTACATTATGCTACTCTTGATACTAGACATTTTCATATCCATTACTTCATT

TAAACTCCACAACCAAATTACTCAATAGGGATTGTTTTCTTTATTTTTGTAAATGAATGTGGAAACCAGC

TCATAAAACTTTAAATTCCTTGCCCCAGTGTCATAAAATTACTGAGTTTGAGTGGCAGTGCTGAGATTAA

AACCTAAGGCTCTAGGATTTCCAGCCCATATTTTTTCTACTGTATCATGTTCTTTCCCAAAGCACTAAAA

AACATTTCCTGGGTAAATGACTGTGGGCTTGGGGACACTATGCCCACTTTCTGGCCTTCATGGGTCTAAG

TTGCTTCTTTGCTTCTTCACTTTTTCTCTCTGATCCTGCTCAGATTAATCTCTAGTAAATCAGATTTCTT

TCCTAGGCTGTCTTATCCCACATATATAGTAGATAATATGTCACATATTTGTGAATGTTAAAAATATTTG

TTTTAGTTTTAAATCTCAAGCTCACACAGTACTTACTCTCTATGTTAACAAAAATGGTTTTTACTATTTT

CTGAAGGTAACGCAGCTGTTGAAGTGAGATCCTACACTGGAACATACAACACTTGGTCTAATATTAAAGT

TCGTGCCAAGAGCTCTCTAAATGTGATTGATGGATTAATTCTGCCTTTATGAAAGATGAACTTATTTTCA

AGATAATAGCTAATTCTTGAATGGGCAACCTATTATGTATCAATGATTGTAATGATGGAGCATTTTAGTC

ATTTGTAGCTGGAAGAGCCTTAGAGATTATCTGGTTCAACCTATTCAAGAGTTAGGTAATTTAACCAAAT

TAACATATGTGGCGATGTGCTCATATAGTGTTCCTTCCACTGTGCTGTGTTGCCGCATATATGCTGATCT

TATGTTTATGCAGTAATAGTCTTCATAATGATAAACAGCTACTGGTTCCTGGTAGGATTTTGGACGTGGA

TATGACTTGAAGTATGATGCCACAGCCTCAAACAAATCTGGGGTTTGAATCCCAGCTCTGTCACTTATGG

ACAAATTACTTAACTTCTCCAAGTTTGTTTCCCTAAGTGTAGAATGTGGATAATATCTACTTTAAAGGGC

TATTGTAAGCATTAAAATAAGATCATGCGTATAAATTGATGAGATGGTGGGGAAGAAGAGGCTACCACAA

AGTAGGCATTCAGTGTAAATGGTGGTAGTTTCTCCAGTATTATTAGGCTATTTGGTTTCACTTATAAAAT

TGCCTTCAAAGAGTTTTTTCATGGCTTTTATGAGTATTATAATATCAGAACTTCCACTCATGCAAATGTA

ACATTTGTGTGTTAGTCTATTCTATAGCTCTGATCGGAGTCTTTATCTTAAATGGGAAAGATTATTTTCC

CTGTCATGTAACTTTAACAGAAAACGATGCTAAAATTAATTTTAAGACTGCTCTATACTATATGTATGTT

ATACTATATTTTTATATACTATACACAAAAATGTATAAGATGTAAAGCTTGTATTTTCAAATGTTATTGC

AGTATCCAGAACAATTGCGACAATTGCTCTTGGAAGCTTCTGTGCTTTTCCACATGCTGTTCCCTGTCTC

TGGAATGGAATGTTCTCTTATTCTGGCTGGCAAACTCCTGAACATAATGTAAACAGTAAGCTCAAACATC

ACTTGCCCTTTCCTTACCATGCCATCCTGTTTCTTCTAAACCTTATAAATGCCTTTATTATAGTACATAT

TGAATCATAATGTAATTATTATTTATCTCTGACTCCTTTGATTCTGGGACTGCCTTGAGGTCACGGTCTA

GGTTTATTTCATCTTTCTGTCTGTTTAGTCAAATTTCCTACCCTGTGTAACTCTCAGAATCTTACTTTAA

GTTAGGAAAGGCCAGTGGCTGGACAGATGCTACAGACCTCCATTGGTACAGATGCCCTTAGCTTCATGTT

GGGAACAAGTGGTTCTGTTTCTTTTTGTTCCTGTGGAGAAGCATCAGAGATACAGGCAGCTGGGGATTTC

TACACCTGTAATAGCAGCAGCTCAGTGATGACACAGAGACATGTTTTTTTTTTTTTTTTTTTTTTGACAG

AAGGCGAAGGGGAAGCAAGGTACTTCTTACATGGCAGAGGGAAAAGGAGAGTTGGAGTGGGGGAGGGGGA

GAGGGAGGAGCAGGAGCAGGGAGACGGGGAGGGAGAGGAAACTGCCCCGAAGACATGGTTTCTAAGATTT

CTCCCAATGAGACAAATCACTTGTCTGGGATGTCAGGGGGAGAAGATGCAGAATCTGAGCCCCTTGTATA

AATAGACAAAAGGTGAAAGCATTGGCAATAGATCAAAAAGGCATTCCACGTAGCAAAAAAATTATTTTTA

AATCTGAAAATTTAAGTGAAATGCCAAGAGCAAAGAAGAGAGGCCATCAGAAAAATTTCAAAGTGACTAC

TCAGGTCATTGTCCTGAAATAAATGATCATTTTATTCTGGTGACTCACCCCTAGCTAGAGTCAGTACTTC

ATAGAAAAGACAGGAGCTGTGAGACACTTAACTGTAATCTTACCCCTTTACAGAAATGCCTCCTATAGTC

AAGCTGGTCTGATGATCTCATGTCATCATGCACAAGGATTAGCAAGCCTTGAAATTTTATCCTGTCAGTG

TAACTCCTCATCTAGTCTCAAGATTGCACTGGCAGTTCTTTCGCAGCTGATCGTACAGGGAGGACCAATA

AGGCTCATAGAACACCCTTGGAGAATGAATGCCTCTGCTGCTGGAGGTCACAGCACCCAGAAAGTGACTA

GAGGGAGCAGCTGATACCTGCTCTAGTGAGTTGTGCTATTCCTGTCACATCCTTGCAGAGGTCCAAGGGT

TGCTGGCAAGTGAAGGAATATCATGCCTGTAGTTTGAAGGATAGATCACTCAACTCTCAACTATTCTCTC

TTCAGCTGTGAACCATACAACGTTTTTGAAAAGCAAAGTCTGAACATTTGCATTCTATACAGAATATTAC

AGTTGGAAAATAGGTGTGGTGTCTAGAGAAATGCTTCTAAATGCTGCAAGCCACCCTATGTGAAGCCTAG

TCTAGAAATCTGCTTTGCTTCATTTATTAACTTCATTGTATCTTTGGAAGTAGAAGTTCTTAAAAGTGTG

TACAGAAGATGAGTCCTCAGCATGAGTTTCACTGCCTGTCTGTGTGAGAGGAAGTGACCTTGAGAGCAGC

TGTAGCCCTAGGGGGACACCCAGGTAGAGGTGCTCACAACATGCTCTGACACCCGGTATGGCTGCATTCC

CAGCTTAAATGATCACATCTGCTCTTGGTTTTTTCACTGTTTACAAAATCTGATTTCTTTAAAGGGAAGG

GATGGAAAAGGTAGTCAATGCTCCTCTGAGCACCCAAAGCATCCCTCCACAAGAACAAAGAGGAAAAATC

ATTACTTGCTCCCAACATTACACAAAGCGCATCTCTACCAATGGAACTCTGCAGTGTCGGTCTAGCCATC

AGCCTTTCCCACCTCACAGCATATTTTCTGTGGGGGTAATTTATTAGAGGGGAATTTGTTCCTTTTTACT

CTTTTTACTCTTCAAATTAGTAATAGAACCCCTCATGTCATTCTAAGTGACTTAAGTTTTAAAGGGAACC

AGAAAAGGAGAGAATATGCTATTGTGGCACTTCTAAAATGCAGAGTGTCCTCAAAGATGGTTATAGTGGG

TAATGGGTTTCATATAAATTGCATACTAGAGTATCTCCATTTCCTCTTTTAATTTTTTAATTGGTTTTTT

AAAGCTTGATATGCTGAGTTAGTTTTCATTTAAAAACAATGCTTGAGTAGGAAAGTTAAAGAAATAGAAA

TTGCCTGTTTTAATCTATTCATCCTGGCAGCAGGTATGTATTGTATCTGTCCAGAGTCCAAAACGTCAGC

TCCCTCCACTTGCCTTCCCCATTTCACATAAACTATAAAAGTTAGAAATAATATATTATGTCATATCCTA

ACATCAGTTCAATGAATATAGTTTTTAAAATACCTACTATGTGCTAGATACTGAGGACAGAATGATAAAA

TACAAAACAAAGCTAACTAAAAACACAGTTTAACTAGTCTTGTCTACGATTACCTGCCCTAACTATAACT

TCATTTAAGGGGAGATACTGCTAAAGGGAGTTCCTTCTGTGACTGCACACTCTGATACCAATATGTGACT

TCTCCCTGGCTACAACTGATTAAATGAGGGAAGAAAACTGATCCAAGGGGAGTTAGTCCAATAGCTTGTA

AATGGTGGGTTCTTATCAGAGTTTTCTCACTAAGTTAGAACCAAGAGGCTGAGGGTTGTGGGGGCTACTT

AAGCAGAAAGTTTCATGGAGTATGGACTGTGCACCATTGTGAAGTTTCCTGTATTTCCACAGGCTGTGGG

GGCAGAGAGCATCAGAGTGCTGATCAGGAAAAAAGAATGAGGCAGATGCAGACAGAGAAGCAAAGAAGCA

TGAGAGGATGAGAATTAGGAAGTAGACTCTTTTTTCATTTTAATTAATTTGACTAGATTATATTTCTTGC

AATCAAAAGGGCCCTGTGCAGAGAAGGTCCTTATCTTCGTTCATTTTTTGTTGCTATAGCTGAATACCCG

AGACTGGGTAATTTATAAAGTAAAGAAATTTATTGCTTACCATTTTAGATGCTGGGAAGTACAAGGTCAA

GGGTTCTCATCTAGTGAGGGCCTTCTTGCTAGTGGGGTCTCTGTAGAGTCCTGAGGTAACACAGAGCATC

ACATGGTGTGGGGGTGGGATTCACCAGAGAGAGCCAAACTGACTTTTATAACAGACTCACTTTTGTGGTA

ACTAACCCACTCCCTTGATAGCCCATCAATCCATTAATCTAGAAATAAATTCATTCACTCATGAGGGCAG

AGCTCTTGTGAACCAATCACCTCCCAGAAGTCCCACCTCTCAACACTGCTGCACTGGGGACCAAATTTCC

AACACATGAACTTTTCGGGGACACATTCAAGCCACAACAGTCCTCAATAAATATTTGTTGAATCAATAAA

TACACAGGACATAGTGTCCTAAATGCTTGGCATGTACATGAACCAGATTTGGTCCTTGATGTCACAGAGC

TTATAATATAGTGGAGGAGACAGACTTTACATGTTTGATCTTGACTGTAAAACTAGGGTCACTACTCATC

ATCTGATGAAGGAGGCAGACAGTAAAACAAATAACAACTATTCAACATGGTAACGTCAAAACTGAACAGA

GGCTCAAGTGAAAGTTTCAATGATAGTAACCATGGCCAATGGATAGGTGAATGCATTGGAAAATTAGTAT

GATGCCATTAGCAAAAACATAAATTGCACTTAACTAAAGTTCATCTGGCTGAATTCTTTCCTTCCTTCCT

TCCTTCCTTCCTTCCTTCCTTCCTTTCTTCCTCTATCTTTCTTTGGTTTTGAGATCTGTCTATAGTATTG

TGCTAGTGCACATATTTTAATTTGGTGCAAGGCATGCTTAACAGAGTAAGTGAAAAGCTATAAAGGTTAG

AGCGTGTATTAGTTTCAATGACGAAGGAGTAATAAATCATTTTGTCAGGTTGCACTCTAACATTCAAATT

CCATTTTTATTATGGCTAACTAGCCAAGCATTTATAATTCTGAACTCCACAGAATGGGGAAAAAGTGTAT

TAACTTATAATAGATCATTGAGCTTGTTAAATGTACCTTTCCTAATTCGTACTTTTTCCCTTTTGCTGAG

AAAAATGCCTTTCTGAATATATAATTTTTACAGGTCATTGGAAAGGAGTACTGCACAACAGGATTATTAT

AATGTGCTAAAAGATGGCACTTGTATCTCATTGCCACATAGAGAGCTCATGTGAAAACTCTTTGATCTTT

GCTGGCTTTTTAGCTGAAAAGATTTGGAAATCTTTTTGGCACTGGCAAAAACAGAACTGTGGTAACTGTT

TATAGTTGGTTGATTTTTAGCAATGGGTAAGAATGTCTTTGTTGAATCCAGTTTATTAATACGTAATGGG

CAACATGTTGCCTTAAAGGCAAAAGCATATTTTTCTCATATCAAAAAGCTCCCTAAATTTTTGCCAATAT

CCAAAGATATTTTAAGATGGTCTTCATTTTCTTTTTCAGTCTGTCCTATGCTGAGAAAAAAAAATTACAA

CCTCTGAACTTCAGCAGTTTTTTAGGTAAAAGGAATTTTTCATGATGTGATTGATATAACTACTCAGCTG

GACATAAGCTTTCAAGGGCTAAGTCCATTTGCCGATTTGTTTGCATTTCCTACTCCAACTAAAATAATTC

AAAGACATGAAGTGTCTATTGTGGAAAATGCATGAATAATGCAAAAGTCATTCTCAAGTGACCTATCAGA

AAGGCCTACCAGAAATGCAGTTACTGCTTTCTAATAAAATCATGAAAAACTTTTTTAAAAAGTATAACCA

TCTTGAATTTGTTGAGGCAATTATAAGAGAGTATATAAGGGCAAGTTTCTCCAAAAGCGGTTCTGTTTGT

TTTGTTTTTTTTTAATAATTCCCATCAACAGCAATCTGTTTTCTCATTGTAGTAGTATAGGTGTTAACAT

AAAAGCTCAAACAGCTTGGGAGAAAAAAAAAGGATATAGCAAACCTAACTGATAACTTTGCTAGTTTCAAA

TCATTATGATGGCCATTCATAGATGACTGAAATATTTAACATTTGAAGTAACAGAATTTTGACTTATGTT

TTCATGGTTTAAGAGTTTATATTTCAAATTTTTTCACATAGTCTGTTTATAACAGTATATAAAACATGTA

TTAACTATATAAGTTAATAAAGTCATGAACTGATGGCCATTTTTTGGTCTTATTCATATAGTATAGGCTG

TTTTGTGAATTAATCCCCTTCCCATGTGTTTGAATTCCCCACTATGTAAGGCTTAGTGGTGTTAGCATGA

TCCAAGGGTGGGATTTTCAGCCCTCTGACATCAAAAGGTGAAGCAGAGGACACAAAAACCCTCACTGCAT

CTTCCCTAGACTGGCCAGAACCACTCCATGGTCAGTGGACTCCCTTAATAGGAAGGAATGCTGGTCAGTT

GTCAAAGAGCAAAAAGGGAGGGGTAGTCATGAGGTTGGTTGTAATCAGAGGTGCAACAGGTCTTTCCAAA

GGGCTGGTTTCTGTTTAACTCTTAGGAAAGAACACCTAATGGTAGTTAGTGAGGAGGGGGTATAATGAGA

TGTGCCCAACCTCCCATCCCATCATGGCCGGGAACTCAGTTTTTAAGGTTTCCCTGAAGTCCTCTTGGCC

AGGAGAGGGTCTGTTTAGTCAAATGTGGGGGGGGAGCTTAGGATTTTATTTTTATTTCCAAGTTATAATG

TTGGACCATCAGAGCATGAATACAGGTTGAGTCTTTAATCTGTAAATACTTAGCAATCACCTTCTTCTCA

GTAACTTTCTCTGAAGGTTGCTGATAAGCCAGTGTTCTTGATGTTTGCCCACTTACCCCTCAAGATGAGG

AGTGAAGAAATTAGATTGCATTGCCAGGAATTTCCTAGAAGAAAAGGTTTTGAGGATGCATGACCTATGA

GGGTGCTGAGGACCCCTTTCCACCAAACATGGACTAACTTCTGGGTTGCTCTTTCACATCTATTGTCTTG

TTCGACGTACACAGAAGTGCAGAGCACAGCCGCAGCCCTCACAGCATCATCTATCTAGGTTCTGAATCCT

GAATGGTCTGAGGCTCTCCTAGAGCTGAGATTCTCCTTTGCAGTTCCAGTGGAGAAAAGCACTTTCTTTG

CCGAGGCAGTGTAACTTCCTTGTTCCCAAGGCTCCAACTGTTGTGGGCACATATGAGTATACATTAACAT

CCATGCCCAATTTATAATCACTGTTTTCCCCACATGTGTACTCAGACCTCCAAGCTTCTGAAAAATATTT

CCTCTCATCTCTCAAAGAATTTTAGTACATTTGCCTGGATGCAGAAACACAAATCATGCTGGCTTTCCTA

CTCAATCAAAAACTGTAGCTTGAGTGGGGTGGCAAGGGTTGTCCCACAGCAGGTTTACTTGGACTCCAAA

ATGATGTAGAGTTTAGTGAGGCAGCTCCTTGAGTTTGAACTTAGAGCCCTTTTGGCTACCAGGGCACCTG

CAGTCCTCCAAGATAGTAGAGTCTGAACCAGTTAAAGAGCCAGCTACCACAGCTTTGCAGAAACCACTGT

TTCTCCTTAACTCTAGGGCTCAGCGGTTGAACTCCTTAGAATAAAACTTTCTGTCTGCGACCCTATCATT

TTCTTTTGTATTTTGGCCTTCACTGAATTCCCACCCCATCCATTCCAACAGACTTGGTTATACGTGTCTG

AACACCAAATGTGACAGAATTTGTGGCCTTGGATCAGGAGGCTATTTGGCCCTTATCTAAATCGGTGGCT

GAGAATGGATTCCAGGGAAGTGTTTTTCTTAGGAGTACCCAAGAAGCTCAGCTCAGGTTGGGCTCCCTGT

TCTATTCTGAGAACTACAAATCAAATTCAAAATGGAGCACAGAAAATTCTCATTTCTAGTTGACTTATTT

GTATTTTGGTGTCCTGTGCCCTAAATATCTTCTCAAGGAAACCTTGGCTTTAAAAGTGATTGTTTACTTA

AACAAATAAAACAACAACATACAAGAAAAGGCAGTAAATCCGAGCGTCCATCAACAAATGAATCAAAAAA

CAAATGAGATATATATGTACGGTGGCATATTATTCAGCCTTAAAAAGGAAAGAAATTTTGACACATGAGA

CAATATGAATGAACCTTGAAGACATTAATTATGCTGAGTGAAATAAGCCAGCCACAAAAGAACAGATATT

GTATAATTCCTCTTATATTAGGTACCTAAAATAGTCAAATTGATAGAAAGTAGAATGTTGGTTGGCAGGG

GCTGGCAAGGAGAGGGAAATGAAGAGAAGGTGTATGAAGGGAACAGAGTTTCAGTTGGGGAAGACGAAAA

AGTTCTGACAGTGGATGATGGTGGTTGTTGCACAGCAACATGAATGTACTTAATGCCACAGAACTGTATG

CTTAAAAATGGTTGAAACGGTAAATTTGATATAATGTGTATTGAACCTATTTCCAGATCAAAACAGAGAG

ATCTACATTCCTTTTGGTGGTGTCAAAATAAACCATTATCTTGAAATAGC

>NM_006164.5 Homo sapiens NFE2 like bZIP transcription factor 2 (NFE2L2), transcript variant 1, mRNA

GATTACCGAGTGCCGGGGAGCCCGGAGGAGCCGCCGACGCAGCCGCCACCGCCGCCGCCGCCGCCACCAG

AGCCGCCCTGTCCGCGCCGCGCCTCGGCAGCCGGAACAGGGCCGCCGTCGGGGAGCCCCAACACACGGTC

CACAGCTCATCATGATGGACTTGGAGCTGCCGCCGCCGGGACTCCCGTCCCAGCAGGACATGGATTTGAT

TGACATACTTTGGAGGCAAGATATAGATCTTGGAGTAAGTCGAGAAGTATTTGACTTCAGTCAGCGACGG

AAAGAGTATGAGCTGGAAAAACAGAAAAAACTTGAAAAGGAAAGACAAGAACAACTCCAAAAGGAGCAAG

AGAAAGCCTTTTTCGCTCAGTTACAACTAGATGAAGAGACAGGTGAATTTCTCCCAATTCAGCCAGCCCA

GCACATCCAGTCAGAAACCAGTGGATCTGCCAACTACTCCCAGGTTGCCCACATTCCCAAATCAGATGCT

TTGTACTTTGATGACTGCATGCAGCTTTTGGCGCAGACATTCCCGTTTGTAGATGACAATGAGGTTTCTT

CGGCTACGTTTCAGTCACTTGTTCCTGATATTCCCGGTCACATCGAGAGCCCAGTCTTCATTGCTACTAA

TCAGGCTCAGTCACCTGAAACTTCTGTTGCTCAGGTAGCCCCTGTTGATTTAGACGGTATGCAACAGGAC

ATTGAGCAAGTTTGGGAGGAGCTATTATCCATTCCTGAGTTACAGTGTCTTAATATTGAAAATGACAAGC

TGGTTGAGACTACCATGGTTCCAAGTCCAGAAGCCAAACTGACAGAAGTTGACAATTATCATTTTTACTC

ATCTATACCCTCAATGGAAAAAGAAGTAGGTAACTGTAGTCCACATTTTCTTAATGCTTTTGAGGATTCC

TTCAGCAGCATCCTCTCCACAGAAGACCCCAACCAGTTGACAGTGAACTCATTAAATTCAGATGCCACAG

TCAACACAGATTTTGGTGATGAATTTTATTCTGCTTTCATAGCTGAGCCCAGTATCAGCAACAGCATGCC

CTCACCTGCTACTTTAAGCCATTCACTCTCTGAACTTCTAAATGGGCCCATTGATGTTTCTGATCTATCA

CTTTGCAAAGCTTTCAACCAAAACCACCCTGAAAGCACAGCAGAATTCAATGATTCTGACTCCGGCATTT

CACTAAACACAAGTCCCAGTGTGGCATCACCAGAACACTCAGTGGAATCTTCCAGCTATGGAGACACACT

ACTTGGCCTCAGTGATTCTGAAGTGGAAGAGCTAGATAGTGCCCCTGGAAGTGTCAAACAGAATGGTCCT

AAAACACCAGTACATTCTTCTGGGGATATGGTACAACCCTTGTCACCATCTCAGGGGCAGAGCACTCACG

TGCATGATGCCCAATGTGAGAACACACCAGAGAAAGAATTGCCTGTAAGTCCTGGTCATCGGAAAACCCC

ATTCACAAAAGACAAACATTCAAGCCGCTTGGAGGCTCATCTCACAAGAGATGAACTTAGGGCAAAAGCT

CTCCATATCCCATTCCCTGTAGAAAAAATCATTAACCTCCCTGTTGTTGACTTCAACGAAATGATGTCCA

AAGAGCAGTTCAATGAAGCTCAACTTGCATTAATTCGGGATATACGTAGGAGGGGTAAGAATAAAGTGGC

TGCTCAGAATTGCAGAAAAAGAAAACTGGAAAATATAGTAGAACTAGAGCAAGATTTAGATCATTTGAAA

GATGAAAAAGAAAAATTGCTCAAAGAAAAAGGAGAAAATGACAAAAGCCTTCACCTACTGAAAAAACAAC

TCAGCACCTTATATCTCGAAGTTTTCAGCATGCTACGTGATGAAGATGGAAAACCTTATTCTCCTAGTGA

ATACTCCCTGCAGCAAACAAGAGATGGCAATGTTTTCCTTGTTCCCAAAAGTAAGAAGCCAGATGTTAAG

AAAAACTAGATTTAGGAGGATTTGACCTTTTCTGAGCTAGTTTTTTTGTACTATTATACTAAAAGCTCCT

ACTGTGATGTGAAATGCTCATACTTTATAAGTAATTCTATGCAAAATCATAGCCAAAACTAGTATAGAAA

ATAATACGAAACTTTAAAAAGCATTGGAGTGTCAGTATGTTGAATCAGTAGTTTCACTTTAACTGTAAAC

AATTTCTTAGGACACCATTTGGGCTAGTTTCTGTGTAAGTGTAAATACTACAAAAACTTATTTATACTGT

TCTTATGTCATTTGTTATATTCATAGATTTATATGATGATATGACATCTGGCTAAAAAGAAATTATTGCA

AAACTAACCACTATGTACTTTTTTATAAATACTGTATGGACAAAAAATGGCATTTTTTATATTAAATTGT

TTAGCTCTGGCAAAAAAAAAAAAATTTTAAGAGCTGGTACTAATAAAGGATTATTATGACTGTTAAA

>NM_003998.4 Homo sapiens nuclear factor kappa B subunit 1 (NFKB1), transcript variant 1, mRNA

GAAGTGCACCAGCGAGCCGGGGCAGGAAGAGGAGGTTTCGCCACCGGAGCGGCCCGGCGACGCGCTGACA

GCTTCCCCTGCCCTTCCCGTCGGTCGGGCCGCCAGCCGCCGCAGCCCTCGGCCTGCACGCAGCCACCGGC

CCCGCTCCCGGAGCCCAGCGCCGCCGAGGCCGCAGCCGCCCGGCCAGTAAGGCGGCGCCGCCGCCCGGCC

ACCGCGCGCCCTGCGCTTCCCTCCGCCCGCGCTGCGGCCATGGCGCGGCGCTGACTGGCCTGGCCCGGCC

CCGCCGCGCTCCCGCTCGCCCCGACCCGCACTCGGGCCCGCCCGGGCTCCGGCCTGCCGCCGCCTCTTCC

TTCTCCAGCCGGCAGGCCCGCGCCGCTTAGGAGGGAGAGCCCACCCGCGCCAGGAGGCCGAACGCGGACT

CGCCACCCGGCTTCAGAATGGCAGAAGATGATCCATATTTGGGAAGGCCTGAACAAATGTTTCATTTGGA

TCCTTCTTTGACTCATACAATATTTAATCCAGAAGTATTTCAACCACAGATGGCACTGCCAACAGCAGAT

GGCCCATACCTTCAAATATTAGAGCAACCTAAACAGAGAGGATTTCGTTTCCGTTATGTATGTGAAGGCC

CATCCCATGGTGGACTACCTGGTGCCTCTAGTGAAAAGAACAAGAAGTCTTACCCTCAGGTCAAAATCTG

CAACTATGTGGGACCAGCAAAGGTTATTGTTCAGTTGGTCACAAATGGAAAAAATATCCACCTGCATGCC

CACAGCCTGGTGGGAAAACACTGTGAGGATGGGATCTGCACTGTAACTGCTGGACCCAAGGACATGGTGG

TCGGCTTCGCAAACCTGGGTATACTTCATGTGACAAAGAAAAAAGTATTTGAAACACTGGAAGCACGAAT

GACAGAGGCGTGTATAAGGGGCTATAATCCTGGACTCTTGGTGCACCCTGACCTTGCCTATTTGCAAGCA

GAAGGTGGAGGGGACCGGCAGCTGGGAGATCGGGAAAAAGAGCTAATCCGCCAAGCAGCTCTGCAGCAGA

CCAAGGAGATGGACCTCAGCGTGGTGCGGCTCATGTTTACAGCTTTTCTTCCGGATAGCACTGGCAGCTT

CACAAGGCGCCTGGAACCCGTGGTATCAGACGCCATCTATGACAGTAAAGCCCCCAATGCATCCAACTTG

AAAATTGTAAGAATGGACAGGACAGCTGGATGTGTGACTGGAGGGGAGGAAATTTATCTTCTTTGTGACA

AAGTTCAGAAAGATGACATCCAGATTCGATTTTATGAAGAGGAAGAAAATGGTGGAGTCTGGGAAGGATT

TGGAGATTTTTCCCCCACAGATGTTCATAGACAATTTGCCATTGTCTTCAAAACTCCAAAGTATAAAGAT

ATTAATATTACAAAACCAGCCTCTGTGTTTGTCCAGCTTCGGAGGAAATCTGACTTGGAAACTAGTGAAC

CAAAACCTTTCCTCTACTATCCTGAAATCAAAGATAAAGAAGAAGTGCAGAGGAAACGTCAGAAGCTCAT

GCCCAATTTTTCGGATAGTTTCGGCGGTGGTAGTGGTGCTGGAGCTGGAGGCGGAGGCATGTTTGGTAGT

GGCGGTGGAGGAGGGGGCACTGGAAGTACAGGTCCAGGGTATAGCTTCCCACACTATGGATTTCCTACTT

ATGGTGGGATTACTTTCCATCCTGGAACTACTAAATCTAATGCTGGGATGAAGCATGGAACCATGGACAC

TGAATCTAAAAAGGACCCTGAAGGTTGTGACAAAAGTGATGACAAAAACACTGTAAACCTCTTTGGGAAA

GTTATTGAAACCACAGAGCAAGATCAGGAGCCCAGCGAGGCCACCGTTGGGAATGGTGAGGTCACTCTAA

CGTATGCAACAGGAACAAAAGAAGAGAGTGCTGGAGTTCAGGATAACCTCTTTCTAGAGAAGGCTATGCA

GCTTGCAAAGAGGCATGCCAATGCCCTTTTCGACTACGCGGTGACAGGAGACGTGAAGATGCTGCTGGCC

GTCCAGCGCCATCTCACTGCTGTGCAGGATGAGAATGGGGACAGTGTCTTACACTTAGCAATCATCCACC

TTCATTCTCAACTTGTGAGGGATCTACTAGAAGTCACATCTGGTTTGATTTCTGATGACATTATCAACAT

GAGAAATGATCTGTACCAGACGCCCTTGCACTTGGCAGTGATCACTAAGCAGGAAGATGTGGTGGAGGAT

TTGCTGAGGGCTGGGGCCGACCTGAGCCTTCTGGACCGCTTGGGTAACTCTGTTTTGCACCTAGCTGCCA

AAGAAGGACATGATAAAGTTCTCAGTATCTTACTCAAGCACAAAAAGGCAGCACTACTTCTTGACCACCC

CAACGGGGACGGTCTGAATGCCATTCATCTAGCCATGATGAGCAATAGCCTGCCATGTTTGCTGCTGCTG

GTGGCCGCTGGGGCTGACGTCAATGCTCAGGAGCAGAAGTCCGGGCGCACAGCACTGCACCTGGCTGTGG

AGCACGACAACATCTCATTGGCAGGCTGCCTGCTCCTGGAGGGTGATGCCCATGTGGACAGTACTACCTA

CGATGGAACCACACCCCTGCATATAGCAGCTGGGAGAGGGTCCACCAGGCTGGCAGCTCTTCTCAAAGCA

GCAGGAGCAGATCCCCTGGTGGAGAACTTTGAGCCTCTCTATGACCTGGATGACTCTTGGGAAAATGCAG

GAGAGGATGAAGGAGTTGTGCCTGGAACCACGCCTCTAGATATGGCCACCAGCTGGCAGGTATTTGACAT

ATTAAATGGGAAACCATATGAGCCAGAGTTTACATCTGATGATTTACTAGCACAAGGAGACATGAAACAG

CTGGCTGAAGATGTGAAGCTGCAGCTGTATAAGTTACTAGAAATTCCTGATCCAGACAAAAACTGGGCTA

CTCTGGCGCAGAAATTAGGTCTGGGGATACTTAATAATGCCTTCCGGCTGAGTCCTGCTCCTTCCAAAAC

ACTTATGGACAACTATGAGGTCTCTGGGGGTACAGTCAGAGAGCTGGTGGAGGCCCTGAGACAAATGGGC

TACACCGAAGCAATTGAAGTGATCCAGGCAGCCTCCAGCCCAGTGAAGACCACCTCTCAGGCCCACTCGC

TGCCTCTCTCGCCTGCCTCCACAAGGCAGCAAATAGACGAGCTCCGAGACAGTGACAGTGTCTGCGACAG

CGGCGTGGAGACATCCTTCCGCAAACTCAGCTTTACCGAGTCTCTGACCAGTGGTGCCTCACTGCTAACT

CTCAACAAAATGCCCCATGATTATGGGCAGGAAGGACCTCTAGAAGGCAAAATTTAGCCTGCTGACAATT

TCCCACACCGTGTAAACCAAAGCCCTAAAATTCCACTGCGTTGTCCACAAGACAGAAGCTGAAGTGCATC

CAAAGGTGCTCAGAGAGCCGGCCCGCCTGAATCATTCTCGATTTAACTCGAGACCTTTTCAACTTGGCTT

CCTTTCTTGGTTCATAAATGAATTTTAGTTTGGTTCACTTACAGATAGTATCTAGCAATCACAACACTGG

CTGAGCGGATGCATCTGGGGATGAGGTTGCTTACTAAGCTTTGCCAGCTGCTGCTGGATCACAGCTGCTT

TCTGTTGTCATTGCTGTTGTCCCTCTGCTACGTTCCTATTGTCATTAAAGGTATCACGGTCGCCACCTGG

CATTCCTTCTGACCACAGCATCATTTTGCATTCAAATTAAGGGTTAAGAAAAGAGATATTTTAAAATGAG

AGTCACTTGATGTGCCATTTTAAAAAAAAAGGCATATTGCTTTTTCTAATGTGGTTATTTCTCTGATTTG

CAAAAAAAAAAAAAAAAAAAATACTTGTCAATATTTAAACATGGTTACAATCATTGCTGAAAATGGTATT

TTCCCCCTTTTCTGCATTTTGCTATTGTAAATATGTTTTTTAGATCAAATACTTTAAAGGAAAAAATGTT

GGATTTATAAATGCTATTTTTTATTTTACTTTTATAATAAAAGGAAAAGCAAATTGATGACCTCA

>NM_145285.3 Homo sapiens NK2 homeobox 3 (NKX2-3), mRNA

AGTGGCTGTAACAAAACCCAGACCCCCAGGTCCCGGCCAATGGAGGCGATTTAGACTGGAGTGGGACCGC

GTCTGTCAAAAGCCCGACTCGGCAGCAGCGGCGGAGTCCAGGAGGAGAGCTGGAGCCGCCGCGCTGCCTC

CCCGCCCCCGCCGGGATTTATTATTTGGACTGGACAATTAAGTGGCCCTGATGATGTTACCAAGCCCGGT

CACCTCCACCCCTTTCTCAGTCAAAGACATTTTGAATCTGGAGCAGCAGCACCAGCACTTCCATGGTGCG

CACTTGCAGGCGGACTTGGAGCACCACTTCCACTCTGCGCCCTGCATGCTGGCCGCCGCTGAGGGGACGC

AATTTTCTGACGGAGGGGAGGAGGACGAGGAAGACGAGGGCGAGAAATTGTCCTATTTGAACTCACTAGC

CGCAGCAGACGGCCACGGGGATTCAGGGCTGTGTCCCCAGGGCTATGTCCACACGGTCCTGCGAGACTCG

TGCAGCGAGCCCAAGGAACATGAAGAGGAGCCCGAGGTCGTGAGGGACCGGAGCCAAAAAAGCTGCCAGC

TGAAGAAGTCTCTAGAGACGGCCGGAGACTGCAAGGCGGCGGAGGAGAGCGAGAGGCCGAAGCCACGCAG

CCGCCGGAAGCCCCGGGTCCTCTTCTCGCAAGCCCAGGTCTTCGAGCTGGAACGCAGGTTCAAGCAGCAG

CGGTACCTGTCGGCACCCGAGCGCGAGCACCTCGCCAGCAGCCTGAAGCTCACATCCACTCAGGTGAAAA

TCTGGTTCCAGAATCGCAGGTACAAGTGCAAGAGACAGCGGCAGGACAAGTCTCTGGAGCTTGGCGCACA

CGCGCCCCCGCCGCCGCCGCGCCGCGTGGCTGTCCCGGTGCTGGTGCGGGACGGCAAGCCGTGCGTCACG

CCCAGCGCGCAGGCCTACGGCGCGCCCTACAGCGTGGGCGCCAGCGCCTACTCCTACAACAGCTTCCCCG

CCTACGGCTATGGGAACTCGGCCGCGGCCGCCGCCGCCGCCGCCGCCGCCGCAGCAGCGGCGGCCTA

CAGCAGCAGCTATGGCTGTGCGTACCCGGCGGGCGGCGGCGGCGGCGGCGGCGGGACCTCCGCGGCGACC

ACTGCCATGCAGCCCGCCTGCAGCGCGGCCGGAGGCGGCCCCTTTGTGAACGTGAGCAACCTAGGAGGCT

TCGGCAGCGGCGGCAGCGCACAGCCGTTGCACCAGGGTACTGCAGCCGGGGCCGCGTGCGCTCAGGGCAC

CTTGCAGGGCATCCGGGCCTGGTAGGGACGGGGCGGGTCACGCGGCGGGCACCCCAGCGCAGCCTGGCGC

CGCGGGACTGAAGCTCGAGAAGGGCCTGACCTAAAGGTCAGGTCCCCTCGTTAAAAAAATATGTACGTCT

AGCTCCTCAGGGCTTCGGATCGCAGCTCACTCGAGGCCTGGGGAAGGGGACTCAGGGGCGAGGAGGATGA

CTGGGTCCGGTCGCCAGGACTGTCTCTGAGGCAGAAACGCCGGCTGGGCGCCGGGGAGGACGATGGCCCC

GACCCTGGCAGCGAGAGGAGACCAGGAGGCTAGGACCCTGGCCGCGCTTGGTTCTTCCAAAGCGAGAAGG

GCTTCTCTCCCTCTGCCTTTCCGCGGCCTCCGCGAAGCGTTGGCGGGGAGCCCAAGGACATAACAAATTA

AAAGCATGAAGGAGAGAAAAATGGGGGTCGTGGCTTGAGAAATTCCAGGCCCTACCGATCCTCTGCCCCC

TTTGCGGGCCTGGAGCGCCATAGCACAGTCGATTTCGTTTCGCAGCTGTCTCCCCTCCGCAGCAGATACC

TCGGTCCAGATCTCCGGATTGTCGGGGGACGCAGGACTCTTCGAGGAAAACCAGCCGAATGAGATCAAAA

GTTGGGGGTGGGGGGAGGCTGAACAAACTCAGGACCTGGTGGCCCACCGGAGGTGTTACCGGGTTTCCTT

TCTGTTTCGTATTCTGTATTCAGCACATGTTATCTATCTATCTATCTATATAACTATAACCACACGCCGT

GTAGACACCCGCTGCCACACACTACAGGAGTCAATAAACAAGGTGCAATATTTTCAAA

>NM_021209.4 Homo sapiens NLR family CARD domain containing 4 (NLRC4), transcript variant 1, mRNA

AAGCCCCTGGCTGTTTATACTCCGGAGGGTGTCCCCGTGCGTCATCGGTGGAGTGGACCAAAACTGGTGA

TCTGTTTGCCCTGTGTGACCTTGCCCAGAACCCTGCTGACTGAGAGAACACATCTGCTGGAAGTCCTCTG

GGATTCAAGGTACAGGGAATGAAGAGTAGTTTTACAGAAAAAAGAGGACAATATTGGGATCACCTTTGAC

CTTTCCATTTGGAAATAATATTTTCTATTGTGTTATAGAAAGGTGGGAAGCTTTCATCCAGAACAATGAA

TTTCATAAAGGACAATAGCCGAGCCCTTATTCAAAGAATGGGAATGACTGTTATAAAGCAAATCACAGAT

GACCTATTTGTATGGAATGTTCTGAATCGCGAAGAAGTAAACATCATTTGCTGCGAGAAGGTGGAGCAGG

ATGCTGCTAGAGGGATCATTCACATGATTTTGAAAAAGGGTTCAGAGTCCTGTAACCTCTTTCTTAAATC

CCTTAAGGAGTGGAACTATCCTCTATTTCAGGACTTGAATGGACAAAGTCTTTTTCATCAGACATCAGAA

GGAGACTTGGACGATTTGGCTCAGGATTTAAAGGACTTGTACCATACCCCATCTTTTCTGAACTTTTATC

CCCTTGGTGAAGATATTGACATTATTTTTAACTTGAAAAGCACCTTCACAGAACCTGTCCTGTGGAGGAA

GGACCAACACCATCACCGCGTGGAGCAGCTGACCCTGAATGGCCTCCTGCAGGCTCTTCAGAGCCCCTGC

ATCATTGAAGGGGAATCTGGCAAAGGCAAGTCCACTCTGCTGCAGCGAATTGCCATGCTCTGGGGCTCCG

GAAAGTGCAAGGCTCTGACCAAGTTCAAATTCGTCTTCTTCCTCCGTCTCAGCAGGGCCCAGGGTGGACT

TTTTGAAACCCTCTGTGATCAACTCCTGGATATACCTGGCACAATCAGGAAGCAGACATTCATGGCCATG

CTGCTGAAGCTGCGGCAGAGGGTTCTTTTCCTTCTTGATGGCTACAATGAATTCAAGCCCCAGAACTGCC

CAGAAATCGAAGCCCTGATAAAGGAAAACCACCGCTTCAAGAACATGGTCATCGTCACCACTACCACTGA

GTGCCTGAGGCACATACGGCAGTTTGGTGCCCTGACTGCTGAGGTGGGGGATATGACAGAAGACAGCGCC

CAGGCTCTCATCCGAGAAGTGCTGATCAAGGAGCTTGCTGAAGGCTTGTTGCTCCAAATTCAGAAATCCA

GGTGCTTGAGGAATCTCATGAAGACCCCTCTCTTTGTGGTCATCACTTGTGCAATCCAGATGGGTGAAAG

TGAGTTCCACTCTCACACACAAACAACGCTGTTCCATACCTTCTATGATCTGTTGATACAGAAAAACAAA

CACAAACATAAAGGTGTGGCTGCAAGTGACTTCATTCGGAGCCTGGACCACTGTGGAGACCTAGCTCTGG

AGGGTGTGTTCTCCCACAAGTTTGATTTCGAACTGCAGGATGTGTCCAGCGTGAATGAGGATGTCCTGCT

GACAACTGGGCTCCTCTGTAAATATACAGCTCAAAGGTTCAAGCCAAAGTATAAATTCTTTCACAAGTCA

TTCCAGGAGTACACAGCAGGACGAAGACTCAGCAGTTTATTGACGTCTCATGAGCCAGAGGAGGTGACCA

AGGGGAATGGTTACTTGCAGAAAATGGTTTCCATTTCGGACATTACATCCACTTATAGCAGCCTGCTCCG

GTACACCTGTGGGTCATCTGTGGAAGCCACCAGGGCTGTTATGAAGCACCTCGCAGCAGTGTATCAACAC

GGCTGCCTTCTCGGACTTTCCATCGCCAAGAGGCCTCTCTGGAGACAGGAATCTTTGCAAAGTGTGAAAA

ACACCACTGAGCAAGAAATTCTGAAAGCCATAAACATCAATTCCTTTGTAGAGTGTGGCATCCATTTATA

TCAAGAGAGTACATCCAAATCAGCCCTGAGCCAAGAATTTGAAGCTTTCTTTCAAGGTAAAAGCTTATAT

ATCAACTCAGGGAACATCCCCGATTACTTATTTGACTTCTTTGAACATTTGCCCAATTGTGCAAGTGCCC

TGGACTTCATTAAACTGGACTTTTATGGGGGAGCTATGGCTTCATGGGAAAAGGCTGCAGAAGACACAGG

TGGAATCCACATGGAAGAGGCCCCAGAAACCTACATTCCCAGCAGGGCTGTATCTTTGTTCTTCAACTGG

AAGCAGGAATTCAGGACTCTGGAGGTCACACTCCGGGATTTCAGCAAGTTGAATAAGCAAGATATCAGAT

ATCTGGGGAAAATATTCAGCTCTGCCACAAGCCTCAGGCTGCAAATAAAGAGATGTGCTGGTGTGGCTGG

AAGCCTCAGTTTGGTCCTCAGCACCTGTAAGAACATTTATTCTCTCATGGTGGAAGCCAGTCCCCTCACC

ATAGAAGATGAGAGGCACATCACATCTGTAACAAACCTGAAAACCTTGAGTATTCATGACCTACAGAATC

AACGGCTGCCGGGTGGTCTGACTGACAGCTTGGGTAACTTGAAGAACCTTACAAAGCTCATAATGGATAA

CATAAAGATGAATGAAGAAGATGCTATAAAACTAGCTGAAGGCCTGAAAAACCTGAAGAAGATGTGTTTA

TTTCATTTGACCCACTTGTCTGACATTGGAGAGGGAATGGATTACATAGTCAAGTCTCTGTCAAGTGAAC

CCTGTGACCTTGAAGAAATTCAATTAGTCTCCTGCTGCTTGTCTGCAAATGCAGTGAAAATCCTAGCTCA

GAATCTTCACAATTTGGTCAAACTGAGCATTCTTGATTTATCAGAAAATTACCTGGAAAAAGATGGAAAT

GAAGCTCTTCATGAACTGATCGACAGGATGAACGTGCTAGAACAGCTCACCGCACTGATGCTGCCCTGGG

GCTGTGACGTGCAAGGCAGCCTGAGCAGCCTGTTGAAACATTTGGAGGAGGTCCCACAACTCGTCAAGCT

TGGGTTGAAAAACTGGAGACTCACAGATACAGAGATTAGAATTTTAGGTGCATTTTTTGGAAAGAACCCT

CTGAAAAACTTCCAGCAGTTGAATTTGGCGGGAAATCGTGTGAGCAGTGATGGATGGCTTGCCTTCATGG

GTGTATTTGAGAATCTTAAGCAATTAGTGTTTTTTGACTTTAGTACTAAAGAATTTCTACCTGATCCAGC

ATTAGTCAGAAAACTTAGCCAAGTGTTATCCAAGTTAACTTTTCTGCAAGAAGCTAGGCTTGTTGGGTGG

CAATTTGATGATGATGATCTCAGTGTTATTACAGGTGCTTTTAAACTAGTAACTGCTTAAATAAAGTGTA

CTCGAAGCCAGTAAAAAAAAAAAAA

>NM_000271.5 Homo sapiens NPC intracellular cholesterol transporter 1 (NPC1), mRNA

CTTCCTGACCGGCGCGCGCAGCCTGCTGCCGCGGTCAGCGCCTGCTCCTGCTCCTCCGCTCCTCCTGCGC

GGGGTGCTGAAACAGCCCGGGGAAGTAGAGCCGCCTCCGGGGAGCCCAACCAGCCGAACGCCGCCGGCGT

CAGCAGCCTTGCGCGGCCACAGCATGACCGCTCGCGGCCTGGCCCTTGGCCTCCTCCTGCTGCTACTGTG

TCCAGCGCAGGTGTTTTCACAGTCCTGTGTTTGGTATGGAGAGTGTGGAATTGCATATGGGGACAAGAGG

TACAATTGCGAATATTCTGGCCCACCAAAACCATTGCCAAAGGATGGATATGACTTAGTGCAGGAACTCT

GTCCAGGATTCTTCTTTGGCAATGTCAGTCTCTGTTGTGATGTTCGGCAGCTTCAGACACTAAAAGACAA

CCTGCAGCTGCCTCTACAGTTTCTGTCCAGATGTCCATCCTGTTTTTATAACCTACTGAACCTGTTTTGT

GAGCTGACATGTAGCCCTCGACAGAGTCAGTTTTTGAATGTTACAGCTACTGAAGATTATGTTGATCCTG

TTACAAACCAGACGAAAACAAATGTGAAAGAGTTACAATACTACGTCGGACAGAGTTTTGCCAATGCAAT

GTACAATGCCTGCCGGGATGTGGAGGCCCCCTCAAGTAATGACAAGGCCCTGGGACTCCTGTGTGGGAAG

GACGCTGACGCCTGTAATGCCACCAACTGGATTGAATACATGTTCAATAAGGACAATGGACAGGCACCTT

TTACCATCACTCCTGTGTTTTCAGATTTTCCAGTCCATGGGATGGAGCCCATGAACAATGCCACCAAAGG

CTGTGACGAGTCTGTGGATGAGGTCACAGCACCATGTAGCTGCCAAGACTGCTCTATTGTCTGTGGCCCC

AAGCCCCAGCCCCCACCTCCTCCTGCTCCCTGGACGATCCTTGGCTTGGACGCCATGTATGTCATCATGT

GGATCACCTACATGGCGTTTTTGCTTGTGTTTTTTGGAGCATTTTTTGCAGTGTGGTGCTACAGAAAACG

GTATTTTGTCTCCGAGTACACTCCCATCGATAGCAATATAGCTTTTTCTGTTAATGCAAGTGACAAAGGA

GAGGCGTCCTGCTGTGACCCTGTCAGCGCAGCATTTGAGGGCTGCTTGAGGCGGCTGTTCACACGCTGGG

GGTCTTTCTGCGTCCGAAACCCTGGCTGTGTCATTTTCTTCTCGCTGGTCTTCATTACTGCGTGTTCGTC

AGGCCTGGTGTTTGTCCGGGTCACAACCAATCCAGTTGACCTCTGGTCAGCCCCCAGCAGCCAGGCTCGC

CTGGAAAAAGAGTACTTTGACCAGCACTTTGGGCCTTTCTTCCGGACGGAGCAGCTCATCATCCGGGCCC

CTCTCACTGACAAACACATTTACCAGCCATACCCTTCGGGAGCTGATGTACCCTTTGGACCTCCGCTTGA

CATACAGATACTGCACCAGGTTCTTGACTTACAAATAGCCATCGAAAACATTACTGCCTCTTATGACAAT

GAGACTGTGACACTTCAAGACATCTGCTTGGCCCCTCTTTCACCGTATAACACGAACTGCACCATTTTGA

GTGTGTTAAATTACTTCCAGAACAGCCATTCCGTGCTGGACCACAAGAAAGGGGACGACTTCTTTGTGTA

TGCCGATTACCACACGCACTTTCTGTACTGCGTACGGGCTCCTGCCTCTCTGAATGATACAAGTTTGCTC

CATGACCCTTGTCTGGGTACGTTTGGTGGACCAGTGTTCCCGTGGCTTGTGTTGGGAGGCTATGATGATC

AAAACTACAATAACGCCACTGCCCTTGTGATTACCTTCCCTGTCAATAATTACTATAATGATACAGAGAA

GCTCCAGAGGGCCCAGGCCTGGGAAAAAGAGTTTATTAATTTTGTGAAAAACTACAAGAATCCCAATCTG

ACCATTTCCTTCACTGCTGAACGAAGTATTGAAGATGAACTAAATCGTGAAAGTGACAGTGATGTCTTCA

CCGTTGTAATTAGCTATGCCATCATGTTTCTATATATTTCCCTAGCCTTGGGGCACATGAAAAGCTGTCG

CAGGCTTCTGGTGGATTCGAAGGTCTCACTAGGCATCGCGGGCATCTTGATCGTGCTGAGCTCGGTGGCT

TGCTCCTTGGGTGTCTTCAGCTACATTGGGTTGCCCTTGACCCTCATTGTGATTGAAGTCATCCCGTTCC

TGGTGCTGGCTGTTGGAGTGGACAACATCTTCATTCTGGTGCAGGCCTACCAGAGAGATGAACGTCTTCA

AGGGGAAACCCTGGATCAGCAGCTGGGCAGGGTCCTAGGAGAAGTGGCTCCCAGTATGTTCCTGTCATCC

TTTTCTGAGACTGTAGCATTTTTCTTAGGAGCATTGTCCGTGATGCCAGCCGTGCACACCTTCTCTCTCT

TTGCGGGATTGGCAGTCTTCATTGACTTTCTTCTGCAGATTACCTGTTTCGTGAGTCTCTTGGGGTTAGA

CATTAAACGTCAAGAGAAAAATCGGCTAGACATCTTTTGCTGTGTCAGAGGTGCTGAAGATGGAACAAGC

GTCCAGGCCTCAGAGAGCTGTTTGTTTCGCTTCTTCAAAAACTCCTATTCTCCACTTCTGCTAAAGGACT

GGATGAGACCAATTGTGATAGCAATATTTGTGGGTGTTCTGTCATTCAGCATCGCAGTCCTGAACAAAGT

AGATATTGGATTGGATCAGTCTCTTTCGATGCCAGATGACTCCTACATGGTGGATTATTTCAAATCCATC

AGTCAGTACCTGCATGCGGGTCCGCCTGTGTACTTTGTCCTGGAGGAAGGGCACGACTACACTTCTTCCA

AGGGGCAGAACATGGTGTGCGGCGGCATGGGCTGCAACAATGATTCCCTGGTGCAGCAGATATTTAACGC

GGCGCAGCTGGACAACTATACCCGAATAGGCTTCGCCCCCTCGTCCTGGATCGACGATTATTTCGACTGG

GTGAAGCCACAGTCGTCTTGCTGTCGAGTGGACAATATCACTGACCAGTTCTGCAATGCTTCAGTGGTTG

ACCCTGCCTGCGTTCGCTGCAGGCCTCTGACTCCGGAAGGCAAACAGAGGCCTCAGGGGGGAGACTTCAT

GAGATTCCTGCCCATGTTCCTTTCGGATAACCCTAACCCCAAGTGTGGCAAAGGGGGACATGCTGCCTAT

AGTTCTGCAGTTAACATCCTCCTTGGCCATGGCACCAGGGTCGGAGCCACGTACTTCATGACCTACCACA

CCGTGCTGCAGACCTCTGCTGACTTTATTGACGCTCTGAAGAAAGCCCGACTTATAGCCAGTAATGTCAC

CGAAACCATGGGCATTAACGGCAGTGCCTACCGAGTATTTCCTTACAGTGTGTTTTATGTCTTCTACGAA

CAGTACCTGACCATCATTGACGACACTATCTTCAACCTCGGTGTGTCCCTGGGCGCGATATTTCTGGTGA

CCATGGTCCTCCTGGGCTGTGAGCTCTGGTCTGCAGTCATCATGTGTGCCACCATCGCCATGGTCTTGGT

CAACATGTTTGGAGTTATGTGGCTCTGGGGCATCAGTCTGAACGCTGTATCCTTGGTCAACCTGGTGATG

AGCTGTGGCATCTCCGTGGAGTTCTGCAGCCACATAACCAGAGCGTTCACGGTGAGCATGAAAGGCAGCC

GCGTGGAGCGCGCGGAAGAGGCACTTGCCCACATGGGCAGCTCCGTGTTCAGTGGAATCACACTTACAAA

ATTTGGAGGGATTGTGGTGTTGGCTTTTGCCAAATCTCAAATTTTCCAGATATTCTACTTCAGGATGTAT

TTGGCCATGGTCTTACTGGGAGCCACTCACGGATTAATATTTCTCCCTGTCTTACTCAGTTACATAGGGC

CATCAGTAAATAAAGCCAAAAGTTGTGCCACTGAAGAGCGATACAAAGGAACAGAGCGCGAACGGCTTCT

AAATTTCTAGCCCTCTCGCAGGGCATCCTGACTGAACTGTGTCTAAGGGTCGGTCGGTTTACCACTGGAC

GGGTGCTGCATCGGCAAGGCCAAGTTGAACACCGGATGGTGCCAACCATCGGTTGTTTGGCAGCAGCTTT

GAACGTAGCGCCTGTGAACTCAGGAATGCACAGTTGACTTGGGAAGCAGTATTACTAGATCTGGAGGCAA

CCACAGGACACTAAACTTCTCCCAGCCTCTTCAGGAAAGAAACCTCATTCTTTGGCAAGCAGGAGGTGAC

ACTAGATGGCTGTGAATGTGATCCGCTCACTGACACTCTGTAAAGGCCAATCAATGCACTGTCTGTCTCT

CCTTTTAGGAGTAAGCCATCCCACAAGTTCTATACCATATTTTTAGTGACAGTTGAGGTTGTAGATACAC

TTTATAACATTTTATAGTTTAAAGAGCTTTATTAATGCAATAAATTAACTTTGTACACATTTTTATATAA

AAAAACAGCAAGTGATTTCAGAATGTTGTAGGCCTCATTAGAGCTTGGTCTCCAAAAATCTGTTTGAAAA

AAGCAACATGTTCTTCACAGTGTTCCCCTAGAAAGGAAGAGATTTAATTGCCAGTTAGATGTGGCATGAA

ATGAGGGACAAAGAAAGCATCTCGTAGGTGTGTCTACTGGGTTTTAACTTATTTTTCTTTAATAAAATAC

ATTGTTTTCCTAAGTTTTGGGGTTACCCTATCTGCTTTGAGAGACAAATACAAAAGCTAAATGGAAGAGA

>NM_030759.5 Homo sapiens nuclear receptor binding factor 2 (NRBF2), transcript variant 1, mRNA

GCTCCTTCAGCGCCTATCGCTGGCTCTTGGGGCGCAGAGAGGGGCCGCAGTCTCCGCGGCTGCGTCGAGC

TCCCTTGCAGTCCCCTCCATGTTCCCCGGCGCCACTACTCCCCTTCCTAAGGCCGCCGCTTACCCCGGGG

TCTATGGAAGTAATGGAAGGACCCCTCAACCTGGCTCATCAACAGAGCAGACGAGCAGACCGTTTATTAG

CTGCAGGCAAATACGAAGAGGCTATTTCTTGTCACAAAAAGGCTGCAGCATATCTTTCTGAAGCCATGAA

GCTGACACAGTCAGAGCAGGCTCATCTTTCACTGGAATTGCAAAGGGATAGCCATATGAAACAGCTCCTC

CTCATCCAAGAGAGATGGAAAAGGGCCCAGCGTGAAGAAAGATTGAAAGCCCAGCAGAACACAGACAAGG

ATGCAGCTGCCCATCTTCAGACATCTCACAAACCCTCTGCAGAGGATGCAGAGGGCCAGAGTCCCCTTTC

TCAGAAGTACAGCCCTTCCACAGAGAAATGCCTGCCTGAGATTCAGGGGATCTTTGACAGGGATCCAGAC

ACACTACTTTATTTACTTCAGCAAAAGAGTGAGCCAGCAGAGCCATGTATTGGAAGCAAAGCCCCAAAAG

ATGATAAAACAATTATAGAGGAGCAGGCAACCAAAATTGCAGATTTGAAGAGGCATGTGGAATTCCTTGT

GGCTGAGAATGAAAGATTAAGGAAAGAAAATAAACAACTAAAGGCTGAAAAGGCCAGACTTCTAAAAGGT

CCAATAGAAAAGGAGCTGGATGTAGATGCTGATTTTGTAGAAACGTCAGAGTTATGGAGCTTGCCACCAC

ATGCAGAAACTGCTACAGCCTCCTCAACCTGGCAGAAGTTCGCAGCAAATACTGGGAAAGCCAAGGACAT

TCCAATCCCCAATCTTCCTCCCTTGGATTTTCCATCTCCAGAACTTCCTCTTATGGAGCTCTCTGAGGAT

ATTCTGAAAGGATTTATGAATAATTAAAATGGAAGGCCACAGAAAAGGGGAAAAGAGGAAATAATACAGT

AATCGTTAATCCAGCAAAAAGAAATGAAAAGGGAAAACCACATAGAAGGGTAATCCCGGAAATGCTTCAT

CTGGTGGACTGTGGGAGCAGAGGCATTGCCAGGACTTGGGAAACAGTCACTGTGAAATGCGCTGCGTATC

TCATTCACTCACTTCAGCTAATGATTCCGACTTGGCAGACGCTAAACTCATGGAGGTTCGGTTTCTCCTG

ATACAAACCAAATGGCTACCTGGAAGAATTTCTTTCAAGCAACAGTTATTTTTCTTATCTTCAGGGTTAA

AATGTATAAAAGTTATGTGTAATTAATCTATAATGCCATAAATGATAATGCAAAACCTAAATAATATGGT

GGCCGGAGGGGCTGCCTTATATTTGAAACATGCTTTCTATCATGCATTGACTGTATGCATTTTGTTAATG

CACATTCTGTTTGTTTAAGGTGTGTAAGATACACACCCTTCTAGATGAAACTATATGTGCCACACTTTGC

ACTACTCATAATGATAACCTCAAGACTATCAGAAGAAATATTTAAATTTCCATTTTATGAAGAAAGGAAC

CAAATTATTATGCTTTTTAAAACAAATTACCAGTTTACATAATTAATCAGGGTGCATTTTAAGTTCTAAC

TTCGTTTATTGTATAATGCATCATTTGAAAATACCAAGGAGGAAATACCCTTTGTTTTTAATGATGCAAG

AGTGGACGTAATGCTAGTTGGCAGTATTTTATTGTAAGAAATCAATAAAGTAATTGTGTTTTA

>NM_001159995.3 Homo sapiens neuregulin 1 (NRG1), transcript variant HRG-beta1c, mRNA

AGTAAGCCTCCGCAGCCCACTCGGACTGCAGCCTGTTTGCCGCCCGTCCTCCCATTGCAGCACTCGGGGC

GACAGAGAGGGAGGAGGCGCGCGGGGACGGGGACGCCCAGGAGGACCCACTCGCGGGTCCCGCTCCGCTC

CGGCAGCAGCATGGGGAAAGGACGCGCGGGCCGAGTTGGCACCACAGCCTTGCCTCCCCGATTGAAAGAG

ATGAAAAGCCAGGAATCGGCTGCAGGTTCCAAACTAGTCCTTCGGTGTGAAACCAGTTCTGAATACTCCT

CTCTCAGATTCAAGTGGTTCAAGAATGGGAATGAATTGAATCGAAAAAACAAACCACAAAATATCAAGAT

ACAAAAAAAGCCAGGGAAGTCAGAACTTCGCATTAACAAAGCATCACTGGCTGATTCTGGAGAGTATATG

TGCAAAGTGATCAGCAAATTAGGAAATGACAGTGCCTCTGCCAATATCACCATCGTGGAATCAAACGAGA

TCATCACTGGTATGCCAGCCTCAACTGAAGGAGCATATGTGTCTTCAGCTACATCTACATCCACCACTGG

GACAAGCCATCTTGTAAAATGTGCGGAGAAGGAGAAAACTTTCTGTGTGAATGGAGGGGAGTGCTTCATG

GTGAAAGACCTTTCAAACCCCTCGAGATACTTGTGCAAGTGCCCAAATGAGTTTACTGGTGATCGCTGCC

AAAACTACGTAATGGCCAGCTTCTACAAGCATCTTGGGATTGAATTTATGGAGGCGGAGGAGCTGTACCA

GAAGAGAGTGCTGACCATAACCGGCATCTGCATCGCCCTCCTTGTGGTCGGCATCATGTGTGTGGTGGCC

TACTGCAAAACCAAGAAACAGCGGAAAAAGCTGCATGACCGTCTTCGGCAGAGCCTTCGGTCTGAACGAA

ACAATATGATGAACATTGCCAATGGGCCTCACCATCCTAACCCACCCCCCGAGAATGTCCAGCTGGTGAA

TCAATACGTATCTAAAAACGTCATCTCCAGTGAGCATATTGTTGAGAGAGAAGCAGAGACATCCTTTTCC

ACCAGTCACTATACTTCCACAGCCCATCACTCCACTACTGTCACCCAGACTCCTAGCCACAGCTGGAGCA

ACGGACACACTGAAAGCATCCTTTCCGAAAGCCACTCTGTAATCGTGATGTCATCCGTAGAAAACAGTAG

GCACAGCAGCCCAACTGGGGGCCCAAGAGGACGTCTTAATGGCACAGGAGGCCCTCGTGAATGTAACAGC

TTCCTCAGGCATGCCAGAGAAACCCCTGATTCCTACCGAGACTCTCCTCATAGTGAAAGGTATGTGTCAG

CCATGACCACCCCGGCTCGTATGTCACCTGTAGATTTCCACACGCCAAGCTCCCCCAAATCGCCCCCTTC

GGAAATGTCTCCACCCGTGTCCAGCATGACGGTGTCCATGCCTTCCATGGCGGTCAGCCCCTTCATGGAA

GAAGAGAGACCTCTACTTCTCGTGACACCACCAAGGCTGCGGGAGAAGAAGTTTGACCATCACCCTCAGC

AGTTCAGCTCCTTCCACCACAACCCCGCGCATGACAGTAACAGCCTCCCTGCTAGCCCCTTGAGGATAGT

GGAGGATGAGGAGTATGAAACGACCCAAGAGTACGAGCCAGCCCAAGAGCCTGTTAAGAAACTCGCCAAT

AGCCGGCGGGCCAAAAGAACCAAGCCCAATGGCCACATTGCTAACAGATTGGAAGTGGACAGCAACACAA

GCTCCCAGAGCAGTAACTCAGAGAGTGAAACAGAAGATGAAAGAGTAGGTGAAGATACGCCTTTCCTGGG

CATACAGAACCCCCTGGCAGCCAGTCTTGAGGCAACACCTGCCTTCCGCCTGGCTGACAGCAGGACTAAC

CCAGCAGGCCGCTTCTCGACACAGGAAGAAATCCAGGCCAGGCTGTCTAGTGTAATTGCTAACCAAGACC

CTATTGCTGTATAAAACCTAAATAAACACATAGATTCACCTGTAAAACTTTATTTTATATAATAAAGTAT

TCCACCTTAAATTAAACAATTTATTTTATTTTAGCAGTTCTGCAAATAGAAAACAGGAAAAAAACTTTTA

TAAATTAAATATATGTATGTAAAAATGTGTTATGTGCCATATGTAGCAATTTTTTACAGTATTTCAAAAC

GAGAAAGATATCAATGGTGCCTTTATGTTATGTTATGTCGAGAGCAAGTTTTGTACAGTTACAGTGATTG

CTTTTCCACAGTATTTCTGCAAAACCTCTCATAGATTCAGTTTTTGCTGGCTTCTTGTGCATTGCATTAT

GATGTTGACTGGATGTATGATTTGCAAGACTTGCAACTGTCCCTCTGTTTGCTTGTAGTAGCACCCGATC

AGTATGTCTTGTAATGGCACATCCATCCAGATATGCCTCTCTTGTGTATGAAGTTTTCTTTGCTTTCAGA

ATATGAAATGAGTTGTGTCTACTCTGCCAGCCAAAGGTTTGCCTCATTGGGCTCTGAGATAATAGTAGAT

CCAACAGCATGCTACTATTAAATACAGCAAGAAACTGCATTAAGTAATGTTAAATATTAGGAAGAAAGTA

ATACTGTGATTTAAAAAAAACTATATTATTAATCAGAAGACAGCTTGCTCTTACTAAAAGGAGCTCTCAT

TTACTTTATTTGATTTTATTTTTCTTGACAAAAAGCAACAGTTTTAGGGATAGCTTAGAAAATGGGTTCT

GGCTTGCTATCAGGGTAAATCTAACACCTTACAAGAGGACTGAGTGTCACTTTCTCTCTGGGGGAATGAT

CCAGCAGCTTATCTAGTTGACAATCAAAACACGGCTGATAAAGGTGCAATCATTTCTGACATGTATTTTT

CACTGATTTTGAAGCTAGTGATTGGTTGTGTCTTCTTGGCTCAAAAAGAAGCATATTACGGCACAAAAAG

CCCAGCCCAGACAGCACATGCAGCATTTTGTCTGAAATACTTCTAGAGTCAAACGTGCCTGCTGTACATA

GCGATGACTTGTCATCATAGGGAAGTATTTCCATCGTAGAGTGTTCAGAAGGAGTGACTGTATAGGTGGA

GAGAAGCTTAGTGACTCCGTTGAAATTTTAAAATGTGGATGACCACCCCTTTCTCCCCCTTATTTTTCTT

TTATCTTTCCATGTTGCCTTGATCAGGTCATAACTATGCATGAACATTTTTTATCAGGAATGGCCGATGT

GTATGTGATTTGTAATCACAAGTAATGATTCATCAGGAAATGTCAATCCTGTTGGAAAGATTGCACCTTT

ACTTGCAGAAGTGACCCCCACCTGTGTCCTGACCTCTCCATTTACAGGCTCTCTCACCCATTTCCCCCAC

CTCCTTTAATTTTTGCTTTACTGTCATAAAGTAGGACTAAGATTGGTCTAAGCATTGCATGTTCTTTTGT

GATGGTAAATCCAAAGGAAGGCCTATAAGTATTAACATTTGAAATAACTGCTAATTCAGGAAAATGGAAG

AAAAAAAATTATTTGAAACACAGAACCCATTTCATGGCCTGCCTGATATCTGTGAAATCAGGGCTGGAGC

TTTACTTAGGATTCACATGGCCTCCTAGGAACCATGGGACAAATGGGAAACAGGTTATCGGGGGATTCAT

GAAGTCAGTGAGAGTAATTGCTTCTTTTTTGCGGGTGAACTGAATGTATTTCTTCACCAAATCTTGATGT

TAACAATTAAAAAGAAGAAATGACATGCAAGTAGGTCTTAGCAGAAAAATGCAGGCTGGGCATGAGTCAT

GTTGTTACCCTCCCACATGCTCCTACAATCCACAGAGATGCCTGTCTGCAGGTTCTTGAAGTTATTGTTA

GTATTTGGTATCTCAAATTTTTCGTCACTGTTCACATGCCACTTTCTCTGTGCACAGTGGTATCCTCATT

TGCTTTTTAACCTACACTGAGGAGTCTTTGTCAGGTTGCACTGATTTTCCAATTCTGCAGTAATGAGTAA

GCTCACGGCATGGGGAAGAAGACAGTCAGTCCAATGAAGTTCTCTAAATTATTTTAACATTGCCTTTGAA

GGCCTTGACTCATCCTTAGCTATTTCAATGAAGAAATTCCTACCATGAATTTAAAACCCTAAAAATTCTG

TTTCAAATTCTTTGGGCATTGGGGTACTCAGATATCCCATTGTGGAAGAATTTTAAGAATAAATAGAAGT

TTCTGTTGAGAACCATGAGCAACATGTTTCTTACAATGAGAATTGCTATGCATTTTAAAATTGCAAATAT

ATATGAAAATTGAAGACAAGAGGAAATTGTATTTCTAACTTGATTCTGATCACTCACAGAGGTGGCATAT

TATTATAGTTGGGACATCCTTTGCACCCTTCATAAAAAAGGCCAGCTGACTGCTCAGCATCACCTGCCAA

GGCCACTAGATTTGTGTTTACAGGGGTATCTCTGTGATGCTTGTCACATCACTCTTGACCACCTCTGTTA

ATAAATTCCGACAGTGCAGTGGCGATCGGAGTGTGAACTTATGTTCCCAGCATATGGAAAGCTATCTTAG

GTTTTAAGGTAGTAGAAATTGCCCAGGAGTTTGACAGCAACTTTGTTTCCCGGGTCTAAAATCGTATCCC

ACTGAGGTGTATGCAGTGGAGCATAATACATGCAAATACATGCAAAACTCCTTTTGTTTCACCTAAGATT

CACTTTCTATCTTACTTTCCCTTCCTGCCTAGTGTGACTTTTGCCCCCAAGAGTGCCTGGACAGCATTCT

AGTTTCTACAAAATGGTCCTCTGTGTAGGTGAATGTGTCCCAAACCTGCTATCACTTTCTTGTTTCAGTG

TGACTGTCTTGTTAGAGGTGAAGTTTATCCAGGGTAACTTGCTCACTAACTATTCCTTTTTATGGCCTGG

GGTTAAAGGGCGCATGGCTCACACTGGTGAAAATAAGGAAGGCCTGGTCTTATCTTGTATTAATAATACT

GGCTGCATTCCACCAGCCAGAGATTTCTATCTGCGAAGACCTATGAAACACTGAAGAGAAATGTAGGCAG

AAGGAAATGGCCACATATCACAAGTTCTATTATATATTCTTTTGTAAATACATATTGTATATTACTTGGA

TGTTTTCTTATATCATTTACTGTCTTTTTGAGTTAATGTCAGTTTTTACTCTCTCAACTTACTATGTAAC

ATTGTAAATAACATAATGTCCTTTATTATTTATATTTAAGCATCTAACATATAGAGTTGTTTTCATATAA

GTTTAAGATAAATGTCAAAAATATATGTTCTTTTGTTTTTCTTTGCTTTAAAATTATGTATCTTTTCCTT

TTCTTTTTTTTAAGAATAATTTATTGTTCAGGAGAAAGAATGTATATGTAACTGAAACTATCTGAAGAAT

GCACATTGAAGGCCGTGAGGTACTGATAAACTAAAGAATTTATTATTCAAAATACTAAGCAATAAGTAAT

TGTGATTTATTTAAAGTTTTGTCCATTTTCCATGAAAGACATACTGCAATAAAAATGCTACTCTGTGGAG

ACCTGGGAGTGTTGCTCAGCAGACTACAGCTTCAGTCTGTTAGACCAGCACCTTTCATCTCATTCCCATA

GTTATGCTAATTTAGGATTGTGTTCCATGGACCCCATGATCACCTTGTCTATACGTTGCTTCTTGTCTGT

CCATTGCTTTTGCCACACCACCTGTTCTCAAATCATCTCCTCCCTACCAATGCTGTTTATCACTTTCTTC

CTTGTTGAAGAGGCCACACAACCAGACAGTACTATGCTTCCTTTTTCCTCCATACACAATAACAGAGAGA

GAATATTCTAGGGCATGACTGCCTGGATCCTGGCTGTTGCTATCTTTTGTAGTGGCAGTAAGAAACTCCT

TCAGACTAATGAAAATGTCAACGTGCCATTCAATCACGAAAGGTAACGAAAAATGCTCTCATGGTTCAAA

TAGTCCAATGGCCCATAGTGGCCTAAAAGGCAGCCAGTTGACACCTGGCCATGCTAAGCTTCCTTATACC

ATCCGCTAATGACTTTCCATTGGGCCCACAATTTACGGATTCATAATTTTAAAAGAGGAGAAGGCCAAGT

TAGGTTCATTCCCCTTATTCTGTCAATAAAACAAATCAAACTCATGTCTATCTAACTGCTCAGGGAGGAG

CCTTTGCATGAGAAAATTCTCATATTCTAAGACTGAGTCATAGAAATGAGGGTATTACTTTTCTTACTGC

AATTAACCTAAACAAAAGCCATATTTTAACAAATAGATATTTGCATGGTACCCTTCATATATTCCAAGCA

TTTCACTCATTATTCCAGGTAGGTTAAGAGCTTCTGAAGTGTATGAAGTAAAGGTCAGCAATCCTTTGGG

GTGAACAGTGGCCTCCTTTGGAGTTTGGGGGTAACCTGAGACTTCCCACCAATGTCCACCTCCATCTGTG

TACCTAATTCCTATTACCTAGTTATGGCTCCTCTAGGATCATTTCCAAACACTCTGGATGTCCAGGAAAT

TTAAATTGTAGCTTTTGACTGAGCTAGTTTTTCCTATTTATATTAATAAATTTTCAAAAATGCTTGAAAT

CTTCACATTTGCAACAACTTTAGTTTTCATGCACATACAAACACAGAGAGACAAAAATTCCAAACAGACA

CTCTCCAAAAGCCACCACACTCTTTCACTTGCTCTATAGTCATTTAGCCAACCAGCCATGCAGAGAATAT

TTAAAACTTAAAGATTGAGACATTATTCTCAGTTTTTGCTGAGGCTTTGTAACGAAATTGAACACTATAA

GCAGCTATTGTAGTAATTTTGGTTAAAATTGTTTGCCTGGGATATAGTATTTGAGGCAGAAGCACGTGTG

TGAAGGAGGTGAGGTGGTTTGGAAAGAGTGAAGACTCGCAGCCAGATTGAATGTCTGGATAATTACTATA

ATTCTCCCTTCTTGGTTGAAACCATGTTCTCTCTTGATTTTTAAACCCAGGCTGCCTCTGGAAACAAGCA

AACCTGAGTCTTTCTAACCTGAGTCTTCCCAATCATTAGATTTCTTTTCTGTCCTAACGATGAATGATAA

AAGGACTTGATGTTCACAATTTGGGGTTATAAGGCAGGTCTGAAATCTGGAGACTCAAGATGCTGGAAGG

AGTGGAAAGTTTCGATGACTTTATATGAATCACTTTGCACTCTATGTTTGGCTTGTCCTCTTTGAAACTG

ATTTACTAAAATAAATGTAAGGGAACTATTACTCCAAAAGATTAACTTGGCAGGAAATACCAATACTTTC

AGTTTATGAAAGACAAAACTGTCTTGTTGCTACAGGAAGCTGCAATGTTCCTAACCTTTAAGGTTGGTGT

TGAATAGGGTGGTCATGCCCTCCCCTGCAGGTATCTTTAGGCTCCTGTTGACCTCCTGGTACTATAACTG

TTCGTCTTCTCTGGGTAGCTATTGATTTTGAACTTTAACATGCTTCAAAACTTTATTCATCAGGGAAATA

GGAAAAGAGTTTTGTTACCTGGAGGAAATCTATTGTGATCTACCTGAGCTTTTTAAAAACAGACCAGGAG

AAGGAAACCAGTAATTTTTAAAGAAGAGACAGAGAATGGGATAATAGTTTCACCCAGGATCTCTTTCTAA

CCCTTTCCCTTCAAATGAACTTATTGGAACAGAATTGGAAAGAAGAAAGGACATCTCTGCCCACCCCACA

GGATGCCAAAAAGGCTAAAGAATTACCTCTGTAGATTTAAACATCTTTAATGGCTTATGTATAGATTTGC

TAATACAGAGAGAAATGAACTATTAAATAAAAATCACATTTTATAATATTTTTATGGCTTAAAACATCCT

TTATCTCCTTTTTGTTCTCTCTACATGATATGGTAAGTGATGAGGAAAATTTAGGCTCAGGAAGGTTAAA

ATCTTTCTTGGAGTTACACATCTAAGAGAGCTGCAGAGCTGACACTTGTACCCAGGTTTTCTGACTGCAA

ATCCAGTTTCTTTCTATTGCGTTCTTCCCCTTTCCCTGCCTCAAGCAGAAACAGGTTTTTTATTTTCAAC

CTTTATGTATACAGTATGTTATGTTACATCTACAGCTAAGTTTCTTTTTAGAAGAATGTGAGCCCTTCTA

GCTTTGGTTTAGAGTGATTCTAGAAGCCAATTTCCTTGGCTTAGTGATTCTATGCACCTTTCCTAAACTT

AGCTTTCTAAGGAAATGAAGTGTACGAGTGAGAATGAATTCACAATTTCGACATGTAGGTAGCATCCTAA

AGTGAAAAGAGGAGGAAATTTGTGGTCAAAGCACTCTCCCCACCACTTAGAAACTTACTGACTGTGGGCA

GCTTCCTCCTCCAAGTTTCCTTCCTGATTTACAAGACCGTGGTGTGGTCAGGATTAAACTTGAATACATG

TAAGGAAGCCTGAAAGTGTCTAACACATAGCGAGTATTCAAATGCCACCTTCTATTTGATCCTTCCCCTC

CAGTTCCTTAAGTTTTGGAATCTAGGTTTCTCAGTTCCAAATGGATTGACATTTGCATATCCCCATTGCA

CAATGGATCAAATAAACTTTATGTTATCATTTCTCCAACATAGTGCCAGTAAGCAAATCCTTTTTAATAA

CAACAGTATGTTGAGAAACATATCACCAAATAATATTTAACTTTGTAGCTTTGATAAGTTCTTTAGGTTT

TGGTTTTGGTTTTGTTTTCTGAGACAGGGTCTTGATCTGTCACCCAGACTGGAGTGCAATGGTTCAATTT

TAGCTCACTGCAACCTGTAACTCCTGAGCTCAAGTGATCCTCCCACCTCAGCCTCTCAAGTAGCTGGGAC

TACAGGTGTGCTCCACCATGCCCAGCGATTTTTTTTTTTTTTTTTTTTTTTTTTTTTGGTAGGGACAAGG

TCTCGCTATGTTGCCCAGGCTGGTCTTCAATTCCTGGCCTTAAGTGATCCTCCTGCCTCGGCCTCCCAAA

GTGCTGGAATTACAGGCATGAGCCACCACCCATAACTTTATGTTTGTTTTTTTGATGCAGTATAAGTTCA

GCTTGCTTCTTATGCAGCCATACCATTTCATGTTAACTCTGATTTTTAGCAGCTTATTACATTAGTGTTT

TATTATTAATATAATTTTACAGAAATTTACTAAACCATGACTCTGTAGAGTTTTAATAATACTACCTCCA

AACATCATTGCAAACATCTAGAAGAATGAACAAAAATGATCTTAGATCGACAGTATATCTGTTTGTCTTA

GTTTCTACACAGGATGTTCAGACATATTCCATTTCTTTAAAAAAAAAAATATATATATATATATATATATA

TAGGCCTGGCACGGTGGCTCATGACTGTAATCCCAGCACTTTGGGAGGCTTAGGCAGGCAAATCACCTGA

GGTCAGGAGTTTGAGACTGGCCTGACCAACATGGTGAAACCTCGTCTCTATTAAAATTACAAAAATTAGC

CGGGCGTGGTGGCACATGCCTGTAATCCCAGCTACTCGGGAGGCTGAGGCAGGAGAATCACTTGAACCTG

GGAGGCAGAGGTTGCAGTGAGTTGAGATCACGCCATTGCACTCCAGCCTTGGTGACAAGAGTGAAACTCC

GTCTCAAAAAAAAAAAAAATATGTATATATATATATATATATATATATATATATATATATATATATATATAT

AAAATCCCACCAAAAGTCTGCAGAGTGACCAAATTAGACGGCTCTGGTTTCAGATTAAATTCTAAATGTG

AGAAACCACATAGCTCCCATGATCCATCCAATAATTCCTCACGTCCTCTTCACTCTTTACTCCATGCATA

AAACAGAATTTTTTTTTCTCATCCTGGGTATGAAGCAATTAATAATTTACGGATTTAGCCTATTTGGATT

CAATCCCTTCAAACTCCATACTACATCCAAGGTGGAAGTGACTTAAACTCTGATATCAATCATCAGGCTT

GTAATATAGGCTTTGTTAATGGCAGGAGAGTCTAATAAAACTTTCTGTTCCTTATCCTTCATTTAAATGA

AAAACTTTTTATTGAAAACAATCATAACTCTAGCTCATCATAAATATAATTCATGAGGACATTTTATTAT

TTTTATATTAAAGAAATAATATTATAGATGTAAACTTTGCACCTTTCTAATTATTATCATGAGTTAAGCT

AATACTTGTCTTCTGGTCCCTAGATGATGATTCTTTTTTGCCTTACTGGAGGAGCCCTTGTCTTGAAGTG

AGTTGCTTCAACAGCAGAGGACTTCTAGTTTCTCCCAGTTGAGCCTAAAGTGAACTTTTCATCTTCTTCA

GAGGAAGGGGCTTCCTTGATTTGTACTTTTGTGGCTCCTCAGATAACACAGACAATTTTATCTTGGATCC

CAGGTTCTCTTCACCATTAAGAATAAGAAAGAGAGAAAATGCTGTGCATGACAGCCACCTACTCCAAACT

ACCCAACCCCCTGTAACCAGGTACCTTCCAACAACGAGATGATTCTGCCCTCACTCAAGAGTCTCCCCCA

CAAAGATTCCATTCTCCCTTTACTTTTTATTTTTTATTTTTTTGCAAAACAAAGGCCTCCTTTAGTACCT

CCTTAGTTTATAACCCTTATCTTCCCAGCTCTTCCCTTCACTGATACCTCTGATTTCAAAAGTTCTGAAG

TCGGAAGACCACACAATTTCAGACTGTGAACAGAAATTCAGTCAGAAATTATTGGAGTTAGAAAGAATTT

AGAGAAATTGTATTGAACTAGAAAGTCCTCTTTGATTAGTGGTGGCCCTTTAGAAAGTTCTAGGGCAGAG

TCCCATGGTGTTTCATCTTTTCCATGATTTGCTTGACCAAAAACTTTTCTTTCACAACATGAAAATATGC

TAATCCACCACACATTTTGGATTCTGCTCTGTTTGCCTGAGGTGTTAGATCTCTAGCCAGGACTGTGAAG

GGAAGGAACTTGAATCCTTCCTATTGAGCTATTAATGCAGAGTCAGTGAGATGAAGGGTTCCACTCGGGG

TCAAAATCATGTCAGTTACCAAGCAAAGGAGCAAGTAAGGGGAAACATCTCCTCATCTGGTTAGTGGAGC

CACATTTCACCCACTGATCAAGCCAGACCTGAGCAATAGTCTAGATTTCTCCCTCCACATCTAATTGGTG

ACAATGGTGATTGTACCACTGACAGGTCACACAAGTCCACACCCTCCTTCACAGCCTCACTGCTTCGGCT

CTTGTTTATGCTCTTATCAGCATCTGTCACTAGGATCCATTTGTCTCCTGACTCATCTACTTCCTTTCAC

TCCCCTGGGCCATCCTTCACATCATGCTAGAAGACTGTGTCTAACTTGCAGAACTTATTGTGTCAATCTC

CTGGTTACGGCCCCTCCATGGCTCCCCACCTGACATAGGAGGCCCTTCACAATCTGGCTTCTGTCACTCA

TAACTTGTCTCCAGCCTTCATTCTTCAGTCTGATTTTATGGTTTTCTAGTTCCCCAATACACCACACCAG

TGTTTATGAAACCCTAGTCATTGGCCTACGAACTTTATGATTATTGACATATTCATGTACCACCTGTATT

ATTTTTTGCATAGTGTTTATTTTTAATTGACTACATTTTTAACTACAATAAAGTAATTTCAACTAAAA

>NM_001010848.4 Homo sapiens neuregulin 3 (NRG3), transcript variant 1, mRNA

GGATTTGCATGCGGCCGCCGCGGCCGCTGCCTGCGCCCGAGCCCGCCGCCGCCGGAGCCCGCGCCCG

CGCCCGCGCCCGGCCCGCGCGGCCCCATGCCTCTGCCGCGGCCCTCGGGGGGGCGAAGGTGAAGACCGGC

TCCTAGGATGAGTGAAGGGGCGGCCGCTGCCTCGCCACCTGGTGCCGCTTCGGCAGCCGCCGCCTCGGCC

GAGGAGGGCACCGCGGCGGCTGCGGCGGCGGCAGCGGCGGGCGGGGGCCCGGACGGCGGCGGCGAAGGGG

CGGCCGAGCCCCCCCGGGAGTTACGCTGTAGCGACTGCATCGTGTGGAACCGGCAGCAGACGTGGCTGTG

CGTGGTACCTCTGTTCATCGGCTTCATCGGCCTGGGGCTCAGCCTCATGCTTCTCAAATGGATCGTGGTG

GGCTCCGTCAAGGAGTACGTGCCCACCGACCTAGTGGACTCCAAGGGGATGGGCCAGGACCCCTTCTTCC

TCTCCAAGCCCAGCTCTTTCCCCAAGGCCATGGAGACCACCACCACTACCACTTCCACCACGTCCCCCGC

CACCCCCTCCGCCGGGGGTGCCGCCTCCTCCAGGACGCCCAACCGGATTAGCACTCGCCTGACCACCATC

ACGCGGGCGCCCACTCGCTTCCCCGGGCACCGGGTGCCCATCCGGGCCAGCCCGCGCTCCACCACAGCAC

GGAACACTGCGGCCCCTGCGACGGTCCCGTCCACCACGGCCCCGTTCTTCAGTAGCAGCACGCTGGGCTC

CCGACCCCCGGTGCCAGGAACTCCAAGTACCCAGGCAATGCCCTCCTGGCCTACTGCGGCATACGCTACC

TCCTCCTACCTTCACGATTCTACTCCCTCCTGGACCCTGTCTCCCTTTCAGGATGCTGCCTCCTCTTCTT

CCTCTTCTTCCTCCTCCGCTACCACCACCACACCAGAAACTAGCACCAGCCCCAAATTTCATACGACGAC

ATATTCCACAGAGCGATCCGAGCACTTCAAACCCTGCCGAGACAAGGACCTTGCATACTGTCTCAATGAT

GGCGAGTGCTTTGTGATCGAAACCCTGACCGGATCCCATAAACACTGTCGGTGCAAAGAAGGCTACCAAG

GAGTCCGTTGTGATCAATTTCTGCCGAAAACTGATTCCATCTTATCGGATCCAACAGACCACTTGGGGAT

TGAATTCATGGAGAGTGAAGAAGTTTATCAAAGGCAGGTGCTGTCAATTTCATGTATCATCTTTGGAATT

GTCATCGTGGGCATGTTCTGTGCAGCATTCTACTTCAAAAGCAAGAAACAAGCTAAACAAATCCAAGAGC

AGCTGAAAGTGCCACAAAATGGTAAAAGCTACAGTCTCAAAGCATCCAGCACAATGGCAAAGTCAGAGAA

CTTGGTGAAGAGCCATGTCCAGCTGCAAAATTATTCAAAGGTGGAAAGGCATCCTGTGACTGCATTGGAG

AAAATGATGGAGTCAAGTTTTGTCGGCCCCCAGTCATTCCCTGAGGTCCCTTCTCCTGACAGAGGAAGCC

AGTCTGTCAAACACCACAGGAGTCTATCCTCTTGCTGCAGCCCAGGGCAAAGAAGTGGCATGCTCCATAG

GAATGCCTTCAGAAGGACACCCCCGTCACCCCGAAGTAGGCTAGGTGGAATTGTGGGACCAGCATATCAG

CAACTCGAAGAATCAAGGATCCCAGACCAGGATACGATACCTTGCCAAGGGTATTCATCCAGTGGTTTAA

AAACCCAACGAAATACATCAATAAATATGCAACTGCCTTCAAGAGAGACAAACCCCTATTTTAATAGCTT

GGAGCAAAAGGACCTGGTGGGCTATTCATCCACAAGGGCCAGTTCTGTGCCCATCATCCCTTCAGTGGGT

TTAGAGGAAACCTGCCTGCAAATGCCAGGGATTTCTGAAGTCAAAAGCATCAAATGGTGCAAAAACTCCT

ATTCAGCTGACGTTGTCAATGTGAGTATTCCAGTCAGCGATTGTCTTATAGCAGAACAACAAGAAGTGAA

AATATTGCTAGAAACTGTCCAGGAGCAGATCCGAATTCTGACTGATGCCAGACGGTCAGAAGACTACGAA

CTGGCCAGCGTAGAAACCGAGGACAGTGCAAGCGAAAACACAGCCTTTCTCCCCCTGAGTCCCACAGCCA

AATCAGAACGAGAGGCGCAATTTGTCTTAAGAAATGAAATACAAAGAGACTCTGCATTGACCAAGTGACT

TGAGATGTAGGAATCTGTGCATTCTATGCTTTGCTCAACAGGAAAGAGAGGAAATCAAATACAAATTATT

TATATGCATTAATTTAAGAGCATCTACTTAGAAGAAACCAAATAGTCTATCGCCCTCATATCATAGTGTT

TTTTAACAAAATATTTTTTTAAGGGAAAGAAATGTTTCAGGAGGGATAAAGCTTACCATTAAAGCTTTTG

GGTAGAATTCTGAATCCAATTTCGTTTAGTCCCAACACTGTCCTCTTTGGCTCTTAGAAGATGTGGGCAA

TTCAGACCCTTGGCCCCACAGTGCCAGTGTCTCATTAAAAAACACTGGCAGTTACCTGGTTTCAAAAAGA

GGAGAGCTGCATCCCGCAGGTGGATGCACCAGTGAGCAGGTGGCTCTTGCCATTTGGCTTGCCAGTCCCA

AGCCCTAAATTTCTATTCACCCAATAGCCTCATCACAGGTTATGTCATGTCGACACATGTAGTGTTTTCT

ATTTGATTTTTGGAGAATGCCACAAAAGACTCTGCAATGGGAAGGAGGACAGGAGATAGAAGGAGAAACC

GGGGGCAATCTTAACTACTCTTGTGTGCCACCTTCCTCTGAGATTTGAATGCACAGATCTCAGCTGGAGG

TATGAAATTATTACAGAAAAAGAGACAAAAAAATCCTTATATCGTGTCACTAAAATCATGCTAATAGAAA

TGTTTTTCCTTGAGATTGTTTAGAGGCTCTGGAGGAGCGTTTCTCAGTTTTTGTGTGCCTGTGTGCACAT

GTGTGTGCGAGCGTGTGTGTGTGTGGACTTGCTCACGCGGGCACATATGCTGTAAGCACATGTGTTCATT

GTGCGTATGTGTGTGCATGTGTGCGCGTATTACGCTTGCTAAAATTTGTTCTGAAACATCAGGGAATCTA

CTATTCAGAAAAATTATTTCCCTCTTAGATCTTTTCACTTTAAATTTTTCCATTAAGTATAAAGTTCAGT

TAGTTCTTAAATCAAGGTCACTTGCATGGTGGGGTCGTATAAAACTCTTGACACTGTCTAGACCATTTTC

TGATGTGAATACTTTTGAGAGGATCAACTATTGGCTCATTAATGATATCAGTATCATAAGGTGAGTTCAT

CAAGGATGTGTGTTTATTTTGAATCATGCCTACTTAAATTATTAATACAAGACAATTAACAAATTGACAG

TTACCATTTGCTTTCTCATCATCCTCATTACCATTTATTTTATAGCAAGTCTGCTATGTGTGGACCAAGG

CTTCGGCTTCTGTGGTTAGTATGGGAAGAATAAATTGTTGAAATAAAAATACCCAGACTATTCAGTTCAC

AAGAAGCCCCCCAAAAGAACAGTAAATTGTGTTGTATGTTCATTTGGAATAAAGCAATATTTTTACCACT

GCTAAGGTGAACTGAAACCTCTCCTGAAGATTTGTCTGTTTATTTACCCTCACTTTCATGGGACATATGA

GGAAATTAGTGTTCACATATCCAGTCTTTTTCCAATTATTGGTGGTGTTGAGTTGTTATGTTTACACTGT

TTTAAATAAAAAGGCACCGTATTTGAAAGCA

>NM_000918.4 Homo sapiens prolyl 4-hydroxylase subunit beta (P4HB), mRNA

GCTCTCGTCGCCCCCGCTGTCCCGGCGGCGCCAACCGAAGCGCCCCGCCTGATCCGTGTCCGACATGCTG

CGCCGCGCTCTGCTGTGCCTGGCCGTGGCCGCCCTGGTGCGCGCCGACGCCCCCGAGGAGGAGGACCACG

TCCTGGTGCTGCGGAAAAGCAACTTCGCGGAGGCGCTGGCGGCCCACAAGTACCTGCTGGTGGAGTTCTA

TGCCCCTTGGTGTGGCCACTGCAAGGCTCTGGCCCCTGAGTATGCCAAAGCCGCTGGGAAGCTGAAGGCA

GAAGGTTCCGAGATCAGGTTGGCCAAGGTGGACGCCACGGAGGAGTCTGACCTGGCCCAGCAGTACGGCG

TGCGCGGCTATCCCACCATCAAGTTCTTCAGGAATGGAGACACGGCTTCCCCCAAGGAATATACAGCTGG

CAGAGAGGCTGATGACATCGTGAACTGGCTGAAGAAGCGCACGGGCCCGGCTGCCACCACCCTGCCTGAC

GGCGCAGCTGCAGAGTCCTTGGTGGAGTCCAGCGAGGTGGCTGTCATCGGCTTCTTCAAGGACGTGGAGT

CGGACTCTGCCAAGCAGTTTTTGCAGGCAGCAGAGGCCATCGATGACATACCATTTGGGATCACTTCCAA

CAGTGACGTGTTCTCCAAATACCAGCTCGACAAAGATGGGGTTGTCCTCTTTAAGAAGTTTGATGAAGGC

CGGAACAACTTTGAAGGGGAGGTCACCAAGGAGAACCTGCTGGACTTTATCAAACACAACCAGCTGCCCC

TTGTCATCGAGTTCACCGAGCAGACAGCCCCGAAGATTTTTGGAGGTGAAATCAAGACTCACATCCTGCT

GTTCTTGCCCAAGAGTGTGTCTGACTATGACGGCAAACTGAGCAACTTCAAAACAGCAGCCGAGAGCTTC

AAGGGCAAGATCCTGTTCATCTTCATCGACAGCGACCACACCGACAACCAGCGCATCCTCGAGTTCTTTG

GCCTGAAGAAGGAAGAGTGCCCGGCCGTGCGCCTCATCACCCTGGAGGAGGAGATGACCAAGTACAAGCC

CGAATCGGAGGAGCTGACGGCAGAGAGGATCACAGAGTTCTGCCACCGCTTCCTGGAGGGCAAAATCAAG

CCCCACCTGATGAGCCAGGAGCTGCCGGAGGACTGGGACAAGCAGCCTGTCAAGGTGCTTGTTGGGAAGA

ACTTTGAAGACGTGGCTTTTGATGAGAAAAAAAACGTCTTTGTGGAGTTCTATGCCCCATGGTGTGGTCA

CTGCAAACAGTTGGCTCCCATTTGGGATAAACTGGGAGAGACGTACAAGGACCATGAGAACATCGTCATC

GCCAAGATGGACTCGACTGCCAACGAGGTGGAGGCCGTCAAAGTGCACAGCTTCCCCACACTCAAGTTCT

TTCCTGCCAGTGCCGACAGGACGGTCATTGATTACAACGGGGAACGCACGCTGGATGGTTTTAAGAAATT

CCTGGAGAGCGGTGGCCAGGATGGGGCAGGGGATGATGACGATCTCGAGGACCTGGAAGAAGCAGAGGAG

CCAGACATGGAGGAAGACGATGATCAGAAAGCTGTGAAAGATGAACTGTAATACGCAAAGCCAGACCCGG

GCGCTGCCGAGACCCCTCGGGGGCTGCACACCCAGCAGCAGCGCACGCCTCCGAAGCCTGCGGCCTCGCT

TGAAGGAGGGCGTCGCCGGAAACCCAGGGAACCTCTCTGAAGTGACACCTCACCCCTACACACCGTCCGT

TCACCCCCGTCTCTTCCTTCTGCTTTTCGGTTTTTGGAAAGGGATCCATCTCCAGGCAGCCCACCCTGGT

GGGGCTTGTTTCCTGAAACCATGATGTACTTTTTCATACATGAGTCTGTCCAGAGTGCTTGCTACCGTGT

TCGGAGTCTCGCTGCCTCCCTCCCGCGGGAGGTTTCTCCTCTTTTTGAAAATTCCGTCTGTGGGATTTTT

AGACATTTTTCGACATCAGGGTATTTGTTCCACCTTGGCCAGGCCTCCTCGGAGAAGCTTGTCCCCCGTG

TGGGAGGGACGGAGCCGGACTGGACATGGTCACTCAGTACCGCCTGCAGTGTCGCCATGACTGATCATGG

CTCTTGCATTTTTGGGTAAATGGAGACTTCCGGATCCTGTCAGGGTGTCCCCCATGCCTGGAAGAGGAGC

TGGTGGCTGCCAGCCCTGGGGCCCGGCACAGGCCTGGGCCTTCCCCTTCCCTCAAGCCAGGGCTCCTCCT

CCTGTCGTGGGCTCATTGTGACCACTGGCCTCTCTACAGCACGGCCTGTGGCCTGTTCAAGGCAGAACCA

CGACCCTTGACTCCCGGGTGGGGAGGTGGCCAAGGATGCTGGAGCTGAATCAGACGCTGACAGTTCTTCA

GGCATTTCTATTTCACAATCGAATTGAACACATTGGCCAAATAAAGTTGAAATTTTA

>NM_001618.4 Homo sapiens poly(ADP-ribose) polymerase 1 (PARP1), mRNA

AGCAATCTATCAGGGAACGGCGGTGGCCGGTGCGGCGTGTTCGGTGGCGGCTCTGGCCGCTCAGGCGCCT

GCGGCTGGGTGAGCGCACGCGAGGCGGCGAGGCGGCAGCGTGTTTCTAGGTCGTGGCGTCGGGCTTCCGG

AGCTTTGGCGGCAGCTAGGGGAGGATGGCGGAGTCTTCGGATAAGCTCTATCGAGTCGAGTACGCCAAGA

GCGGGCGCGCCTCTTGCAAGAAATGCAGCGAGAGCATCCCCAAGGACTCGCTCCGGATGGCCATCATGGT

GCAGTCGCCCATGTTTGATGGAAAAGTCCCACACTGGTACCACTTCTCCTGCTTCTGGAAGGTGGGCCAC

TCCATCCGGCACCCTGACGTTGAGGTGGATGGGTTCTCTGAGCTTCGGTGGGATGACCAGCAGAAAGTCA

AGAAGACAGCGGAAGCTGGAGGAGTGACAGGCAAAGGCCAGGATGGAATTGGTAGCAAGGCAGAGAAGAC

TCTGGGTGACTTTGCAGCAGAGTATGCCAAGTCCAACAGAAGTACGTGCAAGGGGTGTATGGAGAAGATA

GAAAAGGGCCAGGTGCGCCTGTCCAAGAAGATGGTGGACCCGGAGAAGCCACAGCTAGGCATGATTGACC

GCTGGTACCATCCAGGCTGCTTTGTCAAGAACAGGGAGGAGCTGGGTTTCCGGCCCGAGTACAGTGCGAG

TCAGCTCAAGGGCTTCAGCCTCCTTGCTACAGAGGATAAAGAAGCCCTGAAGAAGCAGCTCCCAGGAGTC

AAGAGTGAAGGAAAGAGAAAAGGCGATGAGGTGGATGGAGTGGATGAAGTGGCGAAGAAGAAATCTAAAA

AAGAAAAAGACAAGGATAGTAAGCTTGAAAAAGCCCTAAAGGCTCAGAACGACCTGATCTGGAACATCAA

GGACGAGCTAAAGAAAGTGTGTTCAACTAATGACCTGAAGGAGCTACTCATCTTCAACAAGCAGCAAGTG

CCTTCTGGGGAGTCGGCGATCTTGGACCGAGTAGCTGATGGCATGGTGTTCGGTGCCCTCCTTCCCTGCG

AGGAATGCTCGGGTCAGCTGGTCTTCAAGAGCGATGCCTATTACTGCACTGGGGACGTCACTGCCTGGAC

CAAGTGTATGGTCAAGACACAGACACCCAACCGGAAGGAGTGGGTAACCCCAAAGGAATTCCGAGAAATC

TCTTACCTCAAGAAATTGAAGGTTAAAAAACAGGACCGTATATTCCCCCCAGAAACCAGCGCCTCCGTGG

CGGCCACGCCTCCGCCCTCCACAGCCTCGGCTCCTGCTGCTGTGAACTCCTCTGCTTCAGCAGATAAGCC

ATTATCCAACATGAAGATCCTGACTCTCGGGAAGCTGTCCCGGAACAAGGATGAAGTGAAGGCCATGATT

GAGAAACTCGGGGGGAAGTTGACGGGGACGGCCAACAAGGCTTCCCTGTGCATCAGCACCAAAAAGGAGG

TGGAAAAGATGAATAAGAAGATGGAGGAAGTAAAGGAAGCCAACATCCGAGTTGTGTCTGAGGACTTCCT

CCAGGACGTCTCCGCCTCCACCAAGAGCCTTCAGGAGTTGTTCTTAGCGCACATCTTGTCCCCTTGGGGG

GCAGAGGTGAAGGCAGAGCCTGTTGAAGTTGTGGCCCCAAGAGGGAAGTCAGGGGCTGCGCTCTCCAAAA

AAAGCAAGGGCCAGGTCAAGGAGGAAGGTATCAACAAATCTGAAAAGAGAATGAAATTAACTCTTAAAGG

AGGAGCAGCTGTGGATCCTGATTCTGGACTGGAACACTCTGCGCATGTCCTGGAGAAAGGTGGGAAGGTC

TTCAGTGCCACCCTTGGCCTGGTGGACATCGTTAAAGGAACCAACTCCTACTACAAGCTGCAGCTTCTGG

AGGACGACAAGGAAAACAGGTATTGGATATTCAGGTCCTGGGGCCGTGTGGGTACGGTGATCGGTAGCAA

CAAACTGGAACAGATGCCGTCCAAGGAGGATGCCATTGAGCACTTCATGAAATTATATGAAGAAAAAACC

GGGAACGCTTGGCACTCCAAAAATTTCACGAAGTATCCCAAAAAGTTCTACCCCCTGGAGATTGACTATG
GCCAGGATGAAGAGGCAGTGAAGAAGCTGACAGTAAATCCTGGCACCAAGTCCAAGCTCCCCAAGCCAGT
TCAGGACCTCATCAAGATGATCTTTGATGTGGAAAGTATGAAGAAAGCCATGGTGGAGTATGAGATCGAC
CTTCAGAAGATGCCCTTGGGGAAGCTGAGCAAAAGGCAGATCCAGGCCGCATACTCCATCCTCAGTGAGG
TCCAGCAGGCGGTGTCTCAGGGCAGCAGCGACTCTCAGATCCTGGATCTCTCAAATCGCTTTTACACCCT
GATCCCCCACGACTTTGGGATGAAGAAGCCTCCGCTCCTGAACAATGCAGACAGTGTGCAGGCCAAGGTG
GAAATGCTTGACAACCTGCTGGACATCGAGGTGGCCTACAGTCTGCTCAGGGGAGGGTCTGATGATAGCA
GCAAGGATCCCATCGATGTCAACTATGAGAAGCTCAAAACTGACATTAAGGTGGTTGACAGAGATTCTGA
AGAAGCCGAGATCATCAGGAAGTATGTTAAGAACACTCATGCAACCACACACAATGCGTATGACTTGGAA
GTCATCGATATCTTTAAGATAGAGCGTGAAGGCGAATGCCAGCGTTACAAGCCCTTTAAGCAGCTTCATA
ACCGAAGATTGCTGTGGCACGGGTCCAGGACCACCAACTTTGCTGGGATCCTGTCCCAGGGTCTTCGGAT
AGCCCCGCCTGAAGCGCCCGTGACAGGCTACATGTTTGGTAAAGGGATCTATTTCGCTGACATGGTCTCC
AAGAGTGCCAACTACTGCCATACGTCTCAGGGAGACCCAATAGGCTTAATCCTGTTGGGAGAAGTTGCCC
TTGGAAACATGTATGAACTGAAGCACGCTTCACATATCAGCAAGTTACCCAAGGGCAAGCACAGTGTCAA
AGGTTTGGGCAAAACTACCCCTGATCCTTCAGCTAACATTAGTCTGGATGGTGTAGACGTTCCTCTTGGG
ACCGGGATTTCATCTGGTGTGAATGACACCTCTCTACTATATAACGAGTACATTGTCTATGATATTGCTC
AGGTAAATCTGAAGTATCTGCTGAAACTGAAATTCAATTTTAAGACCTCCCTGTGGTAATTGGGAGAGGT
AGCCGAGTCACACCCGGTGGCTCTGGTATGAATTCACCCGAAGCGCTTCTGCACCAACTCACCTGGCCGC
TAAGTTGCTGATGGGTAGTACCTGTACTAAACCACCTCAGAAAGGATTTTACAGAAACGTGTTAAAGGTT
TTCTCTAACTTCTCAAGTCCCTTGTTTTGTGTTGTGTCTGTGGGGAGGGGTTGTTTTGGGGTTGTTTTTG
TTTTTTCTTGCCAGGTAGATAAAACTGACATAGAGAAAAGGCTGGAGAGAGATTCTGTTGCATAGACTAG
TCCTATGGAAAAAACCAAGCTTCGTTAGAATGTCTGCCTTACTGGTTTCCCCAGGGAAGGAAAAATACAC
TTCCACCCTTTTTTCTAAGTGTTCGTCTTTAGTTTTGATTTTGGAAAGATGTTAAGCATTTATTTTTAGT
TAAAAATAAAAACTAATTTCATACTATTTAGATTTTCTTTTTTATCTTGCACTTATTGTCCCCTTTTTAG
TTTTTTTTGTTTGCCTCTTGTGGTGAGGGGTGTGGGAAGACCAAAGGAAGGAACGCTAACAATTTCTCAT

ACTTAGAAACAAAAAGAGCTTTCCTTCTCCAGGAATACTGAACATGGGAGCTCTTGAAATATGTAGTATT

AAAAGTTGCATTTGAAATTCTTGACTTTCTTATGGGCACTTTTGTCTTCCAAATTAAAACTCTACCACAA

ATATACTTACCCAAGGGCTAATAGTAATACTCGATTAAAAATGCAGATGCCTTCTCTA

>NM_003768.5 Homo sapiens proliferation and apoptosis adaptor protein 15 (PEA15), transcript variant 2, mRNA

GGCTCCGCGGGCGGAAGAGGCGGCGGCGGCGGCAGAAGCGGCGGCGGCGGCGGCGGGAGCCGAGGAGGAG

GTTCCGGACGCTGCTTAGGAACCGGGGACTCAGGAGTGCCCGCGCCCTGAGCGCTCAGCTCCAGAGGCGT

CATGGCTGAGTACGGGACCCTCCTGCAAGACCTGACCAACAACATCACCCTTGAAGATCTAGAACAGCTC

AAGTCGGCCTGCAAGGAAGACATCCCCAGCGAAAAGAGTGAGGAGATCACTACTGGCAGTGCCTGGTTTA

GCTTCCTGGAGAGCCACAACAAGCTGGACAAAGACAACCTCTCCTACATTGAGCACATCTTTGAGATCTC

CCGCCGTCCTGACCTACTCACTATGGTGGTTGACTACAGAACCCGTGTGCTGAAGATCTCTGAGGAGGAT

GAGCTGGACACCAAGCTAACCCGTATCCCCAGTGCCAAGAAGTACAAAGACATTATCCGGCAGCCCTCTG

AGGAAGAGATCATCAAATTGGCTCCCCCACCGAAGAAGGCCTGAGCAAGGGGGAGGAAGAGGAGGAAGGT

TGGACCTTCATCAGACCACTCCCTTCCCCCATCCTCCAGGAGAGGGGGCAAGGGCAACCCACCATCTACC

CACTTACTAACCTGGTCCTAACCCCCTTACTGTGCGCGTGTGTGTGCGTGTGCGCACGCTCTGGCTGTTT

GTCTATATGTCTAGCTCATCTAGTTCCTCTTCTTAAGGGGATGGGGGTCAGGGGCTAGGGGAGGGGGCTG

AGTTTCCCCACTTTAGGAGGAGGTGGGGGCTATTTCTATGCAAATAGAAATCAGCACATTCCTCCTACTT

CCCTTTCCTCCACTCCCCCCATATCTTTAAAGTGTGGAAGCAGAAAGGACCTGCATTTTCCTACATTGAG

GAGCTGACATAGGGGTAAGGTATGGGAGAGGTAGGTGGATCCAGGGAAAAGCAGTGGGGACGGAAGGCAA

AGAGACCACTCAACCCCCACCTGGAAGGGGCAAAGAAAAGCCAGAGTTCCATGTTTGTACTCCTGTGCTG

GACTGTTTCCTGAGTACCAGCAGGTCCCTTTTTGTCTCTCATGGGCCTAGCATAGGTATGAGCCAGGGAT

CCTTTCCTGGTCCCTAAGATCAAACCCCATGGAGCAGCCAGCGTTAGATGCCCCCACCCACCTGTACTCT

GGAGAGACTGTGCTGGGAACATGTACCACTGAGCCTGAGATGGGGATGAGGGCAGAGAGAGGGGAGCCCC

CTCTTCCACTCAGTTGTTCCTACTCAGACTGTTGCACTCTAAACCTAGGGAGGTTGAAGAATGAGACCCT

TAGGTTTTAACACGAATCCTGACACCACCATCTATAGGGTCCCAACTTGGTTATTGTAGGCAACCTTCCC

TCTCTCCTTGGTGAAGAACATCCCAAGCCAGAAAGAAGTTAACTACAGTGTTTTCCTTTGCACCGATCCC

CACCCCAATTCAATCCCGGAAGGGACTTACTTAGGAAACCCTTCTTTACTAGATATCCTGGCCCCCTGGG

CTTGTGAACACCTCCTAGCCACATCACTACAGTACAGTGAGTGACCCCAGCCTCCTGCCTACCCCAAGAT

GCCCCTCCCCACCCTGACCGTGCTAACTGTGTGTACATATATATTCTACATATATGTATATTAAAACTGC

ACTGCCATGTCTGCCCTTTTTTGTGGTGTCTAGCATTAACTTATTGTCTAGGCCAGAGCGGGGGTGGGAG

GGGAATGCCACAGTGAAGGGAGTGGCAGAATCAAATTGCTACATAGTCCAAACAAAAAAGAAGGCTTTTT

CAAAAAACATTAAATTCACATGCAGTCTCAGAGACTATTTAGACAAAGTTCAAGTTAGGAGCTTTTAGGA

TGTGGGAGTAAAACTTTAATGGGAGGGGAGGGCTGGCTGCTGGAAGAAGGAAGAAGCCAGACTGGTTAGA

CAGTACTCTTAACTCCTAGCCCAGCCTAGCGTGCCCTGCCCCTCTGGCCACTGCTGCAGACACCTGCCTT

AACACACACACCTCTAGGACTCCACAGTTTTGCCTTAAAGGACCTTCCCAAGTCTCCCTTTCCCTGTCTG

GCTTCTCCCTTAAGAAGAGAGAGATACTTGTAGAATTGGGTGGGGGGAATGAGCATGAACTGTCCTTCCA

TTTGGGATATGTTACATTAGAGTGAGAGAGAGAATAAGGAGCCTTTCTTATGGAAGAAATGGGAGAAGAG

AGACAGGGTTCTTTTCAGCAGAGTCTAGTAGTTTCTCTGTAAGGCAAAATAATCTAAAAAGACTAACCTG

CCCACCCACTCCTTATATTGCTGTGAGATTGCCCCTATCTTGTGCTCTTCTGTCTGCAGTGTGCACGGCC

TTGTTCTAACCCGGAATAAAGGTGATTGATTGTATTGCAA

>NM_014389.3 Homo sapiens proline, glutamate and leucine rich protein 1 (PELP1), transcript variant 1, mRNA

ACGCCACCACTGGAACCCGGGGGAAGATGGCGGCAGCCGTTCTGAGTGGGCCCTCTGCGGGCTCCGCGGC

TGGGGTTCCTGGCGGGACCGGGGGTCTCTCGGCAGTGAGCTCGGGCCCGCGGCTCCGCCTGCTGCTGCTG

GAGAGTGTTTCTGGTTTGCTGCAACCTCGAACGGGGTCTGCCGTTGCTCCGGTGCATCCCCCAAACCGCT

CGGCCCCACATTTGCCCGGGCTCATGTGCCTATTGCGGCTGCATGGGTCGGTGGGCGGGGCCCAGAACCT

TTCAGCTCTTGGGGCATTGGTGAGTCTCAGTAATGCACGTCTCAGTTCCATCAAAACTCGGTTTGAGGGC

CTGTGTCTGCTGTCCCTGCTGGTAGGGGAGAGCCCCACAGAGCTATTCCAGCAGCACTGTGTGTCTTGGC

TTCGGAGCATTCAGCAGGTGTTACAGACCCAGGACCCGCCTGCCACAATGGAGCTGGCCGTGGCTGTCCT

GAGGGACCTCCTCCGATATGCAGCCCAGCTGCCTGCACTGTTCCGGGACATCTCCATGAACCACCTCCCT

GGCCTTCTCACCTCCCTGCTGGGCCTCAGGCCAGAGTGTGAGCAGTCAGCATTGGAAGGAATGAAGGCTT

GTATGACCTATTTCCCTCGGGCTTGTGGTTCTCTCAAAGGCAAGCTGGCCTCATTTTTTCTGTCTAGGGT

GGATGCCTTGAGCCCTCAGCTCCAACAGTTGGCCTGTGAGTGTTATTCCCGGCTGCCCTCTTTAGGGGCT

GGCTTTTCCCAAGGCCTGAAGCACACCGAGAGCTGGGAGCAGGAGCTACACAGTCTGCTGGCCTCACTGC

ACACCCTGCTGGGGGCCCTGTACGAGGGAGCAGAGACTGCTCCTGTGCAGAATGAAGGCCCTGGGGTGGA

GATGCTGCTGTCCTCAGAAGATGGTGATGCCCATGTCCTTCTCCAGCTTCGGCAGAGGTTTTCGGGACTG

GCCCGCTGCCTAGGGCTCATGCTCAGCTCTGAGTTTGGAGCTCCCGTGTCCGTCCCTGTGCAGGAAATCC

TGGATTTCATCTGCCGGACCCTCAGCGTCAGTAGCAAGAATATTAGCTTGCATGGAGATGGTCCCCTGCG

GCTGCTGCTGCTGCCCTCTATCCACCTTGAGGCCTTGGACCTGCTGTCTGCACTCATCCTCGCGTGTGGA

AGCCGGCTCTTGCGCTTTGGGATCCTGATCGGCCGCCTGCTTCCCCAGGTCCTCAATTCCTGGAGCATCG

GTAGAGATTCCCTCTCTCCAGGCCAGGAGAGGCCTTACAGCACGGTTCGGACCAAGGTGTATGCGATATT

AGAGCTGTGGGTGCAGGTTTGTGGGGCCTCGGCGGGAATGCTTCAGGGAGGAGCCTCTGGAGAGGCCCTG

CTCACCCACCTGCTCAGCGACATCTCCCCGCCAGCTGATGCCCTTAAGCTGCGTAGCCCGCGGGGGAGCC

CTGATGGGAGTTTGCAGACTGGGAAGCCTAGCGCCCCCAAGAAGCTAAAGCTGGATGTGGGGGAAGCTAT

GGCCCCGCCAAGCCACCGGAAAGGGGATAGCAATGCCAACAGCGACGTGTGTGCGGCTGCACTCAGAGGC

CTCAGCCGGACCATCCTCATGTGTGGGCCTCTCATCAAGGAGGAGACTCACAGGAGACTGCATGACCTGG

TCCTCCCCCTGGTCATGGGTGTACAGCAGGGTGAGGTCCTAGGCAGCTCCCCGTACACGAGCTCCCGCTG

CCGCCGTGAACTCTACTGCCTGCTGCTGGCGCTGCTGCTGGCCCCGTCTCCTCGCTGCCCACCTCCTCTT

GCCTGTGCCCTGCAAGCCTTCTCCCTCGGCCAGCGAGAAGATAGCCTTGAGGTCTCCTCTTTCTGCTCAG

AAGCACTGGTGACCTGTGCTGCTCTGACCCACCCCCGGGTTCCTCCCCTGCAGCCCATGGGCCCCACCTG

CCCCACACCTGCTCCAGTTCCCCCTCCTGAGGCCCCATCGCCCTTCAGGGCCCCACCGTTCCATCCTCCG

GGCCCCATGCCCTCAGTGGGCTCCATGCCCTCAGCAGGCCCCATGCCTTCAGCAGGCCCCATGCCCTCAG

CAGGCCCTGTGCCCTCGGCACGCCCTGGACCTCCCACCACAGCCAACCACCTAGGCCTTTCTGTCCCAGG

CCTAGTGTCTGTCCCTCCCCGGCTTCTTCCTGGCCCTGAGAACCACCGGGCAGGCTCAAATGAGGACCCC

ATCCTTGCCCCTAGTGGGACTCCCCCACCTACTATACCCCCAGATGAAACTTTTGGGGGGAGAGTGCCCA

GACCAGCCTTTGTCCACTATGACAAGGAGGAGGCATCTGATGTGGAGATCTCCTTGGAAAGTGACTCTGA

TGACAGCGTGGTGATCGTGCCCGAGGGGCTTCCCCCCCTGCCACCCCCACCACCCTCAGGTGCCACACCA

CCCCCTATAGCCCCCACTGGGCCACCAACAGCCTCCCCTCCTGTGCCAGCGAAGGAGGAGCCTGAAGAAC

TTCCTGCAGCCCCAGGGCCTCTCCCGCCACCCCCACCTCCGCCGCCGCCTGTTCCTGGTCCTGTGACGCT

CCCTCCACCCCAGTTGGTCCCTGAAGGGACTCCTGGTGGGGGAGGACCCCCAGCCCTGGAAGAGGATTTG

ACAGTTATTAATATCAACAGCAGTGATGAAGAGGAGGAGGAAGAGGAAGAAGAGGAAGAAGAAGAAGAGG

AAGAAGAGGAAGAGGAGGAAGACTTTGAGGAAGAGGAAGAGGATGAAGAGGAATATTTTGAAGAGGAAGA

AGAGGAGGAAGAAGAGTTTGAGGAAGAATTTGAGGAAGAAGAAGGTGAGTTAGAGGAAGAAGAAGAAGAG

GAGGATGAGGAGGAGGAAGAAGAACTGGAAGAGGTGGAAGACCTGGAGTTTGGCACAGCAGGAGGGGAGG

TAGAAGAAGGTGCACCTCCACCCCCAACCCTGCCTCCAGCTCTGCCTCCCCCTGAGTCTCCCCCAAAGGT

GCAGCCAGAACCCGAACCTGAACCCGGGCTGCTTTTGGAAGTGGAGGAGCCAGGGACGGAGGAGGAGCGT

GGGGCTGACACAGCTCCCACCCTGGCCCCTGAAGCGCTCCCCTCCCAGGGAGAGGTGGAGAGGGAAGGGG

AAAGCCCTGCGGCAGGGCCCCCTCCCCAGGAGCTTGTTGAAGAAGAGCCCTCTGCTCCCCCAACCCTGTT

GGAAGAGGAGACTGAGGATGGGAGTGACAAGGTGCAGCCCCCACCAGAGACACCTGCAGAAGAAGAGATG

GAGACAGAGACAGAGGCCGAAGCTCTCCAGGAAAAGGAGCAGGATGACACAGCTGCCATGCTGGCCGACT

TCATCGATTGTCCCCCTGATGATGAGAAGCCACCACCTCCCACAGAGCCTGACTCCTAGCCATCTTCTGC

ACCCCACTCTTTGTTTCCAATAAAGTTATGTCCTTAGATAGCGACTGCTGCTTCTGCCTTTTGCCACACC

TGGGTCCCCAGCCAGAAGCTCAAGTGTCTGCTGGGCTTTTCAGGGTGTCCATAGTATTCCCCAGATGTCT

CCATAGCTGTCCTCTTTTTGACGATCCAGAGAGGAACAACTGACATTGGTGTAATGGACTGTCCCATCAC

TCAGCACGATGGTGTCAGAATTGTAGGATTCTGGCTGGATGCTAAACGTGCAGAATTGTGTGTAGGGTTA

AGAATGAGGAGTTATAGAGACACAGCCCTGCTTTCGTGTTTGTATTCGGGGTGGGAAATGTGTGGAGAAT

GCATCCTAGTGAGTGGACCTGTGTCAACACCCACATGCCCACACGCATGCGTGCTCAGCCCCTTGGTCTG

AAAGGGCAGGCTGAGTGATTGCTCAGTCTATGGAAGTTTAGGAAATTAGGAAAAGGTGTGGAGCAGTTTA

GTCATTGAATTCTGGGAAATTTGGACCTTACCCCAAGATGAAAGGGTGTCCGTAGTGGGTACGTTGGGAA

TGACCTCTGTTTTGGGGCTAGTCCTCCTAATGACAGTTCCCCTCACAGCTCCCTGGCAGGAGTTTGATAA

GCTCACCCCAGGCCTTCCTGCTCACTCTTGAGGTGTGGATATTTCCCACCCAGTGTCTTTTCTTTTCTTT

TTTTTTTTGAGATGGACTCCTGCTCTGTCGCCCAGGCTGGAGTGCAGTGGCATGGTCTCAGCTCACTGCA

ACCTCTGCCTCCCAGGTTCAAGCAATTCTCTGCCTCAGCCTCCTGAGTAGCTGGGATTACAGGCGCCTGC

CACTGCCTGGCTAATTTTTGTATTTTTAGTAGAGGTGGGGTTTCACCATGTTGGCCAGGCTGGTCTTGAA

CTCCTGAGCTCATGGTCCACCTGCCTTGGACTCCCAAAGTGCTGGGATCACAAGCATGAGACACCGCACC

TGACTGTCCCAGTGTCTTTTCTTTAAGCACTAATCCTATTCTAGGCCTTGCTGGGATGGACGTGTGAGGA

TGAACCGAGAGATGTGTCTGTGAGAGGGTACCAAGATTGGTGGGCTTGAACTAGGGGCCCCGTGGTTTTT

TTTGCTCAGTCAGTTGAATCATGAACGTAAGTAAAGTGCCTTCGCAATGCAGAAGAGGGAGTCATTTCTC

CCAACATGTATCCTGAAGAGCCCTTTGAGGAGGTGGCATTTGAGCTAATTTTTGGAAACTTCAGTAGGAT

TTTAAAAAGTTACCCCTCAACTTGTTTTCGAAATAAACAGAAAAGTGAGAACAGTATAATAGATAACCTT

TGCCTAGATTTGCCAATTAACATTTAGTCATGTTAACTCTTATCCCTATAAACTGCGTATCTACATGTAG

ACATGTATCTATATTACACCCATATATCTGTCTAATCTATACATCTGTAGTTATACACATTTCTTTTTTC

ATGGGACCACGTGAAAGTAAGCTGTAGACACCGTGGCCCTTCCCATCCCAGATTCTTTAACATGAATTAT

CTTCTAAGATAAGAATGTTTTTCTGCACAATCCTGATACCATTATCAAACCTAAGAAAAATAACAGTAAT

CCAGTGATAATTGTGATATTAAAATTTTCCTATTAATTCCTAAA

>NM_004565.3 Homo sapiens peroxisomal biogenesis factor 14 (PEX14), mRNA

AGTCAGGCCTCAGAAAGATGGCGTCCTCGGAGCAGGCAGAGCAGCCGAGCCAGCCAAGCTCTACTCCAGG

AAGTGAAAATGTGCTGCCTCGAGAGCCGCTGATTGCCACGGCAGTGAAGTTTCTACAGAATTCCCGGGTC

CGCCAGAGCCCACTTGCAACCAGGAGAGCATTCCTAAAGAAGAAAGGGCTGACAGATGAAGAGATTGATA

TGGCCTTCCAGCAGTCGGGCACTGCTGCCGATGAGCCTTCGTCCTTGGGCCCAGCCACACAGGTGGTTCC

TGTCCAGCCCCCTCACCTCATATCTCAGCCATACAGTCCCGCAGGCTCCCGATGGCGAGATTACGGCGCC

CTGGCCATCATCATGGCAGGCATTGCATTTGGCTTTCACCAGCTCTACAAGAAATACCTGCTCCCCCTCA

TCCTGGGCGGCCGAGAGGACAGAAAGCAGCTGGAGAGGATGGAGGCCGGTCTCTCTGAGCTGAGTGGCAG

CGTGGCCCAGACAGTGACTCAGTTACAGACGACCCTCGCCTCCGTCCAGGAGCTGCTGATTCAGCAGCAG

CAGAAGATCCAGGAGCTTGCCCACGAGCTGGCCGCTGCCAAGGCCACCACATCCACCAACTGGATCCTGG

AGTCCCAGAATATCAACGAACTCAAGTCCGAAATTAACTCCTTGAAAGGGCTTCTTTTAAATCGGAGGCA

GTTCCCTCCATCCCCATCAGCCCCGAAGATCCCCTCCTGGCAGATCCCAGTCAAGTCACCGTCACCCTCC

AGCCCTGCGGCCGTGAACCACCACAGCAGCAGCGACATCTCACCTGTCAGCAACGAGTCCACGTCGTCCT

CGCCTGGGAAGGAGGGCCACAGCCCCGAGGGCTCCACGGTCACCTACCACTTGCTGGGCCCCCAGGAGGA

AGGCGAGGGGGTGGTGGACGTCAAGGGCCAGGTGCGGATGGAGGTGCAAGGCGAGGAGGAGAAGAGGGAG

GACAAGGAGGACGAGGAGGATGAGGAGGATGATGATGTGAGCCATGTGGACGAGGAGGACTGCCTGGGGG

TGCAGAGGGAGGACCGCCGGGGCGGGGATGGGCAGATCAACGAGCAGGTGGAGAAGCTGCGGCGGCCCGA

GGGCGCCAGCAACGAGAGTGAGCGGGACTAGGGCTGCGCCTGCTGCCTCCAGCCCTGAGGATGGCATCTA

GTGTGCCCGTGCGTGGCCATACCCTGCCTCCCTCTCTGGCCCTGGGAGGGCAGCTTGGAGCCCAGGTAGG

GGGCAGAGCTGTCCTCAGCTGCACTGCGGCCTGGTGGCAGTGTGGGGAGTCACACTTCTGTCCACCTGGC

CTCCTCTCGCCTGGCCGCCAGCCCCAGCCCCAGCCCCAGCCCCAGGCCCAGCTGCCTTTGGCTTTGATCT

CAAGTCAGGCTGAAGGCAGCGAAGCCTCGGGGCCCAAGCCCCTCCCCAGCCCCCTCTCCCGGACAGACGC

CTTGCCCAGGGTGTGTTTGCTGAGTGTCTTGACTACCGTGACACCACGCATGGCCAGAGCTAGCGTCCCT

ACTGCCTCCCGACTCCTCAGTGGAGGAGGAGCTGCGGTCCCTCTGGTGTCTGCCATCCCCCTCCCTCCCT

GGGCCCGGCCCTGGACCCGTCAGGTGCCTGTCCCCAGCCCCAACCCCACTCATGCCCCGTCGTCCTCCCA

GACAAATGAAACCACGCTGCGCTTCCGATGCCCCCGCTTGCCGTGTAATGGTTCAGCTAATCCCATGGCG

AGATGGGGGCTCACTCCGGAGGAGGAGCCAGGCAGCAGGGCCTTCCTGACCAACAGCCAGCTCTGTCCTT

CCCCCCAGGAAACACATGTTCATTTGTGTGATCATGTATAGACCTCAGAACGGAAGATAGGACTGTATAT

AATTGTAATAAATACCAGTTGCCACTATTTAA

>NM_003630.3 Homo sapiens peroxisomal biogenesis factor 3 (PEX3), mRNA

AGTGAGCGGCGGCGGCTGCGCGGCGGCAGCGGCAGAAAGCGTAGCTGCTTTGCTGTAGTCCACGCCCCCT

TGCCGCTCCGGTGACAGTCTCTGCGGAAAGTCACGTTTGTGATTTCGGGAGAGCACAGAACGGGACGACG

GCGCTCTTGCTGGGTCATCTGGGCCAGGTGACGAAGAAACAGTTTCCTGGTGAAGCAGTCCCTCACCCCT

AGTCAGCCCACACCCCTAGGGCCTAAAGATGCTGAGGTCTGTATGGAATTTTCTGAAACGCCACAAAAAG

AAATGCATCTTCCTGGGCACGGTCCTTGGAGGAGTATATATTCTGGGGAAATATGGACAGAAGAAAATCA

GAGAAATACAGGAAAGGGAGGCTGCAGAATACATTGCCCAAGCACGACGACAATATCATTTTGAAAGTAA

CCAGAGGACTTGCAATATGACAGTGCTGTCCATGCTTCCAACACTGAGAGAGGCCTTAATGCAGCAACTG

AATTCCGAGAGCCTCACAGCTCTGCTAAAAAACAGGCCTTCAAACAAGCTAGAAATATGGGAGGATCTGA

AGATAATAAGTTTCACAAGAAGTACTGTGGCTGTATACAGTACCTGTATGCTGGTTGTTCTTTTGCGGGT

CCAGTTAAACATAATTGGTGGATATATTTACCTGGATAATGCAGCAGTTGGCAAAAATGGCACTACAATT

CTTGCTCCCCCAGATGTCCAACAGCAGTATTTATCAAGTATTCAGCACCTACTTGGAGATGGCCTGACAG

AATTGATCACTGTCATTAAACAAGCTGTGCAGAAGGTTTTAGGAAGTGTTTCTCTTAAACATTCTTTGTC

CCTTTTGGACTTGGAGCAAAAACTAAAAGAAATCAGAAATCTCGTTGAGCAGCATAAGTCTTCTTCTTGG

ATTAATAAAGATGGATCCAAACCTTTATTATGCCATTATATGATGCCAGATGAAGAAACTCCATTAGCAG

TGCAGGCCTGTGGACTTTCTCCTCGAGACATTACCACTATTAAACTTCTCAATGAAACTAGAGACATGTT

GGAAAGCCCAGATTTTAGTACAGTTTTGAATACCTGTTTAAACCGAGGTTTTAGTAGACTTCTAGACAAT

ATGGCTGAGTTCTTTCGACCTACTGAACAGGACCTGCAACATGGTAACTCTATGAATAGTCTTTCCAGTG

TCAGCCTGCCTTTAGCTAAGATAATTCCAATAGTAAACGGACAGATCCATTCAGTTTGCAGTGAAACACC

TAGTCATTTTGTTCAGGATCTGTTGACAATGGAGCAAGTGAAAGACTTTGCTGCTAATGTGTATGAAGCT

TTTAGTACCCCTCAGCAACTGGAGAAATGATTTTTCCTTCAAGAAAAACTACAGTGGGATTCATTTACTT

TTTAAAATACACTGGGTAAATCACCTATACTTAGAGTAACAGTTTGTTATCAAAATGCCTGATAAAATAT

ATTCTTAATAAAAGTCTTCATTTCATAATGAAATCAATTTATTTGGCATCTTAATATATTTTTTTAGATT

CATCAACAGACCAGTTTTTGTGGGCATATATATATACACGTGCAAATATCAGAATTGTTAATAATTTGTT

ACACATGGACATTTGTTCCAAACTGACTAAAAATCAATATAGATATTTTATATACATATATATATATAAA

AATACAAAATTCAGTGTACTTTACCATATTAATACTGAGGAAAAATCTGTTGGAGACATAGGTCTAGGAT

GTGTGAAGTTTGGAAAAATATGCTATTTAATTATAATGTTCCCTAGACTGCTGTAAACAGAAGTGAATCA

GACTTTTCTCCAGCTACCTTTCAAAATAATAAATTATTTGTCTCAAATATACCTTGATGGAGGACTTTTT

TATTCTTATGGAAATAGTGAATTCCAACAACTATGATGAACTATGTTCTTTGCTATTTCTTCACTATATT

TTTTAAGGTTTTATTAAAAAGCCTTAGAAAGTTACATATTGGTTTAGAGGCTAAAATTGTGTTGATGCTG

TTTACTCACCTAATTACATAGTTTTAATCATTTGTACATAATTTTAAAAACTTACTTTGTATTGATTTTG

AATACAGTGAAAATCTTATTGCAATAAACTATTTTAGTAAAATATTATTTTGTTGAGTTAATATTTTTCA

GCTATTAATTTACTTATCCCATAGCATTTTTGTTATTATTTCGGAAGAGAGAGCATGCTAGTTTAAGTTT

CATTTTAATTGGTCGTGTACTTGGTGCTTTTTTGTTGTTGTTAACTTGTTCATTTAGTAAAAAGCAAATA

GCTTTACTTTCTGAAAAAGAAATAATTCATTATCTCTAGTAATACATCTGTAATTCTTCATTTAGAAGGG

TAATTAGGGCAAAAGTAAATAAATCATTATATATCATTAGTATGTTGACATATATTTTCAGTAACATTGT

TTTATGTTCCTCCAGCGTAACATGTAGCTGAACAGTAGAAGGTAGAAAATAAATTATGACATCTCTTCTA

AGGCCTGGCTAGGCCTGAAGCTCACTGCCAGGGAGTTTGTGTCTAGAATGTTTTTAAAAATAGGTAGTGG

AGCCATAGCCCTCACCATAGACACCTCTGAGCCCACTGGCATTGTCTCTGAGCAGAAGATGGCTGTGCCA

CTCACATATGCTAATCTTGGCAAATCTGCCAAGCATGTCTTCACCAAGGGATATGGATTTGGCTTAATAA

AACCTGATTTGAAAACAAAA

>NM_002647.4 Homo sapiens phosphatidylinositol 3-kinase catalytic subunit type 3 (PIK3C3), transcript variant 1, mRNA

ATGTTGTTTTTCCTGTACCTAAGTTCCCGCTGTAGGTGGTACCTTTGCAGACGGTGCGATGGGGGAAGCA

GAGAAGTTTCACTACATCTATAGTTGTGACCTGGATATCAACGTCCAGCTTAAGATAGGAAGCTTGGAAG

GGAAGAGAGAACAAAAGAGTTATAAAGCTGTCCTGGAAGACCCAATGTTGAAGTTCTCAGGACTATATCA

AGAGACATGCTCTGATCTTTATGTTACTTGTCAAGTTTTTGCAGAAGGGAAGCCTTTGGCCTTGCCAGTG

AGAACATCCTACAAAGCATTTAGTACAAGATGGAACTGGAATGAATGGCTGAAACTACCAGTAAAATACC

CTGACCTGCCCAGGAATGCCCAAGTGGCCCTCACCATATGGGATGTGTATGGTCCCGGAAAAGCAGTGCC

TGTAGGAGGAACAACGGTTTCGCTCTTTGGAAAATACGGCATGTTTCGCCAAGGGATGCATGACTTGAAA

GTCTGGCCTAATGTAGAAGCAGATGGATCAGAACCCACAAAAACTCCTGGCAGAACAAGTAGCACTCTCT

CAGAAGATCAGATGAGCCGTCTTGCCAAGCTCACCAAAGCTCATCGACAAGGACACATGGTGAAAGTAGA

TTGGCTGGATAGATTGACATTTAGAGAAATAGAAATGATAAATGAGAGTGAAAAACGAAGTTCTAATTTC

ATGTACCTGATGGTTGAATTTCGATGTGTCAAGTGTGATGATAAGGAATATGGTATTGTTTATTATGAAA

AGGACGGTGATGAATCATCTCCAATTTTAACAAGTTTTGAATTAGTGAAAGTTCCTGACCCCCAGATGTC

TATGGAGAATTTAGTTGAGAGCAAACACCACAAGCTTGCCCGGAGTTTAAGAAGTGGACCTTCTGACCAC

GATCTGAAACCCAATGCTGCCACGAGAGATCAGTTAAATATTATTGTGAGTTATCCACCAACCAAGCAAC

TTACATATGAAGAACAAGATCTTGTTTGGAAGTTTAGATATTATCTTACGAATCAAGAAAAAGCCTTGAC

AAAATTCTTGAAATGTGTTAATTGGGATCTACCTCAAGAGGCCAAACAGGCCTTGGAACTTCTGGGAAAA

TGGAAGCCGATGGATGTAGAGGACTCCTTGGAGCTGTTATCCTCTCATTACACCAACCCAACTGTGAGGC

GTTATGCTGTTGCCCGGTTGCGACAGGCCGATGATGAGGATTTGTTGATGTACCTATTACAATTGGTCCA

GGCTCTCAAATATGAAAATTTTGATGATATAAAGAATGGATTGGAACCTACCAAGAAGGATAGTCAGAGT

TCAGTGTCAGAAAATGTGTCAAATTCTGGAATAAATTCTGCAGAAATAGATAGCTCCCAAATTATAACCA

GCCCCCTTCCTTCAGTCTCTTCACCTCCTCCTGCATCAAAAACAAAAGAAGTTCCAGATGGCGAAAATCT

GGAACAAGATCTCTGTACCTTCTTGATATCGAGAGCCTGCAAAAACTCAACACTGGCTAATTATTTATAC

TGGTATGTGATAGTGGAATGTGAAGATCAAGATACTCAGCAGAGAGATCCAAAGACCCATGAGATGTACT

TGAACGTAATGAGAAGATTCAGCCAAGCATTGTTGAAGGGTGATAAGTCTGTCAGAGTTATGCGTTCTTT

GCTGGCTGCACAACAGACATTTGTAGATCGGTTGGTGCATCTAATGAAGGCAGTACAACGCGAAAGTGGA

AATCGTAAGAAAAAGAATGAGAGACTACAGGCATTGCTTGGAGATAATGAAAAGATGAATTTGTCAGATG

TGGAACTTATCCCGTTGCCTTTAGAACCCCAAGTGAAAATTAGAGGAATAATTCCGGAAACAGCTACACT
GTTTAAAAGTGCCCTTATGCCTGCACAGTTGTTTTTTAAGACGGAAGATGGAGGCAAATATCCAGTTATA
TTTAAGCATGGAGATGATTTACGTCAAGATCAACTTATTCTTCAAATCATTTCACTCATGGACAAGCTGT
TACGGAAAGAAAATCTGGACTTGAAATTGACACCTTATAAGGTGTTAGCCACCAGTACAAAACATGGCTT
CATGCAGTTTATCCAGTCAGTTCCTGTGGCTGAAGTTCTTGATACAGAGGGAAGCATTCAGAACTTTTTT
AGAAAATATGCACCAAGTGAGAATGGGCCAAATGGGATTAGTGCTGAGGTCATGGACACTTACGTTAAAA
GCTGTGCTGGATATTGCGTGATCACCTATATACTTGGAGTTGGAGACAGGCACCTGGATAACCTTTTGCT
AACAAAAACAGGCAAACTCTTCCACATAGACTTTGGATATATTTTGGGTCGGGATCCAAAGCCTCTTCCT
CCACCAATGAAGCTGAATAAAGAAATGGTAGAAGGAATGGGGGGCACACAGAGTGAGCAGTACCAAGAGT
TCCGTAAACAGTGTTACACGGCTTTCCTCCACCTGCGAAGGTATTCTAATCTGATTTTGAACTTGTTTTC
CTTGATGGTTGATGCAAACATTCCAGATATTGCACTTGAACCAGATAAAACTGTGAAAAAGGTTCAGGAT
AAATTCCGCTTAGACCTGTCGGATGAAGAGGCTGTGCATTACATGCAGAGTCTGATTGATGAGAGTGTCC
ATGCTCTTTTTGCTGCAGTGGTGGAACAGATTCACAAGTTTGCCCAGTACTGGAGAAAATGAAACTGGGA
TTGACCCATCAAGATGCTTGGCTCAATAAGAAAACCACGTTAGGAGCAACCTTTGTATATTGGAGACTTC
AGAGTAACCAGCAAGGAAGAGAAATCTTAATCTTCAAGTTACCATATTTTCCAAATATTACATGGTACCT
GAGTTCTGCTTCCTTGGATGTCATTGCTTAAATATAGTCTTGAAGGGCTTGTTTTGAAATATTGTATATA
TTTTTTCAAATGTATACATTGTTAATAAATTAAGAAATGAGAAACATTCTTATTTATGTACATGTTATGA
GAGTTCTGGAGGAAGTAATATGTTGAAATAGTAAAACATTTTAGTTTTTGAGTTTAAAATGTTATTTTTT
CCTCTTATATCTTCATATCAGGACAGCAGTAACCTGAATTTAGATTGTCACAGTTTACAAGTTTTTGTTT
GTTCCTTCTTTCCTCTCTCTTCTCCCACTCACTTTTGTGTGTTGTTTGTTTTTGCCTAATTTACATGTTT
AAAGTTCTACTTAAAATTTTTCTTCAAGTTTTACATGTGATGGCTCATTCTTCTGCAGTTCAAGAATTGC
TTATATTTTTAGAAGATACTCATTTTCTCTTACAAATAATTAATTTTTTGTAAACTACAGTTAATAGTTC
TTCTAGCTTGTAAGTGTGTAAAGTAGAATATGAAAATGTGGCTATTTAGATAACTGGCCAAAAGGTCATA
TGACCACCAGTTTTAGTATTGAGCATAAGATATTTTTACATATTCATGTAAGACAATGTAAATTGTCTTT
CTGCAATAAATTTTTTGGATGCAAATTTAGATTTCAAAAGCTTTTTGATGTTATAAAGGAGTTGAGGAAC

AAAAAGCTCTTTGAAATAGCTCACCTAGACCTGTTAATGTAATATATTCTTTCTGAATTAAGCAAAATTT

TTTTTGTCTTTTAAGACTAGAACTAATCCCTCTAGCTTTTTTACTGGAACTTTGGGGAAAAAGAACAAAT

ACAAATATACAAATGATATGAACAATATGGTAATAATCTTTTGCAACTTAATATGTTACATAGTCATATG

ACTTGGTCTTGGAAGAAAAAGAATTGTTTTGGTGCTGAATAAATATTAGAAATCAAGTGACAGAGAAGAT

GAAGGTAAAATTGTGGCACAGTTTCAGAGAATGCCGTTAATGATAACGTGTTATTTGTCTCAAATTCTTA

GCCCTGGGCTTGCCTCTTCAGAGTGGGGTTACCGGCGAGAGGGCAAGCATGTAATTAAGAGACACTCTAG

AGGCATATGGAATATAGTTTGTATAAAACATACCTGCTGATTAGAAAAATGGTAAAAATGAAAATACCTT

TGTACATCATCTTAAGAGAGCTTATATATAAACTAAATGGGCAGGCTCCATCGTCTACCTTTCTTTTTCT

TTTCTTCACATGTTTATGTCTTTTTTGTTATTAGTCAGACATTTTTAGCCCTCTGTATGCTCATTCTTGT

AGTTCTATTTCTATTGAAACTTCCTCCAAAACTTTTCTCTGAATTACCCTTGTTGAAACTGACCTTTCTT

CCGTGTGAACTCCTACACTTGCATCTCCTATTTCAGGATTTGGCATACTTTTTCTTTAATGGGCTAAGAA

AGTAAATATCTTCAGCTTTGGGGGGCATATGATCCCAATAGCATCAACTTAACTCTGCTAACTGGAAGGC

AAAAGTGTATCATCTTACTCGTCTTTATAAACTCCACAGCACTCTGCATAGATATGACCATGTTTATAAG

GAACTCAATAAATAATTTTGACACAAACATGAAATTCTAGAAGGGCAGACACTGACAACTAGTAAGGAAG

TTGATCATGAGTAACTTTGTATTCAGTGCCACCTTAACAAGAATTGTAAAGAGGATAAACCTTTCTAGGA

AGGTTGATGCGTGTTGGGTGAGGAAGGAGCATCTCTCAGGAGGGTAAACAGTAGCTGTGGTTTTGCTGAG

AGATTTTGGCTGTGAGTGACAAGCCTATATCAAAAAGAAAAGTGGCTTATCAATCTCAGTTTTTATTGCT

CAAAGCAATTCTTTACTCCTATTTTTTCCAAATTCCATTGTTTTTACTCTTTAGAGCTCTATCCTCAGAG

TGTAGTCTGCATATTTCTTTATTATCATTGCCATGTGACTTCTTTGGTGACAAGTTCAGTGATATTTCAA

GCATGATGTTTTGAGTTTGAGAAATGTATTTGTGAGGACACATCACTTTAATTAAAAAAGTGTGAGTACT

ATGGTCATTAAGTTATTCAGAGTGAGATTTCTGAGAGGAGAAGAGAGAATGTAGATGGCAGTTATTGAGA

GTGGGGCTATAGGACAAAAAGAAGAAAAGTATGGTTAACATTGCCAAATAAAGGGTCATACAGCCTTGCT

TTTTGCTGGAGTTAGCAAAAATCATTCTCCATATGATAGTTTTAGATGTTCCCCAGCTCATTTGTATTAT

TGAGTTTTCAGCTGGGTATATATTATAATAACAGTCAACATGATGTAGGGGCTCAGTATTACATGGCAGG

GCTATACTAATTGCTTCATATGCCTTATCTCATTATTCTGTGAGATAGAATTGTCTCCTCATTTTTTAGT

TGAAATAACTGAAGTTTAAAGCAATTGAATCATCTGCCCAAGGATAAGCTGCTGGTGAGAGCAGAGTTGG

GATTTGAAGTCGAGTTAGACCCCAGTGATCACAGTCTTGACGATTAAATTCTTCCAGCTTTCATTTTTCA

CTGAGATAATGGTAGTGATAGTACTGACCTCTAATGTGTGCATTTGTGGGTATGTGGTCCATTCAGCTTT

AATCCCCAGAAGACAAGGCTTATTCCTTTTCTTATTTTTGGTCATGTTTTATTTTTCCATTGCTTTTAAC

AGGATTACCAAAGGCACACTCAGTAGTCAGTAAACACATTTCTAGGAAAGGTGTTGTGTCATCATGCCAC

ATATTCATACTTTCCTTGGGTTGGAAAATAGATCATTCAGTAAAAACATACAGGAAAAATGAATCTTGCC

AATGCAATTGTTAACCTACAACCATAATATACCTTAAGTATATTTTTGCACATAAGTATAACATTGCGAT

TTAAAACAATAAACCAGATTGAGATTCTAAGGAGCATTTTGTAAGTAATTACTAATGTTTATTTTAGAGA

GATCACACAACTTCAAATAAAAACTGACATAGATTGAACACCTTGAGAATAAACTTTAGTGCCAAATGGA

AAATAATTTTTTACAAGTAAATTTGAAGAACAATGTGAACTTTCTATAATTATATACAGAAAATATACTG

ATTTGCCAAAATGAGTAATTTTGATATATTAATATTTCACTTATAAGAATGCATACCACCTGATCCAGGA

TGGGATCCAGGAACAGAAAAAGAACATTAGGTAAAAATGACAGAAATCTGAATATAGTATAGAGTAGCTA

AAAACAAACCAAAAAAAAAAAAAAAAAAAAAGGAAAAACAGAATAAGCCTCAGAAGATAGTGAGCATCCC

ACCTGGGTGACCACTTAGAGAAGCTGAGGTGGGACCCCTGCATCCGATAAGTGGCTGCACTAGACCACAG

GAACCAGACTCCACTGTAAGAACTAGAGTGGGAATGGACATGTCAAAACCTCCTGGGAACCTTATTAAAT

GTGCACATTCCCAATCCCCACTCCTGAGCAGCCAAATCAATCTGCAGGTGGGCCTTGTGAGTTTTCCCAG

GGGATTCCGATGCCTGCTAGATTTAACCAGTAGATGAGATGTTAAGTTATCCTGTAGTCCAGAGATTCCA

CAGGCCAGTGTCAGGGAACAGGGGCCAGGGCTGGGAAGGCATAAGAACAGAAGATAAATTTGAGGCTCTG

TTACGCTGGTCATCCAACCCAACCAGGTATCTCTTGTTATATGCTAACTTCATTTTCCCTTTCTTGTCCA

TAGTGGGAAAAAATAATTTTTAACTGGCTATGTAGTTAAAAAACCTAACAAAAAGCACTTTTCTATATAA

TGAGTGGTTTATATTAAGAATAGGTACAATGTAAATACTGGTAAGTGAAAGTTCATTTTGTTCAGTGCTA

TGGGCTGGTACATTGCATTTCAACACTAGTTTTTTACTTTTATCTACCCTGAGGGTAGATAACCCATATT

CCAAAATACAGTATTATTTTCATTTGCTGTAACATTTTAAGAAATATGTAGTTTCTAAAACTTCATATAC

TTTATATGGAATATGAACAACTTTAATATGAATAAATAGTCTAGAAAAAAATTTAGGAAAAATTGCCATG

AAATCATGAGCTAAGTCAATGGTTATTTTAAACCACTTAATTTTGAGACTAGTATTTCATACAGAAAAAG

CTAATACTGATATCACACACTAATTGTGTATAAAACACTAGGAATGTCAAAATAGAACATAAGAAAATGA

GTCTAGTGGACCCACTGCATAGTGTAACTTTGGGATGAATTTAAAAGAATATTTAAGTCACAGTCAATTT

TCTGTTGGTCTAATTTTACCAAATAACCCCCTAAATAAGAAGTCTGATTTATCTAAAATAGAAGTTAACA

TAGCATCCCAGTATTGCCATTCTAAAACTCTGGCATGTAAATGGGAGAAATGGAAATAAGCTCAAAATGG

GTAGAGTAACCCATTTCTGCATGAACCATAGAATTTAACTAGAATTTGGCTGGCTCATGCCTGTAATGCC

AGCACTTTGGGAGGCCAAGGCAGGCAGATCACTTGAGCCCAGGAGTTTGAGACCAGCCCGGGCAACATGG

TGAAACTCCATCTCTTCAAAAATATATATTTGTGTGTGTGTGTGTGTGTATGTGCGTGCACAAAAATTAG

CTGAGTGTGGTGGTTCACACTTGTAATCACAGCTACCTGGGAGGCTGAGGCAGGAGGATTGCTTGAGCCT

GGGAGGCAGAGGTTGCAGTGAGCTGAGATTGCACCATTGCACTGCAGCCTGGGTAATAGAGCGAGACAAC

TCTATCTCAAAAAAAAAGAATTTCACTAGAATTTATATCTTCAGTGTTTGCATTAGATTGTCACCAGCACT

ATCTGCCTTGTGACTGTCAGGACTAATCCTTATATCATTTATCTTATGTCCTTTACCTCAGTTTATGTCT

GTCACCACAAGAAACCCAGACCTCCCTAATCATACCATATGCCCTGGTCTTACCCACATACAGCTCGGTA

CAACTGCATTTCCCCAAACCTCAGAACTCCTTGGGCCATCACTGTCAGAGACATTTGAACTAGAGCAACT

CCATCTTGAGTAGGGACTGGGTATAATGAAGCTGAGACCCACTGGGCTGCATTCCCAGGAAGTTAGGCAT

TCTTAGTCACAGGATGAGATAGGAGATTGGCTCAAGATAAGGTCACAAAGACCTTGCTGATAAAACAGTC

TGTGGTAAAGAAGCCAGCCAAAACCCACCAAAACCAAGACGGGAATGAAAGTGACCTCCGGTCATCCTCA

CTGCTCATTATACGCTAGTTATAATGCATTGACATGCTAAGAGACACTCCCACCAGCACCATGACAGTTT

ACAAATGCCATAACAACATCAGGAAGTTACCCTATATGGTCTAAAAAGGGGAGGAACCCTCAGTTCTGAG

AATTTCCTTGAAAATTCATGAATAATCCACCCCTTGTTTAGCATATGATCAAGAAATAATCATTAAAAAA

TAGCCAACCAGCAGCTCATGCTGCTGGTCTGCCTATGGAGTAGCCATCCTTTCTTTATTAACCTGCTTTC

ACTTTATGGACTCCCCTGAATTCTTCTGCAGGAGATCCAAGAACCCTCTCTTGGAGTCTTAACCAGGATC

CCTTTCCAGTAACTTCAGCAGGATCTGTGAGTGCCAGTTTCCTTCTGTGGTGCAAAACTAGGTGCTAAGT

ACACTTTTTCCCCCCTTTCCTCTATTTTTAAAAGAACTTTCAGCCCTTTTGAAGTGGGTACATTGTCTGG

AGTACATACCCTGGGGTTCATTGTTATAGGAGAATTTAGGACACAGACACACGAGGAGTTTAGGAGCAGA

GGTTTAATAGGTAGAAGAGAAGGGAAAGAGAAACAGCTTCCTCTATAGATGTCTGCACTGACACCGTGGC

TTTAAGGCATGGCCCATGATGTGTCTCCGAGTGGAAAAGACTGGTTGGTGGTGAATGCGCCAAATTTTAT
AGTCCGGTTTGAGGAGGTGGTTTCTGATTGGTTGCATCATGTATGACGTTTGCAAGGTGCATGTGGAAGG
CTGGTCGCCCCACCCTAATCTTATGCAAATGGGCTTTCCAGTTGATTGGGTCCATCTTGTGTAACACGTG
GCTGGCAGAGAAGGGAAGATGAAGCCACCATCTTGAAAATGTCTAGTCCTTAGTTTCTGCTGGCATTCAC
CCATGCAAGCTGCCAGCTTGTCTATGTCTGCAGCTCAACTTTACAGGCTGCTCTTTGTTAGCAAATGATT
TGGAGCTTCTTTTCATTAAAGAAAAAAGCCTTACCGAGGACTCCCATACCCTTGCTTTCTGCTTAAGTGA
TTTCTTCTTAACTCCTATGTCAATTTTATACAGCATGAAATAATGATGTATCTATAAATGAGCCTTTGCA
AGATTACCATTTATCAAAGAAAACCAGTGATGTTTAGTTAACTGTTTGTAAAAATAAATGGTTAGTAGAA
TGTCTAATTCAATAAATGTTTCATAAATGAGTAAA

>NM_014602.3 Homo sapiens phosphoinositide-3-kinase regulatory subunit 4 (PIK3R4), mRNA
GAGGTCAGACCGGTTGCTTTCCCGGGAGTTCGGCGTTTGCTGGGGCTGCAGCAGCTGAAGTGTAGTGTTT
TCTTGGGACTGGCGGTCTGCACTTCTCTCCCGGGTTCCATCTCCCCCCGCCCGGTGGTGAGGCCCTCGAG
GAGGGCTCGGACGGGTGTAGCGATCCGCGCTAGAGGAAGACGAGGCCCGGGAACGCATGTCCCCCAGGGC
AGGTTAGGGGGCTGGAGGGGTCAAATCCCGGGGTACTTGTGGAGACTCTTTAGCGTGGCTTCTTCTCTCT
GCTGAGACCCCGAGAGCTTTCCCAGTTCTCCTCCCAGGACCACCGGGGTTCCTGAAGATCGGGACTTTTC
TGCGCCCCTCCACCAACAGCCCATCTCCTGTCTATGAAGAAAGACCCTTCGTAGAAACAACTTCCCCGCT
GCTGACGCGTTTTCCCGTCCCGTCCCCGAAGTAGTCTACTATGACCTCGTTGTGAGCCTCTGAACGATTT
TGACACTTTCCCGAGGCCTAGGGTATTATATCCTAACCTTACTAAAGACCACAGAGGTGCTTGCCATTAT
GGGAAATCAGCTTGCTGGCATTGCTCCCTCCCAGATCCTTTCTGTAGAGAGTTATTTTTCAGATATTCAT
GACTTTGAATATGATAAAAGCCTGGGGAGTACTCGGTTTTTTAAAGTTGCTCGAGCCAAGCACCGAGAAG
GCCTGGTCGTTGTGAAGGTTTTTGCAATTCAGGATCCCACATTGCCTTTAACCAGCTATAAACAAGAGCT
GGAGGAACTGAAAATCAGGCTTAATTCTGCACAGAATTGTCTACCTTTCCAGAAAGCATCAGAAAAAGCA

TCTGAGAAAGCAGCTATGCTCTTTAGGCAGTATGTGCGAGACAATCTCTATGATCGCATCAGTACCCGTC

CATTCTTGAATAACATTGAGAAGCGCTGGATTGCTTTCCAGATCCTGACAGCTGTGGACCAAGCACACAA

ATCTGGAGTTCGTCATGGGGACATCAAGACTGAGAATGTGATGGTCACCAGTTGGAATTGGGTTCTTCTA

ACTGATTTTGCCAGTTTTAAGCCCACTTATCTTCCAGAAGACAACCCGGCAGATTTCAATTATTTCTTTG

ACACATCACGGAGGAGAACTTGCTATATTGCTCCTGAACGTTTTGTTGATGGTGGGATGTTTGCCACTGA

GTTAGAATATATGAGAGATCCTTCAACTCCGCTTGTAGACTTAAATAGCAATCAGAGAACAAGAGGAGAG

TTGAAGAGAGCAATGGACATCTTTTCAGCAGGTTGTGTGATAGCTGAGCTTTTTACAGAAGGTGTACCAT

TATTTGATCTCTCTCAACTTTTGGCTTATAGAAATGGACATTTTTTCCCTGAACAAGTGCTAAATAAAAT

TGAAGATCACAGTATCAGAGAATTGGTAACTCAGATGATTCACCGTGAGCCAGATAAACGTTTAGAGGCA

GAAGATTACTTAAAACAGCAGCGTGGCAATGCCTTTCCTGAAATATTTTACACTTTTCTTCAGCCCTACA

TGGCCCAGTTTGCCAAGGAAACGTTTCTTTCTGCAGATGAGCGTATTCTGGTTATACGGAAGGATTTGGG

CAACATTATTCACAATCTCTGTGGACATGATCTGCCAGAAAAAGCCGAAGGAGAGCCTAAGGAAAATGGG

CTGGTTATCTTGGTATCTGTTATAACATCCTGCCTACAGACCCTTAAATACTGTGATTCCAAACTAGCTG

CTTTGGAACTGATTCTTCATTTGGCTCCAAGATTAAGTGTTGAAATCCTTTTGGATCGTATTACTCCATA

TCTTTTGCATTTCAGCAATGACTCTGTTCCTAGGGTGAGGGCTGAAGCCTTGAGGACGTTGACCAAAGTT

CTTGCTCTCGTCAAAGAGGTTCCTCGTAATGATATCAATATTTATCCGGAATACATTCTGCCAGGCATAG

CCCACTTAGCCCAAGATGATGCTACTATCGTTAGACTAGCCTATGCTGAAAACATAGCTCTGCTGGCAGA

AACAGCTCTGAGATTCCTGGAATTAGTACAGTTAAAAAATCTTAATATGGAAAATGACCCCAATAATGAA

GAAATAGATGAGGTTACACATCCAAATGGAAATTATGACACAGAGCTCCAAGCCTTACATGAAATGGTCC

AGCAGAAAGTTGTTACTTTGCTAAGTGACCCTGAAAATATTGTAAAACAAACCTTGATGGAAAATGGAAT

AACACGGCTGTGTGTATTCTTTGGACGTCAGAAAGCCAACGATGTTTTGTTGTCCCACATGATTACTTTC

CTAAATGATAAGAATGATTGGCATCTACGTGGAGCATTTTTTGATAGTATAGTTGGTGTTGCTGCCTATG

TTGGCTGGCAAAGCTCCTCAATTCTCAAGCCTCTGCTGCAACAAGGTCTTAGTGATGCTGAGGAATTTGT

CATTGTGAAAGCTCTTTATGCCCTTACTTGTATGTGCCAGTTAGGACTGCTACAAAAACCCCATGTTTAC

GAATTTGCCAGTGATATTGCCCCCTTCCTGTGTCATCCCAATTTATGGATACGTTATGGTGCCGTGGGAT

TTATCACAGTGGTAGCTCGTCAAATAAGTACAGCTGATGTCTACTGTAAACTGATGCCTTATCTTGACCC

ATATATTACCCAACCAATAATACAGATTGAAAGAAAACTTGTTCTGCTCAGTGTTTTAAAGGAACCAGTA

AGTCGTTCTATATTTGATTATGCTTTGAGGTCTAAAGATATTACTAGCTTGTTCAGACATCTTCACATGC

GTCAGAAGAAACGAAATGGTTCTCTTCCCGACTGCCCTCCGCCAGAGGATCCTGCCATAGCACAGCTTCT

GAAGAAGTTGCTCTCACAGGGAATGACAGAGGAAGAGGAAGACAAACTTCTGGCACTGAAAGACTTCATG

ATGAAATCTAATAAAGCAAAGGCCAATATAGTGGACCAGAGCCATCTTCATGATAGTAGTCAGAAAGGTG

TAATTGACTTGGCAGCTTTAGGCATAACTGGGAGACAAGTTGATCTTGTTAAAACCAAACAAGAACCAGA

TGACAAACGGGCCAGAAAACATGTAAAACAAGACTCAAATGTAAATGAAGAATGGAAAAGCATGTTTGGG

TCACTGGACCCACCAAACATGCCACAGGCCCTACCTAAAGGGAGTGATCAGGAGGTGATTCAGACTGGGA

AACCTCCTCGTTCCGAGTCCTCTGCTGGCATTTGTGTCCCTTTGTCAACTTCTTCACAGGTTCCAGAAGT

GACAACTGTCCAAAATAAAAAACCAGTAATACCGGTTTTAAGTAGTACAATCTTACCATCCACCTATCAG

ATTCGAATTACAACTTGTAAAACTGAACTTCAGCAACTCATCCAGCAAAAGCGGGAGCAGTGCAATGCTG

AGAGAATAGCTAAGCAGATGATGGAAAATGCTGAATGGGAGAGTAAACCACCACCACCTGGATGGCGTCC

TAAAGGGCTGTTAGTTGCCCATCTTCATGAGCATAAATCTGCTGTGAATCGAATTAGAGTCTCTGATGAA

CACTCACTTTTTGCAACATGTTCAAATGATGGCACAGTGAAAATCTGGAACAGTCAAAAGATGGAGGGGA

AGACCACCACTACCAGATCTATTCTTACATACAGCCGAATTGGAGGACGAGTCAAGACGCTCACATTCTG

CCAAGGCTCCCACTATTTAGCCATAGCATCTGATAATGGTGCTGTCCAGCTTCTTGGAATTGAGGCTTCT

AAGCTGCCCAAGTCTCCTAAAATCCATCCTCTACAAAGCAGAATTCTAGATCAGAAGGAGGACGGTTGTG

TTGTGGATATGCATCACTTCAACTCTGGAGCACAGTCTGTTCTTGCCTATGCCACTGTGAATGGCTCTCT

GGTTGGCTGGGACCTTAGGTCTTCAAGCAATGCGTGGACTTTAAAGCATGATTTAAAGTCGGGCCTCATC

ACTTCCTTTGCTGTGGACATCCACCAATGCTGGCTCTGCATTGGTACAAGCAGTGGTACCATGGCTTGTT

GGGACATGAGGTTCCAGTTGCCAATTTCAAGTCACTGTCATCCTTCCAGGGCTCGAATCAGACGCCTCTC

AATGCACCCTCTGTATCAGTCCTGGGTGATTGCAGCTGTTCAGGGCAACAACGAAGTGTCCATGTGGGAC

ATGGAGACTGGTGACAGAAGATTTACTCTCTGGGCCAGCAGTGCACCACCACTTTCTGAATTACAGCCTT

CTCCTCATAGCGTCCATGGTATCTACTGTAGTCCTGCAGATGGAAATCCTATCCTACTAACAGCTGGCTC

AGATATGAAAATAAGGTTTTGGGACTTGGCTTACCCAGAAAGGTCCTATGTTGTTGCAGGAAGTACTAGT

TCCCCATCTGTGTCCTACTACAGGAAAATAATTGAAGGCACTGAAGTTGTCCAGGAAATTCAGAATAAGC

AGAAAGTAGGACCAAGTGATGACACCCCTCGAAGGGGCCCAGAGTCCCTGCCCGTGGGACATCATGACAT

CATCACTGATGTCGCCACATTCCAGACCACACAGGGCTTCATCGTAACTGCTTCTAGAGATGGGATTGTG

AAGGTGTGGAAATAAAACCTACTGATTTGTATAAATTTTAATAGTTATAAATATAATACTATAACTCGAG

AAAAGGCATTTCTAGAGAACAGATTCATTTGCTTAATTTTCAAAATTATGTCTCCATATTACTGTTTCAT

GACTGACTGACTAAATGACACCCAAAATGGTTAAGATGTACTTGACTAGTTTACTTATGCATCTCTTTGC

AAGAATCAGCCAGCCAACAATGTCTGGGATTTTTATTGTATATGTTATAGAGGTGAGAAATGTAAAATAT

GAAAATGAATATGTTTATTTTGTATTGAAAAAGATGGTTGAAAAGATGGTTGTAAGCTATTATAGTATAA

ACACATTTTTGCTATTAAAAATGCTATTCAAAGCAGTTAAACTGTA

>NM_032409.3 Homo sapiens PTEN induced kinase 1 (PINK1), mRNA; nuclear gene for mitochondrial product

AGAGGCACCGCCCCAAGTTTGTTGTGACCGGCGGGGGACGCCGGTGGTGGCGGCAGCGGCGGCTGCGGGG

GCACCGGGCCGCGGCGCCACCATGGCGGTGCGACAGGCGCTGGGCCGCGGCCTGCAGCTGGGTCGAGCGC

TGCTGCTGCGCTTCACGGGCAAGCCCGGCCGGGCCTACGGCTTGGGGCGGCCGGGCCCGGCGGCGGGCTG

TGTCCGCGGGGAGCGTCCAGGCTGGGCCGCAGGACCGGGCGCGGAGCCTCGCAGGGTCGGGCTCGGGCTC

CCTAACCGTCTCCGCTTCTTCCGCCAGTCGGTGGCCGGGCTGGCGGCGCGGTTGCAGCGGCAGTTCGTGG

TGCGGGCCTGGGGCTGCGCGGGCCCTTGCGGCCGGGCAGTCTTTCTGGCCTTCGGGCTAGGGCTGGGCCT

CATCGAGGAAAAACAGGCGGAGAGCCGGCGGGCGGTCTCGGCCTGTCAGGAGATCCAGGCAATTTTTACC

CAGAAAAGCAAGCCGGGGCCTGACCCGTTGGACACGAGACGCTTGCAGGGCTTTCGGCTGGAGGAGTATC

TGATAGGGCAGTCCATTGGTAAGGGCTGCAGTGCTGCTGTGTATGAAGCCACCATGCCTACATTGCCCCA

GAACCTGGAGGTGACAAAGAGCACCGGGTTGCTTCCAGGGAGAGGCCCAGGTACCAGTGCACCAGGAGAA

GGGCAGGAGCGAGCTCCGGGGGCCCCTGCCTTCCCCTTGGCCATCAAGATGATGTGGAACATCTCGGCAG

GTTCCTCCAGCGAAGCCATCTTGAACACAATGAGCCAGGAGCTGGTCCCAGCGAGCCGAGTGGCCTTGGC

TGGGGAGTATGGAGCAGTCACTTACAGAAAATCCAAGAGAGGTCCCAAGCAACTAGCCCCTCACCCCAAC

ATCATCCGGGTTCTCCGCGCCTTCACCTCTTCCGTGCCGCTGCTGCCAGGGGCCCTGGTCGACTACCCTG

ATGTGCTGCCCTCACGCCTCCACCCTGAAGGCCTGGGCCATGGCCGGACGCTGTTCCTCGTTATGAAGAA

CTATCCCTGTACCCTGCGCCAGTACCTTTGTGTGAACACACCCAGCCCCCGCCTCGCCGCCATGATGCTG

CTGCAGCTGCTGGAAGGCGTGGACCATCTGGTTCAACAGGGCATCGCGCACAGAGACCTGAAATCCGACA

ACATCCTTGTGGAGCTGGACCCAGACGGCTGCCCCTGGCTGGTGATCGCAGATTTTGGCTGCTGCCTGGC

TGATGAGAGCATCGGCCTGCAGTTGCCCTTCAGCAGCTGGTACGTGGATCGGGGCGGAAACGGCTGTCTG

ATGGCCCCAGAGGTGTCCACGGCCCGTCCTGGCCCCAGGGCAGTGATTGACTACAGCAAGGCTGATGCCT

GGGCAGTGGGAGCCATCGCCTATGAAATCTTCGGGCTTGTCAATCCCTTCTACGGCCAGGGCAAGGCCCA

CCTTGAAAGCCGCAGCTACCAAGAGGCTCAGCTACCTGCACTGCCCGAGTCAGTGCCTCCAGACGTGAGA

CAGTTGGTGAGGGCACTGCTCCAGCGAGAGGCCAGCAAGAGACCATCTGCCCGAGTAGCCGCAAATGTGC

TTCATCTAAGCCTCTGGGGTGAACATATTCTAGCCCTGAAGAATCTGAAGTTAGACAAGATGGTTGGCTG

GCTCCTCCAACAATCGGCCGCCACTTTGTTGGCCAACAGGCTCACAGAGAAGTGTTGTGTGGAAACAAAA

ATGAAGATGCTCTTTCTGGCTAACCTGGAGTGTGAAACGCTCTGCCAGGCAGCCCTCCTCCTCTGCTCAT

GGAGGGCAGCCCTGTGATGTCCCTGCATGGAGCTGGTGAATTACTAAAAGAACATGGCATCCTCTGTGTC

GTGATGGTCTGTGAATGGTGAGGGTGGGAGTCAGGAGACAAGACAGCGCAGAGAGGGCTGGTTAGCCGGA

AAAGGCCTCGGGCTTGGCAAATGGAAGAACTTGAGTGAGAGTTCAGTCTGCAGTCCTCTGCTCACAGACA

TCTGAAAAGTGAATGGCCAAGCTGGTCTAGTAGATGAGGCTGGACTGAGGAGGGGTAGGCCTGCATCCAC

AGAGAGGATCCAGGCCAAGGCACTGGCTGTCAGTGGCAGAGTTTGGCTGTGACCTTTGCCCCTAACACGA

GGAACTCGTTTGAAGGGGGCAGCGTAGCATGTCTGATTTGCCACCTGGATGAAGGCAGACATCAACATGG

GTCAGCACGTTCAGTTACGGGAGTGGGAAATTACATGAGGCCTGGGCCTCTGCGTTCCCAAGCTGTGCGT

TCTGGACCAGCTACTGAATTATTAATCTCACTTAGCGAAAGTGACGGATGAGCAGTAAGTAAGTAAGTGT

GGGGATTTAAACTTGAGGGTTTCCCTCCTGACTAGCCTCTCTTACAGGAATTGTGAAATATTAAATGCAA

ATTTACAACTGCAGATGACGTATGTGCCTTGAACTGAATATTTGGCTTTAAGAATGATTCTTATACTCTG

AAGGTGAGAATATTTTGTGGGCAGGTATCAACATTGGGGAAGAGATTTCATGTCTAACTAACTAACTTTA

TACATGATTTTTAGGAAGCTATTGCCTAAATCAGCGTCAACATGCAGTAAAGGTTGTCTTCAACTGA

>NM_014330.5 Homo sapiens protein phosphatase 1 regulatory subunit 15A (PPP1R15A), mRNA

GCTCTTATCGGTTCCCATCCCAGTTGTTGATCTTATGCAAGACGCTGCACGACCCCGCGCCCGCTTGTCG

CCACGGCACTTGAGGCAGCCGGAGATACTCTGAGTTACTCGGAGCCCGACGCCTGAGGGTGAGATGAACG

CGCTGGCCTCCCTAACCGTCCGGACCTGTGATCGCTTCTGGCAGACCGAACCGGCGCTCCTGCCCCCGGG

GTGACGCGCAGCTCCCAGCCGCCCAGACACATGGCCCCAGGCCAAGCACCCCATCAGGCTACCCCGTGGA

GGGATGCCCACCCTTTCTTCCTCCTGTCCCCAGTGATGGGCCTCCTCAGCCGCGCCTGGAGCCGCCTGAG

GGGCCTGGGACCTCTAGAGCCCTGGCTGGTGGAAGCAGTAAAAGGAGCAGCTCTGGTAGAAGCTGGCCTG

GAGGGAGAAGCTAGGACTCCTCTGGCAATCCCCCATACCCCTTGGGGCAGACGCCCTGAAGAGGAGGCTG

AAGACAGTGGAGGCCCTGGAGAGGACAGAGAAACACTGGGGCTGAAAACCAGCAGTTCCCTTCCTGAAGC

CTGGGGACTTTTGGATGATGATGATGGCATGTATGGTGAGCGAGAGGCAACCAGTGTCCCTAGAGGGCAG

GGAAGTCAATTTGCAGATGGCCAGCGTGCTCCCCTGTCTCCCAGCCTTCTGATAAGGACACTGCAAGGTT

CTGATAAGAACCCAGGGGAGGAGAAAGCCGAGGAAGAGGGAGTTGCTGAAGAGGAGGGAGTTAACAAGTT

CTCTTATCCACCATCACACCGGGAGTGTTGTCCAGCCGTGGAGGAGGAGGACGATGAAGAAGCTGTAAAG

AAAGAAGCTCACAGAACCTCTACTTCTGCCTTGTCTCCAGGATCCAAGCCCAGCACTTGGGTGTCTTGCC

CAGGGGAGGAAGAGAATCAAGCCACGGAGGATAAAAGAACAGAAAGAAGTAAAGGAGCCAGGAAGACCTC

CGTGTCCCCCCGATCTTCAGGCTCCGACCCCAGGTCCTGGGAGTATCGTTCAGGAGAGGCGTCCGAGGAG

AAGGAGGAAAAGGCACACAAAGAAACTGGGAAAGGAGAAGCTGCCCCAGGGCCGCAATCCTCAGCCCCAG

CCCAGAGGCCCCAGCTCAAGTCCTGGTGGTGCCAACCCAGTGATGAAGAGGAGGGTGAGGTCAAGGCTTT

GGGGGCAGCTGAGAAGGATGGAGAAGCTGAGTGTCCTCCCTGCATCCCCCCACCAAGTGCCTTCCTGAAG

GCCTGGGTGTATTGGCCAGGAGAGGACACAGAGGAAGAGGAAGATGAGGAAGAAGATGAGGACAGTGACT

CTGGATCAGATGAGGAAGAGGGAGAAGCTGAGGCTTCCTCTTCCACTCCTGCTACAGGTGTCTTCTTGAA

GTCCTGGGTCTATCAGCCAGGAGAGGACACAGAGGAGGAGGAAGATGAGGACAGTGATACAGGATCAGCC

GAGGATGAAAGAGAAGCTGAGACTTCTGCTTCCACACCCCCTGCAAGTGCTTTCTTGAAGGCCTGGGTGT

ATCGGCCAGGAGAGGACACGGAGGAGGAGGAAGATGAGGATGTGGATAGTGAGGATAAGGAAGATGATTC

AGAAGCAGCCTTGGGAGAAGCTGAGTCAGACCCACATCCCTCCCACCCGGACCAGAGGGCCCACTTCAGG

GGCTGGGGATATCGACCTGGAAAAGAGACAGAGGAAGAGGAAGCTGCTGAGGACTGGGGAGAAGCTGAGC

CCTGCCCCTTCCGAGTGGCCATCTATGTACCTGGAGAGAAGCCACCGCCTCCCTGGGCTCCTCCTAGGCT

GCCCCTCCGACTGCAAAGGCGGCTCAAGCGCCCAGAAACCCCTACTCATGATCCGGACCCTGAGACTCCC

CTAAAGGCCAGAAAGGTGCGCTTCTCCGAGAAGGTCACTGTCCATTTCCTGGCTGTCTGGGCAGGGCCGG

CCCAGGCCGCCCGCCAGGGCCCCTGGGAGCAGCTTGCTCGGGATCGCAGCCGCTTCGCACGCCGCATCAC

CCAGGCCCAGGAGGAGCTGAGCCCCTGCCTCACCCCTGCTGCCCGGGCCAGAGCCTGGGCACGCCTCAGG

AACCCACCTTTAGCCCCCATCCCTGCCCTCACCCAGACCTTGCCTTCCTCCTCTGTCCCTTCGTCCCCAG

TCCAGACCACGCCCTTGAGCCAAGCTGTGGCCACACCTTCCCGCTCGTCTGCTGCTGCAGCGGCTGCCCT

GGACCTCAGTGGGAGGCGTGGCTGAGACCAACTGGTTTGCCTATAATTTATTAACTATTTATTTTTTCTA

AGTGTGGGTTTATATAAGGAATAAAGCCTTTTGATTTGTA

>NM_006253.5 Homo sapiens protein kinase AMP-activated non-catalytic subunit beta 1 (PRKAB1), mRNA

GACTCCTTCCGCAGGCTCCTTGGGACCCGCGGTTCCGGGAGTCCCTTGCTCAGGGTCCCTTTCCTGCAGT

GAGGCGCCGTCCGCCTTCCCTGTGTCCCCGCAGACCCCCATCATGGGCAATACCAGCAGTGAGCGCGCCG

CGCTGGAGCGGCATGGTGGCCATAAGACGCCCCGGAGGGACAGCTCGGGGGGCACCAAGGACGGGGACAG

GCCCAAGATCCTGATGGACAGCCCCGAAGACGCCGACCTCTTCCACTCCGAGGAAATCAAGGCACCAGAG

AAGGAGGAATTCCTGGCCTGGCAGCATGATCTGGAAGTGAATGATAAAGCTCCCGCCCAGGCTCGGCCAA

CGGTGTTTCGATGGACGGGGGGCGGAAAGGAAGTTTACTTATCTGGGTCCTTCAACAACTGGAGTAAACT

TCCCCTCACCAGAAGCCACAATAACTTTGTAGCCATCCTGGATCTGCCGGAAGGAGAGCATCAGTACAAG

TTCTTTGTGGATGGTCAGTGGACGCACGACCCTTCCGAGCCCATAGTAACCAGCCAGCTTGGCACAGTTA

ACAACATCATTCAAGTGAAGAAAACTGACTTTGAAGTATTTGATGCTTTAATGGTGGATTCCCAAAAGTG

CTCCGATGTGTCTGAGCTGTCCAGTTCTCCCCCAGGACCCTACCATCAGGAGCCCTACGTCTGCAAACCC

GAAGAGCGCTTTCGGGCACCCCCTATTCTCCCCCCACATCTCCTCCAGGTCATCCTGAACAAGGACACGG

GGATTTCCTGTGATCCAGCTTTGCTTCCTGAGCCCAATCACGTCATGCTGAACCACCTATACGCGCTGTC

TATCAAGGATGGAGTGATGGTGCTCAGCGCAACCCACCGGTACAAGAAGAAGTACGTCACCACCTTGTTA

TACAAGCCCATATGAAGAGCTGGGGGCGGATGGTGGCCCAGGAGACAGCACACCACCAGGCTCCACACGT

GCATGCTTTCCCCAAGAGGGAATGGACTGTACATTGCTCATTTCACACTCTTCAGAAGACATTTCATACC

TGCCCTGGTCCTGCTTGAAGGTTTGTCCAGGCAGAGCAGCTCCTGCAGCGCCTCGGTCTGTGACAGTCCT

CCTAGCACCCCCATGGCTTTGAGCCTCGGGGACTCATCAAGTCCAAGAAAAGAGGGAGGGGTGGCAGAGG

ATCTGCAGCCCTGGCCCCGCGGTGCATGAGGCTGGGTGCAGTTCTAAACCTACATTCTCGATTTTTCTTA

AGCCAAAAATGAATGCTAACTCCTTTGCCAGTAAAATTCTGGGAAACAGGGACTGAGGCCACACATCATT

TCCAGTCATCTGTGTGTTTTTAAGGCCAGCCACTTGTCCCTGTTGAGGCCTGGCTATGGAACTAAATACA

GTGTTGGTCTTGCCTGTCCTTCAAAATCAACAACAGATTGTCTCTCGGCTCCAGGGAGGTGTCATTTCTA

TAGAAATTAGAAGCTTTCTGATTTCTAGATGAGGTTTTACAATTGTTTCTTACAGTCATGTGCACTAAGT

ACTCTTTTTGTAAGCAGAGGTGGCTGGCTCTGCAGCCTTAAGGCCATTTTTTAAGTCACCACGTCTAGAA

GTCACATGAACTCTGCTCAGCAATAATCTGTTCTCAGAACAGACTTTTCAACCTGCTGCCGGATTTCTCC

ATTCAGCTGGATGATCCTCAGGACTGACCAGTTAGCTGGCAGGTTGTCCAGCTTTTTATTCCAGTCATAA

TAGGTGACAGTGTTAACCGTGAAAACTTGAGAGGCACTCTGCCCTCTTCCCTATAAAATCACACAGCGTG

ATTTTACAAGGTCCCGTGGCACCTTGCTCAGGACCTCTGCCCCTAGTTAGCAAGACTGCAGCAGTTGCTG

TTGCTTATTCTGAAAGGAATGTAGAACTTGACAGCAGCCTTCTGAGTCTGGGTCAGGAAGATGTCCTTTG

GACCAAAGCAGACTTCTTTATACGCAGCTCAGTTTCCCGGGAGTCGCCACAGATGTACCCACTAGCCCAG

GTTGCTGTGAGTCAGCGGAAGCTCCCGTTATGCCCTTTGCTCCTGGTGGGAGAGGGAGGAGTGAGCTCCC

TGGGTTCCAGTATTTACTTGGTATACCTGAGTTTGGGGGTACCCTTTTTTGTGACTTTTCAAAACAGTGA

ATTACTGTCACCTTGATGGACAAGTTTCAATAAAACTTTGTAAAAATAA

>NM_001316327.2 Homo sapiens protein kinase C delta (PRKCD), transcript variant 3, mRNA

AGTTCCCGTTTCCGAAACTGAGAAACTGAGCCGCGGTCAGCGAAAGTCCCGCCGCGCCGAGACCTGCCGG

TCTGCAGGGAACTGGCCAGGCAAGGGGGCAGGCCCGTTTCTCCTGGTGGTTGGTGCGTTGTAGCAGCAGC

GGGAGCCAGGACTAAGGACAAGCAGGAGCTGGGAGCCCCAGCCCCACTGCAGGCCCCACCATGGCGCCGT

TCCTGCGCATCGCCTTCAACTCCTATGAGCTGGGCTCCCTGCAGGCCGAGGACGAGGCGAACCAGCCCTT

CTGTGCCGTGAAGATGAAGGAGGCGCTCAGCACAGAGCGTGGGAAAACACTGGTGCAGAAGAAGCCGACC

ATGTATCCTGAGTGGAAGTCGACGTTCGATGCCCACATCTATGAGGGGCGCGTCATCCAGATTGTGCTAA

TGCGGGCAGCAGAGGAGCCAGTGTCTGAGGTGACCGTGGGTGTGTCGGTGCTGGCCGAGCGCTGCAAGAA

GAACAATGGCAAGGCTGAGTTCTGGCTGGACCTGCAGCCTCAGGCCAAGGTGTTGATGTCTGTTCAGTAT

TTCCTGGAGGACGTGGATTGCAAACAGTCTATGCGCAGTGAGGACGAGGCCAAGTTCCCAACGATGAACC

GCCGCGGAGCCATCAAACAGGCCAAAATCCACTACATCAAGAACCATGAGTTTATCGCCACCTTCTTTGG

GCAACCCACCTTCTGTTCTGTGTGCAAAGACTTTGTCTGGGGCCTCAACAAGCAAGGCTACAAATGCAGG

CAATGTAACGCTGCCATCCACAAGAAATGCATCGACAAGATCATCGGCAGATGCACTGGCACCGCGGCCA

ACAGCCGGGACACTATATTCCAGAAAGAACGCTTCAACATCGACATGCCGCACCGCTTCAAGGTTCACAA

CTACATGAGCCCCACCTTCTGTGACCACTGCGGCAGCCTGCTCTGGGGACTGGTGAAGCAGGGATTAAAG

TGTGAAGACTGCGGCATGAATGTGCACCATAAATGCCGGGAGAAGGTGGCCAACCTCTGCGGCATCAACC

AGAAGCTTTTGGCTGAGGCCTTGAACCAAGTCACCCAGAGAGCCTCCCGGAGATCAGACTCAGCCTCCTC

AGAGCCTGTTGGGATATATCAGGGTTTCGAGAAGAAGACCGGAGTTGCTGGGGAGGACATGCAAGACAAC

AGTGGGACCTACGGCAAGATCTGGGAGGGCAGCAGCAAGTGCAACATCAACAACTTCATCTTCCACAAGG

TCCTGGGCAAAGGCAGCTTCGGGAAGGTGCTGCTTGGAGAGCTGAAGGGCAGAGGAGAGTACTTTGCCAT

CAAGGCCCTCAAGAAGGATGTGGTCCTGATCGACGACGACGTGGAGTGCACCATGGTTGAGAAGCGGGTG

CTGACACTTGCCGCAGAGAATCCCTTTCTCACCCACCTCATCTGCACCTTCCAGACCAAGGACCACCTGT

TCTTTGTGATGGAGTTCCTCAACGGGGGGGACCTGATGTACCACATCCAGGACAAAGGCCGCTTTGAACT

CTACCGTGCCACGTTTTATGCCGCTGAGATAATGTGTGGACTGCAGTTTCTACACAGCAAGGGCATCATT

TACAGGGACCTCAAACTGGACAATGTGCTGCTGGACCGGGATGGCCACATCAAGATTGCCGACTTTGGGA

TGTGCAAAGAGAACATATTCGGGGAGAGCCGGGCCAGCACCTTCTGCGGCACCCCTGACTATATCGCCCC

TGAGATCCTACAGGGCCTGAAGTACACATTCTCTGTGGACTGGTGGTCTTTCGGGGTCCTTCTGTACGAG

ATGCTCATTGGCCAGTCCCCCTTCCATGGTGATGATGAGGATGAACTCTTCGAGTCCATCCGTGTGGACA

CGCCACATTATCCCCGCTGGATCACCAAGGAGTCCAAGGACATCCTGGAGAAGCTCTTTGAAAGGGAACC

AACCAAGAGGCTGGGAGTGACCGGAAACATCAAAATCCACCCCTTCTTCAAGACCATAAACTGGACTCTG

CTGGAAAAGCGGAGGTTGGAGCCACCTTTCAGGCCCAAAGTGAAGTCACCCAGAGACTACAGTAACTTTG

ACCAGGAGTTCCTGAACGAGAAGGCGCGCCTCTCCTACAGCGACAAGAACCTCATCGACTCCATGGACCA

GTCTGCATTCGCTGGCTTCTCCTTTGTGAACCCCAAATTCGAGCACCTCCTGGAAGATTGAGGTTCCTGG

ACAGATCAGGCTAGCCCTGCCCTCCACCCACACCTGCCCGCTCCCCACGATAAGCACCAGTGGGACTGTG

GTGACTTCTGCTGCTGGCCCCGCCCCTGCCCCCAGAGCGTCCTTGGCTGCCGTCTGGCCGGGCTCTCATG

GTACTTCCTCTGTGAACTGTGTGTGAATCTGCTTTTCCTCTGCCTTCGGAGGGAAATTGTAAATCCTGTG

TTTCATTACTTGAATGTAGTTATCTATTGAAAATATATATTATATACATAGACATATATATATATATAAT

AGGCTGTATATATTGCTCAGTAGAGAAAAACCATGGGGGACTGGTGATATGTTGATCTTTTTCAAAAAAA

TATATATATGACAAAAAAAAAAAAAAAGGAGCACAAGCTGTTTGAACCACCAGGTTTATTTGTGTGTCTA

AATAAACACCAAATAGTACCAA

>NM_004562.3 Homo sapiens parkin RBR E3 ubiquitin protein ligase (PRKN), transcript variant 1, mRNA

AGGATTTAACCCAGGAGAGCCGCTGGTGGGAGGCGCGGCTGGCGCCGCTGCGCGCATGGGCCTGTTCCTG

GCCCGCAGCCGCCACCTACCCAGTGACCATGATAGTGTTTGTCAGGTTCAACTCCAGCCATGGTTTCCCA

GTGGAGGTCGATTCTGACACCAGCATCTTCCAGCTCAAGGAGGTGGTTGCTAAGCGACAGGGGGTTCCGG

CTGACCAGTTGCGTGTGATTTTCGCAGGGAAGGAGCTGAGGAATGACTGGACTGTGCAGAATTGTGACCT

GGATCAGCAGAGCATTGTTCACATTGTGCAGAGACCGTGGAGAAAAGGTCAAGAAATGAATGCAACTGGA

GGCGACGACCCCAGAAACGCGGCGGGAGGCTGTGAGCGGGAGCCCCAGAGCTTGACTCGGGTGGACCTCA

GCAGCTCAGTCCTCCCAGGAGACTCTGTGGGGCTGGCTGTCATTCTGCACACTGACAGCAGGAAGGACTC

ACCACCAGCTGGAAGTCCAGCAGGTAGATCAATCTACAACAGCTTTTATGTGTATTGCAAAGGCCCCTGT

CAAAGAGTGCAGCCGGGAAAACTCAGGGTACAGTGCAGCACCTGCAGGCAGGCAACGCTCACCTTGACCC

AGGGTCCATCTTGCTGGGATGATGTTTTAATTCCAAACCGGATGAGTGGTGAATGCCAATCCCCACACTG

CCCTGGGACTAGTGCAGAATTTTTCTTTAAATGTGGAGCACACCCCACCTCTGACAAGGAAACATCAGTA

GCTTTGCACCTGATCGCAACAAATAGTCGGAACATCACTTGCATTACGTGCACAGACGTCAGGAGCCCCG

TCCTGGTTTTCCAGTGCAACTCCCGCCACGTGATTTGCTTAGACTGTTTCCACTTATACTGTGTGACAAG

ACTCAATGATCGGCAGTTTGTTCACGACCCTCAACTTGGCTACTCCCTGCCTTGTGTGGCTGGCTGTCCC

AACTCCTTGATTAAAGAGCTCCATCACTTCAGGATTCTGGGAGAAGAGCAGTACAACCGGTACCAGCAGT

ATGGTGCAGAGGAGTGTGTCCTGCAGATGGGGGGCGTGTTATGCCCCCGCCCTGGCTGTGGAGCGGGGCT

GCTGCCGGAGCCTGACCAGAGGAAAGTCACCTGCGAAGGGGGCAATGGCCTGGGCTGTGGGTTTGCCTTC

TGCCGGGAATGTAAAGAAGCGTACCATGAAGGGGAGTGCAGTGCCGTATTTGAAGCCTCAGGAACAACTA

CTCAGGCCTACAGAGTCGATGAAAGAGCCGCCGAGCAGGCTCGTTGGGAAGCAGCCTCCAAAGAAACCAT

CAAGAAAACCACCAAGCCCTGTCCCCGCTGCCATGTACCAGTGGAAAAAAATGGAGGCTGCATGCACATG

AAGTGTCCGCAGCCCCAGTGCAGGCTCGAGTGGTGCTGGAACTGTGGCTGCGAGTGGAACCGCGTCTGCA

TGGGGGACCACTGGTTCGACGTGTAGCCAGGGCGGCCGGGCGCCCCATCGCCACATCCTGGGGGAGCATA

CCCAGTGTCTACCTTCATTTTCTAATTCTCTTTTCAAACACACACACACACGCGCGCGCGCACACACA

CTCTTCAAGTTTTTTTCAAAGTCCAACTACAGCCAAATTGCAGAAGAAACTCCTGGATCCCTTTCACTAT

GTCCATGAAAAACAGCAGAGTAAAATTACAGAAGAAGCTCCTGAATCCCTTTCAGTTTGTCCACACAAGA

CAGCAGAGCCATCTGCGACACCACCAACAGGCGTTCTCAGCCTCCGGATGACACAAATACCAGAGCACAG

ATTCAAGTGCAATCCATGTATCTGTATGGGTCATTCTCACCTGAATTCGAGACAGGCAGAATCAGTAGCT

GGAGAGAGAGTTCTCACATTTAATATCCTGCCTTTTACCTTCAGTAAACACCATGAAGATGCCATTGACA

AGGTGTTTCTCTGTAAAATGAACTGCAGTGGGTTCTCCAAACTAGATTCATGGCTTTAACAGTAATGTTC

TTATTTAAATTTTCAGAAAGCATCTATTCCCAAAGAACCCCAGGCAATAGTCAAAAACATTTGTTTATCC

TTAAGAATTCCATCTATATAAATCGCATTAATGAAATACCAACTATGCGTAAATCAACTTGTCACAAAGT

GAGAAATTATGAAAGTTAATTTGAATGTTGAATGTTTGAATTACAGGGAAGAAATCAAGTTAATGTACTT

TCATTCCCTTTCATGATTTGCAACTTTAGAAAGAAATTGTTTTTCTGAAAGTATCACCAAAAAATCTATA

GTTTGATTCTGAGTATTCATTTTGCAACTTGGAGATTTTGCTAATACATTTGGCTCCACTGTAAATTTAA

TAGATAAAGTGCCTATAAAGGAAACACGTTTAGAAATGATTTCAAAATGATATTCAATCTTAACAAAAGT

GAACATTATTAAATCAGAATCTTTAAAGAGGAGCCTTTCCAGAACTACCAAAATGAAGACACGCCCGACT

CTCTCCATCAGAAGGGTTTATACCCCTTTGGCACACCCTCTCTGTCCAATCTGCAAGTCCCAGGGAGCTC

TGCATACCAGGGGTTCCCCAGGAGAGACCTTCTCTTAGGACAGTAAACTCACTAGAATATTCCTTATGTT

GACATGGATTGGATTTCAGTTCAATCAAACTTTCAGCTTTTTTTTCAGCCATTCACAACACAATCAAAAG

ATTAACAACACTGCATGCGGCAAACCGCATGCTCTTACCCACACTACGCAGAAGAGAAAGTACAACCACT

ATCTTTTGTTCTACCTGTATTGTCTGACTTCTCAGGAAGATCGTGAACATAACTGAGGGCATGAGTCTCA

CTAGCACATGGAGGCCCTTTTGGATTTAGAGACTGTAAATTATTAAATCGGCAACAGGGCTTCTCTTTTT

AGATGTAGCACTGAAATCCTTGCTGGAGGGAAGAGAGGGGATGAACTCAAGTTTTCCACATCCTGGGACA

CCTGTCCCTCTTTTCCTAACTGCCTAAGATAACCCATTTCTTCCAACCATCTGAGGACAGTCCCGTCGTC

TCAGAGGCCCTGCACCGGGGAGAGACTGGGCTCTGCAGCAGCCACATCAGCATTCACAGCTTCATGTGGC

TTCACTGTCTGAAAATCTACCGACTCCAACATGGCCCCACGGTGACAACAGACCTGTGACAGGAAGCCCA

AAGCTCACATAGAAATGGTGGACAGATCAAAGTCTCTATAGTAAGGGAAAAAAAGAGAGGTGGCAGGCAT

GAGCCCCCTGCACCCAGTGGCTCGTGTCCATACTGAGTCCAGACCCTGATCAAGGCCTGACTTAGTGTCA

CTGGCAGTCCCACTAAATTACACTTCCTTACACTGGCCCGATGCGACAAATCAGGTGGCTCCCTTCTGTC

ACGTGGAGCACACAGTGTTTTCCATCATCCATAGCTTTCTTCCTGATGGTGTTTGCATTATTGCGCCTTC

CCAATCTGCATGCTGCGTTGGGCTTGCGGTGCCTGAACAAGGTTTGCTCCCATGAGCTCAGGCACCCTAG

GATCCCCTGTTAGACTATTAGGCTGTCCAGCATGGTCTCCTTTCCCTTCTTGGTGGTGGTCTTTTCCCTT

TCCAGAATAGAACAGTGATTCTTAAAATAAGTTAGAGCAGGCCGGGCGCGGTGGCTCATGCCTGTAATCC

CAGCACTTTGGGAGGCCGAGGTGGGTGGATCACGAGGTCAGGAGTTCAAGACCAGCCTGGCCAAGATGAT

GAAACCCCGTCTCTATTAAAAATACAAAAATTAGCTGGGCGTGGTGGCAGGCACCTGTAATCCCAGCTTC

CTGGGAGGCTGAGGCAGGAGAATCACTTGAACCCGGGGGGCAGAGGTTGCAGTGAGCCGAGATCACGCCA

CTGAACTCCAGCCTGGGCAACAGAGTGAGACTCTGTCTCAAAAAAAAAAAAAAAACAAAAACAAAAAAGC

AAGATCATCCACTACACATGAACATGAATCACAGTATTATTTGCACAGGAAGGGTGTAACAAAATATGAA

TGTATCAAAAAATAGAAATAAAGTCTTTGCAGAAAAAGTCTGTTTTTCTCTGAAGTGTGTTGAGATTATC

TGACAACTCTAAGATTGTACTTAAATTGTCAATAAAGCATCAAAAGAG

>NM_000314.8 Homo sapiens phosphatase and tensin homolog (PTEN), transcript variant 1, mRNA

GTTCTCTCCTCTCGGAAGCTGCAGCCATGATGGAAGTTTGAGAGTTGAGCCGCTGTGAGGCGAGGCCGGG

CTCAGGCGAGGGAGATGAGAGACGGCGGCGGCCGCGGCCCGGAGCCCCTCTCAGCGCCTGTGAGCAGCCG

CGGGGGCAGCGCCCTCGGGGAGCCGGCCGGCCTGCGGCGGCGGCAGCGGCGGCGTTTCTCGCCTCCTCTT

CGTCTTTTCTAACCGTGCAGCCTCTTCCTCGGCTTCTCCTGAAAGGGAAGGTGGAAGCCGTGGGCTCGGG

CGGGAGCCGGCTGAGGCGCGGCGGCGGCGGCGGCACCTCCCGCTCCTGGAGCGGGGGGGAGAAGCGGCGG

CGGCGGCGGCCGCGGCGGCTGCAGCTCCAGGGAGGGGGTCTGAGTCGCCTGTCACCATTTCCAGGGCTGG

GAACGCCGGAGAGTTGGTCTCTCCCCTTCTACTGCCTCCAACACGGCGGCGGCGGCGGCTGGCACATCCA

GGGACCCGGGCCGGTTTTAAACCTCCCGTGCGCCGCCGCCGCACCCCCCGTGGCCCGGGCTCCGGAGGCC

GCCGGCGGAGGCAGCCGTTCGGAGGATTATTCGTCTTCTCCCCATTCCGCTGCCGCCGCTGCCAGGCCTC

TGGCTGCTGAGGAGAAGCAGGCCCAGTCGCTGCAACCATCCAGCAGCCGCCGCAGCAGCCATTACCCGGC

TGCGGTCCAGAGCCAAGCGGCGGCAGAGCGAGGGGCATCAGCTACCGCCAAGTCCAGAGCCATTTCCATC

CTGCAGAAGAAGCCCCGCCACCAGCAGCTTCTGCCATCTCTCTCCTCCTTTTTCTTCAGCCACAGGCTCC

CAGACATGACAGCCATCATCAAAGAGATCGTTAGCAGAAACAAAAGGAGATATCAAGAGGATGGATTCGA

CTTAGACTTGACCTATATTTATCCAAACATTATTGCTATGGGATTTCCTGCAGAAAGACTTGAAGGCGTA

TACAGGAACAATATTGATGATGTAGTAAGGTTTTTGGATTCAAAGCATAAAAACCATTACAAGATATACA

ATCTTTGTGCTGAAAGACATTATGACACCGCCAAATTTAATTGCAGAGTTGCACAATATCCTTTTGAAGA

CCATAACCCACCACAGCTAGAACTTATCAAACCCTTTTGTGAAGATCTTGACCAATGGCTAAGTGAAGAT

GACAATCATGTTGCAGCAATTCACTGTAAAGCTGGAAAGGGACGAACTGGTGTAATGATATGTGCATATT

TATTACATCGGGGCAAATTTTTAAAGGCACAAGAGGCCCTAGATTTCTATGGGGAAGTAAGGACCAGAGA

CAAAAAGGGAGTAACTATTCCCAGTCAGAGGCGCTATGTGTATTATTATAGCTACCTGTTAAAGAATCAT

CTGGATTATAGACCAGTGGCACTGTTGTTTCACAAGATGATGTTTGAAACTATTCCAATGTTCAGTGGCG

GAACTTGCAATCCTCAGTTTGTGGTCTGCCAGCTAAAGGTGAAGATATATTCCTCCAATTCAGGACCCAC

ACGACGGGAAGACAAGTTCATGTACTTTGAGTTCCCTCAGCCGTTACCTGTGTGTGGTGATATCAAAGTA

GAGTTCTTCCACAAACAGAACAAGATGCTAAAAAAGGACAAAATGTTTCACTTTTGGGTAAATACATTCT

TCATACCAGGACCAGAGGAAACCTCAGAAAAAGTAGAAAATGGAAGTCTATGTGATCAAGAAATCGATAG

CATTTGCAGTATAGAGCGTGCAGATAATGACAAGGAATATCTAGTACTTACTTTAACAAAAAATGATCTT

GACAAAGCAAATAAAGACAAAGCCAACCGATACTTTTCTCCAAATTTTAAGGTGAAGCTGTACTTCACAA

AAACAGTAGAGGAGCCGTCAAATCCAGAGGCTAGCAGTTCAACTTCTGTAACACCAGATGTTAGTGACAA

TGAACCTGATCATTATAGATATTCTGACACCACTGACTCTGATCCAGAGAATGAACCTTTTGATGAAGAT

CAGCATACACAAATTACAAAAGTCTGAATTTTTTTTTATCAAGAGGGATAAAACACCATGAAAATAAACT

TGAATAAACTGAAAATGGACCTTTTTTTTTTTAATGGCAATAGGACATTGTGTCAGATTACCAGTTATAG

GAACAATTCTCTTTTCCTGACCAATCTTGTTTTACCCTATACATCCACAGGGTTTTGACACTTGTTGTCC

AGTTGAAAAAAGGTTGTGTAGCTGTGTCATGTATATACCTTTTTGTGTCAAAAGGACATTTAAAATTCAA

TTAGGATTAATAAAGATGGCACTTTCCCGTTTTATTCCAGTTTTATAAAAAGTGGAGACAGACTGATGTG

TATACGTAGGAATTTTTTCCTTTTGTGTTCTGTCACCAACTGAAGTGGCTAAAGAGCTTTGTGATATACT

GGTTCACATCCTACCCCTTTGCACTTGTGGCAACAGATAAGTTTGCAGTTGGCTAAGAGAGGTTTCCGAA

GGGTTTTGCTACATTCTAATGCATGTATTCGGGTTAGGGGAATGGAGGGAATGCTCAGAAAGGAAATAAT

TTTATGCTGGACTCTGGACCATATACCATCTCCAGCTATTTACACACACCTTTCTTTAGCATGCTACAGT

TATTAATCTGGACATTCGAGGAATTGGCCGCTGTCACTGCTTGTTGTTTGCGCATTTTTTTTTAAAGCAT

ATTGGTGCTAGAAAAGGCAGCTAAAGGAAGTGAATCTGTATTGGGGTACAGGAATGAACCTTCTGCAACA

TCTTAAGATCCACAAATGAAGGGATATAAAAATAATGTCATAGGTAAGAAACACAGCAACAATGACTTAA

CCATATAAATGTGGAGGCTATCAACAAAGAATGGGCTTGAAACATTATAAAAATTGACAATGATTTATTA

AATATGTTTTCTCAATTGTAACGACTTCTCCATCTCCTGTGTAATCAAGGCCAGTGCTAAAATTCAGATG

CTGTTAGTACCTACATCAGTCAACAACTTACACTTATTTTACTAGTTTTCAATCATAATACCTGCTGTGG

ATGCTTCATGTGCTGCCTGCAAGCTTCTTTTTTCTCATTAAATATAAAATATTTTGTAATGCTGCACAGA

AATTTTCAATTTGAGATTCTACAGTAAGCGTTTTTTTTCTTTGAAGATTTATGATGCACTTATTCAATAG

CTGTCAGCCGTTCCACCCTTTTGACCTTACACATTCTATTACAATGAATTTTGCAGTTTTGCACATTTTT

TAAATGTCATTAACTGTTAGGGAATTTTACTTGAATACTGAATACATATAATGTTTATATTAAAAAGGAC

ATTTGTGTTAAAAAGGAAATTAGAGTTGCAGTAAACTTTCAATGCTGCACACAAAAAAAAGACATTTGAT

TTTTCAGTAGAAATTGTCCTACATGTGCTTTATTGATTTGCTATTGAAAGAATAGGGTTTTTTTTTTTTT

TTTTTTTTTTTTTTTTAAATGTGCAGTGTTGAATCATTTCTTCATAGTGCTCCCCCGAGTTGGGACTAGG

GCTTCAATTTCACTTCTTAAAAAAAATCATCATATATTTGATATGCCCAGACTGCATACGATTTTAAGCG

GAGTACAACTACTATTGTAAAGCTAATGTGAAGATATTATTAAAAAGGTTTTTTTTTCCAGAAATTTGGT

GTCTTCAAATTATACCTTCACCTTGACATTTGAATATCCAGCCATTTTGTTTCTTAATGGTATAAAATTC

CATTTTCAATAACTTATTGGTGCTGAAATTGTTCACTAGCTGTGGTCTGACCTAGTTAATTTACAAATAC

AGATTGAATAGGACCTACTAGAGCAGCATTTATAGAGTTTGATGGCAAATAGATTAGGCAGAACTTCATC

TAAAATATTCTTAGTAAATAATGTTGACACGTTTTCCATACCTTGTCAGTTTCATTCAACAATTTTTAAA

TTTTTAACAAAGCTCTTAGGATTTACACATTTATATTTAAACATTGATATATAGAGTATTGATTGATTGC

TCATAAGTTAAATTGGTAAAGTTAGAGACAACTATTCTAACACCTCACCATTGAAATTTATATGCCACCT

TGTCTTTCATAAAAGCTGAAAATTGTTACCTAAAATGAAAATCAACTTCATGTTTTGAAGATAGTTATAA

ATATTGTTCTTTGTTACAATTTCGGGCACCGCATATTAAAACGTAACTTTATTGTTCCAATATGTAACAT

GGAGGGCCAGGTCATAAATAATGACATTATAATGGGCTTTTGCACTGTTATTATTTTTCCTTTGGAATGT

GAAGGTCTGAATGAGGGTTTTGATTTTGAATGTTTCAATGTTTTTGAGAAGCCTTGCTTACATTTTATGG

TGTAGTCATTGGAAATGGAAAAATGGCATTATATATATTATATATATAAATATATATTATACATACTCTC

CTTACTTTATTTCAGTTACCATCCCCATAGAATTTGACAAGAATTGCTATGACTGAAAGGTTTTCGAGTC

CTAATTAAAACTTTATTTATGGCAGTATTCATAATTAGCCTGAAATGCATTCTGTAGGTAATCTCTGAGT

TTCTGGAATATTTTCTTAGACTTTTTGGATGTGCAGCAGCTTACATGTCTGAAGTTACTTGAAGGCATCA

CTTTTAAGAAAGCTTACAGTTGGGCCCTGTACCATCCCAAGTCCTTTGTAGCTCCTCTTGAACATGTTTG

CCATACTTTTAAAAGGGTAGTTGAATAAATAGCATCACCATTCTTTGCTGTGGCACAGGTTATAAACTTA

AGTGGAGTTTACCGGCAGCATCAAATGTTTCAGCTTTAAAAAATAAAAGTAGGGTACAAGTTTAATGTTT

AGTTCTAGAAATTTTGTGCAATATGTTCATAACGATGGCTGTGGTTGCCACAAAGTGCCTCGTTTACCTT

TAAATACTGTTAATGTGTCATGCATGCAGATGGAAGGGGTGGAACTGTGCACTAAAGTGGGGGCTTTAAC

TGTAGTATTTGGCAGAGTTGCCTTCTACCTGCCAGTTCAAAAGTTCAACCTGTTTTCATATAGAATATAT

ATACTAAAAAATTTCAGTCTGTTAAACAGCCTTACTCTGATTCAGCCTCTTCAGATACTCTTGTGCTGTG

CAGCAGTGGCTCTGTGTGTAAATGCTATGCACTGAGGATACACAAAAATACCAATATGATGTGTACAGGA

TAATGCCTCATCCCAATCAGATGTCCATTTGTTATTGTGTTTGTTAACAACCCTTTATCTCTTAGTGTTA

TAAACTCCACTTAAAACTGATTAAAGTCTCATTCTTGTCATTGTGTGGGTGTTTTATTAAATGAGAGTTT

ATAATTCAAATTGCTTAAGTCCATTGAAGTTTTAATTAATGGGCAGCCAAATGTGAATACAAAGTTTTCA

GTTTTTTTTTTTCCTGCTGTCCTTCAAAGCCTACTGTTTAAAAAAAAAAAAAAAAAAAAACATGGCCTGA

GAGTAGAGTATCTGTCTACTCATGTTTAATTAAGGAAAAACACTTATTTTTAGGGCTTTAGTCATCACTT

CATAAATTGTATAAGCACATTAAATAGCGTTCTAGTCCTGAAAAAGTCCAAGATTCTTAGAAAATTGTGC

ATATTTTTATTATGACAGATGTTTGAAGATAATTCCCCAGAATGGATTTGATACTTTAGATTTCAATTTT

GTGGCTTTTGTCTATTATTCTGTACTCTGCCATCAGCATATGGAAAGCTTCATTTACTCATCATGACTTG

TGCCATATAAAAATTGATATTTCGGAATAGTCTAAAGGACTTTTTGTACTTGAATTTAATCATGTTGTTT

CTAATATTCTTAAAAGCTTGAAGACTAAAGCATATCCTTTCAACAAAGCATAGTAAGGTAATAAGAAAGT

GTAGTTTGTACAAGTGTTAAAAAAATAAAGTAGACAATGTTACAGTGGGACTTATTATTTCAAGTTTACA

TTTTCTCCATGTAATTTTTTAAAAAGTAAATGAAAAAATGTGCAATAATGTAAAATATGAAGTGTATGTG

TACACACATTTTATTTTTCGGTATCTTGGGTATACGTATGGTTGAAAACTATACTGGAGTCTAAAAGTAT

TCTAATTTATAAGAAGACATTTTGGTGATGTTTGAAAAATAGAAATGTGCTAGTTTTGTTTTTATATCAT

GTCCTTTGTACGTTGTAATATGAGCTGGCTTGGTTCAGTAAATGCCATCACCATTTCCATTGAGAATTTA

AAACTCACCAGTGTTTAATATGCAGGCTTCCAAAGGCTTATGAAAAAAATCAAGACCCTTAAATCTAGTT

AATTTGCTGCTAACATGAAACTCTTTGGTTCTTTTATTTTTGCCAGATAATTAGACACACATCTAAAGCT

TAGTCTTAAATGGCTTAAGTGTAGCTATTGATTAGTGCTGTTGCTAGTTCAGAAAGAAATGTTTGTGAAT

GGAAACAAGAATATTCAGTCCAAACTGTTGTAAGGACAGTACCTGAAAACCAGGAAACAGGATAATGGAA

AAAGTCTTTTAAAGATGAAATGTTGGAGCCAACTTTCTTATAGAATTAATTGTATGTGGCTATAGAAAGC

CTAATGATTGTTGCTTATTTTTGAGAGCATATTATTCTTTTATGACCATAATCTTGCTGTTTTTCCATCT

TCCAAAAGATCTTCCTTCTAATATGTATATCAGAATGTGGGTAGCCAGTCAGACAAATTCATATTGGTTG

GTAGCTTTAAAAAGTTTGTAATGTGAAGACAGGAAAGGACAAAATAGTTTGCTTTGGTGGTAGTACTCTG

GTTGTTAAGCTAGGTATTTTGAGACTACTTCCCCATCACAACAACAATAAAATAATCACTCATAATCCTA

TCACCTGGAGACATAGCCATCGTTAATATGTTAGTGACTATACAATCATGTTTTCTTCTGTATATCCATG

TATATTCTTTAAAAATGAAATTTATACTGTACCTGATCTCAAAGCTTTTTAGCTTAGTATATCTGTCATG

AATTTGTAGGATGTTCCATTGCATCAGAAAACGGACAGTGATTTGATTACTTTCTAATGCCACAGATGCA

GATTACATGTAGTTATTGAGAATCCTTTCGAATTCAGTGGCTTAATCATGAATGTCTAAATATTGTTGAC

ATTAGGATGATACATGTAAATTAAAGTTACATTTGTTTAGCATAGACAAGCTTAACATTGTAGATGTTTC

TCTTCAAAAATCATCTTAAACATTTGCATTTGGAATTGTGTTAAATAGAATGTGTGAAACACTGTATTAG

TAAACTTCATCACCTTTCTACTTCCTTATAGTTTGAACTTTTCAGTTTTTGTAGTTCCCAAACAGTTGCT

CAATTTAGAGCAAATTAATTTAACACCTGCCAAAAAAAGGCTGCTGTTGGCTTATCAGTTGTCTTTAAAT

TCAAATGCTCATGTGACTTTTATCACATCAAAAAATATTTCATTAATGATTCACCTTTAGCTCTGAAAAT

TACCGCGTTTAGTAATTATAGTGGGCTTATAAAAACATGCAACTCTTTTTGATAGTTATTTGAGAATTTT

GGTGAAAAATATTTAGCTGAGGGCAGTATAGAACTTATAAACCAATATATTGATATTTTTAAAACATTTT

TACATATAAGTAAACTGCCATCTTTGAGCATAACTACATTTAAAAATAAAGCTGCATATTTTTAAATCAA

GTGTTTAACAAGAATTTATATTTTTTATTTTTTAAAATTAAAAATAATTTATATTTCCTCTGTTGCATGA

GGATTCTCATCTGTGCTTATAATGGTTAGAGATTTTATTTGTGTGGAATGAAGTGAGGCTTGTAGTCATG

GTTCTAGTGTTTCAGTTTGCCAAGTCTGTTTACTGCAGTGAAATTCATCAAATGTTTCAGTGTGGTTTTC

TGTAGCCTATCATTTACTGGCTATTTTTTTATGTACACCTTTAGGATTTTCTGCCTACTCTATCCAGTTG

TCCAAATGATATCCTACATTTTACAAATGCCCTTTCAGTTTCTATTTTCTTTTTCCATTAAATTGCCCTC

ATGTCCTAATGTGCAGTTTGTAAGTGTGTGTGTGTGTCTGTGTGTGTGAATTTGATTTTCAAGAGT

GCTAGACTTCCAATTTGAGAGATTAAATAATTTAATTCAGGCAAACATTTTTCATTGGAATTTCACAGTT

CATTGTAATGAAAATGTTAATCCTGGATGACCTTTGACATACAGTAATGAATCTTGGATATTAATGAATT

TGTTAGTAGCATCTTGATGTGTGTTTTAATGAGTTATTTTCAAAGTTGTGCATTAAACCAAAGTTGGCAT

ACTGGAAGTGTTTATATCAAGTTCCATTTGGCTACTGATGGACAAAAAATAGAAATGCCTTCCTATGGAG

AGTATTTTTCCTTTAAAAAATTAAAAAGGTTAATTATTTTGACTA

>NM_001256358.2 Homo sapiens protein tyrosine kinase 6 (PTK6), transcript variant 2, mRNA

ACGCCCAGCTCTGGGTGCTCCAGCTGGGCCACAGCCTGGTCCTGCCGCTGCGCCCGCCCGCCATGGTGTC

CCGGGACCAGGCTCACCTGGGCCCCAAGTATGTGGGCCTCTGGGACTTCAAGTCCCGGACGGACGAGGAG

CTGAGCTTCCGCGCGGGGGACGTCTTCCACGTGGCCAGGAAGGAGGAGCAGTGGTGGTGGGCCACGCTGC

TGGACGAGGCGGGTGGGGCCGTGGCCCAGGGCTATGTGCCCCACAACTACCTGGCCGAGAGGGAGACGGT

GGAGTCGGAACCTGCGGGACACGCAGGCTGTGCGGCACTACAAGATCTGGCGGCGTGCCGGGGGCCGGCT

GCACCTGAACGAGGCGGTGTCCTTCCTCAGCCTGCCCGAGCTTGTGAACTACCACAGGGCCCAGAGCCTG

TCCCACGGCCTGCGGCTGGCCGCGCCCTGCCGGAAGCACGAGCCTGAGCCCCTGCCCCATTGGGATGACT

GGGAGAGGCCGAGGGAGGAGTTCACGCTCTGCAGGAAGCTGGGGTCCGGCTACTTTGGGGAGGTCTTCGA

GGGGCTCTGGAAAGACCGGGTCCAGGTGGCCATTAAGGTGATTTCTCGAGACAACCTCCTGCACCAGCAG

ATGCTGCAGTCGGAGATCCAGGCCATGAAGAAGCTGCGGCACAAACACATCCTGGCGCTGTACGCCGTGG

TGTCCGTGGGGGACCCCGTGTACATCATCACGGAGCTCATGGCCAAGGGCAGCCTGCTGGAGCTGCTCCG

CGACTCTGATGAGAAAGTCCTGCCCGTTTCGGAGCTGCTGGACATCGCCTGGCAGGTGGCTGAGGGCATG

TGTTACCTGGAGTCGCAGAATTACATCCACCGGGACCTGGCCGCCAGGAACATCCTCGTCGGGGAAAACA

CCCTCTGCAAAGTTGGGGACTTCGGGTTAGCCAGGCTTATCAAGGAGGACGTCTACCTCTCCCATGACCA

CAATATCCCCTACAAGTGGACGGCCCCTGAAGCGCTCTCCCGAGGCCATTACTCCACCAAATCCGACGTC

TGGTCCTTTGGGATTCTCCTGCATGAGATGTTCAGCAGGGGTCAGGTGCCCTACCCAGGCATGTCCAACC

ATGAGGCCTTCCTGAGGGTGGACGCCGGCTACCGCATGCCCTGCCCTCTGGAGTGCCCGCCCAGCGTGCA

CAAGCTGATGCTGACATGCTGGTGCAGGGACCCCGAGCAGAGACCCTGCTTCAAGGCCCTGCGGGAGAGG

CTCTCCAGCTTCACCAGCTACGAGAACCCGACCTGAGCTGCTGTGGAGCGGGCATGGCCGGGCCCTGCTG

AGGAGGGGCCTGGGCAGAGGGCCTGGACCTGGGATCAAGGCCCACGCGCTTCCCTGGGGTTTACTGAGGT

GATGGGTGCAGGAAAGGTTCACAAATGTGGAGTGTCTGCGTCCAATACACGCGTGTGCTCCTCTCCTTAC

TCCATCGTGTGTGCCTTGGGTCTCAGCTGCTGACACGCAGCCTGCTCTGGAGCCTGCAGATGAGATCCGG

GAGACTGACACGAAGCCAGCAGAGGTCAGAGGGGACTCTGACCACAGCCCGCTCTCTGGCTGTCTGTCTG

CAGTGCCCGGCTGAGGGTGGGAGGCAAACACGCCTTGTTCCTGCTCTTCCCAGTTCAGCTTGGTGGGAGA

AAGTCATTCGCGTGGCTCGGGACGCTCATGTAAATTTGGTTTTGGTGCTCAAGGGTTCTTTCCTCCCAGG

GGCAGGTGTTTCTTTCCTGTTTGTCTTGTGTCTTGAGAGCTTGGCCTTATGACCAGTGAGAACTCTCTCC

CTGGTCTCTGCCAGCCCAAGCATCACTGCCCGAGGCGCCAGCTCAGTTTCACCGTCCACGTCCACAAGGG

GCTTTTCCCACCTTCACCTTTGTCGCTGGGTCAGTGCTGGAAAGCGCCCCTCACTCCTGCGCTGACAAGG

GCCCTTCTCTACTGTCTGTGGGGTGGTTCCGGGCTGGGGGGGCTGCCTCCTTTGCACCTGATTTTGAAGG

TGTCTCTTTCATCCATGGTTAAGTCATAAAAAGCTTATTGGTTTTGGTTTTGACTCACCTGAAAGTTTTT

TTGGTTTAAAAGAAGAATAGGCGGGGCACGGTGGCTCATGCCTGTAATCCCAGCACTTTGGGAGGCTGAG

GCAGGTGGATCACGAGGTCAGGAGATCGACACCATCCTGGCTAACACGGTGAAACCCCGTCTCTACTAAA

AAATACAAAAAATTAGCTGGGTGTGGTGGTGGGGGTGGGCGCCTGTAGTCCCAGCTACGTGGGAGGCTGA

GGCAGCAGACTGGTGTGAACCCGGGAGGTGGAGCTTGCAGTGAGCCGAGATCGCGCCACTGCACTCCAGC

CTGGGCGACAGAGCGAGACTCCATCTCAAAAAAAAAAAAAAAAAAAAGAAAGAAAGAAAAATAATTTTGG

GAGTTTCTGGAAAGGTACTAGGATTTCTCAAAAGGATTTGTCTTCTGCCTTGTGAAAGACAGATGTCAGA

CTAATCAGGCTTATCCGATGTGCTACATGAGATGGAAAGCGTGTGAAATAGTAAGTCACACTAAGTCTTC

TGGAGGTTCTATTTACGGGTTTGGTTTTGATATGAATGTTGCAGAAACTCTATAAAATTCCTAGAAATCT

GATACGTTATCCTATGATATATATCCTATCTCACATGCTATTCCTAGAAATCTGGTGCAATGTTATCAGT

CATAATTTTGGTTATTACATTAAAATGCTGCATGCCACAGAAATAACCAAGTTTCCTTGCCAACCGTGTC

ACGAATATAATAAACTCTCATCAGATCCTGA

>NM_001206836.2 Homo sapiens RAB11A, member RAS oncogene family (RAB11A), transcript variant 2, mRNA

AGTTGAAGCTCGGCGCTCGGGTTACCCCTGCAGCGACGCCCCCTGGTCCCACAGATACCACTGCTGCTCC

CGCCCTTTCGCTCCTCGGCCGCGCAATGGGCACCCGCGACGACGAGTACGACTACCTCTTTAAAGTTGTC

CTTATTGGAGATTCTGGTGTTGGAAAGAGTAATCTCCTGTCTCGATTTACTCGAAATGAGTTTAATCTGG

AAAGCAAGAGCACCATTGGAGTAGAGTTTGCAACAAGAAGCATCCAGGTTGATGGAAAAACAATAAAGGC

ACAGATATGGGACACAGCAGGGCAAGAGCGATATCGAGCTATAACATCAGCATATTATCGTGGAGCTGTA

GGTGCCTTATTGGTTTATGACATTGCTAAACATCTCACATATGAAAATGTAGAGCGATGGCTGAAAGAAC

TGAGAGATCATGCTGATAGTAACATTGTTATCATGCTTGTGGGCAATAAGAGTGATCTACGTCATCTCAG

GGCAGTTCCTACAGATGAAGCAAGAGCTTTTGCAGAAAAGAATGAAGCAAATGTCAGACAGACGCGAAAA

TGACATGTCTCCAAGCAACAATGTGGTTCCTATTCATGTTCCACCAACCACTGAAAACAAGCCAAAGGTG

CAGTGCTGTCAGAACATCTAAGGCATTTCTCTTCTCCCCTAGAAGGCTGTGTATAGTCCATTTCCCAGGT

CTGAGATTTAAATATATTTGTAATTCTTGTGTCACTTTTGTGTTTTATTACTTCATACTTATGAATTTTT

CCATGTCCTAAGTCTTTTGATTTTAGCTTTATAAAATCATCCACTTGTCCCGAATGACTGCAGCTTTTTT

TCATGCTATGGCTTCACTAGCCTTAGTTTAATAAACTGAATGTTTGGATTCCTCAGTTATTGTTTACTTT

TCATCATGGAAGCCTGTCACTGTATGTAGGACATAATAGAACTTGATCACTTGAAGCTCAGACCTATTGG

TCTTGATCAAATCAAACTAAGAAGACCTTAGAAATAAGCTACCATTTTGCCACAGAGCAGCTTATAGGTA

ATACACTCTTCTCTCAGTGCAGTGTACATTTCCACAAATCTAAGAATTGCCCTATAAACATAGCAGGATT

TTGAGAGCTTGAAAATTTTCCATTATTCTGGACATGAATTTCTAAAATGCCTTAATAGGTTTATGTAGTT

GAGTAAATTTTGTTTTTTAATTTTTGTAAGCATCAAAGTTGATTAGAGAGGGGGGCACTTTTTCTGGAGA

ATTCTCTTAGTAAACACAAAAGATTGTTACGGTTTCATTAGTAGTATGGTTGTGGGGCCATAAGTTAAAC

AGTGCTGCCTGGTAGGCTGGGAACTGAAGAGACTTGTGGTATTCCATCTCGGGTGCCTCTGTTGGCAATG

ATCAGGCAGCCCAAAAGATTTAAATGATCTATAATAATTTCCAAGCGGTAGATTATGTGGCATTTTATTG

CTCAGGCAATAATTGGTTTAATGCTGGTAGTGTCAAATTTTGAAGTGTTAATTTTGTCTTAGAACCTTCC

AGTAAGTGAAATACAACCTAGTTTTATCACCATATCCACCAGCAGGCATGGATAATTATTTTAACAATGC

TAATATTTGAGTTTTGCAGTATATTATAGAATATAGTCCAGTTAAATCTTTGGTTTCAGTATGTCTGAAG

AGTACAGTGAGAGGTTAATTTCTGCTCAAGTGGTACCACTTAAAGGCATGTATTCTTTTAGTATGTAAAA

TGAAATAGTACCTTGAGTTTAAATAGAATGCATTTAGGCATTGTAGAGATCTGAAATAGTTTTCTTCCAC

TACATTGTTGAAATCAATGAAGCAATTAGTTTCTCATTCAGAAATGTGCACACTAATATTTAGTTTTGCT

TTCTCGTGGATAATATTAAGCACTTACTCTGCAGTTTCCTGGAAGTTGTGTCAACTGCAGTGATACTATT

CAGGATGGTGGGAAATCCCCAAAAATATGTATGTGTGGGCTTGCTTAGATTACTATATTTCATAGTTAAT

CTTTTGTCTCTTGCGGTGCTCATGATGTGTGGGGCACACGGAAGGCATTGCTGTAGTCAGTCATTTTGGT

TTTCTTCTATAGCCATTTTATTATTTTAGTGTATTAGTTATGAAGATAATATTATCTATTTGTAAATTGC

TACTTTGTATTTTATGCATGCTCTGTAATTTGATTTTTTTTTAGTTATTGATTTGGATTATATTCACATT

CTAATAAACAGTTATAGGGGGATTATTTCTTATGCTGTCAGATTACATTTTTCCTTTGAGTGCTTTGGGT

GCAGCCGTGGAATCCTGATGTAAAAGCATAGGTTCTTGCATTACTGAGTAAACATTGAATTGGGAGCATC

AGTGTGTGAATTCAGCTTTGATTTTAGAACTGCAGTTCAATTATGCTTTCCTTGGAAAAAGTATGTCCAA

AAACATTAGGAAAGTAGTGTGTATTTTCATATATAACAGTGTCACCAGACCCAGGAAAAGAAACCATCTC

TATTTTATTGAAAGTTGGTGGTAGATTTTTTACAAAGTAAGGAGAAAAGAGAAAAACTAAATAGTGAATT

GAAAATGAATAAAGTAAACTTAGAATTTTTACCCAAGTGTAGACTTAAATGTTGCTTTGAGTATGGCTGG

ATCTTGGGAGAATTCCAGGAAATATTAGGTATTAGAAAATGACTTTGATTGCATTTCAGCAGGTGTCTTT

ATTCTGAGTAGTATCTTAGGAGACAAACTGTCTCAAGCAAGAAACAATAAATTTTTAAATATTATGACAG

TTCAGGGCTTAAGGCAGTTTAGAAGTATTTTGCTACTTGACCTTGATTTTCAGTTATTTTAACTTTGAAT

TTTTTTTTTTTTTTTTGAGACGGAGTCTTGCTCTGTTGCCCAGGCTGGAGTACAATGGCACCATCTCAGC

TCACTGCAAGCTCCACCTCCAGGGTTCATGCCATTCTCCTGCCTCAGCCTCCCGAGTAGCTGGGATTATA

GGCGCCAGCCACCACGACTGGCTAATTTTTTGTATTTTTAGTAGAGACGGGGTTTCACCATGTTAGCCAG

GGTGGTCTCGATCTCCTGACCTGGTGATACACCCACCTCGGCCTCCCAAAGTGCTGGGATTAAAGGCGTG

AGCCACCGTGCCTGGCCTTGAATTTTTTTTTAACCTGTTATATAGTCTCCTGAATAGTTAGCCATGGTGC

CTTGATTTAATCTTATGTCATTGTTCTCAAATGCCATGATTTCTCTTTAATAGTAGTTGTCATTATGTAA

GGGTAAGTTATTGCCCATTTAATTGTAATTATAAGAAGAGGTGCCAGCTAGTGACCTTACATCAGTGATC

TTTTAAGTTCTGAAGGGTTCTGCAGAAAAGTTACACTTCTTGAGTTTGGGTTGCCCATCAGAGCTGGTCA

CATTTCTTTGATCATTGAAAAAATGAATATCTTAATTTTTGTCTTTGCTGACTGGTATTTATAAAATACA

AATGCTTTTAGTGGGTTGAGCTGTTGTGATAATCTCTTCCATCTACCTTAGAGAAGAAGCTGTACTATTT

AGACAGTTTTGGCCAACACTAGAAGCAAAAGAATTCAAAGGCTGGGTGGATATTTAAGAACTTATTCTAA

CCTGTCAGGTTTCAATTTGTGGGGCTTGGTTTAACAGAGGAAAAAAATATAAATTGATTTTCTGATGTCT

CACAAATATCACATACCTGGAAAAAAATTAAAACAAAAAATGAATTTTGTAAAAGGTTATGTATGACTTC

CATTTGCTCTGTAAAAAAATGAACAAAGATTGTGAAAGGATCCTCTTCCACCACATCATTTAATGGGTTA

GCGGAACAGATAGGGAGCTATGACACTAGCACCAAAGAGAGATTTTTCTTCTGGTTGGGTGGTGCTGGTG

CCACGCCCCTTTCTGGGATTTGACAAGTCTTTCCCACTCACCTATACAATATGCCAAATCAACTGGAGTT

GTCATTCTAATGTTTTAAAGAATGATTGGGTTCCCCCAAACTGTAGGAGGTCGATGGTAACAATAAAGTG

ATACAAAATGCTTATGTCATTATAATTTATGTAAGGGAGACCATTAGGCTGAGAATCAATATGATTGAGG

TTATCTATTCTCCTGAAAAGTGAATTGGAGAATAAAGAAAGAATATTTGAAAGGATGTTGTGTACTAGGC

AATATACTAGCATGTATTAGAAAATACGGTAAACTGTGGGTTAGAAGAGTGAAAGTAGCTGGTATGATAG

AGCACTGGACTGAGTAACGAAGAGATTCTAGTCATGGCTCTTGTGGTCATTTCATTTTTGAGCCTCACAT

TCTTCCAATTAATGAGGGTACCAGATTAAATAAACTCTGGGGATTCATCCCAATTCGAACTCTGTAATTC

TCTGAATGAACTCTCTCTTCAATTAAAGCCAAATTATTAACATTTTAACTCAAGTTTAAATTTAATAGAG

AAGTCAAAATGAGGTATGCACTTTGTGGAATGTTTTTGGTCTTATCACAATAACCTGATTAAAATGGTGC

CTTTATTTCCATGTTTACTCCTTTAAATAAGATGAAAGCTTTCAGAACATTTATTTGCAAAATGACTGTC

AATATCAACAATATGCATCAAAGTACCATATATGCTAAGGGAGAGGAGCACTTGATGGGCTGCTATGTTA

ACTGTAAATTGAGAACTGCTTGTTTTATGGCTTTTGCTGTTAAAAAAAAACTGCTGGCTTTTAATATGTA

CTTTAAAATATACTCATGTCTGGGAGAGAACTTTTAAAAA

>NM_004161.5 Homo sapiens RAB1A, member RAS oncogene family (RAB1A), transcript variant 1, mRNA

ATTTTGGGTGGAAGCGATAGCTGAGTGGCGGCGGCTGCTGATTGTGTTCTAGGGGACGGAGTAGGGGAAG

ACGTTTGCTCTCCCGGAACAGCCTATCTCATTCCTTTCTTTCGATTACCCGTGGCGCGGAGAGTCAGGGC

GGCGGCTGCGGCAGCAAGGGCGGCGGTGGCGGCGGCGGCAGCTGCAGTGACATGTCCAGCATGAATCCCG

AATATGATTATTTATTCAAGTTACTTCTGATTGGCGACTCAGGGGTTGGAAAGTCTTGCCTTCTTCTTAG

GTTTGCAGATGATACATATACAGAAAGCTACATCAGCACAATTGGTGTGGATTTCAAAATAAGAACTATA

GAGTTAGACGGGAAAACAATCAAGCTTCAAATATGGGACACAGCAGGCCAGGAAAGATTTCGAACAATCA

CCTCCAGTTATTACAGAGGAGCCCATGGCATCATAGTTGTGTATGATGTGACAGATCAGGAGTCCTTCAA

TAATGTTAAACAGTGGCTGCAGGAAATAGATCGTTATGCCAGTGAAAATGTCAACAAATTGTTGGTAGGG

AACAAATGTGATCTGACCACAAAGAAAGTAGTAGACTACACAACAGCGAAGGAATTTGCTGATTCCCTTG

GAATTCCGTTTTTGGAAACCAGTGCTAAGAATGCAACGAATGTAGAACAGTCTTTCATGACGATGGCAGC

TGAGATTAAAAAGCGAATGGGTCCCGGAGCAACAGCTGGTGGTGCTGAGAAGTCCAATGTTAAAATTCAG

AGCACTCCAGTCAAGCAGTCAGGTGGAGGTTGCTGCTAAAATTTGCCTCCATCCTTTTCTCACAGCAATG

AATTTGCAATCTGAACCCAAGTGAAAAAACAAAATTGCCTGAATTGTACTGTATGTAGCTGCACTACAAC

AGATTCTTACCGTCTCCACAAAGGTCAGAGATTGTAAATGGTCAATACTGACTTTTTTTTTATTCCCTTG

ACTCAAGACAGCTAACTTCATTTTCAGAACTGTTTTAAACCTTTGTGTGCTGGTTTATAAAATAATGTGT

GTAATCCTTGTTGCTTTCCTGATACCAGACTGTTTCCCGTGGTTGGTTAGAATATATTTTGTTTTGATGT

TTATATTGGCATGTTTAGATGTCAGGTTTAGTCTTCTGAAGATGAAGTTCAGCCATTTTGTATCAAACAG

CACAAGCAGTGTCTGTCACTTTCCATGCATAAAGTTTAGTGAGATGTTATATGTAAGATCTGATTTGCTA

GTTCTTCCTTGTAGAGTTATAAATGGAAAGATTACACTATCTGATTAATAGTTTCTTCATACTCTGCATA

TAATTTGTGGCTGCAGAATATTGTAATTTGTTGCACACTATGTAACAAAACAACTGAAGATATGTTTAAT

AAATATTGTACTTATTGGAAGTAATATCAAACTGTATGGTGATAAGTATTGTTTTGATTCTTATGGTTAA

AGGGAAATAGAGCCTTGCATTATATTCAACACAGCCATTTGTGTGTGCACAATGCAAACTAAGGTATTCT

AGACCTATCTTAGAGCAGCATCCAGTATTTGCTTTCTAGATAATATGCCCAATAACATGACCTAGAGGGG

CTTCTGTGCTGTGTAGGGATTTAACCAACTTCAGTGGTTCAGGGAGCTCAAACTATATGTAAAACAAGTT

TAGAATGTATGCTATCTAGCCCGTTATCTCTGATCCTTCTCTAAAACCATTTGAAATAGCTTCATTGATC

AACATTTCATAAATGCATCTGTGGTAGAGGTAGAAAGCAGCACCTTTCCTAATTGGCAAATGATCAGACT

AATGTGTGCTAATGTTTTTCTTCCATGCTTTCAGTCAGATTCAACTATTTTATCCTCCACAGTTGCTTAA

CTTGGTGTTGGAGGAGGGTTTAAGCATTAAGATAGGAAGCAGGAAATTTGATTGCTCTAAATTTAGAAAT

TATATCCCTAAAAATTAAAACATGAATACTGGGTGGTAATGATAATTGAGGCAAATGTATTTATTTTGGT

GACATTTTGCATATATGAAGATTTTCTGAAATAGGACCTTCAAGATCCTAGGGGGTTTTGTTTGGTTTTT

AATTGTGAGGAATAAAAAATCTTCTGCCCACACTGGCATTTTAAGGTGACTGAGGTCAAACGTTGTTTCC

TTAGGTTGAAATAGCAGCCAAAACATTCTTCACGCAGGGGCTTGGGATATGGCTGCTGGCAACACATTTT

GTTGTGGGCTCCTTAATTTAATGATAAAATTTAAGCTAAACACAAGCCAAAAATGAATAGGTTTTTTTAA

TTTTTATTTTTCACTAAACAGGCAATTGAAATACATGGTACAAAAATAAGTGGTAAGATAATTGTAAAAT

GAAATGGACAGAATATTCAATTTTCCATCTATGAAAATTTCACAATAAAAATCATAGTTTACTTTGTA

>NM_130781.4 Homo sapiens RAB24, member RAS oncogene family (RAB24), transcript variant 2, mRNA

CTCTAGCCCCCTCCCGCGGGAGTCGCGGCGCTGCGGGTAGGAGCCGGGTTGCGGGAGACCCCAGGTTCGG

TTGGGATTCCCAGCCAGAACGGAGCTTAAGCCGGGCAGGCGAGCGAATGACGGAGTAGCGAGCTGCACGG

CGGCGTGCTGCGCTGTTGAGGACGCTGTCCCGCGCGCTCCCAGGCCGCCCCGAGGCTTGGGGTCTTCGAA

GGATAATCGGCGCCCGGGGCCGAACAGCGGGGGCACACGGGGCGCTGCCGAAGTGCAAGGCCACGGCCAG

AGCTCGAGCCCGACGCGCTGTCTGGAGTCGTAGGACCCTGACGTGGCTGAAGCGGCCCCGGGAGCATGAG

CGGGCAGCGCGTGGACGTCAAGGTGGTGATGCTGGGCAAGGAGTACGTGGGCAAGACTAGCCTGGTGGAG

CGCTACGTGCACGACCGCTTTCTGGTGGGGCCTTATCAGAACACCATCGGGGCCGCCTTCGTGGCCAAGG

TGATGTCGGTCGGAGACCGGACTGTGACATTAGGTATTTGGGACACAGCAGGCTCTGAGCGCTATGAGGC

CATGAGTAGAATCTACTATCGGGGTGCCAAGGCTGCCATCGTCTGCTATGACCTCACAGACAGCAGCAGC

TTTGAGCGAGCAAAGTTCTGGGTGAAGGAACTGCGCAGCCTAGAGGAGGGCTGCCAAATCTACTTATGTG

GCACCAAGAGTGACCTGCTGGAAGAAGACCGGAGGCGTCGACGTGTGGACTTCCACGACGTCCAGGACTA

TGCAGACAATATCAAAGCTCAGCTCTTTGAAACATCCAGCAAGACAGGCCAGAGTGTGGACGAGCTCTTC

CAGAAAGTGGCAGAGGATTACGTCAGTGTGGCTGCCTTCCAGGTGATGACAGAGGACAAGGGCGTGGATC

TGGGCCAGAAGCCAAACCCCTACTTCTACAGCTGTTGTCATCACTGAGTCAGCACTCACCTGGCCTGGGG

GAATTAAAGGAATTCCCCGTAAGGGCTGGACCCAGCTCCTTTCTGGGCTTGGGTAGTCAAATGTCTGAGC

TACCCCAGGTCCTCATGTCAGCAGAGTGGCGCCTGCCTGTGCTGGCCCATGGAACGGAGACAGCATTGGG

CTGACTGTGGGCATGAGGAGGGATAAGGCTGATTTGGACCCCAGGCTTCTGCCCTGGACAGCACTTGTGT

CTGCAGATTATTTAAGTGGCTTTTGATCTGTAAATAAAATCAGTGCACTGTGCATCACACCCAGCCCCTT

TCCCTGCTGTGTGGATTAGGTGTCAAGACACCTAGTTCTTCCTGGGGCCACCCGGCTGGCCTCACTGCTT

ATATTAAGGCTCCTCCCAACTCTCATTTTCCTTTGGAAAACAAGACTTTTTTCCCCATGGTTACCGCTGA

GATACTGGGGCTGTAGTAGTATAAAAGCTCACAGTTCCTTCTGAGTGCTGAAAAGAGTGCATGAGTTGCT

TCGAAATAAAAGGGTCAAGCATTCCTACCTGA

>NM_031296.3 Homo sapiens RAB33B, member RAS oncogene family (RAB33B), mRNA

GCTCTTGCGGTGGCGTAATCTCTCAGCCTTTCTGTGTCTCCTTTCCTCCGCCTCAGTTTGGGGCGGGTCG

GGGGAATGGCTGAGGAGATGGAGTCGTCGCTCGAGGCAAGCTTTTCGTCCAGCGGGGCAGTGTCAGGGGC

CTCAGGGTTTTTGCCTCCTGCCCGCTCCCGCATCTTCAAGATAATCGTGATCGGCGACTCCAATGTGGGC

AAGACATGCCTGACCTACCGCTTCTGCGCTGGCCGCTTCCCCGACCGCACCGAGGCCACGATAGGGGTGG

ATTTCCGAGAACGAGCGGTGGAGATTGATGGGGAGCGCATCAAGATCCAGCTATGGGACACAGCAGGACA

AGAACGATTCAGAAAGAGCATGGTTCAGCACTACTACAGAAATGTACATGCTGTTGTCTTCGTGTATGAT

ATGACCAACATGGCTAGTTTTCATAGCCTACCATCTTGGATAGAAGAATGCAAACAACATTTGCTAGCCA

ATGATATACCACGGATTCTTGTTGGAAATAAATGTGACTTGAGAAGTGCCATACAGGTACCCACAGACTT

GGCACAAAAATTTGCTGACACACACAGTATGCCTTTGTTTGAAACGTCTGCTAAAAACCCCAATGATAAT

GACCATGTGGAAGCTATATTTATGACCTTGGCTCATAAGCTTAAGAGCCACAAACCATTAATGCTTAGTC

AGCCCCCTGATAATGGAATTATCCTGAAGCCTGAACCAAAGCCTGCAATGACGTGCTGGTGCTAAATAAC

AGTCTTTATTATATTATCTAATTTTGACTAAAGAAATACTTTTGAAGTATGACAGTATTAAGTCATAAGA

TTTAATCTCAACTATAATGGGTCATCTTGACACTTTGCTGTTTGTCATTGTCACGCTTTTGTATTTTGTA

TCTACTTAAGTTTGTCACTGTGACAACACAGGAAAAGTTGGTTTTCAGGTGAGATTGAAAATGAAGCAAA

GATAGGATGAATCTGAACATCTCTCCATCTAGAGCCCAATGAAGGAAGCTTCAAATGAGAACATGATGGA

ATCAGTAACCATTCAATCTTTTGTCCTAGGATTGGAAAAAAATGTTAAAGGTTTAGGACACACCTAATAG

TATGTCCTTTGAATGGGAAGTGTTCTTAATAGGATAAAAACTGGTATTTGCCTCTCCCCAGAGTACTTTT

TTTGTTTTTTCATAGAGACGGGGTCTTGCTATGTTGTCCAGGCTGGCCTTGAGCTCCTGGGCTCAAGCAA

TCCTCCCACCTCAGCCTCCCCAGTACTGGGACTGTAGGTACTCACCACTGCATCTGGCCTTTCTTTGTTT

TATTAACATATTTAGTTTTGTTATTATTGGTATGTTTTAGAGCCAAGACTTTAGTTCCAGTGGGATAAGA

AGGCATAGAATGTTTTCTGGTTCCCAGTCCATAAAGAATGACTTTTCCAAGAGTTCTAGATGTTTGATTT

TCTAATTAATACTTATCAGATCTACAAAAATCATTATTATTTTAAAAGAGTTATTTGAGTTTCTTTCTTT

CTTTTTTTTTTTTTTTTTTTTTTGAGATGGAGTCTCGCTCTGTCTCCAGGCTGGAGTGCAGTGGCGTCATC

TCGGCTCACTGCAACCTCCGCCTCCCAGGTTCAAGTGATTCTCCTGCCTCAGCCTCCCGAGTAGCTGGGA

TTACAGGCGTGCACTACCACACCCAGCTGATTTTTGTATTTTTAGTATAGATGGGGTTTCACCATGTTGG

CCAGGATGGTCTCGATCTCTTGACCTCATGATCCACCCGCCTCAGCTTCCCAAAGTGCTGGGATTACAGG

TGTGAGTCACTGCACCCAGCCGAGTTGCTTTCTTACTAAATCCTATTAAAATATGCAAAAATAAGTCAGA

TTTTAAGGCAAATAAAGTGACATAAGGTGCTTTATATTTTATTTTGGTATATTTAAACAGTGAAAAACTA

ACTGAAAGCACATGAAGAGTTGTAACTTGGGGGAAAATAGGTAAACATAGCTTCTAGCTAACACAGGAGA

CCTATTCTTAGCCTTTACTAATTTCAAGCAGTGTATCCCATATGGTATCTCTTGCTCTTCCTTCAACTCC

AATAAATTTAATGACTAAATGCCAAGTTAACAAATCAACTTCCATTTGGATTGTAGGTGTGAAGGCACAA

CTCTAATTGCTATTAGTCTACATGTATTTCTGTAATAGTATTGTGTCATATCAATTTTTAAGATGTCTAA

ATTTTATGGTCACAAGTTATCCCTCCTCAGTATGAAAAATAAATTAGATATTGAAAAATGTCTAAACTTC

AGTGATGGAAAGAATATTTCAAGAAGTTTTTTAACCTAAATACTTTTATTTTGAATTTAAGTCTTTGCAC

ATAAAATATAGCAAGCTTACATATTAAACTATTTACGTAAATGGAATGTAAGCCATGACTTTAACTGAAG

TGTTCACATTCACTAATTTTGATAGATTGCTGTCCTTAATAATTTTGGAGGAAATTAAGCCAAATGATTA

TTGTACTACAGTATTTTCAGAATATGGGAAATCAATTAAAAATGTAATCTAATCTAGTTTAAGATTTTTG

TTTAATCATCATGGTGGTCCTACCTGGATAATTTAACTATAAAGACAAAGTAATTCTATTAAATGAACTA

ACTGAAAATAATAATTATAGGAAGTGATTATTCCATTTTAAGTATTAGAGCTCAAATTGGCTTTATTTGC

ATTTAGGGAGATCATGTTTTCTTAATCATGCTGGAATTTAAAAATTGTTTTACTTGTATCGAAATTAACC

TTGATTTATAACTATTTTTGTAATAAAACAATGACAGCTGTAGTAACTATGATGGGTGTAACAACATTTT

TTTAAAGAAGGGAATCTGTTTATCGCTTTTCAAAATATTTTCTAAAGTGGGGGAAGAAAGTTTATAGACT

TTCCAAGCACATTTATGGTTTTTTATTACTATTATTATGGTTTTAAAAAGAGTAACTTTATTTCTTTTTG

TAAGGAATTAAGTAATATCCTTTACAGTTCTGTGAAAGGACTTATTTTTTAACTGTAATATTTATTAGTT

TTAAAATATTTGTATCTCATTTGTAACAATTTGTTTTAATTTTTTATATATATGTTTTTATTTTTAAAAA

ACATACCAGTTGAATGGGGTTAAAGCTTTCAATATCTTAAAATATTTATAAAACATTTCACTGTTGCAAA

ATCACTTCCAAAATGATAGCTATCTAACAACTAATTACTAATTTTTAAAGAACAAATCACACATTTAAAA

AATCTGTAGAATTTATTTTAACTATGACCTTTAATTGAAAATAAATAATTAAAATATCAGACATGTTTTG

GAAAAGTCTTAATTTGAGAACACCAAAGGAAACTACCCCAGAATCTAATGTAGTTCGCTATTAATAACAA

TGCATTATTGAAAGTATATTGCAAATACATGTTTCCTCATGAAATCTAAGTAATTTTGTTGTGGAATAGT

GTCACTGTTACATTTCCCCCATGAAGTTCAATAAACCAGCTTAGCCACAAAGAAATTGTGTTTTATTTTC

TTTGTTTGGTTACTGGGTAGAGTATAAAATAAAACAATCTTTTTTTCCTCCTGGTTTCCAGATAATATTA

TATATATGATACACTTTTGAAAACAACAAAAAGCCCCCAACCCATTGGACAATTATTTTCTTGTATACAT

TTGAGGTCAAAGAACACTTCAGGAAAAAAACTCAACAAAGTATTAAAATTAGCTGATAACACAGCTTTTG

TATTTGTGTGTTTTAGTAATCCAGCTTGTTTTAGAATAGTTCAGTGCAAAAAGCAAATGGTGCAAGAGGA

GTTGATGTGTATTTAGACGTAAAAGCATTTAACAGAATGAATAACTATAGAAAGTATGAGAAAAGAGAAC

GAACTACGTCTCAGGTATAAGATTGCAGTTACTGATTTAGACACCATAGGATTAGGGATGTGGGGGCCTC

TGATGTTGATTCTGCTTCCCCTGGTCAGTTTATTTTACGTATAAGTTACCCTTGTGTTCAAACACAAAAT

TGACATTATTCATGAAGGACTGCTTTCTTAATCAGATATTAAGAAGTTCGTCATGTATTTTGGGAGCACG

TTTGCTACAGCCTGTGTGGCAAGGGCGAATGCACTTGGTTTGTTGGAATAACCTGATTTATCAAACATTT

ATCAAGTATGGGTCATGTGTTCTCTATTTGTGTTAGTGGAGCTACATA

>NM_004162.5 Homo sapiens RAB5A, member RAS oncogene family (RAB5A), transcript variant 1, mRNA

AGTTCGCTGCGTGCAGCGACGTGGCGGCGGGGCCGGCACCGGGCAGCGGAAGTGGCTCCGGCGGTGGGAC

TTGAGTGTTTGTGTTTTGGTTCGTGAAGGAGCCGGCGGCTGGCCTTAGGGGAGGAGGCAGAGGGAGGAGG

AGGAGGAAGAATTAGTCGGAACTCCAGCGCCGGCGGCGGCGGCGGCGGCGGAGGAGGAGAAAGGAAAGAG

GAAGGGGGAGCGGCGAGAGGCGGAGACGGAGCCCGACAGGGGCGGCACCACGGCACGAGCCCCGCACAGT

CCAGTGTGAGGGGGAGCGGCGCTAAGAGCAGGCGACGCCGCCGCCGCCACCACCACCGCCATAGATACACT

CTCATCCTACGGGCCACGCCTGGGCCTTGCTGCCAGGAAGCTTCGGCCCCGCAGCTCGGCTTGCTGCGGT

CTCAGGTTTCTTTACCTCCAGAAAGAAGAATATTGGCCCCTTGAATTCTGGAAGTTCATTGAAGAGTCTG

AAATTAGGGACTTATTTCAAATTTGGACATGGCTAGTCGAGGCGCAACAAGACCCAACGGGCCAAATACG

GGAAATAAAATATGCCAGTTCAAACTAGTACTTCTGGGAGAGTCCGCTGTTGGCAAATCAAGCCTAGTGC

TTCGTTTTGTGAAAGGCCAATTTCATGAATTTCAAGAGAGTACCATTGGGGCTGCTTTTCTAACCCAAAC

TGTATGTCTTGATGACACTACAGTAAAGTTTGAAATATGGGATACAGCTGGTCAAGAACGATACCATAGC

CTAGCACCAATGTACTACAGAGGAGCACAAGCAGCCATAGTTGTATATGATATCACAAATGAGGAGTCCT

TTGCAAGAGCAAAAAATTGGGTTAAAGAACTTCAGAGGCAAGCAAGTCCTAACATTGTAATAGCTTTATC

GGGAAACAAGGCCGACCTAGCAAATAAAAGAGCAGTAGATTTCCAGGAAGCACAGTCCTATGCAGATGAC

AATAGTTTATTATTCATGGAGACATCCGCTAAAACATCAATGAATGTAAATGAAATATTCATGGCAATAG

CTAAAAAATTGCCAAAGAATGAACCACAAAATCCAGGAGCAAATTCTGCCAGAGGAAGAGGAGTAGACCT

TACCGAACCCACACAACCAACCAGGAATCAGTGTTGTAGTAACTAAACCTCTAGTTTGAACTAGCTGGAA

TAGTCTTCTGCTTCCTAAATGTTAATAACAATGGAATTGGAGCATTTAACCAGCCCAGTATGACTTCCAA

AAGAAGAGACTTATGATAGAGTCAAGTTTCTAATACAGAATTATTTTAAGTGTTTTGAACTTAATTTTTA

ATAACATGCATGGGTCCCTCTCACTAATGTTTCAACAATAGGGAAAAATGAGAACTATGTGGACACTTGT

TTCATTGGAAGGTTAGGGGGAATAATTTCTCATCACTAGGAATATAGACAAATGACTGTCTGGGCCCACA

CAGTTAACCAGCCCATTTCTCCACACTGGTACAGTAGTCACCTGTGAAAAAAAAATTGGAACTTACTAAT

TTGGGCTTTTCAAAAACATTCTTTGTTTAGAAGGAGATTCTAAAGTTATTTATGATGCTTAGCCATAGTA

TTCAGGCAAATGTTCATTTCTCCTGGTACCTGTATTTAAAATGTACATTCCACATTTTAATAAATTAACC

ACAAGAAAATAATCCCACATATACAAGGTCAGGGGTGGGGAAGAGTATTAATGGTATCTTAATTATACCC

AGTCTGGTTTTTTTTTTTTAAATGGGGTAAAAATCAAATGCAACCCCATCTTGTTTTAGGAATTTTGAGA

ACTAATAAATGCACCTTAATGGTCAGTGTTCCTTTCAAACATGTGAGTTCTTTAACAAAAATGAAATAAA

CCAGGTGTCTGTGATTTCTAATTAATCACCGCTGGCCATTACACAGGTTTTGTTGTTTGGGGTGGGGAGG

GGGCTTTTGTTCCCTTTTGACATAATATAGTCAATGCACTAACAATTATGTATATTCAAACTTGATTATT

TTAAATTCGATCTTCAGCTGTACTGTAAATAGGGTACTGCATTGTAGTCTCCATATCTGTATTACTTTTC

TGTAATATTTAAGAGTTGCTTAAAAGCATACAAAATGTACTGTTACTAAAACAGCTAATTATTTCTCTCT

CCCCCTTTGACAGGAAGGGGCTTCAGTTGTTCCTCCATGGCTAGAACCATAATAAACAATGTACCCGTAA

TTTGTAACATAAAGTATTGCAATATGTTAGTAACAATCTTGCAGCCTTCCTTTCCAAAGTTCATTTTATT

TTGATCAGTTCAGTATATTGCACTAATTATTTTAGGTATTTTCATTATATGAAAGCTACCATGTGTCAGA

GATGATTTAATCTATTTAAGTGTTGGACTGCTAGGAGAACTTGTACATTTATGATAATGCAGAATTAGGA

AAACGGTTCACCAGTGTTTAGTTTTATATTGAGGTGCTCAGGTTGGAATAAAGTGGTATAAAAAGCAA

>NM_004637.6 Homo sapiens RAB7A, member RAS oncogene family (RAB7A), mRNA

AGTCTTGGCCATAAAGCCTGAGGCGGCGGCAGCGGCGGAGTTGGCGGCTTGGAGAGCTCGGGAGAGTTCC

CTGGAACCAGAACTTGGACCTTCTCGCTTCTGTCCTCCGTTTAGTCTCCTCCTCGGCGGGAGCCCTCGCG

ACGCGCCCGGCCCGGAGCCCCCAGCGCAGCGGCCGCGTTTGAAGGATGACCTCTAGGAAGAAAGTGTTGC

TGAAGGTTATCATCCTGGGAGATTCTGGAGTCGGGAAGACATCACTCATGAACCAGTATGTGAATAAGAA

ATTCAGCAATCAGTACAAAGCCACAATAGGAGCTGACTTTCTGACCAAGGAGGTGATGGTGGATGACAGG

CTAGTCACAATGCAGATATGGGACACAGCAGGACAGGAACGGTTCCAGTCTCTCGGTGTGGCCTTCTACA

GAGGTGCAGACTGCTGCGTTCTGGTATTTGATGTGACTGCCCCCAACACATTCAAAACCCTAGATAGCTG

GAGAGATGAGTTTCTCATCCAGGCCAGTCCCCGAGATCCTGAAAACTTCCCATTTGTTGTGTTGGGAAAC

AAGATTGACCTCGAAAACAGACAAGTGGCCACAAAGCGGGCACAGGCCTGGTGCTACAGCAAAAACAACA

TTCCCTACTTTGAGACCAGTGCCAAGGAGGCCATCAACGTGGAGCAGGCGTTCCAGACGATTGCACGGAA

TGCACTTAAGCAGGAAACGGAGGTGGAGCTGTACAACGAATTTCCTGAACCTATCAAACTGGACAAGAAT

GACCGGGCCAAGGCCTCGGCAGAAAGCTGCAGTTGCTGAGGGGGCAGTGAGAGTTGAGCACAGAGTCCTT

CACAAACCAAGAACACACGTAGGCCTTCAACACAATTCCCCTCTCCTCTTCCAAACAAAACATACATTGA

TCTCTCACATCCAGCTGCCAAAAGAAAACCCCATCAAACACAGTTACACCCCACATATCTCTCACACACA

CACACACACGCACACACACACACACAGATCTGACGTAATCAAACTCCAGCCCTTGCCCGTGATGGCTCCT

TGGGGTCTGCCTGCCCACCCACATGAGCCCGCGAGTATGGCAGCAGGACAAGCCAGCGGTGGAAGTCATT

CTGATATGGAGTTGGCATTGGAAGCTTATTCTTTTTGTTCACTGGAGAGAGAGAGAACTGTTTACAGTTA

ATCTGTGTCTAATTATCTGATTTTTTTTATTGGTCTTGTGGTCTTTTTACCCCCCCTTTCCCCTCCCTCC

TTGAAGGCTACCCCTTGGGAAGGCTGGTGCCCCATGCCCCATTACAGGCTCACACCCAGTCTGATCAGGC

TGAGTTTTGTATGTATCTATCTGTTAATGCTTGTTACTTTTAACTAATCAGATCTTTTTACAGTATCCAT

TTATTATGTAATGCTTCTTAGAAAAGAATCTTATAGTACATGTTAATATATGCAACCAATTAAAATGTAT

AAATTAGTGTAAGAAATTCTTGGATTATGTGTTTAAGTCCTGTAATGCAGGCCTGTAAGGTGGAGGGTTG

AACCCTGTTTGGATTGCAGAGTGTTACTCAGAATTGGGAAATCCAGCTAGCGGCAGTATTCTGTACAGTA

GACACAAGAATTATGTACGCCTTTTATCAAAGACTTAAGAGCCAAAAAGCTTTTCATCTCTCCAGGGGGA

AAACTGTCTAGTTCCCTTCTGTGTCTAAATTTTCCAAAACGTTGATTTGCATAATACAGTGGTATGTGCA

ATGGATAAATTGCCGTTATTTCAAAAATTAAAATTCTCATTTTCTTTCTTTTTTTTCCCCCCTGCTCCAC

ACTTCAAAACTCCCGTTAGATCAGCATTCTACTACAAGAGTGAAAGGAAAACCCTAACAGATCTGTCCTA

GTGATTTTACCTTTGTTCTAGAAGGCGCTCCTTTCAGGGTTGTGGTATTCTTAGGTTAGCGGAGCTTTTT

CCTCTTTTCCCCACCCATCTCCCCAATATTGCCCATTATTAATTAACCTCTTTCTTTGGTTGGAACCCTG

GCAGTTCTGCTCCCTTCCTAGGATCTGCCCCTGCATTGTAGCTTGCTTAACGGAGCACTTCTCCTTTTTC

CAAAGGTCTACATTCTAGGGTGTGGGCTGAGTTCTTCTGTAAAGAGATGAACGCAATGCCAATAAAATTG

AACAAGAACAATGAT

>NM_018890.4 Homo sapiens Rac family small GTPase 1 (RAC1), transcript variant Rac1b, mRNA

CTGTTTCTCTGCAGTTTTCCTCAGCTTTGGGTGGTGGCCGCTGCCGGGCATCGGCTTCCAGTCCGCGGAG

GGCGAGGCGGCGTGGACAGCGGCCCCGGCACCCAGCGCCCCGCCGCCCGCAAGCCGCGCGCCCGTCCGCC

GCGCCCCGAGCCCGCCGCTTCCTATCTCAGCGCCCTGCCGCCGCCGCCGCGGCCCAGCGAGCGGCCCTGA

TGCAGGCCATCAAGTGTGTGGTGGTGGGAGACGGAGCTGTAGGTAAAACTTGCCTACTGATCAGTTACAC

AACCAATGCATTTCCTGGAGAATATATCCCTACTGTCTTTGACAATTATTCTGCCAATGTTATGGTAGAT

GGAAAACCGGTGAATCTGGGCTTATGGGATACAGCTGGACAAGAAGATTATGACAGATTACGCCCCCTAT

CCTATCCGCAAACAGTTGGAGAAACGTACGGTAAGGATATAACCTCCCGGGGCAAAGACAAGCCGATTGC

CGATGTGTTCTTAATTTGCTTTTCCCTTGTGAGTCCTGCATCATTTGAAAATGTCCGTGCAAAGTGGTAT

CCTGAGGTGCGGCACCACTGTCCCAACACTCCCATCATCCTAGTGGGAACTAAACTTGATCTTAGGGATG

ATAAAGACACGATCGAGAAACTGAAGGAGAAGAAGCTGACTCCCATCACCTATCCGCAGGGTCTAGCCAT

GGCTAAGGAGATTGGTGCTGTAAAATACCTGGAGTGCTCGGCGCTCACACAGCGAGGCCTCAAGACAGTG

TTTGACGAAGCGATCCGAGCAGTCCTCTGCCCGCCTCCCGTGAAGAAGAGGAAGAGAAAATGCCTGCTGT

TGTAAATGTCTCAGCCCCTCGTTCTTGGTCCTGTCCCTTGGAACCTTTGTACGCTTTGCTCAAAAAAAAA

CAAAAAAAAAAAACAAAAAAAAAAAACAACGGTGGAGCCTTCGCACTCAATGCCAACTTTTTGTTACAGA

TTAATTTTTCCATAAAACCATTTTTTGAACCAATCAGTAATTTTAAGGTTTTGTTTGTTCTAAATGTAAG

AGTTCAGACTCACATTCTATTAAAATTTAGCCCTAAAATGACAAGCCTTCTTAAAGCCTTATTTTTCAAA

AGCGCCCCCCCCATTCTTGTTCAGATTAAGAGTTGCCAAAATACCTTCTGAACTACACTGCATTGTTGTG

CCGAGAACACCGAGCACTGAACTTTGCAAAGACCTTCGTCTTTGAGAAGACGGTAGCTTCTGCAGTTAGG

AGGTGCAGACACTTGCTCTCCTATGTAGTTCTCAGATGCGTAAAGCAGAACAGCCTCCCGAATGAAGCGT

TGCCATTGAACTCACCAGTGAGTTAGCAGCACGTGTTCCCGACATAACATTGTACTGTAATGGAGTGAGC

GTAGCAGCTCAGCTCTTTGGATCAGTCTTTGTGATTTCATAGCGAGTTTTCTGACCAGCTTTTGCGGAGA

TTTTGAACAGAACTGCTATTTCCTCTAATGAAGAATTCTGTTTAGCTGTGGGTGTGCCGGGTGGGGTGTG

TGTGATCAAAGGACAAAGACAGTATTTTGACAAAATACGAAGTGGAGATTTACACTACATTGTACAAGGA

ATGAAAGTGTCACGGGTAAAAACTCTAAAAGGTTAATTTCTGTCAAATGCAGTAGATGATGAAAGAAAGG

TTGGTATTATCAGGAAATGTTTTCTTAAGCTTTTCCTTTCTCTTACACCTGCCATGCCTCCCCAAATTGG

GCATTTAATTCATCTTTAAACTGGTTGTTCTGTTAGTCGCTAACTTAGTAAGTGCTTTTCTTATAGAACC

CCTTCTGACTGAGCAATATGCCTCCTTGTATTATAAAATCTTTCTGATAATGCATTAGAAGGTTTTTTTG

TCGATTAGTAAAAGTGCTTTCCATGTTACTTTATTCAGAGCTAATAAGTGCTTTCCTTAGTTTTCTAGTA

ACTAGGTGTAAAAATCATGTGTTGCAGCTTTATAGTTTTTAAAATATTTTAGATAATTCTTAAACTATGA

ACCTTCTTAACATCACTGTCTTGCCAGATTACCGACACTGTCACTTGACCAATACTGACCCTCTTTACCT

CGCCCACGCGGACACACGCCTCCTGTAGTCGCTTTGCCTATTGATGTTCCTTTGGGTCTGTGAGGTTCTG

TAAACTGTGCTAGTGCTGACGATGTTCTGTACAACTTAACTCACTGGCGAGAATACAGCGTGGGACCCTT

CAGCCACTACAACAGAATTTTTTAAATTGACAGTTGCAGAATTGTGGAGTGTTTTTACATTGATCTTTTG

CTAATGCAATTAGCATTATGTTTTGCATGTATGACTTAATAAATCCTTGAATCATA

>NM_006098.5 Homo sapiens receptor for activated C kinase 1 (RACK1), mRNA

CTCTCTTTCACTGCAAGGCGGCGGCAGGAGAGGTTGTGGTGCTAGTTTCTCTAAGCCATCCAGTGCCATC

CTCGTCGCTGCAGCGACACACGCTCTCGCCGCCGCCATGACTGAGCAGATGACCCTTCGTGGCACCCTCA

AGGGCCACAACGGCTGGGTAACCCAGATCGCTACTACCCCGCAGTTCCCGGACATGATCCTCTCCGCCTC

TCGAGATAAGACCATCATCATGTGGAAACTGACCAGGGATGAGACCAACTATGGAATTCCACAGCGTGCT

CTGCGGGGTCACTCCCACTTTGTTAGTGATGTGGTTATCTCCTCAGATGGCCAGTTTGCCCTCTCAGGCT

CCTGGGATGGAACCCTGCGCCTCTGGGATCTCACAACGGGCACCACCACGAGGCGATTTGTGGGCCATAC

CAAGGATGTGCTGAGTGTGGCCTTCTCCTCTGACAACCGGCAGATTGTCTCTGGATCTCGAGATAAAACC

ATCAAGCTATGGAATACCCTGGGTGTGTGCAAATACACTGTCCAGGATGAGAGCCACTCAGAGTGGGTGT

CTTGTGTCCGCTTCTCGCCCAACAGCAGCAACCCTATCATCGTCTCCTGTGGCTGGGACAAGCTGGTCAA

GGTATGGAACCTGGCTAACTGCAAGCTGAAGACCAACCACATTGGCCACACAGGCTATCTGAACACGGTG

ACTGTCTCTCCAGATGGATCCCTCTGTGCTTCTGGAGGCAAGGATGGCCAGGCCATGTTATGGGATCTCA

ACGAAGGCAAACACCTTTACACGCTAGATGGTGGGGACATCATCAACGCCCTGTGCTTCAGCCCTAACCG

CTACTGGCTGTGTGCTGCCACAGGCCCCAGCATCAAGATCTGGGATTTAGAGGGAAAGATCATTGTAGAT

GAACTGAAGCAAGAAGTTATCAGTACCAGCAGCAAGGCAGAACCACCCCAGTGCACCTCCCTGGCCTGGT

CTGCTGATGGCCAGACTCTGTTTGCTGGCTACACGGACAACCTGGTGCGAGTGTGGCAGGTGACCATTGG

CACACGCTAGAAGTTTATGGCAGAGCTTTACAAATAAAAAAAAAAACTGGCTTTTCTGACTTTTAGGTTTT

TTTTTCTTATATGCAAAAAA

>NM_002880.4 Homo sapiens Raf-1 proto-oncogene, serine/threonine kinase (RAF1), transcript variant 2, mRNA

AATCGCGGGCGCTTGGGCCGCCATCTTAGATGGCGGGAGTAAGAGGAAAACGATTGTGAGGCGGGAACGG

CTTTCTGCTGCCTTTTTTGGGCCCCGAAAAGGGTCAGCTGGCCGGGCTTTGGGGCGCGTGCCCTGAGGCG

CGGAGCGCGTTTGCTACGATGCGGGGGCTGCTCGGGGCTCCGTCCCCTGGGCTGGGGACGCGCCGAATGT

GACCGCCTCCCGCTCCCTCACCCGCCGCGGGGAGGAGGAGCGGGCGAGAAGCTGCCGCCGAACGACAGGA

CGTTGGGGCGGCCTGGCTCCCTCAGGTTTAAGAATTGTTTAAGCTGCATCAATGGAGCACATACAGGGAG

CTTGGAAGACGATCAGCAATGGTTTTGGATTCAAAGATGCCGTGTTTGATGGCTCCAGCTGCATCTCTCC

TACAATAGTTCAGCAGTTTGGCTATCAGCGCCGGGCATCAGATGATGGCAAACTCACAGATCCTTCTAAG

ACAAGCAACACTATCCGTGTTTTCTTGCCGAACAAGCAAAGAACAGTGGTCAATGTGCGAAATGGAATGA

GCTTGCATGACTGCCTTATGAAAGCACTCAAGGTGAGGGGCCTGCAACCAGAGTGCTGTGCAGTGTTCAG

ACTTCTCCACGAACACAAAGGTAAAAAAGCACGCTTAGATTGGAATACTGATGCTGCGTCTTTGATTGGA

GAAGAACTTCAAGTAGATTTCCTGGATCATGTTCCCCTCACAACACACAACTTTGCTCGGAAGACGTTCC

TGAAGCTTGCCTTCTGTGACATCTGTCAGAAATTCCTGCTCAATGGATTTCGATGTCAGACTTGTGGCTA

CAAATTTCATGAGCACTGTAGCACCAAAGTACCTACTATGTGTGTGGACTGGAGTAACATCAGACAACTC

TTATTGTTTCCAAATTCCACTATTGGTGATAGTGGAGTCCCAGCACTACCTTCTTTGACTATGCGTCGTA

TGCGAGAGTCTGTTTCCAGGATGCCTGTTAGTTCTCAGCACAGATATTCTACACCTCACGCCTTCACCTT

TAACACCTCCAGTCCCTCATCTGAAGGTTCCCTCTCCCAGAGGCAGAGGTCGACATCCACACCTAATGTC

CACATGGTCAGCACCACCCTGCCTGTGGACAGCAGGATGATTGAGGATGCAATTCGAAGTCACAGCGAAT

CAGCCTCACCTTCAGCCCTGTCCAGTAGCCCCAACAATCTGAGCCCAACAGGCTGGTCACAGCCGAAAAC

CCCCGTGCCAGCACAAAGAGAGCGGGCACCAGTATCTGGGACCCAGGAGAAAAACAAAATTAGGCCTCGT

GGACAGAGAGATTCAAGCTATTATTGGGAAATAGAAGCCAGTGAAGTGATGCTGTCCACTCGGATTGGGT

CAGGCTCTTTTGGAACTGTTTATAAGGGTAAATGGCACGGAGATGTTGCAGTAAAGATCCTAAAGGTTGT

CGACCCAACCCCAGAGCAATTCCAGGCCTTCAGGAATGAGGTGGCTGTTCTGCGCAAAACACGGCATGTG

AACATTCTGCTTTTCATGGGGTACATGACAAAGGACAACCTGGCAATTGTGACCCAGTGGTGCGAGGGCA

GCAGCCTCTACAAACACCTGCATGTCCAGGAGACCAAGTTTCAGATGTTCCAGCTAATTGACATTGCCCG

GCAGACGGCTCAGGGAATGGACTATTTGCATGCAAAGAACATCATCCATAGAGACATGAAATCCAACAAT

ATATTTCTCCATGAAGGCTTAACAGTGAAAATTGGAGATTTTGGTTTGGCAACAGTAAAGTCACGCTGGA

GTGGTTCTCAGCAGGTTGAACAACCTACTGGCTCTGTCCTCTGGATGGCCCCAGAGGTGATCCGAATGCA

GGATAACAACCCATTCAGTTTCCAGTCGGATGTCTACTCCTATGGCATCGTATTGTATGAACTGATGACG

GGGGAGCTTCCTTATTCTCACATCAACAACCGAGATCAGATCATCTTCATGGTGGGCCGAGGATATGCCT

CCCCAGATCTTAGTAAGCTATATAAGAACTGCCCCAAAGCAATGAAGAGGCTGGTAGCTGACTGTGTGAA

GAAAGTAAAGGAAGAGAGGCCTCTTTTTCCCCAGATCCTGTCTTCCATTGAGCTGCTCCAACACTCTCTA

CCGAAGATCAACCGGAGCGCTTCCGAGCCATCCTTGCATCGGGCAGCCCACACTGAGGATATCAATGCTT

GCACGCTGACCACGTCCCCGAGGCTGCCTGTCTTCTAGTTGACTTTGCACCTGTCTTCAGGCTGCCAGGG

GAGGAGGAGAAGCCAGCAGGCACCACTTTTCTGCTCCCTTTCTCCAGAGGCAGAACACATGTTTTCAGAG

AAGCTGCTGCTAAGGACCTTCTAGACTGCTCACAGGGCCTTAACTTCATGTTGCCTTCTTTTCTATCCCT

TTGGGCCCTGGGAGAAGGAAGCCATTTGCAGTGCTGGTGTGTCCTGCTCCCTCCCCACATTCCCCATGCT

CAAGGCCCAGCCTTCTGTAGATGCGCAAGTGGATGTTGATGGTAGTACAAAAAGCAGGGGCCCAGCCCCA

GCTGTTGGCTACATGAGTATTTAGAGGAAGTAAGGTAGCAGGCAGTCCAGCCCTGATGTGGAGACACATG

GGATTTTGGAAATCAGCTTCTGGAGGAATGCATGTCACAGGCGGGACTTTCTTCAGAGAGTGGTGCAGCG

CCAGACATTTTGCACATAAGGCACCAAACAGCCCAGGACTGCCGAGACTCTGGCCGCCCGAAGGAGCCTG

CTTTGGTACTATGGAACTTTTCTTAGGGGACACGTCCTCCTTTCACAGCTTCTAAGGTGTCCAGTGCATT

GGGATGGTTTTCCAGGCAAGGCACTCGGCCAATCCGCATCTCAGCCCTCTCAGGGAGCAGTCTTCCATCA

TGCTGAATTTTGTCTTCCAGGAGCTGCCCCTATGGGGCGGGGCCGCAGGGCCAGCCTTGTTTCTCTAACA

AACAAACAAACAAACAGCCTTGTTTCTCTAGTCACATCATGTGTATACAAGGAAGCCAGGAATACAGGTT

TTCTTGATGATTTGGGTTTTAATTTTGTTTTTATTGCACCTGACAAAATACAGTTATCTGATGGTCCCTC

AATTATGTTATTTTAATAAAATAAATTAAATTTAGGTGTAA

>NM_000321.3 Homo sapiens RB transcriptional corepressor 1 (RB1), transcript variant 1, mRNA

AGTTGCCGGGCGGGGGAGGGCGCGTCCGGTTTTTCTCAGGGGACGTTGAAATTATTTTTGTAACGGGAGT

CGGGAGAGGACGGGGCGTGCCCCGACGTGCGCGCGTCGTCCTCCCCGGCGCTCCTCCACAGCTCGCTG

GCTCCCGCCGCGGAAAGGCGTCATGCCGCCCAAAACCCCCCGAAAAACGGCCGCCACCGCCGCCGCTGCC

GCCGCGGAACCCCCGGCACCGCCGCCGCCGCCCCCTCCTGAGGAGGACCCAGAGCAGGACAGCGGCCCGG

AGGACCTGCCTCTCGTCAGGCTTGAGTTTGAAGAAACAGAAGAACCTGATTTTACTGCATTATGTCAGAA

ATTAAAGATACCAGATCATGTCAGAGAGAGAGCTTGGTTAACTTGGGAGAAAGTTTCATCTGTGGATGGA

GTATTGGGAGGTTATATTCAAAAGAAAAAGGAACTGTGGGGAATCTGTATCTTTATTGCAGCAGTTGACC

TAGATGAGATGTCGTTCACTTTTACTGAGCTACAGAAAAACATAGAAATCAGTGTCCATAAATTCTTTAA

CTTACTAAAAGAAATTGATACCAGTACCAAAGTTGATAATGCTATGTCAAGACTGTTGAAGAAGTATGAT

GTATTGTTTGCACTCTTCAGCAAATTGGAAAGGACATGTGAACTTATATATTTGACACAACCCAGCAGTT

CGATATCTACTGAAATAAATTCTGCATTGGTGCTAAAAGTTTCTTGGATCACATTTTTATTAGCTAAAGG

GGAAGTATTACAAATGGAAGATGATCTGGTGATTTCATTTCAGTTAATGCTATGTGTCCTTGACTATTTT

ATTAAACTCTCACCTCCCATGTTGCTCAAAGAACCATATAAAACAGCTGTTATACCCATTAATGGTTCAC

CTCGAACACCCAGGCGAGGTCAGAACAGGAGTGCACGGATAGCAAAACAACTAGAAAATGATACAAGAAT

TATTGAAGTTCTCTGTAAAGAACATGAATGTAATATAGATGAGGTGAAAAATGTTTATTTCAAAAATTTT

ATACCTTTTATGAATTCTCTTGGACTTGTAACATCTAATGGACTTCCAGAGGTTGAAAATCTTTCTAAAC

GATACGAAGAAATTTATCTTAAAAATAAAGATCTAGATGCAAGATTATTTTTGGATCATGATAAAACTCT

TCAGACTGATTCTATAGACAGTTTTGAAACACAGAGAACACCACGAAAAAGTAACCTTGATGAAGAGGTG

AATGTAATTCCTCCACACACTCCAGTTAGGACTGTTATGAACACTATCCAACAATTAATGATGATTTTAA

ATTCAGCAAGTGATCAACCTTCAGAAAATCTGATTTCCTATTTTAACAACTGCACAGTGAATCCAAAAGA

AAGTATACTGAAAAGAGTGAAGGATATAGGATACATCTTTAAAGAGAAATTTGCTAAAGCTGTGGGACAG

GGTTGTGTCGAAATTGGATCACAGCGATACAAACTTGGAGTTCGCTTGTATTACCGAGTAATGGAATCCA

TGCTTAAATCAGAAGAAGAACGATTATCCATTCAAAATTTTAGCAAACTTCTGAATGACAACATTTTTCA

TATGTCTTTATTGGCGTGCGCTCTTGAGGTTGTAATGGCCACATATAGCAGAAGTACATCTCAGAATCTT

GATTCTGGAACAGATTTGTCTTTCCCATGGATTCTGAATGTGCTTAATTTAAAAGCCTTTGATTTTTACA

AAGTGATCGAAAGTTTTATCAAAGCAGAAGGCAACTTGACAAGAGAAATGATAAAACATTTAGAACGATG

TGAACATCGAATCATGGAATCCCTTGCATGGCTCTCAGATTCACCTTTATTTGATCTTATTAAACAATCA

AAGGACCGAGAAGGACCAACTGATCACCTTGAATCTGCTTGTCCTCTTAATCTTCCTCTCCAGAATAATC

ACACTGCAGCAGATATGTATCTTTCTCCTGTAAGATCTCCAAAGAAAAAAGGTTCAACTACGCGTGTAAA

TTCTACTGCAAATGCAGAGACACAAGCAACCTCAGCCTTCCAGACCCAGAAGCCATTGAAATCTACCTCT

CTTTCACTGTTTTATAAAAAAGTGTATCGGCTAGCCTATCTCCGGCTAAATACACTTTGTGAACGCCTTC

TGTCTGAGCACCCAGAATTAGAACATATCATCTGGACCCTTTTCCAGCACACCCTGCAGAATGAGTATGA

ACTCATGAGAGACAGGCATTTGGACCAAATTATGATGTGTTCCATGTATGGCATATGCAAAGTGAAGAAT

ATAGACCTTAAATTCAAAATCATTGTAACAGCATACAAGGATCTTCCTCATGCTGTTCAGGAGACATTCA

AACGTGTTTTGATCAAAGAAGAGGAGTATGATTCTATTATAGTATTCTATAACTCGGTCTTCATGCAGAG

ACTGAAAACAAATATTTTGCAGTATGCTTCCACCAGGCCCCCTACCTTGTCACCAATACCTCACATTCCT

CGAAGCCCTTACAAGTTTCCTAGTTCACCCTTACGGATTCCTGGAGGGAACATCTATATTTCACCCCTGA

AGAGTCCATATAAAATTTCAGAAGGTCTGCCAACACCAACAAAAATGACTCCAAGATCAAGAATCTTAGT

ATCAATTGGTGAATCATTCGGGACTTCTGAGAAGTTCCAGAAAATAAATCAGATGGTATGTAACAGCGAC

CGTGTGCTCAAAAGAAGTGCTGAAGGAAGCAACCCTCCTAAACCACTGAAAAAACTACGCTTTGATATTG

AAGGATCAGATGAAGCAGATGGAAGTAAACATCTCCCAGGAGAGTCCAAATTTCAGCAGAAACTGGCAGA

AATGACTTCTACTCGAACACGAATGCAAAAGCAGAAAATGAATGATAGCATGGATACCTCAAACAAGGAA

GAGAAATGAGGATCTCAGGACCTTGGTGGACACTGTGTACACCTCTGGATTCATTGTCTCTCACAGATGT

GACTGTATAACTTTCCCAGGTTCTGTTTATGGCCACATTTAATATCTTCAGCTCTTTTTGTGGATATAAA

ATGTGCAGATGCAATTGTTTGGGTGATTCCTAAGCCACTTGAAATGTTAGTCATTGTTATTTATACAAGA

TTGAAAATCTTGTGTAAATCCTGCCATTTAAAAAGTTGTAGCAGATTGTTTCCTCTTCCAAAGTAAAATT

GCTGTGCTTTATGGATAGTAAGAATGGCCCTAGAGTGGGAGTCCTGATAACCCAGGCCTGTCTGACTACT

TTGCCTTCTTTTGTAGCATATAGGTGATGTTTGCTCTTGTTTTTATTAATTTATATGTATATTTTTTTAA

TTTAACATGAACACCCTTAGAAAATGTGTCCTATCTATCTTCCAAATGCAATTTGATTGACTGCCCATTC

ACCAAAATTATCCTGAACTCTTCTGCAAAAATGGATATTATTAGAAATTAGAAAAAAATTACTAATTTTA

CACATTAGATTTTATTTTACTATTGGAATCTGATATACTGTGTGCTTGTTTTATAAAATTTTGCTTTTAA

TTAAATAAAAGCTGGAAGCAAAGTATAACCATATGATACTATCATACTACTGAAACAGATTTCATACCTC

AGAATGTAAAAGAACTTACTGATTATTTTCTTCATCCAACTTATGTTTTTAAATGAGGATTATTGATAGT

ACTCTTGGTTTTTATACCATTCAGATCACTGAATTTATAAAGTACCCATCTAGTACTTGAAAAAGTAAAG

TGTTCTGCCAGATCTTAGGTATAGAGGACCCTAACACAGTATATCCCAAGTGCACTTTCTAATGTTTCTG

GGTCCTGAAGAATTAAGATACAAATTAATTTTACTCCATAAACAGACTGTTAATTATAGGAGCCTTAATT

TTTTTTTCATAGAGATTTGTCTAATTGCATCTCAAAATTATTCTGCCCTCCTTAATTTGGGAAGGTTTGT

GTTTTCTCTGGAATGGTACATGTCTTCCATGTATCTTTTGAACTGGCAATTGTCTATTTATCTTTTATTT

TTTTAAGTCAGTATGGTCTAACACTGGCATGTTCAAAGCCACATTATTTCTAGTCCAAAATTACAAGTAA

TCAAGGGTCATTATGGGTTAGGCATTAATGTTTCTATCTGATTTTGTGCAAAAGCTTCAAATTAAAACAG

CTGCATTAGAAAAAGAGGCGCTTCTCCCCTCCCCTACACCTAAAGGTGTATTTAAACTATCTTGTGTGAT

TAACTTATTTAGAGATGCTGTAACTTAAAATAGGGGATATTTAAGGTAGCTTCAGCTAGCTTTTAGGAAA

ATCACTTTGTCTAACTCAGAATTATTTTTAAAAAGAAATCTGGTCTTGTTAGAAAACAAAATTTTATTTT

GTGCTCATTTAAGTTTCAAACTTACTATTTTGACAGTTATTTTGATAACAATGACACTAGAAAACTTGAC

TCCATTTCATCATTGTTTCTGCATGAATATCATACAAATCAGTTAGTTTTTAGGTCAAGGGCTTACTATT

TCTGGGTCTTTTGCTACTAAGTTCACATTAGAATTAGTGCCAGAATTTTAGGAACTTCAGAGATCGTGTA

TTGAGATTTCTTAAATAATGCTTCAGATATTATTGCTTTATTGCTTTTTTGTATTGGTTAAAACTGTACA

TTTAAAATTGCTATGTTACTATTTTCTACAATTAATAGTTTGTCTATTTTAAAATAAATTAGTTGTTAAG

AGTCTTAA

>NM_014781.5 Homo sapiens RB1 inducible coiled-coil 1 (RB1CC1), transcript variant 1, mRNA

GGAGTCGACAATAACAAACCAAGCCGCGGCGGTGTCCGCGGCCCTGCCGAGCCCTCGGCGTTGCCTCAGA

ATCCCCCAGTCGCCTGGGCCCCTCGGCTCTGACAGGCCGCGGCCTTCTGTCCCCCGGCCCCAGACCCAGA

GCCGAGGGGCCTGCTCGCGTCCTTGTCCGCCCGGACCCCTCCCTGCCTCCTAGAGTTCGGGGCCGCGGCG

GGCGGGCGCCCGGGACGCCGGCGGTTGTGTCGGCTTAGCGGTGCCGAATGGGCGGTTGGTAACCGCTGCC

GAGGACTAGGCGGCGGCGGAAGATGGTGCCGGGGGTCGCTGGCTCTGCTGCTGCCGCCGGCGAAGGAGGA

GGCGTTGCCGGTTTTCTGAGTTTAACCAGTAATGCCATTCAGTTGCCAATCTCAAGCAAAGCAAACATAA

GCCAGTTTTAATCTACTTTTTAAGAAAAGTGGTAGTCCTTTTCACAGTGCCTGACGTAACTGTATCAGAG

GGTGAGGTATAAGCTCACAGAATTCAGATAAATCATCATGAAGTTATATGTATTTCTGGTTAACACTGGA

ACTACTCTAACATTTGACACTGAACTTACAGTGCAAACTGTGGCAGACCTTAAGCATGCCATTCAAAGCA

AATACAAGATTGCTATTCAACACCAGGTGCTGGTGGTCAATGGAGGAGAATGCATGGCTGCAGATCGAAG

AGTGTGTACCTACAGTGCTGGGACGGATACAAATCCAATTTTTCTTTTTAACAAAGAAATGATCTTATGT

GATCGTCCACCTGCTATTCCTAAAACTACCTTTTCGACAGAAAATGACATGGAAATAAAAGTTGAAGAAT

CTCTTATGATGCCTGCAGTTTTTCATACTGTTGCTTCAAGGACACAGCTTGCATTGGAAATGTATGAAGT

TGCCAAGAAACTTTGTTCTTTTTGTGAAGGTCTTGTACATGATGAACATCTTCAACACCAAGGCTGGGCT

GCAATCATGGCCAACCTGGAGGACTGTTCAAATTCATACCAAAAGCTACTTTTCAAGTTTGAAAGTATTT

ATTCAAATTATCTGCAGTCCATAGAAGACATCAAGTTAAAACTTACTCATTTAGGAACTGCAGTTTCAGT

AATGGCCAAGATTCCACTGTTGGAGTGCCTAACCAGACATAGTTACAGAGAATGTTTGGGAAGACTGGAT

TCTTTACCTGAACATGAAGACTCAGAAAAAGCTGAGATGAAAAGATCCACTGAACTGGTGCTCTCTCCTG

ATATGCCTAGAACAACTAACGAATCTTTGTTAACCTCATTTCCCAAGTCAGTGGAACATGTGTCCCCAGA

TACCGCAGATGCTGAAAGTGGCAAAGAAATTAGGGAATCTTGTCAAAGTACTGTTCATCAGCAAGATGAA

ACTACGATTGACACTAAAGATGGTGATCTGCCCTTTTTTAATGTCTCTTTGTTAGACTGGATAAATGTTC

AAGATAGACCTAATGATGTGGAATCTTTGGTCAGGAAGTGCTTTGATTCTATGAGCAGGCTTGATCCAAG

GATTATTCGACCATTTATAGCAGAATGCCGTCAAACTATTGCCAAACTTGATAATCAGAATATGAAAGCC

ATTAAAGGACTTGAAGATCGGCTCTACGCCCTGGACCAGATGATTGCTAGCTGTGGCCGACTGGTGAATG

AACAGAAAGAGCTTGCTCAGGGATTTTTAGCTAATCAGAAGAGAGCTGAAAACTTAAAGGATGCATCTGT

ATTACCTGATTTATGCCTGAGTCACGCAAATCAGTTGATGATTATGTTGCAAAATCATAGAAAACTGTTA

GATATTAAGCAGAAGTGTACCACTGCCAAACAAGAACTAGCAAATAACCTACATGTCAGACTGAAGTGGT

GTTGCTTTGTAATGCTTCATGCTGATCAAGATGGAGAGAAGTTACAAGCTTTGCTCCGCCTCGTAATAGA

GCTGTTAGAAAGAGTCAAAATTGTTGAAGCTCTTAGTACAGTTCCTCAGATGTACTGCTTAGCTGTTGTT

GAGGTTGTAAGAAGAAAAATGTTCATAAAACACTACAGGGAGTGGGCTGGTGCTTTAGTCAAAGATGGAA

AGAGATTATATGAAGCAGAAAAATCAAAAAGGGAATCCTTTGGGAAATTATTTAGGAAGTCTTTTTTAAG

AAATCGTCTGTTTAGGGGACTGGACTCCTGGCCCCCTTCCTTTTGTACTCAAAAGCCTCGAAAGTTTGAC

TGTGAACTTCCAGATATTTCATTAAAAGATTTACAGTTTCTGCAATCATTTTGTCCTTCGGAAGTTCAGC

CATTCCTCAGGGTTCCCTTACTTTGTGACTTTGAACCTCTACACCAGCATGTACTTGCTCTACATAATTT

GGTAAAAGCAGCACAAAGTTTGGATGAAATGTCACAGACCATTACAGATCTACTGAGTGAACAAAAGGCA

TCTGTGAGTCAGACATCCCCACAGTCTGCTTCTTCACCAAGGATGGAAAGTACAGCAGGAATTACAACTA

CTACCTCACCGAGAACTCCTCCACCACTGACTGTTCAGGATCCCTTATGTCCTGCAGTTTGTCCCTTAGA

AGAATTATCTCCAGATAGTATTGATGCACATACGTTTGATTTTGAAACTATTCCCCATCCAAACATAGAA

CAGACTATTCACCAAGTTTCTTTAGACTTGGATTCATTAGCAGAAAGTCCTGAATCAGATTTTATGTCTG

CTGTGAATGAGTTTGTAATAGAAGAAAATTTGTCGTCTCCTAATCCTATAAGTGATCCACAAAGCCCAGA

AATGATGGTGGAATCACTTTATTCATCAGTTATCAATGCGATAGACAGTAGACGAATGCAGGATACAAAT

GTATGTGGTAAGGAGGATTTTGGAGATCATACTTCTCTGAATGTCCAGTTGGAAAGATGTAGAGTTGTTG

CCCAAGACTCTCACTTCAGTATACAAACCATTAAGGAAGACCTTTGCCACTTTAGAACATTTGTACAAAA

AGAACAGTGTGACTTCTCAAATTCATTAAAATGTACAGCAGTAGAAATAAGAAACATTATTGAAAAAGTA

AAATGTTCTCTGGAAATAACACTAAAAGAAAAACATCAAAAAGAACTACTGTCTTTAAAAAATGAATATG

AAGGTAAACTTGACGGACTAATAAAGGAAACTGAAGAGAATGAAAACAAAATTAAAAAATTGAAGGGAGA

GTTAGTATGCCTTGAGGAGGTTTTACAAAATAAAGATAATGAATTTGCTTTGGTTAAACATGAAAAAGAA

GCTGTAATCTGCCTGCAGAATGAAAAGGATCAGAAGTTGTTAGAGATGGAAAATATAATGCACTCTCAAA

ATTGTGAAATTAAAGAACTGAAGCAGTCACGAGAAATAGTGTTAGAAGACTTAAAAAAGCTCCATGTTGA

AAATGATGAGAAGTTACAGTTATTGAGGGCAGAACTTCAGTCCTTGGAGCAAAGTCATCTAAAGGAATTA

GAGGACACACTTCAGGTTAGGCACATACAAGAGTTTGAGAAGGTTATGACAGACCACAGAGTTTCTTTGG

AGGAATTAAAAAAGGAAAACCAACAAATAATTAATCAAATACAAGAATCTCATGCTGAAATTATCCAGGA

AAAAGAAAAACAGTTACAGGAATTAAAACTCAAGGTTTCTGATTTGTCAGACACGAGATGCAAGTTAGAG

GTTGAACTTGCGTTGAAGGAAGCAGAAACTGATGAAATAAAAATTTTGCTGGAAGAAAGCAGAGCCCAGC

AGAAGGAGACCTTGAAATCTCTTCTTGAACAAGAGACAGAAAATTTGAGAACAGAAATTAGTAAACTCAA

CCAAAAGATTCAGGATAATAATGAAAATTATCAGGTGGGCTTAGCAGAGCTAAGAACTTTAATGACAATT

GAAAAAGATCAGTGTATTTCCGAGTTAATTAGTAGACATGAAGAAGAATCTAATATACTTAAAGCTGAAT

TAAACAAAGTAACATCTTTGCATAACCAAGCATTTGAAATAGAAAAAAACCTAAAAGAACAAATAATTGA

ACTGCAGAGTAAATTGGATTCAGAATTGAGTGCTCTTGAAAGACAAAAAGATGAAAAAATTACCCAACAA

GAAGAGAAATACGAAGCTATTATCCAGAACCTTGAGAAAGACAGACAAAAATTGGTCAGCAGCCAGGAGC

AAGACAGAGAACAGTTAATTCAGAAGCTTAATTGTGAAAAAGATGAAGCTATTCAGACTGCCCTAAAAGA

ATTTAAATTGGAGAGAGAAGTTGTTGAGAAAGAGTTATTAGAAAAAGTTAAACATCTTGAGAATCAAATA

GCAAAAAGTCCTGCCATTGACTCTACCAGAGGAGATTCTTCAAGCTTAGTTGCTGAACTTCAAGAAAAGC

TTCAGGAAGAAAAAGCTAAGTTTCTAGAACAACTTGAAGAGCAAGAAAAAAGAAAGAATGAAGAAATGCA

AAATGTTCGAACATCTTTGATTGCGGAACAACAGACCAATTTTAACACTGTTTTAACAAGAGAGAAAATG

AGAAAAGAAAACATAATAAATGATCTTAGTGATAAGTTGAAAAGTACAATGCAGCAACAAGAACGGGATA

AAGATTTGATAGAGTCACTTTCTGAAGATCGAGCTCGTTTGCTTGAGGAAAAGAAAAAGCTTGAAGAAGA

AGTCAGTAAGTTGCGTAGTAGCAGTTTTGTTCCTTCACCATATGTAGCTACAGCCCCAGAACTTTATGGA

GCTTGTGCACCTGAACTCCCAGGTGAATCAGATAGATCCGCTGTGGAAACAGCAGATGAAGGAAGAGTGG

ATTCAGCAATGGAGACAAGCATGATGTCTGTACAAGAAAATATTCATATGTTGTCTGAAGAAAAACAGCG

GATAATGCTGTTAGAACGAACATTGCAATTGAAAGAAGAAGAAAATAAACGGTTAAATCAAAGACTGATG

TCTCAGAGCATGTCTTCAGTATCTTCAAGGCATTCTGAAAAGATAGCTATTAGAGATTTTCAGGTGGGAG

ATTTGGTACTCATCATCCTAGACGAACGCCATGACAATTATGTGTTATTTACTGTTAGTCCTACTTTATA

TTTTCTACATTCAGAGTCTCTACCTGCCCTGGATCTCAAACCAGGTGAGGGTGCTTCAGGTGCATCTAGA

AGACCCTGGGTACTTGGAAAAGTAATGGAAAAAGAATACTGTCAAGCCAAAAAGGCACAAAACAGATTTA

AAGTTCCTTTGGGGACAAAGTTTTACAGAGTGAAAGCCGTATCATGGAATAAGAAAGTATAACTTATGGA

CAAAATTAATACATTCTATGACATTTTTTTCTGATTTGTCCTGCAGTGCTCATTCATCACTCCAAAAACA

GCAGGCCATCTTTTTATGCAAAAGTCAGCGTGACAATATACTTCACTGGTGTACATCGTTTACTTTTTAA

CTGGCTTCATTTTAGGAATAATAAATTCATCAGAATCCTTGGCTGAATTAAAATGGTTTTTGTTTTTTGG

TTTTTTTTTTTACCCAGACAACTCTAGAAATGCGGACCAAACTACTTCATTTTCTCAAAGGGCATACCTT

GTGCATTGTGGCTTATGATGAGCCATATTAATTGCCTGTTAAATATACACTAGCTTGAACTTAGATGTTA

AATGTTATTATTACCAGCATTTGTCCTTTTGTGAAATCAGTATCAGAATACTTGCACTCTTTAACACATT

CTTTATAAAATGTATAAATTATTCAGAACTATTTAAAATAAAGAGGAGTGTTATTGCATGCTGATAATCA

TTTTGAGTTTGCCTCAGTAGATACTAAAGCAAATTGTTTCAGTTTTTTTAAATGCCCTTTGATGTTTCAA

AAAAAAAAAGGAACTGTAATTTGATTGACTGATTTTAAGATCAGCCATAAGTAATCAGCAATCTTCAAAA

GCACTTTCAGTGGATTGGTCATCTGGGTTCTAAAGGGAAGAGTCTGTGCTACTAACCATTTCAAATGCAG

ACTCAAACCTTCCCAACATCTTTATGACTCTAGAATAATCATATTGATGAAATCGTAATTCATGGTTGAG

TTTCAGAACAAAAGATATTCATTGCACATTAACCATTTAGAGGTCATTTAAATAACAAAATATTGTATTG

TAAAAGAACTGTACAATTTTAAAACAATAAAGATTTGAACCTGTAAATGTGTGTGCCTTTTAAAGAAGGA

TACATTTTTAATATATTTGAGTGATTGCTGGGAAGTGTGAAAATATTGTTATGTATCATATCAAAGAGAA

ACATGTTTATTACAAAAATGTTCTTTAACTATATACTATGTAACAGGGTAAACAGTGTTATGTAGAATAG

AATTGTGTAAACTAGATCTTTAGAGAAGTTGCCATTGAGCAAAGTTATTTAAATGAGTTAGTTGAGTTGG

ATGAGAATTGTTTGAGGTTTGTTGCTAGAGAACAATAATAAAATAATTCTTTTTCAGAAAATATTTAATT

TCTTCATAAAAATAAGTTAAATATTTTTTTAAATATGTATATCTAATAGTACAAAATGGAATAAACATCA

TAGTGTATAGAAAACTGAATTTGACAAGTTAATGAATAAATGAACAAATGATTTCA

>NM_021975.4 Homo sapiens RELA proto-oncogene, NF-kB subunit (RELA), transcript variant 1, mRNA

ATTTCCGCCTCTGGCGAATGGCTCGTCTGTAGTGCACGCCGCGGGCCCAGCTGCGACCCCGGCCCCGCCC

CCGGGACCCCGGCCATGGACGAACTGTTCCCCCTCATCTTCCCGGCAGAGCCAGCCCAGGCCTCTGGCCC

CTATGTGGAGATCATTGAGCAGCCCAAGCAGCGGGGCATGCGCTTCCGCTACAAGTGCGAGGGGCGCTCC

GCGGGCAGCATCCCAGGCGAGAGGAGCACAGATACCACCAAGACCCACCCCACCATCAAGATCAATGGCT

ACACAGGACCAGGGACAGTGCGCATCTCCCTGGTCACCAAGGACCCTCCTCACCGGCCTCACCCCCACGA

GCTTGTAGGAAAGGACTGCCGGGATGGCTTCTATGAGGCTGAGCTCTGCCCGGACCGCTGCATCCACAGT

TTCCAGAACCTGGGAATCCAGTGTGTGAAGAAGCGGGACCTGGAGCAGGCTATCAGTCAGCGCATCCAGA

CCAACAACAACCCCTTCCAAGTTCCTATAGAAGAGCAGCGTGGGGACTACGACCTGAATGCTGTGCGGCT

CTGCTTCCAGGTGACAGTGCGGGACCCATCAGGCAGGCCCCTCCGCCTGCCGCCTGTCCTTTCTCATCCC

ATCTTTGACAATCGTGCCCCCAACACTGCCGAGCTCAAGATCTGCCGAGTGAACCGAAACTCTGGCAGCT

GCCTCGGTGGGGATGAGATCTTCCTACTGTGTGACAAGGTGCAGAAAGAGGACATTGAGGTGTATTTCAC

GGGACCAGGCTGGGAGGCCCGAGGCTCCTTTTCGCAAGCTGATGTGCACCGACAAGTGGCCATTGTGTTC

CGGACCCCTCCCTACGCAGACCCCAGCCTGCAGGCTCCTGTGCGTGTCTCCATGCAGCTGCGGCGGCCTT

CCGACCGGGAGCTCAGTGAGCCCATGGAATTCCAGTACCTGCCAGATACAGACGATCGTCACCGGATTGA

GGAGAAACGTAAAAGGACATATGAGACCTTCAAGAGCATCATGAAGAAGAGTCCTTTCAGCGGACCCACC

GACCCCCGGCCTCCACCTCGACGCATTGCTGTGCCTTCCCGCAGCTCAGCTTCTGTCCCCAAGCCAGCAC

CCCAGCCCTATCCCTTTACGTCATCCCTGAGCACCATCAACTATGATGAGTTTCCCACCATGGTGTTTCC

TTCTGGGCAGATCAGCCAGGCCTCGGCCTTGGCCCCGGCCCCTCCCCAAGTCCTGCCCCAGGCTCCAGCC

CCTGCCCCTGCTCCAGCCATGGTATCAGCTCTGGCCCAGGCCCCAGCCCCTGTCCCAGTCCTAGCCCCAG

GCCCTCCTCAGGCTGTGGCCCCACCTGCCCCCAAGCCCACCCAGGCTGGGGAAGGAACGCTGTCAGAGGC

CCTGCTGCAGCTGCAGTTTGATGATGAAGACCTGGGGGCCTTGCTTGGCAACAGCACAGACCCAGCTGTG

TTCACAGACCTGGCATCCGTCGACAACTCCGAGTTTCAGCAGCTGCTGAACCAGGGCATACCTGTGGCCC

CCCACACAACTGAGCCCATGCTGATGGAGTACCCTGAGGCTATAACTCGCCTAGTGACAGGGGCCCAGAG

GCCCCCCGACCCAGCTCCTGCTCCACTGGGGGCCCCGGGGCTCCCCAATGGCCTCCTTTCAGGAGATGAA

GACTTCTCCTCCATTGCGGACATGGACTTCTCAGCCCTGCTGAGTCAGATCAGCTCCTAAGGGGGTGACG

CCTGCCCTCCCCAGAGCACTGGGTTGCAGGGGATTGAAGCCCTCCAAAAGCACTTACGGATTCTGGTGGG

GTGTGTTCCAACTGCCCCCAACTTTGTGGATGTCTTCCTTGGAGGGGGGAGCCATATTTTATTCTTTTAT

TGTCAGTATCTGTATCTCTCTCTCTTTTTGGAGGTGCTTAAGCAGAAGCATTAACTTCTCTGGAAAGGGG

GGAGCTGGGGAAACTCAAACTTTTCCCCTGTCCTGATGGTCAGCTCCCTTCTCTGTAGGGAACTCTGGGG

TCCCCCATCCCCATCCTCCAGCTTCTGGTACTCTCCTAGAGACAGAAGCAGGCTGGAGGTAAGGCCTTTG

AGCCCACAAAGCCTTATCAAGTGTCTTCCATCATGGATTCATTACAGCTTAATCAAAATAACGCCCCAGA

TACCAGCCCCTGTATGGCACTGGCATTGTCCCTGTGCCTAACACCAGCGTTTGAGGGGCTGGCCTTCCTG

CCCTACAGAGGTCTCTGCCGGCTCTTTCCTTGCTCAACCATGGCTGAAGGAAACCAGTGCAACAGCACTG

GCTCTCTCCAGGATCCAGAAGGGGTTTGGTCTGGGACTTCCTTGCTCTCCCTCTTCTCAAGTGCCTTAAT

AGTAGGGTAAGTTGTTAAGAGTGGGGGAGAGCAGGCTGGCAGCTCTCCAGTCAGGAGGCATAGTTTTTAC

TGAACAATCAAAGCACTTGGACTCTTGCTCTTTCTACTCTGAACTAATAAATCTGTTGCCAAGCTGG

>NM_005873.3 Homo sapiens regulator of G protein signaling 19 (RGS19), transcript variant 1, mRNA

GGATCTGGCAGCCACTCAGCCCTTCGAGCTGTAGGGGAGGCGCTGCGCCCCCGGCCCCAGCCGATCGGGG

GCTCCTGGCGCTAGCCTCCCTCCAGAGCGCCGCAGGAGTCCCTCCTGCCGTCTGACTTGAGTCCCTGCTG

CTGCAGTGCACGCCCCTTCGGCAGCCAGGGTGGGGCCCAGACCCAACCTGGCCCCTCCTGCCCCCACCCC

GCCTTTGGGTCGCTGACCCCCAGGCTGTGGTGAGGGTCTGAGAGCTGGTACCACGGAGCCTGGGCAACCT

CCTCCGCCCACCCATGCCCACCCCGCATGAGGCTGAGAAGCAGATCACAGGGCCAGAGGAGGCGGACCGG

CCCCCTTCAATGTCCAGTCATGATACAGCCTCTCCAGCGGCCCCCAGCCGCAACCCCTGCTGCCTGTGCT

GGTGCTGCTGCTGTAGCTGCTCCTGGAACCAAGAGCGGCGGCGCGCGTGGCAGGCCTCCCGGGAGAGCAA

GCTGCAGCCCCTCCCCAGCTGTGAAGTATGTGCCACGCCAAGTCCTGAGGAGGTGCAGAGCTGGGCGCAG

TCTTTTGACAAGCTGATGCACAGCCCAGCGGGACGCAGCGTGTTCCGGGCGTTCCTGCGGACAGAGTACA

GCGAGGAGAACATGCTCTTCTGGTTGGCCTGCGAGGAGCTGAAGGCCGAGGCCAACCAGCATGTGGTAGA

CGAGAAGGCGAGGCTCATCTACGAGGACTACGTATCCATCCTGTCCCCCAAGGAGGTGAGCCTGGACTCC

CGTGTGCGGGAGGGCATCAACAAGAAGATGCAGGAGCCGTCCGCACACACGTTCGACGACGCGCAGCTGC

AGATCTACACGCTCATGCACCGGGACTCCTACCCCCGCTTCCTCAGCTCTCCCACCTACCGTGCCCTGCT

GCTGCAGGGGCCATCACAGTCCTCCTCCGAGGCCTAGGCCGCCCCCAGCAGCACAGACCCCGCCGCCTCC

TACGGCCGACTCTGGGTTCCCTTCAGGTGTTGTTGTCGGCAGGGCTGTCAGGGCACATGCCTGGCCGGCT

GGGGTCCCCACCCGCGGAGACGGTCCTAGACCCAGTGGGGAGTGCTGTGTCTCCTGGGTCTGCCCACCCG

TGGCCAAGCAGGAACTCCAGGTGCAGGGCGGGCCTCCAGGTGCAGGGCGGGCCAGAAGCCCCCTCATCGG

CCCGCAGCTGGCTGGCCCAGTGCTCCTGGCTGGGGGCTCTTGCGTGGTGAGAGTAGGGGTCCCCCCAGGA

GCCATGCTGGACCCATGAGCCTCGCTGGGGCCTCCTCCCCAGGCAGCCAATGGGCCCCAGACAAGCCTTT

GGTGGGGAACAGAACCTCCGCATCGTGTAGTTTTGTGACATAAGGAGACCTCCTACTTGAGCTGTCTGTA

CCCCAGAATCAAACACAGAACCTCAGAACCAGATTTAGGACCTCAGAATCCTGCACTCAAGGTGGCAGGA

CCCAATTTCTGTTTTATATGTTCATGAACTTTAAACCTGGAAACATGTCCTTACTAGGTGTTTTATCAAA

AAAAAGTTTTCATATTTATCTCTATCCCTAACCCCCCAAAAATAGGATGTCTTATTATTGAAGATATTTT

TAAAGCAAA

>NM_005614.4 Homo sapiens Ras homolog, mTORC1 binding (RHEB), mRNA

GGGAGGGTCATGACGCAGCGAGTTTCAGTCGTGACTTTTCTGGGGGCATCGCGGCGTCCCCTTTTTTTGC

CTTTAAAGTAAAACGTCGCCCCGACGCACCCCCCGCGTATTTCGGGGGGCGGAGGCGGCGGGCCACGGCG

CGAAGAGGGGCGGTGCTGACGCCGGCCGGTCACGTGGGCGTGTTGTGGGGGGGAGGGGCGCCGCCGCGCG

GTCGGTTCCGGGCGGTTGGGAGCGCGCGAGCTAGCGAGCGAGAGGCAGCCGCGCCCGCCGCCGCCCCTGC

TCTGTATGCCGCTCTCTCCCGGCGCGGCCGCCGCCGATCACAGCAGCAGGAGCCACCGCCGCCGCGGTTG

ATGTGGTTGGGCCGGGGCTGAGGAGGCCGCCAAGATGCCGCAGTCCAAGTCCCGGAAGATCGCGATCCTG

GGCTACCGGTCTGTGGGGAAATCCTCATTGACGATTCAATTTGTTGAAGGCCAATTTGTGGACTCCTACG

ATCCAACCATAGAAAACACTTTTACAAAGTTGATCACAGTAAATGGACAAGAATATCATCTTCAACTTGT

AGACACAGCCGGGCAAGATGAATATTCTATCTTTCCTCAGACATACTCCATAGATATTAATGGCTATATT

CTTGTGTATTCTGTTACATCAATCAAAAGTTTTGAAGTGATTAAAGTTATCCATGGCAAATTGTTGGATA

TGGTGGGGAAAGTACAAATACCTATTATGTTGGTTGGGAATAAGAAAGACCTGCATATGGAAAGGGTGAT

CAGTTATGAAGAAGGGAAAGCTTTGGCAGAATCTTGGAATGCAGCTTTTTTGGAATCTTCTGCTAAAGAA

AATCAGACTGCTGTGGATGTTTTTCGAAGGATAATTTTGGAGGCAGAAAAAATGGACGGGGCAGCTTCAC

AAGGCAAGTCTTCATGCTCGGTGATGTGATTCTGCTGCAAAGCCTGAGGACACTGGGAATATATTCTACC

TGAAGAAGCAAACTGCCCGTTCTCCTTGAAGATAAACTATGCTTCTTTTTTCTTCTGTTAACCTGAAAGA

TATCATTTGGGTCAGAGCTCCCCTCCCTTCAGATTATGTTAACTCTGAGTCTGTCCAAATGAGTTCACTT

CCATTTTCAAATTTTAAGCAATCATATTTTCAATTTATATATTGTATTTCTTAATATTATGACCAAGAAT

TTTATCGGCATTAATTTTTCAGTGTAGTTTGTTGTTTAAAATAATGTAATCATCAAAATGATGCATATTG

TTACACTACTATTAACTAGGCTTCAGTATATCAGTGTTTATTTCATTGTGTTAAATGTATACTTGTAAAT

AAAATAGCTGCAAACCTCAGTCCTTTGTGCTACTTGATGTGGCTTTCAAAGAAGAGAAGCCTTGTCCTGA

GTTTCTCACTTGGCTTCAGGAAGGCCCCAGGTTGGATTCCAGAAACCAGTGAAGATGTGGCCACAGGAGG

AGGTGTGCTGAGGTGGCTGCTGACCGTGGACTCCCTGCGCAGTGGCCTGCAGATGTTGGGGCTGGGTTAC

AGCTGATTGAAGCTGAGTGGCCCTGGGGGGTCTGTGAGGGGAGTTCCTCCCCAGTGATGAAATTCTCTCC

TTCCACCCTCAAATCCCTAGACCTTGACTGAAATGCTCCGTGGTCGGGAGCCTGGTCAAGGAGGAGGAGC

TGCTGAGAGGCATTGTTCGCCCTTGCTCATAGCTTAGCTCGATGTCCGTGTCAGACAGGAGATGATTGAG

AACAGCCTTGCCTGTCACTGTCCTAGAACACCCTGGAGTTTAGTGTTCTGTGTCAGAGTCTTGGGAGCCT

CCTTCAGACCCAGATGACGGGCCTCCCTCTGTCCAAGGAGCAGCTGTAAAGGAGAAGAGGGATTTCATTT

GTTTGGTGGCTGTTACCTTGTCTGTAAGTCAAACTTGGAGTTGAGCAGTGCTTTTTAAACGATTCCCTTT

TGCAGCTAAAATTTCACAGGGCTATTTCTAATACGTAAGCAAATGTTACCATTGACTTTATTAATAAAAT

ATAGTTTTGCTTTGCA

>NM_003161.4 Homo sapiens ribosomal protein S6 kinase B1 (RPS6KB1), transcript variant 1, mRNA

GCACTGAGCCTAAGCAGCCGGTGATGGCGGCAGCGGCTGTGGTGGCTGCGGCGGGTCCGGGCCCATGAGG

CGACGAAGGAGGCGGGACGGCTTTTACCCAGCCCCGGACTTCCGAGACAGGGAAGCTGAGGACATGGCAG

GAGTGTTTGACATAGACCTGGACCAGCCAGAGGACGCGGGCTCTGAGGATGAGCTGGAGGAGGGGGGTCA

GTTAAATGAAAGCATGGACCATGGGGGAGTTGGACCATATGAACTTGGCATGGAACATTGTGAGAAATTT

GAAATCTCAGAAACTAGTGTGAACAGAGGGCCAGAAAAAATCAGACCAGAATGTTTTGAGCTACTTCGGG

TACTTGGTAAAGGGGGCTATGGAAAGGTTTTTCAAGTACGAAAAGTAACAGGAGCAAATACTGGGAAAAT

ATTTGCCATGAAGGTGCTTAAAAAGGCAATGATAGTAAGAAATGCTAAAGATACAGCTCATACAAAAGCA

GAACGGAATATTCTGGAGGAAGTAAAGCATCCCTTCATCGTGGATTTAATTTATGCCTTTCAGACTGGTG

GAAAACTCTACCTCATCCTTGAGTATCTCAGTGGAGGAGAACTATTTATGCAGTTAGAAAGAGAGGGAAT

ATTTATGGAAGACACTGCCTGCTTTTACTTGGCAGAAATCTCCATGGCTTTGGGGCATTTACATCAAAAG

GGGATCATCTACAGAGACCTGAAGCCGGAGAATATCATGCTTAATCACCAAGGTCATGTGAAACTAACAG

ACTTTGGACTATGCAAAGAATCTATTCATGATGGAACAGTCACACACACATTTTGTGGAACAATAGAATA

CATGGCCCCTGAAATCTTGATGAGAAGTGGCCACAATCGTGCTGTGGATTGGTGGAGTTTGGGAGCATTA

ATGTATGACATGCTGACTGGAGCACCCCCATTCACTGGGGAGAATAGAAAGAAAACAATTGACAAAATCC

TCAAATGTAAACTCAATTTGCCTCCCTACCTCACACAAGAAGCCAGAGATCTGCTTAAAAAGCTGCTGAA

AAGAAATGCTGCTTCTCGTCTGGGAGCTGGTCCTGGGGACGCTGGAGAAGTTCAAGCTCATCCATTCTTT

AGACACATTAACTGGGAAGAACTTCTGGCTCGAAAGGTGGAGCCCCCCTTTAAACCTCTGTTGCAATCTG

AAGAGGATGTAAGTCAGTTTGATTCCAAGTTTACACGTCAGACACCTGTCGACAGCCCAGATGACTCAAC

TCTCAGTGAAAGTGCCAATCAGGTCTTTCTGGGTTTTACATATGTGGCTCCATCTGTACTTGAAAGTGTG

AAAGAAAAGTTTTCCTTTGAACCAAAAATCCGATCACCTCGAAGATTTATTGGCAGCCCACGAACACCTG

TCAGCCCAGTCAAATTTTCTCCTGGGGATTTCTGGGGAAGAGGTGCTTCGGCCAGCACAGCAAATCCTCA

GACACCTGTGGAATACCCAATGGAAACAAGTGGCATAGAGCAGATGGATGTGACAATGAGTGGGGAAGCA

TCGGCACCACTTCCAATACGACAGCCGAACTCTGGGCCATACAAAAAACAAGCTTTTCCCATGATCTCCA

AACGGCCAGAGCACCTGCGTATGAATCTATGACAGAGCAATGCTTTTAATGAATTTAAGGCAAAAAAGGT

GGAGAGGGAGATGTGTGAGCATCCTGCAAGGTGAAACGACTCAAAATGACAGTTTCAGAGAGTCAATGTC

ATTACATAGAACACTTCAGACACAGGAAAAATAAACGTGGATTTTAAAAAATCAATCAATGGTGCAAAAA

AAAACTTAAAGCAAAATAGTATTGCTGAACTCTTAGGCACATCAATTAATTGATTCCTCGCGACATCTTC

TCAACCTTATCAAGGATTTTCATGTTGATGACTCGAAACTGACAGTATTAAGGGTAGGATGTTGCTTCTG

AATCACTGTTGAGTTCTGATTGTGTTGAAGAAGGGTTATCCTTTCATTAGGCAAAGTACAAAATTGCCTA

TAATACTTGCAACTAAGGACAAATTAGCATGCAAGCTTGGTCAAACTTTTTCCAGCAAAATGGAAGCAAA

GACAAAAGAAACTTACCAATTGATGTTTTACGTGCAAACAACCTGAATCTTTTTTTTATATAAATATATA

TTTTTCAAATAGATTTTTGATTCAGCTCATTATGAAAAACATCCCAAACTTTAAAATGCGAAATTATTGG

TTGGTGTGAAGAAAGCCAGACAACTTCTGTTTCTTCTCTTGGTGAAATAATAAAATGCAAATGAATCATT

GTTAACCACAGCTGTGGCTCGTTTGAGGGATTGGGGTGGACCTGGGGTTTATTTTCAGTAACCCAGCTGC

AATACCTGTCTGTAATATGAGAAAAAAAAAAATGAATCTATTTAATCATTTCTACTTGCAGTACTGCTATG

TGCTAAGCTTAACTGGAAGCCTTGGAATGGGCATAAGTTGTATGTCCTACATTTCATCATTGTCCCGGGC

CTGCATTGCACTGGAAAAAAAAATCGCCACCTGTTCTTACACCAGTATTTGGTTCAAGACACCAAATGTC

TTCAGCCCATGGCTGAAGAACAACAGAAGAGAGTCAGGATAAAAAATACATACTGTGGTCGGCAAGGTGA

GGGAGATAGGGATATCCAGGGGAAGAGGGTGTTGCTGTGGCCCACTCTCTGTCTAATCTCTTTACAGCAA

ATTGGTAAGATTTTCAGTTTTACTTCTTTCTACTGTTTCTGCTGTCTACCTTCCTTATATTTTTTTCCTC

AACAGTTTTAAAAAGAAAAAAAGGTCTATTTTTTTTTCTCCTATACTTGGGCTACATTTTTTGATTGTAA

AAATATTTGATGGCCTTTTGATGAATGTCTTCCACAGTAAAGAAAACTTAGTGGCTTAATTTAGGAAACA

TGTTAACAGGACACTATGTTTTTGAAATTGTAACAAAATCTACATAAATGATTTACAGGTTAAAAGAATA

AAAATAAAGGTAACTTTACCTTTCTTAAATATTTCCTGCCTTAAAGAGAGCATTTCCATGACTTTAGCTG

GTGAAAGGGTTTAATATCTGCAGAGCTTTATAAAAATATATTTCAGTGCATACTGGTATAATAGATGATC

ATGCAGTTGCAGTTGAGTTGTATCACCTTTTTTGTTTGTCTTTTATAATGTCTTCAGTCTGAGTGTGCAA

AGTCAATTTGTAATATTTTGCAACCCTAGGATTTTTTTAAATAGATGCTGCTTGCTATGTTTTCAAACCT

TTTTGAGCCATAGGATCCAAGCCATAAAATTCTTTATGCATGTTGAATTCAGTCAGAAAAGAGCAAGGCT

TTGCTTTTTGAAATTGCAACTCAAATGAGATGGGATGAAATCCTATGACAGTAAGCAAAAACAGAACCAT

GAAAAATGATTGGACATACACCTTTTCAATTGTGGCAATAATTGAAAGAATCGATAAAAGTTCATCTTTG

GACAGAAAGCCTTTAAAAAAAAAATCACTCCCTCTTCCCCCTCCTCCCTTATTGCAGCAGCCTACTGAGA

ACTTTGACTGTTGCTGGTAAATTAGAAGCTACAATAATAATTAAGGGCAGAAATTATACTTAAAAAGTGC

AGATCCTTGTTCTTTGACAATTTGTGATGTCTGAAAAAACAGAACCCGAAAAGCTATGGTGATATGTACA

GGCATTATTTCAGACTGTAAATGGCTTGTGATACTCTTGATACTTGTTTTCAAATATGTTTACTAACTGT

AGTGTTGACTGCCTGACCAAATTCCAGTGAAACTTATACACCAAAATATTCTTCCTAGGTCCTATTTGCT

AGTAACATGAGCACTGTGATTGGCTGGCTATAACCACCCCAGTTAAACCATTTTCATAATTAGTAGTGCC
AGCAATAGTGGCAAACACTGCAACTTTTCTGCATAAAAAGCATTAATTGCACAGCTACCATCCACACAAA
TACATAGTTTTTCTGACTTCACATTTATTAAGTGAAATTTATTTCCCATGCTGTGGAAAGTTTATTGAGA
ACTTGTTTCATAAATGGATATCCCTACTATGACTGTGAAAACATGTCAAGTGTCACATTAGTGTCACAGA
CAGAAAGCACACACCTATGCAATATGGCTTATCTATATTTATTTGTAAAAATCCAAGCATAGTTTAAAAT
ATGATGTCGATATTACTAGTCTTGAGTTTCTAAGAGGGTTCTTTATGTTATACCAGGTAAGTGTATAAAA
GAGATTAAGTGCTTTTTTTTCATCACTTGATTATTTTCTTTAAAATCAGCTATTACAGGATATTTTTTTA
TTTTATACATGCTGTTTTTTAATTAAAATATAATCACTGAAGTTTACTAATTTGATTTTATAAGGTTTGT
AGCATTACAGAATAACTAAACTGGGATTTATAAACCAGCTGTGATTAACAATGTAAAGTATTAATTATTG
AACTTTGAACCAGATTTTTAGGAAAATTATGTTCTTTTTCCCCCTTTATGGTCTTAACTAATTTGAATCC
TTCAAGAAGGATTTTTCCATACTATTTTTTAAGATAGAAGATAATTTGTGGGCAGGGGTGGAGGATGCAT
GTATGATACTCCATAAATTCAACATTCTTTACTATAGGTAATGAATGATTATAAACAAGATGCATCTTAG
ATAGTATTAATATACTGAGCCTTGGATTATATATTTAATATAGGACCTATTTTGAATATTCAGTTAATCA
TATGGTTCCTAGCTTACAAGGGCTAGATCTAAGATTATTCCCATGAGAAATGTTGAATTTATGAAGAATA
GATTTTAAGGCTTTGAAAATGGTTAATTTCTCAAAAACATCAATGTCCAAACATCTACCTTTTTTCATAG
GAGTAGACACTAGCAAGCTGGACAAACTATCACAAAAGTATTTGTCACACATAACCTGTGGTCTGTTGCT
GATTAATACAGTACTTTTTCTTGTGTGATTCTTAACATTATAGCACAAGTATTATCTCAGTGGATTATCC
GGAATAACATCTGAAAGATGGGTTCATCTATGTTTGTGTTTGCTCTTTAAACTATTGTTTCTCCTATCCC
AAGTTCGCTTTGCATCTATCAGTAAATAAAATTCTTCAGCTGCCTTATTAGGAGTGCTATGAGGGTAACA
CCTGTTCTGCTTTTCATCTTGTATTTAGTTGACTGTATTATTTGATTTCGGATTGAATGAATGTAAATAG
AAATTAAATGCAAATTTGAATGAACATAAATAGAAGTGATTTATTTTTTTATTATCTTAAAGATAGGAAG
GAATTAGTATACGTATCAGGTGTATAAATGGTTTAAATTTATTTTAACATGTCAGATGTGTTCAAGTTGT
CATATAAATAAACTATGTATGTAAGGAATGGGTTAAAA

>NM_020761.3 Homo sapiens regulatory associated protein of MTOR complex 1 (RPTOR), transcript variant 1, mRNA

GTCCTGGCAATATGGCGTCCTCCTTGATGGGCTGATGAGATGAGTTTCACTGTAGCTCCAAACCAGAGGG

CAAAGCTCCCATGACCCAATAAGCCCACATTGTCCCTTTCCTCCGTGGTTCCGTGTCGCCCGTTTCTCAG

GACTCGTTCTCAGGCAGGAGAGAGCCTCGGGGCTGAAGGCCAGGACCAGCCAGGCCGCGCGGACCTGAGG

TTGAGGAACCGGGTGCAGGCGAGCACGATGGGCCGGTCGTGGCTCTGGTTGCAGCAGCTCAGACGAGTGC

GGGACCCGCAGGGCTGAGAGTGGCTGGAGGAGACCCAGGGCCCTTTGAACCCGATCCCTTGGCCGGAGAC

CTCAGCCCAGTCGGCCCAGTGGGCGAACCGGCACCAAGAGCGGCCTGCCTGTCTTCGGAACTGCTGAGGC

GGTGGAGGCCGAGAGCAGGGTCATCGTGAGGCCTGAAGTCTCTTACGCTTTTGGCAGCTCCCCTCGCAGC

CCCTCTGGAAACGTACAGCCTCAGGAGCAGCCAGTGGCTTGGGACCTGGGGTGGTGTGTGTCTGCGGAGC

TTCTTGGGCTGCCCCATTTCCTAGCGGCCCCCACCTCCCCACTTCCCGCTCAGAGTTAGAGATAAGGATC

TCAGACTTTTGCCTGAGTAAGGGTCTCCGCACTCTTTATCCATTTGGTTTTCGATTTCCCGTTTTTGTTT

CTTATTTCACCAATTCTGGTACACGCTAGTTTTTAAGGCTGGAGGTTCTCGAGCGCTTGCTGCCAAGGAC

TCCCCCACCCCCTCCCCCACTGATGGAGTCCGAAATGCTGCAATCGCCTCTTCTGGGCCTGGGGGAGGAA

GATGAGGCTGATCTTACAGACTGGAACCTACCTTTGGCTTTTATGAAAAAGAGGCACTGTGAGAAAATTG

AAGGCTCCAAATCCTTAGCTCAGAGCTGGAGGATGAAGGATCGGATGAAGACAGTCAGTGTTGCCTTAGT

TTTGTGCCTGAATGTTGGTGTGGACCCTCCCGATGTGGTGAAGACCACGCCCTGTGCACGCTTGGAATGC

TGGATCGATCCTCTGTCGATGGGTCCTCAGAAAGCTCTGGAAACCATCGGTGCAAATTTACAGAAGCAGT

ACGAGAACTGGCAGCCAAGGGCCCGGTACAAGCAGAGCCTTGACCCAACTGTGGATGAAGTCAAGAAGCT

CTGCACGTCCTTACGTCGCAACGCCAAGGAGGAGCGAGTCCTCTTTCACTACAATGGCCACGGGGTGCCC

CGGCCCACAGTCAACGGGGAGGTCTGGGTCTTCAACAAGAACTACACGCAGTACATCCCTCTGTCCATAT

ATGACCTGCAGACGTGGATGGGCAGCCCGTCGATCTTCGTCTACGACTGCTCCAATGCTGGCTTGATCGT

CAAGTCCTTCAAGCAGTTCGCACTACAGCGGGAGCAGGAGCTGGAGGTAGCTGCAATCAACCCAAATCAC

CCTCTTGCTCAGATGCCTTTGCCTCCGTCGATGAAAAACTGCATCCAGCTGGCAGCCTGCGAGGCCACCG

AGCTGCTGCCCATGATCCCCGACCTCCCGGCTGACCTATTCACCTCCTGCCTCACCACCCCCATCAAGAT

CGCCCTGCGCTGGTTTTGCATGCAGAAATGTGTCAGTCTGGTGCCTGGCGTCACACTGGATTTGATAGAA

AAGATCCCTGGCCGCCTGAACGACAGGAGGACGCCCCTGGGTGAACTGAACTGGATCTTCACAGCCATCA

CAGACACCATCGCGTGGAACGTGCTCCCCCGGGATCTCTTCCAAAAGCTCTTCAGACAGGACTTGCTGGT

GGCTAGTCTGTTTCGAAATTTTTTATTGGCGGAAAGGATTATGAGGTCGTATAACTGCACTCCCGTCAGC

AGCCCGCGTCTGCCGCCCACGTACATGCACGCCATGTGGCAAGCCTGGGACCTGGCTGTTGACATCTGTC

TGTCTCAGCTGCCGACGATCATCGAGGAAGGCACTGCGTTTCGGCACAGCCCGTTCTTCGCCGAGCAGCT

GACCGCATTCCAGGTGTGGCTCACCATGGGCGTGGAGAACCGAAACCCACCCGAACAGCTGCCCATCGTC

CTGCAGGTGCTGTTAAGCCAAGTGCACCGGCTGAGAGCATTGGACTTGCTTGGAAGATTTTTGGACCTGG

GTCCCTGGGCAGTGAGCCTGGCCTTGTCTGTCGGCATCTTCCCCTACGTGCTGAAGCTGCTCCAGAGCTC

GGCCCGAGAGCTGCGGCCACTTCTCGTTTTCATCTGGGCCAAGATCCTCGCAGTGGACAGCTCGTGCCAA

GCGGACCTCGTGAAGGACAACGGCCACAAGTACTTCCTGTCGGTCCTGGCGGACCCCTACATGCCAGCTG

AACACCGGACCATGACGGCTTTCATTCTCGCCGTGATCGTCAACAGCTATCACACGGGGCAGGAAGCCTG

CCTTCAGGGAAACCTCATTGCCATCTGCCTGGAGCAGCTCAACGACCCGCACCCCTTGCTGCGCCAGTGG

GTGGCCATCTGCCTCGGCAGGATCTGGCAGAACTTCGACTCGGCGAGGTGGTGCGGCGTGAGGGACAGCG

CTCATGAGAAGCTCTACAGCCTCCTCTCCGACCCCATTCCCGAGGTCCGCTGCGCAGCGGTCTTCGCCCT

TGGCACGTTCGTGGGCAACTCTGCAGAGAGGACGGACCACTCCACCACCATCGACCACAACGTGGCCATG

ATGCTGGCCCAGCTGGTCAGCGACGGGAGCCCCATGGTCCGGAAGGAGCTGGTGGTGGCTCTGAGTCATC

TTGTGGTTCAGTATGAAAGCAATTTCTGCACCGTGGCCCTGCAGTTCATAGAAGAGGAAAAGAACTACGC

CTTGCCTTCTCCAGCAACCACAGAGGGAGGGAGTTTGACCCCAGTGCGAGACAGCCCGTGCACCCCCAGA

CTTCGTTCTGTGAGCTCCTATGGAAACATCCGTGCTGTCGCCACAGCCAGGAGCCTCAACAAATCTTTGC

AGAACCTGAGTTTGACAGAGGAATCTGGTGGCGCGGTGGCGTTCTCCCCCGGAAACCTCAGCACCAGCAG

CAGCGCCAGCAGCACCCTGGGCAGCCCCGAGAATGAGGAGCATATCCTGTCCTTCGAGACCATCGACAAG

ATGCGCCGCGCCAGCTCCTACTCCTCCCTCAACTCCCTCATCGGAGTTTCCTTTAACAGTGTTTACACTC

AGATTTGGAGAGTCCTGCTGCACCTGGCTGCTGACCCCTATCCAGAGGTCTCGGACGTGGCCATGAAAGT

ACTCAACAGCATCGCCTACAAGGCCACCGTGAACGCCCGGCCGCAGCGCGTCCTGGACACCTCCTCCCTC

ACGCAGTCGGCCCCCGCCAGCCCCACCAACAAGGGCGTGCACATCCACCAGGCGGGGGGCTCCCCTCCGG

CGTCCAGCACCAGCAGCTCCAGCCTGACCAACGATGTGGCCAAGCAGCCGGTCAGCCGAGACTTGCCTTC

TGGCCGGCCGGGCACCACAGGCCCCGCTGGGGCGCAGTACACCCCTCACTCCCACCAGTTCCCCCGGACA

CGGAAGATGTTCGACAAGGGCCCAGAGCAGACTGCGGACGACGCGGACGATGCTGCTGGACACAAAAGTT

TCATCTCCGCCACGGTGCAGACGGGGTTCTGCGACTGGAGCGCCCGCTATTTTGCCCAGCCCGTCATGAA

GATCCCAGAAGAGCACGACCTGGAGAGTCAGATCCGCAAGGAGCGGGAGTGGCGGTTCCTGCGAAACAGC

CGTGTCAGGAGGCAGGCCCAGCAAGTCATTCAGAAGGGCATTACGAGATTGGACGACCAAATATTTCTGA

ACAGGAACCCCGGCGTCCCCTCTGTGGTGAAATTCCACCCCTTCACGCCGTGCATCGCCGTAGCCGACAA

GGACAGCATCTGCTTTTGGGACTGGGAGAAAGGGGAGAAGCTGGATTATTTCCACAATGGGAACCCTCGG

TACACGAGGGTCACTGCCATGGAGTATCTGAACGGCCAGGACTGCTCGCTTCTGCTGACGGCCACAGACG

ATGGTGCCATCAGGGTCTGGAAGAATTTTGCTGATTTGGAAAAGAACCCAGAGATGGTGACCGCGTGGCA

GGGGCTCTCGGACATGCTGCCAACGACGCGAGGAGCTGGGATGGTGGTGGACTGGGAGCAGGAGACCGGC

CTCCTCATGAGCTCAGGAGACGTGCGGATCGTCCGGATCTGGGACACAGACCGTGAGATGAAGGTGCAGG

ACATCCCTACGGGCGCAGACAGCTGTGTGACGAGTCTGTCCTGTGATTCCCACCGCTCACTCATCGTGGC

TGGCCTCGGTGACGGCTCCATCCGCGTCTACGACAGAAGGATGGCACTCAGCGAATGCCGCGTCATGACG

TACCGGGAGCACACAGCCTGGGTGGTGAAGGCCTCCCTGCAGAAGCGTCCCGACGGCCACATCGTGAGTG

TGAGCGTCAATGGAGATGTGCGCATCTTTGATCCCCGGATGCCTGAGTCGGTAAATGTGCTTCAGATCGT

GAAGGGGCTGACGGCCCTGGACATCCACCCCCAGGCGGACCTGATCGCATGTGGCTCCGTCAATCAGTTC

ACCGCCATCTACAACAGCAGCGGAGAGCTCATCAACAACATCAAGTACTACGACGGCTTCATGGGCCAGC

GGGTCGGCGCCATCAGCTGCCTGGCCTTCCACCCGCACTGGCCTCACCTGGCCGTGGGAAGCAACGACTA

CTACATCTCCGTGTACTCGGTGGAGAAGCGTGTCAGATAGCGGCGTGACCCGGGCCCACCAGGCCACGGC

CGCCTGCTGTACATAGTGAAGCTGTCACTCGCCGGGGCACGGGGCGTCGGCTGCTGCGGCCCCGCAGTGT

GAACGTTGGCTGCTGCCTTAGCTGCTGATGACGGCAGGAGGGCCCTGCTACTCGCTTTTGTCTGTCTTCG

CTGTCGTGTCTGGAATGTCAGGGAAGGGGAGGGCTCGGGTTGACGGTGGCTTCCCACTGAGCACCAGCAT

CCAGGTGCACCCCCGCGGCCACGGCGCCTCTGTCCCTCTCCTGTTCTGTGTTTCTCTGAGACGCTGAAAG

GGGAAACACCTCACTTTATTTCCATGTAATCAGAGCATTAGCTGCAGAAAAACCCCCGACAGAGCCCTG

GCGGAGAGGCAGGCGCTGGGGCTCCTACGGGTCCCTGGGGCAGCTGTCCCCATCAGGCCAAGAGCGAGCG

AGAGGCGCTGCCCCAGCCAGGCCCACCACCTCTCACAGTCAGTGCACGCAAGCAGGGACATTTCCTAGCC

AGCTGGGGGACACTGGAAATTCGGGAAACCAAGAGAGAGGAAGAAGGAGACGCCCCTCCAACTGGCGGGT

GTGAAGGAAGCCGCCCAGGGGTCCGGGCTGTCCTTGGCCGCTGGCAGCATCACTGAGCAGGAAGCGCACA

GCCCACCCTCCCCGCACCTCCAGGTCTCTGGACTCCAGTTTTGGCCCCTCTCACACAGAGCTGTCAGCAG

GGGCCGCTGTGGCGGTGCACAGGGGAGGCAGGTCCTTGGCGAGGTAGCCCCTGCCTTAATCCACGGGGCT

CCTTTCCCTCCGAAGGGCTGCTCTTCCCCACAGGCGCGGGGACAGCAGCCCGACCTGTGGTCTCCATGCC

TGTGCCCTCACACAGGTGTAGCACACGCATGTGCAGATGGCACCACGGCCGGCACCTGGGGGCACACACA

TGCAGGCGGCGTGGTCTCCCTGCTCTGTCCCCACACGTTCCTCACATACAGGCAAGAGGCACTGCCGGGT

CCCGGACGGCTCCGGGTGACACCAGCCCCGTCTCCAGCCTTGAGCCGCCCATGCTGATGCGACCTCGGCT

GACAGCTGGGCCTGTGGTGCAGACAGGAGCTGTGTGGACAGTCCCGCCCAGGAGGGGCCGCAGGGCGTGT

ATGAGCAGTTTTGCAAACAGAACACAACCACAATGATGGTATTTTGAAAAGTGTTCTTTCCGTGTTCGTC

GGGAATCAGGATTATTGAGAGGTGAAGGAGCCAGGTGGCTTCATTCTGGCGGTGAGAGGCCCACGACCAC

GGGAGTGAGAGCTGGTGTGGCGAGGCCCGGCTCTCCTGCGGTGTGGCTGGTGGCCTGCCGTGGCCAAGAG

CATCTTCTGGGTGGATGGAACCCTGCCTGGTCACATTTGGCCAGAGACACACCTGGCCCTCAGGGGGCTG

AGCTGGAGACTGAGCTGGGGCTGGCCGGGACGTGACAAGGCAGGACAGAGGCGGCCCCTCCGCTGCTCCT

TTTTGGAATGCGAGCTCCCACCAGAAGAAGGTTCCGGCACGAATCCCATCCCCACGTCTGGGCCGAGAAA

GCAGCCCGGGTCCGGAAGGTGTAGAGAGTCCCGGCCTCACTCAGCTCACAGGGCGTGCCAGGCGGCAACA

CCAGAATCTTCCAGAAGCCCAGCTCCACCCGCACACGCAGCTTCCCATCCAGTCCTTCAACTCAATTCTT

ACCCAACACGCGTTTCTGTTTGTTTTGAGACAAAATCACCACCTGTCAAAAGGCAGGTGGCTCCAGAGGG

GTCAAGACCCCCCCCCGCCCCCGCTCCACCCTGGAGCCCACCCCCATGGGCACCGCGTGCCGCCTGCACG

TGGGCTGTCTTCACAGGTCTGATGTGAAAATTCAATCACGACGTTAACCGGCTCGAGAGAGCGCCGGCCT

AGAGGCTCATTATCTATTTATTTTACCAAACGCGAATTGAGACGGACTTTGACAAAACACGAAATGGTAA

TGTGAAGCTAAGAGCAGAGAGTGACCAACAGTAAACAACACGCGCAGA

>NM_001145642.5 Homo sapiens rubicon autophagy regulator (RUBCN), transcript variant 1, mRNA

GCCACCGACACTCGCGGGCGACCAGAGCGGCCCCGCGCCGTGACTCGCTCCTAGCGGCCCTCGCAGGATC

GGACGAGGCATTGCAGCCGCTGTGCTAGGCGCTCGCGAGGACCGCCGTTGTGAGCAGTTCGACTTTCCGC

CCAAACTGGGGGATGCTAGACCGCGGGCACCTCTGAATCTTATGCCTTCAGTGATATAATCCTCCGTGTC

CTTCGAGTCCCTCCTGCTGCAGACAGGTCCCCTATACTTCGGTACACACGAGCCTTTCGCGCCCACCGCA

ACGCAGCCTCGGGAGGCAGGACAGACCCCTGCGAGTGTAGATTGCAGCTGCATCCCAGCTCCCCCCTCCC

TGGCACGCTCCTCCACCTACAGCTGCGCTGAGCTGCCGCATCTGAACGCAAAGCAAACGCTGCAGTGACG

GTTCCTGCGTCACCTTAACTCCAAAGTAAATGCTAACGTTTCCCGTGTCTGTACCTTCTCTCAATCGGCA

CTCTCAAAACATCTGGCACGGTGTTCACTACCCTAATAAGACCTCAGCTTCCCTCGGGAGGGGAGGGAGC

ACTGGCAGTTGCTGGGTAATTTGAAGACGACGGTGGAGGGTTTGGTATCAACCAACAGCCCCAACGTCTG

GTCTAAGTATGGTGGCTTGGAGCGGCTTTGCAGGGACATGCAGAGCATCCTCTATCACGGGCTTATCCGT

GACCAGGCGTGCCGCCGCCAGACGGATTACTGGCAGTTCGTGAAAGACATCCGGTGGCTCAGTCCCCACT

CAGCCCTTCACGTGGAGAAGTTCATCAGCGTGCACGAGAACGACCAGAGCAGTGCTGATGGTGCCAGTGA

ACGTGCTGTTGCCGAGCTGTGGCTGCAGCACAGCCTGCAGTACCACTGCCTCTCAGCCCAGCTCCGGCCC

CTGCTCGGGGATAGACAGTATATCAGAAAATTCTACACAGATGCTGCCTTCCTGCTAAGTGACGCTCATG

TCACGGCCATGCTGCAGTGCCTGGAAGCAGTGGAACAGAACAACCCCCGCCTCCTGGCTCAGATCGATGC

GTCCATGTTTGCCAGAAAGCACGAGAGCCCGCTCCTGGTGACAAAGAGCCAGAGCCTGACAGCCCTGCCC

AGTTCCACATACACCCCTCCAAACAGCTATGCTCAGCATTCCTACTTTGGGTCCTTCTCTAGCCTCCACC

AATCCGTGCCCAACAATGGCTCAGAGAGAAGATCTACTTCCTTTCCACTCTCTGGCCCTCCCCGGAAACC

TCAAGAAAGCAGAGGGCACGTCTCACCAGCAGAGGATCAAACCATCCAAGCCCCCCAGTTTCAGTCTCT

GCACTAGCCAGGGATTCCCCTTTGACCCCAAATGAAATGAGCTCCAGTACTCTGACCAGCCCCATAGAGG

CATCCTGGGTCAGCAGCCAGAATGATTCCCCAGGTGATGCCAGTGAGGGGCCTGAGTACCTGGCCATTGG

CAACTTGGACCCCCGAGGCCGGACTGCCAGCTGTCAGAGTCACAGCAGCAATGCCGAGAGCAGCAGTTCC

AATTTGTTCTCCTCCAGCAGCTCCCAGAAGCCAGATTCTGCTGCCTCTTCCTTAGGGGACCAGGAAGGAG

GTGGGGAGAGCCAGCTGTCCAGTGTCCTCCGCAGGTCCAGCTTCTCAGAGGGGCAGACACTCACTGTCAC

CAGTGGGGCAAAGAAAAGCCACATTCGCTCCCATTCGGATACCAGCATTGCCTCCAGGGGAGCTCCAGGA

GGCCCCAGGAATATCACCATTATAGTTGAAGATCCCATTGCAGAATCCTGCAATGATAAGGCGAAGTTGA

GAGGCCCTTTGCCCTACTCTGGTCAAAGCAGTGAAGTCAGCACACCCAGCTCTCTGTACATGGAATATGA

AGGTGGTCGGTACCTGTGCTCAGGGGAAGGCATGTTCCGAAGACCATCAGAAGGACAGTCCCTCATCAGC

TACCTCTCTGAGCAAGACTTCGGCAGCTGTGCCGACCTGGAAAAGGAGAATGCCCACTTCAGCATCTCAG

AGTCCTTAATTGCTGCCATCGAGCTAATGAAGTGCAACATGATGAGCCAGTGCCTAGAGGAGGAGGAAGT

GGAAGAGGAAGACAGTGATAGAGAGATCCAGGAGCTGAAGCAGAAGATCCGCCTTCGGCGCCAGCAAATC

CGCACCAAGAACCTGCTCCCCATGTACCAGGAGGCTGAGCACGGAAGCTTTCGGGTCACCTCCAGCAGCT

CCCAGTTCAGCTCACGTGATTCGGCACAGCTCTCTGACTCTGGCTCTGCTGATGAGGTTGATGAATTTGA

AATCCAAGATGCTGACATCAGAAGGAACACAGCCTCAAGCAGCAAATCCTTCGTTTCCTCCCAGTCCTTC

TCCCACTGCTTCCTGCACTCCACGTCTGCTGAGGCGGTGGCCATGGGGCTCCTGAAGCAGTTTGAGGGGA

TGCAGCTTCCAGCCGCCTCGGAGCTGGAGTGGCTTGTCCCGGAGCATGATGCCCCTCAGAAGCTCCTGCC

CATTCCTGACTCACTGCCCATCTCACCGGATGACGGGCAGCACGCTGACATCTACAAGCTGCGGATTCGT

GTTCGTGGCAACTTGGAGTGGGCCCCGCCCCGGCCTCAGATAATTTTTAATGTTCATCCAGCCCCAACGA

GGAAAATTGCCGTGGCCAAGCAGAATTACCGCTGTGCAGGATGTGGCATCCGGACTGACCCTGATTACAT

CAAGCGACTGCGGTACTGTGAGTACCTGGGCAAGTACTTCTGCCAGTGCTGCCACGAGAATGCCCAGATG

GCCATCCCCAGCCGGGTTCTGCGCAAGTGGGACTTCAGCAAGTACTACGTCAGCAACTTCTCCAAGGACC

TGCTCATTAAGATCTGGAATGATCCTCTCTTCAACGTGCAGGACATAAACAGTGCCCTCTATAGGAAGGT

CAAGCTGCTCAATCAAGTCCGGCTGCTGCGGGTCCAGCTGTGTCACATGAAGAACATGTTCAAGACTTGC

CGACTGGCCAAGGAGCTTCTGGATTCCTTTGACACAGTCCCAGGCCACCTGACAGAGGACCTCCACCTGT

ACTCACTGAATGACCTGACTGCGACCAGGAAGGGGGAGCTGGGGCCCCGGCTTGCTGAGCTCACCAGGGC

AGGGGCTACCCATGTGGAGAGATGCATGCTCTGCCAAGCCAAAGGCTTCATCTGTGAGTTCTGTCAGAAT

GAGGATGACATCATCTTTCCCTTTGAGCTCCATAAGTGCCGGACCTGTGAAGAGTGTAAAGCGTGTTACC

ATAAAGCCTGCTTCAAGTCTGGAAGCTGTCCGCGCTGCGAGCGGCTGCAGGCCCGGCGGGAGGCACTGGC

CAGGCAGAGCCTGGAGTCTTACCTGTCAGACTACGAGGAGGAGCCCGCGGAAGCGCTGGCCCTGGAAGCC

GCCGTCCTGGAGGCCACCTGAAGAAAGCACGTGCAGCCCTCCCTCCGGGCCGGGTCACACCTGTTGCAGA

ACTGAGCCACTCTTTGAAGGACTCGCCCCACCTGGGGCTTCTTTTTTTTTTTTTTTTTTAATTATCATC

ATCTTTTTTTTTTTTTTTACTGACTTGTCTGACGTCTGTGTGCAGTCAGCCGTCGGCAGGTTGATGGGTC

CAGAGTCTGTGGTGACAGATAATTTGTAAACACCAGGTGTTTCCATCAGAACTGACATGCGGGTCCTTCA

GTGAAGCTTCTAGTGCCTCTGTCAGTGGAAGAGACAGCAAGACCAAGTTCTTCCAGCGTCTGTGGCCTTC

TCCTCTAGGTTTCACCTGCATGTCAGGTATCATTTCCAATTTTCCTTTGTTTCAGTTCTGGAGCTTCTGA

GCCAGGCCTTTCTCAACCACCTCTCCTGCTGCTGAAACGGGGATGGCGTTTTCCCTCTCCCTGTCCTGGA

CTGGGGTCAGACTGTGCCCCGAGGAGAAGCAGCAGAGAATAGGACTACGTCATGGGCATTTCGTCCACTT

ATTTGGGTATTTTGGGGGCCACAGAACAATCCTGACTATCCTAGACTCCTCAGAGACCTCAGAGGCAGCT

GTGAATGTCCCTATGTTGCCGGGAGTTCCTGTTTGAAATATTTGAAGCATAGAGGATGCCACAAGCTGAC

TTTCTTCATCTACCTTGGTGATCTTGAAGCAAAGAACAGAACTGATGCTCAGGCCAGGCTCACCTGTAGC

CTTACGCCGCAAGCATACGTGAGGCGCCAGCTCTGTCGCTGAAGGAGCGCTTACTCAGAGGAGCGGTCGG

CCCCCTCTTGGTGTTAAGGTCTCTTAGTTAACCTGGCTTTTTGGTGCAGGTGTGATCTTTGAAGCTCAGG

CAGGTCCCTGATGCCATCCTAAGGTGAGGACAGGAACCTCACCCACCATCTTCTTAGCGTGTCCCTGATG

ACTCTGTCCTCTGTTAGATGGTCGTTGTGCTTCTGAGTAAAAGTACAACCCGACTCCGTTCTCTCCCCTT

CCTGCAGCAGAGCTGGGTCCTTCCCTGGTGGCCGAGTCTCTCTTGCCTTAGCTTCTTTGGTCAAAGTTGG

AGAAAAGCTTCCTGCTATTAGTGCTGTTACAGAACTTGACGGTTTGTGGATGTGAGTGTGAATGTCCCTG

TGTTCTTGGGATAACAAGAGCCTTTATGCCAATTATGCACTTAACTCTGTGTAGCCTGGTAATGTTTATC

TGTTCATTTGATAATGCTGATTTTAGTGTGCTGCCCCCCTCCCCCCGTTAATGTGTGTTGATGGTGAAGT

CCTTTTGATAATGCTGATTTTGGTGTGCTGCCTCCCCCTTCCCCCCCGTTAATGTGTGTGTTGACAGTGA

AGTCCTTGGGTGGGGCCATGTGTGTGTTTGTGATGTTCCTTAAGTTGATGCAGCTTCTAACCTCTGTGAA

AACACTGGTCAGAGTGGCTTCTCCAAGAGCTGGCAGCTCTGTGAACTAAAGCCTGCATCATTTTTGTTCT

GGGATTGAATTCTGCCCATGGGCATGTCTTCTCATAGTTGCTTGCTGGTAGGAAAGAAATGGGCGTGGGT

GCTGCCCTGGAAGCTGAGCGGAAAGTTGCCTGTGGTTGGTGGAAGCTGATGAGAGCTTGAGCTGGCGGTA

AGAAGGAGTCTCCCAGGGAAGTGGGAGAGGCATTAAGGTGATGGCCAGGGCTGAGGCTCCACCAGCGTGA

GAGGGAACATGTGGGAACTGGCCCCTGCCCTTGATTCCTCTGCCTCAAAGTTGGGATCTGAAAGCCATGT

AGGGCTAGAAGACCCTGAGGCTGTTCTCCCTTCTGTTCATAGTGAGACTCAAAAAGCCAAGTCCCAGAAG

TTCTGAAGGGCTGTGACTAGAAGTGCCCAGGTCCTTCAGGGAGCTTTAAGAATGACCCCACAGAACTCAA

GTTTAACTAGGGGTTAGGTCCCAGATTCAGACCCAGGAGTTTATAAAAATGAGCTCTACTTCCAGTTTTG

GTTTAAATTACACATCCAGGCCAGGCACAGTGGCTCACACCTGTAATCCCAGCACTTTGGGAGGCCAGTG

CGGGCGGATCATGAGGTCAGGAGTTTGAGACCAGCCTGGCCAATGTGGTGAAACCCTGTCTCTTCCAAAA

ATACAAAAATTAGCTGGGCGTGGTGGCACACGCCTGTAATCCCAGCTACTTGGGAGGCTGAGGCAGGAGA

ATCGCTTGAACCTGGGAGGCAGAGGTTGCAGTGAGCCGAGATTGCGCCACCGCACTCCAGCCTGGGTGAC

AGAGTGAGACTCCGTCTCAAAAAACAAAAAGGTGACACATCCAGCTCTTTCTCCAGGTCACTGCGCTGGA

GGACAGATGTGCCGTCTTGTCCTGCCTGTTTCACATCAGCATAGGATCAAAGGATGACAATGCTGACAGC

TTCTGAAGCCGAACTCAACAGTCTCATAGGCTCCTCACTTGTCACTTATTTTTCCCTAGCTCCCTCAACC

GCACCCCATCCCTTTAGATCGTGCGTCTGTTTTAGTGACTCTGACACGATGCCGTCCTCACCTTCCAAAT

ACCCAGTTATTTATTCAAGAGGGGGGAAGTGGGTAGAGGATGGGATGTTTTGGAAGCACTTTGCAAGTTA

CCACTATCTGAAAATCCCCTGCTGTTGCGGGGAGAAGCTTTGAATGCACTGAAGAGAATTCCTTCTAAAT

GAAGGCAGGTGATAGTGTTCTTTCTGTAAGTAAAGGGAAAGAAAAAAAACATAGTTTGCTTACCAGGTGG

AGACAAGATTCAAGACATAGCAGAAGAGTGGAAGACAAATATTTTCCACTTAAATGAGGCTGTTTTTGAC

GTTCTCTGCCAAGGATTTAGAGCTTTCGTTGAACTAACATAAAAGGAGTGCGAGTCTTAGTAGAGATGTT

CCGTGTGTGCCGCCCGTGCTCTGAACTGCGTTTCCACCTGCTGTGGTGCTTGTGCAGCCTGGCAGTTCAT

TGTCATCTTTAATAAACTAAGGAAATAAAATTCTGTCTTTCGTCAGCCTGCCTTGACTGTCAAGAAATGG

CATGTGTCCACTGTAAAGCAGGTATATCTGAACATTGAGAAGGTTTCTGTGTTATAATGTAAAGCAAACT

GGACCACACACATCAAGGAAAGTGAGGTGGAGTGGCTGTGTCTCTAACTTGCCATGAGACCTCGAGCGAA

CCACTTCACTTCTCTGGGTTTCTGGTGCATGGTGTAACTGTCACTTAAAATGAGGTGTCTTCCTCTCCGC

AACATCTGACTTGCCTTAGATGCTTCAGTTCCCACTTCTGTGAGCAGCTAGCATCTTTCTCTTCTGCTCC

TGGCCCACTGGAGCTCCAGCTCTACGCTGCAACTCTCTCAACATTTCCCCTTAAATGTCCCCTTGTCATC

TCCAACCAGTGTGTCTAAAACCAGCATAAATTCCGTATTTCCAAAAACCTAACCATTAGTCACCCAGGTT

TGAAAATTAATTGTCTTCAACTTCCTCTGTAAGTGGCACACTGAGCTCATCACGCATCGTCTCATTCCTA

CTCCCTGTCATCAAGTTCTGTGGATGTTTTCCTCTAAAATAAATCTCTCATTACTTTTCTGCCACTAGCA

CACAAATTCAGGTGTTCTTGATCATACATTATTTTCCTCCCTGCGCCTCAGTGGAACAATGATTAGAAGC

ACCTTGTGGGATGAGGAGCCGGGCGCGGTGGCTCACGAGTGTCATCCAGTACTTTGAGAGACCAAGGCAG

GGGGATCACCTGAGGTCAGGAATTCAAGACCAGCCTGGCTAACATCACAAAACCTCATCTCTACAAAAAT

ATAAAAATTAGCCAGGCATGGTGGCATGTGCTTGTAATCCCAGCTACTCGGGAGGCTGAATCATGAGAAT

CACTTGAACCTGGAAGGCGAGGGCTGCAGTGAGCCAAGCTTGCTCCATTGCACTCCATCCTGGATGAAGA

GCGAGACCCTGTCTCAAAAAAACAAAACAAAACAAAAAAAGCACCAGCACACTGTGGCATGAGGGGCGGG

ATTGTGACCACCTCTACCTCCCCTCTTAGTGCTCACCAAGTCTCTGCAGTAGTGAGCGTGTCCTCATCTC

CTGTCTTCTCCCTCAGCTACTTTGTCTGCAACTGCTCCTGTTGTGTTTGTCCAGCAGTCATCATCAGAGG

ACACACATCCCAAATGTCCTTCAGTCACTTTATTATTAAACTGCCAGCTCTTTCCTTGCCAGGGACTAAA

CCAGGAGATAAGATGATATTTATATGCTGTAAACACACCCTACATATTTAAAAATCTTCATATTTACAAA

TTAAACTGAGAGGAGAATGAAAAAGCCATGTGAAAGACTGCTGAAGTTGATAAAATGTTCAAATGTTTTT

CAACTAGAGATAGGAAAATAGCTTACAGTACAAAACCTTCACTTTGTTCTAAGGCCCTTTTGTTCATTGT

TACAAACTGGTGTAGTTCACAGGAAGCCAAAGCTTGATTTCCAGTAAGTTCCCAAACTTCTGTTCTCAAC

ATTTAACTTAGATTTTAGATGATCATATTCCAAAATTGGAAAGACTGTTGAAGAGGAAAACAAGGACCAT

TGATTCACAATGGCAGCCCAGCATTTATTTATTCATTCAATCATCCGTCAATCCCAAACATCTATAGATG

CCTCCGTGTTCCAGACACCATGCCAGAGCTGAGAGTACAAAGAATCACAGAATATTGTTTAAAGATGTTT

ACTGCAGCATAATTTATGAAAGTAAAAAGTTAATGGAAAATAAAAAAATTGATGCAACCATCAAAATGAC

AGTTATAAAAATTATGATAGTGAGCCGCACACAGTGGCTCACGCCTGTAATCCCAGCACTTTGGGAGGCC

GAGGCGGGCGGATCACGAGGTCAAGAGATTGGGACCATCCTGGCCAACATGGTGAAACCTTGTCTCTACT

AAAAATACAAAAGCTAGTTGGGCATGATGGCGGGCGCCTGTAATCCCAGCTACTCGCGAGGTTGAGACAG

GAGAATTGCTTGAACCCGGGTGGCGGAGGTTGCAGTGAGCCGAGATCACGCCACTGCACTCCACCCTGGG

CAACAGAGTGGGACTCTGTCTCAAACAAAAAAATAAATTATGATAGTAACATGGGGAAAAAGTCTTGTAA

TACAATGTTATGTGAGAAGAAGTTAGAAAAAGTGTATGTCAAATATATATTTATTAGTCCATACATACAT

AAATAAATGACCAAATAAATAAATTCTCACAGAAGAATTCTAAACAAAATACGTAAATTCTCCCAGGAGG

TGGAGTTTAATTCCTTCTCCCATTGAGGGTGGACTGGAATTAGTGATTCACTTTTTTAAATAAAAGGATA

TAGAAAGAGGAAAATAGCAACTTTAGAGTAGAGAGAGCTAACACGACCTTAACCAAGTAATCAACGTTAA

CATCTCCAAAGATGTCATTGGATGTCATGTACCTACTAATACAATGAAAGGGGTACATCTTCCCTGACAT

TCATTCAGAAACCCATAACCTCAGTCTAATCATGAGAAAAACAACAAACAACATAAACACAGGAGCAAAA

TTCGAGGAACTTTCTACAAAACACTTGACCCTACTCAAAATTGCCAAGGTCTCGAAAACAAGGAAAGAGA

TGCCGTCACGGACGAGAGGAGACTAAGCAGATACGATGACTGAATGCCATGTGGGATCCCGGGTGGGATC

CCGAAATGGAGAAAGGATATTAGTGAAAAAACTGGCAAAATCTGAATACAATTTGGAGTTTAGTTAAAAG

TAACATACCAATGTTGGTTCCTTAGTTTTGGGAAATGTCCAGTACTACAGTAATGTAAGATGGTAACATT

AGGGAGAGCTGAAATCGGGTGAGGGCTATGTGACGAATTTGTACTATCTTTGCAACTTTACTGTGGATCT

AAAATTATTCCAGATTAAGAAGTTTACCTATATAAAAAGCATATGTACATCATAATTACAGTTATGCAAA

AATATGGTTAGAAAAAATACATAAGGAAAACATACGTAAAAATTAGTGTGGTTCTGTTAGAATGGTGGCA

TTGTGGAGTATTTTTTCCTCTTATCTGCTTACTGAATTAGCTGTAAATGAGTCATTTTCCATAAATTAAA

GAGTTAATTTTA

>NM_001142648.2 Homo sapiens secretion associated Ras related GTPase 1A (SAR1A), transcript variant 1, mRNA

GTACATCCGGCGAGTAGCTGGCGGTCCCGGGTGCTGCTGGTTAGTGTGCTCTGAGGGAGGGTCCGAGCCA

GCCGCTGTTTTGCCGGAGGAGCCCCTCAGGCCGGCTGCTTCCAACCTTCAATTTGTAAAAAAACCAGAAA

ACCTCAGGATCCACAAAGTATCATCAAATGAGGTAGTAAGCATTAATAATGTCTTTCATCTTTGAGTGGA

TCTACAATGGCTTCAGCAGTGTGCTCCAGTTCCTAGGACTGTACAAGAAATCTGGAAAACTTGTATTCTT

AGGTTTGGATAATGCAGGCAAAACCACTCTTCTTCACATGCTCAAAGATGACAGATTGGGCCAACATGTT

CCAACACTACATCCGACATCAGAAGAGCTAACAATTGCTGGAATGACCTTTACAACTTTTGATCTTGGTG

GGCACGAGCAAGCACGTCGCGTTTGGAAAAATTATCTCCCAGCAATTAATGGGATTGTCTTTCTGGTGGA

CTGTGCAGATCATTCTCGCCTCGTGGAATCCAAAGTTGAGCTTAATGCTTTAATGACTGATGAAACAATA

TCCAATGTGCCAATCCTTATCTTGGGTAACAAAATTGACAGAACAGATGCAATCAGTGAAGAAAAACTCC

GTGAGATATTTGGGCTTTATGGACAGACCACAGGAAAGGGGAATGTGACCCTGAAGGAGCTGAATGCTCG

CCCCATGGAAGTGTTCATGTGCAGTGTGCTCAAGAGGCAAGGTTACGGCGAGGGTTTCCGCTGGCTCTCC

CAGTATATTGACTGATGTTTGGACGGTGAAAATAAAAGAGTTTTACTTCTCTGGACTGATCCTATTCACA

GCTTCCTCATGAACTTTTCTAATAGAACAAGGAAAGCTCTCCAACCATGTCTGGCGTTGAGAAGCCAAGA

GTCTCTGTCAACTCTCTCATTGCCCAGTGGTGACATGTGCTCTTCTCCACACTGTTGGGAGGTAATGCTG

CCCCACGTGCTGGTGCAGGTCAGTATCCTGGGACTTGGAAGCTGGCAGGATTTGCCGGGTAAAGCTGTAT

GCCATCATGGGGCACCTGAAAAGAAAAACACGTCTCACCACTGTGGTTGATTCAAAAGAAAGTGATTCTA

TTTTTTAAAGAAAGCGTTGTTAATGTAATTGGTATCCCTCCTAACTTTTTGAGTTCACAATTTACTTGGT

CCAGAGTTTTCTATTCTTTTTTTTTTTTAAACTAATGAATGACATTTAGATACTTCATAAAATTATGAAC

AGATATGGAGGCCAGAGCTCATTTGGGTAAACTTACTCCTGCTGAGTTAGCAGGTTGGTGAGAGAAGCTC

CCCTGAGCTCACCTGTCTCTCTGACTGCCTTGGAGTAGGTGGCATAACCTTGTGCACAGAGAACTAGAAA

AGGGGCAGAACCCCGGCCTTGCAGTTGTGGCAGGTTTCCACTGTGGTAAGCTAGGTTCATTCCTCATCAA

GGAATGTGTAGCAGATTGTTCACTGTGGAGGAGTTAATTATAGAATGGGTTATTGTTGTTATTCTTACTC

ATGAAGTTACAGATTTTAGCCAGTCTTTGCTTTTATACTTTTGTGAAATTTAATTTCTCTCTATAGCACC

TTCCTTTTTCGTTTTCAGTTATCAAAAGTGACTTTGACCTCATAAAAGAGTTGAGAACATCTCTCGTGTC

ACATACTGCAGGTGCATCAGTTACTTTTGCACAGATTCTAGGGGGACATTTTTCTGAATAGGAAGACAGG

ACAAAGTTAACAGCTTAAGGGCTCTTAATTCTGTGAGTTGAGGACTTAAAAGTATTGTAGCATTTGTTTG

GATCCATGAAAAATGTATTCAGTGGGCTTTAAAATTTCCATTTGCAGAATTTGGTCTCTCAGGCTGTTTG

GGAGCTCTTTTTTTTACATTTTTTCTCCTTTGACACCTATTTTATTGGTGTTTAAAGTAAAGGTTAACAT

CTGTAGCTTTTCCAGGTTTTTTTTTTTTTTTTTTTTTTTTTGGTATGAAATTGTCTTTCTCCATTGCAGA

AATAAGCTAGGGAAACACTAACCCAAAAACTTTCTGTAGAGCTGTTCCTTTGGAGGCAGCATCACTTATT

GGCAGTAAAGACTCAGTATAAAAGCACCGGCATCCCTACTTGGGTGATGGGGATTAATTTTATAGCATTC

CATTTTCCTAGTGCCACATGTGAAATTGGATTTTGATGATCTTAATCTATATTCTACCCTTATAATAAAA

GATCAAAAGATATATCTCCTATGAACAGATTGGAGATAGGAGATGAAAAGTTGGGAGGATGTCTTTATTC

TAATGTGAGGGTAGGGAAAATGTGGATAACATTACTGGGGTGAGGGAGGCATTGTTCTTTAGTTGGAGTT

CTCATTCTTATTCTCCAGTACTGACTTGTGGGGAAAGCATACTTTTTCACTGCCAGGTACTGAATGCAGA

GGCTCAGTGAAGTATATATGTGGGAAGTGCATGCATTTCGTTTATTAGCAAACATAGCTGGATTAAGACA

AAGTTGTTGGTTTGGAAAGGGGTTAAAGCCTTAAGTGAACAAATCTAGCTAACAGTGAATGAACTAGGTA

ATATAACTTGCATATTTTTAATTTCCTTTGGTTAAAGGTCCCCCATACTTCTCTGTTCGGAGACATGAGA

AGTATGATTACTTCAGTGTTAGTTTTCTTAATTTTTTTTTTCCCCTATTTGTCCCTTGTCACTTTGTTGC

AAGCTAGAAATCTGTGGGTTATACATAGGGCAGCTCTTTGTGAAAGTGGTTTATTCCACTGGAGAAAGGG

GATTGAAAATCAGTTAGAACCAATGTATTTCTTGCCCCACGGAACACTATTCCTATAAGATAGCTGAAAG

AAGCTGCTGTGAGGAGCTCAGCTCCAAACACAGGATCAGCACCTTGTATAGGAATTCCCATGAATTATGA

CTTCTCATTCTGTTTTATCAGAGTGCATATATGTCCTACTTCAGGAAAAGTAAAACAGTCATTTACGAAA

GAAAGTCAATCTGTATCCTAAGCATTTTAATAAAAAGTTAAAACAAATACGCTATTTCCTTTTTTTTTTT

TCATTGGTTTGTTCTGAGCTCTGAGTTTGAACAATGCCGTTTATTTGGGGAGGCAACCAAAAGTTCCCTG

CATAGAGGAAGAGACATTTAGGGTGCGTGTGAGATACTTGAATTTTGCCACCAGGAGGGGGAAAAAGGCT

GGGGTACTAGAAGTAATAGATTTAGTACTACTTGAGAGATTTGGAACATGCTGTGGACCCAGGGTTGTTG

GTCATCTCTGCAACAGGATGGGGATGGCAGATGTTTGAGCAAAATCTATATTAACATGCTAGCAAGGACT

AGTTCTAGTTTAGCTGTGTTTCACTCATTTTACACAGAATTAGAATTGGCCAGGTGTGGTGGCTCACGCT

GTAATCCTAGCACTTTGGGAGGCTGAGGTGGGTGGATTGTCTGAGCTCAGGAGTTTGAGACCAGCCTGGG

GAACAGGGTGAAACCCCATCACTACTAAAATACAAAAAAATTAGCTGGGCATTGTGGTACATGGAGTAGC

TCCCAGCTACTCTGGAGGCTGAGGCGGAGAATCGCTTGAACTAGGAGGTGGAGATTGAAGTGAGCCGAGA

TCATGCCACTGTACTCCTGCCTGGGTGACAGAGCAAGACTCTCTCTAGCTCAAAAAAAAAAAAAAAAAAA

AAATCAATTAAATGCTACATTTGGCAAAATGAGAAAGAAAGAATTTCCTTTACTAGGCTTGTAGGAAGAC

TGCTTTTACAAAAACCCAAATGAGATCATAAAACTAAAGTACTGGGACAAACAGTGGGAAACAGTGCAAT

GGGTTCTTTTCAACCAAGTATTAAAGTATTGGAGATATTGCCGGGCGCGGTGGCTCACGTCTGTAATCCT

AGCGCTTTGGGAGGCCAAGGCGGGCGGGTCATGAGGTCAGGAGTTCAAGGCCAGCCTGACCAACATGGCA

GAGCTCCGTCTCTACTAAAAATACAAAAATTAGCCAGGCACGGTGGGACGCACCTTTAATCCCAGCTACT

CAGGAGGCTGAAGCAGGAGAATCGCTTGAATCGGGGAAGCGGAGGTTGCAGTGAGCCGAGATCGCGCCAC

TGCACTCCAGCCTGGGTGACTCCCATCTCAAAAAAAATAAAAAGTACTGGAGGTGTCCTGTTGTCCTTGT

TACTGTGAAAGGATGCCATGGGTTGTATGGGCAAATGAGTTGTTTTGTTTGAGGCAGAGTGGAAAATAAG

ATTAGGCTCAGTCCATGAGCCTAGCACTCAACAGATCTTACTTTGGCGTTCTGGCTGTGACTTGGGACTT

CTTTTTACCATGGTCTGCTGTAAACACAGAAAAACCCAATGCCTAACAGTTCATGCTGGAGTCTTGAGCG

TTGAGGGAGAATCACTGGACTCGAGAGAGAAAGTTTGTTTGTTTGTTTGTTTGTTTGTTTTTGAGTTGG

TCTCACTCTGTCGCCCAGGCTGGAGTGCAGTGGTATGAACATAGGTCACTGCAGCCTTGACCTCCTGGGC

TTAAGTGATCCTCCCATCTCAGCCTCCCAAGTAGCTTGGGACCACAGGGTTGCACCACCGTGCTCAGCTA

GTTTTCAAAATTTTTGTAGAGTTAAGGTCTCATCATGTTGCCCAGGCTGGTCTCAAACTCCTGGGCTCAG

GAAATTCTCCTACCTCAGCCTCCCAACGTGCTGGGATTCCAAGTGTGAGCCCCTGTGCTCCACCAAGAGA

AAAACTCTTTGATTGCTAGGAAGTTGGGATAAAGAAATTGTGCTGTGATCAAGCCACCTTCTGTGTCATA

GAACCAAAAGAAGACCGCCTGCTATGGCAAGATGGCCTAATTTTATTCCTGCCTCATACTATCATGGATA

ATCGTTTTCCTTTCTCTAGCAGGTTTGTGTCACTGGGAGTACTGGGAGCATTATCCTGTGTTGTGCTGGG

GTTATACCAAGCAGTGTTAACTTTATTTAAAGAATTCAGGCAGAAAGATTAGAGAATTTAAGTAGCAATT

GTATATGGTAAGCAATACAGTAACCTTAATAAAAATTAATGATGGCCGGGTACGGTGGCTCACGCCTGTA

ATCCCAGCACTTTGGGAGGCCGAGGCGGGCGGATCACAAGGTCAGGAGATCGAGACCATCCTGGCTAACA

CGGTGAAACCCCGTCTCTACTAAAAATACAAAAAATTAGCTGGGCGTGGTGGCAGGCGCCTGTAGTCCCA

GCGACTGGGGAGGCTGAGGCAGGAGAATAGCGTGAACCCAGGAGGCGGAGTTTGCAGTGAGCGGAGATCG

CACCATTGCACTCCAGCCTGGGCGACAGAGCGAGACTCCGTCTCAAAAAAATAAATAAATAAATAAAAAA

TAATGTGTATTTTATCAATTCAGTGATAAAGCAGACCTTTGTAAACTGTCTAGCTTGTGGTGACTGATTG

AGGGTATCATGAAGCTCCGCGCCCCCCCACTCCCCGACATACTCGTTCATCCTCCTCTGTCCTAAAGCGT

ACTTCTTTGGAAAAGTAGAGCAGTTTATGTTAGTGTACACTAGAGATGGTAAACTATGCCTTGTGGGTCA

AATCGGGCCTGCTGCCTGTGTTTGTATGGCCTGTGAGCAAAGAAAGGTTTTTACATATTTAAATGGTTGG

AAAAAATATTTAAAGAATGCTATTTTTGGACACAAAAAATTGTATGAAATTAAAATTTCAATGTCTGTAT

CTTTTTATTGGGACACAGCCACCGCCATTCCTTTAGCTTTTGTATGCAACAGCTGCAGAGTTGACTATTT

GAGACAAGTTGTATGGTCCCCCCAAACTGGAACTGTTTCCATTCTGGCCTTCTACAGAAAGTATTTGGAC

CCTGGCCTAGATAAATCCTGGCTGGTGGTTATGTTATGTGAAATGTGCACACTGTAAAATATAGGCCTGT

ATGATTAAAATTTTTGATTTCA

>NM_001002235.3 Homo sapiens serpin family A member 1 (SERPINA1), transcript variant 3, mRNA

AGAGTCCTGAGCTGAACCAAGAAGGAGGAGGGGGTCGGGCCTCCGAGGAAGGCCTAGCCGCTGCTGCTGC

CAGGAATTCCAGGTTGGAGGGGCGGCAACCTCCTGCCAGCCTTCAGGCCACTCTCCTGTGCCTGCCAGAA

GAGACAGAGCTTGAGGAGAGCTTGAGGAGAGCAGGAAAGGACAATGCCGTCTTCTGTCTCGTGGGGCATC

CTCCTGCTGGCAGGCCTGTGCTGCCTGGTCCCTGTCTCCCTGGCTGAGGATCCCCAGGGAGATGCTGCCC

AGAAGACAGATACATCCCACCATGATCAGGATCACCCAACCTTCAACAAGATCACCCCCAACCTGGCTGA

GTTCGCCTTCAGCCTATACCGCCAGCTGGCACACCAGTCCAACAGCACCAATATCTTCTTCTCCCCAGTG

AGCATCGCTACAGCCTTTGCAATGCTCTCCCTGGGGACCAAGGCTGACACTCACGATGAAATCCTGGAGG

GCCTGAATTTCAACCTCACGGAGATTCCGGAGGCTCAGATCCATGAAGGCTTCCAGGAACTCCTCCGTAC

CCTCAACCAGCCAGACAGCCAGCTCCAGCTGACCACCGGCAATGGCCTGTTCCTCAGCGAGGGCCTGAAG

CTAGTGGATAAGTTTTTGGAGGATGTTAAAAAGTTGTACCACTCAGAAGCCTTCACTGTCAACTTCGGGG

ACACCGAAGAGGCCAAGAAACAGATCAACGATTACGTGGAGAAGGGTACTCAAGGGAAAATTGTGGATTT

GGTCAAGGAGCTTGACAGAGACACAGTTTTTGCTCTGGTGAATTACATCTTCTTTAAAGGCAAATGGGAG

AGACCCTTTGAAGTCAAGGACACCGAGGAAGAGGACTTCCACGTGGACCAGGTGACCACCGTGAAGGTGC

CTATGATGAAGCGTTTAGGCATGTTTAACATCCAGCACTGTAAGAAGCTGTCCAGCTGGGTGCTGCTGAT
GAAATACCTGGGCAATGCCACCGCCATCTTCTTCCTGCCTGATGAGGGGAAACTACAGCACCTGGAAAAT
GAACTCACCCACGATATCATCACCAAGTTCCTGGAAAATGAAGACAGAAGGTCTGCCAGCTTACATTTAC
CCAAACTGTCCATTACTGGAACCTATGATCTGAAGAGCGTCCTGGGTCAACTGGGCATCACTAAGGTCTT
CAGCAATGGGGCTGACCTCTCCGGGGTCACAGAGGAGGCACCCCTGAAGCTCTCCAAGGCCGTGCATAAG
GCTGTGCTGACCATCGACGAGAAAGGGACTGAAGCTGCTGGGGCCATGTTTTTAGAGGCCATACCCATGT
CTATCCCCCCCGAGGTCAAGTTCAACAAACCCTTTGTCTTCTTAATGATTGAACAAAATACCAAGTCTCC
CCTCTTCATGGGAAAAGTGGTGAATCCCACCCAAAAATAACTGCCTCTCGCTCCTCAACCCCTCCCCTCC
ATCCCTGGCCCCCTCCCTGGATGACATTAAAGAAGGGTTGAGCTGGTCCCTGCCTGCATGTGACTGTAAA
TCCCTCCCATGTTTTCTCTGAGTCTCCCTTTGCCTGCTGAGGCTGTATGTGGGCTCCAGGTAACAGTGCT
GTCTTCGGGCCCCCTGAACTGTGTTCATGGAGCATCTGGCTGGGTAGGCACATGCTGGGCTTGAATCCAG
GGGGGACTGAATCCTCAGCTTACGGACCTGGGCCCATCTGTTTCTGGAGGGCTCCAGTCTTCCTTGTCCT
GTCTTGGAGTCCCCAAGAAGGAATCACAGGGGAGGAACCAGATACCAGCCATGACCCCAGGCTCCACCAA
GCATCTTCATGTCCCCCTGCTCATCCCCCACTCCCCCCCACCCAGAGTTGCTCATCCTGCCAGGGCTGGC
TGTGCCCACCCCAAGGCTGCCCTCCTGGGGGCCCCAGAACTGCCTGATCGTGCCGTGGCCCAGTTTTGTG
GCATCTGCAGCAACACAAGAGAGAGGACAATGTCCTCCTCTTGACCCGCTGTCACCTAACCAGACTCGGG
CCCTGCACCTCTCAGGCACTTCTGGAAAATGACTGAGGCAGATTCTTCCTGAAGCCCATTCTCCATGGGG
CAACAAGGACACCTATTCTGTCCTTGTCCTTCCATCGCTGCCCCAGAAAGCCTCACATATCTCCGTTTAG
AATCAGGTCCCTTCTCCCCAGATGAAGAGGAGGGTCTCTGCTTTGTTTTCTCTATCTCCTCCTCAGACTT
GACCAGGCCCAGCAGGCCCCAGAAGACCATTACCCTATATCCCTTCTCCTCCCTAGTCACATGGCCATAG
GCCTGCTGATGGCTCAGGAAGGCCATTGCAAGGACTCCTCAGCTATGGGAGAGGAAGCACATCACCCATT
GACCCCCGCAACCCCTCCCTTTCCTCCTCTGAGTCCCGACTGGGGCCACATGCAGCCTGACTTCTTTGTG
CCTGTTGCTGTCCCTGCAGTCTTCAGAGGGCCACCGCAGCTCCAGTGCCACGGCAGGAGGCTGTTCCTGA
ATAGCCCCTGTGGTAAGGGCCAGGAGAGTCCTTCCATCCTCCAAGGCCCTGCTAAAGGACACAGCAGCCA
GGAAGTCCCCTGGGCCCCTAGCTGAAGGACAGCCTGCTCCCTCCGTCTCTACCAGGAATGGCCTTGTCCT

ATGGAAGGCACTGCCCCATCCCAAACTAATCTAGGAATCACTGTCTAACCACTCACTGTCATGAATGTGT

ACTTAAAGGATGAGGTTGAGTCATACCAAATAGTGATTTCGATAGTTCAAAATGGTGAAATTAGCAATTC

TACATGATTCAGTCTAATCAATGGATACCGACTGTTTCCCACACAAGTCTCCTGTTCTCTTAAGCTTACT

CACTGACAGCCTTTCACTCTCCACAAATACATTAAAGATATGGCCATCACCAAGCCCCCTAGGATGACAC

CAGACCTGAGAGTCTGAAGACCTGGATCCAAGTTCTGACTTTTCCCCCTGACAGCTGTGTGACCTTCGTG

AAGTCGCCAAACCTCTCTGAGCCCCAGTCATTGCTAGTAAGACCTGCCTTTGAGTTGGTATGATGTTCAA

GTTAGATAACAAAATGTTTATACCCATTAGAACAGAGAATAAATAGAACTACATTTCTTGCA

>NM_031459.5 Homo sapiens sestrin 2 (SESN2), mRNA

GCTGGCAGTTCCGCGGCGGGGATGCTGAGGAGCGCTGGGTCCGGGAGCAGCCCTGGCCCCTGCGGACTTC

CGAGGCCGTGAAAACCCCTGCGCTGCGGCCCTTCCCAGGCCCCCGAGGCCGTTCGCCGTTCCCGAAGCCC

GACTGGGGGAAGAGTCCAGCACCAAAGCGGCCGTTCTCGGATTCCGGAGCGTTCTGGAGCCCCGAGAGAC

GCCCCGGGGTTCTAGAAGCTCCCCGGCGGCGCCCAGTCCCGGCTTCATTCGGGCGTCCCTCCGAAACCCA

CTCGGGTGCACGGGTCGTCGGCGAGCCGCGACCGGGTCCTGGCGCGCACCATGATCGTGGCGGACTCCGA

GTGCCGCGCAGAGCTCAAGGACTACCTGCGGTTCGCCCCGGGCGGCGTCGGCGACTCGGGCCCCGGAGAG

GAGCAGAGGGAGAGCCGGGCTCGGCGAGGCCCTCGAGGGCCCAGCGCCTTCATCCCCGTGGAGGAGGTCC

TTCGGGAGGGGGCTGAGAGCCTCGAGCAGCACCTGGGGCTGGAGGCACTGATGTCCTCTGGGCGAGTAGA

CAACCTGGCAGTGGTGATGGGCCTGCACCCTGACTACTTTACCAGCTTCTGGCGCCTGCACTACCTGCTG

CTGCACACGGATGGTCCCTTGGCCAGCTCCTGGCGCCACTACATTGCCATCATGGCTGCCGCCCGCCATC

AGTGTTCTTACCTGGTAGGCTCCCACATGGCCGAGTTTCTGCAGACTGGTGGTGACCCTGAGTGGCTGCT

GGGCCTCCACCGGGCCCCCGAGAAGCTGCGCAAACTCAGCGAGATCAACAAGTTGCTGGCGCATCGGCCA

TGGCTCATCACCAAGGAACACATCCAGGCCTTGCTGAAGACCGGCGAGCACACTTGGTCCCTGGCCGAGC

TCATTCAGGCTCTGGTCCTGCTCACCCACTGCCACTCGCTCTCCTCCTTCGTGTTTGGCTGTGGCATCCT

CCCTGAGGGGGATGCAGATGGCAGCCCTGCCCCCCAGGCACCTACACCCCCTAGTGAACAGAGCAGCCCC

CCAAGCAGGGACCCGTTGAACAACTCTGGGGGCTTTGAGTCTGCCCGCGACGTGGAGGCGCTGATGGAGC

GCATGCAGCAGCTGCAGGAGAGCCTGCTGCGGGATGAGGGGACGTCCCAGGAGGAGATGGAGAGCCGCTT

TGAGCTGGAGAAGTCAGAGAGCCTGCTGGTGACCCCCTCAGCTGACATCCTGGAGCCCTCTCCACACCCA

GACATGCTGTGCTTTGTGGAAGACCCTACTTTCGGATATGAGGACTTCACTCGGAGAGGGGCTCAGGCAC

CCCCTACCTTCCGGGCCCAGGATTATACCTGGGAAGACCATGGCTACTCGCTGATCCAGCGGCTTTACCC

TGAGGGTGGGCAGCTGCTGGATGAGAAGTTCCAGGCAGCCTATAGCCTCACCTACAATACCATCGCCATG

CACAGTGGTGTGGACACCTCCGTGCTCCGCAGGGCCATCTGGAACTATATCCACTGCGTCTTTGGCATCA

GATATGATGACTATGATTATGGGGAGGTGAACCAGCTCCTGGAGCGGAACCTCAAGGTCTATATCAAGAC

AGTGGCCTGCTACCCAGAGAAGACCACCCGAAGAATGTACAACCTCTTCTGGAGGCACTTCCGCCACTCA

GAGAAGGTCCACGTGAACTTGCTGCTCCTGGAGGCGCGCATGCAAGCCGCTCTGCTGTACGCCCTCCGTG

CCATCACCCGCTACATGACCTGACTCCTGAGCAGGACCTGGGCCCGGTTCAGCTCCCCACAAGGACTTCT

CTGTCTGGAGACAGCCCCAGACCCTTTTGTGTCCCATGCCCACCCTCCCCACGCTGCAGTGGGCTTGTGT

GTGATGTGCAGTCCCGAAGCCACACCCTCCCTTTTCCTCACTGGAATGGACAGTTCATTGCACTGACTCT

GGGATCTCAGCCCTGCTCCTGGGAGCTGGAAGAGCACTTGGAGATCCTAAGGGACCACACCCTTCCTCCT

TCCCCTGCCCACAGAGGCAGAGGGCACAGGAAAGAAGCCGGGCCAAGCTCGGAATTAATGTGCCACAAGT

GTTGTGGCCTTCCTGAACTGGGAAGTCCCTGGCTGGCCCCCGGGGGAGAGGGGCAAATGCCTCCGGGACT

GACACTCCAGGCAGCTTTGCCTTCTCTCCCCTGTCATTTCCAGATTTCATTACCTCCTACTTGCCATTCA

CCCATCAATGTGAAAGTCAGGGTCACAGCTGGTCTGTGTGTCCAGTTCCCTAAAAGCCTGTTCTGTTGGG

CAGCCTGAGGCTGTTGCCCGAATCCTAGTTCAGTTTTTTGACTTCCTTTGCCCTTTTTCCCTTTTCTCCA

TGCTTAATGGTGTGAGGCGTCAGGAGAGAGGCCAAGTACATAAAAAAAAAAAAAAGCAGATTATCTCTAGA

GAGTTTGAGCCTTTGCTGGTCACATTGCCTTCTGAAGAGGAGGGAGTATTAGATTATAAATCCTCTTTAT

TTTGGTCCTTTATGCTTGAGGTTCCAACCTGGAGCCACAGTGTGTGAGAGGAGGAGGAGAGGGAGAATTC

TGTTCTCCCAGAGCTGCACCTGCCTCGCAGAGGCCAGCACCCCACTCTCCTGCCTCCAGTGGCCCTGCCG

CAGATGTCTCCCAAAAAGTTGAGCCTTTCTAGATGGCTTAGGTGGCACCATGGCTCAGCAGGAGGGGCGG

GAGGCACCAGGGTTCTTGTTTGGACCCTGCCCCTGGGCCATGGCCAGGTGACCATGGCTACATTGCCAAA

CCTCTGACTGCCACAGCTGCAGACTGAGAGGGTGGGTCTGAGTCCCCACAATGTCTGAAGCTGCCCCTGG

GATTCTCAGGCCAACCTGCCAACAGCAAGCGGATTTTCTTGCAAGATCAGGGACCCCATTTCTGCAGCCA

GTGTCTCCTGGGTGCCTTCTGAGGACTCCCACCCCCATCCCAGTATCTCATCTGTCCCCTCTCCTGGGGC

TTAAGTGGGTTGCTTCCAGGCAGAAGCAGCCAAGGACCGATTCCAGGCACTTTCTGTAGCAAATGACTGT

GAATTACGACTTCTCTTGCCCTTCTTCTAGCAGTCTGTGCCTCCTCTCTGACCAGTTTGGAGGGCACTGA

AGAAAGGCAAGGGCCGTGCTGCTGCTGGGCGGGGCAGGAGAGGAGCCTGGCCAGTGTGCCACATTAAATA

CCCGTGCAGGCGCGGAGAAGCAACCGGCACCCCCTTCCGGCCTGAAAGCCCTCCCTGCAAGAAGGTGTGC

AGGAGAGAAGAGGCCCCGGCATGGGGATCTGGGTTCTAGAGGGCATGTGATGACTGTAAATGTTCACTGG

GTGGGTAGGGAGTGGTATCCAGTGTTCAAGTGCAGAAATCTTTGGCTTTGCTACCAGTTCCATATGATGA

GAAATAAACGTTCGCTGAGGTTTTGTTTCATA

>NM_016009.5 Homo sapiens SH3 domain containing GRB2 like, endophilin B1 (SH3GLB1), transcript variant 1, mRNA

GTTTTTCCCTTGGGACCCGGGTCCACACGGCGGGGTCGCCCGTCCATCTCCGGCTCGCCCGCGGGGCCCA

TCGTCGACGTTAGCGGCCGTTCTCCGAGCCGACTGACCCATCCTTGGCGCTGCCGCCGCGCGCTTGTTCT

CCTCCCTCGCCCCGCCTTCATCCTCCCCGTTCACGGAAACGACAGCTGCGGCTGCGGGGCTGGCGCCGCC

TCCCTCCACCTACCACGTCTGCCCTCGCCGCTCTAGCCCTGCGCCCCAGCCCGGCCGCGGCACCTCCGCC

TCGCCGCCGCTAGGTCGGCCGGCTCCGCCCGGCTGCCGCCTAGGATGAATATCATGGACTTCAACGTGAA

GAAGCTGGCGGCCGACGCAGGCACCTTCCTCAGTCGCGCCGTGCAGTTCACAGAAGAAAAGCTTGGCCAG

GCTGAGAAGACAGAATTGGATGCTCACTTAGAGAACCTCCTTAGCAAAGCTGAATGTACCAAAATATGGA

CAGAAAAAATAATGAAACAAACTGAAGTGTTATTGCAGCCAAATCCAAATGCCAGGATAGAAGAATTTGT

TTATGAGAAACTGGATAGAAAAGCTCCAAGTCGTATAAACAACCCAGAACTTTTGGGACAATATATGATT

GATGCAGGGACTGAGTTTGGCCCAGGAACAGCTTATGGTAATGCCCTTATTAAATGTGGAGAAACCCAAA

AAAGAATTGGAACAGCAGACAGAGAACTGATTCAAACGTCAGCCTTAAATTTTCTTACTCCTTTAAGAAA

CTTTATAGAAGGAGATTACAAAACAATTGCTAAAGAAAGGAAACTATTGCAAAATAAGAGACTGGATTTG

GATGCTGCAAAAACGAGACTAAAAAAGGCAAAAGCTGCAGAAACTAGAAATTCATCTGAACAGGAATTAA

GAATAACTCAAAGTGAATTTGATCGTCAAGCAGAGATTACCAGACTTCTGCTAGAGGGAATCAGCAGTAC

ACATGCCCATCACCTTCGCTGTCTGAATGACTTTGTAGAAGCCCAGATGACTTACTATGCACAGTGTTAC

CAGTATATGTTGGACCTCCAGAAACAACTGGGAAGTTTTCCATCCAATTATCTTAGTAACAACAATCAGA

CTTCTGTGACACCTGTACCATCAGTTTTACCAAATGCGATTGGTTCTTCTGCCATGGCTTCAACAAGTGG

CCTAGTAATCACCTCTCCTTCCAACCTCAGTGACCTTAAGGAGTGTAGTGGCAGCAGAAAGGCCAGGGTT

CTCTATGATTATGATGCAGCAAACAGTACTGAATTATCACTTCTGGCAGATGAGGTGATCACTGTGTTCA

GTGTTGTTGGAATGGATTCAGACTGGCTAATGGGGGAAAGGGGAAACCAGAAGGGCAAGGTGCCAATTAC

CTACTTAGAACTGCTCAATTAAGTAGGTGGACTATGGAAAGGTTGCCCATCATGACTTTGTATTTATATA

CAATTAACTCTAAATAAAGCAGGTTAAGTATCTTCCATGTTAATGTGTTAAGAGACTGAAAATACCAGCC

ATCAGAAACTGGCCTTTCTGCCAATAAAGTTGCATGGTAAATATTTCATTACAGAATTTATGTTAGAGCT

TTCATGCCAAGAATGTTTTCTTACAAAATTCTCTTTTTATTGAGGTTTCACTAATAAGCAGCTTCTACTT

TTGAGCCTCAACTTAAAGCAGAACTGTTTTCTACTGGATTTTTCATTAACAGCAAGCTTTTTCTTTTATG

TAAAATAAATCTATTGTGAATTGATATCAGCGACTCATTTATTAGGTATAAATAATAGCCAAAGAATAAA

ATTAAAATTTATTTTAAACTTTCGGTCTTAAAAAGAGCTGTAGGATATAAACCTCTTTTGTGAAAAGTAG

AATTTTCTGAATGCTTTCATAGACAAAAACGCATTTAAACTATGTTTACCTGGTTTTCCAACAGAAATCA

AATGATTTTAATATTATGTTTTGATGGGTTATTTTCAGAGTTGTTTGGTTTTTTAAACACTACTGAATGG

TTTCTTTTAAATTTAAGAACAACTAGTTCTTGAAAAGCAATACCGTATTTCTCTGATAAATTTGTAGTTA

AGGTTCTAGAAAATCTAGGACAAATTTACTTCATTAAACATAAAGTTTTTAAGATTATTCAGTTGGCACA

ATTTAATGCATAATTGGGATTGGATCCATTTACTGTACTGTGGTACAGTAAAGAGAATGTGTTCTTATTG

AACAGCAGATTTGTATACAAATACGGAATAATGCAACTACATTATAAATTGTATACGAACAAAATAAATT

GGGTCACTCTGAGCTTACTATTAAAACATTGAAAATTTATTAATGAACACAGGCTATCTTGATGAGGATC

TCTGCTTATGAAACACATACTAATGAAATTATTAGTGACACATTGGAAGCTAGAAAATTGCTGTTGGGGC

CATTGCTATAAATCATTGTTTCCATGTTAAGACTTCTGTTCTAATTCATTCATTCAACAAACATTTGTTG

AGGGCCAGATACTGTGCTGGTCAACTGGGGATACTGTCCTGAACAATATGCACACAGTTCTTGCCCCTGC

AGAGCTTAAAGACCATTAAAGGATACAGAAAATTAAGCAATTATCCTATAGTAGGATTCGTGGTGTGATG

GGGAAAATACAAGGTACTGTATTTGGGAGTCATGTAACTCCAGTTTTGGGAGTGAGAGAGGGTCTGGGGA

AGAACTGTTCCAGATAAAAAGATGTGTAACCAAGATAGCATAGCACATTCTGACAACTAAAGCAATTTAC

TCTAACTTAAGCATGAAGTACAAAAGTATAGTTCATTTATTGAGGGAATGTTAAGCTATTAAAATGATAC

TTTTTGGTACTTTTAAAAAAATGAATACTTAAACACCGTTTTAAAATTTTGTGTTAAGAGCTCCTAATAA

GAACTTGGTAAACATGTTGACAATTTAACTCACAGTCTAAACTTTAAAAGTAGTAGTTACCCTTTTATTT

CTGTGCCATGAGACAAATTCTTCTGCAGTTTTATAGTTGTACAAGATGTTTTGACTCACTCTCAATTGCC

ATATTCTTAAAGAAACCCTGAAAAGCTGCTCAAGCTGGTCAGTTTTCTGTAAAACCAAGTAAATGAAATT

TTCTCTCCCCTCAGAAAGGAAGTTCTACAGCCTAGAGTACCATCTATTGTGTTTTATAAGTCCAGATTTA

GGCTTTGAAAGATCATACCAAAACTTCAGATCGACTTAAACAGCAACTATGAATTAGGTTTATGGGAAAT

GTAGTAATACAGGGGAAAGAATATCGGTAATGGAGTGAAATGGTCCTGGGTTCAAATCCAGTCTTCCTCA

CATATTGTGCTCTTTAAGCTTCAAATGAGATGTATAACTGCCTTTTCCTTACCAGTAAAATGAGGACAAT

AATTATGACACCATAGGGTGGTTGTGAGGATTAGCAAATGTTATCTTGTATTTCAGGTGCCTTTCAGGGA

AAGGCTTGGCACATAGTAAACACATCCTGTAAATATCAGTTTCCCCTTGTTAGCCCCTGTAGCATCCTGA

CCTACCAAGTCTATAGGCCTTGCATATCACTGTGCACCAGAGATACCTCTTTTACATCTGGGCTCTGTTT

TCTCTCTATCAATCCTATCCTCCACCCTCTTTAAATCAGACCCCAGGTTTAACCTTCCCACAGTCAGTAT

AAATCATTTTTCAGAACTTTCAGCTTTCCATCAAGTGAAAGTTGATTTTAGTTACCCAGTAATGTACAGA

AACCTTCAATAATGTTAAGCTTCAAAGGCTTCTGGTACGTTTGTCTACCACAGAACCACCACAATATATA

TGCTTGTTCTTAGGTTTATTGCTTTTTATTTTAATGGGGTTTGGTTGTTTGGGTTTTATTTTGGTTTTGG

CTTATTTTAAAGTCAAGTTTAGTTTTTACTCAGGTAAAGGCCTGAAGTTTCTTGGGCGTGGAAGAAAAAG

CTTGGGAAGCCTATTAATTCATGGTGCAGTTCAGAATTGTTTAAATAAGCTCTGTGCCACTGAGCTTAAT

CTGAAAGTATAGTGACATGTGAAGGATGCACATATGAATGAAAAACTAAAATAATTGTAGGCTCTTCTAT

AAATGAAAAAAAAAGTGATTGGATTGTGTCTACCTTTTTCCTCAAAGTAATCTAACCTAAACGGTCATGG

TGTTAAAACTTTTTATGGTGCTAATCAGAAAAATTGATGTTGCAAGAAATGAGGTCTCAAAAATGGGAAT

GTGCCTCCTCACTCTGCGTCACATGGGAAATAAATCCTGAGGCCAGGATCACCAGTGGGGTAGAGTCATC

TTCATAGTCTCCTGTTATCCTCCCCCTGAACAAAGAACAAAGTGAGACTGGGAAGCTAGTGTTGGGGAAA

CATTTCTGGGATAAACTGTGGAGTGGAGAAGACAATGGAGGGCAAGGCAAAAACCCACAGACTTGTTTAC

CCATAGAGGCCCCACTCACCTCCATGCTGACAGAATTCCAGAAAAATTCCTTCCCTATCTCCCAAACACC

TGAAATTCTGAAATATTGCCCTACTATTTAGCACTTTAAGCTTCAGTGAGGTGCTGTAATGGAGAGATAG

CTTCATAGTTTAGCCTATAGCCAACTTTGCCTCAAGACCTCCAGATAAGATGAAGTGGCTTTTACCTCAT

GTGAGTCTCAAACGCTGGAGAGAGAGTTCCACAACCACCCCTGCCCTTTCTCCCATATTACGCTCATTAG

AAAGTCTACTCAGGGCTGGGTGCCGTGGCTCATGCCTGTAATCCCAGCACTTTGGAGGCTGAGGCGGGTG

GATCATCTGAGGTCAGGAGTTCGAGACCAGCCTGGCCAACATGGGGAAACCCTGTCTCTACTGAAAATAT

AAAAATTAGCCGGGCATGGTGGCGTGCACCTGTAATTCCAGCTACTCGGGAGGCTGAGGCAGGAGAATCA

CTTGAACCCCAGAGGCAGAGGCTACAGTGAGCCAAGATTGCACCACTGCACTCCAACCTGGGTGATAGAA

TAAGACTCTTGTCTCAAGGAAAGAAAAAATGTCTACTCAGTAGAGCAGTGATTGGCAAAGTGTGAGACCC

CAGACCGGCAATATTGGCATCTCCTGGAGACTTGTTTAAAATGCTGATTACTATTTTCAAGAACTTTCTA

CCTTCATTTTTACTTATTTTTTGTTTTCAAGGGCTCATGGAATCAGTCTTCCATGTGACAGCTTTTTAAA

TATTTTAAGACAAAAACTGAGTCTTCTAATCTTAATTGTGTTCTAGCAGTAGTCATTGTAATTATAGTTA

TTTTTATAATTTATTTGTTTAGTGTATATCTGTCACTCTAGAATGTAGGCTCCCTGAGATAAAAACTATA

ATTCTTTTGATGGCAGTTACATTCCCAACTCTTAGCACAAGATCTAGGACATAGTATGAACTCAATAAAT

TTTTGTTGAATGAAAGAAAAATGCAAATTTATAAGCCTTTTCAGAGACCTACTGAATCAAACTCTGAGAG

TGGAGCCCAGCAATCTGTTGTAAAAGCTGTTTGAGTAATACTGTTGCACTCTAAGCTTGAGAACCACTGC

AATAGAACTAAGAGAACATTCATTTCACCTTCAGGCCTATTCTCTGACATCACTTGAAAAATCCTCTCTG

CCTTCTGTAACCCTTAACCAACAACAGTGCCCTGTCCATGTAGGGGAAACTTCTGGGCTGTATTACAAGT

GGGTAGGAAGGTATAAGGATATCATAGTGGGAAGTATTGCTTAGAATGCCAGTACTTGTTTGCTGTGGAA

GGTTTCTAGGAATGGATTGCTTAGTCTAAAGTAAGCACTACCACCATCCACGTTTTCACTCAACACAGTG

CCTTTTAATTGGTGACTAACATCAAAACACCTTGACAAACCGTTAAAACAGTATCAGAAACCATGTGTAC

ATCCCTGTGCCTCTTAGATTGCTAAGTTCTGCTCTCATTCTTACTTCAAAGGACACTTTTTAAAGAATTG

TTAATGCTTTTTTATAATACAAGTCATAGCCCTGTTGGCAAAAAGAAAAAAGATTCAGTGAGAAAGAAAA

ATCACTGATAATTCCATCATCCAGGGAGAACCACTGTTATTTAAGTATATGGCCTTCCAGACTTTTCTGT

GCATTTATAGATATTTTTAATGGGATCAAAGTGTACATACTGTTTTGTAACTTTTTTTCACTCAACAATA

CCATGACTGTCTTGCTGTGTCAAGTATATTTCTACATCTCTTAATGGCTGCATGATATTCCATCATATGT

GCCACATTTTATTAAACTACTGTTCAATTTTTAGGTTTCCAACTTTCATTAATAAAGTGCTTTAAAGAAC

A

>NM_012238.5 Homo sapiens sirtuin 1 (SIRT1), transcript variant 1, mRNA

GCCAGTGCCGCGCGTCGAGCGGGAGCAGAGGAGGCGAGGGAGGAGGGCCAGAGAGGCAGTTGGAAGATGG

CGGACGAGGCGGCCCTCGCCCTTCAGCCCGGCGGCTCCCCCTCGGCGGCGGGGGCCGACAGGGAGGCCGC

GTCGTCCCCCGCCGGGGAGCCGCTCCGCAAGAGGCCGCGGAGAGATGGTCCCGGCCTCGAGCGGAGCCCG

GGCGAGCCCGGTGGGGCGGCCCCAGAGCGTGAGGTGCCGGCGGCGGCCAGGGGCTGCCCGGGTGCGGCGG

CGGCGGCGCTGTGGCGGGAGGCGGAGGCAGAGGCGGCGGCGGCAGGCGGGGAGCAAGAGGCCCAGGCGAC

TGCGGCGGCTGGGGAAGGAGACAATGGGCCGGGCCTGCAGGGCCCATCTCGGGAGCCACCGCTGGCCGAC

AACTTGTACGACGAAGACGACGACGACGAGGGCGAGGAGGAGGAAGAGGCGGCGGCGGCGGCGATTGGGT

ACCGAGATAACCTTCTGTTCGGTGATGAAATTATCACTAATGGTTTTCATTCCTGTGAAAGTGATGAGGA

GGATAGAGCCTCACATGCAAGCTCTAGTGACTGGACTCCAAGGCCACGGATAGGTCCATATACTTTTGTT

CAGCAACATCTTATGATTGGCACAGATCCTCGAACAATTCTTAAAGATTTATTGCCGGAAACAATACCTC

CACCTGAGTTGGATGATATGACACTGTGGCAGATTGTTATTAATATCCTTTCAGAACCACCAAAAAGGAA

AAAAAGAAAAGATATTAATACAATTGAAGATGCTGTGAAATTACTGCAAGAGTGCAAAAAAATTATAGTT

CTAACTGGAGCTGGGGTGTCTGTTTCATGTGGAATACCTGACTTCAGGTCAAGGGATGGTATTTATGCTC

GCCTTGCTGTAGACTTCCCAGATCTTCCAGATCCTCAAGCGATGTTTGATATTGAATATTTCAGAAAAGA

TCCAAGACCATTCTTCAAGTTTGCAAAGGAAATATATCCTGGACAATTCCAGCCATCTCTCTGTCACAAA

TTCATAGCCTTGTCAGATAAGGAAGGAAAACTACTTCGCAACTATACCCAGAACATAGACACGCTGGAAC

AGGTTGCGGGAATCCAAAGGATAATTCAGTGTCATGGTTCCTTTGCAACAGCATCTTGCCTGATTTGTAA

ATACAAAGTTGACTGTGAAGCTGTACGAGGAGATATTTTTAATCAGGTAGTTCCTCGATGTCCTAGGTGC

CCAGCTGATGAACCGCTTGCTATCATGAAACCAGAGATTGTGTTTTTTGGTGAAAATTTACCAGAACAGT

TTCATAGAGCCATGAAGTATGACAAAGATGAAGTTGACCTCCTCATTGTTATTGGGTCTTCCCTCAAAGT

AAGACCAGTAGCACTAATTCCAAGTTCCATACCCCATGAAGTGCCTCAGATATTAATTAATAGAGAACCT

TTGCCTCATCTGCATTTTGATGTAGAGCTTCTTGGAGACTGTGATGTCATAATTAATGAATTGTGTCATA

GGTTAGGTGGTGAATATGCCAAACTTTGCTGTAACCCTGTAAAGCTTTCAGAAATTACTGAAAAACCTCC

ACGAACACAAAAAGAATTGGCTTATTTGTCAGAGTTGCCACCCACACCTCTTCATGTTTCAGAAGACTCA

AGTTCACCAGAAAGAACTTCACCACCAGATTCTTCAGTGATTGTCACACTTTTAGACCAAGCAGCTAAGA

GTAATGATGATTTAGATGTGTCTGAATCAAAAGGTTGTATGGAAGAAAAACCACAGGAAGTACAAACTTC

TAGGAATGTTGAAAGTATTGCTGAACAGATGGAAAATCCGGATTTGAAGAATGTTGGTTCTAGTACTGGG

GAGAAAAATGAAAGAACTTCAGTGGCTGGAACAGTGAGAAAATGCTGGCCTAATAGAGTGGCAAAGGAGC

AGATTAGTAGGCGGCTTGATGGTAATCAGTATCTGTTTTTGCCACCAAATCGTTACATTTTCCATGGCGC

TGAGGTATATTCAGACTCTGAAGATGACGTCTTATCCTCTAGTTCTTGTGGCAGTAACAGTGATAGTGGG

ACATGCCAGAGTCCAAGTTTAGAAGAACCCATGGAGGATGAAAGTGAAATTGAAGAATTCTACAATGGCT

TAGAAGATGAGCCTGATGTTCCAGAGAGAGCTGGAGGAGCTGGATTTGGGACTGATGGAGATGATCAAGA

GGCAATTAATGAAGCTATATCTGTGAAACAGGAAGTAACAGACATGAACTATCCATCAAACAAATCATAG

TGTAATAATTGTGCAGGTACAGGAATTGTTCCACCAGCATTAGGAACTTTAGCATGTCAAAATGAATGTT

TACTTGTGAACTCGATAGAGCAAGGAAACCAGAAAGGTGTAATATTTATAGGTTGGTAAAATAGATTGTT

TTTCATGGATAATTTTTAACTTCATTATTTCTGTACTTGTACAAACTCAACACTAACTTTTTTTTTTTTA

AAAAAAAAAAGGTACTAAGTATCTTCAATCAGCTGTTGGTCAAGACTAACTTTCTTTTAAAGGTTCATTT

GTATGATAAATTCATATGTGTATATATAATTTTTTTTGTTTTGTCTAGTGAGTTTCAACATTTTTAAAGT

TTTCAAAAAGCCATCGGAATGTTAAATTAATGTAAAGGGAACAGCTAATCTAGACCAAAGAATGGTATTT

TCACTTTTCTTTGTAACATTGAATGGTTTGAAGTACTCAAAATCTGTTACGCTAAACTTTTGATTCTTTA

ACACAATTATTTTTAAACACTGGCATTTTCCAAAACTGTGGCAGCTAACTTTTTAAAATCTCAAATGACA

TGCAGTGTGAGTAGAAGGAAGTCAACAATATGTGGGGAGAGCACTCGGTTGTCTTTACTTTTAAAAGTAA

TACTTGGTGCTAAGAATTTCAGGATTATTGTATTTACGTTCAAATGAAGATGGCTTTTGTACTTCCTGTG

GACATGTAGCAATGTCTATATTGGCTCATAAAACTAACCTGAAAAACAAATAAATGCTTTGGAAATGTTT

CAGTTGCTTTAGAAACATTAGTGCCTGCCTGGATCCCCTTAGTTTTGAAATATTTGCCATTGTTGTTTAA

ATACCTATCACTGTGGTAGAGCTTGCATTGATCTTTTCCACAAGTATTAAACTGCCAAAATGTGAATATG

CAAAGCCTTTCTGAATCTATAATAATGGTACTTCTACTGGGGAGAGTGTAATATTTTGGACTGCTGTTTT

CCATTAATGAGGAGAGCAACAGGCCCCTGATTATACAGTTCCAAAGTAATAAGATGTTAATTGTAATTCA

GCCAGAAAGTACATGTCTCCCATTGGGAGGATTTGGTGTTAAATACCAAACTGCTAGCCCTAGTATTATG

GAGATGAACATGATGATGTAACTTGTAATAGCAGAATAGTTAATGAATGAAACTAGTTCTTATAATTTAT

CTTTATTTAAAAGCTTAGCCTGCCTTAAAACTAGAGATCAACTTTCTCAGCTGCAAAAGCTTCTAGTCTT

TCAAGAAGTTCATACTTTATGAAATTGCACAGTAAGCATTTATTTTTCAGACCATTTTTGAACATCACTC

CTAAATTAATAAAGTATTCCTCTGTTGCTTTAGTATTTATTACAATAAAAAGGGTTTGAAATATAGCTGT

TCTTTATGCATAAAACACCCAGCTAGGACCATTACTGCCAGAGAAAAAAATCGTATTGAATGGCCATTTC

CCTACTTATAAGATGTCTCAATCTGAATTTATTTGGCTACACTAAAGAATGCAGTATATTTAGTTTTCCA

TTTGCATGATGTTTGTGTGCTATAGATGATATTTTAAATTGAAAAGTTTGTTTTAAATTATTTTTACAGT

GAAGACTGTTTTCAGCTCTTTTTATATTGTACATAGTCTTTTATGTAATTTACTGGCATATGTTTTGTAG

ACTGTTTAATGACTGGATATCTTCCTTCAACTTTTGAAATACAAAACCAGTGTTTTTTACTTGTACACTG

TTTTAAAGTCTATTAAAATTGTCATTTGACTTTTTTCTGTTAACTTA

>NM_001193286.2 Homo sapiens sirtuin 2 (SIRT2), transcript variant 3, mRNA

AGTCGGTGACAGGACAGAGCAGTCGGTGACGGGACACAGTGGTTGGTGACGGGACAGAGCGGTCGGTGAC

AGCCTCAAGGGCTTCAGCACCGCGCCCATGGCAGAGCCAGACCGACTCAGATTCAGACTCTGAGGGAGGA

GCCGCTGGTGGAGAAGCAGACATGGACTTCCTGCGGAACTTATTCTCCCAGACGCTCAGCCTGGGCAGCC

AGAAGGAGCGTCTGCTGGACGAGCTGACCTTGGAAGGGGTGGCCCGGTACATGCAGAGCGAACGCTGTCG

CAGAGTCATCTGTTTGGTGGGAGCTGGAATCTCCACATCCGCAGGCATCCCCGACTTTCGCTCTCCATCC

ACCGGCCTCTATGACAACCTAGAGAAGTACCATCTTCCCTACCCAGAGGCCATCTTTGAGATCAGCTATT

TCAAGAAACATCCGGAACCCTTCTTCGCCCTCGCCAAGGAACTCTATCCTGGGCAGTTCAAGCCAACCAT

CTGTCACTACTTCATGCGCCTGCTGAAGGACAAGGGGCTACTCCTGCGCTGCTACACGCAGAACATAGAT

ACCCTGGAGCGAATAGCCGGGCTGGAACAGGAGGACTTGGTGGAGGCGCACGGCACCTTCTACACATCAC

ACTGCGTCAGCGCCAGCTGCCGGCACGAATACCCGCTAAGCTGGATGAAAGAGAAGATCTTCTCTGAGGT

GACGCCCAAGTGTGAAGACTGTCAGAGCCTGGTGAAGCCTGATATCGTCTTTTTTGGTGAGAGCCTCCCA

GCGCGTTTCTTCTCCTGTATGCAGTCAGACTTCCTGAAGGTGGACCTCCTCCTGGTCATGGGTACCTCCT

TGCAGGGACGTGGCCTGGCTGGGTGAATGCGACCAGGGCTGCCTGGCCCTTGCTGAGCTCCTTGGATGGA

AGAAGGAGCTGGAGGACCTTGTCCGGAGGGAGCACGCCAGCATAGATGCCCAGTCGGGGGCGGGGGTCCC

CAACCCCAGCACTTCAGCTTCCCCCAAGAAGTCCCCGCCACCTGCCAAGGACGAGGCCAGGACAACAGAG

AGGGAGAAACCCCAGTGACAGCTGCATCTCCCAGGCGGGATGCCGAGCTCCTCAGGGACAGCTGAGCCCC

AACCGGGCCTGGCCCCCTCTTAACCAGCAGTTCTTGTCTGGGGAGCTCAGAACATCCCCCAATCTCTTAC

AGCTCCCTCCCCAAAACTGGGGTCCCAGCAACCCTGGCCCCCAACCCCAGCAAATCTCTAACACCTCCTA

GAGGCCAAGGCTTAAACAGGCATCTCTACCAGCCCCACTGTCTCTAACCACTCCTGGGCTAAGGAGTAAC

CTCCCTCATCTCTAACTGCCCCCACGGGGCCAGGGCTACCCCAGAACTTTTAACTCTTCCAGGACAGGGA

GCTTCGGGCCCCCACTCTGTCTCCTGCCCCCGGGGGCCTGTGGCTAAGTAAACCATACCTAACCTACCCC

AGTGTGGGTGTGGGCCTCTGAATATAACCCACACCCAGCGTAGGGGGAGTCTGAGCCGGGAGGGCTCCCG

AGTCTCTGCCTTCAGCTCCCAAAGTGGGTGGTGGGCCCCCTTCACGTGGGACCCACTTCCCATGCTGGAT

GGGCAGAAGACATTGCTTATTGGAGACAAATTAAAAACAAAAACAACTAACAA

>NM_182965.3 Homo sapiens sphingosine kinase 1 (SPHK1), transcript variant 2, mRNA

AGTGCCCTCCCCGCTCCGCGGCGCCGGCTGCGAAGTTGAGCGAAAAGTTTGAGGCCGGAGGGAGCGAGGC

CGGGGAGTCCGCTCCAGCGGGGCGCTCCAGTCCCTCAGACGTGGGCTGAGCTTGGGACGAGCTGCGTTCC

GCCCCAGGCCACTGTAGGGAACGGCGGTGGCGCCTCCCCAGCAAACCGGACCGACTGGGTAGGGCCGCCC

ACCCTGCCTTCGCGCCGCTCGTGGCTCCTGTGCGGCCCGCCCTCGCGGGGCCCCGGGAACTGGGCCACTT

GTCGCTTGGGCGAGAGCAGGCGGCAGCTGGTGGCCCGGTTCGGGCTTGGCTTTGGCGCGACCCGGGAGCG

GCTCCCACGAGCGCCGCGCGCGTCGCAACGGAGCGGGGCCCTGAGAAGCGCGCGCCGCGGCTCCCACCGC

TCTGGAGCTCCGGGCAGGGGACACGGCAACCTGGATGGCTGGGGCAGGGATCCTCTCCCAGAACTTCGTG

CCCAGCAATGTCCGCTCAAGTTCTGGGATTTTTACGCAGCTGGACTCCCCTCCCCCTGGCAGCCCCGAGG

GGTCCAGCCGCCGCAGGGAATGACGCCGGTGCTCCTGCAGCCACGGCTCCGGGCGGGGAAGGCGAGCCCC

ACAGCCGGCCCTGCGACGCCCGCCTGGGCAGCACCGATAAGGAGCTGAAGGCAGGAGCCGCCGCCACGGG

CAGCGCCCCCACAGCGCCAGGGACCCCCTGGCAGCGGGAGCCGCGGGTCGAGGTTATGGATCCAGCGGGC

GGCCCCCGGGGCGTGCTCCCGCGGCCCTGCCGCGTGCTGGTGCTGCTGAACCCGCGCGGCGGCAAGGGCA

AGGCCTTGCAGCTCTTCCGGAGTCACGTGCAGCCCCTTTTGGCTGAGGCTGAAATCTCCTTCACGCTGAT

GCTCACTGAGCGGCGGAACCACGCGCGGGAGCTGGTGCGGTCGGAGGAGCTGGGCCGCTGGGACGCTCTG

GTGGTCATGTCTGGAGACGGGCTGATGCACGAGGTGGTGAACGGGCTCATGGAGCGGCCTGACTGGGAGA

CCGCCATCCAGAAGCCCCTGTGTAGCCTCCCAGCAGGCTCTGGCAACGCGCTGGCAGCTTCCTTGAACCA

TTATGCTGGCTATGAGCAGGTCACCAATGAAGACCTCCTGACCAACTGCACGCTATTGCTGTGCCGCCGG

CTGCTGTCACCCATGAACCTGCTGTCTCTGCACACGGCTTCGGGGCTGCGCCTCTTCTCTGTGCTCAGCC

TGGCCTGGGGCTTCATTGCTGATGTGGACCTAGAGAGTGAGAAGTATCGGCGTCTGGGGGAGATGCGCTT

CACTCTGGGCACCTTCCTGCGTCTGGCAGCCCTGCGCACCTACCGCGGCCGACTGGCCTACCTCCCTGTA

GGAAGAGTGGGTTCCAAGACACCTGCCTCCCCCGTTGTGGTCCAGCAGGGCCCGGTAGATGCACACCTTG

TGCCACTGGAGGAGCCAGTGCCCTCTCACTGGACAGTGGTGCCCGACGAGGACTTTGTGCTAGTCCTGGC

ACTGCTGCACTCGCACCTGGGCAGTGAGATGTTTGCTGCACCCATGGGCCGCTGTGCAGCTGGCGTCATG

CATCTGTTCTACGTGCGGGCGGGAGTGTCTCGTGCCATGCTGCTGCGCCTCTTCCTGGCCATGGAGAAGG

GCAGGCATATGGAGTATGAATGCCCCTACTTGGTATATGTGCCCGTGGTCGCCTTCCGCTTGGAGCCCAA

GGATGGGAAAGGTGTGTTTGCAGTGGATGGGGAATTGATGGTTAGCGAGGCCGTGCAGGGCCAGGTGCAC

CCAAACTACTTCTGGATGGTCAGCGGTTGCGTGGAGCCCCCGCCCAGCTGGAAGCCCCAGCAGATGCCAC

CGCCAGAAGAGCCCTTATGACCCCTGGGCCGCGCTGTGCCTTAGTGTCTACTTGCAGGACCCTTCCTCCT

TCCCTAGGGCTGCAGGGCCTGTCCACAGCTCCTGTGGGGGTGGAGGAGACTCCTCTGGAGAAGGGTGAGA

AGGTGGAGGCTATGCTTTGGGGGGACAGGCCAGAATGAAGTCCTGGGTCAGGAGCCCAGCTGGCTGGGCC

CAGCTGCCTATGTAAGGCCTTCTAGTTTGTTCTGAGACCCCCACCCCACGAACCAAATCCAAATAAAGTG

ACATTCCCA

>NM_032038.3 Homo sapiens sphingolipid transporter 1 (putative) (SPNS1), transcript variant 1, mRNA

ACATGACCGGCTTTAAGCAACATGGCGGCTGCCGTGGTGCAGCGCCCGGGCTGAGCGACAGCAAGTGCAG

CGGGCTCCTACCCCGGGTGAGGGGTGGCCTCCGCGTGGGATCGTGCCCTCTTCAGCCCGCTCCTGTCCCC

GACATCACGTGTATTCCGCACGTCCCCTCCGCGCTGTGTGTCTACTGAGACGGGGAGGCGTGACAGGGCC

CGGGTCCCTTCTCAGTGGTGCTCTGTGCTTCAGGGCAAGCTCCCCGTCTCCGGGCGCACTTCCCTCGCCT

GTGTTCGGTCCATCCTCCTTTCTCCAGCCTCCTCCCCTCGCAGGTGGGATCGTCGGTGGGACCGGAGCGC

GGGCGGGCGCGGCCCCCCGGGACCATGGCCGGGTCCGACACCGCGCCCTTCCTCAGCCAGGCGGATGACC

CGGACGACGGGCCAGTGCCTGGCACCCCGGGGTTGCCAGGGTCCACGGGGAACCCGAAGTCCGAGGAGCC

CGAGGTCCCGGACCAGGAGGGGCTGCAGCGCATCACCGGCCTGTCTCCCGGCCGTTCGGCTCTCATAGTG

GCGGTGCTGTGCTACATCAATCTCCTGAACTACATGGACCGCTTCACCGTGGCTGGCGTCCTTCCCGACA

TCGAGCAGTTCTTCAACATCGGGGACAGTAGCTCTGGGCTCATCCAGACCGTGTTCATCTCCAGTTACAT

GGTGTTGGCACCTGTGTTTGGCTACCTGGGTGACAGGTACAATCGGAAGTATCTCATGTGCGGGGGCATT

GCCTTCTGGTCCCTGGTGACACTGGGGTCATCCTTCATCCCCGGAGAGCATTTCTGGCTGCTCCTCCTGA

CCCGGGGCCTGGTGGGGGTCGGGGAGGCCAGTTATTCCACCATCGCGCCCACTCTCATTGCCGACCTCTT

TGTGGCCGACCAGCGGAGCCGGATGCTCAGCATCTTCTACTTTGCCATTCCGGTGGGCAGTGGTCTGGGC

TACATTGCAGGCTCCAAAGTGAAGGATATGGCTGGAGACTGGCACTGGGCTCTGAGGGTGACACCGGGTC

TAGGAGTGGTGGCCGTTCTGCTGCTGTTCCTGGTAGTGCGGGAGCCGCCAAGGGGAGCCGTGGAGCGCCA

CTCAGATTTGCCACCCCTGAACCCCACCTCGTGGTGGGCAGATCTGAGGGCTCTGGCAAGAAATCCTAGT

TTCGTCCTGTCTTCCCTGGGCTTCACTGCTGTGGCCTTTGTCACGGGCTCCCTGGCTCTGTGGGCTCCGG

CATTCCTGCTGCGTTCCCGCGTGGTCCTTGGGGAGACCCCACCCTGCCTTCCCGGAGACTCCTGCTCTTC

CTCTGACAGTCTCATCTTTGGACTCATCACCTGCCTGACCGGAGTCCTGGGTGTGGGCCTGGGTGTGGAG

ATCAGCCGCCGGCTCCGCCACTCCAACCCCCGGGCTGATCCCCTGGTCTGTGCCACTGGCCTCCTGGGCT

CTGCACCCTTCCTCTTCCTGTCCCTTGCCTGCGCCCGTGGTAGCATCGTGGCCACTTATATTTTCATCTT

CATTGGAGAGACCCTCCTGTCCATGAACTGGGCCATCGTGGCCGACATTCTGCTGTACGTGGTGATCCCT

ACCCGACGCTCCACCGCCGAGGCCTTCCAGATCGTGCTGTCCCACCTGCTGGGTGATGCTGGGAGCCCCT

ACCTCATTGGCCTGATCTCTGACCGCCTGCGCCGGAACTGGCCCCCCTCCTTCTTGTCCGAGTTCCGGGC

TCTGCAGTTCTCGCTCATGCTCTGCGCGTTTGTTGGGGCACTGGGCGGCGCAGCCTTCCTGGGCACCGCC

ATCTTCATTGAGGCCGACCGCCGGCGGGCACAGCTGCACGTGCAGGGCCTGCTGCACGAAGCAGGGTCCA

CAGACGACCGGATTGTGGTGCCCCAGCGGGGCCGCTCCACCCGCGTGCCCGTGGCCAGTGTGCTCATCTG

AGAGGCTGCCGCTCACCTACCTGCACATCTGCCACAGCTGGCCCTGGGCCCACCCCACGAAGGGCCTGGG

CCTAACCCCTTGGCCTGGCCCAGCTTCCAGAGGGACCCTGGGCCGTGTGCCAGCTCCCAGACACTACCTG

GGTAGCTCAGGGGAGGAGGTGGGGGTCCAGGAGGGGGATCCCTCTCCACAGGGGCAGCCCCAAGGGCTCG

GTGCTATTTGTAACGGAATAAAATTTGTAGCCAGA

>NM_001142298.2 Homo sapiens sequestosome 1 (SQSTM1), transcript variant 2, mRNA

GGCTTCCGGCCGCCTTCCGCGGCCACCGCCGGGCCCGCTCCCGCCGCCGACGCCCAGGTGCGCCAGGTGC

GGGCCGGGCGGGGGTCGCGCTCACCTTTCTGGCCGCTGAGTGCCGCGTACCAGGACAGCGAGAGGAAGGC

GCACAGGCAGAAGAGCAGCAGCGTCAGGAAGGTGCCATTGCGGAGCCTCATCTCCTCGGTGTCTGCGAGA

TTAATCTCTCATGGCCGCTGCACAAGAACCTGGCTTTTAGCTGAACTAAGGAGAAAGTCCTACAACAGTT

TGGCGTGCAACATGGGGCTTGAGAAAGGATGAGGACGGGGACTTGGTTGCCTTTTCCAGTGACGAGGAAT

TGACAATGGCCATGTCCTACGTGAAGGATGACATCTTCCGAATCTACATTAAAGAGAAAAAAGAGTGCCG

GCGGGACCACCGCCCACCGTGTGCTCAGGAGGCGCCCCGCAACATGGTGCACCCCAATGTGATCTGCGAT

GGCTGCAATGGGCCTGTGGTAGGAACCCGCTACAAGTGCAGCGTCTGCCCAGACTACGACTTGTGTAGCG

TCTGCGAGGGAAAGGGCTTGCACCGGGGGCACACCAAGCTCGCATTCCCCAGCCCCTTCGGGCACCTGTC

TGAGGGCTTCTCGCACAGCCGCTGGCTCCGGAAGGTGAAACACGGACACTTCGGGTGGCCAGGATGGGAA

ATGGGTCCACCAGGAAACTGGAGCCCACGTCCTCCTCGTGCAGGGGAGGCCCGCCCTGGCCCCACGGCAG

AATCAGCTTCTGGTCCATCGGAGGATCCGAGTGTGAATTTCCTGAAGAACGTTGGGGAGAGTGTGGCAGC

TGCCCTTAGCCCTCTGGGCATTGAAGTTGATATCGATGTGGAGCACGGAGGGAAAAGAAGCCGCCTGACC

CCCGTCTCTCCAGAGAGTTCCAGCACAGAGGAGAAGAGCAGCTCACAGCCAAGCAGCTGCTGCTCTGACC

CCAGCAAGCCGGGTGGGAATGTTGAGGGCGCCACGCAGTCTCTGGCGGAGCAGATGAGGAAGATCGCCTT

GGAGTCCGAGGGGCGCCCTGAGGAACAGATGGAGTCGGATAACTGTTCAGGAGGAGATGATGACTGGACC

CATCTGTCTTCAAAAGAAGTGGACCCGTCTACAGGTGAACTCCAGTCCCTACAGATGCCAGAATCCGAAG

GGCCAAGCTCTCTGGACCCCTCCCAGGAGGGACCCACAGGGCTGAAGGAAGCTGCCTTGTACCCACATCT

CCCGCCAGAGGCTGACCCGCGGCTGATTGAGTCCCTCTCCCAGATGCTGTCCATGGGCTTCTCTGATGAA

GGCGGCTGGCTCACCAGGCTCCTGCAGACCAAGAACTATGACATCGGAGCGGCTCTGGACACCATCCAGT

ATTCAAAGCATCCCCCGCCGTTGTGACCACTTTTGCCCACCTCTTCTGCGTGCCCCTCTTCTGTCTCATA

GTTGTGTTAAGCTTGCGTAGAATTGCAGGTCTCTGTACGGGCCAGTTTCTCTGCCTTCTTCCAGGATCAG

GGGTTAGGGTGCAAGAAGCCATTTAGGGCAGCAAAACAAGTGACATGAAGGGAGGGTCCCTGTGTGTGTG

TGTGCTGATGTTTCCTGGGTGCCCTGGCTCCTTGCAGCAGGGCTGGGCCTGCGAGACCCAAGGCTCACTG

CAGCGCGCTCCTGACCCCTCCCTGCAGGGGCTACGTTAGCAGCCCAGCACATAGCTTGCCTAATGGCTTT

CACTTTCTCTTTTGTTTTAAATGACTCATAGGTCCCTGACATTTAGTTGATTATTTTCTGCTACAGACCT

GGTACACTCTGATTTTAGATAAAGTAAGCCTAGGTGTTGTCAGCAGGCAGGCTGGGGAGGCCAGTGTTGT

GGGCTTCCTGCTGGGACTGAGAAGGCTCACGAAGGGCATCCGCAATGTTGGTTTCACTGAGAGCTGCCTC

CTGGTCTCTTCACCACTGTAGTTCTCTCATTTCCAAACCATCAGCTGCTTTTAAAATAAGATCTCTTTGT

AGCCATCCTGTTAAATTTGTAAACAATCTAATTAAATGGCATCAGCACTTTAACCAATGACGTTTGCATA

GAGAGAAATGATTGACAGTAAGTTTATTGTTAATGGTTCTTACAGAGTATCTTTAAAAGTGCCTTAGGGG

AACCCTGTCCCTCCTAACAAGTGTATCTCGATTAATAACCTGCCAGTCCCAGATCACACATCATCATCGA

AGTCTTCCCCAGTTATAAAGAGGTCACATAGTCGTGTGGGTCGAGGATTCTGTGCCTCCAGGACCAGGGG

CCCACCCTCTGCCCAGGGAGTCCTTGCGTCCCATGAGGTCTTCCCGCAAGGCCTCTCAGACCCAGATGTG

ACGGGGTGTGTGGCCCGAGGAAGCTGGACAGCGGCAGTGGGCCTGCTGAGGCCTTCTCTTGAGGCCTGTG

CTCTGGGGGTCCCTTGCTTAGCCTGTGCTGGACCAGCTGGCCTGGGGTCCCTCTGAAGAGACCTTGGCTG

CTCACTGTCCACATGTGAACTTTTTCTAGGTGGCAGGACAAATTGCGCCCATTTAGAGGATGTGGCTGTA

ACCTGCTGGATGGGACTCCATAGCTCCTTCCCAGGACCCCTCAGCTCCCCGGCACTGCAGTCTGCAGAGT

TCTCCTGGAGGCAGGGGCTGCTGCCTTGTTTCACCTTCCATGTCAGGCCAGCCTGTCCCTGAAAGAGAAG

ATGGCCATGCCCTCCATGTGTAAGAACAATGCCAGGGCCCAGGAGGACCGCCTGCCCTGCCTGGGCCTTG

GCTGGGCCTCTGGTTCTGACACTTTCTGCTGGAAGCTGTCAGGCTGGGACAGGCTTTGATTTTGAGGGTT

AGCAAGACAAAGCAAATAAATGCCTTCCACCTCACCGCAAA

>NM_003932.5 Homo sapiens ST13 Hsp70 interacting protein (ST13), transcript variant 1, mRNA

CTTGCGGCAGCCGCCCCCTTCTGCGCGGTCACGCCGAGCCAGCGCCTGGGCCTGGAACCGGGCCGTAGCC

CCCCCAGTTTCGCCCACCACCTCCCTACCATGGACCCCCGCAAAGTGAACGAGCTTCGGGCCTTTGTGAA

AATGTGTAAGCAGGATCCGAGCGTTCTGCACACCGAGGAAATGCGCTTCCTGAGGGAGTGGGTGGAGAGC

ATGGGTGGTAAAGTACCACCTGCTACTCAGAAAGCTAAATCAGAAGAAAATACCAAGGAAGAAAAACCTG

ATAGTAAGAAGGTGGAGGAAGACTTAAAGGCAGACGAACCATCAAGTGAGGAAAGTGATCTAGAAATTGA

TAAAGAAGGTGTGATTGAACCAGACACTGATGCTCCTCAAGAAATGGGAGATGAAAATGCGGAGATAACG

GAGGAGATGATGGATCAGGCAAATGATAAAAAAGTGGCTGCTATTGAAGCCCTAAATGATGGTGAACTCC

AGAAAGCCATTGACTTATTCACAGATGCCATCAAGCTGAATCCTCGCTTGGCCATTTTGTATGCCAAGAG

GGCCAGTGTCTTCGTCAAATTACAGAAGCCAAATGCTGCCATCCGAGACTGTGACAGAGCCATTGAAATA

AATCCTGATTCAGCTCAGCCTTACAAGTGGCGGGGGAAAGCACACAGACTTCTAGGCCACTGGGAAGAAG

CAGCCCATGATCTTGCCCTTGCCTGTAAATTGGATTATGATGAAGATGCTAGTGCAATGCTGAAAGAAGT

TCAACCTAGGGCACAGAAAATTGCAGAACATCGGAGAAAGTATGAGCGAAAACGTGAAGAGCGAGAGATC

AAAGAAAGAATAGAACGAGTTAAGAAGGCTCGAGAAGAGCATGAGAGAGCCCAGAGGGAGGAAGAAGCCA

GACGACAGTCAGGAGCTCAGTATGGCTCTTTTCCAGGTGGCTTTCCTGGGGGAATGCCTGGTAATTTTCC

CGGAGGAATGCCTGGAATGGGAGGGGGCATGCCTGGAATGGCTGGAATGCCTGGACTCAATGAAATTCTT

AGTGATCCAGAGGTTCTTGCAGCCATGCAGGATCCAGAAGTTATGGTGGCTTTCCAGGATGTGGCTCAGA

ACCCAGCAAATATGTCAAAATACCAGAGCAACCCAAAGGTTATGAATCTCATCAGTAAATTGTCAGCCAA

ATTTGGAGGTCAAGCGTAATGTCCTTCTGATAAATAAAGCCCTTGCTGAAGGAAAAGCAACCTAGATCAC

CTTATGGATGTCGCAATAATACAAACCAGTGTACCTCTGACCTTCTCATCAAGAGAGCTGGGGTGCTTTG

AAGATAATCCCTACCCCTCTCCCCCAAATGCAGCTGAAGCATTTTACAGTGGTTTGCCATTAGGGTATTC

ATTCAGATAATGTTTTCCTACTAGGAATTACAAACTTTAAACACTTTTTAAATCTTCAAAATATTTAAAA

CAAATTTAAAGGGCCTGTTAATTCTTATATTTTTCTTTACTAATCATTTTGGATTTTTTTCTTTGAATTA

TTGGCAGGGAATATACTTATGTATGGAAGATTACTGCTCTGAGTGAAATAAAAGTTATTAGTGCGAGGCA

AACATAACTCATTTGAGGATAAAGTTTGTGTTGGATATGTGGTTCCTGATGCATTTTGACTTGTCTTTTT

AAATGCTTTATCTTTTTCTTTAAAGATTTATTTCAATAAAACTAATTGGGACCACCCGTATTTCAGTAGG

ACCTGGGTAGGGATTGGAAGTACTTGGCAGGGCAGCAGCAATCTTGCTGTGTTTGATATAACATGCATCC

TTGGGCAGGTTGCCCTTAAATCTTACACTGTGGTGAAGGGATGTTTTTTTTGTAATGCTGCAGTAGAGTT

GGAGTACTTAGTTCTCTTGTTGTCCAGTATATCTAATAAGTGTTTTTCATATTATTTCCACGTAAGGGAA

ATAAGGTAGTACTTTTCTTTTTATATTTCTATGCTTAAAATTCTCTTTCCTAGTCAAAAATTGCCCAAAT

CTGTGTTTGCTTTCTGCTTGCTACATTTGTCTCCCTTACTTTTCTTGAGCTAAAGACAGGCTTTTTCCAC

CGGCATCATCACTGCTATCATCATTAACAGCGTAATTATACAAGCATATTTAATGCTGAGTTTAATTTAA

TATGTAATACATATGGTAATTGTAGGGTAATACCCACAACAACTGTAGTTTCTTACTTGGCCAAGAGAAT

GCTTATTTAAGTGTTAGACTTCCATTCTGGCAAAATCTTGCCTTATCAGAAGACATTGGAAAGAGGGATT

CCCTTTGGTGTTTGGTCTTCTACTTAGAAAAACCTATTGCAGTTAGTTTATCTTGTAGTATTCATCTTTG

TATTCTGAAGATAAGGTTTGAATTAAATTGATACACACAGAGGGGAACCGATTTTTTTTATCCAATGTGA

ATTATAAATGAGATAATCCACAGTTATTCATTGTGGAGTTGTTGAGACTATGAAAGACTCATTGTCTTTG

TATTCAGCTCTTAAATAGTGTAACTATATCCCCACCTCTGCTTGCTTTCTTTCCCTCCCCTCCAATGATA

AAGAAAATGATAAATTTTCTGTTGTGCATTCAATTCTTATTTTAAATAAGACTAAGTATAGGCATTGTAC

CTGACATTGCTACGTTTCTACCAGTGTTTCAATTTAAAGTGCTAGTGTTTAAAAACATTTTCAAGGGATA

AGGCCTTCTGTACTTTGCTTATTTGAAGAATCAGTGGTAGGAGCAGTGAAGTAAATTCTATGGAGTACAT

TTCTAAAATACCACATTTCTGAAATCATAAATAAGTTTATTCAGGTTCTAACCCTTTGCTGTACACAAGC

AGACAGAAATGCATCTGTTACATAAATGAGAAAAAGCTATTATGCTGATGGAGCATGCTTTTTAAATCCT

TTAAAAACACTCACCATATAAACTTGCATTTGAGCTTGTGTGTTCTTTTGTTAATGTGTAGAGTTCTCCT

TTCTCGAAATTGCCAGTGTGTACTTGGCTTAACTCAAGAACAGTTTCTTCTGGATTCCTTATTTGATTTA

TTTAACCTAATTATATTCTAATATTGCAAATATTACCATAAGTGGGTAAAAGTAAAATTCCTCTTCTGAA

AATGTGTCCTGTGCTTTTAGATTTTTAAATTCCATAATATACATTCTTAATTTTCAACTCAA

>NM_000455.5 Homo sapiens serine/threonine kinase 11 (STK11), transcript variant 1, mRNA

GAGGTAAACAAGATGGCGGCGGCGTGTCGGGCGCGGAAGGGGGAGGCGGCCCGGGGCGCCCGCGAGTGAG

GCGCGGGGCGGCGAAGGGAGCGCGGGTGGCGGCACTTGCTGCCGCGGCCTTGGATGGGCTGGGCCCCCCT

CGCCGCTCCGCCTCCTCCACACGCGCGGCGGCCGCGGCGAGGGGGACGCGCCGCCCGGGGCCCGGCACCT

TCGGGAACCCCCCGGCCCGGAGCCTGCGGCCTGCGCCGCCTCGGCCGCCGGGAGCCCCGTGGAGCCCCCG

CCGCCGCGCCGCCCCGCGGACCGGACGCTGAGGGCACTCGGGGCGGGGCGCGCTCGGGCAGACGTTTG

CGGGGAGGGGGGCGCCTGCCGGGCCCCGGCGACCACCTTGGGGGTCGCGGGCCGGCTCGGGGGGCGCCCA

GTGCGGGCCCTCGCGGGCGCCGGGCAGCGACCAGCCCTGAGCGGAGCTGTTGGCCGCGGCGGGAGGCCTC

CCGGACGCCCCCAGCCCCCCGAACGCTCGCCCGGGCCGGCGGGAGTCGGCGCCCCCCGGGAGGTCCGCTC

GGTCGTCCGCGGCGGAGCGTTTGCTCCTGGGACAGGCGGTGGGACCGGGGCGTCGCCGGAGACGCCCCCA

GCGAAGTTGGGCTCTCCAGGTGTGGGGGTCCCGGGGGGTAGCGACGTCGCGGACCCGGCCTGTGGGATGG

GCGGCCCGGAGAAGACTGCGCTCGGCCGTGTTCATACTTGTCCGTGGGCCTGAGGTCCCCGGAGGATGAC

CTAGCACTGAAAAGCCCCGGCCGGCCTCCCCAGGGTCCCCGAGGACGAAGTTGACCCTGACCGGGCCGTC

TCCCAGTTCTGAGGCCCGGGTCCCACTGGAACTCGCGTCTGAGCCGCCGTCCCGGACCCCCGGTGCCCGC

CGGTCCGCAGACCCTGCACCGGGCTTGGACTCGCAGCCGGGACTGACGTGTAGAACAATCGTTTCTGTTG

GAAGAAGGGTTTTTCCCTTCCTTTTGGGGTTTTTGTTGCCTTTTTTTTTTCTTTTTTCTTTGTAAAATTT

TGGAGAAGGGAAGTCGGAACACAAGGAAGGACCGCTCACCCGCGGACTCAGGGCTGGCGGCGGGACTCCA

GGACCCTGGGTCCAGCATGGAGGTGGTGGACCCGCAGCAGCTGGGCATGTTCACGGAGGGCGAGCTGATG

TCGGTGGGTATGGACACGTTCATCCACCGCATCGACTCCACCGAGGTCATCTACCAGCCGCGCCGCAAGC

GGGCCAAGCTCATCGGCAAGTACCTGATGGGGGACCTGCTGGGGGAAGGCTCTTACGGCAAGGTGAAGGA

GGTGCTGGACTCGGAGACGCTGTGCAGGAGGGCCGTCAAGATCCTCAAGAAGAAGAAGTTGCGAAGGATC

CCCAACGGGGAGGCCAACGTGAAGAAGGAAATTCAACTACTGAGGAGGTTACGGCACAAAAATGTCATCC

AGCTGGTGGATGTGTTATACAACGAAGAGAAGCAGAAAATGTATATGGTGATGGAGTACTGCGTGTGTGG

CATGCAGGAAATGCTGGACAGCGTGCCGGAGAAGCGTTTCCCAGTGTGCCAGGCCCACGGGTACTTCTGT

CAGCTGATTGACGGCCTGGAGTACCTGCATAGCCAGGGCATTGTGCACAAGGACATCAAGCCGGGGAACC

TGCTGCTCACCACCGGTGGCACCCTCAAAATCTCCGACCTGGGCGTGGCCGAGGCACTGCACCCGTTCGC

GGCGGACGACACCTGCCGGACCAGCCAGGGCTCCCCGGCTTTCCAGCCGCCCGAGATTGCCAACGGCCTG

GACACCTTCTCCGGCTTCAAGGTGGACATCTGGTCGGCTGGGGTCACCCTCTACAACATCACCACGGGTC

TGTACCCCTTCGAAGGGGACAACATCTACAAGTTGTTTGAGAACATCGGGAAGGGGAGCTACGCCATCCC

GGGCGACTGTGGCCCCCCGCTCTCTGACCTGCTGAAAGGGATGCTTGAGTACGAACCGGCCAAGAGGTTC

TCCATCCGGCAGATCCGGCAGCACAGCTGGTTCCGGAAGAAACATCCTCCGGCTGAAGCACCAGTGCCCA

TCCCACCGAGCCCAGACACCAAGGACCGGTGGCGCAGCATGACTGTGGTGCCGTACTTGGAGGACCTGCA

CGGCGCGGACGAGGACGAGGACCTCTTCGACATCGAGGATGACATCATCTACACTCAGGACTTCACGGTG

CCCGGACAGGTCCCAGAAGAGGAGGCCAGTCACAATGGACAGCGCCGGGGCCTCCCCAAGGCCGTGTGTA

TGAACGGCACAGAGGCGGCGCAGCTGAGCACCAAATCCAGGGCGGAGGGCCGGGCCCCCAACCCTGCCCG

CAAGGCCTGCTCCGCCAGCAGCAAGATCCGCCGGCTGTCGGCCTGCAAGCAGCAGTGAGGCTGGCCGCCT

GCAGCCCGTGTCCAGGAGCCCCGCCAGGTGCCCGCGCCAGGCCCTCAGTCTTCCTGCCGGTTCCGCCCGC

CCTCCCGGAGAGGTGGCCGCCATGCTTCTGTGCCGACCACGCCCCAGGACCTCCGGAGCGCCCTGCAGGG

CCGGGCAGGGGGACAGCAGGGACCGGGCGCAGCCCTCCCCCCTCGGCCGCCCGGCAGTGCACGCGGCTTG

TTGACTTCGCAGCCCCGGGCGGAGCCTTCCCGGGCGGGCGTGGGAGGAGGGAGGCGGCCTCCATGCACTT

TATGTGGAGACTACTGGCCCCGCCCGTGGCCTCGTGCTCCGCAGGGCGCCCAGCGCCGTCCGGCGGCCCC

GCCGCAGACCAGCTGGCGGGTGTGGAGACCAGGCTCCTGACCCCGCCATGCATGCAGCGCCACCTGGAAG

CCGCGCGGCCGCTTTGGTTTTTTGTTTGGTTGGTTCCATTTTCTTTTTTTCTTTTTTTTTTTAAGAAAAA

ATAAAAGGTGGATTTGAGCTGTGGCTGTGAGGGGTGTTTGGGAGCTGCTGGGTGGCAGGGGGGCTGTGGG

GTCGGGCTCACGTCGCGGCCGCCTTTGCGCTCTCGGGTCACCCTGCTTTGGCGGCCCGGCCGGAGGGCAG

GACCCTCACCTCTCCCCCAAGGCCACTGCGCTCTTGGGACCCCAGAGAAAACCCGGAGCAAGCAGGAGTG

TGCGGTCAATATTTATATCATCCAGAAAAGAAAAACACGAGAAACGCCATCGCGGGATGGTGCAGACGCG

GCGGGGACTCGGAGGGTGCCGTGCGGGCGAGGCCGCCCAAATTTGGCAATAAATAAAGCTTGGGAAGCTT

GGA

>NM_013254.4 Homo sapiens TANK binding kinase 1 (TBK1), mRNA

GGAAGTGTCCTGAGTCTCGAGGAGGCCGCGGGAGCCCGCCGGCGGTGGCGCGGCGGAGACCCGGCTGGTA

TAACAAGAGGATTGCCTGATCCAGCCAAGATGCAGAGCACTTCTAATCATCTGTGGCTTTTATCTGATAT

TTTAGGCCAAGGAGCTACTGCAAATGTCTTTCGTGGAAGACATAAGAAAACTGGTGATTTATTTGCTATC

AAAGTATTTAATAACATAAGCTTCCTTCGTCCAGTGGATGTTCAAATGAGAGAATTTGAAGTGTTGAAAA

AACTCAATCACAAAAATATTGTCAAATTATTTGCTATTGAAGAGGAGACAACAACAAGACATAAAGTACT

TATTATGGAATTTTGTCCATGTGGGAGTTTATACACTGTTTTAGAAGAACCTTCTAATGCCTATGGACTA

CCAGAATCTGAATTCTTAATTGTTTTGCGAGATGTGGTGGGTGGAATGAATCATCTACGAGAGAATGGTA

TAGTGCACCGTGATATCAAGCCAGGAAATATCATGCGTGTTATAGGGGAAGATGGACAGTCTGTGTACAA

ACTCACAGATTTTGGTGCAGCTAGAGAATTAGAAGATGATGAGCAGTTTGTTTCTCTGTATGGCACAGAA

GAATATTTGCACCCTGATATGTATGAGAGAGCAGTGCTAAGAAAAGATCATCAGAAGAAATATGGAGCAA

CAGTTGATCTTTGGAGCATTGGGGTAACATTTTACCATGCAGCTACTGGATCACTGCCATTTAGACCCTT

TGAAGGGCCTCGTAGGAATAAAGAAGTGATGTATAAAATAATTACAGGAAAGCCTTCTGGTGCAATATCT

GGAGTACAGAAAGCAGAAAATGGACCAATTGACTGGAGTGGAGACATGCCTGTTTCTTGCAGTCTTTCTC

GGGGTCTTCAGGTTCTACTTACCCCTGTTCTTGCAAACATCCTTGAAGCAGATCAGGAAAAGTGTTGGGG

TTTTGACCAGTTTTTTGCAGAAACTAGTGATATACTTCACCGAATGGTAATTCATGTTTTTTCGCTACAA

CAAATGACAGCTCATAAGATTTATATTCATAGCTATAATACTGCTACTATATTTCATGAACTGGTATATA

AACAAACCAAAATTATTTCTTCAAATCAAGAACTTATCTACGAAGGGCGACGCTTAGTCTTAGAACCTGG

AAGGCTGGCACAACATTTCCCTAAAACTACTGAGGAAAACCCTATATTTGTAGTAAGCCGGGAACCTCTG

AATACCATAGGATTAATATATGAAAAAATTTCCCTCCCTAAAGTACATCCACGTTATGATTTAGACGGGG

ATGCTAGCATGGCTAAGGCAATAACAGGGGTTGTGTGTTATGCCTGCAGAATTGCCAGTACCTTACTGCT

TTATCAGGAATTAATGCGAAAGGGGATACGATGGCTGATTGAATTAATTAAAGATGATTACAATGAAACT

GTTCACAAAAAGACAGAAGTTGTGATCACATTGGATTTCTGTATCAGAAACATTGAAAAAACTGTGAAAG

TATATGAAAAGTTGATGAAGATCAACCTGGAAGCGGCAGAGTTAGGTGAAATTTCAGACATACACACCAA

ATTGTTGAGACTTTCCAGTTCTCAGGGAACAATAGAAACCAGTCTTCAGGATATCGACAGCAGATTATCT

CCAGGTGGATCACTGGCAGACGCATGGGCACATCAAGAAGGCACTCATCCGAAAGACAGAAATGTAGAAA

AACTACAAGTCCTGTTAAATTGCATGACAGAGATTTACTATCAGTTCAAAAAAGACAAAGCAGAACGTAG

ATTAGCTTATAATGAAGAACAAATCCACAAATTTGATAAGCAAAAACTGTATTACCATGCCACAAAAGCT

ATGACGCACTTTACAGATGAATGTGTTAAAAAGTATGAGGCATTTTTGAATAAGTCAGAAGAATGGATAA

GAAAGATGCTTCATCTTAGGAAACAGTTATTATCGCTGACTAATCAGTGTTTTGATATTGAAGAAGAAGT

ATCAAAATATCAAGAATATACTAATGAGTTACAAGAAACTCTGCCTCAGAAAATGTTTACAGCTTCCAGT

GGAATCAAACATACCATGACCCCAATTTATCCAAGTTCTAACACATTAGTAGAAATGACTCTTGGTATGA

AGAAATTAAAGGAAGAGATGGAAGGGGTGGTTAAAGAACTTGCTGAAAATAACCACATTTTAGAAAGGTT

TGGCTCTTTAACCATGGATGGTGGCCTTCGCAACGTTGACTGTCTTTAGCTTTCTAATAGAAGTTTAAGA

AAAGTTTCCGTTTGCACAAGAAAATAACGCTTGGGCATTAAATGAATGCCTTTATAGATAGTCACTTGTT

TCTACAATTCAGTATTTGATGTGGTCGTGTAAATATGTACAATATTGTAAATACATAAAAAATATACAAA

TTTTTGGCTGCTGTGAAGATGTAATTTTATCTTTTAACATTTATAATTATATGAGGAAATTTGACCTCAG

TGATCACGAGAAGAAAGCCATGACCGACCAATATGTTGACATACTGATCCTCTACTCTGAGTGGGGCTAA

ATAAGTTATTTTCTCTGACCGCCTACTGGAAATATTTTTAAGTGGAACCAAAATAGGCATCCTTACAAAT

CAGGAAGACTGACTTGACACGTTTGTAAATGGTAGAACGGTGGCTACTGTGAGTGGGGAGCAGAACCGCA

CCACTGTTATACTGGGATAACAATTTTTTTGAGAAGGATAAAGTGGCATTATTTTATTTTACAAGGTGCC

CAGATCCCAGTTATCCTTGTATCCATGTAATTTCAGATGAATTATTAAGCAAACATTTTAAAGTGAATTC

ATTATTAAAAACTATTCATTTTTTTCCTTTGGCCATAAATGTGTAATTGTCATTAAAATTCTAAGGTCAT

TTCAACTGTTTTAAGCTGTATATTTCTTTAATTCTGCTTACTATTTCATGGAAAAAAATAAATTTCTCAA

TTTTAATGTAAA

>NM_006405.7 Homo sapiens transmembrane 9 superfamily member 1 (TM9SF1), transcript variant 1, mRNA

ACCGCGCCGCTGTGGGAGAGACCCCAGCAGGAGCCCAAAGGCAGCTACGGGGGCGCGAAGGCCGCTGGCG

CCGCCTCGGCCAGCCCTTCCCGCGCGGTTCCACTGCCTTAAGGATGACAGTCGTAGGGAACCCTCGAAGT

TGGAGCTGCCAGTGGTTGCCAATCCTGATACTGTTGCTGGGCACAGGCCATGGGCCAGGGGTGGAAGGCG

TGACACACTACAAGGCCGGCGACCCTGTTATTCTGTATGTCAACAAAGTGGGACCCTACCATAACCCTCA

GGAAACTTACCACTACTATCAGCTTCCAGTCTGCTGCCCTGAGAAGATACGTCACAAAAGCCTTAGCCTG

GGTGAAGTGCTGGATGGGGACCGAATGGCTGAGTCTTTGTATGAGATCCGCTTTCGGGAAAACGTGGAGA

AGAGAATTCTGTGCCACATGCAGCTCAGTTCTGCACAGGTGGAGCAGCTGCGCCAGGCCATTGAAGAACT

GTACTACTTTGAATTTGTGGTAGATGACTTGCCAATCCGGGGCTTTGTGGGCTACATGGAGGAGAGTGGT

TTCCTGCCACACAGCCACAAGATAGGACTCTGGACCCATTTGGACTTCCACCTAGAATTCCATGGAGACC

GAATTATATTTGCCAATGTTTCAGTGCGGGACGTCAAGCCCCACAGCTTGGATGGGTTACGACCTGACGA

GTTCCTAGGCCTTACCCACACTTATAGCGTGCGCTGGTCTGAGACTTCAGTGGAGCGTCGGAGTGACAGG

CGCCGTGGTGACGATGGTGGTTTCTTTCCTCGAACACTGGAAATCCATTGGTTGTCCATCATCAACTCCA

TGGTGCTTGTGTTTTTACTGGTGGGTTTTGTGGCTGTCATTCTAATGCGTGTGCTTCGGAATGACCTGGC

TCGGTACAACTTAGATGAGGAGACCACCTCTGCAGGTTCTGGTGATGACTTTGACCAGGGTGACAATGGC

TGGAAAATTATCCATACAGATGTCTTCCGCTTCCCCCCATACCGTGGTCTGCTCTGTGCTGTGCTTGGCG

TGGGTGCCCAGTTCCTGGCCCTTGGCACTGGCATTATTGTCATGGCACTGCTGGGCATGTTCAATGTGCA

CCGTCATGGGGCCATTAACTCAGCAGCCATCTTGTTGTATGCCCTGACCTGCTGCATCTCTGGCTACGTG

TCCAGCCACTTCTACCGGCAGATTGGAGGCGAGCGTTGGGTGTGGAACATCATTCTCACCACCAGTCTCT

TCTCTGTGCCTTTCTTCCTGACGTGGAGTGTGGTGAACTCAGTGCATTGGGCCAATGGTTCGACACAGGC

TCTGCCAGCCACAACCATCCTGCTGCTTCTGACGGTTTGGCTGCTGGTGGGCTTTCCCCTCACTGTCATT

GGAGGCATCTTTGGGAAGAACAACGCCAGCCCCTTTGATGCACCCTGTCGCACCAAGAACATCGCCCGGG

AGATTCCACCCCAGCCCTGGTACAAGTCTACTGTCATCCACATGACTGTTGGAGGCTTCCTGCCTTTCAG

TGCCATCTCTGTGGAGCTGTACTACATCTTTGCCACAGTATGGGGTCGGGAGCAGTACACTTTGTACGGC

ATCCTCTTCTTTGTCTTCGCCATCCTGCTGAGTGTGGGGGCTTGCATCTCCATTGCACTCACCTACTTCC

AGTTGTCTGGGGAGGATTACCGCTGGTGGTGGCGATCTGTGCTGAGTGTTGGCTCCACCGGCCTCTTCAT

CTTCCTCTACTCAGTTTTCTATTATGCCCGGCGCTCCAACATGTCTGGGGCAGTACAGACAGTAGAGTTC

TTCGGCTACTCCTTACTCACTGGTTATGTCTTCTTCCTCATGCTGGGCACCATCTCCTTTTTTTCTTCCC

TAAAGTTCATCCGGTATATCTATGTTAACCTCAAGATGGACTGAGTTCTGTATGGCAGAACTATTGCTGT

TCTCTCCCTTTCTTCATGCCCTGTTGAACTCTCCTACCAGCTTCTCTTCTGATTGACTGAATTGTGTGAT

GGCATTGTTGCCTTCCCTTTTGCCCTTTGGGCATTCCTTCCCCAGAGAGGGCCTGGAAATTATAAATCTC

TATCACATAAGGATTATATATTTGAACTTTTTAAGTTGCCTTTAGTTTTGGTCCTGATTTTTCTTTTTAC

AATTACCAAAATAAAATTTATTAAGAAAAA

>NM_001165030.3 Homo sapiens transmembrane protein 41B (TMEM41B), transcript variant 2, mRNA

GCAGTACTCGCTGGGCTGAGGTCGTCTCGGGTGAGGAGGTCGCGCCGGAAGTGGCTCTAGCCGTGGCGCT

TCGGCGTAGTAAGGAGCCTGCAACAGAGTTTTGTTGTTTAGGGGGCGCGGCACCGCACCGCTCCCTTCAC

CTTGCAGCAGCCATGGCGAAAGGCAGAGTCGCCGAACGATCGCAGTTGGGCGCTCACCACACGACCCCCG

TGGGGGACGGGGCAGCGGGGACGCGGGGTCTCGCGGCGCCTGGCAGCAGAGACCACCAGAAGGAAAAATC

CTGGGTAGAAGCTGGATCAGCAAGAATGTCACTCCTTATATTGGTGTCCATTTTCTTATCTGCAGCTTTT

GTTATGTTTTTGGTATATAAAAATTTTCCTCAGCTTAGTGAAGAAGAAAGAGTGAATATGAAGGTTCCCA

GAGATATGGATGATGCCAAGGCTCTAGGAAAAGTTTTATCCAAATACAAGGACACCTTTTATGTTCAAGT

ACTTGTAGCTTATTTTGCTACATATATTTTGTATCCTTTTAACTGAAAAAATGTTTTCTAATTTTGTTCA

AGAAAAAGTGTAAAAGGTCTGCTCTTCATTTATACATAACTATTTTCCTTTTAAAATTTGTTATTGCAGT

GTGACAATATTAAATAATTGAAAAATTAAAATGGAAAAAAATTATCACTTTAA

>NM_153015.3 Homo sapiens transmembrane protein 74 (TMEM74), transcript variant 1, mRNA

GGAGCCGAGCGGCGTGCAGAGGCTACAAGTGCCGTAGCTGGTGATTGGGGGACTTTCTCCGGGAACCGTG

CCGGGAGAGCGCGCGGTGCTGGAGCCGCACCGGGTGGCCGAAGCAGAAGACTTTCCGGAAGCTGCTGGGG

GATGTCTGACTAGCTCTCATGGAGCTCCACTACCTTGCTAAGAAGAGCAACCAGGCAGACCTCTGTGATG

CCAGGGACTGGAGTTCAAGAGGGCTGCCTGGTGACCAGGCAGATACAGCAGCCACAAGAGCTGCTCTCTG

CTGTCAGAAACAGTGTGCATCCACCCCAAGAGCAACCGAGATGGAAGGGTCTAAACTTAGTTCTTCTCCA

GCATCCCCCTCCTCCTCTCTGCAAAACAGTACTCTTCAGCCAGATGCCTTTCCACCAGGACTTCTCCACT

CAGGGAACAACCAAATAACAGCGGAACGGAAAGTCTGTAACTGCTGCAGCCAGGAATTAGAAACTTCTTT

TACCTATGTGGACAAAAACATCAACTTGGAGCAGCGGAACCGGAGCTCGCCATCAGCAAAAGGGCATAAT

CACCCTGGGGAGCTTGGCTGGGAAAATCCAAATGAGTGGTCCCAAGAGGCTGCCATATCTTTGATATCTG

AAGAGGAGGATGATACAAGTTCAGAAGCCACGTCTTCAGGGAAGTCTATAGACTATGGTTTCATCAGCGC

CATCTTGTTCTTGGTCACTGGGATCCTGCTCGTGATCATCTCTTACATCGTCCCACGGGAAGTGACTGTG

GACCCCAACACTGTGGCAGCCCGGGAGATGGAGCGCCTGGAGAAGGAGAGTGCGAGGCTGGGGGCTCACC

TGGACCGCTGTGTGATTGCGGGGCTCTGCCTCCTCACGCTGGGGGGCGTCATCCTGTCCTGCTTGTTAAT

GATGTCCATGTGGAAGGGGGAGCTCTATCGTCGAAACAGATTTGCCTCTTCCAAAGAGTCTGCAAAACTC

TATGGTTCTTTCAACTTCAGGATGAAAACCAGCACGAATGAAAACACTCTGGAACTGTCCTTGGTAGAGG

AAGATGCGCTTGCTGTACAGAGTTAATTCTGGTTGTGAATATCTTGAGAGTCTGCCTTGGCATTTTATAA

TATGAAAAAAGTTAATTTATAAAAATTCACAGTGCAATTTATTTGCCTGGCAAGAAAAGTTTATTTCACA

AACCAACAGCCAGTAAGTGTTTTTGTTCTCTATGTGTCTTCTATTTAGAAGAAAAGCCATGTAAGATGTA

TAAGAAACCACAACCAGCCACACCTATCCTTCTGAAGAGCTGAAGGCTAATTAATCTGTAATGGCCAAGA

ACTTCTACTTCGATAGAAAAATATTTCTAATGACCCAGTCTACAAATTATTTCTTTTACACAAATATATG

ATGTTATTCTTTGGACACTAGGTGGTCCTACACACAGTAGGATCAATTGCTAATCTACTTTGTGAAAAAG

AACTAAGCACTAATCAATAATAAGGCTTACATCTAATTCTCAAAGGTGCTTATCCATTTTCTTGCTAAAT

TATCCTTCTTGTAATTTGGCTAAACACTAAAACATGGAATTTTTAGTTTGAATATTTTGAAGTTTGAGGA

TGTTGGGCTTTCCTTATTGTAAAAAATGTTATGTTTGAAATTATTCCTGTTTTCAAAAATGGTAATTAAG

TCATTAGGATAAACTTTCTAATAAAAAAAAATTATGTAAATCATTGTAAGACCAATGTGAATTTAAACCA

CAGTGTTGGTGCCAGATATTAGCCAGGGTCACAGGCTCTTTTTTAAAATGAAGTCAAATTAGTTACTGAT

ATAAGTTTTATATTTTGTGATTTTGCCATCATACTGGTTATCTGTGCTCTTGAAGTCTGCTGGTTCTCAC

ACCAGCAGCAAGAGGCAAGGCATGAGACATTTTAAATATATTTCCAGACATCTAAACATCTAGACAGGCC

TGAGTCATGATTGGCTCTGGTGTGCTTCCAGGTAACATCTTGTTATTGCTGTTTGGGGAAAACCACCAAT

AGAATTGCAGCTAGACTTTTGGGGACTGTGCCTCGCAGGCTGTATCACTTGCTGTAGGCCAAGCAACGAG
CACTTCCCTCATGGTAGCAACAGTCCTTAAGCCCAAAGCCAAAAAAAAATAAAATGGAAAGAGAGATTCA
ATACACTGATTTAATGGGGACTGTAGTCATCGTCAGAACTCACCTCCCACTCACAGTGGTCTAGATGTCA
TTCAGCAACTGTGAACTCTTGCCTCATGCAGCACAAACTCTTCTCTAAGAGCAGCATGATGAGCAAGGTG
CTCACTTTGCAAGACACACACTGGGTGTTCACAGTGACAGACCGCCTCATTATGGTGACGGTTCAGAAAA
GGGATGGGAAATGCAGCCTCAGAAATACCTTACATTTTGCAACTCTGCCTGGAGGTTTTTCCAACCTGTG
CTCTAAGAGTTGTTGTAACTTCCATGATTGTGTTACCAGGATCAGGGGGTCATTCTTAAAAAGAGGACAG
GAGCTGGGAATTGGTATGAGACAGCAGGAATGAGGAAGTAAGAAAGTGATTTCTTTGCAGATTTTTAAAG
GCTATTTCAAATGTCTGAAGTTTGTGGTGTTGTGGACAACTGTGGAATACATGGGTGACTTTGGCAAAAT
GTGTGAATCAGGATATTTATGTGGATGAGCTCTCATGGGTGTCAGATTGATTTGATGAGTGAGGTATGCT
GCATATGACTTAAGGGTTGAACTGAAATACAAAAAGGGAGGCTCAGAAGATAGTAAAAAGAGAGGATGGG
CATATGAAAGAATTATTAAGTAAAAATGAGAAATTATATGGCCATCAGAGGAAGCTAGATGTAGTTAATT
GCCTTATGTTATAGAGCAATACTGATTACTGAAAAGAAAGAATTGAGATGACATAAACATGATTTTTGCT
TTCTGTTGCTGTATTTTTGTGCTTTTTGAACAAACTTTGTTTTACTGTGACTCTCATTTCATGATTTTTG
TTGAGGCTGCCTTACCAGTGGTATAAGTTTTATATTCTGAAGGTTTCTAAGTCCAGCTCTAGCCTGCTTA
AGGATCCACTTTGGATGATGTTGAAACAAGAAGGAAACGATCTGCTGAGTGAGTAGGTCTGAGCTGTGTG
TTAAAAAAAGAAGGCATTAAAAAAAAAGAAGGCATTAAAGAAAAAACTAATGTTTATTCATAGAAGCTAAA
TGAGATCAACTGAGTTTTTATTTATTGGTGAAACAGGATTAATTAGCCAAATGGCAGACTAAACTGAATT
AATGTGGAATCAATTTTCAGTTTATGAAGGAAGAGGAGTTTGGGAATCAAGTGTTAGGAGGTCTTTTCAC
TTGATAATAAAGCTTTTCATGTGGAAGCATTTAATGAATCTTGCAGGGCTACCAAATAATATAGATTTGA
TCTAGAGGTAACATTTTGAAAAATGCCATTTTGGAAAAAAAATATTCAACGGGGGAGAAAAAGACCTAAC
ACATCAAAATACATTTCCTTTCTTTTCCCTAGAATGCTATAATTTCCTTTAAAATTTAAAATCGAAATGT
GGTAAGTAAATGCACACTTCAACATTATTTAAATCTGCTAACATATAGAGCACCTCTCTTTTAAGGTTCT
ACTTATCTGAGGGCAAACCATTACACATTACATGATTTTGAAAATCATCTGAATGACTTCTTTTGGGCTG
TGGTGACATTATTCAGATACTTGCCTTAATCGACTTTTCCCTGGGTATTTGGTTTATGTAGCCCTTATCC

ATCCTGCCTGAATTCAATATGAAGATTACTTGAATTTAAAATGATGGTAACACAGGCAAAGAAAAGCCAT

GTGCTTTATTTCCTGTTTTTCCCAATCTGACAAAGAGTGTTTTTGGCTCTAGAGGTACTTTGGCAAGACC

AGGTGATGCATTCTATAGACATTGGGCATACAATACCTATAGTTTTGGTCTTGCATCCTTCATGGAGATT

TGTGACCTTCCGTCTGTCTCTTCAATATGGTGCTGGTTGTTACCTGTGAAAGTTTTTATTGCTAGTCCTA

GAGAAGAAAAACATATTTCAGTGGTTCTAGGTCTTTAGCTTATTTACATGGTTTTAGAAATCTAAACTCA

AGAGTCATCTTCCTAGTCTCAATGAGAAGACAGCTCGTCTTTCAGCAGTGTGGCCCAATGGGGTGAGTTA

CAGAAAGAAAGCTGTCTTTCTCCTTTATTACTGGGATCTACACGCTGCTGGTGGATGATTTAAGGCTCCT

AATATCTTTATAAGCCAGTTCTTTTTAGGGCACCTTAAACTGTTAGTGGTAGAGTTTGCAAAGAGTTTTC

TTTGCAGCGCTGTGTGTCCTTCCCACCCATTGGGAGCTCTTAAAGCAGATCACATAATAAGATGTACTGG

CCCCTCATATAAAGAACAGCTTTTCGTCATGCTCCCCACTGTGTAGGTGATTTATAGTTTACAGGAAGAC

AAGACTGCTTGGAACCATGAAGTTCTTGGAGGCCATGGTTGCTTTTACCCACATGACTGCTATAACCCTT

GATTCTGTTCTATCAAGTTACTTGCCTTGATATCATCACATGCTTTGCTGTCTGCTTAAAATATGAGTTC

ATCCCCCTCATACTTATTTGTTTTAGGGACCCAATTGGACCAGTGCTGGCACATGCCCTTCAAGGTACAA

TTTCTGAAAAGAGACTGCGCTGCCAAACTTATGTGTGCCTTTACCTCAGTCCCCCCAGTATAAGGGGTGA

CTTAATCTTGATAAATGAGTGACTGAGCACCATGCTAGTTAAATATTGAGAATTAAAAGCCTGAACCCAA

TGGCTCTATTTTTTTTTACCACCTTCTGACAAGCAAAAATTCTAGGCAGGAGCATGTGAACATTTTGGTC

TTCATCTTTCACGTGTGCAATGATAGGTGTATGCAAAATTTTACTTGGATTAATTGTCAAGTTAAAAAAG

TGTTCTATATGTATATCTCTTAGGAGAAGGTTTTTCAACCTGAAGGTCAGATTTCATTGCAGCAATTAAT

GCCATCAAAAAAACGTGGTCTGACAAAAAAGATTCACAACCTTTGCCAGTGGTTCACCTATTACAGCAAG

ATGTAATACTATCAATACTTACTGTTCTCTGTAATGGGTTTAACCTTTCCCAGAATTGGACATAAAGAAT

GACTACGATGAGAAGATTATAATGAAGTCTTACTGGCTTTTCCTAGTCTAAAAAGAGTACTTAAGAAAGA

GAATTAACATATCATTTCCTTTTGGCTGATTTTTAGCTATATCTGTTTTTGGTTCATAATATTCTATATT

ATCATCTCACAAATTTTATAAGTCTTGGGAAATAATTTGCCTGCCTAGTGAATTGACACTGTAAAAAGCA

AGACGTTATTTTAAACACATAAAACTAATTTCAAGAATCTGTGGCTTACACTCAAGAGTGTACTCGTGGT

TTATGTTTGTGTGCAGATTTATGTGCACTCAAGTCTATGTGCATTTGGACTATATTTTATAGTTATACAG

CTTTAATATCCAAAGTTATTGTTAGAATAATTTTATTATAGGAATTTGGCATGCCTTACTATTGACATTA

AGAAGAAGAGAAGTCTTTCACAAGTCATGAATGAAGAATTTGGAATCAGCCTCAGAATGAGATGCCATAA

TTATTATAAACCTGTTTTGGTTGTTCAGCCTCCCACAAATATTTATTAATTTGTTTTTAACCTCTCAAT

TTCAGGCTCATGAGAGTGTGAGATATTTGGAAATTATTTTACCGTGAATTGTTATTTTATTTCAGGGGAC

TATGGGGAGTATATTCCTTTGTTTCAAATTTGGGTAACTCTCCTGTGTTTCAGGAACAGCTCTCCCTATT

TTTTCACTGCCATCTTCAGGCTTCCTAGCCAGGCTCCAAACAAATGTCATATAAGAAAGAAAGATTAATT

ATTACATATTGTGTATTGAGAGTTTGTACCCTGTGGTTCTCATCGTGCTGTTAATTTTACTTTTCTTGCC

AAACAAAAATAATTTGACATTGATTATGTGCTTTTTGATATTTTCAAGGGCAATTATTAAGGTTATGAGC

TCTTTCTTAGGGCACTTGATGCCTTATATGTTAATAGCAAGTGTGTCATATCATTTTAAAATAAAACTAT

TCAAAGGCTTTATTGTGTTTATTAAGCTTAATTTAAATTGGAATCTGTAATGCTGATTTGGTCTTTTAAA

ATTATATTTGAAATTATTTGTCCAAAAAAATTAAAATGAATATTTACCCCTAA

>NM_001190942.2 Homo sapiens TNF superfamily member 10 (TNFSF10), transcript variant 2, mRNA

GACCGGCTGCCTGGCTGACTTACAGCAGTCAGACTCTGACAGGATCATGGCTATGATGGAGGTCCAGGGG

GGACCCAGCCTGGGACAGACCTGCGTGCTGATCGTGATCTTCACAGTGCTCCTGCAGTCTCTCTGTGTGG

CTGTAACTTACGTGTACTTTACCAACGAGCTGAAGCAGATGCAGGACAAGTACTCCAAAAGTGGCATTGC

TTGTTTCTTAAAAGAAGATGACAGTTATTGGGACCCCAATGACGAAGAGAGTATGAACAGCCCCTGCTGG

CAAGTCAAGTGGCAACTCCGTCAGCTCGTTAGAAAGACTCCAAGAATGAAAAGGCTCTGGGCCGCAAAAT

AAACTCCTGGGAATCATCAAGGAGTGGGCATTCATTCCTGAGCAACTTGCACTTGAGGAATGGTGAACTG

GTCATCCATGAAAAAGGGTTTTACTACATCTATTCCCAAACATACTTTCGATTTCAGGAGGAAATAAAAG

AAAACACAAAGAACGACAAACAAATGGTCCAATATATTTACAAATACACAAGTTATCCTGACCCTATATT

GTTGATGAAAAGTGCTAGAAATAGTTGTTGGTCTAAAGATGCAGAATATGGACTCTATTCCATCTATCAA

GGGGGAATATTTGAGCTTAAGGAAAATGACAGAATTTTTGTTTCTGTAACAAATGAGCACTTGATAGACA

TGGACCATGAAGCCAGTTTTTTTGGGGCCTTTTTAGTTGGCTAACTGACCTGGAAAGAAAAAGCAATAAC

CTCAAAGTGACTATTCAGTTTTCAGGATGATACACTATGAAGATGTTTCAAAAAATCTGACCAAAACAAA

CAAACAGAAAACAGAAAACAAAAAAACCTCTATGCAATCTGAGTAGAGCAGCCACAACCAAAAAATTCTA

CAACACACACTGTTCTGAAAGTGACTCACTTATCCCAAGAGAATGAAATTGCTGAAAGATCTTTCAGGAC

TCTACCTCATATCAGTTTGCTAGCAGAAATCTAGAAGACTGTCAGCTTCCAAACATTAATGCAATGGTTA

ACATCTTCTGTCTTTATAATCTACTCCTTGTAAAGACTGTAGAAGAAAGAGCAACAATCCATCTCTCAAG

TAGTGTATCACAGTAGTAGCCTCCAGGTTTCCTTAAGGGACAACATCCTTAAGTCAAAAGAGAGAAGAGG

CACCACTAAAAGATCGCAGTTTGCCTGGTGCAGTGGCTCACACCTGTAATCCCAACATTTTGGGAACCCA

AGGTGGGTAGATCACGAGATCAAGAGATCAAGACCATAGTGACCAACATAGTGAAACCCCATCTCTACTG

AAAGTACAAAAATTAGCTGGGTGTGTTGGCACATGCCTGTAGTCCCAGCTACTTGAGAGGCTGAGGCAAG

AGAATTGTTTGAACCCGGGAGGCAGAGGTTGCAGTGTGGTGAGATCATGCCACTACACTCCAGCCTGGCG

ACAGAGCGAGACTTGGTTTCAAAAAAAAAAAAAAAAAAAACTTCAGTAAGTACGTGTTATTTTTTTCAAT

AAAATTCTATTACAGTATGTCATGTTTGCTGTAGTGCTCATATTTATTGTTGTTTTTGTTTTAGTACTCA

CTTGTTTCATAATATCAAGATTACTAAAAATGGGGGAAAAGACTTCTAATCTTTTTTTCATAATATCTTT

GACACATATTACAGAAGAAATAAATTTCTTACTTTTAATTTAATATGA

>NM_000546.6 Homo sapiens tumor protein p53 (TP53), transcript variant 1, mRNA

CTCAAAAGTCTAGAGCCACCGTCCAGGGAGCAGGTAGCTGCTGGGCTCCGGGGACACTTTGCGTTCGGGC

TGGGAGCGTGCTTTCCACGACGGTGACACGCTTCCCTGGATTGGCAGCCAGACTGCCTTCCGGGTCACTG

CCATGGAGGAGCCGCAGTCAGATCCTAGCGTCGAGCCCCCTCTGAGTCAGGAAACATTTTCAGACCTATG

GAAACTACTTCCTGAAAACAACGTTCTGTCCCCCTTGCCGTCCCAAGCAATGGATGATTTGATGCTGTCC

CCGGACGATATTGAACAATGGTTCACTGAAGACCCAGGTCCAGATGAAGCTCCCAGAATGCCAGAGGCTG

CTCCCCCCGTGGCCCCTGCACCAGCAGCTCCTACACCGGCGGCCCCTGCACCAGCCCCCTCCTGGCCCCT

GTCATCTTCTGTCCCTTCCCAGAAAACCTACCAGGGCAGCTACGGTTTCCGTCTGGGCTTCTTGCATTCT

GGGACAGCCAAGTCTGTGACTTGCACGTACTCCCCTGCCCTCAACAAGATGTTTTGCCAACTGGCCAAGA

CCTGCCCTGTGCAGCTGTGGGTTGATTCCACACCCCCGCCCGGCACCCGCGTCCGCGCCATGGCCATCTA

CAAGCAGTCACAGCACATGACGGAGGTTGTGAGGCGCTGCCCCCACCATGAGCGCTGCTCAGATAGCGAT

GGTCTGGCCCCTCCTCAGCATCTTATCCGAGTGGAAGGAAATTTGCGTGTGGAGTATTTGGATGACAGAA

ACACTTTTCGACATAGTGTGGTGGTGCCCTATGAGCCGCCTGAGGTTGGCTCTGACTGTACCACCATCCA

CTACAACTACATGTGTAACAGTTCCTGCATGGGCGGCATGAACCGGAGGCCCATCCTCACCATCATCACA

CTGGAAGACTCCAGTGGTAATCTACTGGGACGGAACAGCTTTGAGGTGCGTGTTTGTGCCTGTCCTGGGA

GAGACCGGCGCACAGAGGAAGAGAATCTCCGCAAGAAAGGGGAGCCTCACCACGAGCTGCCCCCAGGGAG

CACTAAGCGAGCACTGCCCAACAACACCAGCTCCTCTCCCCAGCCAAAGAAGAAACCACTGGATGGAGAA

TATTTCACCCTTCAGATCCGTGGGCGTGAGCGCTTCGAGATGTTCCGAGAGCTGAATGAGGCCTTGGAAC

TCAAGGATGCCCAGGCTGGGAAGGAGCCAGGGGGGAGCAGGGCTCACTCCAGCCACCTGAAGTCCAAAAA

GGGTCAGTCTACCTCCCGCCATAAAAAACTCATGTTCAAGACAGAAGGGCCTGACTCAGACTGACATTCT

CCACTTCTTGTTCCCCACTGACAGCCTCCCACCCCCATCTCTCCCTCCCCTGCCATTTTGGGTTTTGGGT

CTTTGAACCCTTGCTTGCAATAGGTGTGCGTCAGAAGCACCCAGGACTTCCATTTGCTTTGTCCCGGGGC

TCCACTGAACAAGTTGGCCTGCACTGGTGTTTTGTTGTGGGGAGGAGGATGGGGAGTAGGACATACCAGC

TTAGATTTTAAGGTTTTTACTGTGAGGGATGTTTGGGAGATGTAAGAAATGTTCTTGCAGTTAAGGGTTA

GTTTACAATCAGCCACATTCTAGGTAGGGGCCCACTTCACCGTACTAACCAGGGAAGCTGTCCCTCACTG

TTGAATTTTCTCTAACTTCAAGGCCCATATCTGTGAAATGCTGGCATTTGCACCTACCTCACAGAGTGCA

TTGTGAGGGTTAATGAAATAATGTACATCTGGCCTTGAAACCACCTTTTATTACATGGGGTCTAGAACTT

GACCCCCTTGAGGGTGCTTGTTCCCTCTCCCTGTTGGTCGGTGGGTTGGTAGTTTCTACAGTTGGGCAGC

TGGTTAGGTAGAGGGAGTTGTCAAGTCTCTGCTGGCCCAGCCAAACCCTGTCTGACAACCTCTTGGTGAA

CCTTAGTACCTAAAAGGAAATCTCACCCCATCCCACACCCTGGAGGATTTCATCTCTTGTATATGATGAT

CTGGATCCACCAAGACTTGTTTTATGCTCAGGGTCAATTTCTTTTTTCTTTTTTTTTTTTTTTTTTCTTT

TTCTTTGAGACTGGGTCTCGCTTTGTTGCCCAGGCTGGAGTGGAGTGGCGTGATCTTGGCTTACTGCAGC

CTTTGCCTCCCCGGCTCGAGCAGTCCTGCCTCAGCCTCCGGAGTAGCTGGGACCACAGGTTCATGCCACC

ATGGCCAGCCAACTTTTGCATGTTTTGTAGAGATGGGGTCTCACAGTGTTGCCCAGGCTGGTCTCAAACT

CCTGGGCTCAGGCGATCCACCTGTCTCAGCCTCCCAGAGTGCTGGGATTACAATTGTGAGCCACCACGTC

CAGCTGGAAGGGTCAACATCTTTTACATTCTGCAAGCACATCTGCATTTTCACCCCACCCTTCCCCTCCT

TCTCCCTTTTTATATCCCATTTTTATATCGATCTCTTATTTTACAATAAAACTTTGCTGCCA

>NM_021202.3 Homo sapiens tumor protein p53 inducible nuclear protein 2 (TP53INP2), transcript variant 1, mRNA

GCACAGACTCAAAGCCCCGCGGGCGAGCTCAGCAGCCCGGAGCGACCGCGGCCCCGCCGCCTCCCCCGCG

AGTCCCGGCGATGCGGCCCGGCCTGTGAGCGGCCGGCGACCCTGGGACGCCCCGCCGCACAACTACCTCA

ACGCCGTGCCGCCCCCCTCCCGCCCCCAGGGAATCTCTGGAGATTGGTTCACCTTTTGTGGTCCTGACCC

CTTCTGTCCTCACTGTACCTTGAAGTCCTAGAGTCCAATAAAATCGCTGCCTCCCAGCTGTTTGGATCAC

AGAGAGGTTTTTGCACTGCCATAGGGCGCCCCGTGAGGCGCTTCGCCCCCCACCATGTTCCAGCGCCTC

TCCAGCCTCTTCTTCAGCACCCCCTCGCCCCCCGAAGACCCCGACTGCCCCCGCGCCTTCGTGTCGGAGG

AGGATGAAGTGGACGGCTGGCTCATCATTGACCTGCCGGACAGCTACGCGGCTCCACCCAGCCCCGGGGC

CGCCCCTGCCCCCGCGGGCCGCCCTCCGCCCGCGCCCTCCTTGATGGACGAGAGCTGGTTTGTTACCCCT

CCCGCCTGTTTTACGGCAGAGGGGCCTGGACTCGGTCCCGCCCGCCTCCAGAGCAGTCCCCTGGAGGACC

TCCTCATCGAGCACCCCAGCATGTCCGTTTACGTCACCGGCAGCACCATAGTGCTAGAGCCCGGGTCCCC

TTCCCCGCTCCCGGACGCGGCCCTGCCTGACGGCGACCTCAGCGAAGGGGAATTGACGCCCGCCCGCCGC

GAGCCGCGGGCCGCGCGCCACGCCGCTCCTCTCCCAGCGCGGGCGGCGCTGCTGGAGAAGGCGGGCCAGG

TGCGGCGGCTGCAGCGGGCCCGGCAGCGGGCAGAGCGCCACGCGCTGAGCGCCAAGGCGGTGCAGCGGCA

GAACCGAGCCCGCGAGAGCCGTCCGCGCCGGTCCAAGAACCAGAGCAGCTTCATCTACCAGCCGTGCCAG

CGCCAGTTCAACTACTGAGCGTCCACCGGCCGCGCCACGAACCCCTTGCCGATCCCGATCCCTGTCGGGC

TCCTCCGACTCCTCGGGCTGGACACCGAAACCTCCCTTCTTAAAGCGTGTGAGGTTGGGTGATAGCCGTT

CCTTCCCCGACACCCTCAATTTCCCCATCTCTGATCCTCTAATCTGCCTCTGAACCCATTCACCCTTCAC

CCTCACTCCTGGTCCCCATACCCAGCATCTAATCATCCATGCCCCCTACTCCTGGCCCCTCCATCCTTTC

TTCTCTGGTCCCCATCCCTGTCTCTCCCTTTCACCCTTGCCCTCCAGTCCTCTACCTCTGGCCTGCCCCT

ATTTCTGAAAGCTTCTTCCAGTCCCTGATCTGGCTCATTCCCCACCTTCAACTCCCACCTTACATGTCTC

ACACTATCCCATGGTTGGCATTACACTCACTCCTGTTCCCTTATTCTTCATTCCCAGTAATTCCCTACCA

AATGGTGGGGACCCTGAGCCCAGCTCTGACCAGGTAGAGCCTGTGCAGCCTGGGCTGCTGTCATTGCCCT

CCAGTAAGGGCTCAGGGTTTTGCTTTTAGTCTCCCCTTCCTTCTGCCTTGGGGGCGGTACTCTGTGGAGC

TGCTTAGGCCTGGAAAGGTACAGTATGTAGAAGAGGACTGTGAGACGTGAGTTAGAGGGAGAAGATGGAG

GGAATCTAGGGAACGAGGCAGCCTATTGGAGATGCGGACAGGACAGACACGTTGAGAAGCTGCAGGGAGC

AGGGCACCAAGGGAGTGTGCACTGTGCTTGCTCAGAGAGGCCAAACCCTGCTCCCAGGCTGAAGCCTGGA

GTCTGCCCCCACTTCCCTTTTCTATAATCCACCCTTCTGCAGGCCCTGAAATCTGAAGGGCTTAGTTAGT

ACCTTGCCACTCTACCCCCAACACGTCACCCGAGTAGAAGCTGGGCAAGGCCCTACCATCCTGGCCGTCT

GTTCACAAGCCCAAGGTGCTAGAACTAGCTTAGGAGACATGCAGGCCACAGGGCTTCTAGGCAGGGAAAG

GGCACACACCCTAGGTCAGCGTGCAGAGCAGTGATGCTGGAGGACACACCATGGGGTGAAGCCATCCCAA

GGCTGACAGCTCAGTCTTCACCTTGCCTCTGGCCTTGTATTTCACACCCTGCTCAGTATGGCTAGCCAGC

AGTCCTGAGTAGGAGTCCAGGACTCCCACTGTCTCAATCTGCAAATTGTTCCTATCCAGGTTCAGGGCCT

TCAGGGGGCCTCTTTTCATTTTCTCCCACAGGCCTCTCTTTCTTTCTAGGTTGCTGGGGAGAAATGGGTA

CCCTATGATCCCCCTCCCCTTCTCCTCCAGTAAATACCTGGAAGAGGGAACCTGAATCCCTGGGGGAGAC

AGAAGGGGCAGGGGCCACCAGCCTCCCCTTCTTGTGGTGAGACTGAATTTTGGGCTCAGACACCAGCAAC

AGCCTCTTGGGATGCCCTGAGTTGCTTCCCATTTCCTCTTTCTAGCCGTCCTTTTCTAGTGTGTGCTCAC

TCTTCCTAGGAACTTTTAAGACTTCTTGGTACCTATGAACATAGGTCCTCCCCTCACCCCAACTCTAGGT

TTCCAGGCCTCACAGCCAAGCTGAAGCTTGGAGAAAACTCTGCATTCCCATGAGGGCAAAGGCAGCTGCC

CTCCCTGACCCTATAGCCCCAGGCCTCATGGGGGGTATGTGGGGAAGGGATGGGGTATCCCCATGGATGC

TGGGATGAGGACAGAGGAAGACCTGATGGGGTCTCCTATTCCAGGGAATAAGCCAAATTAACACTAAAAA

CGGATCAAAGCTCCCACGCCAGTCCACTAGGGCCCCAGTAGTTGACAGCCTTGCTCCTCTCCCAAGTTCT

CCTTCAGAACCTAGTTGCTTTTATTCTTCCAGCTACCACTTGGGCACTTCACAGCCAGCCTAGGGTCTTC

GGCACCTCCAAGAGCTGAATCTCCCTCCAACCCTTCTTGCCTACTCCTCACTGCCAGCTGGGACCTAGGC

TCAGTCCTGTGTGGTGCCCATGATCCTTCTGGTGGGGGAAGAGTTTAAGTTATAGGGCATTTGGCTCAAA

TTTTAAAAGGCCTTTTGTTTACCTATATTTCTGGAGGCTCCTGTATTCTAGAACCCAATCTCTCACCTGC

TTGGTTGCAAGGCTCATATTTTTTTGTACCTTTCCTATAGATTCTGTAGCATTTGAGTGTGGCAATATTT

TAATTGTGTATAGATTTCTAAGAACCAACACTACTCAGTCTCCTGCTAGTCTGACTCCTGAAGCATCAGC

CCTTGTCATACTGTATTGACTGTGTACGTGCCTTTCACCTTGAGCATGCTTCAGGATTTTTTTTTAAACC

ACAGAACTTGAATACATGAGGGAACCAGAGTTCAAAGTCCTATGCAACCTTAGGAGGGGGTTAGAGAGTC

TGTTTTGATTGATGTTTTCTGAGGCCCTAGAGGAGTTTGTATCAATTTGTGAGTATTAATGTCAGTACTA

CCAGCACTTTGCCAAAACTGTCAGAGGGACCCGTTTCTAGAGTGAGTCCCAGTTACATCAAACAGTGACT

TCCAGTTATTCCCCAGTAAGTCTGAGTGGTTCCTTCAAGCTGGGTGTCTTTCCAGCCTTTGCCAGTCTAG

CCCCAGCAGGGCACCGTGTATGAATGCAGTTTGGTGCTGTTTTAGAGTATGCCTGCTCCCCAGCCCCCTG

CCTGGAACCCTCTGAGCAACTTGCTCTGACCTATAATGTCTTAGGTGCAACACGGACCCCACCAGAGCTC

TTGGATACCCCCCTAGATCCATGTGGCTTTATGTGAGGGGACTGAATGCAGACACACCATAGCCCCCTTC

TACTACTTTCCCTCTCGCCCTGCCACCTAGTTCCACATGGAACCAACAAGTTGAGTGCATCCCTGTTGGG

TGTTTTGTGTTGAGACTGGCTGAAATGAGGAGACTTTGACCATGTGACGTGTCAACAGACTCAAGGAGAC

AACCACCTCAACTGGGTCATGTGGCATGCCTGTGTATGTGTGTAACAGAATTCTGATTGTTAGACTGTAA

TGCTATTCCTCTATGGGAGAAAAAAATTAATATAAAGAAAAACAAATAAAAATATATTTAAAGCACA

>NM_003722.5 Homo sapiens tumor protein p63 (TP63), transcript variant 1, mRNA

CTATGTCTGATAGCATTTGACCCTATTGCTTTTAGCCTCCCGGCTTTATATCTATATATACACAGGTATA

TGTGTATATTTTATATAATTGTTCTCCGTTCGTTGATATCAAAGACAGTTGAAGGAAATGAATTTTGAAA

CTTCACGGTGTGCCACCCTACAGTACTGCCCTGACCCTTACATCCAGCGTTTCGTAGAAACCCCAGCTCA

TTTCTCTTGGAAAGAAAGTTATTACCGATCCACCATGTCCCAGAGCACACAGACAAATGAATTCCTCAGT

CCAGAGGTTTTCCAGCATATCTGGGATTTTCTGGAACAGCCTATATGTTCAGTTCAGCCCATTGACTTGA

ACTTTGTGGATGAACCATCAGAAGATGGTGCGACAAACAAGATTGAGATTAGCATGGACTGTATCCGCAT

GCAGGACTCGGACCTGAGTGACCCCATGTGGCCACAGTACACGAACCTGGGGCTCCTGAACAGCATGGAC

CAGCAGATTCAGAACGGCTCCTCGTCCACCAGTCCCTATAACACAGACCACGCGCAGAACAGCGTCACGG

CGCCCTCGCCCTACGCACAGCCCAGCTCCACCTTCGATGCTCTCTCTCCATCACCCGCCATCCCCTCCAA

CACCGACTACCCAGGCCCGCACAGTTTCGACGTGTCCTTCCAGCAGTCGAGCACCGCCAAGTCGGCCACC

TGGACGTATTCCACTGAACTGAAGAAACTCTACTGCCAAATTGCAAAGACATGCCCCATCCAGATCAAGG

TGATGACCCCACCTCCTCAGGGAGCTGTTATCCGCGCCATGCCTGTCTACAAAAAAGCTGAGCACGTCAC

GGAGGTGGTGAAGCGGTGCCCCAACCATGAGCTGAGCCGTGAATTCAACGAGGGACAGATTGCCCCTCCT

AGTCATTTGATTCGAGTAGAGGGGAACAGCCATGCCCAGTATGTAGAAGATCCCATCACAGGAAGACAGA

GTGTGCTGGTACCTTATGAGCCACCCCAGGTTGGCACTGAATTCACGACAGTCTTGTACAATTTCATGTG

TAACAGCAGTTGTGTTGGAGGGATGAACCGCCGTCCAATTTTAATCATTGTTACTCTGGAAACCAGAGAT

GGGCAAGTCCTGGGCCGACGCTGCTTTGAGGCCCGGATCTGTGCTTGCCCAGGAAGAGACAGGAAGGCGG

ATGAAGATAGCATCAGAAAGCAGCAAGTTTCGGACAGTACAAAGAACGGTGATGGTACGAAGCGCCCGTT

TCGTCAGAACACACATGGTATCCAGATGACATCCATCAAGAAACGAAGATCCCCAGATGATGAACTGTTA

TACTTACCAGTGAGGGGCCGTGAGACTTATGAAATGCTGTTGAAGATCAAAGAGTCCCTGGAACTCATGC

AGTACCTTCCTCAGCACACAATTGAAACGTACAGGCAACAGCAACAGCAGCAGCACCAGCACTTACTTCA

GAAACAGACCTCAATACAGTCTCCATCTTCATATGGTAACAGCTCCCCACCTCTGAACAAAATGAACAGC

ATGAACAAGCTGCCTTCTGTGAGCCAGCTTATCAACCCTCAGCAGCGCAACGCCCTCACTCCTACAACCA

TTCCTGATGGCATGGGAGCCAACATTCCCATGATGGGCACCCACATGCCAATGGCTGGAGACATGAATGG

ACTCAGCCCCACCCAGGCACTCCCTCCCCCACTCTCCATGCCATCCACCTCCCACTGCACACCCCCACCT

CCGTATCCCACAGATTGCAGCATTGTCAGTTTCTTAGCGAGGTTGGGCTGTTCATCATGTCTGGACTATT

TCACGACCCAGGGGCTGACCACCATCTATCAGATTGAGCATTACTCCATGGATGATCTGGCAAGTCTGAA

AATCCCTGAGCAATTTCGACATGCGATCTGGAAGGGCATCCTGGACCACCGGCAGCTCCACGAATTCTCC

TCCCCTTCTCATCTCCTGCGGACCCCAAGCAGTGCCTCTACAGTCAGTGTGGGCTCCAGTGAGACCCGGG

GTGAGCGTGTTATTGATGCTGTGCGATTCACCCTCCGCCAGACCATCTCTTTCCCACCCCGAGATGAGTG

GAATGACTTCAACTTTGACATGGATGCTCGCCGCAATAAGCAACAGCGCATCAAAGAGGAGGGGGAGTGA

GCCTCACCATGTGAGCTCTTCCTATCCCTCTCCTAACTGCCAGCCCCCTAAAAGCACTCCTGCTTAATCT

TCAAAGCCTTCTCCCTAGCTCCTCCCCTTCCTCTTGTCTGATTTCTTAGGGGAAGGAGAAGTAAGAGGCT

ACCTCTTACCTAACATCTGACCTGGCATCTAATTCTGATTCTGGCTTTAAGCCTTCAAAACTATAGCTTG

CAGAACTGTAGCTGCCATGGCTAGGTAGAAGTGAGCAAAAAAGAGTTGGGTGTCTCCTTAAGCTGCAGAG

ATTTCTCATTGACTTTTATAAAGCATGTTCACCCTTATAGTCTAAGACTATATATATAAATGTATAAATA

TACAGTATAGATTTTTGGGTGGGGGGCATTGAGTATTGTTTAAAATGTAATTTAAATGAAAGAAAATTGA

GTTGCACTTATTGACCATTTTTTAATTTACTTGTTTTGGATGGCTTGTCTATACTCCTTCCCTTAAGGGG

TATCATGTATGGTGATAGGTATCTAGAGCTTAATGCTACATGTGAGTGACGATGATGTACAGATTCTTTC

AGTTCTTTGGATTCTAAATACATGCCACATCAAACCTTTGAGTAGATCCATTTCCATTGCTTATTATGTA

GGTAAGACTGTAGATATGTATTCTTTTCTCAGTGTTGGTATATTTTATATTACTGACATTTCTTCTAGTG

ATGATGGTTCACGTTGGGGTGATTTAATCCAGTTATAAGAAGAAGTTCATGTCCAAACGTCCTCTTTAGT

TTTTGGTTGGGAATGAGGAAAATTCTTAAAAGGCCCATAGCAGCCAGTTCAAAAACACCCGACGTCATGT

ATTTGAGCATATCAGTAACCCCCTTAAATTTAATACCAGATACCTTATCTTACAATATTGATTGGGAAAA

CATTTGCTGCCATTACAGAGGTATTAAAACTAAATTTCACTACTAGATTGACTAACTCAAATACACATTT

GCTACTGTTGTAAGAATTCTGATTGATTTGATTGGGATGAATGCCATCTATCTAGTTCTAACAGTGAAGT

TTTACTGTCTATTAATATTCAGGGTAAATAGGAATCATTCAGAAATGTTGAGTCTGTACTAAACAGTAAG

ATATCTCAATGAACCATAAATTCAACTTTGTAAAAATCTTTTGAAGCATAGATAATATTGTTTGGTAAAT

GTTTCTTTTGTTTGGTAAATGTTTCTTTTAAAGACCCTCCTATTCTATAAAACTCTGCATGTAGAGGCTT

GTTTACCTTTCTCTCTCTAAGGTTTACAATAGGAGTGGTGATTTGAAAAATATAAAATTATGAGATTGGT

TTTCCTGTGGCATAAATTGCATCACTGTATCATTTTCTTTTTTAACCGGTAAGAGTTTCAGTTTGTTGGA

AAGTAACTGTGAGAACCCAGTTTCCCGTCCATCTCCCTTAGGGACTACCCATAGACATGAAAGGTCCCCA

CAGAGCAAGAGATAAGTCTTTCATGGCTGCTGTTGCTTAAACCACTTAAACGAAGAGTTCCCTTGAAACT

TTGGGAAAACATGTTAATGACAATATTCCAGATCTTTCAGAAATATAACACATTTTTTTGCATGCATGCA

AATGAGCTCTGAAATCTTCCCATGCATTCTGGTCAAGGGCTGTCATTGCACATAAGCTTCCATTTTAATT

TTAAAGTGCAAAAGGGCCAGCGTGGCTCTAAAAGGTAATGTGTGGATTGCCTCTGAAAAGTGTGTATATA

TTTTGTGTGAAATTGCATACTTTGTATTTTGATTATTTTTTTTTTCTTCTTGGGATAGTGGGATTTCCAG

AACCACACTTGAAACCTTTTTTTATCGTTTTTGTATTTTCATGAAAATACCATTTAGTAAGAATACCACA

TCAAATAAGAAATAATGCTACAATTTTAAGAGGGGAGGGAAGGGAAAGTTTTTTTTATTATTTTTTTAA

AATTTTGTATGTTAAAGAGAATGAGTCCTTGATTTCAAAGTTTTGTTGTACTTAAATGGTAATAAGCACT

GTAAACTTCTGCAACAAGCATGCAGCTTTGCAAACCCATTAAGGGGAAGAATGAAAGCTGTTCCTTGGTC

CTAGTAAGAAGACAAACTGCTTCCCTTACTTTGCTGAGGGTTTGAATAAACCTAGGACTTCCGAGCTATG

TCAGTACTATTCAGGTAACACTAGGGCCTTGGAAATTCCTGTACTGTGTCTCATGGATTTGGCACTAGCC

AAAGCGAGGCACCCTTACTGGCTTACCTCCTCATGGCAGCCTACTCTCCTTGAGTGTATGAGTAGCCAGG

GTAAGGGGTAAAAGGATAGTAAGCATAGAAACCACTAGAAAGTGGGCTTAATGGAGTTCTTGTGGCCTCA

GCTCAATGCAGTTAGCTGAAGAATTGAAAAGTTTTTGTTTGGAGACGTTTATAAACAGAAATGGAAAGCA

GAGTTTTCATTAAATCCTTTTACCTTTTTTTTTTCTTGGTAATCCCCTAAAATAACAGTATGTGGGATAT

TGAATGTTAAAGGGATATTTTTTTCTATTATTTTTATAATTGTACAAAATTAAGCAAATGTTAAAAGTTT

TATATGCTTTATTAATGTTTTCAAAAGGTATTATACATGTGATACATTTTTTAAGCTTCAGTTGCTTGTC

TTCTGGTACTTTCTGTTATGGGCTTTTGGGGAGCCAGAAGCCAATCTACAATCTCTTTTTGTTTGCCAGG

ACATGCAATAAAATTTAAAAAATAAATAAAAACTAATTAAGAAA

>NM_005427.4 Homo sapiens tumor protein p73 (TP73), transcript variant 1, mRNA

GCCCTGCCTCCCCGCCCGCGCACCCGCCCGGAGGCTCGCGCGCCCGCGAAGGGGACGCAGCGAAACCGGG

GCCCGCGCCAGGCCAGCCGGGACGGACGCCGATGCCCGGGGCTGCGACGGCTGCAGAGCGAGCTGCCCTC

GGAGGCCGGCGTGGGGAAGATGGCCCAGTCCACCGCCACCTCCCCTGATGGGGGCACCACGTTTGAGCAC

CTCTGGAGCTCTCTGGAACCAGACAGCACCTACTTCGACCTTCCCCAGTCAAGCCGGGGGAATAATGAGG

TGGTGGGCGGAACGGATTCCAGCATGGACGTCTTCCACCTGGAGGGCATGACTACATCTGTCATGGCCCA

GTTCAATCTGCTGAGCAGCACCATGGACCAGATGAGCAGCCGCGCGGCCTCGGCCAGCCCCTACACCCCA

GAGCACGCCGCCAGCGTGCCCACCCACTCGCCCTACGCACAACCCAGCTCCACCTTCGACACCATGTCGC

CGGCGCCTGTCATCCCCTCCAACACCGACTACCCCGGACCCCACCACTTTGAGGTCACTTTCCAGCAGTC

CAGCACGGCCAAGTCAGCCACCTGGACGTACTCCCCGCTCTTGAAGAAACTCTACTGCCAGATCGCCAAG

ACATGCCCCATCCAGATCAAGGTGTCCACCCCGCCACCCCCAGGCACCGCCATCCGGGCCATGCCTGTTT

ACAAGAAAGCGGAGCACGTGACCGACGTCGTGAAACGCTGCCCCAACCACGAGCTCGGGAGGGACTTCAA

CGAAGGACAGTCTGCTCCAGCCAGCCACCTCATCCGCGTGGAAGGCAATAATCTCTCGCAGTATGTGGAT

GACCCTGTCACCGGCAGGCAGAGCGTCGTGGTGCCCTATGAGCCACCACAGGTGGGGACGGAATTCACCA

CCATCCTGTACAACTTCATGTGTAACAGCAGCTGTGTAGGGGGCATGAACCGGCGGCCCATCCTCATCAT

CATCACCCTGGAGATGCGGGATGGGCAGGTGCTGGGCCGCCGGTCCTTTGAGGGCCGCATCTGCGCCTGT

CCTGGCCGCGACCGAAAAGCTGATGAGGACCACTACCGGGAGCAGCAGGCCCTGAACGAGAGCTCCGCCA

AGAACGGGGCCGCCAGCAAGCGTGCCTTCAAGCAGAGCCCCCCTGCCGTCCCCGCCCTTGGTGCCGGTGT

GAAGAAGCGGCGGCATGGAGACGAGGACACGTACTACCTTCAGGTGCGAGGCCGGGAGAACTTTGAGATC

CTGATGAAGCTGAAAGAGAGCCTGGAGCTGATGGAGTTGGTGCCGCAGCCACTGGTGGACTCCTATCGGC

AGCAGCAGCAGCTCCTACAGAGGCCGAGTCACCTACAGCCCCCGTCCTACGGGCCGGTCCTCTCGCCCAT

GAACAAGGTGCACGGGGGCATGAACAAGCTGCCCTCCGTCAACCAGCTGGTGGGCCAGCCTCCCCCGCAC

AGTTCGGCAGCTACACCCAACCTGGGGCCCGTGGGCCCCGGGATGCTCAACAACCATGGCCACGCAGTGC

CAGCCAACGGCGAGATGAGCAGCAGCCACAGCGCCCAGTCCATGGTCTCGGGGTCCCACTGCACTCCGCC

ACCCCCCTACCACGCCGACCCCAGCCTCGTCAGTTTTTAACAGGATTGGGGTGTCCAAACTGCATCGAG

TATTTCACCTCCCAAGGGTTACAGAGCATTTACCACCTGCAGAACCTGACCATTGAGGACCTGGGGGCCC

TGAAGATCCCCGAGCAGTACCGCATGACCATCTGGCGGGGCCTGCAGGACCTGAAGCAGGGCCACGACTA

CAGCACCGCGCAGCAGCTGCTCCGCTCTAGCAACGCGGCCACCATCTCCATCGGCGGCTCAGGGGAACTG

CAGCGCCAGCGGGTCATGGAGGCCGTGCACTTCCGCGTGCGCCACACCATCACCATCCCCAACCGCGGCG

GCCCAGGCGGCGGCCCTGACGAGTGGGCGGACTTCGGCTTCGACCTGCCCGACTGCAAGGCCCGCAAGCA

GCCCATCAAGGAGGAGTTCACGGAGGCCGAGATCCACTGAGGGCCTCGCCTGGCTGCAGCCTGCGCCACC

GCCCAGAGACCCAAGCTGCCTCCCCTCTCCTTCCTGTGTGTCCAAAACTGCCTCAGGAGGCAGGACCTTC

GGGCTGTGCCCGGGGAAAGGCAAGGTCCGGCCCATCCCCAGGCACCTCACAGGCCCCAGGAAAGGCCCAG

CCACCGAAGCCGCCTGTGGACAGCCTGAGTCACCTGCAGAACCTTCTGGAGCTGCCCTAGTGCTGGGCTT

GTGGGGCGGGGGCTGGCCCACTCTCAGCCCTGCCACTGCCCCGGCGTGCTCCATGGCAGGCGTGGGTGGG

GACCGCAGCGTCGGCTCCGACTTCCAGGCTTCATCCTAGAGACTGTCATCTCCCAACCAGGCGAGGTCCT

TCCAAAGGAAAGGATCCTCTTTGCTGATGGACTGCCAAAAAGTATTTTGCGACATCTTTTGGTTCTGGAT

AGTAGTGAGCAGCCAAGTGACTGTGTCTGAAACACCAGTGTATTTTCAGGGAATGTCCCTAACTGCGTCT

TGCCCGCGCCGGGGGCTGGGGACTCTCTCTGCTGGACTTGGGACTGGCCTCTGCCCCCAGCACGCTGTAT

TCTGCAGGACCGCCTCCTTCCTGCCCCTAACAACAACCACAGTGTTGCTGAAATTGGAGAAAACTGGGGA

GGGCGCAACCCCCCCCAGGCGCGGGGAAGCATGTGGTACCGCCTCAGCCAGTGCCCCTCAGCCTGGCCAC

AGTCGCCTCTCCTCGGGGACCCCTCAGCAGAAAGGGACAGCCTGTCCTTAGAGGACTGGAAATTGTCAAT

ATTTGATAAAATGATACCCTTTTCTACATGGTGGGTCAGCTTTTTTTTTTTTTTTTTAACTTTCTTTCT

CAGCATTCTCTTTGGAGTTCAACCTAGCGCCCATGAGCCAGGCTGAGGAAGCTGAGTGAGAAGCCAGGTG

GGCGGGACTTGTTCCCAGGAAGGCCGGGTGGGGAGGAAGCCTAGAGGGAACCCCAGGAAGGGCAAATCCA

GGCAAATCTGCAGGAATGCTCTGCCATGGGAGCAGCTCCTCCCTTGCCACGGCCACCTTCTCTAGCACTG

CAAGGTCCACAGGGCATTGCTTTCCTTTCTAGGCGGTGGCAGTCAGGGAACAGACTGAGGTAGGTGTAGG

GGGGTCTAGGCCTTCGTGGAGCACCCCAGGGAGTTAGTAGGCCCCGGGGAGACAGAGTCTGCACAGGCCC

TTTCTGGGGCCACCTCCATCCACGAGGAGCAGCCTGAGCCTTGGTGGCCGAACCTTGACCGTCCCGGAGC

ACAGCTTCAGGGCAGGGAACCGGAGCCCCTGGGGGGCCTCACGGGTGTGACGAGGCCCTTCATTGCAGGC

AGGTGGGCCAATGGGAGCCCTCACCCACGCAAGCCGAGACACCACCCAGAGTGCAGGCTGCCTGGCCCCT

TCTGGCACGGCCAGCTCCACACCCCCTGCCTAGGGTATGTGTGGTCCTAAGGGCTAGGAGCTTCCCCTAC

TAACATCTCCCAGAAAAAGCAGTTAAGCCCCTCAGGGCACAGCAAGGTTAGACACAGCCCCCATCCCCAG

ATCAGGACTCCATCTTGCTAAGTGGCATCACCGTCACCAGCCTCCCCTTATTTAAAAGCAGCGACTGGTG

TTGCCGCAGGTACCTGGTCTACGAAGACGCAGGCATCCCTCTCCCACCGTCCACCTCCCCGGGGGCCGCT

GACAGCACAGTCGCCTGGGTGCACGCTTGTGGGGGCAGCAGGAACGGGGCTGTCGGCTCTCAGGGGATCT

GGCTGCAGCCAGGGCGAGGGCCTGGCCCTTCCTTCCAGCTCCTTCCGGCTCCTTCCAGCTGAAGGGCAGG

AAGCTCTGGCCGCTTAGCTTCTAGGGTTCCATCTCCCTAGAAAGGTGCCCACGCCCAGGGCATCAGTCAG

TAGCGGCAGCAGCAGCAGACTCGGGGCTTTCCCAGGGTGGCGCAGCCACCCCAGCTGCATGTCACCTCAG

CTCTCCATCTTATTGCCATTTTGTAGATGAGGAAGCTGAGACCAGAAAGGCTAAGACCCATGCCCCAGGC

ACCACACCCATCTCTTGGGGGCTGGGCACCTGCTACCCGAGGCCACCTCCTGAAGCCCCCACTCTTCCCC

CATGTTCCACTTCAGGAGCCGCGGGGGCCCATCCTGACACCCGGGGTTCCTCAGCCCAGCGCAGATGTGC

TTCAGTTCCAGAGGGCTTGTTGATTTGTTTCTTAGGTACGTTACCTGTCCACCCTGAGTCCAGTGAGGCT

GTCCCAAGAGCCCCTGTAGTGTGCTCCTGGGAAGGGCTGGGGGGGCTGGGGGGGCTGGGAGAGGCCCAGG

GGCAGCTGTCACTGGAACCCCAGCCAGATGTCCAAGGAAGCCGGCCAGAACACGGAGCAGCCAGATGGCC

CCAGCTGCACCTGTCTAGGGAGCCCATGCAGCCTCCTTGCACTGGAGAAGCAGCTGTGAAAGTAGACAGA

GTTGAGACTTCGCCGTGGTCAGGAGAAAATGCAAATTCCCAGGAACAAGAATCCTTTAAGTGATATGTTT

TTATAAAACTAAACAAATCAACAAATAAATCTTGAAGGCGGATGGTTTTCCCAGCAGTGCAGGGGTTGGA

GGGAGGCTGCTGGCACTCCTGGGGCCAAGGGGGACAGGCAGTGGTCCTGAGTCTGCTCAGAGAGGCAAGG

CAGAAGGAGCTCGCCAGGCAGGTCAGCTCACATCTGTCCAAGTCGCTCTGGTCAGAAACAGCGACTCTCC

CCCATTCCCCCAGCGTTCCCACCAGGCCTGGGCTGCTGGGAAGCCCTTGCTGTACCCAGGAGCCCGACCC

GCAGTATCCTGGCACAGAGCCACTTGTCACTCAGAACAGTCAGTGTCTCCAACGCACAAACATCCACTCC

TCTGTTACCAGTTAAAGCACTTTAATGCTTTAAGGTGAAAACGAAATCCCATCCGTGTTTTTCGTGTAAG

ATCGTGCTTCTCCGAGCAGTATTAATGGACGCCCTCCAATGACATAACAACTGTTTTTGGTAATGTAATC

TTGGGAAAATGTGTTATTTTTTTAGCTGTGTTTCAGTGGGGATTTTTGTTTTTGTAACATAATAAAGTGT

ATGTTCCAATGA

>NM_000368.5 Homo sapiens TSC complex subunit 1 (TSC1), transcript variant 1, mRNA

GTACGTCCAAGATGGCGGCGCCCTGTAGGCTGGAGGGACTGTGAGGTAAACAGCTGAGGGGGAGGAGACG

GTGGTGACCATGAAAGACACCAGGTTGACAGCACTGGAAACTGAAGTACCAGTTGTCGCTAGAACAGTTT

GGTAGTGGCCCCAATGAAGAACCTTCAGAACCTGTAGCACACGTCCTGGAGCCAGCACAGCGCCTTCGAG

CGAGAGAATGGCCCAACAAGCAAATGTCGGGGAGCTTCTTGCCATGCTGGACTCCCCCATGCTGGGTGTG

CGGGACGACGTGACAGCTGTCTTTAAAGAGAACCTCAATTCTGACCGTGGCCCTATGCTTGTAAACACCT

TGGTGGATTATTACCTGGAAACCAGCTCTCAGCCGGCATTGCACATCCTGACCACCTTGCAAGAGCCACA

TGACAAGCACCTCTTGGACAGGATTAACGAATATGTGGGCAAAGCCGCCACTCGTTTATCCATCCTCTCG

TTACTGGGTCATGTCATAAGACTGCAGCCATCTTGGAAGCATAAGCTCTCTCAAGCACCTCTTTTGCCTT

CTTTACTAAAATGTCTCAAGATGGACACTGACGTCGTTGTCCTCACAACAGGCGTCTTGGTGTTGATAAC

CATGCTACCAATGATTCCACAGTCTGGGAAACAGCATCTTCTTGATTTCTTTGACATTTTTGGCCGTCTG

TCATCATGGTGCCTGAAGAAACCAGGCCACGTGGCGGAAGTCTATCTCGTCCATCTCCATGCCAGTGTGT

ACGCACTCTTTCATCGCCTTTATGGAATGTACCCTTGCAACTTCGTCTCCTTTTTGCGTTCTCATTACAG

TATGAAAGAAAACCTGGAGACTTTTGAAGAAGTGGTCAAGCCAATGATGGAGCATGTGCGAATTCATCCG

GAATTAGTGACTGGATCCAAGGACCATGAACTGGACCCTCGAAGGTGGAAGAGATTAGAAACTCATGATG

TTGTGATCGAGTGTGCCAAAATCTCTCTGGATCCCACAGAAGCCTCATATGAAGATGGCTATTCTGTGTC

TCACCAAATCTCAGCCCGCTTTCCTCATCGTTCAGCCGATGTCACCACCAGCCCTTATGCTGACACACAG

AATAGCTATGGGTGTGCTACTTCTACCCCTTACTCCACGTCTCGGCTGATGTTGTTAAATATGCCAGGGC

AGCTACCTCAGACTCTGAGTTCCCCATCGACACGGCTGATAACTGAACCACCACAAGCTACTCTTTGGAG

CCCATCTATGGTTTGTGGTATGACCACTCCTCCAACTTCTCCTGGAAATGTCCCACCTGATCTGTCACAC

CCTTACAGTAAAGTCTTTGGTACAACTGCAGGTGGAAAAGGAACTCCTCTGGGAACCCCAGCAACCTCTC

CTCCTCCAGCCCCACTCTGTCATTCGGATGACTACGTGCACATTTCACTCCCCCAGGCCACAGTCACACC

CCCCAGGAAGGAAGAGAGAATGGATTCTGCAAGACCATGTCTACACAGACAACACCATCTTCTGAATGAC

AGAGGATCAGAAGAGCCACCTGGCAGCAAAGGTTCTGTCACTCTAAGTGATCTTCCAGGGTTTTTAGGTG

ATCTGGCCTCTGAAGAAGATAGTATTGAAAAAGATAAAGAAGAAGCTGCAATATCTAGAGAACTTTCTGA

GATCACCACAGCAGAGGCAGAGCCTGTGGTTCCTCGAGGAGGCTTTGACTCTCCCTTTTACCGAGACAGT

CTCCCAGGTTCTCAGCGGAAGACCCACTCGGCAGCCTCCAGTTCTCAGGGCGCCAGCGTGAACCCTGAGC

CTTTACACTCCTCCCTGGACAAGCTTGGGCCTGACACACCAAAGCAAGCCTTTACTCCCATAGACCTGCC

CTGCGGCAGTGCTGATGAAAGCCCTGCGGGAGACAGGGAATGCCAGACTTCTTTGGAGACCAGTATCTTC

ACTCCCAGTCCTTGTAAAATTCCACCTCCGACGAGAGTGGGCTTTGGAAGCGGGCAGCCTCCCCCGTATG

ATCATCTTTTTGAGGTGGCATTGCCAAAGACAGCCCATCATTTTGTCATCAGGAAGACTGAGGAGCTGTT

AAAGAAAGCAAAAGGAAACACAGAGGAAGATGGTGTGCCCTCTACCTCCCCAATGGAAGTGCTGGACAGA

CTGATACAGCAGGGAGCAGACGCGCACAGCAAGGAGCTGAACAAGTTGCCTTTACCCAGCAAGTCTGTCG

ACTGGACCCACTTTGGAGGCTCTCCTCCTTCAGATGAGATCCGCACCCTCCGAGACCAGTTGCTTTTACT

GCACAACCAGTTACTCTATGAGCGTTTTAAGAGGCAGCAGCATGCCCTCCGGAACAGGCGGCTCCTCCGC

AAGGTGATCAAAGCAGCAGCTCTGGAGGAACATAATGCTGCCATGAAAGATCAGTTGAAGTTACAAGAGA

AGGACATCCAGATGTGGAAGGTTAGTCTGCAGAAAGAACAAGCTAGATACAATCAGCTCCAGGAGCAGCG

TGACACTATGGTAACCAAGCTCCACAGCCAGATCAGACAGCTGCAGCATGACCGAGAGGAATTCTACAAC

CAGAGCCAGGAATTACAGACGAAGCTGGAGGACTGCAGGAACATGATTGCGGAGCTGCGGATAGAACTGA

AGAAGGCCAACAACAAGGTGTGTCACACTGAGCTGCTGCTCAGTCAGGTTTCCCAAAAGCTCTCAAACAG

TGAGTCGGTCCAGCAGCAGATGGAGTTCTTGAACAGGCAGCTGTTGGTTCTTGGGGAGGTCAACGAGCTC

TATTTGGAACAACTGCAGAACAAGCACTCAGATACCACAAAGGAAGTAGAAATGATGAAAGCCGCCTATC

GGAAAGAGCTAGAAAAAAACAGAAGCCATGTTCTCCAGCAGACTCAGAGGCTTGATACCTCCCAAAAACG

GATTTTGGAACTGGAATCTCACCTGGCCAAGAAAGACCACCTTCTTTTGGAACAGAAGAAATATCTAGAG

GATGTCAAACTCCAGGCAAGAGGACAGCTGCAGGCCGCAGAGAGCAGGTATGAGGCTCAGAAAAGGATAA

CCCAGGTGTTTGAATTGGAGATCTTAGATTTATATGGCAGGTTGGAGAAAGATGGCCTCCTGAAAAAACT

TGAAGAAGAAAAAGCAGAAGCAGCTGAAGCAGCAGAAGAAAGGCTTGACTGTTGTAATGACGGGTGCTCA

GATTCCATGGTAGGGCACAATGAAGAGGCATCTGGCCACAACGGTGAGACCAAGACCCCCAGGCCCAGCA

GCGCCCGGGGCAGTAGTGGAAGCAGAGGTGGTGGAGGCAGCAGCAGCAGCAGCAGCGAGCTTTCTACCCC

AGAGAAACCCCCACACCAGAGGGCAGGCCCATTCAGCAGTCGGTGGGAGACGACTATGGGAGAAGCGTCT

GCCAGCATCCCCACCACTGTGGGCTCACTTCCCAGTTCAAAAAGCTTCCTGGGTATGAAGGCTCGAGAGT

TATTTCGTAATAAGAGCGAGAGCCAGTGTGATGAGGACGGCATGACCAGTAGCCTTTCTGAGAGCCTAAA

GACAGAACTGGGCAAAGACTTGGGTGTGGAAGCCAAGATTCCCCTGAACCTAGATGGCCCTCACCCGTCT

CCCCCGACCCCGGACAGTGTTGGACAGCTACATATCATGGACTACAATGAGACTCATCATGAACACAGCT

AAGGAATGATGGTCAATCAGTGTTAACTTGCATATTGTTGGCACAGAACAGGAGGTGTGAATGCACGTTT

CAAAGCTTTCCTGTTTCCAGGGTCTGAGTGCAAGTTCATGTGTGGAAATGGGACGGAGGTCCTTTGGACA

GCTGACTGAATGCAGAACGGTTTTTGGATCTGGCATTGAAATGCCTCTTGACCTTCCCCTCCACCCGCCC

TAACCCCCTCTCATTTACCTCGCAGTGTGTTCTAATCCAAGGGCCAGTTGGTGTTCCTCAGTAGCTTTAC

TTTCTTCCTTTCCCCCCCAAATGGTTGCGTCCTTTGAACCTGTGCAATATGAGGCCAAATTTAATCTTTG

AGTCTAACACACCACTTTCTGCTTTCCCGAAGTTCAGATAACTGGGTTGGCTCTCAATTAGACCAGGTAG

TTTGTTGCATTGCAGGTAAGTCTGGTTTTGTCCCTTCCAGGAGGACATAGCCTGCAAAGCTGGTTGTCTT

TACATGAAAGCGTTTACATGAGACTTTCCGACTGCTTTTTTGATTCTGAAGTTCAGCATCTAAAGCAGCA

GGTCTAGAAGAACAACGGTTTATTCATACTTGCATTCTTTTGGCAGTTCTGATAAGCTTCCTAGAAAGTT

CTGTGTAAACAGAAGCCTGTTTCAGAAATCTGGAGCTGGCACTGTGGAGACCACACACCCTTTGGGAAAG

CTCTTGTCTCTTCTTCCCCCACTACCTCTTATTTATTTGGTGTTTGCTTGAATGCTGGTACTATTGTGAC

CACAGGCTGGTGTGTAGGTGGTAAAACCTGTTCTCCATAGGAGGGAAGGAGCAGTCACTGGGAGAGGTTA

CCCGAGAAGCACTTGAGCATGAGGAACTGCACCTTTAGGCCATCTCAGCTTGCTGGGCCTTTTGTTAAAC

CCTTCTGTCTACTGGCCTCCCTTTGTGTGCATACGCCTCTTGTTCATGTCAGCTTATATGTGACACTGCA

GCAGAAAGGCTCTGAAGGTCCAAAGAGTTTCTGCAAAGTGTATGTGACCATCATTTCCCAGGCCATTAGG

GTTGCCTCACTGTAGCAGGTTCTAGGCTACCAGAAGAGGGGCAGCTTTTTCATACCAATTCCAACTTTCA

GGGGCTGACTCTCCAGGGAGCTGATGTCATCACACTCTCCATGTTAGTAATGGCAGAGCAGTCTAAACAG

AGTCCGGGAGAATGCTGGCAAAGGCTGGCTGTGTATACCCACTAGGCTGCCCCACGTGCTCCCGAGAGAT

GACACTAGTCAGAAAATTGGCAGTGGCAGAGAATCCAAACTCAACAAGTGCTCCTGAAAGAAACGCTAGA

AGCCTAAGAACTGTGGTCTGGTGTTCCAGCTGAGGCAGGGGGATTTGGTAGGAAGGAGCCAGTGAACTTG

GCTTTCCTGTTTCTATCTTTCATTAAAAAGAATAGAAGGATTCAGTCATAAAGAGGTAAAAAACTGTCAC

GGTACGAAATCTTAGTGCCCACGGAGGCCTCGAGCAGAGAGAATGAAAGTCTTTTTTTTTTTTTTTTTT

TTTAGCATGGCAATAAATATTCTAGCATCCCTAACTAAAGGGGACTAGACAGTTAGAGACTCTGTCACCC

TAGCTATACCAGCAGAAAACCTGTTCAGGCAGGCTTTCTGGGTGTGACTGATTCCCAGCCTGTGGCAGGG

CGTGGTCCCAACTACTCAGCCTAGCACAGGCTGGCAGTTGGTACTGAATTGTCAGATGTGGAGTATTAGT

GACACCACACATTTAATTCAGCTTTGTCCAAAGGAAAGCTTAAAACCCAATACAGTCTAGTTTCCTGGTT

CCGTTTTAGAAAAGGAAAACGTGAACAAACTTAGAAAGGGAAGGAAATCCCATCAGTGAATCCTGAAACT

GGTTTTAAGTGCTTTCCTTCTCCTCATGCCCAAGAGATCTGTGCCATAGAACAAGATACCAGGCACTTAA

AGCCTTTTCCTGAATTGGAAAGGAAAAGAGGCCCAAGTGCAAAAGAAAAAACATTTTAGAAACGGACAGC

TTATAAAAATAAAGGGAAGAAAGGAGGCAGCATGGAGAGAGGCCTGTGCTAGAAGCTCCATGGACGTGTC

TGCACAGGGTCCTCAGCTCATCCATGCGGCCTGGGTGTCCTTTTACTCAGCTTTATAACAAATGTGGCTC

CAAGCTCAGGTGCCTTTGAGTTCTAGGAGGCTGTGGGTTTTATTCAACTACGGTTGGGAGAATGAGACCT

GGAGTCATGTTGAAGGTGCCCAACCTAAAAATGTAGGCTTTCATGTTGCAAAGAACTCCAGAGTCAGTAG

TTAGGTTTGGTTTGGTTTTGGACATGATAAACCTGCCAAGAGTCAACAGGTCACTTGATCATGCTGCAGT

GGGTAGTTCTAAGGATGGAAAGGTGACAGTATTACTCTCGAGAGGCAATTCAGTCCTGGGCAAAGGTATT

AGTACAATAAGCGTTAAGGGCAGAGTCTACCTTGAAACCAATTAAGCAGCTTGGTATTCATAAATATTGG

GATTGGATGGCCTCCATCCAGAAATCACTATGGGTGAGCATACCTGTCTCAGCTGTTTGGCCAATGTGCA

TAACCTACTCGGATCCCCACCTGACACTAACCAGAGTCAGCACAGGCCCCGAGGAGCCCGAAGTCTGCTG

CTGTGCAGCATGGAATTCCTTTAAAAAGGTGCACTACAGTTTTAGCGGGGAGGGGGATAGGAAGACGCAG

AGCAAATGAGCTCCGGAGTCCCTGCAGGTGAATAAACACACAGATCTGCATCTGATAGAACTTTGATGGA

TTTTCAAAAAGCCGTTGACAAGGCTCTGCTATACAGTCTATAAAAATTGTTATTATGGGATTGGAAGAAA

CACGTGGTCATGAATAGAAAAAAAACAAACCCAAAGGTAGGAAGGTCAAGGTCATTTCTTAGATGGAGAA

GTTGTGAAAGATGTCCTTGGAGATGAGTTTTAGGACCAGCATTACTAAGGCAGGTGGGCAGACAGTGACC

TCTCTAGGTGTGTCCACAGAGTTTTTCAGGAGAGAAAACTGCCTGACCTTTGGGACTAAGCTGCGGAATC

TTCTTACTAAGCTTGAAGAGTGGAGAGGCGAGAGGTGAGCTACTTTGTGAGCCAAAGCTTATGTGACATG

GTTGGGGAAACAGTCCAAACTGTTCTGAGAAGGTGAACTGTTACGACCCAGGACAATTAGAAAAATTCAC

CCACCATGCCGCACATTACTGGGTAAAAGCAGGGCAGCAGGGAACAAAACTCCAGACTCTTGGGCCGTCC

CCATTTGCAACAGCACACATAGTTTCTGGTATATTTGTTGGGAAAGATAAAACTCTAGCAGTTGTTGAGG

GGAGGATGTATAAAATGGTCATGGGGATGAAAGGATCTCTGAGACCACAGAGGCTCAGACTCACTGTTAA

GAATAGAAAACTGGGTATGCGTTTCATGTAGCCAGCAGAACTGAAGTGTGCTGTGACAAGCCAATGTGAA

TTTCTACCAAATAGTAGAGCATACCACTTGAAGAAGGAAAGAACCGAAGAGCAAACAAAAGTTCTGCGTA

ATGAGACTCACCTTTTCTCGCTGAAAGCACTAAGAGGTGGGAGGAGGCCTGCACAGGCTGGAGGAGGGTT

TGGGCAGAGCGAAGACCCGGCCAGGACCTTGGTGAGATGGGGTGCCGCCCACCTCCTGCGGATACTCTTG

GAGAGTTGTTCCCCCAGGGGGCTCTGCCCCACCTGGAGAAGGAAGCTGCCTGGTGTGGAGTGACTCAAAT

CAGTATACCTATCTGCTGCACCTTCACTCTCCAGGGTACATGCTTTAAAACCGACCCGCAACAAGTATTG

GAAAAATGTATCCAGTCTGAAGATGTTTGTGTATCTGTTTACATCCAGAGTTCTGTGACACATGCCCCCC

AGATTGCTGCAAAGATCCCAAGGCATTGATTGCACTTGATTAAGCTTTTGTCTGTAGGTGAAAGAACAAG

TTTAGGTCGAGGACTGGCCCCTAGGCTGCTGCTGTGACCCTTGTCCCATGTGGCTTGTTTGCCTGTCCGG

GACTCTTCGATGTGCCCAGGGGAGCGTGTTCCTGTCTCTTCCATGCCGTCCTGCAGTCCTTATCTGCTCG

CCTGAGGGAAGAGTAGCTGTAGCTACAAGGGAAGCCTGCCTGGAAGAGCCGAGCACCTGTGCCCATGGCT

TCTGGTCATGAAACGAGTTAATGATGGCAGAGGAGCTTCCTCCCCACTTCGCAGCGCCACATTATCCATC

CTCTGAGATAAGTAGGCTGGTTTAACCATTGGAATGGACCTTTCAGTGGAAACCCTGAGAGTCTGAGAAC

CCCCAGACCAACCCTTCCCTCCCTTTCCCCACCTCTTACAGTGTTTGGACAGGAGGGTATGGTGCTGCTC

TGTGTAGCAAGTACTTTGGCTTATGAAAGAGGCAGCCACGCATTTTGCACTAGGAAGAATCAGTAATCAC

TTTTCAGAAGACTTCTATGGACCACAAATATATTACGGAGGAACAGATTTTGCTAAGACATAATCTAGTT

TTATAACTCAATCATGAATGAACCATGTGTGGCAAACTTGCAGTTTAAAGGGGTCCCATCAGTGAAAGAA

ACTGATTTTTTTTAACGGACTGCTTTTAGTTAAATTGAAGAAAGTCAGCTCTTGTCAAAAGGTCTAAACT

TTCCCGCCTCAATCCTAAAAGCATGTCAACAATCCACATCAGATGCCATAAATATGAACTGCAGGATAAA

ATGGTACAATCTTAGTGAATGGGAATTGGAATCAAAAGAGTTTGCTGTCCTTCTTAGAATGTTCTAAAAT

GTCAAGGCAGTTGCTTGTGTTTAACTGTGAACAAATAAAAATTTATTGTTTTGCACTA

>NM_000548.5 Homo sapiens TSC complex subunit 2 (TSC2), transcript variant 1, mRNA

GCTTCCGGCGGCGTCCCGGGGCCAGGGGGGTGCGCCTTTCTCCGCGTCGGGGCGGCCCGGAGCGCGGTGG

CGCGGCGCGGGAGGGGTTTTCTGGTGCGTCCTGGTCCACCATGGCCAAACCAACAAGCAAAGATTCAGGC

TTGAAGGAGAAGTTTAAGATTCTGTTGGGACTGGGAACACCGAGGCCAAATCCCAGGTCTGCAGAGGGTA

AACAGACGGAGTTTATCATCACCGCGGAAATACTGAGAGAACTGAGCATGGAATGTGGCCTCAACAATCG

CATCCGGATGATAGGGCAGATTTGTGAAGTCGCAAAAACCAAGAAATTTGAAGAGCACGCAGTGGAAGCA

CTCTGGAAGGCGGTCGCGGATCTGTTGCAGCCGGAGCGGCCGCTGGAGGCCCGGCACGCGGTGCTGGCTC

TGCTGAAGGCCATCGTGCAGGGGCAGGGCGAGCGTTTGGGGGTCCTCAGAGCCCTCTTCTTTAAGGTCAT

CAAGGATTACCCTTCCAACGAAGACCTTCACGAAAGGCTGGAGGTTTTCAAGGCCCTCACAGACAATGGG

AGACACATCACCTACTTGGAGGAAGAGCTGGCTGACTTTGTCCTGCAGTGGATGGATGTTGGCTTGTCCT

CGGAATTCCTTCTGGTGCTGGTGAACTTGGTCAAATTCAATAGCTGTTACCTCGACGAGTACATCGCAAG

GATGGTTCAGATGATCTGTCTGCTGTGCGTCCGGACCGCGTCCTCTGTGGACATAGAGGTCTCCCTGCAG

GTGCTGGACGCCGTGGTCTGCTACAACTGCCTGCCGGCTGAGAGCCTCCCGCTGTTCATCGTTACCCTCT

GTCGCACCATCAACGTCAAGGAGCTCTGCGAGCCTTGCTGGAAGCTGATGCGGAACCTCCTTGGCACCCA

CCTGGGCCACAGCGCCATCTACAACATGTGCCACCTCATGGAGGACAGAGCCTACATGGAGGACGCGCCC

CTGCTGAGAGGAGCCGTGTTTTTTGTGGGCATGGCTCTCTGGGGAGCCCACCGGCTCTATTCTCTCAGGA

ACTCGCCGACATCTGTGTTGCCATCATTTTACCAGGCCATGGCATGTCCGAACGAGGTGGTGTCCTATGA

GATCGTCCTGTCCATCACCAGGCTCATCAAGAAGTATAGGAAGGAGCTCCAGGTGGTGGCGTGGGACATT

CTGCTGAACATCATCGAACGGCTCCTTCAGCAGCTCCAGACCTTGGACAGCCCGGAGCTCAGGACCATCG

TCCATGACCTGTTGACCACGGTGGAGGAGCTGTGTGACCAGAACGAGTTCCACGGGTCTCAGGAGAGATA

CTTTGAACTGGTGGAGAGATGTGCGGACCAGAGGCCTGAGTCCTCCCTCCTGAACCTGATCTCCTATAGA

GCGCAGTCCATCCACCCGGCCAAGGACGGCTGGATTCAGAACCTGCAGGCGCTGATGGAGAGATTCTTCA

GGAGCGAGTCCCGAGGCGCCGTGCGCATCAAGGTGCTGGACGTGCTGTCCTTTGTGCTGCTCATCAACAG

GCAGTTCTATGAGGAGGAGCTGATTAACTCAGTGGTCATCTCGCAGCTCTCCCACATCCCCGAGGATAAA

GACCACCAGGTCCGAAAGCTGGCCACCCAGTTGCTGGTGGACCTGGCAGAGGGCTGCCACACACACCACT

TCAACAGCCTGCTGGACATCATCGAGAAGGTGATGGCCCGCTCCCTCTCCCCACCCCCGGAGCTGGAAGA

AAGGGATGTGGCCGCATACTCGGCCTCCTTGGAGGATGTGAAGACAGCCGTCCTGGGGCTTCTGGTCATC

CTTCAGACCAAGCTGTACACCCTGCCTGCAAGCCACGCCACGCGTGTGTATGAGATGCTGGTCAGCCACA

TTCAGCTCCACTACAAGCACAGCTACACCCTGCCAATCGCGAGCAGCATCCGGCTGCAGGCCTTTGACTT

CCTGTTGCTGCTGCGGGCCGACTCACTGCACCGCCTGGGCCTGCCCAACAAGGATGGAGTCGTGCGGTTC

AGCCCCTACTGCGTCTGCGACTACATGGAGCCAGAGAGAGGCTCTGAGAAGAAGACCAGCGGCCCCCTTT

CTCCTCCCACAGGGCCTCCTGGCCCGGCGCCTGCAGGCCCCGCCGTGCGGCTGGGGTCCGTGCCCTACTC

CCTGCTCTTCCGCGTCCTGCTGCAGTGCTTGAAGCAGGAGTCTGACTGGAAGGTGCTGAAGCTGGTTCTG

GGCAGGCTGCCTGAGTCCCTGCGCTATAAAGTGCTCATCTTTACTTCCCCTTGCAGTGTGGACCAGCTGT

GCTCTGCTCTCTGCTCCATGCTTTCAGGCCCAAAGACACTGGAGCGGCTCCGAGGCGCCCCAGAAGGCTT

CTCCAGAACTGACTTGCACCTGGCCGTGGTTCCAGTGCTGACAGCATTAATCTCTTACCATAACTACCTG

GACAAAACCAAACAGCGCGAGATGGTCTACTGCCTGGAGCAGGGCCTCATCCACCGCTGTGCCAGCCAGT

GCGTCGTGGCCTTGTCCATCTGCAGCGTGGAGATGCCTGACATCATCATCAAGGCGCTGCCTGTTCTGGT

GGTGAAGCTCACGCACATCTCAGCCACAGCCAGCATGGCCGTCCCACTGCTGGAGTTCCTGTCCACTCTG

GCCAGGCTGCCGCACCTCTACAGGAACTTTGCCGCGGAGCAGTATGCCAGTGTGTTCGCCATCTCCCTGC

CGTACACCAACCCCTCCAAGTTTAATCAGTACATCGTGTGTCTGGCCCATCACGTCATAGCCATGTGGTT

CATCAGGTGCCGCCTGCCCTTCCGGAAGGATTTTGTCCCTTTCATCACTAAGGGCCTGCGGTCCAATGTC

CTCTTGTCTTTTGATGACACCCCCGAGAAGGACAGCTTCAGGGCCCGGAGTACTAGTCTCAACGAGAGAC

CCAAGAGTCTGAGGATAGCCAGACCCCCCAAACAAGGCTTGAATAACTCTCCACCCGTGAAAGAATTCAA

GGAGAGCTCTGCAGCCGAGGCCTTCCGGTGCCGCAGCATCAGTGTGTCTGAACATGTGGTCCGCAGCAGG

ATACAGACGTCCCTCACCAGTGCCAGCTTGGGGTCTGCAGATGAGAACTCCGTGGCCCAGGCTGACGATA

GCCTGAAAAACCTCCACCTGGAGCTCACGGAAACCTGTCTGGACATGATGGCTCGATACGTCTTCTCCAA

CTTCACGGCTGTCCCGAAGAGGTCTCCTGTGGGCGAGTTCCTCCTAGCGGGTGGCAGGACCAAAACCTGG

CTGGTTGGGAACAAGCTTGTCACTGTGACGACAAGCGTGGGAACCGGGACCCGGTCGTTACTAGGCCTGG

ACTCGGGGGAGCTGCAGTCCGGCCCGGAGTCGAGCTCCAGCCCCGGGGTGCATGTGAGACAGACCAAGGA

GGCGCCGGCCAAGCTGGAGTCCCAGGCTGGGCAGCAGGTGTCCCGTGGGGCCCGGGATCGGGTCCGTTCC

ATGTCGGGGGGCCATGGTCTTCGAGTTGGCGCCCTGGACGTGCCGGCCTCCCAGTTCCTGGGCAGTGCCA

CTTCTCCAGGACCACGGACTGCACCAGCCGCGAAACCTGAGAAGGCCTCAGCTGGCACCCGGGTTCCTGT

GCAGGAGAAGACGAACCTGGCGGCCTATGTGCCCCTGCTGACCCAGGGCTGGGCGGAGATCCTGGTCCGG

AGGCCCACAGGGAACACCAGCTGGCTGATGAGCCTGGAGAACCCGCTCAGCCCTTTCTCCTCGGACATCA

ACAACATGCCCCTGCAGGAGCTGTCTAACGCCCTCATGGCGGCTGAGCGCTTCAAGGAGCACCGGGACAC

AGCCCTGTACAAGTCACTGTCGGTGCCGGCAGCCAGCACGGCCAAACCCCCTCCTCTGCCTCGCTCCAAC

ACAGTGGCCTCTTTCTCCTCCCTGTACCAGTCCAGCTGCCAAGGACAGCTGCACAGGAGCGTTTCCTGGG

CAGACTCCGCCGTGGTCATGGAGGAGGGAAGTCCGGGCGAGGTTCCTGTGCTGGTGGAGCCCCCAGGGTT

GGAGGACGTTGAGGCAGCGCTAGGCATGGACAGGCGCACGGATGCCTACAGCAGGTCGTCCTCAGTCTCC

AGCCAGGAGGAGAAGTCGCTCCACGCGGAGGAGCTGGTTGGCAGGGGCATCCCCATCGAGCGAGTCGTCT

CCTCGGAGGGTGGCCGGCCCTCTGTGGACCTCTCCTTCCAGCCCTCGCAGCCCCTGAGCAAGTCCAGCTC

CTCTCCCGAGCTGCAGACTCTGCAGGACATCCTCGGGGACCCTGGGGACAAGGCCGACGTGGGCCGGCTG

AGCCCTGAGGTTAAGGCCCGGTCACAGTCAGGGACCCTGGACGGGGAAAGTGCTGCCTGGTCGGCCTCGG

GCGAAGACAGTCGGGGCCAGCCCGAGGGTCCCTTGCCTTCCAGCTCCCCCCGCTCGCCCAGTGGCCTCCG

GCCCCGAGGTTACACCATCTCCGACTCGGCCCCATCACGCAGGGGCAAGAGAGTAGAGAGGGACGCCTTA

AAGAGCAGAGCCACAGCCTCCAATGCAGAGAAAGTGCCAGGCATCAACCCCAGTTTCGTGTTCCTGCAGC

TCTACCATTCCCCCTTCTTTGGCGACGAGTCAAACAAGCCAATCCTGCTGCCCAATGAGTCACAGTCCTT

TGAGCGGTCGGTGCAGCTCCTCGACCAGATCCCATCATACGACACCCACAAGATCGCCGTCCTGTATGTT

GGAGAAGGCCAGAGCAACAGCGAGCTCGCCATCCTGTCCAATGAGCATGGCTCCTACAGGTACACGGAGT

TCCTGACGGGCCTGGGCCGGCTCATCGAGCTGAAGGACTGCCAGCCGGACAAGGTGTACCTGGGAGGCCT

GGACGTGTGTGGTGAGGACGGCCAGTTCACCTACTGCTGGCACGATGACATCATGCAAGCCGTCTTCCAC

ATCGCCACCCTGATGCCCACCAAGGACGTGGACAAGCACCGCTGCGACAAGAAGCGCCACCTGGGCAACG

ACTTTGTGTCCATTGTCTACAATGACTCCGGTGAGGACTTCAAGCTTGGCACCATCAAGGGCCAGTTCAA

CTTTGTCCACGTGATCGTCACCCCGCTGGACTACGAGTGCAACCTGGTGTCCCTGCAGTGCAGGAAAGAC

ATGGAGGGCCTTGTGGACACCAGCGTGGCCAAGATCGTGTCTGACCGCAACCTGCCCTTCGTGGCCCGCC

AGATGGCCCTGCACGCAAATATGGCCTCACAGGTGCATCATAGCCGCTCCAACCCCACCGATATCTACCC

CTCCAAGTGGATTGCCCGGCTCCGCCACATCAAGCGGCTCCGCCAGCGGATCTGCGAGGAAGCCGCCTAC

TCCAACCCCAGCCTACCTCTGGTGCACCCTCCGTCCCATAGCAAAGCCCCTGCACAGACTCCAGCCGAGC

CCACACCTGGCTATGAGGTGGGCCAGCGGAAGCGCCTCATCTCCTCGGTGGAGGACTTCACCGAGTTTGT

GTGAGGCCGGGGCCCTCCCTCCTGCACTGGCCTTGGACGGTATTGCCTGTCAGTGAAATAAATAAAGTCC

TGACCCCAGTGCACAGACATAGAGGCACAGATTGCAGTCAGACAGCTCTTTTATTGACTTTGTCTGCTTG

GTGCGGGGGTTGGGGGGGTGTCGAGGCTCTAGAAGCGGCCATGCCCACAGAAGTGGTACACAGAAGCAGG

CACAGCCAGCTCCGAGGGCCTTGAGGCTGCCTGGGCCATACAGCACACTCGCGCGTGCGCGCGCGCACAC

ACACACACACACAGTCACCTTCCTCCACCCTGGGAGCCAGCCCCCAGGAGGAGTCTTTTCCTCTAACCAC

CCTGGGGTCCTCTGACATGCCTAGTCCTGCTACTTGCCCAGACCTGATGCCAGCAGGCCTGGGCGCTGCT

CTCTTGCTACCTGGCCTGGGGCAAGGGAGGATGACAAGGCCTCTGGGGTGATGAGAGTGCCTGGCAGACA

GCTGTGCCCCCAGCACCGGCCCAAGGCCAAGCTCGCATCCAAGCAGCAGCCGGGCTGCCATAACGCCACC

ACACCTACCAAGCGCAGCAGGTGTTGGGGGAGGCCAGCTCTGGGCGCAGGCCCCTCAGCCCTAGTGAAAA

TAGTGACATACAAAAATATACACATTTTAACACCATATAAATTACTGACACGAGACACACAGTGAGACGG

TGCAGGGAGTACGGTAGGAACTGGAGAGGTAATAACTTAGGGGCAGGGTGGCGGCGGTGCAGGCTAACCC

TCCCTGAAGCCAGCAGCCTTAGCAGTGGGGGACATCTGCCCAGGGGGTGGGGCCGGGCACAGCCCGCTGT

ACCTGAGGACTCGGGGAAATAAATTAGCATCTCAGAGGCTAGAAA

>NM_001004125.3 Homo sapiens tumor suppressor candidate 1 (TUSC1), mRNA

AGCAGCCGCCGCCCGCGACCTCCTCTCCTCCTCCGTCGTCACCCCGGCCGCCCTGCCAGCCCCGAACTTG

CCCGCGCCTGGCCGCGCGGCCCCTCAGCCCACGGGGCTGGCGGCGCGGTTGGGATGGGACCGATGTGGCG

CATGCGTGGTGGCGCCACTAGGCGCGGGAGTTGCTGCGGCGGGGACGGTGCTGCGGACGGCCGAGGGCCA

GGCCGCTCCGGCCGGGCTCGTGGTGGGGGCAGCCCCAGCGGCGGCGGCGGCGGCGTGGGCTGGCGAGGCC

GCGCGGACGGCGCCCGACAGCAGCTGGAGGAGCGGTTTGCCGACCTGGCGGCGAGCCACTTGGAGGCCAT

CCGTGCGCGGGACGAGTGGGACCGGCAGAACGCGCGGCTGCGTCAGGAGAACGCCCGGCTGCGGCTCGAG

AACCGGCGGCTGAAGCGCGAGAACCGCAGCCTCTTCCGTCAGGCTTTGCGGCTCCCCGGCGAAGGCGGCA

ACGGGACGCCCGCGGAGGCACGCCGGGTCCCTGAAGAGGCCAGCACGAACCGGAGGGCTAGAGACAGCGG

CCGAGAGGACGAGCCGGGCAGCCCCAGGGCCCTGAGGGCCCGACTTGAGAAGCTGGAAGCCATGTACCGC

CGGGCCCTGCTGCAGCTGCACCTCGAGCAGCGGGGACCACGTCCGAGCGGGGACAAGGAGGAGCAGCCTC

TACAGGAACCCGACTCCGGCCTCCGCTCCCGGGACTCGGAGCCCTCTGGGCCCTGGCTGTAGCCGAGGCC

GCAGCCGGTGAGAGGCGGAGCCTCGAGCGGGCCCCTCCTCCTCCGTTCCCAGCTACCCCCGACCTCGCGC

ACGCCCTGGCCCGCTCCCTGCCCGCGGATACCTTGGACTACGGTGCCTTCCCGCCCCCACCAGTGGACAC

TTCCGGGTGGCGCTTGGCCATCCGCTTGGTGGAGGTCGAGTGGGTGCAAGGGCCCTCAGGACCTCCCACA

TGTACAGTTCCCCTGCCTCTTGCCCCGGCTAGTTTCCCTGGAGAGAGGCCTAGCCAACAACGAAGGGACG

GATTTTAAAACAGCGAGAACTGGGTGCCAAGAAACACTTCATATTCCCTGAACTTTTCTGGATGTTGTGC

GAACCTGTTGTATTTTTTTTTTTCCTGTATTTTGGTTCTGAGGGATGAGTAATGTGTTTTTAGTTGGTTG

AGTTTATCCATTCTTCAATCCCCTAAAGCCTTCAGCTATTCTTTCATATGTGGCCAACATTTTAAGTACA

GTGTTTGCTTTTATAAGTCGGTGTTACTTGGATGTCCTATAAAATACCCACTTTTTATAAAGATGATATT

TATAACCTAGGAACTCATGCCTGTCTTGGCTGCAGTCCCTGTACATCTAATATATACTGATTTGTTTGCC

CCCAGAGAATTTTTAAGGTATCTGCCTATATCGTGATCTTTTGAAATGGTAAATAAATCTGGTGCATTAA

AAATTA

>NM_003565.4 Homo sapiens unc-51 like autophagy activating kinase 1 (ULK1), mRNA

AGTGCGGCTGCGCGGGCGTCTCAGGCTCTGAGGCCCGGGCGCCGCGGCTCTTTTGTTTCTCCGTTGGGGC

CGAGCCCGGGCCCTAGTTGGAGCCGGAGTCGGAGTCGGAGTCGGATCCGGATTCGGATTAGCAGCCCGGG

AAGAGTGCCGTGGCACAGGCGCCGGAGGGAGCGCGACCCTCGGACCCCGCCTGGCCCGCGGGGCTGGGAC

CCGGCCCCGGCCTGCCCGATGGGGCGCGCGGCCCCGGAGATGCGCCCTCGCCCGGCCCCGCGCCCCCGGC

CCCGCGCCCCCGGCCCGCCCGCCCCGGCCCGCGCCTCCGCCTGAGTCCCCCGCGCCTTGGCCCGCCACCC

CCCGCCCCGCGCCCCCGGCCCGCCTGCGCCATGGAGCCCGGCCGCGGCGGCACAGAGACCGTGGGCAAGT

TCGAGTTCTCCCGCAAGGACCTGATCGGCCACGGCGCCTTCGCGGTGGTCTTCAAGGGCCGCCACCGCGA

GAAGCACGATTTGGAGGTCGCCGTCAAGTGCATTAACAAGAAGAACCTCGCCAAGTCTCAGACGCTGCTG

GGGAAGGAAATCAAAATCCTGAAGGAACTGAAACATGAAAACATCGTGGCCCTGTACGACTTCCAGGAAA

TGGCTAATTCTGTCTACCTGGTTATGGAGTACTGCAACGGTGGGGACCTGGCCGACTACCTGCACGCCAT

GCGCACGCTGAGCGAGGACACCATCAGGCTCTTCCTGCAGCAGATCGCGGGCGCCATGCGGCTTCTGCAC

AGCAAAGGCATCATCCACCGCGACCTGAAACCGCAGAACATCCTGCTGTCCAACCCCGCCGGCCGCCGCG

CCAACCCCAACAGCATCCGCGTCAAGATCGCTGACTTCGGCTTCGCGCGGTACCTCCAGAGCAACATGAT

GGCGGCCACACTCTGCGGCTCCCCCATGTACATGGCCCCCGAGGTCATCATGTCCCAGCACTACGACGGG

AAGGCGGACCTGTGGAGCATCGGCACCATCGTCTACCAGTGCCTGACGGGGAAGGCGCCCTTCCAGGCCA

GCAGCCCCCAGGACCTGCGCCTGTTCTACGAGAAGAACAAGACGTTGGTCCCCACCATCCCCCGGGAGAC

CTCGGCCCCGCTGCGGCAGCTGCTCCTGGCCCTACTGCAACGCAACCACAAGGACCGCATGGACTTCGAT

GAGTTTTTTCATCACCCTTTCCTCGATGCCAGCCCCTCGGTCAGGAAATCCCCACCCGTGCCTGTGCCCT

CGTACCCAAGCTCGGGGTCCGGCAGCAGCTCCAGCAGCAGCTCCACCTCCCACCTGGCCTCCCCGCCGTC

CCTGGGCGAGATGCAGCAGCTGCAGAAGACCCTGGCCTCCCCGGCTGACACCGCTGGCTTCCTGCACAGC

TCCCGGGACTCTGGTGGCAGCAAGGACTCTTCCTGTGACACAGACGACTTCGTCATGGTCCCCGCGCAGT

TTCCAGGTGACCTGGTGGCTGAGGCGCCCAGTGCCAAACCCCCGCCAGACAGCCTGATGTGCAGTGGGAG

CTCACTGGTGGCCTCTGCGGGCTTGGAGAGCCACGGCCGGACCCCATCTCCATCCCCACCCTGCAGCAGC

TCCCCCAGTCCCTCAGGCCGGGCTGGCCCGTTCTCCAGCAGCAGGTGCGGCGCCTCTGTCCCCATCCCAG

TCCCCACGCAGGTGCAGAACTACCAGCGCATTGAGCGAAACCTGCAGTCACCCACCCAGTTCCAAACACC

TCGGTCCTCTGCCATCCGCAGGTCAGGCAGCACCAGCCCCCTGGGCTTTGCAAGGGCCAGCCCCTCGCCC

CCTGCCCACGCTGAGCATGGAGGCGTCCTGGCCAGGAAGATGTCTCTGGGTGGAGGCCGGCCCTACACGC

CATCTCCTCAAGTTGGAACCATCCCTGAGCGGCCAGGCTGGAGCGGGACGCCCTCCCCACAGGGAGCTGA

GATGCGGGGTGGCAGGTCCCCTCGTCCAGGCTCCTCTGCACCCGAGCACTCTCCCCGCACTTCCGGGCTG

GGCTGCCGCCTGCACAGCGCCCCCAACCTGTCTGACTTGCACGTCGTCCGCCCCAAGCTGCCCAAACCCC

CCACGGACCCCCTGGGAGCTGTGTTCAGCCCACCACAGGCCAGCCCTCCCCAGCCGTCCCACGGCCTGCA

GTCCTGCCGGAACCTGCGGGGCTCACCCAAGCTGCCCGACTTCCTGCAGCGAAACCCCCTGCCCCCCATC

CTGGGCTCCCCCACCAAGGCTGTGCCCTCCTTTGACTTCCCGAAGACCCCCAGCTCCCAGAACCTGCTGG

CCCTCCTAGCCCGGCAGGGCGTGGTGATGACGCCCCCTCGAAACCGGACGCTGCCCGACCTCTCGGAGGT

GGGACCCTTCCATGGTCAGCCGTTGGGCCCTGGCCTGCGGCCAGGCGAGGACCCCAAGGGCCCCTTTGGC

CGGTCTTTCAGCACCAGCCGCCTCACTGACCTGCTCCTTAAGGCGGCGTTTGGGACACAAGCCCCGGACC

CGGGCAGCACGGAGAGCCTGCAGGAGAAGCCCATGGAGATCGCACCCTCAGCTGGCTTTGGAGGGAGCCT

GCACCCAGGAGCCCGTGCTGGGGGCACCAGCAGCCCTTCCCCGGTGGTCTTCACCGTGGGCTCTCCCCCG

AGCGGGAGCACGCCCCCCCAGGGCCCCCGCACCAGGATGTTCTCAGCGGGCCCCACTGGCTCTGCCAGCT

CTTCTGCCCGCCACCTGGTGCCTGGGCCCTGCAGCGAGGCCCCAGCCCCTGAGCTCCCTGCTCCAGGACA

CGGCTGCAGCTTTGCCGACCCCATTACTGCGAACCTGGAGGGGGCTGTGACCTTCGAGGCCCCCGACCTC

CCTGAGGAGACCCTCATGGAGCAAGAGCACACGGAGATCCTGCGTGGCCTGCGCTTCACGCTGCTGTTCG

TGCAGCACGTCCTGGAGATCGCAGCCCTGAAGGGCAGCGCCAGTGAGGCGGCGGGGGGCCCTGAGTACCA

GCTGCAGGAGAGTGTGGTGGCCGACCAGATCAGCCTGCTGAGCCGAGAATGGGGCTTCGCGGAACAGCTG

GTGCTGTACCTGAAGGTGGCCGAGCTACTGTCCTCCGGCCTGCAAAGTGCCATCGACCAGATCCGGGCCG

GCAAGCTCTGCCTGTCGTCCACTGTGAAGCAGGTGGTGCGCAGGCTGAATGAGCTGTACAAGGCCAGCGT

GGTGTCCTGCCAGGGCCTGAGCCTGCGGCTGCAGCGCTTCTTCCTGGACAAGCAGCGGCTCCTGGACCGC

ATTCACAGCATCACTGCCGAGAGGCTCATCTTCAGCCACGCTGTGCAGATGGTGCAGTCGGCTGCCCTGG

ACGAGATGTTCCAGCACCGTGAGGGCTGCGTCCCACGCTACCACAAGGCCCTGCTGCTCCTGGAGGGGCT

GCAGCACATGCTCTCGGACCAGGCCGACATCGAGAACGTCACCAAGTGCAAGCTGTGCATTGAGCGGAGA

CTCTCGGCGCTGCTGACTGGCATCTGTGCCTGACCTTTCTGGCCTGGCTGGGCCCCCGTCCTGCCGAGC

CCTGCAGAGTGGGCTCTGTGTGCTGGCTGGACTCCTCGGGACAAGCCCATGGCGCTGATCGCTGGTGCTG

AGCCCTGCCCTGGGCCCCACGGACAGTCAGCCTGCCGGCCTCCCTGCAGCTCACGGGGCAGAACCAGCAC

ATCTGGAGCCACACAGCTTGGGGGGTGTCTCCCATCTTTTACAGGTGGGGATCACAGAATTTCTGCCCCT

CCAGCTGCCTGGCTCAGCAGGCGTGGGTGCCACCACCCTCTAGCCCCAGGGCAGCCCCGGAGGACAGGCA

AGGGCCTGAGACCACTGCCGACTCAAAGCCAAAGCGAGCTCCTGCTTAGGGAAGGTCAGCAGGCACTGTG

CCCAGGAAGAGCCTGCGGCCTCGGCGTCCCCCAGTCTCCAGGAGCCTCTCCCTCCGAGATACCCACCCAG

CTTTGTCAATCACCCAAGCACTTTATGCATATAGAGACAGAACCTGGACCTCACCAGGGACTGCTGGGCA

GCGATTCCTGGCAGTGGCCTGGTGTTTGTACATACACATATGCAGACACATGCCAGGGCCCCCAAGCCC

GAGCACCGGACCACGTTGCTGCCCAGGTCTGGACCTCAGCGGGAGAACTGGCTCCGGGGGGAGTGGGGCC

CTGCGCTAGAGGCAGAGGCAGTTCTTTGTTCAAGCGTTCCTCTGGGGACCGGCAGCAGAGGCACCGTGTT

CTCTCAGCCCTGGATACGTCTTGTAATCTTTCACACTTTATTCCTAAAACGTGTCTTATTTTTATGCAGC

TCATTTTTTCTTTAAAGGAGAAAACTTGTAGGTGTTTAAGAATTGGTTTTGGGAGGGCGAGGACTGGGCC

AGGTTAGAGGCAGATGGCACAGGGGCGTGTGGCGGGCGGGTGAGGCTGCTTTGCACACCTGTGTTGGTGG

CTGTCCCCTGCCGCCCCTCCCTGTGGCAGCAGCAGGACAGGTGTGTGCCCAGCACCCTCCCTACCTGGGC

CTGGAAGCAGATGAGGGGAATACTTCATGCAAAGAAAAAAGTAACATGTGCAAAAGCTCCCCGTCCAGCT

TTGACAGTCAGTTTTGATGTCAGCTCCTCGGCAGGGTAGGCCTGATGACAGCCCTGTCCCTCCCTGCCTC

TGCCTTGCCCAAGGCCACGGAGGGCATCTGCAGAGAGGCCTGCCTTCCGGATTCCAGGCGGGCATGCCCT

GCAAACCCCGCCTGGGCCTCCCTTGGTCTGCCCAGCCCTCGATTAGCCCTGCCTGAATCAGTAGATACTT

GAACGAGTCCCCAGTCTGCGGGAGGCAGTGGTGGGGCCATGGACCCATGCGGGGGGTTCCAGGGTCACAC

GCCACATAACAGACAAAAATACACACACGTGTGTTTTTCTTTGCAATACTTGAAATATTGCCACTGTGCT

TGGACTTAGAAGAAGAAAATCCCCGTGACTTCTTCCTCATCACCTTGATGGCTTTATTCTCACCTTGTGG

GGCATGTTTGTATTTATTGCTTCATGGCCGACTGGAATCCTGAGTCCTGGGAAGCTGGCACTGCGGGGAT

CTTGCCCGGTGTCCTGGTCCTCTTGCTTCCGTCGCGGCCGCATGTGCGTGTGTCCAAGCAGGTCCTGGGC

GCCTCAACTGCTGCCCCTGGTTGAATGTTCTCTTGATAGTGCTGGACCCTTTGTCTATTTTAAAGCGAAT

TTTGTGTGATTTCCTGCCCTTTGCGTTATATTGTATAATACCAACGTAAGGAAATAAACCTTTGGAATTG

TT

>NM_014683.4 Homo sapiens unc-51 like autophagy activating kinase 2 (ULK2), transcript variant 1, mRNA

AGTCGCGCGGGAGCGGGTAGCGCGCGAGGGCGTTGGGCGCCGCCGCGAGGCGGGGAAGCGCGGGGCCG

CGGCGGTGCGGGTTCTAGGGCGGCGGCCGTCGCCGTCGCAGCAGCGCCCCGAGCGGGGAGGGCCGAGGAG

GCCCGACGAGCTGGGGATGGAGAGTACCGGGCCCCTCACTGCCTCAGAGCGCGTGTGCGGCTCTGGGCGC

GCACAGTGACGGTGACGGCACCCCTGGCCCGGCAGCGCCGAGGCCGCTTCGCCAGACAGCCAGCGGCCGG

CGGCAGGCCGGGCCATGAGCGGCAGGGGCCGGGCCGGGCCTCGCTGACCCTGGCTCCGCGCGGCAGCTTC

CCCAGTTTCCGCTCCGGTCTCTCGGCATGAGAGTCCGCCCGGGCCCGGGGCTGCGGCTGCCCCAGACCCG

CCGCACGCTGGCGCGCTCCGGGCCCGCGGAGCCGCGGTGCTGATACCTGCGCCGCACTGCGCCGCCCGCC

CGTCCGCTGTGTGCCCCGGGGGCGCGGCCATGGAGGTGGTGGGTGACTTCGAGTACAGCAAGAGGGATCT

CGTGGGACACGGGGCCTTCGCCGTGGTCTTCCGGGGGCGGCACCGCCAGAAAACTGATTGGGAGGTAGCT

ATTAAAAGTATTAATAAAAAGAACTTGTCAAAATCACAAATACTGCTTGGAAAGGAAATTAAAATCTTAA

AGGAACTTCAGCATGAAAATATTGTAGCACTCTATGATGTTCAGGAATTACCCAACTCTGTCTTTTTGGT

GATGGAGTATTGCAATGGTGGAGACCTCGCAGATTATTTGCAAGCGAAAGGGACTCTCAGTGAAGACACG

ATCAGAGTGTTTCTGCATCAGATTGCTGCTGCCATGCGAATCCTGCACAGCAAAGGAATCATCCACAGAG

ATCTCAAACCACAGAACATCTTGCTGTCCTATGCCAATCGCAGAAAATCAAGTGTCAGTGGTATTCGCAT

CAAAATAGCGGATTTTGGTTTTGCTCGTTACCTACATAGTAACATGATGGCTGCAACACTGTGTGGATCC

CCGATGTACATGGCTCCTGAGGTTATTATGTCTCAACATTATGATGCTAAGGCTGACTTGTGGAGCATAG

GAACAGTGATATACCAATGCCTAGTTGGAAAACCACCTTTTCAGGCCAATAGTCCTCAAGACTTAAGGAT

GTTTTATGAAAAAAACAGGAGCTTAATGCCTAGTATTCCCAGAGAAACATCACCTTATTTGGCTAATCTC

CTTTTGGGTTTGCTTCAGAGAAACCAAAAAGATAGAATGGACTTTGAAGCATTTTTTAGCCATCCTTTTC

TTGAGCAAGGTCCAGTAAAAAAATCTTGCCCAGTTCCAGTGCCCATGTATTCTGGTTCTGTCTCTGGAAG

CTCCTGTGGCAGCTCTCCATCTTGTCGTTTTGCTTCTCCACCATCCCTTCCAGATATGCAGCATATTCAG

GAAGAAAACTTATCTTCCCCACCATTGGGTCCTCCCAACTATCTACAAGTTTCCAAAGATTCTGCCAGTA

CTAGTAGCAAGAACTCTTCTTGTGACACGGATGACTTTGTTTTGGTGCCACACAACATCTCGTCAGACCA

CTCATGTGATATGCCAGTGGGGACTGCTGGCAGACGTGCTTCAAATGAATTCTTGGTGTGTGGAGGGCAG

TGTCAGCCTACTGTGTCACCTCACAGCGAAACAGCACCAATTCCAGTTCCTACTCAAATAAGGAATTATC

AGCGCATAGAGCAGAATCTTACATCTACTGCCAGCTCAGGCACAAATGTACATGGTTCTCCAAGATCTGC

AGTGGTACGAAGGTCCAACACCAGCCCCATGGGCTTCCTCCGGCCGGGATCATGCTCCCCAGTACCAGCA

GACACAGCACAGACAGTTGGACGAAGGCTCTCCACTGGGTCTTCTAGGCCTTACTCACCTTCCCCTTTGG

TTGGTACCATTCCTGAGCAATTCAGTCAGTGCTGCTGTGGGCATCCTCAGGGCCATGACTCCAGGAGTAG

AAACTCCTCAGGTTCTCCAGTGCCACAAGCTCAGTCCCCACAGTCTCTCTTATCGGGTGCTAGACTGCAG

AGCGCCCCCACCCTCACTGACATCTATCAGAACAAGCAGAAGCTCAGAAAACAGCACTCTGACCCCGTGT

GCCCATCCCATACTGGGGCTGGGTACAGCTACTCGCCTCAGCCCAGTCGGCCTGGCAGCCTTGGAACTTC

TCCCACCAAGCACTTGGGGTCCTCTCCACGGAGTTCTGACTGGTTCTTTAAAACTCCTTTGCCAACAATC

ATTGGCTCTCCTACTAAGACCACAGCTCCTTTCAAAATCCCTAAAACTCAAGCATCTTCCAACCTGTTAG

CCTTGGTTACTCGTCATGGGCCTGCTGAAGAACAGTCGAAAGATGGGAATGAGCCACGGGAATGTGCCCA

TTGCCTCTTAGTGCAAGGAAGTGAGAGGCAGCGGGCCGAGCAGCAGAGCAAGGCAGTGTTTGGCAGATCT

GTCAGTACCGGGAAGTTATCAGATCAACAAGGAAAGACTCCTATATGTCGACATCAGGGCAGCACAGACA

GTTTAAATACAGAACGACCAATGGATATAGCTCCGGCAGGAGCCTGTGGTGGTGTTCTGGCACCTCCTGC

AGGTACAGCAGCAAGTTCCAAGGCTGTCCTCTTCACTGTAGGGTCTCCTCCACACAGTGCGGCAGCCCCC

ACTTGTACCCACATGTTCCTTCGAACAAGAACAACCTCAGTGGGGCCCAGCAACTCCGGGGGCTCTCTTT

GTGCCATGAGTGGCCGCGTGTGCGTGGGGTCCCCGCCTGGCCCAGGCTTCGGCTCTTCCCCTCCAGGAGC

AGAGGCAGCTCCCAGCCTGAGATACGTGCCTTACGGTGCTTCACCCCCCAGCCTAGAGGGGCTCATCACC

TTTGAAGCCCCTGAACTGCCGGAGGAGACGCTGATGGAGCGGGAACACACAGACACCTTACGCCATCTGA

ATGTGATGCTGATGTTCACTGAGTGTGTGCTGGACCTGACAGCCATGAGGGGAGGAAACCCTGAGCTGTG

CACATCTGCTGTGTCCTTGTACCAGATCCAGGAGAGTGTGGTGGTGGACCAGATCAGTCAGCTGAGCAAA

GACTGGGGGCGGGTGGAGCAGCTGGTGTTGTACATGAAAGCAGCACAGCTGCTTGCGGCTTCTCTGCATC

TTGCCAAAGCCCAGATCAAGTCCGGGAAACTGAGCCCATCCACAGCTGTGAAACAAGTTGTCAAGAATCT

GAACGAACGATATAAATTCTGCATCACCATGTGCAAGAAACTTACAGAAAAGCTGAATCGATTCTTCTCT

GACAAACAGAGGTTTATTGATGAAATCAACAGTGTGACTGCAGAGAAACTCATCTATAATTGTGCTGTAG

AAATGGTTCAGTCTGCAGCCCTGGATGAGATGTTTCAGCAGACCGAAGATATTGTTTATCGCTATCATAA

GGCAGCCCTTCTTTTGGAAGGCCTAAGTAGGATTCTACAGGACCCTGCAGATATTGAAAATGTGCATAAA

TATAAATGTAGTATTGAGAGAAGACTGTCGGCGCTCTGCCATAGCACCGCAACCGTGTGAGCAGCAGGCT

CATCCCGTGGACCGGTGGTGGGAACGTGAGGTGATGCCTTTGGGATTACAGCTTGAGTTCTGTCACCCCA

TCCCCAGGAAACTGTAGCTTCTTAACTGGTGACTACCAAAGAACAAGCAGTGATTTGAAAAAGGAAAAAC

AATCCAAAAACTACATATTTGTAGGAAATCTGCCTTATTGGAGAAAATCACCCTTTCCCTTTTTCTTTGT

AGAAGCAGGAGCAAGAGTGTTTGGCTCCCAGTTTGGACTTGGTGAATAAATGTACCTTAGAACTAGGATA

ATCGGTACAGTTATTCTTAAAGATAATTAAAAATGAAACAAAGTGAGTGCTCGTCACTGGGTTCATCAGA

GCAGTGTGTGAAATTCCATGTGTTTGCTGAGGTGTAAAGGTAAATGTATTCACCCCTCATCCAGGCAGTT

TGATATTTGGAGTAAGTTTGTTTAAATCTGAGCATGCATCTTTAAACAGCTCAGGAAGAAATAGCTTAAG

AAGAAGTGAAACATGGATCTTGGAAGAAATTTTGAAATCTTCAATTTGATCCTAATATGGATACATGTTA

ATCTTCCAAAATCTTTCATATTGCACTAATTTATTAAAACAACTGTGTATTGGATTTTGTAATTTAACTA

AGGCACAATGGACTTGTTTAAAATATTTTACTTGATTGTATACATAGACCCTTTCCAGAATTCACATGTA

ATCTCCAGTGAACTTTTAAGTGGTTAAAACTTGTATTCATGTGAACCTTTGCACATTTTTTTTTTTTACT

TCTTTATCTACACCTACAGATTTTCTCAGTAATGTTTTTGTTAGCTTTTGGTTCCATTTTTTATTGTGCA

TGCAGAATGTACATTGATGCCTGTGACCTTAGGTTTATTAAAGGCTAGGTTTATTTGGGCAGTATTAGAA

ACAAAATCATGGATCAAGAGATACTCTTGATAATTTGAATAGGGCCAAAACAAAGTTGGTGACCTAAAGG

CTTGTTAGTGATGTGGAGTTCCTACATGCAGTGAGTGGAAAATGAAGTTCGTTTTCTCTTAGGAAAATGG

GCAGCTGTCTTCTGCCTAATGTGTATTTTTCATGTTAATTCTGACAGTTCACCAAATAGCTAGTCATGGA

GAATGCAGGCAGTTAACTTAATATCCCTCCAGGAATGGTTCCTACGTTGTGTATTACTTGGTTTCTTTTA

CTTACCTGCTTGAATACTTGAATAAACCATTCACCAATTTTAATCCTTTTATTTTAATCCTTTTACATAA

AATAATCTGAACTCTTTGACAAATTGCACAGAGCTCTTTGGCATTAATCTAATTTTAATGTACTGATAAA

AACAAACATGGTTTTCCTTTACTTTGACAAAGTAATGTAATTTTTACCTTATTTATCTGTATGAAATTCC

AGTAGTTAATTTGAACATTTATTTATATGACGTTTGTATTTTTAGGTCTTTAATACAGTGTTTCTACCTC

TCATTTGTAACTGCATGCATTATTCTTGAAACTAGGTAAAACTCACTGAATTGTTGTGTAATAGCCTTTT

TATTATTGCCTGTACAAATGTATATTAAGGTAAAATAAAACTGACAAAGTGTTTCTAGGGTGTAGCTGGG

TACATATTAAGTGGCTTGTTGAGCCAGGTACTTCCTTAGTGAGTTTAGAGACTTGGCCATGAATATCCTT

TGTCCTGCCCCAGGATTTAGATCTTGGCTACTGTCATGCAGGCTTCCAGGAACATAGACTGTTTTACCTC

CACAACCCTATTTGTTATTAGTGATACTTTATTTTATATAATATTTTTTATTCACAGTGAAATTTCATTC

ATGTTCTTTCAGTTATCACCTGTGTTATCTCAGTTGTAGGTTTATTCTATCCTCTCCTCTTCCTCTCCCA

TTTCTTTTTTAAGACAGGATGAAACAGGTTCAGAGAGGGGAAGTGATTGGCCTAAAGTCAGGAACTAGGC

AAGTGGTCAAGCCATGCTTTGTGACTTTCAAGTTAATTCTTCTTGTTCTTGTATATTAAAGGTCTTGGGG

TAGATGGTGTGTGTGAAACAGTGAAGTCTCAACAGCAGAAAAGAACAAAATGTAAATTCATGAATAATGG

TTCTGGTTATACTTCCATTATCAAGGCTAATTAAGAGATTTTGCCTTGAGTATAGCAATAATAAACAAAT

GCTTTATGTTTCCCTGAGTATTTTTAAATTTCATATCGTGTTTCATAGAGTGGAAATGTACCAGTTCATA

CATTCATGGGTTGACACCTGTATTCAAGCCATAACCTGGAAAATTGCTGACCAGCTATTCCTGCTCTAAG

GGTTTGCTTTAACTATACCCTCCACTGGACTATATATAGAGAGAGAGCCTATGCTGTATATAGTTGGTTT

CCCACAGTCCCTGTCCCCTCCTTCCATTGAGAGAAGTAACTAGAGCAACAGTTGTTCTCCCTCGCTGGAG

TGGTGAATAGGAGCCGGGAGGAGGGGACTGTTAGGGTCGGGCTGCCTCAGTCTCCACAGTAATTAAGAGG

AAATTGAGTTGGATACAGCGGACATGAGGCCTGGAATGGGTACCCCAAACCCATGCATCTTAAGTGTCTG

GGTCCCCACGCCCCCTTCTGCCTGCCAGGAGTACTCTGTGCAGCCATCCTGTAACTGCTTCAGTCCAGAG

AAGGATAGCATCCATCCCTCCCTCCAGCTCGCCTCTCTGCTGAGGCTTTCTCCTCTCCACTCAGCATTTG

CAACAGCTTCTCTCCTGGCACTTGTCTGGGAATATCAAGTTAAAAATTACAACACCCAATTTTGATAACT

TTAGTTGAGAGAAACAACCTCTAGTGAAGACTGATAAGTAACTTATTGCCCGTAGTCCCATGTTCCTTTG

TCTCCACCAAGAGGAATTACCAGCCCAGCAGTTCTGCCCTCACTAGAGTCCCGTCCAGGAGCTTGAGAGG

AGAGGATTTTTAGGGCTGGATTGCCTCAGTCTGCACAGTAATAAGTTGAGTTAGACACAGTGGACATGAG

GACCTGGACTCAGATATCTGAAGAATCCTTATTATTTTTTTGTTGGCTTGTTTTGAGACAGAGTCACCCT

GTTGCCCAGGCTGGAGTGCATTGGCGCAATCTTGGCTCACTGCAACCTCTGCCTCCCAGGTTCAAGTGAT

TCTTCTTCCTCAGCCTCCCAGGTAGCTAGGATTACAGACATGCGCCACCACGCTCAGCTATTTTTCGTAT

TTTTAGTGGAGATGGGGTTTCACCATGTTGGTCAGGCTGGTCTTGAACACCAGCCTCAGCCTCCCCAAGT

GCTGGGATTACAGGCATGAGCCACCGTGCCCGGCCAAAATCCTTAATGTTTTTGTTTTTTGGTTTTTTTT

TTTGAGACAGTCTTGCTCTGTCACCCAGGCTGGAGTGCAGTGGCACAATCTTGGCTCACTGCAAGCTCCG

CCTCCGGGGTTCATGCCATTCTCCCGCCTCAGCCTCCCGAGTAGCTGGGACTACAGGCGCCTGCCACCAC

GCCCGGCTGATTTTTTGTATTTTTTAGTAGAGGCAGGGTTTCACCGTGTTAGGATGGTCTCGATCTCCTG

ACCTCGTGATCCGCCCGCCTCGGCCTCCCAAAGTGCTGGGATTACAGGCGTGAGCCACCGCGCCCGGGCT

ATCCTTAATGTTGATATTAGTGGTTAGCACATGTTTTGGAGGAGGACTTCACACTTTTTAAACTATGCTC

CCTCGTATTTTCTATAAGCAGTCTTAAATTCTTTCTGGAATAAAGCAAGATTGATCAGTATTTCTCAAGG

AGGATTATTTGGCAGGCAGTAGCTACTGCTCAGAGAACTTGGTATTGTTACATGCATCCTGGAAGTATCT

GTGCCTATTTCTAGACGGCCTCAGACCACTTTTTTCTGAGTCCTGGACCCTTGAATCTGCAGCCAAGCAA

GCTCTTGGGTCCCTCCCAAGTCTCATGTTTAGTAAAGCCCTGTGAGAATAATTTTATTGGTGGAGGAGTC

AAGATGATACTTCCCTCACCTGCATCAGAGGAAACCACATCAGAAACAATCCACCTAGAAAAACACTGCA

TACGGGAAAGCTGACAACTTGCCCTGAGAAATCACAGCTTTGGTGCAAGAGGTGAAGCCGAACTGGTAAA

AGATCAAAGTGAATTCAGAGATCAAACTGTACTAGGGAAAGGAGCAGAGGCTGAGATCTTGCTGCCAGCG

TGACCTCTCACAGAGTGGCCCAGCAGAGGCTGTGCAGAGATGGCCAGAGCCAGGACCCAGCCCTGTGGAG

CCCCTGGGCACATGTGGTCCCGAGTGAAGCCGTGCCTGTTGCTCTGAACAGGTGGGATCAATCTGTAGGT

TTTTCCACTTTTCACAGAGTGTGGCTCTCTTTAGGTCTGCACTCCCTCTCTGCGCAGGGCGGACCTCCCT

GGGCTCTGCAGCATGATCTCCTGTCCCAAGACAGTCGTGCTCTTGGACAGTTAGCTTCTGTCATTAGGAA

GTGCCCACATGAGCTCTTGTTTATTGGAAACCGTGTTCTATGCCTCCTTTGGGGTTTCTGCAACTCAATT

TGGCAGGTAGCTTAGTGGTCCACTTGGTCTCAAATTCCAGCTCCACCATGTGCAGTTGTGTGTTCTTTGG

CAAGTTCCTCCCCTCCTCCGAATCTGTCAGTGGGGGAAGAGTGCATGTCTCACAGAGTTGAGGTGAGAAA

TAAGTTAATGAATATGAAATTCTTTGCCCAGCCCCTGGCACGTGGAAGGGCTCAGCAATCCTGTATCCGT

TGTAGTGGTCATCACTCTGTGGCTCTTCACTGCTTTTGTTTTGTTTTGGGAGGGGGTGGAGAACAATTTG

TTCTGTGTTTGTGTGGGGCAAGGCCTGTCTTCAGGGCTCTCTCCAACCACGTACGCTCTGGCTGGAGCTT

CTGAAAGGACACAGTTCTTGCATAGAAGTTGCTTTCTCCAGCTTGCCCACCAGGCAGTCTGGCTTCAGAC

ATCTATGTTGCCCTTTTAAGTCAATTTGGCTGTTTAAGTGGAATGTTTGTGTCAGTCTGATAATTGTTAA

TTCATTTGATCCTCATACCAACCCTTACTTGCCTCCTTTTACAGATGGAAAAATCAAACTTGGACAGTAA

TTTCCACAAGGAAAAGCCCCACAGCTGGAAAGCGGTGGGAGCAGGAGGCCACACTGGGTACAGGAGTACC

CATCTCCCCACAGGAGCCCCACACTGTCTCACCCTTAGGAAGCCCAAATGCTCACTCCTTGAGTTTCTAA

TGCATTCTTTCTGATGTACAGGAAGAGGGGAAGGAAGGAAGAGCTTTTCCATTTGGTGCTCCAATGTCTC

CTGCTGGACCCATCTGCCTAGTGGAAGGCAGCAAAATTTCAAGAAACAGGTGAGGTTGAGCAGCTTGGTG

CAACCCCATGGGGCCTGGAGTTGGAGCTCAACAGCAATGGATTTCAGAGACCACCCTGAAACTCCCAGTA

AAAAAGACTTGGGAGACATGTTAATAAACTCAAGCATTTGATCGACCCA

>NR_104309.3 Homo sapiens unc-51 like kinase 3 (ULK3), transcript variant 6, non-coding RNA

GGAACCGCCGCCCGCAGCGAGGAAGCGCCCGCGCGGGCGCAGGCGGCCGGAATGGCGGGGCCCGGCTGGG

GTCCCCCGCGCCTGGACGGCTTCATCCTCACCGAGCGCCTGGGCAGCGGCACGTACGCCACGGTGTACAA

GGCCTACGCCAAGAAGGACACTCGTGAAGTGGTAGCCATAAAGTGTGTAGCCAAGAAAAGTCTGAACAAG

GCATCGGTGGAGAACCTCCTCACGGAGATTGAGATCCTCAAGGGCATTCGACATCCCCACATTGTGCAGC

TGAAAGACTTTCAGTGGGACAGTGACAATATCTACCTCATCATGGAGTTTTGCGCAGGGGGCGACCTGTC

TCGCTTCATCCATACCCGCAGGATTCTGCCTGAGAAGGTGGCGCGTGTCTTCATGCAGCAATTAGCTAGC

GCCCTGCAATTCCTGCATGAACGGAATATCTCTCACCTGGATCTGAAGCCACAGAACATTCTACTGAGCT

CCTTGGAGAAGCCCCACCTAAAACTGGCAGACTTTGGTTTCGCACAACACATGTCCCCGTGGGATGAGAA

GCACGTGCTCCGTGGCTCCCCCCTCTACATGGCCCCCGAGATGGTGTGCCAGCGGCAGTATGACGCCCGC

GTGGACCTCTGGTCCATGGGGGTCATCCTGTATGGTGAGACCTCTTTTCCCTGCTTCTCACCCTGAGGCC

TACAGTTTCCTCAGGCAAGGCCTCTCTGGGCCACTTCTGAAGGGTTCTGATGAAAACTGACTGCTGGCCC

AGGGCCCCTGCTGAGGGGCGTCAGGCCAGGCTTGACCCATTCCCTCCCAGCCTCCGCTCTGCCTCCCTTC

CACAGAAGCCCTCTTCGGGCAGCCCCCCTTTGCCTCCAGGTCGTTCTCGGAGCTGGAAGAGAAGATCCGT

AGCAACCGGGTCATCGAGCTCCCCTTGCGGCCCCTGCTCTCCCGAGACTGCCGGGACCTACTGCAGCGGC

TCCTGGAGCGGGACCCCAGCCGTCGCATCTCCTTCCAGGACTTTTTTGCGCACCCCTGGGTGGACCTGGA

GCACATGCCCAGTGGGGAGAGTCTGGGGCGAGCAACCGCCCTGGTGGTGCAGGCTGTGAAGAAAGACCAG

GAGGGGGATTCAGCAGCTGCCTTATCACTCTACTGCAAGGCTCTGGACTTCTTTGTACCTGCCCTGCACT

ATGAAGTGGATGCCCAGCGGAAGGAGGCAATTAAGGCAAAGGTGGGGCAGTACGTGTCCCGGGCTGAGGA

GCTCAAGGCCATCGTCTCCTCTTCCAATCAGGCCCTGCTGAGGCAGGGGACCTCTGCCCGAGACCTGCTC

AGAGAGATGGCCCGGGACAAGCCACGCCTCCTAGCTGCCCTGGAAGTGGCTTCAGCTGCCATGGCCAAGG

AGGAGGCCGCCGGCGGGGAGCAGGATGCCCTGGACCTGTACCAGCACAGCCTGGGGGAGCTACTGCTGTT

GCTGGCAGGCGGGAGCTGCTTCACACTGAGGTTCAGAACCTCATGGCCCGAGCTGAATACTTGAAGGAGC

AGGTCAAGATGAGGGAATCTCGCTGGGAAGCTGACACCCTGGACAAAGAGGGACTGTCGGAATCTGTTCG

TAGCTCTTGCACCCTTCAGTGACCCTAGAAGAATGATTGGACAGATGTGAGCCATCTGGAGCAGAGGGGC

ACTAACCCAGGCTGACGCCAAGAATGAAGTGGCCCACTGCAGCCCTGGCGAGCAGGCTTCTTGGATGGAC

AGTGCTGAGACCCCCATATCCCAGAGTCCCCAGCCTCCCTCAGGTTACTCTGCACCCCACAGATGGTTTG

ATGGCTGTGCTGTATACTGGAGGGGAGGGCAGGACTCTGGGAGAACAGCACTTCTTTCATGAGACCTTTG

TTACTCGGTGGTTACTGGGTCCTGTGCCTGTCCGTTTTGGGGCATGCAGCCCTCTATCATTTTTGGCTCC

GAGAAGAGGGCAAGGGGCCCCGCAGGGTACTTCTGTGCTTGCCCTCGCCCTGCCAGCAGGCAGCTGTGC

CCCTGGCCTGCCCTTCCCGGGACCCCTTATTCCAACTCAGCTCCTCTTTGCACTGGAATGGGGCACTCCA

ACACCCCTCAGGGACCACCCTCCCCACAGTATGCACTCAGCCCCACAGAACCCACCAGTCTTTCTGGGAA

CTCACACCTGCCCGCCATCTTGGTACTTTAGGTTAATCCCTCAAGCATGAAAGCTGGATCTTTTGGGGTT

TAAGAAGCCCAAGCCTTGTTCCTGCCCTGGCCTAGGGAGCACTCAGGAGGGTTCCTTGGTCCTCATCTCT

CCCACCTCCGTTCCCTCTGGGCCCCACACTAGCCACAGCGCGGGCCTTGTGCTGGAGTTTGAGCCTGGGA

CAGGGAGAGGGAGGCTTGGAGACAGTCTGACCCAGTGCCCTCTAGGCCACCCACTTCTAGGCCTGCCCTG

CCGCCGTGGAGCCCTGGGCAAGCTCTTTCCCCTTTCTGGGCCTGGGTCTCCCCATCTCTTCAATGGGGCT

GATACCTTCACAGCCCACAGCATGGGCACTTATGAGGACAAAGTGAATTTAACCTGGAAAAGAATGTATT

TGAGAGTTTCTTTTAAATAATCAGCGGGTGTTGGTGATTTGTAGCCCTTCTGCCCTTAAATGCTTCCTTG

GGCAAGAGCTGTCTGTCCTCCCTGCAGGAGGCTGAGTGTAAAGAGTATCATTCATTGTTTCTCTATTAAA

TTATTTTCTCTA

>NM_001272075.2 Homo sapiens ubiquitin specific peptidase 10 (USP10), transcript variant 1, mRNA

GAGTGTGTATGTGCGGGCGAGAAGATGGCGGCGGCGGGGGAAGCAGCGTGAGCAGCCGGAGGATCGCGGA

GTCCCAATGAAACGGGCAGCCATGGCCCTCCACAGCCCGCAGCTCCTGGGCCATGATCCCATTTTCATCA

GATGACTTGAGAACCCAGAAGCTCTACCAGCACTGCCATTCTGTCCCGTCTTGAAACATCATGCCCTGGT

TGCCCTCTCCTGGAATAGGGCAGTATATTTTTGGAGATTTTAGCCCTGATGAATTCAATCAATTCTTTGT

GACTCCTCGATCTTCAGTTGAGCTTCCTCCATACAGTGGAACAGTTCTGTGTGGCACACAGGCTGTGGAT

AAACTACCTGATGGACAAGAATATCAGAGAATTGAGTTTGGTGTCGATGAAGTCATTGAACCCAGTGACA

CTTTGCCGAGAACCCCCAGCTACAGTATTTCAAGCACACTGAACCCTCAGGCCCCTGAATTTATTCTCGG

TTGTACAGCTTCCAAAATAACCCCTGATGGTATCACTAAAGAAGCAAGCTATGGCTCCATCGACTGCCAG

TACCCAGGCTCTGCCCTCGCTTTGGATGGAAGTTCTAATGTGGAGGCGGAAGTTTTGGAAAATGATGGTG

TCTCAGGTGGTCTTGGACAAAGGGAGCGTAAAAAGAAGAAAAAGCGGCCACCTGGATATTACAGCTATTT

GAAAGATGGTGGCGATGATAGTATCTCCACAGAAGCCCTGGTCAATGGCCATGCCAATTCAGCAGTCCCG

AACAGTGTCAGTGCAGAGGATGCAGAATTTATGGGTGACATGCCCCCGTCAGTTACGCCCAGGACTTGTA

ACAGCCCCCAGAACTCCACAGACTCTGTCAGTGACATTGTGCCTGACAGTCCTTTCCCCGGAGCACTCGG

CAGTGACACCAGGACTGCAGGGCAGCCAGAGGGGGGCCCCGGGGCTGATTTTGGTCAGTCCTGCTTCCCT

GCAGAGGCTGGCAGAGACACCCTGTCAAGGACAGCTGGGGCTCAGCCCTGCGTTGGTACCGATACTACTG

AAAACCTTGGAGTTGCTAATGGACAAATACTTGAATCCTCGGGTGAGGGCACAGCTACCAACGGGGTGGA

GTTGCACACCACGGAAAGCATAGACTTGGACCCAACCAAACCCGAGAGTGCATCACCTCCTGCTGACGGC

ACGGGCTCTGCATCAGGCACCCTTCCTGTCAGCCAGCCCAAGTCCTGGGCCAGCCTCTTTCATGATTCTA

AGCCCTCTTCCTCCTCGCCGGTGGCCTATGTGGAAACTAAGTATTCCCCTCCCGCCATATCTCCCCTGGT

TTCTGAAAAGCAGGTTGAAGTCAAAGAAGGGCTTGTTCCGGTTTCAGAGGATCCTGTAGCCATAAAGATT

GCAGAGTTGCTGGAGAATGTAACCCTAATCCATAAACCAGTGTCGTTGCAACCCCGTGGGCTGATCAATA

AAGGGAACTGGTGCTACATTAATGCTACACTGCAGGCATTGGTTGCTTGCCCGCCGATGTACCACCTGAT

GAAGTTCATTCCTCTGTATTCCAAAGTGCAAAGGCCTTGTACGTCAACACCCATGATAGACAGCTTTGTT

CGGCTAATGAATGAGTTCACTAATATGCCAGTACCTCCAAAACCCCGACAAGCTCTTGGAGATAAAATCG

TGAGGGATATTCGCCCTGGAGCTGCCTTTGAGCCCACATATATTTACAGACTCCTGACAGTTAACAAGTC

AAGCCTGTCTGAAAAGGGTCGACAAGAAGATGCTGAGGAATACTTAGGCTTCATTCTAAATGGACTTCAT

GAGGAAATGTTGAACCTAAAGAAGCTTCTCTCACCAAGTAATGAAAAACTTACGATTTCCAACGGCCCCA

AAAACCACTCGGTCAATGAAGAAGAGCAGGAAGAACAAGGTGAAGGAAGCGAGGATGAATGGGAACAAGT

GGGCCCCCGGAACAAGACTTCCGTCACCCGCCAGGCGGATTTTGTTCAGACTCCAATCACCGGCATTTTT

GGTGGACACATCAGGTCTGTGGTTTACCAGCAGAGTTCAAAAGAATCTGCCACTTTGCAGCCATTTTTCA

CGTTGCAGTTGGATATCCAGTCAGACAAGATACGCACAGTCCAGGATGCACTGGAGAGCTTGGTGGCAAG

AGAATCTGTCCAAGGTTATACCACAAAAACCAAACAAGAGGTTGAGATAAGTCGAAGAGTGACTCTGGAA

AAACTCCCTCCTGTCCTCGTGCTGCACCTGAAACGATTCGTTTATGAGAAGACTGGTGGGTGCCAGAAGC

TTATCAAAAATATTGAATATCCTGTGGACTTGGAAATTAGTAAAGAACTGCTTTCTCCAGGGGTTAAAAA

TAAGAATTTTAAATGCCACCGAACCTATCGGCTCTTTGCAGTGGTCTACCATCACGGCAACAGTGCGACG

GGCGGCCATTACACTACAGACGTCTTCCAGATCGGTCTGAATGGCTGGCTGCGCATCGATGACCAGACAG

TCAAGGTGATCAACCAGTACCAGGTGGTGAAACCAACTGCTGAACGCACAGCCTACCTCCTGTATTACCG

CCGAGTGGACCTGCTGTAAACCCTGTGTGCGCTGTGTGTGCGCCCAGTGCCCGCTTCGTAGGACACCACC

TCACACTCACTTCCCGCCTCTCTTTAGTGGCTCTTTAGAGAGAAACTCTTTCTCCCTTTGCAAAAATGGG

CTAGAATGAAAAGGAGATGCCTTGGGGTTCGTGCACAACACAGCTTCTGTTGACTCTAACTTCCAAATCA

AAATCATTTGGTTGAAACAGACTGTTGCTTGATTTTAGAAAATACACAAAAACCCATATTTCTGAAATAA

TGCTGATTCCTGAGATAAGAAAGTGGATTTGATCCCCAGTCTCATTGCTTAGTAGAATAAATCCTGCACC

AGCAACAACACTTGTAAATTTGTGAAAATGAATTTTATCTTTCCTTAAAAAAGAAATTTTTTAATCCATC

ACACTTTTCTTCCCTACCCTTTAGTTTTTGATAAATGATAAAAATGAGCCAGTTATCAAAGAAGAACTAG

TTCTTACTTCAAAAGAAAAATAAACATAAAAAATAAGTTGCTGGTTCCTAACAGGAAAAATTTTAATAAT

TGTACTGAGAGAAACTGCTTACGTACACATTGCAGATCAAATATTTGGAGTTAAAATGTTAGTCTACATA

GATGGGTGATTGTAACTTTATTGCCATTAAAAGATTTCAAATTGCATTCATGCTTCTGTGTACACATAAT

GAAAAATGGGCAAATAATGAAGATCTCTCCTTCAGTCTGCTCTGTTTAATTCTGCTGTCTGCTCTTCTCT

AATGCTGCGTCCCTAATTGTACACAGTTTAGTGATATCTAGGAGTATAAAGTTGTCGCCCATCAATAAAA

ATCACAAAGTTGGTTTAAA

>NM_003369.4 Homo sapiens UV radiation resistance associated (UVRAG), transcript variant 2, mRNA

GGTAATATGGCTCTTCCTTAGCCAGCGGCGGCAACGGCGGCAGCGGCGGCAGCGGCGGCTACTGTCT

GGGCTGAGCAGTAGTGCCTCTCGGGTGGCGGGTTTCTAGGCTGCAGGGGCTTGGTAGGTGGTGGCAAGGG

GGCGGCGGCGGATGCCGGAAGAGTGCCCGCCCCGCCGCTTGGCGGCCCCTGGATCGAGATGAGCGCCTCC

GCGTCGGTCGGGGGCCCCGTCCCCCAGCCACCCCCGGGCCCGGCCGCTGCTCTGCCTCCCGGTTCTGCCG

CGCGGGCCCTGCATGTGGAGCTGCCGTCTCAGCAGCGGCGTCTTCGACATCTTCGGAACATTGCTGCCCG

GAACATTGTTAATAGAAATGGCCATCAGCTCCTTGATACCTACTTTACACTTCACTTGTGTAGTACTGAA

AAGATATATAAAGAATTTTATAGAAGTGAAGTGATTAAGAATTCCTTGAATCCCACGTGGCGAAGTCTCG

ATTTTGGAATTATGCCAGACCGTCTTGATACATCTGTGTCTTGTTTCGTGGTGAAGATATGGGGTGGAAA

GGAGAACATCTACCAGCTGTTGATTGAATGGAAAGTCTGTTTGGATGGGCTGAAATACTTGGGTCAGCAG

ATTCATGCCCGAAACCAAAATGAAATAATTTTTGGGCTGAATGATGGATACTATGGTGCTCCATTTGAAC

ATAAGGGTTATTCAAATGCTCAGAAGACTATTCTTCTGCAGGTGGATCAGAACTGTGTTCGCAATTCTTA

CGATGTCTTCTCTTTGCTACGGCTTCATAGAGCCCAGTGTGCAATTAAACAGACTCAGGTAACTGTTCAG

AAAATTGGAAAGGAAATTGAAGAAAAACTAAGACTCACATCTACAAGCAATGAACTGAAAAAAAAAAGTG

AATGCCTGCAGTTAAAAATTTTGGTGCTTCAGAATGAACTGGAACGGCAGAAGAAAGCTTTGGGACGGGA

GGTGGCATTACTGCATAAGCAACAAATTGCATTACAAGACAAAGGAAGTGCATTTTCAGCTGAGCACCTC

AAACTTCAACTCCAGAAGGAATCCCTAAATGAGCTGAGGAAGGAGTGCACTGCAAAAAGAGAACTCTTCT

TGAAGACTAATGCTCAGTTGACAATTCGTTGCAGGCAGTTACTCTCTGAGCTTTCCTACATTTACCCTAT

TGATTTGAATGAACATAAGGATTACTTTGTATGCGGTGTCAAGTTGCCTAATTCTGAGGACTTCCAAGCA

AAAGATGATGGAAGCATTGCTGTTGCCCTTGGTTATACTGCACATCTGGTCTCCATGATTTCCTTTTTCC

TACAAGTGCCCCTCAGATATCCTATAATTCATAAGGGGTCTAGATCAACAATCAAAGACAATATCAATGA

CAAACTGACGGAAAAGGAGAGAGAGTTTCCACTGTATCCAAAAGGAGGGGAGAAGTTGCAGTTTGATTAT

GGTGTCTATCTTCTGAACAAAAATATAGCACAGCTAAGATATCAACATGGACTAGGGACTCCAGACTTGC

GGCAAACCCTTCCCAACCTGAAAAACTTCATGGAGCATGGACTAATGGTCAGGTGTGACAGACATCACAC

CTCCAGTGCAATCCCTGTTCCTAAGAGACAAAGCTCCATATTTGGGGGTGCAGATGTAGGCTTCTCTGGG

GGGATCCCTTCACCAGACAAAGGACATCGAAAACGGGCCAGCTCTGAGAATGAGAGACTTCAGTACAAAA

CCCCTCCTCCCAGTTACAACTCAGCATTAGCCCAGCCTGTGACCACCGTCCCCTCCATGGGAGAGACCGA

GAGAAAGATAACATCTCTATCCTCCTCCTTGGATACCTCCTTGGACTTCTCCAAAGAAAACAAGAAAAAA

GGAGAGGATCTAGTTGGCAGCTTAAACGGAGGCCACGCGAATGTGCACCCTAGCCAAGAACAAGGAGAAG

CCCTCTCCGGGCACCGGGCCACAGTCAATGGCACTCTCCTACCCAGCGAGCAGGCCGGGTCCGCCAGTGT

CCAGCTTCCAGGCGAGTTCCACCCAGTCTCAGAAGCTGAGCTCTGCTGTACTGTGGAGCAAGCAGAAGAA

ATCATCGGGCTGGAAGCCACAGGTTTCGCCTCAGGTGATCAGCTAGAAGCATTTAACTGCATCCCAGTGG

ACAGTGCTGTGGCAGTAGAGTGTGACGAACAAGTTCTGGGAGAATTTGAAGAGTTCTCCCGAAGGATCTA

TGCACTGAATGAAAACGTATCCAGCTTCCGCCGGCCGCGCAGGAGTTCCGATAAGTGAAGTGAGCAGGTC

AACAGTAGGACTGGGGCAGAAGCTCTGCCTAAAATGAAGTGAAAGCTGCACTTAACCCTTTGTGATAATG

ATGACACAAAATGAATATTAATGGAGGATATTCCTCGGAAAAACAGACTTTGGGAATGAAGGAGGGACTC

AGGATCATTGTTATCAGTGGGCCAAAGTTAGATTTTGCTTTCAAGATTTGCTTTTCGGGCCTGATGATTT

TAAAGCAAAAATCACCCTCTAGTTGAAAGAGCTTACAGCTCGAGTCACCTTTTAGCTATTTGTCTGCTTT

TTATTTACCCTTGTATGTTATCCTCAGAGGGAAGATGATAATATATAAATAATATAATGAACACACCCTT

AGTTTCTCATAAGCATTTGCCCTCACCATGGTTTATAAAACTTTGGGAAAACGGAATATTCAGAAATAGG

TTTCCGCCATGTACTGAAAGGTCTGTGGCCATCTGTGAGGTAGATGAAGAAGCAGCATAGTGGTCTCCTT

ACATCTAGGCCTAACTGTCCCTCTTCCTGCCCCCGGGTACCACAGTCCACCTTTAGACCCTACTGTCGCC

CCATCTTCTCCGTGGATGGGCCATGCGTCCTGAAAACAGGACATCAGATTCACTGGTTCTGTAACCCAGT

AGCTGTGACGTTCCATCTCTTCTAACCAGCCATGGCCTTCCCCTCCTCTGCCATACCCTTAATGCGGCCC

TCAGATTAGATGAAAAACTTGCTCCTGGTGGATCCCAAGGGACCCTCAAGGACCTCGAGGTTACTGCAGT

CAGATGCCATCTCATCCCCTGTGGGGGCCAAAGTTTTTATGTGGGCAGATGCTGTGGTCAGGAACTAGGC

ATGCTTTCTGGCAATGCACTCACCAGACAAAAATCCCTTGATGTAAATCCCATGTTAATTTATTAAATTT

CAGTCAGAAGGTCAGCATTTACATGACAGAATGTATGTAGAGAGTTGGGGTGTCTGGTAGGCAAACTGCA

AGGCAGTTGAGATAGTTGGATTAAGAGGCTAGACGAGACATAGAATACTATTGGTATGTGTGCAATTTCA

TGAATATTAAATTATGTTTCGAAGTCCAGTTGTCATTCCCGCATTCAGATTTCATTTGCTGTTGCTTTAT

ACGTTACGTACCCAAGGACATTGCCTCAGGGTTGCAAACTCTTTAAAGGAAAATTTATCCATATATCCAT

GTATTATATAGAAGAATAAAAATTGAGTTTACTTCACCCGACCTGATTTTTTTTTACCAGCTTGGAAATT

CCTCTTGAACCTACGTCTCCATATGTCACATCATGACAGGACTAGCCTGAACAAAAGCCATGTCTATCTA

AGCGGAGGCTGTTGACTTCATTCAGTTTGCATATTGTATATAGCAACACAAACACTTGACAGGTATATAC

TCCAGTCGCCACATTTGTCCTGCATAACAGCTTCACTCACAGGCCTCACCGTCACTTTATTTTGTGTCCA

AGCATTCCTGGGCTCAAGTTTAATGTATAGCTACATTGTTGTTTTCCATGTAGAGAACTCACAGGATGAC

TACATCACAAATAAACCCAACTCTCAGGCAGTCGAAAGCTTTAACTGGATCTGCAGAAGCCCATCTTCCT

CCACATGAAGAAGTGGGGCTCCTTCACCTAGAGCAGGAGCTTCTCTGCAGTGTACTGTCTGTCGCTCATT

TTGGAGTCAGTGTGGGTGGAGACAGCATCGTGGATATGCACGTGCTGCGGGGCCCTCCAGGGAAGTAACA

TTTACCAAAAAAAAAGAAAAGTTCTGAAGGGCAGTGTTAGGATTGCAGAATGGAAGGTCAACCCCGTGGG

ACTTACGATTGCCCCAGGTGCGGGATGGACTTAAATGTCACACCAAACTCAAAGTAGGTACACTGGGTTG

CAGGGCATCCAGCCTGGCGCTGGCACCACCAAGGAGCAGGACTCCTCATGATTACAGTGTCCATCTCAGG

CCCACACAACCATGAGAGCCTGGACTCTGGGGTCTACCCGTCAGTGCCCCCCACCGCTGTGCAGACTCCC

TGGTTGATCCTGGGCTTGTGGCTTTTCACCGCACGGCGGGGAGCCCTGCTCTTGAATGTCATCGGGCTGC

TCAGAGCTGATTGCTAGGTGCCTACACATTTGCCTCGACCCACACAGCCCCGTGGTGATGCCTCAAAACA
CCACTTAGGTTTGGGTTCGTTTAGTTCGAGTTTGGGGTTTTCATTTGAACTTGTTTGATGTCTGCAGTTT
CTGCCATGACCTGGGTAGAACCTATGGGATTACCCGTCTCCTGGAATAACTGTTTGACGTTTTCCAAAGT
TGTAGAGTTAGTATGGCCTCTGTTTAAAGTGGCTGGGGCCAAAATAAGGGATGAGTTATTATCTCCTACT
AACCTTTTAGTTTTGTAAATCACTAAGAAAATTGTTTGCTTGGAAGATATAAGTTGAATTGGAAACTCAT
TACTATCTATTTCTGAACTGCTAAGAACCCTTTCAGTTTTCTTACAGCTGAGACACTTTTAAACAGCGGG
CAAATGTTATCAAATGAATATTTGATTGTGTTTTTTCTCTTCACTTCCCCTTAACCACTTTAGAAATTCC
AGAGATTTTTTTCCCCTCAGAATATTAGTTTTGGAAGATTGTGCCCAGCTATATATTTTTTAGCAGTTCT
AATGGTGCCCATTTATCCTGACCTAACCAGTTATTTAAATAATTTTTTAAACCACCACGAATAATAAATG
GCATGTGAAACTGATCTGTTGGTAACTGGAAGAAAACTCAGCATCTGTATTTATACAATAAAATTGATTA
GTATTTA

>NM_004781.4 Homo sapiens vesicle associated membrane protein 3 (VAMP3), mRNA
GTCCCACCCATCTCCCTGGCCTCCGGTCCCAACTTCGCTTCTCTGCTGACCCTCTCTCGTCGCCGCTGCC
GCCGCCGCAGCTGCCAAAATGTCTACAGGTCCAACTGCTGCCACTGGCAGTAATCGAAGACTTCAGCAGA
CACAAAATCAAGTAGATGAGGTGGTGGACATAATGCGAGTTAACGTGGACAAGGTTCTGGAAAGAGACCA
GAAGCTCTCTGAGTTAGACGACCGTGCAGACGCACTGCAGGCAGGCGCTTCTCAATTTGAAACGAGCGCA
GCCAAGTTGAAGAGGAAATATTGGTGGAAGAATTGCAAGATGTGGGCAATCGGGATTACTGTTCTGGTTA
TCTTCATCATCATCATCATCGTGTGGGTTGTCTCTTCATGAAGAACCAGCGGAACTCAAAACTGCTGTTC
AAGAAACCTCTTCAAGACTTTTGACTTAGAACCTGCTATATTATCAAGCTTACCTACTGTTATCTCTAAA
ATTTTTTTTGTGTTAATGTAAAGTTGAATTTCTAGGAAACGTGCCTTTGTTTTTTAATATGCACTCCAAA
TTAGAAGGCCGGCCCCGTCCACATTTTGCACAGTGCCTTTACAGATTTACGTATGGGCTGATGAAGAGGC
CTTCTTAAGTTCCAGAGTGCTATAATCTAGATGTAATGTTGTCACTAATTAATTGCCATTACTCCCAGTT

AGTTACCCTTGTCATTTGGCATTATTTTCAGAACCACATTTTAAACCTTTGGGTAATCAGATTTCCAACT

TATGCCTTCCAGAAAAAAACACTACTGCCTAACACAAATCTGTGATAACAACAGGCTGTGCCTTATTTTG

ATAATTTTCTGATTCCCTAGAAGAGAACCCTCTACTTTTTGTAAGCACTACTGACTCTCGCTGTATTTAA

GATGCTGGTGAAGAGCTTTTGCTCTTGCATTAGATTTGAAGATGTTTACATTGTTGTTATTGTTATGTAT

CACTTGCTAAAAATATTGTTTTAATCAGAGATAACCTCTTTAAAAAAATTTTTAAAGAACTATGGCTATG

ACCAAAGCTTCTATTTTGCCAAAAAGTTAAATACCGATAAAATGGCCTTAAGTGTATTCCTGACAGTTAA

ATTCAGAAACGTGCCAAATGGAACTCAAGGTGCCCCTTCAGAATTAAAATCATTACCTTGTGTGTGAACC

TTCTACATCTTCATAGGCCTTTCTTCCTTTTGAAAGGCTGTAGACAGTGTGGCTCCCCTTCTGATTCAGT

ATTTTGCATGGGGGTTAGAGAAGGTTTGAGGTAGACTCTGACCGTCTCATAAAAGAGTTCTACCCAGCAG

TTGGCAGATTATCAGCTGTGGACTCCAGCATGTTTCTGATAATTATGCAAGCAACAATTCTGTAGCCTCA

AGTAAGACCACCTGTGAACTTGATCATTATCTGGCCCAAATATGAAGATAAACTATAACTTTGGAGTTTG

TTTCCTATTTGTATTCACATTCTGCTTCCTAAATCAGTTTTCTAAATTATGCCTGCAATTAGGCATTGGT

CAGGGGTGAATGGCTCTTTTCACAGAGAGTAGCCAACCAGAGACCTTTGCTTTGATATCATCAACTGCAG

AGAATGCTGTTGATGGGAATGCTGGAAGCAGAAACTTTGTCATCGGAAAAACTTTTCTTGTATGCATGAG

ACTCAACATCAGGATCCACAGCTTAAAGATGGGAATTCAGGTATGAAAGAAAACAGGCAAGGAGGCACTG

AGGGAGAAAGACACAGACTTTATCGCTCTGTGGCTCATTGTTACTGGAATATTCTAAAACTCTTGTTCAC

ATGCTATTATGACTTATAAAGCAGCAACAGCTGAGGCGCACCAGGACACAGCTTCCATTTCTTTAACGTC

TGTTCCCTTAACATCGCTGAAATGATTTACTGTTGAAGAGATGCCTTGCGGTGTGGCCAGCTGTGAGGAG

AAAGCAGCTGGCAGTGTTAGGACATTAGTCCACCTTCAGCGCAGGGTCTCTGGCCGGGTCTGACTCAGAA

ACCTTGGTACTCGCCCCTTGGCCACAGTGCCCAGACCCATGTAACCCACTGGCTCCTGCATTAACCCAGA

AATACCTCGCTTCTATCTGTGCACTTAGCTGGGAACTTACCCACTGTAATCACCTAAATAAAGTGTTTAT

AAACATGA

>NM_005638.6 Homo sapiens vesicle associated membrane protein 7 (VAMP7), transcript variant 1, mRNA

GGCGTCCGGTCCCAGCCTCCTCTGGGAGCGGGCAGTTGGCGACCCTGCACTGACCCGCGTCCCTCCGTCC

CGAGCCCGCGCGCCCTCAGAGGGTGCCCGGACAGACTGAAGCCATGGCGATTCTTTTTGCTGTTGTTGCC

AGGGGGACCACTATCCTTGCCAAACATGCTTGGTGTGGAGGAAACTTCCTGGAGGTGACAGAGCAGATTC

TGGCTAAGATACCTTCTGAAAATAACAAACTAACGTACTCACATGGCAATTATTTGTTTCATTACATCTG

CCAAGACAGGATTGTATATCTTTGTATCACTGATGATGATTTTGAACGTTCCCGAGCCTTTAATTTTCTG

AATGAGATAAAGAAGAGGTTCCAGACTACTTACGGTTCAAGAGCACAGACAGCACTTCCATATGCCATGA

ATAGCGAGTTCTCAAGTGTCTTAGCTGCACAGCTGAAGCATCACTCTGAGAATAAGGGCCTAGACAAAGT

GATGGAGACTCAAGCCCAAGTGGATGAACTGAAAGGAATCATGGTCAGAAACATAGATCTGGTAGCTCAG

CGAGGAGAAAGATTGGAATTATTGATTGACAAAACAGAAAATCTTGTGGATTCTTCTGTCACCTTCAAAA

CTACCAGCAGAAATCTTGCTCGAGCCATGTGTATGAAGAACCTCAAGCTCACTATTATCATCATCATCGT

ATCAATTGTGTTCATCTATATCATTGTTTCACCTCTCTGTGGTGGATTTACATGGCCAAGCTGTGTGAAG

AAATAGGAAAGAAGAAGTTACCATTAACCAAGGATATGAGAGAACAAGGAGTTAAAAGCAATCCATGTGA

CTCAAGCCTTTCACATACTGACAGATGGTATCTGCCAGTCTCTTCAACCCTCTTCTCACTTTTTAAAATC

TTGTTCCATGCCTCCAGGTTTATCTTTGTCTTATCTACCAGTTTATTCCTGTGAACTTCAGATTGAACCA

TTCATTGCAGCAGTAGCCTTAAAAAGGCTTTTGTTTATTTCTTTGGTTTGTTAACTAGTGTCATCTATTT

AGAGAAACATTTTTGTTTTTAATTGCTCAAAGCTGTCGCCGCTAGTCTTATGAGCTATCTACTAAAACTA

TGGAGAAACTTTGTATGTGCACACAAAAGTATTCAAGAGACAGTATTGCTAACATCTCATCTTAATGTCT

TTTGTTATTGAGAAGTTTTAGGTGCTTCAAAACAATATAAATGGATAATAGTTGTTATTTGGGGAATTGT

AATGATGTTGGTGCTGCTTCCTTCTAAGAGCTCAGACAAGTAAAGTATGAAACATTCTTATTTCAGTTAG

ATGGGGAACATTTTGCTAGCCCATTAGAAGCACACAGAATTATCCTTGTCCTCCTAATATTGACTTTCAG

GAATAAAGTTCAGTGTGCTGATCATTCACAATACAGTGGATAGCTTGATATCTTCTGTTTTCCCATTGCA

GTTGATTTGAGAAGATGAAGGTTTAAATATTGTTGAAAGTTGCAGTTTTTTAAATGTGTTCCTTTTTCTT

CTGTGAATATTTAGGGCAATCGTGTCGCTAATAGAATATGTAGTAGAGGGGGTGGGGAGGTAAATTCCTC

TGACTTGCCAAAGAAAAAGAAGGGAACCACAGTGGATATGCTAGCATTTTAGCTGTGCAAAGGGAGGTAG

TGTGGGAAAAGTGTTTCCATTCTGGGAAAAGCCCAAACCGAATACGGTCAGCAGTCAACTCCAGGGTTTG

GGCTTGATTCCTGTTGAATAATAGTTTTGAGCATTCTTTGTGGTTAAATAAATTCTTAAATCTGCCTAGT

TTTGATGAATTCTTTTGTGAAACTTGAAAGAGAATAGACAGTATGACATATAGAATTAATACAAAACAGT

TTAACAACCATTTAACTGCAGTGTAAGAAAATTGGACTGTAATCATATCGCTACTGGCATCTGTTATCTA

GTATGCATTTCTGGTGTGTATCTGAAAGGAAGACATTTTCTACCCTAGATCCAATTGCATTTATTTATCA

ATAAGTGCCATTAAATTGAAATTATATTACATTTTACACTTTCTCAATGAATGAACAAATTAGTCTGTAG

AATCTAGCCACCTGTTTAGCCTAGTCATGTGCCTTGAACATATATGTGTCCCATAATCTGGCTCATGGTA

CCTGTTCTTCTATCCAAACCTTTCAATTCATGCTACCTGATTCATTTATTTGACATAGATCTTAGGCCCA

CTTGAACTCTTTTCTTGTTTATCTAGCATAGCACAAACGTTTTTCCAGTCTTCTTTATCAACACTAATGC

CTCTTAATTGCATCAGTATTTCCTATTGGAAAATACATCTGTTCCAGAAAAACATTTGGCATTCCTGAAT

AATTTCCAAATGTTTTTAATCCAAAGAAAAAGGTTTAAAGCTTATTTCCCTTTCTTATACACACCTGAAT

AAAATTGATGTGCATGTTTTAGGGATCAATTACCTAACTGTTCCTTGGTCTATTTATGTATAAGAATGCT

TTTTAAAGCACATGTCTCATTTTAAATGACGCACAAACTGAAGATGTTAATAAAATTTAAGAGTAATACA

ATGA

>NM_001025366.3 Homo sapiens vascular endothelial growth factor A (VEGFA), transcript variant 1, mRNA

GCGGAGGCTTGGGGCAGCCGGGTAGCTCGGAGGTCGTGGCGCTGGGGGCTAGCACCAGCGCTCTGTCGGG

AGGCGCAGCGGTTAGGTGGACCGGTCAGCGGACTCACCGGCCAGGGCGCTCGGTGCTGGAATTTGATATT

CATTGATCCGGGTTTTATCCCTCTTCTTTTTTCTTAAACATTTTTTTTTAAAACTGTATTGTTTCTCGTT

TTAATTTATTTTTGCTTGCCATTCCCCACTTGAATCGGGCCGACGGCTTGGGGAGATTGCTCTACTTCCC

CAAATCACTGTGGATTTTGGAAACCAGCAGAAAGAGGAAAGAGGTAGCAAGAGCTCCAGAGAGAAGTCGA

GGAAGAGAGAGACGGGGTCAGAGAGAGCGCGCGGGCGTGCGAGCAGCGAAAGCGACAGGGGCAAAGTGAG

TGACCTGCTTTTGGGGGTGACCGCCGGAGCGCGGCGTGAGCCCTCCCCCTTGGGATCCCGCAGCTGACCA

GTCGCGCTGACGGACAGACAGACAGACACCGCCCCCAGCCCCAGCTACCACCTCCTCCCCGGCCGGCGGC

GGACAGTGGACGCGGCGGCGAGCCGCGGGCAGGGGCCGGAGCCCGCGCCCGGAGGCGGGGTGGAGGGGGT

CGGGGCTCGCGGCGTCGCACTGAAACTTTTCGTCCAACTTCTGGGCTGTTCTCGCTTCGGAGGAGCCGTG

GTCCGCGCGGGGGAAGCCGAGCCGAGCGGAGCCGCGAGAAGTGCTAGCTCGGGCCGGGAGGAGCCGCAGC

CGGAGGAGGGGGAGGAGGAAGAAGAGAAGGAAGAGGAGAGGGGGCCGCAGTGGCGACTCGGCGCTCGGAA

GCCGGGCTCATGGACGGGTGAGGCGGCGGTGTGCGCAGACAGTGCTCCAGCCGCGCGCGCTCCCCAGGCC

CTGGCCCGGGCCTCGGGCCGGGGAGGAAGAGTAGCTCGCCGAGGCGCCGAGGAGAGCGGGCCGCCCCACA

GCCCGAGCCGGAGAGGGAGCGCGAGCCGCGCCGGCCCCGGTCGGGCCTCCGAAACCATGAACTTTCTGCT

GTCTTGGGTGCATTGGAGCCTTGCCTTGCTGCTCTACCTCCACCATGCCAAGTGGTCCCAGGCTGCACCC

ATGGCAGAAGGAGGAGGGCAGAATCATCACGAAGTGGTGAAGTTCATGGATGTCTATCAGCGCAGCTACT

GCCATCCAATCGAGACCCTGGTGGACATCTTCCAGGAGTACCCTGATGAGATCGAGTACATCTTCAAGCC

ATCCTGTGTGCCCCTGATGCGATGCGGGGGCTGCTGCAATGACGAGGGCCTGGAGTGTGTGCCCACTGAG

GAGTCCAACATCACCATGCAGATTATGCGGATCAAACCTCACCAAGGCCAGCACATAGGAGAGATGAGCT

TCCTACAGCACAACAAATGTGAATGCAGACCAAAGAAAGATAGAGCAAGACAAGAAAAAAAATCAGTTCG

AGGAAAGGGAAAGGGGCAAAAACGAAAGCGCAAGAAATCCCGGTATAAGTCCTGGAGCGTGTACGTTGGT

GCCCGCTGCTGTCTAATGCCCTGGAGCCTCCCTGGCCCCCATCCCTGTGGGCCTTGCTCAGAGCGGAGAA

AGCATTTGTTTGTACAAGATCCGCAGACGTGTAAATGTTCCTGCAAAAACACAGACTCGCGTTGCAAGGC

GAGGCAGCTTGAGTTAAACGAACGTACTTGCAGATGTGACAAGCCGAGGCGGTGAGCCGGGCAGGAGGAA

GGAGCCTCCCTCAGGGTTTCGGGAACCAGATCTCTCACCAGGAAAGACTGATACAGAACGATCGATACAG

AAACCACGCTGCCGCCACCACACCATCACCATCGACAGAACAGTCCTTAATCCAGAAACCTGAAATGAAG

GAAGAGGAGACTCTGCGCAGAGCACTTTGGGTCCGGAGGGCGAGACTCCGGCGGAAGCATTCCCGGGCGG

GTGACCCAGCACGGTCCCTCTTGGAATTGGATTCGCCATTTTATTTTTCTTGCTGCTAAATCACCGAGCC

CGGAAGATTAGAGAGTTTTATTTCTGGGATTCCTGTAGACACACCCACCCACATACATACATTTATATAT

ATATATATTATATATATATAAAAATAAATATCTCTATTTTATATATATAAAATATATATATTCTTTTTTT

AAATTAACAGTGCTAATGTTATTGGTGTCTTCACTGGATGTATTTGACTGCTGTGGACTTGAGTTGGGAG

GGGAATGTTCCCACTCAGATCCTGACAGGGAAGAGGAGGAGATGAGAGACTCTGGCATGATCTTTTTTTT

GTCCCACTTGGTGGGGCCAGGGTCCTCTCCCCTGCCCAGGAATGTGCAAGGCCAGGGCATGGGGGCAAAT

ATGACCCAGTTTTGGGAACACCGACAAACCCAGCCCTGGCGCTGAGCCTCTCTACCCCAGGTCAGACGGA

CAGAAAGACAGATCACAGGTACAGGGATGAGGACACCGGCTCTGACCAGGAGTTTGGGGAGCTTCAGGAC

ATTGCTGTGCTTTGGGGATTCCCTCCACATGCTGCACGCGCATCTCGCCCCCAGGGGCACTGCCTGGAAG

ATTCAGGAGCCTGGGCGGCCTTCGCTTACTCTCACCTGCTTCTGAGTTGCCCAGGAGACCACTGGCAGAT

GTCCCGGCGAAGAGAAGAGACACATTGTTGGAAGAAGCAGCCCATGACAGCTCCCCTTCCTGGGACTCGC

CCTCATCCTCTTCCTGCTCCCCTTCCTGGGGTGCAGCCTAAAAGGACCTATGTCCTCACACCATTGAAAC

CACTAGTTCTGTCCCCCCAGGAGACCTGGTTGTGTGTGTGTGAGTGGTTGACCTTCCTCCATCCCCTGGT

CCTTCCCTTCCCTTCCCGAGGCACAGAGAGACAGGGCAGGATCCACGTGCCCATTGTGGAGGCAGAGAAA

AGAGAAAGTGTTTTATATACGGTACTTATTTAATATCCCTTTTTAATTAGAAATTAAAACAGTTAATTTA

ATTAAAGAGTAGGGTTTTTTTTCAGTATTCTTGGTTAATATTTAATTTCAACTATTTATGAGATGTATCT

TTTGCTCTCTCTTGCTCTCTTATTTGTACCGGTTTTTGTATATAAAATTCATGTTTCCAATCTCTCTCTC

CCTGATCGGTGACAGTCACTAGCTTATCTTGAACAGATATTTAATTTTGCTAACACTCAGCTCTGCCCTC

CCCGATCCCCTGGCTCCCCAGCACACATTCCTTTGAAATAAGGTTTCAATATACATCTACATACTATATA

TATATTTGGCAACTTGTATTTGTGTGTATATATATATATATATGTTTATGTATATATGTGATTCTGATAA

AATAGACATTGCTATTCTGTTTTTTATATGTAAAAACAAAACAAGAAAAAATAGAGAATTCTACATACTA

AATCTCTCTCCTTTTTTAATTTTAATATTTGTTATCATTTATTTATTGGTGCTACTGTTTATCCGTAATA

ATTGTGGGGAAAAGATATTAACATCACGTCTTTGTCTCTAGTGCAGTTTTTCGAGATATTCCGTAGTACA

TATTTATTTTTAAACAACGACAAAGAAATACAGATATATCTTAAAAAAAAAAAAGCATTTTGTATTAAAG

AATTTAATTCTGATCTCAAA

>NM_030938.5 Homo sapiens vacuole membrane protein 1 (VMP1), transcript variant 1, mRNA

GGGTTCCGGTTGTCTGGAGCCCAGCGGCGGGTGTGAGAGTCCGTAAGGAGCAGCTTCCAGGATCCTGAGA

TCCGGAGCAGCCGGGGTCGGAGCGGCTCCTCAAGAGTTACTGATCTATGAAATGGCAGAGAATGGAAAAA

ATTGTGACCAGAGACGTGTAGCAATGAACAAGGAACATCATAATGGAAATTTCACAGACCCCTCTTCAGT

GAATGAAAAGAAGAGGAGGGAGCGGGAAGAAAGGCAGAATATTGTCCTGTGGAGACAGCCGCTCATTACC

TTGCAGTATTTTTCTCTGGAAATCCTTGTAATCTTGAAGGAATGGACCTCAAAATTATGGCATCGTCAAA

GCATTGTGGTGTCTTTTTTACTGCTGCTTGCTGTGCTTATAGCTACGTATTATGTTGAAGGAGTGCATCA

ACAGTATGTGCAACGTATAGAGAAACAGTTTCTTTTGTATGCCTACTGGATAGGCTTAGGAATTTTGTCT

TCTGTTGGGCTTGGAACAGGGCTGCACACCTTTCTGCTTTATCTGGGTCCACATATAGCCTCAGTTACAT

TAGCTGCTTATGAATGCAATTCAGTTAATTTTCCCGAACCACCCTATCCTGATCAGATTATTTGTCCAGA

TGAAGAGGGCACTGAAGGAACCATTTCTTTGTGGAGTATCATCTCAAAAGTTAGGATTGAAGCCTGCATG

TGGGGTATCGGTACAGCAATCGGAGAGCTGCCTCCATATTTCATGGCCAGAGCAGCTCGCCTCTCAGGTG

CTGAACCAGATGATGAAGAGTATCAGGAATTTGAAGAGATGCTGGAACATGCAGAGTCTGCACAAGACTT

TGCCTCCCGGGCCAAACTGGCAGTTCAAAAACTAGTACAGAAAGTTGGATTTTTTGGAATTTTGGCCTGT

GCTTCAATTCCAAATCCTTTATTTGATCTGGCTGGAATAACGTGTGGACACTTTCTGGTACCTTTTTGGA

CCTTCTTTGGTGCAACCCTAATTGGAAAAGCAATAATAAAAATGCATATCCAGAAAATTTTTGTTATAAT

AACATTCAGCAAGCACATAGTGGAGCAAATGGTGGCTTTCATTGGTGCTGTCCCCGGCATAGGTCCATCT

CTGCAGAAGCCATTTCAGGAGTACCTGGAGGCTCAACGGCAGAAGCTTCACCACAAAAGCGAAATGGGCA

CACCACAGGGAGAAAACTGGTTGTCCTGGATGTTTGAAAAGTTGGTCGTTGTCATGGTGTGTTACTTCAT

CCTATCTATCATTAACTCCATGGCACAAAGTTATGCCAAACGAATCCAGCAGCGGTTGAACTCAGAGGAG

AAAACTAAATAAGTAGAGAAAGTTTTAAACTGCAGAAATTGGAGTGGATGGGTTCTGCCTTAAATTGGGA

GGACTCCAAGCCGGGAAGGAAAATTCCCTTTTCCAACCTGTATCAATTTTTACAACTTTTTTCCTGAAAG

CAGTTTAGTCCATACTTTGCACTGACATACTTTTTCCTTCTGTGCTAAGGTAAGGTATCCACCCTCGATG

CAATCCACCTTGTGTTTTCTTAGGGTGGAATGTGATGTTCAGCAGCAAACTTGCAACAGACTGGCCTTCT

GTTTGTTACTTTCAAAAGGCCCACATGATACAATTAGAGAATTCCCACCGCACAAAAAAAGTTCCTAAGT

ATGTTAAATATGTCAAGCTTTTTAGGCTTGTCACAAATGATTGCTTTGTTTTCCTAAGTCATCAAAATGT

ATATAAATTATCTAGATTGGATAACAGTCTTGCATGTTTATCATGTTACAATTTAATATTCCATCCTGCC

CAACCCTTCCTCTCCCATCCTCAAAAAAGGGCCATTTTATGATGCATTGCACACCCTCTGGGGAAATTGA

TCTTTAAATTTTGAGACAGTATAAGGAAAATCTGGTTGGTGTCTTACAAGTGAGCTGACACCATTTTTTA

TTCTGTGTATTTAGAATGAAGTCTTGAAAAAAACTTTATAAAGACATCTTTAATCATTCCAAAATTGTGT

CCGTTTTCTTGAGCGTTTTGATTTTTTACTTTTAGCTTATACCAGCTGAATGGCAGCCTTGCCTAATCCA

CCTACAACAAGAATTTCTTAAGCTTTCTTTTATTTGCATGAGAGAGCCACTACCAAGGCATGTTTTGTTA

TGCTGAAACTGGGCTGCTGCATACTGCTAAATGGCACCTCTGGGATTGGCCTACCTGGGGATTTCTTGGT

TTGTGAAAACAGGAGAGGAGAAATATCTCATACAAGTGAAAGGATACTGGAGAGAGAAATTACCCATTTC

TAAAAAAAAACCACACTCTGTCGTATCTGTGTTAATGTTTTCTAGCATGTACTCTGGTTTCAACAGACAC

AAATTTATATGTTAACCCAGTTTTCTTGCCGTTCTGTAAGTGTTTTATTCTTAGTGTGATTTTTTTCCAT

TGGGATGTTTTTGATTGAACTTGTTCATTTTGTTTTGCTTGGGAGGAAAATAAACAATTTTACTTTTTTC

CTTTAGGAGCATTATGAGCATTATGTCAGAATAGAATAGAATTGGGGTTCGATCTTAACAGGCCAGAAAT

GCCTGGGTTTTTTTGGTTTGTTTTTGTTTTTGTTTTTTTATCAAATCCTGCCTGACTGTCTGCTTGTTTT

GCCTACCATCGTGACATCTCCATGGCTGTACCACCTTGTCGGGTAGCTTATCAGACTGATGTTGACTGTT

GAATCTCATGGCAACACCAGTCGATGGGCTGTCTGACATTTTGGTATCTTTCATCTGACCATCCATATCC

AATGTTCTCATTTAAACATTACCCAGCATCATTGTTTATAATCAGAAACTCTGGTCCTTCTGTCTGGTGG

CACTTAGAGTCTTTTGTGCCATAATGCAGCAGTATGGAGGGAGGATTTTATGGAGAAATGGGGATAGTCT

TCATGACCACAAATAAATAAAGGAAAACTAAGCTGCATTGTGGGTTTTGAAAAGGTTATTATACTTCTTA

ACAATTCTTTTTTTCAGGGACTTTTCTAGCTGTATGACTGTTACTTGACCTTCTTTGAAAAGCATTCCCA

AAATGCTCTATTTTAGATAGATTAACATTAACCAACATAATTTTTTTTAGATCGAGTCAGCATAAATTTC

TAAGTCAGCCTCTAGTCGTGGTTCATCTCTTTCACCTGCATTTTATTTGGTGTTTGTCTGAAGAAAGGAA

AGAGGAAAGCAAATACGAATTGTACTATTTGTACCAAATCTTTGGGATTCATTGGCAAATAATTTCAGTG

TGGTGTATTATTAAATAGAAAAAAAAAATTTTGTTTCCTAGGTTGAAGGTCTAATTGATACGTTTGACTT

ATGATGACCATTTATGCACTTTCAAATGAATTTGCTTTCAAAATAAATGAAGAGCAGCTGTCCTTCTTTC

CTCTTTTAAGTGTTCAGCTGTGGCATGCTCAGAGGTTCCTGCTGGATTCCAGCTGGAGCGGTGTGATACC

CTTCTTTTTCAGCTGTTCGTGCCTTCCTTTCTTGTATCCACCAAAGTGGAGACAAATACATGATCTCAAA

GATACACAGTACCTACTTAATTCCAGCTGATGGGAGACCAAAGAATTTGCAAGTGGATGGTTTGGTATCA

CTGTAAATAAAAAGAGGGCCTGGGAATTCTTGCGATTCCATCTCTA

>NM_014991.6 Homo sapiens WD repeat and FYVE domain containing 3 (WDFY3), mRNA

AGACAGCCAGCGGGAGGTGGAGAAAGCAGGAGGAGGAGGAGGATTAAAGATGGCCACCAACAGCTGCGGG

AAACGGCAACAACCCCTCACTTTCCGGGATGGTCCCTGCGGGTCGGCCCGGCCTTGATGGAGAGAAGAAA

CCCGAGGAGCGCCGAGGCTGAGGCGGCGGCGGCGGGGACCCAGCGAGGACGAGGACGCGGCGGAGCAGGG

ACGGGGGCAGGAGAAGGGAAAGGCGGCGGCGTCGCTGCCCCTGCTGCCTAGCACCGCTGCCTGGCCCGGC

GGACCGGTTCCCATACCTCGCGGCCGCAGAATCGAGCTCGGGCCCCGGCCCCCGGCCCGCGGCGCGGGGC

TCCCGGGCCCCGCCGCGGACGTCGCGCCGGTCGCCCCTTCCCCGTAGCCCGTGCGCCCTCGGCGCGGAGC

CCCGGCCCGCCGCGGTCCCGTCTCCTGGGCCTGTCCCGCCCGCGCCCTCCGCCGGCCCTCAGGTATAATA

CTTCTCCACGTCTGCTTCAGGAAGAAAGTGCCTGCCATTCTTATCATTTCTAAGCAGGTTCATGCCAGCC

CAGAACAGAGAATCAGCTGGAGCCCAGATTTCAAGTTTTGAGTAAAATACCTTCAAGCGAATGGGCCCTA

TTGTGCTCACACATTCAGAACCTGTTACCCAAGGAATTCCCTAAAGAATTAGAAGTGCGTCTCACCAACC

AGCCAAGATGAACATGGTGAAGAGGATCATGGGGCGGCCGAGGCAGGAGGAGTGCAGCCCACAAGACAAC

GCCTTAGGACTGATGCACCTCCGCCGGCTCTTCACGGAGTTGTGCCATCCTCCCCGGCACATGACTCAGA

AGGAACAAGAAGAGAAACTGTATATGATGCTGCCAGTGTTTAACAGGGTTTTTGGAAATGCTCCGCCGAA

TACAATGACAGAAAAATTTTCTGATCTTCTGCAGTTCACAACACAAGTCTCACGACTAATGGTGACAGAA

ATTCGAAGGAGAGCATCAAACAAATCCACAGAGGCTGCAAGTCGGGCCATAGTTCAGTTCCTAGAGATTA

ATCAGAGTGAAGAAGCCAGTAGAGGCTGGATGCTTCTAACGACAATTAATTTGTTAGCTTCCTCTGGTCA

GAAAACCGTGGACTGCATGACAACAATGTCAGTGCCTTCCACCCTGGTTAAATGTTTATATCTGTTTTTT

GACCTTCCACATGTGCCTGAGGCAGTTGGAGGTGCACAGAATGAGCTACCTCTAGCAGAACGTCGAGGAC

TACTCCAGAAAGTTTTTGTACAGATCTTAGTGAAACTGTGCAGTTTTGTTTCCCCTGCGGAGGAGCTGGC

TCAGAAAGATGATCTCCAGCTTCTATTCAGTGCAATAACCTCTTGGTGCCCTCCCTATAACCTGCCTTGG

AGAAAGAGTGCTGGAGAAGTCCTCATGACCATATCTCGTCATGGTCTTAGTGTCAATGTAGTGAAGTATA

TTCATGAGAAAGAGTGTTTATCTACATGTGTTCAGAATATGCAGCAATCAGATGACCTGTCTCCCCTAGA

AATTGTCGAAATGTTTGCTGGGCTTTCTTGTTTCCTCAAAGATTCCAGCGATGTTTCCCAAACACTTCTG

GATGATTTTCGGATATGGCAAGGATATAATTTTCTTTGTGATCTCTTGCTTAGATTGGAACAAGCAAAAG

AGGCAGAATCCAAAGATGCCTTGAAAGATCTGGTTAATCTGATAACTTCCCTAACAACATATGGTGTCAG

TGAACTAAAACCAGCTGGTATTACCACAGGGGCACCCTTTTTATTGCCTGGATTTGCAGTACCTCAGCCT

GCAGGCAAAGGTCACAGTGTGAGAAACGTCCAGGCCTTTGCAGTTCTTCAGAATGCATTTTTAAAAGCAA

AAACCAGCTTCCTTGCCCAAATCATCCTTGATGCTATCACAAATATTTACATGGCTGACAATGCCAATTA

CTTCATCCTAGAGTCACAGCACACATTGTCACAGTTTGCAGAGAAGATTTCTAAACTCCCAGAAGTACAA

AACAAATACTTTGAGATGCTGGAGTTTGTTGTTTTTAGCTTAAATTATATACCTTGTAAAGAACTTATTA

GTGTCAGTATCCTCTTAAAATCTAGCTCTTCTTATCACTGTAGCATTATTGCAATGAAAACACTTCTTAA

GTTTACAAGACATGACTACATATTTAAAGACGTGTTCAGGGAGGTTGGCCTTTTGGAGGTCATGGTAAAC

CTTTTGCATAAATATGCTGCCCTGTTGAAGGATCCAACTCAGGCACTAAATGAACAAGGGGACTCAAGAA

ATAATAGTTCAGTTGAAGACCAAAAACACCTGGCTTTATTGGTTATGGAGACCTTGACAGTGCTTCTTCA

AGGATCAAACACAAATGCAGGAATTTTTCGAGAATTTGGAGGTGCAAGATGTGCACATAATATAGTAAAG

TACCCTCAATGCCGGCAGCATGCCTTGATGACTATCCAACAGCTGGTGCTCTCCCCAAATGGGGACGATG

ACATGGGCACTCTCCTGGGGCTAATGCATTCAGCCCCACCGACGGAATTGCAGTTGAAGACTGATATTTT

AAGGGCCCTCCTGTCGGTCCTTCGAGAAAGCCATCGTTCAAGAACAGTTTTTAGGAAAGTTGGAGGATTT

GTGTACATTACATCCTTGCTCGTTGCTATGGAAAGATCTTTGAGCTGTCCACCCAAGAATGGCTGGGAGA

AAGTGAACCAGAATCAAGTGTTTGAACTTCTTCACACTGTGTTCTGCACGTTGACTGCAGCAATGCGCTA

TGAGCCAGCCAACTCTCATTTCTTCAAAACAGAGATTCAGTATGAGAAGTTGGCAGATGCTGTTCGATTT

CTTGGCTGCTTCTCAGACCTAAGAAAAATAAGCGCCATGAATGTCTTCCCCTCAAATACACAGCCATTTC

AAAGACTTTTAGAGGAAGATGTAATCTCAATAGAATCAGTGTCACCCACGTTACGGCACTGCAGTAAACT

TTTTATTTATCTTTACAAAGTAGCCACAGATTCTTTTGACAGTCGTGCAGAACAGATCCCTCCTTGCCTG

ACAAGTGAGTCTTCTCTCCCCTCTCCTTGGGGTACACCAGCTTTGTCCAGGAAAAGGCATGCATATCATT

CTGTTTCAACTCCCCCTGTTTACCCTCCTAAAAATGTTGCCGACCTGAAACTACATGTGACAACTTCATC

TCTGCAGAGTTCTGATGCAGTCATCATTCATCCTGGAGCCATGCTTGCCATGCTGGACCTACTGGCCTCT

GTTGGGTCAGTGACACAGCCAGAACATGCTTTGGATCTTCAACTTGCCGTGGCAAATATTTTACAATCCC

TGGTGCACACAGAAAGGAACCAGCAAGTCATGTGTGAAGCTGGTCTTCATGCACGACTGCTGCAGAGGTG

CAGTGCTGCATTGGCTGATGAGGACCACTCACTGCACCCGCCCCTGCAGCGGATGTTTGAACGATTAGCC

TCTCAGGCTCTGGAACCCATGGTGTTGAGGGAGTTTTTACGTTTGGCAAGTCCTTTAAATTGTGGTGCCT

GGGACAAAAAACTGCTAAAACAATATAGGGTCCACAAACCAAGTTCACTGAGTTATGAACCAGAAATGAG

AAGTAGTATGATCACATCTCTGGAAGGTCTGGGTACTGATAATGTTTTTAGCTTACATGAAGATAACCAT

TACCGGATAAGCAAGAGCCTGGTAAAATCTGCGGAAGGAAGTACTGTACCCCTGACCAGGGTGAAGTGTC

TGGTCTCCATGACAACCCCACATGACATCAGACTTCATGGGTCATCAGTTACTCCAGCTTTTGTTGAATT

TGACACATCACTTGAAGGGTTTGGATGTCTTTTTTTGCCCAGTTTGGCCCCTCATAATGCTCCTACAAAT

AATACCGTCACAACAGGTCTTATTGATGGGGCTGTGGTCAGTGGCATTGGTTCTGGTGAAAGATTCTTCC

CTCCTCCCTCCGGCTTAAGTTACTCTAGCTGGTTTTGTATTGAACATTTTAGTTCTCCTCCAAATAACCA

CCCTGTCAGACTTCTTACTGTTGTGCGCCGAGCAAATTCTTCTGAGCAACATTACGTGTGCCTTGCAATA

GTTCTATCAGCAAAAGACCGATCTCTGATTGTTTCCACCAAAGAGGAACTCCTCCAAAATTATGTTGATG

ATTTTAGTGAAGAGTCCTCATTTTATGAAATTCTCCCATGCTGTGCTCGCTTTCGATGTGGAGAGCTTAT

CATTGAGGGACAGTGGCATCATTTGGTCCTGGTAATGAGCAAAGGCATGTTGAAAAACAGTACTGCAGCC

CTTTATATTGATGGACAGCTTGTTAACACTGTAAAGCTTCATTATGTCCACAGTACTCCAGGGGGTTCAG

GTTCGGCAAATCCACCAGTGGTGAGCACGGTCTATGCCTACATTGGTACTCCACCTGCCCAACGCCAAAT

TGCCTCATTGGTTTGGCGCCTGGGACCCACACATTTTCTAGAAGAAGTTTTACCTTCTTCAAATGTTACT

ACCATTTATGAACTTGGACCAAATTATGTTGGAAGCTTTCAGGCTGTATGTATGCCATGTAAAGATGCAA

AATCCGAAGGGGTGGTGCCATCCCCTGTGTCATTAGTACCAGAGGAGAAAGTGTCATTTGGCCTCTATGC

ACTCTCTGTGTCGTCTCTAACAGTGGCAAGAATCCGGAAAGTGTATAACAAATTGGATAGCAAAGCCATT

GCTAAGCAGTTAGGCATTTCCTCACATGAGAATGCCACTCCTGTGAAGTTGATACACAATTCAGCAGGAC

ATCTTAATGGATCTGCACGGACAATTGGGGCCGCTCTGATTGGATACTTGGGAGTAAGAACATTTGTCCC

TAAGCCTGTTGCCACTACTTTGCAGTACGTTGGTGGAGCTGCAGCCATCCTGGGCCTGGTGGCCATGGCC

TCTGATGTGGAAGGGTTATATGCAGCAGTCAAGGCCCTGGTTTGTGTGGTCAAGAGTAACCCACTAGCCA

GCAAAGAAATGGAAAGAATCAAGGGCTACCAGTTGCTGGCAATGTTGCTTAAGAAGAAACGTTCCCTTCT

TAACAGCCACATCCTCCATCTAACTTTTTCTTTGGTGGGAACTGTTGATAGTGGACATGAGACCTCCATT

ATTCCAAATTCAACTGCTTTCCAGGACCTCCTCTGTGATTTTGAAGTCTGGCTCCATGCACCATATGAAC

TTCATCTTTCCTTATTTGAACACTTTATTGAACTGCTCACAGAGTCCAGTGAAGCCTCAAAGAATGCCAA

ATTAATGAGAGAATTCCAGTTAATCCCAAAGCTGCTCCTGACTCTTCGAGATATGTCTTTATCCCAGCCT

ACTATTGCTGCTATTAGTAATGTCCTGAGCTTCTTACTGCAAGGTTTTCCTAGCAGCAATGATCTGCTCA

GATTTGGGCAGTTTATTTCTTCTACTTTGCCAACCTTTGCGGTTTGTGAGAAATTTGTAGTAATGGAAAT

AAATAATGAAGAGAAGCTTGACACTGGAACTGAAGAGGAGTTTGGAGGTCTTGTATCAGCTAATCTTATA

CTTTTGAGGAACAGACTTCTGGATATCTTGCTAAAACTAATTTATACATCTAAAGAAAAGACAAGCATTA

ATTTGCAAGCTTGTGAAGAACTGGTGAAGACACTGGGTTTTGACTGGATCATGATGTTTATGGAGGAACA

CTTACATTCCACCACAGTTACAGCAGCCATGAGGATTCTTGTTGTCCTACTAAGTAATCAGTCTATTCTC

ATCAAGTTTAAAGAAGGACTCAGTGGTGGAGGATGGCTTGAACAGACAGATTCTGTCTTAACTAATAAGA

TTGGAACTGTATTAGGATTCAACGTGGGCAGAAGTGCTGGTGGGAGATCGACGGTCAGGGAGATTAACCG

AGATGCTTGTCATTTTCCTGGTTTTCCAGTCCTTCAGTCATTCCTTCCTAAACACACTAATGTCCCTGCC

CTCTATTTTCTCCTCATGGCCTTGTTTCTGCAGCAGCCAGTTAGTGAGCTGCCTGAGAACCTGCAGGTCA

GTGTGCCTGTCATCAGCTGCCGGAGTAAGCAGGGTTGCCAGTTTGATTTGGATTCCATTTGGACATTCAT

CTTTGGAGTTCCTGCCTCCAGCGGAACTGTGGTCTCTTCTATCCATAACGTATGCACAGAAGCTGTTTTT

TTATTATTGGGAATGCTCCGCAGCATGCTGACTTCACCTTGGCAATCAGAAGAAGAGGGATCTTGGCTCC

GAGAATATCCTGTGACCCTGATGCAGTTCTTCAGATATTTGTATCACAACGTGCCAGACCTTGCCTCCAT

GTGGATGAGCCCTGACTTCCTGTGTGCATTAGCAGCCACCGTCTTCCCCTTCAATATTCGCCCTTACTCA

GAGATGGTGACTGACCTTGATGATGAAGTTGGATCTCCAGCAGAAGAGTTTAAAGCGTTTGCAGCAGACA

CAGGGATGAACAGGAGCCAATCAGAGTACTGCAATGTGGGCACCAAGACATATCTGACCAATCACCCGGC

TAAAAAGTTCGTTTTTGACTTCATGCGGGTCTTAATCATAGACAACCTCTGTCTCACTCCTGCCAGCAAG

CAAACTCCACTAATTGATCTTTTGTTGGAGGCTTCCCCTGAAAGGTCTACAAGAACTCAGCAAAAAGAAT

TTCAAACTTACATTTTGGATAGCGTGATGGACCATTTGCTTGCAGCTGATGTGTTATTAGGGGAAGATGC

ATCTCTGCCTATTACCAGTGGAGGAAGCTACCAGGTATTGGTGAACAATGTGTTTTATTTCACACAGCGT

GTGGTGGACAAGCTTTGGCAAGGCATGTTCAACAAAGAATCTAAACTTCTTATAGATTTTATAATTCAAC

TAATTGCACAGTCAAAGAGAAGATCACAGGGATTGTCACTGGATGCAGTGTATCATTGCCTCAATAGGAC

CATCTTGTACCAGTTCTCACGGGCACACAAAACCGTTCCTCAGCAAGTAGCTCTGCTTGATTCACTCAGG

GTCCTCACTGTAAACAGAAACTTGATCCTGGGACCTGGGAACCATGACCAAGAATTCATTAGCTGTCTGG

CCCACTGCTTGATAAATCTACATGTTGGAAGCAACGTGGATGGATTTGGACTGGAAGCAGAAGCCCGCAT

GACCACATGGCACATTATGATCCCCTCGGACATTGAACCAGATGGTAGTTACAGCCAAGATATTAGTGAA

GGGCGTCAGCTTCTCATAAAAGCTGTCAACAGAGTTTGGACTGAACTGATACATAGTAAGAAACAAGTCT

TAGAGGAACTTTTCAAAGTAACTCTACCTGTGAATGAAAGGGGCCACGTGGACATAGCTACAGCAAGGCC

ACTCATTGAAGAAGCTGCCCTGAAGTGCTGGCAGAATCATTTGGCCCATGAAAAGAAATGCATAAGTCGA

GGAGAAGCTTTAGCGCCCACCACACAGTCCAAATTATCCCGTGTCAGCAGTGGCTTTGGTCTTTCCAAGT

TAACAGGATCAAGAAGGAATCGAAAAGAAAGTGGTCTTAATAAACACAGTCTTTCCACCCAGGAGATTTC

GCAGTGGATGTTTACTCACATTGCTGTTGTTCGTGACTTAGTAGATACACAATATAAAGAATATCAGGAG

CGTCAGCAGAATGCCCTGAAGTACGTGACAGAAGAGTGGTGTCAGATCGAGTGCGAGCTGTTGAGGGAGC

GGGGGCTGTGGGGCCCTCCCATCGGCTCCCACCTCGACAAGTGGATGCTGGAGATGACAGAAGGGCCCTG

CAGGATGAGGAAAAAGATGGTGCGAAATGATATGTTTTATAACCATTACCCTTACGTGCCAGAAACTGAG

CAAGAGACAAATGTGGCGTCTGAGATCCCAAGTAAACAGCCTGAGACACCCGATGATATTCCTCAAAAGA

AACCTGCTCGATATAGAAGAGCCGTAAGTTATGACAGTAAAGAGTACTACATGCGACTGGCCTCTGGCAA

TCCCGCCATTGTCCAAGACGCCATTGTGGAGAGTTCAGAAGGTGAAGCTGCTCAGCAAGAACCAGAGCAT

GGGGAAGACACTATTGCTAAAGTCAAAGGTTTGGTCAAGCCTCCTCTAAAACGCTCCCGATCTGCACCTG

ATGGAGGAGATGAGGAGAACCAGGAGCAGCTACAAGACCAGATTGCTGAGGGCAGCTCCATAGAAGAGGA

GGAGAAAACAGATAATGCTACCTTACTGCGCCTGTTAGAGGAAGGAGAAAAGATCCAACACATGTACCGC

TGTGCTCGAGTCCAGGGCCTAGATACCAGTGAGGGGCTCCTTCTTTTTGGTAAAGAGCATTTTTATGTGA

TTGATGGATTTACCATGACAGCAACCAGGGAAATAAGAGATATTGAAACCTTACCTCCAAATATGCATGA

GCCTATTATTCCTAGAGGAGCCAGGCAAGGCCCTAGTCAACTCAAGAGAACATGCAGCATTTTTGCATAT

GAAGATATCAAGGAAGTTCATAAAAGGAGATATCTCCTGCAGCCTATTGCTGTGGAAGTTTTCTCTGGAG

ATGGACGGAATTACCTCCTTGCTTTTCAGAAAGGAATCAGAAACAAAGTCTATCAAAGGTTTTTGGCTGT

AGTGCCATCTCTAACGGACAGTTCAGAATCTGTATCTGGGCAACGACCAAACACGAGTGTGGAGCAGGGA

TCTGGGTTACTTAGCACTTTGGTTGGAGAGAAGTCTGTGACTCAGAGATGGGAGAGAGGTGAAATCAGCA

ACTTCCAATATTTGATGCATTTGAACACTTTGGCTGGCAGATCATATAATGATCTCATGCAGTATCCTGT

CTTCCCCTGGATCCTTGCAGATTATGACTCAGAGGAGGTGGATCTTACTAATCCCAAGACGTTTAGAAAC

CTGGCTAAGCCAATGGGAGCACAAACAGATGAACGATTAGCTCAGTATAAGAAGCGGTATAAAGACTGGG

AGGATCCTAATGGAGAAACTCCTGCATACCACTATGGGACCCACTATTCATCTGCAATGATTGTGGCCTC

ATACCTTGTAAGGATGGAGCCTTTCACACAGATATTCTTAAGGCTACAGGGTGGCCACTTTGACCTGGCT

GACCGGATGTTTCACAGTGTGCGCGAGGCCTGGTATTCAGCGTCAAAGCACAATATGGCAGATGTAAAAG

AACTTATCCCAGAGTTCTTTTATTTACCAGAATTCCTGTTCAATTCCAACAACTTTGATCTAGGCTGTAA

ACAAAATGGCACCAAGCTTGGAGATGTTATCCTTCCACCCTGGGCAAAAGGGGACCCACGAGAATTCATC

AGAGTCCATCGTGAGGCTTTGGAGTGTGATTACGTGAGTGCCCATCTACATGAGTGGATTGACTTAATCT

TCGGTTATAAACAGCAAGGCCCTGCTGCAGTAGAAGCTGTAAATGTCTTCCATCATCTTTTTTATGAGGG

TCAAGTGGATATCTACAACATCAATGACCCACTAAAGGAGACAGCCACAATTGGGTTCATTAATAACTTC

GGTCAGATCCCTAAACAGTTATTTAAAAAACCTCATCCACCAAAGCGAGTGAGAAGTCGACTCAATGGAG

ACAATGCAGGAATCTCTGTCCTACCAGGATCTACAAGTGACAAGATCTTTTTTCATCATCTAGACAACTT

GAGGCCTTCTCTAACACCTGTAAAAGAACTCAAAGAACCTGTAGGACAAATCGTATGTACAGATAAAGGT

ATTCTTGCGGTGGAACAGAATAAGGTTCTTATCCCACCAACCTGGAATAAAACTTTTGCTTGGGGCTATG

CAGACCTCAGTTGCAGACTGGGAACCTATGAGTCAGACAAGGCCATGACTGTTTATGAATGCTTGTCTGA

GTGGGGCCAGATTCTCTGTGCAATCTGCCCCAACCCCAAGCTGGTCATCACGGGTGGAACAAGCACGGTT

GTGTGTGTGTGGGAGATGGGCACCTCCAAAGAAAAGGCCAAGACCGTCACCCTCAAACAGGCCTTACTGG

GCCACACTGATACCGTCACCTGCGCCACAGCATCATTAGCCTATCACATAATTGTCAGTGGGTCCCGTGA

TCGAACCTGTATCATTTGGGATTTGAACAAACTGTCATTTCTAACCCAGCTTCGAGGGCATCGAGCTCCA

GTTTCTGCTCTTTGTATCAATGAATTAACAGGGGACATTGTGTCCTGCGCTGGCACATATATCCATGTGT

GGAGCATCAATGGGAACCCTATCGTGAGTGTCAACACGTTCACAGGTAGGAGCCAGCAGATCATCTGCTG

CTGCATGTCGGAGATGAACGAATGGGACACGCAGAACGTCATAGTGACAGGACACTCAGATGGAGTGGTT

CGGTTTTGGAGAATGGAATTTTTGCAAGTTCCTGAAACACCAGCTCCTGAGCCTGCTGAAGTCCTAGAAA

TGCAGGAAGACTGTCCAGAAGCACAAATAGGGCAGGAAGCCCAAGACGAGGACAGCAGTGATTCAGAAGC

AGATGAGCAGAGCATCAGCCAGGACCCTAAGGACACTCCAAGCCAACCCAGCAGCACCAGCCACAGGCCC

CGGGCAGCCTCCTGCCGCGCAACAGCCGCCTGGTGTACTGACAGTGGCTCTGACGACTCCAGACGCTGGT

CCGACCAGCTCAGTCTAGATGAGAAAGACGGCTTCATATTTGTGAACTATTCAGAGGGCCAGACCAGAGC

CCATCTGCAGGGCCCCCTTAGCCACCCCCACCCCAATCCCATTGAGGTGCGGAATTACAGCAGATTGAAA

CCTGGGTACCGATGGGAACGGCAGCTGGTGTTCAGGAGTAAGCTGACTATGCACACAGCCTTTGATCGAA

AGGACAATGCACACCCAGCTGAGGTCACTGCCTTGGGCATCTCCAAGGATCACAGTAGGATCCTCGTTGG

TGACAGTCGAGGCCGAGTTTTCAGCTGGTCTGTGAGTGACCAGCCAGGCCGTTCTGCTGCTGATCACTGG

GTGAAGGATGAAGGTGGTGACAGCTGCTCAGGCTGCTCGGTGAGGTTTTCACTCACAGAAAGACGACACC

ATTGCAGGAACTGTGGTCAGCTCTTCTGCCAGAAGTGCAGTCGCTTTCAATCTGAAATCAAACGCTTGAA

AATCTCATCCCCGGTGCGTGTTTGTCAGAACTGTTATTATAACTTACAGCATGAGAGAGGTTCAGAAGAT

GGGCCTCGAAATTGTTGAAGATTCAACAAGCTGAGTGGAGACCATGGTCTGTAGACCCCTTCCCGATTCT

CCTGTCCCAGCTTGGAAGGCATTGAAAACAGTCTCCGTTTACACATCTCTTCATACCACGTGTTTGAAGT

GTTAAAATTCAAAGGGATCATTGAATAAAACGGGTGTAGAGTACAGGAATGGGGCAGACGCGATTCAGGT

GAACAGCACAAGAAGAATATGAGGTGGTTCCTAGGAGCAACACTTTCGACCTCCAGTTCTCCCTGATGAC

AGTAGCTGTCTCCAAGAGAAAAATCCTCACTTATTAACTCTCTTTTCTTGCATCTCATTTTTATAGAGCT

ACTCATCCTTATTTGGAAAAACCAACAACAAAAAAGGCTTTTAGAAAATGGTTGTAAATCTGACTTCTTT

GCAAGTAACTATGTATATTGTAAATAGATATAAAAGGCCTTTTTTCTAAATAAGGACTTAACTGCCTGTA

ACATGAAACTTCAAACTAAACCACTAACTCAATGAACTACTTATGGTTTGTCTGACATCCCTCACTTACC

AATTAATTATAAATATGTTTTTTTAAATCCCCAAAGACATTATCTGTGGTCTTTTTTTCCTTTCAAGCTC

AGCCTGTGTGCCTGATGTCATTTCTTTCAAGTTGCCCACAGTATCTCCACTTAAACTAGGCTAGTAACCA

AAATAATGTGGACCTTCTTTAGGAAACAGTGTGGGAGAATAGGAGTCCAGCCGTAAGATAAACTGGAAAT

ATTTGGGCGTCTTGTACCTGGCTACGCACCACCTCAGTGTTGTTCCTACATAAACAGGGCCCCTTTTAAA

CTTGTATGTGGACTGCTGTTTGGTCAAAGAATACCTTCTTAGCATTGCAGAAAGGTGGTCAGATGACCAG

TGTAGTGCAGGAAACAGCCCTGTCTCAACTAATGGAAATATATTTGCATGTAACCCAAAATTAGCTTATC

TTGCATAGAACATAATAAGTATGTGTCTTTGGTGACACTAATGTTCTACTATAGCTTATTTTCAAACAAG

GGGTAAAAAAAGGAAAGAAAGAAGTGTACAGAATTAACATATAAACTTTGTTGTAAAACTGAATCATGTC

AGAACTGCTTAAAATTAACCTTTACCATTTAATGTCATCTACCTGAAAACAGTGAGATTTATACTGTATC

AATGTCTATTTTTTTGTTTTTGCTATGAATATAATTACAGTATTTTAATATTTAGTTATTTAATTTGTTC

TACTAGTTGGATACAGAACACACAAATCCAGGGGGATTAAAGCTGGAAGGGGCTAAGAGATTAGTTTACA

GAGAAAAGGCTTGGTGGTGGGATTTTTTTAAATGTGTGTTATGTACATATATATATATATATAATATATA

TTAAAAATGAAACAATTAATCTAGATTTTAACATTTTCAGAAACTTAGTGATAACATTATGAACAATTCT

AAAAGCCCTGTGATTTGAAAAATATAGAATCATTAATGGCCCAAGATAGGCCTTCACACCTTCACAGGTG

CGAAAGGAAAGGCCTTCACACCCTCACAGAGGCATCATGCAAAGGACAGCGGCTTTGGCTTTTCCAATTT

TCCATCTTTAGGCCCTGGTGAGAGGCACACTTATGCACTAAAATGCACATATATGCACATGCATTCAAAA

ATAGGCATTTGGTACAATGGTGATCTTGTACCTGATGGGCTGAAACCAGCTTAAGAACAAATTTGTTCTT

CCTGATATGATAACTAGGTCTCCAAGAGAAAATAGAAAGGCTGCTTTAGTGCCTTACGCTTACTAAATTT

AAATCTTTATTTACCTGGGTTTGAGCCTACAGTCTATTTATGATTACATATCAAAATTGATTAAAACACT

TCCATTTCTAAAAGTTCAAATATACTTGTTAATAAAAGGATTATCGGCATTAATACTTTAATTTAAAGAA

AAGTTGTGTTCTGTTTTCCTTTCTGTGTCTTACTCCCCCCACACTCTCCCTCCCCCATCACCATCTTCAA

TTCTAATAAATAATGCTGATGTTCAACAGTTGCAGAAATTGTGCTATTATGTAACTGTGGGCCTTGCCCC

TGTCTGGCCCTCTAGATGATTTGTAGCAGTGTTATTCTACACTTTTTAAAAGAAGCGTCCTCCTTTTGTC

CATGAATCATGTTTACCCCATACCCAGTGGCAGAGGTGTTCTTTAAAGACTTGAATATATGAATGTGTGT

GTGTAGTTACTTAAAGGTTATTCCTCTTTGTAATAGGAAACTATATGGGATGAACACTTTTAAACTTTCC

GACACAACTTCCATTACTAACTTTCTAACAGAACTTCCATAACTAGAAGGTGGAAACCAAAACCCTCATG

GTAGTATTTCCTCTGGCAGCTGGTGCTGTGGGCAACTGTTTTGTTCAATCGGGTTTCTTTTCTTTTTGCC

TCTAATGCAGAAATCAACAGAATCACTCACACATACAAGTACACTCACATACATAAACTAATTATTTCTC

TGGATATCTTTCTGTGTTCCATGTAAATTTATTTACCAACATCTATTGTCAACATGTACATCTACCTTAG

TATGGTCTGCATTCTTTTTCTGAGAGTACCTCATAGGGCTCCTGCCTGATCTTTGTAGTTTGTTCATTCA

TCCATCCACCTGTTCATTTGTTCATCCATGTATTCTAACATTTCTATGTAGTGTGCAACTCTAATGTCAT

GCTTTTGAAGAAGAGAATAGCTGCCCATAGCAGCCATCCGTCTGGATAATAGCAAAACACTCTAGATAAG

TTATTTTGCACTTTCTTATGTATAAAGTTGGTAGAAACTTATTTTTGCTTTGTATCATTTAAATACATTT

TGTTTTGGTAAATGAACTGTGTATAAAATATTTATGCCGTTAAAACTGTTTTTAGAAAGTATTTTTAATT

TCAGCAAGTTTGGTTACTTGTTGCATGACTCTTAACACAGCTGACTTTTTGTGTCAGTGCAATGTATATT

TTTTGTCCTGTTATTAACTTGTAAGCCCTAGTAATGGCCAATTATTTGTACAGCAACAGAAGTAAATTGA

AGATACTGGCTAAGACTGGATTGATTGTGGACTTTTATACTATATTGCAGAAACCAATATCTGTTTCTTG

GTGGTTATGTAAAAGACCTGAAGAATTACTATCTAGTGTGCAGTCTGTGATATCTGAATGTTCATTGTAT

ATTTGTCTCTGATGCAAAAAGGTAGAGTAACACAATTACAATACATGATTAAATGCAATAGTCCAGGTA

>NM_007075.4 Homo sapiens WD repeat domain 45 (WDR45), transcript variant 1, mRNA

GGGTGCCATTCAGTGCCTGGGCTCCGGGAAAGTCGTGCTTTTACGGTTCCGGGTACTGGCTCCCACACCA

CTGCCTCGTGTGGGGTTGTTCGCCCGTGAAGGGGCAGGACAGGGTGCGCGCTGGTGGAGGTTGAAATTGT

TACATTTTGGCCGGGCGCGGTGGCTCACGCCTGTAATCCCAGCACTTTGGGAGGCCGAGGCAGGTGGATC

ACGAGGTCAGGAGATCGAGACCATCCTGGCTAACACAGTGAAACCCCGTCTCTACTAAAAAAATACAAAA

AATCAGCCGGGCGTGGTGACGGGCGCCTGTAGTCCCAGCTACTCAGGAGGCTGAGGCAGGAGGATGGCGT

GAACCCGGGAGGCGGAGCTTGCAGTGAGCCGAGATCGCGCCAGTGCACTCCAGCCTGGGCGACAGAGCGA

AACTACGTCTCAAAAAAAAAAAAAAAAGAAAGAAAAGAAATTGTTACATTTTATATATATAGGAACAATCC

TGCACCATGACTCAACAGCCACTTCGAGGAGTGACCAGCCTGCGTTTCAACCAAGACCAAAGCTGCTTTT

GCTGCGCCATGGAGACAGGTGTGCGCATCTACAACGTGGAGCCCTTGATGGAGAAGGGGCATCTGGACCA

CGAGCAGGTGGGCAGCATGGGCTTGGTGGAGATGCTGCACCGCTCCAACCTTCTGGCCTTGGTGGGCGGT

GGTAGTAGTCCCAAGTTCTCAGAGATCTCAGCAGTGCTGATCTGGGACGATGCCCGGGAGGGCAAGGACT

CCAAGGAGAAGCTGGTGCTGGAGTTCACCTTCACCAAGCCAGTGCTTTCTGTGCGCATGCGCCATGACAA

GATCGTGATCGTGCTGAAGAACCGCATCTATGTGTACTCCTTCCCCGACAATCCCCGAAAGCTGTTTGAG

TTTGATACCCGGGACAACCCCAAGGGGCTCTGTGACCTCTGCCCCAGCCTGGAGAAGCAACTGCTAGTGT

TCCCGGGACACAAGTGTGGGAGTCTGCAACTTGTGGACCTGGCGAGCACAAAGCCTGGCACCTCGTCTGC

TCCATTCACGATCAATGCACATCAGAGTGACATAGCCTGTGTGTCTCTAAACCAGCCAGGCACTGTAGTG

GCCTCAGCCTCCCAGAAGGGTACCCTTATTCGCCTCTTTGACACACAATCCAAGGAGAAACTGGTGGAGC

TGCGCCGAGGCACTGACCCTGCCACCCTCTACTGCATTAACTTCAGCCACGACTCCTCCTTCCTCTGCGC

TTCCAGTGATAAGGGTACTGTCCATATCTTTGCTCTCAAGGATACCCGCCTCAACCGCCGCTCCGCGCTG

GCTCGCGTGGGCAAGGTGGGGCCTATGATTGGGCAGTACGTGGACTCTCAGTGGAGCCTGGCGAGCTTCA

CTGTGCCTGCTGAGTCAGCTTGCATCTGCGCCTTCGGTCGCAATACTTCCAAGAACGTCAACTCTGTCAT

TGCCATCTGCGTAGATGGGACCTTCCACAAATATGTCTTCACTCCTGATGGAAACTGCAACAGAGAGGCT

TTCGACGTGTACCTTGACATCTGTGATGATGATGACTTTTAAGGACCCTGGGGGCTGTGCTAGGGACCTG

CAGTGGCAGAACTGCAGAGCTGAGCCTTGGCAGTGGGGCGTGCTTGGAAGCCACCAGCCAGCAAGCATTA

ATGGGGCTGGTGCCCACTTTCCACTCAGCAGAGCTATGTCTAAATAAAGAGCTCACTTCCCCCCAGCACT

TCTTGATGACTGTGTGCCCCAAGGGCCAGGCCAGAGACCCAGGAAGGCAGCGACCCCTTGGGATCCCTAA

CCTGGAGGAAATTGCCAGGGACCCAGAGGGAGTGCCCTAATCCAACCTGGGGATTTTTTAAAAGCTTCCT

AGGAAGAGATGATCTCCGATGTGATGAATACGAATAAAAGGCCCTTAATGGCA

>NM_019613.4 Homo sapiens WD repeat domain 45B (WDR45B), mRNA

ACTTCCGGCCGCGGCGGCCTCAGCGCCGGCCCGAAGGGACCAGGCCGCCGTCCCCAGCGAGAGGCATGCA

GCGCTGAGGAGCGGCGACCCAGCACGGCGGCGCCATGAACCTCCTGCCGTGTAACCCTCACGGCAACGGG

CTGCTCTACGCCGGCTTCAACCAGGACCACGGATGCTTTGCGTGTGGGATGGAAAATGGATTCCGAGTCT

ATAACACTGATCCACTAAAAGAAAAAGAGAAACAAGAATTTCTAGAAGGAGGAGTTGGCCATGTTGAAAT

GTTATTTCGCTGCAACTATTTAGCTTTAGTTGGTGGTGGAAAAAAGCCGAAATACCCTCCCAACAAAGTA

ATGATCTGGGATGACCTGAAGAAGAAGACTGTTATTGAAATAGAATTTTCTACAGAAGTCAAGGCAGTCA

AGCTGCGGCGAGATAGAATTGTGGTGGTTTTGGACTCCATGATTAAGGTGTTCACATTCACACACAATCC

CCATCAGTTGCACGTCTTCGAAACCTGCTATAACCCCAAAGGCCTCTGTGTCCTTTGTCCCAATAGTAAC

AACTCCCTCCTGGCCTTTCCGGGCACGCACACGGGCCATGTGCAGCTTGTGGACCTGGCCAGCACGGAGA

AGCCACCCGTGGACATTCCTGCACACGAGGGTGTCCTGAGCTGCATTGCACTCAACCTGCAGGGAACAAG

AATTGCAACTGCATCCGAGAAAGGGACGCTTATAAGAATATTTGATACTTCATCAGGGCATTTAATCCAG

GAACTGCGAAGAGGATCTCAAGCAGCCAATATTTACTGCATCAACTTCAATCAGGATGCGTCCCTCATCT

GCGTATCCAGCGACCACGGCACAGTGCATATTTTTGCAGCTGAAGATCCAAAAAGGAATAAACAGTCCAG

TTTGGCCTCAGCCAGTTTCCTTCCAAAATACTTCAGTTCCAAGTGGAGTTTCTCCAAGTTTCAGGTTCCC

TCAGGCTCTCCGTGCATTTGTGCCTTTGGAACAGAGCCAAACGCCGTCATTGCAATTTGTGCAGACGGCA

GCTACTACAAATTCCTGTTCAACCCCAAGGGGGAGTGCATCCGAGATGTCTACGCGCAGTTTCTAGAGAT

GACCGATGACAAGCTGTGACTCCAGCTGGGGGCGCCACAGCACCCACCACCTGCCGCCTTCAGACTCTCG

GGGCTGGTGCCAGTGCCCCAGGGGCCTCCTGGGCCACGGGCTGGAGGGGCTGCCCAGGGACCTTGGTCTC

GAAGCCATACGTGGTTGTCTGCTTTCCTAAGGACTCCCATTTCCAGTATTAAAGAGAGAATCATCATCAA

GGCACCGTAGGTAACTCAGTGGCTGTGACCAGCTCGACTGGCGGCCACTGGCTGTTCCCATGAGTTCAGC

TGTGACGTTAGCTTCAGTGGCTCCGCCGCATCCTCACACTGACGGGGGCTCCATACGGACCTGGGGACTG

GGCTGAGAGGGTGGACGAGTTCAGGTTTGTTTTTGCAGCAGATTCCGTCGTTCTTACTGAGTCTGCAGCG

GGGGAGTGAACAAGTGTGCAGATGTAAGTTCTTACATGATAAGCAGATTGAATACAACACCAGCAGCTTG

CCTTAGAAAAGGAGAAAGGAATTCCTTTTCCCGCCCGAACATGAAGAAAAATGACCTGACCCTGTAGAGA

GAACACAGTGTGAATGTTTCCCCTCGTGTGAGCCCAGCCTGTGGTCTTCTCCGTACCCGCAACGTGGTCA

TCTGTGCCCGTGACGTCACCTGTGCCCGTGCGTGGCGTCCCCGTCTCCGTTGGGGCCATTAGAATGAGGC

AGACACCAGGCCACTCTAGAAGCCGAGCCGTCACACCTCAGGCGTGTGCGGGGCGGGGACGGGGGGTCTC

CTGGTTACATTTTGGATTAAACCTGTTTCCCGGTTATGTGTAGGGAACAGCAGAGTGATGCACGAACTTT

GAACATTCGTTATGGGGAAAACATCCTTTAACTTCGGGGTCGTCTGCCAGAGCAGGGTCTGGGAGGGTCC

ATGCAGTTCCCGCTGGTGTGGAGGGAAATGCCCTGGTCTGGCCTCCGAGCCCCCAGGTCCACCGTCTCCC

CTCCCCTCATTTGTAAGAATAGCTACACACTAACATTTTGGGAAGGAGAGGCACATAACTTTTTTTAACA

TTTGGTAACTAGGTTATGGGCTCTACATTGTCAGCTACTTGGGATATATATTTAATTTTCTTAAATTCCC

GTTAAACTCTATTTTATGGTTTTGATTTCAGATTGCAAACATGTAAAACCTGCATAGCAGCGAGTTCTCG

GTTTTGCCGGTTTCTTTAGTTCTTTACTGTCACTGTCATGTAATCAGCTAATTCTCTGTGGATGTTGCTG

TAAAGTATGCATGTTCCTTTCATGTGTATTTAATCATGATGTTTAATTTTGCACACTTATTTGTAATGTT

TCTTTTAAATAAAAGTGACTAATTTTGTTGTAGTCTGGACCTGTAA

>NM_017983.7 Homo sapiens WD repeat domain, phosphoinositide interacting 1 (WIPI1), transcript variant 1, mRNA

GTGGAGCCGGCGCGGGCGGGCTGCTGAGGTGGCTGTCGCCGGCTCCGAGCTGCGGCTTCCCGGGCCGAGC

CCCCGATGGAGGCCGAGGCCGCGGACGCTCCCCCGGGCGGGGTTGAGTCGGCGCTCAGCTGCTTCTCTTT

CAACCAGGACTGCACATCCCTAGCAACTGGAACTAAAGCCGGGTATAAGCTGTTTTCTCTGAGTTCTGTG

GAGCAGCTGGATCAAGTCCACGGAAGCAATGAAATCCCGGACGTCTACATCGTGGAGCGCCTCTTCTCCA

GCAGCCTGGTGGTGGTAGTCAGTCACACAAAACCACGGCAGATGAACGTGTATCACTTCAAGAAAGGCAC

AGAGATCTGTAATTACAGCTACTCCAGCAACATCTTGTCCATAAGGCTGAACCGGCAAAGGCTGCTGGTT

TGCCTAGAAGAGTCCATTTATATTCACAACATTAAAGACATGAAGCTGTTGAAGACCCTCCTGGATATTC

CTGCAAACCCAACAGGTCTATGTGCTCTCTCTATCAACCATTCCAATTCTTACCTGGCCTATCCTGGAAG

CCTGACTTCAGGGGAGATTGTGCTTTATGATGGAAACTCCCTGAAAACAGTCTGCACTATTGCTGCCCAT

GAGGGAACACTAGCTGCCATCACCTTCAATGCCTCAGGCTCCAAACTAGCAAGTGCGTCTGAAAAAGGCA

CAGTCATCCGGGTGTTCTCTGTCCCTGATGGGCAAAAGCTCTATGAGTTCCGGAGAGGGATGAAAAGGTA

TGTGACAATCAGCTCTCTAGTGTTCAGTATGGATTCACAATTCCTCTGCGCCTCCAGTAACACCGAGACG

GTACACATCTTCAAGCTGGAACAGGTCACCAACAGTCGACCAGAAGAGCCTTCGACCTGGAGTGGCTACA

TGGGAAAGATGTTTATGGCTGCTACCAACTACCTCCCTACCCAGGTGTCAGACATGATGCATCAGGACAG

GGCTTTTGCCACTGCACGCTTGAACTTCTCCGGACAGAGGAACATCTGTACCCTCTCAACGATCCAGAAG

TTGCCACGGCTGCTAGTTGCGTCATCCAGTGGACACCTTTATATGTACAATTTGGATCCTCAGGATGGAG

GAGAGTGTGTCTTAATCAAAACCCACAGCTTGCTTGGCTCAGGAACAACAGAAGAGAATAAAGAAAATGA

CCTCAGACCTTCCTTACCTCAGTCTTATGCAGCGACCGTAGCCAGACCAAGTGCATCTTCAGCCTCCACG

GTGCCAGGTTATTCTGAGGACGGCGGGGCGCTGCGAGGAGAAGTTATTCCTGAACATGAGTTTGCGACGG

GACCAGTGTGTCTTGATGATGAGAATGAGTTTCCTCCTATAATCTTGTGCCGTGGAAATCAGAAGGGCAA

AACGAAGCAGTCATGATGAGAAGCACACCTCAGAAATCAGGACATCCCCCCTATCAGGTGGTTTTGGAGA

AAACAAGGAAGGCGGAAGAATGGAGTGCAATTGTGTGAGCAGAAAGGGGGGCAGGAATCCCGGGTGCTCC

ACTGCTTAAACCACAGGACCTGGTTAACTCCTCACCAAGCTTCCCACGACCCTGGTTGCCAATGGGCGCG

GGAGACATTGTATACACATCATGCTATTTAAAATACGTTCAAACTATAGTGTAAATGCTAATTAACCATA

TTGGTATATAACCGGAATTTTATATTAAAAGGGGCCTCCTTTTTAAATATATGCCGTGTAAAAAATGTAC

TTATAGGAACATCTCTTTGAATTGTATTTCTTGTATATTACATACTTAGAGAGAGACTCTTTTAGCCAGG

CAAAGTCTTTTTTGGCTGTGGCTGGAATAAATCATTTATTACTTGGGAGTCCCATTTTGGACACTAATAA

TAAAATCATGGCAATGCA

>NM_015610.4 Homo sapiens WD repeat domain, phosphoinositide interacting 2 (WIPI2), transcript variant 1, mRNA

GTACCGGGTGCCCCGGCTCTGGAGCATAAACAAGAGCGGGGACGGGATGAGGCGGCGGTTGATCCCAGGG

TGGCGAGTGGCGGCGACCGAGGCGGCGAGCGGGGCCCGGCGCCGACCCTGAGTGCAGCCTGACCCGCCCT

CGCGCGCGCGCCCTCCCCGGCCGGGCCCACTCGCCGCGCGCCCAGCCATGAACCTGGCGAGCCAGAGCGG

GGAGGCCGGCGCCGGCCAGCTGCTCTTCGCCAACTTCAACCAGGACAACACAGAAGTGAAAGGGGCATCA

AGAGCAGCTGGTCTTGGCCGTCGCGCTGTTGTCTGGTCCCTAGCTGTTGGTAGTAAGTCCGGTTATAAAT

TTTTCTCCCTTTCTTCTGTGGATAAGCTGGAACAGATCTATGAATGCACCGATACGGAAGATGTGTGCAT

TGTAGAGAGATTGTTCTCCAGCAGCCTAGTGGCCATCGTCAGCCTTAAAGCACCAAGGAAGCTAAAGGTT

TGCCACTTTAAGAAGGGAACTGAGATCTGCAACTACAGCTACTCCAACACGATTCTGGCTGTGAAGCTCA

ACAGGCAGAGGCTGATAGTATGCCTGGAGGAGTCCCTGTACATCCACAACATTCGGGACATGAAGGTGCT

GCATACGATCAGGGAGACGCCTCCAAACCCTGCAGGCCTGTGTGCGCTGTCAATCAACAACGACAACTGC

TACTTGGCGTACCCAGGGAGCGCGACCATCGGAGAGGTGCAGGTCTTCGATACCATTAATTTGAGAGCTG

CAAACATGATTCCGGCTCACGACAGTCCTTTAGCGGCACTGGCCTTTGACGCAAGTGGAACTAAACTTGC

CACGGCTTCGGAGAAGGGGACCGTGATTAGGGTATTTTCCATTCCAGAAGGACAAAAACTCTTTGAGTTT

CGGAGAGGAGTAAAGAGGTGCGTGAGCATCTGCTCCCTGGCCTTCAGCATGGACGGCATGTTCCTCTCCG

CCTCCAGCAACACTGAGACCGTGCACATCTTCAAACTCGAGACTGTGAAAGAAAAACCCCCAGAGGAGCC

CACCACCTGGACCGGGTACTTCGGGAAAGTGCTCATGGCCTCCACCAGCTACCTGCCTTCCCAAGTGACA

GAAATGTTCAACCAGGGCAGAGCCTTCGCCACGGTCCGCCTGCCATTCTGCGGCCACAAAAACATCTGCT

CGCTAGCCACAATTCAGAAGATCCCGCGGTTGTTGGTGGGTGCCGCCGACGGGTACCTGTACATGTACAA

CCTGGACCCCCAGGAGGGCGGCGAGTGTGCCCTGATGAAGCAGCACCGGCTGGACGGCAGTCTGGAAACG

ACCAATGAGATCTTGGACTCTGCCTCTCACGACTGCCCCTTAGTCACTCAGACATACGGCGCAGCTGCAG

GAAAAGGTACTTACGTGCCTTCATCCCCAACGAGACTTGCCTACACAGACGACCTGGGTGCTGTGGGTGG

CGCCTGCCTGGAGGACGAGGCCAGCGCCCTGCGCCTGGATGAGGACAGCGAGCACCCGCCCATGATTCTT

CGGACTGACTGAACTTGACCTGTGACCTCTGACCCGGGGAGCAGAGAACACTGGCTTCACAGAGGACTTT

GTGCATTGCTGCTATGAACTTTGACCTGAGTCGGGGGAGAGGATGGCAGAGACTTTATTAAAAAAAAAAA

AAGATTGTAGTGGTAGTCTAACTCCATAACGCTGAGGAAATACATCATTTTCACTTCAGTGGCTTTTAAA

TCCTGCTTATGAATTTTAGCTTTTTGTTTGTTTGTTTTCTCTTTTTGCCAAAATTAACTGTTTGGTGAAG

CCCGCAAAACCTCCTCGCTTTGCATGCATGAACGTGCCAAGCCAGCATAGGGGAGCTAGAAGCCACTTTC

CAGCCACCTGCCGTTGGGTTTTTTCATATCTGTACATAATGCCGAGTGCGTAAGGAAACCGTGGCGTCGC

GCACAGTGGGTCTGCTTGTCAAGGCCAGTTCTGCAGTGACAGGCCCAGGGGCTGCCCACCAGGTGTGCTG

GGCAGACTTCAGCTGGGACAGAAGTCCGATCTCCCTAGGGCCCCACCTGGACCATTTTCCCTCCGTTTTA

TTTTGTTAATTAAATTCTTTCCAAATTGGATCGCTCTGGGATTTCTTCCATGGTGGACTTTTGTTTCTGA

TCTTGTTTTCCCTGTGGATATTGGAGGACAGCGAGGTTCTTTCTGATACTAAAAACCTTTCTTTCAGGCA

GCAAATGAACTTGAAAGGTTGCCTGGACTCGCTGGAGCAAAGGAAAGCGATTTTGTTTGTATAATTAAAT

GATCTGTTCTTCTACTTCACTCTTCCTGTTGAAAAACTGTGTGATTTTTTTTTTTTTTTAAGTAAAGTTT

GTAGCTCCTCCCATGCCCAGGAAAGCACAGAACTCAAGTGTGGTGGCCGTCTGAGCTGTCCTTTCGCTGG

CCCCGCTGTCCGCAGGGGCTTCCTACCTGTGTGAGAGGTCGTAGCGGGAGACAGCAACAGAGAGTAGGCG

GCTGGGCCACGTCCTTCACAGGGCGTCATGTGCCTTTCTATTTTCATCTTAGAAAATTTCCTCAGCAAAC

CGATGAGAGATTGTGGTTGCAAGCTTCAGTATTTGCCTCGCTTCCCTTCCCTTTTCATATTTACAGAATT

AAAACAACCTCAAGTACCTCAGACTCTGCATTCCAAACCAAGGCACCCAGCAGCCAAAGTGTCGAGGGCA

TTTAAGTGGCATTAATGGCAGGAGAGATGGTTTTAGAATCTATGGAGTGGTGGAAGTTACGGATAGAAGG

GAAAAGGCAAAAACTATTTACCCTGCCTTTGCAGGCTGGGGTTTTTGAACCGAGGAAGGCTGGGACGCCT

GTTTCCAGATGGTTGTCATGGTCACGCTGGGCGAAGAGCTGGAGGGGAGTTGTCCCCTCAGCTGAGCGGC

TGCGGTGAAATGCCCCAGTGTTCCTGGGTTGGCTTTACGGCAAACTAAATGCAGGGGACGCTGGAGTCCG

ACTCACCTACACCGGCTTCCTCCCAGCCGCTGGTGTGCGGCACACACAACATCTGCATTAGGCAGAGGTG

CAAGTGGGCTGATTGAACTTTTCCTTCAAAACCTGCTTGTCTGTCCTGGACCTTTGATGAAATGGGATCC

CGGTCACGCAGGCTGAGACAGTGGGGACCGCCGAGGCCAGAGTGGGCTATGCTTGAGCAGGGATGAGAAG

GGCCGCGGCAGCACGCAGCCTTGACCCACGCCTGCGTCTTGTGGTGCAAGGCCAGAGGGCTCTCTCTAGA

ACCTGACCGTGCACTCCATCTCCTGGGAGCCACTTTTGGCAAGAGTGAGTGTGGGGGGAAAAAGTGTGCA

CAAGAGATACGGAACCCTGAGCTAGGGTTTCCTGTCACCAAAGAGTGAGCTGCATTTCCAGTTCTGCGTT

AAAGTATCGTTTGTTCTGGTGCCTGCCTGTGGTGGTGATTTGGAGACTCAAATTTCCTTTGCCAGGTCTC

TTTTGTCTTTCTTTGGGATTGGTAGTATAGAAGATGCTGGGAATTGTCTTCCTCGCTCAGCCTCGGCTTC

CCGCACTGTAAGATGAGGCAGTTGCAGAGGAGGCCTCGGACGTCCTGTGACCCTCGGCCCACACTCCCCC

TTGGGTGGCCCTGGCAGGGCTGGCTAGGTGCGTTCCCTGCAGACCACGGGAAGCCCTGTGCTTGCCTGGG

TCAGGGCTAAGCTGTGCGCCCTAGAGGAGGGAGGAGGACTGCAGATTCTTGGTCGAGAAGAATGAAGAGG

ATTTCTGTGTTTGCTGAGCAGCCATTGTGGAGGGTTACCCGCCTCCACTTTCCCGTTGCCCATTTCACTG

CCACCAGCTCCTCTTCCTCTGCTCGAACCTATGAGTCCCAGCCTCACTGGCGGCCTCAGACATGCTCAGG

AGTGACGGGGACAGAGGGAGGCCGCTGAGTTGCCCGTTAGAACTCTTACTGCTGCGCCAGTGACCCAGGT

GGAGAGGGACCCTGAACCAAACAGAACGTGTGCTAATTTTCCGAACTCCAAACTGTACACTCATATTCAT

TTTTAAATTGTATTTTTCCAAACTTCAAAAAGGACGATGAGCGTGGGGGATAGGAAACAAAACCTGTAAA

CGTTGTGAATGGGCTCAGTGGACTCTGGGACCAAACCTTCTGTAATCTCTAAAACAATGGGACCAAGAGC

TGGATGGAACCTGGAGTCAAAAAGAACTGCTTCAGTCCCCGCTGTACCGCCTGCCTAGCTGTGGGAGCAG

GCAGGGCGCCTGGGAGCCTGCGTTTTCTGGACCGTTCCATGGGACTCATCCCTACCTCACAGGGCTGTTG

TGAGGTGTTGTGTGACTGCGTGTCCTGCAAACGCCCAGCTCGGTGCCCAGCCAGCGGTGGGCACCCAATA

AACGCTACAACATAAATGTGTCTTCTGCAATTAAA

>NM_021260.4 Homo sapiens zinc finger FYVE-type containing 1 (ZFYVE1), transcript variant 1, mRNA

ACTCCGATCAGCTGATCCCAACTGACAACAGGAGAGGAGGAAGCCCGGGAGGCAACGAAGGAGGAGGGTG

GCGGAGATGGAGATGAGGATGGATCTGCCGGTGTCCTGAGGAATAGCCTCTGCCCCCACTGGCGCCCTGC

GGCCCCCCGACGCCGCCTTGCTGCGGCCGAGCTTCTCAGTGGTATCCCCTGAAATACTGACTTCAGGTCG

AATTATATTGAAAAGCTCCTGACCACTTTCTTTCATTACCAAAACTTTGTAGCTGATGTCCAACCGATGA

ACCCACCACCGTGAACCCATCAGACCTCTCTCAGATAGCCATAAAAGACCCTTCCAAGTCAATTTTGACC

ACATCTTTGCTTGCACTTTATGGAGGATGAAACCATCAAACCAAATCAACGTTGCTGCTAATACAAGAGT

CTTAGAGGCAGCAAATTAAAAATTTGAACATTTGTTTGTGAAGAACTATAACAGGACATGAAAGGTGTTC

TTTTTTAAAGTGTTCAGAACCCTGTGGAAGTTTCGTGCAGTCTTCAGACTCAAATCTTCGTCTTCACCCC

CGGGGCAAGCTCAGTGACTATTATATGGTGGGTGTGTTTCCTTACCAGCGTGAGTATGAGTGCCCAGACT

TCCCCAGCAGAGAAGGGCCTGAATCCGGGGCTGATGTGCCAGGAAAGTTACGCTTGCAGCGGGACTGATG

AAGCTATCTTTGAGTGTGATGAGTGCTGCAGTCTGCAGTGTCTCCGCTGCGAGGAGGAGCTCCATCGGCA

GGAGCGCCTGAGAAACCATGAGCGGATAAGACTCAAACCTGGCCATGTCCCTTACTGTGACCTCTGCAAG

GGTCTCAGTGGGCATTTACCAGGTGTTAGGCAGAGGGCAATAGTGAGGTGCCAGACCTGCAAAATTAACT

TGTGCCTGGAGTGCCAGAAGAGGACTCATTCTGGGGGTAACAAAAGGAGACACCCTGTTACTGTGTACAA

TGTCAGTAATCTCCAGGAGTCACTGGAGGCAGAAGAGATGGATGAGGAGACCAAGAGGAAGAAGATGACT

GAGAAGGTTGTGAGTTTCCTCCTAGTAGACGAAAATGAAGAAATTCAGGTAACAAATGAAGAAGACTTTA

TTAGAAAATTGGACTGCAAACCTGATCAGCATCTGAAAGTGGTTTCCATTTTTGGAAATACTGGTGATGG

AAAGTCTCATACTCTCAACCACACTTTCTTTTATGGTCGTGAAGTCTTTAAAACCTCCCCGACCCAGGAG

TCCTGCACTGTGGGAGTGTGGGCAGCCTATGACCCAGTTCACAAAGTAGCAGTGATCGATACGGAAGGGC

TCCTGGGGGCCACCGTGAATCTAAGCCAGAGAACACGGCTGCTGCTTAAGGTCCTGGCCATCTCAGACCT

CGTCATCTATCGAACTCATGCAGACCGGCTGCATAACGACCTCTTCAAATTCCTTGGGGATGCCTCAGAA

GCTTATCTGAAGCACTTCACCAAGGAGCTCAAGGCCACCACTGCTCGCTGTGGCCTGGATGTCCCTTTAT

CCACACTGGGCCCTGCAGTTATCATCTTCCATGAGACCGTGCACACCCAGCTACTGGGCTCTGATCATCC

CTCAGAGGTGCCAGAGAAGCTCATCCAGGACCGGTTCCGGAAGCTGGGCCGTTTCCCTGAAGCCTTTAGT

TCCATTCACTACAAGGGAACGAGGACTTACAACCCTCCCACGGACTTTTCTGGGCTTCGGCGTGCTTTGG

AGCAGCTACTAGAGAATAACACCACCCGTTCTCCCCGGCACCCGGGAGTCATCTTCAAAGCCCTGAAGGC

ACTAAGTGACCGCTTCAGCGGTGAGATCCCCGATGACCAGATGGCGCACAGCTCCTTTTTTCCAGATGAG

TATTTCACCTGCTCCTCCTTGTGCCTCAGCTGTGGGGTTGGATGTAAGAAAAGCATGAATCATGGGAAGG

AAGGAGTGCCTCATGAAGCCAAGAGCCGCTGCAGATACTCCCACCAGTATGACAACCGAGTGTATACCTG

CAAGGCCTGCTATGAGAGAGGCGAGGAAGTCAGTGTAGTGCCCAAAACATCTGCTTCCACTGACTCCCCC

TGGATGGGTCTCGCAAAATATGCCTGGTCTGGGTATGTGATCGAATGTCCTAACTGTGGCGTGGTCTATC

GTAGTCGGCAGTACTGGTTTGGAAACCAAGATCCTGTGGATACGGTGGTGCGGACAGAGATTGTGCATGT

GTGGCCTGGAACTGATGGGTTTCTGAAGGACAACAACAATGCTGCCCAGCGCCTGTTGGACGGGATGAAC

TTCATGGCTCAGTCGGTGTCCGAGCTTAGCCTTGGACCCACCAAGGCTGTGACTTCCTGGCTGACAGACC

AGATCGCCCCTGCCTACTGGAGGCCCAACTCCCAGATTCTGAGCTGCAACAAGTGTGCGACGTCCTTTAA

AGATAACGACACTAAGCATCACTGCCGAGCCTGTGGGGAGGGCTTCTGTGACAGCTGTTCATCAAAGACT

CGGCCAGTGCCTGAGCGGGGCTGGGGCCCTGCGCCAGTGCGGGTCTGTGACAACTGCTACGAAGCCAGGA

ACGTCCAGTTAGCTGTTACCGAGGCACAAGTGGACGATGAAGGTGGAACGCTCATTGCTCGGAAGGTGGG

CGAGGCCGTGCAGAACACTCTGGGAGCCGTGGTGACAGCCATTGACATACCACTAGGTCTGGTAAAGGAC

GCGGCCAGGCCTGCGTACTGGGTGCCTGACCACGAAATCCTCCACTGCCACAACTGCCGGAAGGAGTTCA

GCATCAAGCTCTCCAAGCACCACTGCCGGGCCTGCGGACAGGGCTTCTGTGATGAGTGCTCCCATGACCG

CCGGGCTGTTCCTTCTCGTGGCTGGGACCATCCCGTCCGAGTCTGCTTCAACTGCAATAAAAAGCCCGGT

GACCTTTAACCCCAGCCCCCTCTCCGAGTCCTTCACAATTCCTTAGGTTCTCAGGGTTAGAAACAGTCTT

GCGAGGTAGGCCCTCCTCCCAGTCACCTGCTGTGGTGTGTGTCCTCTCCTCTCCGCATCCAGGGCCACTT

TCCCTCAGTGGGGGTGAGCCTGGCGGCAGGCCCGAAGGTGTGGACCCCTCAGGGCAGGGGACCTTGCAAC

TTATCGCAAAGGGGAATGAACCTGAATCCGTTGCATTTATTTCAGTTAAAAATAATGAATATATATGTGT

ATATCTCTCTCTCATATATACATATGAAAGGCACTCGGGGCGTATCGAGGCTGCTGCTGGCTGTGAAGAC

TTCGCACAGTCTCCTCCGCACAGGGTGAGGTGGCAGTGGCAGCACGTCTTCCTCATGAGCCGAGCCAGGT

CCATGGCCACCACGTGGCTGGCCCCTTCCTCTGCTGCTCTTGGAGCCTTGGAAGCCTCTCCTGTCCTTGG

CTCTTCCCTCCATGCCTGTCAGCTGCCTGGGGAGTGAGCCTCCCTGGTCCTTCCTGCCTGAAACAGCCTG

AAGGGAATTCTCCCTAGGTCTCCTGGGAGTCGAGTCCCAATTCTTGGCTTAAGCCTGTTTTAGTCAGAGA

CCACCCAACTTAGCGTGCAGGTCACCGGAGTGGGTGGAGGGTCAGAGGTCGGGTCTTCGGCCCTGAGAAG

TAGAAATGCAGGGGCCGTGCTGTCCCTGGTCCCCCAGGGAACAGCAAGGAAGGAACTGAGCCTTCTCCAG

CAGGGCTTCCTGTCCCGATGCTTCTGTCTCCACTCGGCTTTCCCAAAAGGCGGCACCCAGCTCCTCAATC

GAAGCATCTGCCTCCCACCCCTCGGCCCCCTCAAGCCCACCATCTGCTTCTGAGTGTCGCACTAGGATTT

TCATTGCTTATTTTAAAGTGTCTTAATCCTTTGTTCCCAGACACACAACCCCTCTAGCTCTCGGAGGGGC

GATCATGAGAAACCTTCCAGGGAAACTGAGCACAGGATGAACTGTTAGTTGTTTTTAAAAGTCTATATAA

ATATTTCAACAGATCGTAAAGAAAAAATTTATCTCTTTGGTCCTTGCAAGAGAAGTCAAAGGAACTTTTG

TTTCTCCTCAAGAGCCTGGACATCTCTGTCTGTCATGACTGGAAAGGGCCCGTTGTGCTGAAATCCTATC

ATCATGGTGGATTTGATCTTCAGTGGCCAAACACGAATTAAAGTATAATTCTTAACTGAACTGGTGGGTG

GGTTGAGCTTTAGGGAAGTATATGGCGGTCATTCCTGGTGCGCCTATCGGTGTGACATGAGCCCTGGAGT

GTGTCGTCCCTCTCAGCCCTGCTCCTCCTGCCTCCTGGGGTCCAGTGACTGGGACCCTACTCTAGAACTG

TGTACCATCCAATTCGCCATCATAAAGGAATCTTCCTGCA
