# Supplementary material for: Analysis of the Compositional Features and Codon Usage Pattern of Genes Involved in Human Autophagy
Source: Cells. 2022 Oct 12;11(20):3203. doi: 10.3390/cells11203203 (PMC9601114; doi:10.3390/cells11203203)
Supplement: Supplementary file 1 [file cells-11-03203-s001.zip › Supplementary Table S4.pdf]

Supplementary Table S4. Improved effective number of codons (ENC) of all the human autophagy genes.

SL. No.	Gene	ENC
1	AMBRA1	49.45
2	APOL1	49.72
3	ARNT	50.48
4	ARSA	37.34
5	ARSB	51.06
6	ATF4	46.92
7	ATF6	50.64
8	ATG10	47.73
9	ATG101	35.28
10	ATG12	42.79
11	ATG13	49.49
12	ATG14	51.53
13	ATG16L1	52.7
14	ATG16L2	39.59
15	ATG2A	39.08
16	ATG2B	51.23
17	ATG3	44.06
18	ATG4A	50.55
19	ATG4B	45.5
20	ATG4C	44.08
21	ATG4D	37.59
22	ATG5	41.76
23	ATG7	53.45
24	ATG9A	44.05
25	ATG9B	41
26	ATIC	50.7
27	BAG1	46.4
28	BAG3	46.41
29	BAK1	38.94
30	BAX	36.15
31	BCL2	36.08
32	BCL2L1	40.16
33	BECN1	46.89
34	BECN2	41.44
35	BID	38.4
36	BIRC5	42.56
37	BIRC6	51.06
38	BNIP1	49.98

39	BNIP3	44.95
40	BNIP3L	51.88
41	CALCOCO2	48.54
42	CAMKK2	41.47
43	CANX	46.49
44	CAPN1	36.69
45	CAPN10	38.81
46	CAPN2	45.83
47	CAPNS1	43.47
48	CASP1	51
49	CASP3	45.63
50	CASP4	49.1
51	CASP8	49.82
52	CCL2	36.95
53	CCR2	47.79
54	CD46	46.51
55	CDKN1A	42.06
56	CDKN1B	44.22
57	CDKN2A	35.06
58	CFLAR	49.43
59	CHMP2B	40.89
60	CHMP4B	45.76
61	CLN3	42.85
62	CTSB	42.81
63	CTSD	33.26
64	CTSL	45.07
65	CX3CL1	42.47
66	CXCR4	43.9
67	DAPK1	48.37
68	DAPK2	40.49
69	DDIT3	41.33
70	DIRAS3	33.65
71	DLC1	53.83
72	DNAJB1	47.34
73	DNAJB9	44.81
74	DRAM1	52.02
75	EDEM1	50.74
76	EEF2	34.14
77	EEF2K	45.63
78	EGFR	47.86
79	EIF2AK2	47.94

80	EIF2AK3	53.28
81	EIF2S1	48.47
82	EIF4EBP1	37.11
83	EIF4G1	51.02
84	ERBB2	41.53
85	ERN1	44.44
86	ERO1A	48.3
87	FADD	35.55
88	FAS	47.91
89	FKBP1A	36.52
90	FKBP1B	39.68
91	FOS	42.4
92	FOXO1	50.97
93	FOXO3	46.1
94	GAA	34.98
95	GABARAP	39.33
96	GABARAPL1	46.05
97	GABARAPL2	41.53
98	GAPDH	39.29
99	GNAI3	52.11
100	GOPC	49.98
101	GRID1	42.43
102	GRID2	54.6
103	HDAC1	48.98
104	HDAC6	46.69
105	HGS	36.89
106	HIF1A	47.83
107	HSP90AB1	46.81
108	HSPA5	48.46
109	HSPA8	47.18
110	HSPB8	42.79
111	IFNG	45.49
112	IKBKB	44.51
113	IKBKE	39.43
114	IL24	42.35
115	IRGM	43.96
116	ITGA3	40.57
117	ITGA6	53.57
118	ITGB1	50.24
119	ITGB4	36.17
120	ITPR1	51.69

121	KIF5B	49.31
122	KLHL24	50.02
123	LAMP1	44.56
124	LAMP2	48.43
125	MAP1LC3A	33.44
126	MAP1LC3B	45.28
127	MAP1LC3C	36.23
128	MAP2K7	35.81
129	MAPK1	49.34
130	MAPK3	36.3
131	MAPK8	50.71
132	MAPK8IP1	40.19
133	MAPK9	48.53
134	MBTPS2	51.39
135	MLST8	35.48
136	MTMR14	45.03
137	MTOR	48.74
138	MYC	42.97
139	NAF1	50.26
140	NAMPT	49.32
141	NBR1	51.52
142	NCKAP1	50.31
143	NFE2L2	51.54
144	NFKB1	52.19
145	NKX2-3	38.6
146	NLRC4	52.42
147	NPC1	52.64
148	NRBF2	46.79
149	NRG1	52.69
150	NRG2	39.02
151	NRG3	51.99
152	P4HB	38.79
153	PARP1	50.69
154	PEA15	37.36
155	PELP1	48.21
156	PEX14	40.43
157	PEX3	47.71
158	PIK3C3	49.5
159	PIK3R4	51.87
160	PINK1	44.64
161	PPP1R15A	47.84

162	PRKAB1	45.71
163	PRKAR1A	51.43
164	PRKCD	40.28
165	PRKCQ	52.11
166	PRKN	49.97
167	PTEN	48.25
168	PTK6	36.65
169	RAB11A	42.53
170	RAB1A	46.93
171	RAB24	36.79
172	RAB33B	48.32
173	RAB5A	42.26
174	RAB7A	42.71
175	RAC1	49.19
176	RACK1	41.09
177	RAF1	53.38
178	RB1	49.93
179	RB1CC1	46.5
180	RELA	42.01
181	RGS19	34.4
182	RHEB	39.62
183	RPS6KB1	48.45
184	RPTOR	43
185	RUBCN	45.75
186	SAR1A	47.35
187	SERPINA1	41.36
188	SESN2	37.76
189	SH3GLB1	47.32
190	SIRT1	50.47
191	SIRT2	40.72
192	SPHK1	40
193	SPNS1	37.43
194	SQSTM1	46.27
195	ST13	47.92
196	STK11	35.01
197	SUPT20H	50.58
198	TBK1	46.77
199	TM9SF1	46.38
200	TMEM41B	42.19
201	TMEM74	47.65
202	TNFSF10	39.4

203	TP53	47.62
204	TP53INP2	35.67
205	TP63	50.55
206	TP73	36.06
207	TSC1	52.87
208	TSC2	39.75
209	TUSC1	35.13
210	ULK1	34.8
211	ULK2	53.45
212	ULK3	40.78
213	USP10	54.48
214	UVRAG	54.02
215	VAMP3	39.42
216	VAMP7	48.69
217	VEGFA	47.69
218	VMP1	48.97
219	WDFY3	54.5
220	WDR45	39.85
221	WDR45B	51.15
222	WIPI1	51.58
223	WIPI2	45.27
224	ZFYVE1	49.56
