# Supplementary material for: Analysis of the Compositional Features and Codon Usage Pattern of Genes Involved in Human Autophagy
Source: Cells. 2022 Oct 12;11(20):3203. doi: 10.3390/cells11203203 (PMC9601114; doi:10.3390/cells11203203)
Supplement: Supplementary file 1 [file cells-11-03203-s001.zip › Supplementary Table S5.pdf]

Supplementary Table S5. Minimum free energy (mFE) of mRNA of human autophagy genes.

SL. No	Human Autophagy Gene	mFE of mRNA (kcal/mol) (Absolute values)
1	*AMBRA1*	1942.6
2	*APOL1*	1279.5
3	*ARNT*	1452.5
4	*ARSA*	2036.5
5	*ARSB*	1567.1
6	*ATF4*	809.5
7	*ATF6*	2068.2
8	*ATG10*	1038.7
9	*ATG101*	626.3
10	*ATG12*	1083.62
11	*ATG13*	1491.7
12	*ATG14*	1671.8
13	*ATG16L1*	1123.9
14	*ATG16L2*	962.8
15	*ATG2A*	2808.9
16	*ATG2B**	-
17	*ATG3*	485.2
18	*ATG4A*	598.2
19	*ATG4B*	1129.8
20	*ATG4C*	708.7
21	*ATG4D*	896.6
22	*ATG5*	815.5
23	*ATG7*	1772.5
24	*ATG9A*	1497
25	*ATG9B*	1949.1
26	*ATIC*	602.9
27	*BAG1*	1290.8
28	*BAG3*	902.1
29	*BAK1*	939
30	*BAX*	267.3
31	*BCL2*	2110.5
32	*BCL2L1*	1005.4
33	*BECN1*	659.5
34	*BECN2*	549
35	*BID*	836
36	*BIRC5*	927.5
37	*BIRC6**	-

38	*BNIP1*	390.4
39	*BNIP3*	416.7
40	*BNIP3L*	862.1
41	*CALCOCO2*	1027.1
42	*CAMKK2*	1842.6
43	*CANX*	1316.8
44	*CAPN1*	1216.8
45	*CAPN10*	1276.8
46	*CAPN2*	1119.87
47	*CAPNS1*	566.9
48	*CASP1*	405
49	*CASP3*	703.8
50	*CASP4*	348.2
51	*CASP8*	936.4
52	*CCL2*	170.2
53	*CCR2*	716.1
54	*CD46*	843
55	*CDKN1A*	842.9
56	*CDKN1B*	772.4
57	*CDKN2A*	400.5
58	*CFLAR**	-
59	*CHMP2B*	600.5
60	*CHMP4B*	561.6
61	*CLN3*	776.1
62	*CTSB*	1596.4
63	*CTSD*	895.7
64	*CTSL*	545.3
65	*CX3CL1*	1383.8
66	*CXCR4*	524.1
67	*DAPK1*	2049.9
68	*DAPK2*	1057.6
69	*DDIT3*	298.4
70	*DIRAS3*	441.3
71	*DLC1*	2207.2
72	*DNAJB1*	784.9
73	*DNAJB9*	636.8
74	*DRAM1*	1007.2
75	*EDEM1*	1990.3
76	*EEF2*	1255
77	*EEF2K*	2753.1
78	*EGFR*	3138

79	*EIF2AK2**	-
80	*EIF2AK3*	1383.2
81	*EIF2S1*	1094.4
82	*EIF4EBP1*	325.6
83	*EIF4G1*	2194.5
84	*ERBB2*	1919.3
85	*ERN1*	2689.6
86	*ERO1A*	1278.6
87	*FADD*	702.9
88	*FAS*	923.8
89	*FKBP1A*	471.8
90	*FKBP1B*	313.3
91	*FOS*	722.9
92	*FOXO1*	1827.9
93	*FOXO3*	2461.8
94	*GAA*	1754.4
95	*GABARAP*	419.5
96	*GABARAPL1*	330.84
97	*GABARAPL2*	272.3
98	*GAPDH*	468.4
99	*GNAI3*	2641.4
100	*GOPC*	1249.44
101	*GRID1*	2369.12
102	*GRID2*	1349.41
103	*HDAC1*	687.2
104	*HDAC6*	1630.4
105	*HGS*	1219.1
106	*HIF1A*	960.8
107	*HSP90AB1*	762.1
108	*HSPA5*	1228.6
109	*HSPA8*	683.2
110	*HSPB8*	668.8
111	*IFNG*	274.6
112	*IKBKB*	1329.5
113	*IKBKE*	1283.1
114	*IL24*	628.9
115	*IRGM*	535.6
116	*ITGA3*	2102.1
117	*ITGA6*	1678.7
118	*ITGB1*	1012.1
119	*ITGB4*	2524.9

120	*ITPR1*	3087.2
121	*KIF5B*	1548.6
122	*KLHL24*	1917.1
123	*LAMP1*	1028
124	*LAMP2*	936.87
125	*MAP1LC3A*	385.9
126	*MAP1LC3B*	557.6
127	*MAP1LC3C*	413.6
128	*MAP2K7*	1525.37
129	*MAPK1*	1758.1
130	*MAPK3*	435.2
131	*MAPK8*	1470.7
132	*MAPK8IP1*	1362.5
133	*MAPK9*	639.1
134	*MBTPS2*	1223.3
135	*MLST8*	713.7
136	*MTMR14*	815.6
137	*MTOR*	3063.3
138	*MYC*	1494.8
139	*NAF1*	550.1
140	*NAMPT*	1032.27
141	*NBR1*	1444.9
142	*NCKAP1**	-
143	*NFE2L2*	628.3
144	*NFKB1*	1349.6
145	*NKX2-3*	941.5
146	*NLRC4*	1008.7
147	*NPC1*	1564.3
148	*NRBF2*	497.9
149	*NRG1**	-
150	*NRG2*	1664.5
151	*NRG3*	1227.8
152	*P4HB*	935.1
153	*PARP1*	1342.6
154	*PEA15*	857.3
155	*PELP1*	1929.6
156	*PEX14*	804.6
157	*PEX3*	680.6
158	*PIK3C3*	2472
159	*PIK3R4*	1456.4
160	*PINK1*	1116.7

161	*PPP1R15A*	920.91
162	*PRKAB1*	758.5
163	*PRKAR1A*	1189.7
164	*PRKCD*	951
165	*PRKCQ*	990.7
166	*PRKN*	1327.3
167	*PTEN*	2197.1
168	*PTK6*	1179
169	*RAB11A*	1227.4
170	*RAB1A*	633.5
171	*RAB24*	623.6
172	*RAB33B*	1046.3
173	*RAB5A*	694.5
174	*RAB7A*	610.1
175	*RAC1*	663.7
176	*RACK1*	393.2
177	*RAF1*	1090.8
178	*RB1*	1155.9
179	*RB1CC1*	1682.34
180	*RELA*	970.1
181	*RGS19*	695
182	*RHEB*	690.2
183	*RPS6KB1*	1365.4
184	*RPTOR*	2777.3
185	*RUBCN*	3155.12
186	*SAR1A*	1945.07
187	*SERPINA1*	1081
188	*SESN2*	1454.6
189	*SH3GLB1*	1661.8
190	*SIRT1*	1181.3
191	*SIRT2*	659.4
192	*SPHK1*	1050.4
193	*SPNS1*	1011.7
194	*SQSTM1*	1168.6
195	*ST13*	850.8
196	*STK11*	1633.9
197	*SUPT20H*	808.7
198	*TBK1*	744.3
199	*TM9SF1*	761.5
200	*TMEM41B*	212.2
201	*TMEM74*	1704.1

202	*TNFSF10*	441.6
203	*TP53*	892.4
204	*TP53INP2*	1483.3
205	*TP63*	1329.2
206	*TP73*	2174.4
207	*TSC1*	2753.3
208	*TSC2*	2663.9
209	*TUSC1*	646.7
210	*ULK1*	2352.7
211	*ULK2*	3028.3
212	*ULK3*	1124.1
213	*USP10*	1036.2
214	*UVRAG*	1589.1
215	*VAMP3*	597.4
216	*VAMP7*	638.4
217	*VEGFA*	1345.7
218	*VMP1*	1037
219	*WDFY3**	-
220	*WDR45*	743.6
221	*WDR45B*	834
222	*WIPI1*	591.2
223	*WIPI2*	1657.9
224	*ZFYVE1*	1590.2
